# Effectiveness of contrast-associated acute kidney injury prevention methods; a systematic review and network meta-analysis

**DOI:** 10.1186/s12882-018-1113-0

**Published:** 2018-11-13

**Authors:** Khalid Ahmed, Terri McVeigh, Raminta Cerneviciute, Sara Mohamed, Mohammad Tubassam, Mohammad Karim, Stewart Walsh

**Affiliations:** 1 Lambe Institute for Translational Research, Discipline of Surgery National University of Ireland, Galway, Republic of Ireland; 2 Department of Vascular surgery, Galway University Hospital, Galway, Republic of Ireland; 3 School of Population and Public Health, University of British Columbia, Scientist / Biostatistician, Centre for Health Evaluation and Outcome Sciences (CHEOS), St. Paul’s Hospital, Vancouver, Canada; 4 HRB Clinical Research Facility Galway, Galway, Republic of Ireland

**Keywords:** Contrast induced acute kidney injury, Contrast nephropathy, Prevention methods, Contrast associated acute kidney injury

## Abstract

**Background:**

Different methods to prevent contrast-associated acute kidney injury (CA-AKI) have been proposed in recent years. We performed a mixed treatment comparison to evaluate and rank suggested interventions.

**Methods:**

A comprehensive Systematic review and a Bayesian network meta-analysis of randomised controlled trials was completed. Results were tabulated and graphically represented using a network diagram; forest plots and league tables were shown to rank treatments by the surface under the cumulative ranking curve (SUCRA). A stacked bar chart rankogram was generated. We performed main analysis with 200 RCTs and three analyses according to contrast media and high or normal baseline renal profile that includes 173, 112 & 60 RCTs respectively.

**Results:**

We have included 200 trials with 42,273 patients and 44 interventions. The primary outcome was CI-AKI, defined as ≥25% relative increase or ≥ 0.5 mg/dl increase from baseline creatinine one to 5 days post contrast exposure. The top ranked interventions through different analyses were Allopurinol, Prostaglandin E1 (PGE1) & Oxygen (0.9647, 0.7809 & 0.7527 in the main analysis). Comparatively, reference treatment intravenous hydration was ranked lower but better than Placebo (0.3124 VS 0.2694 in the main analysis).

**Conclusion:**

Multiple CA-AKI preventive interventions have been tested in RCTs. This network evaluates data for all the explored options. The results suggest that some options (particularly allopurinol, PGE1 & Oxygen) deserve further evaluation in a larger well-designed RCTs.

**Electronic supplementary material:**

The online version of this article (10.1186/s12882-018-1113-0) contains supplementary material, which is available to authorized users.

## Background

### Rationale

Contrast Associated acute kidney injury (CA-AKI) also known as Contrast-induced acute kidney injury (CI-AKI) previously known as contrast induced nephropathy (CIN) is the third leading cause of hospital-acquired acute renal injury, accounting for 12% of cases [1]. It is defined as an abrupt deterioration in renal function following exposure to contrast media (CM) in the absence of other aetiological factors [2]. The absolute and relative values used to define CI-AKI vary, but are most commonly quoted as a relative increase of > 25% or an absolute increase of 0.5 mg/dL and ≥ 0.3 mg from baseline serum creatinine measurement within 1–3 (4–5 days less frequently used) of contrast exposure [3–7]. In CI-AKI, the serum creatinine level begins to rise within 24 h of contrast exposure, peaking after 72 h, and usually returning to baseline within 1–3 weeks [6].

The proposed pathophysiology of CI-AKI is acute tubular necrosis. The underlying mechanisms are thought to be vasoconstriction, leading to cellular hypoxia, or direct toxicity of contrast media to renal tubular cells [8, 9]. Multiple therapies have been postulated to prevent CI-AKI act by affecting these mechanisms or their metabolic mediators.

There is ongoing discussion about the impact of new contrast media on the size of the problem and the outcomes of prevention methods or even the existence of the problem, on the other side these conclusions were challenged as coming only from retrospective studies that does not take in account patients factors or indications for using contrast media in deferent cases with deferent baseline renal profile [10, 11].

In recent years, there have been many systematic reviews and meta-analyses for direct pair-wise comparisons of individual interventions suggested for CI-AKI prevention. With so many options explored, it is difficult to determine the treatment options most likely to show benefit in large-scale trials. Unlike conventional meta-analysis, Network facilitates simultaneous comparison of indirect relationships between multiple interventions. The network can establish an estimate of comparative efficacy between two or more treatments compared to the same control intervention [12–14]. We undertook a network-meta-analysis of preventive strategies for CA-AKI to determine the treatment most likely to be beneficial based upon currently available evidence.

## Methods

We conducted a systematic review and network meta-analysis in accordance with the PRISMA extension for Network Meta-Analyses [15].

### Protocol and registration

No registered protocol.

### Eligibility criteria

We consider all randomized controlled trials in which patients underwent a contrast-enhanced procedure with CI-AKI as a primary or secondary outcome. We evaluate studies in which a prevention method was compared to placebo, control or other intervention. Excluded from the analysis were other research designs, including non-randomised control trials; clinical trials; trials comparing different doses of the same intervention and trials using re-randomization of the same sample (Crossover design). For this review, we defined CI-AKI as an increase of more than or equal to 0.5 mg/dl and/or 25% increase in baseline serum Creatinine one to 5 days post contrast exposure [3].

### Information sources

We searched for English-language trials in PubMed, Embase and Cochrane Central Register of Controlled Trials without any date restrictions. The final search was undertaken on 25th April 2017.

### Search strategy and study selection

Two authors (Ahmed, Walsh) searched Electronic databases using Mesh terms “contrast nephropathy”, “contrast nephropathy prophylaxis”, “contrast nephropathy prevention”, with the Boolean operator “OR” as appropriate. Titles and abstracts of identified studies were assessed first, with full texts reviewed thereafter. The study was included if the methodology fulfilled inclusion criterion.

### Data collection

Data were recorded concerning sample size, adverse events, procedures performed, study inclusion and exclusion criteria, intervention type and dose, contrast media volume, CI-AKI definition, and contrast medium type and osmolality.

### The geometry of the network

A network diagram was created using NetMetaXL tool to graphically represent the size of the trial and the number of pairwise comparisons between interventions. The size of each intervention node is proportional to a number of patients included in the trial, while the thickness of interconnecting lines is proportional to the number of pairwise comparisons between any two interventions.

### Risk of bias

The Cochrane tool for risk of bias assessment (RevMan 5.3) was used to assess bias within individual studies. A bias graph was generated to portray the risk of bias overall across the included trials.

### Summary measures

Odds ratios with 95% confidence intervals were calculated and presented in the form of Forest plots we generated a league table, which ranks summary estimates in order of the impact of the intervention on the primary outcome measure [10]. In the league table, interventions were ranked from those with the highest effect to the lowest. A stacked bar chart rankogram was also created to represent ranking probabilities and their uncertainty.

### Analysis methods

Data with respect to events and number of patients in individual trials were prepared and entered using NetMetaXL [16], to facilitate completion of a Bayesian network meta-analysis using WinBUGS version 1.4.3 from within Microsoft excel. We used the Markov Chain Monte Carlo method of parameter estimation to obtain posterior estimates of effects. Both vague prior and informative prior results were presented in the Forest Plot. Zero cells were adjusted using an adjusted continuity correction factor accounting for potential differences in sample size, centered around 0.5.

As NetMetaXL is a relatively new tool, we run a separate set of analyses for the same data on GeMTC R package to validate our results with no noticeable differences.

We performed analysis with both fixed effects models and random effects random-effect hierarchical models. For Bayesian computation; detailed statistical approach and diagnostics are provided in Additional file 1.

### Assessment of consistency, model fit, and convergence

In NetMetaXL, ‘inconsistency plot’ was generated to facilitate visual assessment of conflicts between direct and indirect evidence with limitation in our analysis due to a substantial number of nodes on excel. Heterogeneity for vague and informative priors was provided within the forest plot results & Monte Carlo error < 5% of the standard deviation (SD) used to assess convergence.

For GeMTC R package Gelman-Rubin statistics used numerically and graphically to evaluate convergence while deviance information criterion (DIC) was used for determining model fits and the model with smaller DIC value was considered better.

### Additional analyses

In addition to the main analysis we performed three other analysis, the first excluding RCTs with any partial use of hyperosmolar contrast media and in the other two RCTs were divided according to baseline renal profile.

For each of the four analyses we performed sub-analysis excluding studies with zero values as corresponding effects estimates may be subject to numerical instability, generally over-estimate the effect, and that can be observed in the wide associated confidence intervals.

## Results

### Study selection

**Fig. 1:**
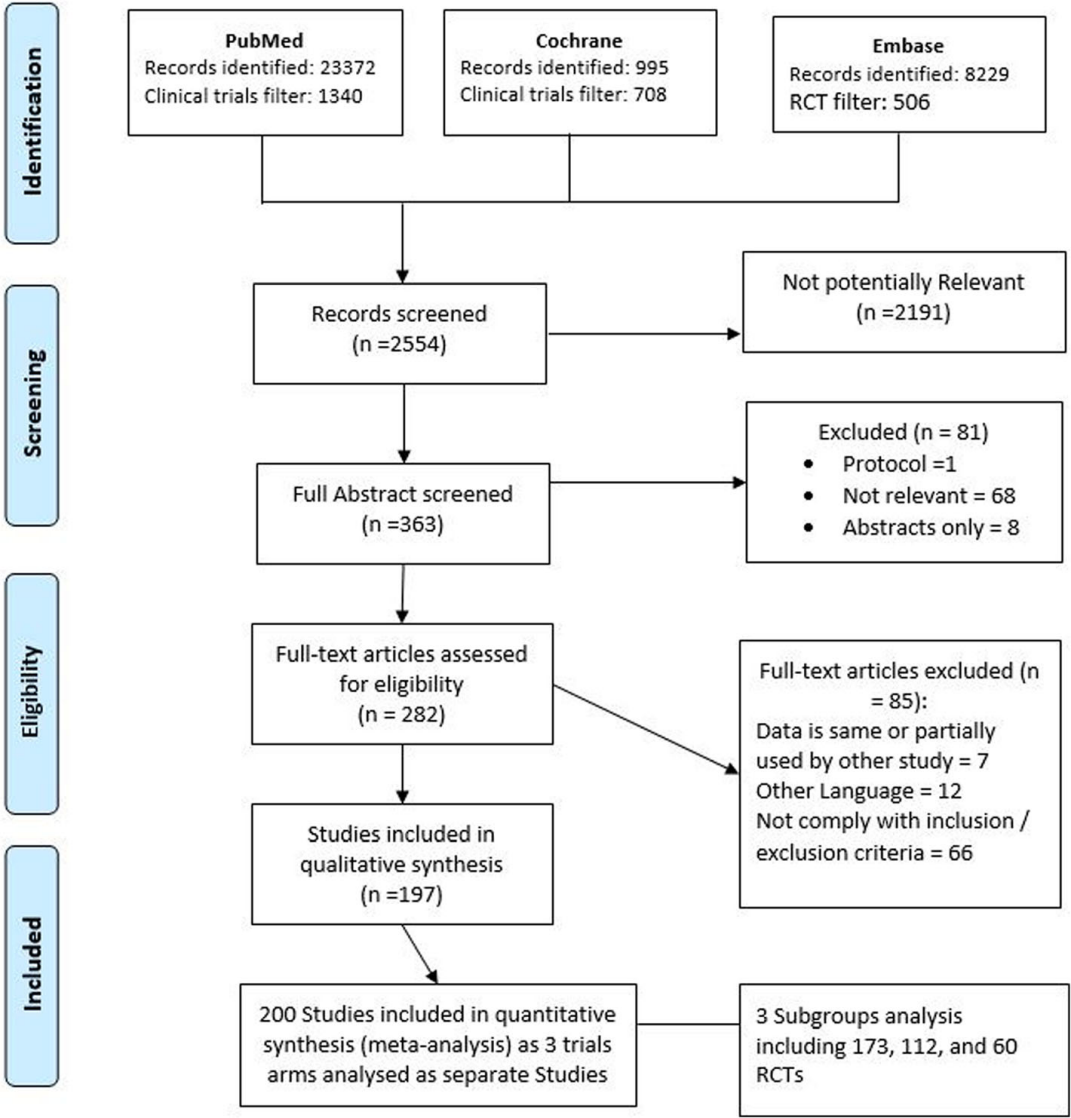
Flow Diagram

A total of 32,596 study titles were identified in the initial literature search, of which 200 fulfilled criteria for inclusion [4, 5, 7, 17–209] (Fig. 1). Some studies were excluded as some data were partially included or re-analyzed in a follow-up study involved in our review [210–215]. A total of 32,399 studies were excluded after remove duplication the most common reasons for exclusions after full examination included observational methodology; different outcome measures, inadequate definition of CI-AKI; unclear evidence of randomization; old studies that did not comply with eligibility criteria for more than one reason [216–279]. The twelve studies published in a non-English language included those from centers in Germany [280, 281], China [282–287], Spain [288], France [289], Turkey [290] and Italy [291]. Eight further potentially suitable studies were identified in abstract form only, but were excluded as no full-text article could be identified [292–299].

### Study characteristics

Additional file 2 outlines individual study characteristics (study inclusion and exclusion criteria; procedure performed; baseline renal function; definition of CI-AKI used in the study; contrast medium volume and osmolality). In total, 197studies fulfilled the inclusion criteria, including three which had multiple trial arms requiring separate analyses (Yang 2014, Kumar 2014 & Chen 2008). A total of 200 comparative analyses were therefore included in our analyses. Coronary angiography accounted for 145 (72.5%) of the contrast-dependent procedures were. Less frequently reported procedures included contrast-enhanced CT imaging (*n* = 16, 8%), peripheral angiography with/without angioplasty and stenting (*n* = 3, 1.5%) endovascular aneurysm repairs (EVAR) (n = 1, 0.5. %). Multiple procedures were included in 35 studies (17.5%). Low osmolar contrast agents were used in 111 (55.5%), iso-osmolar agents in 44 studies (22%), and hi-osmolar media in 3 studies (1.5%). Twenty-six (13%) trials permitted physician discretion in the selection of contrast media, while a further 16 (8%) did not specify the contrast medium utilized. More recent studies we observed better design with an exclusion for patients using alternative CI-AKI prevention interventions from participation or stratified those methods among arms of the trial.

### Network structure

**Fig. 2:**
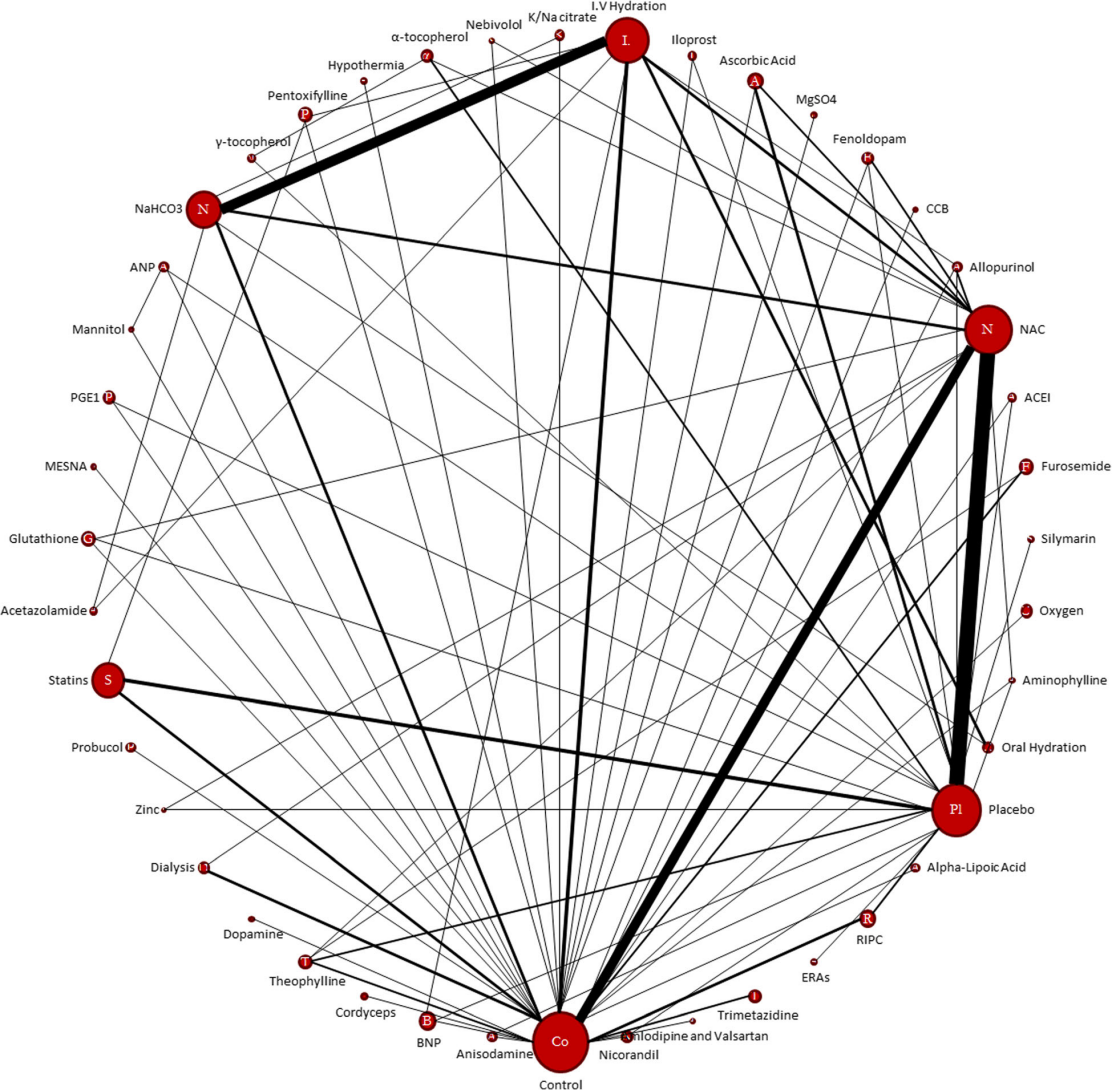
Network Diagram: The size of each intervention node is proportional to the number of patients included in the trials, while the thickness of interconnecting lines is proportional to the number of pairwise comparisons between any two interventions

The relationship and comparisons between included studies are demonstrated in the network diagram (Fig. 2). Forty-four interventions are included in this network (Table 1).

**Table 1:** Interventions within Network Diagram

NO	Drug	Abbreviation	Patients
1	I.V Hydration	I.V	5136
2	Statins	Sta	3040
3	Furosemide	Fur	554
4	NAC	NAC	6095
5	Trimetazidine	Tri	352
6	NaHCO3	NaH	3393
7	PGE1	PGE	304
8	MgSO4	MgS	62
9	Pentoxifylline	Pen	438
10	Placebo	Pla	7044
11	Control	Con	9120
12	Allopurinol	All	204
13	BNP	BNP	744
14	Probucol	Pro	198
15	α-tocopherol	α-t	312
16	γ-tocopherol	γ-t	102
17	Oxygen	Oxy	346
18	Amlodipine and Valsartan	Aml	45
19	K/Na citrate	K/N	203
20	Nicorandil	Nic	291
21	Ascorbic Acid	Asc	552
22	Alpha-Lipoic Acid	Alp	139
23	Oral Hydration	Ora	254
24	Nebivolol	Neb	40
25	Anisodamine	Ani	192
26	RIPC	RIP	608
27	Theophylline	The	384
28	Hypothermia	Hyp	58
29	Glutathione	Glu	421
30	MESNA	MES	51
31	ACEI	AC	129
32	Aminophylline	Ami	45
33	Iloprost	Ilo	118
34	Acetazolamide	Ace	94
35	ANP	ANP	202
36	Zinc	Zin	18
37	Dialysis	Dia	293
38	Fenoldopam	Fe	333
39	ERAs	ER	77
40	CCB	CC	42
41	Dopamine	Do	48
42	Mannitol	Ma	35
43	Cordyceps	Co	88
44	Silymarin	Si	69

*ACEI* Angiotensin Converting-Enzyme Inhibitor, *ANP Atrial Natriuretic Peptide*, *BNP* B-Type Natriuretic Peptide, *CCB* Calcium Channels Blockers, *CI-AKI* Contrast Induced Acute Kidney Injury, *CIN* Contrast Induced Nephropathy, *ERAs* Endothelin Receptor Antagonism, *MESNA* 2-Mercaptoethane Sulfonate Sodium, *MgSo4* Magnesium Sulphate, *NAC N*-*acetyl cysteine*, *NaHco3* Sodium Bicarbonate, *PGE1* Prostaglandin E1, *RIPC* Remote Ischemic Preconditioning

### Network geometry

Data from 42,273 patients recruited to 200 trials investigating 44 interventions were included in our analyses; a summary of network characteristics is provided in (Table 2). Nine hundred and forty-six pair-wise comparisons were possible, of which 81 used data from direct comparisons in Additional file 3. The most commonly investigated comparisons are between N-acetylcysteine (NAC) and placebo (36 studies, 8,202patients); and intravenous normal saline and intravenous sodium bicarbonate (24 studies, 5,481patients). The interventions most commonly investigated were NAC, NaHCO3, Statins, Intravenous Hydration (I.V), and placebo or control. The characteristics of individual interventions are outlined in Additional file 3.

**Table 2:** Network Characteristics

Characteristic	Number
Number of Interventions	44
Number of Studies	200
Total Number of Patients in Network	42,273
Total Number of Events in Network	4602
Total Possible Pairwise Comparisons	946
Total Number Pairwise Comparisons with Direct Data	81
Number of Two-arm Studies	179
Number of Multi-Arms Studies	21
Number of Studies with No Zero Events	184
Number of Studies With At Least One Zero Event	16
Number of Studies with All Zero Events	2

### Risk of bias

**Fig. 3:**
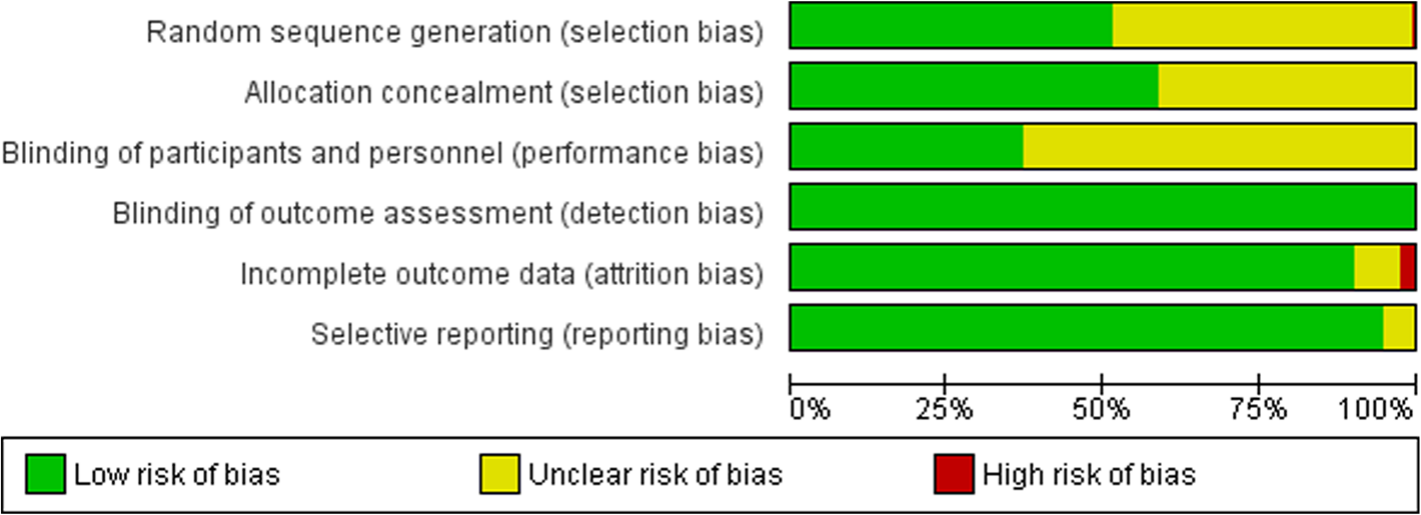
Risk of Bias Graph

Risk of bias assessed by two authors (Khalid, Walsh). In case of disagreement, other authors were consulted. Summary for individual studies provided in Additional file 4 while (Fig. 3) shows the risk of bias graph across all studies. Most of the studies demonstrated unclear to low risk of bias while most of the high risk of bias were observed in attrition bias domain. As the outcome measure (CA-AKI) is dependent on laboratory results it seems reasonable to assume the risk of bias attributed to blinding of outcome assessment domain was low by default.

### Synthesis of results

The Renal Association, British Cardiovascular Intervention Society and the Royal College of Radiologists among many other medical bodies recommend using intravenous volume expansion as a prevention method for CA-AKI [300]. Thus, we considered intravenous hydration clinically the reference intervention in this analysis, in addition to the node size and the multiple arms within the network which make it very good comparator.

**Fig. 4:**
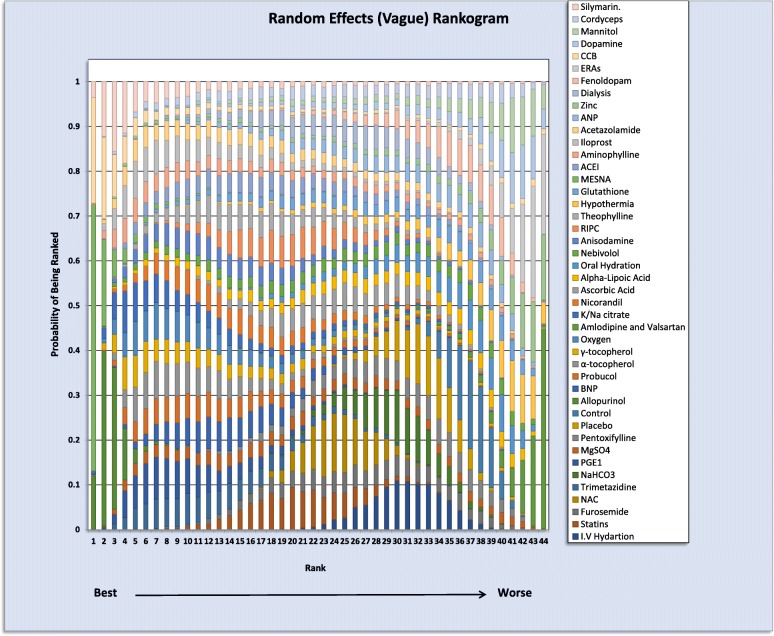
Rankogram: ranking the interventions for the probability of being the best, the interventions are colour coded; the first column represents the chance of being first best and 2nd column is the chance of being 2nd best and so on. i.e. the first column represent the chance of being first best cmparing all interventions out of100% and the second represent the chance of being second best out of 100% up to last column in this case number 44 (nuber of interventions); the overall ranking for each treatment is the sum of scores through out the 44 compasrisons. The overall numerical value is presented in Table 3

A forest plot was generated to demonstrate odds ratio generated from direct and indirect pair-wise comparisons. Effect estimates, and confidence intervals were included for both vague and informative priors using a random effects model. The overall heterogeneity for the vague prior was 0.54 (95% CI 0.41–0.69), while that for informative prior was 0.498 (95% CI 0.366–0.6403). The SUCRA (surface under the cumulative ranking curve) was utilized to generate a stacked bar chart rankogram (Fig. 4). A league table arranging summary of effect estimate, and ranking interventions according to impact on the outcome can be found in Additional file 3 in addition to the Forest Plot, characteristics of interventions and comparisons and analysis specifications. The probabilities of being ranked for the best each intervention is summarized in (Table 3) while the numerical values follow the Rankogram results the list of interventions in the first column follow the league table hierarchy and a good example is Allopurinol which included in 4 studies ranked best in both Rankogram (0.9647) and League Table while Silymarin was 3rd (0.7934) and last respectively and was included in one study.

**Table 3:** Interventions ranking the treatments names column follow the league table (which arranges the presentation of summary estimates by ranking the treatments in order of most pronounced impact on the outcome under consideration) the numerical values represents the cumulative results of the probability of being best in which the highest score is 1 or 100% (see Rankogram)

Treatment	SUCRA	Treatment	SUCRA
Allopurinol	0.9647	NaHCO3	0.3419
MESNA	0.9427	Pentoxifylline	0.3391
PGE1	0.7809	I.V Hydration	0.3124
α-tocopherol	0.7614	Placebo	0.2694
Oxygen	0.7527	Oral Hydration	0.2517
K/Na citrate	0.7469	Hypothermia	0.2021
Trimetazidine	0.7151	Control	0.1658
Probucol	0.7042	Amlodipine and Valsartan	0.05485
γ-tocopherol	0.689	ACEI	0.5783
BNP	0.6767	Aminophylline	0.6593
Anisodamine	0.6594	Iloprost	0.7481
Nicorandil	0.6442	Acetazolamide	0.6242
Theophylline	0.629	ANP	0.3291
RIPC	0.5692	Zinc	0.198
Statins	0.5497	Dialysis	0.4319
MgSO4	0.5177	Fenoldopam	0.2296
NAC	0.4592	ERAs	0.06734
Nebivolol	0.4543	CCB	0.7249
Ascorbic Acid	0.4433	Dopamine	0.1916
Alpha-Lipoic Acid	0.4322	Mannitol	0.1905
Furosemide	0.4027	Cordyceps	0.4459
Glutathione	0.3554	Silymarin	0.7934
*Analysis*	Random Effects (Vague)		

### Sensitivity analysis

Flow chart for the main analyses and sub-analyses is included in Additional file 1. From the main analysis 200 RCTs we run sub-analysis that includes 184 RCTS in which we exclude all studies with zero values (*n* = 7). All figures and tables are included in Additional file 3.

The second analysis involved 173 RCTs after excluding studies reporting any use of hyperosmolar contrast media, the sub-analysis without zero values RCTs include 159 RCTS.

Trials with high baseline renal profile were in analysis 3 which includes 112 RCTs and sub-analysis for 105 RCTS. The 4th analysis includes 60 and 53 RCTs respectively. Analysis specifications, figures and tables provided in Additional file 5, Additional file 6 and Additional file 7.

When interpreting sub-analyses results in conventional direct pairwise comparisons the main effect results from the size of the excluded studies because there is no exclusion for interventions and they will always be present at both sides of the forest plot. This impact the overall diamond shape effect estimates size and confidence interval will either shift towards one treatment or touching the line of no effect indicating no superiority for any intervention. This is different in Network Meta-analysis in which we can see changes in connections dynamic (Network Diagram) and interventions numbers represented by node sizes and number of connections between them both can be affected or totally removed by the excluding studies. In the latter case the Network Diagram and characteristics of interventions and comparisons provide detailed visualization to help compare the main vs sub-analysis. In Additional file 3, Additional file 5, Additional file 6 and Additional file 7 we detailed all excluded studies, the affected interventions, Network Diagrams and the characteristics of the interventions and comparisons.

### Assessment of consistency

**Fig. 5:**
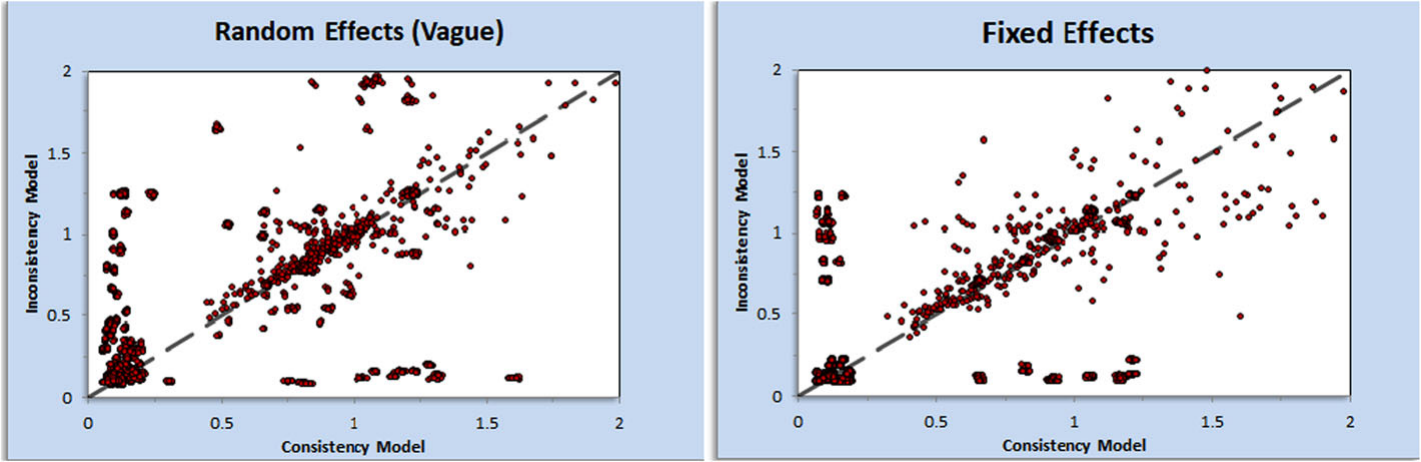
Inconsistency Plot: Inconsistency is similar to heterogeneity in conventional meta-analysis, but consistency concerns the relation between the direct and indirect evidence. The consistency plot shows individual data points’ posterior mean deviance contributions for the consistency model (horizontal axis) and the unrelated mean effects model (vertical axis) along with the line of equality

An ‘inconsistency plot’ (Fig. 5) was generated to assess inconsistency. Inconsistency in network meta-analysis is similar to heterogeneity in conventional meta-analysis but consistency concerns the relation between the treatments whereas heterogeneity concerns the variation between trials within a pairwise comparison between two treatments. Inconsistency is caused by imbalances in the distribution of effect modifiers in the direct and indirect evidence. Effects modifiers in this large sample include but are not limited to patient factors, drug interactions, contrast media volume and type and renal function pre-intervention. Inevitably, some modifiers exist that cannot be completely eliminated in large multi-treatment network meta-analysis, leading to some inconsistency, indicating a need for careful interpretation of the results [301]. The consistency plot shows individual data points’ posterior mean deviance contributions for the consistency model (horizontal axis) and the unrelated mean effects model (vertical axis) along with the line of equality. In our analysis, the main limitation is excel inability to handle a large amount of nodes. However, there should be a consideration of individual pairwise comparisons effect estimates generated within the forest plot.

In GeMTC R analyses I^2^ statistics and DIC was much smaller for Random effect indicating less heterogeneity compared with a fixed effect which is expected to provide the nature of the network. Detailed scores are presented in Additional file 1 while Gelman and Rubin’s convergence diagnostics were added to corresponded analyses in Additional file 3, Additional file 5, Additional file 6 and Additional file 7.

In general, the main analysis reviled some interesting results with Allopurinol, Prostaglandin E1 (PGE1) & Oxygen were ranked high with good both statistical and clinical outcomes in relatively fewer number of studies comparing with other interventions studied in larger number of RCTs e.g. NAC, Statins, Hydration, NaHco3 and RIPC. The results were stable throughout different sub analysis considering the changes in network diagram being affected by excluded studies in all 7 networks. The model fitting and the consistency within the network was good considering the large size and it is understandable that it was better fitted in the 7 sub-groups analysis specially after excluding zero values studies. It is very important here to remember in network ranking is the probability of being the best within the interventions and we need to look at the forest plot for each comparison.

## Discussion

### Summary of evidence

This is a systematic review and network meta-analysis (multi-treatment comparison) of studies investigating methods for the prevention of contrast-induced nephropathy. We identified 200 eligible trials, of which 3 had 2 different arms and thus analysed separately. Data from a total of 42,273 patients undergoing 44 different interventions were included. Intravenous hydration (Nacl) was used as the reference treatment as there is a consensus supported by evidence accepting it as a method of prevention with no clear superiority for other I.V fluids [81]. in our network it was also included in many multiple arms RCTs which make it statistically a very good comparator. While only randomized control trials were included, defining the outcome and inclusion criteria, help to minimize the number of effect modifiers at play in different studies, thus minimising inconsistency. However, the assumption of homogeneity should be accepted with caution in light of the large numbers of trials and patients included.

It is very important for readers more familiar with general probability measure in which the value one is assigned to the entire probability space to recognize that SUCRA use posterior probabilities for each treatment to be among the *n* - best options (cumulative probabilities) thus the sum add to > 1. The word best referred to the number of times that an intervention ranks first out of the total number of random samples [14] In Rankogram the first column represent the chance of being first best out of100% and the second represent the chance of being second best up to last column; the overall ranking for each treatment is the sum and that the reason each treatment probability is calculated out of 100%.

We can generally categorize the 44 ranked interventions in groups. The first group is high ranked interventions with relatively fewer number of studies and this group is mainly for further research consideration despite good design RCTs, good clinical outcomes, and our conscious effort to eliminate the effect of small node effect on the network and the fact we accommodate and accounted for the different in interventions size when calculating the probability but we cannot ignore that this may still play in favour of small studies and we think they deserve another look with larger well-designed trials, this group includes mainly Allopurinol, Prostaglandin E1 (PGE1) & Oxygen; Allopurinol a xanthine oxidase Inhibitor used for treatment of gout and management of hyperuricemia associated with chemotherapy and was assessed in 4 trials with 204 patients with recent published evidence suggesting some benefits [302] while PGE1 in 4 trails with total 304 patients. Interestingly Oxygen was highly ranked before and after exclusion of zero events studies and the total number of patients was 346 in 2 studies.

The scorned group is the middle group which included in decent number of studies and the interventions in this group with safe and or well tested profile can be used in patient care at the same time continuously evaluated and this group can include RIPC, Statins (which usually in use specially by cardiac and vascular patients), NAC, NaHco3, I.V hydration, Oral hydration and hypothermia. This group needed the physician to consult his local guidelines after evaluating each patient individually and some interventions like hypothermia is not applicable for all patients.

The sub-analyses in our network for was performed after excluding studies with zero events to eliminate favorable effect profile. It produced better statistical results and helped compare the results without the interventions involved in a small number of trials.

### Research & Clinical impact

For health care providers, the results of this meta-analysis do not suggest changes to current clinical practice. The prevention methods assessed in large studies should be evaluated on a case-by-case basis, bearing in mind the comorbidities, clinical needs and prior risk factors of the individual patient with special consideration to national and local guidelines. Interventions with safe profile and supportive evidence from direct pair-wise meta-analysis can be considered as additional or second-line therapies for CA-AKI prevention. For clinical researchers, the highly-ranked treatments with relatively small number of trials merit further examonation in larger RCTs.

### Limitations

One limitation of this meta-analysis is the exclusion of non-English language studies (*n* = 12). The inclusion of these studies may add to the supportive evidence for the use of some interventions, although the effect size of these trials is likely to be minimal in light of the sample sizes in question. Another limitation is the difference in contrast media used which may affect the outcomes; we excluded studies that used hyperosmolar contrast media to minimise this effect with some evidence suggesting similar CIN incidence for iso and low-osmolar CM in coronary angiography patients [303]. In large Network, another consideration is our inability to account for other possible effect modifiers, and our assumptions regarding homogeneity and similarity across a large number of studies thus it is important to look at each intervention ranking through the multiple analyses provided in the supplemnts.

While preparing this network meta-analysis a pairwise meta-analysis was published .comparing *N*-acetylcysteine, sodium bicarbonate, statins and ascorbic acid for CA-AKI reduction [304]. The data was obtained from controlled trials that used intravenous (IV) or intra-arterial contrast. The results of statins plus I.V saline vs I.V saline show clinically but not statistically significant difference. When comparing Sodium bicarbonate to I.V saline it was clinically better, but again the difference was not statistically significant. However Ascorbic acid was better both clinically and statistically vs I.V saline and show no such difference when compared with NAC. A similar result can be observed in our ranking table with 0.5497, 0.4433, 0.3419 and 0.3124 probability of being rank for statins, ascorbic acid, Sodium bicarbonate and I.V saline consequently. Although direct comparisons results were provided within forest plot in our network, we think the results from pairwise reviews is important; the nature of conventional meta-analysis prevent utilization of multiple arms trials and creating indirect comparison but it can be used to look at sections of more comprehensive network-meta-analysis in addition to the fact that It is more flexible in terms of subgroup analysis and thus assessment of effects modifiers e.g. type of contrast media in this case.

## Conclusion

This systematic review and network meta-analysis provide a comprehensive analysis of currently utilized CA-AKI prevention interventions. Results arising from this network identified some highly-ranked interventions throughout analyses and sub-analyses (e.g., Allopurinol, PGE1 & Oxygen) which were included in small number of trials and merit further examination on a larger scale in the context of a well-designed RCTs.

## Additional files

Additional file 1: Analysis flow chart and statistical approach. (DOCX 125 kb)

Additional file 2: Studies characteristics. (DOCX 889 kb)

Additional file 3: Main Analysis 200–184 RCTs. (DOCX 46108 kb)

Additional file 4: Risk of Bias Table. (DOCX 1056 kb)

Additional file 5: Excluding hyperosmolar 173–159 RCTs. (DOCX 41962 kb)

Additional file 6: High Baseline Renal Profile 112–105 RCTs. (DOCX 32278 kb)

Additional file 7: Normal Baseline Renal Profile 60–53 RCTs. (DOCX 16081 kb)

## Abbreviations

ACEI: Angiotensin Converting-Enzyme Inhibitor
ANP: Atrial Natriuretic Peptide
BNP: B-Type Natriuretic Peptide
CCB: Calcium Channels Blockers
CI-AKI: Contrast Induced Acute Kidney Injury
CIN: Contrast Induced Nephropathy
CM: Contrast Media
ERAs: Endothelin Receptor Antagonism
MESNA: 2-Mercaptoethane Sulfonate Sodium
MgSo4: Magnesium Sulphate
NAC: N*-*acetyl cysteine
NaHco3: Sodium Bicarbonate
PGE1: Prostaglandin E1
RCT: Randomise Control Trails
RIPC: Remote Ischemic Preconditioning
