# Supplementary material for: Effectiveness of contrast-associated acute kidney injury prevention methods; a systematic review and network meta-analysis
Source: BMC Nephrol. 2018 Nov 13;19:323. doi: 10.1186/s12882-018-1113-0 (PMC6234687; doi:10.1186/s12882-018-1113-0)
Supplement: Supplementary file 1 — Analysis flow chart and statistical approach. (DOCX 125 kb) [file 12882_2018_1113_MOESM1_ESM.docx]

Supplement 1

**Statistical Methods**

**Analyses Flow Chart:**

Main Analysis 200 RCTs

Sub-analysis excluding RCTs with zero values = 184 RCTs

Sub-analysis excluding RCTs with partial use of hyperosmolar contrast media = 173 RCTs

Sub-analysis excluding RCTs with zero values = 159 RCTs

Sub-analysis: RCTs with Normal renal baseline = 60

Sub-analysis: RCTs with Abnormal renal baseline = 112

Sub-analysis excluding RCTs with zero values = 105RCTs

Sub-analysis excluding RCTs with zero values = 53

**Statistical Analysis**

In this study, we compared different interventions to prevent CI-AKI in various recent clinical trials. For such a comparison, we resort to a Bayesian version of the network meta-analysis (NMA)(1, 2) that allows us to perform both direct and indirect comparisons of the interventions and produces comparative effectiveness estimates, expressed as odds ratios as well as the corresponding 95% credible intervals. These credible intervals are the Bayesian analog to confidence intervals frequently utilized in traditional frequentist methods. Contrary to regular meta-analysis, NMA allows us to compare the interventions both directly and indirectly, irrespective of whether they were evaluated directly in any head-to-head clinical trial or not. Performing NMA in a Bayesian manner enables us to rank of different interventions and evaluate the comparative effectiveness in a probabilistic sense by ordering them in terms of likelihood of being the best with respect to achieving the favorable outcome.

We use NetMetaXL and GeMTC R (3-5). The first is excel based sheet for tabulating data and send it to WinBug 1.4.3 and generate graphical figures after receiving results from the statistical package (6). It is a relatively new tool thus validating results was necessary using GeMTC R. There was no substantial difference in results, and the tool can be improved more for future usage.

**Model Specifications**

In our settings, we considered binomial likelihoods with a logit link function and performed analysis with both fixed effects models and random effects random-effect hierarchical models. For Bayesian computation, we assumed vague prior distributions for the study baseline, the effect parameters, and precision. To estimate posterior distributions in the Bayesian analysis, we repeated Monte Carlo simulations 20000 (10000in NetMetaXL) times after a 5000-iteration training phase (burn- in) and considered the thinning interval between consecutive observations to be 20. For the analysis of 200 trials, we needed more simulations: 10,000 simulations as burn-in and additional 50,000 simulations for the main analysis

**Assessment of Model Fit and Convergence:**

In NetMetaXL ‘inconsistency plot’ was generated to facilitate visual assessment of conflicts between direct and indirect evidence with limitation in our analysis due to a substantial number of nodes on excel. Heterogeneity for vague and informative priors was provided within the forest plot results & Monte Carlo error < 5% of the standard deviation (SD) used to assess convergence (7). Within GeMTC R Convergence diagnostics such as Gelman-Rubin statistic (8)and it’s multivariate version (9) were evaluated both numerically and graphically. For evaluating model fits, we used the deviance information criterion (DIC): the model with smaller DIC value was considered to be better.

Each main analysis results were generated using NetMetaXL which generates in addition to Forest Plot a Network Diagram, Rankogram & summary data tables (10).

**Sensitivity Analysis:**

Ability to perform indirect and mixed pairwise comparisons by NMA comes at a price of a number of required restrictive assumptions, for which we need assess how much heterogeneity was accounted for using the chosen model. Higgins I^2^ statistics was used for heterogeneity assessment within the network (11-13). I^2^ close to 30 may be an indication that mild heterogeneity exists in the study (14).

**Handling Zero Values:**

For the studies with zero events in any given intervention arm, it is possible to perform the analysis by using continuity correction (say, proportional to the sample size in the other treatment arm and the respective study sizes) (15, 16). Such corrections are generally meant for one single study with zero events (16). However, these corrections may lead to bias and the size and direction of them are unclear (17, 18). In more extreme scenarios, if more than one the treatment arms have zero events, they provide no information on the magnitude of the corresponding effects and may not contribute to the non-null combined treatment effect estimate. Therefore, those studies are generally excluded from comparisons (19-25). Even for the studies with zero event in either arm, the corresponding effects estimates may be subject to numerical instability, generally over-estimate the effect, the associated confidence intervals are sometimes prohibitively wide and skewed, MCMC samples have poor convergence, and in extreme cases, no converge at all (1, 23). Our case includes multiple zero events occurred in one or more arms within a study and many such studies existed within the network. Therefore, instability was high while considering all these studies, which could not be resolved by increasing Monte Carlo burn-in or additional thinning. Therefore, obtaining stable estimate required excluding these studies with zero events (16, 21). Clinically these studies has better outcomes thus we performed each analysis without excluding and sub-analysis after zero events RCTs exclusion and ranking were compared in each case. In our conclusion, we highlight the need for further large-scale trials to examine these interventions

Diagnostics:

For specification of settings and Gelman Rubin diagnostics referred to correlated supplements

**Diagnostics:**

**1. Main Analysis:**

| **NetMetaXL: Main Analysis (200 RCTs)** | | | |
| --- | --- | --- | --- |
|  | FE | RE Informative Prior | RE Vague Prior |
| Dbar | 2087.32 | 1935.38 | 1928.32 |
| Dhat | 1846.18 | 1623.38 | 1609.99 |
| pD | 241.148 | 312.614 | 318.331 |
| DIC | 2328.47 | 2248.6 | 2246.66 |
| **NetMetaXL: Main Analysis After Excluding RCTs with zero Events (184 RCTs)** | | | |
| Dbar | 1944.51 | 1815.28 | 1807.76 |
| Dhat | 1720.29 | 1528.57 | 1515.44 |
| pD | 224.219 | 286.712 | 292.321 |
| DIC | 2168.73 | 2101.99 | 2100.08 |
| **GeMTC R: Main Analysis After Excluding RCTs with zero Events (184 RCTs)** | | | |
| Dbar | 531.4341 | N/A | 394.5256 |
| I^2^ | 28% | N/A | 3% |
| pD | 228.6844 | N/A | 294.5448 |
| DIC | 1185 | N/A | 689.0705 |

**2. Excluding RCTs with Partial Use of Hyperosmolar Contrast media:**

| **NetMetaXL: Main Analysis (173 RCTs)** | | | |
| --- | --- | --- | --- |
|  | FE | RE Informative Prior | RE Vague Prior |
| Dbar | N/A | 1672.71 | 1664.32 |
| Dhat | N/A | 1405.35 | 1390.76 |
| pD | N/A | 267.361 | 273.561 |
| DIC |  | 1940.07 | 1937.88 |
| **Excluding RCTs with zero Events (159 RCTs)** | | | |
| Dbar | 1654.04 | 1563.66 | 1555.58 |
| Dhat | 1457.87 | 1319.3 | 1305.24 |
| pD | 196.161 | 244.36 | 250.34 |
| DIC | 1850.2 | 1808.02 | 1805.92 |
| **GeMTC R:After Excluding RCTs with zero Events (173 RCTs)** | | | |
| Dbar | 435.4910 | N/A | 336.4317 |
| I^2^ | 24% | N/A | 2% |
| pD | 199.9267 | N/A | 252.4373 |
| DIC | 635.4177 | N/A | 588.6890 |

**3. RCTs with High Baseline Renal Profile:**

| **NetMetaXL: Main Analysis (112 RCTs)** | | | |
| --- | --- | --- | --- |
|  | FE | RE Informative Prior | RE Vague Prior |
| Dbar | 1139.36 | 1073.87 | 1064.8 |
| Dhat | 995.995 | 897.224 | 881.453 |
| pD | 143.361 | 176.645 | 183.351 |
| DIC | 1282.72 | 1250.51 | 1248.15 |
| **NetMetaXL: Main Analysis After Excluding RCTs with zero Events (105 RCTs)** | | | |
| Dbar | 1088.56 | 1026.02 | 1016.49 |
| Dhat | 953.342 | 858.667 | 843.001 |
| pD | 135.217 | 167.351 | 173.487 |
| DIC | 1223.78 | 1193.37 | 1189.98 |
| **GeMTC R: After Excluding RCTs with zero Events** | | | |
| Dbar | 298.4699 | N/A | 225.5229 |
| I^2^ | 26% | N/A | 2% |
| pD | 138.7850 | N/A | 175.4850 |
| DIC | 2549 | N/A | 401.0079 |

**4. RCTs with Normal Baseline Renal Profile:**

| **NetMetaXL: Main Analysis (60 RCTs)** | | | |
| --- | --- | --- | --- |
|  | FE | RE Informative Prior | RE Vague Prior |
| Dbar | 627.844 | 598.857 | 590.462 |
| Dhat | 546.119 | 503.07 | 489.548 |
| pD | 81.725 | 95.787 | 100.915 |
| DIC | 709.57 | 694.644 | 691.377 |
| **NetMetaXL: Main Analysis After Excluding RCTs with zero Events (53 RCTs)** | | | |
| Dbar | 555.729 | 538.741 | 531.311 |
| Dhat | 482.178 | 455.817 | 442.882 |
| pD | 73.551 | 82.924 | 88.43 |
| DIC | 629.28 | 621.665 | 619.741 |
| **GeMTC R: After Excluding RCTs with zero Events ( RCTs)** | | | |
| Dbar | 133.12656 | N/A | 108.98182 |
| I^2^ | 19% | N/A | 0.9% |
| pD | 75.27316 | N/A | 90.14556 |
| DIC | 208.39972 | N/A | 199.12738 |

**GeMTC R: Gelman and Rubin's convergence diagnostic**

**1. Main Analysis after Excluding RCTs with zero Events (184 RCTs)**

**Random effect Model:**

**Potential scale reduction factors:**

|  | Point est. Upper C.I. | |
| --- | --- | --- |
| d.Control.Alpha_Lipoic_Acid | 0.999 | 1.00 |
| d.Control.Amlodipine_and_Valsartan | 1.002 | 1.01 |
| d.Control.Cordyceps | 1.002 | 1.01 |
| d.Control.Dopamine | 1.000 | 1.00 |
| d.Control.Hypothermia | 1.000 | 1.00 |
| d.Control.Iloprost | 0.999 | 1.00 |
| d.Control.Mannitol | 1.002 | 1.01 |
| d.Control.MgSO4 | 1.001 | 1.00 |
| d.Control.Oxygen | 1.002 | 1.01 |
| d.Control.Pentoxifylline | 1.000 | 1.00 |
| d.Control.Probucol | 1.002 | 1.01 |
| d.Control.Trimetazidine | 1.001 | 1.00 |
| d.NAC.Allopurinol | 1.000 | 1.00 |
| d.NAC.Ascorbic_Acid | 1.002 | 1.00 |
| d.NAC.Control | 1.001 | 1.00 |
| d.NAC.Dialysis | 1.001 | 1.00 |
| d.NAC.Fenoldopam | 1.000 | 1.00 |
| d.NAC.I_V_Hydartion | 1.000 | 1.00 |
| d.NAC.NaHCO3 | 1.000 | 1.00 |
| d.NAC.Nebivolol | 1.001 | 1.00 |
| d.NAC.Placebo | 1.001 | 1.00 |
| d.NAC.Theophylline | 0.999 | 1.00 |
| d.NAC.Zinc | 1.001 | 1.01 |
| d.NaHCO3.Acetazolamide | 1.002 | 1.00 |
| d.NaHCO3.K_Na_citrate | 1.002 | 1.00 |
| d.NaHCO3.Oral_Hydration | 1.001 | 1.00 |
| d.Placebo.ACEI | 1.001 | 1.00 |
| d.Placebo.alpha_tocopherol | 1.000 | 1.00 |

| d.Placebo.Aminophylline | 1.000 | 1.00 |
| --- | --- | --- |
| d.Placebo.Anisodamine | 1.005 | 1.02 |
| d.Placebo.ANP | 1.002 | 1.01 |
| d.Placebo.BNP | 1.000 | 1.00 |
| d.Placebo.ERAs | 1.000 | 1.00 |
| d.Placebo.gamma_tocopherol | 1.000 | 1.00 |
| d.Placebo.Glutathione | 1.000 | 1.00 |
| d.Placebo.Nicorandil | 1.000 | 1.00 |
| d.Placebo.PGE1 | 1.000 | 1.00 |
| d.Placebo.RIPC | 1.001 | 1.01 |
| d.Placebo.Silymarin_ | 1.001 | 1.00 |
| d.Placebo.Statins | 1.002 | 1.01 |
| d.Theophylline.Furosemide | 0.999 | 1.00 |
| sd.d | 1.007 | 1.02 |

**Empirical mean and standard deviation for each variable, plus standard error of the mean:**

|  | Mean | SD Naive SE Time-series SE | | |
| --- | --- | --- | --- | --- |
| d.Control.Alpha_Lipoic_Acid | -0.55793 | 0.64809 | 0.010247 | 0.010627 |
| d.Control.Amlodipine_and_Valsartan 1.21351 | | 0.90805 | 0.014358 | 0.014355 |
| d.Control.Cordyceps | -0.58010 | 0.63806 | 0.010089 | 0.010191 |
| d.Control.Dopamine | 0.19102 | 0.69128 | 0.010930 | 0.011241 |
| d.Control.Hypothermia | 0.16682 | 0.65450 | 0.010348 | 0.010350 |
| d.Control.Iloprost | -1.24015 | 0.67933 | 0.010741 | 0.010524 |
| d.Control.Mannitol | 0.20294 | 0.70384 | 0.011129 | 0.011316 |
| d.Control.MgSO4 | -0.79020 | 0.69915 | 0.011055 | 0.010703 |
| d.Control.Oxygen | -1.41012 | 0.49538 | 0.007833 | 0.007829 |
| d.Control.Pentoxifylline | -0.36506 | 0.38655 | 0.006112 | 0.006232 |
| d.Control.Probucol | -1.26403 | 0.51461 | 0.008137 | 0.008133 |
| d.Control.Trimetazidine | -1.26002 | 0.40543 | 0.006410 | 0.006329 |
| d.NAC.Allopurinol | -0.52666 | 0.72788 | 0.011509 | 0.012311 |
| d.NAC.Ascorbic_Acid | 0.02113 | 0.30332 | 0.004796 | 0.004938 |
| d.NAC.Control | 0.68781 | 0.14015 | 0.002216 | 0.002332 |
| d.NAC.Dialysis | 0.10117 | 0.35792 | 0.005659 | 0.005617 |
| d.NAC.Fenoldopam | 0.52897 | 0.32661 | 0.005164 | 0.005165 |
| d.NAC.I_V_Hydartion | 0.32736 | 0.18730 | 0.002961 | 0.003047 |
| d.NAC.NaHCO3 | 0.27502 | 0.19532 | 0.003088 | 0.003139 |
| d.NAC.Nebivolol | 0.05250 | 0.65948 | 0.010427 | 0.010423 |

| d.NAC.Placebo | 0.36872 | 0.12016 | 0.001900 | 0.002030 |
| --- | --- | --- | --- | --- |
| d.NAC.Theophylline | -0.40057 | 0.39358 | 0.006223 | 0.006477 |
| d.NAC.Zinc | 1.00422 | 1.05186 | 0.016631 | 0.018942 |
| d.NaHCO3.Acetazolamide | -0.70645 | 0.69729 | 0.011025 | 0.012333 |
| d.NaHCO3.K_Na_citrate | -1.07520 | 0.65310 | 0.010326 | 0.010448 |
| d.NaHCO3.Oral_Hydration | 0.25811 | 0.42235 | 0.006678 | 0.006727 |
| d.Placebo.ACEI | -0.67130 | 0.50884 | 0.008045 | 0.008369 |
| d.Placebo.alpha_tocopherol | -1.16724 | 0.42119 | 0.006660 | 0.006606 |
| d.Placebo.Aminophylline | -0.53045 | 0.88798 | 0.014040 | 0.014534 |
| d.Placebo.Anisodamine | -0.82673 | 0.49550 | 0.007835 | 0.008133 |
| d.Placebo.ANP | -0.03726 | 0.47580 | 0.007523 | 0.007390 |
| d.Placebo.BNP | -0.86020 | 0.35333 | 0.005587 | 0.005587 |
| d.Placebo.ERAs | 1.11509 | 0.60674 | 0.009593 | 0.009481 |
| d.Placebo.gamma_tocopherol | -1.04950 | 0.70075 | 0.011080 | 0.010763 |
| d.Placebo.Glutathione | 0.03807 | 0.59622 | 0.009427 | 0.009793 |
| d.Placebo.Nicorandil | -0.79661 | 0.46459 | 0.007346 | 0.007470 |
| d.Placebo.PGE1 | -1.18863 | 0.39778 | 0.006289 | 0.006277 |
| d.Placebo.RIPC | -0.58417 | 0.29643 | 0.004687 | 0.004683 |
| d.Placebo.Silymarin_ | -1.58526 | 1.02831 | 0.016259 | 0.016621 |
| d.Placebo.Statins | -0.52345 | 0.21980 | 0.003475 | 0.003550 |
| d.Theophylline.Furosemide | 0.56257 | 0.49085 | 0.007761 | 0.007209 |
| sd.d | 0.50197 | 0.06842 | 0.001082 | 0.001438 |

| **Quantiles for each variable**: |  |  |  |  |  |
| --- | --- | --- | --- | --- | --- |
|  | 2.5% | 25% | 50% | 75% | 97.5% |
| d.Control.Alpha_Lipoic_Acid | -1.88029 | -0.97636 | -0.54805 | -0.12630 | 0.70790 |

d.Control.Amlodipine_and_Valsartan -0.50098 0.60060 1.19120 1.77897 3.04104

| d.Control.Cordyceps | -1.88043 | -1.00845 | -0.56449 | -0.13443 | 0.63025 |
| --- | --- | --- | --- | --- | --- |
| d.Control.Dopamine | -1.17223 | -0.26934 | 0.18233 | 0.67156 | 1.54353 |
| d.Control.Hypothermia | -1.11929 | -0.26748 | 0.15379 | 0.61252 | 1.45757 |
| d.Control.Iloprost | -2.57479 | -1.70336 | -1.25110 | -0.78476 | 0.05362 |

| d.Control.Mannitol | -1.17202 | -0.25940 | 0.20995 | 0.66960 | 1.56108 |
| --- | --- | --- | --- | --- | --- |
| d.Control.MgSO4 | -2.15851 | -1.25627 -0.78113 | | -0.30993 | 0.56123 |
| d.Control.Oxygen | -2.39571 | -1.73036 -1.40055 | | -1.07172 -0.42870 | |
| d.Control.Pentoxifylline | -1.12457 | -0.62431 -0.36402 | | -0.10899 | 0.38851 |
| d.Control.Probucol | -2.29885 | -1.60196 -1.25296 | | -0.92466 -0.24867 | |
| d.Control.Trimetazidine | -2.06654 | -1.52865 -1.25013 | | -0.98316 -0.48913 | |
| d.NAC.Allopurinol | -1.98522 | -1.00926 -0.50900 | | -0.03012 | 0.88578 |
| d.NAC.Ascorbic_Acid | -0.55840 | -0.18878 | 0.01921 | 0.22527 | 0.63195 |
| d.NAC.Control | 0.40785 | 0.59465 | 0.68837 | 0.78256 | 0.95957 |
| d.NAC.Dialysis | -0.60199 | -0.14614 | 0.10346 | 0.34457 | 0.81189 |
| d.NAC.Fenoldopam | -0.11097 | 0.31426 | 0.53097 | 0.74955 | 1.16930 |
| d.NAC.I_V_Hydartion | -0.04103 | 0.19923 | 0.32561 | 0.45808 | 0.69124 |
| d.NAC.NaHCO3 | -0.11111 | 0.14492 | 0.27639 | 0.40497 | 0.65677 |
| d.NAC.Nebivolol | -1.20328 | -0.40461 | 0.04544 | 0.50613 | 1.34592 |
| d.NAC.Placebo | 0.13421 | 0.28689 | 0.36717 | 0.44725 | 0.60954 |
| d.NAC.Theophylline | -1.19731 | -0.65540 -0.40088 | | -0.13214 | 0.34997 |
| d.NAC.Zinc | -1.07178 | 0.32881 | 0.99010 | 1.69198 | 3.05803 |
| d.NaHCO3.Acetazolamide | -2.12377 | -1.15909 -0.69444 | | -0.25178 | 0.63101 |
| d.NaHCO3.K_Na_citrate | -2.40866 | -1.49561 -1.06716 | | -0.62879 | 0.17105 |
| d.NaHCO3.Oral_Hydration | -0.57290 | -0.03113 | 0.25312 | 0.55554 | 1.08111 |
| d.Placebo.ACEI | -1.69757 | -1.01711 -0.66073 | | -0.33256 | 0.28710 |
| d.Placebo.alpha_tocopherol | -2.00661 | -1.44628 -1.16020 | | -0.88374 -0.35807 | |
| d.Placebo.Aminophylline | -2.25600 | -1.13910 -0.51865 | | 0.09454 | 1.18960 |
| d.Placebo.Anisodamine | -1.79080 | -1.16765 -0.83365 | | -0.49389 | 0.12953 |
| d.Placebo.ANP | -0.98492 | -0.35903 -0.02472 | | 0.28278 | 0.88345 |
| d.Placebo.BNP | -1.54189 | -1.09391 -0.86287 | | -0.63012 -0.14252 | |
| d.Placebo.ERAs | -0.03704 | 0.70232 | 1.11856 | 1.51449 | 2.29228 |
| d.Placebo.gamma_tocopherol | -2.44768 | -1.53100 -1.03624 | | -0.57905 | 0.29659 |
| d.Placebo.Glutathione | -1.11372 | -0.35895 | 0.04036 | 0.43424 | 1.22442 |
| d.Placebo.Nicorandil | -1.71567 | -1.11075 -0.78967 | | -0.48575 | 0.10764 |
| d.Placebo.PGE1 | -1.94602 | -1.45611 -1.19410 | | -0.92335 -0.38811 | |
| d.Placebo.RIPC | -1.16103 | -0.78495 -0.58720 | | -0.38654 -0.01246 | |

| d.Placebo.Silymarin_ | | | -3.75797 | -2.22412 | -1.53087 | -0.88742 | 0.32286 |
| --- | --- | --- | --- | --- | --- | --- | --- |
| d.Placebo.Statins | |  | -0.96481 | -0.66952 | -0.51915 | -0.37471 | -0.10612 |
| d.Theophylline.Furosemide | | | -0.41169 | 0.24010 | 0.54729 | 0.88770 | 1.54929 |
| sd.d |  |  | 0.37211 | 0.45674 | 0.49967 | 0.54596 | 0.64393 |

**Fixed effect Model:**

**Potential scale reduction factors**

|  | | Point est. Upper C.I. | | |
| --- | --- | --- | --- | --- |
| d.Control.Alpha_Lipoic_Acid | | 1.001 | | 1.01 |
| d.Control.Amlodipine_and_Valsartan | | 1.000 | | 1.00 |
| d.Control.Cordyceps | | 1.000 | | 1.00 |
| d.Control.Dopamine | | 0.999 | | 1.00 |
| d.Control.Hypothermia | | 1.001 | | 1.00 |
| d.Control.Iloprost | | 1.002 | | 1.00 |
| d.Control.Mannitol | | 1.000 | | 1.00 |
| d.Control.MgSO4 | | 1.000 | | 1.00 |
| d.Control.Oxygen | | 1.001 | | 1.00 |
| d.Control.Pentoxifylline | | 1.000 | | 1.00 |
| d.Control.Probucol | | 1.001 | | 1.00 |
| d.Control.Trimetazidine | | 1.000 | | 1.00 |
| d.NAC.Allopurinol | | 1.000 | | 1.00 |
| d.NAC.Ascorbic_Acid | | 1.001 | | 1.00 |
| d.NAC.Control | | 1.000 | | 1.00 |
| d.NAC.Dialysis | | 1.000 | | 1.00 |
| d.NAC.Fenoldopam | | 1.000 | | 1.00 |
| d.NAC.I_V_Hydartion | | 1.000 | | 1.00 |
| d.NAC.NaHCO3 | | 1.002 | | 1.00 |
| d.NAC.Nebivolol | | 1.000 | | 1.00 |
| d.NAC.Placebo | | 1.000 | | 1.00 |
| d.NAC.Theophylline | | 1.002 | | 1.01 |
| d.NAC.Zinc | | 1.001 | | 1.01 |
| d.NaHCO3.Acetazolamide | | 1.001 | | 1.00 |
| d.NaHCO3.K_Na_citrate | | 1.003 | | 1.01 |
| d.NaHCO3.Oral_Hydration | | 1.002 | | 1.01 |
| d.Placebo.ACEI | | 1.001 | | 1.00 |
| d.Placebo.alpha_tocopherol | | 0.999 | | 1.00 |
| d.Placebo.Aminophylline | 1.000 | | 1.00 | |
| d.Placebo.Anisodamine | 1.000 | | 1.00 | |
| d.Placebo.ANP | 1.002 | | 1.01 | |
| d.Placebo.BNP | 1.001 | | 1.00 | |
| d.Placebo.ERAs | 1.000 | | 1.00 | |
| d.Placebo.gamma_tocopherol | 1.001 | | 1.00 | |
| d.Placebo.Glutathione | 1.001 | | 1.00 | |
| d.Placebo.Nicorandil | 1.000 | | 1.00 | |
| d.Placebo.PGE1 | 1.002 | | 1.01 | |
| d.Placebo.RIPC | 1.000 | | 1.00 | |
| d.Placebo.Silymarin_ | 1.000 | | 1.00 | |
| d.Placebo.Statins | 1.001 | | 1.00 | |
| d.Theophylline.Furosemide | 1.001 | | 1.00 | |

**Empirical mean and standard deviation for each variable, plus standard error of the mean:**

|  | Mean | SD Naive SE Time-series SE | | |
| --- | --- | --- | --- | --- |
| d.Control.Alpha_Lipoic_Acid | -0.58029 | 0.54375 | 0.008597 | 0.008743 |
| d.Control.Amlodipine_and_Valsartan 1.22724 | | 0.75967 | 0.012011 | 0.012013 |
| d.Control.Cordyceps | -0.59123 | 0.52648 | 0.008324 | 0.008327 |
| d.Control.Dopamine | 0.16293 | 0.58444 | 0.009241 | 0.009127 |
| d.Control.Hypothermia | 0.14596 | 0.42550 | 0.006728 | 0.006397 |
| d.Control.Iloprost | -1.24535 | 0.45341 | 0.007169 | 0.007170 |
| d.Control.Mannitol | 0.24735 | 0.59941 | 0.009478 | 0.009851 |
| d.Control.MgSO4 | -0.79459 | 0.47291 | 0.007477 | 0.007363 |
| d.Control.Oxygen | -1.22556 | 0.24044 | 0.003802 | 0.003657 |
| d.Control.Pentoxifylline | -0.40179 | 0.26418 | 0.004177 | 0.004075 |
| d.Control.Probucol | -1.26506 | 0.35192 | 0.005564 | 0.005566 |
| d.Control.Trimetazidine | -1.26259 | 0.28860 | 0.004563 | 0.004563 |
| d.NAC.Allopurinol | -0.59589 | 0.57676 | 0.009119 | 0.009241 |
| d.NAC.Ascorbic_Acid | -0.16296 | 0.19011 | 0.003006 | 0.003004 |
| d.NAC.Control | 0.66807 | 0.09309 | 0.001472 | 0.001579 |
| d.NAC.Dialysis | 0.01940 | 0.24274 | 0.003838 | 0.003883 |
| d.NAC.Fenoldopam | 0.45552 | 0.19916 | 0.003149 | 0.003024 |
| d.NAC.I_V_Hydartion | 0.30745 | 0.11366 | 0.001797 | 0.001962 |
| d.NAC.NaHCO3 | 0.32343 | 0.11939 | 0.001888 | 0.002054 |
| d.NAC.Nebivolol | 0.04827 | 0.48023 | 0.007593 | 0.007639 |

| d.NAC.Placebo | 0.22330 | 0.06595 | 0.001043 | 0.001101 |
| --- | --- | --- | --- | --- |
| d.NAC.Theophylline | -0.46085 | 0.30819 | 0.004873 | 0.005586 |
| d.NAC.Zinc | 0.91698 | 0.93971 | 0.014858 | 0.014606 |
| d.NaHCO3.Acetazolamide | -0.81395 | 0.53694 | 0.008490 | 0.008990 |
| d.NaHCO3.K_Na_citrate | -1.24161 | 0.49724 | 0.007862 | 0.007941 |
| d.NaHCO3.Oral_Hydration | 0.19610 | 0.33039 | 0.005224 | 0.005151 |
| d.Placebo.ACEI | -0.60898 | 0.42489 | 0.006718 | 0.006718 |
| d.Placebo.alpha_tocopherol | -1.11426 | 0.29531 | 0.004669 | 0.004750 |
| d.Placebo.Aminophylline | -0.55114 | 0.72683 | 0.011492 | 0.011312 |
| d.Placebo.Anisodamine | -0.66682 | 0.32081 | 0.005072 | 0.004989 |
| d.Placebo.ANP | -0.03942 | 0.32278 | 0.005104 | 0.005098 |
| d.Placebo.BNP | -0.85260 | 0.20378 | 0.003222 | 0.003332 |
| d.Placebo.ERAs | 1.11316 | 0.33250 | 0.005257 | 0.005257 |
| d.Placebo.gamma_tocopherol | -1.04021 | 0.50451 | 0.007977 | 0.007895 |
| d.Placebo.Glutathione | 0.04261 | 0.32835 | 0.005192 | 0.005192 |
| d.Placebo.Nicorandil | -0.80789 | 0.32785 | 0.005184 | 0.005217 |
| d.Placebo.PGE1 | -1.13790 | 0.27207 | 0.004302 | 0.004219 |
| d.Placebo.RIPC | -0.52148 | 0.22588 | 0.003571 | 0.003487 |
| d.Placebo.Silymarin_ | -1.59084 | 0.89176 | 0.014100 | 0.013955 |
| d.Placebo.Statins | -0.30367 | 0.13220 | 0.002090 | 0.002090 |
| d.Theophylline.Furosemide | 0.50865 | 0.34950 | 0.005526 | 0.006053 |

| **Quantiles for each variable:** |  |  |  |  |  |
| --- | --- | --- | --- | --- | --- |
|  | 2.5% | 25% | 50% | 75% | 97.5% |
| d.Control.Alpha_Lipoic_Acid | -1.67477 | -0.93879 | -0.57236 | -0.21501 | 0.46555 |

d.Control.Amlodipine_and_Valsartan -0.16799 0.70495 1.19558 1.68624 2.82890

| d.Control.Cordyceps | -1.65240 -0.93760 | | -0.58553 | -0.23028 | 0.40047 |
| --- | --- | --- | --- | --- | --- |
| d.Control.Dopamine | -1.01400 | -0.21784 | 0.16306 | 0.55577 | 1.31365 |
| d.Control.Hypothermia | -0.70187 | -0.13735 | 0.14710 | 0.43448 | 0.97632 |
| d.Control.Iloprost | -2.16302 | -1.53182 | -1.23568 | -0.93109 -0.38673 | |
| d.Control.Mannitol | -0.95727 | -0.15421 | 0.25321 | 0.64708 | 1.40129 |

| d.Control.MgSO4 | | -1.71966 | -1.11095 -0.79101 | | | -0.47502 | | 0.14440 |
| --- | --- | --- | --- | --- | --- | --- | --- | --- |
| d.Control.Oxygen | | -1.68514 | -1.39022 -1.21913 | | | -1.05994 -0.77477 | | |
| d.Control.Pentoxifylline | | -0.93918 | -0.58053 -0.39553 | | | -0.22514 | | 0.09506 |
| d.Control.Probucol | | -1.98476 | -1.49845 -1.25551 | | | -1.02554 -0.60160 | | |
| d.Control.Trimetazidine | | -1.83384 | -1.46139 -1.25870 | | | -1.06064 -0.71570 | | |
| d.NAC.Allopurinol | | -1.81269 | -0.96794 -0.57977 | | | -0.20079 | | 0.45872 |
| d.NAC.Ascorbic_Acid | | -0.53208 | -0.29233 -0.16193 | | | -0.03565 | | 0.21610 |
| d.NAC.Control | | 0.48640 | 0.60364 | | 0.66971 | 0.73104 | | 0.85032 |
| d.NAC.Dialysis | | -0.47269 | -0.14030 | | 0.02154 | 0.17755 | | 0.48599 |
| d.NAC.Fenoldopam | | 0.06187 | 0.32301 | | 0.45504 | 0.59130 | | 0.84202 |
| d.NAC.I_V_Hydartion | | 0.08577 | 0.23267 | | 0.30606 | 0.38284 | | 0.53593 |
| d.NAC.NaHCO3 | | 0.08781 | 0.24353 | | 0.32211 | 0.40512 | | 0.56453 |
| d.NAC.Nebivolol | | -0.90939 | -0.26714 | | 0.06093 | 0.36932 | | 0.98086 |
| d.NAC.Placebo | | 0.09633 | 0.17990 | | 0.22236 | 0.26715 | | 0.35172 |
| d.NAC.Theophylline | | -1.07503 | -0.66426 -0.45809 | | | -0.24621 | | 0.13328 |
| d.NAC.Zinc | | -0.89747 | 0.26974 | | 0.91857 | 1.54127 | | 2.73440 |
| d.NaHCO3.Acetazolamide | | -1.95550 | -1.15358 -0.79366 | | | -0.44672 | | 0.18846 |
| d.NaHCO3.K_Na_citrate | | -2.29258 | -1.56079 -1.21631 | | | -0.89985 -0.32258 | | |
| d.NaHCO3.Oral_Hydration | | -0.45724 | -0.02669 | | 0.19531 | 0.41828 | | 0.85003 |
| d.Placebo.ACEI | | -1.48609 | -0.89185 -0.60153 | | | -0.32437 | | 0.20025 |
| d.Placebo.alpha_tocopherol | | -1.71931 | -1.31549 -1.10831 | | | -0.91429 -0.54704 | | |
| d.Placebo.Aminophylline | | -2.06390 | -1.00352 -0.54294 | | | -0.06460 | | 0.85551 |
| d.Placebo.Anisodamine | | -1.30804 | -0.88333 -0.66488 | | | -0.44494 -0.05564 | | |
| d.Placebo.ANP | | -0.65725 | -0.25666 -0.03887 | | | 0.18097 | | 0.59008 |
| d.Placebo.BNP | | -1.25365 | -0.99136 -0.84907 | | | -0.71248 -0.46330 | | |
| d.Placebo.ERAs | | 0.47731 | 0.89003 | | 1.11343 | 1.34087 | | 1.76148 |
| d.Placebo.gamma_tocopherol | | -2.07149 | -1.36218 -1.02685 | | | -0.69694 -0.08725 | | |
| d.Placebo.Glutathione | | -0.58992 | -0.17482 | | 0.04074 | 0.25551 | | 0.70825 |
| d.Placebo.Nicorandil | | -1.46312 | -1.02990 -0.79968 | | | -0.58431 -0.17129 | | |
| d.Placebo.PGE1 | | -1.69518 | -1.31456 -1.12913 | | | -0.95391 -0.61738 | | |
| d.Placebo.RIPC | | -0.94978 | -0.67516 -0.52251 | | | -0.36880 -0.08394 | | |
| d.Placebo.Silymarin_ | | -3.49752 | -2.14432 -1.53296 | | | -0.96278 -0.02815 | | |
| d.Placebo.Statins | -0.56775 | | -0.39016 | -0.30452 | | -0.21312 | -0.04578 | |
| d.Theophylline.Furosemide | -0.16023 | | 0.26716 | 0.50896 | | 0.73795 | 1.20879 | |

**2. Excluding RCTs with Partial Use of Hyperosmolar Contrast media (173 RCTs):**

**Random effect Model:**

**Potential scale reduction factors**

|  | Point est. Upper C.I. | |
| --- | --- | --- |
| d.Control.Alpha_Lipoic_Acid | 1 | 1.00 |
| d.Control.Amlodipine_and_Valsartan | 1 | 1.00 |
| d.Control.ANP | 1 | 1.00 |
| d.Control.Cordyceps | 1 | 1.00 |
| d.Control.Dopamine | 1 | 1.01 |
| d.Control.Hypothermia | 1 | 1.01 |
| d.Control.MgSO4 | 1 | 1.00 |
| d.Control.Oxygen | 1 | 1.00 |
| d.Control.Pentoxifylline | 1 | 1.00 |
| d.Control.Probucol | 1 | 1.00 |
| d.Control.Trimetazidine | 1 | 1.00 |
| d.NAC.Allopurinol | 1 | 1.01 |
| d.NAC.Ascorbic_Acid | 1 | 1.01 |
| d.NAC.Control | 1 | 1.00 |
| d.NAC.Dialysis | 1 | 1.00 |
| d.NAC.Fenoldopam | 1 | 1.00 |
| d.NAC.I_V_Hydartion | 1 | 1.01 |
| d.NAC.NaHCO3 | 1 | 1.01 |
| d.NAC.Nebivolol | 1 | 1.00 |
| d.NAC.Placebo | 1 | 1.00 |
| d.NAC.Theophylline | 1 | 1.00 |
| d.NAC.Zinc | 1 | 1.00 |
| d.NaHCO3.Acetazolamide | 1 | 1.01 |
| d.NaHCO3.K_Na_citrate | 1 | 1.00 |
| d.NaHCO3.Oral_Hydration | 1 | 1.01 |
| d.Placebo.ACEI | 1 | 1.00 |
| d.Placebo.alpha_tocopherol | 1 | 1.01 |
| d.Placebo.Aminophylline | 1 | 1.00 |
| d.Placebo.Anisodamine | 1 | 1.00 |
| d.Placebo.BNP | 1 | 1.01 |
| d.Placebo.ERAs | 1 | 1.00 |
| d.Placebo.Glutathione | 1 | 1.00 |
| d.Placebo.Nicorandil | 1 | 1.00 |
| d.Placebo.PGE1 | 1 | 1.00 |
| d.Placebo.RIPC | 1 | 1.01 |
| d.Placebo.Silymarin | 1 | 1.00 |
| d.Placebo.Statins | 1 | 1.01 |
| d.Theophylline.Furosemide | 1 | 1.00 |
| sd.d | 1 | 1.00 |

**Empirical mean and standard deviation for each variable, plus standard error of the mean:**

|  | | Mean | SD Naive SE Time-series SE | | |
| --- | --- | --- | --- | --- | --- |
| d.Control.Alpha_Lipoic_Acid | | -0.53256 | 0.6522 | 0.010312 | 0.010450 |
| d.Control.Amlodipine_and_Valsartan 1.26297 | | | 0.9079 | 0.014354 | 0.015648 |
| d.Control.ANP | | -1.49899 | 0.7646 | 0.012090 | 0.012963 |
| d.Control.Cordyceps | | -0.58145 | 0.6162 | 0.009743 | 0.009775 |
| d.Control.Dopamine | | 0.90783 | 1.1103 | 0.017555 | 0.021921 |
| d.Control.Hypothermia | | 0.16118 | 0.6466 | 0.010223 | 0.009943 |
| d.Control.MgSO4 | | -0.79684 | 0.6786 | 0.010730 | 0.010583 |
| d.Control.Oxygen | | -1.38444 | 0.4772 | 0.007546 | 0.007568 |
| d.Control.Pentoxifylline | | -0.38018 | 0.3713 | 0.005871 | 0.005999 |
| d.Control.Probucol | | -1.26323 | 0.4870 | 0.007699 | 0.008063 |
| d.Control.Trimetazidine | | -1.26017 | 0.3895 | 0.006158 | 0.006346 |
| d.NAC.Allopurinol | | -0.52290 | 0.7254 | 0.011470 | 0.012842 |
| d.NAC.Ascorbic_Acid | | 0.19182 | 0.3259 | 0.005153 | 0.005566 |
| d.NAC.Control | | 0.71017 | 0.1523 | 0.002409 | 0.002613 |
| d.NAC.Dialysis | | 0.12011 | 0.3437 | 0.005435 | 0.005457 |
| d.NAC.Fenoldopam | | 0.55337 | 0.3141 | 0.004967 | 0.004894 |
| d.NAC.I_V_Hydartion | | 0.32033 | 0.2063 | 0.003262 | 0.003543 |
| d.NAC.NaHCO3 | | 0.15966 | 0.2196 | 0.003472 | 0.003728 |
| d.NAC.Nebivolol | | 0.06817 | 0.6416 | 0.010144 | 0.009976 |
| d.NAC.Placebo | | 0.40331 | 0.1279 | 0.002023 | 0.002104 |
| d.NAC.Theophylline | -0.35623 | | 0.3931 | 0.006215 | 0.006297 |
| d.NAC.Zinc | 1.01876 | | 1.0434 | 0.016498 | 0.018792 |
| d.NaHCO3.Acetazolamide | -0.65304 | | 0.7036 | 0.011124 | 0.012000 |
| d.NaHCO3.K_Na_citrate | -0.98054 | | 0.6569 | 0.010386 | 0.010936 |
| d.NaHCO3.Oral_Hydration | -0.16715 | | 0.4788 | 0.007571 | 0.007965 |
| d.Placebo.ACEI | -0.66956 | | 0.9134 | 0.014442 | 0.015728 |
| d.Placebo.alpha_tocopherol | -0.84340 | | 0.6419 | 0.010149 | 0.010428 |
| d.Placebo.Aminophylline | -0.53532 | | 0.8869 | 0.014023 | 0.014616 |
| d.Placebo.Anisodamine | -0.84700 | | 0.4802 | 0.007593 | 0.007890 |
| d.Placebo.BNP | -0.87836 | | 0.3597 | 0.005688 | 0.005600 |
| d.Placebo.ERAs | 1.11450 | | 0.5881 | 0.009299 | 0.009301 |
| d.Placebo.Glutathione | 0.04575 | | 0.5767 | 0.009119 | 0.009433 |
| d.Placebo.Nicorandil | -0.81074 | | 0.4489 | 0.007097 | 0.007099 |
| d.Placebo.PGE1 | -1.21161 | | 0.3909 | 0.006180 | 0.006181 |
| d.Placebo.RIPC | -0.64583 | | 0.3124 | 0.004940 | 0.005122 |
| d.Placebo.Silymarin | -1.60447 | | 0.9923 | 0.015689 | 0.016980 |
| d.Placebo.Statins | -0.52241 | | 0.2157 | 0.003411 | 0.003470 |
| d.Theophylline.Furosemide | 0.70143 | | 0.5614 | 0.008877 | 0.008980 |
| sd.d | 0.47333 | | 0.0759 | 0.001200 | 0.001648 |

| **Quantiles for each variable:** |  |  |  |  |  |
| --- | --- | --- | --- | --- | --- |
|  | 2.5% | 25% | 50% | 75% | 97.5% |
| d.Control.Alpha_Lipoic_Acid | -1.85840 | -0.95558 | -0.53910 | -0.09477 | 0.72155 |

d.Control.Amlodipine_and_Valsartan -0.44426 0.64350 1.24729 1.84700 3.10184

| d.Control.ANP | -3.07120 -1.97356 | | -1.47145 | -0.99293 -0.03715 | |
| --- | --- | --- | --- | --- | --- |
| d.Control.Cordyceps | -1.82417 -0.98797 | | -0.57308 | -0.18174 | 0.62449 |
| d.Control.Dopamine | -1.17431 | 0.12987 | 0.86908 | 1.62369 | 3.19206 |
| d.Control.Hypothermia | -1.11382 -0.26348 | | 0.17155 | 0.59697 | 1.41101 |
| d.Control.MgSO4 | -2.16962 | -1.23087 | -0.77508 | -0.34820 | 0.50016 |
| d.Control.Oxygen | -2.34385 | -1.69604 | -1.38055 | -1.07051 -0.46911 | |
| d.Control.Pentoxifylline | -1.10543 | -0.62586 | -0.38167 | -0.13147 | 0.36634 |

| d.Control.Probucol | -2.20639 | -1.59422 -1.26414 | | -0.95451 -0.30411 | |
| --- | --- | --- | --- | --- | --- |
| d.Control.Trimetazidine | -2.06076 | -1.50662 -1.25065 | | -0.99743 -0.50860 | |
| d.NAC.Allopurinol | -2.00449 | -1.00408 -0.51062 | | -0.03240 | 0.88411 |
| d.NAC.Ascorbic_Acid | -0.44823 | -0.02635 | 0.19263 | 0.40764 | 0.82780 |
| d.NAC.Control | 0.40873 | 0.60857 | 0.70800 | 0.81669 | 1.00708 |
| d.NAC.Dialysis | -0.53633 | -0.10777 | 0.11919 | 0.34900 | 0.80245 |
| d.NAC.Fenoldopam | -0.05919 | 0.33763 | 0.55291 | 0.77085 | 1.16651 |
| d.NAC.I_V_Hydartion | -0.09286 | 0.18264 | 0.32089 | 0.45504 | 0.73148 |
| d.NAC.NaHCO3 | -0.26967 | 0.01024 | 0.15862 | 0.30712 | 0.58697 |
| d.NAC.Nebivolol | -1.18882 | -0.35652 | 0.06660 | 0.50783 | 1.30672 |
| d.NAC.Placebo | 0.16292 | 0.31895 | 0.40109 | 0.48884 | 0.65673 |
| d.NAC.Theophylline | -1.14674 | -0.61481 -0.34778 | | -0.09667 | 0.40665 |
| d.NAC.Zinc | -1.04200 | 0.34319 | 1.02914 | 1.71526 | 3.06242 |
| d.NaHCO3.Acetazolamide | -2.11085 | -1.12344 -0.63522 | | -0.18574 | 0.66732 |
| d.NaHCO3.K_Na_citrate | -2.29044 | -1.41580 -0.96725 | | -0.52991 | 0.27755 |
| d.NaHCO3.Oral_Hydration | -1.11906 | -0.48280 -0.16744 | | 0.15671 | 0.76278 |
| d.Placebo.ACEI | -2.55755 | -1.27311 -0.63916 | | -0.04797 | 1.06865 |
| d.Placebo.alpha_tocopherol | -2.11548 | -1.25111 -0.83789 | | -0.42953 | 0.40284 |
| d.Placebo.Aminophylline | -2.27316 | -1.12362 -0.53302 | | 0.04651 | 1.20641 |
| d.Placebo.Anisodamine | -1.82635 | -1.15209 -0.84781 | | -0.52940 | 0.07213 |
| d.Placebo.BNP | -1.58958 | -1.11581 -0.87675 | | -0.64104 -0.17507 | |
| d.Placebo.ERAs | -0.05794 | 0.72100 | 1.11813 | 1.50944 | 2.24773 |
| d.Placebo.Glutathione | -1.11085 | -0.33256 | 0.05399 | 0.42464 | 1.20089 |
| d.Placebo.Nicorandil | -1.69774 | -1.11063 -0.81027 | | -0.51001 | 0.06682 |
| d.Placebo.PGE1 | -2.01491 | -1.46787 -1.20515 | | -0.94703 -0.47446 | |
| d.Placebo.RIPC | -1.24107 | -0.85711 -0.64554 | | -0.43197 -0.05119 | |
| d.Placebo.Silymarin | -3.76926 | -2.20649 -1.55457 | | -0.92738 | 0.14540 |
| d.Placebo.Statins | -0.94973 | -0.66853 -0.51882 | | -0.37308 -0.12257 | |
| d.Theophylline.Furosemide | -0.40883 | 0.31981 | 0.69681 | 1.08187 | 1.81624 |
| sd.d | 0.32828 | 0.42154 | 0.47179 | 0.52443 | 0.62709 |

**Fixed effect Model:**

**Potential scale reduction factors:**

|  | Point est. Upper C.I. | |
| --- | --- | --- |
| d.Control.Alpha_Lipoic_Acid | 1.002 | 1.007 |
| d.Control.Amlodipine_and_Valsartan | 1.001 | 1.004 |
| d.Control.ANP | 1.004 | 1.012 |
| d.Control.Cordyceps | 1.001 | 1.003 |
| d.Control.Dopamine | 1.000 | 1.001 |
| d.Control.Hypothermia | 1.001 | 1.002 |
| d.Control.MgSO4 | 0.999 | 0.999 |
| d.Control.Oxygen | 1.000 | 1.001 |
| d.Control.Pentoxifylline | 1.000 | 1.002 |
| d.Control.Probucol | 1.000 | 1.001 |
| d.Control.Trimetazidine | 1.000 | 1.001 |
| d.NAC.Allopurinol | 1.001 | 1.003 |
| d.NAC.Ascorbic_Acid | 1.001 | 1.003 |
| d.NAC.Control | 1.002 | 1.007 |
| d.NAC.Dialysis | 1.000 | 1.002 |
| d.NAC.Fenoldopam | 1.000 | 1.000 |
| d.NAC.I_V_Hydartion | 1.004 | 1.013 |
| d.NAC.NaHCO3 | 1.004 | 1.012 |
| d.NAC.Nebivolol | 1.000 | 1.001 |
| d.NAC.Placebo | 0.999 | 1.000 |
| d.NAC.Theophylline | 1.000 | 1.001 |
| d.NAC.Zinc | 1.001 | 1.002 |
| d.NaHCO3.Acetazolamide | 1.002 | 1.009 |
| d.NaHCO3.K_Na_citrate | 1.001 | 1.004 |
| d.NaHCO3.Oral_Hydration | 1.000 | 1.002 |
| d.Placebo.ACEI | 1.001 | 1.003 |
| d.Placebo.alpha_tocopherol | 1.000 | 1.001 |
| d.Placebo.Aminophylline | 1.000 | 1.001 |

| d.Placebo.Anisodamine | 1.001 | 1.003 |
| --- | --- | --- |
| d.Placebo.BNP | 1.001 | 1.002 |
| d.Placebo.ERAs | 1.002 | 1.006 |
| d.Placebo.Glutathione | 1.001 | 1.002 |
| d.Placebo.Nicorandil | 1.000 | 1.002 |
| d.Placebo.PGE1 | 1.000 | 1.002 |
| d.Placebo.RIPC | 1.000 | 1.001 |
| d.Placebo.Silymarin | 1.001 | 1.005 |
| d.Placebo.Statins | 1.001 | 1.006 |
| d.Theophylline.Furosemide | 1.000 | 1.001 |

**Empirical mean and standard deviation for each variable, plus standard error of the mean:**

|  | Mean | SD Naive SE Time-series SE | | |
| --- | --- | --- | --- | --- |
| d.Control.Alpha_Lipoic_Acid | -0.56680 | 0.54780 | 0.008662 | 0.008672 |
| d.Control.Amlodipine_and_Valsartan 1.24243 | | 0.76053 | 0.012025 | 0.012090 |
| d.Control.ANP | -1.48070 | 0.60806 | 0.009614 | 0.010177 |
| d.Control.Cordyceps | -0.58595 | 0.51820 | 0.008193 | 0.008443 |
| d.Control.Dopamine | 0.89238 | 0.98656 | 0.015599 | 0.015502 |
| d.Control.Hypothermia | 0.15486 | 0.42122 | 0.006660 | 0.006786 |
| d.Control.MgSO4 | -0.78566 | 0.45850 | 0.007250 | 0.007252 |
| d.Control.Oxygen | -1.22694 | 0.23594 | 0.003731 | 0.003656 |
| d.Control.Pentoxifylline | -0.40522 | 0.25985 | 0.004109 | 0.004162 |
| d.Control.Probucol | -1.25345 | 0.35989 | 0.005690 | 0.005692 |
| d.Control.Trimetazidine | -1.25828 | 0.28845 | 0.004561 | 0.004529 |
| d.NAC.Allopurinol | -0.55819 | 0.58319 | 0.009221 | 0.009810 |
| d.NAC.Ascorbic_Acid | 0.04828 | 0.21227 | 0.003356 | 0.003356 |
| d.NAC.Control | 0.70448 | 0.10647 | 0.001683 | 0.001994 |
| d.NAC.Dialysis | 0.04823 | 0.25055 | 0.003962 | 0.004019 |
| d.NAC.Fenoldopam | 0.51135 | 0.19494 | 0.003082 | 0.003119 |
| d.NAC.I_V_Hydartion | 0.27363 | 0.13368 | 0.002114 | 0.002554 |
| d.NAC.NaHCO3 | 0.12727 | 0.14748 | 0.002332 | 0.002894 |
| d.NAC.Nebivolol | 0.07413 | 0.49311 | 0.007797 | 0.007703 |
| d.NAC.Placebo | 0.31523 | 0.08234 | 0.001302 | 0.001335 |

| d.NAC.Theophylline | -0.38319 | 0.31926 | 0.005048 | 0.004989 |
| --- | --- | --- | --- | --- |
| d.NAC.Zinc | 0.99253 | 0.94015 | 0.014865 | 0.014866 |
| d.NaHCO3.Acetazolamide | -0.72202 | 0.52731 | 0.008338 | 0.009033 |
| d.NaHCO3.K_Na_citrate | -1.05257 | 0.51103 | 0.008080 | 0.007985 |
| d.NaHCO3.Oral_Hydration | -0.14387 | 0.37683 | 0.005958 | 0.005756 |
| d.Placebo.ACEI | -0.63410 | 0.76534 | 0.012101 | 0.012263 |
| d.Placebo.alpha_tocopherol | -0.84945 | 0.41023 | 0.006486 | 0.006488 |
| d.Placebo.Aminophylline | -0.51074 | 0.73844 | 0.011676 | 0.011675 |
| d.Placebo.Anisodamine | -0.70406 | 0.32020 | 0.005063 | 0.004886 |
| d.Placebo.BNP | -0.94190 | 0.20942 | 0.003311 | 0.003700 |
| d.Placebo.ERAs | 1.11274 | 0.33049 | 0.005226 | 0.005224 |
| d.Placebo.Glutathione | 0.05646 | 0.32431 | 0.005128 | 0.004767 |
| d.Placebo.Nicorandil | -0.81299 | 0.33163 | 0.005243 | 0.005245 |
| d.Placebo.PGE1 | -1.14546 | 0.26984 | 0.004267 | 0.004072 |
| d.Placebo.RIPC | -0.62622 | 0.24588 | 0.003888 | 0.003901 |
| d.Placebo.Silymarin | -1.61741 | 0.90354 | 0.014286 | 0.014240 |
| d.Placebo.Statins | -0.34152 | 0.13728 | 0.002171 | 0.002171 |
| d.Theophylline.Furosemide | 0.73252 | 0.42953 | 0.006792 | 0.006650 |

| **Quantiles for each variable:** |  |  |  |  |  |
| --- | --- | --- | --- | --- | --- |
|  | 2.5% | 25% | 50% | 75% | 97.5% |
| d.Control.Alpha_Lipoic_Acid | -1.66773 | -0.92725 | -0.55185 | -0.19372 | 0.48798 |

d.Control.Amlodipine_and_Valsartan -0.15734 0.72311 1.19592 1.74448 2.80973

| d.Control.ANP | -2.74786 -1.87684 | | -1.45507 | -1.06919 -0.35059 | |
| --- | --- | --- | --- | --- | --- |
| d.Control.Cordyceps | -1.64195 -0.92412 | | -0.57723 | -0.24068 | 0.40276 |
| d.Control.Dopamine | -0.89267 | 0.23413 | 0.82781 | 1.49924 | 3.03351 |
| d.Control.Hypothermia | -0.70921 -0.11948 | | 0.15870 | 0.43137 | 0.95785 |
| d.Control.MgSO4 | -1.70682 -1.08188 | | -0.78826 | -0.47814 | 0.09066 |
| d.Control.Oxygen | -1.68583 | -1.39090 | -1.22380 | -1.06011 -0.77801 | |
| d.Control.Pentoxifylline | -0.93179 | -0.57516 | -0.40439 | -0.23229 | 0.09911 |
| d.Control.Probucol | -1.97580 | -1.49118 | -1.25155 | -1.00902 -0.56411 | |

| d.Control.Trimetazidine | -1.86293 | -1.44914 -1.24812 | | -1.05631 -0.72264 | |
| --- | --- | --- | --- | --- | --- |
| d.NAC.Allopurinol | -1.74853 | -0.93268 -0.54391 | | -0.15233 | 0.52889 |
| d.NAC.Ascorbic_Acid | -0.38244 | -0.09244 | 0.04962 | 0.19068 | 0.45951 |
| d.NAC.Control | 0.49821 | 0.63253 | 0.70362 | 0.77561 | 0.91988 |
| d.NAC.Dialysis | -0.43727 | -0.12236 | 0.04950 | 0.21561 | 0.53980 |
| d.NAC.Fenoldopam | 0.13389 | 0.37773 | 0.51133 | 0.64728 | 0.88636 |
| d.NAC.I_V_Hydartion | 0.01805 | 0.18022 | 0.27332 | 0.36722 | 0.53236 |
| d.NAC.NaHCO3 | -0.15980 | 0.02625 | 0.12688 | 0.22748 | 0.41777 |
| d.NAC.Nebivolol | -0.92358 | -0.25034 | 0.08293 | 0.40992 | 0.99250 |
| d.NAC.Placebo | 0.15305 | 0.26111 | 0.31442 | 0.37005 | 0.47934 |
| d.NAC.Theophylline | -1.00912 | -0.60139 -0.37692 | | -0.16511 | 0.22897 |
| d.NAC.Zinc | -0.79709 | 0.37832 | 0.97247 | 1.61332 | 2.85561 |
| d.NaHCO3.Acetazolamide | -1.80127 | -1.06374 -0.70040 | | -0.35847 | 0.25826 |
| d.NaHCO3.K_Na_citrate | -2.10536 | -1.38934 -1.03692 | | -0.69928 -0.11456 | |
| d.NaHCO3.Oral_Hydration | -0.88222 | -0.39188 -0.14901 | | 0.11600 | 0.57653 |
| d.Placebo.ACEI | -2.22123 | -1.13152 -0.61509 | | -0.12210 | 0.82997 |
| d.Placebo.alpha_tocopherol | -1.67468 | -1.11534 -0.83757 | | -0.56361 -0.08768 | |
| d.Placebo.Aminophylline | -1.98575 | -0.98136 -0.49456 | | -0.02837 | 0.89179 |
| d.Placebo.Anisodamine | -1.33717 | -0.91914 -0.70021 | | -0.49034 -0.08686 | |
| d.Placebo.BNP | -1.36160 | -1.08061 -0.93990 | | -0.80186 -0.53890 | |
| d.Placebo.ERAs | 0.48599 | 0.87907 | 1.10959 | 1.33255 | 1.78586 |
| d.Placebo.Glutathione | -0.57850 | -0.16103 | 0.05833 | 0.26977 | 0.69020 |
| d.Placebo.Nicorandil | -1.47111 | -1.03598 -0.80365 | | -0.58997 -0.16973 | |
| d.Placebo.PGE1 | -1.67398 | -1.32069 -1.14916 | | -0.96566 -0.62667 | |
| d.Placebo.RIPC | -1.11041 | -0.79434 -0.62725 | | -0.45412 -0.15517 | |
| d.Placebo.Silymarin | -3.58621 | -2.15301 -1.55365 | | -0.98557 -0.04714 | |
| d.Placebo.Statins | -0.60589 | -0.43268 -0.34236 | | -0.25000 -0.06883 | |
| d.Theophylline.Furosemide | -0.09049 | 0.43956 | 0.72742 | 1.01574 | 1.57579 |

**3. RCTs with High Baseline Renal Profile (105):**

**Random effect Model:**

**Potential scale reduction factors**

|  | | Point est. Upper C.I. | |
| --- | --- | --- | --- |
| d.Ascorbic_Acid.Control | | 0.999 | 1.00 |
| d.Ascorbic_Acid.NAC | | 1.000 | 1.00 |
| d.Ascorbic_Acid.Placebo | | 1.000 | 1.00 |
| d.Control.Anisodamine | | 1.000 | 1.00 |
| d.Control.ANP | | 1.001 | 1.00 |
| d.Control.BNP | | 1.000 | 1.00 |
| d.Control.Cordyceps | | 1.000 | 1.00 |
| d.Control.Dialysis | | 1.000 | 1.00 |
| d.Control.Dopamine | | 1.001 | 1.00 |
| d.Control.Fenoldopam | | 1.001 | 1.00 |
| d.Control.Furosemide | | 1.000 | 1.00 |
| d.Control.Hypothermia | | 1.001 | 1.00 |
| d.Control.I_V_Hydartion | | 1.001 | 1.00 |
| d.Control.NaHCO3 | | 1.000 | 1.00 |
| d.Control.Nebivolol | | 1.001 | 1.00 |
| d.Control.Nicorandil | | 1.001 | 1.00 |
| d.Control.Oxygen | | 1.001 | 1.00 |
| d.Control.RIPC | | 1.000 | 1.00 |
| d.Control.Statins | | 1.001 | 1.00 |
| d.Control.Theophylline | | 1.001 | 1.00 |
| d.Control.Trimetazidine | | 1.000 | 1.00 |
| d.I_V_Hydartion.Oral_Hydration | | 1.002 | 1.00 |
| d.NAC.Allopurinol | | 1.000 | 1.00 |
| d.NAC.Zinc | | 1.000 | 1.00 |
| d.NaHCO3.K_Na_citrate | | 1.001 | 1.00 |
| d.Placebo.ACEI | | 1.001 | 1.00 |
| d.Placebo.alpha_tocopherol | | 1.000 | 1.00 |
| d.Placebo.Aminophylline | | 1.000 | 1.00 |
| d.Placebo.ERAs | 1.000 | | 1.00 |
| d.Placebo.PGE1 | 1.001 | | 1.00 |
| d.Statins.Pentoxifylline | 1.001 | | 1.01 |
| sd.d | 1.001 | | 1.00 |

**Empirical mean and standard deviation for each variable, plus standard error of the mean:**

|  | Mean | SD Naive SE Time-series SE | | |
| --- | --- | --- | --- | --- |
| d.Ascorbic_Acid.Control | 0.450511 | 0.3843 | 0.006077 | 0.008785 |
| d.Ascorbic_Acid.NAC | -0.241799 | 0.3564 | 0.005635 | 0.008457 |
| d.Ascorbic_Acid.Placebo | 0.245469 | 0.3519 | 0.005564 | 0.008667 |
| d.Control.Anisodamine | -0.833516 | 0.6705 | 0.010601 | 0.010289 |
| d.Control.ANP | -1.514412 | 0.8303 | 0.013129 | 0.013615 |
| d.Control.BNP | -1.235438 | 0.7567 | 0.011965 | 0.012486 |
| d.Control.Cordyceps | -0.826626 | 0.9830 | 0.015542 | 0.016761 |
| d.Control.Dialysis | -0.580348 | 0.3465 | 0.005479 | 0.005481 |
| d.Control.Dopamine | 0.883876 | 1.1526 | 0.018224 | 0.020028 |
| d.Control.Fenoldopam | -0.124072 | 0.3653 | 0.005775 | 0.005887 |
| d.Control.Furosemide | -0.359255 | 0.5068 | 0.008014 | 0.008129 |
| d.Control.Hypothermia | 0.134221 | 0.7037 | 0.011127 | 0.011129 |
| d.Control.I_V_Hydartion | -0.237662 | 0.2477 | 0.003916 | 0.004100 |
| d.Control.NaHCO3 | -0.533058 | 0.2610 | 0.004126 | 0.004227 |
| d.Control.Nebivolol | -0.650262 | 0.6917 | 0.010937 | 0.010935 |
| d.Control.Nicorandil | -1.057833 | 0.4901 | 0.007749 | 0.007610 |
| d.Control.Oxygen | -2.761998 | 1.4328 | 0.022655 | 0.026698 |
| d.Control.RIPC | -1.191506 | 0.4895 | 0.007740 | 0.007738 |
| d.Control.Statins | -0.583327 | 0.3663 | 0.005792 | 0.005486 |
| d.Control.Theophylline | -1.043921 | 0.4228 | 0.006685 | 0.006685 |

| d.Control.Trimetazidine | -1.257669 | 0.4317 | 0.006826 | 0.006345 |
| --- | --- | --- | --- | --- |
| d.I_V_Hydartion.Oral_Hydration -2.029350 | | 1.4586 | 0.023063 | 0.030814 |
| d.NAC.Allopurinol | -0.464363 | 0.7719 | 0.012204 | 0.013559 |
| d.NAC.Zinc | 1.108796 | 1.0714 | 0.016941 | 0.018450 |
| d.NaHCO3.K_Na_citrate | 0.006571 | 1.2736 | 0.020138 | 0.023279 |
| d.Placebo.ACEI | -0.675800 | 0.9759 | 0.015430 | 0.016306 |
| d.Placebo.alpha_tocopherol | -0.836930 | 0.7089 | 0.011209 | 0.011351 |
| d.Placebo.Aminophylline | -0.525175 | 0.9244 | 0.014615 | 0.014969 |
| d.Placebo.ERAs | 1.127875 | 0.6526 | 0.010319 | 0.010319 |
| d.Placebo.PGE1 | -1.653025 | 0.8181 | 0.012936 | 0.012794 |
| d.Statins.Pentoxifylline | 0.813603 | 0.9470 | 0.014973 | 0.015288 |
| sd.d | 0.555895 | 0.1016 | 0.001607 | 0.002391 |

**Quantiles for each variable:**

|  | 2.5% | 25% | 50% | 75% | 97.5% |
| --- | --- | --- | --- | --- | --- |
| d.Ascorbic_Acid.Control | -0.3247 | 0.199098 | 0.45972 | 0.7084906 | 1.18273 |
| d.Ascorbic_Acid.NAC | -0.9608 | -0.474717 -0.23616 | | 0.0042868 | 0.43890 |
| d.Ascorbic_Acid.Placebo | -0.4592 | 0.009571 | 0.25763 | 0.4815471 | 0.90545 |
| d.Control.Anisodamine | -2.1963 | -1.248562 -0.83058 | | -0.3921122 | 0.47622 |
| d.Control.ANP | -3.1887 | -2.063836 -1.47818 | | -0.9500450 | 0.07893 |
| d.Control.BNP | -2.7425 | -1.722361 -1.22495 | | -0.7387070 | 0.20277 |
| d.Control.Cordyceps | -2.8145 | -1.477054 -0.82359 | | -0.1594784 | 1.03749 |
| d.Control.Dialysis | -1.2605 | -0.809626 -0.58117 | | -0.3492190 | 0.09168 |
| d.Control.Dopamine | -1.2086 | 0.104475 | 0.84720 | 1.6040865 | 3.25726 |
| d.Control.Fenoldopam | -0.8328 | -0.370387 -0.12033 | | 0.1136796 | 0.60903 |
| d.Control.Furosemide | -1.3599 | -0.705704 -0.36380 | | -0.0131911 | 0.64938 |
| d.Control.Hypothermia | -1.2625 | -0.333975 | 0.13961 | 0.6057220 | 1.48610 |
| d.Control.I_V_Hydartion | -0.7156 | -0.403117 -0.24026 | | -0.0756737 | 0.23928 |
| d.Control.NaHCO3 | -1.0535 | -0.701841 -0.53122 | | -0.3604424 -0.02287 | |
| d.Control.Nebivolol | -2.0465 | -1.108055 -0.63948 | | -0.1827974 | 0.67612 |
| d.Control.Nicorandil | -2.0367 | -1.383918 -1.06091 | | -0.7275765 -0.09474 | |

| d.Control.Oxygen | -6.0214 | -3.573535 -2.61394 | | -1.7689836 -0.42567 | |
| --- | --- | --- | --- | --- | --- |
| d.Control.RIPC | -2.1699 | -1.515994 -1.18289 | | -0.8705149 -0.23216 | |
| d.Control.Statins | -1.3021 | -0.830256 -0.58299 | | -0.3400904 | 0.14282 |
| d.Control.Theophylline | -1.8740 | -1.320890 -1.04890 | | -0.7585986 -0.21802 | |
| d.Control.Trimetazidine | -2.1234 | -1.538269 -1.25406 | | -0.9634597 -0.43900 | |
| d.I_V_Hydartion.Oral_Hydration | -5.3816 | -2.856544 -1.86740 | | -1.0389946 | 0.36152 |
| d.NAC.Allopurinol | -2.0459 | -0.959291 -0.44468 | | 0.0541225 | 0.99967 |
| d.NAC.Zinc | -1.0065 | 0.407052 | 1.08950 | 1.8337312 | 3.16918 |
| d.NaHCO3.K_Na_citrate | -2.4608 | -0.803254 | 0.01015 | 0.8302168 | 2.55141 |
| d.Placebo.ACEI | -2.6852 | -1.319132 -0.64307 | | -0.0004861 | 1.21325 |
| d.Placebo.alpha_tocopherol | -2.2374 | -1.313237 -0.83324 | | -0.3576588 | 0.54709 |
| d.Placebo.Aminophylline | -2.4129 | -1.126157 -0.52562 | | 0.0900294 | 1.24779 |
| d.Placebo.ERAs | -0.1652 | 0.703279 | 1.15092 | 1.5682446 | 2.38287 |
| d.Placebo.PGE1 | -3.3193 | -2.180622 -1.64474 | | -1.1030908 -0.09251 | |
| d.Statins.Pentoxifylline | -0.9574 0.187371 0.80095 1.4058997 2.75017 | | | | |
| sd.d | 0.3637 | 0.487792 | 0.55187 | 0.6209740 | 0.76668 |

**Fixed effect Model:**

**Potential scale reduction factors**

|  | Point est. Upper C.I. | |
| --- | --- | --- |
| d.Ascorbic_Acid.Control | 1.001 | 1.01 |
| d.Ascorbic_Acid.NAC | 1.002 | 1.01 |
| d.Ascorbic_Acid.Placebo | 1.003 | 1.01 |
| d.Control.Anisodamine | 1.000 | 1.00 |
| d.Control.ANP | 1.001 | 1.00 |
| d.Control.BNP | 1.000 | 1.00 |
| d.Control.Cordyceps | 1.001 | 1.00 |
| d.Control.Dialysis | 1.001 | 1.00 |
| d.Control.Dopamine | 1.001 | 1.00 |
| d.Control.Fenoldopam | 1.000 | 1.00 |
| d.Control.Furosemide | 0.999 | 1.00 |
| d.Control.Hypothermia | 1.001 | 1.00 |
| d.Control.I_V_Hydartion | 1.001 | 1.00 |
| d.Control.NaHCO3 | 1.001 | 1.00 |
| d.Control.Nebivolol | 1.000 | 1.00 |
| d.Control.Nicorandil | 1.000 | 1.00 |
| d.Control.Oxygen | 1.000 | 1.00 |
| d.Control.RIPC | 1.000 | 1.00 |
| d.Control.Statins | 1.000 | 1.00 |
| d.Control.Theophylline | 1.002 | 1.01 |
| d.Control.Trimetazidine | 1.000 | 1.00 |
| d.I_V_Hydartion.Oral_Hydration | 1.005 | 1.01 |
| d.NAC.Allopurinol | 1.001 | 1.00 |
| d.NAC.Zinc | 1.002 | 1.00 |
| d.NaHCO3.K_Na_citrate | 1.006 | 1.02 |
| d.Placebo.ACEI | 1.002 | 1.01 |
| d.Placebo.alpha_tocopherol | 1.000 | 1.00 |
| d.Placebo.Aminophylline | 1.000 | 1.00 |

| d.Placebo.ERAs | 1.000 | 1.00 |
| --- | --- | --- |
| d.Placebo.PGE1 | 1.001 | 1.01 |
| d.Statins.Pentoxifylline | 1.001 | 1.00 |

**Empirical mean and standard deviation for each variable, plus standard error of the mean:**

|  | Mean | SD Naive SE Time-series SE | | |
| --- | --- | --- | --- | --- |
| d.Ascorbic_Acid.Control | 0.56796 | 0.2358 | 0.003728 | 0.005322 |
| d.Ascorbic_Acid.NAC | -0.09460 | 0.2126 | 0.003361 | 0.004926 |
| d.Ascorbic_Acid.Placebo | 0.31196 | 0.2102 | 0.003324 | 0.005038 |
| d.Control.Anisodamine | -0.85671 | 0.3751 | 0.005931 | 0.005932 |
| d.Control.ANP | -1.49501 | 0.6176 | 0.009764 | 0.010001 |
| d.Control.BNP | -1.24842 | 0.5128 | 0.008107 | 0.008174 |
| d.Control.Cordyceps | -0.81065 | 0.7894 | 0.012481 | 0.012738 |
| d.Control.Dialysis | -0.64399 | 0.2330 | 0.003685 | 0.003524 |
| d.Control.Dopamine | 0.87913 | 0.9920 | 0.015685 | 0.016623 |
| d.Control.Fenoldopam | -0.09985 | 0.2222 | 0.003514 | 0.003416 |
| d.Control.Furosemide | -0.35479 | 0.3469 | 0.005485 | 0.005486 |
| d.Control.Hypothermia | 0.15866 | 0.4366 | 0.006903 | 0.006842 |
| d.Control.I_V_Hydartion | -0.30079 | 0.1399 | 0.002212 | 0.002331 |
| d.Control.NaHCO3 | -0.52541 | 0.1642 | 0.002597 | 0.002804 |
| d.Control.Nebivolol | -0.61836 | 0.4851 | 0.007670 | 0.007671 |
| d.Control.Nicorandil | -1.13279 | 0.3396 | 0.005369 | 0.005059 |
| d.Control.Oxygen | -2.75896 | 1.3417 | 0.021215 | 0.020797 |
| d.Control.RIPC | -1.20286 | 0.3446 | 0.005449 | 0.005809 |
| d.Control.Statins | -0.52062 | 0.1799 | 0.002844 | 0.002864 |
| d.Control.Theophylline | -1.04304 | 0.3129 | 0.004947 | 0.004958 |

| d.Control.Trimetazidine | -1.26041 | 0.2934 | 0.004639 | 0.004779 |
| --- | --- | --- | --- | --- |
| d.I_V_Hydartion.Oral_Hydration -2.05320 | | 1.3889 | 0.021960 | 0.021379 |
| d.NAC.Allopurinol | -0.51790 | 0.5949 | 0.009406 | 0.009196 |
| d.NAC.Zinc | 1.01701 | 0.9418 | 0.014891 | 0.014642 |
| d.NaHCO3.K_Na_citrate | 0.04283 | 1.1289 | 0.017849 | 0.017829 |
| d.Placebo.ACEI | -0.64002 | 0.7949 | 0.012569 | 0.012555 |
| d.Placebo.alpha_tocopherol | -0.84249 | 0.4041 | 0.006389 | 0.006390 |
| d.Placebo.Aminophylline | -0.53677 | 0.7364 | 0.011643 | 0.012016 |
| d.Placebo.ERAs | 1.12169 | 0.3438 | 0.005436 | 0.005517 |
| d.Placebo.PGE1 | -1.63924 | 0.6052 | 0.009569 | 0.009459 |
| d.Statins.Pentoxifylline | 0.82002 | 0.7621 | 0.012050 | 0.012051 |

**Quantiles for each variable:**

|  | 2.5% | | 25% | 50% | 75% | 97.5% |
| --- | --- | --- | --- | --- | --- | --- |
| d.Ascorbic_Acid.Control | 0.10671 | | 0.4108 | 0.56755 | 0.72313 | 1.03329 |
| d.Ascorbic_Acid.NAC | -0.50202 | | -0.2413 -0.09920 | | 0.04634 | 0.32308 |
| d.Ascorbic_Acid.Placebo | -0.09327 | | 0.1659 | 0.30877 | 0.44937 | 0.71658 |
| d.Control.Anisodamine | -1.61057 | | -1.0969 -0.84747 | | -0.61058 -0.13103 | |
| d.Control.ANP | -2.78388 | | -1.8854 -1.46625 | | -1.06989 -0.36997 | |
| d.Control.BNP | -2.29026 | | -1.5729 -1.22528 | | -0.89804 -0.29446 | |
| d.Control.Cordyceps | -2.46721 | | -1.3021 -0.77777 | | -0.28003 | 0.66062 |
| d.Control.Dialysis | -1.11228 | | -0.8019 -0.64615 | | -0.48970 -0.19069 | |
| d.Control.Dopamine | -0.93191 | | 0.1872 | 0.81780 | 1.50924 | 2.96354 |
| d.Control.Fenoldopam | -0.53265 | | -0.2506 -0.09907 | | 0.05279 | 0.32769 |
| d.Control.Furosemide | -1.06592 | | -0.5820 -0.35330 | | -0.11905 | 0.30765 |
| d.Control.Hypothermia | -0.66727 | | -0.1367 | 0.15881 | 0.44958 | 1.01623 |
| d.Control.I_V_Hydartion | -0.57498 | | -0.3941 -0.30333 | | -0.20619 -0.02964 | |
| d.Control.NaHCO3 | -0.85047 | | -0.6379 -0.52895 | | -0.41315 -0.20353 | |
| d.Control.Nebivolol | -1.58689 | | -0.9410 -0.61245 | | -0.28203 | 0.31320 |
| d.Control.Nicorandil | -1.81802 | | -1.3582 -1.12180 | | -0.89527 -0.49416 | |
| d.Control.Oxygen | -5.86079 | | -3.4813 -2.57070 | | -1.82951 -0.70046 | |
| d.Control.RIPC | | -1.91302 | -1.4335 -1.19386 | | -0.96836 -0.55059 | |
| d.Control.Statins | | -0.86731 | -0.6394 -0.52068 | | -0.39963 -0.16968 | |
| d.Control.Theophylline | | -1.63540 | -1.2552 -1.03820 | | -0.83418 -0.44397 | |
| d.Control.Trimetazidine | | -1.85767 | -1.4554 -1.25565 | | -1.05705 -0.71506 | |
| d.I_V_Hydartion.Oral_Hydration | | -5.31073 | -2.7877 -1.86769 | | -1.09497 | 0.11526 |
| d.NAC.Allopurinol | | -1.74032 | -0.9067 -0.50020 | | -0.10898 | 0.60038 |
| d.NAC.Zinc | | -0.81793 | 0.3715 | 1.00910 | 1.64987 | 2.87454 |
| d.NaHCO3.K_Na_citrate | | -2.16559 | -0.6801 | 0.04720 | 0.75062 | 2.33736 |
| d.Placebo.ACEI | | -2.22077 | -1.1456 -0.63433 | | -0.10681 | 0.89145 |
| d.Placebo.alpha_tocopherol | | -1.64958 | -1.1107 -0.83181 | | -0.56118 -0.09177 | |
| d.Placebo.Aminophylline | | -2.05615 | -0.9836 -0.51645 | | -0.04400 | 0.87751 |
| d.Placebo.ERAs | | 0.46397 | 0.8901 | 1.11207 | 1.35052 | 1.81705 |
| d.Placebo.PGE1 | | -2.85886 | -2.0266 -1.63164 | | -1.24090 -0.48834 | |
| d.Statins.Pentoxifylline | | -0.60925 | 0.3113 | 0.79718 | 1.28601 | 2.37709 |

**4. RCTs with Normal Baseline Renal Profile (53):**

**Random effect Model:**

**Potential scale reduction factors**

|  | Point est. Upper C.I. | |
| --- | --- | --- |
| d.Control.Alpha_Lipoic_Acid | 1 | 1.00 |
| d.Control.Amlodipine_and_Valsartan | 1 | 1.00 |
| d.Control.Cordyceps | 1 | 1.00 |
| d.Control.K_Na_citrate | 1 | 1.00 |
| d.Control.MgSO4 | 1 | 1.00 |
| d.Control.Oxygen | 1 | 1.00 |
| d.Control.Pentoxifylline | 1 | 1.00 |
| d.Control.PGE1 | 1 | 1.00 |
| d.Control.Probucol | 1 | 1.00 |
| d.Control.RIPC | 1 | 1.00 |
| d.Control.Statins | 1 | 1.01 |
| d.I_V_Hydartion.Oral_Hydration | 1 | 1.00 |
| d.NAC.Control | 1 | 1.00 |
| d.NAC.I_V_Hydartion | 1 | 1.00 |
| d.NAC.NaHCO3 | 1 | 1.00 |
| d.NAC.Placebo | 1 | 1.00 |
| d.NaHCO3.Acetazolamide | 1 | 1.00 |
| d.Placebo.Anisodamine | 1 | 1.00 |
| d.Placebo.BNP | 1 | 1.01 |
| d.Placebo.Glutathione | 1 | 1.00 |
| d.Placebo.Silymarin | 1 | 1.00 |
| sd.d | 1 | 1.01 |

**Empirical mean and standard deviation for each variable, plus standard error of the mean:**

|  | Mean | SD Naive SE Time-series SE | | |
| --- | --- | --- | --- | --- |
| d.Control.Alpha_Lipoic_Acid | -0.54494 | 0.6502 | 0.010281 | 0.012059 |
| d.Control.Amlodipine_and_Valsartan 1.23852 | | 0.8987 | 0.014209 | 0.013114 |
| d.Control.Cordyceps | -0.45076 | 0.8449 | 0.013358 | 0.013585 |
| d.Control.K_Na_citrate | -1.93695 | 0.7630 | 0.012064 | 0.012298 |
| d.Control.MgSO4 | -0.76880 | 0.6520 | 0.010309 | 0.010168 |
| d.Control.Oxygen | -1.14905 | 0.5175 | 0.008182 | 0.008428 |
| d.Control.Pentoxifylline | -0.51422 | 0.4034 | 0.006378 | 0.006864 |
| d.Control.PGE1 | -1.57252 | 0.4821 | 0.007623 | 0.007922 |
| d.Control.Probucol | -1.25090 | 0.4805 | 0.007598 | 0.007729 |
| d.Control.RIPC | -0.79882 | 0.4258 | 0.006733 | 0.007046 |
| d.Control.Statins | -1.11916 | 0.2956 | 0.004674 | 0.004786 |
| d.I_V_Hydartion.Oral_Hydration | -0.04514 | 0.5212 | 0.008240 | 0.009032 |
| d.NAC.Control | 0.85963 | 0.3017 | 0.004771 | 0.005341 |
| d.NAC.I_V_Hydartion | 0.25033 | 0.3408 | 0.005388 | 0.005983 |
| d.NAC.NaHCO3 | 0.39619 | 0.3870 | 0.006118 | 0.006638 |
| d.NAC.Placebo | 0.21568 | 0.2202 | 0.003481 | 0.003658 |
| d.NaHCO3.Acetazolamide | -0.83013 | 0.6834 | 0.010805 | 0.013844 |
| d.Placebo.Anisodamine | -1.30176 | 0.7515 | 0.011883 | 0.013463 |
| d.Placebo.BNP | -0.81088 | 0.4039 | 0.006385 | 0.006752 |
| d.Placebo.Glutathione | 0.04459 | 0.5567 | 0.008802 | 0.008594 |

| d.Placebo.Silymarin | -1.59308 | 1.0031 | 0.015860 | 0.017857 | |
| --- | --- | --- | --- | --- | --- |
| sd.d | 0.42683 | 0.1322 | 0.002090 | 0.004373 | |
| **Quantiles for each variable:** |  |  |  |  |  |
|  | 2.5% | 25% | 50% | 75% | 97.5% |
| d.Control.Alpha_Lipoic_Acid | -1.8216 | -0.97839 -0.54413 | | -0.1125 | 0.74732 |

d.Control.Amlodipine_and_Valsartan -0.4612 0.62101 1.21699 1.8211 3.05349

| d.Control.Cordyceps | -2.1270 -0.99531 | | -0.43708 | 0.1076 | 1.12168 |
| --- | --- | --- | --- | --- | --- |
| d.Control.K_Na_citrate | -3.4690 -2.42437 | | -1.91775 | -1.4277 -0.50290 | |
| d.Control.MgSO4 | -2.0687 -1.19421 | | -0.76630 | -0.3466 | 0.57101 |
| d.Control.Oxygen | -2.1749 -1.48029 | | -1.14113 | -0.8226 -0.11387 | |
| d.Control.Pentoxifylline | -1.2885 -0.78553 | | -0.51662 | -0.2552 | 0.31009 |
| d.Control.PGE1 | -2.5367 -1.87767 | | -1.56854 | -1.2570 -0.63509 | |
| d.Control.Probucol | -2.1717 -1.57629 | | -1.25138 | -0.9252 -0.31201 | |
| d.Control.RIPC | -1.6436 -1.07266 | | -0.79324 | -0.5188 | 0.03713 |
| d.Control.Statins | -1.7168 -1.31566 | | -1.10755 | -0.9263 -0.54868 | |
| d.I_V_Hydartion.Oral_Hydration | -1.0456 -0.39066 | | -0.05614 | 0.2921 | 0.99279 |
| d.NAC.Control | 0.2830 | 0.66408 | 0.85863 | 1.0492 | 1.46474 |
| d.NAC.I_V_Hydartion | -0.4130 | 0.02741 | 0.25365 | 0.4713 | 0.92640 |
| d.NAC.NaHCO3 | -0.3472 | 0.13135 | 0.40254 | 0.6547 | 1.16015 |
| d.NAC.Placebo | -0.2081 | 0.06916 | 0.21046 | 0.3577 | 0.65599 |
| d.NaHCO3.Acetazolamide | -2.2278 -1.26510 | | -0.82036 | -0.3868 | 0.48292 |
| d.Placebo.Anisodamine | -2.8079 -1.78505 | | -1.27162 | -0.7941 | 0.13738 |
| d.Placebo.BNP | -1.5897 -1.08031 | | -0.81625 | -0.5494 -0.01411 | |
| d.Placebo.Glutathione | -1.0760 -0.31771 | | 0.04786 | 0.4094 | 1.12414 |
| d.Placebo.Silymarin | -3.7460 -2.20268 | | -1.53052 | -0.9040 | 0.18154 |
| sd.d | 0.1629 | 0.33984 | 0.42639 | 0.5107 | 0.69823 |

**Fixed effect Model:**

**Potential scale reduction factors**

|  | Point est. Upper C.I. | |
| --- | --- | --- |
| d.Control.Alpha_Lipoic_Acid | 1 | 1.00 |
| d.Control.Amlodipine_and_Valsartan | 1 | 1.00 |
| d.Control.Cordyceps | 1 | 1.00 |
| d.Control.K_Na_citrate | 1 | 1.00 |
| d.Control.MgSO4 | 1 | 1.00 |
| d.Control.Oxygen | 1 | 1.00 |
| d.Control.Pentoxifylline | 1 | 1.00 |
| d.Control.PGE1 | 1 | 1.00 |
| d.Control.Probucol | 1 | 1.00 |
| d.Control.RIPC | 1 | 1.00 |
| d.Control.Statins | 1 | 1.00 |
| d.I_V_Hydartion.Oral_Hydration | 1 | 1.00 |
| d.NAC.Control | 1 | 1.00 |
| d.NAC.I_V_Hydartion | 1 | 1.01 |
| d.NAC.NaHCO3 | 1 | 1.01 |
| d.NAC.Placebo | 1 | 1.01 |
| d.NaHCO3.Acetazolamide | 1 | 1.00 |
| d.Placebo.Anisodamine | 1 | 1.00 |
| d.Placebo.BNP | 1 | 1.00 |
| d.Placebo.Glutathione | 1 | 1.01 |
| d.Placebo.Silymarin | 1 | 1.00 |

**Empirical mean and standard deviation for each variable, plus standard error of the mean:**

|  | Mean | SD Naive SE Time-series SE | | |
| --- | --- | --- | --- | --- |
| d.Control.Alpha_Lipoic_Acid | -0.54432 | 0.5451 | 0.008620 | 0.008748 |
| d.Control.Amlodipine_and_Valsartan 1.21784 | | 0.7662 | 0.012115 | 0.012265 |
| d.Control.Cordyceps | -0.44072 | 0.7101 | 0.011228 | 0.011637 |
| d.Control.K_Na_citrate | -1.92768 | 0.5815 | 0.009194 | 0.009093 |
| d.Control.MgSO4 | -0.79062 | 0.4623 | 0.007309 | 0.007190 |
| d.Control.Oxygen | -1.14330 | 0.2483 | 0.003926 | 0.003927 |
| d.Control.Pentoxifylline | -0.51352 | 0.2774 | 0.004386 | 0.004388 |
| d.Control.PGE1 | -1.68731 | 0.3329 | 0.005264 | 0.005512 |
| d.Control.Probucol | -1.25479 | 0.3506 | 0.005543 | 0.005813 |
| d.Control.RIPC | -0.87223 | 0.3426 | 0.005416 | 0.005368 |
| d.Control.Statins | -1.07288 | 0.2045 | 0.003233 | 0.003288 |
| d.I_V_Hydartion.Oral_Hydration | -0.03391 | 0.4204 | 0.006647 | 0.006503 |
| d.NAC.Control | 0.87548 | 0.1937 | 0.003063 | 0.003890 |
| d.NAC.I_V_Hydartion | 0.24938 | 0.2142 | 0.003387 | 0.004332 |
| d.NAC.NaHCO3 | 0.29756 | 0.2478 | 0.003918 | 0.004661 |
| d.NAC.Placebo | 0.13806 | 0.1360 | 0.002150 | 0.002215 |
| d.NaHCO3.Acetazolamide | -0.80712 | 0.5409 | 0.008553 | 0.009302 |
| d.Placebo.Anisodamine | -1.32335 | 0.6307 | 0.009973 | 0.010369 |
| d.Placebo.BNP | -0.84716 | 0.2554 | 0.004038 | 0.004348 |
| d.Placebo.Glutathione | 0.04504 | 0.3189 | 0.005043 | 0.005042 |

| d.Placebo.Silymarin | -1.59913 0.8976 0.014192 | 0.014191 |
| --- | --- | --- |

**Quantiles for each variable:**

|  | 2.5% | 25% | 50% | 75% | 97.5% |
| --- | --- | --- | --- | --- | --- |
| d.Control.Alpha_Lipoic_Acid | -1.6578 | -0.89484 | -0.53665 | -0.18591 | 0.49644 |

d.Control.Amlodipine_and_Valsartan -0.1385 0.68796 1.17322 1.68655 2.82902

| d.Control.Cordyceps |  | -1.8638 -0.90784 | | -0.43055 | 0.03201 | 0.91796 |
| --- | --- | --- | --- | --- | --- | --- |
| d.Control.K_Na_citrate |  | -3.1531 -2.28170 | | -1.89337 | -1.51266 -0.88501 | |
| d.Control.MgSO4 |  | -1.7350 -1.08804 | | -0.78678 | -0.48674 | 0.09190 |
| d.Control.Oxygen |  | -1.6301 -1.30938 | | -1.14549 | -0.97157 -0.65760 | |
| d.Control.Pentoxifylline |  | -1.0642 -0.69704 | | -0.51171 | -0.33001 | 0.04172 |
| d.Control.PGE1 |  | -2.3613 -1.90617 | | -1.68364 | -1.46046 -1.04138 | |
| d.Control.Probucol |  | -1.9610 -1.49009 | | -1.25054 | -1.00945 -0.59982 | |
| d.Control.RIPC |  | -1.5645 -1.10301 | | -0.87012 | -0.64100 -0.20383 | |
| d.Control.Statins |  | -1.4796 -1.21016 | | -1.06567 | -0.93473 -0.67938 | |
| d.I_V_Hydartion.Oral_Hydration | | -0.8650 -0.31131 | | -0.03294 | 0.25277 | 0.78425 |
| d.NAC.Control |  | 0.5078 | 0.74358 | 0.87224 | 1.00555 | 1.26603 |
| d.NAC.I_V_Hydartion |  | -0.1616 | 0.10522 | 0.24910 | 0.38842 | 0.67256 |
| d.NAC.NaHCO3 |  | -0.1971 | 0.12980 | 0.29589 | 0.46392 | 0.77205 |
| d.NAC.Placebo |  | -0.1262 | 0.04484 | 0.13781 | 0.22855 | 0.41609 |
| d.NaHCO3.Acetazolamide |  | -1.9214 -1.15403 | | -0.78580 | -0.43320 | 0.20065 |
| d.Placebo.Anisodamine |  | -2.6377 -1.72697 | | -1.28815 | -0.89237 -0.14898 | |
| d.Placebo.BNP |  | -1.3430 -1.01677 | | -0.84875 | -0.67323 -0.34871 | |
| d.Placebo.Glutathione |  | -0.5699 -0.17103 | | 0.04111 | 0.26153 | 0.68193 |
| d.Placebo.Silymarin |  | -3.5916 -2.12044 | | -1.53861 | -0.98298 -0.03301 | |

**References:**

1. Dias S, Sutton AJ, Ades AE, Welton NJ. Evidence synthesis for decision making 2: a generalized linear modeling framework for pairwise and network meta-analysis of randomized controlled trials. Med Decis Making. 2013;33(5):607-17.

2. Jansen JP, Fleurence R, Devine B, Itzler R, Barrett A, Hawkins N, et al. Interpreting indirect treatment comparisons and network meta-analysis for health-care decision making: report of the ISPOR Task Force on Indirect Treatment Comparisons Good Research Practices: part 1. Value Health. 2011;14(4):417-28.

3. Neupane B, Richer D, Bonner AJ, Kibret T, Beyene J. Network meta-analysis using R: a review of currently available automated packages. PLoS One. 2014;9(12):e115065.

4. van Valkenhoef G, Lu G, de Brock B, Hillege H, Ades AE, Welton NJ. Automating network meta-analysis. Res Synth Methods. 2012;3(4):285-99.

5. van Valkenhoef G, Tervonen T, de Brock B, Hillege H. Algorithmic parameterization of mixed treatment comparisons. Statistics and Computing. 2012;22(5):1099-111.

6. Brown S, Hutton B, Clifford T, Coyle D, Grima D, Wells G, et al. A Microsoft-Excel-based tool for running and critically appraising network meta-analyses--an overview and application of NetMetaXL. Syst Rev. 2014;3:110.

7. van der Linden WJ. Handbook of Item Response Theory, Volume One: Models: CRC Press; 2016.

8. Gelman A, Rubin DB. Inference from Iterative Simulation Using Multiple Sequences. Statist Sci. 1992;7(4):457-72.

9. Brooks SP, Gelman A. General Methods for Monitoring Convergence of Iterative Simulations. Journal of Computational and Graphical Statistics. 1998;7(4):434-55.

10. Salanti G, Del Giovane C, Chaimani A, Caldwell DM, Higgins JP. Evaluating the quality of evidence from a network meta-analysis. PLoS One. 2014;9(7):e99682.

11. Higgins JP, Thompson SG. Quantifying heterogeneity in a meta-analysis. Stat Med. 2002;21(11):1539-58.

12. Krahn U, Binder H, Konig J. A graphical tool for locating inconsistency in network meta-analyses. BMC Med Res Methodol. 2013;13:35.

13. Valkenhoef G. Modeling inconsistency as heterogeneity in network meta-analysis. Society for Research Synthesis Methods Annual Meeting. 2013.

14. Hoaglin DC, Hawkins N, Jansen JP, Scott DA, Itzler R, Cappelleri JC, et al. Conducting indirect-treatment-comparison and network-meta-analysis studies: report of the ISPOR Task Force on Indirect Treatment Comparisons Good Research Practices: part 2. Value Health. 2011;14(4):429-37.

15. Higgins JPT, Green S. Cochrane Handbook for Systematic Reviews of Interventions: Wiley; 2011.

16. Spittal MJ, Pirkis J, Gurrin LC. Meta-analysis of incidence rate data in the presence of zero events. BMC Med Res Methodol. 2015;15:42.

17. Böhning D, Mylona K, Kimber A. Meta-analysis of clinical trials with rare events. Biometrical Journal. 2015;57(4):633-48.

18. Tu YK. Using Generalized Linear Mixed Models to Evaluate Inconsistency within a Network Meta-Analysis. Value Health. 2015;18(8):1120-5.

19. Bradburn MJ, Deeks JJ, Berlin JA, Russell Localio A. Much ado about nothing: a comparison of the performance of meta-analytical methods with rare events. Stat Med. 2007;26(1):53-77.

20. Cheng J, Pullenayegum E, Marshall JK, Iorio A, Thabane L. Impact of including or excluding both-armed zero-event studies on using standard meta-analysis methods for rare event outcome: a simulation study. BMJ Open. 2016;6(8):e010983.

21. Diamond GA, Bax L, Kaul S. Uncertain effects of rosiglitazone on the risk for myocardial infarction and cardiovascular death. Ann Intern Med. 2007;147(8):578-81.

22. Liu J, Li L, Deng K, Xu C, Busse JW, Vandvik PO, et al. Incretin based treatments and mortality in patients with type 2 diabetes: systematic review and meta-analysis. BMJ. 2017;357:j2499.

23. Sweeting MJ, Sutton AJ, Lambert PC. What to add to nothing? Use and avoidance of continuity corrections in meta-analysis of sparse data. Stat Med. 2004;23(9):1351-75.

24. Trelle S, Reichenbach S, Wandel S, Hildebrand P, Tschannen B, Villiger PM, et al. Cardiovascular safety of non-steroidal anti-inflammatory drugs: network meta-analysis. BMJ. 2011;342:c7086.

25. Whitehead A, Whitehead J. A general parametric approach to the meta-analysis of randomized clinical trials. Stat Med. 1991;10(11):1665-77.
