# Supplementary material for: Effectiveness of contrast-associated acute kidney injury prevention methods; a systematic review and network meta-analysis
Source: BMC Nephrol. 2018 Nov 13;19:323. doi: 10.1186/s12882-018-1113-0 (PMC6234687; doi:10.1186/s12882-018-1113-0)
Supplement: Supplementary file 2 — Studies characteristics. (DOCX 889 kb) [file 12882_2018_1113_MOESM2_ESM.docx]

| **Trial** | **Inclusion and Exclusion Criteria** | **Procedure** | **Number of patients in analysis** | **CA-AKI Definition** | **Contrast / OSM** | **I.V Fluid** | **Intervention** | **Dose** | | **Contrast Volume (ml)** | | | **Baseline Renal function** | |
| --- | --- | --- | --- | --- | --- | --- | --- | --- | --- | --- | --- | --- | --- | --- |
| A.C.T Investigators 2011(75) | Inclusion Criteria: Patients undergoing  1. Coronary or peripheral arterial diagnostic intravascular angiography OR  2. Percutaneous intervention  With  ≥1 risk factor for CI-AKI  1. Age >70 years 2. CRF (stable S. Creat. >132.6 mmol/L [1.5 mg/dL]) 3. Diabetes mellitus  4. Clinical evidence of congestive heart failure 5. LVEF<0.45 6. Hypotension  Exclusion Criteria:  1. Patients on dialysis 2. Primary angioplasty for STEMI 3. Pregnancy 4. Breastfeeding 5. Women of childbearing age not on contraception | 1. Peripheral vascular angiography 2. Coronary diagnostic angiography 3. PCI | 2561 | >25% increase in S. Creat. from baseline, 48-96 hours post-angiography | 1. Hi-Osm 2. Iso-Osm 3. Low-Osm | • 0.9% Normal Saline 1 mL/kg/hr from 6-12 hours pre- and 6-12 hours post-angiography  (Changes in volume/ speed of administration permitted) | NAC | 1200 mg orally every 12 hours • 2 doses pre- and 2 doses post- procedure | | Median (IQR): 100 (70-130) | | | Mean S. Creat. 1.2±0.5mg/dL | |
|  |  |  |  |  |  |  | Placebo | 1200 mg orally every 12 hours • 2 doses pre- and 2 doses post- procedure | | Median (IQR): 100 (70-130) | | | Mean S. Creat. 1.2±0.5mg/dL | |
| Abouzeid 2016(187) | Inclusion Criteria:  Adult patients > 18 years with renal dysfunction [estimated glomerular filtration rate (eGFR), 60 mL/min/1.73 m^2^or less]  Exclusion Criteria:  1. End-stage renal insufficiency (eGFR <15 mL/min)  2. Acute renal insufficiency  3.History of reaction to contrast media  4. use of potentially nephrotoxic medicines (48 h before and 24 h after the procedure)  5. pulmonary oedema  6. Multiple myeloma  7. Exposure to contrast media within seven days before the procedure  8. Pregnancy  9. Noncompliance of the patient  10. Use of N-acetylcysteine, dopamine, fenoldopam, and mannitol before CAG. | 1.Coronary Angiography | 210 | Increase of more than absolute 0.5 mg/dL and/or relative 25% in serum creatinine after 48 h | Iohexol Low-Osm | All patients were hydrated with intravenous (i.v.) normal saline at 1 mL/kg/h for 12 h before exposure to the contrast and 12 h after coronary angiography | Na/K citrate | 5 g of granules diluted in 200 mL of water 12, 6, and 1 h before procedure followed by another two doses after 2 and 8 h post procedure. | | Mean+SD:  103.93±47.70 | | | S. Creat.  Mean+SD:  2.30±0.46 | |
|  |  |  |  |  |  |  | NaHco3 | solution (150 mEq/L) for 8 h before procedure continued until 6 h post procedure | | Mean+SD:  105.24±45.38 | | | S. Creat.  Mean+SD:  2.28±0.44 | |
| Abaci  2015(17) | Inclusion Criteria:  Elective coronary or peripheral angiography    Exclusion Criteria  1. Emergency coronary angiography 2. Acute or end-stage renal failure requiring dialysis 3. eGFRs <30 or ≥60ml/min/1.73 m2 4. Congestive heart failure  5. Coronary artery disease 6. Severe coronary occlusion for CABG or PCI  7. Allergy to contrast media 8. Contrast media administration within the previous 14 days 9. Current statin treatment 10. Contraindications to statin treatment 11. Severe co-morbidities 12. Pregnancy 13. Patient refusal | 1.Coronary Angiography | 235 | Absolute increase in S. Creat. of ≥0.5 mg/dl OR Relative increase of ≥25% 48-72 hours post- procedure | Low-Osm (Optiray - Ioversol) | Isotonic saline (1ml/kg/h) for 12 hours before and 24 hours after contrast exposure | Rosuvastatin | 40 mg on admission Followed by 20 mg daily | | Mean ± SD.:  139.2 ± 77.4 | | | eGFRs <60 ml/min/1.73 m2 measured on day preceding procedure | |
|  |  |  |  |  |  |  | Control (no statin) | - | | Mean ± SD.:  117.7+/-56.8 | | |  | |
| Adolph 2008(18) | Inclusion Criteria:  1. Patients > 18y 2. Elective diagnostic/ therapeutic coronary angiography 3. Stable renal insufficiency (2 repeated S. Creat. levels > 106µmol/L (>1.2mg/dL) within 12 weeks before procedures, with <5% difference between levels  Exclusion Criteria:  1. Acute MI requiring primary or rescue PCI 2. Allergy to trial medication 3. Exposure to contrast media within 7 days of procedure 4. Thyroid dysfunction 5. Pregnancy 6. Uncontrolled hypertension 7. Life-limiting co-morbidity 8. Pulmonary Oedema 9. Chronic dialysis 10. Administration of Dopamine, Mannitol, Fenoldopam, or NAC during study | 1. Coronary Angiography 2. PCI | 150 | ≥25% or ≥0.5-mg/dL increase in the S. Creat. at day 0 , or 2 of contrast administration | Iodixanol Iso-Osm | 2ml/Kg/hour for 2 hours before procedure and 1ml/kg/hour during and for 6 hours after | NaCl | 154 mEq/l infusion of sodium chloride in 5% dextrose | | Mean ± SD (Range): 138±52 (51-282) | | | Mean S. Creat. ±SD (Range): 1.57±0.36 (1.20-2.60) mg/dL | |
|  |  |  |  |  |  |  | NaHCo_3_ | 154 mEq/l infusion of sodium bicarbonate in 5% dextrose | | Mean±SD (Range): 141±50 (39-270) | | | Mean S. Creat. ±SD (Range): 1.54±0.51 (1.20-4.60) mg/dL | |
| Akyuz  2014(19) | Inclusion Criteria:  1. Patients undergoing cardiac intervention  2. High risk of CA-AKI   Exclusion Criteria:  1. < 18 years old 2. Type 1 DM 3. History of Dialysis 4. No enough time for volume admission pre-procedure 5. Known allergy for contrast media 6. Exposure to contrast media within 3 days of procedure 7. Nephrotoxic drugs within 7 days of procedure  8. Pregnancy or breast feeding 9. Uncontrolled hypertension 10. AKI of alternative aetiology  11. Contraindication to volume expanders | 1. Coronary Angiography 2. PCI | 241 | ≥25% relative or ≥ 0.5mg/dL absolute increase in S. Creat. from baseline 48 hours post-contrast | Low-Osm Ultravist | As per intervention protocol | I.V Hydration with isotonic (0.9%) saline | 1ml/kg/hr for 12 hours pre-procedure and continued for 12 hours post-procedure | | Mean±SD: 108±76 | | | Median S. Creat (IQR): 0.9 (0.4) mg/dL | |
|  |  |  |  |  |  |  | Oral Hydration | Patients encourage to drink water as much as possible starting 12 hours pre-procedure until 2 hours directly prior to procedure, and continue the same for the 12 hours post-procedure | | Mean±SD: 107±70 | | | Median S. Creat (IQR): 0.9 (0.3) mg/dL | |
| Albabtain 2013(20) | Inclusion Criteria:  1. Patients > 18 years undergoing coronary angiography/ PCI 2. S. Creat. ≥ 1.3 mg/dL OR taking medication for Diabetes mellitus   Exclusion Criteria:  1. Known acute renal failure 2. End-stage renal disease requiring dialysis 3. Intravascular administration of contrast medium within 6 days of procedure 4. Anticipated re-administration of contrast medium within 6 days post-procedure 5. Use of Vitamin C supplements on a daily basis during the week before the procedure 6. Inability to administer the study medication at least 2 hours before the procedure   Note: A fourth study group using combination of trial interventions was excluded from analysis | 1. Coronary Angiography 2. PCI | 195 | 1. 0.5 mg/dL absolute increase in S. Creat.  AND/OR  2. 25% relative decrease in creatinine clearance | Ioxaglate Low-Osm | Normal Saline 50-125 mL/h IV from point of randomization until at least 6 hours post-procedure | NAC | 600 mg orally twice daily for 2 days, starting evening pre-procedure | | Mean±SD:  70.1 ± 60.4 | | | Mean S. Creat. ±SD:  1.45 ± 0.56mg/dL | |
|  |  |  |  |  |  |  | Ascorbic acid | Ascorbic acid as effervescent tablets as three doses:  1. 3g 2 hours pre-procedure 2. 2 g immediately post-procedure 3. 2 g 24 hours post-procedure | | Mean±SD:  88.3 ± 64.8 | | | Mean S. Creat. ±SD:  1.24± 0.44mg/dL | |
|  |  |  |  |  |  |  | Placebo | Placebo as effervescent tablets as three doses:  1. 2 hours pre-procedure 2. Immediately post-procedure 3. 24 hours post-procedure | | Mean±SD:  97.4±99.4 | | | Mean S. Creat. ±SD:  1.22 ± 0.40 mg/dL | |
| Allaqaband 2002(21) | Inclusion Criteria:  Patients undergoing cardiovascular interventions requiring the use of a radio contrast agent  AND  1. Baseline creatinine ≥1.6 mg/dl  OR   2. eGFR ≤ 60 ml/min  Exclusion Criteria: Not specified | 1.PCI | 143 | Increase in S. Creat. level > 0.5 mg/dl after 48 hours | Low-Osm | I.V. saline (0.45%) at 1ml/kg/hr for 12 hr prior to, during, and for 12 hr after the procedure | NAC | 600 mg orally twice daily starting the day before the procedure | | Mean±SD:  1.52±0.81 (ml/kg) | | | Mean S. Creat. ±SD:  2.20±0.73 mg/dL | |
|  |  |  |  |  |  |  | Fenoldopam | I.V. Fenoldopam at 0.1 µg/kg/min starting 4 hr prior to and continued for 4 hr after contrast dose | | Mean±SD:  1.63±0.67 (ml/kg) | | | Mean S. Creat. ±SD:  1.94±0.38 mg/dL | |
|  |  |  |  |  |  |  | Control | I.V fluid only as specified for all groups | | Mean±SD:  1.47±0.90 (ml/kg) | | | Mean S. Creat. ±SD:  2.03±0.79 mg/dL | |
| Amini 2009(22) | Inclusion Criteria  1. Elective diagnostic coronary angiography 2. > 18 years  3. Diabetes mellitus for at least one year AND  4. CKD (S. Creat. ≥ 1.5 mg/dL (male) or ≥ 1.4 mg/dL (female))   Exclusion Criteria  1. Acute coronary syndrome requiring primary or rescue coronary intervention within 12 h of procedure 2. Cardiogenic shock 3. Current peritoneal or haemodialysis 4. Known allergy to NAC | 1.Coronary Angiography | 98 | Increase in serum creatinine ≥ 0.5 mg/ dL (44.2 μmol/L) or ≥ 25% above baseline at 48 h after exposure to contrast | Iohexol Low-Osm Iodixanol Iso-Osm | 1L normal saline (0.9%) commenced in the catheterization laboratory Patients were also encouraged to drink fluids (≥8 glasses of ~200ml) over 12 h pre-procedure | NAC | 600 mg orally twice daily, starting the day before the procedure and continuing for 2 doses post-procedure | | Mean±SD:  118.00±35.20 | | | Mean S. Creat. ±SD:  1.736±0.42 mg/dL | |
|  |  |  |  |  |  |  | Placebo | 600 mg orally twice daily, starting the day before the procedure and continuing for 2 doses post-procedure | | Mean±SD:  121.11±43.95 | | | Mean S. Creat. ±SD:  1.736±0.17 mg/dL | |
| Angoulvant 2009(23) | Inclusion Criteria:  1. Patients 18-80 years  2. Elective coronary angiography ± Percutaneous transluminal coronary angioplasty  AND  3. Baseline S. Creat. < 140µmol/L (1.58 mg/dL)   Exclusion Criteria:  1. NYHA Class IV congestive heart failure 2. Pregnancy 3. Significant valvular heart disease 4. Non-ischaemic dilated cardiomyopathy 5. Active cancer or any life-threatening disease | 1.Coronary Angiography ± PCI | 213 | Increase in serum creatinine > 25% 3 days after contrast exposure | Hexabrix Low-Osm | As per intervention protocol | I.V Hydration | 1000mL of 0.9% saline infusion, started at the beginning of the procedure and continued for the next 24 hours | | Mean±SD:  231.4±7.8 | | | Mean S. Creat. ±SD:  86.7±1.7 µmol/L | |
|  |  |  |  |  |  |  | Control | Received no additional hydration | | Mean±SD:  242.7±8.6 | | | Mean S. Creat. ±SD:  85.6±1.5 µmol/L | |
| Arabmomeni 2015(188) | Inclusion Criteria:  Patients with at least moderate risk for CIN as defined by the Mehran risk score  Exclusion Criteria:  1. unstable angina  2. Myocardial infarction  3. Cardiac arrhythmia  4. Acute or chronic renal failure  5. I.V contrast media in the past month  6. Using theophylline or NAC in the past month  7. Known hypersensitivity to Trial Drugs | Coronary angiography with or without angioplasty | 75 | increase in creatinine of ≥ 0.5 mg/dl or ≥ 25% from the baseline | Iodixanol  Low-Osm | 0.9% sodium chloride (1 ml/kg/h) for 24 h, started 12 h before operation. Patients with left-ventricular ejection fraction of less than 40% or New York Heart Association functional class of III-IV were hydrated at rate of 0.5 ml/kg/h | Theophylline | | 200 mg slow-release theophylline tablet plus placebo | Mean+SD:  124.0 ± 115.2 | | S. Creat.  Mean+SD:  1.14 ± 0.40 | | |
|  |  |  |  |  |  |  | NAC | | 600 mg tablet plus placebo | Mean+SD:  155.6 ± 114.9 | | S. Creat.  Mean+SD:  1.08 ± 0.22 | | |
|  |  |  |  |  |  |  | Theophylline plus NAc (this group were excluded from this analysis) | | Both tablets  All groups receive tablets twice daily, from 24 h before to 48 h after administration of contrast material | Mean+SD:  128.9 ± 89.4 | | S. Creat.  Mean+SD:  1.08 ± 0.22 | | |
| Aslanger 2012(24) | Inclusion Criteria:  1. Patients with STEMI undergoing primary angiography within 12 h of symptoms  2. > 30 years old  Exclusion Criteria:  1. Known allergies to NAC 2. Undergoing chronic dialysis 3. No written informed consent | Primary Angioplasty | 257 | Increase in S. Creat. > 25% 3 days after contrast exposure | Ioxaglate Low-Osm | Isotonic saline hydration (0.9%) for 12 h at 1 ml/kg/h | I.V. NAC | 1200mg bolus during the procedure and 1200mg orally, twice daily for 48h post-procedure | | Mean±SD:  193±57 | | | Mean S. Creat. ±SD:  0.9±0.3 mg/dL | |
|  |  |  |  |  |  |  | Intra-renal NAC   **This group is excluded from our analysis** | 600mg bolus into both renal arteries before and after the procedure, as well as 1200mg of NAC orally twice daily for 48h | |  | | | Mean S. Creat. ±SD:  0.89±0.4 mg/dL | |
|  |  |  |  |  |  |  | Placebo | 12ml bolus of IV saline during procedure and oral doses twice daily for 48h post procedure | | Mean±SD:  204±67 | | | Mean S. Creat. ±SD:  0.86±0.3 mg/dL | |
| Baker 2003(25) | Inclusion Criteria:  Patients with stable renal dysfunction (S. Creat. > 1.36mg/dL OR creatinine clearance <50ml/min)  Exclusion Criteria:  1. Acute Renal failure 2. End stage renal failure on dialysis 3. Patients who received a NSAID (except Aspirin 75-150 mg) within 24h of procedure 4. Systolic blood pressure <90mmHg 5. Hemodynamically significant valvular heart disease 6. Signs of Cardiac failure | 1. Coronary Angiography 2. PCI | 90 | Increase in S. Creat. ≥ 25% 2-4 days after contrast administration | Iodixanol Iso-Osm | Free oral fluids were commenced immediately post-procedure in all patients | I.V. NAC | 150 mg/kg in 500 ml saline (0.9%) over 30 min immediately before contrast exposure followed by 50 mg/kg in 500 ml saline (0.9%) over the subsequent 4 hours | | Mean±SD:  238±155 | | | Mean S. Creat. ±SD:  1.85±0.59 mg/dL | |
|  |  |  |  |  |  |  | Control (IV Fluids) | Normal Saline at 1 ml/kg/h for 12 h pre- and post-procedure. | | Mean±SD:  222±162 | | | Mean S. Creat. ±SD:  1.75±0.41 mg/dL | |
| Balderramo 2004(26) | Inclusion Criteria:  Patients with chronic renal impairment (stable S. Creat. > 1.5mg/dL OR eGFR <50ml/min)) undergoing a same day angiography  Exclusion Criteria 1. Haemodynamic instability 2. Congestive Heart failure 3. Pregnancy 4. Lactation 5. Severe Asthma 6. Contrast media use within preceding 10 days | 1.Coronary angiography  2.Peripheral angiography | 64 | Increase in S. Creat. >0.5mg/dL 48 hours after the procedure | Amidotrizate  Ioxitalamate Iopamidol Iohexol | All patients receive 0.9% normal saline at 4ml/kg/hr for 3 hours prior to, and 2ml/kg/hr for 6 hours after radiocontrast exposure | NAC | 1200mg orally with 125ml of orange juice 3 hours before and 3 hours after catheterization | | Mean±SD:  158±60.5 | | | Mean S. Creat. ±SD:  1.46±0.46mg/dL | |
|  |  |  |  |  |  |  | Placebo | 125ml of orange juice 3 hours before and 3 hours after catheterization | | Mean±SD:  155.5±108.1 | | | Mean S. Creat. ±SD:  1.42±0.37mg/dL | |
| Baskurt 2009(27) | Inclusion Criteria:  1. Patients with moderate CKD (eGFR 30-60 mL/min) undergoing coronary angiography   Exclusion Criteria: 1. Acute coronary syndrome 2. Cardiogenic shock 3. Chronic haemodialysis 4. Overt congestive heart failure 5. Exposure to radiocontrast medium within preceding 14 days 6. Emergent procedures 7. Patients with eGFR < 30 or ≥60 mL/min 8. Age < 21 years 9. Known allergy to NAC, theophylline or contrast agents 10. Contraindications to theophylline 11. Medication shown exerting pharmacokinetic interaction with theophylline | 1.Coronary Angiography | 157 | Increase in S. Creat. >0.5mg/dL 48 hours after the procedure | Ioversol Low-Osm | Isotonic saline 1 mL/kg/h for 12 hours before and after contrast | NAC | 600 mg orally twice daily day preceding and day of angiography | | Mean±SD:  115.61±35.2 | | | Mean S. Creat. ±SD:  1.39±0.24mg/dL | |
|  |  |  |  |  |  |  | NAC+ Theophylline Group: This Group is not included in this analysis | | | | | | | |
|  |  |  |  |  |  |  | Control | I.V Hydration only | | Mean±SD:  113.54±37.7 | | | Mean S. Creat. ±SD:  1.30±0.20mg/dL | |
| Berwanger 2013(28) | Inclusion Criteria  1. Diabetes Mellitus  Exclusion Criteria 1. Dialysis 2. STEMI  3. Pregnancy 4. Breastfeeding 5. Females aged <45 years not using contraception | 1. Angiography 2. Percutaneous intervention | 1421 | Increase in S. Creat. >0.5mg/dL | High-Osm Low-Osm Iso-Osm | 0.9% saline, 1 mL/kg/hr 6-12 hours pre- to 6-12 hours post-angiography | NAC | 1200 mg orally every 12 hours • 2 doses pre- and 2 doses post- procedure | | Median (IQR): 100 (70-130) | | | Mean S. Creat. ±SD:  1.1±0.5mg/dL | |
|  |  |  |  |  |  |  | Placebo | 1200 mg orally every 12 hours • 2 doses pre- and 2 doses post- procedure | | Median (IQR): 100 (70-130) | | | Mean S. Creat. ±SD:  1.1±0.6mg/dL | |
| Bidram 2015(29) | Inclusion Criteria   1. Chronic stable angina  Exclusion Criteria  1. Pregnancy 2. Diabetes mellitus 3. Renal failure 4. Single kidney 5. Cardiogenic shock 6. Unstable angina 7. MI 8. Hypersensitivity to statins 9. Previous intravascular contrast injection within month of admission | 1.PCI | 203 | Increase in serum creatinine > 0.5mg/dL 48 hours after the procedure Increase in S. Creat. of >0.5 mg/dl or >25% from baseline | Iso-Osm Iodixanol | 1 ml/kg/h of isotonic saline solution 12 h pre- and post-contrast | Atorvastatin (patients previously on statins excluded from analysis) | 80mg | | 30-40cc | | | Mean S. Creat. ±SD:  1.18±0.02mg/dL | |
|  |  |  |  |  |  |  | Placebo |  | | 30-40cc | | | Mean S. Creat. ±SD:  1.14±0.02mg/dL | |
| Bilasy 2012(30) | Inclusion Criteria   1. Stable S. Creat.  2. Moderate risk for CI-AKI  3. Elective intervention  serum creatinine and at least moderate risk for CI-AKI   Exclusion Criteria   1. Patients with unstable S. Creat.  2. Recent i.v. administration of Contrast within month of procedure  3. Shock 4. End-stage renal disease 5. Haemodialysis 6. Hypersensitivity to NAC or theophylline 7. Serious cardiac arrhythmias 8. Seizures 9. Acute renal failure | 1. Coronary Angiography 2. PCI | 66 | Elevation of the S. Creat. by ≥0.5mg/dL or ≥25% within 3 days of contrast injection | Iopamidol Low-Oam | 0.9% sodium chloride (1 mL/kg/hr for 24 hours beginning 12 hours before the procedure.  All patients received NAC 600 mg twice daily, on the day before and the day of procedure | Theophylline | 200 mg in 100 mL sodium chloride (0.9%) I.V 30 minutes before Contrast | | Mean±SD:  116.33±59.44 | | | Mean S. Creat. ±SD:  1.54±0.73mg/dL | |
|  |  |  |  |  |  |  | Placebo | 100 mL sodium chloride (0.9%) 30 minutes before the procedure. | | Mean±SD:  117.17 ± 63.13 | | | Mean S. Creat. ±SD:  1.34±0.66mg/dL | |
| Boscheri 2007(31) | Inclusion Criteria   1. Chronic renal failure and stable S. Creat. > 120 µmol/l)  Exclusion Criteria  1. MI within 3 months 2. Cardiogenic Shock 3. Use of Vasopressors 4. Ejection Fraction < 25% 5. Acute renal failure  6. Current peritoneal dialysis or haemodialysis 7. Pregnancy 8. Exposure to contrast dye or medication with NAC up to 72 hours prior to study entry | 1. Coronary Angiography 2. PCI | 151 | ≥ 25% Absolute rise of the S. Creat. day 2 compared to baseline | Iodixanol Iso-Osm | 500 ml of normal saline 2 hours prior to procedure. 500 ml during the procedure and for 6 hours after | Ascorbic acid | 1 g ascorbic acid orally 20 minutes prior to contrast exposure | | Mean ±SD: 99±46 | | | Mean S. Creat. ±SD:  1.75±0.4mg/dL | |
|  |  |  |  |  |  |  | Placebo | 1 g placebo orally 20 minutes prior to contrast exposure | | Mean ±SD: 112±67 | | | Mean S. Creat. ±SD:  1.73±0.4mg/dL | |
| Boucek 2013(32) | Inclusion Criteria   1. >18 Years old 2. Diabetic patients with impaired renal function (Serum creatinine ≥100 mmol/L) 3. Use of low-osmolar contrast media.  Exclusion Criteria   1. End-stage renal disease 2. Chronic dialysis treatment 3. Kidney transplant 4. Pre-planned dialysis following the contrast-involving procedure 5. Emergency procedures 6. Acute kidney injury (serum creatinine increase ≥50 mmol/L during the previous 24-h period) 7. Volume overload with left ventricular failure 8. Uncontrolled hypertension (systolic BP ≥ 180 or diastolic BP ≥ 110 mmHg) 9. Hemodynamic instability (systolic BP <90 and diastolic BP <50 mmHg) 10. Contrast use in the previous 48-h period 11. Multiple myeloma 12. Pregnancy 13. Breastfeeding 14. Pre-planned use of any other measure for CI-AKI prevention | 1. Elective procedure with use of low-osmolar contrast media | 132 | Increase in S. Creat. ≥ 25% and/or ≥ 44 mmol/L within 2 days after contrast administration | Low-Osm | Both groups receive infusion solutions 1 hour pre-procedure at 3 mL/kg/h to maximum 330mL; and for 6 hours after at 1mL/kg/h to maximum of 660 mL | NaHCO3 | 154 mL of 8.4% NaHCO3 add to 846 mL of 5% glucose | | Mean ±SD: 115±47 | | | Mean S. Creat. ±SD:  170±84 mmol/L | |
|  |  |  |  |  |  |  | Control (IV Fluids) | 154 mL of 5.85% NaCl added to 846 mL of 5% Glucose. | | Mean ±SD: 104±32 | | | Mean S. Creat. ±SD:  160±74 mmol/L | |
| Brar 2008(33) | Inclusion Criteria   1. Age ≥ 18 years  2. Estimated GFR ≤60 mL/min/1.73 m2  2. One or more of  a. Diabetes Mellitus b. congestive heart failure c. hypertension d. Age > 75 years  Exclusion Criteria   1. Inability to obtain consent 2. Receipt of NaHco3 infusion prior to randomization 3. Emergency cardiac catheterization 4. Intra-aortic balloon counter-pulsation 5. Dialysis 6. Exposure to radiographic contrast media within the preceding 2 days 7. Allergy to contrast media 8. Acutely decompensated CHF 9. Severe valvular Abnormality 10. Single functioning kidney 11. History of kidney or heart transplantation 12. Change in eGFR ≥ 7.5% per day or cumulative change ≥ 15% in preceeding 2 or more days. | 1.Coronary Angiography | 379 | ≥25% or ≥0.5-mg/dL increase in S. Creat. | Ioxilan Low-Osm | As per intervention protocol | NaHCO3 | 3 mL/kg for 1 hour before coronary angiography, decreased to 1.5 mL/kg/hr during the procedure and for 4 hours after | | Median (IQR): 126 (80-214) | | | Median S. Creat. (IQR), 1.49 (0.36) mg/dL | |
|  |  |  |  |  |  |  | NaCl | 3 mL/kg for 1 hour before coronary angiography, decreased to 1.5 mL/kg/hr during the procedure and for 4 hours after | | Median (IQR): 137 (89-247) | | | Median S. Creat. (IQR), 1.49 (0.36) mg/dL | |
| Briguori 2002(36) | Inclusion Criteria   1. Chronic renal impairment (S. Creat. >1.2 mg/dl and/or eGFR <70 ml/min) 2. Elective procedure  Exclusion Criteria  None specified | 1. Coronary Angiography ± Angioplasty 2. Peripheral Angiography ± Angioplasty | 199 | 1. Increase of ≥25% in S. Creat. 48 hours after the procedure OR 2. Need for dialysis post contrast | Iopromide Low-Osm | 0.45% saline I.V. at 1ml/kg/hour for 12hours pre- and 12 hours post-procedure | NAC | 600 mg orally every 12 hours • before and on day of administration, total of 2 days | | Mean ±SD: 194±127 | | | Mean S. Creat. ±SD:  1.52±0.43 mg/dL | |
|  |  |  |  |  |  |  | Control | I.V fluid only | | Mean ±SD: 200±144 | | | Mean S. Creat. ±SD:  1.54±0.36 mg/dL | |
| Briguori 2004(35) | Inclusion Criteria   1. Elective patients  2. Chronic renal impairment (S. Creat. ≥1.5 mg/dl and/or creatinine clearance <60 ml/min)  3. Stable S. Creat.  Exclusion Criteria  None specified | 1. Coronary Angiography ± Angioplasty 2. Peripheral Angiography ± Angioplasty | 209 | 1. Increase in S. Creat. by ≥0.5mg/dL 48 hours after the procedure OR 2. Need for dialysis post contrast | Iodixanol Iso-Osm | 0.45% saline I.V. at 1ml/kg/hour (or 0.5ml/kg/hour in patients with LVEF<40%) for 12hours pre- and 12 hours post-procedure | NAC | 1200 mg orally every 12 hours • before and on day of administration, total of 2 days | | Mean ±SD: 160±82 | | | Median S. Creat. (IQR): 1.72 (1.55-1.90) mg/dL | |
|  |  |  |  |  |  |  | Fenoldopam | Infusion started at least 1 h pre-procedure at 0.10 µg/kg/min, continued during and for 12 h post-procedure | | Mean ±SD: 168±104 | | | Median S. Creat. (IQR): 1.75 (1.62-2.01) mg/dL | |
| Briguori 2007(34) | Inclusion Criteria   1. Patients ≥18 years 2. Chronic kidney disease (stable S. Creat. ≥2.0 mg/dL and/or estimated glomerular filtration rate <40 mL/min)  Exclusion Criteria   1. S. Creat. ≥8 mg/dL 2. History of dialysis 3. Multiple myeloma 4. Pulmonary oedema 5. Acute MI 6. Recent exposure to radiographic contrast within 2 days of the study 7. Pregnancy 8. Administration of theophylline, dopamine, mannitol, or fenoldopam | 1. Coronary Angiography 2. Peripheral Angiography | 238 | Increase of ≥25% in S. Creat. 48 hours after the procedure | Iodixanol Iso-Osm | All patients received NAC orally at a dose of 1200 mg twice daily on the day before and the day of administration of the contrast agent  3^rd^ Group with mixed treatments excluded | NaCl | Isotonic saline (0.90%) I.V at a rate of 1 mL/kg/hr (0.5 mL/kg for patients with LVEF<40%) for 12 hours before and 12 hours after administration of the contrast agent | | Mean ±SD: 179±102 | | | Median S. Creat. (IQR), 1.95 (1.69-2.26) mg/dL | |
|  |  |  |  |  |  |  | NaHCO3 | 154 mEq/L NaHCO_3_ in dextrose and H_2_O bolus 3ml/kg/h for 1 hour immediately before contrast; then infusion at 1 mL/kg/h during contrast exposure and for 6 hours after the procedure. | | Mean ±SD: 169±92 | | | Median S. Creat. (IQR): 2.04 (1.86-2.36) mg/dL | |
| Brueck 2013(37) | Inclusion Criteria   1. Chronic Renal Impairment (S. Creat. ≥1.3 mg/dL)  2. Elective procedure  Exclusion Criteria   1. Variability of ≥0.3mg/dL in S. Creat. measurements in 7 days pre-procedure 2. Exposure to contrast agents or nephrotoxic medication within week of procedure  3. Renal transplant recipients 4. Patients with Plasmocytoma, Oxalosis, Nephrolithiasis or Hyperthyroidism  5. Inadequate time for study measurements pre-procedure  study procedures 6. Previously known sensitivity to NAC or ascorbic acid. 7. Pregnancy  8. Breast feeding women 9. Child-bearing females not using contraception | 1.Cardiac Catheterization | 622 | 1. Increase in S. Creat. by ≥0.5mg/dL (≥44.2 µmol/L) with 72 hours of procedure | Ultravist Low-Osm | 0.9% sodium chloride (1 mL/kg/hr for 12 hours pre- and 12 hours post-procedure. | I.V. NAC | 600 mg in 250 ml saline (0.9%) over 30 min 24 hours and one hour before contrast exposure | | Median (IQR): 110 (80-160) | | | Median S. Creat. (IQR): 1.5 (1.3-1.8) mg/dL | |
|  |  |  |  |  |  |  | Ascorbic acid | 500mg in 250 ml saline (0.9%) over 30 min 24 hours and one hour before contrast exposure | | Median (IQR): 115 (90-150) | | | Median S. Creat. (IQR): 1.5 (1.3-1.7) mg/dL | |
|  |  |  |  |  |  |  | Control (IV Fluids) | as per fluid protocol | | Median (IQR): 110 (80-150) | | | Median S. Creat. (IQR): 1.5 (1.3-1.7) mg/dL | |
| Burns 2010(38) | Inclusion Criteria   1. Critically ill adult patients 2. ≥ 18 years of age 3. Undergoing contrast-enhanced CT of any organ 4. Patient considered at risk of CI-AKI  Exclusion Criteria   1. CK > 5,000 2. Myoglobinuria 3. Known hypersensitivity to contrast dye or NAC 4. Serious illness with low likelihood of survival within 48-hours or poor prognosis 5. Pregnancy 6. Cardiogenic shock 7. Known or suspected nephritic, nephrotic or pulmonary-renal syndromes 8. Post-renal aetiology of renal impairment 9. Previous renal transplant 10. Known solitary kidney 11. Serum creatinine > 200 µmol/L 12. Recent exposure to radiographic contrast within 14 days of randomization. | Contrast Enhanced CT | 46 | 1. Increase in S. Creat. by > 50 µmol/L from the time of randomization up to day 5 following contrast exposure | not specified | Normal saline 12 hours pre- and 12 hours post CT | I.V. NAC | 5 g in 100 ml D5W before CT, 2.5 g in 50 ml D5W at 6 and 12 hours post-CT | | not specified | | | Mean S. Creat. ±SD:  102±40.9 mmol/L | |
|  |  |  |  |  |  |  | Placebo | 5 g in 100 ml D5W before CT, 2.5 g in 50 ml D5W at 6 and 12 hours post-CT | | not specified | | | Mean S. Creat. ±SD:  118.1±26.3 mmol/L | |
| Carbonell 2007(39) | Inclusion Criteria  1. Patients with ACS syndrome 2. Normal renal function (Stable S. Creat. <1.4 mg/dl (123.76 μmol/l) or a creatinine clearance >60 ml/min)  Exclusion Criteria   1. Hemodynamic instability (Systolic B.P. <90 mm Hg)  2. Known allergy to NAC or contrast agent 3. Untreated gastrointestinal bleeding 4. Current or previous treatment with theophylline, Mannitol or nephrotoxic drugs. | 1. Coronary Angiography | 238 | Increase in S. Creat. ≥0.5 mg/dL (44 µmol/L) or >25% increase above baseline at 48 h post-contrast | Iopromide Low-Osm | As per intervention protocol | I.V. NAC | 600 mg in 50 ml saline (0.9%) over 30 min twice daily for total of 4 doses, starting at least 6 hours pre-procedure | | Mean ±SD: 193±11 | | | Mean S. Creat. ±SD:  0.94±0.16 mg/dL | |
|  |  |  |  |  |  |  | Control (IV Fluids) | 50 mL of 0.9% saline solution for 30 minutes twice daily for a total of 4 doses. | | Mean ±SD: 183±10 | | | Mean S. Creat. ±SD:  0.96±0.17 mg/dL | |
| Carbonell 2010(40) | Inclusion Criteria   1. Patients with chronic kidney disease (S. Creat. ≥1.4 mg/dL (123.76 µmol/L)9 Or <60 mL/min creatinine clearance)  Exclusion Criteria   1. Hemodynamic instability (Systolic B.P. <90 mm Hg)  2. Known allergy to NAC or contrast agent 3. Untreated gastrointestinal bleeding 4. Current or previous treatment with theophylline, Mannitol or nephrotoxic drugs. | 1. Coronary Angiography | 93 | Increase in S. Creat. ≥0.5 mg/dL (44 µmol/L) or >25% increase above baseline at 48 h post-contrast | Iopromide Low-Osm | As per intervention protocol | I.V. NAC | 600 mg in 50 ml saline (0.9%) over 30 min twice daily for total of 4 doses, starting at least 6 hours pre-procedure | | Mean ±SD: 134.79±13 | | | Mean S. Creat. ±SD:  2.01±0.77 mg/dL | |
|  |  |  |  |  |  |  | Control (IV Fluids) | 50 mL of 0.9% saline solution for 30 minutes twice daily for a total of 4 doses. | | Mean ±SD: 184.66±21 | | | Mean S. Creat. ±SD:  1.87±0.7 mg/dL | |
| Castini 2010(4) | Inclusion Criteria   1. Patients aged 18 years or older  2. Stable S. Creat. ≥1.2mg/dL  Exclusion Criteria   1. S. Creat. >4mg/dL 2. History of dialysis 3. Multiple myeloma 4. Pulmonary oedema 5. Cardiogenic shock 6. Acute MI 7. Emergency catheterization  8. Exposure to contrast media within 7 days of the study 9. Allergy to iodinate contrast media or NAC 10. Previous enrolment in the same or other protocols 11. Pregnancy 12. Administration of theophylline, mannitol, dopamine, dobutamine, nonsteroidal anti-inflammatory drugs, or fenoldopam. | 1. Coronary Angiography ± PCI | 117 | CI-AKI1: ≥25% increase in S. Creat. over baseline at any of 3 predefined time-points.  (24 hours, 48 hours and 5 days)  CI-AKI2: Absolute increase of ≥0.5mg/dL in S. Creat at the same time-points   NB : CI-AKI1 used for this analysis | Iodixanol Iso-Osm | As per intervention protocol | NaCl | 1mL/kg/hr for 12 hours pre- and 12 hours post-contrast exposure | | Mean ±SD: 196.4±127.7 | | | Mean S. Creat. ±SD:  1.59±0.38 mg/dL | |
|  |  |  |  |  |  |  | NaHCO3 | 154 mEq/L at 3mL/kg/hr for one 1 hour immediately pre-contrast injection, then 1mL/kg/hr during contrast exposure and for 6 hours post-procedure. | | Mean ±SD: 179.2±125.1 | | | Mean S. Creat. ±SD:  1.59±0.38 mg/dL | |
|  |  |  |  |  |  |  | NAC and IV NaCl | Excluded from analysis | | Mean ±SD: 210.5±140.6 | | | Mean S. Creat. ±SD:  1.57±0.38 mg/dL | |
| Chen 2008(41) | Inclusion Criteria   1. Myocardial ischemia   Patients divided according to S. Creat.: <1.5mg/dL: Normal arm >1.5mg/dL: Abnormal arm before further randomization.   Normal and abnormal arms are considered separately  Exclusion Criteria   1. Coronary anatomy not suitable for PCI 2. Emergency CABG required 3. Patients on peritoneal or haemodialysis 4. Acute MI 5. No written formal consent  6. Patients with normal coronary arteries | 1.PCI | 1185 | Absolute increase in S. Creat of 0.5mg/dl at 48 h post-procedure | Iso-Osm | As per intervention protocol | IV hydration* *Abnormal group received 1200mg NAC 12hours pre-procedure and again post procedure | Both arms: 0.45% saline I.V at 1ml/kg/h starting 12h pre-procedure | | Mean ±SD: Normal Arm: 285±107 Abnormal Arm: 298±125 | | | Mean S. Creat. ±SD:  Normal Arm: 1.3±0.3 mg/dL Abnormal Arm: 2.5±0.1 mg/dL | |
|  |  |  |  |  |  |  | Control (no hydration) * *Abnormal group received 1200mg NAC 12hours pre-procedure and again post procedure | Control Group: 1. Normal Arm: No hydration 2.Abnormal Arm: No Hydration | |  |  |  |  |  |
| Cho 2010(42) | Inclusion Criteria   1. Chronic kidney disease 2. 18 years 3. Stable S. Creat. of 1.1 mg/dL or estimated creatinine clearance <60mL/min  4. Diagnostic, elective Cardiac angiography  Exclusion Criteria   1. Dialysis 2. Multiple myeloma 3. Myeloproliferative disease 4. Current decompensated heart failure or significant change in NYHA symptom Class 5. Current MI 6. Symptomatic hypokalaemia 7. Uncontrolled hypertension (SBP >200mmHg or DBP >100mmHg) 8. Exposure to radiocontrast within 7 days of enrolment 9. Emergency catheterization  10. Allergy to contrast 11. Pregnancy 12. Administration of dopamine, mannitol, fenoldapam or NAC during the study 13. Exacerbation of COPD  14. S. HCO3>28mEq/L 15. S. Sodium <133 mEq/L | 1.Coronary Angiography | 79 | Increase in S. Creat. ≥0.5 mg/dL (44 µmol/L) or >25% increase above baseline at 72 h post-contrast | Isoversol Low-Osm | As per intervention protocol | NaCl | 154 mEq/L at 3mL/kg/hr for one 1 hour immediately pre-contrast injection, then 1mL/kg/hr for 6 hours post-procedure. | |  | | | Mean S. Creat.:  1.38mg/dL | |
|  |  |  |  |  |  |  | NaHCO3 | 154 mEq/L at 3mL/kg/hr for one 1 hour immediately pre-contrast injection, then 1mL/kg/hr for 6 hours post-procedure. | |  | | | Mean S. Creat.:  1.38mg/dL | |
|  |  |  |  |  |  |  | Oral Hydration | 500 mL of water to be started 4 hours prior to contrast exposure and stopped 2 hours prior to procedure and 600 mL of water post-procedure | |  | | | Mean S. Creat.:  1.38mg/dL | |
|  |  |  |  |  |  |  | NaHCO3 and Oral Hydration | Excluded from analysis | | | | | | |
| Cicek 2013(43) | Inclusion Criteria  1. Diabetic patients 2. Elective coronary angiography  Exclusion Criteria   1. S. Creat. >1.5 mg/dL 2. Known malignancy 3. Liver disease 4. Allergy to contrast media 5. Use of any nephrotoxic agent within 48 h 6. Exposure to contrast agent within 7 days 7. Unstable angina 8. Hemodynamically unstable patients 9. Patients requiring PCI | 1.Coronary Angiography | 84 | Increase in S. Creat. ≥0.5 mg/dL (44 µmol/L) or >25% increase above baseline at 48 h post-contrast | Iohexol Low-Osm | saline infusion at 1 mL/kg/h from 3 h before procedure continued for 24 h | Alpha-Lipoic Acid | 600 mg Thioctacid in 3 doses before meal; 30 min pre- and at 24 and 48-hour post-Angiography | | Median (IQR): 75 (50-100) | | | Mean S. Creat. ±SD:  0.86±0.18mg/dL | |
|  |  |  |  |  |  |  | Control | nil | | Median (IQR): 80 (60-120) | | | Mean S. Creat. ±SD:  0.89±0.18mg/dL | |
| Coyle 2006(44) | Inclusion Criteria   1. Age >18 years  2. Diabetes Mellitus  Exclusion Criteria   1. Emergency procedure  2. Inability to complete hydration protocol 3. History of Contrast Nephropathy | 1.Coronary Angiography | 141 | Increase in S. Creat. ≥0.5 mg/dL (44 µmol/L) above baseline at 48 h post-contrast | Mixed: Selection and volume of contrast at the discretion of the operator | Oral hydration with 1litre of clear fluids over 10 hours before angiography.  IV hydration from start of procedure with 0.45% saline at 300ml/hour for 6 hours. | NAC | 600 mg orally every 12 hours • 2 doses before and 2 days after administration of contrast | | Mean ±SD: 88±61 | | | Mean S. Creat. ±SD:  1.16±0.38mg/dL | |
|  |  |  |  |  |  |  | Control | No intervention | | Mean ±SD: 98±65 | | | Mean S. Creat. ±SD:  1.10±0.44mg/dL | |
| Durham 2002(45) | Inclusion Criteria   1. S. Creat. ≥1.7 mg/dL  Exclusion Criteria   1. < 18 years 2. Renal disease with reversible component 3. Unwilling or unable to provide informed consent 4. Inadequate time to perform the study procedures 5. Evidence of active athero-embolic disease 6. Known allergy to NAC 7. Severe asthma 8. Breast feeding 9. Severe peptic ulcer disease 10. Respiratory depression 11. >15% variability in S. Creat. over three days prior to angiography 12. Women of child bearing not using contraception | 1. Coronary Angiography ± PCI | 98 | Increase in S. Creat. ≥0.5 mg/dL (44 µmol/L) | Iohexol Low-Osm | 0.45% saline at 1ml/kg/hr for 12 hours pre-contrast, continuing for 12 hours post-procedure | NAC | 1200 mg orally 1 hour pre- and 3 hours post-contrast | | Mean ±SD: 77.4±35.9 | | | Mean S. Creat. ±SD:  2.2±0.4mg/dL | |
|  |  |  |  |  |  |  | Placebo | 1200 mg orally 1 hour pre- and 3 hours post-contrast | |  | | | Mean S. Creat. ±SD:  2.3±0.5mg/dL | |
| Dussol 2006(46) | Inclusion Criteria  1. Chronic renal failure (creatinine clearance 15-60 ml/min) 2. Radiological procedures necessitating a contrast medium injection   Exclusion Criteria   1. < 18-years 2. Women of child-bearing age not on contraception 3. Breast-feeding women 4. LVEF<30%, 5. Uncontrolled hypertension (BP >180/110mmHg) 6. Obvious extracellular over-hydration 7. Respiratory depression 8. Known prior intolerance to theophylline or furosemide 9. Previous exposure to contrast media in the 14 days before randomization 10. No informed consent | 1.Contrast Enhanced Imaging procedure | 258 | Increase in S. Creat. ≥0.5 mg/dL (44 µmol/L) above baseline at 48 h post-contrast | Low-Osm Contrast medium | 0.9% NaCl 15ml/kg/hr I.V. for 6h pre-procedure. 4th Group use oral Nacl only excluded from this analysis | Furosemide | 3mg/kg intravenously just after the procedure | | Mean ±SD: 119±42 | | | Mean S. Creat. ±SD:  201±81 mmol/L | |
|  |  |  |  |  |  |  | Theophylline | 5mg/kg one hour before the procedure | | Mean ±SD: 133±70 | | | Mean S. Creat. ±SD:  214±113 mmol/L | |
|  |  |  |  |  |  |  | Control | nil extra | | Mean ±SD: 115±57 | | | Mean S. Creat. ±SD:  208±84 mmol/L | |
| Dvorsak 2013(47) | Inclusion Criteria   1. Chronic Renal Impairment with stable S. Creat. >107 mmol/L   Exclusion Criteria   1. Regular vitamin C-containing medication 2. Acute renal failure 3. End-stage renal disease 4. Radiocontrast procedure in the last 3months 5. Cardiogenic shock 6. Acute MI | 1. Coronary Angiography ± PCI | 86 | >25% increase in S. Creat or >25% increase in S. Cystatin C from baseline, 3-4 days post-procedure | Iopamidol Iso-Osm | Normal saline 50–100 mL/h for 2 h pre- and for at least 6 h post-procedure | Ascorbic acid | 3g orally pre-procedure and 2g orally post-procedure and again the next morning | | Mean ±SD: 144.6±86 | | | Mean S. Creat. ±SD:  139.4±24 mmol/L | |
|  |  |  |  |  |  |  | Placebo | 3g orally pre-procedure and 2g orally post-procedure and again the next morning | |  | | | Mean S. Creat. ±SD:  133.3±30.9 mmol/L | |
| Efrati 2003(48) | Inclusion Criteria   1. S. Creat > 106µmol/L  2. Elective coronary angiography  Exclusion Criteria   1. Acute renal failure 2. Acute MI 3. Non-compensated CHF 4. Hemodynamic instability 5. Known sensitivity to contrast media | 1.Coronary Angiography | 51 | Increase of ≥25% in S. Creat. 96 hours after the procedure | Ultravist Low-Osm | 0.45% saline at 1ml/kg/hr for 12 hours pre-contrast, continuing for 12 hours post-procedure | NAC | Mucomyst 20% solution diluted in 30ml of regular Coca-Cola, as 1 g orally twice daily 24 hours pre- and 24 hours post-angiography | | Mean ±SD: 142±25.3 | | | Mean S. Creat. ±SD:  135.25±6.19 mmol/L | |
|  |  |  |  |  |  |  | Placebo | Regular Coca Cola alone orally twice daily 24 hours pre- and 24 hours post-angiography | | Mean ±SD: 138±33.7 | | | Mean S. Creat. ±SD:  131.7±6.19 mmol/L | |
| Er, F. 2012 | Inclusion Criteria  1.> 18 years of age  2. Presented with stable angina Pectoris with  4. Renal function test revealed impaired renal function (elevated serum creatinine of > 1.4 mg/dL or reduced eGFR <60 mL/min/1.73 m^2^,)  Exclusion Criteria:  Patients who do not meet inclusion criteria | 1.Coronary Angiography | 126 | Increase in serum creatinine≥25% or≥ 0.5 mg/dL above baseline at 48 hours after contrast medium exposure | Iohexol  Low-Osm |  | RIPC | Intermittent arm ischemia through 4 cycles of 5-minute inflation and 5-minute deflation of a blood pressure cuff started immediately before procedure. | | Mean ±SD: 124±44 | | | Mg/dl  1.63 (1.47–1.81) | |
|  |  |  |  |  |  |  | Placebo | Sham (RIPC)) | | Mean ±SD: 103±41 | | | Mg/dl  1.62 (1.39–1.93) | |
| Eric Chong 2015(190) | Inclusion Criteria:  adults >21 years of age with a glomerular filtration rate (GFR) of 15-60mL/min/1.73m2 and who were able to receive pre-hydration for 12 h  Exclusion Criteria:  1. End-stage renal failure with GFR of <15 mL/min/1.73 m2,  2. Acute renal failure with a >44 μmol/L increase in serum Cr levels in the previous 24 h,  3. Pre-existing dialysis, pulmonary oedema or moderate to severe congestive heart failure (New York Heart Association III–IV).  4. Inability to withstand the fluid load and presence of haemodynamic compromise, uncontrolled hypertension (untreated systolic blood pressure >160mmHg, or diastolic blood pressure >100mmHg)  5. Emergency cardiac catheterisation  6. Exposure to contrast or study drugs within the last 48  7. Allergies to contrast or trial medications  8. Clinical conditions requiring  continuous fluid therapy  9. Potentially renal-toxic  Drugs within 48 h of cardiac catheterisation and throughout the study | 1.Elective Cardiac catheterization | 331 | ≥25% increase of serum Cr concentration or a ≥44 μmol/L (0.5mg/dL) increase in serum Cr within 48 h of cardiac catheterisation or PCI [ | - | See Groups | NAC | | 154mEq/L sustained sodium chloride regime (1 mL/kg/h 12 h before, during and 6 h after the procedure) with oral NAC at 1.2 g bid for 3 days | Contrast(ml) Mean(SD):  116 (83.5) | | S. Creat.  Mean(SD):  134.0 (35.5) | | |
|  |  |  |  |  |  |  | NaHco3 | | 154 mEq/L abbreviated SOB regime at 3 mL/kg/h 1 h before the procedure, and 1mL/kg/h during and 6 h after the procedure | Contrast(ml) Mean(SD):  115 (85.4) | | S. Creat.  Mean(SD):  141.8 (44.8) | | |
|  |  |  |  |  |  |  | Combination (Excluded) | | combination | Contrast(ml) Mean(SD):  116 (84.5) | | S. Creat.  Mean(SD):  138.7 (36.6) | | |
| Erley 1999(50) | Inclusion Criteria:   1. Chronic renal insufficiency (S. Creat. >1.5 mg/dl )  2. Receive ≥ 80 ml low-osmolality contrast media (Iopromide)  Exclusion Criteria:  1. Allergy to contrast media or theophylline 2. Pregnancy 3. Uncontrolled hypertension 4. Severe heart failure 5. Liver failure 6. Nephrotic syndrome | 1.CT Digital Subtraction Angiography | 67 | Increase in S. Creat. ≥0.5 mg/dL (44 µmol/L) | Iopromide Low-Osm | All patients received hydration with 2–2.5 ml fluid (either orally as mineral water or I.V with 0.45% saline) starting at least 24 h before and continued for 24 hours post-contrast | Theophylline | 810 mg daily in divided doses, 270mg mané and 540mg tardé started 2 days before and continued until 3 days post-contrast | | Mean ±SD: 118±48 | | | Mean S. Creat. ±SD:  1.9±0.5mg/dL | |
|  |  |  |  |  |  |  | Placebo | Divided doses, mané and tardé started 2 days before and continued until 3 days post-contrast | | Mean ±SD: 110±44 | | | Mean S. Creat. ±SD:  1.7±0.4mg/dL | |
| Erol 2013(51) | Inclusion Criteria   1. S. Creat >1.1mg/dL 2. cardiac catheterization  Exclusion Criteria   1. Acute myocardial infarction requiring PCI within 24h 2. Cardiogenic shock 3. Acute renal failure 4. Current or planned peritoneal or hemo-dialysis 5. Previous administration of contrast agents or anticipated re-administration of contrast agents within the following 4 days. | 1.Coronary Angiography / PCI | 165 | Increase of ≥25% in S. Creat. 48-96 hours after the procedure | Iohexol Low-Osm | 1mg /kg/h saline for 12 h pre- and post-contrast | Allopurinol | 300mg orally 24 h before administration of contrast agent | | Mean ±SD: 121±25 | | | Median S. Creat. (IQR): 1.43(1.1-4.15) mg/dL | |
|  |  |  |  |  |  |  | Control | fluid only | | Mean ±SD: 119±26 | | | Median S. Creat. (IQR): 1.48(1.1-2.96) mg/dL | |
| Erturk 2014(52) | Inclusion Criteria   1. Age > 21years 2. eGFR <60ml/min/1.732  3. Elective intra-arterial procedure  Exclusion Criteria    1. Dialysis  2. eGFR <15ml/min/1.732 3. Uncontrolled HTN 4. Radiocontrast medium within 7 days of trial 5. Acute/chronic inflammatory disease 6. NSAID or Metformin up to 2 days before procedure 7. Pregnancy 8. Allergy to contrast or NAC 9. Patients receiving Fenoldopam, Mannitol, Dopamine and Theophylline | 1. Coronary Angiography 2. PCI 3. Peripheral intervention | 225 | >25% increase in S. Creat or >25% increase in S. Cystatin C from baseline, 48hours post-procedure |  | 0.9% normal saline for 12 hours pre- and 12 hours post-procedure | Intravenous NAC | 2400mg 1 hour pre-procedure and 4800mg 4-6 hours post-procedure **for this Analysis only I.V NAC included | | Mean ±SD: 122±67 | | | eGFR 30-59ml:  n=97  eGFR 15-29ml:  n=5 | |
|  |  |  |  |  |  |  | Oral NAC | 1200 mg orally every 12 hours for 24 hours pre- and 48 hours post-procedure | | Mean ±SD: 127±89 | | | eGFR 30-59ml:  n=95  eGFR 15-29ml:  n=7 | |
|  |  |  |  |  |  |  | IV hydration | normal saline only | | Mean ±SD: 127±66 | | | eGFR 30-59ml:  n=92  eGFR 15-29ml:  n=11 | |
| Eshraghi 2017(191) | Inclusion Criteria:  Patients with ST elevation MI and above 18 years of age who referred for emergency angioplasty  Exclusion Criteria:  1. Serum creatinine more  than 1.5 mg/dl  2. heart failure, history of end-stage renal failure or being on dialysis  3. Use of NAC, theophylline, aminoglycosides non-steroidal anti-inflammatory medicines  4. I.V contrast media administration within the last 2 days.  5. Pulmonary edema, multiple myeloma, and uncontrolled hypertension | 1.Emergency coronary angioplasty | 189 | 0.5 mg/dl or 25% increase in serum creatinine above the baseline, 48 hours after exposure to contrast media | Iodixanol  Iso-Osm | Normal saline 1-1.5 cc/kg was administered from 6 hours before to 6 hours after procedure | Pentoxifylline | | 400 mg 3 times a day from referral day until 24 hours after the procedure | Mean+SD:  190.88 ± 75.82 | | S. Creat.  Mean+SD:  1.147 ±0.424 | | |
|  |  |  |  |  |  |  | Control | | I.V hydration only | Mean+SD:  231.29 ± 105.10 | | S. Creat.  Mean+SD:  1.12 ±0.26 | | |
| Fan, Y 2016(192) | Inclusion Criteria:  Patients> 18 years with an estimated glomerular filtration rate (eGFR) of 60 mL/min or less undergoing elective cardiac catheterization  Exclusion Criteria:  1. cardiogenic shock  2. Aute ST-segment elevated MI requiring primary PCI  3. eft ventricular ejection fraction <30%  4. Allergy to contrast agent or nicorandil  5. Previous contrast media exposure within 1 week  6. Uraemia or renal failure receiving dialysis  7. Administration of NAC, metformin, or NaHco3 within 48 h of the procedure | 2.Elective coronary angiography | 269 | 25 % increase in serum creatinine from baseline or 44 µmol/L (0.5 mg/dL) increase in absolute value within 72 h after exposure to contrast medium | Ultravist  Low-Osm | 0.9 % saline at a rate of 1 mL/kg/h (0.5 mL/kg/h for patients with LVEF <40 %) at least 6 h before and 12 h after procedure | Nicorandil | | 10 mg three times daily from 2 days before to 3 days after contrast media exposure | Mean+SD:  145.3 ± 51.6 | | S. Creat.  Mean+SD:  123.55 ± 10.77 | | |
|  |  |  |  |  |  |  | Placebo | | Matching Placebo | Mean+SD:  149.2 ± 57.0 | | S. Creat.  Mean+SD:  122.99 ± 10.39 | | |
| Ferrario 2009(53) | Inclusion Criteria   1. Age >18 years  2. Elective coronary and/or peripheral angiography and/or angioplasty 3. Creatinine clearance <55ml/min with only ±10% variation in S. Creat. 3-30 days before the procedure.  Exclusion Criteria   1. NYHA symptoms class III to IV 2. Ongoing acute myocardial infarction 3. Acute coronary syndrome 4. Renal replacement therapy 5. Allergy to NAC 6. Need for theophylline, dopamine, fenoldopam, mannitol or nephrotoxic drugs within 1 week of the procedure 7. Presence of clinical signs of dehydration and systemic hypotension. | 1.Coronary / Peripheral Angiography ± Angioplasty | 214 | Increase in S. Creat. ≥0.5 mg/dL (44 µmol/L) or >25% increase above baseline within 3 days of contrast | Iodixanol Iso-Osm | Normal saline (0.9%) at 1ml/kg/h in the 12–24 h pre- and 24h post-procedure.  Oral clear fluid intake was not restricted pre- or post-procedure. | NAC | 600 mg orally every 12 hours on day pre- and day of procedure | | Mean ±SD: 180±104.4 | | | Mean Creat. Clearance±SD:  37±11.5 ml/min | |
|  |  |  |  |  |  |  | Placebo | Glucose tablet orally every 12 hours on day pre- and day of procedure | | Mean ±SD: 168±103.3 | | | Mean Creat. Clearance±SD:  40±9.3 ml/min | |
| Firouzi 2012(54) | Inclusion Criteria   1.Non-emergent coronary angiography and Intervention  Exclusion Criteria   1. S. Creat. >2mg/dl 2. Acute MI 3. Unstable coronary syndrome 4. Cardiogenic shock 5. End-stage renal failure  6. Dialysis 7. Use of NAC 8. History of intravenous contrast media administration within the previous 10 days | 1.Angiography / PCI | 318 | Increase in S. Creat. ≥0.5 mg/dL (44 µmol/L) or >25% increase above baseline within 48 hours of contrast | Iodixanol Iso-Osm Iopromide Iso-Osm | Normal saline 1–1.5 cc/kg from 6 h before to 6 h after procedure | Pentoxyfylline | 400 mg three times a day from 24 h pre- to 24h post-procedure | | Mean ±SD: 319.28±98.1 | | | Mean S. Creat. ±SD:  1.17±0.22mg/dL | |
|  |  |  |  |  |  |  | Control | IV hydration only | | Mean ±SD: 325.34±101.41 | | | Mean S. Creat. ±SD:  1.21±0.24mg/dL | |
| Firouzi 2015(55) | Inclusion Criteria   1. Acute MI 2. Age >20 years  Exclusion Criteria   1. S. Creat. > 2mg/dl 2. Cardiogenic shock 3. LVEF <40% 4. End-stage renal failure 5. Dialysis 6. N-acetylcysteine use 7. History of intravenous contrast media administration within 10 days 8. Metabolic disorder with impairment of serum magnesium level. | 2.PCI | 152 | Increase in S. Creat. ≥0.5 mg/dL (44 µmol/L) or >25% increase above baseline within 48 hours of contrast | Iso-Osm Iodixanol or  Low-Osm iopromide | Normal saline infusion 1–1.5 cc/kg from start of procedure until 6 h post-procedure | MgSO4 | 1 g just before the procedure | | Mean ±SD: 319.28±98.1 | | | Mean S. Creat. ±SD:  1.17±0.22mg/dL | |
|  |  |  |  |  |  |  | Control | IV hydration only | | Mean ±SD: 325.34±101.41 | | | Mean S. Creat. ±SD:  1.21±0.24mg/dL | |
| Fung 2004(56) | Inclusion Criteria   1. Moderate to severe renal insufficiency (S. Creat 1.69-4.52 mg/dL) 2. Elective coronary angiography and/or intervention  Exclusion Criteria    1. Cardiogenic shock 2. Current dialysis therapy 3. Use of dopamine, theophylline, or mannitol 4. Allergy to NAC or contrast agents | 1.Coronary Angiography / PCI | 105 | Increase in S. Creat. ≥0.5 mg/dL (44 µmol/L) or > 25% reduction in eGFR 48 hours of contrast | Iopromide Low-Osm | Normal saline at 100 mL/h from 12 hours pre- until 12 hours post-procedure | NAC | 400 mg, thrice daily the day pre- and day of contrast procedure | | Mean ±SD: 135.8±66.6 | | | Mean S. Creat. ±SD:  2.27±0.54mg/dL | |
|  |  |  |  |  |  |  | Control | IV hydration only | | Mean ±SD: 121±66.2 | | | Mean S. Creat. ±SD:  2.37±0.61mg/dL | |
| Gare 1999(57) | Inclusion Criteria   1. Mild-moderate chronic renal failure AND/OR  2. Diabetes Mellitus   Exclusion Criteria   1. Severe renal insufficiency (S. Creat. >200 µmol/l) 2. Acute coronary events (MI or unstable angina) <48h pre-procedure 3. Known intolerance to dopamine 4. Allergy to contrast material 5. Pheochromocytoma | 1.Coronary angiography | 72 | Increase in S. Creat. by 40% from baseline after the injection of the radiocontrast agents | Lopromide Low-Osm | 0.45% Saline/D5W for 8-12h pre-and 36-48h post-angiography at 100 ml/h (increased depending on urinary output). | Dopamine | 120ml of 0.9% saline plus 2mg/kg dopamine per day for 48h | | Mean ±SD: 173.8±13 | | | Mean S. Creat. ±SD:  100.3±5.4 mmol/L | |
|  |  |  |  |  |  |  | Control | IV hydration only | | Mean ±SD: 163.2±13.2 | | | Mean S. Creat. ±SD:  100.6±5.2 mmol/L | |
| Geng 2012(58) | Inclusion Criteria   1. Type 2 DM 2. eGFR ≤60 mL/min   Exclusion Criteria  1. Allergy to contrast media 2. Contraindication for Anisodamine 3. Emergency Cardiac Catheterization 4. Congestive Heart Failure 5. Intra-Aortic Balloon Counter-pulsation 6. Dialysis 7. Exposure to Contrast media within the last 7 days | 1.Coronary Angiography / PCI | 299 | Increase in S. Creat. ≥0.5 mg/dL (44 µmol/L) or >25% increase above baseline within 3 days of contrast | Ultravist Low-Osm | Saline at 1ml/kg/hr 12 hours pre- and 12 hours post-procedure | Anisodamine | 0.2 µg/kg/min 12 hours pre- and continued for 12 hours post-procedure | | Mean ±SD: 119±25 | | | Mean S. Creat. ±SD:  120.9±19.2 mmol/L | |
|  |  |  |  |  |  |  | Control | IV hydration only | | Mean ±SD: 124±23 | | | Mean S. Creat. ±SD:  122±19.5 mmol/L | |
| Goldenberg 2004(59) | Inclusion Criteria   1. Chronic renal insufficiency (S. Creat. ≥1.5mg/dl or creatinine clearance <50ml/min)  Exclusion Criteria   1. Acute renal failure 2. Acute MI requiring primary or rescue PCI within 12hours 3. Cardiogenic shock 4. Current peritoneal or haemodialysis 5. Planned post-contrast dialysis 6. Known allergy to acetylcysteine | 1.Coronary Angiography / PCI | 87 | Increase in S. Creat. ≥0.5 mg/dL (44 µmol/L) within 48 hours | Iopamidol Low-Osm | 0.45% Saline at 1ml/kg/hr 12 hours pre- and 12 hours post-procedure | NAC | 600 mg orally thrice daily for 2 days, starting 24h pre-procedure | | Mean ±SD: 111±43 | | | Mean S. Creat. ±SD:  2±0.4mg/dL | |
|  |  |  |  |  |  |  | Placebo | placebo orally thrice daily for 2 days, starting 24h pre-procedure | | Mean ±SD: 121±49 | | | Mean S. Creat. ±SD:  1.9±0.3mg/dL | |
| Gomes 2005(61) | Inclusion Criteria   1. S. Creat > 106.08mol/l, creatinine clearance <50ml/min, or drug treated diabetes mellitus 2. Elective intervention   Exclusion Criteria   1. Age <18 years 2. Use of contrast media within 21 days of randomisation 3. Current dialysis 4. Haemodynamic instability before the procedure (SBP ≤ 90 mm Hg or DBP≤ 60 mm Hg) 5. History of sensitivity to N-acetylcysteine. | 1.Coronary Angiography / PCI | 172 | Increase in S. Creat. ≥0.5 mg/dL (44 µmol/L) within 48 hours | Ioxaglate Low-Osm | 0.9% Saline at 1ml/kg/hr 12 hours pre- and 12 hours post-procedure | NAC | 600 mg orally twice daily for 2 days, starting 24h pre-procedure (2 doses pre- and 2 doses post-procedure) | | Mean ±SD: 102.5±47.3 | | | Mean S. Creat. ±SD:  123.76±45.08 mmol/L | |
|  |  |  |  |  |  |  | Placebo | placebo orally twice daily for 2 days, starting 24h pre-procedure (2 doses pre- and 2 doses post-procedure) | | Mean ±SD: 102.8±60.4 | | | Mean S. Creat. ±SD:  111.38±30.94 mmol/L | |
| Gomes 2012(60) | Inclusion Criteria   1. S. Creat. ≥ 1.2 mg/dL or eGFR < 50mL/min  Exclusion Criteria   1. Age < 18 years 2. Use of contrast media during the last 21 days 3. History of dialysis 4. Cardiac insufficiency (NYHA III-IV) 5. Emergency procedures. | 1.Coronary Angiography / PCI | 319 | Increase in S. Creat. ≥0.5 mg/dL (44 µmol/L) within 48 hours | Ioxaglate Low-Osm | IV bolus of assigned fluid at 3 mL/kg/h for 1 hour immediately pre-contrast.  IV hydration to continue at 1mL/kg/hr during and for 6 hours post-procedure | NaHCO3 | 154 mEq/l in D5W | | Mean ±SD: 124±65 | | | Mean S. Creat. ±SD:  1.5±0.4mg/dL | |
|  |  |  |  |  |  |  | NaCl | 154 mEq/l in D5W | | Mean ±SD: 125±87 | | | Mean S. Creat. ±SD:  1.49±0.5mg/dL | |
| Gu 2013(62) | Exclusion Criteria   1. NYHA stage IV heart failure | 1.Coronary Angiography / PCI | 859 | Increase in S. Creat. ≥0.5 mg/dL (44 µmol/L) or >25% increase above baseline within 48 hours of contrast |  | 1/ml/kg/h saline for 4 hr pre- and for 24 hr post-procedure | Furosemide | 20mg over 30 seconds | | Mean ±SD: 100±40 | | | Mean S. Creat. ±SD:  91±21.3 mmol/L | |
|  |  |  |  |  |  |  | Control | IV hydration only | | Mean ±SD: 100±40 | | | Mean S. Creat. ±SD:  89.2±17.6 mmol/L | |
| Gulel 2005(63) | Inclusion Criteria   1. Elective coronary intervention 2. Chronic renal impairment (stable S. Creat. > 1.3 mg/dl)   Exclusion Criteria   1. Acute renal failure  2. End stage Renal failure on regular dialysis 3. Clinically evident heart failure  4. Allergy against contrast medium 5. Serious hepatic dysfunction 6. Planned for percutaneous coronary intervention | 1.Diagnostic Coronary Angiography | 55 | Increase in S. Creat. ≥0.5 mg/dL (44 µmol/L) within 48 hours | Ioxaglat Low-Osm | 0.9% Saline at 1ml/kg/hr 12 hours pre- and 12 hours post-procedure | NAC | 600 mg orally twice daily for 2 days, starting 24h pre-procedure | |  | | | Mean S. Creat. ±SD:  1.6±0.4mg/dL | |
|  |  |  |  |  |  |  | Control | IV hydration only | |  | | | Mean S. Creat. ±SD:  1.8±0.6mg/dL | |
| Gunebakmaz 2012(64) | Inclusion Criteria   1. Patients scheduled for coronary angiography and ventriculography 2. S. Creat. ≥1.2 mg/dL  Exclusion Criteria   1. Dialysis 2. Recent exposure to contrast media or a nephrotoxic agent within 7 days before the study 3. Urgent PCI 4. Requiring loop diuretics, theophylline, aminophylline or dopamine throughout the study 5. Hemodynamically unstable patients 6. Contraindications for β-blocker prescription. | 1.Coronary Angiography / PCI | 148 | Increase in S. Creat. ≥0.5 mg/dL (44 µmol/L) or >25% increase above baseline within 2-5 days of contrast | Iopromide Low-Osm | Intravenous isotonic saline at 1mL/kg/hour, for 6 hours pre- and 12 hours post-procedure | NAC | 600 mg orally twice daily for 4days, 4 doses pre-procedure, 2 doses day of procedure and 2 doses day post-procedure | | Mean: 63.4 | | | Mean S. Creat. ±SD:  1.42±0.13mg/dL | |
|  |  |  |  |  |  |  | Nebivolol | 5mg every 24 hours for 4 days: 2 doses pre-procedure, 1 dose day of procedure, and 1 dose day post- procedure | | Mean: 61.8 | | | Mean S. Creat. ±SD:  1.4±0.11mg/dL | |
|  |  |  |  |  |  |  | Control | IV hydration only | | Mean: 64.2 | | | Mean S. Creat. ±SD:  1.43±0.14mg/dL | |
| Gupta 1999(65) | Inclusion Criteria   1.Diabetes Mellitus    Exclusion Criteria  1. S. Creat. >6mg/dL 2. Patients already on ACEI 3. Contraindication to ACEI 4. LVEF < 30% 5. Known allergy to contrast media | 1.Coronary Angiography | 83 | Increase in S. Creat. ≥0.5 mg/dL (44 µmol/L) within 24 hours |  | Dextrose saline (with insulin) at 1ml/kg/hour started 3 hours pre- and continued for 6 hours post-procedure | Captopril | 25mg po q8h started 1h pre-contrast, continued for 3 days | | Mean ±SD: 116.6±11.4 | | | Mean S. Creat. ±SD:  1.38±0.27mg/dL | |
|  |  |  |  |  |  |  | Control | IV hydration only | | Mean ±SD: 118.4±9.3 | | |  | |
| Hafiz 2012(66) | Inclusion Criteria   1. Chronic kidney disease (S. Creat. >1.6mg/dl in non-diabetics and >1.4 mg/dl in diabetics) or an estimated glomerular filtration rate (eGFR) of <50 ml/min/1.73 m2  2. Age>18 years   Exclusion Criteria   1. Dialysis 2. Unstable renal function (>0.4 mg/dl change in S. Creat. 48 hr pre-procedure) 3. Pulmonary oedema 4. S. bicarbonate >34 mmol/L 5. Received Fenoldopam, Mannitol, Dopamine, or NAC within 48 hr pre-procedure 6. Cardiogenic shock 7. Allergic to contrast media 8. Pregnancy 9. Unable to provide informed consent. | 1. Coronary Angiography 2. PCI 3. Peripheral intervention | 176 | Increase in S. Creat. ≥0.5 mg/dL (44 µmol/L) or >25% increase above baseline within 48 hours of contrast | Iodixanol Iopamidol Ioversol All are: Low-Osm | As per intervention protocol | NaCl | Normal Saline 1ml/kg/hr for 12h pre- and 12h post-procedure | | Median (IQR): 100 (80-140) | | | Mean S. Creat.: 1.6mg/dL | |
|  |  |  |  |  |  |  | NaHCO3 | D5W containing 154 mEq/L of NaHCO3 at 3ml/kg/hr for 1 hr pre-procedure, 1ml/kg/hr for 6 hr post-procedure | | Median (IQR): 110 (75-155) | | | Mean S. Creat.: 1.7mg/dL | |
| Han  2014(67) | Inclusion Criteria   1. Type 2 DM 2. Chronic Kidney disease (stage 2 or 3)   Exclusion Criteria   1. Hypersensitivity to contrast medium or statins 2. Type 1 DM 3. Ketoacidosis 4. Lactic acidosis 5. CKD stages 0/1/4/5 6. Acute STEMI within preceding 4 weeks 5. NYHA Class IV heart failure 6. Hemodynamic instability 7. Administration of iodinated contrast medium during the 2 weeks before randomization 8. LDL Cholesterol <1.82mmol/L 9. Hepatic dysfunction (S ALT >3xnormal) 10. Thyroid insufficiency 11. Renal artery stenosis (unilateral>70% or bilateral >50%) | 1. Coronary Angiography 2. PCI 3. Peripheral intervention | 3095 | Increase in S. Creat. ≥0.5 mg/dL (44 µmol/L) or >25% increase above baseline within 72 hours of contrast | Iodixanol Iso-Osm | 0.9% saline at 1 ml/kg/h from 12h pre- until 24h post-procedure (physician’s discretion allowed) | Statin | Rosuvastatin 10mg tardé from 2 days pre- until 3 days post-procedure (total dose 50 mg over 5 days) | | Median (IQR): 120 (100-200) | | |  | |
|  |  |  |  |  |  |  | Control | no statin | | Median (IQR): 110 (100-200) | | | Mean S. Creat. ±SD:  94.95±20.84 mmol/L | |
| Hashemi 2005(68) | Exclusion Criteria   1. Amount of contrast used <100 or >300 mls 2. Calcium antagonists, ACE-I, theophylline prescribed within 2 days before the procedure 3. Baseline S. Creat. > 2mg/dl. | 1.Coronary Angioplasty | 98 | Increase in S. Creat. ≥0.5 mg/dL (44 µmol/L) within 48 hours | Meglumin compound (76%, DarooPakhsh, Iran) (370 mg/ 20 ml) | Normal saline 0.9% infusion (total volume of 1.5L) at 60 ml/hr from 12hr pre- until 12 hr post-procedure. | Captopril | 12.5mg po q8h started 2h pre-contrast, continued for 48hr | | Mean ±SD: 225±120 | | | Mean S. Creat. ±SD:  0.98±0.43mg/dL | |
|  |  |  |  |  |  |  | Placebo | placebo po q8h started 2h pre-contrast, continued for 48hr | | Mean ±SD: 223.3±130 | | | Mean S. Creat. ±SD:  1.05±0.39mg/dL | |
| Healy, D 2015(193) | Inclusion Criteria:  Patients aged over 17 years scheduled for abdomino-pelvic CECT-scans who were likely to remain in hospital for at least two days after the scan  Exclusion Criteria:  1. Allergy or hypersensitivity to iodinated contrast  2. Hospital admission SCr >150 µmol/dL (a contraindication to iodinated contrast)  3. Renal transplant  4. History of acute renal failure that required management by a nephrologist  5.Current use of either sulphonlyurea or nicorandil. | 1.Contrast enhanced CT | 98 | increase of >25% or an increase of ≥0.5 mg/dl in SCr from the baseline value 48 h after exposure to the contrast medium | Iohexol  Low-Osm  Iopamidol  Iso-Osm  Iodixanol  Iso-Osm | Use of hydration prior to the procedure was at the discretion of the physician who ordered the scan. | RIPC | 40 minutes before contrast was given five-minute cycles of arm ischemia with three minutes of reperfusion between by inflating blood pressure cuff positioned on the patient’s arm to a pressure of 200 mmHg or 15 mmHg above systolic pressure if that was >200 mmHg | | Most patients receive a dose of 90 mL, but patients heavier than 110 kg may receive 120 mL. All patients with eGFR <60 mL/min/1.73m2 receive iodixanol | | | Median (IQR)  73 (59, 85) | |
|  |  |  |  |  |  |  | Control | No intervention | |  | | | Median (IQR)  75 (62, 85) | |
| Heguilen 2013(69) | Inclusion Criteria   1. Age>18 years 2. Stable S. Creat. ~1.25 mg/dl (110 lmol/l) 3. eCr. Clearance <45 ml/min   Exclusion Criteria   1. S. Creat. 4.5 mg/dl ([364.5 lmol/l) 2. Change in S. Creat. ≥0.5 mg/dl (44.2 lmol/l) within the previous week 3. Emergency catheterization 4. Pulmonary oedema 5. Pre-existing dialysis 6. Recent exposure to contrast media 7. Multiple Myeloma 8. Uncontrolled hypertension 9. Hemodynamic instability 10. Current treatment with dopamine, mannitol, Fenoldopam, Aminophylline, Theophylline, Ascorbic acid or NAC 11. Pregnant or childbearing women 12. Known hypersensitivity to Contrast media or NAC 13. Patients undergoing interventions limited to the major renal vasculature | 1.Contrast Study including cardiac catheterization or arteriography | 101 | >25% increase above baseline S. Creat. within 48 -72 hours of contrast | Ioversol Low-Osm | As per intervention protocol | NaHCO3 | D5W containing 154 mEq/L of NaHCO3 at 3ml/kg/hr for 2 hr pre-procedure, 1ml/kg/hr during and for 6-12 hr post-procedure | | Mean ±SD: 179.8±14.2 | | | Mean S. Creat. ±SD:  1.57±0.03mg/dL | |
|  |  |  |  |  |  |  | NAC | 600 mg orally twice daily day preceding and day of procedure | | Mean ±SD: 186.6±15.4 | | | Mean S. Creat. ±SD:  1.60±0.07mg/dL | |
|  |  |  |  |  |  |  | Both NAC + NaHCO3 | Excluded from analysis | | | | | | |
| Heng 2008(70) | Inclusion Criteria   1. Stable CRF (eGFR <56ml/min and S. Creat variability <0.1mg/dl between levels measured 1-2 months before procedure and the baseline level taken within 24 hours before intervention  Exclusion Criteria   1. Age <18 years 2. Pregnancy 3. Allergy to NAC 4. Dialysis 5. Acute Renal Failure 6. Haemodynamic instability 7. Overt Congestive cardiac failure 8. Administration of Iodinated Contrast media, nephrotoxic agent or NAC within the last 30 days prior to inclusion 9 Patients on nephrotoxic agents such as NSAIDs or aminosides | 1.Coronary Angiography / PCI | 65 | Increase in S. Creat. ≥0.5 mg/dL (44 µmol/L), >25% increase S. Creat. above baseline or >5ml decrease in eGFR within 48 hours of contrast | Iomeprol Low-Osm Iodixanol Iso-Osm | Bicarbonate saline 1.4% I.V at 1ml/kg/hr for 12 hours pre- and 12hr post-procedure (6 hours for patients with heart failure) | NAC | 1200 mg orally twice daily day preceding and day of procedure | | Mean ±SD: 208±70 | | | Mean S. Creat. ±SD:  178±53mmol/L | |
|  |  |  |  |  |  |  | Placebo | placebo orally twice daily day preceding and day of procedure | | Mean ±SD: 198±76 | | | Mean S. Creat. ±SD:  193±76mmol/L | |
| Hoole 2009(71) | Inclusion Criteria   1. Age ≥18 years  2. Elective PCI 3. Able to give informed consent  Exclusion Criteria   1. Emergency PCI 2. Elevation of cTnI pre-PCI 3. Women of child-bearing age 4. Nicorandil or glibenclamide use  5. Severe comorbidity (estimated life expectancy <6 months) | 1.PCI | 218 | >25% increase above baseline S. Creat. within 24 hours of contrast | Iopromide Low-Osm |  | RIPC | Blood pressure cuff placed around their non-dominant upper arm. The cuff inflated to 200-mm Hg pressure for 5 minutes, followed by 5 minutes of deflation and repeated 2 more times to 3 cycles in total | | Mean ±SD: 196.7±80.1 | | | eGFR, median (IQR):  72.0 (55.8–86.0) | |
|  |  |  |  |  |  |  | Placebo | Placebo Group: similar cuff placed around the upper arm, but it was not inflated | | Mean ±SD: 187.5±74.2 | | | eGFR, median (IQR):  75.5 (65.3–89.8) | |
| Hsu 2012(72) | Inclusion Criteria   1. Adult patients undergoing abdominal or chest contrast-enhanced CT in Emergency Department  Exclusion Criteria   1. long-term haemodialysis 2. Peritoneal dialysis 3. Received another dose of contrast medium within 72 hours 4. Refusal to sign consent forms 5. Known allergy to NAC | 1.Contrast Enhanced CT | 241 | Increase in S. Creat. ≥0.5 mg/dL (44 µmol/L) or >25% increase above baseline within 48-72 hours of contrast | Iohexol Iobitridol Iopromide All are: Low-Osm | 3 mL/kg/h of 0.9% NaCl for 1hr pre-CT, continued at 1mL/kg/hr for 6hr post-CT (decreased to 0.5mL/kg/hr in patients with clinical evidence of CHF) | Intravenous NAC | 600 mg of NAC in 0.9% sodium chloride at 3 mL/kg for 1hr pre-contrast | | Mean ±SD: 91.1±10.0 | | | Mean S. Creat. ±SD:  1.40±0.58mg/dL | |
|  |  |  |  |  |  |  | Control | IV hydration only | | Mean ±SD: 88.1±10.0 | | | Mean S. Creat. ±SD:  1.26±0.43mg/dL | |
| Huber 2003(73) | Inclusion Criteria   1. Chronic renal insufficiency (S. Creat. ≥1.3mg/dl) undergoing coronary angiography   Exclusion Criteria   1. Pregnancy 2. Contraindications to theophylline | 1.Coronary Angiography | 112 | Increase in S. Creat. ≥0.5 mg/dL (44 µmol/L) within 48 hours | Iomeprol Low-Osm | 100ml Nacl 30 minutes before coronary angiography, fluid intake ≥2 L/day was advised | Theophylline | 200 mg IV 30 minutes before coronary angiography | | Mean ±SD: 196.5±84.1 | | | Mean S. Creat. ±SD:  1.65±0.41mg/dL | |
|  |  |  |  |  |  |  | Placebo | IV hydration only | | Mean ±SD: 216.6±95.0 | | | Mean S. Creat. ±SD:  1.72±0.69mg/dL | |
| Inda-Filho 2014(74) | Inclusion Criteria  1. Age> 18 years 2. Elective coronary angiography or ventriculography   Exclusion Criteria   1. Received an iodinated contrast medium intravascularly within 30 days pre-procedure  2. Emergency coronary catheterization 3. Pulmonary oedema 4. Acute decompensated congestive heart failure 5. On nonsteroidal anti-inflammatory drugs or metformin at the time of the study 6. Declined to participate | 1.Angiography / Ventriculography | 561 | Increase of ≥ 0.3mg in or absolute value ≥1.1mg/dL of S. Creat. at 72 hours | Ioxitalamate Hi-Osm | All patients receive Nacl (0.9%, isotonic) given intravenously at 1 mL/kg/h. Patients received medications intravenously 60 minutes immediately before, during, and 6 hours immediately after contrast | Intravenous NAC | NAC in D5W at 150mg/kg/hr as bolus prior to contrast, then at 50 mg/kg/h | | Mean ±SD: 21±16.7 | | | Mean S. Creat. ±SD:  1.00±0.25mg/dL | |
|  |  |  |  |  |  |  | NaHCO3 | 150 mEq of 8.4% NaHCO3 in 1L 5% dextrose given as bolus at 3.5ml/kg/hr pre-contrast, then at 1.18ml/kg/hr | | Mean ±SD: 22±17.6 | | | Mean S. Creat. ±SD:  1.00±0.24mg/dL | |
|  |  |  |  |  |  |  | Control | IV hydration only | | Mean ±SD: 28±22.4 | | | Mean S. Creat. ±SD:  1.04±0.41mg/dL | |
|  |  |  |  |  |  |  | Both NAC + NaHCO3 | Excluded from analysis | | | | | | |
| Izani 2008(194) | Inclusion Criteria:  Patients electively admitted for coronary angiography > 18 yaers with creatinine clearance 40-90ml/min  Exclusion Criteria:  1. Severe renal failure  2. Severe peptic ulcer  3. Allergy to NAC or Asthma  4. Pregnancy or breast feeding  5. | 1.Coronary Angiography ± PCI | 108 | 25% increase in serum creatinine above the baseline, 48 hours after exposure to contrast media | Iohexol  Low-Osm | 0.45% normal saline 12 hours before contrast start and after at rate of 1ml/kg/h | NAC | 600mg BD for 4 days started 12 hours before contrast administration | | Contrast(ml) Mean(SD):  136.73(100.23) | | | S. Creat.  Mean(SD):  123.7(17.08) | |
|  |  |  |  |  |  |  | Control | Only I.V saline | | Contrast(ml) Mean(SD):  126.67(94.37) | | | S. Creat.  Mean(SD):  124.4(21.89) | |
| Jaffery 2012(76) | Inclusion Criteria   1. Diagnosis of ACS 2. Age >18years   Exclusion Criteria   1. End-stage renal disease (ESRD) requiring dialysis 2. Known hypersensitivity to NAC 3. History of life-threatening contrast reaction. | 1.Coronary Angiography / PCI | 456 | >25% increase above baseline S. Creat. within 72 hours of contrast | Iodixanol Iso-Osm | Additional intravenous normal saline (0.9% NaCl in water) was administered such that the total volume of fluid infused was equal to 1 cc/kg/hr for 24 hr | Intravenous NAC | 1,200 mg bolus followed by 200 mg/hr for 24 hr | | Mean ±SD: 169.5±94.5 | | | Mean S. Creat. ±SD:  1.09±0.4mg/dL | |
|  |  |  |  |  |  |  | Placebo | matched placebo | | Mean ±SD: 161.3±83.4 | | | Mean S. Creat. ±SD:  1.07±0.4mg/dL | |
| Jo  2013(77) | Inclusion Criteria   1. Age >19 years  2. Creat. clearance ≤ 60ml/min  3. Elective procedure  Exclusion Criteria   1. Acute Coronary Syndrome 2. Cardiogenic shock 3. Pulmonary oedema 4. Emergency Coronary angiography 5. Acute renal Failure 6. End Stage Renal Disease requiring Dialysis 7. Previous Exposure to Contrast Medium within seven days of study 8. Pregnancy 9. Lactation 10. Hypersensitivity to Contrast media 11. Mechanical ventilation 12 Parenteral Use of Diuretics 13. Multiple Myeloma 14. Use of Metformin or Non-steroidal anti-inflammatory drugs within 48 hours of procedure | 1.Coronary Angiography / PCI | 212 | Increase in S. Creat. ≥0.5 mg/dL (44 µmol/L) or >25% increase above baseline | Iodixanol Iso-Osm Ipromide Low-Osm Iobitridol Low-Osm | Isotonic Normal Saline at 1ml/kg/hr for 12 hours pre- and 12 hours post-procedure | Alpha Lipoic Acid | 600 mg orally 8-hourly commenced day of procedure, up to a total of 3600mg | | Mean ±SD: 165.2±83.6 | | | Mean S. Creat. ±SD:  120.8±69.8mmol/L | |
|  |  |  |  |  |  |  | Control | IV hydration only | | Mean ±SD: 174.6±103.6 | | | Mean S. Creat. ±SD:  108.2±37.5mmol/L | |
| Jo  2008(78) | Inclusion Criteria   1. Age >19 years  2. Baseline S. Creat. ≥1.1mg/dL or Creat. clearance ≤ 60ml/min   Exclusion Criteria   1. Acute Coronary Syndrome 2. Cardiogenic shock 3. Pulmonary oedema 4. Emergency Coronary angiography 5. Acute renal Failure 6. End Stage Renal Disease requiring Dialysis 7. Previous Exposure to Contrast Medium within seven days of study 8. Pregnancy 9. Lactation 10. Hypersensitivity to Contrast media 11. Mechanical ventilation 12 Parenteral use of diuretics Use of NAC, Ascorbic acid, metformin or nonsteroidal anti-inflammatory drugs within 48 hours of the procedure 13. Multiple Myeloma 14. Statin use within 30 days of procedure | 1.Coronary Angiography | 243 | Increase in S. Creat. ≥0.5 mg/dL (44 µmol/L) or >25% increase above baseline | Iodixanol Iso-Osm | Half-isotonic Normal Saline at 1ml/kg/hr for 12 hours pre- and 12 hours post-procedure | Simvastatin | 40mg twice daily for 2 days, twice pre- and twice post-angiography to total 160mg | | Mean ±SD: 173.3±99.3 | | | Mean S. Creat. ±SD:  1.285±0.409mg/dL | |
|  |  |  |  |  |  |  | Placebo | placebo twice daily for 2 days, twice pre- and twice post-angiography to total 160mg | | Mean ±SD: 190.9±133.5 | | | Mean S. Creat. ±SD:  1.234±0.358mg/dL | |
| Jo  2009(79) | Inclusion Criteria   1. Age >19 years  2. Baseline S. Creat. ≥1.1mg/dL or Creat. clearance ≤ 60ml/min   Exclusion Criteria   1. Acute Coronary Syndrome 2. Cardiogenic shock 3. Pulmonary oedema 4. Emergency Coronary angiography 5. Acute renal Failure 6. End Stage Renal Disease requiring Dialysis 7. Previous Exposure to Contrast Medium within seven days of study 8. Pregnancy 9. Lactation 10. Hypersensitivity to Contrast media 11. Mechanical ventilation 12 Parenteral use of diuretics Use of NAC, Ascorbic acid, metformin or nonsteroidal anti-inflammatory drugs within 48 hours of the procedure 13. Multiple Myeloma | 1.Coronary Angiography | 179 | Increase in S. Creat. ≥0.5 mg/dL (44 µmol/L) or >25% increase above baseline within 48 hours of contrast | Iodixanol Iso-Osm | Half-isotonic Normal Saline at 1ml/kg/hr for 12 hours pre- and 12 hours post-procedure | NAC | 1,200 mg orally twice daily, twice pre- and twice post-angiography, to a total of 4,800 mg | | Mean ±SD: 203.6±141.9 | | | Mean S. Creat. ±SD:  1.38±0.52mg/dL | |
|  |  |  |  |  |  |  | Ascorbic acid | 5mg pre-procedure in divided doses of 3g and 2g with 12h interval, two doses of 2g at 12h intervals post-procedure | | Mean ±SD: 216.4±136.1 | | | Mean S. Creat. ±SD:  1.27±0.35mg/dL | |
| Jurado-Roman  2015(80) | Inclusion Criteria   1. Patients with STEMI undergoing primary PCI   Exclusion Criteria   1. End-stage renal failure requiring dialysis 2. Cardiac arrest 3. Severe heart failure (Killip III to IV) | 1.PCI | 473 | Increase in S. Creat. ≥0.5 mg/dL (44 µmol/L) or >25% increase above baseline within 3 days of contrast | Iodixanol Iso-Osm | As per intervention protocol | IV hydration | Isotonic Normal Saline at 1ml/kg/hr from start of procedure continued for 24h | | Mean: 180 | | | Mean GFR ±SD: 90±21  mg/min/1.73m2 | |
|  |  |  |  |  |  |  | Control | no hydration | | Mean: 173 | | | Mean GFR ±SD: 88±54  mg/min/1.73m2 | |
| Kai, Z 2015(195) | Inclusion Criteria:  Patients > 18 years and < 80 years with Type 2 diabetes whose estimated glomerular filtration rate  (eGFR) was ≤ 60 mL/min·1.73m2  Exclusion Criteria:  1. Hyperpyrexia or allergic to iodine  2. Tumors  3. Severe heart failure  4. Severe kidney failure  5. Severe liver failure  6. Disorders of the immune system  7. Blood diseases. | 1.Coronary angiograph | 89 | Relative (≥ 25%) or an absolute (≥ 44.2 μmol/L) increase in serum creatinine from the baseline value within 3 days after intravascular administration of contrast medium | Iopamidol  Low-Osm | intravenous isotonic saline (0.9%) at a rate of approximately 1 mL/kg per hour for 6 h before, and 12 h after, contrast exposure | Standard Cordyceps | | 2-g corbrin capsules, 3 times/d, 3 days before and after procedure | | Mean+SD:  248.87±48.69 | | | S. Creat.  Mean+SD:  121±19 |
|  |  |  |  |  |  |  | Control  (basic treatment group) | | No Cordyceps | | Mean+SD:  246.85±49.74 | | | S. Creat.  Mean+SD:  122±19 |
|  |  |  |  |  |  |  | Intensive Cordyceps  This group was not analysed | | 3-g corbrin capsules, 3 times/d, 3 days before and after procedure | | Mean+SD:  250.87±50.72 | | | S. Creat.  Mean+SD:  122±19 |
| Kama 2014(81) | Inclusion Criteria   1.Patients who has CE-CT in Emergency Department 2. Age> 18 years 3. Mehran risk score for CI-AKI moderate and high-risk groups (>5 points)   Exclusion Criteria   1. Low risk for CI-AKI 2. Allergy to contrast 3. Hemodynamically unstable requiring excessive fluid resuscitation or surgery 4. Renal replacement therapy 5. Did not provide informed consent | 1.CE-CT | 123 | Increase in S. Creat. ≥0.5 mg/dL (44 µmol/L) or >25% increase above baseline within 48-72 hours of contrast | Low-Osm Iohexal All Patients get Less than 100 ml volume | 1,000 mL of 0.9% NaCl at a rate of 350 mL/hour | Nacl | 150 mg/kg 1L 0.9% NaCl at 350 mL/hour | |  | | | Median S. Creat. (IQR): 1.62 (1.4-2.07) mg/dL | |
|  |  |  |  |  |  |  | Control | IV hydration only | |  | | | Median S. Creat. (IQR): 1.47 (1.0-1.68) mg/dL | |
|  |  |  |  |  |  |  | NaHCO3 | 150 mEq in 1L 0.9% NaCl at 350 mL/hour | |  | | | Median S. Creat. (IQR): 1.49 (1.12-1.85) mg/dL | |
| Kay 2003(82) | Inclusion Criteria   1. Patients with stable moderate renal insufficiency (S. Creat >1.2mg/dL (106 µmol/L) or creatinine clearance < 60mL/min) 2. Elective coronary angiography and/or intervention  Exclusion Criteria   1. Dialysis 2. Acute renal failure  3. Contrast media or nephrotoxic agent within the last 30 days 4. Overt congestive heart failure  5. Severe valvular disease  6. LVEF< 35% 7. COPD or asthma 8. Allergy to NAC | 1.Coronary Angiography / PCI | 216 | >25% increase above baseline S. Creat. within 48 hours of contrast | Low-Osm | NaCl at 1mL/kg/hr for 12 hr pre- and 6hr post-contrast.  Liberal intake of oral fluid was encouraged | NAC | 600 mg orally twice daily day preceding and day of procedure, total 2 days | | Mean (IQR): 130 (75-320) | | | Median S. Creat. (IQR): 1.24 (0.77-2.99) mg/dL | |
|  |  |  |  |  |  |  | Placebo | placebo orally twice daily day preceding and day of procedure, total 2 days | | Mean (IQR): 120 (70-380) | | | Median S. Creat. (IQR): 1.26 (0.75-3.64) mg/dL | |
| Kefer 2003(83) | Exclusion Criteria   1. S. Creat. >3mg/dl 2. Acute Renal failure | 1.Coronary Angiography  2.PCI | 109 | Increase in S. Creat. ≥0.5 mg/dL (44 µmol/L) or >25% increase above baseline within 24 hours of contrast | Low-Osm Iopromide or Iohexol Mean CV/ml per procedure: 199±77 |  | Intravenous NAC | 1200mg in 200ml 0.9% NaCl in two 60 minute infusions, first 12hr pre-procedure, and second following administration of contrast. | |  | | |  | |
|  |  |  |  |  |  |  | Placebo | placebo in 200ml 0.9% NaCl in two 60 minute infusions, first 12hr pre-procedure, and second following administration of contrast. | |  | | |  | |
| Khosravi 2016(196) | Inclusion Criteria:  Patients aged between 55 and 75 who had diabetes (fasting blood sugar > 126 mg/dL, random blood sugar > 200 mg/dL, and glucose tolerance test > 200 mg/dL) and chronic renal failure (creatinine > 1.5 mg/dL or 15 < glomerular filtration rate [GFR] < 60 mls/min/1.73m2) and were candidates for elective angiography an  Exclusion Criteria:  1. Recent treatment with 80 mg of statin (high dose)  2. Need for emergency angiography  3. Contraindications to statin prescription  4. previous contrast-media administration during the preceding 10days  5. Chronic dialysis treatment, and informed refusal of consent | 1.Coronary Angiography | 229 | Increase in serum creatinine more than 0.5 mg/dL or more than 25% from the baseline (for this analysis we took 48 hours incidences) | Iso-Osm | Isotonic saline (0.9% sodium chloride or half saline, 1 3 mL/kg/h), intravenously, and N-acetylcysteine (NAC) 1200 mg, orally, twice a day, 1 day before to 2 days after intervention started from1hour before angiography until 4 hours after | Atorvastatin | 80mg/d from 48h before angiography | | < 200 ml for 96.3% of patients | | | S. Creat.  Mean+SD:  1.53±0.44 | |
|  |  |  |  |  |  |  | Placebo | Matching Placebo | | < 200 ml for 98.2% of patients | | | S. Creat.  Mean+SD:  1.47±0.42 | |
| Khoury 1995(84) | Inclusion Criteria  1. Patients undergoing radiocontrast study   Exclusion Criteria   1. NSAID use 2. Use of nephrotoxic drugs 3. Contrast media administration within 72 hours of procedure  4. LVEF < 30% | 1.CT 2.Angiography 3.IVP 4.Venogram | 87 | >25% increase above baseline S. Creat. to above 124 µmol/L | Radiologist preference allowed | Normal saline 0.5-1.5 L pre-procedure and 0.5 L after | Nifedipine | 10mg 1 hour pre-procedure | | Mean ±SD: 126±34.8 | | | Mean S. Creat. ±SD:  92.4±53.1mmol/L | |
|  |  |  |  |  |  |  | Control | no intervention | | Mean ±SD: 118.1±41.8 | | | Mean S. Creat. ±SD:  92.5±31.6mmol/L | |
| Kimmel 2008(85) | Inclusion Criteria   1. Mild to moderately impaired kidney function  2. Age> 18 years 3. S. Creat. ≥1.2 mg/dl or Creatinine clearance < 50 ml/min measured by a 12- or 24-h urine collection  Exclusion Criteria   1. Acute inflammatory disease 2. Medication with NSAID or metformin up to 3 days before entering study 3. Abnormal findings in physical examinations, e.g. signs of dehydration or inflammation. | 1.Coronary Angiography | 60 | Increase in S. Creat. ≥0.5 mg/dL (44 µmol/L) or >25% increase above baseline | Iomeperole Low-Osm | 1ml/kg/h infusion of 0.45% saline for 24 h (12 h before and 12 h after exposure to contrast media | NAC | 600 mg orally twice daily day preceding and day of procedure, total 2 days | | Mean ±SD: 187±88 | | | Mean S. Creat. ±SD:  1.51±0.23mg/dL | |
|  |  |  |  |  |  |  | Zinc | 60 mg once daily on the day before missing doses replaced by placebo | | Mean ±SD: 173±85 | | | Mean S. Creat. ±SD:  1.60±0.49mg/dL | |
|  |  |  |  |  |  |  | Placebo | placebo orally twice daily day preceding and day of procedure, total 2 days | | Mean ±SD: 219±105 | | | Mean S. Creat. ±SD:  1.65±0.65mg/dL | |
| Kinbara 2010(86, 87) | Inclusion Criteria   1. Patients with stable S. Creat. (baseline ± ≤0.1mg/dL at 12-24h pre-procedure) concentrations undergoing coronary angiography± PCI   Exclusion Criteria    1. Acute MI requiring primary or rescue PCI 2. Use of vasopressors before PCI 3. Cardiogenic shock 4. Current peritoneal dialysis or haemodialysis, 5. Planned post-contrast dialysis 6. Allergies to the medications being studied 7. Overt congestive heart failure 8. Severe valvular disease 9. LVEF<30% 10. Pregnancy 11. Multiple myeloma 12. Amyloidosis | 1.Coronary Angiography  2.PCI | 49 | Increase in S. Creat. ≥0.5 mg/dL (44 µmol/L) after 48h | Iopamidol  Low-Osm | Nacl 1 ml/kg/hr for 30 min pre- and 10h post-procedure | NAC | 704 mg orally twice daily day preceding and day of procedure, total 2 days | | Mean ±SD: 147±23 | | | Mean S. Creat. ±SD:  1.00±0.36mg/dL | |
|  |  |  |  |  |  |  | Control | IV hydration only | | Mean ±SD: 141±14 | | | Mean S. Creat. ±SD:  0.97±0.29mg/dL | |
|  |  |  |  |  |  |  | Aminophylline | 250mg as a short infusion (100ml saline, 0.9%) 30 min pre-procedure | | Mean ±SD: 142±15 | | | Mean S. Creat. ±SD:  0.94±0.21mg/dL | |
| Kitzler 2012 | Inclusion Criteria   1. Chronic kidney disease stage 1–4  2. Elective CT with non-ionic radiocontrast agents  3. Age >18years  4. S. Creat.> 1.25 mg/dL for males and 1.09 mg/dL for females  4. No renal replacement therapy  Exclusion Criteria  1. Acute kidney injury 2. Increase of >0.2mg/dL in S. Creat. from baseline to enrolment  3. Administration of vitamin E, NAC, or other antioxidant therapy within 4 weeks of study 4. Participation in an investigational clinical trial within 1 month prior to the start of the study 5. Known or suspected allergy to the investigational drugs 6. Current use of a theophylline, dopamine, furosemide, or mannitol. | 1.Elective CT | 30 | >25% increase above baseline S. Creat. at 48 hours | Low-Osm Iopromide | 0.45% saline infusion at 1 ml/kg/hr for 12 h before and after CT. | NAC | 1200gm NAC granules+ placebo emulsion oral route, 12 and 6 h before and 6 and 12 h post-procedure, as well as 0.45 % saline infusion (1:1 dilution of 0.9 % saline with 5 % glucose) | | 100ml | | | Mean S. Creat. ±SD:  1.37±0.51mg/dL | |
|  |  |  |  |  |  |  | Vitamin E | Placebo granules + Vitamin E emulsion (540 mg) intravenously 12 and 6 h before and 6 and 12 h post-procedure, as well as 0.45 % saline infusion | | 100ml | | | Mean S. Creat. ±SD:  1.37±0.2mg/dL | |
|  |  |  |  |  |  |  | Placebo | Placebo granules + placebo emulsion as well as 0.45 % saline infusion | | 100 | | | Mean S. Creat. ±SD:  1.33±0.12mg/dL | |
| Klima 2012(88) | Inclusion Criteria   1. Patients undergoing intra-arterial / intravenous radiological contrast study with renal dysfunction (S. Creat.>0.93 µmol/L for women and 0.117 µmol/L for men, or  eGFR <60mL/min/1.73 m2  Exclusion Criteria   1. Age <18 years 2. Dialysis 3. Allergy to radiographic contrast 4. Pregnancy 5. NYHA class III and IV heart failure  6. NAC ≤24h before contrast 7. Clinical condition requiring continuous fluid therapy, e.g. severe sepsis | 1.Contrast Study | 185 | Increase in S. Creat. ≥0.5 mg/dL (44 µmol/L) or >25% increase above baseline at 48h | Low-Osm Iso-Osm Iopromid Iomeprol Iopentol Iohexol Iobitridol Iodixanol | Additional oral fluid intake was encouraged in all groups | IV hydration | NaCl at 1 mL/kg/h beginning from 8 p.m. on the day pre- and for ≥12 h post-procedure. CV median (IQR), mL: 100 (80–163) | | Median (IQR): 100 (80-163) | | | Median S. Creat. (IQR): 141 (112-158) mmol/L | |
|  |  |  |  |  |  |  | NaHCO3 | Initial intravenous bolus 3 mL/kg/h of 166 mEq/L NaHCO3 for 1hr pre-contrast. Following this, patients received the same fluid at 1mL/kg/h during and for 6h post-procedure | | Median (IQR): 100 (80-143) | | | Median S. Creat. (IQR): 141 (115-164) mmol/L | |
|  |  |  |  |  |  |  | Intravenous and oral NaHCO3 | Excluded from analysis | | | | | | |
| Ko 2013(89) | Inclusion Criteria   1. eGFR <60 mL/min | 1.Coronary Angiography | 159 | Increase in S. Creat. ≥0.5 mg/dL (44 µmol/L) or >25% increase above baseline at 48h | Iodixanol Iso-Osm | 0.45% saline at 1mL/kg/hr (0.5 mL/kg/hr if LVEF <40%) administered ≥ 8hr pre- and post-procedure | Nicorandil | 12 mg dissolved in 100 mL of 0.9% saline | | Mean ±SD: 125.6±69.1 | | | Mean S. Creat. ±SD:  1.73±0.6mg/dL | |
|  |  |  |  |  |  |  | Control | 100ml of 0.9% saline | | Mean ±SD: 126.9±74.6 | | | Mean S. Creat. ±SD:  1.61±0.44mg/dL | |
| Koc 2012(91) | Inclusion Criteria   1. Age ≥18 years  2. Creatinine clearance ≤60mL/min and/or S. Creat. ≥1.1mg/dL   Exclusion Criteria   1. Contrast-agent hypersensitivity 2. Pregnancy  3. Lactation 4. Decompensated heart failure 5. Pulmonary oedema 6. Emergency catheterization 7. Acute renal failure 8. End-stage renal failure | 1.Coronary Angiography  2.PCI | 241 | Increase in S. Creat. ≥0.5 mg/dL (44 µmol/L) or >25% increase above baseline at 48h | Iohexol Low-Osm | As per intervention protocol | Intravenous NAC | IV bolus of 600 mg twice daily before and on the day of procedure (total=2.4 g) plus IV 0.9% saline 1 mL/kg/h before, on and after the day of procedure | | Median (IQR): 130 (100-155) | | | Mean CrCl. ±SD:  59±16mL/min | |
|  |  |  |  |  |  |  | Control | IV 0.9% saline 1 mL/kg/h 12h pre- and 12h post-procedure | | Median (IQR): 130 (119-150) | | | Mean CrCl. ±SD:  63±15mL/min | |
|  |  |  |  |  |  |  | IV hydration | IV 0.9% saline 1mL/kg/h before, on and after the day of coronary procedure | | Median (IQR): 120 (100-150) | | | Mean CrCl. ±SD:  58±16mL/min | |
| Koc 2013(90) | Inclusion Criteria   1. Diabetes Mellitus  2. Age>18 years   Exclusion Criteria   1. Hypersensitivity to contrast 2. Decompensated Heart failure 3. Pulmonary Oedema 4. Pregnancy 5. Lactation 6. Severe renal impairment 7. Emergency Procedure 8. Contrast Medium administration within 7 days | 1.Coronary Angiography  2.PCI | 216 | Increase in S. Creat. ≥0.5 mg/dL (44 µmol/L) or >25% increase above baseline at 48h | Iohexol Low-Osm Preferred | As per intervention protocol | NaHCO3 | 154 mL of 8.4% NaHCO3 add to 846 mL of 5% glucose given at 1ml/kg/hr 6hr pre- and 6h post-procedure | | Median (IQR): 90 (85-100) | | | Median S. Creat. (IQR): 1.0 (0.8-1.3) mg/dL | |
|  |  |  |  |  |  |  | IV hydration | I.V Hydration Group: 1 ml/Kg/hour for 12h pre- and 12h post-procedure | | Median (IQR): 90 (90-100) | | | Median S. Creat. (IQR): 1.0 (0.87-1.3) mg/dL | |
| Koch 2000(92) | Inclusion Criteria   1. Age >18  2. Stable impaired renal function (S. Creat. ≥1.5 mg/dl)   Exclusion Criteria   1. MI 2. Cerebral stroke 3. Chronic cardiac insufficiency 4. Unstable angina pectoris 5. Significant arrhythmias 6. Intake of digitalis 7. Clinically relevant respiratory, gastrointestinal, hematologic, or neurologic illness 8. Haemodialysis or progressive renal failure 9. Severe liver damage 10. Multiple myeloma 11. Autoimmune illnesses or severe allergies 12 Severe uncontrollable HTN (systolic >220 mmHg) 13. Arterial hypotension (systolic <80 mmHg) 14. Cardiogenic Shock 15. Infectious diseases or fever. | 1. Coronary Angiography 2. Peripheral Angiography | 83 | Maximum creatinine increase over 48 h was separated according to the cut off values ≥0.5mg/dl , ≥1.0mg/dl The Cut-off point ≥0.5mg/dl used for the purpose of this analysis | Iso-Osm (320& 340 mg/ml) | 2000 ml (1000 ml NaCl solution/1000 ml 5% glucose solution) for 24 h pre- and post-procedure | PGE1 | 20 ng/kg/min 1 h prior to radiocontrast administration and continued for total of 6 h. | | Mean ±SD: 158.5±73.86 (20-445) | | | Mean S. Creat. ±SD:  2.07±0.48mg/dL | |
|  |  |  |  |  |  |  | PGE1 | The other 2 arms with 10,40 ng concentration is not included in this analysis | | | | | | |
|  |  |  |  |  |  |  | Placebo | placebo prior to radiocontrast administration and continued for total of 6 h. | | Mean ±SD: 158.5±73.86 (20-445) | | | Mean S. Creat. ±SD:  2.41±0.72mg/dL | |
| Kong 2012(93) | Inclusion Criteria   1. Suspected or definitive coronary artery disease 2. Age 18- 80 years old 3. Normal renal function   Exclusion Criteria   1. Acute MI 2. LVEF<45% 3. Blood electrolyte disturbance 4. Liver dysfunction | 1.Coronary Angiography  2.PCI | 85 | Increase in S. Creat. ≥0.5 mg/dL (44 µmol/L) or >25% increase above baseline at 48h-72h | Iopromide Low-Osm | Patients Allowed to drink tap water or other fluid freely in all groups | IV hydration | NaCl at 1ml/kg/hr 12h pre- and 24h post-procedure | | Mean ±SD: 139.6±54.3 with PCI; 79.4±22.4 without PCI | | | Mean S. Creat. ±SD:  102±25.9mmol/L | |
|  |  |  |  |  |  |  | Oral Hydration | 500ml of tap water pre- procedure and 2000ml within 24 hours post procedure | | Mean ±SD: 142.8±56.1 with PCI; 74.7±20.2 without PCI | | | Mean S. Creat. ±SD:  108±32.78mmol/L | |
|  |  |  |  |  |  |  | Oral Hydration | 2000ml oral tap water within 24 hours post-procedure  NB: this group was excluded from our analysis (post intervention hydration only) | | | | | | |
| Kooiman 2014a(95) | Inclusion Criteria   1. Chronic renal impairment (eGFR <60ml/min) 2. High clinical suspicion of acute PE   Exclusion Criteria   1. Pregnancy 2. Previous contrast administration within the past 7 days 3. Documented allergy for iodinated contrast media 4. Hemodynamic instability (SBP < 100 mm Hg) | 1.CTPA | 145 | Increase in S. Creat. ≥0.5 mg/dL (44 µmol/L) or >25% increase above baseline at 48h-96h | Iopromide Low-Osm Iobitridol Low-Osm Iodixanol Iso-Osm Contrast type according to each hospital guidelines | As per intervention protocol | NaHCO3 | 250 mL I.V 1.4% NaHCO3 1 hour before CTPA without hydration after CTPA | | Mean ±SD: 73.8±8.1 | | | Mean eGFR ±SD:  48.2±15.4 | |
|  |  |  |  |  |  |  | Control | No intervention | | Mean ±SD: 74.5±10.3 | | | Mean eGFR ±SD:  50.2±15.5 | |
| Kooiman 2014b(94) | Inclusion Criteria   1. Elective CE-CT 2. Age> 18y 3. CKD (eGFR < 60mL/min/1.73m2  Exclusion Criteria   1. Pregnancy 2. Previous contrast administration within the last 7 days 3. Documented allergy for iodinated contrast media 4. Haemodynamic instability (SBP<100mmHg) 5. Previous participation in the trial | 1.CE-CT | 560 | Increase in S. Creat. ≥0.5 mg/dL (44 µmol/L) or >25% increase above baseline | low-Osm Iomeprol Iobitridol Iodixanol | As per intervention protocol | NaHCO3 | 250 mL I.V 1.4% NaHCO3 1 hour pre-CE-CT | | Mean ±SD: 105.7±21 | | |  | |
|  |  |  |  |  |  |  | I.V Hydration Group: | 2L 0.9% NaCl, 1L pre- and 1L post-CE-CT | | Mean ±SD: 104.7±21.6 | | |  | |
| Kotlyar 2005(96) | Inclusion Criteria   1. S. Creat. ≥0.13 mmol/l, 2. Elective coronary, carotid or peripheral angiography and/or PTCA and stenting  Exclusion Criteria   1. Allergy to the study medication 2. Unstable renal function (Creat. increase ≥0.04 mmol/l day) 3. Patients on dialysis 4. Uncontrolled asthma 5. Pregnancy 6. Breastfeeding | 1.Coronary  angiography  2.Carotid  angiography  3.Peripheral angiography  4. PTCA | 40 | Increase in the serum creatinine concentration of at least 0.044 mmol/l 48-96 hours post procedure | Iopromide Low-Osm | 0.9% saline at 200ml/h 2h pre-and continued for a further 5h post-procedure | Intravenous NAC | 600mg in 100ml of 5% dextrose administered over 20 min, 1–2h before angiography and again 2–4h after angiography | | Mean ±SD: 89±32 | | | Median S. Creat. (Range):  27.5±5.8mmol/L | |
|  |  |  |  |  |  |  | Placebo | matched placebo | | Mean ±SD: 86±41 | | | Median S. Creat. (Range):  27.5±5.8mmol/L | |
| Kumar 2014(97) | Inclusion Criteria:   1. Patients without risk factors for AKI 2. Patients receiving < maximum permissible dose of the dye   Exclusion Criteria  1. Patients receiving >maximum permissible dose of dye 2. Use of nephrotoxic Drugs 3. Gout or serum uric acid levels >10mg/dl 4. Hypersensitivity or intolerance to allopurinol 5. Congestive heart failure or LVEF < 40% 6. Inability to give consent | 1.PCI | 289 | Increase in S. Creat. ≥0.5 mg/dL (44 µmol/L) or >25% increase above baseline | Patents Randomized in 2 groups:  1. Iow-Osm Omnipaqu 2.Iso-Osm Visipaque | 0.9% saline at 1ml/kg/min (to max of 100 ml/hr) for 12h pre- and 12h post-procedure. Patients randomized first to Omnipaque and Visipaque arms which is analysed separately in this study | NAC | 600 mg orally twice daily 12h pre- and post- contrast | |  | | | Mean S. Creat. (Range):  Omnipaque:  1.0 (0.9-1.3) Visipaque  1.1 (0.9-1.2) | |
|  |  |  |  |  |  |  | Allopurinol | 300 mg orally 12h pre- and post- contrast | |  |  |  |  |  |
|  |  |  |  |  |  |  | I.V Hydration | IV hydration only | |  |  |  |  |  |
| Kurnik  1990(99) | Inclusion Criteria   1. Patients with stable S. Creat ≥ 1.8mg/L 2. Elective cardiac catheterization  Exclusion Criteria   1. Hypersensitivity to peptide drugs, local anaesthetics, heparin or radiocontrast 2. Pregnancy 3. NYHA Class IV CHF 4. Radiocontrast exposure within the last 7 days 5. MI within the last 2 weeks 6. Unstable renal function 7. Medical instability | 1.PCI | 28 | Increase in S. Creat. ≥0.5 mg/dL (44 µmol/L) above baseline at 24h post-contrast | MD 76 Hi-Osm | 75mmol/L NaCl in water at 100ml/hr starting 12 hr pre-procedure | ANP | Bolus: 50µg, followed by 1µg/min mixed in 75mmol Nacl for 2 hours | |  | | | Mean S. Creat. ±SEM:  2.4±0.7mg/dL | |
|  |  |  |  |  |  |  | Mannitol | 15% mannitol for 2 hours before and during procedure | |  |  |  | Mean S. Creat. ±SEM:  2.5±0.8mg/dL | |
| Kurnik  1998(98) | Inclusion Criteria   1. Patients aged 18-85years  2. Patients with stable S. Creat ≥1.8mg/L OR ≥ 1.5mg/L and < 1.8mg/L with Creat. Clearance ≤65ml/min  3. Elective cardiac catheterization  Exclusion Criteria   1. Hypersensitivity to peptide drugs, local anaesthetics, heparin or radiocontrast 2. Dialysis  3. SBP ≤100 mmHg before study 3. Dialysis  4. Major surgery or radiocontrast exposure within the last 7 days 5. Expected Dose of radiocontrast <than 75 mL 6. NYHA Class IV CHF 7. Scheduled surgical procedure within 48hr post-procedure 8. Pregnancy 9. Lack of consent  10. Co-morbidity | 1.Contrast-enhanced radiographic procedures | 152 | Increase in S. Creat. ≥0.5 mg/dL (44 µmol/L) or >25% increase above baseline | Choice of radiocontrast agent determined by the angiographer | Intravenous 0.45% saline for 12hr pre= and continuing for 12hrpost-contrast | ANP | 0.05 µg/kg/min 30 minutes before and continuing for 30 minutes after radiocontrast administration. Other 2 arms (0.1 and 0.01 µ µg/kg/min were excluded from this analysis) | | Mean ±SD: 141±58 | | | Mean S. Creat. ±SD:  2.1±0.9mg/dL | |
|  |  |  |  |  |  |  | Placebo | matched placebo | | Mean ±SD: 132±54 | | | Mean S. Creat. ±SD:  2.1±0.56mg/dL | |
| Lawlor 2007(101) | Inclusion Criteria   1. Pre-existing renal impairment 2. Angioplasty for peripheral vascular disease   Exclusion Criteria   1. Acute Renal Failure 2. Haemodynamic instability 3. Unable to tolerate hydration protocol for medical reasons 4. Known sensitivity to NAC 5. Unable to provide informed consent | 1.Peripheral Angioplasty | 54 | Increase in S. Creat. ≥0.5 mg/dL (44 µmol/L) or >25% increase above baseline at 48h |  | 0.9% normal saline 1 ml/kg/hr for 12 hours pre- and post-procedure | NAC | 600 mg in 30ml of ginger ale orally twice daily, day prior to and day of angioplasty | |  | | | Mean S. Creat. ±SD:  167±46mmol/L | |
|  |  |  |  |  |  |  | Placebo | 3ml of 0.9% normal saline in 30ml of ginger ale orally twice daily, day prior to and day of angioplasty | |  | | | Mean S. Creat. ±SD:  172±48mmol/L | |
|  |  |  |  |  |  |  | Control | Out-patient oral hydration followed by IV hydration, *excluded from analysis* | | | | | | |
| Lee 2011(102) | Inclusion Criteria   1. Patients undergoing arteriography or intervention 2. S. Creat ≥1.1mg/dl, eGFR <60ml/min/1.73m2 3. Age >18yr 4. Diabetes mellitus  Exclusion Criteria   1. Inability to obtain informed consent 2. S. Creat >8mg/dl, eGFR <15ml/min/1.73 m2 at rest 3. End-stage renal disease on haemodialysis 4. Multiple myeloma 5. Pulmonary oedema 6. Uncontrolled hypertension (SBP >160 mmHg or DBP >100 mmHg) 7. Acute STEMI while undergoing primary PCI 8. Emergency coronary angioplasty or angiography 9. Use of contrast media within the previous 2 days 10. Pregnancy 11. Allergy to contrast medium or medications such as theophylline, Dopamine, Mannitol, Fenoldopam, and NAC | Coronary and endovascular angiography ± Intervention | 402 | Increase in S. Creat. ≥0.5 mg/dL (44 µmol/L) or >25% increase above baseline at 48h | Iodixanol Iso-Osm | As per intervention protocol. Also, NAC 1,200 mg twice daily for 2 days starting day before procedure. | NaHCO3 | 154 mEq/L in dextrose and water starting at 3 ml/kg/hr, 1hr pre-procedure, decreasing to 1 ml/kg/hr during procedure and for 6hr post-procedure (decreased to 0.5 l/kg/hour in patients with LVEF <45%) | | Mean (Range): 113 (80-200) | | | Mean S. Creat. (Range):  1.5 (1.3-1.9) mg/dL | |
|  |  |  |  |  |  |  | IV hydration | 0.9% sodium chloride 1 ml/kg/hour for 12 hours pre- and post- procedure  (decreased to 0.5 l/kg/hour in patients with LVEF <45%) | | Mean (Range): 120 (79-223) | | | Mean S. Creat. (Range):  1.5 (1.3-1.7) mg/dL | |
| Lehnert 1998(103) | Inclusion Criteria   1. Stable S. Creat. ≥1.4 mg/dl (124 µmol/l) 2. Angiography (contrast medium dose ≥1.2 ml/kg)  Exclusion Criteria  1. End-stage renal disease 2. Allergy to contrast medium 3. Prior exposure to contrast medium within 14 days before 4. Age <30yr 5. Pregnancy | 1.Angiography | 44 | Increase in S. Creat. ≥0.5 mg/dL (44 µmol/L) above baseline at 48h post-contrast | Iopentol Low-Osm | 0.9% saline I.V at 83 ml/h starting 12h pre- and for 12h post-procedure | Haemodialysis | Haemodialysis start with injection of the last part of contrast media and continue for 3 hours | | Mean CV (ml/kg) ±SEM: 3.5±0.6 | | | Mean S. Creat. ±SD:  2.58±0.25mg/dL | |
|  |  |  |  |  |  |  | Control | IV hydration only | | Mean CV (ml/kg) ±SEM: 3.0±0.4 | | | Mean S. Creat. ±SD:  2.26±0.2mg/dL | |
| Leoncini 2014(104) | Inclusion Criteria   1. Non-STEMI 2. Early invasive strategy  Exclusion Criteria   1. Current statin treatment 2. High-risk features warranting emergency coronary angiography (within 2 h) 3. Acute renal failure or end-stage renal failure requiring dialysis 4. Serum creatinine ≥ 3mg/dl 5. Severe comorbidities which precluded early invasive strategy 6. Contraindications to statin treatment 7. Contrast medium administration within the previous 10 days 8. Pregnancy 9. Refusal of consent | 1.PCI | 559 | Increase in S. Creat. ≥0.5 mg/dL (44 µmol/L) or >25% increase above baseline | Iso-Osm Iodixanol [Visipaque] | 0.9% saline I.V at 1ml/kg/h starting 12h pre- and for 12h post-procedure. NAC 1,200 mg twice daily for 2 days starting day before procedure.  (Fluid decreased to 0.5 l/kg/hour in patients with LVEF <40%) | Statin | 40 mg Rosuvastatin on time of randomization followed by 20 mg/day | | Mean ±SD: 183±80 | | | Mean S. Creat. ±SD:  0.95±0.27mg/dL | |
|  |  |  |  |  |  |  | Control | No Statins | | Mean ±SD: 127±72 | | | Mean S. Creat. ±SD:  0.96±0.28mg/dL | |
| Li, W 2012(106) | Inclusion Criteria   1. Patients with acute STEMI 2. Emergency PCI`  Exclusion Criteria   1. Statin treatment within preceding 3 months  2. Renal or hepatic dysfunction 3. Dialysis  4. Prior fibrinolysis 5. Unconsciousness on arrival 6. Cardiogenic shock with intra-aortic balloon pumping 7. Uncontrolled hypertension 8. Stroke 9. Major operation within the last 3 months 10. Refuse PCI | 1.PCI | 176 | Increase in S. Creat. ≥0.5 mg/dL (44 µmol/L) above baseline 3days post-contrast | Low-Osm Ultravist | 0.9% saline I.V at 1ml/kg/h starting 12h pre- and for 12h post-procedure | Statin | 80mg Atorvastatin pre-procedure, and continued long-term, 40mg/day | | Mean ±SD: 100±25.9 | | | Mean S. Creat. ±SD:  82.3±11.2mmol/L | |
|  |  |  |  |  |  |  | Placebo | matched placebo | | Mean ±SD: 103.6±26.2 | | | Mean S. Creat. ±SD:  82.6±11.3mmol/L | |
| Li 2009(105) | Inclusion Criteria   1. Patients undergoing planned coronary angiography or intervention  Exclusion Criteria   1. NYHA Class IV CHF 2. S. Creat.  >3.0 mg/dl | 1.Coronary Angiography  2.PCI | 228 | Increase in S. Creat. ≥0.5 mg/dL (44 µmol/L) above baseline 3days post-contrast | Iohexol Low-Osm | 0.9% saline I.V at 1ml/kg/h for 12h post-procedure | probucol | 500 mg orally twice daily for 3 days before and after the procedure | | Mean ±SD: 116±65 | | | Mean S. Creat. ±SD:  0.99±0.4mg/dL | |
|  |  |  |  |  |  |  | Control | No Probucol | | Mean ±SD: 121±56 | | | Mean S. Creat. ±SD:  1.08±0.71mg/dL | |
| Li 2011(108) | Inclusion Criteria   1. Mild to moderate renal insufficiency (eGFR 60-89ml/min)  Exclusion Criteria  1. Diagnostic only procedure 2. S. Creat ≥176 µmol/L  3. NYHA class IV CHF  4. Renal artery stenosis 5. Diagnosed during angiography 6. Allergy to contrast medium 7 ACEI intolerance 8. Autoimmune disease 9. End Stage Renal Failure requiring dialysis 10. Administration of contrast medium within the last 6 days or within the next flowing 2 days 11. Pregnancy | 1.PCI | 123 | Increase in S. Creat. ≥0.5 mg/dL (44 µmol/L) or >25% increase above baseline at 72 h post-contrast | Iohexol Iow-Osm | 0.9% sodium chloride at 1ml/kg/hr for 6 hours pre- and 6 hours post-procedure | ACE-inhibitor | 10mg Benazepril daily for at least 3 days pre-procedure | | Mean ±SD: 167.37±51.23 | | | Mean S. Creat. ±SD:  83.2±15.44mmol/L | |
|  |  |  |  |  |  |  | Placebo | matched placebo | | Mean ±SD: 159.90±51.58 | | | Mean S. Creat. ±SD:  83.4±16.71mmol/L | |
| Li 2014(107) | Inclusion Criteria   1. Patients undergoing coronary intervention procedure  Exclusion Criteria   1. Patients who used nephrotoxic drugs during pre-operative period 2. Severe hepatic and renal dysfunction  (eGFR) <30ml/min/1.73 m2) 3. Active cancer  4. NYHA class IV CHF 5. LVEF<35 % 6. Thyroid or adrenal dysfunction 7. Acute or chronic infectious diseases 8. Hyperpyrexia | 1.PCI | 175 | Increase in S. Creat. ≥0.5 mg/dL (44 µmol/L) or >25% increase above baseline at 48 h post-angiography | Iohexol Iow-Osm | 0.9 % sodium chloride solution for routine hydration (volume/rate not specified) | PGE1 | Intravenous infusion at 20ng/kg/min for 6 h before and after the administration of contrast | | Mean ±SD: 172±32 | | | Mean S. Creat. ±SD:  0.98±0.14mg/dL | |
|  |  |  |  |  |  |  | Control | IV hydration only | | Mean ±SD: 168±41 | | | Mean S. Creat. ±SD:  0.96±0.17mg/dL | |
| Liu, W 2015(198) | Inclusion Criteria:  Patients 18-75 yaesr old with mild to modrate CKD (eGFR 30-89/min)  Exclusion Criteria:  1. Acute renal failure  2. End stage renal disease that needs dialysis  3. unstable renal function  4. Uncontrolled DM, HTN or Hyperthyroidism  5. Class IV cardiac failure or; left ejection fraction < 35%  6. Acute myocardial infarction require primary or rescue coronary intervention  7. Cardiogenic shock  8. Administration of contrast media from 7 days before to 72 hours after study intervention  8. Agents for CIN prevention (such as NAC) or intake of nephrotoxic drugs from 24 before to 24 hours after  9. patients treated with ascorbic acid within last 30 days  10 Allergy to Trimetazidine | 1.Coronary angiography | 151 | increase of >25% or an absolute increase of ≥0.5 mg/dl in SCr from the baseline value 48 -72 hours of contrast administration | Iodixanol  Iso-Osm | 1-1.5 ml/ kg per hour start 3-12 hours before angiography and up to 12 hours thereafter | Trimetazidine | 20mg three times daily orally 48 hours before and 24 hours after coronary angiography | | Mean+SD:  124.94±31.65 | | | Mean S. Creat. ±SD:  107.74±24.03 | |
|  |  |  |  |  |  |  | Control | No Trimetazidine | | Mean+SD:  119.69±34.28 | | | Mean S. Creat. ±SD:  103.38±19.43 | |
| Liu 2014(107) | Inclusion Criteria   1. Patients with unstable angina  Exclusion Criteria   1. Early invasive therapy (within 12-24 hours) 2. Patients with refractory angina or hemodynamic or electrical instability 3. Patients at increased risk for clinical events (CHF, serious ventricular ar- rhythmias) 4. High-risk unstable angina (resting angina within 48 hours or infarction angina, ST-segment depression more than 1 mm and 20 minutes, or increased cardiac bio- markersdtroponin T or I) 5. Stable angina 6.STEMI 7. NSTEMI 8. Pre-existing renal dysfunction. | 1.Coronary Angiography  2.PCI | 1100 | Increase in S. Creat. ≥0.5 mg/dL (44 µmol/L) or >25% increase above baseline at 48 h post-angiography | Ultravist  Iopromide | As per intervention protocol | Human recombinant (Brain Natriuretic Peptide) (rhBNP) | 0.005 µg/kg/min for 24 hours before procedure | | Median (Range): 128 (60-185) | | | Mean S. Creat. ±SD:  79.2±14.2mmol/L | |
|  |  |  |  |  |  |  | IV hydration | 0.9%, Normal saline at 1 mL/kg/h for 24 hours before PCI CV/mL: 119 (63,172) | | Median (Range): 119 (63-172) | | | Mean S. Creat. ±SD:  81.5±16.7mmol/L | |
| Liu 2016(197) | Inclusion Criteria  patients with CKD (eGFR between  15 and 60 mL/min/1.73m2) aged 18 to 80 years and undergoing coronary angiography or elective PCI  Exclusion Criteria   1. Emergency PCI 2. Patients with refractory angina or hemodynamic or electrical instability 3. Patients at increased risk for clinical events (CHF, serious ventricular  arrhythmias) 4. High-risk unstable angina (resting angina within 48 hours or infarction angina, ST-segment depression more than 1 mm and 20 minutes, or increased cardiac biomarkers  5. Heart dysfunction 6. Hypersensitivity to Contrast media or BNP  7. End-stage renal failure 8. systolic blood pressure ≤100mmHg before study drug infusion  9. CM administered within the past 7 days; BNP infusion within 1month; dopamine, NAC, Nahco3 and fenoldopam during the study. | 1.Coronary Angiography  2.PCI | 232 | Relative (≥25%) or absolute (≥0.5mg/dL, 44 µmol/L) increase in SCr from baseline within 48 h after CM exposure | Iodixanol  Iso-Osm | 0.9% NaCl at 1.0mL/kg/h for 12h before and 12h aſter CM administration | Human recombinant (Brain Natriuretic Peptide) (rhBNP) | 0.005 µg/kg/min for 24 hours before procedure | | Mean+SD:  102 ± 17.2 | | | Mean S. Creat. ±SD:  117.2 ± 13.1 | |
|  |  |  |  |  |  |  | Control | Hydration only | | Mean+SD:  96 ± 14.5 | | | Mean S. Creat. ±SD:  120.5 ± 14.7 | |
| Ludwig 2011(109) | Inclusion Criteria   1. Patients scheduled for cardiac catheterization, arteriography or computed tomography with contrast agents  2. S. Creat. ≥ 150 µmol/l (1.7mg/dl)  Exclusion Criteria   1. Patients undergoing dialysis 2. Acute Renal Failure 3. Received iodinated contrast media within 7 days prior to the study 4. Known allergy to MESNA 5. Pregnancy 6. Patients receiving dopamine, mannitol, or NAC | 1.Coronary Angiography  2.PCI  3.CE-CT | 107 | >25% increase above baseline S. Creat. at 48 hours | Iopromide Low-Osm | 1000 ml of 0.9% saline pre- and 500ml post-procedure in both groups. No further oral fluid intake was allowed. | MESNA | Infusion of 1600 mg of sodium 2-mercaptoethanesulfonate (MESNA) CV/mL: 140 (120-200) | | Median (Range): 140 (120-200) | | |  | |
|  |  |  |  |  |  |  | Control | IV hydration only | | Median (Range): 150 (120-180) | | |  | |
| Luo  2014(111) | Inclusion Criteria   1. STEMI  2. Primary PCI  Exclusion Criteria  1. Chronic peritoneal or haemodialysis treatment 2. Exposure to radiographic contrast within the previous two days 3. Allergies to radiographic contrast medium 4. Coronary anatomy not suitable for PCI or primary CABG | 1.PCI | 276 | Increase in S. Creat. ≥0.5 mg/dL (44 µmol/L) or >25% increase above baseline at 72h post-angiography | Iopamidol Low-Osm | As per intervention protocol | IV hydration | 0.9% normal saline at 1mL/kg/hr for 12 hours post-procedure | | Mean ±SD: 228.6±84.5 | | | Mean S. Creat. ±SD:  76±14mmol/L | |
|  |  |  |  |  |  |  | Control | no hydration | | Mean ±SD: 241.2±101.4 | | | Mean S. Creat. ±SD:  78±13mmol/L | |
| Luo 2013(110) | Inclusion Criteria   1. Age ≥18 years  2. Elective PCI 3. Informed consent  Exclusion Criteria   1. Emergency PCI 2. Baseline troponin ≥ 0.04ng/mL 3. Nicorandil or glibenclamide use  4. Inability to cooperate with trial protocol  5. Lack of informed consent 6. Second procedure of staged elective PCI | 1.PCI | 208 | Increase in S. Creat. ≥0.5 mg/dL (44 µmol/L) or >25% increase above baseline | Iopromide Low-Osm | I.V saline infusion at 1mL/kg/hr for 12 hr pre- and 12h post-contrast. Patients encouraged to drink oral fluids post procedure | RIPC | Blood pressure cuff placed around their non-dominant upper arm. The cuff inflated to 200-mm Hg pressure for 5 minutes, followed by 5 minutes of deflation and repeated 2 more times to 3 cycles in total <2h pre-procedure | |  | | | Mean eGFR ±SD:  101±20mL/min | |
|  |  |  |  |  |  |  | Control | no intervention | |  | | | Mean eGFR ±SD:  100±20mL/min | |
| MacNeill 2003(112) | Inclusion Criteria   1. Cardiac catheterization  2. Mild-moderate renal dysfunction (S. Creat ≥ 1.5 mg/dl)  Exclusion Criteria   1. Acute renal failure 2. Dialysis-dependent chronic renal failure 3. Exposure to contrast within the preceding 5 days 4. Emergent procedures 5. Pregnancy 6. Age < 21 years 7. Known sensitivity to acetylcysteine | 1.Coronary Angiography2.PCI | 51 | >25% increase above baseline S. Creat. at 72 hours | Iopromid Low-Osm Ioxilan Low-Osm | Pre-procedure: 0.45% saline at 1ml/kg/hr for 12h (in-patients) and 2ml/kg/hr for 4h (day-case patients). Post-procedure: 0.45% saline at 75 ml/hr for 12h | NAC | 600mg twice daily commenCI-AKIg day of procedure, for total of 5 doses | | Mean ±SD: 103±52 | | | Mean S. Creat. ±SD:  1.89±0.38mg/dL | |
|  |  |  |  |  |  |  | Placebo | matched placebo | | Mean ±SD: 116±63.3 | | | Mean S. Creat. ±SD:  1.88±0.41mg/dL | |
| Maioli 2008(5) | Inclusion Criteria   1. Planned coronary angiographic procedures  2. Renal Dysfunction (estimated Creat. Clearance <60ml/min)  Exclusion Criteria   1. Creat. Clearance ≥ 60ml/min 2. Refusal to participate 3. Administration of contrast medium within the previous 10 days 4. End stage renal disease | 1.Coronary Angiography | 556 | Increase in S. Creat. ≥0.5 mg/dL (44 µmol/L) above baseline 5days post-contrast | Iodixanol Iso-Osm | as per intervention protocol | NaCl | 0.9% sodium chloride at 1mL/kg/hr for 12 h pre- and post-procedure | | Mean (IQR): 170 (120-230) | | | Mean S. Creat. ±SD:  1.20±0.3mg/dL | |
|  |  |  |  |  |  |  | NaHCO3 | 154 mEq/l in dextrose and water at 3 ml/kg/h for 1 h pre-procedure, 1ml/kg/h for 6h post-procedure | | Mean (IQR): 160 (120-220) | | | Mean S. Creat. ±SD:  1.21±0.3mg/dL | |
| Maioli 2011(113) | Inclusion Criteria   1. STEMI 2. Primary PCI  Exclusion Criteria   1. Contrast medium administration within the previous 10 days 2. End-stage renal failure requiring dialysis 3. Refusal to give informed consent | 1.PCI | 543 | Increase in S. Creat. ≥0.5 mg/dL (44 µmol/L) or >25% increase above baseline within 3 days of contrast | Iodixanol Iso-Osm | as per intervention protocol | NaHCO3 | 154 mEq/L in dextrose and water at 3ml/kg/h, starting in the emergency room for 1h, followed by infusion of 1 mL/kg/h for 12 hours after PCI | | Mean ±SD: 208±92 | | | Mean S. Creat. ±SD:  1.09±0.3mg/dL | |
|  |  |  |  |  |  |  | NaCl | 0.9% sodium chloride at 1mL/kg/h for 12h immediately post-PCI | | Mean ±SD: 216±101 | | | Mean S. Creat. ±SD:  1.10±0.4mg/dL | |
|  |  |  |  |  |  |  | Control | no hydration | | Mean ±SD: 224±94 | | | Mean S. Creat. ±SD:  1.08±0.3mg/dL | |
| Malhis 2010(114) | Inclusion Criteria   1. Radiographic imaging with contrast   Exclusion Criteria   1. Acute renal failure 2. Maintenance dialysis 3. History of acute MI 4. LVEF ≤ 25% 5. Allergy to contrast media 6. Pregnancy 7. Contraindications for theophylline use  8. Use of acetylcysteine | 1.Coronary Angiography2.PCI  3.CE-CT | 294 | Increase in serum creatinine of at least 0.5 mg/dL in patients with a baseline serum creatinine less than 2 mg/dL or an increase of 25% in base- line Serum Creatinine with a baseline serum creatinine more than or equal to 2 mg/dL at 48 h after administration of contrast media | Iodixanol  Iso-Osm | 1-2 L of intravenous bicarbonate solution (150 meq/L) for 12h after the procedure | Theophylline | 200 mg twice daily starting 24h pre-procedure and continuing for 48 h post-procedure; OR 200 mg theophylline as a short intravenous infusion 30 minutes pre-procedure, and continuing with 200 mg twice daily oral theophylline for 48 h post-procedure | | Mean ±SD: 137±76 | | | Mean S. Creat. ±SD:  1.38±0.79mg/dL | |
|  |  |  |  |  |  |  | Control | IV hydration only | | Mean ±SD: 144±78 | | | Mean S. Creat. ±SD:  1.21±0.48mg/dL | |
| Marenzi 2003(117) | Inclusion Criteria   1. Chronic renal failure (S. Creat. >2mg/dL (176.8 µmol/L) OR creat. clearance < 50ml/min)  2. Coronary angiography or elective PCI  Exclusion Criteria   1. Acute coronary syndrome 2. Cardiogenic shock 3. Long-term peritoneal dialysis or haemodialysis treatment 4. Overt CHF 5. Recent major bleeding 6. Contraindications to anticoagulant therapy | 1.Coronary Angiography2.PCI  3.Aortic angiography  2.Peripheral angioplasty/ Renal angioplasty | 145 | >25% increase above baseline S. Creat. | Iopentol Low-Osm | as per intervention protocol | Hemofiltration | Hemofiltration starting 4-6 h pre-procedure; stopped during and resumed post-procedure, and continued for 18- | | Mean ±SD: 247±125 | | | Mean S. Creat. ±SD:  3.0±1.0mg/dL | |
|  |  |  |  |  |  |  | Control | Normal saline at 1ml/Kg/hour (reduced to 0.5ml/kg/h if LVEF<40%) for 6-8h pre- and 24h post-procedure | | Mean ±SD: 258±132 | | | Mean S. Creat. ±SD:  3.1±1.0mg/dL | |
| Marenzi 2006(115) | Inclusion Criteria   1. Primary angioplasty for acute STEMI within 12h of presentation (18 hours in cases of cardiogenic shock)  Exclusion Criteria   1. Long-term dialysis 2. Known allergy to N-acetylcysteine | 1.Coronary Angiography | 291 | >25% increase above baseline S. Creat. at 72 hours | Iohexol Low-Osm | Normal saline at 1ml/Kg/hour (reduced to 0.5ml/kg/h if LVEF<40%) for 12h post-procedure | NAC | Intravenous bolus of 600 mg pre-procedure and 600mg orally twice daily for 48h post-procedure, to a total dose 3000mg fter intervention (total dose of 3000 mg) | | Mean ±SD: 264±146 | | | Median S. Creat.:  1.01mg/dL | |
|  |  |  |  |  |  |  | Control  3^rd^ NAC Double Dose Group were excluded | - | | Mean ±SD: 274±113 | | | Median S. Creat.:  1.06mg/dL | |
| Marenzi 2006(115) | Inclusion Criteria   1. Severe chronic kidney disease (Creat. clearance <30mL/min 2. Diagnostic and therapeutic cardiovascular procedures  Exclusion Criteria   1. Acute coronary syndrome 2. Cardiogenic shock 3. Acute renal failure 4. Chronic peritoneal or haemodialysis  5. Overt CHF 6. Recent major bleeding 7. Contraindications to anticoagulation | 1.Coronary Angiography2.PCI | 81 | >25% increase above baseline S. Creat. | Iopentol Low-Osm | Normal saline at 1ml/Kg/hour (reduced to 0.5ml/kg/h if LVEF<40%) | Hemofiltration post-procedure | I.V isotonic saline, for 12h pre-contrast followed by hemofiltration for 18-24h post-contrast | | Mean ±SD: 237±122 | | | Mean S. Creat. ±SD:  3.6±0.7mg/dL | |
|  |  |  |  |  |  |  | Control | I.V isotonic saline for 12h pre- and 12h post-contrast | | Mean ±SD: 232±144 | | | Mean S. Creat. ±SD:  3.6±0.8mg/dL | |
|  |  |  |  |  |  |  | Hemofiltration pre- and post-procedure | Hemofiltration pre- and post-procedure | | excluded from analysis | | | | |
| Marenzi 2012(116) | Inclusion Criteria   1. Age 18-85 2. Chronic Kidney disease (eGFR <60ml/min/1.73m2)   Exclusion Criteria   1. Primary or rescue PCI 2. Angiography procedures requiring a direct renal injection of contrast 3. Cardiogenic shock 4. Overt CHF 5. Acute respiratory Insufficiency 6. Recent acute kidney injury 7. Chronic peritoneal or haemodialysis 8. Known furosemide hypersensitivity 9. Receipt of intra- venous contrast within 10 days before the procedure 10. Another planned contrast-enhanced procedure in the following 72hours 11. Contraindications to placement of a Foley catheter in the bladder | 1.Coronary Angiography2.PCI | 189 | Increase in S. Creat. ≥0.5 mg/dL (44 µmol/L) or >25% increase above baseline | Iomeprol Low-Osm | as per intervention protocol | Furosemide | Single intravenous bolus of 0.5 mg/kg (up to a maximum of 50 mg) after initial bolus of 205ml normal saline. Renal Guard system used for fluid replacement (Matched hydration) | | Mean ±SD: 181±104 | | | Mean S. Creat. ±SD:  1.8±0.6mg/dL | |
|  |  |  |  |  |  |  | Control | Normal saline at 1ml/Kg/hour (reduced to 0.5ml/kg/h if LVEF<40%) for 12h pre- and 12h post-procedure | | Mean ±SD: 158±109 | | | Mean S. Creat. ±SD:  1.7±0.5mg/dL | |
| Markota 2013(118) | Inclusion Criteria   1. Age >18   Exclusion Criteria   1. End-stage renal insufficiency (eGFR <15 mL/min) 2. Acute renal insufficiency 3. History of reaction to contrast media 4. Use of nephrotoxic MediCI-AKIes 5. Pulmonary Oedema 6. Multiple myeloma 7. Factors predisposing to kidney injury  8. Exposure to contrast media within 7 days before the procedure 9. Pregnancy 10. Non-compliance 11. Use of NAC, teofiline, dopamine, fenoldopam, manitol, or NaHCO3 within 48h of procedure | 1.Coronary Angiography2.PCI | 227 | Increase in S. Creat. ≥0.5 mg/dL (44 µmol/L) or >25% increase above baseline, OR >25% decrease in eGFR within 48h of procedure | Iopamiro 370 Low-Osm | eGFR <60 mL/min/1.73m2: IV normal saline at 1mL/kg/h for 2h pre- and 12h post-procedure.   eGFR >60 mL/min/1.73m2: Hydration orally | Na/K citrate | 5 g of granules diluted in 200 mL of water | | Mean ±SD: 222.3±102.3 | | | Mean S. Creat. ±SD:  93.96±18.39mmol/L | |
|  |  |  |  |  |  |  | Control | 200ml water | | Mean ±SD: 231.2±95.85 | | | Mean S. Creat. ±SD:  89.35±23.97mmol/L | |
| Masuda 2007(119) | Inclusion Criteria   1. Chronic kidney disease (S. Creat >1.1mg/dl or eGFR <60ml/min)  2. Emergency coronary procedure   Exclusion Criteria   1. Change in serum creatinine concentration of at least 0.5mg/dl during the previous 24 hours 2. Pre-existing dialysis 3. Recent exposure to radiographic contrast media within 2 days of the study 4. Allergy to radiographic contrast media 5. Pregnancy 6. Previous or planned administration of mannitol, fenoldopam, N-acetylcysteine or non-study sodium bicarbonate | 1.Emergency coronary procedure | 71 | Increase in S. Creat. ≥0.5 mg/dL (44 µmol/L) or >25% increase above baseline within 2days of procedure | Iopamidol Low-Osm | Initial intravenous bolus of 3ml/kg/h for 1h, if possible, pre-procedure. Same fluid at 1ml/kg/h during and for 6h post-procedure | NaHCO3 | 154 mEq/L of sodium bicarbonate | | Mean ±SD: 112±89 | | | Mean S. Creat. ±SD:  1.31±0.52mg/dL | |
|  |  |  |  |  |  |  | NaCl | 154 mEq/L sodium chloride | | Mean ±SD: 120±61 | | | Mean S. Creat. ±SD:  1.32±0.65mg/dL | |
| Matejka 2010(120) | Inclusion Criteria  1. Age > 18 years 2. S. Creat. persistently >1.47 mg/dl (130 µmol/l)  Exclusion Criteria  1. Long-term dialysis 2. Pregnancy 3. Lactation 4. Epilepsy 5. Thyrotoxicosis 6. Theophylline allergy 7. Previous theophylline medication 8. Arrhythmias with hemodynamic instability 9. Severe liver dysfunction 10. Clinical signs of dehydration 11. Inability to take oral fluids. 12. Use of ACE inhibitors 13. Use of NSAIDs | 1.Coronary Angiography2.PCI | 59 | Increase in S. Creat. ≥0.5 mg/dL (44 µmol/L) or >25% increase above baseline within 48h of procedure | Iodixanol Iso-Osm | Continuous normal saline infusion was started immediately post-procedure at 0.5ml/kg/hr, stopped on day three | Theophylline Group | 1 h infusion of 205.7 mg theophylline in 500ml 0.9% normal saline | | Mean ±SD: 95±38 | | | Mean S. Creat. ±SD:  2.02±0.45mg/dL | |
|  |  |  |  |  |  |  | Control | 500ml 0.9% normal saline | | Mean ±SD: 94±35 | | | Mean S. Creat. ±SD:  2.06±0.59mg/dL | |
| Menting 2015(121) | Inclusion Criteria   1. Interventional or diagnostic radiological procedure expected to use >100 mL intravascular contrast 2. Fulfil risk criteria for CI-AKI according to Dutch guidelines  Exclusion Criteria   1. Age <18 years 2. Haemodialysis or peritoneal dialysis 3. Simultaneous participation in another interventional study 4. Percutaneous coiling/embolization procedures of the kidney 5. Impossibility to perform RIPC 6. No written informed consent | 1. Contrast Enhanced Radiological studies | 77 | Increase in S. Creat. ≥0.5 mg/dL (44 µmol/L) or >25% increase above baseline within 48h-72h of procedure | Xenetrix Low-Osm | Normal saline at 3-4ml/Kg/hour for 4h pre- and 4h post-procedure (reduced to 1ml/kg/h for 12h pre- and post-procedure if signs of CHF or eGFR <30ml/min/1.73m^2^) | RIPC | Four cycles of ischemia and reperfusion of the forearm by inflating a blood pressure cuff around the upper arm at 50 mmHg above the actual systolic pressure for 5 minutes, followed by 5 minutes of reperfusion | | Mean ±SD: 99±29 | | | Mean S. Creat. ±SD:  115±27mmol/L | |
|  |  |  |  |  |  |  | Placebo | Placebo Group: similar cuff placed around the upper arm, but it was not inflated | | Mean ±SD: 98±29 | | | Mean S. Creat. ±SD:  119±32mmol/L | |
| Merten 2004(122) | Inclusion Criteria   1. Age >18 years 2. Stable S. Creat. >1.1mg/dL 3. Scheduled cardiac catheterization, CT, diagnostic or therapeutic arteriography, or transjugular intrahepatic portal systemic shunt placement  Exclusion Criteria   1. S. Creat. >8mg/dL 2. Change in S. Creat. >0.5mg/dL within last 24 hours 3. Pre-existing dialysis 4. Multiple myeloma 5. Pulmonary oedema 6. Uncontrolled hypertension 7. Emergency catheterization 8. Recent exposure to radiographic contrast within 2days of the study 9. Allergy to radiographic contrast 10. Pregnancy 11. Administration of dopamine, mannitol, fenoldopam, or NAC during the study. | 1.Angiography  2.CT  3.TIPSS | 128 | >25% increase above baseline S. Creat. within 2days of procedure | Iopamidol Low-Osm | Assigned fluid at 3mL/kg per hour for 1h immediately before radiocontrast injection, followed by same fluid at 1mL/kg/h during contrast exposure and for 6 h post-procedure | NaCl | 154 mEq/L of sodium chloride in D5W | | Mean ±SD: 134±63 | | | Mean S. Creat. ±SD, (Range):  1.71±0.42 (1.1-3.7) mg/dL | |
|  |  |  |  |  |  |  | NaHCO3 | NaHco3 Group: 154 mEq/L of sodium bicarbonate in D5W | | Mean ±SD: 130±72 | | | Mean S. Creat. ±SD, (Range):  1.89±0.69 (1.2-5.2) mg/dL | |
| Miao 2013(123) | Inclusion Criteria  1. Age ≥ 70 years 2. Stable clinical state 3. Scheduled for contrast enhanced CT  Exclusion Criteria   1. Uncontrolled DM, hypertension, CHF (LVEF <40%), or other uncontrolled clinical diseases 2. Fever or infectious diseases 3. Unstable renal function or chronic renal failure [S. Creat >265.2 mmol/l] 4. Hypersensitivity to iodine-containing compounds or hyperthyroidism 5. Hypersensitivity to Alprostadil 6. Ulcers 7. Coagulation abnormalities 8. Other diseases with contraindications to alprostadil 9. Patients who had received any iodinated contrast agent or other drugs that affect renal function within 7 days | 1.CE-CT | 383 | Increase in S. Creat. ≥0.5 mg/dL (44 µmol/L) or >25% increase above baseline within 3days of procedure | Low-Osm Iohexol | I.V Fluid >800 ml 6h pre- and post-CE-CT | PGE1 | Alprostadil (0.4lg/kg/day) in 100 ml sterile saline pre- and post-procedure | | 100 | | | Mean S. Creat. ±SD:  87.18±24.6mmol/L | |
|  |  |  |  |  |  |  | Placebo | 100 ml sterile saline | | 100 | | | Mean S. Creat. ±SD:  88.11±24.19mmol/L | |
| Miner 2004(124) | Inclusion Criteria  1. Planned PCI or urgent coronary angiography with a high likelihood of ad hoc PCI. 2. Creatinine Clearance <50 mL/min, (<100mL/min if Diabetic) or S. Creat. >200 µmol/L   Exclusion Criteria  1. Renal replacement therapy 2. Reactive airway disease requiring oral steroids 3. Baseline SBP <80 mm Hg 4. Active CHF 5. Acute MI 6. Enrolment in another clinical trial 7. Inability to provide informed consent 8. Ongoing need for intravenous nitroglycerin 9. Treatment with NAC within 72 hours of planned PCI 10. Women of childbearing age | 1.Coronary Angiography2.PCI | 208 | >25% increase above baseline S. Creat. 48-72h | Omnipaque Iso-Osm | 0.45% saline at 75 mL/hour for at least 24 hours beginning at the time of enrolment. Changes in hydration were allowed at the discretion of the cardiologist | NAC | 6000mg (4000mg for day / case patients) | | Mean ±SD: 344±211 | | | Mean S. Creat. ±SD:  124±49mmol/L | |
|  |  |  |  |  |  |  | Placebo | IV hydration only | | Mean ±SD: 350±187 | | | Mean S. Creat. ±SD:  130±58mmol/L | |
| Minoo 2016(199) | Inclusion Criteria:  Patients ≥ 35 years undergoing elective coronary angiography  Exclusion Criteria:  1. Baseline serum creatinine concentrations greater than 1.5 mg/dL,  2. Need for emergency catheterization  3. Receiving contrast media for diagnostic or therapeutic interventions in the past 3 months  4. Uncontrolled congestive heart failure  5. Uncontrolled chronic obstructive pulmonary disease  6. History of allergy to contrast  Media  7. Pregnancy or lactation | 1.Elective coronary angiography | 453 | Increase of 25% or more in serum creatinine concentrations, or an increment of at least 0.5 mg/dL in serum creatinine concentrations 48 hours after catheterization | Iopromide  Low-Osm | normal saline at the dose of 1 mL/kg/h, 12 hours leading to the procedure and 12 hours after the procedure | Oxygen | nasal cannula at the rate of 2 L/min to 3 L/min beginning 10 minutes before the procedure until the end of the procedure | | Median (IQR):  200 (100-250 | | | Mean S. Creat. ±SD:  0.96 ± 0.24 | |
|  |  |  |  |  |  |  | Control | I.V hydration only | | Median (IQR):  150 (100-257) | | | Mean S. Creat. ±SD:  0.93 ± 0.15 | |
| Moore 2006(125) | Inclusion Criteria  1. Elective EVAR | 1.EVAR | 23 | >25% increase above baseline S. Creat. | Niopam Low-Osm | Median IV fluids received (IQR): NAC: 5.9 (3.0–5.75) L Control: 4 (3–7) L | NAC | 600 mg orally twice daily for 2 days, starting day pre-procedure (total 3 doses pre-op) | | Median (IQR): 258 (210-285) | | | Median S. Creat. (IQR):  102 (76-112) mmol/L | |
|  |  |  |  |  |  |  | Control | IV hydration only | | Median (IQR): 258 (200-355) | | | Median S. Creat. (IQR):  86 (81.5-99) mmol/L | |
| Morikawa 2009(126) | Inclusion Criteria   1. Chronic renal insufficiency (S. Creat. 1.3-6mg/dl)  Exclusion Criteria   1. Pregnancy 2. Lactation 3. Acute renal failure 4. End-stage renal failure on dialysis 5. Acute MI 6. Multiple myeloma 7. Pulmonary oedema 8. Cardiogenic shock 9. SBP < 110 mm Hg 10. Dehydration 11. History of allergies to contrast media or ANP 12. Received contrast media within 7 days of the study entry 13. Received an infusion of ANP within 1 month of the study entry 13. Parenteral use of diuretics 14. Administration of dopamine, N-acetylcysteine, metformin, NaHco3, fenoldopam, mannitol, or NSAIDs during the study | 1.Coronary Angiography2.PCI | 273 | Increase in S. Creat. ≥0.5 mg/dL (44 µmol/L) or >25% increase above baseline within 48h of procedure | Iomeprol Low-Osm | oral hydration encouraged | ANP | 0.042 µg/kg/min of ANP + 1.3ml/kg/h Ringer's Lactate IV 4-6h pre- and continued for 48h post-procedure | | Mean ±SD: 139±62 | | | Median S. Creat. (IQR):  1.57(1.34-1.95) mg/dL | |
|  |  |  |  |  |  |  | Control | IV hydration only | | Mean ±SD: 140±72 | | | Median S. Creat. (IQR):  1.55(1.36-1.90) mg/dL | |
| Motohiro 2011(127) | Inclusion Criteria  1. Age > 20years  2. eGFR < 60ml/min/1.73m2   Exclusion Criteria   1. S. Creat. >4 mg/dl 2. Change in S. Creat. >0.5 mg/dl in preceding 24 hours 3. Pre-existing dialysis 4. Pulmonary oedema 5. Uncontrolled hypertension (treated SBP >160 mmHg or DBP >100 mmHg) 6. Emergency catheterization 7. Exposure to radiographic contrast within previous 2 days 8.Any Allergy to radiographic contrast medium | 1.Coronary Angiography | 167 | Increase in S. Creat. ≥0.5 mg/dL (44 µmol/L) or >25% increase above baseline within 48h of procedure | Iopamidol Low-Osm | 0.9% sodium chloride at 1ml/kg/h for 12h pre- and post-procedure | NaHCO3 | 1ml/kg/hour continued from 3 hours pre- to 6 hours post-procedure | | Mean ±SD: 140±50 | | | Mean S. Creat. ±SD:  1.54±0.43mg/dL | |
|  |  |  |  |  |  |  | Control | IV hydration only | | Mean ±SD: 130±40 | | | Mean S. Creat. ±SD:  1.55±0.44mg/dL | |
| Nawa 2015(200) | Inclusion Criteria:  Patients with poor renal function and who had a high cystatin C level (> 0.95 mg/L in males and 0.87 mg/dL in females)  Exclusion Criteria:  1. End-stage renal failure on dialysis, a single functioning kidney, or history of kidney transplantation  2. Hypotension with systolic blood pressures below 100 mmHg  3. Acute myocardial infarction  4. Acute heart failure, left ventricular ejection fraction (LVEF) less than 30% on echocardiogram or evidenced by pulmonary edema  5.Multiple myeloma  6. Pregnancy  7. History of allergies to CM or nicorandil  8. Received CM within 7 days of study or nicorandil within 1month  9. Parenteral use of diuretics, and the administration of NAC, Metformin, NaHco3, theophylline, fenoldopam, mannitol, or a phosphodiesterase inhibitor during the study. | 1.Elective PCI | 204 | 25% increase in serum creatinine or an increase in creatinine of 0.5mg/dL from base- line at 48 h | Iomeprol  Low-Osm  Iohexol  Low-Osm | 0.9% saline hydration intravenously infused at 1.0 mL/kg/h (nicorandil group) or 0.9% saline infusion only at 1.1 mL/kg/h  Infusions were initiated 4 h prior to elective PCI and were continued for 24 h after the procedure | Nicorandil | 2 vials (48 mg/V) dissolve in 100 mL 0.9% saline, and dripped it at speed of 0.1 mL/kg/h) | | Mean+SD:  135.2±57.0 | | | Mean S. Creat. ±SD:  0.99±0.29 | |
|  |  |  |  |  |  |  | Control | I.V saline Only | | Mean+SD:  146.3±63.6 | | | Mean S. Creat. ±SD:  1.02±0.35 | |
| Ng 2006(128) | Inclusion Criteria   1. High risk for the development of CI-AKI  2. Pre-existing stable renal insufficiency (S. Creat. >1.2 mg/dL with no change greater than ±0.1m mg/dL)   Exclusion Criteria  1. Acute Renal failure 2. History of renal transplantation 3. Receiving N-acetylcysteine or fenoldopam 4. Known contraindication or hypersensitivity to N-acetylcysteine or fenoldopam 5. Pregnancy | 1.Coronary Angiography | 97 | Increase in S. Creat. ≥0.5 mg/dL (44 µmol/L) or >25% increase above baseline | Omnipaque Visipaque Hexabrix. | Normal saline (or 5% dextrose in normal saline in diabetics on insulin) at 1mL/kg/h beginning 1–2 h pre-procedure and continuing for 6–12h | NAC | 600 mg orally twice daily for 2 days, starting day pre-procedure (total 4 doses) | | Mean ±SD: 172.2±73.2 | | |  | |
|  |  |  |  |  |  |  | Fenoldopam | 0.1 mcg/kg/min IV commenCI-AKIg 1–2 h pre- and continued for 6h post-procedure | | Mean ±SD: 164.4±85 | | |  | |
| Nijssen 2017(201) | Inclusion Criteria:  Patients aged 18 years and older, referred for an elective procedure requiring intravascular iodinated contrast material with estimated glomerular filtration rate (eGFR) between 45 and 59 mL per min/1·73 m² combined with either diabetes, or at least two predefined risk factors (age >75 years; anaemia defined as haematocrit values <0·39 L/L for men, and <0·36 L/L for women; cardiovascular disease; non-steroidal anti-inflammatory drug or diuretic nephrotoxic medication); or eGFR between 30 and 45 mL per min/1·73 m²; or multiple myeloma or lymphoplasmacytic lymphoma with small chain proteinuria  Exclusion Criteria:  1. Inability to obtain informed consent  2. eGFR <30 mL per min/1·73 m²  3. Renal replacement therapy, emergency procedures  4. Intensive care patients  5. Known inability to plan primary endpoint data collection  6. No referral for prophylactic hydration  7. Participation in another randomised trial  8. Isolation (infection control). | 1.Elective procedure with intravascular contrast media administration | 599 | Increase in serum creatinine by more than 25% or 44 µmol/L within 2–6 days of contrast exposure (2–5 days was aimed for, but day 6 was allowed if no other option was available) | Iopromide  Low-Osm | See Groups | Hydration | standard protocol intravenous 0·9% NaCl 3–4 mL/kg per h during 4 h before and 4 h after contrast administration | | Contrast(ml) Mean(SD):  92 (41) | | | S. Creat.  Mean(SD):  118·78 (27·63) | |
|  |  |  |  |  |  |  | Non-Hydration (Control) | No Hydration | | Contrast(ml) Mean(SD):  89 (41) | | | S. Creat.  Mean(SD):  117·71 (24·62) | |
| Ochoa 2004(129) | Inclusion Criteria   1. Elective or urgent coronary angiography ± PCI  2. Chronic renal insufficiency (S. Creat. >1.8 mg/dL males, >1.6 mg/dL females), or Creat. Clearance <50mL/min   Exclusion Criteria   1. >0.5 mg/dL increase S. Creat. in preceding 6 weeks 2. Current or planned dialysis 3. Contrast exposure within preceding 48 hours 4. Known allergy to NAC 5. History of anaphylactic reaction to contrast 6. Recent decompensated CHF within preceding 4 weeks 7. Cardiogenic shock or use of intravenous vasopressors within preceding 1 week 8. Known or suspected severe aortic valve stenosis (area <1.0 m2, mean gradient >50 mmHg) 9. Recent (<4 weeks) initiation of diuretics or ACE inhibitors | 1.Coronary Angiography2.PCI | 94 | Increase in S. Creat. ≥0.5 mg/dL (44 µmol/L) or >25% increase above baseline within 48h of procedure | Ioxaglate Low-Osm Iohexol Low-Osm Iodixonal Iso-Osm | Normal saline at 150 mL/h beginning 4 hours pre- and continued for 6 hours post-procedure | NAC | 1000 mg [5 mL] diluted in 20 mL of diet cola)  administered orally 1h pre- and 4h post-procedure | |  | | | Mean S. Creat. ±SD:  2.02±0.56mg/dL | |
|  |  |  |  |  |  |  | Placebo | 5mL 0.9% normal saline diluted in 20 mL of diet cola)  administered orally 1h pre- and 4h post-procedure | |  | | | Mean S. Creat. ±SD:  1.93±0.53mg/dL | |
| Oguzhan 2013(130) | Inclusion Criteria   1. S. Creat. <2.1 mg/dL  Exclusion Criteria   1. Acute STEMI 2. NYHA class IV CHF 3. Hemodynamic instability (SBP<90mmHg on ≥2 measurements or patients requiring pressors) 4. Exposure to contrast within preceding 7 days 5. Use of a nephrotoxic drug within preceding 48h 6. Contraindication to amlodipine or valsartan | 1.Coronary Angiography | 101 | Increase in S. Creat. ≥0.5 mg/dL (44 µmol/L) or >25% increase above baseline within 48-72h of procedure | Iopromide  Low-Osm | Isotonic sodium chloride 1 mL/kg/h for 12h pre- and post-procedure | Amlodipine /Valsartan Group: | Amlodipine/Valsartan (5/160mg) mané commenCI-AKIg day prior to procedure (total 3 doses) | | Median (Range): 60 (30-200) | | | Mean S. Creat. ±SD:  1.13±0.33mg/dL | |
|  |  |  |  |  |  |  | Control | IV hydration only | | Median (Range): 60 (25-250) | | | Mean S. Creat. ±SD:  1.07±0.23mg/dL | |
| Oldemeyer 2003(131) | Inclusion Criteria   1. Age ≥19 years 2. Creat. clearance <50 mL/min and S. Creat > 1.2mg/dL 3. Elective coronary angiography  Exclusion Criteria  1. Acute kidney failure 2. Dialysis 3. Unstable renal function (Change ≥0.5 mg/dL or ≥25% in S. Creat. in preceding 10 days) 4. Known allergy to contrast or acetylcysteine 5. Administration of mannitol, intravenous catecholamines, parenteral diuretics, theophylline, or a contrast agent within days of study entry 6. Mechanical ventilation 7. Cardiogenic shock 8. Emergent angiography. | 1.Coronary Angiography | 103 | Increase in S. Creat. ≥0.5 mg/dL (44 µmol/L) or >25% increase above baseline within 24-48h of procedure | Iopamidol Low-Osm | (0.45%) saline at 1 mL/kg/h for 12h pre- and 12h post-procedure | NAC | 1500 mg BD orally in 120 mL of carbonated beverage commenced evening pre-procedure (total 4 doses) | | Mean ±SD: 134±71 | | |  | |
|  |  |  |  |  |  |  | Placebo | equivalent volume of normal saline BD orally in 120 mL of carbonated beverage commenCI-AKIg evening pre-procedure (total 4 doses) | | Mean ±SD: 127±73 | | |  | |
| Onbasili 2007(132) | Inclusion Criteria   1. S. Creat ≥1.2 mg/dl (≥106 mmol/l) or creat. clearance <50 ml/min   Exclusion Criteria  1. Acute renal failure 2. Acute MI requiring primary or rescue coronary intervention 3. Cardiogenic shock 4. Renal replacement treatments 5. Known allergy to Trimetazidine | 1.Coronary Angiography2.Angioplasty | 90 | Increase in S. Creat. ≥0.5 mg/dL (44 µmol/L) or >25% increase above baseline within 24-48h of procedure | Iopramide Low-Osm | Isotonic saline at 1 mL/kg/h for 12h pre- and 12h post-procedure | Trimetazidine | 20 mg TDS orally for 72h starting 48h pre-procedure | | Median: 225 | | | Mean S. Creat. ±SD:  1.31±0.25mg/dL | |
|  |  |  |  |  |  |  | Control | IV hydration only | | Median: 240 | | | Mean S. Creat. ±SD:  1.26±0.16mg/dL | |
| Ozcan 2007(133) | Inclusion Criteria   1. S. Creat. >1.2 mg/dL  Exclusion Criteria   1. Uncontrolled hypertension (SBP>160 mm Hg and DBP>110 mm Hg) 2. Emergency catheterization 3. Recent exposure to radiocontrast medium within preceding 2 days 4. Volume overload 5. S. Creat. levels >4 mg/dL | 1.Coronary Angiography2.PCI | 192 | Increase in S. Creat. ≥0.5 mg/dL (44 µmol/L) or >25% increase above baseline within 48h of procedure | Ioxaglate Low-Osm | Assigned fluid at 1 mL/kg/h (max 100ml/hr) for 6h pre- and 6h post-procedure | NaCl | 1 mL/kg/h (max 100ml/hr) for 6h pre- and 6h post-procedure | | Median (Range): 110 (30-270) | | | Mean S. Creat. (Range):  1.40 (1.2-2.3) mg/dL | |
|  |  |  |  |  |  |  | NaHCO3 | 154 mL of 1000-mEq/L NaHCO3 in 846 mL of D5W at 1 mL/kg/h (max 100ml/hr) for 6h pre- and 6h post-procedure | | Median (Range): 100 (50-300) | | | Mean S. Creat. (Range):  1.36 (1.2-3.8) mg/dL | |
|  |  |  |  |  |  |  | NAC+ NaCl: | Excluded from analysis | | | | | | |
| Ozhan 2010(134) | Inclusion Criteria:   1. Patients undergoing coronary angiography  Exclusion Criteria   1. Known allergy for contrast agents 2. Renal insufficiency (S. Creat. >1.5 mg/dL or eGFR <70 mL/min | 1.Coronary Angiography | 139 | Increase in S. Creat. ≥0.5 mg/dL (44 µmol/L) or >25% increase above baseline | Iopamidol Iso-Osm | 1L saline infusion in 6h post-procedure | Statin | 80 mg atorvastatin plus 600 mg NAC twice daily on day of procedure followed by 80 mg atorvastatin for 2 days after the procedure | |  | | | Mean S. Creat. ±SD:  0.88±0.2mg/dL | |
|  |  |  |  |  |  |  | Control | 600 mg NAC twice daily on day of procedure | |  | | | Mean S. Creat. ±SD:  0.88±0.19mg/dL | |
| Pakfetrat 2009(135) | Exclusion Criteria  1. Exposure to contrast media within preceding two days 2. Hypotension 3. Intra-aortic balloon pump 4. Pulmonary oedema 5. Dialysis 6. Electrolyte and acid-base disturbances 7. Known sensitivity to Acetazolamide 8. Medications affecting Renal Function e.g. mannitol, dopamine, and theophylline 9. Unwilling to give written informed consent | 1.Coronary Angiography2.PCI | 311 | RIFLE criteria: S. Creat. increased by 1.5-2 times baseline | Iodixanol Iso-Osm |  | NaHCO3 | 3 mL/kg of 154mEq/l NaHCO3 in Dextrose solution for 1 hour before coronary angiography, decreased to 1 mL/kg/hr for 6 hours post-procedure | | Mean ±SD: 58±32.7 | | | Mean S. Creat. ±SD:  1.1±0.3mg/dL | |
|  |  |  |  |  |  |  | NaCl Group: | 1 ml/kg/h starting 6h pre- and continued 6h post-procedure | | Mean ±SD: 67±41.1 | | | Mean S. Creat. ±SD:  1.1±0.2mg/dL | |
|  |  |  |  |  |  |  | Acetazolamide Group: | 250 mg orally 2h pre- and 6h post-contrast | | Mean ±SD: 70.1±67.9 | | | Mean S. Creat. ±SD:  1.1±0.3mg/dL | |
| Patti 2011(136) | Inclusion Criteria  1. Statin naive patients with non–STEMI or unstable angina 2. Planned intervention within 48 hours  Exclusion Criteria  1.Current or previous (<3 months) statin treatment 2. Non-STE ACS with high-risk features warranting emergency coronary angiography (<2 hours) 3. Any baseline increases in liver enzymes  4. LVEF <30% 5. Renal failure with S. Creat. > 3mg/dl 6. History of liver or muscle disease. | 1.Coronary Angiography2.PCI | 263 | Increase in S. Creat. ≥0.5 mg/dL (44 µmol/L) or >25% increase above baseline | Iobitridol Low-Osm | Patients with pre-existing renal failure (S. Creat. ≥1.5 mg/dl or CrCl ≥60 ml/min) received IV hydration with normal saline at 1ml/hour/kg for ≥12h pre- and ≥24 hours post-procedure | Statin | 80-mg loading dose of Atorvastatin given ~12h pre-procedure, with a further 40mg ~2h pre-procedure | | Mean ±SD: 209±72 | | | Mean S. Creat. ±SD:  79.8±29.4mmol/l | |
|  |  |  |  |  |  |  | Placebo | matched placebo | | Mean ±SD: 213±13 | | | Mean S. Creat. ±SD:  77±27.6mmol/l | |
| Poletti 2013(137) | Inclusion Criteria   1. Patients admitted as an emergency requiring CT 2. Creat. clearance <60 ml/min/1.73 m2 by MDRD (eGFR ~42 ml/min/1.73 m2) and need CT Scan   Exclusion Criteria  1. Asthma 2. Pregnancy  3. Obstructive nephropathy 4. Patient refusal | 1.CT | 128 | Increase in S. Creat. ≥0.5 mg/dL (44 µmol/L) or >25% increase above baseline | Low-Osm Iohexol (Accupaque®) | 250 ml of 0.45% NaCl pre-CT and 1L post-CT | NAC | 6000 mg NAC diluted in 100 ml 0.45% normal saline IV 1h pre-CT | | Mean ±SD: 117.4±1.8 | | | Mean S. Creat. ±SD:  132.4±34.8mmol/l | |
|  |  |  |  |  |  |  | Placebo | matched placebo | | Mean ±SD: 117.7±3.2 | | | Mean S. Creat. ±SD:  133.5±34.8mmol/l | |
| Qiao 2015(138) | Inclusion Criteria   1. Diabetes Mellitus 2. Mild-to-moderate chronic kidney disease (eGFR 30-89 ml/min/1.73 m2 3. Total contrast volume ≥ 100 ml  Exclusion Criteria   1. Pregnancy 2. Lactation 3. Ketoacidosis 4. Lactic acidosis 5. Contrast administration within 7 days of study  6. Emergent coronary angiography 7. History of hypersensitivity reaction to contrast or statins 8. NYHA class IV CHF 9. Unstable renal function 10. Use of: Aminophylline, Prostaglandin or E1 within 7 days of the procedure. 11. Recent statin use within 14 days | 1.PCI | 124 | Increase in S. Creat. ≥0.5 mg/dL (44 µmol/L) or >25% increase above baseline within 72h | Iso-Osm Iodixanol | Isotonic saline 0.9% at 1-1.15 mL/kg/h for 3-12h pre- and 6- 24 hours post-procedure | Statin | Rosuvastatin 10 mg/day) 2 days pre-and 3 days post-contrast | | Mean ±SD: 204.3±74.7 | | | eGFR 30-89 ml/min/1.73 m^2^ | |
|  |  |  |  |  |  |  | Control | IV hydration only | | Mean ±SD: 212.3±84.6 | | |  |  |
| Quintavalle 2012(139) | Inclusion Criteria  1. Statin-naïve patients 2. Elective coronary angiography due to symptomatic coronary artery disease OR PCI in de novo lesions in native coronary  Exclusion Criteria   1. Primary or rescue PCI 2. ACS with elevated cardiac biomarkers 3. Coronary artery restenosis 4. Treatment of a saphenous vein graft or left internal mammary artery graft 5. Active statin therapy | 1.Coronary Angiography2.PCI | 430 | Increase in S. Creat. ≥0.5 mg/dL (44 µmol/L) or >25% increase above baseline within 48h | Iodixanol Iso-Osm | NAC 1200mg orally twice daily, the days pre- and of contrast, as well as hydration with 154 mEq/L NaHCO3 in dextrose and H2O. with the initial IV bolus of 3 mL/kg/h for 1 hour immediately before CM injection, followed by 1 mL/kg/h during and for 6 h post-procedure | Statin | Atorvastatin (80 mg) within 24 hours | | Mean ±SD: 177±74 | | | Mean S. Creat. (Range):  1.16 (1.0-1.32) mg/dL | |
|  |  |  |  |  |  |  | Control | IV hydration only | | Mean ±SD: 184±78 | | | Mean S. Creat. (Range):  1.18 (1.0-1.35) mg/dL | |
| Rahman 2012(140) | Inclusion Criteria   1. Elective coronary angiography for ACD, chronic stable Angina, dilated/ischemic cardiomyopathy and preoperative assessment 2. S. Creat. 1.2-2.5mg/dl  Exclusion Criteria   1. Diabetes Mellitus 2. Acute renal failure  3. Acute MI requiring primary or rescue PCI 4. Cardiogenic shock 5. Patients on renal replacement therapy 6. Patients undergoing PTCA 7. COPD 8. Exacerbation of asthma  9. Allergy to TMZ | 1.Coronary Angiography | 436 | Increase in S. Creat. ≥0.5 mg/dL (44 µmol/L) or >25% increase above baseline within 24-48h | Iopamiro Low-Osm | Normal saline 1mg/kg/h for at least 12h pre- and 12h post-procedure | Trimetazidine | 35mg twice daily commenCI-AKIg 48h pre-procedure, for 96h | | Mean ±SD: 95.34±4.25 | | | Mean S. Creat. ±SD:  1.23±0.23mg/dl | |
|  |  |  |  |  |  |  | Control | IV hydration only | | Mean ±SD: 97.45±5.62 | | | Mean S. Creat. ±SD:  1.4±0.24mg/dl | |
| Rashid 2004(141) | Inclusion Criteria   1. Peripheral vascular disease  2. Elective angiography or angioplasty 3. Candidates subdivided into 2 groups: i. Normal S. Creat. (<120mmol/L[1.32mg/dl] for men and <97mmol/L[1.07mg/dl] for women) | 1.Peripheral Angiography  2.Angioplasty | 100 | Increase in S. Creat. ≥0.5 mg/dL (44 µmol/L) or >25% increase above baseline within 48h | Omnipaque Low-Osm | All patients received normal saline (500 mL over 4-6 hours) 6-12h pre-and post-procedure | NAC | 1g per bag of normal saline | | Mean ±SD: 135.4±62.7 | | | Mean S. Creat. ±SD:  109.9±41.15mmol/L | |
|  |  |  |  |  |  |  | Control | IV hydration only | | Mean ±SD: 151.2±75.6 | | | Mean S. Creat. ±SD:  124.3±63.47mmol/L | |
| Reinecke 2007(142) | Inclusion Criteria   1. S. Creat. 1.3-3.5 mg/dl  2. Elective coronary angiography≥ 1.3 mg/dl-3.5 mg/dl  Exclusion Criteria   1. Acute or recent MI within 30 days 2. NYHA class IV CHF 3. Recipient of transplanted organs 4. Monoclonal gammopathy 5. Previous contrast medium administration within 7 days | 1.Coronary Angiography | 431 | Increase in S. Creat. ≥0.5 mg/dL (44 µmol/L) 48-72h post-procedure | Iopromide Iso-Osm | 500 ml 5% glucose and 500 ml isotonic NaCl over 12h pre- and for 12h post-procedure | NAC | 600 mg evening pre-procedure, second dose morning pre-procedure, third evening post-procedure, and the last dose was given on morning post-procedure | | Mean ±SD: 197±80 | | | Mean S. Creat. (Range):  1.5 (1.3-1.9) mg/dl | |
|  |  |  |  |  |  |  | Dialysis | Haemodialysis performed within 20 min after catheterization | | Mean ±SD: 184±80 | | | Mean S. Creat. (Range):  1.5 (1.3-2.2) mg/dl | |
|  |  |  |  |  |  |  | Control | IV hydration only | | Mean ±SD: 188±79 | | | Mean S. Creat. (Range):  1.4 (1.3-1.9) mg/dl | |
| Rezaei 2016(202) | Inclusion Criteria:  patients aged ≥18 years with baseline estimated glomerular filtration rate (eGFR) <60 mL/min per 1.73 m2  Exclusion Criteria:  1. Acute ST-segment elevation  myocardial infarction or high-risk NSTE-ACS warranting emergency coronary angiography (<2 hours)  2. Cardiogenic shock  3. Pulmonary edema, overt heart failure and/or ejection fraction <30%  4. ACS undergoing coronary angiography or angioplasty during the previous 5 days  5. Sensitivity to contrast medium, recent administration of contrast medium for any reason  6. AKI or history of dialysis  7. Pregnancy  8. Newly prescribed angiotensin-converting enzyme inhibitors or angiotensin receptor blockers  9. Bleeding and/or coagulopathy disease  10 Consumption of nephrotoxic drugs, vitamin E, vitamin C, or NAC at least 48 hours before intervention | 1.Coronary Angiography | 329 | Absolute increase ≥0.5 mg/dL or a relative increase ≥25% over baseline serum creatinine concentration within 72 hours after administration of contrast media | Iodixanol  Iso-Osm | See Groups | vitamin E  (a-tocopherol) | 0.9% saline infusions (1 mL/kg) for 12 hours prior to and after coronary angiography combined with 600 mg oral vitamin E at 12 hours before plus 400 mg  2 hours before intervention | | Median (IQR):  1.3(1.2-1.5) | | | S CrMedian (IQR):  1.3(1.2-1.5) | |
|  |  |  |  |  |  |  | Placebo | Matching placebo (No Vit E) | | Median (IQR):  1.3(1.2-1.5) | | | S CrMedian (IQR):  1.3(1.2-1.5) | |
| Rohani 2010(143) | Inclusion Criteria  1. Chronic renal failure (stable S. Creat. >1.3 mg/dl)   Exclusion Criteria   1. Pregnancy 2. Contraindications to aminophylline (history of seizures, arrhythmia resulting in circulatory instability) | 1.Coronary Angiography | 70 | Increase in S. Creat. ≥0.5 mg/dL (44 µmol/L) 48h post-procedure | Omnipaque Low-Osm | Isotonic crystalloid 1.0-1.5 ml/kg/hr for 3-12 h pre- and for 6-24 hours post-procedure. Additional hydration was performed according to clinical examination, X-ray, and central venous pressures, if available. | Aminophylline | 250mg as a short infusion (100ml saline, 0.9%) 30 min pre-procedure | | Mean ±SD: 200±89 | | | Mean S. Creat. ±SD:  1.93±0.21mg/dl | |
|  |  |  |  |  |  |  | Placebo | Short infusion of 100 ml saline, 0.9% 30 min pre-procedure | | Mean ±SD: 210±90 | | | Mean S. Creat. ±SD:  1.84±0.54mg/dl | |
| Sadat 2011(144) | Inclusion Criteria  1. Peripheral Arterial Disease  Exclusion Criteria   1. Established renal failure 2. Renal replacement therapy | 1.Peripheral Angiography | 44 | >25% increase above baseline S. Creat. 72h post-procedure | Iopamidol  Low-Osm | 1L 0.9% normal saline IV infusion over 12h pre- and 1L over 12h post-procedure | NAC | 600 mg twice daily on the day pre- and 600 mg twice on the day of procedure | | Mean ±SD: 70±20 | | | Mean S. Creat. (Range):  97 (72-125) mmol/l | |
|  |  |  |  |  |  |  | Control | IV hydration only | | Mean ±SD: 75±25 | | | Mean S. Creat. (Range):  88 (68-142) mmol/l | |
| Sadineni 2017(203) | Inclusion Criteria:  Patients > 30 years undergoing coronary angiography ± PCI for angina, non-ST-segment elevation myocardial infarction (NSTEMI) and acute myocardial infarction/STEMI with serum creatinine ≥1.2 mg/dl on their most recent sample drawn within 3 months of planned procedure.  Exclusion Criteria:  1. Patients with acute renal failure or endstage renal disease requiring dialysis  2. Intravascular administration of contrast material within previous 6 days  3. Pregnancyor lactation  4. Emergency coronary angiography  5. History of hypersensitivity reaction to contrast media  6. Cardiogenic shock  7. Pulmonary edema  8. Mechanical ventilator  9. Parenteral use of diuretics  10 Recent use of NAC or ascorbic acid  11. Use of metformin or NSAIDS within 48 h of procedure | 1.Coronary angiography  2.PCI (non-emergency) | 118 | Relative increase in serum creatinine from baseline of ≥25% or an absolute increase of ≥0.3 mg/dl (44.2 µmol/L) during days 1 and 2. | Iodixanol  Iso-Osm | Normal saline 0.5 ml/kg/h 12 h prior to the procedure and was continued for 12 h after contrast administration (total 24 h). Patients who had low ejection fraction (<40%) received NS at rate of 0.3 ml/kg/h | NAC | | 600 mg orally twice daily, the day before and the day of the procedure | Mean+SD:  61.4±34.8 | | Mean S. Creat. ±SD:  2.24±0.9 | | |
|  |  |  |  |  |  |  | Allopurinol | | 300 mg single dose given the day before the procedure | Mean+SD:  68.7±46.77 | | Mean S. Creat. ±SD:  1.91±0.72 | | |
|  |  |  |  |  |  |  | Placebo | | Normal saline only | Mean+SD:  77.33±43.30 | | Mean S. Creat. ±SD:  2.19±1.01 | | |
| Saitoh 2011(145) | Inclusion Criteria   1. S Creat. >1.5 mg/dl ± creatinine clearance <60 ml/min 2. Elective coronary angiography | 1.Coronary Angiography | 23 | Increase in S. Creat. ≥0.5 mg/dL (44 µmol/L) or >25% increase above baseline within 48h | Iomeprol Low-Osm | 0.9% saline at 1 ml/kg/h for 24 h started 12 h pre-procedure and continued until 12h after | NAC | 704 mg twice daily orally from 1 day pre-procedure for a total of 2 days | | Mean ±SD: 117.1±9.0 | | |  | |
|  |  |  |  |  |  |  | Control | IV hydration only | | Mean ±SD: 113.6±14.5 | | |  |  |
|  |  |  |  |  |  |  | Glutathione | 100mg/min from 30min pre-procedure to max of 3000mg | | Mean ±SD: 130.7±19.3 | | |  |  |
| Sandhu 2006(146) | Inclusion Criteria   1. Non-coronary diagnostic angiography (head and neck, extremity and visceral abdominal angiography) 2. With or without renal impairment  Exclusion Criteria   1. Acute renal failure 2. Renal transplant | 1.Diagnostic angiography | 109 | Increase in S. Creat. ≥0.5 mg/dL (44 µmol/L) above baseline within 48h | Iodixanol Iso-Osm Iopamiol Low-Osm |  | NAC | 600 mg twice daily the day pre- and day of the procedure | |  | | | Mean S. Creat. ±SD:  116±48.9mmol/l | |
|  |  |  |  |  |  |  | Control | no intervention | |  | | | Mean S. Creat. ±SD:  103.6±48.6mmol/l | |
| Sanei 2014(147) | Inclusion Criteria  1. Elective CTA 2. Normal renal function  Exclusion Criteria   1. Unstable angina 2. MI 3. Cardiac arrhythmias 4. Heart failure 5. Acute or chronic renal failure 6. S. Creat. level >1.5 mg/dl 7. IV administration of contrast material in the past month 8. Known hypersensitivity to statins 9. Lost to follow-up | 1.CTA | 247 | Increase in S. Creat. ≥0.5 mg/dL (44 µmol/L) or >25% increase above baseline within 48h | Low-Osm Iopromide |  | Statin | 80mg dose of Atorvastatin daily commenCI-AKIg 24h pre- and continued for 48h post-procedure | | All patients received a total of 100 ml of the contrast material; 15 ml for the test bolus and 85 ml for the imaging (6ml/s injected with injector device) | | | Mean S. Creat. ±SD:  1.0±0.16mg/dl | |
|  |  |  |  |  |  |  | Placebo | matched placebo | |  |  |  | Mean S. Creat. ±SD:  1.03±0.17mg/dl | |
| Sar 2010(148) | Inclusion Criteria   1. Type 2 Diabetes Mellitus 2.Normal renal function  3. Elective radiological investigation requiring intravenous contrast media administration   Exclusion Criteria   1. BMI <21 or > 30 2. Concomitant systematic disease  3. Use of nephrotoxic drug or drug that can interact with ACEI within the last 30 days | 1.CT | 48 | Increase in S. Creat. ≥0.3 mg/dL or >20% increase above baseline, or >20% decrease in eGFR | Low-Osm Iohexaol | Normal saline 0.9% at 1ml/kg/h for 12h pre- and 24h post-procedure | NAC | 1200mg IV 1 h pre-procedure, 1200mg orally for 2 days | | 100 mg standard dose | | | Mean S. Creat. ±SD:  0.83±0.15mg/dl | |
|  |  |  |  |  |  |  | Placebo | IV hydration only | |  |  |  | Mean S. Creat. ±SD:  0.81±0.17mg/dl | |
| Savaj 2014(149) | Inclusion Criteria   1. Diabetic patients  Exclusion Criteria   1. CHF 2. Complications during angiography | 1.Coronary Angiography | 102 | Increase in S. Creat. ≥0.3 mg/dL or >30% increase above baseline at 24h |  | All of the patients received 1L of normal saline before procedure | RIPC | Blood pressure cuff placed around their non-dominant upper arm. The cuff inflated to 200-mm Hg pressure for 5 minutes, followed by 5 minutes of deflation and repeated for 3 cycles in total, starting 15min pre-procedure | | Mean ±SD: 126.6±77.2 | | | Mean S. Creat. ±SD:  1.3±0.4mg/dl | |
|  |  |  |  |  |  |  | Control | no intervention | | Mean ±SD: 123.8±66.6 | | | Mean S. Creat. ±SD:  1.1±0.3mg/dl | |
| Sedighifard 2016(204) | Inclusion Criteria:  Patients with mild to moderate risk for CIN were included in the study. referred for elective coronary angiography  Exclusion Criteria:  1. Unstable angina  2. Myocardial infarction  3. Cardiac arrhythmias  4. Acute or chronic renal insufficiency/failure (estimated glomerular filtration rate [eGFR] <60 mL/min/1.73 m2)  5. Acute or decompensate heart failure  6. Diabetes  7. Intravascular administration of contrast material in the past month | 1.Elective coronary angiography | 153 | Increase in serum creatinine of ≥0.5 mg/dL or ≥25% of the baseline creatinine after 48 h of contrast material injection | Iodixanol  Iso-Osm | 0.9% sodium chloride (1 mL/kg/h) for 12 h, started 6 h before and continued to 6 h after the procedure | Silymarin | Single dose (280 mg) tablet 2 h before administration of the contrast material. | | Average, 45 mL | | | Mean S. Creat. ±SD:  0.85±0.16 | |
|  |  |  |  |  |  |  | Placebo | Single dose match placebo tablet 2 h before administration of the contrast material. | | Average, 45 mL | | | Mean S. Creat. ±SD:  0.94±0.15 | |
| Sekiguchi 2013(150) | Inclusion Criteria   1. Elective coronary angiography ± PCI  Exclusion Criteria   1. ACS 2. End-stage renal failure 3. Dialysis 4. Cardiogenic shock 5. CHF 6. COPD 7. Oxygen saturation levels<90% | 1.PCI | 359 | >25% increase above baseline S. Creat. 48h post-procedure |  | 0.9% normal saline at 1 ml/kg/h 12h pre-procedure until 12h post-procedure | Oxygen | Administration via nasal cannula; 2 l/min of oxygen from 10 min before the procedure to the end of the procedure | |  | | |  | |
|  |  |  |  |  |  |  | Room air |  | |  |  |  |  |  |
| Seyon 2007(151) | Inclusion Criteria   1. Age ≥ 18 years  2. ACS 3. Renal dysfunction  (S. Creat. ≥1.4 mg/dL in males or ≥1.3 mg/dL in females; ± Creat. clearance < 50mL/min 4. Informed consent 5. Available for follow-up  Exclusion Criteria   1. Hemodynamic instability requiring inotropic support 2. Pregnancy  3. Acute gastrointestinal disorder (unable to tolerate oral medication) 4. NYHA class III or IV CHF  5. Unsuitable to receive intravenous hydration therapy as per cardiologist  6. Known sensitivity to NAC 7. Current treatment with theophylline or mannitol 8. Dialysis therapy 9. Participation in another study or use of experimental drug | 1.Coronary Angiography | 43 | Increase in S. Creat. ≥0.5 mg/dL (44 µmol/L) above baseline within 48h | Iohexol Low-Osm Iodixanol Iso-Osm | 0.45% normal saline at 1 mL/kg/h 4-6h pre- and 12h post-procedure | NAC | 600 mg orally, for a total of 4 doses, with the first dose at 8:00 A.M. the day of the procedure and 3 doses post-procedure | | Mean ±SD: 147.5±74.75 | | |  | |
|  |  |  |  |  |  |  | Placebo | matched placebo | | Mean ±SD: 133.68±58.04 | | |  |  |
| Shehata 2014(152) | Inclusion Criteria   1. Diabetes Mellitus  2. Chronic stable angina 3. Mild-to-moderate renal dysfunction (mean eGFR 48±16 ml/min/ 1.73m2)  Exclusion Criteria   1. Severe CKD (eGFR <30 ml/min/1.73m2) 2. End-stage renal disease 3. Hemodialysis 4. Acute MI requiring emergency coronary intervention 5. Cardiogenic shock 6. History of ACS, PCI or CABG 7. CHD or myocardial disease other than ischemia 8. Limited life expectancy 9. Positive pre-procedural cTnI result 10. Previous treatment with Trimetazidine 11. Contraindications for aspirin, Clopidogrel, or Trimetazidine Use 12. Parkinson disease and other motion disorders | 1.Elective PCI | 108 | Increase in S. Creat. ≥0.5 mg/dL (44 µmol/L) or >25% increase above baseline within 48h | Low-Osm Ultravist . | 0.9% Isotonic saline solution at 1 ml/kg/h starting 12h pre-procedure and up to 24h. N-acetylcysteine (1,200 mg) 24h pre- and post-procedure. | Trimetazidine | 35 mg twice daily for 72 hours, starting 48 hours before PCI | | Mean ±SD: 270±10 | | | Mean S. Creat. ±SD:  2.0±0.5mg/dl | |
|  |  |  |  |  |  |  | Placebo | IV hydration only | | Mean ±SD: 280±15 | | | Mean S. Creat. ±SD:  2.0±0.4mg/dl | |
| Shehata 2015(153) | Inclusion Criteria   1. Diabetes Mellitus  2. Mild-to-moderate chronic kidney disease  Exclusion Criteria   1. Severe CKD (eGFR <30 mL/min/1.73m2) 2. End-stage renal disease (or patients on hemodialysis) 3. Intake of potentially nephrotoxic drugs 4. Acute MI requiring emergency coronary intervention 5. Cardiogenic shock 6. Prior history of ACS 7. Prior history of PCI or CABG 8. Congenital heart disease or any myocardial disease apart from ischemia 9. Known skeletal muscle disorder 10. Chronic liver disease 11. Limited life expectancy due to coexistent disease 12. Contraindications for aspirin and/or clopidogrel | 1.PCI | 148 | Increase in S. Creat. ≥0.5 mg/dL (44 µmol/L) or >25% increase above baseline within 48h | Low-Osm Iopromide, Non-ionic Ultravist 370/100 | 0.9% Isotonic saline at 1 mL/kg/h starting 12 h pre- and up to 24 h post-procedure. NAC (1200 mg) administered to both groups 24 h pre- and post-procedure | Statin | 80mg dose of Atorvastatin daily for 48h pre-procedure | | Mean ±SD: 274±8 | | | Mean GFR of 48.5±16 mL/min/1.73 m^2^ | |
|  |  |  |  |  |  |  | Placebo | IV hydration only | | Mean ±SD: 278±11 | | |  |  |
| Shyu 2002(154) | Inclusion Criteria   1. Chronic renal insufficiency (S. Creat.2.0-6.0mg/dl or creat. clearance 8-40 ml/min)  2. Stable S. Creat. (difference of ≤0.1 mg/dl compared with baseline at 12 to 24 h before procedure)  Exclusion Criteria   1. Acute MI requiring primary or rescue PCI  2. Use of vasopressors before the procedure 3. Cardiogenic shock 4. Current peritoneal dialysis or haemodialysis 5. Planned post-contrast dialysis 6. Allergies to the study medications | 1.Coronary Angiography | 138 | Increase in S. Creat. ≥0.5 mg/dL (44 µmol/L) above baseline within 48h | Iopamidol Low-Osm | 0.45% normal saline IV at 1 ml/kg/h for 12 h pre- and 12 h post-procedure. All patients were encouraged to drink if they were thirsty. | NAC | 400 mg twice daily orally, on the day pre- and day of procedure, for a total of two days | | Mean ±SD: 119±3 | | | Mean S. Creat. ±SD:  2.8±0.8mg/dl | |
|  |  |  |  |  |  |  | Placebo | matched placebo | | Mean ±SD: 115±48 | | | Mean S. Creat. ±SD:  2.8±0.8mg/dl | |
| Singh 2016(189) | Inclusion Criteria:  patients with diabetes scheduled for elective PCI with eGFR60 ml/min/1.73m2 or urinary albumin creatinine ratio of >300 mg/g  Exclusion Criteria:  1. Patients with ST-elevation MI or decompensated heart failure in the preceding 6months  3. End stage renal disease on maintenance dialysis  4. Cerebrovascular disease  5. Chronic liver disease  6. chronic obstructive pulmonary disease  7. Gastrointestinal bleeding  8. Acute or chronic infection  9 Malignancy | 1.Elective PCI | 116 | Serum creatinine rise of ≥0.5mg/dl from baseline and/or an increase in creatinine of ≥25% from baseline within 48 hours after contrast exposure | Visipaque  Iso-Osm | Intravenous 0.9%NaCl infusion at 60ml/hour 6 hours before and after PCI procedure | RIPC | Right upper arm cuff manually inflated to  200mmHg for 5minutes, followed by deflation for 5minutes to allow reperfusion and this Cycle was performed 3 times 30minutes before the PCI. | | Mean+SD:  197.5 ± 114.3 | | | S. Creat.  Mean+SD:  1.42 ± 0.58 | |
|  |  |  |  |  |  |  | Placebo | Sham cuff applied for 30minutes with no inflation | | Mean+SD:  196.3 ± 118.8 | | | S. Creat.  Mean+SD:  1.41 ± 0.34 | |
| Solomon 1994(155) | Inclusion Criteria   1. S. Creat. > 1.6 mg/dL (140 μmol/L) or Creat. clearance < 60ml/min | Coronary Angiography | 63 | Increase in S. Creat. ≥0.5 mg/dL (44 µmol/L) above baseline within 48h | The choice of radiocontrast agent was made by the cardiologist | 0.45% normal saline IV at 1 ml/kg/h for 12 h pre- and 12 h post-procedure. All patients were encouraged to drink if they were thirsty. | Mannitol | 25g intravenously during the 60 minutes immediately before angiography | | Mean ±SD: 130±56 | | | Mean S. Creat. ±SD:  2.1±0.64mg/dl | |
|  |  |  |  |  |  |  | Furosemide | 80 mg IV during 30 min pre-procedure | | Mean ±SD: 132±48 | | | Mean S. Creat. ±SD:  2.1±0.59mg/dl | |
|  |  |  |  |  |  |  | Control | IV hydration only | | Mean ±SD: 125±45 | | | Mean S. Creat. ±SD:  2.1±0.54mg/dl | |
| Solomon 2015(205) | Inclusion Criteria:  Patients > 18 years scheduled for elective coronary or peripheral angiography, and if their calculated  eGFR was ,45 ml/min per 1.73 m2  Exclusion Criteria:  1. Hemodynamic  2. Instability per investigator judgment  3. Renal replacement thereby  4. Hypocalcaemia | Elective Coronary or peripheral angiography | 412 | ≥ 0.5mg/dl or 25%rise in creatinine from baseline during the first 3 days. | No details (Multicentre study) | Infusion rate in all groups were 5 ml/kg 60 minutes before and 1.5 ml/kg per h during and for 4 hours after angiography. In patients with a low ejection fraction (,40%), history of congestive heart failure, or significant oedema, the same total dose of study fluid could be infused over 5 hours post-angiography at the discretion of the investigator | NaHco3 | 50 ml ampules of 1 m sodium bicarbonate (154 mEq/L) and a partially filled 1 l bag with 830 ml sterile water | | Mean+SD:  110±66 | | | Mean S. Creat. ±SD:  1.98±0.62 | |
|  |  |  |  |  |  |  | Nacl | Standard 0.9% sodium chloride | | Mean+SD:  104±72 | | | Mean S. Creat. ±SD:  1.85±0.49 | |
| Spargias  2004(7) | Inclusion Criteria   1. Non-emergent coronary angiography  2. S. Creat. ≥1.2 mg/dL (106µmol/L) within 3 months of the planned procedure  Exclusion Criteria   1. Known acute renal failure 2. End-stage renal disease requiring dialysis 3. Intravascular administration of contrast medium within the previous 6 days 4. Anticipated re-administration of contrast medium within the following 6 days 5. Use of vitamin C supplements on a daily basis during the week before the procedure 6. Inability to administer the study medication at least 2h pre-procedure. | 1.Coronary Angiography  2.PCI | 265 | Increase in S. Creat. ≥0.5 mg/dL (44 µmol/L) or >25% increase above baseline within 2-5 days of contrast | The choice of the type of contrast agent was left to the interventional cardiologist performing the procedure, but use of a non-ionic, low- or iso-osmolar contrast agent was encouraged. | 50-125 mL/h normal saline IV from randomization until at least 6h post-procedure. All patients were encouraged to drink if they were thirsty | Ascorbic Acid | 3g chewable tablets at least 2 hours pre-procedure, followed by 2g the night and the morning post-procedure | | Mean ±SD: 287±148 | | | Mean S. Creat. ±SD:  1.52±0.64mg/dl | |
|  |  |  |  |  |  |  | Placebo | 3g chewable tablets at least 2 hours pre-procedure, followed by 2g the night and the morning post-procedure | | Mean ±SD: 261±128 | | | Mean S. Creat. ±SD:  1.50±0.54mg/dl | |
| Spargias  2006(157) | Inclusion Criteria   1. S. Creat. ≥ 1.4 mg/dL within 1 month  2. Elective procedure  Exclusion Criteria   1. Circulatory shock for any reason 2. SBP <95 mmHg 3. Acute renal failure 4. End-stage renal disease requiring dialysis 5. IV administration of contrast medium within preceding 10 days or anticipated re-administration of contrast medium within the following 6 days 6. Inability to administer study medication at least 30 min pre-procedure 6. Primary intervention for acute STEMI | 1.Coronary Angiography  2.PCI | 33 | Increase in S. Creat. ≥0.5 mg/dL (44 µmol/L) or >25% increase above baseline within 2 days of contrast | The choice of the type of contrast agent was left to the interventional cardiologist performing the procedure, but use of a non-ionic, low- or iso-osmolar contrast agent was encouraged. | 1.5 mL/kg/h normal saline for ≥ 4h pre-procedure and continued for at least 12h post-procedure | Iloprost | 1ng/kg/min beginning 30-90 min prior to and ending 4h post-procedure | | Mean ±SD: 217±118 | | | Mean S. Creat. ±SD:  1.71±0.7mg/dl | |
|  |  |  |  |  |  |  | Iloprost | 2ng/kg/min beginning 30-90 min prior to and ending 4h post-procedure | | excluded from analysis | | | | |
|  |  |  |  |  |  |  | Placebo | Normal saline beginning 30-90 min prior to and ending 4h post-procedure | |  | | | Mean S. Creat. ±SD:  1.66±0.64mg/dl | |
| Spargias  2009(156) | Inclusion Criteria   1. S. Creat. ≥1.4 mg/dL within 1 month before the planned procedure   Exclusion Criteria   1. Circulatory shock for any reason 2. SBP <95 mmHg 3. Acute renal failure 4. End-stage renal disease requiring dialysis 5. Intravascular administration of contrast medium within the previous 10 days or anticipated re-administration of contrast medium within the following 6 days 6. Inability to administer study medication at least 30 minutes before the procedure 7. Primary intervention for acute STEMI | 1.Coronary Angiography  2.PCI | 239 | Increase in S. Creat. ≥0.5 mg/dL (44 µmol/L) or >25% increase above baseline within 2 days of contrast | Use of a non-ionic, iso-osmolar, or low-osmolar contrast medium was encouraged. | 1.5 mL/kg/h normal saline for ≥ 4h pre-procedure and continued for at least 12h post-procedure | Iloprost | 1ng/kg/min beginning 30-90 min prior to and ending 4h post-procedure | | Mean ±SD: 257±129 | | | Mean S. Creat. ±SD:  1.63±0.49mg/dl | |
|  |  |  |  |  |  |  | Placebo | Normal saline beginning 30-90 min prior to and ending 4h post-procedure | | Mean ±SD: 249±137 | | | Mean S. Creat. ±SD:  1.59±0.49mg/dl | |
| Stone 2003(158) | Inclusion Criteria   1. Age > 18years 2. Risk for developing contrast-induced nephropathy (creat. clearance <60mL/min (1.00 mL/s) 3. Diagnostic ± interventional cardiology procedures.  Exclusion Criteria   1. Known severe allergy to contrast media or fenoldopam or its infusion components 2. Acute renal failure or unstable renal function 3. SBP < 100mmHg 4. Acute MI 5. Decompensated heart or respiratory failure 6. Contraindication to dopaminergic agents 7. Current use of mannitol or dopamine 8. Planned addition, discontinuation, or dose adjustment of trimethoprim, cimetidine, metoclopramide, bromocriptine, levodopa, nonsteroidal anti-inflammatory drugs, or catechol-O-methyltransferase inhibitors during the study 9. Exposure to iodinated contrast within the previous 10 days 10. Other serious medical conditions likely to interfere with data collection or follow-up 11. Participation in other investigational protocols within 30 days | 1.Diagnostic ± interventional cardiology procedures | 373 | Increase in serum creatinine level of ≥ 25% from baseline to the maximum value obtained at 24-96h | Low-Osm | 0.45% normal saline infusions at 1.5 mL/kg/h (or 1.0 mL/kg/h if heart failure was present) for 2-12h prior to trial | Fenoldopam | 0.05 µg/kg/min and increased in 20 minutes to 0.10 µg/kg/min if tolerated. The infusion was then maintained during angiography and percutaneous intervention and continued for 12 hours | | Mean ±SD: 153±107 | | | Mean S. Creat. ±SD:  1.82±0.71mg/dl | |
|  |  |  |  |  |  |  | Placebo | matched placebo | | Mean ±SD: 162±110 | | | Mean S. Creat. ±SD:  1.81±0.83mg/dl | |
| Stone 2011(159) | Inclusion Criteria  1. Age >18 years  2. CKD (Creat. clearance 20-50 ml/min) 3. Elective coronary arteriography (± PCI) 4. Use of iodinated contrast ≥50ml  Exclusion Criteria   1. Acute renal failure 2. Unstable renal function and current or planned dialysis 3. Renal artery angiography, renal drug infusion, or known renal artery stenosis 4. Use of mannitol or intravenous diuretics 5. Decompensated heart failure 6. Respiratory failure 7. Hypotension 8. Acute or recent STEMI 9. Allergy to Contrast Heparin, meperidine, or buspirone that could not be adequately premedicated 10. Recent monoamine oxidase inhibitor 11. Use of additional contrast administration 10 days before or after the procedure 12. Inferior vena cava filter 13. Height <1.5 m 14. Hypersensitivity to hypothermia (Raynaud) 15. Bleeding diathesis, coagulopathy or sickle cell disease 16. Hepatic impairment 17. Cryoglobulinemia 18. Untreated hypothyroidism 19. Addison disease 20. Prostatic hypertrophy or urethral stricture 21. Would refuse blood transfusions 22. Pregnancy 23. Inability or unwillingness to sign informed consent 24. Enrolment in another investigational drug or device trial 25. Condition possibly leading to noncompliance with any study procedures. | 1.Angiography  2.PCI  3.Renal Angiography | 157 | >25% relative increase above baseline within 96h of contrast, or increase in S. Creat. ≥0.5 mg/dL (44 µmol/L) | Iodixanol Low-Osm Other low osmolar contrast agents were permitted according to the operator’s choice. | 0.9% saline at 1.5 ml/kg/hour (1.0ml/kg/hour of 0.45% normal saline for patients with CHF or left ventricular dysfunction).  1h before the procedure NaCl was replaced with NaHCO3 (150 mEq in 1L D5W) at 0.45 mEq/kg (3ml/kg/hour) for the first hour and then 0.15 mEq/kg/hour (1 ml/kg/hour) for 5-7 hours after the procedure | Hypothermia | Reprieve endovascular temperature therapy system used to achieve a central temperature of 33°C-34°C before first contrast injection and for 3 hours after the procedure | | Mean ±SD: 150±94.3 | | | Mean S. Creat. ±SD:  12.7±3.0mg/dl | |
|  |  |  |  |  |  |  | Control | Normo-thermia. IV hydration only | | Mean ±SD: 138±78.3 | | | Mean S. Creat. ±SD:  12.1±2.2mg/dl | |
| Sun, C 2015(206) | Inclusion Criteria:  Patients > 18 years referred for coronary angiography and / or PCI  Exclusion Criteria:  1. Acute myocardial infarction requiring emergency catheterization  2. Chronic heart failure  3. Previous PCI or coronary artery bypass grafts  4. Contrast media within 7 days  5. Acute respiratory insufficiency  6. Acute renal failure  7. End-stage renal disease requiring dialysis  8. Patients on metformin, nonsteroidal anti-inflammatory drugs, diuretics, mannitol, dopamine, theophylline, ascorbic acid and N-acetylcysteine during the study period. | 1.Coronary angiography ± PCI | 141 | acute decline in renal function characterized by an absolute rise of 0.5 mg/dL (44.2 µmol/L) in SCr or a >25% increase from baseline to 48 hours after angiography | Iodixanol  Iso-Osm  Iopromide  Low-Osm | See Groups | BNP | 1.5 μg/kg bolus followed by an adjusted dose infusion of 0.01 μg/kg/ min | | Mean+SD:  145.56±75.86 | | | Mean S. Creat. ±SD:  75.46±17.25 | |
|  |  |  |  |  |  |  | I.V Hydration | isotonic saline (0.9%) at 6 hours before and 12 hours after the procedure | | Mean+SD:  149.36±73.28 | | | Mean S. Creat. ±SD:  75.10±14.59 | |
| Tamura 2009(160) | Inclusion Criteria   1. Patients with mild renal insufficiency (S. Creat. 1.1-2.0 mg/dl)  2. Elective coronary procedure   Exclusion Criteria   1. Allergy to contrast medium 2. Pregnancy 3. History of dialysis 4. Exposure to contrast medium within the preceding 48 hours of the study 5. ACS within the preceding 1 month of the study 6. NYHA class IV CHF 7.LVEF<25% 8. Severe chronic respiratory disease 9. Single functioning kidney 10. Administration of N-acetylcysteine, theophylline, dopamine or mannitol | 1.Diagnostic ± interventional cardiology procedures | 154 | Increase in S. Creat. ≥0.5 mg/dL (44 µmol/L) or >25% increase above baseline within 3 days of contrast | Iohexol Low-Osm | 0.9% Isotonic saline at 1ml/kg/h (0.5 ml/kg/h for patients with LVEF <40%) for 12h pre- and 12h post-procedure | NaHco3 Group: | Standard hydration with NaCl plus single-bolus IV administration of NaHCO3 [20 ml = 20 mEq], 5 minutes pre-procedure  CV/mL Mean(SD): 82.1± 40.4 | | Mean ±SD: 82.1±40.4 | | | Mean S. Creat. ±SD:  1.36±0.18mg/dl | |
|  |  |  |  |  |  |  | Control | IV hydration only | | Mean ±SD: 87.8±44.9 | | | Mean S. Creat. ±SD:  1.38±0.19mg/dl | |
| Tanaka 2011(161) | Inclusion Criteria   1. Primary Angioplasty for Acute MI  Exclusion Criteria   1. Dialysis 2. Known allergy to NAC 3. Inability to take NAC orally | 1.PCI | 83 | >25% relative increase above baseline within 72h of contrast | Iopamidol Low-Osm | Intravenous Ringer’s lactate solution at 1-2 mL/kg/h for >12h post-procedure | NAC | 705 mg orally before and at 12, 24, and 36h post-procedure (to a total of 2,820 mg) | | Mean ±SD: 205±80 | | | Mean S. Creat. ±SD:  0.88±0.25mg/dl | |
|  |  |  |  |  |  |  | Placebo | 4ml water | | Mean ±SD: 216±85 | | | Mean S. Creat. ±SD:  0.80±0.19mg/dl | |
| Tasanarong 2009(162) | Inclusion Criteria   1. S. Creat. ≥1.2 mg/dL or Creat. clearance ≤60mL/min  Exclusion Criteria   1. Renal Failure 2. End stage Renal disease requiring dialysis 3. Unstable renal function 4. Known allergy to contrast agents 5. Receiving mechanical ventilation 6. Congestive heart failure 7. Cardiogenic shock 8. Emergency angiography 9. Receiving NAC, mannitol, diuretics, theophylline, dopamine, ascorbic acid or contrast agents within 14 days before study 10. Use of α-or γ-tocopherol supplements on a daily basis during the week prior to the study. | 1.Coronary Angiography  2.PCI | 118 | Increase in S. Creat. ≥0.5 mg/dL (44 µmol/L) or >25% increase above baseline within 48h |  | 0.9% Isotonic saline at 1ml/kg/h for 12h pre- and 12h post-procedure | α-tocopherol | 525 IU daily commening 48h pre-procedure for total 3 doses. | | Mean ±SD: 150±83 | | | Mean S. Creat. ±SD:  1.62±0.44mg/dl | |
|  |  |  |  |  |  |  | Placebo | matched placebo | | Mean±SD: 132±58 | | | Mean S. Creat. ±SD:  1.67±0.53mg/dl | |
| Tasanarong 2013(163) | Inclusion Criteria  1. Chronic kidney disease   Exclusion Criteria  1. Acute Kidney Injury  2. CKD Stage 5 3. Unstable renal function 4. Known allergy to contrast agents 5. Receiving mechanical ventilation 6. Congestive heart failure 7. Cardiogenic shock 8. Emergency angiography 9. Receiving NAC, mannitol, diuretics, theophylline, dopamine, ascorbic acid or contrast agents within 14 days before study 10. Use of α-or γ-tocopherol supplements on a daily basis during the week prior to the study. | 1.PCI | 331 | Increase in S. Creat. ≥0.5 mg/dL (44 µmol/L) or >25% increase above baseline within 48h |  | 0.9% Isotonic saline at 1ml/kg/h for 12h pre- and 12h post-procedure | α-tocopherol | 350 mg/day 5 Days before and 2 days after procedure | | Mean±SD: 134±73 | | | Mean S. Creat. ±SD:  1.58±0.48mg/dl | |
|  |  |  |  |  |  |  | γ-tocopherol | 300 mg/day 5 Days before and 2 days after procedure | | Mean±SD: 137±75 | | | Mean S. Creat. ±SD:  1.48±0.48mg/dl | |
|  |  |  |  |  |  |  | Placebo | 350 mg/day 5 Days before and 2 days after procedure | | Mean±SD: 134±69 | | | Mean S. Creat. ±SD:  1.63±0.53mg/dl | |
| Tepel 2000(164) | Inclusion Criteria  1. Elective computed tomography (CT)  2. Chronic renal insufficiency (S. Creat. >1.2 mg/dL (106 µmol/L) or creat. clearance <50 ml/min)  Exclusion Criteria  1. Acute renal failure | 1.CT | 93 | Increase in S. Creat. ≥0.5 mg/dL (44 µmol/L) above baseline within 48h |  | 0.45% saline at 1ml/kg/h for 12h pre- and 12h post-procedure. Patients were encouraged to drink if thirsty. | NAC | 600 mg orally twice daily on the day prior to and day of CT | |  | | | Mean S. Creat. ±SD:  2.5±1.3mg/dl | |
|  |  |  |  |  |  |  | Control | IV hydration only | |  | | | Mean S. Creat. ±SD:  2.4±1.3mg/dl | |
| Thiele 2010(165) | Inclusion Criteria   1. Patients with ST-elevation undergoing primary angioplasty   Exclusion Criteria  1. Previous fibrinolysis <12h 2. Known NAC allergy 3. Chronic dialysis 4. Pregnancy 5. Contra- indications to magnetic resonance imaging (MRI) | 1.Coronary Angioplasty | 292 | >25% relative increase above baseline within 72h of contrast | Iopromide Low-Osm | 0.9% Isotonic saline at 1ml/kg/h (0.5 ml/kg/h for patients in overt heart failure) for 12h | NAC | Intravenous bolus of 1,200 mg before angioplasty and 1,200 mg intravenously twice daily for 48 h after (total dose 6,000 mg) | | Median (Range): 180 (140-230) | | | Median S. Creat. (Range):  81 (69-97) mmol/l | |
|  |  |  |  |  |  |  | Placebo | matched times - 10 ml of 0.9% NaCl at each injection | | Median (Range): 160 (120-220) | | | Median S. Creat. (Range):  78 (67-90) mmol/l | |
| Toso 2010(166) | Inclusion Criteria   1. Chronic kidney disease (estimated Creat. Clearance <60 ml/min) 2. Elective coronary angiography and/or other intervention  Exclusion Criteria   1. Current treatment with a statin 2. Contraindication to statin treatment 3. Previous contrast media administration within 10days of study entry 4. End-stage renal failure requiring dialysis 5. Informed refusal of consent | 1.Coronary Angiography | 353 | Increase in S. Creat. ≥0.5 mg/dL (44 µmol/L) or >25% increase above baseline within 5 days | Iodixanol Iso-Osm | 0.9% Isotonic saline at 1ml/kg/h (0.5 ml/kg/h for patients in overt heart failure) for 12h. Oral NAC (1200mg) twice daily from the day before to the day after the procedure | Statin | 80mg Atorvastatin daily starting 48h pre-procedure, continued for 48h post-procedure | | Mean±SD: 164±99 | | | Mean S. Creat. ±SD:  1.2±0.35mg/dl | |
|  |  |  |  |  |  |  | Placebo | matched placebo starting 48h pre-procedure, continued for 48h post-procedure | | Mean±SD: 151±95 | | | Mean S. Creat. ±SD:  1.18±0.33mg/dl | |
| Traub 2013(167) | Inclusion Criteria   1. Patients undergoing chest, abdominal, or pelvic CE-CT 2. Age > 18 years 3. ≥ one risk factor for CI-AKI  Exclusion Criteria   1. Unable or unwilling to provide written informed consent 2. End-stage renal disease 3. Pregnancy 4. Allergy to NAC  5. Clinical instability | 1.CE-CT | 383 | Increase in S. Creat. ≥0.5 mg/dL (44 µmol/L) or >25% increase above baseline within 48-72h of procedure | Low-Osm Isovue Low-Osm Optiray Iso_osm Visipaque | as per intervention protocol | NAC | 3g in 500mL NaCl as an IV bolus and then 200mg/h as 67mL/h for up to 24 hours | | Mean: 113.11 | | | Mean S. Creat. ±SD:  1.00±0.28mg/dl | |
|  |  |  |  |  |  |  | Placebo | 500mL NaCl as an IV bolus and then 200mg/h as 67mL/h for up to 24 hours | | Mean: 115.24 | | | Mean S. Creat. ±SD:  0.99±0.27mg/dl | |
| Trivedi 2003(168) | Inclusion Criteria  1. Age > 18 years  2. Elective Coronary Angiography  Exclusion Criteria   1. Creat. clearance < 20ml/min 2. Clinically decompensated heart failure 3. Decrease effective arterial volume (e.g. Nephrotic syndrome, liver cirrhosis) 4. Unwilling to participate | 1.Coronary Angiography | 63 | Increase in S. Creat. ≥0.5 mg/dL (44 µmol/L) above baseline within 48h of procedure |  | as per intervention protocol | IV fluid | 0.9% normal saline at 1ml/kg/h for 24h pre-procedure | |  | | | Mean S. Creat. ±SD:  101±21mmol/l | |
|  |  |  |  |  |  |  | Oral fluid | unrestricted oral fluid | |  | | | Mean S. Creat. ±SD:  112±33mmol/l | |
| Tumlin 2002(169) | Inclusion Criteria   1. Age ≥ 18 years  2. Moderate-severe renal failure (S. Creat. 2.0-5.0 mg/dL)   Exclusion Criteria   1. Uncontrolled ventricular arrhythmia 2. Chronic  hemodialysis or peritoneal dialysis 3. Metformin therapy 4. Acute cerebral vascular accidents 5. Oxygen saturation <90% on room air or possible overt pulmonary edema 6. Known glaucoma 7. Unstable clinical condition that would not allow for 3 hours of pre-hydration 8. S. Creat. <2.0mg/dL or >5.0 mg/dL 9. Known hypersensitivity to fenoldopam mesylate 10. Informed consent not possible 11. Patients who received prophylactic furosemide, mannitol, or renal dose dopamine before randomization 12. Pregnancy or lactation | 1.Coronary Aniography  2.Peripheral Angiography | 59 | Increase in S. Creat. ≥0.5 mg/dL (44 µmol/L) or >25% increase above baseline within 48 hours of contrast | Low-Osm Iso-Osm | Pre-hydration with 1/3 normal saline at 100 mL/h for 3h pre-procedure | Fenoldopam | 0.1 µg/kg/min starting 1h pre-procedure and continued for 4 hours with 1/3 normal saline at 100ml/h | | Mean±SEM: 80±15 | | | Mean S. Creat. ±SEM:  13±56mg/dl | |
|  |  |  |  |  |  |  | Control | normal saline only at same time and schedule | | Mean±SEM: 96±19 | | | Mean S. Creat. ±SEM:  11±50mg/dl | |
| Vasheghani-Farahani 2009(171) | Inclusion Criteria   1. Elective coronary angiography 2. S. Creat. ≥1.5 mg/dL  Exclusion Criteria   1. Unstable serum creatinine 2. Previous history of dialysis 3. eGFR <20ml/min 4. Emergency catheterization 5. Recent exposure to radiocontrast agent within 2 days prior to study  6. Allergy to contrast agent 7. Pregnancy 8. Administration of Dopamine, Fenoldopam or NAC during the intended time of the study 9. Need for continuous hydration | 1.Coronary Angiography | 281 | Increase in S. Creat. ≥0.5 mg/dL (44 µmol/L) or >25% increase above baseline within 48 hours of contrast | Iohexol Low-Osm | 3ml/kg for 1 hour immediately before contrast injection followed by infusion of 1ml/kg/h for 6h post-procedure | NaCl | 1075 ml of 0.45% Normal Saline at specified rate | | Mean±SD: 125±96.2 | | | Mean S. Creat. ±SD:  1.63±0.32mg/dl | |
|  |  |  |  |  |  |  | NaHCO3 | 75ml of 8.4% NaHCO3 added to 1L of 0.45% Normal Saline | | Mean±SD: 129±96.3 | | | Mean S. Creat. ±SD:  1.66±0.50mg/dl | |
| Vasheghani-Farahani 2010(170) | Inclusion Criteria   1. Elective coronary angiography 2. S. Creat. ≥1.5 mg/dL 3. Uncontrolled hypertension 4. Compensated severe heart failure 5. History of pulmonary oedema  Exclusion Criteria   1. Unstable serum creatinine 2. Previous history of dialysis 3. eGFR <20ml/min 4. Emergency catheterization 5. Recent exposure to radiocontrast agent within 2 days prior to study  6. Allergy to contrast agent 7. Pregnancy 8. Administration of Dopamine, Fenoldopam or NAC during the intended time of the study 9. Need for continuous hydration | 1.Coronary Angiography | 76 | Increase in S. Creat. ≥0.5 mg/dL (44 µmol/L) or >25% increase above baseline within 48 hours of contrast | Iohexol Low-Osm | 3ml/kg for 1 hour immediately before contrast injection followed by infusion of 1ml/kg/h for 6h post-procedure | NaCl | 1075 ml of 0.45% Normal Saline at specified rate | | Mean±SD: 123±31 | | | Mean S. Creat. ±SD:  1.77±0.52mg/dl | |
|  |  |  |  |  |  |  | NaHCO3 | 75ml of 8.4% NaHCO3 added to 1L of 0.45% Normal Saline | | Mean±SD: 112±33 | | | Mean S. Creat. ±SD:  1.71±0.45mg/dl | |
| Vogt 2001(172) | Inclusion Criteria   1. Chronic stable renal insufficiency 2. S. Creat. >200 µm/L (>2.3 mg/dL) | 1.Renal angioplasty  2.Peripheral angioplasty  3.CE-CT  4.Coronary angiography | 143 | Increase in S. Creat. ≥0.5 mg/dL (44 µmol/L) or >25% increase above baseline within 6 days | Low-Osm | I.V. saline at 1ml/kg/h for 12h pre-procedure | Dialysis | Haemodialysis starting as soon as possible after radiographic investigation | | Mean±SD: 210±143 | | | Mean S. Creat. ±SD:  308±106mmol/l | |
|  |  |  |  |  |  |  | Control | I.V. saline at 1ml/kg/h for 12h pre-procedure | | Mean±SD: 143±115 | | | Mean S. Creat. ±SD:  316±112mmol/l | |
| Wang, C 2015(207) | Inclusion Criteria:  18–80 years old undergoing non-emergency coronary angiography and/or intervention  Exclusion Criteria:  1. Serum creatinine ≥8 mg/dl(707mmol/L)  2. Known acute kidney injury, current peritoneal or haemodialysis  3. Acute myocardial infraction or cardiogenic shock  4. Hypersensitivity to iodine-containing compounds  5. Intravascular administration of contrast medium within the previous 1 week  6. Use of antioxidant on a daily basis during the week before the procedure  7. Pregnancy or lactation  8. Malignancy | 1.Non –emergency Coronary angiography | 866 | Absolute increase of serum creatinine concentration of at least 0.5mg/dl (44.2mmol/l) or a relative increase of at least 25% from baseline within 48 hours after the procedure. | Iopamidol  Iso-Osm | 1 ml/ kg body weight per hour intravenous normal saline 500 ml was administered in all patients at the beginning of the procedure | Glutathione | 1.8 g reduced glutathione in 500 ml of normal saline | | Mean+SD:  135.6±70.1 | | | Serum Cr (mmol/L):  74(64,84) | |
|  |  |  |  |  |  |  | Placebo | Saline | | Mean+SD:  132.8±66.7 | | | Serum Cr (mmol/L):  75 (63.3,84.0) | |
| Wang 2000(173) | Inclusion Criteria   1. Adult patients undergoing cardiac angiography 2. S. Creat. ≥2.0 mg/dL (176.8 µmol/L) within 48h of procedure  Exclusion Criteria   1. SBP <100/70 mm Hg or heart rate >100 bpm at baseline 2. Acute renal failure 3. Chronic renal failure requiring dialysis 4. Inability to adhere to the hydration regimen 5. Diuretic therapy within 12h or during infusion of study drug 6. Dopamine therapy within six hours or during infusion of study drug 7. Administration of NSAIDs other than aspirin (≤325 mg/day) within 24h of study drug 8. Uncontrolled cardiac arrhythmia 9. Hepatic dysfunction 10. Cerebrovascular accident within 1 week 11. Women of child-bearing potential | 1.Coronary Angiography | 225 | Increase in S. Creat. ≥0.5 mg/dL (44 µmol/L) or >25% increase above baseline within 48 hours of contrast | Low-Osm Type determined by consultant | 0.45% saline at 1ml/kg/h beginning 2-12h pre- and continuing for ≥ 12h post-procedure. Patients were encouraged to drink if thirsty. | ERAs Group: Endothelin A and B receptor antagonist (SB 290670) | 100 µg/kg over 10min, then 1.0µg/kg/min starting 30-150min pre-contrast, and continued for 12 hours after CV/mL Mean±SD: 104.0 64.8 | | Mean±SD: 104±64.8 | | | Mean S. Creat. ±SD:  2.7±0.9mg/dl | |
|  |  |  |  |  |  |  | Placebo | matched placebo | | Mean±SD: 122.4±86.2 | | | Mean S. Creat. ±SD:  2.8±1.1mg/dl | |
| Wang 2011(174) | Inclusion Criteria   1. Patients with ST-Segment elevation undergoing primary PCI  Exclusion Criteria   1. Cardiogenic shock 2. Heart rate > 100bpm on admission  3. End stage renal disease requiring dialysis  4. Not agree to consent 5. Known allergic condition 6. Candidate not suitable to use anisodamine | 1.PCI | 143 | Increase in S. Creat. ≥0.5 mg/dL (44 µmol/L) or >25% increase above baseline within 48-72 hours of contrast | Ultravist Low-Osm | Normal saline at 1ml/kg/h pre-procedure and for 24h after | Anisodamine | 50 µg/kg bolus dose followed by adjusted dose (0.1-0.2 µg/kg/min) for 24h after | | Mean±SD: 256.8±71.9 | | | Mean S. Creat. ±SD:  83.1±17.7mmol/l | |
|  |  |  |  |  |  |  | Placebo | matched placebo | | Mean±SD: 259.8±79.1 | | | Mean S. Creat. ±SD:  82.2±14.1mmol/l | |
| Webb 2004(175) | Inclusion Criteria   1. Renal dysfunction (eGFR < 50mL/min)  2. Cardiac catheterization  Exclusion Criteria   1. Suspected acute renal failure 2. S. Creat. >400µmol/L 3. Current dialysis 4. Hemodynamic instability 5. NAC administration within 48 hours 6. Age <18 years 7. Lack of informed consent 8. Inability to comply with follow-up 9. Recent creatinine elevation after a diagnostic angiogram | 1.Coronary Angiography  2.PCI | 496 | >25% relative increase above baseline S. Creat. | Ioversol Low-Osm | 200ml isotonic saline pre-procedure, then at 1.5 mL/kg/h for 6h, unless contraindicated | NAC | 500 mg I.V. immediately pre-procedure | | Median (IQR): 120 (80-186) | | | Median (IQR): 141 (125-166) mmol/l | |
|  |  |  |  |  |  |  | Placebo | IV hydration only | | Median (IQR): 120 (80-155) | | | Median (IQR): 142 (124-167) mmol/l | |
| Weisberg 1993(176) | Inclusion Criteria   1. Stable S. Creat. ≥1.8mg/L  2. Elective cardiac catheterization  Exclusion Criteria   1. NYHA class IV CHF 2. Haemodynamic instability 3. Allergy to radiocontrast agent or prior exposure within the last 7days 4. Pregnancy | 1.Cardiac Catheterization | 41 | >25% relative increase above baseline S. Creat. | MD 76 Hi-Osm | as per intervention protocol | Dopamine | 2µ/kg/min in 0.45% Nacl | |  | | | Mean S. Creat. ±SD:  2.37±0.17mg/dl | |
|  |  |  |  |  |  |  | Control | 0.45% normal saline 100ml/h starting 12h pre-procedure, continued throughout procedure and for total of 120 min | |  | | | Mean S. Creat. ±SD:  2.66±0.15mg/dl | |
| Wrobel 2010(177) | Inclusion Criteria   1. Patients with cardiovascular disease 2. Diabetes Mellitus   Exclusion Criteria   1. Contraindications for invasive procedures 2. Pregnancy 3. Breast-feeding 4. Symptoms and signs of infection 5. Antibiotic treatment 6. Participation in other studies within the preceding 30 days 7. History of hypersensitivity to contrast agents 8. Cancer  9. Acute renal failure of alternative aetiology | 1.Coronary Angiography  2.PCI | 107 | Increase in S. Creat. ≥0.5 mg/dL (44 µmol/L) or >25% increase above baseline | Loversol Low-Osm | as per intervention protocol | IV fluid | Intravenous infusion of isotonic 0.9% NaCl at 1 mL/kg/h started 6h pre-procedure, continued up to 12h post-procedure | | Mean±SD: 101.1±36.62 | | | Mean S. Creat. ±SD:  1.235±0.4454mg/dl | |
|  |  |  |  |  |  |  | Oral fluid | Weight-adjusted quantity of neutral fluid (commercially available still mineral water or boiled water) administered at 1mL/kg/h between 12-6 hours pre-procedure, continued up to 12h post-procedure | | Mean±SD: 110.4±65.28 | | | Mean S. Creat. ±SD:  1.172±0.3872mg/dl | |
| Xu, R. H 2013(208) | Inclusion Criteria:  Patients (aged ≥60 years) with coronary artery disease who were admitted for PCI  Exclusion Criteria:  1. Refusal to participate  2. Refusal of PCI  3. Use of any nephrotoxic drugs during the perioperative period  4. Severe hepatic and renal failure  5. Serious infectious disease  6. New York Heart Association Functional Classification (NYHA) >3  7. Hemodynamic instability (including systolic blood pressure <90 mmHg  8. Coronary lesions below the threshold for clinical revascularization therapy  9. Coronary lesions not suitable for PCI due to coronary anatomy  10 Allergic reaction to contrast media and alprostadil | 1.PCI | 67 | Relative increase of >25% or an absolute increase of ≥0.5 mg/dl in SCr from the baseline value 72 h after exposure to the contrast medium | Iopromide  Low-Iso | 1 ml/kg/h normal saline for 6 h prior to PCI and 12 h following PCI | Alprostadi | | 10 µg (diluted with 100 ml normal saline) twice a day by intravenous drip for the 3 days following PCI. | Mean+SD:  133.71±32.46 | | Mean S. Creat. ±SD:  83.63±23.59 | | |
|  |  |  |  |  |  |  | Control | | I.V hydration Only | Mean+SD:  123.57±37.14 | | Mean S. Creat. ±SD:  76.82±19.45 | | |
|  |  |  |  |  |  |  | The original control Group in this study were excluded | | No intervention | Mean+SD:  134.09±36.99 | | Mean S. Creat. ±SD:  88.27±27.40 | | |
| Xu 2014(178) | Inclusion Criteria   1. Diabetes Mellitus  2. Age ≥65 years  Exclusion Criteria   1. Previous use of trimatezidine, nicorandil or glibenclamide 2. Uncontrolled Diabetes Mellitus  3. Elevated cardiac biomarker at admission 4. MI within 6 months, stent implantation or CABG within 6 months 5. Cardiogenic shock 6. LVEF <50% 7. Congenital or valvular heart disease requiring further surgery 8. Moderate or severe renal insufficiency with eGFR <30 mL/min 9. Ongoing bleeding, or a history of bleeding diathesis 10. Expected life span <12months | 1.Coronary Angiography | 207 | >25% relative increase above baseline S. Creat. |  |  | RIPC | Blood pressure cuff placed around their non-dominant upper arm within 2h pre-procedure. The cuff inflated to 200-mm Hg pressure for 5 minutes, followed by 5 minutes of deflation and repeated 2 more times to 3 cycles in total | | Mean±SD: 171.8±37.9 | | | Mean eGFR ±SD:  99.1±20.6mmol/l | |
|  |  |  |  |  |  |  | Control | no cuff | | Mean±SD: 163.3±39 | | | Mean eGFR ±SD:  100.8±28.2mmol/l | |
| Yamanaka 2015(179) | Inclusion Criteria   1. Age ≥ 20 years  2. Suspected STEMI undergoing PCI  Exclusion Criteria   1. Left bundle branch block 2. Previous CABG 3. Severe heart failure requiring percutaneous cardiopulmonary support 4. Severe chronic kidney disease requiring dialysis or continuous hemodiafiltration | 1.PCI | 109 | Increase in S. Creat. ≥0.5 mg/dL (44 µmol/L) or >25% increase above baseline 48-72h post-procedure | low-osm contrast media used in all cases | 0.9% Isotonic saline at 1ml/kg/h (0.5 ml/kg/h for patients in overt heart failure) beginning 24h pre-PCI and continuing for 24h | RIPC | Blood pressure cuff placed around their non-dominant upper arm. The cuff inflated to 200-mm Hg pressure for 5 minutes, followed by 5 minutes of deflation and repeated 2 more times to 3 cycles in total | |  | | | Mean S. Creat. ±SD:  0.82±0.21mg/dl | |
|  |  |  |  |  |  |  | Control | no cuff | |  | | | Mean S. Creat. ±SD:  0.87±0.44mg/dl | |
| Yang 2014(180) | Inclusion Criteria   1. Age > 18 years  2. Elective cardiovascular procedures  Exclusion Criteria   1. Severe renal dysfunction (eGFR <30 mL min) 2. Chronic renal failure-induced incomplete dialysis therapy 3. Exposure to radiographic contrast within 10 days 4. A second contrast-enhanced procedure planned within 72h 5. Previous emergent PCI 6. Heart failure and LVEF<40% 7. Previous CABG 8. Preoperative SBP<90mmHg 9. Kidney transplantation or congenital unilateral renal agenesis 10. Use of nephrotoxic medications  11. Valvular disorders 12. Active cancer 13. Uncontrolled hypertension 14. Previous dialysis 15. Autoimmune diseases 16. Chronic or acute infectious disease 17. Overt CHF 18. Recent acute kidney injury 19. Allergy to radiographic contrast combined with severe liver and lung diseases 20. Gastrointestinal bleeding 21. Infection 22. CarCI-AKIoma 23. Anaemia (Hb < 9gm/L) 24. Hyperthyroidism 25. Autoimmune diseases | 1.Coronary Angiography  2.PCI | 655 | Increase in S. Creat. ≥0.5 mg/dL (44 µmol/L) or >25% increase above baseline 3days post-procedure | Iopromide Low-Osm | as per intervention protocol | NaCl | 0.9 % sodium chloride at 1.5mL/kg/h 6h pre-procedure, and continued for 6h after | | Mean±SD: 124±63.81; | | | Mean eGFR. ±SD:  93.46±22.45 ml/min/1.73m^2^ | |
|  |  |  |  |  |  |  | NaHCO3 | 1.5 % NaHCO3 at 1.5 mL kg/h for 6h pre-procedure, continuing for 6h after | | Mean±SD: 127±48.09; | | | Mean eGFR. ±SD:  92.77±22.98 ml/min/1.73m^2^ | |
|  |  |  |  |  |  |  | NaCl | 0.9 % sodium chloride at 1.5mL/kg/h 6h pre-procedure, and continued for 6h after plus 600mg NAC BD 24h pre- and post-procedure | | Mean±SD: 129±46.77 | | | Mean eGFR. ±SD:  93.84±21.98 ml/min/1.73m^2^ | |
|  |  |  |  |  |  |  | NaHCO3 | 1.5 % NaHCO3 at 1.5 mL kg/h for 6h pre-procedure, continuing for 6h after plus 600mg NAC BD 24h pre- and post-procedure | | Mean±SD: 126±57.97 | | | Mean eGFR. ±SD:  92.76±23.05 ml/min/1.73m^2^ | |
| Yavari 2014(181) | Inclusion Criteria   1. Age 18-65 years 2. Baseline S. Creat. ≤132.6 lmol/l (1.5mg/dl)  Exclusion Criteria   1. Acute MI 2. Congestive heart failure 3. Hemodynamic instability during or after the procedure 4. Known allergy or previous administration of Pentoxifylline, and use of concomitant nephrotoxic agents (e.g. NSAIDs, aminoglycosides 5. Recent contrast injection 6. Diuretics | 1.PCI | 211 | >25% relative increase above baseline S. Creat. at 48h post-procedure | iso-osm Iodixanol | 0.9% saline at 1ml/kg/h for 6 h prior to, during, and 6h post-procedure | Pentoxyfylline | 400mg TDS starting day of procedure and day after | | Mean±SD: 191.96±94.32 | | | Mean S. Creat. ±SD:  1.06±0.16mg/dl | |
|  |  |  |  |  |  |  | IV fluid | Normal Saline only | | Mean±SD: 185.88±81.06 | | | Mean S. Creat. ±SD:  1.04±0.16mg/dl | |
| Yeganehkhah 2014(182) | Inclusion Criteria   1. Patients undergoing coronary angiography with at least one of the following risk factors:  i) Congestive heart failure (LVEF <40%) ii) History of Diabetes Mellitus iii) Age >65 years iv) Renal failure (eGFR <60 mL/min/1.73m2)  Exclusion Criteria   1. Pregnancy and lactation 2. History of allergic reaction to contrast agents 3. Cardiogenic shock 4. Pulmonary edema 5. Multiple myeloma 6. Mechanical ventilation 7. Urgent coronary angio¬graphy 8. S. Creat. >4 mg/dL 9. End-stage re¬nal disease (ESRD) 10.Receiving contrast agents two days prior to the study and 48h after 11. Using diuretics, NAC, sodium bicarbonate, theophylline, dopamine, mannitol, fenoldopam, metformin, and NSAIDs during the study 12. DBP >100mmHg  13. Need for further fluid therapy 14. Hypertension | 1.Coronary Angiography | 183 | >25% relative increase above baseline S. Creat. at 48h post-procedure | Iow-Osm Iohexol | 150 mL 8.4% NaHCO3 added to 850 mL isotonic normal saline | NaHCO3 | 3 mL/kg/h | |  | | | Mean S. Creat. ±SD:  1.17±0.32mg/dl | |
|  |  |  |  |  |  |  | NAC | NAC 600mg twice daily orally starting one day pre-procedure, in addition to isotonic normal saline at 1ml/kg/h (max 100ml/h) for 12h before and after angiography | | Mean±SD: 45.7±21.6 | | | Mean S. Creat. ±SD:  1.17±0.43mg/dl | |
|  |  |  |  |  |  |  | IV fluid | Isotonic normal saline at 1ml/kg/h (max 100ml/h) for 12h before and after angiography | | Mean±SD: 41.9±17.7 | | | Mean S. Creat. ±SD:  1.08±0.32mg/dl | |
| Yin 2013(183) | Inclusion Criteria   1. Consecutive patients admitted to the coronary care unit including:  i) Acute STEMI requiring PCI  ii) Acute NSTEMI requiring urgent coronary intervention | 1.PCI | 231 | Increase in S. Creat. ≥0.5 mg/dL (44 µmol/L) or >25% increase above baseline 72h post-procedure | low-osmolality contrast medium, Ultravist (Iopromide) | 0.9% Isotonic saline at 1ml/kg/h (0.5 ml/kg/h for patients in overt heart failure) for 24h | probucol | 1000 mg orally before primary or urgent angioplasty and 500 mg twice daily for 3 days after intervention | | Mean±SD: 168.89±79.77 | | | Mean S. Creat. ±SD:  0.8±0.23mg/dl | |
|  |  |  |  |  |  |  | Control | IV hydration only | | Mean±SD: 157.9±69.9 | | | Mean S. Creat. ±SD:  0.88±0.38mg/dl | |
| Zagidullin 2017(209) | Inclusion Criteria:  Patients undergoing coronary angiography with impaired renal function (eGFR <80ml/min)  Exclusion Criteria:  1. Acute coronary syndrome,  2. Acute kidney injury/decompensation of chronic kidney disease | 1.Coronary Angiography | 59 | Absolute (44 mmol/L) or relative increase in creatinine (by 25%) retested in day 2± 12 hours | Omnipaque | Body weight adapted 0.9% normal saline 6-12 hours before procedure | RIPC | 5-minutes cycle cuff inflation on the upper arm (systolic blood pressure + 50mmHg) with blood pressure cuff and with a 5-minutes rest between the cycles | | Mean+SD:  155.8±16.9 | | | Mean S. Creat. ±SD:  106.9±10.0 | |
|  |  |  |  |  |  |  | Placebo  (sham RIPC) | Cuff inflated to diastolic blood pressure | | Mean+SD:  148.3±16.7 | | | Mean S. Creat. ±SD:  124.8±11.04 | |
| Zhang 2010(184) | Inclusion Criteria   1. STEMI with heart failure within 24 h of onset of symptoms  2. Primary PCI  Exclusion Criteria   1. Cardiogenic shock 2. Inadequate blood volume 3. Mechanical complications of Acute MI 4. Known allergy | 1.PCI | 185 | Increase in S. Creat. ≥0.5 mg/dL (44 µmol/L) or >25% increase above baseline 72h post-procedure | Iodixanol Iso-Osm (used in patients with chronic kidney diseases) Iohexol Low-Osm (All other patients) | 0.9% Isotonic saline at an adjusted rate of 0.5–1.5 ml/kg/h depending on degree of heart failure from admission to 12 h after PCI | Human recombinant (Brain Natriuretic Peptide) (rhBNP) | 1.5 µg/kg bolus followed by adjusted dose infusion of 0.0075–0.030 µg/kg/min for 24 h after PCI | | Mean±SD: 189.86±51.66 | | | Mean S. Creat. ±SD:  90.89±17.64mmol/l | |
|  |  |  |  |  |  |  | Placebo | IV hydration only | | Mean±SD: 181.27±50.05 | | | Mean S. Creat. ±SD:  90.44±15.37mmol/l | |
| Zhao 2014(185) | Inclusion Criteria  1. Age 18-75 years 2. Acute coronary syndrome 3. Elective PCI  Exclusion Criteria   1. Cardiac shock State supported by device, such as intra-aortic balloon pump (IABP) 2. Hyperpyrexia 3. Allergy to iodine | 1.PCI | 110 | Increase in S. Creat. ≥0.5 mg/dL (44 µmol/L) or >25% increase above baseline 3 days post-procedure | Iodixanol Iso-Osm | 0.45% saline at 1ml/kg/h beginning 2-12h pre- and continuing for ≥ 12h post-procedure. | Cordyceps | 2g TDS for 3days before and after PCI | | Mean±SD: 248.86±48.68 | | | Mean S. Creat. ±SD:  82.8±19.2mmol/l | |
|  |  |  |  |  |  |  | Control | No cordyceps | | Mean±SD: 246.87±49.76 | | | Mean S. Creat. ±SD:  83.2±18.6mmol/l | |
|  |  |  |  |  |  |  | Cordyceps | 3g TDS for 3days before and after PCI | | excluded from analysis | | | | |
| Zhou 2012(186) | Inclusion Criteria  1. Age >18 years 2. Chronic renal insufficiency (eGFR<60 mL/min/1.73 m2 or baseline S. Creat. ≥ 1.1mg/dL)  Exclusion Criteria   1. Acute renal failure 2. End-stage renal disease requiring dialysis 3. Unstable renal function 4. Uncontrolled Diabetes Mellitus 5. NYHA class IV CHF or LVEF < 35% 6. Intraarterial or intravenous administration of iodinated CM from 7 days before to 72 hours after the administration of the study agents 7. Administration of any medication to prevent CI-AKI such as NAC 8. Intake of nephrotoxic medications from 24 hours before to 24 hours after the administration of the study agent 9. Recent ascorbic acid users (within 30 days before the procedure) | 1.Coronary Angiography  2.PCI | 166 | Increase in S. Creat. ≥0.5 mg/dL (44 µmol/L) or >25% increase above baseline | The choice of the type of contrast medium was left to the interventional cardiologist performing the procedure, but use of a non-ionic, low- or iso-osmolar contrast agent was encouraged Agents used: Iodixanol Iopromide Iohexol | Normal saline at 1 mg/kg/h for 4h pre- and for ≥12h post-procedure | Ascorbic Acid Group: | 3g IV pre-procedure, then 0.5 g orally BD for 2 days post-procedure: once before coronary angiography, starting the morning before the procedure and twice after coronary angiography, beginning the evening of procedure (total 5g) | | Mean±SD: 136±71.6 | | | Mean S. Creat. ±SD:  1.286±0.418mg/dl | |
|  |  |  |  |  |  |  | Control | IV hydration only | | Mean±SD: 133.7±74.4 | | | Mean S. Creat. ±SD:  1.248±0.364mg/dl | |
| **Trial** | **Inclusion and Exclusion Criteria** | **Procedure** | **Number of patients in analysis** | **CA-AKI Definition** | **Contrast / OSM** | **I.V Fluid** | **Intervention** | **Dose** | | **Contrast Volume (ml)** | | | **Baseline Renal function** | |
| A.C.T Investigators 2011(73) | Inclusion Criteria: Patients undergoing  1. Coronary or peripheral arterial diagnostic intravascular angiography OR  2. Percutaneous intervention  With  ≥1 risk factor for CI-AKI  1. Age >70 years 2. CRF (stable S. Creat. >132.6 mmol/L [1.5 mg/dL]) 3. Diabetes mellitus  4. Clinical evidence of congestive heart failure 5. LVEF<0.45 6. Hypotension  Exclusion Criteria:  1. Patients on dialysis 2. Primary angioplasty for STEMI 3. Pregnancy 4. Breastfeeding 5. Women of childbearing age not on contraception | 1. Peripheral vascular angiography 2. Coronary diagnostic angiography 3. PCI | 2561 | >25% increase in S. Creat. from baseline, 48-96 hours post-angiography | 1. Hi-Osm 2. Iso-Osm 3. Low-Osm | • 0.9% Normal Saline 1 mL/kg/hr from 6-12 hours pre- and 6-12 hours post-angiography  (Changes in volume/ speed of administration permitted) | NAC | 1200 mg orally every 12 hours • 2 doses pre- and 2 doses post- procedure | | Median (IQR): 100 (70-130) | | | Mean S. Creat. 1.2±0.5mg/dL | |
|  |  |  |  |  |  |  | Placebo | 1200 mg orally every 12 hours • 2 doses pre- and 2 doses post- procedure | | Median (IQR): 100 (70-130) | | | Mean S. Creat. 1.2±0.5mg/dL | |
| Abouzeid 2016(185) | Inclusion Criteria:  Adult patients > 18 years with renal dysfunction [estimated glomerular filtration rate (eGFR), 60 mL/min/1.73 m^2^or less]  Exclusion Criteria:  1. End-stage renal insufficiency (eGFR <15 mL/min)  2. Acute renal insufficiency  3.History of reaction to contrast media  4. use of potentially nephrotoxic medicines (48 h before and 24 h after the procedure)  5. pulmonary oedema  6. Multiple myeloma  7. Exposure to contrast media within seven days before the procedure  8. Pregnancy  9. Noncompliance of the patient  10. Use of N-acetylcysteine, dopamine, fenoldopam, and mannitol before CAG. | 1.Coronary Angiography | 210 | Increase of more than absolute 0.5 mg/dL and/or relative 25% in serum creatinine after 48 h | Iohexol Low-Osm | All patients were hydrated with intravenous (i.v.) normal saline at 1 mL/kg/h for 12 h before exposure to the contrast and 12 h after coronary angiography | Na/K citrate | 5 g of granules diluted in 200 mL of water 12, 6, and 1 h before procedure followed by another two doses after 2 and 8 h post procedure. | | Mean+SD:  103.93±47.70 | | | S. Creat.  Mean+SD:  2.30±0.46 | |
|  |  |  |  |  |  |  | NaHco3 | solution (150 mEq/L) for 8 h before procedure continued until 6 h post procedure | | Mean+SD:  105.24±45.38 | | | S. Creat.  Mean+SD:  2.28±0.44 | |
| Abaci  2015(15) | Inclusion Criteria:  Elective coronary or peripheral angiography    Exclusion Criteria  1. Emergency coronary angiography 2. Acute or end-stage renal failure requiring dialysis 3. eGFRs <30 or ≥60ml/min/1.73 m2 4. Congestive heart failure  5. Coronary artery disease 6. Severe coronary occlusion for CABG or PCI  7. Allergy to contrast media 8. Contrast media administration within the previous 14 days 9. Current statin treatment 10. Contraindications to statin treatment 11. Severe co-morbidities 12. Pregnancy 13. Patient refusal | 1.Coronary Angiography | 235 | Absolute increase in S. Creat. of ≥0.5 mg/dl OR Relative increase of ≥25% 48-72 hours post- procedure | Low-Osm (Optiray - Ioversol) | Isotonic saline (1ml/kg/h) for 12 hours before and 24 hours after contrast exposure | Rosuvastatin | 40 mg on admission Followed by 20 mg daily | | Mean ± SD.:  139.2 ± 77.4 | | | eGFRs <60 ml/min/1.73 m2 measured on day preceding procedure | |
|  |  |  |  |  |  |  | Control (no statin) | - | | Mean ± SD.:  117.7+/-56.8 | | |  | |
| Adolph 2008(16) | Inclusion Criteria:  1. Patients > 18y 2. Elective diagnostic/ therapeutic coronary angiography 3. Stable renal insufficiency (2 repeated S. Creat. levels > 106µmol/L (>1.2mg/dL) within 12 weeks before procedures, with <5% difference between levels  Exclusion Criteria:  1. Acute MI requiring primary or rescue PCI 2. Allergy to trial medication 3. Exposure to contrast media within 7 days of procedure 4. Thyroid dysfunction 5. Pregnancy 6. Uncontrolled hypertension 7. Life-limiting co-morbidity 8. Pulmonary Oedema 9. Chronic dialysis 10. Administration of Dopamine, Mannitol, Fenoldopam, or NAC during study | 1. Coronary Angiography 2. PCI | 150 | ≥25% or ≥0.5-mg/dL increase in the S. Creat. at day 0 , or 2 of contrast administration | Iodixanol Iso-Osm | 2ml/Kg/hour for 2 hours before procedure and 1ml/kg/hour during and for 6 hours after | NaCl | 154 mEq/l infusion of sodium chloride in 5% dextrose | | Mean ± SD (Range): 138±52 (51-282) | | | Mean S. Creat. ±SD (Range): 1.57±0.36 (1.20-2.60) mg/dL | |
|  |  |  |  |  |  |  | NaHCo_3_ | 154 mEq/l infusion of sodium bicarbonate in 5% dextrose | | Mean±SD (Range): 141±50 (39-270) | | | Mean S. Creat. ±SD (Range): 1.54±0.51 (1.20-4.60) mg/dL | |
| Akyuz  2014(17) | Inclusion Criteria:  1. Patients undergoing cardiac intervention  2. High risk of CA-AKI   Exclusion Criteria:  1. < 18 years old 2. Type 1 DM 3. History of Dialysis 4. No enough time for volume admission pre-procedure 5. Known allergy for contrast media 6. Exposure to contrast media within 3 days of procedure 7. Nephrotoxic drugs within 7 days of procedure  8. Pregnancy or breast feeding 9. Uncontrolled hypertension 10. AKI of alternative aetiology  11. Contraindication to volume expanders | 1. Coronary Angiography 2. PCI | 241 | ≥25% relative or ≥ 0.5mg/dL absolute increase in S. Creat. from baseline 48 hours post-contrast | Low-Osm Ultravist | As per intervention protocol | I.V Hydration with isotonic (0.9%) saline | 1ml/kg/hr for 12 hours pre-procedure and continued for 12 hours post-procedure | | Mean±SD: 108±76 | | | Median S. Creat (IQR): 0.9 (0.4) mg/dL | |
|  |  |  |  |  |  |  | Oral Hydration | Patients encourage to drink water as much as possible starting 12 hours pre-procedure until 2 hours directly prior to procedure, and continue the same for the 12 hours post-procedure | | Mean±SD: 107±70 | | | Median S. Creat (IQR): 0.9 (0.3) mg/dL | |
| Albabtain 2013(18) | Inclusion Criteria:  1. Patients > 18 years undergoing coronary angiography/ PCI 2. S. Creat. ≥ 1.3 mg/dL OR taking medication for Diabetes mellitus   Exclusion Criteria:  1. Known acute renal failure 2. End-stage renal disease requiring dialysis 3. Intravascular administration of contrast medium within 6 days of procedure 4. Anticipated re-administration of contrast medium within 6 days post-procedure 5. Use of Vitamin C supplements on a daily basis during the week before the procedure 6. Inability to administer the study medication at least 2 hours before the procedure   Note: A fourth study group using combination of trial interventions was excluded from analysis | 1. Coronary Angiography 2. PCI | 195 | 1. 0.5 mg/dL absolute increase in S. Creat.  AND/OR  2. 25% relative decrease in creatinine clearance | Ioxaglate Low-Osm | Normal Saline 50-125 mL/h IV from point of randomization until at least 6 hours post-procedure | NAC | 600 mg orally twice daily for 2 days, starting evening pre-procedure | | Mean±SD:  70.1 ± 60.4 | | | Mean S. Creat. ±SD:  1.45 ± 0.56mg/dL | |
|  |  |  |  |  |  |  | Ascorbic acid | Ascorbic acid as effervescent tablets as three doses:  1. 3g 2 hours pre-procedure 2. 2 g immediately post-procedure 3. 2 g 24 hours post-procedure | | Mean±SD:  88.3 ± 64.8 | | | Mean S. Creat. ±SD:  1.24± 0.44mg/dL | |
|  |  |  |  |  |  |  | Placebo | Placebo as effervescent tablets as three doses:  1. 2 hours pre-procedure 2. Immediately post-procedure 3. 24 hours post-procedure | | Mean±SD:  97.4±99.4 | | | Mean S. Creat. ±SD:  1.22 ± 0.40 mg/dL | |
| Allaqaband 2002(19) | Inclusion Criteria:  Patients undergoing cardiovascular interventions requiring the use of a radio contrast agent  AND  1. Baseline creatinine ≥1.6 mg/dl  OR   2. eGFR ≤ 60 ml/min  Exclusion Criteria: Not specified | 1.PCI | 143 | Increase in S. Creat. level > 0.5 mg/dl after 48 hours | Low-Osm | I.V. saline (0.45%) at 1ml/kg/hr for 12 hr prior to, during, and for 12 hr after the procedure | NAC | 600 mg orally twice daily starting the day before the procedure | | Mean±SD:  1.52±0.81 (ml/kg) | | | Mean S. Creat. ±SD:  2.20±0.73 mg/dL | |
|  |  |  |  |  |  |  | Fenoldopam | I.V. Fenoldopam at 0.1 µg/kg/min starting 4 hr prior to and continued for 4 hr after contrast dose | | Mean±SD:  1.63±0.67 (ml/kg) | | | Mean S. Creat. ±SD:  1.94±0.38 mg/dL | |
|  |  |  |  |  |  |  | Control | I.V fluid only as specified for all groups | | Mean±SD:  1.47±0.90 (ml/kg) | | | Mean S. Creat. ±SD:  2.03±0.79 mg/dL | |
| Amini 2009(20) | Inclusion Criteria  1. Elective diagnostic coronary angiography 2. > 18 years  3. Diabetes mellitus for at least one year AND  4. CKD (S. Creat. ≥ 1.5 mg/dL (male) or ≥ 1.4 mg/dL (female))   Exclusion Criteria  1. Acute coronary syndrome requiring primary or rescue coronary intervention within 12 h of procedure 2. Cardiogenic shock 3. Current peritoneal or haemodialysis 4. Known allergy to NAC | 1.Coronary Angiography | 98 | Increase in serum creatinine ≥ 0.5 mg/ dL (44.2 μmol/L) or ≥ 25% above baseline at 48 h after exposure to contrast | Iohexol Low-Osm Iodixanol Iso-Osm | 1L normal saline (0.9%) commenced in the catheterization laboratory Patients were also encouraged to drink fluids (≥8 glasses of ~200ml) over 12 h pre-procedure | NAC | 600 mg orally twice daily, starting the day before the procedure and continuing for 2 doses post-procedure | | Mean±SD:  118.00±35.20 | | | Mean S. Creat. ±SD:  1.736±0.42 mg/dL | |
|  |  |  |  |  |  |  | Placebo | 600 mg orally twice daily, starting the day before the procedure and continuing for 2 doses post-procedure | | Mean±SD:  121.11±43.95 | | | Mean S. Creat. ±SD:  1.736±0.17 mg/dL | |
| Angoulvant 2009(21) | Inclusion Criteria:  1. Patients 18-80 years  2. Elective coronary angiography ± Percutaneous transluminal coronary angioplasty  AND  3. Baseline S. Creat. < 140µmol/L (1.58 mg/dL)   Exclusion Criteria:  1. NYHA Class IV congestive heart failure 2. Pregnancy 3. Significant valvular heart disease 4. Non-ischaemic dilated cardiomyopathy 5. Active cancer or any life-threatening disease | 1.Coronary Angiography ± PCI | 213 | Increase in serum creatinine > 25% 3 days after contrast exposure | Hexabrix Low-Osm | As per intervention protocol | I.V Hydration | 1000mL of 0.9% saline infusion, started at the beginning of the procedure and continued for the next 24 hours | | Mean±SD:  231.4±7.8 | | | Mean S. Creat. ±SD:  86.7±1.7 µmol/L | |
|  |  |  |  |  |  |  | Control | Received no additional hydration | | Mean±SD:  242.7±8.6 | | | Mean S. Creat. ±SD:  85.6±1.5 µmol/L | |
| Arabmomeni 2015(186) | Inclusion Criteria:  Patients with at least moderate risk for CIN as defined by the Mehran risk score  Exclusion Criteria:  1. unstable angina  2. Myocardial infarction  3. Cardiac arrhythmia  4. Acute or chronic renal failure  5. I.V contrast media in the past month  6. Using theophylline or NAC in the past month  7. Known hypersensitivity to Trial Drugs | Coronary angiography with or without angioplasty | 75 | increase in creatinine of ≥ 0.5 mg/dl or ≥ 25% from the baseline | Iodixanol  Low-Osm | 0.9% sodium chloride (1 ml/kg/h) for 24 h, started 12 h before operation. Patients with left-ventricular ejection fraction of less than 40% or New York Heart Association functional class of III-IV were hydrated at rate of 0.5 ml/kg/h | Theophylline | | 200 mg slow-release theophylline tablet plus placebo | Mean+SD:  124.0 ± 115.2 | | S. Creat.  Mean+SD:  1.14 ± 0.40 | | |
|  |  |  |  |  |  |  | NAC | | 600 mg tablet plus placebo | Mean+SD:  155.6 ± 114.9 | | S. Creat.  Mean+SD:  1.08 ± 0.22 | | |
|  |  |  |  |  |  |  | Theophylline plus NAc (this group were excluded from this analysis) | | Both tablets  All groups receive tablets twice daily, from 24 h before to 48 h after administration of contrast material | Mean+SD:  128.9 ± 89.4 | | S. Creat.  Mean+SD:  1.08 ± 0.22 | | |
| Aslanger 2012(22) | Inclusion Criteria:  1. Patients with STEMI undergoing primary angiography within 12 h of symptoms  2. > 30 years old  Exclusion Criteria:  1. Known allergies to NAC 2. Undergoing chronic dialysis 3. No written informed consent | Primary Angioplasty | 257 | Increase in S. Creat. > 25% 3 days after contrast exposure | Ioxaglate Low-Osm | Isotonic saline hydration (0.9%) for 12 h at 1 ml/kg/h | I.V. NAC | 1200mg bolus during the procedure and 1200mg orally, twice daily for 48h post-procedure | | Mean±SD:  193±57 | | | Mean S. Creat. ±SD:  0.9±0.3 mg/dL | |
|  |  |  |  |  |  |  | Intra-renal NAC   **This group is excluded from our analysis** | 600mg bolus into both renal arteries before and after the procedure, as well as 1200mg of NAC orally twice daily for 48h | |  | | | Mean S. Creat. ±SD:  0.89±0.4 mg/dL | |
|  |  |  |  |  |  |  | Placebo | 12ml bolus of IV saline during procedure and oral doses twice daily for 48h post procedure | | Mean±SD:  204±67 | | | Mean S. Creat. ±SD:  0.86±0.3 mg/dL | |
| Baker 2003(23) | Inclusion Criteria:  Patients with stable renal dysfunction (S. Creat. > 1.36mg/dL OR creatinine clearance <50ml/min)  Exclusion Criteria:  1. Acute Renal failure 2. End stage renal failure on dialysis 3. Patients who received a NSAID (except Aspirin 75-150 mg) within 24h of procedure 4. Systolic blood pressure <90mmHg 5. Hemodynamically significant valvular heart disease 6. Signs of Cardiac failure | 1. Coronary Angiography 2. PCI | 90 | Increase in S. Creat. ≥ 25% 2-4 days after contrast administration | Iodixanol Iso-Osm | Free oral fluids were commenced immediately post-procedure in all patients | I.V. NAC | 150 mg/kg in 500 ml saline (0.9%) over 30 min immediately before contrast exposure followed by 50 mg/kg in 500 ml saline (0.9%) over the subsequent 4 hours | | Mean±SD:  238±155 | | | Mean S. Creat. ±SD:  1.85±0.59 mg/dL | |
|  |  |  |  |  |  |  | Control (IV Fluids) | Normal Saline at 1 ml/kg/h for 12 h pre- and post-procedure. | | Mean±SD:  222±162 | | | Mean S. Creat. ±SD:  1.75±0.41 mg/dL | |
| Balderramo 2004(24) | Inclusion Criteria:  Patients with chronic renal impairment (stable S. Creat. > 1.5mg/dL OR eGFR <50ml/min)) undergoing a same day angiography  Exclusion Criteria 1. Haemodynamic instability 2. Congestive Heart failure 3. Pregnancy 4. Lactation 5. Severe Asthma 6. Contrast media use within preceding 10 days | 1.Coronary angiography  2.Peripheral angiography | 64 | Increase in S. Creat. >0.5mg/dL 48 hours after the procedure | Amidotrizate  Ioxitalamate Iopamidol Iohexol | All patients receive 0.9% normal saline at 4ml/kg/hr for 3 hours prior to, and 2ml/kg/hr for 6 hours after radiocontrast exposure | NAC | 1200mg orally with 125ml of orange juice 3 hours before and 3 hours after catheterization | | Mean±SD:  158±60.5 | | | Mean S. Creat. ±SD:  1.46±0.46mg/dL | |
|  |  |  |  |  |  |  | Placebo | 125ml of orange juice 3 hours before and 3 hours after catheterization | | Mean±SD:  155.5±108.1 | | | Mean S. Creat. ±SD:  1.42±0.37mg/dL | |
| Baskurt 2009(25) | Inclusion Criteria:  1. Patients with moderate CKD (eGFR 30-60 mL/min) undergoing coronary angiography   Exclusion Criteria: 1. Acute coronary syndrome 2. Cardiogenic shock 3. Chronic haemodialysis 4. Overt congestive heart failure 5. Exposure to radiocontrast medium within preceding 14 days 6. Emergent procedures 7. Patients with eGFR < 30 or ≥60 mL/min 8. Age < 21 years 9. Known allergy to NAC, theophylline or contrast agents 10. Contraindications to theophylline 11. Medication shown exerting pharmacokinetic interaction with theophylline | 1.Coronary Angiography | 157 | Increase in S. Creat. >0.5mg/dL 48 hours after the procedure | Ioversol Low-Osm | Isotonic saline 1 mL/kg/h for 12 hours before and after contrast | NAC | 600 mg orally twice daily day preceding and day of angiography | | Mean±SD:  115.61±35.2 | | | Mean S. Creat. ±SD:  1.39±0.24mg/dL | |
|  |  |  |  |  |  |  | NAC+ Theophylline Group: This Group is not included in this analysis | | | | | | | |
|  |  |  |  |  |  |  | Control | I.V Hydration only | | Mean±SD:  113.54±37.7 | | | Mean S. Creat. ±SD:  1.30±0.20mg/dL | |
| Berwanger 2013(26) | Inclusion Criteria  1. Diabetes Mellitus  Exclusion Criteria 1. Dialysis 2. STEMI  3. Pregnancy 4. Breastfeeding 5. Females aged <45 years not using contraception | 1. Angiography 2. Percutaneous intervention | 1421 | Increase in S. Creat. >0.5mg/dL | High-Osm Low-Osm Iso-Osm | 0.9% saline, 1 mL/kg/hr 6-12 hours pre- to 6-12 hours post-angiography | NAC | 1200 mg orally every 12 hours • 2 doses pre- and 2 doses post- procedure | | Median (IQR): 100 (70-130) | | | Mean S. Creat. ±SD:  1.1±0.5mg/dL | |
|  |  |  |  |  |  |  | Placebo | 1200 mg orally every 12 hours • 2 doses pre- and 2 doses post- procedure | | Median (IQR): 100 (70-130) | | | Mean S. Creat. ±SD:  1.1±0.6mg/dL | |
| Bidram 2015(27) | Inclusion Criteria   1. Chronic stable angina  Exclusion Criteria  1. Pregnancy 2. Diabetes mellitus 3. Renal failure 4. Single kidney 5. Cardiogenic shock 6. Unstable angina 7. MI 8. Hypersensitivity to statins 9. Previous intravascular contrast injection within month of admission | 1.PCI | 203 | Increase in serum creatinine > 0.5mg/dL 48 hours after the procedure Increase in S. Creat. of >0.5 mg/dl or >25% from baseline | Iso-Osm Iodixanol | 1 ml/kg/h of isotonic saline solution 12 h pre- and post-contrast | Atorvastatin (patients previously on statins excluded from analysis) | 80mg | | 30-40cc | | | Mean S. Creat. ±SD:  1.18±0.02mg/dL | |
|  |  |  |  |  |  |  | Placebo |  | | 30-40cc | | | Mean S. Creat. ±SD:  1.14±0.02mg/dL | |
| Bilasy 2012(28) | Inclusion Criteria   1. Stable S. Creat.  2. Moderate risk for CI-AKI  3. Elective intervention  serum creatinine and at least moderate risk for CI-AKI   Exclusion Criteria   1. Patients with unstable S. Creat.  2. Recent i.v. administration of Contrast within month of procedure  3. Shock 4. End-stage renal disease 5. Haemodialysis 6. Hypersensitivity to NAC or theophylline 7. Serious cardiac arrhythmias 8. Seizures 9. Acute renal failure | 1. Coronary Angiography 2. PCI | 66 | Elevation of the S. Creat. by ≥0.5mg/dL or ≥25% within 3 days of contrast injection | Iopamidol Low-Oam | 0.9% sodium chloride (1 mL/kg/hr for 24 hours beginning 12 hours before the procedure.  All patients received NAC 600 mg twice daily, on the day before and the day of procedure | Theophylline | 200 mg in 100 mL sodium chloride (0.9%) I.V 30 minutes before Contrast | | Mean±SD:  116.33±59.44 | | | Mean S. Creat. ±SD:  1.54±0.73mg/dL | |
|  |  |  |  |  |  |  | Placebo | 100 mL sodium chloride (0.9%) 30 minutes before the procedure. | | Mean±SD:  117.17 ± 63.13 | | | Mean S. Creat. ±SD:  1.34±0.66mg/dL | |
| Boscheri 2007(29) | Inclusion Criteria   1. Chronic renal failure and stable S. Creat. > 120 µmol/l)  Exclusion Criteria  1. MI within 3 months 2. Cardiogenic Shock 3. Use of Vasopressors 4. Ejection Fraction < 25% 5. Acute renal failure  6. Current peritoneal dialysis or haemodialysis 7. Pregnancy 8. Exposure to contrast dye or medication with NAC up to 72 hours prior to study entry | 1. Coronary Angiography 2. PCI | 151 | ≥ 25% Absolute rise of the S. Creat. day 2 compared to baseline | Iodixanol Iso-Osm | 500 ml of normal saline 2 hours prior to procedure. 500 ml during the procedure and for 6 hours after | Ascorbic acid | 1 g ascorbic acid orally 20 minutes prior to contrast exposure | | Mean ±SD: 99±46 | | | Mean S. Creat. ±SD:  1.75±0.4mg/dL | |
|  |  |  |  |  |  |  | Placebo | 1 g placebo orally 20 minutes prior to contrast exposure | | Mean ±SD: 112±67 | | | Mean S. Creat. ±SD:  1.73±0.4mg/dL | |
| Boucek 2013(30) | Inclusion Criteria   1. >18 Years old 2. Diabetic patients with impaired renal function (Serum creatinine ≥100 mmol/L) 3. Use of low-osmolar contrast media.  Exclusion Criteria   1. End-stage renal disease 2. Chronic dialysis treatment 3. Kidney transplant 4. Pre-planned dialysis following the contrast-involving procedure 5. Emergency procedures 6. Acute kidney injury (serum creatinine increase ≥50 mmol/L during the previous 24-h period) 7. Volume overload with left ventricular failure 8. Uncontrolled hypertension (systolic BP ≥ 180 or diastolic BP ≥ 110 mmHg) 9. Hemodynamic instability (systolic BP <90 and diastolic BP <50 mmHg) 10. Contrast use in the previous 48-h period 11. Multiple myeloma 12. Pregnancy 13. Breastfeeding 14. Pre-planned use of any other measure for CI-AKI prevention | 1. Elective procedure with use of low-osmolar contrast media | 132 | Increase in S. Creat. ≥ 25% and/or ≥ 44 mmol/L within 2 days after contrast administration | Low-Osm | Both groups receive infusion solutions 1 hour pre-procedure at 3 mL/kg/h to maximum 330mL; and for 6 hours after at 1mL/kg/h to maximum of 660 mL | NaHCO3 | 154 mL of 8.4% NaHCO3 add to 846 mL of 5% glucose | | Mean ±SD: 115±47 | | | Mean S. Creat. ±SD:  170±84 mmol/L | |
|  |  |  |  |  |  |  | Control (IV Fluids) | 154 mL of 5.85% NaCl added to 846 mL of 5% Glucose. | | Mean ±SD: 104±32 | | | Mean S. Creat. ±SD:  160±74 mmol/L | |
| Brar 2008(31) | Inclusion Criteria   1. Age ≥ 18 years  2. Estimated GFR ≤60 mL/min/1.73 m2  2. One or more of  a. Diabetes Mellitus b. congestive heart failure c. hypertension d. Age > 75 years  Exclusion Criteria   1. Inability to obtain consent 2. Receipt of NaHco3 infusion prior to randomization 3. Emergency cardiac catheterization 4. Intra-aortic balloon counter-pulsation 5. Dialysis 6. Exposure to radiographic contrast media within the preceding 2 days 7. Allergy to contrast media 8. Acutely decompensated CHF 9. Severe valvular Abnormality 10. Single functioning kidney 11. History of kidney or heart transplantation 12. Change in eGFR ≥ 7.5% per day or cumulative change ≥ 15% in preceeding 2 or more days. | 1.Coronary Angiography | 379 | ≥25% or ≥0.5-mg/dL increase in S. Creat. | Ioxilan Low-Osm | As per intervention protocol | NaHCO3 | 3 mL/kg for 1 hour before coronary angiography, decreased to 1.5 mL/kg/hr during the procedure and for 4 hours after | | Median (IQR): 126 (80-214) | | | Median S. Creat. (IQR), 1.49 (0.36) mg/dL | |
|  |  |  |  |  |  |  | NaCl | 3 mL/kg for 1 hour before coronary angiography, decreased to 1.5 mL/kg/hr during the procedure and for 4 hours after | | Median (IQR): 137 (89-247) | | | Median S. Creat. (IQR), 1.49 (0.36) mg/dL | |
| Briguori 2002(34) | Inclusion Criteria   1. Chronic renal impairment (S. Creat. >1.2 mg/dl and/or eGFR <70 ml/min) 2. Elective procedure  Exclusion Criteria  None specified | 1. Coronary Angiography ± Angioplasty 2. Peripheral Angiography ± Angioplasty | 199 | 1. Increase of ≥25% in S. Creat. 48 hours after the procedure OR 2. Need for dialysis post contrast | Iopromide Low-Osm | 0.45% saline I.V. at 1ml/kg/hour for 12hours pre- and 12 hours post-procedure | NAC | 600 mg orally every 12 hours • before and on day of administration, total of 2 days | | Mean ±SD: 194±127 | | | Mean S. Creat. ±SD:  1.52±0.43 mg/dL | |
|  |  |  |  |  |  |  | Control | I.V fluid only | | Mean ±SD: 200±144 | | | Mean S. Creat. ±SD:  1.54±0.36 mg/dL | |
| Briguori 2004(33) | Inclusion Criteria   1. Elective patients  2. Chronic renal impairment (S. Creat. ≥1.5 mg/dl and/or creatinine clearance <60 ml/min)  3. Stable S. Creat.  Exclusion Criteria  None specified | 1. Coronary Angiography ± Angioplasty 2. Peripheral Angiography ± Angioplasty | 209 | 1. Increase in S. Creat. by ≥0.5mg/dL 48 hours after the procedure OR 2. Need for dialysis post contrast | Iodixanol Iso-Osm | 0.45% saline I.V. at 1ml/kg/hour (or 0.5ml/kg/hour in patients with LVEF<40%) for 12hours pre- and 12 hours post-procedure | NAC | 1200 mg orally every 12 hours • before and on day of administration, total of 2 days | | Mean ±SD: 160±82 | | | Median S. Creat. (IQR): 1.72 (1.55-1.90) mg/dL | |
|  |  |  |  |  |  |  | Fenoldopam | Infusion started at least 1 h pre-procedure at 0.10 µg/kg/min, continued during and for 12 h post-procedure | | Mean ±SD: 168±104 | | | Median S. Creat. (IQR): 1.75 (1.62-2.01) mg/dL | |
| Briguori 2007(32) | Inclusion Criteria   1. Patients ≥18 years 2. Chronic kidney disease (stable S. Creat. ≥2.0 mg/dL and/or estimated glomerular filtration rate <40 mL/min)  Exclusion Criteria   1. S. Creat. ≥8 mg/dL 2. History of dialysis 3. Multiple myeloma 4. Pulmonary oedema 5. Acute MI 6. Recent exposure to radiographic contrast within 2 days of the study 7. Pregnancy 8. Administration of theophylline, dopamine, mannitol, or fenoldopam | 1. Coronary Angiography 2. Peripheral Angiography | 238 | Increase of ≥25% in S. Creat. 48 hours after the procedure | Iodixanol Iso-Osm | All patients received NAC orally at a dose of 1200 mg twice daily on the day before and the day of administration of the contrast agent  3^rd^ Group with mixed treatments excluded | NaCl | Isotonic saline (0.90%) I.V at a rate of 1 mL/kg/hr (0.5 mL/kg for patients with LVEF<40%) for 12 hours before and 12 hours after administration of the contrast agent | | Mean ±SD: 179±102 | | | Median S. Creat. (IQR), 1.95 (1.69-2.26) mg/dL | |
|  |  |  |  |  |  |  | NaHCO3 | 154 mEq/L NaHCO_3_ in dextrose and H_2_O bolus 3ml/kg/h for 1 hour immediately before contrast; then infusion at 1 mL/kg/h during contrast exposure and for 6 hours after the procedure. | | Mean ±SD: 169±92 | | | Median S. Creat. (IQR): 2.04 (1.86-2.36) mg/dL | |
| Brueck 2013(35) | Inclusion Criteria   1. Chronic Renal Impairment (S. Creat. ≥1.3 mg/dL)  2. Elective procedure  Exclusion Criteria   1. Variability of ≥0.3mg/dL in S. Creat. measurements in 7 days pre-procedure 2. Exposure to contrast agents or nephrotoxic medication within week of procedure  3. Renal transplant recipients 4. Patients with Plasmocytoma, Oxalosis, Nephrolithiasis or Hyperthyroidism  5. Inadequate time for study measurements pre-procedure  study procedures 6. Previously known sensitivity to NAC or ascorbic acid. 7. Pregnancy  8. Breast feeding women 9. Child-bearing females not using contraception | 1.Cardiac Catheterization | 622 | 1. Increase in S. Creat. by ≥0.5mg/dL (≥44.2 µmol/L) with 72 hours of procedure | Ultravist Low-Osm | 0.9% sodium chloride (1 mL/kg/hr for 12 hours pre- and 12 hours post-procedure. | I.V. NAC | 600 mg in 250 ml saline (0.9%) over 30 min 24 hours and one hour before contrast exposure | | Median (IQR): 110 (80-160) | | | Median S. Creat. (IQR): 1.5 (1.3-1.8) mg/dL | |
|  |  |  |  |  |  |  | Ascorbic acid | 500mg in 250 ml saline (0.9%) over 30 min 24 hours and one hour before contrast exposure | | Median (IQR): 115 (90-150) | | | Median S. Creat. (IQR): 1.5 (1.3-1.7) mg/dL | |
|  |  |  |  |  |  |  | Control (IV Fluids) | as per fluid protocol | | Median (IQR): 110 (80-150) | | | Median S. Creat. (IQR): 1.5 (1.3-1.7) mg/dL | |
| Burns 2010(36) | Inclusion Criteria   1. Critically ill adult patients 2. ≥ 18 years of age 3. Undergoing contrast-enhanced CT of any organ 4. Patient considered at risk of CI-AKI  Exclusion Criteria   1. CK > 5,000 2. Myoglobinuria 3. Known hypersensitivity to contrast dye or NAC 4. Serious illness with low likelihood of survival within 48-hours or poor prognosis 5. Pregnancy 6. Cardiogenic shock 7. Known or suspected nephritic, nephrotic or pulmonary-renal syndromes 8. Post-renal aetiology of renal impairment 9. Previous renal transplant 10. Known solitary kidney 11. Serum creatinine > 200 µmol/L 12. Recent exposure to radiographic contrast within 14 days of randomization. | Contrast Enhanced CT | 46 | 1. Increase in S. Creat. by > 50 µmol/L from the time of randomization up to day 5 following contrast exposure | not specified | Normal saline 12 hours pre- and 12 hours post CT | I.V. NAC | 5 g in 100 ml D5W before CT, 2.5 g in 50 ml D5W at 6 and 12 hours post-CT | | not specified | | | Mean S. Creat. ±SD:  102±40.9 mmol/L | |
|  |  |  |  |  |  |  | Placebo | 5 g in 100 ml D5W before CT, 2.5 g in 50 ml D5W at 6 and 12 hours post-CT | | not specified | | | Mean S. Creat. ±SD:  118.1±26.3 mmol/L | |
| Carbonell 2007(37) | Inclusion Criteria  1. Patients with ACS syndrome 2. Normal renal function (Stable S. Creat. <1.4 mg/dl (123.76 μmol/l) or a creatinine clearance >60 ml/min)  Exclusion Criteria   1. Hemodynamic instability (Systolic B.P. <90 mm Hg)  2. Known allergy to NAC or contrast agent 3. Untreated gastrointestinal bleeding 4. Current or previous treatment with theophylline, Mannitol or nephrotoxic drugs. | 1. Coronary Angiography | 238 | Increase in S. Creat. ≥0.5 mg/dL (44 µmol/L) or >25% increase above baseline at 48 h post-contrast | Iopromide Low-Osm | As per intervention protocol | I.V. NAC | 600 mg in 50 ml saline (0.9%) over 30 min twice daily for total of 4 doses, starting at least 6 hours pre-procedure | | Mean ±SD: 193±11 | | | Mean S. Creat. ±SD:  0.94±0.16 mg/dL | |
|  |  |  |  |  |  |  | Control (IV Fluids) | 50 mL of 0.9% saline solution for 30 minutes twice daily for a total of 4 doses. | | Mean ±SD: 183±10 | | | Mean S. Creat. ±SD:  0.96±0.17 mg/dL | |
| Carbonell 2010(38) | Inclusion Criteria   1. Patients with chronic kidney disease (S. Creat. ≥1.4 mg/dL (123.76 µmol/L)9 Or <60 mL/min creatinine clearance)  Exclusion Criteria   1. Hemodynamic instability (Systolic B.P. <90 mm Hg)  2. Known allergy to NAC or contrast agent 3. Untreated gastrointestinal bleeding 4. Current or previous treatment with theophylline, Mannitol or nephrotoxic drugs. | 1. Coronary Angiography | 93 | Increase in S. Creat. ≥0.5 mg/dL (44 µmol/L) or >25% increase above baseline at 48 h post-contrast | Iopromide Low-Osm | As per intervention protocol | I.V. NAC | 600 mg in 50 ml saline (0.9%) over 30 min twice daily for total of 4 doses, starting at least 6 hours pre-procedure | | Mean ±SD: 134.79±13 | | | Mean S. Creat. ±SD:  2.01±0.77 mg/dL | |
|  |  |  |  |  |  |  | Control (IV Fluids) | 50 mL of 0.9% saline solution for 30 minutes twice daily for a total of 4 doses. | | Mean ±SD: 184.66±21 | | | Mean S. Creat. ±SD:  1.87±0.7 mg/dL | |
| Castini 2010(4) | Inclusion Criteria   1. Patients aged 18 years or older  2. Stable S. Creat. ≥1.2mg/dL  Exclusion Criteria   1. S. Creat. >4mg/dL 2. History of dialysis 3. Multiple myeloma 4. Pulmonary oedema 5. Cardiogenic shock 6. Acute MI 7. Emergency catheterization  8. Exposure to contrast media within 7 days of the study 9. Allergy to iodinate contrast media or NAC 10. Previous enrolment in the same or other protocols 11. Pregnancy 12. Administration of theophylline, mannitol, dopamine, dobutamine, nonsteroidal anti-inflammatory drugs, or fenoldopam. | 1. Coronary Angiography ± PCI | 117 | CI-AKI1: ≥25% increase in S. Creat. over baseline at any of 3 predefined time-points.  (24 hours, 48 hours and 5 days)  CI-AKI2: Absolute increase of ≥0.5mg/dL in S. Creat at the same time-points   NB : CI-AKI1 used for this analysis | Iodixanol Iso-Osm | As per intervention protocol | NaCl | 1mL/kg/hr for 12 hours pre- and 12 hours post-contrast exposure | | Mean ±SD: 196.4±127.7 | | | Mean S. Creat. ±SD:  1.59±0.38 mg/dL | |
|  |  |  |  |  |  |  | NaHCO3 | 154 mEq/L at 3mL/kg/hr for one 1 hour immediately pre-contrast injection, then 1mL/kg/hr during contrast exposure and for 6 hours post-procedure. | | Mean ±SD: 179.2±125.1 | | | Mean S. Creat. ±SD:  1.59±0.38 mg/dL | |
|  |  |  |  |  |  |  | NAC and IV NaCl | Excluded from analysis | | Mean ±SD: 210.5±140.6 | | | Mean S. Creat. ±SD:  1.57±0.38 mg/dL | |
| Chen 2008(39) | Inclusion Criteria   1. Myocardial ischemia   Patients divided according to S. Creat.: <1.5mg/dL: Normal arm >1.5mg/dL: Abnormal arm before further randomization.   Normal and abnormal arms are considered separately  Exclusion Criteria   1. Coronary anatomy not suitable for PCI 2. Emergency CABG required 3. Patients on peritoneal or haemodialysis 4. Acute MI 5. No written formal consent  6. Patients with normal coronary arteries | 1.PCI | 1185 | Absolute increase in S. Creat of 0.5mg/dl at 48 h post-procedure | Iso-Osm | As per intervention protocol | IV hydration* *Abnormal group received 1200mg NAC 12hours pre-procedure and again post procedure | Both arms: 0.45% saline I.V at 1ml/kg/h starting 12h pre-procedure | | Mean ±SD: Normal Arm: 285±107 Abnormal Arm: 298±125 | | | Mean S. Creat. ±SD:  Normal Arm: 1.3±0.3 mg/dL Abnormal Arm: 2.5±0.1 mg/dL | |
|  |  |  |  |  |  |  | Control (no hydration) * *Abnormal group received 1200mg NAC 12hours pre-procedure and again post procedure | Control Group: 1. Normal Arm: No hydration 2.Abnormal Arm: No Hydration | |  |  |  |  |  |
| Cho 2010(40) | Inclusion Criteria   1. Chronic kidney disease 2. 18 years 3. Stable S. Creat. of 1.1 mg/dL or estimated creatinine clearance <60mL/min  4. Diagnostic, elective Cardiac angiography  Exclusion Criteria   1. Dialysis 2. Multiple myeloma 3. Myeloproliferative disease 4. Current decompensated heart failure or significant change in NYHA symptom Class 5. Current MI 6. Symptomatic hypokalaemia 7. Uncontrolled hypertension (SBP >200mmHg or DBP >100mmHg) 8. Exposure to radiocontrast within 7 days of enrolment 9. Emergency catheterization  10. Allergy to contrast 11. Pregnancy 12. Administration of dopamine, mannitol, fenoldapam or NAC during the study 13. Exacerbation of COPD  14. S. HCO3>28mEq/L 15. S. Sodium <133 mEq/L | 1.Coronary Angiography | 79 | Increase in S. Creat. ≥0.5 mg/dL (44 µmol/L) or >25% increase above baseline at 72 h post-contrast | Isoversol Low-Osm | As per intervention protocol | NaCl | 154 mEq/L at 3mL/kg/hr for one 1 hour immediately pre-contrast injection, then 1mL/kg/hr for 6 hours post-procedure. | |  | | | Mean S. Creat.:  1.38mg/dL | |
|  |  |  |  |  |  |  | NaHCO3 | 154 mEq/L at 3mL/kg/hr for one 1 hour immediately pre-contrast injection, then 1mL/kg/hr for 6 hours post-procedure. | |  | | | Mean S. Creat.:  1.38mg/dL | |
|  |  |  |  |  |  |  | Oral Hydration | 500 mL of water to be started 4 hours prior to contrast exposure and stopped 2 hours prior to procedure and 600 mL of water post-procedure | |  | | | Mean S. Creat.:  1.38mg/dL | |
|  |  |  |  |  |  |  | NaHCO3 and Oral Hydration | Excluded from analysis | | | | | | |
| Cicek 2013(41) | Inclusion Criteria  1. Diabetic patients 2. Elective coronary angiography  Exclusion Criteria   1. S. Creat. >1.5 mg/dL 2. Known malignancy 3. Liver disease 4. Allergy to contrast media 5. Use of any nephrotoxic agent within 48 h 6. Exposure to contrast agent within 7 days 7. Unstable angina 8. Hemodynamically unstable patients 9. Patients requiring PCI | 1.Coronary Angiography | 84 | Increase in S. Creat. ≥0.5 mg/dL (44 µmol/L) or >25% increase above baseline at 48 h post-contrast | Iohexol Low-Osm | saline infusion at 1 mL/kg/h from 3 h before procedure continued for 24 h | Alpha-Lipoic Acid | 600 mg Thioctacid in 3 doses before meal; 30 min pre- and at 24 and 48-hour post-Angiography | | Median (IQR): 75 (50-100) | | | Mean S. Creat. ±SD:  0.86±0.18mg/dL | |
|  |  |  |  |  |  |  | Control | nil | | Median (IQR): 80 (60-120) | | | Mean S. Creat. ±SD:  0.89±0.18mg/dL | |
| Coyle 2006(42) | Inclusion Criteria   1. Age >18 years  2. Diabetes Mellitus  Exclusion Criteria   1. Emergency procedure  2. Inability to complete hydration protocol 3. History of Contrast Nephropathy | 1.Coronary Angiography | 141 | Increase in S. Creat. ≥0.5 mg/dL (44 µmol/L) above baseline at 48 h post-contrast | Mixed: Selection and volume of contrast at the discretion of the operator | Oral hydration with 1litre of clear fluids over 10 hours before angiography.  IV hydration from start of procedure with 0.45% saline at 300ml/hour for 6 hours. | NAC | 600 mg orally every 12 hours • 2 doses before and 2 days after administration of contrast | | Mean ±SD: 88±61 | | | Mean S. Creat. ±SD:  1.16±0.38mg/dL | |
|  |  |  |  |  |  |  | Control | No intervention | | Mean ±SD: 98±65 | | | Mean S. Creat. ±SD:  1.10±0.44mg/dL | |
| Durham 2002(43) | Inclusion Criteria   1. S. Creat. ≥1.7 mg/dL  Exclusion Criteria   1. < 18 years 2. Renal disease with reversible component 3. Unwilling or unable to provide informed consent 4. Inadequate time to perform the study procedures 5. Evidence of active athero-embolic disease 6. Known allergy to NAC 7. Severe asthma 8. Breast feeding 9. Severe peptic ulcer disease 10. Respiratory depression 11. >15% variability in S. Creat. over three days prior to angiography 12. Women of child bearing not using contraception | 1. Coronary Angiography ± PCI | 98 | Increase in S. Creat. ≥0.5 mg/dL (44 µmol/L) | Iohexol Low-Osm | 0.45% saline at 1ml/kg/hr for 12 hours pre-contrast, continuing for 12 hours post-procedure | NAC | 1200 mg orally 1 hour pre- and 3 hours post-contrast | | Mean ±SD: 77.4±35.9 | | | Mean S. Creat. ±SD:  2.2±0.4mg/dL | |
|  |  |  |  |  |  |  | Placebo | 1200 mg orally 1 hour pre- and 3 hours post-contrast | |  | | | Mean S. Creat. ±SD:  2.3±0.5mg/dL | |
| Dussol 2006(44) | Inclusion Criteria  1. Chronic renal failure (creatinine clearance 15-60 ml/min) 2. Radiological procedures necessitating a contrast medium injection   Exclusion Criteria   1. < 18-years 2. Women of child-bearing age not on contraception 3. Breast-feeding women 4. LVEF<30%, 5. Uncontrolled hypertension (BP >180/110mmHg) 6. Obvious extracellular over-hydration 7. Respiratory depression 8. Known prior intolerance to theophylline or furosemide 9. Previous exposure to contrast media in the 14 days before randomization 10. No informed consent | 1.Contrast Enhanced Imaging procedure | 258 | Increase in S. Creat. ≥0.5 mg/dL (44 µmol/L) above baseline at 48 h post-contrast | Low-Osm Contrast medium | 0.9% NaCl 15ml/kg/hr I.V. for 6h pre-procedure. 4th Group use oral Nacl only excluded from this analysis | Furosemide | 3mg/kg intravenously just after the procedure | | Mean ±SD: 119±42 | | | Mean S. Creat. ±SD:  201±81 mmol/L | |
|  |  |  |  |  |  |  | Theophylline | 5mg/kg one hour before the procedure | | Mean ±SD: 133±70 | | | Mean S. Creat. ±SD:  214±113 mmol/L | |
|  |  |  |  |  |  |  | Control | nil extra | | Mean ±SD: 115±57 | | | Mean S. Creat. ±SD:  208±84 mmol/L | |
| Dvorsak 2013(45) | Inclusion Criteria   1. Chronic Renal Impairment with stable S. Creat. >107 mmol/L   Exclusion Criteria   1. Regular vitamin C-containing medication 2. Acute renal failure 3. End-stage renal disease 4. Radiocontrast procedure in the last 3months 5. Cardiogenic shock 6. Acute MI | 1. Coronary Angiography ± PCI | 86 | >25% increase in S. Creat or >25% increase in S. Cystatin C from baseline, 3-4 days post-procedure | Iopamidol Iso-Osm | Normal saline 50–100 mL/h for 2 h pre- and for at least 6 h post-procedure | Ascorbic acid | 3g orally pre-procedure and 2g orally post-procedure and again the next morning | | Mean ±SD: 144.6±86 | | | Mean S. Creat. ±SD:  139.4±24 mmol/L | |
|  |  |  |  |  |  |  | Placebo | 3g orally pre-procedure and 2g orally post-procedure and again the next morning | |  | | | Mean S. Creat. ±SD:  133.3±30.9 mmol/L | |
| Efrati 2003(46) | Inclusion Criteria   1. S. Creat > 106µmol/L  2. Elective coronary angiography  Exclusion Criteria   1. Acute renal failure 2. Acute MI 3. Non-compensated CHF 4. Hemodynamic instability 5. Known sensitivity to contrast media | 1.Coronary Angiography | 51 | Increase of ≥25% in S. Creat. 96 hours after the procedure | Ultravist Low-Osm | 0.45% saline at 1ml/kg/hr for 12 hours pre-contrast, continuing for 12 hours post-procedure | NAC | Mucomyst 20% solution diluted in 30ml of regular Coca-Cola, as 1 g orally twice daily 24 hours pre- and 24 hours post-angiography | | Mean ±SD: 142±25.3 | | | Mean S. Creat. ±SD:  135.25±6.19 mmol/L | |
|  |  |  |  |  |  |  | Placebo | Regular Coca Cola alone orally twice daily 24 hours pre- and 24 hours post-angiography | | Mean ±SD: 138±33.7 | | | Mean S. Creat. ±SD:  131.7±6.19 mmol/L | |
| Er, F. 2012 | Inclusion Criteria  1.> 18 years of age  2. Presented with stable angina Pectoris with  4. Renal function test revealed impaired renal function (elevated serum creatinine of > 1.4 mg/dL or reduced eGFR <60 mL/min/1.73 m^2^,)  Exclusion Criteria:  Patients who do not meet inclusion criteria | 1.Coronary Angiography | 126 | Increase in serum creatinine≥25% or≥ 0.5 mg/dL above baseline at 48 hours after contrast medium exposure | Iohexol  Low-Osm |  | RIPC | Intermittent arm ischemia through 4 cycles of 5-minute inflation and 5-minute deflation of a blood pressure cuff started immediately before procedure. | | Mean ±SD: 124±44 | | | Mg/dl  1.63 (1.47–1.81) | |
|  |  |  |  |  |  |  | Placebo | Sham (RIPC)) | | Mean ±SD: 103±41 | | | Mg/dl  1.62 (1.39–1.93) | |
| Eric Chong 2015(188) | Inclusion Criteria:  adults >21 years of age with a glomerular filtration rate (GFR) of 15-60mL/min/1.73m2 and who were able to receive pre-hydration for 12 h  Exclusion Criteria:  1. End-stage renal failure with GFR of <15 mL/min/1.73 m2,  2. Acute renal failure with a >44 μmol/L increase in serum Cr levels in the previous 24 h,  3. Pre-existing dialysis, pulmonary oedema or moderate to severe congestive heart failure (New York Heart Association III–IV).  4. Inability to withstand the fluid load and presence of haemodynamic compromise, uncontrolled hypertension (untreated systolic blood pressure >160mmHg, or diastolic blood pressure >100mmHg)  5. Emergency cardiac catheterisation  6. Exposure to contrast or study drugs within the last 48  7. Allergies to contrast or trial medications  8. Clinical conditions requiring  continuous fluid therapy  9. Potentially renal-toxic  Drugs within 48 h of cardiac catheterisation and throughout the study | 1.Elective Cardiac catheterization | 331 | ≥25% increase of serum Cr concentration or a ≥44 μmol/L (0.5mg/dL) increase in serum Cr within 48 h of cardiac catheterisation or PCI [ | - | See Groups | NAC | | 154mEq/L sustained sodium chloride regime (1 mL/kg/h 12 h before, during and 6 h after the procedure) with oral NAC at 1.2 g bid for 3 days | Contrast(ml) Mean(SD):  116 (83.5) | | S. Creat.  Mean(SD):  134.0 (35.5) | | |
|  |  |  |  |  |  |  | NaHco3 | | 154 mEq/L abbreviated SOB regime at 3 mL/kg/h 1 h before the procedure, and 1mL/kg/h during and 6 h after the procedure | Contrast(ml) Mean(SD):  115 (85.4) | | S. Creat.  Mean(SD):  141.8 (44.8) | | |
|  |  |  |  |  |  |  | Combination (Excluded) | | combination | Contrast(ml) Mean(SD):  116 (84.5) | | S. Creat.  Mean(SD):  138.7 (36.6) | | |
| Erley 1999(48) | Inclusion Criteria:   1. Chronic renal insufficiency (S. Creat. >1.5 mg/dl )  2. Receive ≥ 80 ml low-osmolality contrast media (Iopromide)  Exclusion Criteria:  1. Allergy to contrast media or theophylline 2. Pregnancy 3. Uncontrolled hypertension 4. Severe heart failure 5. Liver failure 6. Nephrotic syndrome | 1.CT Digital Subtraction Angiography | 67 | Increase in S. Creat. ≥0.5 mg/dL (44 µmol/L) | Iopromide Low-Osm | All patients received hydration with 2–2.5 ml fluid (either orally as mineral water or I.V with 0.45% saline) starting at least 24 h before and continued for 24 hours post-contrast | Theophylline | 810 mg daily in divided doses, 270mg mané and 540mg tardé started 2 days before and continued until 3 days post-contrast | | Mean ±SD: 118±48 | | | Mean S. Creat. ±SD:  1.9±0.5mg/dL | |
|  |  |  |  |  |  |  | Placebo | Divided doses, mané and tardé started 2 days before and continued until 3 days post-contrast | | Mean ±SD: 110±44 | | | Mean S. Creat. ±SD:  1.7±0.4mg/dL | |
| Erol 2013(49) | Inclusion Criteria   1. S. Creat >1.1mg/dL 2. cardiac catheterization  Exclusion Criteria   1. Acute myocardial infarction requiring PCI within 24h 2. Cardiogenic shock 3. Acute renal failure 4. Current or planned peritoneal or hemo-dialysis 5. Previous administration of contrast agents or anticipated re-administration of contrast agents within the following 4 days. | 1.Coronary Angiography / PCI | 165 | Increase of ≥25% in S. Creat. 48-96 hours after the procedure | Iohexol Low-Osm | 1mg /kg/h saline for 12 h pre- and post-contrast | Allopurinol | 300mg orally 24 h before administration of contrast agent | | Mean ±SD: 121±25 | | | Median S. Creat. (IQR): 1.43(1.1-4.15) mg/dL | |
|  |  |  |  |  |  |  | Control | fluid only | | Mean ±SD: 119±26 | | | Median S. Creat. (IQR): 1.48(1.1-2.96) mg/dL | |
| Erturk 2014(50) | Inclusion Criteria   1. Age > 21years 2. eGFR <60ml/min/1.732  3. Elective intra-arterial procedure  Exclusion Criteria    1. Dialysis  2. eGFR <15ml/min/1.732 3. Uncontrolled HTN 4. Radiocontrast medium within 7 days of trial 5. Acute/chronic inflammatory disease 6. NSAID or Metformin up to 2 days before procedure 7. Pregnancy 8. Allergy to contrast or NAC 9. Patients receiving Fenoldopam, Mannitol, Dopamine and Theophylline | 1. Coronary Angiography 2. PCI 3. Peripheral intervention | 225 | >25% increase in S. Creat or >25% increase in S. Cystatin C from baseline, 48hours post-procedure |  | 0.9% normal saline for 12 hours pre- and 12 hours post-procedure | Intravenous NAC | 2400mg 1 hour pre-procedure and 4800mg 4-6 hours post-procedure **for this Analysis only I.V NAC included | | Mean ±SD: 122±67 | | | eGFR 30-59ml:  n=97  eGFR 15-29ml:  n=5 | |
|  |  |  |  |  |  |  | Oral NAC | 1200 mg orally every 12 hours for 24 hours pre- and 48 hours post-procedure | | Mean ±SD: 127±89 | | | eGFR 30-59ml:  n=95  eGFR 15-29ml:  n=7 | |
|  |  |  |  |  |  |  | IV hydration | normal saline only | | Mean ±SD: 127±66 | | | eGFR 30-59ml:  n=92  eGFR 15-29ml:  n=11 | |
| Eshraghi 2017(189) | Inclusion Criteria:  Patients with ST elevation MI and above 18 years of age who referred for emergency angioplasty  Exclusion Criteria:  1. Serum creatinine more  than 1.5 mg/dl  2. heart failure, history of end-stage renal failure or being on dialysis  3. Use of NAC, theophylline, aminoglycosides non-steroidal anti-inflammatory medicines  4. I.V contrast media administration within the last 2 days.  5. Pulmonary edema, multiple myeloma, and uncontrolled hypertension | 1.Emergency coronary angioplasty | 189 | 0.5 mg/dl or 25% increase in serum creatinine above the baseline, 48 hours after exposure to contrast media | Iodixanol  Iso-Osm | Normal saline 1-1.5 cc/kg was administered from 6 hours before to 6 hours after procedure | Pentoxifylline | | 400 mg 3 times a day from referral day until 24 hours after the procedure | Mean+SD:  190.88 ± 75.82 | | S. Creat.  Mean+SD:  1.147 ±0.424 | | |
|  |  |  |  |  |  |  | Control | | I.V hydration only | Mean+SD:  231.29 ± 105.10 | | S. Creat.  Mean+SD:  1.12 ±0.26 | | |
| Fan, Y 2016(190) | Inclusion Criteria:  Patients> 18 years with an estimated glomerular filtration rate (eGFR) of 60 mL/min or less undergoing elective cardiac catheterization  Exclusion Criteria:  1. cardiogenic shock  2. Aute ST-segment elevated MI requiring primary PCI  3. eft ventricular ejection fraction <30%  4. Allergy to contrast agent or nicorandil  5. Previous contrast media exposure within 1 week  6. Uraemia or renal failure receiving dialysis  7. Administration of NAC, metformin, or NaHco3 within 48 h of the procedure | 2.Elective coronary angiography | 269 | 25 % increase in serum creatinine from baseline or 44 µmol/L (0.5 mg/dL) increase in absolute value within 72 h after exposure to contrast medium | Ultravist  Low-Osm | 0.9 % saline at a rate of 1 mL/kg/h (0.5 mL/kg/h for patients with LVEF <40 %) at least 6 h before and 12 h after procedure | Nicorandil | | 10 mg three times daily from 2 days before to 3 days after contrast media exposure | Mean+SD:  145.3 ± 51.6 | | S. Creat.  Mean+SD:  123.55 ± 10.77 | | |
|  |  |  |  |  |  |  | Placebo | | Matching Placebo | Mean+SD:  149.2 ± 57.0 | | S. Creat.  Mean+SD:  122.99 ± 10.39 | | |
| Ferrario 2009(51) | Inclusion Criteria   1. Age >18 years  2. Elective coronary and/or peripheral angiography and/or angioplasty 3. Creatinine clearance <55ml/min with only ±10% variation in S. Creat. 3-30 days before the procedure.  Exclusion Criteria   1. NYHA symptoms class III to IV 2. Ongoing acute myocardial infarction 3. Acute coronary syndrome 4. Renal replacement therapy 5. Allergy to NAC 6. Need for theophylline, dopamine, fenoldopam, mannitol or nephrotoxic drugs within 1 week of the procedure 7. Presence of clinical signs of dehydration and systemic hypotension. | 1.Coronary / Peripheral Angiography ± Angioplasty | 214 | Increase in S. Creat. ≥0.5 mg/dL (44 µmol/L) or >25% increase above baseline within 3 days of contrast | Iodixanol Iso-Osm | Normal saline (0.9%) at 1ml/kg/h in the 12–24 h pre- and 24h post-procedure.  Oral clear fluid intake was not restricted pre- or post-procedure. | NAC | 600 mg orally every 12 hours on day pre- and day of procedure | | Mean ±SD: 180±104.4 | | | Mean Creat. Clearance±SD:  37±11.5 ml/min | |
|  |  |  |  |  |  |  | Placebo | Glucose tablet orally every 12 hours on day pre- and day of procedure | | Mean ±SD: 168±103.3 | | | Mean Creat. Clearance±SD:  40±9.3 ml/min | |
| Firouzi 2012(52) | Inclusion Criteria   1.Non-emergent coronary angiography and Intervention  Exclusion Criteria   1. S. Creat. >2mg/dl 2. Acute MI 3. Unstable coronary syndrome 4. Cardiogenic shock 5. End-stage renal failure  6. Dialysis 7. Use of NAC 8. History of intravenous contrast media administration within the previous 10 days | 1.Angiography / PCI | 318 | Increase in S. Creat. ≥0.5 mg/dL (44 µmol/L) or >25% increase above baseline within 48 hours of contrast | Iodixanol Iso-Osm Iopromide Iso-Osm | Normal saline 1–1.5 cc/kg from 6 h before to 6 h after procedure | Pentoxyfylline | 400 mg three times a day from 24 h pre- to 24h post-procedure | | Mean ±SD: 319.28±98.1 | | | Mean S. Creat. ±SD:  1.17±0.22mg/dL | |
|  |  |  |  |  |  |  | Control | IV hydration only | | Mean ±SD: 325.34±101.41 | | | Mean S. Creat. ±SD:  1.21±0.24mg/dL | |
| Firouzi 2015(53) | Inclusion Criteria   1. Acute MI 2. Age >20 years  Exclusion Criteria   1. S. Creat. > 2mg/dl 2. Cardiogenic shock 3. LVEF <40% 4. End-stage renal failure 5. Dialysis 6. N-acetylcysteine use 7. History of intravenous contrast media administration within 10 days 8. Metabolic disorder with impairment of serum magnesium level. | 2.PCI | 152 | Increase in S. Creat. ≥0.5 mg/dL (44 µmol/L) or >25% increase above baseline within 48 hours of contrast | Iso-Osm Iodixanol or  Low-Osm iopromide | Normal saline infusion 1–1.5 cc/kg from start of procedure until 6 h post-procedure | MgSO4 | 1 g just before the procedure | | Mean ±SD: 319.28±98.1 | | | Mean S. Creat. ±SD:  1.17±0.22mg/dL | |
|  |  |  |  |  |  |  | Control | IV hydration only | | Mean ±SD: 325.34±101.41 | | | Mean S. Creat. ±SD:  1.21±0.24mg/dL | |
| Fung 2004(54) | Inclusion Criteria   1. Moderate to severe renal insufficiency (S. Creat 1.69-4.52 mg/dL) 2. Elective coronary angiography and/or intervention  Exclusion Criteria    1. Cardiogenic shock 2. Current dialysis therapy 3. Use of dopamine, theophylline, or mannitol 4. Allergy to NAC or contrast agents | 1.Coronary Angiography / PCI | 105 | Increase in S. Creat. ≥0.5 mg/dL (44 µmol/L) or > 25% reduction in eGFR 48 hours of contrast | Iopromide Low-Osm | Normal saline at 100 mL/h from 12 hours pre- until 12 hours post-procedure | NAC | 400 mg, thrice daily the day pre- and day of contrast procedure | | Mean ±SD: 135.8±66.6 | | | Mean S. Creat. ±SD:  2.27±0.54mg/dL | |
|  |  |  |  |  |  |  | Control | IV hydration only | | Mean ±SD: 121±66.2 | | | Mean S. Creat. ±SD:  2.37±0.61mg/dL | |
| Gare 1999(55) | Inclusion Criteria   1. Mild-moderate chronic renal failure AND/OR  2. Diabetes Mellitus   Exclusion Criteria   1. Severe renal insufficiency (S. Creat. >200 µmol/l) 2. Acute coronary events (MI or unstable angina) <48h pre-procedure 3. Known intolerance to dopamine 4. Allergy to contrast material 5. Pheochromocytoma | 1.Coronary angiography | 72 | Increase in S. Creat. by 40% from baseline after the injection of the radiocontrast agents | Lopromide Low-Osm | 0.45% Saline/D5W for 8-12h pre-and 36-48h post-angiography at 100 ml/h (increased depending on urinary output). | Dopamine | 120ml of 0.9% saline plus 2mg/kg dopamine per day for 48h | | Mean ±SD: 173.8±13 | | | Mean S. Creat. ±SD:  100.3±5.4 mmol/L | |
|  |  |  |  |  |  |  | Control | IV hydration only | | Mean ±SD: 163.2±13.2 | | | Mean S. Creat. ±SD:  100.6±5.2 mmol/L | |
| Geng 2012(56) | Inclusion Criteria   1. Type 2 DM 2. eGFR ≤60 mL/min   Exclusion Criteria  1. Allergy to contrast media 2. Contraindication for Anisodamine 3. Emergency Cardiac Catheterization 4. Congestive Heart Failure 5. Intra-Aortic Balloon Counter-pulsation 6. Dialysis 7. Exposure to Contrast media within the last 7 days | 1.Coronary Angiography / PCI | 299 | Increase in S. Creat. ≥0.5 mg/dL (44 µmol/L) or >25% increase above baseline within 3 days of contrast | Ultravist Low-Osm | Saline at 1ml/kg/hr 12 hours pre- and 12 hours post-procedure | Anisodamine | 0.2 µg/kg/min 12 hours pre- and continued for 12 hours post-procedure | | Mean ±SD: 119±25 | | | Mean S. Creat. ±SD:  120.9±19.2 mmol/L | |
|  |  |  |  |  |  |  | Control | IV hydration only | | Mean ±SD: 124±23 | | | Mean S. Creat. ±SD:  122±19.5 mmol/L | |
| Goldenberg 2004(57) | Inclusion Criteria   1. Chronic renal insufficiency (S. Creat. ≥1.5mg/dl or creatinine clearance <50ml/min)  Exclusion Criteria   1. Acute renal failure 2. Acute MI requiring primary or rescue PCI within 12hours 3. Cardiogenic shock 4. Current peritoneal or haemodialysis 5. Planned post-contrast dialysis 6. Known allergy to acetylcysteine | 1.Coronary Angiography / PCI | 87 | Increase in S. Creat. ≥0.5 mg/dL (44 µmol/L) within 48 hours | Iopamidol Low-Osm | 0.45% Saline at 1ml/kg/hr 12 hours pre- and 12 hours post-procedure | NAC | 600 mg orally thrice daily for 2 days, starting 24h pre-procedure | | Mean ±SD: 111±43 | | | Mean S. Creat. ±SD:  2±0.4mg/dL | |
|  |  |  |  |  |  |  | Placebo | placebo orally thrice daily for 2 days, starting 24h pre-procedure | | Mean ±SD: 121±49 | | | Mean S. Creat. ±SD:  1.9±0.3mg/dL | |
| Gomes 2005(59) | Inclusion Criteria   1. S. Creat > 106.08mol/l, creatinine clearance <50ml/min, or drug treated diabetes mellitus 2. Elective intervention   Exclusion Criteria   1. Age <18 years 2. Use of contrast media within 21 days of randomisation 3. Current dialysis 4. Haemodynamic instability before the procedure (SBP ≤ 90 mm Hg or DBP≤ 60 mm Hg) 5. History of sensitivity to N-acetylcysteine. | 1.Coronary Angiography / PCI | 172 | Increase in S. Creat. ≥0.5 mg/dL (44 µmol/L) within 48 hours | Ioxaglate Low-Osm | 0.9% Saline at 1ml/kg/hr 12 hours pre- and 12 hours post-procedure | NAC | 600 mg orally twice daily for 2 days, starting 24h pre-procedure (2 doses pre- and 2 doses post-procedure) | | Mean ±SD: 102.5±47.3 | | | Mean S. Creat. ±SD:  123.76±45.08 mmol/L | |
|  |  |  |  |  |  |  | Placebo | placebo orally twice daily for 2 days, starting 24h pre-procedure (2 doses pre- and 2 doses post-procedure) | | Mean ±SD: 102.8±60.4 | | | Mean S. Creat. ±SD:  111.38±30.94 mmol/L | |
| Gomes 2012(58) | Inclusion Criteria   1. S. Creat. ≥ 1.2 mg/dL or eGFR < 50mL/min  Exclusion Criteria   1. Age < 18 years 2. Use of contrast media during the last 21 days 3. History of dialysis 4. Cardiac insufficiency (NYHA III-IV) 5. Emergency procedures. | 1.Coronary Angiography / PCI | 319 | Increase in S. Creat. ≥0.5 mg/dL (44 µmol/L) within 48 hours | Ioxaglate Low-Osm | IV bolus of assigned fluid at 3 mL/kg/h for 1 hour immediately pre-contrast.  IV hydration to continue at 1mL/kg/hr during and for 6 hours post-procedure | NaHCO3 | 154 mEq/l in D5W | | Mean ±SD: 124±65 | | | Mean S. Creat. ±SD:  1.5±0.4mg/dL | |
|  |  |  |  |  |  |  | NaCl | 154 mEq/l in D5W | | Mean ±SD: 125±87 | | | Mean S. Creat. ±SD:  1.49±0.5mg/dL | |
| Gu 2013(60) | Exclusion Criteria   1. NYHA stage IV heart failure | 1.Coronary Angiography / PCI | 859 | Increase in S. Creat. ≥0.5 mg/dL (44 µmol/L) or >25% increase above baseline within 48 hours of contrast |  | 1/ml/kg/h saline for 4 hr pre- and for 24 hr post-procedure | Furosemide | 20mg over 30 seconds | | Mean ±SD: 100±40 | | | Mean S. Creat. ±SD:  91±21.3 mmol/L | |
|  |  |  |  |  |  |  | Control | IV hydration only | | Mean ±SD: 100±40 | | | Mean S. Creat. ±SD:  89.2±17.6 mmol/L | |
| Gulel 2005(61) | Inclusion Criteria   1. Elective coronary intervention 2. Chronic renal impairment (stable S. Creat. > 1.3 mg/dl)   Exclusion Criteria   1. Acute renal failure  2. End stage Renal failure on regular dialysis 3. Clinically evident heart failure  4. Allergy against contrast medium 5. Serious hepatic dysfunction 6. Planned for percutaneous coronary intervention | 1.Diagnostic Coronary Angiography | 55 | Increase in S. Creat. ≥0.5 mg/dL (44 µmol/L) within 48 hours | Ioxaglat Low-Osm | 0.9% Saline at 1ml/kg/hr 12 hours pre- and 12 hours post-procedure | NAC | 600 mg orally twice daily for 2 days, starting 24h pre-procedure | |  | | | Mean S. Creat. ±SD:  1.6±0.4mg/dL | |
|  |  |  |  |  |  |  | Control | IV hydration only | |  | | | Mean S. Creat. ±SD:  1.8±0.6mg/dL | |
| Gunebakmaz 2012(62) | Inclusion Criteria   1. Patients scheduled for coronary angiography and ventriculography 2. S. Creat. ≥1.2 mg/dL  Exclusion Criteria   1. Dialysis 2. Recent exposure to contrast media or a nephrotoxic agent within 7 days before the study 3. Urgent PCI 4. Requiring loop diuretics, theophylline, aminophylline or dopamine throughout the study 5. Hemodynamically unstable patients 6. Contraindications for β-blocker prescription. | 1.Coronary Angiography / PCI | 148 | Increase in S. Creat. ≥0.5 mg/dL (44 µmol/L) or >25% increase above baseline within 2-5 days of contrast | Iopromide Low-Osm | Intravenous isotonic saline at 1mL/kg/hour, for 6 hours pre- and 12 hours post-procedure | NAC | 600 mg orally twice daily for 4days, 4 doses pre-procedure, 2 doses day of procedure and 2 doses day post-procedure | | Mean: 63.4 | | | Mean S. Creat. ±SD:  1.42±0.13mg/dL | |
|  |  |  |  |  |  |  | Nebivolol | 5mg every 24 hours for 4 days: 2 doses pre-procedure, 1 dose day of procedure, and 1 dose day post- procedure | | Mean: 61.8 | | | Mean S. Creat. ±SD:  1.4±0.11mg/dL | |
|  |  |  |  |  |  |  | Control | IV hydration only | | Mean: 64.2 | | | Mean S. Creat. ±SD:  1.43±0.14mg/dL | |
| Gupta 1999(63) | Inclusion Criteria   1.Diabetes Mellitus    Exclusion Criteria  1. S. Creat. >6mg/dL 2. Patients already on ACEI 3. Contraindication to ACEI 4. LVEF < 30% 5. Known allergy to contrast media | 1.Coronary Angiography | 83 | Increase in S. Creat. ≥0.5 mg/dL (44 µmol/L) within 24 hours |  | Dextrose saline (with insulin) at 1ml/kg/hour started 3 hours pre- and continued for 6 hours post-procedure | Captopril | 25mg po q8h started 1h pre-contrast, continued for 3 days | | Mean ±SD: 116.6±11.4 | | | Mean S. Creat. ±SD:  1.38±0.27mg/dL | |
|  |  |  |  |  |  |  | Control | IV hydration only | | Mean ±SD: 118.4±9.3 | | |  | |
| Hafiz 2012(64) | Inclusion Criteria   1. Chronic kidney disease (S. Creat. >1.6mg/dl in non-diabetics and >1.4 mg/dl in diabetics) or an estimated glomerular filtration rate (eGFR) of <50 ml/min/1.73 m2  2. Age>18 years   Exclusion Criteria   1. Dialysis 2. Unstable renal function (>0.4 mg/dl change in S. Creat. 48 hr pre-procedure) 3. Pulmonary oedema 4. S. bicarbonate >34 mmol/L 5. Received Fenoldopam, Mannitol, Dopamine, or NAC within 48 hr pre-procedure 6. Cardiogenic shock 7. Allergic to contrast media 8. Pregnancy 9. Unable to provide informed consent. | 1. Coronary Angiography 2. PCI 3. Peripheral intervention | 176 | Increase in S. Creat. ≥0.5 mg/dL (44 µmol/L) or >25% increase above baseline within 48 hours of contrast | Iodixanol Iopamidol Ioversol All are: Low-Osm | As per intervention protocol | NaCl | Normal Saline 1ml/kg/hr for 12h pre- and 12h post-procedure | | Median (IQR): 100 (80-140) | | | Mean S. Creat.: 1.6mg/dL | |
|  |  |  |  |  |  |  | NaHCO3 | D5W containing 154 mEq/L of NaHCO3 at 3ml/kg/hr for 1 hr pre-procedure, 1ml/kg/hr for 6 hr post-procedure | | Median (IQR): 110 (75-155) | | | Mean S. Creat.: 1.7mg/dL | |
| Han  2014(65) | Inclusion Criteria   1. Type 2 DM 2. Chronic Kidney disease (stage 2 or 3)   Exclusion Criteria   1. Hypersensitivity to contrast medium or statins 2. Type 1 DM 3. Ketoacidosis 4. Lactic acidosis 5. CKD stages 0/1/4/5 6. Acute STEMI within preceding 4 weeks 5. NYHA Class IV heart failure 6. Hemodynamic instability 7. Administration of iodinated contrast medium during the 2 weeks before randomization 8. LDL Cholesterol <1.82mmol/L 9. Hepatic dysfunction (S ALT >3xnormal) 10. Thyroid insufficiency 11. Renal artery stenosis (unilateral>70% or bilateral >50%) | 1. Coronary Angiography 2. PCI 3. Peripheral intervention | 3095 | Increase in S. Creat. ≥0.5 mg/dL (44 µmol/L) or >25% increase above baseline within 72 hours of contrast | Iodixanol Iso-Osm | 0.9% saline at 1 ml/kg/h from 12h pre- until 24h post-procedure (physician’s discretion allowed) | Statin | Rosuvastatin 10mg tardé from 2 days pre- until 3 days post-procedure (total dose 50 mg over 5 days) | | Median (IQR): 120 (100-200) | | |  | |
|  |  |  |  |  |  |  | Control | no statin | | Median (IQR): 110 (100-200) | | | Mean S. Creat. ±SD:  94.95±20.84 mmol/L | |
| Hashemi 2005(66) | Exclusion Criteria   1. Amount of contrast used <100 or >300 mls 2. Calcium antagonists, ACE-I, theophylline prescribed within 2 days before the procedure 3. Baseline S. Creat. > 2mg/dl. | 1.Coronary Angioplasty | 98 | Increase in S. Creat. ≥0.5 mg/dL (44 µmol/L) within 48 hours | Meglumin compound (76%, DarooPakhsh, Iran) (370 mg/ 20 ml) | Normal saline 0.9% infusion (total volume of 1.5L) at 60 ml/hr from 12hr pre- until 12 hr post-procedure. | Captopril | 12.5mg po q8h started 2h pre-contrast, continued for 48hr | | Mean ±SD: 225±120 | | | Mean S. Creat. ±SD:  0.98±0.43mg/dL | |
|  |  |  |  |  |  |  | Placebo | placebo po q8h started 2h pre-contrast, continued for 48hr | | Mean ±SD: 223.3±130 | | | Mean S. Creat. ±SD:  1.05±0.39mg/dL | |
| Healy, D 2015(191) | Inclusion Criteria:  Patients aged over 17 years scheduled for abdomino-pelvic CECT-scans who were likely to remain in hospital for at least two days after the scan  Exclusion Criteria:  1. Allergy or hypersensitivity to iodinated contrast  2. Hospital admission SCr >150 µmol/dL (a contraindication to iodinated contrast)  3. Renal transplant  4. History of acute renal failure that required management by a nephrologist  5.Current use of either sulphonlyurea or nicorandil. | 1.Contrast enhanced CT | 98 | increase of >25% or an increase of ≥0.5 mg/dl in SCr from the baseline value 48 h after exposure to the contrast medium | Iohexol  Low-Osm  Iopamidol  Iso-Osm  Iodixanol  Iso-Osm | Use of hydration prior to the procedure was at the discretion of the physician who ordered the scan. | RIPC | 40 minutes before contrast was given five-minute cycles of arm ischemia with three minutes of reperfusion between by inflating blood pressure cuff positioned on the patient’s arm to a pressure of 200 mmHg or 15 mmHg above systolic pressure if that was >200 mmHg | | Most patients receive a dose of 90 mL, but patients heavier than 110 kg may receive 120 mL. All patients with eGFR <60 mL/min/1.73m2 receive iodixanol | | | Median (IQR)  73 (59, 85) | |
|  |  |  |  |  |  |  | Control | No intervention | |  | | | Median (IQR)  75 (62, 85) | |
| Heguilen 2013(67) | Inclusion Criteria   1. Age>18 years 2. Stable S. Creat. ~1.25 mg/dl (110 lmol/l) 3. eCr. Clearance <45 ml/min   Exclusion Criteria   1. S. Creat. 4.5 mg/dl ([364.5 lmol/l) 2. Change in S. Creat. ≥0.5 mg/dl (44.2 lmol/l) within the previous week 3. Emergency catheterization 4. Pulmonary oedema 5. Pre-existing dialysis 6. Recent exposure to contrast media 7. Multiple Myeloma 8. Uncontrolled hypertension 9. Hemodynamic instability 10. Current treatment with dopamine, mannitol, Fenoldopam, Aminophylline, Theophylline, Ascorbic acid or NAC 11. Pregnant or childbearing women 12. Known hypersensitivity to Contrast media or NAC 13. Patients undergoing interventions limited to the major renal vasculature | 1.Contrast Study including cardiac catheterization or arteriography | 101 | >25% increase above baseline S. Creat. within 48 -72 hours of contrast | Ioversol Low-Osm | As per intervention protocol | NaHCO3 | D5W containing 154 mEq/L of NaHCO3 at 3ml/kg/hr for 2 hr pre-procedure, 1ml/kg/hr during and for 6-12 hr post-procedure | | Mean ±SD: 179.8±14.2 | | | Mean S. Creat. ±SD:  1.57±0.03mg/dL | |
|  |  |  |  |  |  |  | NAC | 600 mg orally twice daily day preceding and day of procedure | | Mean ±SD: 186.6±15.4 | | | Mean S. Creat. ±SD:  1.60±0.07mg/dL | |
|  |  |  |  |  |  |  | Both NAC + NaHCO3 | Excluded from analysis | | | | | | |
| Heng 2008(68) | Inclusion Criteria   1. Stable CRF (eGFR <56ml/min and S. Creat variability <0.1mg/dl between levels measured 1-2 months before procedure and the baseline level taken within 24 hours before intervention  Exclusion Criteria   1. Age <18 years 2. Pregnancy 3. Allergy to NAC 4. Dialysis 5. Acute Renal Failure 6. Haemodynamic instability 7. Overt Congestive cardiac failure 8. Administration of Iodinated Contrast media, nephrotoxic agent or NAC within the last 30 days prior to inclusion 9 Patients on nephrotoxic agents such as NSAIDs or aminosides | 1.Coronary Angiography / PCI | 65 | Increase in S. Creat. ≥0.5 mg/dL (44 µmol/L), >25% increase S. Creat. above baseline or >5ml decrease in eGFR within 48 hours of contrast | Iomeprol Low-Osm Iodixanol Iso-Osm | Bicarbonate saline 1.4% I.V at 1ml/kg/hr for 12 hours pre- and 12hr post-procedure (6 hours for patients with heart failure) | NAC | 1200 mg orally twice daily day preceding and day of procedure | | Mean ±SD: 208±70 | | | Mean S. Creat. ±SD:  178±53mmol/L | |
|  |  |  |  |  |  |  | Placebo | placebo orally twice daily day preceding and day of procedure | | Mean ±SD: 198±76 | | | Mean S. Creat. ±SD:  193±76mmol/L | |
| Hoole 2009(69) | Inclusion Criteria   1. Age ≥18 years  2. Elective PCI 3. Able to give informed consent  Exclusion Criteria   1. Emergency PCI 2. Elevation of cTnI pre-PCI 3. Women of child-bearing age 4. Nicorandil or glibenclamide use  5. Severe comorbidity (estimated life expectancy <6 months) | 1.PCI | 218 | >25% increase above baseline S. Creat. within 24 hours of contrast | Iopromide Low-Osm |  | RIPC | Blood pressure cuff placed around their non-dominant upper arm. The cuff inflated to 200-mm Hg pressure for 5 minutes, followed by 5 minutes of deflation and repeated 2 more times to 3 cycles in total | | Mean ±SD: 196.7±80.1 | | | eGFR, median (IQR):  72.0 (55.8–86.0) | |
|  |  |  |  |  |  |  | Placebo | Placebo Group: similar cuff placed around the upper arm, but it was not inflated | | Mean ±SD: 187.5±74.2 | | | eGFR, median (IQR):  75.5 (65.3–89.8) | |
| Hsu 2012(70) | Inclusion Criteria   1. Adult patients undergoing abdominal or chest contrast-enhanced CT in Emergency Department  Exclusion Criteria   1. long-term haemodialysis 2. Peritoneal dialysis 3. Received another dose of contrast medium within 72 hours 4. Refusal to sign consent forms 5. Known allergy to NAC | 1.Contrast Enhanced CT | 241 | Increase in S. Creat. ≥0.5 mg/dL (44 µmol/L) or >25% increase above baseline within 48-72 hours of contrast | Iohexol Iobitridol Iopromide All are: Low-Osm | 3 mL/kg/h of 0.9% NaCl for 1hr pre-CT, continued at 1mL/kg/hr for 6hr post-CT (decreased to 0.5mL/kg/hr in patients with clinical evidence of CHF) | Intravenous NAC | 600 mg of NAC in 0.9% sodium chloride at 3 mL/kg for 1hr pre-contrast | | Mean ±SD: 91.1±10.0 | | | Mean S. Creat. ±SD:  1.40±0.58mg/dL | |
|  |  |  |  |  |  |  | Control | IV hydration only | | Mean ±SD: 88.1±10.0 | | | Mean S. Creat. ±SD:  1.26±0.43mg/dL | |
| Huber 2003(71) | Inclusion Criteria   1. Chronic renal insufficiency (S. Creat. ≥1.3mg/dl) undergoing coronary angiography   Exclusion Criteria   1. Pregnancy 2. Contraindications to theophylline | 1.Coronary Angiography | 112 | Increase in S. Creat. ≥0.5 mg/dL (44 µmol/L) within 48 hours | Iomeprol Low-Osm | 100ml Nacl 30 minutes before coronary angiography, fluid intake ≥2 L/day was advised | Theophylline | 200 mg IV 30 minutes before coronary angiography | | Mean ±SD: 196.5±84.1 | | | Mean S. Creat. ±SD:  1.65±0.41mg/dL | |
|  |  |  |  |  |  |  | Placebo | IV hydration only | | Mean ±SD: 216.6±95.0 | | | Mean S. Creat. ±SD:  1.72±0.69mg/dL | |
| Inda-Filho 2014(72) | Inclusion Criteria  1. Age> 18 years 2. Elective coronary angiography or ventriculography   Exclusion Criteria   1. Received an iodinated contrast medium intravascularly within 30 days pre-procedure  2. Emergency coronary catheterization 3. Pulmonary oedema 4. Acute decompensated congestive heart failure 5. On nonsteroidal anti-inflammatory drugs or metformin at the time of the study 6. Declined to participate | 1.Angiography / Ventriculography | 561 | Increase of ≥ 0.3mg in or absolute value ≥1.1mg/dL of S. Creat. at 72 hours | Ioxitalamate Hi-Osm | All patients receive Nacl (0.9%, isotonic) given intravenously at 1 mL/kg/h. Patients received medications intravenously 60 minutes immediately before, during, and 6 hours immediately after contrast | Intravenous NAC | NAC in D5W at 150mg/kg/hr as bolus prior to contrast, then at 50 mg/kg/h | | Mean ±SD: 21±16.7 | | | Mean S. Creat. ±SD:  1.00±0.25mg/dL | |
|  |  |  |  |  |  |  | NaHCO3 | 150 mEq of 8.4% NaHCO3 in 1L 5% dextrose given as bolus at 3.5ml/kg/hr pre-contrast, then at 1.18ml/kg/hr | | Mean ±SD: 22±17.6 | | | Mean S. Creat. ±SD:  1.00±0.24mg/dL | |
|  |  |  |  |  |  |  | Control | IV hydration only | | Mean ±SD: 28±22.4 | | | Mean S. Creat. ±SD:  1.04±0.41mg/dL | |
|  |  |  |  |  |  |  | Both NAC + NaHCO3 | Excluded from analysis | | | | | | |
| Izani 2008(192) | Inclusion Criteria:  Patients electively admitted for coronary angiography > 18 yaers with creatinine clearance 40-90ml/min  Exclusion Criteria:  1. Severe renal failure  2. Severe peptic ulcer  3. Allergy to NAC or Asthma  4. Pregnancy or breast feeding  5. | 1.Coronary Angiography ± PCI | 108 | 25% increase in serum creatinine above the baseline, 48 hours after exposure to contrast media | Iohexol  Low-Osm | 0.45% normal saline 12 hours before contrast start and after at rate of 1ml/kg/h | NAC | 600mg BD for 4 days started 12 hours before contrast administration | | Contrast(ml) Mean(SD):  136.73(100.23) | | | S. Creat.  Mean(SD):  123.7(17.08) | |
|  |  |  |  |  |  |  | Control | Only I.V saline | | Contrast(ml) Mean(SD):  126.67(94.37) | | | S. Creat.  Mean(SD):  124.4(21.89) | |
| Jaffery 2012(74) | Inclusion Criteria   1. Diagnosis of ACS 2. Age >18years   Exclusion Criteria   1. End-stage renal disease (ESRD) requiring dialysis 2. Known hypersensitivity to NAC 3. History of life-threatening contrast reaction. | 1.Coronary Angiography / PCI | 456 | >25% increase above baseline S. Creat. within 72 hours of contrast | Iodixanol Iso-Osm | Additional intravenous normal saline (0.9% NaCl in water) was administered such that the total volume of fluid infused was equal to 1 cc/kg/hr for 24 hr | Intravenous NAC | 1,200 mg bolus followed by 200 mg/hr for 24 hr | | Mean ±SD: 169.5±94.5 | | | Mean S. Creat. ±SD:  1.09±0.4mg/dL | |
|  |  |  |  |  |  |  | Placebo | matched placebo | | Mean ±SD: 161.3±83.4 | | | Mean S. Creat. ±SD:  1.07±0.4mg/dL | |
| Jo  2013(75) | Inclusion Criteria   1. Age >19 years  2. Creat. clearance ≤ 60ml/min  3. Elective procedure  Exclusion Criteria   1. Acute Coronary Syndrome 2. Cardiogenic shock 3. Pulmonary oedema 4. Emergency Coronary angiography 5. Acute renal Failure 6. End Stage Renal Disease requiring Dialysis 7. Previous Exposure to Contrast Medium within seven days of study 8. Pregnancy 9. Lactation 10. Hypersensitivity to Contrast media 11. Mechanical ventilation 12 Parenteral Use of Diuretics 13. Multiple Myeloma 14. Use of Metformin or Non-steroidal anti-inflammatory drugs within 48 hours of procedure | 1.Coronary Angiography / PCI | 212 | Increase in S. Creat. ≥0.5 mg/dL (44 µmol/L) or >25% increase above baseline | Iodixanol Iso-Osm Ipromide Low-Osm Iobitridol Low-Osm | Isotonic Normal Saline at 1ml/kg/hr for 12 hours pre- and 12 hours post-procedure | Alpha Lipoic Acid | 600 mg orally 8-hourly commenced day of procedure, up to a total of 3600mg | | Mean ±SD: 165.2±83.6 | | | Mean S. Creat. ±SD:  120.8±69.8mmol/L | |
|  |  |  |  |  |  |  | Control | IV hydration only | | Mean ±SD: 174.6±103.6 | | | Mean S. Creat. ±SD:  108.2±37.5mmol/L | |
| Jo  2008(76) | Inclusion Criteria   1. Age >19 years  2. Baseline S. Creat. ≥1.1mg/dL or Creat. clearance ≤ 60ml/min   Exclusion Criteria   1. Acute Coronary Syndrome 2. Cardiogenic shock 3. Pulmonary oedema 4. Emergency Coronary angiography 5. Acute renal Failure 6. End Stage Renal Disease requiring Dialysis 7. Previous Exposure to Contrast Medium within seven days of study 8. Pregnancy 9. Lactation 10. Hypersensitivity to Contrast media 11. Mechanical ventilation 12 Parenteral use of diuretics Use of NAC, Ascorbic acid, metformin or nonsteroidal anti-inflammatory drugs within 48 hours of the procedure 13. Multiple Myeloma 14. Statin use within 30 days of procedure | 1.Coronary Angiography | 243 | Increase in S. Creat. ≥0.5 mg/dL (44 µmol/L) or >25% increase above baseline | Iodixanol Iso-Osm | Half-isotonic Normal Saline at 1ml/kg/hr for 12 hours pre- and 12 hours post-procedure | Simvastatin | 40mg twice daily for 2 days, twice pre- and twice post-angiography to total 160mg | | Mean ±SD: 173.3±99.3 | | | Mean S. Creat. ±SD:  1.285±0.409mg/dL | |
|  |  |  |  |  |  |  | Placebo | placebo twice daily for 2 days, twice pre- and twice post-angiography to total 160mg | | Mean ±SD: 190.9±133.5 | | | Mean S. Creat. ±SD:  1.234±0.358mg/dL | |
| Jo  2009(77) | Inclusion Criteria   1. Age >19 years  2. Baseline S. Creat. ≥1.1mg/dL or Creat. clearance ≤ 60ml/min   Exclusion Criteria   1. Acute Coronary Syndrome 2. Cardiogenic shock 3. Pulmonary oedema 4. Emergency Coronary angiography 5. Acute renal Failure 6. End Stage Renal Disease requiring Dialysis 7. Previous Exposure to Contrast Medium within seven days of study 8. Pregnancy 9. Lactation 10. Hypersensitivity to Contrast media 11. Mechanical ventilation 12 Parenteral use of diuretics Use of NAC, Ascorbic acid, metformin or nonsteroidal anti-inflammatory drugs within 48 hours of the procedure 13. Multiple Myeloma | 1.Coronary Angiography | 179 | Increase in S. Creat. ≥0.5 mg/dL (44 µmol/L) or >25% increase above baseline within 48 hours of contrast | Iodixanol Iso-Osm | Half-isotonic Normal Saline at 1ml/kg/hr for 12 hours pre- and 12 hours post-procedure | NAC | 1,200 mg orally twice daily, twice pre- and twice post-angiography, to a total of 4,800 mg | | Mean ±SD: 203.6±141.9 | | | Mean S. Creat. ±SD:  1.38±0.52mg/dL | |
|  |  |  |  |  |  |  | Ascorbic acid | 5mg pre-procedure in divided doses of 3g and 2g with 12h interval, two doses of 2g at 12h intervals post-procedure | | Mean ±SD: 216.4±136.1 | | | Mean S. Creat. ±SD:  1.27±0.35mg/dL | |
| Jurado-Roman  2015(78) | Inclusion Criteria   1. Patients with STEMI undergoing primary PCI   Exclusion Criteria   1. End-stage renal failure requiring dialysis 2. Cardiac arrest 3. Severe heart failure (Killip III to IV) | 1.PCI | 473 | Increase in S. Creat. ≥0.5 mg/dL (44 µmol/L) or >25% increase above baseline within 3 days of contrast | Iodixanol Iso-Osm | As per intervention protocol | IV hydration | Isotonic Normal Saline at 1ml/kg/hr from start of procedure continued for 24h | | Mean: 180 | | | Mean GFR ±SD: 90±21  mg/min/1.73m2 | |
|  |  |  |  |  |  |  | Control | no hydration | | Mean: 173 | | | Mean GFR ±SD: 88±54  mg/min/1.73m2 | |
| Kai, Z 2015(193) | Inclusion Criteria:  Patients > 18 years and < 80 years with Type 2 diabetes whose estimated glomerular filtration rate  (eGFR) was ≤ 60 mL/min·1.73m2  Exclusion Criteria:  1. Hyperpyrexia or allergic to iodine  2. Tumors  3. Severe heart failure  4. Severe kidney failure  5. Severe liver failure  6. Disorders of the immune system  7. Blood diseases. | 1.Coronary angiograph | 89 | Relative (≥ 25%) or an absolute (≥ 44.2 μmol/L) increase in serum creatinine from the baseline value within 3 days after intravascular administration of contrast medium | Iopamidol  Low-Osm | intravenous isotonic saline (0.9%) at a rate of approximately 1 mL/kg per hour for 6 h before, and 12 h after, contrast exposure | Standard Cordyceps | | 2-g corbrin capsules, 3 times/d, 3 days before and after procedure | | Mean+SD:  248.87±48.69 | | | S. Creat.  Mean+SD:  121±19 |
|  |  |  |  |  |  |  | Control  (basic treatment group) | | No Cordyceps | | Mean+SD:  246.85±49.74 | | | S. Creat.  Mean+SD:  122±19 |
|  |  |  |  |  |  |  | Intensive Cordyceps  This group was not analysed | | 3-g corbrin capsules, 3 times/d, 3 days before and after procedure | | Mean+SD:  250.87±50.72 | | | S. Creat.  Mean+SD:  122±19 |
| Kama 2014(79) | Inclusion Criteria   1.Patients who has CE-CT in Emergency Department 2. Age> 18 years 3. Mehran risk score for CI-AKI moderate and high-risk groups (>5 points)   Exclusion Criteria   1. Low risk for CI-AKI 2. Allergy to contrast 3. Hemodynamically unstable requiring excessive fluid resuscitation or surgery 4. Renal replacement therapy 5. Did not provide informed consent | 1.CE-CT | 123 | Increase in S. Creat. ≥0.5 mg/dL (44 µmol/L) or >25% increase above baseline within 48-72 hours of contrast | Low-Osm Iohexal All Patients get Less than 100 ml volume | 1,000 mL of 0.9% NaCl at a rate of 350 mL/hour | Nacl | 150 mg/kg 1L 0.9% NaCl at 350 mL/hour | |  | | | Median S. Creat. (IQR): 1.62 (1.4-2.07) mg/dL | |
|  |  |  |  |  |  |  | Control | IV hydration only | |  | | | Median S. Creat. (IQR): 1.47 (1.0-1.68) mg/dL | |
|  |  |  |  |  |  |  | NaHCO3 | 150 mEq in 1L 0.9% NaCl at 350 mL/hour | |  | | | Median S. Creat. (IQR): 1.49 (1.12-1.85) mg/dL | |
| Kay 2003(80) | Inclusion Criteria   1. Patients with stable moderate renal insufficiency (S. Creat >1.2mg/dL (106 µmol/L) or creatinine clearance < 60mL/min) 2. Elective coronary angiography and/or intervention  Exclusion Criteria   1. Dialysis 2. Acute renal failure  3. Contrast media or nephrotoxic agent within the last 30 days 4. Overt congestive heart failure  5. Severe valvular disease  6. LVEF< 35% 7. COPD or asthma 8. Allergy to NAC | 1.Coronary Angiography / PCI | 216 | >25% increase above baseline S. Creat. within 48 hours of contrast | Low-Osm | NaCl at 1mL/kg/hr for 12 hr pre- and 6hr post-contrast.  Liberal intake of oral fluid was encouraged | NAC | 600 mg orally twice daily day preceding and day of procedure, total 2 days | | Mean (IQR): 130 (75-320) | | | Median S. Creat. (IQR): 1.24 (0.77-2.99) mg/dL | |
|  |  |  |  |  |  |  | Placebo | placebo orally twice daily day preceding and day of procedure, total 2 days | | Mean (IQR): 120 (70-380) | | | Median S. Creat. (IQR): 1.26 (0.75-3.64) mg/dL | |
| Kefer 2003(81) | Exclusion Criteria   1. S. Creat. >3mg/dl 2. Acute Renal failure | 1.Coronary Angiography  2.PCI | 109 | Increase in S. Creat. ≥0.5 mg/dL (44 µmol/L) or >25% increase above baseline within 24 hours of contrast | Low-Osm Iopromide or Iohexol Mean CV/ml per procedure: 199±77 |  | Intravenous NAC | 1200mg in 200ml 0.9% NaCl in two 60 minute infusions, first 12hr pre-procedure, and second following administration of contrast. | |  | | |  | |
|  |  |  |  |  |  |  | Placebo | placebo in 200ml 0.9% NaCl in two 60 minute infusions, first 12hr pre-procedure, and second following administration of contrast. | |  | | |  | |
| Khosravi 2016(194) | Inclusion Criteria:  Patients aged between 55 and 75 who had diabetes (fasting blood sugar > 126 mg/dL, random blood sugar > 200 mg/dL, and glucose tolerance test > 200 mg/dL) and chronic renal failure (creatinine > 1.5 mg/dL or 15 < glomerular filtration rate [GFR] < 60 mls/min/1.73m2) and were candidates for elective angiography an  Exclusion Criteria:  1. Recent treatment with 80 mg of statin (high dose)  2. Need for emergency angiography  3. Contraindications to statin prescription  4. previous contrast-media administration during the preceding 10days  5. Chronic dialysis treatment, and informed refusal of consent | 1.Coronary Angiography | 229 | Increase in serum creatinine more than 0.5 mg/dL or more than 25% from the baseline (for this analysis we took 48 hours incidences) | Iso-Osm | Isotonic saline (0.9% sodium chloride or half saline, 1 3 mL/kg/h), intravenously, and N-acetylcysteine (NAC) 1200 mg, orally, twice a day, 1 day before to 2 days after intervention started from1hour before angiography until 4 hours after | Atorvastatin | 80mg/d from 48h before angiography | | < 200 ml for 96.3% of patients | | | S. Creat.  Mean+SD:  1.53±0.44 | |
|  |  |  |  |  |  |  | Placebo | Matching Placebo | | < 200 ml for 98.2% of patients | | | S. Creat.  Mean+SD:  1.47±0.42 | |
| Khoury 1995(82) | Inclusion Criteria  1. Patients undergoing radiocontrast study   Exclusion Criteria   1. NSAID use 2. Use of nephrotoxic drugs 3. Contrast media administration within 72 hours of procedure  4. LVEF < 30% | 1.CT 2.Angiography 3.IVP 4.Venogram | 87 | >25% increase above baseline S. Creat. to above 124 µmol/L | Radiologist preference allowed | Normal saline 0.5-1.5 L pre-procedure and 0.5 L after | Nifedipine | 10mg 1 hour pre-procedure | | Mean ±SD: 126±34.8 | | | Mean S. Creat. ±SD:  92.4±53.1mmol/L | |
|  |  |  |  |  |  |  | Control | no intervention | | Mean ±SD: 118.1±41.8 | | | Mean S. Creat. ±SD:  92.5±31.6mmol/L | |
| Kimmel 2008(83) | Inclusion Criteria   1. Mild to moderately impaired kidney function  2. Age> 18 years 3. S. Creat. ≥1.2 mg/dl or Creatinine clearance < 50 ml/min measured by a 12- or 24-h urine collection  Exclusion Criteria   1. Acute inflammatory disease 2. Medication with NSAID or metformin up to 3 days before entering study 3. Abnormal findings in physical examinations, e.g. signs of dehydration or inflammation. | 1.Coronary Angiography | 60 | Increase in S. Creat. ≥0.5 mg/dL (44 µmol/L) or >25% increase above baseline | Iomeperole Low-Osm | 1ml/kg/h infusion of 0.45% saline for 24 h (12 h before and 12 h after exposure to contrast media | NAC | 600 mg orally twice daily day preceding and day of procedure, total 2 days | | Mean ±SD: 187±88 | | | Mean S. Creat. ±SD:  1.51±0.23mg/dL | |
|  |  |  |  |  |  |  | Zinc | 60 mg once daily on the day before missing doses replaced by placebo | | Mean ±SD: 173±85 | | | Mean S. Creat. ±SD:  1.60±0.49mg/dL | |
|  |  |  |  |  |  |  | Placebo | placebo orally twice daily day preceding and day of procedure, total 2 days | | Mean ±SD: 219±105 | | | Mean S. Creat. ±SD:  1.65±0.65mg/dL | |
| Kinbara 2010(84, 85) | Inclusion Criteria   1. Patients with stable S. Creat. (baseline ± ≤0.1mg/dL at 12-24h pre-procedure) concentrations undergoing coronary angiography± PCI   Exclusion Criteria    1. Acute MI requiring primary or rescue PCI 2. Use of vasopressors before PCI 3. Cardiogenic shock 4. Current peritoneal dialysis or haemodialysis, 5. Planned post-contrast dialysis 6. Allergies to the medications being studied 7. Overt congestive heart failure 8. Severe valvular disease 9. LVEF<30% 10. Pregnancy 11. Multiple myeloma 12. Amyloidosis | 1.Coronary Angiography  2.PCI | 49 | Increase in S. Creat. ≥0.5 mg/dL (44 µmol/L) after 48h | Iopamidol  Low-Osm | Nacl 1 ml/kg/hr for 30 min pre- and 10h post-procedure | NAC | 704 mg orally twice daily day preceding and day of procedure, total 2 days | | Mean ±SD: 147±23 | | | Mean S. Creat. ±SD:  1.00±0.36mg/dL | |
|  |  |  |  |  |  |  | Control | IV hydration only | | Mean ±SD: 141±14 | | | Mean S. Creat. ±SD:  0.97±0.29mg/dL | |
|  |  |  |  |  |  |  | Aminophylline | 250mg as a short infusion (100ml saline, 0.9%) 30 min pre-procedure | | Mean ±SD: 142±15 | | | Mean S. Creat. ±SD:  0.94±0.21mg/dL | |
| Kitzler 2012 | Inclusion Criteria   1. Chronic kidney disease stage 1–4  2. Elective CT with non-ionic radiocontrast agents  3. Age >18years  4. S. Creat.> 1.25 mg/dL for males and 1.09 mg/dL for females  4. No renal replacement therapy  Exclusion Criteria  1. Acute kidney injury 2. Increase of >0.2mg/dL in S. Creat. from baseline to enrolment  3. Administration of vitamin E, NAC, or other antioxidant therapy within 4 weeks of study 4. Participation in an investigational clinical trial within 1 month prior to the start of the study 5. Known or suspected allergy to the investigational drugs 6. Current use of a theophylline, dopamine, furosemide, or mannitol. | 1.Elective CT | 30 | >25% increase above baseline S. Creat. at 48 hours | Low-Osm Iopromide | 0.45% saline infusion at 1 ml/kg/hr for 12 h before and after CT. | NAC | 1200gm NAC granules+ placebo emulsion oral route, 12 and 6 h before and 6 and 12 h post-procedure, as well as 0.45 % saline infusion (1:1 dilution of 0.9 % saline with 5 % glucose) | | 100ml | | | Mean S. Creat. ±SD:  1.37±0.51mg/dL | |
|  |  |  |  |  |  |  | Vitamin E | Placebo granules + Vitamin E emulsion (540 mg) intravenously 12 and 6 h before and 6 and 12 h post-procedure, as well as 0.45 % saline infusion | | 100ml | | | Mean S. Creat. ±SD:  1.37±0.2mg/dL | |
|  |  |  |  |  |  |  | Placebo | Placebo granules + placebo emulsion as well as 0.45 % saline infusion | | 100 | | | Mean S. Creat. ±SD:  1.33±0.12mg/dL | |
| Klima 2012(86) | Inclusion Criteria   1. Patients undergoing intra-arterial / intravenous radiological contrast study with renal dysfunction (S. Creat.>0.93 µmol/L for women and 0.117 µmol/L for men, or  eGFR <60mL/min/1.73 m2  Exclusion Criteria   1. Age <18 years 2. Dialysis 3. Allergy to radiographic contrast 4. Pregnancy 5. NYHA class III and IV heart failure  6. NAC ≤24h before contrast 7. Clinical condition requiring continuous fluid therapy, e.g. severe sepsis | 1.Contrast Study | 185 | Increase in S. Creat. ≥0.5 mg/dL (44 µmol/L) or >25% increase above baseline at 48h | Low-Osm Iso-Osm Iopromid Iomeprol Iopentol Iohexol Iobitridol Iodixanol | Additional oral fluid intake was encouraged in all groups | IV hydration | NaCl at 1 mL/kg/h beginning from 8 p.m. on the day pre- and for ≥12 h post-procedure. CV median (IQR), mL: 100 (80–163) | | Median (IQR): 100 (80-163) | | | Median S. Creat. (IQR): 141 (112-158) mmol/L | |
|  |  |  |  |  |  |  | NaHCO3 | Initial intravenous bolus 3 mL/kg/h of 166 mEq/L NaHCO3 for 1hr pre-contrast. Following this, patients received the same fluid at 1mL/kg/h during and for 6h post-procedure | | Median (IQR): 100 (80-143) | | | Median S. Creat. (IQR): 141 (115-164) mmol/L | |
|  |  |  |  |  |  |  | Intravenous and oral NaHCO3 | Excluded from analysis | | | | | | |
| Ko 2013(87) | Inclusion Criteria   1. eGFR <60 mL/min | 1.Coronary Angiography | 159 | Increase in S. Creat. ≥0.5 mg/dL (44 µmol/L) or >25% increase above baseline at 48h | Iodixanol Iso-Osm | 0.45% saline at 1mL/kg/hr (0.5 mL/kg/hr if LVEF <40%) administered ≥ 8hr pre- and post-procedure | Nicorandil | 12 mg dissolved in 100 mL of 0.9% saline | | Mean ±SD: 125.6±69.1 | | | Mean S. Creat. ±SD:  1.73±0.6mg/dL | |
|  |  |  |  |  |  |  | Control | 100ml of 0.9% saline | | Mean ±SD: 126.9±74.6 | | | Mean S. Creat. ±SD:  1.61±0.44mg/dL | |
| Koc 2012(89) | Inclusion Criteria   1. Age ≥18 years  2. Creatinine clearance ≤60mL/min and/or S. Creat. ≥1.1mg/dL   Exclusion Criteria   1. Contrast-agent hypersensitivity 2. Pregnancy  3. Lactation 4. Decompensated heart failure 5. Pulmonary oedema 6. Emergency catheterization 7. Acute renal failure 8. End-stage renal failure | 1.Coronary Angiography  2.PCI | 241 | Increase in S. Creat. ≥0.5 mg/dL (44 µmol/L) or >25% increase above baseline at 48h | Iohexol Low-Osm | As per intervention protocol | Intravenous NAC | IV bolus of 600 mg twice daily before and on the day of procedure (total=2.4 g) plus IV 0.9% saline 1 mL/kg/h before, on and after the day of procedure | | Median (IQR): 130 (100-155) | | | Mean CrCl. ±SD:  59±16mL/min | |
|  |  |  |  |  |  |  | Control | IV 0.9% saline 1 mL/kg/h 12h pre- and 12h post-procedure | | Median (IQR): 130 (119-150) | | | Mean CrCl. ±SD:  63±15mL/min | |
|  |  |  |  |  |  |  | IV hydration | IV 0.9% saline 1mL/kg/h before, on and after the day of coronary procedure | | Median (IQR): 120 (100-150) | | | Mean CrCl. ±SD:  58±16mL/min | |
| Koc 2013(88) | Inclusion Criteria   1. Diabetes Mellitus  2. Age>18 years   Exclusion Criteria   1. Hypersensitivity to contrast 2. Decompensated Heart failure 3. Pulmonary Oedema 4. Pregnancy 5. Lactation 6. Severe renal impairment 7. Emergency Procedure 8. Contrast Medium administration within 7 days | 1.Coronary Angiography  2.PCI | 216 | Increase in S. Creat. ≥0.5 mg/dL (44 µmol/L) or >25% increase above baseline at 48h | Iohexol Low-Osm Preferred | As per intervention protocol | NaHCO3 | 154 mL of 8.4% NaHCO3 add to 846 mL of 5% glucose given at 1ml/kg/hr 6hr pre- and 6h post-procedure | | Median (IQR): 90 (85-100) | | | Median S. Creat. (IQR): 1.0 (0.8-1.3) mg/dL | |
|  |  |  |  |  |  |  | IV hydration | I.V Hydration Group: 1 ml/Kg/hour for 12h pre- and 12h post-procedure | | Median (IQR): 90 (90-100) | | | Median S. Creat. (IQR): 1.0 (0.87-1.3) mg/dL | |
| Koch 2000(90) | Inclusion Criteria   1. Age >18  2. Stable impaired renal function (S. Creat. ≥1.5 mg/dl)   Exclusion Criteria   1. MI 2. Cerebral stroke 3. Chronic cardiac insufficiency 4. Unstable angina pectoris 5. Significant arrhythmias 6. Intake of digitalis 7. Clinically relevant respiratory, gastrointestinal, hematologic, or neurologic illness 8. Haemodialysis or progressive renal failure 9. Severe liver damage 10. Multiple myeloma 11. Autoimmune illnesses or severe allergies 12 Severe uncontrollable HTN (systolic >220 mmHg) 13. Arterial hypotension (systolic <80 mmHg) 14. Cardiogenic Shock 15. Infectious diseases or fever. | 1. Coronary Angiography 2. Peripheral Angiography | 83 | Maximum creatinine increase over 48 h was separated according to the cut off values ≥0.5mg/dl , ≥1.0mg/dl The Cut-off point ≥0.5mg/dl used for the purpose of this analysis | Iso-Osm (320& 340 mg/ml) | 2000 ml (1000 ml NaCl solution/1000 ml 5% glucose solution) for 24 h pre- and post-procedure | PGE1 | 20 ng/kg/min 1 h prior to radiocontrast administration and continued for total of 6 h. | | Mean ±SD: 158.5±73.86 (20-445) | | | Mean S. Creat. ±SD:  2.07±0.48mg/dL | |
|  |  |  |  |  |  |  | PGE1 | The other 2 arms with 10,40 ng concentration is not included in this analysis | | | | | | |
|  |  |  |  |  |  |  | Placebo | placebo prior to radiocontrast administration and continued for total of 6 h. | | Mean ±SD: 158.5±73.86 (20-445) | | | Mean S. Creat. ±SD:  2.41±0.72mg/dL | |
| Kong 2012(91) | Inclusion Criteria   1. Suspected or definitive coronary artery disease 2. Age 18- 80 years old 3. Normal renal function   Exclusion Criteria   1. Acute MI 2. LVEF<45% 3. Blood electrolyte disturbance 4. Liver dysfunction | 1.Coronary Angiography  2.PCI | 85 | Increase in S. Creat. ≥0.5 mg/dL (44 µmol/L) or >25% increase above baseline at 48h-72h | Iopromide Low-Osm | Patients Allowed to drink tap water or other fluid freely in all groups | IV hydration | NaCl at 1ml/kg/hr 12h pre- and 24h post-procedure | | Mean ±SD: 139.6±54.3 with PCI; 79.4±22.4 without PCI | | | Mean S. Creat. ±SD:  102±25.9mmol/L | |
|  |  |  |  |  |  |  | Oral Hydration | 500ml of tap water pre- procedure and 2000ml within 24 hours post procedure | | Mean ±SD: 142.8±56.1 with PCI; 74.7±20.2 without PCI | | | Mean S. Creat. ±SD:  108±32.78mmol/L | |
|  |  |  |  |  |  |  | Oral Hydration | 2000ml oral tap water within 24 hours post-procedure  NB: this group was excluded from our analysis (post intervention hydration only) | | | | | | |
| Kooiman 2014a(93) | Inclusion Criteria   1. Chronic renal impairment (eGFR <60ml/min) 2. High clinical suspicion of acute PE   Exclusion Criteria   1. Pregnancy 2. Previous contrast administration within the past 7 days 3. Documented allergy for iodinated contrast media 4. Hemodynamic instability (SBP < 100 mm Hg) | 1.CTPA | 145 | Increase in S. Creat. ≥0.5 mg/dL (44 µmol/L) or >25% increase above baseline at 48h-96h | Iopromide Low-Osm Iobitridol Low-Osm Iodixanol Iso-Osm Contrast type according to each hospital guidelines | As per intervention protocol | NaHCO3 | 250 mL I.V 1.4% NaHCO3 1 hour before CTPA without hydration after CTPA | | Mean ±SD: 73.8±8.1 | | | Mean eGFR ±SD:  48.2±15.4 | |
|  |  |  |  |  |  |  | Control | No intervention | | Mean ±SD: 74.5±10.3 | | | Mean eGFR ±SD:  50.2±15.5 | |
| Kooiman 2014b(92) | Inclusion Criteria   1. Elective CE-CT 2. Age> 18y 3. CKD (eGFR < 60mL/min/1.73m2  Exclusion Criteria   1. Pregnancy 2. Previous contrast administration within the last 7 days 3. Documented allergy for iodinated contrast media 4. Haemodynamic instability (SBP<100mmHg) 5. Previous participation in the trial | 1.CE-CT | 560 | Increase in S. Creat. ≥0.5 mg/dL (44 µmol/L) or >25% increase above baseline | low-Osm Iomeprol Iobitridol Iodixanol | As per intervention protocol | NaHCO3 | 250 mL I.V 1.4% NaHCO3 1 hour pre-CE-CT | | Mean ±SD: 105.7±21 | | |  | |
|  |  |  |  |  |  |  | I.V Hydration Group: | 2L 0.9% NaCl, 1L pre- and 1L post-CE-CT | | Mean ±SD: 104.7±21.6 | | |  | |
| Kotlyar 2005(94) | Inclusion Criteria   1. S. Creat. ≥0.13 mmol/l, 2. Elective coronary, carotid or peripheral angiography and/or PTCA and stenting  Exclusion Criteria   1. Allergy to the study medication 2. Unstable renal function (Creat. increase ≥0.04 mmol/l day) 3. Patients on dialysis 4. Uncontrolled asthma 5. Pregnancy 6. Breastfeeding | 1.Coronary  angiography  2.Carotid  angiography  3.Peripheral angiography  4. PTCA | 40 | Increase in the serum creatinine concentration of at least 0.044 mmol/l 48-96 hours post procedure | Iopromide Low-Osm | 0.9% saline at 200ml/h 2h pre-and continued for a further 5h post-procedure | Intravenous NAC | 600mg in 100ml of 5% dextrose administered over 20 min, 1–2h before angiography and again 2–4h after angiography | | Mean ±SD: 89±32 | | | Median S. Creat. (Range):  27.5±5.8mmol/L | |
|  |  |  |  |  |  |  | Placebo | matched placebo | | Mean ±SD: 86±41 | | | Median S. Creat. (Range):  27.5±5.8mmol/L | |
| Kumar 2014(95) | Inclusion Criteria:   1. Patients without risk factors for AKI 2. Patients receiving < maximum permissible dose of the dye   Exclusion Criteria  1. Patients receiving >maximum permissible dose of dye 2. Use of nephrotoxic Drugs 3. Gout or serum uric acid levels >10mg/dl 4. Hypersensitivity or intolerance to allopurinol 5. Congestive heart failure or LVEF < 40% 6. Inability to give consent | 1.PCI | 289 | Increase in S. Creat. ≥0.5 mg/dL (44 µmol/L) or >25% increase above baseline | Patents Randomized in 2 groups:  1. Iow-Osm Omnipaqu 2.Iso-Osm Visipaque | 0.9% saline at 1ml/kg/min (to max of 100 ml/hr) for 12h pre- and 12h post-procedure. Patients randomized first to Omnipaque and Visipaque arms which is analysed separately in this study | NAC | 600 mg orally twice daily 12h pre- and post- contrast | |  | | | Mean S. Creat. (Range):  Omnipaque:  1.0 (0.9-1.3) Visipaque  1.1 (0.9-1.2) | |
|  |  |  |  |  |  |  | Allopurinol | 300 mg orally 12h pre- and post- contrast | |  |  |  |  |  |
|  |  |  |  |  |  |  | I.V Hydration | IV hydration only | |  |  |  |  |  |
| Kurnik  1990(97) | Inclusion Criteria   1. Patients with stable S. Creat ≥ 1.8mg/L 2. Elective cardiac catheterization  Exclusion Criteria   1. Hypersensitivity to peptide drugs, local anaesthetics, heparin or radiocontrast 2. Pregnancy 3. NYHA Class IV CHF 4. Radiocontrast exposure within the last 7 days 5. MI within the last 2 weeks 6. Unstable renal function 7. Medical instability | 1.PCI | 28 | Increase in S. Creat. ≥0.5 mg/dL (44 µmol/L) above baseline at 24h post-contrast | MD 76 Hi-Osm | 75mmol/L NaCl in water at 100ml/hr starting 12 hr pre-procedure | ANP | Bolus: 50µg, followed by 1µg/min mixed in 75mmol Nacl for 2 hours | |  | | | Mean S. Creat. ±SEM:  2.4±0.7mg/dL | |
|  |  |  |  |  |  |  | Mannitol | 15% mannitol for 2 hours before and during procedure | |  |  |  | Mean S. Creat. ±SEM:  2.5±0.8mg/dL | |
| Kurnik  1998(96) | Inclusion Criteria   1. Patients aged 18-85years  2. Patients with stable S. Creat ≥1.8mg/L OR ≥ 1.5mg/L and < 1.8mg/L with Creat. Clearance ≤65ml/min  3. Elective cardiac catheterization  Exclusion Criteria   1. Hypersensitivity to peptide drugs, local anaesthetics, heparin or radiocontrast 2. Dialysis  3. SBP ≤100 mmHg before study 3. Dialysis  4. Major surgery or radiocontrast exposure within the last 7 days 5. Expected Dose of radiocontrast <than 75 mL 6. NYHA Class IV CHF 7. Scheduled surgical procedure within 48hr post-procedure 8. Pregnancy 9. Lack of consent  10. Co-morbidity | 1.Contrast-enhanced radiographic procedures | 152 | Increase in S. Creat. ≥0.5 mg/dL (44 µmol/L) or >25% increase above baseline | Choice of radiocontrast agent determined by the angiographer | Intravenous 0.45% saline for 12hr pre= and continuing for 12hrpost-contrast | ANP | 0.05 µg/kg/min 30 minutes before and continuing for 30 minutes after radiocontrast administration. Other 2 arms (0.1 and 0.01 µ µg/kg/min were excluded from this analysis) | | Mean ±SD: 141±58 | | | Mean S. Creat. ±SD:  2.1±0.9mg/dL | |
|  |  |  |  |  |  |  | Placebo | matched placebo | | Mean ±SD: 132±54 | | | Mean S. Creat. ±SD:  2.1±0.56mg/dL | |
| Lawlor 2007(99) | Inclusion Criteria   1. Pre-existing renal impairment 2. Angioplasty for peripheral vascular disease   Exclusion Criteria   1. Acute Renal Failure 2. Haemodynamic instability 3. Unable to tolerate hydration protocol for medical reasons 4. Known sensitivity to NAC 5. Unable to provide informed consent | 1.Peripheral Angioplasty | 54 | Increase in S. Creat. ≥0.5 mg/dL (44 µmol/L) or >25% increase above baseline at 48h |  | 0.9% normal saline 1 ml/kg/hr for 12 hours pre- and post-procedure | NAC | 600 mg in 30ml of ginger ale orally twice daily, day prior to and day of angioplasty | |  | | | Mean S. Creat. ±SD:  167±46mmol/L | |
|  |  |  |  |  |  |  | Placebo | 3ml of 0.9% normal saline in 30ml of ginger ale orally twice daily, day prior to and day of angioplasty | |  | | | Mean S. Creat. ±SD:  172±48mmol/L | |
|  |  |  |  |  |  |  | Control | Out-patient oral hydration followed by IV hydration, *excluded from analysis* | | | | | | |
| Lee 2011(100) | Inclusion Criteria   1. Patients undergoing arteriography or intervention 2. S. Creat ≥1.1mg/dl, eGFR <60ml/min/1.73m2 3. Age >18yr 4. Diabetes mellitus  Exclusion Criteria   1. Inability to obtain informed consent 2. S. Creat >8mg/dl, eGFR <15ml/min/1.73 m2 at rest 3. End-stage renal disease on haemodialysis 4. Multiple myeloma 5. Pulmonary oedema 6. Uncontrolled hypertension (SBP >160 mmHg or DBP >100 mmHg) 7. Acute STEMI while undergoing primary PCI 8. Emergency coronary angioplasty or angiography 9. Use of contrast media within the previous 2 days 10. Pregnancy 11. Allergy to contrast medium or medications such as theophylline, Dopamine, Mannitol, Fenoldopam, and NAC | Coronary and endovascular angiography ± Intervention | 402 | Increase in S. Creat. ≥0.5 mg/dL (44 µmol/L) or >25% increase above baseline at 48h | Iodixanol Iso-Osm | As per intervention protocol. Also, NAC 1,200 mg twice daily for 2 days starting day before procedure. | NaHCO3 | 154 mEq/L in dextrose and water starting at 3 ml/kg/hr, 1hr pre-procedure, decreasing to 1 ml/kg/hr during procedure and for 6hr post-procedure (decreased to 0.5 l/kg/hour in patients with LVEF <45%) | | Mean (Range): 113 (80-200) | | | Mean S. Creat. (Range):  1.5 (1.3-1.9) mg/dL | |
|  |  |  |  |  |  |  | IV hydration | 0.9% sodium chloride 1 ml/kg/hour for 12 hours pre- and post- procedure  (decreased to 0.5 l/kg/hour in patients with LVEF <45%) | | Mean (Range): 120 (79-223) | | | Mean S. Creat. (Range):  1.5 (1.3-1.7) mg/dL | |
| Lehnert 1998(101) | Inclusion Criteria   1. Stable S. Creat. ≥1.4 mg/dl (124 µmol/l) 2. Angiography (contrast medium dose ≥1.2 ml/kg)  Exclusion Criteria  1. End-stage renal disease 2. Allergy to contrast medium 3. Prior exposure to contrast medium within 14 days before 4. Age <30yr 5. Pregnancy | 1.Angiography | 44 | Increase in S. Creat. ≥0.5 mg/dL (44 µmol/L) above baseline at 48h post-contrast | Iopentol Low-Osm | 0.9% saline I.V at 83 ml/h starting 12h pre- and for 12h post-procedure | Haemodialysis | Haemodialysis start with injection of the last part of contrast media and continue for 3 hours | | Mean CV (ml/kg) ±SEM: 3.5±0.6 | | | Mean S. Creat. ±SD:  2.58±0.25mg/dL | |
|  |  |  |  |  |  |  | Control | IV hydration only | | Mean CV (ml/kg) ±SEM: 3.0±0.4 | | | Mean S. Creat. ±SD:  2.26±0.2mg/dL | |
| Leoncini 2014(102) | Inclusion Criteria   1. Non-STEMI 2. Early invasive strategy  Exclusion Criteria   1. Current statin treatment 2. High-risk features warranting emergency coronary angiography (within 2 h) 3. Acute renal failure or end-stage renal failure requiring dialysis 4. Serum creatinine ≥ 3mg/dl 5. Severe comorbidities which precluded early invasive strategy 6. Contraindications to statin treatment 7. Contrast medium administration within the previous 10 days 8. Pregnancy 9. Refusal of consent | 1.PCI | 559 | Increase in S. Creat. ≥0.5 mg/dL (44 µmol/L) or >25% increase above baseline | Iso-Osm Iodixanol [Visipaque] | 0.9% saline I.V at 1ml/kg/h starting 12h pre- and for 12h post-procedure. NAC 1,200 mg twice daily for 2 days starting day before procedure.  (Fluid decreased to 0.5 l/kg/hour in patients with LVEF <40%) | Statin | 40 mg Rosuvastatin on time of randomization followed by 20 mg/day | | Mean ±SD: 183±80 | | | Mean S. Creat. ±SD:  0.95±0.27mg/dL | |
|  |  |  |  |  |  |  | Control | No Statins | | Mean ±SD: 127±72 | | | Mean S. Creat. ±SD:  0.96±0.28mg/dL | |
| Li, W 2012(104) | Inclusion Criteria   1. Patients with acute STEMI 2. Emergency PCI`  Exclusion Criteria   1. Statin treatment within preceding 3 months  2. Renal or hepatic dysfunction 3. Dialysis  4. Prior fibrinolysis 5. Unconsciousness on arrival 6. Cardiogenic shock with intra-aortic balloon pumping 7. Uncontrolled hypertension 8. Stroke 9. Major operation within the last 3 months 10. Refuse PCI | 1.PCI | 176 | Increase in S. Creat. ≥0.5 mg/dL (44 µmol/L) above baseline 3days post-contrast | Low-Osm Ultravist | 0.9% saline I.V at 1ml/kg/h starting 12h pre- and for 12h post-procedure | Statin | 80mg Atorvastatin pre-procedure, and continued long-term, 40mg/day | | Mean ±SD: 100±25.9 | | | Mean S. Creat. ±SD:  82.3±11.2mmol/L | |
|  |  |  |  |  |  |  | Placebo | matched placebo | | Mean ±SD: 103.6±26.2 | | | Mean S. Creat. ±SD:  82.6±11.3mmol/L | |
| Li 2009(103) | Inclusion Criteria   1. Patients undergoing planned coronary angiography or intervention  Exclusion Criteria   1. NYHA Class IV CHF 2. S. Creat.  >3.0 mg/dl | 1.Coronary Angiography  2.PCI | 228 | Increase in S. Creat. ≥0.5 mg/dL (44 µmol/L) above baseline 3days post-contrast | Iohexol Low-Osm | 0.9% saline I.V at 1ml/kg/h for 12h post-procedure | probucol | 500 mg orally twice daily for 3 days before and after the procedure | | Mean ±SD: 116±65 | | | Mean S. Creat. ±SD:  0.99±0.4mg/dL | |
|  |  |  |  |  |  |  | Control | No Probucol | | Mean ±SD: 121±56 | | | Mean S. Creat. ±SD:  1.08±0.71mg/dL | |
| Li 2011(106) | Inclusion Criteria   1. Mild to moderate renal insufficiency (eGFR 60-89ml/min)  Exclusion Criteria  1. Diagnostic only procedure 2. S. Creat ≥176 µmol/L  3. NYHA class IV CHF  4. Renal artery stenosis 5. Diagnosed during angiography 6. Allergy to contrast medium 7 ACEI intolerance 8. Autoimmune disease 9. End Stage Renal Failure requiring dialysis 10. Administration of contrast medium within the last 6 days or within the next flowing 2 days 11. Pregnancy | 1.PCI | 123 | Increase in S. Creat. ≥0.5 mg/dL (44 µmol/L) or >25% increase above baseline at 72 h post-contrast | Iohexol Iow-Osm | 0.9% sodium chloride at 1ml/kg/hr for 6 hours pre- and 6 hours post-procedure | ACE-inhibitor | 10mg Benazepril daily for at least 3 days pre-procedure | | Mean ±SD: 167.37±51.23 | | | Mean S. Creat. ±SD:  83.2±15.44mmol/L | |
|  |  |  |  |  |  |  | Placebo | matched placebo | | Mean ±SD: 159.90±51.58 | | | Mean S. Creat. ±SD:  83.4±16.71mmol/L | |
| Li 2014(105) | Inclusion Criteria   1. Patients undergoing coronary intervention procedure  Exclusion Criteria   1. Patients who used nephrotoxic drugs during pre-operative period 2. Severe hepatic and renal dysfunction  (eGFR) <30ml/min/1.73 m2) 3. Active cancer  4. NYHA class IV CHF 5. LVEF<35 % 6. Thyroid or adrenal dysfunction 7. Acute or chronic infectious diseases 8. Hyperpyrexia | 1.PCI | 175 | Increase in S. Creat. ≥0.5 mg/dL (44 µmol/L) or >25% increase above baseline at 48 h post-angiography | Iohexol Iow-Osm | 0.9 % sodium chloride solution for routine hydration (volume/rate not specified) | PGE1 | Intravenous infusion at 20ng/kg/min for 6 h before and after the administration of contrast | | Mean ±SD: 172±32 | | | Mean S. Creat. ±SD:  0.98±0.14mg/dL | |
|  |  |  |  |  |  |  | Control | IV hydration only | | Mean ±SD: 168±41 | | | Mean S. Creat. ±SD:  0.96±0.17mg/dL | |
| Liu, W 2015(196) | Inclusion Criteria:  Patients 18-75 yaesr old with mild to modrate CKD (eGFR 30-89/min)  Exclusion Criteria:  1. Acute renal failure  2. End stage renal disease that needs dialysis  3. unstable renal function  4. Uncontrolled DM, HTN or Hyperthyroidism  5. Class IV cardiac failure or; left ejection fraction < 35%  6. Acute myocardial infarction require primary or rescue coronary intervention  7. Cardiogenic shock  8. Administration of contrast media from 7 days before to 72 hours after study intervention  8. Agents for CIN prevention (such as NAC) or intake of nephrotoxic drugs from 24 before to 24 hours after  9. patients treated with ascorbic acid within last 30 days  10 Allergy to Trimetazidine | 1.Coronary angiography | 151 | increase of >25% or an absolute increase of ≥0.5 mg/dl in SCr from the baseline value 48 -72 hours of contrast administration | Iodixanol  Iso-Osm | 1-1.5 ml/ kg per hour start 3-12 hours before angiography and up to 12 hours thereafter | Trimetazidine | 20mg three times daily orally 48 hours before and 24 hours after coronary angiography | | Mean+SD:  124.94±31.65 | | | Mean S. Creat. ±SD:  107.74±24.03 | |
|  |  |  |  |  |  |  | Control | No Trimetazidine | | Mean+SD:  119.69±34.28 | | | Mean S. Creat. ±SD:  103.38±19.43 | |
| Liu 2014(105) | Inclusion Criteria   1. Patients with unstable angina  Exclusion Criteria   1. Early invasive therapy (within 12-24 hours) 2. Patients with refractory angina or hemodynamic or electrical instability 3. Patients at increased risk for clinical events (CHF, serious ventricular ar- rhythmias) 4. High-risk unstable angina (resting angina within 48 hours or infarction angina, ST-segment depression more than 1 mm and 20 minutes, or increased cardiac bio- markersdtroponin T or I) 5. Stable angina 6.STEMI 7. NSTEMI 8. Pre-existing renal dysfunction. | 1.Coronary Angiography  2.PCI | 1100 | Increase in S. Creat. ≥0.5 mg/dL (44 µmol/L) or >25% increase above baseline at 48 h post-angiography | Ultravist  Iopromide | As per intervention protocol | Human recombinant (Brain Natriuretic Peptide) (rhBNP) | 0.005 µg/kg/min for 24 hours before procedure | | Median (Range): 128 (60-185) | | | Mean S. Creat. ±SD:  79.2±14.2mmol/L | |
|  |  |  |  |  |  |  | IV hydration | 0.9%, Normal saline at 1 mL/kg/h for 24 hours before PCI CV/mL: 119 (63,172) | | Median (Range): 119 (63-172) | | | Mean S. Creat. ±SD:  81.5±16.7mmol/L | |
| Liu 2016(195) | Inclusion Criteria  patients with CKD (eGFR between  15 and 60 mL/min/1.73m2) aged 18 to 80 years and undergoing coronary angiography or elective PCI  Exclusion Criteria   1. Emergency PCI 2. Patients with refractory angina or hemodynamic or electrical instability 3. Patients at increased risk for clinical events (CHF, serious ventricular  arrhythmias) 4. High-risk unstable angina (resting angina within 48 hours or infarction angina, ST-segment depression more than 1 mm and 20 minutes, or increased cardiac biomarkers  5. Heart dysfunction 6. Hypersensitivity to Contrast media or BNP  7. End-stage renal failure 8. systolic blood pressure ≤100mmHg before study drug infusion  9. CM administered within the past 7 days; BNP infusion within 1month; dopamine, NAC, Nahco3 and fenoldopam during the study. | 1.Coronary Angiography  2.PCI | 232 | Relative (≥25%) or absolute (≥0.5mg/dL, 44 µmol/L) increase in SCr from baseline within 48 h after CM exposure | Iodixanol  Iso-Osm | 0.9% NaCl at 1.0mL/kg/h for 12h before and 12h aſter CM administration | Human recombinant (Brain Natriuretic Peptide) (rhBNP) | 0.005 µg/kg/min for 24 hours before procedure | | Mean+SD:  102 ± 17.2 | | | Mean S. Creat. ±SD:  117.2 ± 13.1 | |
|  |  |  |  |  |  |  | Control | Hydration only | | Mean+SD:  96 ± 14.5 | | | Mean S. Creat. ±SD:  120.5 ± 14.7 | |
| Ludwig 2011(107) | Inclusion Criteria   1. Patients scheduled for cardiac catheterization, arteriography or computed tomography with contrast agents  2. S. Creat. ≥ 150 µmol/l (1.7mg/dl)  Exclusion Criteria   1. Patients undergoing dialysis 2. Acute Renal Failure 3. Received iodinated contrast media within 7 days prior to the study 4. Known allergy to MESNA 5. Pregnancy 6. Patients receiving dopamine, mannitol, or NAC | 1.Coronary Angiography  2.PCI  3.CE-CT | 107 | >25% increase above baseline S. Creat. at 48 hours | Iopromide Low-Osm | 1000 ml of 0.9% saline pre- and 500ml post-procedure in both groups. No further oral fluid intake was allowed. | MESNA | Infusion of 1600 mg of sodium 2-mercaptoethanesulfonate (MESNA) CV/mL: 140 (120-200) | | Median (Range): 140 (120-200) | | |  | |
|  |  |  |  |  |  |  | Control | IV hydration only | | Median (Range): 150 (120-180) | | |  | |
| Luo  2014(109) | Inclusion Criteria   1. STEMI  2. Primary PCI  Exclusion Criteria  1. Chronic peritoneal or haemodialysis treatment 2. Exposure to radiographic contrast within the previous two days 3. Allergies to radiographic contrast medium 4. Coronary anatomy not suitable for PCI or primary CABG | 1.PCI | 276 | Increase in S. Creat. ≥0.5 mg/dL (44 µmol/L) or >25% increase above baseline at 72h post-angiography | Iopamidol Low-Osm | As per intervention protocol | IV hydration | 0.9% normal saline at 1mL/kg/hr for 12 hours post-procedure | | Mean ±SD: 228.6±84.5 | | | Mean S. Creat. ±SD:  76±14mmol/L | |
|  |  |  |  |  |  |  | Control | no hydration | | Mean ±SD: 241.2±101.4 | | | Mean S. Creat. ±SD:  78±13mmol/L | |
| Luo 2013(108) | Inclusion Criteria   1. Age ≥18 years  2. Elective PCI 3. Informed consent  Exclusion Criteria   1. Emergency PCI 2. Baseline troponin ≥ 0.04ng/mL 3. Nicorandil or glibenclamide use  4. Inability to cooperate with trial protocol  5. Lack of informed consent 6. Second procedure of staged elective PCI | 1.PCI | 208 | Increase in S. Creat. ≥0.5 mg/dL (44 µmol/L) or >25% increase above baseline | Iopromide Low-Osm | I.V saline infusion at 1mL/kg/hr for 12 hr pre- and 12h post-contrast. Patients encouraged to drink oral fluids post procedure | RIPC | Blood pressure cuff placed around their non-dominant upper arm. The cuff inflated to 200-mm Hg pressure for 5 minutes, followed by 5 minutes of deflation and repeated 2 more times to 3 cycles in total <2h pre-procedure | |  | | | Mean eGFR ±SD:  101±20mL/min | |
|  |  |  |  |  |  |  | Control | no intervention | |  | | | Mean eGFR ±SD:  100±20mL/min | |
| MacNeill 2003(110) | Inclusion Criteria   1. Cardiac catheterization  2. Mild-moderate renal dysfunction (S. Creat ≥ 1.5 mg/dl)  Exclusion Criteria   1. Acute renal failure 2. Dialysis-dependent chronic renal failure 3. Exposure to contrast within the preceding 5 days 4. Emergent procedures 5. Pregnancy 6. Age < 21 years 7. Known sensitivity to acetylcysteine | 1.Coronary Angiography2.PCI | 51 | >25% increase above baseline S. Creat. at 72 hours | Iopromid Low-Osm Ioxilan Low-Osm | Pre-procedure: 0.45% saline at 1ml/kg/hr for 12h (in-patients) and 2ml/kg/hr for 4h (day-case patients). Post-procedure: 0.45% saline at 75 ml/hr for 12h | NAC | 600mg twice daily commenCI-AKIg day of procedure, for total of 5 doses | | Mean ±SD: 103±52 | | | Mean S. Creat. ±SD:  1.89±0.38mg/dL | |
|  |  |  |  |  |  |  | Placebo | matched placebo | | Mean ±SD: 116±63.3 | | | Mean S. Creat. ±SD:  1.88±0.41mg/dL | |
| Maioli 2008(5) | Inclusion Criteria   1. Planned coronary angiographic procedures  2. Renal Dysfunction (estimated Creat. Clearance <60ml/min)  Exclusion Criteria   1. Creat. Clearance ≥ 60ml/min 2. Refusal to participate 3. Administration of contrast medium within the previous 10 days 4. End stage renal disease | 1.Coronary Angiography | 556 | Increase in S. Creat. ≥0.5 mg/dL (44 µmol/L) above baseline 5days post-contrast | Iodixanol Iso-Osm | as per intervention protocol | NaCl | 0.9% sodium chloride at 1mL/kg/hr for 12 h pre- and post-procedure | | Mean (IQR): 170 (120-230) | | | Mean S. Creat. ±SD:  1.20±0.3mg/dL | |
|  |  |  |  |  |  |  | NaHCO3 | 154 mEq/l in dextrose and water at 3 ml/kg/h for 1 h pre-procedure, 1ml/kg/h for 6h post-procedure | | Mean (IQR): 160 (120-220) | | | Mean S. Creat. ±SD:  1.21±0.3mg/dL | |
| Maioli 2011(111) | Inclusion Criteria   1. STEMI 2. Primary PCI  Exclusion Criteria   1. Contrast medium administration within the previous 10 days 2. End-stage renal failure requiring dialysis 3. Refusal to give informed consent | 1.PCI | 543 | Increase in S. Creat. ≥0.5 mg/dL (44 µmol/L) or >25% increase above baseline within 3 days of contrast | Iodixanol Iso-Osm | as per intervention protocol | NaHCO3 | 154 mEq/L in dextrose and water at 3ml/kg/h, starting in the emergency room for 1h, followed by infusion of 1 mL/kg/h for 12 hours after PCI | | Mean ±SD: 208±92 | | | Mean S. Creat. ±SD:  1.09±0.3mg/dL | |
|  |  |  |  |  |  |  | NaCl | 0.9% sodium chloride at 1mL/kg/h for 12h immediately post-PCI | | Mean ±SD: 216±101 | | | Mean S. Creat. ±SD:  1.10±0.4mg/dL | |
|  |  |  |  |  |  |  | Control | no hydration | | Mean ±SD: 224±94 | | | Mean S. Creat. ±SD:  1.08±0.3mg/dL | |
| Malhis 2010(112) | Inclusion Criteria   1. Radiographic imaging with contrast   Exclusion Criteria   1. Acute renal failure 2. Maintenance dialysis 3. History of acute MI 4. LVEF ≤ 25% 5. Allergy to contrast media 6. Pregnancy 7. Contraindications for theophylline use  8. Use of acetylcysteine | 1.Coronary Angiography2.PCI  3.CE-CT | 294 | Increase in serum creatinine of at least 0.5 mg/dL in patients with a baseline serum creatinine less than 2 mg/dL or an increase of 25% in base- line Serum Creatinine with a baseline serum creatinine more than or equal to 2 mg/dL at 48 h after administration of contrast media | Iodixanol  Iso-Osm | 1-2 L of intravenous bicarbonate solution (150 meq/L) for 12h after the procedure | Theophylline | 200 mg twice daily starting 24h pre-procedure and continuing for 48 h post-procedure; OR 200 mg theophylline as a short intravenous infusion 30 minutes pre-procedure, and continuing with 200 mg twice daily oral theophylline for 48 h post-procedure | | Mean ±SD: 137±76 | | | Mean S. Creat. ±SD:  1.38±0.79mg/dL | |
|  |  |  |  |  |  |  | Control | IV hydration only | | Mean ±SD: 144±78 | | | Mean S. Creat. ±SD:  1.21±0.48mg/dL | |
| Marenzi 2003(115) | Inclusion Criteria   1. Chronic renal failure (S. Creat. >2mg/dL (176.8 µmol/L) OR creat. clearance < 50ml/min)  2. Coronary angiography or elective PCI  Exclusion Criteria   1. Acute coronary syndrome 2. Cardiogenic shock 3. Long-term peritoneal dialysis or haemodialysis treatment 4. Overt CHF 5. Recent major bleeding 6. Contraindications to anticoagulant therapy | 1.Coronary Angiography2.PCI  3.Aortic angiography  2.Peripheral angioplasty/ Renal angioplasty | 145 | >25% increase above baseline S. Creat. | Iopentol Low-Osm | as per intervention protocol | Hemofiltration | Hemofiltration starting 4-6 h pre-procedure; stopped during and resumed post-procedure, and continued for 18- | | Mean ±SD: 247±125 | | | Mean S. Creat. ±SD:  3.0±1.0mg/dL | |
|  |  |  |  |  |  |  | Control | Normal saline at 1ml/Kg/hour (reduced to 0.5ml/kg/h if LVEF<40%) for 6-8h pre- and 24h post-procedure | | Mean ±SD: 258±132 | | | Mean S. Creat. ±SD:  3.1±1.0mg/dL | |
| Marenzi 2006(113) | Inclusion Criteria   1. Primary angioplasty for acute STEMI within 12h of presentation (18 hours in cases of cardiogenic shock)  Exclusion Criteria   1. Long-term dialysis 2. Known allergy to N-acetylcysteine | 1.Coronary Angiography | 291 | >25% increase above baseline S. Creat. at 72 hours | Iohexol Low-Osm | Normal saline at 1ml/Kg/hour (reduced to 0.5ml/kg/h if LVEF<40%) for 12h post-procedure | NAC | Intravenous bolus of 600 mg pre-procedure and 600mg orally twice daily for 48h post-procedure, to a total dose 3000mg fter intervention (total dose of 3000 mg) | | Mean ±SD: 264±146 | | | Median S. Creat.:  1.01mg/dL | |
|  |  |  |  |  |  |  | Control  3^rd^ NAC Double Dose Group were excluded | - | | Mean ±SD: 274±113 | | | Median S. Creat.:  1.06mg/dL | |
| Marenzi 2006(113) | Inclusion Criteria   1. Severe chronic kidney disease (Creat. clearance <30mL/min 2. Diagnostic and therapeutic cardiovascular procedures  Exclusion Criteria   1. Acute coronary syndrome 2. Cardiogenic shock 3. Acute renal failure 4. Chronic peritoneal or haemodialysis  5. Overt CHF 6. Recent major bleeding 7. Contraindications to anticoagulation | 1.Coronary Angiography2.PCI | 81 | >25% increase above baseline S. Creat. | Iopentol Low-Osm | Normal saline at 1ml/Kg/hour (reduced to 0.5ml/kg/h if LVEF<40%) | Hemofiltration post-procedure | I.V isotonic saline, for 12h pre-contrast followed by hemofiltration for 18-24h post-contrast | | Mean ±SD: 237±122 | | | Mean S. Creat. ±SD:  3.6±0.7mg/dL | |
|  |  |  |  |  |  |  | Control | I.V isotonic saline for 12h pre- and 12h post-contrast | | Mean ±SD: 232±144 | | | Mean S. Creat. ±SD:  3.6±0.8mg/dL | |
|  |  |  |  |  |  |  | Hemofiltration pre- and post-procedure | Hemofiltration pre- and post-procedure | | excluded from analysis | | | | |
| Marenzi 2012(114) | Inclusion Criteria   1. Age 18-85 2. Chronic Kidney disease (eGFR <60ml/min/1.73m2)   Exclusion Criteria   1. Primary or rescue PCI 2. Angiography procedures requiring a direct renal injection of contrast 3. Cardiogenic shock 4. Overt CHF 5. Acute respiratory Insufficiency 6. Recent acute kidney injury 7. Chronic peritoneal or haemodialysis 8. Known furosemide hypersensitivity 9. Receipt of intra- venous contrast within 10 days before the procedure 10. Another planned contrast-enhanced procedure in the following 72hours 11. Contraindications to placement of a Foley catheter in the bladder | 1.Coronary Angiography2.PCI | 189 | Increase in S. Creat. ≥0.5 mg/dL (44 µmol/L) or >25% increase above baseline | Iomeprol Low-Osm | as per intervention protocol | Furosemide | Single intravenous bolus of 0.5 mg/kg (up to a maximum of 50 mg) after initial bolus of 205ml normal saline. Renal Guard system used for fluid replacement (Matched hydration) | | Mean ±SD: 181±104 | | | Mean S. Creat. ±SD:  1.8±0.6mg/dL | |
|  |  |  |  |  |  |  | Control | Normal saline at 1ml/Kg/hour (reduced to 0.5ml/kg/h if LVEF<40%) for 12h pre- and 12h post-procedure | | Mean ±SD: 158±109 | | | Mean S. Creat. ±SD:  1.7±0.5mg/dL | |
| Markota 2013(116) | Inclusion Criteria   1. Age >18   Exclusion Criteria   1. End-stage renal insufficiency (eGFR <15 mL/min) 2. Acute renal insufficiency 3. History of reaction to contrast media 4. Use of nephrotoxic MediCI-AKIes 5. Pulmonary Oedema 6. Multiple myeloma 7. Factors predisposing to kidney injury  8. Exposure to contrast media within 7 days before the procedure 9. Pregnancy 10. Non-compliance 11. Use of NAC, teofiline, dopamine, fenoldopam, manitol, or NaHCO3 within 48h of procedure | 1.Coronary Angiography2.PCI | 227 | Increase in S. Creat. ≥0.5 mg/dL (44 µmol/L) or >25% increase above baseline, OR >25% decrease in eGFR within 48h of procedure | Iopamiro 370 Low-Osm | eGFR <60 mL/min/1.73m2: IV normal saline at 1mL/kg/h for 2h pre- and 12h post-procedure.   eGFR >60 mL/min/1.73m2: Hydration orally | Na/K citrate | 5 g of granules diluted in 200 mL of water | | Mean ±SD: 222.3±102.3 | | | Mean S. Creat. ±SD:  93.96±18.39mmol/L | |
|  |  |  |  |  |  |  | Control | 200ml water | | Mean ±SD: 231.2±95.85 | | | Mean S. Creat. ±SD:  89.35±23.97mmol/L | |
| Masuda 2007(117) | Inclusion Criteria   1. Chronic kidney disease (S. Creat >1.1mg/dl or eGFR <60ml/min)  2. Emergency coronary procedure   Exclusion Criteria   1. Change in serum creatinine concentration of at least 0.5mg/dl during the previous 24 hours 2. Pre-existing dialysis 3. Recent exposure to radiographic contrast media within 2 days of the study 4. Allergy to radiographic contrast media 5. Pregnancy 6. Previous or planned administration of mannitol, fenoldopam, N-acetylcysteine or non-study sodium bicarbonate | 1.Emergency coronary procedure | 71 | Increase in S. Creat. ≥0.5 mg/dL (44 µmol/L) or >25% increase above baseline within 2days of procedure | Iopamidol Low-Osm | Initial intravenous bolus of 3ml/kg/h for 1h, if possible, pre-procedure. Same fluid at 1ml/kg/h during and for 6h post-procedure | NaHCO3 | 154 mEq/L of sodium bicarbonate | | Mean ±SD: 112±89 | | | Mean S. Creat. ±SD:  1.31±0.52mg/dL | |
|  |  |  |  |  |  |  | NaCl | 154 mEq/L sodium chloride | | Mean ±SD: 120±61 | | | Mean S. Creat. ±SD:  1.32±0.65mg/dL | |
| Matejka 2010(118) | Inclusion Criteria  1. Age > 18 years 2. S. Creat. persistently >1.47 mg/dl (130 µmol/l)  Exclusion Criteria  1. Long-term dialysis 2. Pregnancy 3. Lactation 4. Epilepsy 5. Thyrotoxicosis 6. Theophylline allergy 7. Previous theophylline medication 8. Arrhythmias with hemodynamic instability 9. Severe liver dysfunction 10. Clinical signs of dehydration 11. Inability to take oral fluids. 12. Use of ACE inhibitors 13. Use of NSAIDs | 1.Coronary Angiography2.PCI | 59 | Increase in S. Creat. ≥0.5 mg/dL (44 µmol/L) or >25% increase above baseline within 48h of procedure | Iodixanol Iso-Osm | Continuous normal saline infusion was started immediately post-procedure at 0.5ml/kg/hr, stopped on day three | Theophylline Group | 1 h infusion of 205.7 mg theophylline in 500ml 0.9% normal saline | | Mean ±SD: 95±38 | | | Mean S. Creat. ±SD:  2.02±0.45mg/dL | |
|  |  |  |  |  |  |  | Control | 500ml 0.9% normal saline | | Mean ±SD: 94±35 | | | Mean S. Creat. ±SD:  2.06±0.59mg/dL | |
| Menting 2015(119) | Inclusion Criteria   1. Interventional or diagnostic radiological procedure expected to use >100 mL intravascular contrast 2. Fulfil risk criteria for CI-AKI according to Dutch guidelines  Exclusion Criteria   1. Age <18 years 2. Haemodialysis or peritoneal dialysis 3. Simultaneous participation in another interventional study 4. Percutaneous coiling/embolization procedures of the kidney 5. Impossibility to perform RIPC 6. No written informed consent | 1. Contrast Enhanced Radiological studies | 77 | Increase in S. Creat. ≥0.5 mg/dL (44 µmol/L) or >25% increase above baseline within 48h-72h of procedure | Xenetrix Low-Osm | Normal saline at 3-4ml/Kg/hour for 4h pre- and 4h post-procedure (reduced to 1ml/kg/h for 12h pre- and post-procedure if signs of CHF or eGFR <30ml/min/1.73m^2^) | RIPC | Four cycles of ischemia and reperfusion of the forearm by inflating a blood pressure cuff around the upper arm at 50 mmHg above the actual systolic pressure for 5 minutes, followed by 5 minutes of reperfusion | | Mean ±SD: 99±29 | | | Mean S. Creat. ±SD:  115±27mmol/L | |
|  |  |  |  |  |  |  | Placebo | Placebo Group: similar cuff placed around the upper arm, but it was not inflated | | Mean ±SD: 98±29 | | | Mean S. Creat. ±SD:  119±32mmol/L | |
| Merten 2004(120) | Inclusion Criteria   1. Age >18 years 2. Stable S. Creat. >1.1mg/dL 3. Scheduled cardiac catheterization, CT, diagnostic or therapeutic arteriography, or transjugular intrahepatic portal systemic shunt placement  Exclusion Criteria   1. S. Creat. >8mg/dL 2. Change in S. Creat. >0.5mg/dL within last 24 hours 3. Pre-existing dialysis 4. Multiple myeloma 5. Pulmonary oedema 6. Uncontrolled hypertension 7. Emergency catheterization 8. Recent exposure to radiographic contrast within 2days of the study 9. Allergy to radiographic contrast 10. Pregnancy 11. Administration of dopamine, mannitol, fenoldopam, or NAC during the study. | 1.Angiography  2.CT  3.TIPSS | 128 | >25% increase above baseline S. Creat. within 2days of procedure | Iopamidol Low-Osm | Assigned fluid at 3mL/kg per hour for 1h immediately before radiocontrast injection, followed by same fluid at 1mL/kg/h during contrast exposure and for 6 h post-procedure | NaCl | 154 mEq/L of sodium chloride in D5W | | Mean ±SD: 134±63 | | | Mean S. Creat. ±SD, (Range):  1.71±0.42 (1.1-3.7) mg/dL | |
|  |  |  |  |  |  |  | NaHCO3 | NaHco3 Group: 154 mEq/L of sodium bicarbonate in D5W | | Mean ±SD: 130±72 | | | Mean S. Creat. ±SD, (Range):  1.89±0.69 (1.2-5.2) mg/dL | |
| Miao 2013(121) | Inclusion Criteria  1. Age ≥ 70 years 2. Stable clinical state 3. Scheduled for contrast enhanced CT  Exclusion Criteria   1. Uncontrolled DM, hypertension, CHF (LVEF <40%), or other uncontrolled clinical diseases 2. Fever or infectious diseases 3. Unstable renal function or chronic renal failure [S. Creat >265.2 mmol/l] 4. Hypersensitivity to iodine-containing compounds or hyperthyroidism 5. Hypersensitivity to Alprostadil 6. Ulcers 7. Coagulation abnormalities 8. Other diseases with contraindications to alprostadil 9. Patients who had received any iodinated contrast agent or other drugs that affect renal function within 7 days | 1.CE-CT | 383 | Increase in S. Creat. ≥0.5 mg/dL (44 µmol/L) or >25% increase above baseline within 3days of procedure | Low-Osm Iohexol | I.V Fluid >800 ml 6h pre- and post-CE-CT | PGE1 | Alprostadil (0.4lg/kg/day) in 100 ml sterile saline pre- and post-procedure | | 100 | | | Mean S. Creat. ±SD:  87.18±24.6mmol/L | |
|  |  |  |  |  |  |  | Placebo | 100 ml sterile saline | | 100 | | | Mean S. Creat. ±SD:  88.11±24.19mmol/L | |
| Miner 2004(122) | Inclusion Criteria  1. Planned PCI or urgent coronary angiography with a high likelihood of ad hoc PCI. 2. Creatinine Clearance <50 mL/min, (<100mL/min if Diabetic) or S. Creat. >200 µmol/L   Exclusion Criteria  1. Renal replacement therapy 2. Reactive airway disease requiring oral steroids 3. Baseline SBP <80 mm Hg 4. Active CHF 5. Acute MI 6. Enrolment in another clinical trial 7. Inability to provide informed consent 8. Ongoing need for intravenous nitroglycerin 9. Treatment with NAC within 72 hours of planned PCI 10. Women of childbearing age | 1.Coronary Angiography2.PCI | 208 | >25% increase above baseline S. Creat. 48-72h | Omnipaque Iso-Osm | 0.45% saline at 75 mL/hour for at least 24 hours beginning at the time of enrolment. Changes in hydration were allowed at the discretion of the cardiologist | NAC | 6000mg (4000mg for day / case patients) | | Mean ±SD: 344±211 | | | Mean S. Creat. ±SD:  124±49mmol/L | |
|  |  |  |  |  |  |  | Placebo | IV hydration only | | Mean ±SD: 350±187 | | | Mean S. Creat. ±SD:  130±58mmol/L | |
| Minoo 2016(197) | Inclusion Criteria:  Patients ≥ 35 years undergoing elective coronary angiography  Exclusion Criteria:  1. Baseline serum creatinine concentrations greater than 1.5 mg/dL,  2. Need for emergency catheterization  3. Receiving contrast media for diagnostic or therapeutic interventions in the past 3 months  4. Uncontrolled congestive heart failure  5. Uncontrolled chronic obstructive pulmonary disease  6. History of allergy to contrast  Media  7. Pregnancy or lactation | 1.Elective coronary angiography | 453 | Increase of 25% or more in serum creatinine concentrations, or an increment of at least 0.5 mg/dL in serum creatinine concentrations 48 hours after catheterization | Iopromide  Low-Osm | normal saline at the dose of 1 mL/kg/h, 12 hours leading to the procedure and 12 hours after the procedure | Oxygen | nasal cannula at the rate of 2 L/min to 3 L/min beginning 10 minutes before the procedure until the end of the procedure | | Median (IQR):  200 (100-250 | | | Mean S. Creat. ±SD:  0.96 ± 0.24 | |
|  |  |  |  |  |  |  | Control | I.V hydration only | | Median (IQR):  150 (100-257) | | | Mean S. Creat. ±SD:  0.93 ± 0.15 | |
| Moore 2006(123) | Inclusion Criteria  1. Elective EVAR | 1.EVAR | 23 | >25% increase above baseline S. Creat. | Niopam Low-Osm | Median IV fluids received (IQR): NAC: 5.9 (3.0–5.75) L Control: 4 (3–7) L | NAC | 600 mg orally twice daily for 2 days, starting day pre-procedure (total 3 doses pre-op) | | Median (IQR): 258 (210-285) | | | Median S. Creat. (IQR):  102 (76-112) mmol/L | |
|  |  |  |  |  |  |  | Control | IV hydration only | | Median (IQR): 258 (200-355) | | | Median S. Creat. (IQR):  86 (81.5-99) mmol/L | |
| Morikawa 2009(124) | Inclusion Criteria   1. Chronic renal insufficiency (S. Creat. 1.3-6mg/dl)  Exclusion Criteria   1. Pregnancy 2. Lactation 3. Acute renal failure 4. End-stage renal failure on dialysis 5. Acute MI 6. Multiple myeloma 7. Pulmonary oedema 8. Cardiogenic shock 9. SBP < 110 mm Hg 10. Dehydration 11. History of allergies to contrast media or ANP 12. Received contrast media within 7 days of the study entry 13. Received an infusion of ANP within 1 month of the study entry 13. Parenteral use of diuretics 14. Administration of dopamine, N-acetylcysteine, metformin, NaHco3, fenoldopam, mannitol, or NSAIDs during the study | 1.Coronary Angiography2.PCI | 273 | Increase in S. Creat. ≥0.5 mg/dL (44 µmol/L) or >25% increase above baseline within 48h of procedure | Iomeprol Low-Osm | oral hydration encouraged | ANP | 0.042 µg/kg/min of ANP + 1.3ml/kg/h Ringer's Lactate IV 4-6h pre- and continued for 48h post-procedure | | Mean ±SD: 139±62 | | | Median S. Creat. (IQR):  1.57(1.34-1.95) mg/dL | |
|  |  |  |  |  |  |  | Control | IV hydration only | | Mean ±SD: 140±72 | | | Median S. Creat. (IQR):  1.55(1.36-1.90) mg/dL | |
| Motohiro 2011(125) | Inclusion Criteria  1. Age > 20years  2. eGFR < 60ml/min/1.73m2   Exclusion Criteria   1. S. Creat. >4 mg/dl 2. Change in S. Creat. >0.5 mg/dl in preceding 24 hours 3. Pre-existing dialysis 4. Pulmonary oedema 5. Uncontrolled hypertension (treated SBP >160 mmHg or DBP >100 mmHg) 6. Emergency catheterization 7. Exposure to radiographic contrast within previous 2 days 8.Any Allergy to radiographic contrast medium | 1.Coronary Angiography | 167 | Increase in S. Creat. ≥0.5 mg/dL (44 µmol/L) or >25% increase above baseline within 48h of procedure | Iopamidol Low-Osm | 0.9% sodium chloride at 1ml/kg/h for 12h pre- and post-procedure | NaHCO3 | 1ml/kg/hour continued from 3 hours pre- to 6 hours post-procedure | | Mean ±SD: 140±50 | | | Mean S. Creat. ±SD:  1.54±0.43mg/dL | |
|  |  |  |  |  |  |  | Control | IV hydration only | | Mean ±SD: 130±40 | | | Mean S. Creat. ±SD:  1.55±0.44mg/dL | |
| Nawa 2015(198) | Inclusion Criteria:  Patients with poor renal function and who had a high cystatin C level (> 0.95 mg/L in males and 0.87 mg/dL in females)  Exclusion Criteria:  1. End-stage renal failure on dialysis, a single functioning kidney, or history of kidney transplantation  2. Hypotension with systolic blood pressures below 100 mmHg  3. Acute myocardial infarction  4. Acute heart failure, left ventricular ejection fraction (LVEF) less than 30% on echocardiogram or evidenced by pulmonary edema  5.Multiple myeloma  6. Pregnancy  7. History of allergies to CM or nicorandil  8. Received CM within 7 days of study or nicorandil within 1month  9. Parenteral use of diuretics, and the administration of NAC, Metformin, NaHco3, theophylline, fenoldopam, mannitol, or a phosphodiesterase inhibitor during the study. | 1.Elective PCI | 204 | 25% increase in serum creatinine or an increase in creatinine of 0.5mg/dL from base- line at 48 h | Iomeprol  Low-Osm  Iohexol  Low-Osm | 0.9% saline hydration intravenously infused at 1.0 mL/kg/h (nicorandil group) or 0.9% saline infusion only at 1.1 mL/kg/h  Infusions were initiated 4 h prior to elective PCI and were continued for 24 h after the procedure | Nicorandil | 2 vials (48 mg/V) dissolve in 100 mL 0.9% saline, and dripped it at speed of 0.1 mL/kg/h) | | Mean+SD:  135.2±57.0 | | | Mean S. Creat. ±SD:  0.99±0.29 | |
|  |  |  |  |  |  |  | Control | I.V saline Only | | Mean+SD:  146.3±63.6 | | | Mean S. Creat. ±SD:  1.02±0.35 | |
| Ng 2006(126) | Inclusion Criteria   1. High risk for the development of CI-AKI  2. Pre-existing stable renal insufficiency (S. Creat. >1.2 mg/dL with no change greater than ±0.1m mg/dL)   Exclusion Criteria  1. Acute Renal failure 2. History of renal transplantation 3. Receiving N-acetylcysteine or fenoldopam 4. Known contraindication or hypersensitivity to N-acetylcysteine or fenoldopam 5. Pregnancy | 1.Coronary Angiography | 97 | Increase in S. Creat. ≥0.5 mg/dL (44 µmol/L) or >25% increase above baseline | Omnipaque Visipaque Hexabrix. | Normal saline (or 5% dextrose in normal saline in diabetics on insulin) at 1mL/kg/h beginning 1–2 h pre-procedure and continuing for 6–12h | NAC | 600 mg orally twice daily for 2 days, starting day pre-procedure (total 4 doses) | | Mean ±SD: 172.2±73.2 | | |  | |
|  |  |  |  |  |  |  | Fenoldopam | 0.1 mcg/kg/min IV commenCI-AKIg 1–2 h pre- and continued for 6h post-procedure | | Mean ±SD: 164.4±85 | | |  | |
| Nijssen 2017(199) | Inclusion Criteria:  Patients aged 18 years and older, referred for an elective procedure requiring intravascular iodinated contrast material with estimated glomerular filtration rate (eGFR) between 45 and 59 mL per min/1·73 m² combined with either diabetes, or at least two predefined risk factors (age >75 years; anaemia defined as haematocrit values <0·39 L/L for men, and <0·36 L/L for women; cardiovascular disease; non-steroidal anti-inflammatory drug or diuretic nephrotoxic medication); or eGFR between 30 and 45 mL per min/1·73 m²; or multiple myeloma or lymphoplasmacytic lymphoma with small chain proteinuria  Exclusion Criteria:  1. Inability to obtain informed consent  2. eGFR <30 mL per min/1·73 m²  3. Renal replacement therapy, emergency procedures  4. Intensive care patients  5. Known inability to plan primary endpoint data collection  6. No referral for prophylactic hydration  7. Participation in another randomised trial  8. Isolation (infection control). | 1.Elective procedure with intravascular contrast media administration | 599 | Increase in serum creatinine by more than 25% or 44 µmol/L within 2–6 days of contrast exposure (2–5 days was aimed for, but day 6 was allowed if no other option was available) | Iopromide  Low-Osm | See Groups | Hydration | standard protocol intravenous 0·9% NaCl 3–4 mL/kg per h during 4 h before and 4 h after contrast administration | | Contrast(ml) Mean(SD):  92 (41) | | | S. Creat.  Mean(SD):  118·78 (27·63) | |
|  |  |  |  |  |  |  | Non-Hydration (Control) | No Hydration | | Contrast(ml) Mean(SD):  89 (41) | | | S. Creat.  Mean(SD):  117·71 (24·62) | |
| Ochoa 2004(127) | Inclusion Criteria   1. Elective or urgent coronary angiography ± PCI  2. Chronic renal insufficiency (S. Creat. >1.8 mg/dL males, >1.6 mg/dL females), or Creat. Clearance <50mL/min   Exclusion Criteria   1. >0.5 mg/dL increase S. Creat. in preceding 6 weeks 2. Current or planned dialysis 3. Contrast exposure within preceding 48 hours 4. Known allergy to NAC 5. History of anaphylactic reaction to contrast 6. Recent decompensated CHF within preceding 4 weeks 7. Cardiogenic shock or use of intravenous vasopressors within preceding 1 week 8. Known or suspected severe aortic valve stenosis (area <1.0 m2, mean gradient >50 mmHg) 9. Recent (<4 weeks) initiation of diuretics or ACE inhibitors | 1.Coronary Angiography2.PCI | 94 | Increase in S. Creat. ≥0.5 mg/dL (44 µmol/L) or >25% increase above baseline within 48h of procedure | Ioxaglate Low-Osm Iohexol Low-Osm Iodixonal Iso-Osm | Normal saline at 150 mL/h beginning 4 hours pre- and continued for 6 hours post-procedure | NAC | 1000 mg [5 mL] diluted in 20 mL of diet cola)  administered orally 1h pre- and 4h post-procedure | |  | | | Mean S. Creat. ±SD:  2.02±0.56mg/dL | |
|  |  |  |  |  |  |  | Placebo | 5mL 0.9% normal saline diluted in 20 mL of diet cola)  administered orally 1h pre- and 4h post-procedure | |  | | | Mean S. Creat. ±SD:  1.93±0.53mg/dL | |
| Oguzhan 2013(128) | Inclusion Criteria   1. S. Creat. <2.1 mg/dL  Exclusion Criteria   1. Acute STEMI 2. NYHA class IV CHF 3. Hemodynamic instability (SBP<90mmHg on ≥2 measurements or patients requiring pressors) 4. Exposure to contrast within preceding 7 days 5. Use of a nephrotoxic drug within preceding 48h 6. Contraindication to amlodipine or valsartan | 1.Coronary Angiography | 101 | Increase in S. Creat. ≥0.5 mg/dL (44 µmol/L) or >25% increase above baseline within 48-72h of procedure | Iopromide  Low-Osm | Isotonic sodium chloride 1 mL/kg/h for 12h pre- and post-procedure | Amlodipine /Valsartan Group: | Amlodipine/Valsartan (5/160mg) mané commenCI-AKIg day prior to procedure (total 3 doses) | | Median (Range): 60 (30-200) | | | Mean S. Creat. ±SD:  1.13±0.33mg/dL | |
|  |  |  |  |  |  |  | Control | IV hydration only | | Median (Range): 60 (25-250) | | | Mean S. Creat. ±SD:  1.07±0.23mg/dL | |
| Oldemeyer 2003(129) | Inclusion Criteria   1. Age ≥19 years 2. Creat. clearance <50 mL/min and S. Creat > 1.2mg/dL 3. Elective coronary angiography  Exclusion Criteria  1. Acute kidney failure 2. Dialysis 3. Unstable renal function (Change ≥0.5 mg/dL or ≥25% in S. Creat. in preceding 10 days) 4. Known allergy to contrast or acetylcysteine 5. Administration of mannitol, intravenous catecholamines, parenteral diuretics, theophylline, or a contrast agent within days of study entry 6. Mechanical ventilation 7. Cardiogenic shock 8. Emergent angiography. | 1.Coronary Angiography | 103 | Increase in S. Creat. ≥0.5 mg/dL (44 µmol/L) or >25% increase above baseline within 24-48h of procedure | Iopamidol Low-Osm | (0.45%) saline at 1 mL/kg/h for 12h pre- and 12h post-procedure | NAC | 1500 mg BD orally in 120 mL of carbonated beverage commenced evening pre-procedure (total 4 doses) | | Mean ±SD: 134±71 | | |  | |
|  |  |  |  |  |  |  | Placebo | equivalent volume of normal saline BD orally in 120 mL of carbonated beverage commenCI-AKIg evening pre-procedure (total 4 doses) | | Mean ±SD: 127±73 | | |  | |
| Onbasili 2007(130) | Inclusion Criteria   1. S. Creat ≥1.2 mg/dl (≥106 mmol/l) or creat. clearance <50 ml/min   Exclusion Criteria  1. Acute renal failure 2. Acute MI requiring primary or rescue coronary intervention 3. Cardiogenic shock 4. Renal replacement treatments 5. Known allergy to Trimetazidine | 1.Coronary Angiography2.Angioplasty | 90 | Increase in S. Creat. ≥0.5 mg/dL (44 µmol/L) or >25% increase above baseline within 24-48h of procedure | Iopramide Low-Osm | Isotonic saline at 1 mL/kg/h for 12h pre- and 12h post-procedure | Trimetazidine | 20 mg TDS orally for 72h starting 48h pre-procedure | | Median: 225 | | | Mean S. Creat. ±SD:  1.31±0.25mg/dL | |
|  |  |  |  |  |  |  | Control | IV hydration only | | Median: 240 | | | Mean S. Creat. ±SD:  1.26±0.16mg/dL | |
| Ozcan 2007(131) | Inclusion Criteria   1. S. Creat. >1.2 mg/dL  Exclusion Criteria   1. Uncontrolled hypertension (SBP>160 mm Hg and DBP>110 mm Hg) 2. Emergency catheterization 3. Recent exposure to radiocontrast medium within preceding 2 days 4. Volume overload 5. S. Creat. levels >4 mg/dL | 1.Coronary Angiography2.PCI | 192 | Increase in S. Creat. ≥0.5 mg/dL (44 µmol/L) or >25% increase above baseline within 48h of procedure | Ioxaglate Low-Osm | Assigned fluid at 1 mL/kg/h (max 100ml/hr) for 6h pre- and 6h post-procedure | NaCl | 1 mL/kg/h (max 100ml/hr) for 6h pre- and 6h post-procedure | | Median (Range): 110 (30-270) | | | Mean S. Creat. (Range):  1.40 (1.2-2.3) mg/dL | |
|  |  |  |  |  |  |  | NaHCO3 | 154 mL of 1000-mEq/L NaHCO3 in 846 mL of D5W at 1 mL/kg/h (max 100ml/hr) for 6h pre- and 6h post-procedure | | Median (Range): 100 (50-300) | | | Mean S. Creat. (Range):  1.36 (1.2-3.8) mg/dL | |
|  |  |  |  |  |  |  | NAC+ NaCl: | Excluded from analysis | | | | | | |
| Ozhan 2010(132) | Inclusion Criteria:   1. Patients undergoing coronary angiography  Exclusion Criteria   1. Known allergy for contrast agents 2. Renal insufficiency (S. Creat. >1.5 mg/dL or eGFR <70 mL/min | 1.Coronary Angiography | 139 | Increase in S. Creat. ≥0.5 mg/dL (44 µmol/L) or >25% increase above baseline | Iopamidol Iso-Osm | 1L saline infusion in 6h post-procedure | Statin | 80 mg atorvastatin plus 600 mg NAC twice daily on day of procedure followed by 80 mg atorvastatin for 2 days after the procedure | |  | | | Mean S. Creat. ±SD:  0.88±0.2mg/dL | |
|  |  |  |  |  |  |  | Control | 600 mg NAC twice daily on day of procedure | |  | | | Mean S. Creat. ±SD:  0.88±0.19mg/dL | |
| Pakfetrat 2009(133) | Exclusion Criteria  1. Exposure to contrast media within preceding two days 2. Hypotension 3. Intra-aortic balloon pump 4. Pulmonary oedema 5. Dialysis 6. Electrolyte and acid-base disturbances 7. Known sensitivity to Acetazolamide 8. Medications affecting Renal Function e.g. mannitol, dopamine, and theophylline 9. Unwilling to give written informed consent | 1.Coronary Angiography2.PCI | 311 | RIFLE criteria: S. Creat. increased by 1.5-2 times baseline | Iodixanol Iso-Osm |  | NaHCO3 | 3 mL/kg of 154mEq/l NaHCO3 in Dextrose solution for 1 hour before coronary angiography, decreased to 1 mL/kg/hr for 6 hours post-procedure | | Mean ±SD: 58±32.7 | | | Mean S. Creat. ±SD:  1.1±0.3mg/dL | |
|  |  |  |  |  |  |  | NaCl Group: | 1 ml/kg/h starting 6h pre- and continued 6h post-procedure | | Mean ±SD: 67±41.1 | | | Mean S. Creat. ±SD:  1.1±0.2mg/dL | |
|  |  |  |  |  |  |  | Acetazolamide Group: | 250 mg orally 2h pre- and 6h post-contrast | | Mean ±SD: 70.1±67.9 | | | Mean S. Creat. ±SD:  1.1±0.3mg/dL | |
| Patti 2011(134) | Inclusion Criteria  1. Statin naive patients with non–STEMI or unstable angina 2. Planned intervention within 48 hours  Exclusion Criteria  1.Current or previous (<3 months) statin treatment 2. Non-STE ACS with high-risk features warranting emergency coronary angiography (<2 hours) 3. Any baseline increases in liver enzymes  4. LVEF <30% 5. Renal failure with S. Creat. > 3mg/dl 6. History of liver or muscle disease. | 1.Coronary Angiography2.PCI | 263 | Increase in S. Creat. ≥0.5 mg/dL (44 µmol/L) or >25% increase above baseline | Iobitridol Low-Osm | Patients with pre-existing renal failure (S. Creat. ≥1.5 mg/dl or CrCl ≥60 ml/min) received IV hydration with normal saline at 1ml/hour/kg for ≥12h pre- and ≥24 hours post-procedure | Statin | 80-mg loading dose of Atorvastatin given ~12h pre-procedure, with a further 40mg ~2h pre-procedure | | Mean ±SD: 209±72 | | | Mean S. Creat. ±SD:  79.8±29.4mmol/l | |
|  |  |  |  |  |  |  | Placebo | matched placebo | | Mean ±SD: 213±13 | | | Mean S. Creat. ±SD:  77±27.6mmol/l | |
| Poletti 2013(135) | Inclusion Criteria   1. Patients admitted as an emergency requiring CT 2. Creat. clearance <60 ml/min/1.73 m2 by MDRD (eGFR ~42 ml/min/1.73 m2) and need CT Scan   Exclusion Criteria  1. Asthma 2. Pregnancy  3. Obstructive nephropathy 4. Patient refusal | 1.CT | 128 | Increase in S. Creat. ≥0.5 mg/dL (44 µmol/L) or >25% increase above baseline | Low-Osm Iohexol (Accupaque®) | 250 ml of 0.45% NaCl pre-CT and 1L post-CT | NAC | 6000 mg NAC diluted in 100 ml 0.45% normal saline IV 1h pre-CT | | Mean ±SD: 117.4±1.8 | | | Mean S. Creat. ±SD:  132.4±34.8mmol/l | |
|  |  |  |  |  |  |  | Placebo | matched placebo | | Mean ±SD: 117.7±3.2 | | | Mean S. Creat. ±SD:  133.5±34.8mmol/l | |
| Qiao 2015(136) | Inclusion Criteria   1. Diabetes Mellitus 2. Mild-to-moderate chronic kidney disease (eGFR 30-89 ml/min/1.73 m2 3. Total contrast volume ≥ 100 ml  Exclusion Criteria   1. Pregnancy 2. Lactation 3. Ketoacidosis 4. Lactic acidosis 5. Contrast administration within 7 days of study  6. Emergent coronary angiography 7. History of hypersensitivity reaction to contrast or statins 8. NYHA class IV CHF 9. Unstable renal function 10. Use of: Aminophylline, Prostaglandin or E1 within 7 days of the procedure. 11. Recent statin use within 14 days | 1.PCI | 124 | Increase in S. Creat. ≥0.5 mg/dL (44 µmol/L) or >25% increase above baseline within 72h | Iso-Osm Iodixanol | Isotonic saline 0.9% at 1-1.15 mL/kg/h for 3-12h pre- and 6- 24 hours post-procedure | Statin | Rosuvastatin 10 mg/day) 2 days pre-and 3 days post-contrast | | Mean ±SD: 204.3±74.7 | | | eGFR 30-89 ml/min/1.73 m^2^ | |
|  |  |  |  |  |  |  | Control | IV hydration only | | Mean ±SD: 212.3±84.6 | | |  |  |
| Quintavalle 2012(137) | Inclusion Criteria  1. Statin-naïve patients 2. Elective coronary angiography due to symptomatic coronary artery disease OR PCI in de novo lesions in native coronary  Exclusion Criteria   1. Primary or rescue PCI 2. ACS with elevated cardiac biomarkers 3. Coronary artery restenosis 4. Treatment of a saphenous vein graft or left internal mammary artery graft 5. Active statin therapy | 1.Coronary Angiography2.PCI | 430 | Increase in S. Creat. ≥0.5 mg/dL (44 µmol/L) or >25% increase above baseline within 48h | Iodixanol Iso-Osm | NAC 1200mg orally twice daily, the days pre- and of contrast, as well as hydration with 154 mEq/L NaHCO3 in dextrose and H2O. with the initial IV bolus of 3 mL/kg/h for 1 hour immediately before CM injection, followed by 1 mL/kg/h during and for 6 h post-procedure | Statin | Atorvastatin (80 mg) within 24 hours | | Mean ±SD: 177±74 | | | Mean S. Creat. (Range):  1.16 (1.0-1.32) mg/dL | |
|  |  |  |  |  |  |  | Control | IV hydration only | | Mean ±SD: 184±78 | | | Mean S. Creat. (Range):  1.18 (1.0-1.35) mg/dL | |
| Rahman 2012(138) | Inclusion Criteria   1. Elective coronary angiography for ACD, chronic stable Angina, dilated/ischemic cardiomyopathy and preoperative assessment 2. S. Creat. 1.2-2.5mg/dl  Exclusion Criteria   1. Diabetes Mellitus 2. Acute renal failure  3. Acute MI requiring primary or rescue PCI 4. Cardiogenic shock 5. Patients on renal replacement therapy 6. Patients undergoing PTCA 7. COPD 8. Exacerbation of asthma  9. Allergy to TMZ | 1.Coronary Angiography | 436 | Increase in S. Creat. ≥0.5 mg/dL (44 µmol/L) or >25% increase above baseline within 24-48h | Iopamiro Low-Osm | Normal saline 1mg/kg/h for at least 12h pre- and 12h post-procedure | Trimetazidine | 35mg twice daily commenCI-AKIg 48h pre-procedure, for 96h | | Mean ±SD: 95.34±4.25 | | | Mean S. Creat. ±SD:  1.23±0.23mg/dl | |
|  |  |  |  |  |  |  | Control | IV hydration only | | Mean ±SD: 97.45±5.62 | | | Mean S. Creat. ±SD:  1.4±0.24mg/dl | |
| Rashid 2004(139) | Inclusion Criteria   1. Peripheral vascular disease  2. Elective angiography or angioplasty 3. Candidates subdivided into 2 groups: i. Normal S. Creat. (<120mmol/L[1.32mg/dl] for men and <97mmol/L[1.07mg/dl] for women) | 1.Peripheral Angiography  2.Angioplasty | 100 | Increase in S. Creat. ≥0.5 mg/dL (44 µmol/L) or >25% increase above baseline within 48h | Omnipaque Low-Osm | All patients received normal saline (500 mL over 4-6 hours) 6-12h pre-and post-procedure | NAC | 1g per bag of normal saline | | Mean ±SD: 135.4±62.7 | | | Mean S. Creat. ±SD:  109.9±41.15mmol/L | |
|  |  |  |  |  |  |  | Control | IV hydration only | | Mean ±SD: 151.2±75.6 | | | Mean S. Creat. ±SD:  124.3±63.47mmol/L | |
| Reinecke 2007(140) | Inclusion Criteria   1. S. Creat. 1.3-3.5 mg/dl  2. Elective coronary angiography≥ 1.3 mg/dl-3.5 mg/dl  Exclusion Criteria   1. Acute or recent MI within 30 days 2. NYHA class IV CHF 3. Recipient of transplanted organs 4. Monoclonal gammopathy 5. Previous contrast medium administration within 7 days | 1.Coronary Angiography | 431 | Increase in S. Creat. ≥0.5 mg/dL (44 µmol/L) 48-72h post-procedure | Iopromide Iso-Osm | 500 ml 5% glucose and 500 ml isotonic NaCl over 12h pre- and for 12h post-procedure | NAC | 600 mg evening pre-procedure, second dose morning pre-procedure, third evening post-procedure, and the last dose was given on morning post-procedure | | Mean ±SD: 197±80 | | | Mean S. Creat. (Range):  1.5 (1.3-1.9) mg/dl | |
|  |  |  |  |  |  |  | Dialysis | Haemodialysis performed within 20 min after catheterization | | Mean ±SD: 184±80 | | | Mean S. Creat. (Range):  1.5 (1.3-2.2) mg/dl | |
|  |  |  |  |  |  |  | Control | IV hydration only | | Mean ±SD: 188±79 | | | Mean S. Creat. (Range):  1.4 (1.3-1.9) mg/dl | |
| Rezaei 2016(200) | Inclusion Criteria:  patients aged ≥18 years with baseline estimated glomerular filtration rate (eGFR) <60 mL/min per 1.73 m2  Exclusion Criteria:  1. Acute ST-segment elevation  myocardial infarction or high-risk NSTE-ACS warranting emergency coronary angiography (<2 hours)  2. Cardiogenic shock  3. Pulmonary edema, overt heart failure and/or ejection fraction <30%  4. ACS undergoing coronary angiography or angioplasty during the previous 5 days  5. Sensitivity to contrast medium, recent administration of contrast medium for any reason  6. AKI or history of dialysis  7. Pregnancy  8. Newly prescribed angiotensin-converting enzyme inhibitors or angiotensin receptor blockers  9. Bleeding and/or coagulopathy disease  10 Consumption of nephrotoxic drugs, vitamin E, vitamin C, or NAC at least 48 hours before intervention | 1.Coronary Angiography | 329 | Absolute increase ≥0.5 mg/dL or a relative increase ≥25% over baseline serum creatinine concentration within 72 hours after administration of contrast media | Iodixanol  Iso-Osm | See Groups | vitamin E  (a-tocopherol) | 0.9% saline infusions (1 mL/kg) for 12 hours prior to and after coronary angiography combined with 600 mg oral vitamin E at 12 hours before plus 400 mg  2 hours before intervention | | Median (IQR):  1.3(1.2-1.5) | | | S CrMedian (IQR):  1.3(1.2-1.5) | |
|  |  |  |  |  |  |  | Placebo | Matching placebo (No Vit E) | | Median (IQR):  1.3(1.2-1.5) | | | S CrMedian (IQR):  1.3(1.2-1.5) | |
| Rohani 2010(141) | Inclusion Criteria  1. Chronic renal failure (stable S. Creat. >1.3 mg/dl)   Exclusion Criteria   1. Pregnancy 2. Contraindications to aminophylline (history of seizures, arrhythmia resulting in circulatory instability) | 1.Coronary Angiography | 70 | Increase in S. Creat. ≥0.5 mg/dL (44 µmol/L) 48h post-procedure | Omnipaque Low-Osm | Isotonic crystalloid 1.0-1.5 ml/kg/hr for 3-12 h pre- and for 6-24 hours post-procedure. Additional hydration was performed according to clinical examination, X-ray, and central venous pressures, if available. | Aminophylline | 250mg as a short infusion (100ml saline, 0.9%) 30 min pre-procedure | | Mean ±SD: 200±89 | | | Mean S. Creat. ±SD:  1.93±0.21mg/dl | |
|  |  |  |  |  |  |  | Placebo | Short infusion of 100 ml saline, 0.9% 30 min pre-procedure | | Mean ±SD: 210±90 | | | Mean S. Creat. ±SD:  1.84±0.54mg/dl | |
| Sadat 2011(142) | Inclusion Criteria  1. Peripheral Arterial Disease  Exclusion Criteria   1. Established renal failure 2. Renal replacement therapy | 1.Peripheral Angiography | 44 | >25% increase above baseline S. Creat. 72h post-procedure | Iopamidol  Low-Osm | 1L 0.9% normal saline IV infusion over 12h pre- and 1L over 12h post-procedure | NAC | 600 mg twice daily on the day pre- and 600 mg twice on the day of procedure | | Mean ±SD: 70±20 | | | Mean S. Creat. (Range):  97 (72-125) mmol/l | |
|  |  |  |  |  |  |  | Control | IV hydration only | | Mean ±SD: 75±25 | | | Mean S. Creat. (Range):  88 (68-142) mmol/l | |
| Sadineni 2017(201) | Inclusion Criteria:  Patients > 30 years undergoing coronary angiography ± PCI for angina, non-ST-segment elevation myocardial infarction (NSTEMI) and acute myocardial infarction/STEMI with serum creatinine ≥1.2 mg/dl on their most recent sample drawn within 3 months of planned procedure.  Exclusion Criteria:  1. Patients with acute renal failure or endstage renal disease requiring dialysis  2. Intravascular administration of contrast material within previous 6 days  3. Pregnancyor lactation  4. Emergency coronary angiography  5. History of hypersensitivity reaction to contrast media  6. Cardiogenic shock  7. Pulmonary edema  8. Mechanical ventilator  9. Parenteral use of diuretics  10 Recent use of NAC or ascorbic acid  11. Use of metformin or NSAIDS within 48 h of procedure | 1.Coronary angiography  2.PCI (non-emergency) | 118 | Relative increase in serum creatinine from baseline of ≥25% or an absolute increase of ≥0.3 mg/dl (44.2 µmol/L) during days 1 and 2. | Iodixanol  Iso-Osm | Normal saline 0.5 ml/kg/h 12 h prior to the procedure and was continued for 12 h after contrast administration (total 24 h). Patients who had low ejection fraction (<40%) received NS at rate of 0.3 ml/kg/h | NAC | | 600 mg orally twice daily, the day before and the day of the procedure | Mean+SD:  61.4±34.8 | | Mean S. Creat. ±SD:  2.24±0.9 | | |
|  |  |  |  |  |  |  | Allopurinol | | 300 mg single dose given the day before the procedure | Mean+SD:  68.7±46.77 | | Mean S. Creat. ±SD:  1.91±0.72 | | |
|  |  |  |  |  |  |  | Placebo | | Normal saline only | Mean+SD:  77.33±43.30 | | Mean S. Creat. ±SD:  2.19±1.01 | | |
| Saitoh 2011(143) | Inclusion Criteria   1. S Creat. >1.5 mg/dl ± creatinine clearance <60 ml/min 2. Elective coronary angiography | 1.Coronary Angiography | 23 | Increase in S. Creat. ≥0.5 mg/dL (44 µmol/L) or >25% increase above baseline within 48h | Iomeprol Low-Osm | 0.9% saline at 1 ml/kg/h for 24 h started 12 h pre-procedure and continued until 12h after | NAC | 704 mg twice daily orally from 1 day pre-procedure for a total of 2 days | | Mean ±SD: 117.1±9.0 | | |  | |
|  |  |  |  |  |  |  | Control | IV hydration only | | Mean ±SD: 113.6±14.5 | | |  |  |
|  |  |  |  |  |  |  | Glutathione | 100mg/min from 30min pre-procedure to max of 3000mg | | Mean ±SD: 130.7±19.3 | | |  |  |
| Sandhu 2006(144) | Inclusion Criteria   1. Non-coronary diagnostic angiography (head and neck, extremity and visceral abdominal angiography) 2. With or without renal impairment  Exclusion Criteria   1. Acute renal failure 2. Renal transplant | 1.Diagnostic angiography | 109 | Increase in S. Creat. ≥0.5 mg/dL (44 µmol/L) above baseline within 48h | Iodixanol Iso-Osm Iopamiol Low-Osm |  | NAC | 600 mg twice daily the day pre- and day of the procedure | |  | | | Mean S. Creat. ±SD:  116±48.9mmol/l | |
|  |  |  |  |  |  |  | Control | no intervention | |  | | | Mean S. Creat. ±SD:  103.6±48.6mmol/l | |
| Sanei 2014(145) | Inclusion Criteria  1. Elective CTA 2. Normal renal function  Exclusion Criteria   1. Unstable angina 2. MI 3. Cardiac arrhythmias 4. Heart failure 5. Acute or chronic renal failure 6. S. Creat. level >1.5 mg/dl 7. IV administration of contrast material in the past month 8. Known hypersensitivity to statins 9. Lost to follow-up | 1.CTA | 247 | Increase in S. Creat. ≥0.5 mg/dL (44 µmol/L) or >25% increase above baseline within 48h | Low-Osm Iopromide |  | Statin | 80mg dose of Atorvastatin daily commenCI-AKIg 24h pre- and continued for 48h post-procedure | | All patients received a total of 100 ml of the contrast material; 15 ml for the test bolus and 85 ml for the imaging (6ml/s injected with injector device) | | | Mean S. Creat. ±SD:  1.0±0.16mg/dl | |
|  |  |  |  |  |  |  | Placebo | matched placebo | |  |  |  | Mean S. Creat. ±SD:  1.03±0.17mg/dl | |
| Sar 2010(146) | Inclusion Criteria   1. Type 2 Diabetes Mellitus 2.Normal renal function  3. Elective radiological investigation requiring intravenous contrast media administration   Exclusion Criteria   1. BMI <21 or > 30 2. Concomitant systematic disease  3. Use of nephrotoxic drug or drug that can interact with ACEI within the last 30 days | 1.CT | 48 | Increase in S. Creat. ≥0.3 mg/dL or >20% increase above baseline, or >20% decrease in eGFR | Low-Osm Iohexaol | Normal saline 0.9% at 1ml/kg/h for 12h pre- and 24h post-procedure | NAC | 1200mg IV 1 h pre-procedure, 1200mg orally for 2 days | | 100 mg standard dose | | | Mean S. Creat. ±SD:  0.83±0.15mg/dl | |
|  |  |  |  |  |  |  | Placebo | IV hydration only | |  |  |  | Mean S. Creat. ±SD:  0.81±0.17mg/dl | |
| Savaj 2014(147) | Inclusion Criteria   1. Diabetic patients  Exclusion Criteria   1. CHF 2. Complications during angiography | 1.Coronary Angiography | 102 | Increase in S. Creat. ≥0.3 mg/dL or >30% increase above baseline at 24h |  | All of the patients received 1L of normal saline before procedure | RIPC | Blood pressure cuff placed around their non-dominant upper arm. The cuff inflated to 200-mm Hg pressure for 5 minutes, followed by 5 minutes of deflation and repeated for 3 cycles in total, starting 15min pre-procedure | | Mean ±SD: 126.6±77.2 | | | Mean S. Creat. ±SD:  1.3±0.4mg/dl | |
|  |  |  |  |  |  |  | Control | no intervention | | Mean ±SD: 123.8±66.6 | | | Mean S. Creat. ±SD:  1.1±0.3mg/dl | |
| Sedighifard 2016(202) | Inclusion Criteria:  Patients with mild to moderate risk for CIN were included in the study. referred for elective coronary angiography  Exclusion Criteria:  1. Unstable angina  2. Myocardial infarction  3. Cardiac arrhythmias  4. Acute or chronic renal insufficiency/failure (estimated glomerular filtration rate [eGFR] <60 mL/min/1.73 m2)  5. Acute or decompensate heart failure  6. Diabetes  7. Intravascular administration of contrast material in the past month | 1.Elective coronary angiography | 153 | Increase in serum creatinine of ≥0.5 mg/dL or ≥25% of the baseline creatinine after 48 h of contrast material injection | Iodixanol  Iso-Osm | 0.9% sodium chloride (1 mL/kg/h) for 12 h, started 6 h before and continued to 6 h after the procedure | Silymarin | Single dose (280 mg) tablet 2 h before administration of the contrast material. | | Average, 45 mL | | | Mean S. Creat. ±SD:  0.85±0.16 | |
|  |  |  |  |  |  |  | Placebo | Single dose match placebo tablet 2 h before administration of the contrast material. | | Average, 45 mL | | | Mean S. Creat. ±SD:  0.94±0.15 | |
| Sekiguchi 2013(148) | Inclusion Criteria   1. Elective coronary angiography ± PCI  Exclusion Criteria   1. ACS 2. End-stage renal failure 3. Dialysis 4. Cardiogenic shock 5. CHF 6. COPD 7. Oxygen saturation levels<90% | 1.PCI | 359 | >25% increase above baseline S. Creat. 48h post-procedure |  | 0.9% normal saline at 1 ml/kg/h 12h pre-procedure until 12h post-procedure | Oxygen | Administration via nasal cannula; 2 l/min of oxygen from 10 min before the procedure to the end of the procedure | |  | | |  | |
|  |  |  |  |  |  |  | Room air |  | |  |  |  |  |  |
| Seyon 2007(149) | Inclusion Criteria   1. Age ≥ 18 years  2. ACS 3. Renal dysfunction  (S. Creat. ≥1.4 mg/dL in males or ≥1.3 mg/dL in females; ± Creat. clearance < 50mL/min 4. Informed consent 5. Available for follow-up  Exclusion Criteria   1. Hemodynamic instability requiring inotropic support 2. Pregnancy  3. Acute gastrointestinal disorder (unable to tolerate oral medication) 4. NYHA class III or IV CHF  5. Unsuitable to receive intravenous hydration therapy as per cardiologist  6. Known sensitivity to NAC 7. Current treatment with theophylline or mannitol 8. Dialysis therapy 9. Participation in another study or use of experimental drug | 1.Coronary Angiography | 43 | Increase in S. Creat. ≥0.5 mg/dL (44 µmol/L) above baseline within 48h | Iohexol Low-Osm Iodixanol Iso-Osm | 0.45% normal saline at 1 mL/kg/h 4-6h pre- and 12h post-procedure | NAC | 600 mg orally, for a total of 4 doses, with the first dose at 8:00 A.M. the day of the procedure and 3 doses post-procedure | | Mean ±SD: 147.5±74.75 | | |  | |
|  |  |  |  |  |  |  | Placebo | matched placebo | | Mean ±SD: 133.68±58.04 | | |  |  |
| Shehata 2014(150) | Inclusion Criteria   1. Diabetes Mellitus  2. Chronic stable angina 3. Mild-to-moderate renal dysfunction (mean eGFR 48±16 ml/min/ 1.73m2)  Exclusion Criteria   1. Severe CKD (eGFR <30 ml/min/1.73m2) 2. End-stage renal disease 3. Hemodialysis 4. Acute MI requiring emergency coronary intervention 5. Cardiogenic shock 6. History of ACS, PCI or CABG 7. CHD or myocardial disease other than ischemia 8. Limited life expectancy 9. Positive pre-procedural cTnI result 10. Previous treatment with Trimetazidine 11. Contraindications for aspirin, Clopidogrel, or Trimetazidine Use 12. Parkinson disease and other motion disorders | 1.Elective PCI | 108 | Increase in S. Creat. ≥0.5 mg/dL (44 µmol/L) or >25% increase above baseline within 48h | Low-Osm Ultravist . | 0.9% Isotonic saline solution at 1 ml/kg/h starting 12h pre-procedure and up to 24h. N-acetylcysteine (1,200 mg) 24h pre- and post-procedure. | Trimetazidine | 35 mg twice daily for 72 hours, starting 48 hours before PCI | | Mean ±SD: 270±10 | | | Mean S. Creat. ±SD:  2.0±0.5mg/dl | |
|  |  |  |  |  |  |  | Placebo | IV hydration only | | Mean ±SD: 280±15 | | | Mean S. Creat. ±SD:  2.0±0.4mg/dl | |
| Shehata 2015(151) | Inclusion Criteria   1. Diabetes Mellitus  2. Mild-to-moderate chronic kidney disease  Exclusion Criteria   1. Severe CKD (eGFR <30 mL/min/1.73m2) 2. End-stage renal disease (or patients on hemodialysis) 3. Intake of potentially nephrotoxic drugs 4. Acute MI requiring emergency coronary intervention 5. Cardiogenic shock 6. Prior history of ACS 7. Prior history of PCI or CABG 8. Congenital heart disease or any myocardial disease apart from ischemia 9. Known skeletal muscle disorder 10. Chronic liver disease 11. Limited life expectancy due to coexistent disease 12. Contraindications for aspirin and/or clopidogrel | 1.PCI | 148 | Increase in S. Creat. ≥0.5 mg/dL (44 µmol/L) or >25% increase above baseline within 48h | Low-Osm Iopromide, Non-ionic Ultravist 370/100 | 0.9% Isotonic saline at 1 mL/kg/h starting 12 h pre- and up to 24 h post-procedure. NAC (1200 mg) administered to both groups 24 h pre- and post-procedure | Statin | 80mg dose of Atorvastatin daily for 48h pre-procedure | | Mean ±SD: 274±8 | | | Mean GFR of 48.5±16 mL/min/1.73 m^2^ | |
|  |  |  |  |  |  |  | Placebo | IV hydration only | | Mean ±SD: 278±11 | | |  |  |
| Shyu 2002(152) | Inclusion Criteria   1. Chronic renal insufficiency (S. Creat.2.0-6.0mg/dl or creat. clearance 8-40 ml/min)  2. Stable S. Creat. (difference of ≤0.1 mg/dl compared with baseline at 12 to 24 h before procedure)  Exclusion Criteria   1. Acute MI requiring primary or rescue PCI  2. Use of vasopressors before the procedure 3. Cardiogenic shock 4. Current peritoneal dialysis or haemodialysis 5. Planned post-contrast dialysis 6. Allergies to the study medications | 1.Coronary Angiography | 138 | Increase in S. Creat. ≥0.5 mg/dL (44 µmol/L) above baseline within 48h | Iopamidol Low-Osm | 0.45% normal saline IV at 1 ml/kg/h for 12 h pre- and 12 h post-procedure. All patients were encouraged to drink if they were thirsty. | NAC | 400 mg twice daily orally, on the day pre- and day of procedure, for a total of two days | | Mean ±SD: 119±3 | | | Mean S. Creat. ±SD:  2.8±0.8mg/dl | |
|  |  |  |  |  |  |  | Placebo | matched placebo | | Mean ±SD: 115±48 | | | Mean S. Creat. ±SD:  2.8±0.8mg/dl | |
| Singh 2016(187) | Inclusion Criteria:  patients with diabetes scheduled for elective PCI with eGFR60 ml/min/1.73m2 or urinary albumin creatinine ratio of >300 mg/g  Exclusion Criteria:  1. Patients with ST-elevation MI or decompensated heart failure in the preceding 6months  3. End stage renal disease on maintenance dialysis  4. Cerebrovascular disease  5. Chronic liver disease  6. chronic obstructive pulmonary disease  7. Gastrointestinal bleeding  8. Acute or chronic infection  9 Malignancy | 1.Elective PCI | 116 | Serum creatinine rise of ≥0.5mg/dl from baseline and/or an increase in creatinine of ≥25% from baseline within 48 hours after contrast exposure | Visipaque  Iso-Osm | Intravenous 0.9%NaCl infusion at 60ml/hour 6 hours before and after PCI procedure | RIPC | Right upper arm cuff manually inflated to  200mmHg for 5minutes, followed by deflation for 5minutes to allow reperfusion and this Cycle was performed 3 times 30minutes before the PCI. | | Mean+SD:  197.5 ± 114.3 | | | S. Creat.  Mean+SD:  1.42 ± 0.58 | |
|  |  |  |  |  |  |  | Placebo | Sham cuff applied for 30minutes with no inflation | | Mean+SD:  196.3 ± 118.8 | | | S. Creat.  Mean+SD:  1.41 ± 0.34 | |
| Solomon 1994(153) | Inclusion Criteria   1. S. Creat. > 1.6 mg/dL (140 μmol/L) or Creat. clearance < 60ml/min | Coronary Angiography | 63 | Increase in S. Creat. ≥0.5 mg/dL (44 µmol/L) above baseline within 48h | The choice of radiocontrast agent was made by the cardiologist | 0.45% normal saline IV at 1 ml/kg/h for 12 h pre- and 12 h post-procedure. All patients were encouraged to drink if they were thirsty. | Mannitol | 25g intravenously during the 60 minutes immediately before angiography | | Mean ±SD: 130±56 | | | Mean S. Creat. ±SD:  2.1±0.64mg/dl | |
|  |  |  |  |  |  |  | Furosemide | 80 mg IV during 30 min pre-procedure | | Mean ±SD: 132±48 | | | Mean S. Creat. ±SD:  2.1±0.59mg/dl | |
|  |  |  |  |  |  |  | Control | IV hydration only | | Mean ±SD: 125±45 | | | Mean S. Creat. ±SD:  2.1±0.54mg/dl | |
| Solomon 2015(203) | Inclusion Criteria:  Patients > 18 years scheduled for elective coronary or peripheral angiography, and if their calculated  eGFR was ,45 ml/min per 1.73 m2  Exclusion Criteria:  1. Hemodynamic  2. Instability per investigator judgment  3. Renal replacement thereby  4. Hypocalcaemia | Elective Coronary or peripheral angiography | 412 | ≥ 0.5mg/dl or 25%rise in creatinine from baseline during the first 3 days. | No details (Multicentre study) | Infusion rate in all groups were 5 ml/kg 60 minutes before and 1.5 ml/kg per h during and for 4 hours after angiography. In patients with a low ejection fraction (,40%), history of congestive heart failure, or significant oedema, the same total dose of study fluid could be infused over 5 hours post-angiography at the discretion of the investigator | NaHco3 | 50 ml ampules of 1 m sodium bicarbonate (154 mEq/L) and a partially filled 1 l bag with 830 ml sterile water | | Mean+SD:  110±66 | | | Mean S. Creat. ±SD:  1.98±0.62 | |
|  |  |  |  |  |  |  | Nacl | Standard 0.9% sodium chloride | | Mean+SD:  104±72 | | | Mean S. Creat. ±SD:  1.85±0.49 | |
| Spargias  2004(7) | Inclusion Criteria   1. Non-emergent coronary angiography  2. S. Creat. ≥1.2 mg/dL (106µmol/L) within 3 months of the planned procedure  Exclusion Criteria   1. Known acute renal failure 2. End-stage renal disease requiring dialysis 3. Intravascular administration of contrast medium within the previous 6 days 4. Anticipated re-administration of contrast medium within the following 6 days 5. Use of vitamin C supplements on a daily basis during the week before the procedure 6. Inability to administer the study medication at least 2h pre-procedure. | 1.Coronary Angiography  2.PCI | 265 | Increase in S. Creat. ≥0.5 mg/dL (44 µmol/L) or >25% increase above baseline within 2-5 days of contrast | The choice of the type of contrast agent was left to the interventional cardiologist performing the procedure, but use of a non-ionic, low- or iso-osmolar contrast agent was encouraged. | 50-125 mL/h normal saline IV from randomization until at least 6h post-procedure. All patients were encouraged to drink if they were thirsty | Ascorbic Acid | 3g chewable tablets at least 2 hours pre-procedure, followed by 2g the night and the morning post-procedure | | Mean ±SD: 287±148 | | | Mean S. Creat. ±SD:  1.52±0.64mg/dl | |
|  |  |  |  |  |  |  | Placebo | 3g chewable tablets at least 2 hours pre-procedure, followed by 2g the night and the morning post-procedure | | Mean ±SD: 261±128 | | | Mean S. Creat. ±SD:  1.50±0.54mg/dl | |
| Spargias  2006(155) | Inclusion Criteria   1. S. Creat. ≥ 1.4 mg/dL within 1 month  2. Elective procedure  Exclusion Criteria   1. Circulatory shock for any reason 2. SBP <95 mmHg 3. Acute renal failure 4. End-stage renal disease requiring dialysis 5. IV administration of contrast medium within preceding 10 days or anticipated re-administration of contrast medium within the following 6 days 6. Inability to administer study medication at least 30 min pre-procedure 6. Primary intervention for acute STEMI | 1.Coronary Angiography  2.PCI | 33 | Increase in S. Creat. ≥0.5 mg/dL (44 µmol/L) or >25% increase above baseline within 2 days of contrast | The choice of the type of contrast agent was left to the interventional cardiologist performing the procedure, but use of a non-ionic, low- or iso-osmolar contrast agent was encouraged. | 1.5 mL/kg/h normal saline for ≥ 4h pre-procedure and continued for at least 12h post-procedure | Iloprost | 1ng/kg/min beginning 30-90 min prior to and ending 4h post-procedure | | Mean ±SD: 217±118 | | | Mean S. Creat. ±SD:  1.71±0.7mg/dl | |
|  |  |  |  |  |  |  | Iloprost | 2ng/kg/min beginning 30-90 min prior to and ending 4h post-procedure | | excluded from analysis | | | | |
|  |  |  |  |  |  |  | Placebo | Normal saline beginning 30-90 min prior to and ending 4h post-procedure | |  | | | Mean S. Creat. ±SD:  1.66±0.64mg/dl | |
| Spargias  2009(154) | Inclusion Criteria   1. S. Creat. ≥1.4 mg/dL within 1 month before the planned procedure   Exclusion Criteria   1. Circulatory shock for any reason 2. SBP <95 mmHg 3. Acute renal failure 4. End-stage renal disease requiring dialysis 5. Intravascular administration of contrast medium within the previous 10 days or anticipated re-administration of contrast medium within the following 6 days 6. Inability to administer study medication at least 30 minutes before the procedure 7. Primary intervention for acute STEMI | 1.Coronary Angiography  2.PCI | 239 | Increase in S. Creat. ≥0.5 mg/dL (44 µmol/L) or >25% increase above baseline within 2 days of contrast | Use of a non-ionic, iso-osmolar, or low-osmolar contrast medium was encouraged. | 1.5 mL/kg/h normal saline for ≥ 4h pre-procedure and continued for at least 12h post-procedure | Iloprost | 1ng/kg/min beginning 30-90 min prior to and ending 4h post-procedure | | Mean ±SD: 257±129 | | | Mean S. Creat. ±SD:  1.63±0.49mg/dl | |
|  |  |  |  |  |  |  | Placebo | Normal saline beginning 30-90 min prior to and ending 4h post-procedure | | Mean ±SD: 249±137 | | | Mean S. Creat. ±SD:  1.59±0.49mg/dl | |
| Stone 2003(156) | Inclusion Criteria   1. Age > 18years 2. Risk for developing contrast-induced nephropathy (creat. clearance <60mL/min (1.00 mL/s) 3. Diagnostic ± interventional cardiology procedures.  Exclusion Criteria   1. Known severe allergy to contrast media or fenoldopam or its infusion components 2. Acute renal failure or unstable renal function 3. SBP < 100mmHg 4. Acute MI 5. Decompensated heart or respiratory failure 6. Contraindication to dopaminergic agents 7. Current use of mannitol or dopamine 8. Planned addition, discontinuation, or dose adjustment of trimethoprim, cimetidine, metoclopramide, bromocriptine, levodopa, nonsteroidal anti-inflammatory drugs, or catechol-O-methyltransferase inhibitors during the study 9. Exposure to iodinated contrast within the previous 10 days 10. Other serious medical conditions likely to interfere with data collection or follow-up 11. Participation in other investigational protocols within 30 days | 1.Diagnostic ± interventional cardiology procedures | 373 | Increase in serum creatinine level of ≥ 25% from baseline to the maximum value obtained at 24-96h | Low-Osm | 0.45% normal saline infusions at 1.5 mL/kg/h (or 1.0 mL/kg/h if heart failure was present) for 2-12h prior to trial | Fenoldopam | 0.05 µg/kg/min and increased in 20 minutes to 0.10 µg/kg/min if tolerated. The infusion was then maintained during angiography and percutaneous intervention and continued for 12 hours | | Mean ±SD: 153±107 | | | Mean S. Creat. ±SD:  1.82±0.71mg/dl | |
|  |  |  |  |  |  |  | Placebo | matched placebo | | Mean ±SD: 162±110 | | | Mean S. Creat. ±SD:  1.81±0.83mg/dl | |
| Stone 2011(157) | Inclusion Criteria  1. Age >18 years  2. CKD (Creat. clearance 20-50 ml/min) 3. Elective coronary arteriography (± PCI) 4. Use of iodinated contrast ≥50ml  Exclusion Criteria   1. Acute renal failure 2. Unstable renal function and current or planned dialysis 3. Renal artery angiography, renal drug infusion, or known renal artery stenosis 4. Use of mannitol or intravenous diuretics 5. Decompensated heart failure 6. Respiratory failure 7. Hypotension 8. Acute or recent STEMI 9. Allergy to Contrast Heparin, meperidine, or buspirone that could not be adequately premedicated 10. Recent monoamine oxidase inhibitor 11. Use of additional contrast administration 10 days before or after the procedure 12. Inferior vena cava filter 13. Height <1.5 m 14. Hypersensitivity to hypothermia (Raynaud) 15. Bleeding diathesis, coagulopathy or sickle cell disease 16. Hepatic impairment 17. Cryoglobulinemia 18. Untreated hypothyroidism 19. Addison disease 20. Prostatic hypertrophy or urethral stricture 21. Would refuse blood transfusions 22. Pregnancy 23. Inability or unwillingness to sign informed consent 24. Enrolment in another investigational drug or device trial 25. Condition possibly leading to noncompliance with any study procedures. | 1.Angiography  2.PCI  3.Renal Angiography | 157 | >25% relative increase above baseline within 96h of contrast, or increase in S. Creat. ≥0.5 mg/dL (44 µmol/L) | Iodixanol Low-Osm Other low osmolar contrast agents were permitted according to the operator’s choice. | 0.9% saline at 1.5 ml/kg/hour (1.0ml/kg/hour of 0.45% normal saline for patients with CHF or left ventricular dysfunction).  1h before the procedure NaCl was replaced with NaHCO3 (150 mEq in 1L D5W) at 0.45 mEq/kg (3ml/kg/hour) for the first hour and then 0.15 mEq/kg/hour (1 ml/kg/hour) for 5-7 hours after the procedure | Hypothermia | Reprieve endovascular temperature therapy system used to achieve a central temperature of 33°C-34°C before first contrast injection and for 3 hours after the procedure | | Mean ±SD: 150±94.3 | | | Mean S. Creat. ±SD:  12.7±3.0mg/dl | |
|  |  |  |  |  |  |  | Control | Normo-thermia. IV hydration only | | Mean ±SD: 138±78.3 | | | Mean S. Creat. ±SD:  12.1±2.2mg/dl | |
| Sun, C 2015(204) | Inclusion Criteria:  Patients > 18 years referred for coronary angiography and / or PCI  Exclusion Criteria:  1. Acute myocardial infarction requiring emergency catheterization  2. Chronic heart failure  3. Previous PCI or coronary artery bypass grafts  4. Contrast media within 7 days  5. Acute respiratory insufficiency  6. Acute renal failure  7. End-stage renal disease requiring dialysis  8. Patients on metformin, nonsteroidal anti-inflammatory drugs, diuretics, mannitol, dopamine, theophylline, ascorbic acid and N-acetylcysteine during the study period. | 1.Coronary angiography ± PCI | 141 | acute decline in renal function characterized by an absolute rise of 0.5 mg/dL (44.2 µmol/L) in SCr or a >25% increase from baseline to 48 hours after angiography | Iodixanol  Iso-Osm  Iopromide  Low-Osm | See Groups | BNP | 1.5 μg/kg bolus followed by an adjusted dose infusion of 0.01 μg/kg/ min | | Mean+SD:  145.56±75.86 | | | Mean S. Creat. ±SD:  75.46±17.25 | |
|  |  |  |  |  |  |  | I.V Hydration | isotonic saline (0.9%) at 6 hours before and 12 hours after the procedure | | Mean+SD:  149.36±73.28 | | | Mean S. Creat. ±SD:  75.10±14.59 | |
| Tamura 2009(158) | Inclusion Criteria   1. Patients with mild renal insufficiency (S. Creat. 1.1-2.0 mg/dl)  2. Elective coronary procedure   Exclusion Criteria   1. Allergy to contrast medium 2. Pregnancy 3. History of dialysis 4. Exposure to contrast medium within the preceding 48 hours of the study 5. ACS within the preceding 1 month of the study 6. NYHA class IV CHF 7.LVEF<25% 8. Severe chronic respiratory disease 9. Single functioning kidney 10. Administration of N-acetylcysteine, theophylline, dopamine or mannitol | 1.Diagnostic ± interventional cardiology procedures | 154 | Increase in S. Creat. ≥0.5 mg/dL (44 µmol/L) or >25% increase above baseline within 3 days of contrast | Iohexol Low-Osm | 0.9% Isotonic saline at 1ml/kg/h (0.5 ml/kg/h for patients with LVEF <40%) for 12h pre- and 12h post-procedure | NaHco3 Group: | Standard hydration with NaCl plus single-bolus IV administration of NaHCO3 [20 ml = 20 mEq], 5 minutes pre-procedure  CV/mL Mean(SD): 82.1± 40.4 | | Mean ±SD: 82.1±40.4 | | | Mean S. Creat. ±SD:  1.36±0.18mg/dl | |
|  |  |  |  |  |  |  | Control | IV hydration only | | Mean ±SD: 87.8±44.9 | | | Mean S. Creat. ±SD:  1.38±0.19mg/dl | |
| Tanaka 2011(159) | Inclusion Criteria   1. Primary Angioplasty for Acute MI  Exclusion Criteria   1. Dialysis 2. Known allergy to NAC 3. Inability to take NAC orally | 1.PCI | 83 | >25% relative increase above baseline within 72h of contrast | Iopamidol Low-Osm | Intravenous Ringer’s lactate solution at 1-2 mL/kg/h for >12h post-procedure | NAC | 705 mg orally before and at 12, 24, and 36h post-procedure (to a total of 2,820 mg) | | Mean ±SD: 205±80 | | | Mean S. Creat. ±SD:  0.88±0.25mg/dl | |
|  |  |  |  |  |  |  | Placebo | 4ml water | | Mean ±SD: 216±85 | | | Mean S. Creat. ±SD:  0.80±0.19mg/dl | |
| Tasanarong 2009(160) | Inclusion Criteria   1. S. Creat. ≥1.2 mg/dL or Creat. clearance ≤60mL/min  Exclusion Criteria   1. Renal Failure 2. End stage Renal disease requiring dialysis 3. Unstable renal function 4. Known allergy to contrast agents 5. Receiving mechanical ventilation 6. Congestive heart failure 7. Cardiogenic shock 8. Emergency angiography 9. Receiving NAC, mannitol, diuretics, theophylline, dopamine, ascorbic acid or contrast agents within 14 days before study 10. Use of α-or γ-tocopherol supplements on a daily basis during the week prior to the study. | 1.Coronary Angiography  2.PCI | 118 | Increase in S. Creat. ≥0.5 mg/dL (44 µmol/L) or >25% increase above baseline within 48h |  | 0.9% Isotonic saline at 1ml/kg/h for 12h pre- and 12h post-procedure | α-tocopherol | 525 IU daily commening 48h pre-procedure for total 3 doses. | | Mean ±SD: 150±83 | | | Mean S. Creat. ±SD:  1.62±0.44mg/dl | |
|  |  |  |  |  |  |  | Placebo | matched placebo | | Mean±SD: 132±58 | | | Mean S. Creat. ±SD:  1.67±0.53mg/dl | |
| Tasanarong 2013(161) | Inclusion Criteria  1. Chronic kidney disease   Exclusion Criteria  1. Acute Kidney Injury  2. CKD Stage 5 3. Unstable renal function 4. Known allergy to contrast agents 5. Receiving mechanical ventilation 6. Congestive heart failure 7. Cardiogenic shock 8. Emergency angiography 9. Receiving NAC, mannitol, diuretics, theophylline, dopamine, ascorbic acid or contrast agents within 14 days before study 10. Use of α-or γ-tocopherol supplements on a daily basis during the week prior to the study. | 1.PCI | 331 | Increase in S. Creat. ≥0.5 mg/dL (44 µmol/L) or >25% increase above baseline within 48h |  | 0.9% Isotonic saline at 1ml/kg/h for 12h pre- and 12h post-procedure | α-tocopherol | 350 mg/day 5 Days before and 2 days after procedure | | Mean±SD: 134±73 | | | Mean S. Creat. ±SD:  1.58±0.48mg/dl | |
|  |  |  |  |  |  |  | γ-tocopherol | 300 mg/day 5 Days before and 2 days after procedure | | Mean±SD: 137±75 | | | Mean S. Creat. ±SD:  1.48±0.48mg/dl | |
|  |  |  |  |  |  |  | Placebo | 350 mg/day 5 Days before and 2 days after procedure | | Mean±SD: 134±69 | | | Mean S. Creat. ±SD:  1.63±0.53mg/dl | |
| Tepel 2000(162) | Inclusion Criteria  1. Elective computed tomography (CT)  2. Chronic renal insufficiency (S. Creat. >1.2 mg/dL (106 µmol/L) or creat. clearance <50 ml/min)  Exclusion Criteria  1. Acute renal failure | 1.CT | 93 | Increase in S. Creat. ≥0.5 mg/dL (44 µmol/L) above baseline within 48h |  | 0.45% saline at 1ml/kg/h for 12h pre- and 12h post-procedure. Patients were encouraged to drink if thirsty. | NAC | 600 mg orally twice daily on the day prior to and day of CT | |  | | | Mean S. Creat. ±SD:  2.5±1.3mg/dl | |
|  |  |  |  |  |  |  | Control | IV hydration only | |  | | | Mean S. Creat. ±SD:  2.4±1.3mg/dl | |
| Thiele 2010(163) | Inclusion Criteria   1. Patients with ST-elevation undergoing primary angioplasty   Exclusion Criteria  1. Previous fibrinolysis <12h 2. Known NAC allergy 3. Chronic dialysis 4. Pregnancy 5. Contra- indications to magnetic resonance imaging (MRI) | 1.Coronary Angioplasty | 292 | >25% relative increase above baseline within 72h of contrast | Iopromide Low-Osm | 0.9% Isotonic saline at 1ml/kg/h (0.5 ml/kg/h for patients in overt heart failure) for 12h | NAC | Intravenous bolus of 1,200 mg before angioplasty and 1,200 mg intravenously twice daily for 48 h after (total dose 6,000 mg) | | Median (Range): 180 (140-230) | | | Median S. Creat. (Range):  81 (69-97) mmol/l | |
|  |  |  |  |  |  |  | Placebo | matched times - 10 ml of 0.9% NaCl at each injection | | Median (Range): 160 (120-220) | | | Median S. Creat. (Range):  78 (67-90) mmol/l | |
| Toso 2010(164) | Inclusion Criteria   1. Chronic kidney disease (estimated Creat. Clearance <60 ml/min) 2. Elective coronary angiography and/or other intervention  Exclusion Criteria   1. Current treatment with a statin 2. Contraindication to statin treatment 3. Previous contrast media administration within 10days of study entry 4. End-stage renal failure requiring dialysis 5. Informed refusal of consent | 1.Coronary Angiography | 353 | Increase in S. Creat. ≥0.5 mg/dL (44 µmol/L) or >25% increase above baseline within 5 days | Iodixanol Iso-Osm | 0.9% Isotonic saline at 1ml/kg/h (0.5 ml/kg/h for patients in overt heart failure) for 12h. Oral NAC (1200mg) twice daily from the day before to the day after the procedure | Statin | 80mg Atorvastatin daily starting 48h pre-procedure, continued for 48h post-procedure | | Mean±SD: 164±99 | | | Mean S. Creat. ±SD:  1.2±0.35mg/dl | |
|  |  |  |  |  |  |  | Placebo | matched placebo starting 48h pre-procedure, continued for 48h post-procedure | | Mean±SD: 151±95 | | | Mean S. Creat. ±SD:  1.18±0.33mg/dl | |
| Traub 2013(165) | Inclusion Criteria   1. Patients undergoing chest, abdominal, or pelvic CE-CT 2. Age > 18 years 3. ≥ one risk factor for CI-AKI  Exclusion Criteria   1. Unable or unwilling to provide written informed consent 2. End-stage renal disease 3. Pregnancy 4. Allergy to NAC  5. Clinical instability | 1.CE-CT | 383 | Increase in S. Creat. ≥0.5 mg/dL (44 µmol/L) or >25% increase above baseline within 48-72h of procedure | Low-Osm Isovue Low-Osm Optiray Iso_osm Visipaque | as per intervention protocol | NAC | 3g in 500mL NaCl as an IV bolus and then 200mg/h as 67mL/h for up to 24 hours | | Mean: 113.11 | | | Mean S. Creat. ±SD:  1.00±0.28mg/dl | |
|  |  |  |  |  |  |  | Placebo | 500mL NaCl as an IV bolus and then 200mg/h as 67mL/h for up to 24 hours | | Mean: 115.24 | | | Mean S. Creat. ±SD:  0.99±0.27mg/dl | |
| Trivedi 2003(166) | Inclusion Criteria  1. Age > 18 years  2. Elective Coronary Angiography  Exclusion Criteria   1. Creat. clearance < 20ml/min 2. Clinically decompensated heart failure 3. Decrease effective arterial volume (e.g. Nephrotic syndrome, liver cirrhosis) 4. Unwilling to participate | 1.Coronary Angiography | 63 | Increase in S. Creat. ≥0.5 mg/dL (44 µmol/L) above baseline within 48h of procedure |  | as per intervention protocol | IV fluid | 0.9% normal saline at 1ml/kg/h for 24h pre-procedure | |  | | | Mean S. Creat. ±SD:  101±21mmol/l | |
|  |  |  |  |  |  |  | Oral fluid | unrestricted oral fluid | |  | | | Mean S. Creat. ±SD:  112±33mmol/l | |
| Tumlin 2002(167) | Inclusion Criteria   1. Age ≥ 18 years  2. Moderate-severe renal failure (S. Creat. 2.0-5.0 mg/dL)   Exclusion Criteria   1. Uncontrolled ventricular arrhythmia 2. Chronic  hemodialysis or peritoneal dialysis 3. Metformin therapy 4. Acute cerebral vascular accidents 5. Oxygen saturation <90% on room air or possible overt pulmonary edema 6. Known glaucoma 7. Unstable clinical condition that would not allow for 3 hours of pre-hydration 8. S. Creat. <2.0mg/dL or >5.0 mg/dL 9. Known hypersensitivity to fenoldopam mesylate 10. Informed consent not possible 11. Patients who received prophylactic furosemide, mannitol, or renal dose dopamine before randomization 12. Pregnancy or lactation | 1.Coronary Aniography  2.Peripheral Angiography | 59 | Increase in S. Creat. ≥0.5 mg/dL (44 µmol/L) or >25% increase above baseline within 48 hours of contrast | Low-Osm Iso-Osm | Pre-hydration with 1/3 normal saline at 100 mL/h for 3h pre-procedure | Fenoldopam | 0.1 µg/kg/min starting 1h pre-procedure and continued for 4 hours with 1/3 normal saline at 100ml/h | | Mean±SEM: 80±15 | | | Mean S. Creat. ±SEM:  13±56mg/dl | |
|  |  |  |  |  |  |  | Control | normal saline only at same time and schedule | | Mean±SEM: 96±19 | | | Mean S. Creat. ±SEM:  11±50mg/dl | |
| Vasheghani-Farahani 2009(169) | Inclusion Criteria   1. Elective coronary angiography 2. S. Creat. ≥1.5 mg/dL  Exclusion Criteria   1. Unstable serum creatinine 2. Previous history of dialysis 3. eGFR <20ml/min 4. Emergency catheterization 5. Recent exposure to radiocontrast agent within 2 days prior to study  6. Allergy to contrast agent 7. Pregnancy 8. Administration of Dopamine, Fenoldopam or NAC during the intended time of the study 9. Need for continuous hydration | 1.Coronary Angiography | 281 | Increase in S. Creat. ≥0.5 mg/dL (44 µmol/L) or >25% increase above baseline within 48 hours of contrast | Iohexol Low-Osm | 3ml/kg for 1 hour immediately before contrast injection followed by infusion of 1ml/kg/h for 6h post-procedure | NaCl | 1075 ml of 0.45% Normal Saline at specified rate | | Mean±SD: 125±96.2 | | | Mean S. Creat. ±SD:  1.63±0.32mg/dl | |
|  |  |  |  |  |  |  | NaHCO3 | 75ml of 8.4% NaHCO3 added to 1L of 0.45% Normal Saline | | Mean±SD: 129±96.3 | | | Mean S. Creat. ±SD:  1.66±0.50mg/dl | |
| Vasheghani-Farahani 2010(168) | Inclusion Criteria   1. Elective coronary angiography 2. S. Creat. ≥1.5 mg/dL 3. Uncontrolled hypertension 4. Compensated severe heart failure 5. History of pulmonary oedema  Exclusion Criteria   1. Unstable serum creatinine 2. Previous history of dialysis 3. eGFR <20ml/min 4. Emergency catheterization 5. Recent exposure to radiocontrast agent within 2 days prior to study  6. Allergy to contrast agent 7. Pregnancy 8. Administration of Dopamine, Fenoldopam or NAC during the intended time of the study 9. Need for continuous hydration | 1.Coronary Angiography | 76 | Increase in S. Creat. ≥0.5 mg/dL (44 µmol/L) or >25% increase above baseline within 48 hours of contrast | Iohexol Low-Osm | 3ml/kg for 1 hour immediately before contrast injection followed by infusion of 1ml/kg/h for 6h post-procedure | NaCl | 1075 ml of 0.45% Normal Saline at specified rate | | Mean±SD: 123±31 | | | Mean S. Creat. ±SD:  1.77±0.52mg/dl | |
|  |  |  |  |  |  |  | NaHCO3 | 75ml of 8.4% NaHCO3 added to 1L of 0.45% Normal Saline | | Mean±SD: 112±33 | | | Mean S. Creat. ±SD:  1.71±0.45mg/dl | |
| Vogt 2001(170) | Inclusion Criteria   1. Chronic stable renal insufficiency 2. S. Creat. >200 µm/L (>2.3 mg/dL) | 1.Renal angioplasty  2.Peripheral angioplasty  3.CE-CT  4.Coronary angiography | 143 | Increase in S. Creat. ≥0.5 mg/dL (44 µmol/L) or >25% increase above baseline within 6 days | Low-Osm | I.V. saline at 1ml/kg/h for 12h pre-procedure | Dialysis | Haemodialysis starting as soon as possible after radiographic investigation | | Mean±SD: 210±143 | | | Mean S. Creat. ±SD:  308±106mmol/l | |
|  |  |  |  |  |  |  | Control | I.V. saline at 1ml/kg/h for 12h pre-procedure | | Mean±SD: 143±115 | | | Mean S. Creat. ±SD:  316±112mmol/l | |
| Wang, C 2015(205) | Inclusion Criteria:  18–80 years old undergoing non-emergency coronary angiography and/or intervention  Exclusion Criteria:  1. Serum creatinine ≥8 mg/dl(707mmol/L)  2. Known acute kidney injury, current peritoneal or haemodialysis  3. Acute myocardial infraction or cardiogenic shock  4. Hypersensitivity to iodine-containing compounds  5. Intravascular administration of contrast medium within the previous 1 week  6. Use of antioxidant on a daily basis during the week before the procedure  7. Pregnancy or lactation  8. Malignancy | 1.Non –emergency Coronary angiography | 866 | Absolute increase of serum creatinine concentration of at least 0.5mg/dl (44.2mmol/l) or a relative increase of at least 25% from baseline within 48 hours after the procedure. | Iopamidol  Iso-Osm | 1 ml/ kg body weight per hour intravenous normal saline 500 ml was administered in all patients at the beginning of the procedure | Glutathione | 1.8 g reduced glutathione in 500 ml of normal saline | | Mean+SD:  135.6±70.1 | | | Serum Cr (mmol/L):  74(64,84) | |
|  |  |  |  |  |  |  | Placebo | Saline | | Mean+SD:  132.8±66.7 | | | Serum Cr (mmol/L):  75 (63.3,84.0) | |
| Wang 2000(171) | Inclusion Criteria   1. Adult patients undergoing cardiac angiography 2. S. Creat. ≥2.0 mg/dL (176.8 µmol/L) within 48h of procedure  Exclusion Criteria   1. SBP <100/70 mm Hg or heart rate >100 bpm at baseline 2. Acute renal failure 3. Chronic renal failure requiring dialysis 4. Inability to adhere to the hydration regimen 5. Diuretic therapy within 12h or during infusion of study drug 6. Dopamine therapy within six hours or during infusion of study drug 7. Administration of NSAIDs other than aspirin (≤325 mg/day) within 24h of study drug 8. Uncontrolled cardiac arrhythmia 9. Hepatic dysfunction 10. Cerebrovascular accident within 1 week 11. Women of child-bearing potential | 1.Coronary Angiography | 225 | Increase in S. Creat. ≥0.5 mg/dL (44 µmol/L) or >25% increase above baseline within 48 hours of contrast | Low-Osm Type determined by consultant | 0.45% saline at 1ml/kg/h beginning 2-12h pre- and continuing for ≥ 12h post-procedure. Patients were encouraged to drink if thirsty. | ERAs Group: Endothelin A and B receptor antagonist (SB 290670) | 100 µg/kg over 10min, then 1.0µg/kg/min starting 30-150min pre-contrast, and continued for 12 hours after CV/mL Mean±SD: 104.0 64.8 | | Mean±SD: 104±64.8 | | | Mean S. Creat. ±SD:  2.7±0.9mg/dl | |
|  |  |  |  |  |  |  | Placebo | matched placebo | | Mean±SD: 122.4±86.2 | | | Mean S. Creat. ±SD:  2.8±1.1mg/dl | |
| Wang 2011(172) | Inclusion Criteria   1. Patients with ST-Segment elevation undergoing primary PCI  Exclusion Criteria   1. Cardiogenic shock 2. Heart rate > 100bpm on admission  3. End stage renal disease requiring dialysis  4. Not agree to consent 5. Known allergic condition 6. Candidate not suitable to use anisodamine | 1.PCI | 143 | Increase in S. Creat. ≥0.5 mg/dL (44 µmol/L) or >25% increase above baseline within 48-72 hours of contrast | Ultravist Low-Osm | Normal saline at 1ml/kg/h pre-procedure and for 24h after | Anisodamine | 50 µg/kg bolus dose followed by adjusted dose (0.1-0.2 µg/kg/min) for 24h after | | Mean±SD: 256.8±71.9 | | | Mean S. Creat. ±SD:  83.1±17.7mmol/l | |
|  |  |  |  |  |  |  | Placebo | matched placebo | | Mean±SD: 259.8±79.1 | | | Mean S. Creat. ±SD:  82.2±14.1mmol/l | |
| Webb 2004(173) | Inclusion Criteria   1. Renal dysfunction (eGFR < 50mL/min)  2. Cardiac catheterization  Exclusion Criteria   1. Suspected acute renal failure 2. S. Creat. >400µmol/L 3. Current dialysis 4. Hemodynamic instability 5. NAC administration within 48 hours 6. Age <18 years 7. Lack of informed consent 8. Inability to comply with follow-up 9. Recent creatinine elevation after a diagnostic angiogram | 1.Coronary Angiography  2.PCI | 496 | >25% relative increase above baseline S. Creat. | Ioversol Low-Osm | 200ml isotonic saline pre-procedure, then at 1.5 mL/kg/h for 6h, unless contraindicated | NAC | 500 mg I.V. immediately pre-procedure | | Median (IQR): 120 (80-186) | | | Median (IQR): 141 (125-166) mmol/l | |
|  |  |  |  |  |  |  | Placebo | IV hydration only | | Median (IQR): 120 (80-155) | | | Median (IQR): 142 (124-167) mmol/l | |
| Weisberg 1993(174) | Inclusion Criteria   1. Stable S. Creat. ≥1.8mg/L  2. Elective cardiac catheterization  Exclusion Criteria   1. NYHA class IV CHF 2. Haemodynamic instability 3. Allergy to radiocontrast agent or prior exposure within the last 7days 4. Pregnancy | 1.Cardiac Catheterization | 41 | >25% relative increase above baseline S. Creat. | MD 76 Hi-Osm | as per intervention protocol | Dopamine | 2µ/kg/min in 0.45% Nacl | |  | | | Mean S. Creat. ±SD:  2.37±0.17mg/dl | |
|  |  |  |  |  |  |  | Control | 0.45% normal saline 100ml/h starting 12h pre-procedure, continued throughout procedure and for total of 120 min | |  | | | Mean S. Creat. ±SD:  2.66±0.15mg/dl | |
| Wrobel 2010(175) | Inclusion Criteria   1. Patients with cardiovascular disease 2. Diabetes Mellitus   Exclusion Criteria   1. Contraindications for invasive procedures 2. Pregnancy 3. Breast-feeding 4. Symptoms and signs of infection 5. Antibiotic treatment 6. Participation in other studies within the preceding 30 days 7. History of hypersensitivity to contrast agents 8. Cancer  9. Acute renal failure of alternative aetiology | 1.Coronary Angiography  2.PCI | 107 | Increase in S. Creat. ≥0.5 mg/dL (44 µmol/L) or >25% increase above baseline | Loversol Low-Osm | as per intervention protocol | IV fluid | Intravenous infusion of isotonic 0.9% NaCl at 1 mL/kg/h started 6h pre-procedure, continued up to 12h post-procedure | | Mean±SD: 101.1±36.62 | | | Mean S. Creat. ±SD:  1.235±0.4454mg/dl | |
|  |  |  |  |  |  |  | Oral fluid | Weight-adjusted quantity of neutral fluid (commercially available still mineral water or boiled water) administered at 1mL/kg/h between 12-6 hours pre-procedure, continued up to 12h post-procedure | | Mean±SD: 110.4±65.28 | | | Mean S. Creat. ±SD:  1.172±0.3872mg/dl | |
| Xu, R. H 2013(206) | Inclusion Criteria:  Patients (aged ≥60 years) with coronary artery disease who were admitted for PCI  Exclusion Criteria:  1. Refusal to participate  2. Refusal of PCI  3. Use of any nephrotoxic drugs during the perioperative period  4. Severe hepatic and renal failure  5. Serious infectious disease  6. New York Heart Association Functional Classification (NYHA) >3  7. Hemodynamic instability (including systolic blood pressure <90 mmHg  8. Coronary lesions below the threshold for clinical revascularization therapy  9. Coronary lesions not suitable for PCI due to coronary anatomy  10 Allergic reaction to contrast media and alprostadil | 1.PCI | 67 | Relative increase of >25% or an absolute increase of ≥0.5 mg/dl in SCr from the baseline value 72 h after exposure to the contrast medium | Iopromide  Low-Iso | 1 ml/kg/h normal saline for 6 h prior to PCI and 12 h following PCI | Alprostadi | | 10 µg (diluted with 100 ml normal saline) twice a day by intravenous drip for the 3 days following PCI. | Mean+SD:  133.71±32.46 | | Mean S. Creat. ±SD:  83.63±23.59 | | |
|  |  |  |  |  |  |  | Control | | I.V hydration Only | Mean+SD:  123.57±37.14 | | Mean S. Creat. ±SD:  76.82±19.45 | | |
|  |  |  |  |  |  |  | The original control Group in this study were excluded | | No intervention | Mean+SD:  134.09±36.99 | | Mean S. Creat. ±SD:  88.27±27.40 | | |
| Xu 2014(176) | Inclusion Criteria   1. Diabetes Mellitus  2. Age ≥65 years  Exclusion Criteria   1. Previous use of trimatezidine, nicorandil or glibenclamide 2. Uncontrolled Diabetes Mellitus  3. Elevated cardiac biomarker at admission 4. MI within 6 months, stent implantation or CABG within 6 months 5. Cardiogenic shock 6. LVEF <50% 7. Congenital or valvular heart disease requiring further surgery 8. Moderate or severe renal insufficiency with eGFR <30 mL/min 9. Ongoing bleeding, or a history of bleeding diathesis 10. Expected life span <12months | 1.Coronary Angiography | 207 | >25% relative increase above baseline S. Creat. |  |  | RIPC | Blood pressure cuff placed around their non-dominant upper arm within 2h pre-procedure. The cuff inflated to 200-mm Hg pressure for 5 minutes, followed by 5 minutes of deflation and repeated 2 more times to 3 cycles in total | | Mean±SD: 171.8±37.9 | | | Mean eGFR ±SD:  99.1±20.6mmol/l | |
|  |  |  |  |  |  |  | Control | no cuff | | Mean±SD: 163.3±39 | | | Mean eGFR ±SD:  100.8±28.2mmol/l | |
| Yamanaka 2015(177) | Inclusion Criteria   1. Age ≥ 20 years  2. Suspected STEMI undergoing PCI  Exclusion Criteria   1. Left bundle branch block 2. Previous CABG 3. Severe heart failure requiring percutaneous cardiopulmonary support 4. Severe chronic kidney disease requiring dialysis or continuous hemodiafiltration | 1.PCI | 109 | Increase in S. Creat. ≥0.5 mg/dL (44 µmol/L) or >25% increase above baseline 48-72h post-procedure | low-osm contrast media used in all cases | 0.9% Isotonic saline at 1ml/kg/h (0.5 ml/kg/h for patients in overt heart failure) beginning 24h pre-PCI and continuing for 24h | RIPC | Blood pressure cuff placed around their non-dominant upper arm. The cuff inflated to 200-mm Hg pressure for 5 minutes, followed by 5 minutes of deflation and repeated 2 more times to 3 cycles in total | |  | | | Mean S. Creat. ±SD:  0.82±0.21mg/dl | |
|  |  |  |  |  |  |  | Control | no cuff | |  | | | Mean S. Creat. ±SD:  0.87±0.44mg/dl | |
| Yang 2014(178) | Inclusion Criteria   1. Age > 18 years  2. Elective cardiovascular procedures  Exclusion Criteria   1. Severe renal dysfunction (eGFR <30 mL min) 2. Chronic renal failure-induced incomplete dialysis therapy 3. Exposure to radiographic contrast within 10 days 4. A second contrast-enhanced procedure planned within 72h 5. Previous emergent PCI 6. Heart failure and LVEF<40% 7. Previous CABG 8. Preoperative SBP<90mmHg 9. Kidney transplantation or congenital unilateral renal agenesis 10. Use of nephrotoxic medications  11. Valvular disorders 12. Active cancer 13. Uncontrolled hypertension 14. Previous dialysis 15. Autoimmune diseases 16. Chronic or acute infectious disease 17. Overt CHF 18. Recent acute kidney injury 19. Allergy to radiographic contrast combined with severe liver and lung diseases 20. Gastrointestinal bleeding 21. Infection 22. CarCI-AKIoma 23. Anaemia (Hb < 9gm/L) 24. Hyperthyroidism 25. Autoimmune diseases | 1.Coronary Angiography  2.PCI | 655 | Increase in S. Creat. ≥0.5 mg/dL (44 µmol/L) or >25% increase above baseline 3days post-procedure | Iopromide Low-Osm | as per intervention protocol | NaCl | 0.9 % sodium chloride at 1.5mL/kg/h 6h pre-procedure, and continued for 6h after | | Mean±SD: 124±63.81; | | | Mean eGFR. ±SD:  93.46±22.45 ml/min/1.73m^2^ | |
|  |  |  |  |  |  |  | NaHCO3 | 1.5 % NaHCO3 at 1.5 mL kg/h for 6h pre-procedure, continuing for 6h after | | Mean±SD: 127±48.09; | | | Mean eGFR. ±SD:  92.77±22.98 ml/min/1.73m^2^ | |
|  |  |  |  |  |  |  | NaCl | 0.9 % sodium chloride at 1.5mL/kg/h 6h pre-procedure, and continued for 6h after plus 600mg NAC BD 24h pre- and post-procedure | | Mean±SD: 129±46.77 | | | Mean eGFR. ±SD:  93.84±21.98 ml/min/1.73m^2^ | |
|  |  |  |  |  |  |  | NaHCO3 | 1.5 % NaHCO3 at 1.5 mL kg/h for 6h pre-procedure, continuing for 6h after plus 600mg NAC BD 24h pre- and post-procedure | | Mean±SD: 126±57.97 | | | Mean eGFR. ±SD:  92.76±23.05 ml/min/1.73m^2^ | |
| Yavari 2014(179) | Inclusion Criteria   1. Age 18-65 years 2. Baseline S. Creat. ≤132.6 lmol/l (1.5mg/dl)  Exclusion Criteria   1. Acute MI 2. Congestive heart failure 3. Hemodynamic instability during or after the procedure 4. Known allergy or previous administration of Pentoxifylline, and use of concomitant nephrotoxic agents (e.g. NSAIDs, aminoglycosides 5. Recent contrast injection 6. Diuretics | 1.PCI | 211 | >25% relative increase above baseline S. Creat. at 48h post-procedure | iso-osm Iodixanol | 0.9% saline at 1ml/kg/h for 6 h prior to, during, and 6h post-procedure | Pentoxyfylline | 400mg TDS starting day of procedure and day after | | Mean±SD: 191.96±94.32 | | | Mean S. Creat. ±SD:  1.06±0.16mg/dl | |
|  |  |  |  |  |  |  | IV fluid | Normal Saline only | | Mean±SD: 185.88±81.06 | | | Mean S. Creat. ±SD:  1.04±0.16mg/dl | |
| Yeganehkhah 2014(180) | Inclusion Criteria   1. Patients undergoing coronary angiography with at least one of the following risk factors:  i) Congestive heart failure (LVEF <40%) ii) History of Diabetes Mellitus iii) Age >65 years iv) Renal failure (eGFR <60 mL/min/1.73m2)  Exclusion Criteria   1. Pregnancy and lactation 2. History of allergic reaction to contrast agents 3. Cardiogenic shock 4. Pulmonary edema 5. Multiple myeloma 6. Mechanical ventilation 7. Urgent coronary angio¬graphy 8. S. Creat. >4 mg/dL 9. End-stage re¬nal disease (ESRD) 10.Receiving contrast agents two days prior to the study and 48h after 11. Using diuretics, NAC, sodium bicarbonate, theophylline, dopamine, mannitol, fenoldopam, metformin, and NSAIDs during the study 12. DBP >100mmHg  13. Need for further fluid therapy 14. Hypertension | 1.Coronary Angiography | 183 | >25% relative increase above baseline S. Creat. at 48h post-procedure | Iow-Osm Iohexol | 150 mL 8.4% NaHCO3 added to 850 mL isotonic normal saline | NaHCO3 | 3 mL/kg/h | |  | | | Mean S. Creat. ±SD:  1.17±0.32mg/dl | |
|  |  |  |  |  |  |  | NAC | NAC 600mg twice daily orally starting one day pre-procedure, in addition to isotonic normal saline at 1ml/kg/h (max 100ml/h) for 12h before and after angiography | | Mean±SD: 45.7±21.6 | | | Mean S. Creat. ±SD:  1.17±0.43mg/dl | |
|  |  |  |  |  |  |  | IV fluid | Isotonic normal saline at 1ml/kg/h (max 100ml/h) for 12h before and after angiography | | Mean±SD: 41.9±17.7 | | | Mean S. Creat. ±SD:  1.08±0.32mg/dl | |
| Yin 2013(181) | Inclusion Criteria   1. Consecutive patients admitted to the coronary care unit including:  i) Acute STEMI requiring PCI  ii) Acute NSTEMI requiring urgent coronary intervention | 1.PCI | 231 | Increase in S. Creat. ≥0.5 mg/dL (44 µmol/L) or >25% increase above baseline 72h post-procedure | low-osmolality contrast medium, Ultravist (Iopromide) | 0.9% Isotonic saline at 1ml/kg/h (0.5 ml/kg/h for patients in overt heart failure) for 24h | probucol | 1000 mg orally before primary or urgent angioplasty and 500 mg twice daily for 3 days after intervention | | Mean±SD: 168.89±79.77 | | | Mean S. Creat. ±SD:  0.8±0.23mg/dl | |
|  |  |  |  |  |  |  | Control | IV hydration only | | Mean±SD: 157.9±69.9 | | | Mean S. Creat. ±SD:  0.88±0.38mg/dl | |
| Zagidullin 2017(207) | Inclusion Criteria:  Patients undergoing coronary angiography with impaired renal function (eGFR <80ml/min)  Exclusion Criteria:  1. Acute coronary syndrome,  2. Acute kidney injury/decompensation of chronic kidney disease | 1.Coronary Angiography | 59 | Absolute (44 mmol/L) or relative increase in creatinine (by 25%) retested in day 2± 12 hours | Omnipaque | Body weight adapted 0.9% normal saline 6-12 hours before procedure | RIPC | 5-minutes cycle cuff inflation on the upper arm (systolic blood pressure + 50mmHg) with blood pressure cuff and with a 5-minutes rest between the cycles | | Mean+SD:  155.8±16.9 | | | Mean S. Creat. ±SD:  106.9±10.0 | |
|  |  |  |  |  |  |  | Placebo  (sham RIPC) | Cuff inflated to diastolic blood pressure | | Mean+SD:  148.3±16.7 | | | Mean S. Creat. ±SD:  124.8±11.04 | |
| Zhang 2010(182) | Inclusion Criteria   1. STEMI with heart failure within 24 h of onset of symptoms  2. Primary PCI  Exclusion Criteria   1. Cardiogenic shock 2. Inadequate blood volume 3. Mechanical complications of Acute MI 4. Known allergy | 1.PCI | 185 | Increase in S. Creat. ≥0.5 mg/dL (44 µmol/L) or >25% increase above baseline 72h post-procedure | Iodixanol Iso-Osm (used in patients with chronic kidney diseases) Iohexol Low-Osm (All other patients) | 0.9% Isotonic saline at an adjusted rate of 0.5–1.5 ml/kg/h depending on degree of heart failure from admission to 12 h after PCI | Human recombinant (Brain Natriuretic Peptide) (rhBNP) | 1.5 µg/kg bolus followed by adjusted dose infusion of 0.0075–0.030 µg/kg/min for 24 h after PCI | | Mean±SD: 189.86±51.66 | | | Mean S. Creat. ±SD:  90.89±17.64mmol/l | |
|  |  |  |  |  |  |  | Placebo | IV hydration only | | Mean±SD: 181.27±50.05 | | | Mean S. Creat. ±SD:  90.44±15.37mmol/l | |
| Zhao 2014(183) | Inclusion Criteria  1. Age 18-75 years 2. Acute coronary syndrome 3. Elective PCI  Exclusion Criteria   1. Cardiac shock State supported by device, such as intra-aortic balloon pump (IABP) 2. Hyperpyrexia 3. Allergy to iodine | 1.PCI | 110 | Increase in S. Creat. ≥0.5 mg/dL (44 µmol/L) or >25% increase above baseline 3 days post-procedure | Iodixanol Iso-Osm | 0.45% saline at 1ml/kg/h beginning 2-12h pre- and continuing for ≥ 12h post-procedure. | Cordyceps | 2g TDS for 3days before and after PCI | | Mean±SD: 248.86±48.68 | | | Mean S. Creat. ±SD:  82.8±19.2mmol/l | |
[truncated: 1,747 more chars]
