# Supplementary material for: Effectiveness of contrast-associated acute kidney injury prevention methods; a systematic review and network meta-analysis
Source: BMC Nephrol. 2018 Nov 13;19:323. doi: 10.1186/s12882-018-1113-0 (PMC6234687; doi:10.1186/s12882-018-1113-0)
Supplement: Supplementary file 3 — Main Analysis 200–184 RCTs. (DOCX 46108 kb) [file 12882_2018_1113_MOESM3_ESM.docx]

**Supplement:**

Results from Analysis 1 (200 RCTs)

Number of Studies: 200 RCTs (see Manuscript)

Figures and Tables:

1. Network Diagram (included in the main manuscript)

2. Tables:

A. Network Characteristics (included in the main manuscript)

B. Interventions Characteristics

C Direct comparisons characteristics

3. Rankogram (included in the main manuscript)

4. Ranking and probability of being the best (included in the main manuscript)

5. Forest Plot

| Software | Spec | Convergence | Analysis |
| --- | --- | --- | --- |
| Netmetaxl / WinBUGS14 version 1.4.3 | Burn 5000  Model 10000 | good convergence (FE MC error 5% of the SD) | Random Effects (Vague)  Random Effects (Informative) |

Table 1Interventions Characteristics

| **Treatment** | **# Studies** | **# Events** | **# Patients** | **Aggregate Rate** |
| --- | --- | --- | --- | --- |
| **I.V Hydration** | 41 | 539 | 5136 | 0.1049 |
| **Statins** | 14 | 123 | 3040 | 0.0405 |
| **Furosemide** | 3 | 50 | 554 | 0.0903 |
| **NAC** | 68 | 667 | 6095 | 0.1094 |
| **Trimetazidine** | 4 | 17 | 352 | 0.0483 |
| **NaHCO3** | 32 | 366 | 3393 | 0.1079 |
| **PGE1** | 4 | 24 | 304 | 0.0789 |
| **MgSO4** | 1 | 9 | 62 | 0.1452 |
| **Pentoxifylline** | 4 | 30 | 438 | 0.0685 |
| **Placebo** | 70 | 938 | 7044 | 0.1332 |
| **Control** | 88 | 1238 | 9120 | 0.1357 |
| **Allopurinol** | 4 | 5 | 204 | 0.0245 |
| **BNP** | 4 | 52 | 744 | 0.0699 |
| **Probucol** | 2 | 12 | 198 | 0.0606 |
| **α-tocopherol** | 4 | 18 | 312 | 0.0577 |
| **γ-tocopherol** | 1 | 6 | 102 | 0.0588 |
| **Oxygen** | 2 | 33 | 346 | 0.0954 |
| **Amlodipine and Valsartan** | 1 | 8 | 45 | 0.1778 |
| **K/Na citrate** | 2 | 6 | 203 | 0.0296 |
| **Nicorandil** | 3 | 15 | 291 | 0.0515 |
| **Ascorbic Acid** | 7 | 52 | 552 | 0.0942 |
| **Alpha-Lipoic Acid** | 2 | 6 | 139 | 0.0432 |
| **Oral Hydration** | 5 | 23 | 254 | 0.0906 |
| **Nebivolol** | 1 | 8 | 40 | 0.2000 |
| **Anisodamine** | 2 | 17 | 192 | 0.0885 |
| **RIPC** | 10 | 38 | 608 | 0.0625 |
| **Theophylline** | 7 | 21 | 384 | 0.0547 |
| **Hypothermia** | 1 | 14 | 58 | 0.2414 |
| **Glutathione** | 2 | 21 | 421 | 0.0499 |
| **MESNA** | 1 |  | 51 | 0.0000 |
| **ACEI** | 3 | 10 | 129 | 0.0775 |
| **Aminophylline** | 2 | 4 | 45 | 0.0889 |
| **Iloprost** | 2 | 8 | 118 | 0.0678 |
| **Acetazolamide** | 1 | 5 | 94 | 0.0532 |
| **ANP** | 3 | 24 | 202 | 0.1188 |
| **Zinc** | 1 | 3 | 18 | 0.1667 |
| **Dialysis** | 5 | 43 | 293 | 0.1468 |
| **Fenoldopam** | 5 | 78 | 333 | 0.2342 |
| **ERAs** | 1 | 43 | 77 | 0.5584 |
| **CCB** | 1 |  | 42 | 0.0000 |
| **Dopamine** | 2 | 9 | 48 | 0.1875 |
| **Mannitol** | 2 | 10 | 35 | 0.2857 |
| **Cordyceps** | 2 | 7 | 88 | 0.0795 |
| **Silymarin.** | 1 | 2 | 69 | 0.0290 |

Table 2 Direct comparisons characteristics

| **Comparison** | **# Studies** | **# Patients** | **# Events** |
| --- | --- | --- | --- |
| NAC vs. Placebo | 36 | 8,202 | 945 |
| Statins vs. Control | 6 | 4,382 | 200 |
| NaHCO3 vs. K/Na citrate | 1 | 206 | 4 |
| I.V Hydration vs. NaHCO3 | 24 | 5,481 | 515 |
| I.V Hydration vs. Oral Hydration | 5 | 509 | 43 |
| NAC vs. Ascorbic Acid | 3 | 583 | 88 |
| Placebo vs. Ascorbic Acid | 5 | 869 | 139 |
| NAC vs. Control | 21 | 2,474 | 367 |
| NAC vs. Fenoldopam | 3 | 359 | 44 |
| Control vs. Fenoldopam | 2 | 123 | 26 |
| I.V Hydration vs. Control | 8 | 2,884 | 396 |
| NAC vs. Theophylline | 1 | 62 | 13 |
| Statins vs. Placebo | 7 | 1,508 | 125 |
| Placebo vs. Theophylline | 3 | 224 | 21 |
| NaHCO3 vs. Oral Hydration | 1 | 43 | 3 |
| Control vs. Alpha-Lipoic Acid | 2 | 280 | 16 |
| Furosemide vs. Control | 3 | 1,089 | 131 |
| Furosemide vs. Theophylline | 1 | 159 | 18 |
| Control vs. Theophylline | 3 | 493 | 27 |
| Control vs. RIPC | 6 | 782 | 68 |
| NAC vs. NaHCO3 | 5 | 805 | 211 |
| Control vs. Allopurinol | 1 | 159 | 6 |
| I.V Hydration vs. NAC | 6 | 681 | 109 |
| Pentoxifylline vs. Control | 2 | 461 | 46 |
| Placebo vs. Nicorandil | 1 | 240 | 29 |
| MgSO4 vs. Control | 1 | 126 | 26 |
| Control vs. Dopamine | 2 | 96 | 17 |
| Control vs. Anisodamine | 1 | 260 | 39 |
| NAC vs. Nebivolol | 1 | 80 | 17 |
| Control vs. Nebivolol | 1 | 80 | 19 |
| Control vs. ACEI | 1 | 71 | 12 |
| Placebo vs. ACEI | 2 | 202 | 19 |
| Placebo vs. RIPC | 4 | 427 | 43 |
| NaHCO3 vs. Control | 6 | 1,056 | 231 |
| Control vs. Cordyceps | 2 | 180 | 19 |
| Statins vs. Pentoxifylline | 1 | 220 | 9 |
| Control vs. CCB | 1 | 85 | 2 |
| NAC vs. Zinc | 1 | 37 | 4 |
| Placebo vs. Zinc | 1 | 35 | 5 |
| NAC vs. Aminophylline | 1 | 30 | 0 |
| Control vs. Aminophylline | 1 | 30 | 4 |
| NAC vs. α-tocopherol | 1 | 20 | 0 |
| Placebo vs. α-tocopherol | 4 | 624 | 66 |
| Control vs. Nicorandil | 2 | 341 | 22 |
| PGE1 vs. Placebo | 2 | 392 | 74 |
| I.V Hydration vs. Allopurinol | 2 | 185 | 31 |
| NAC vs. Allopurinol | 3 | 215 | 30 |
| ANP vs. Mannitol | 1 | 20 | 8 |
| Placebo vs. ANP | 1 | 126 | 26 |
| Control vs. Dialysis | 5 | 588 | 112 |
| Control vs. Probucol | 2 | 409 | 50 |
| PGE1 vs. Control | 2 | 226 | 16 |
| Control vs. BNP | 1 | 209 | 23 |
| I.V Hydration vs. BNP | 2 | 1,128 | 113 |
| Trimetazidine vs. Control | 4 | 714 | 71 |
| Control vs. MESNA | 1 | 100 | 7 |
| Control vs. K/Na citrate | 1 | 202 | 25 |
| Control vs. Oxygen | 2 | 697 | 115 |
| Control vs. ANP | 1 | 254 | 19 |
| Control vs. Amlodipine and Valsartan | 1 | 90 | 11 |
| I.V Hydration vs. Acetazolamide | 1 | 190 | 21 |
| NaHCO3 vs. Acetazolamide | 1 | 190 | 9 |
| NAC vs. Dialysis | 1 | 275 | 11 |
| Placebo vs. Aminophylline | 1 | 60 | 10 |
| Placebo vs. Allopurinol | 1 | 60 | 16 |
| NAC vs. Glutathione | 1 | 14 | 1 |
| Control vs. Glutathione | 1 | 14 | 1 |
| Placebo vs. Silymarin. | 1 | 143 | 10 |
| Control vs. Mannitol | 1 | 53 | 10 |
| Placebo vs. Iloprost | 1 | 30 | 3 |
| Control vs. Iloprost | 1 | 208 | 31 |
| Placebo vs. Fenoldopam | 1 | 283 | 90 |
| Control vs. Hypothermia | 1 | 128 | 29 |
| Placebo vs. γ-tocopherol | 1 | 203 | 21 |
| α-tocopherol vs. γ-tocopherol | 1 | 204 | 11 |
| Placebo vs. ERAs | 1 | 158 | 67 |
| Placebo vs. Glutathione | 1 | 825 | 41 |
| Placebo vs. Anisodamine | 1 | 126 | 17 |
| I.V Hydartion vs. Pentoxifylline | 1 | 199 | 12 |
| Placebo vs. BNP | 1 | 149 | 36 |
| Control vs. Ascorbic Acid | 1 | 156 | 10 |

Figure 1Forest Plot


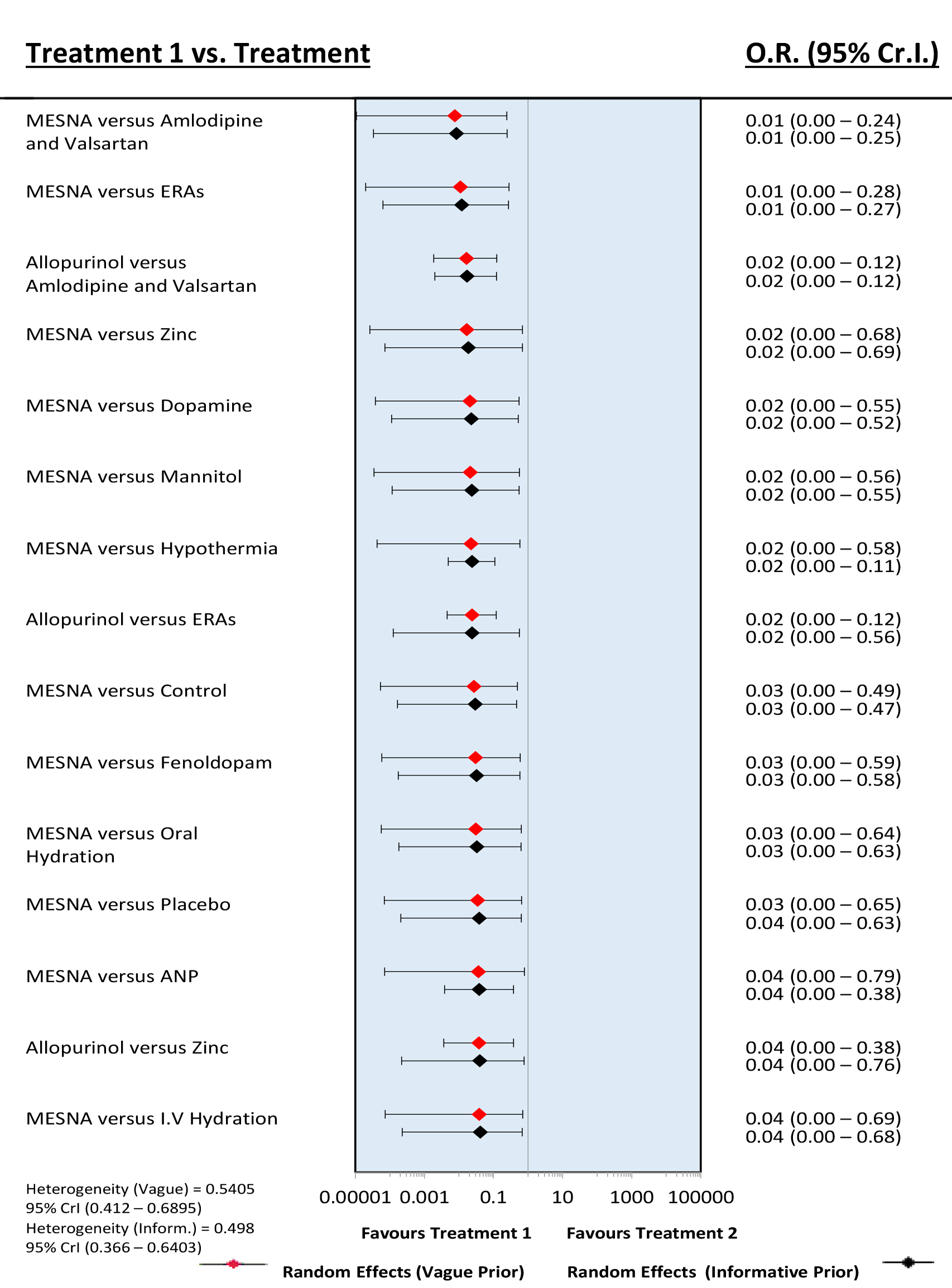


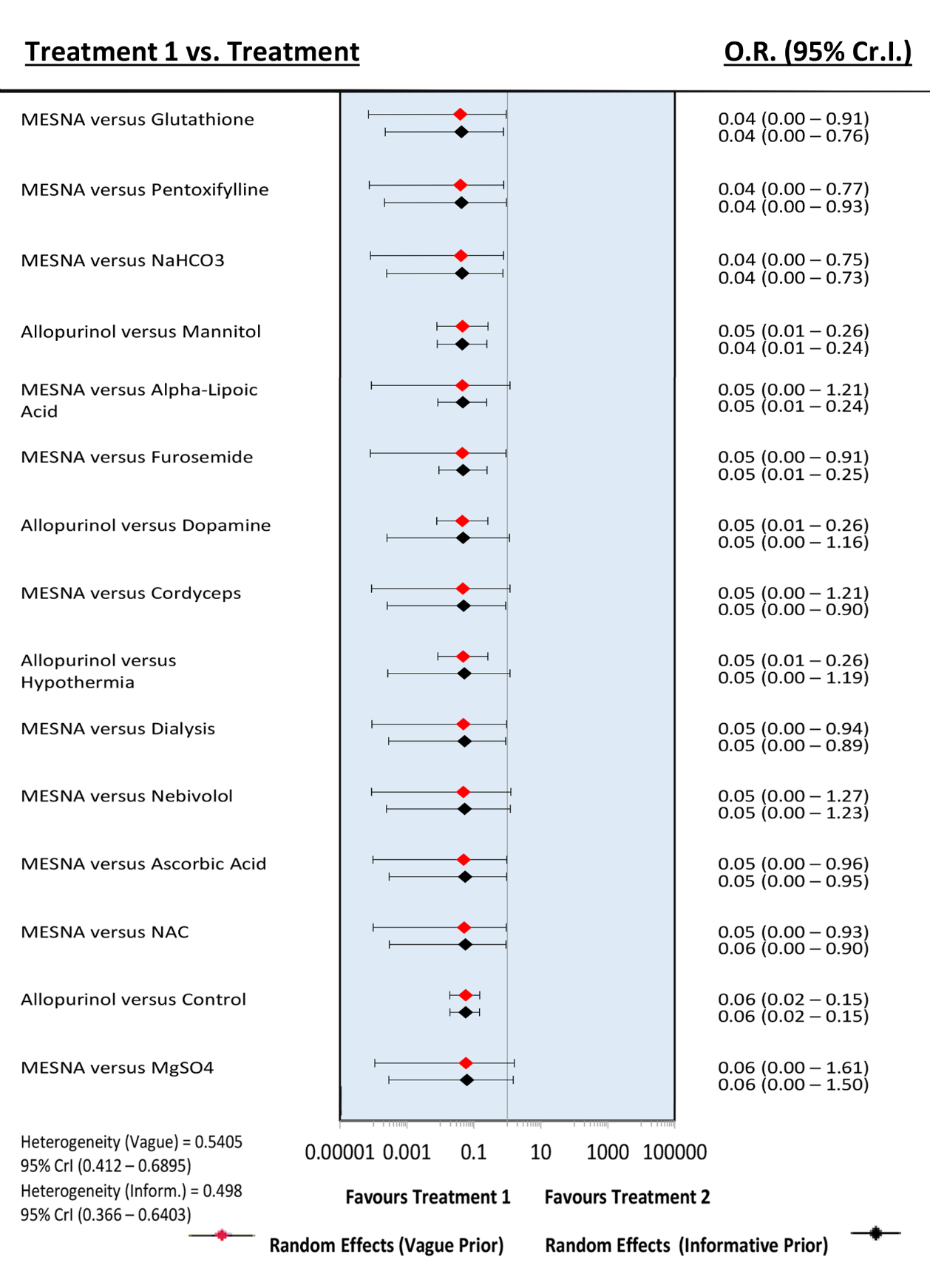


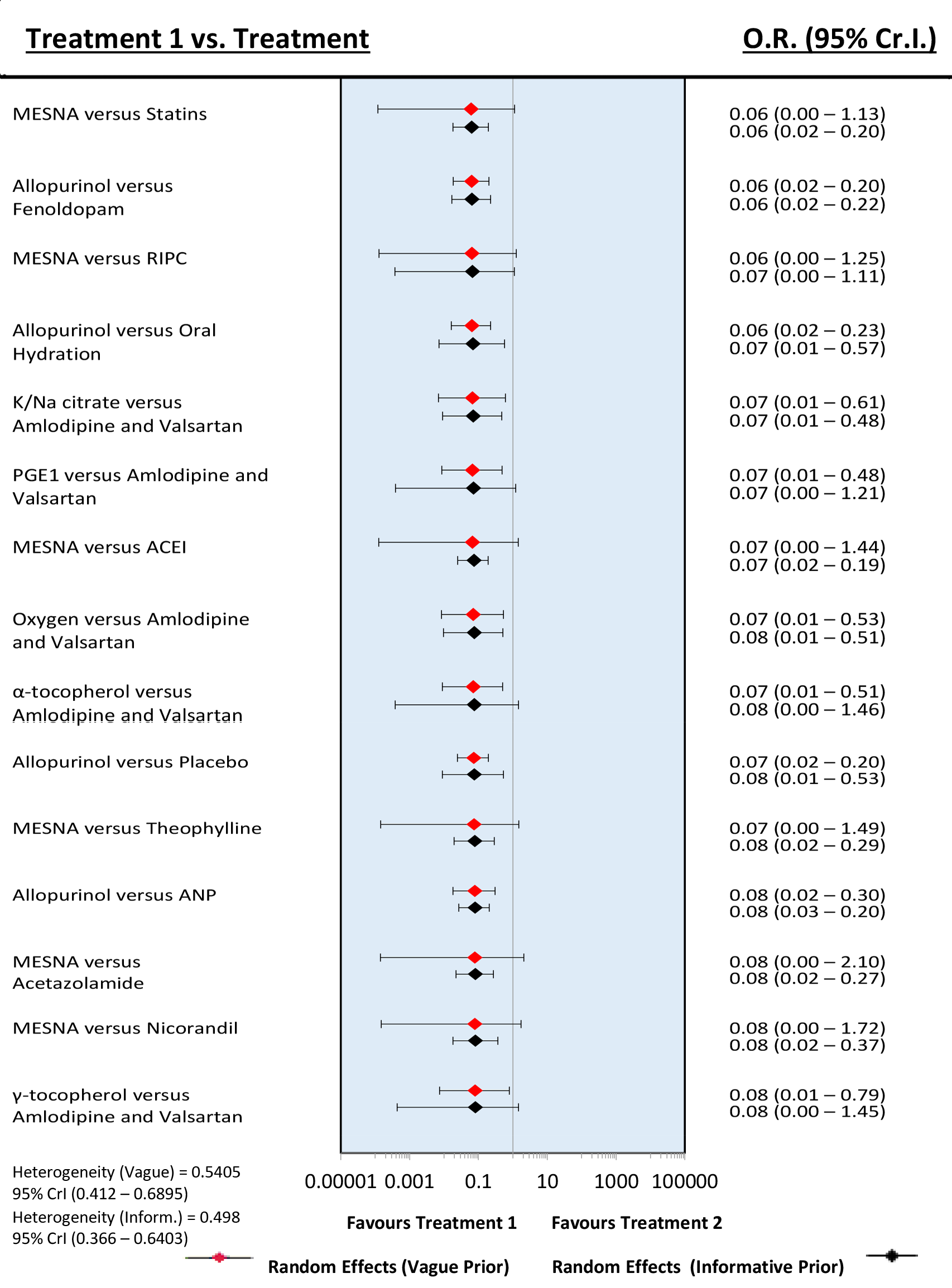


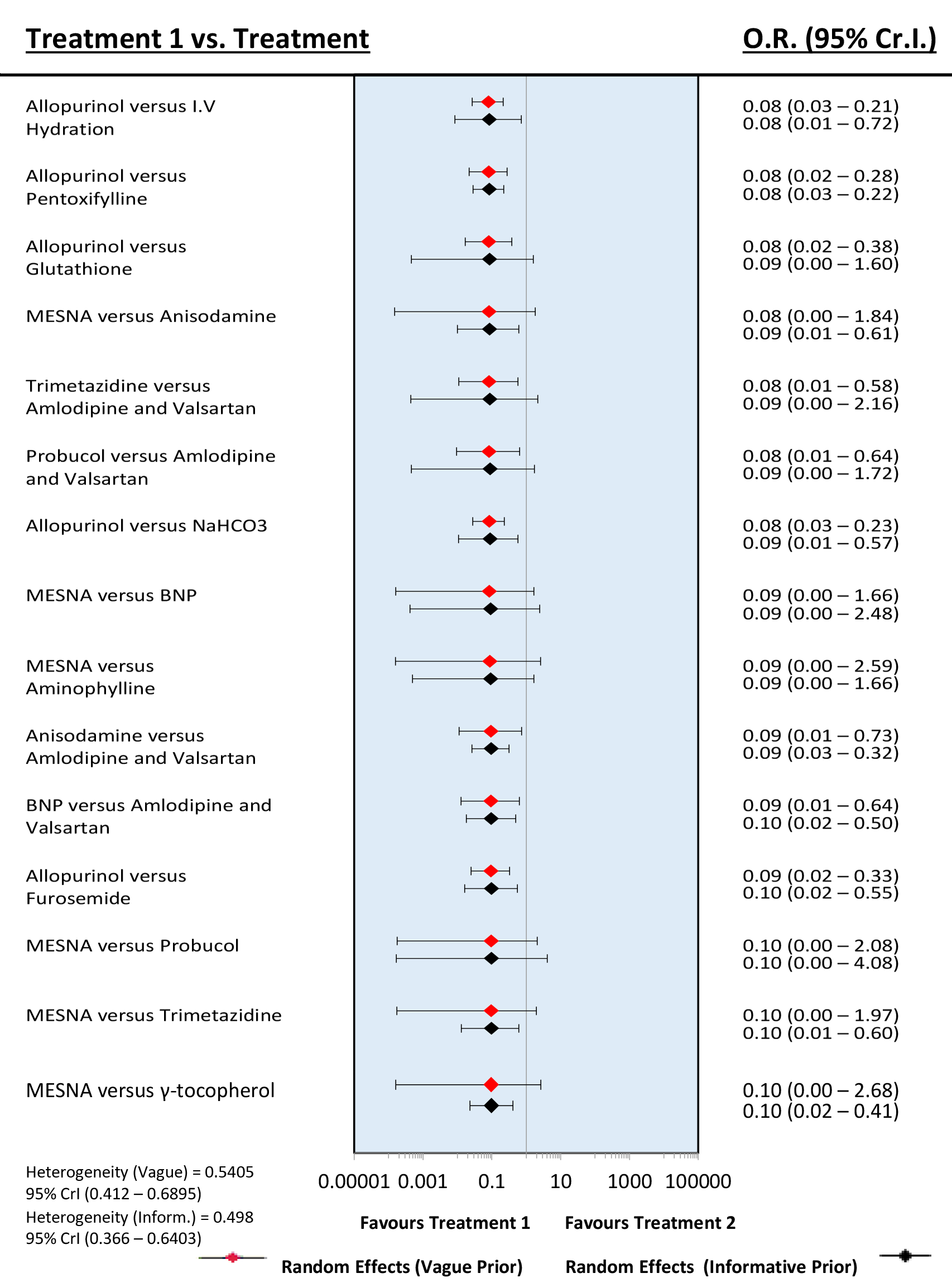


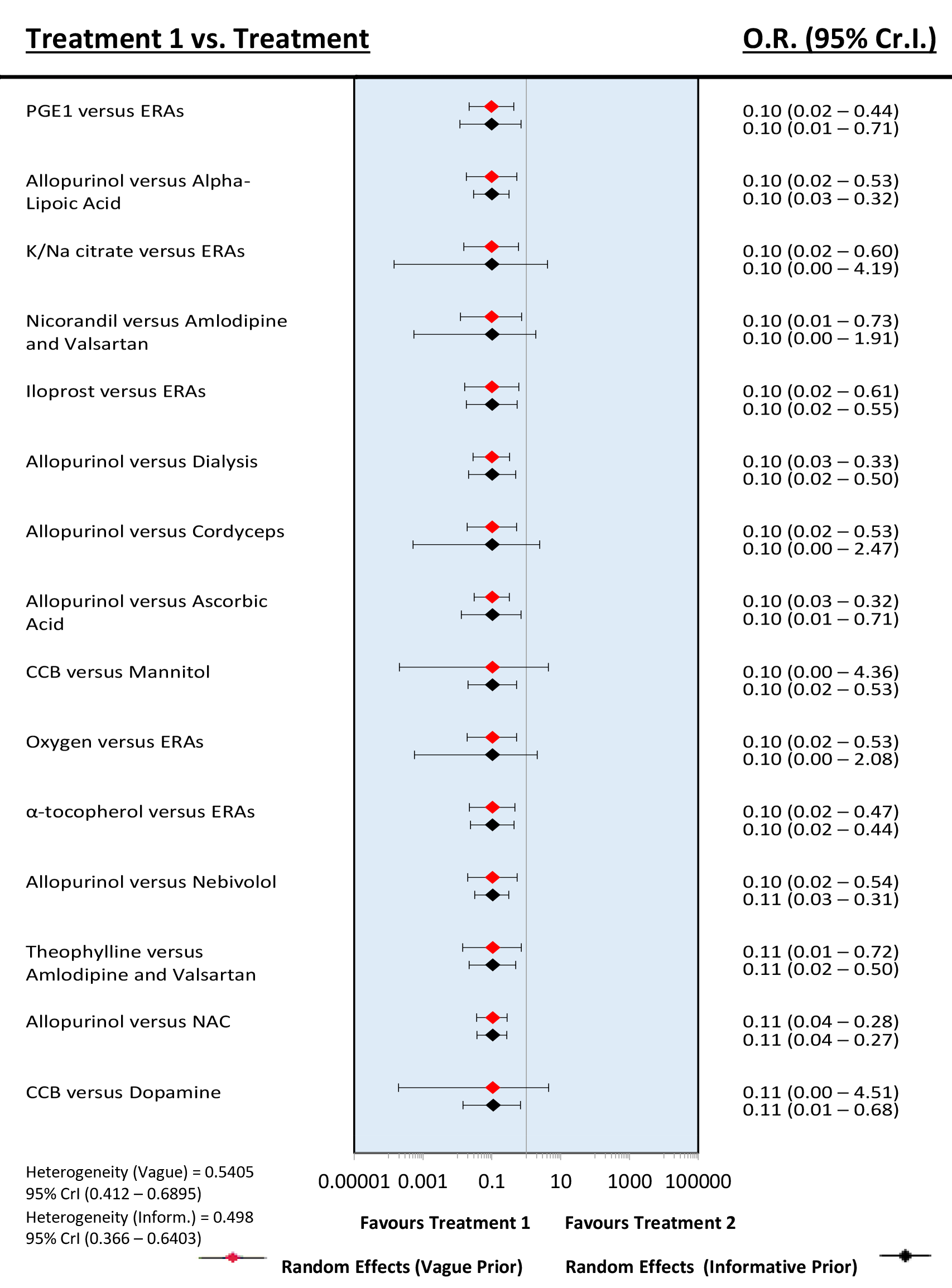


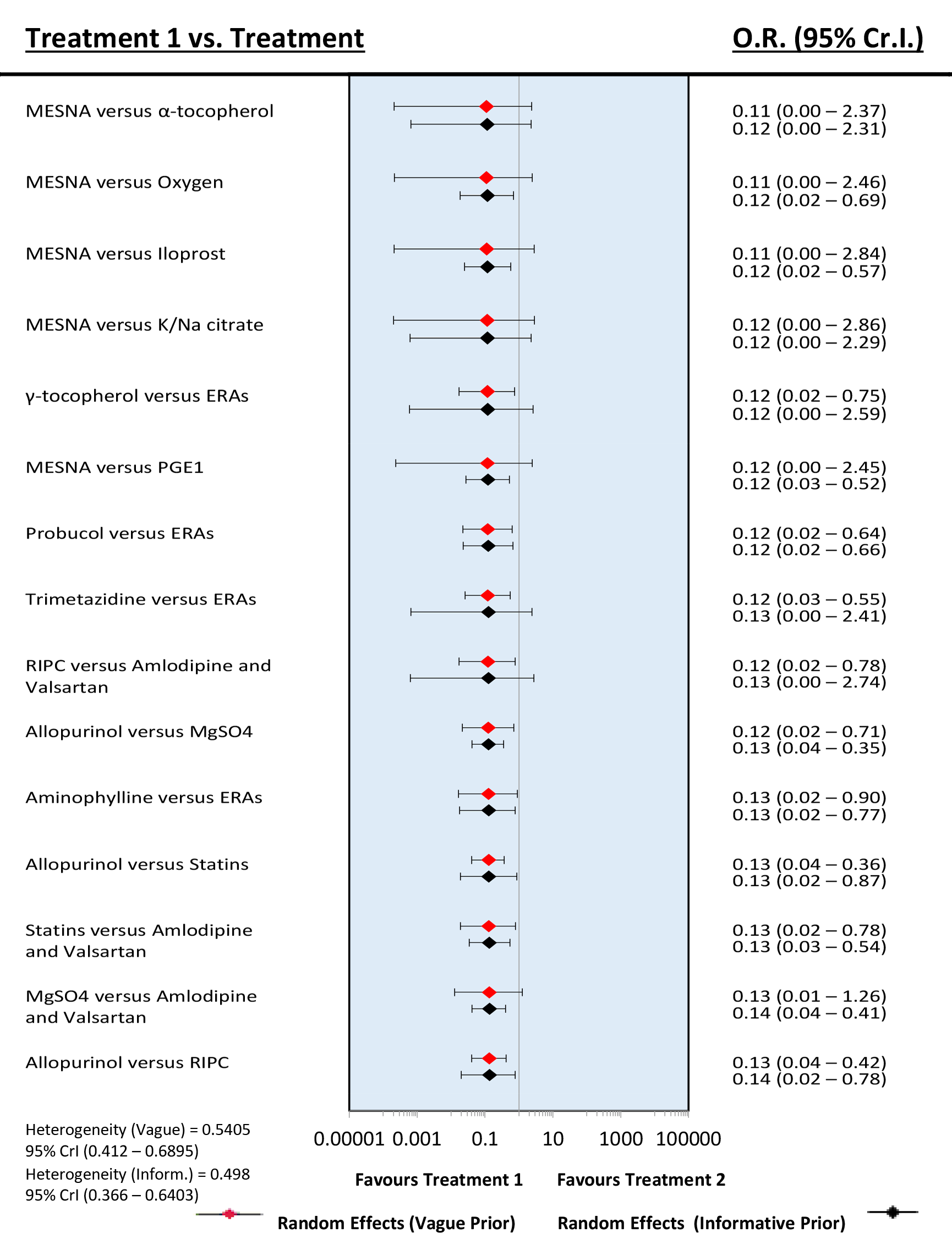


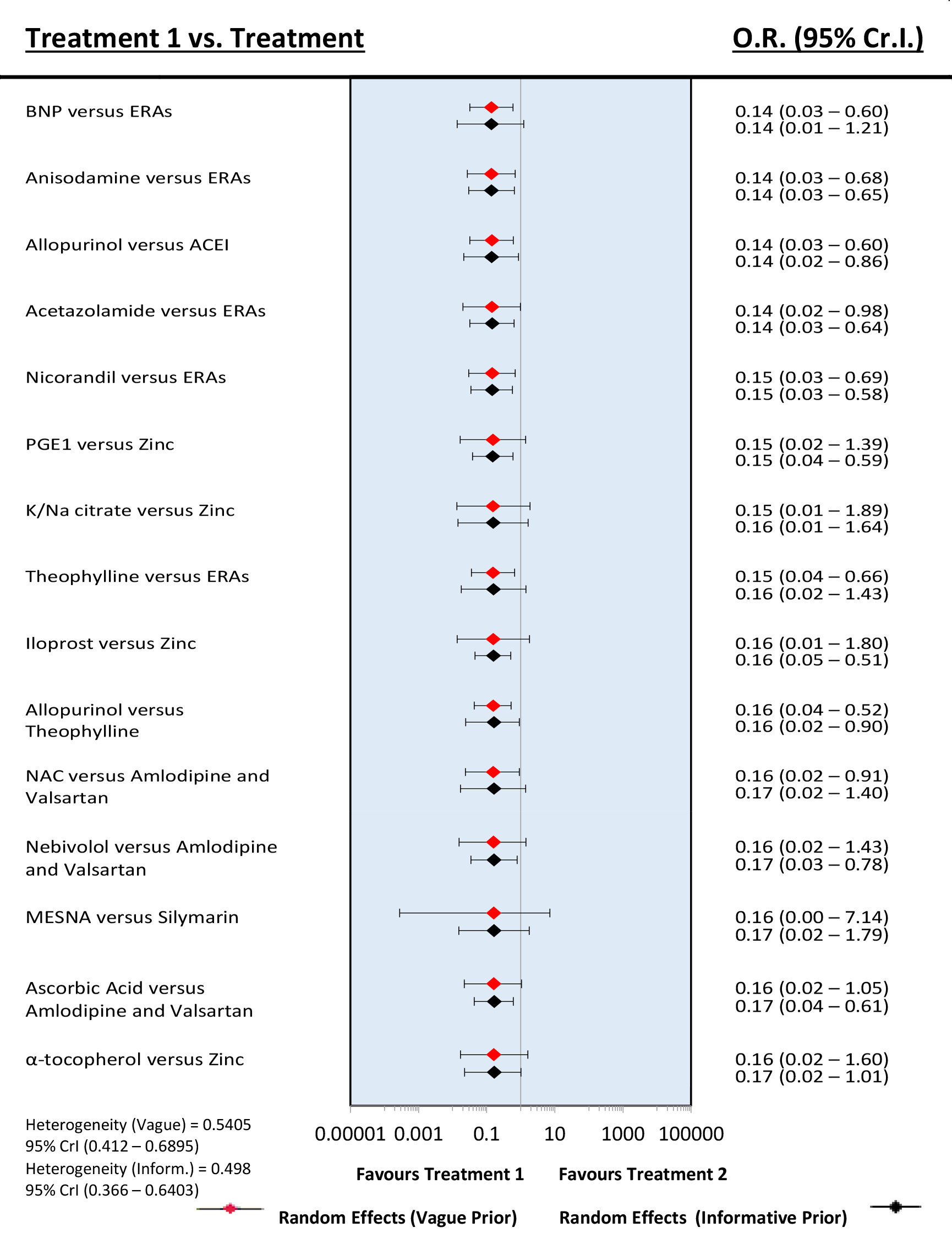


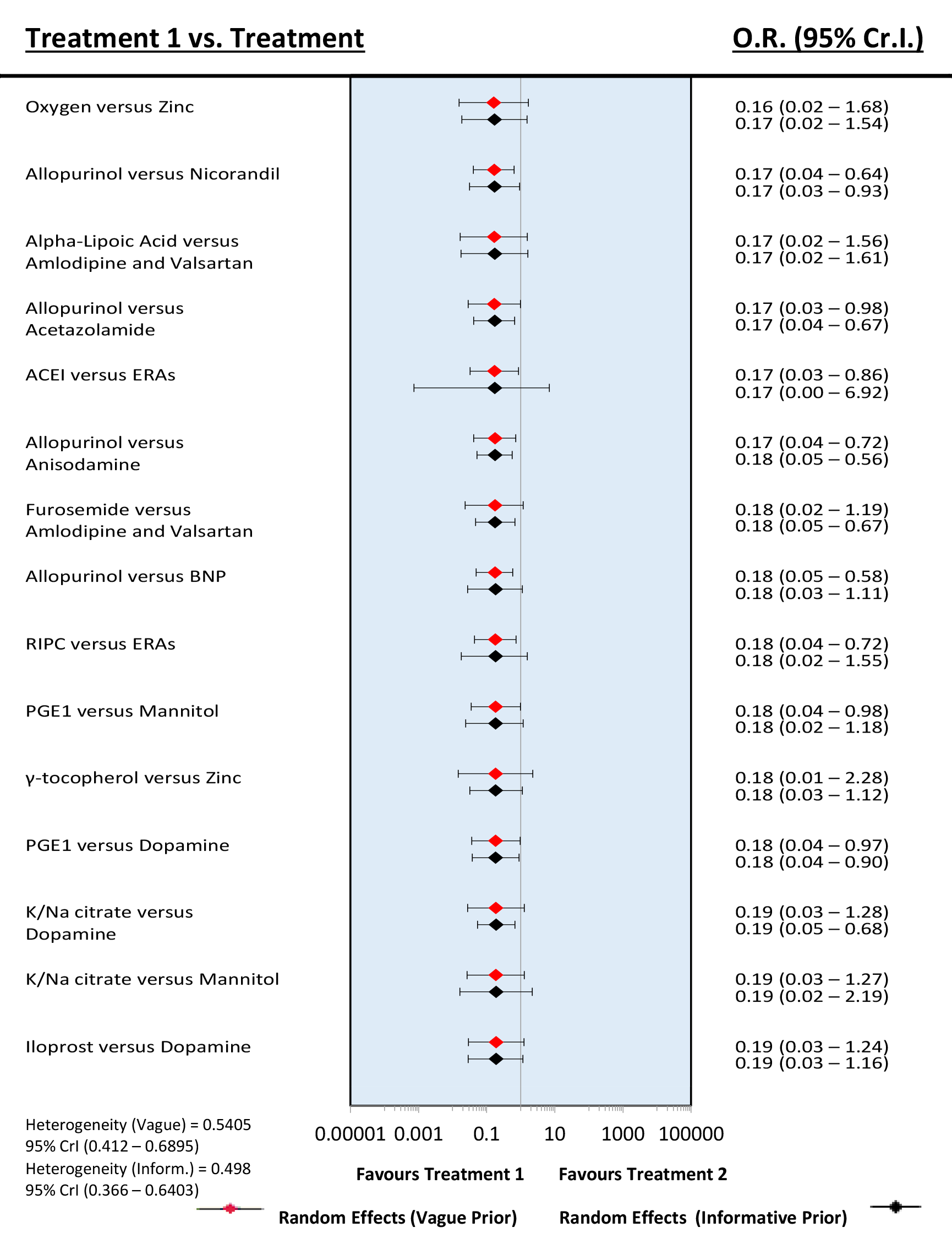


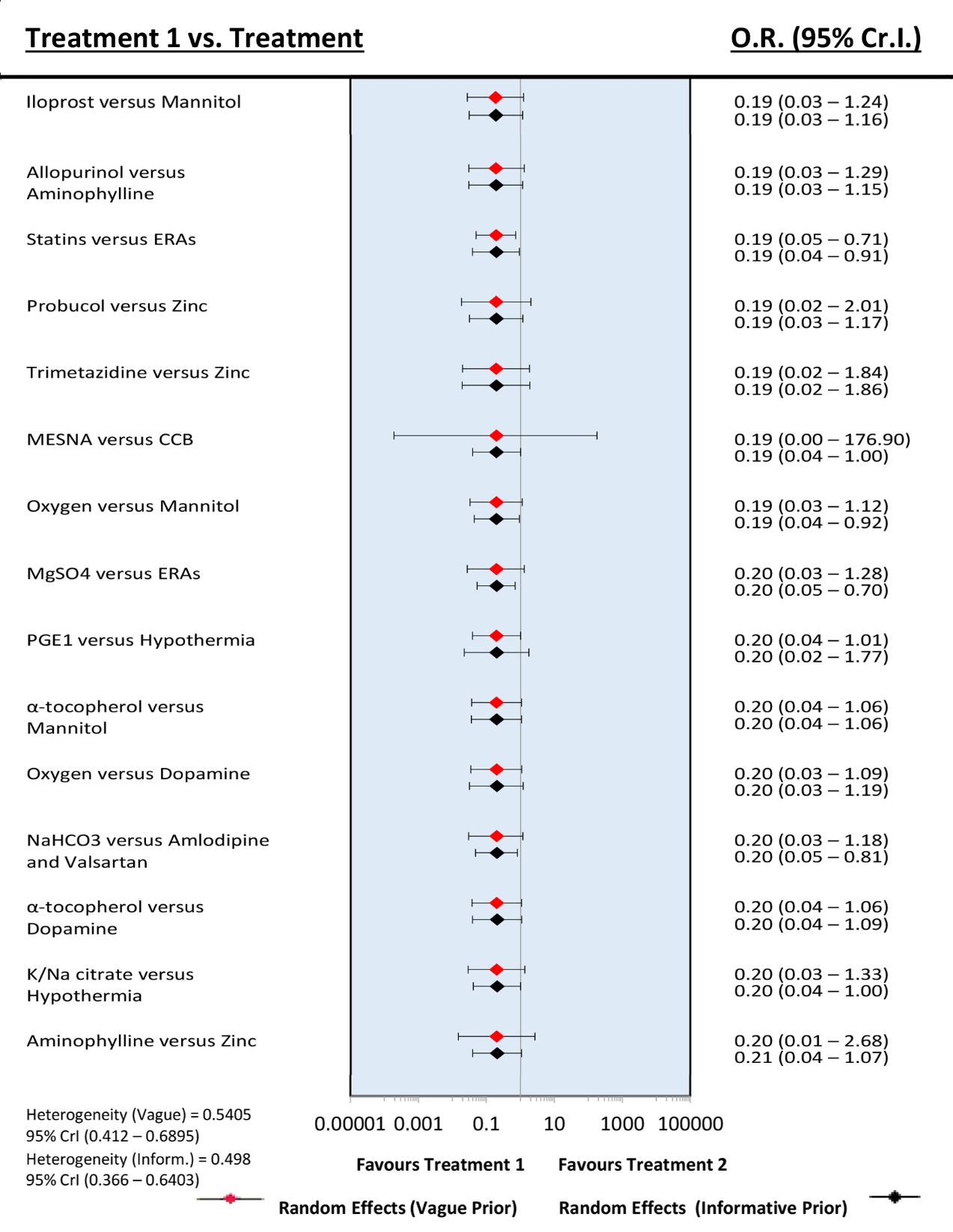


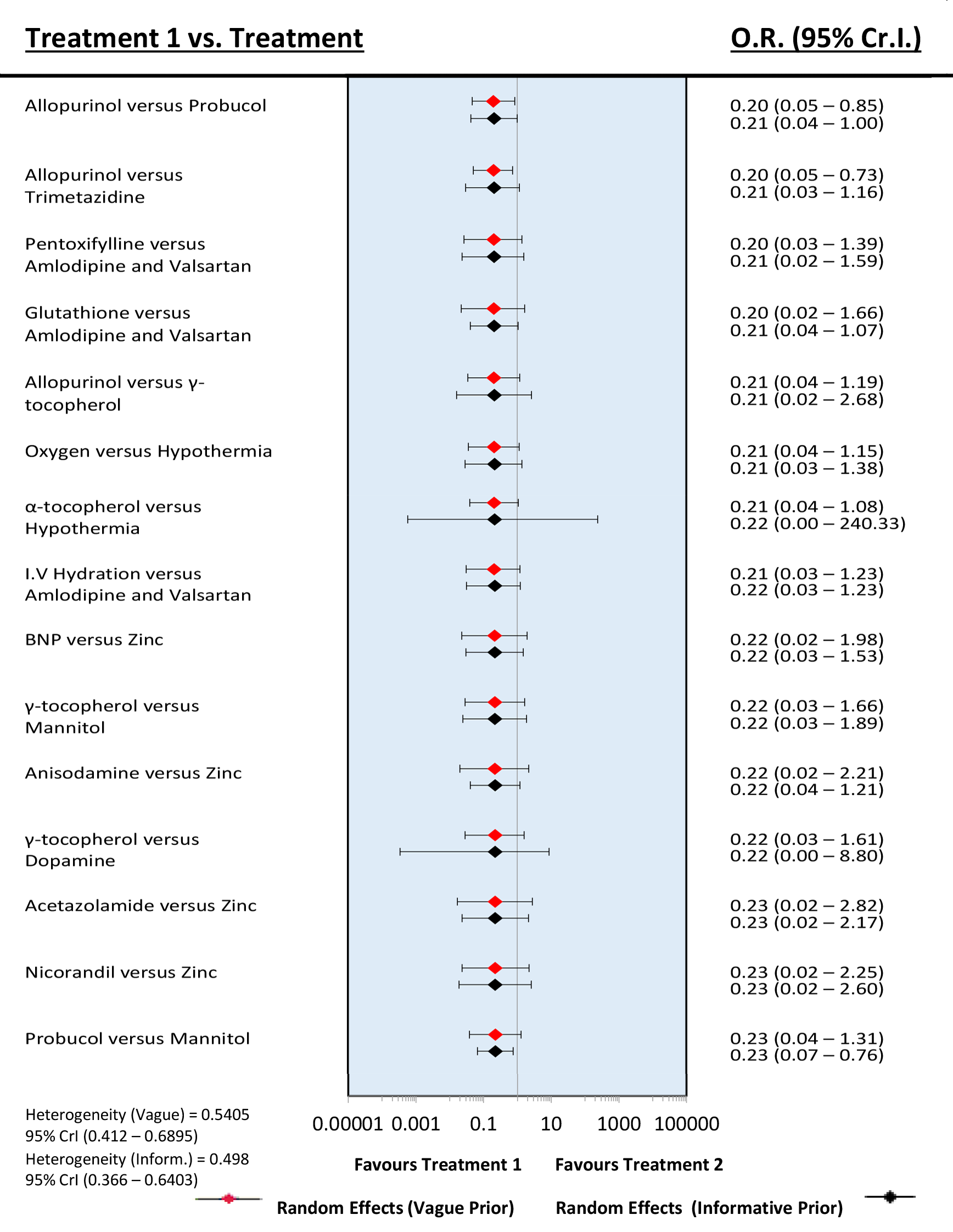


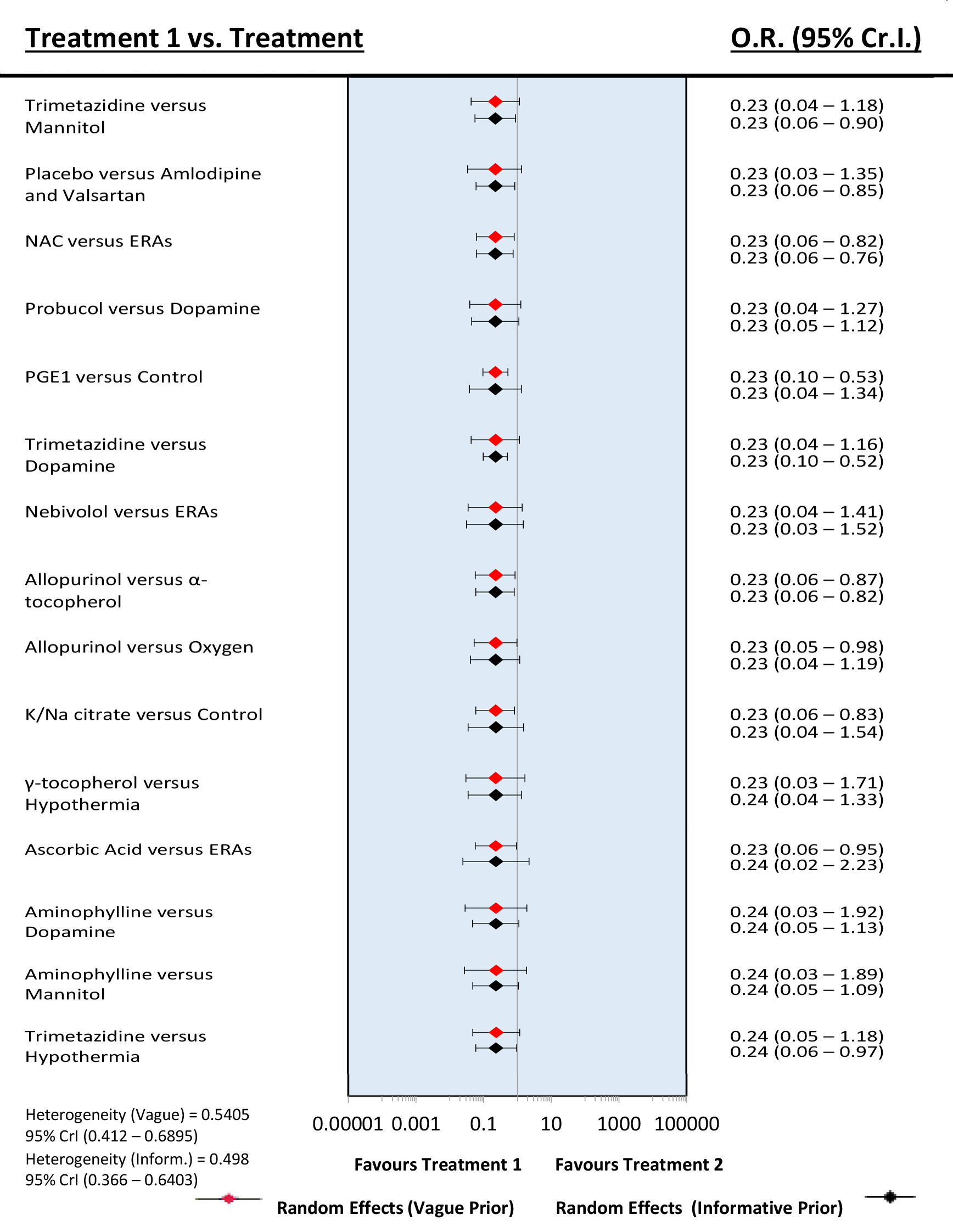


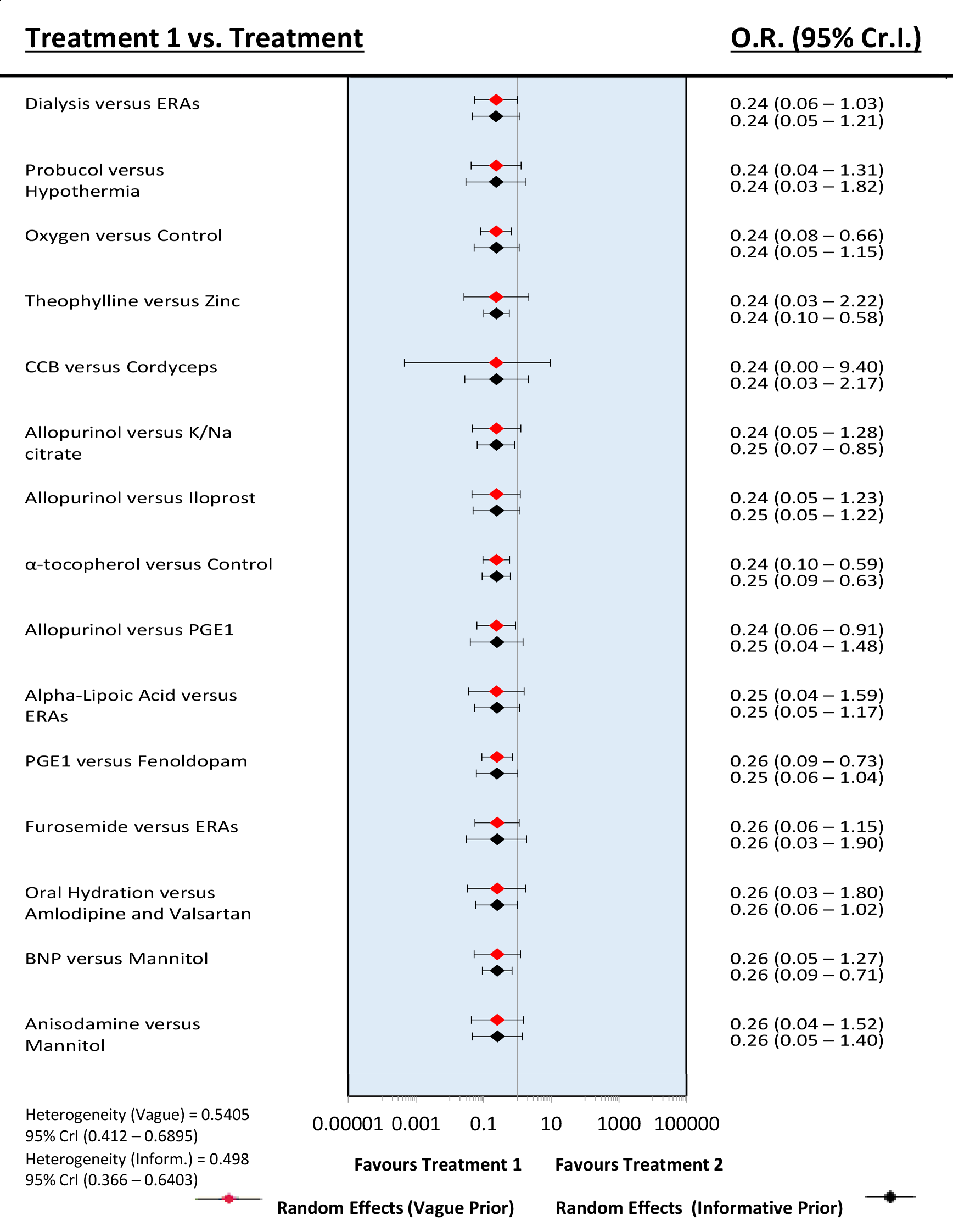


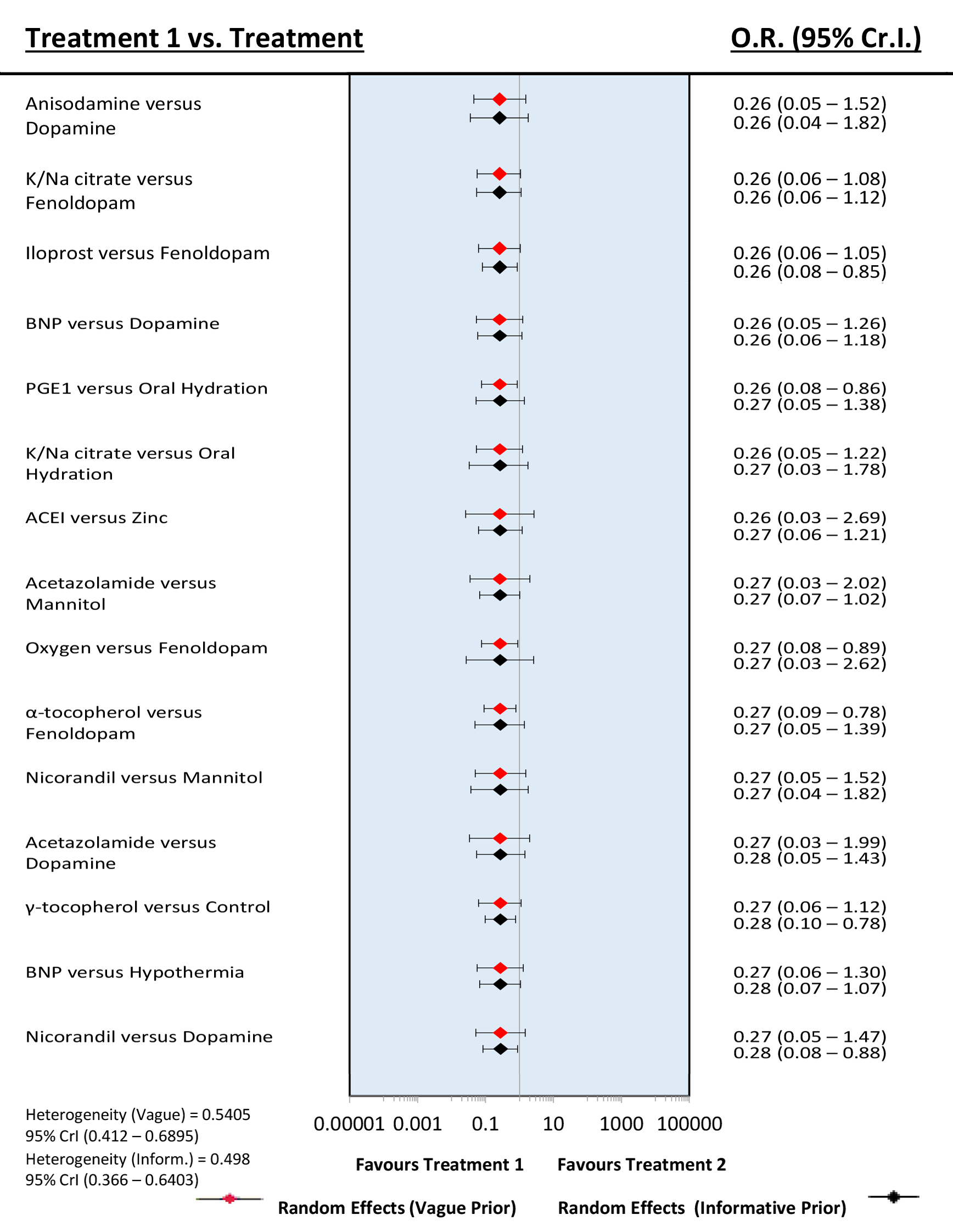


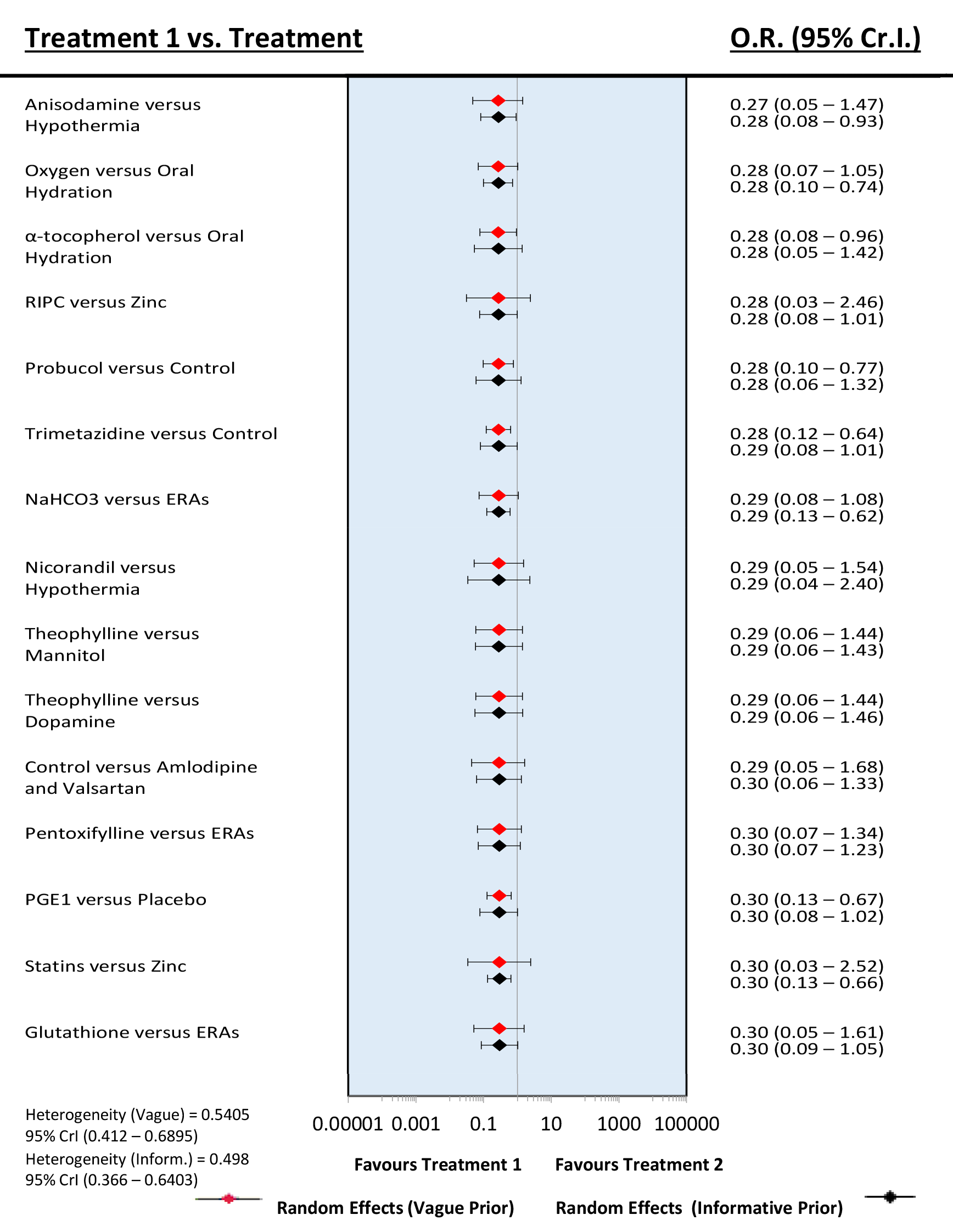


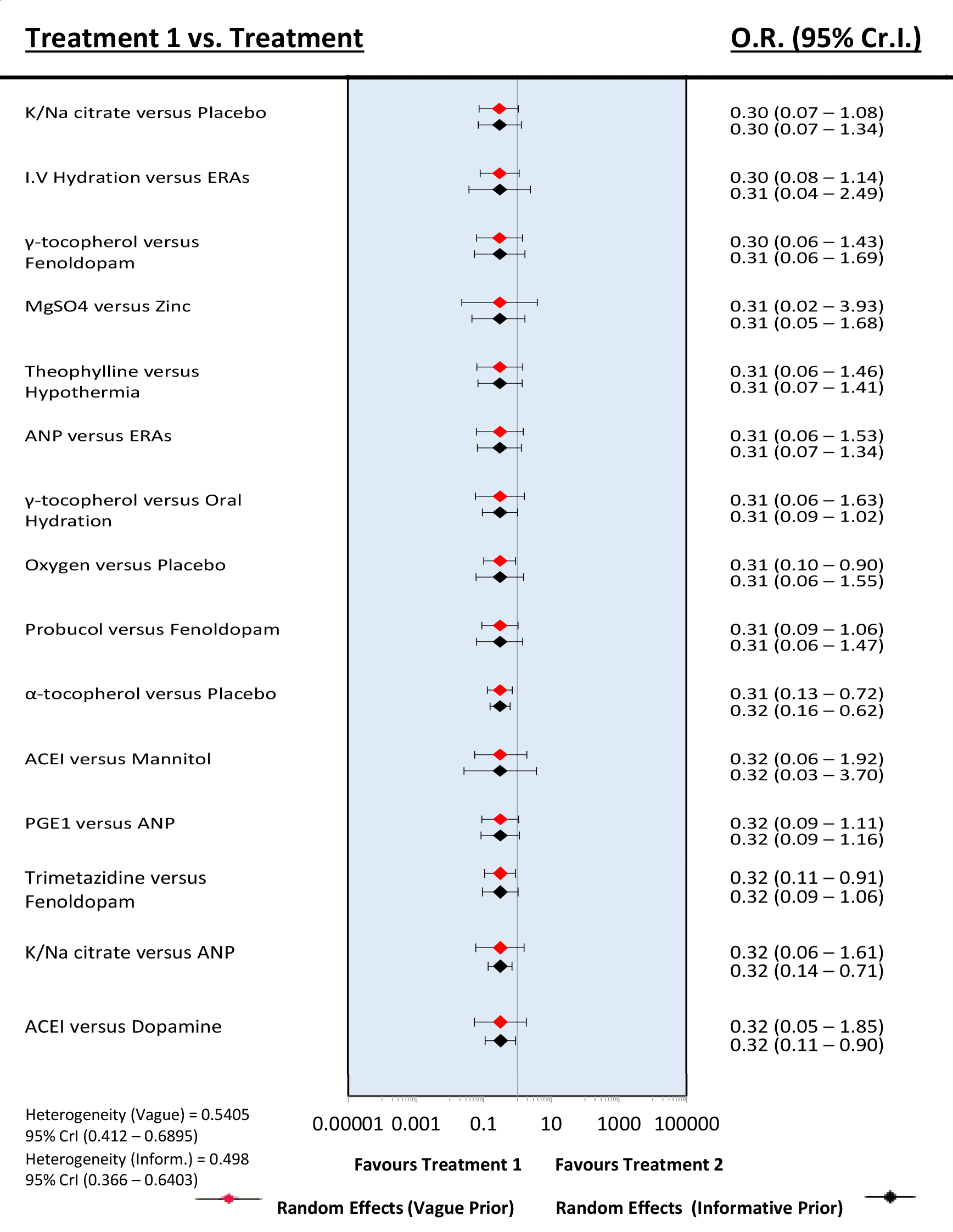


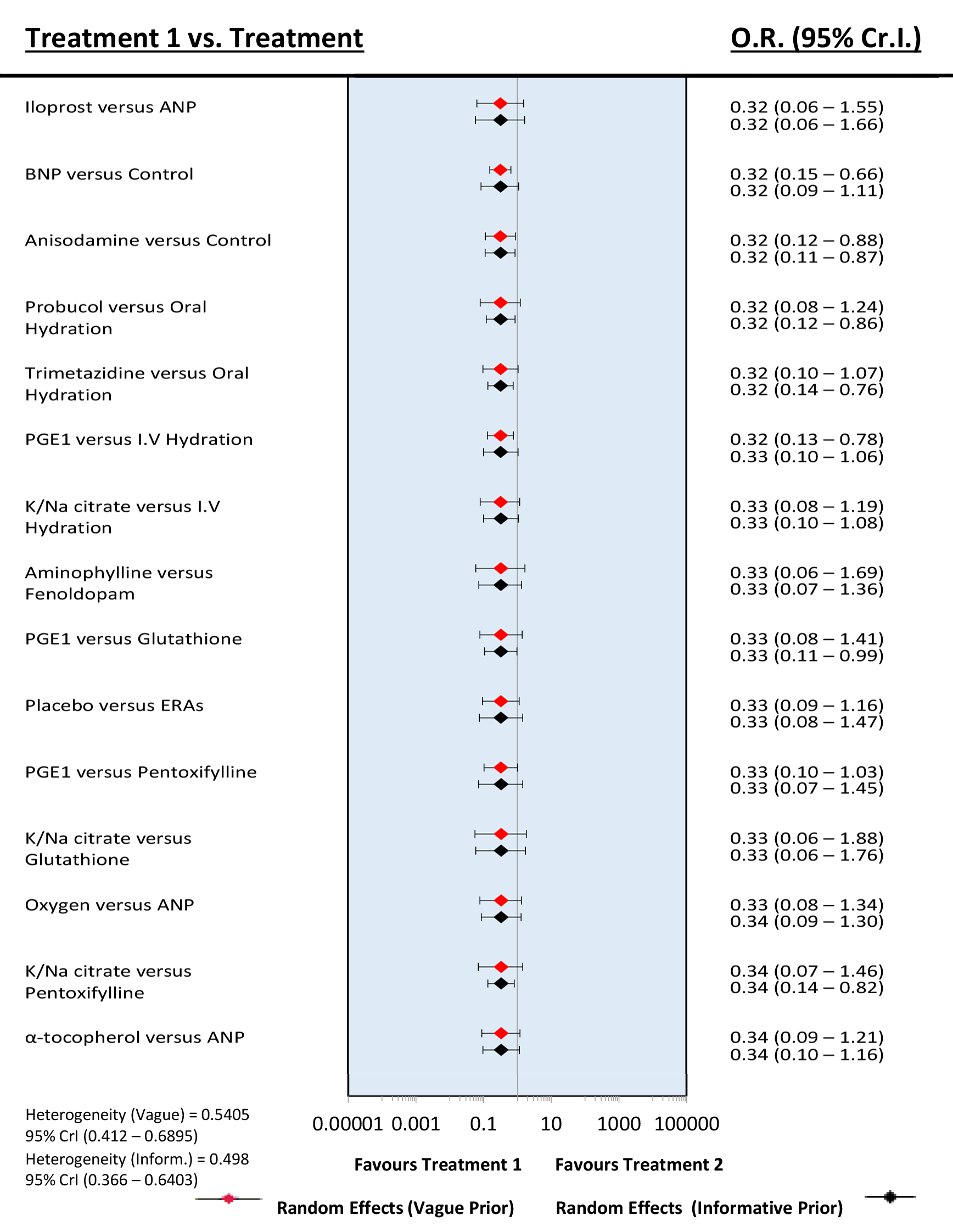


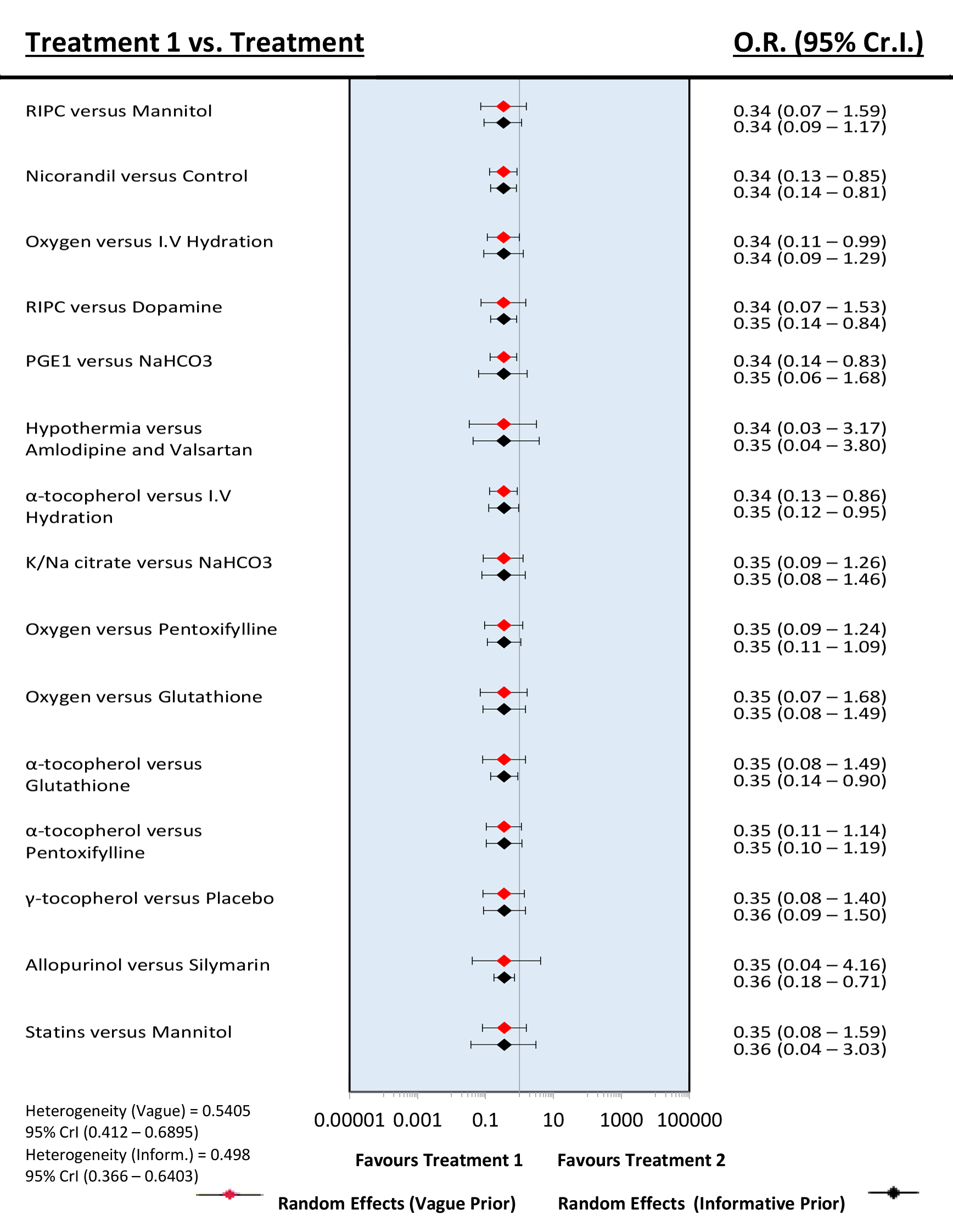


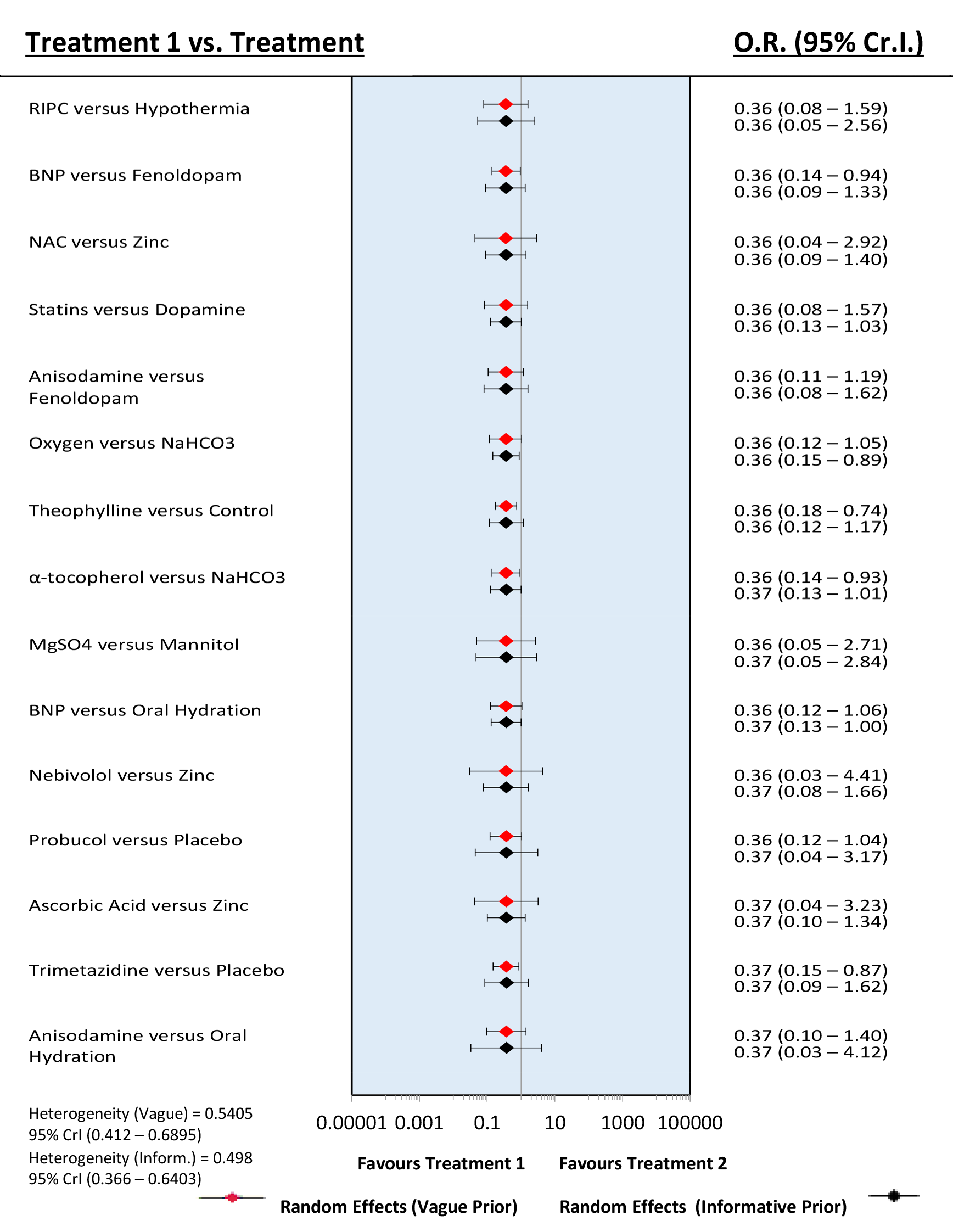


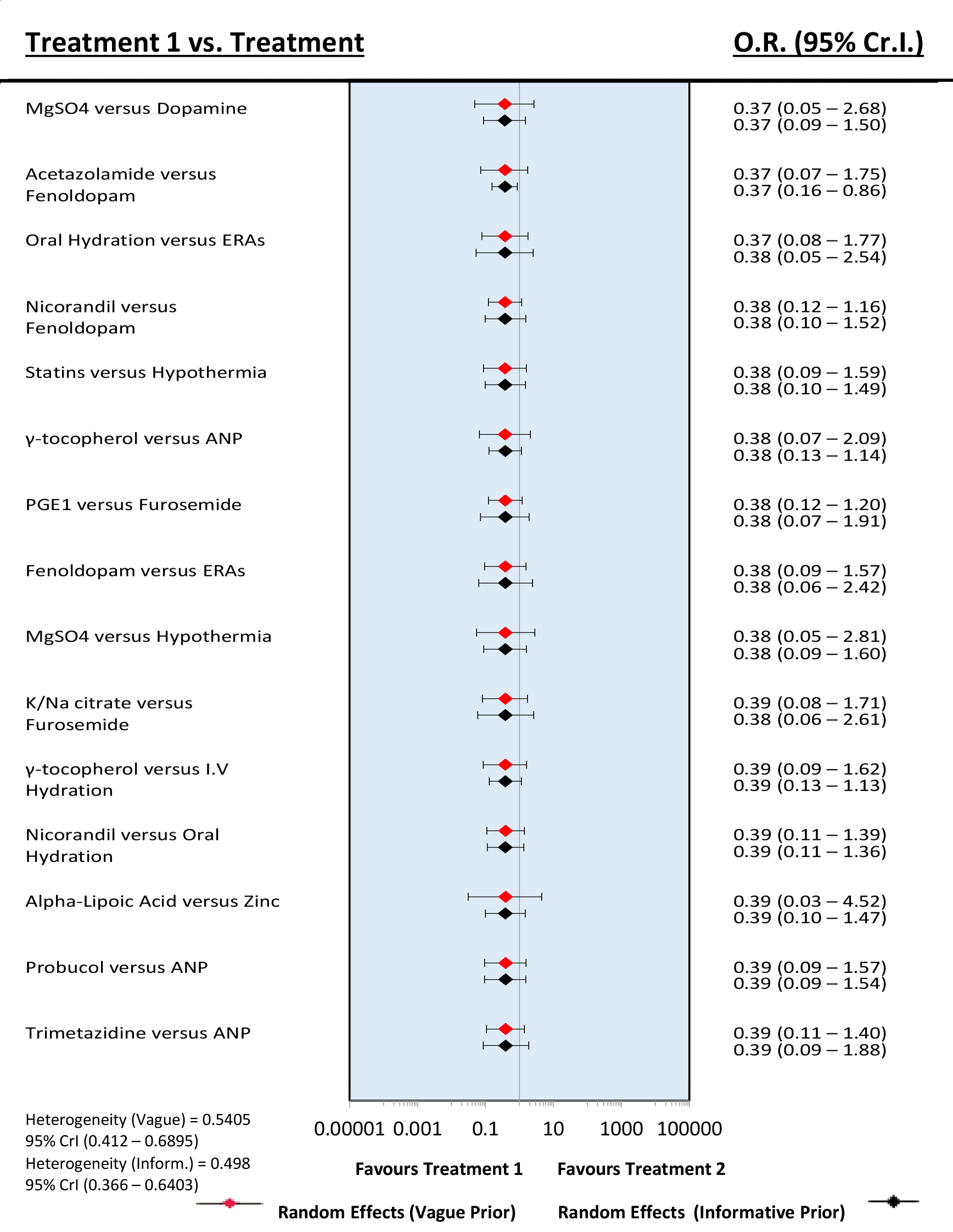


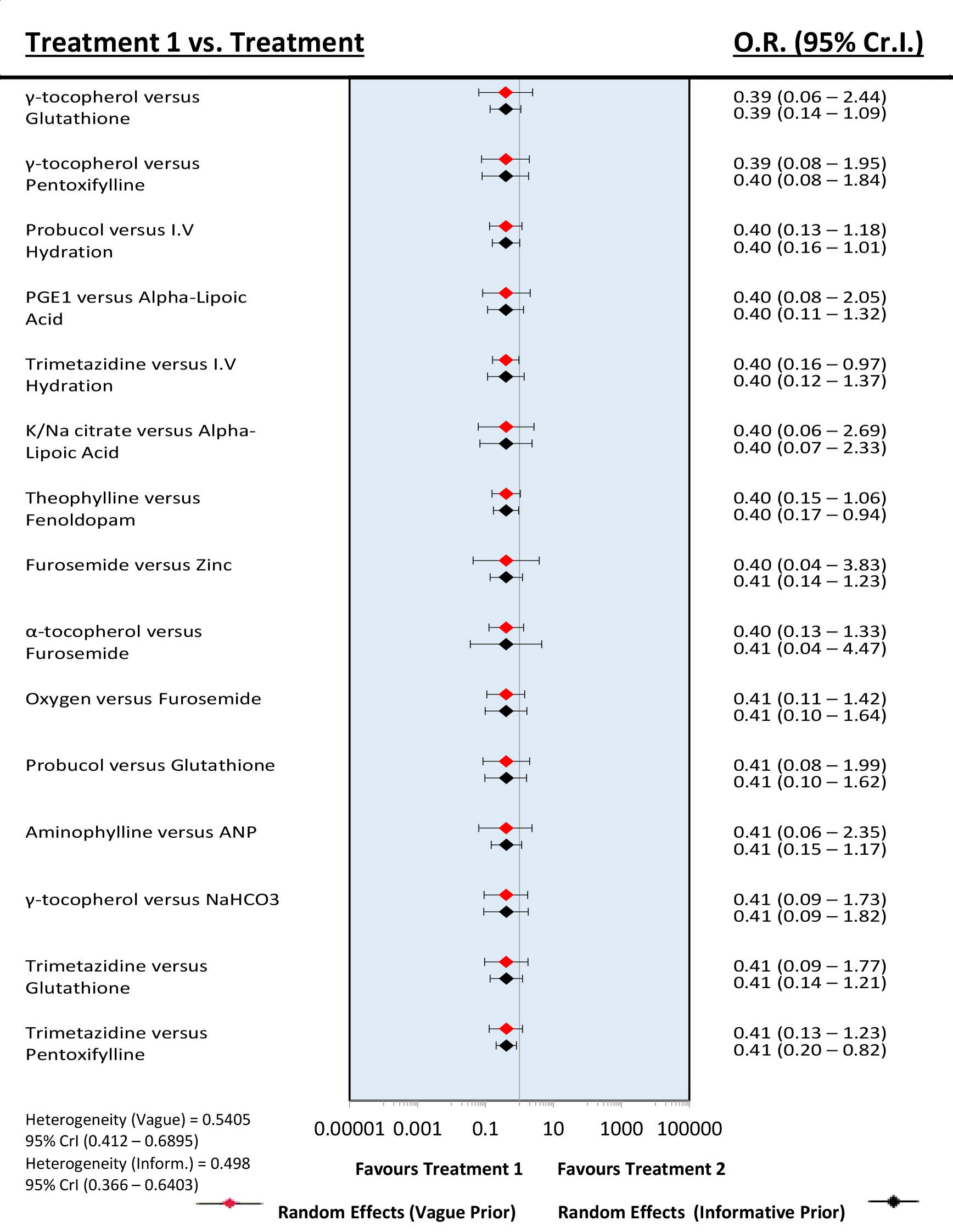


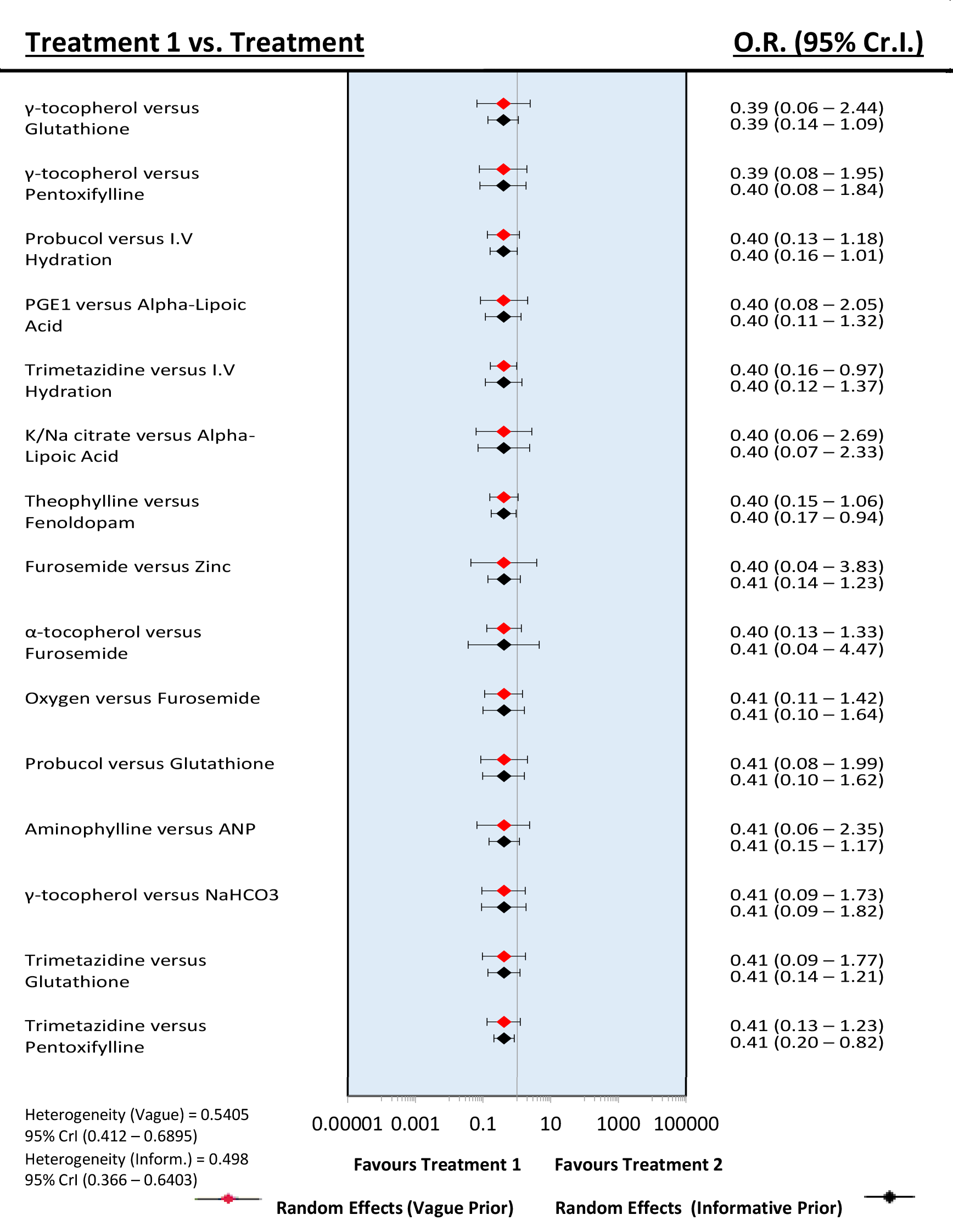


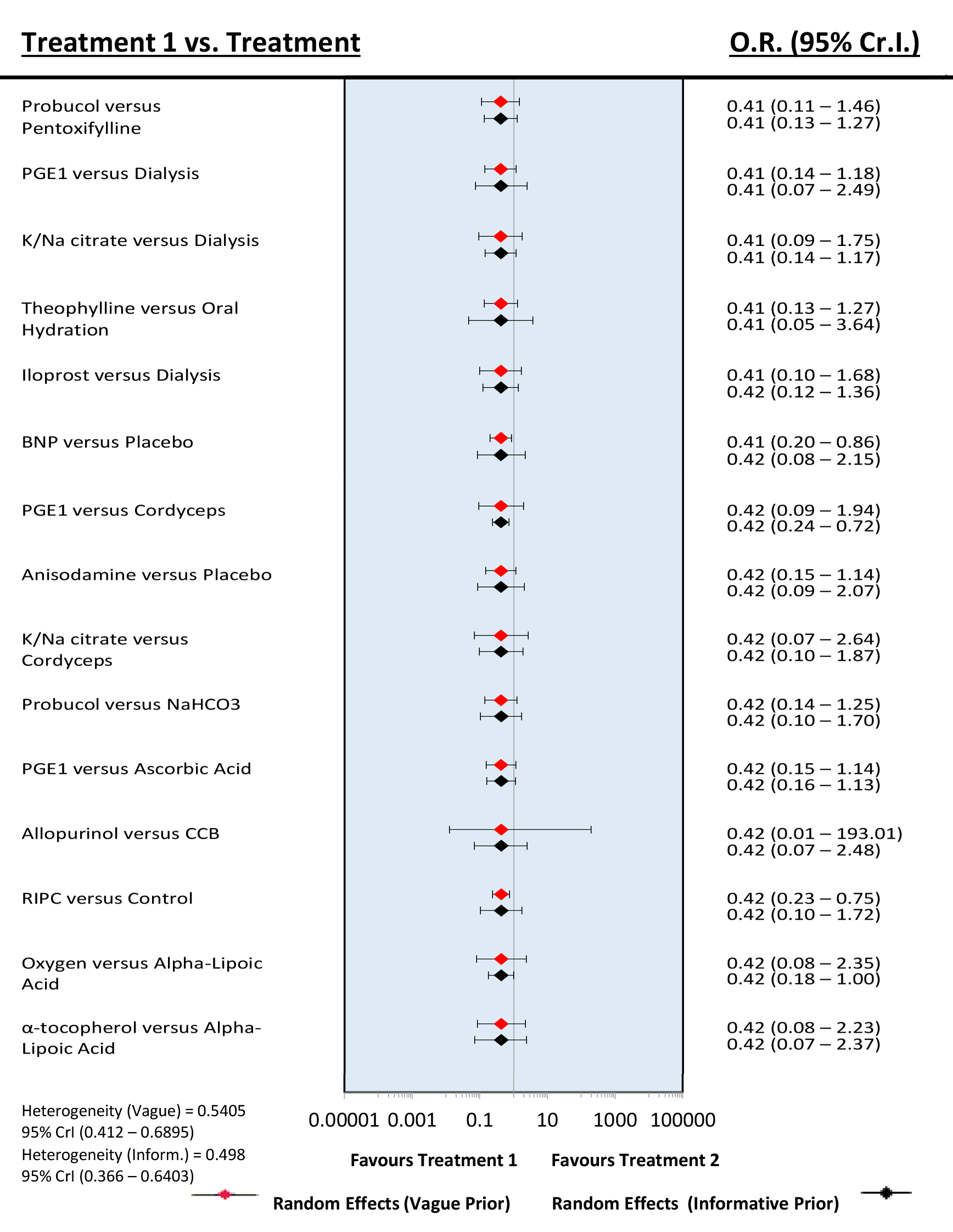


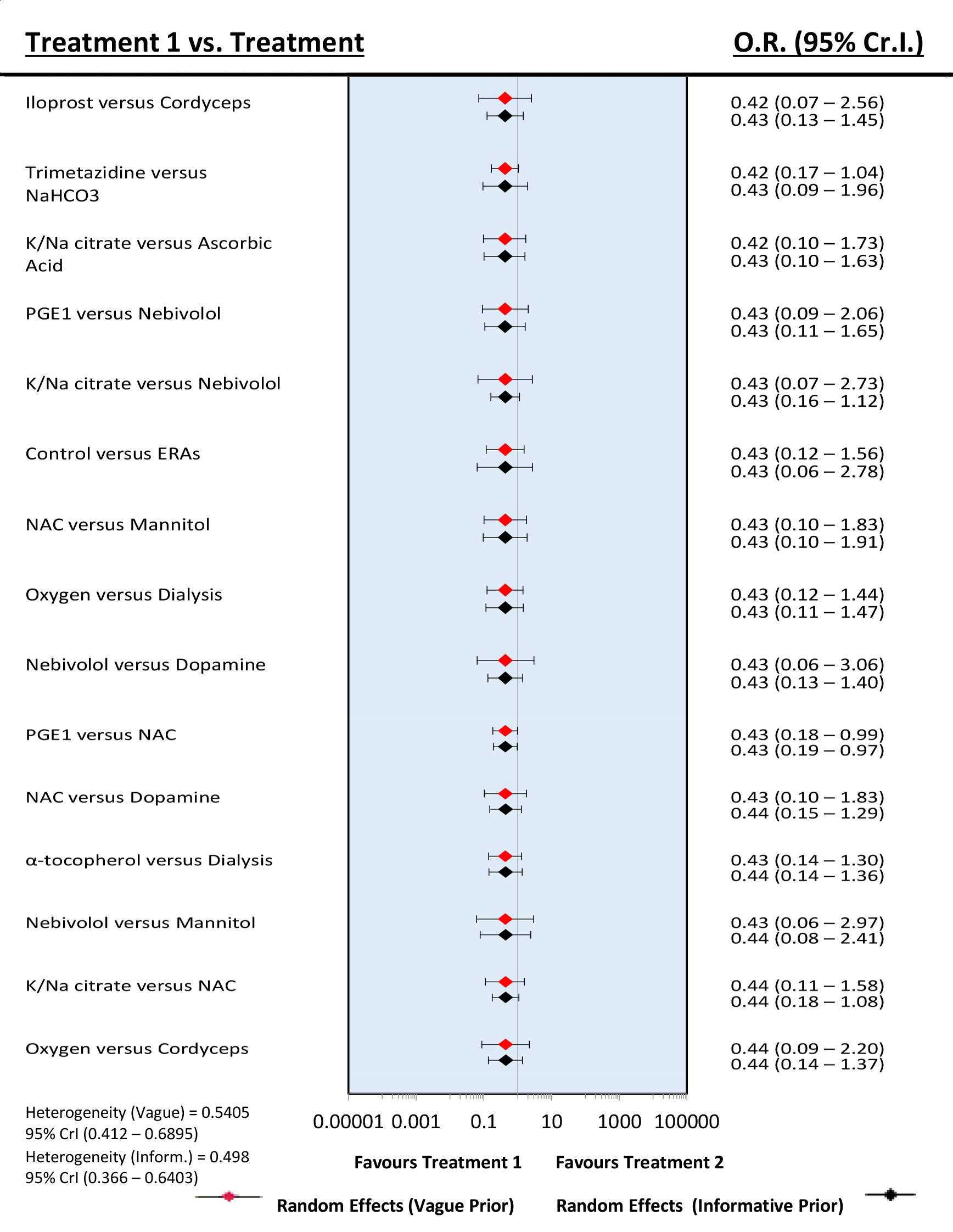


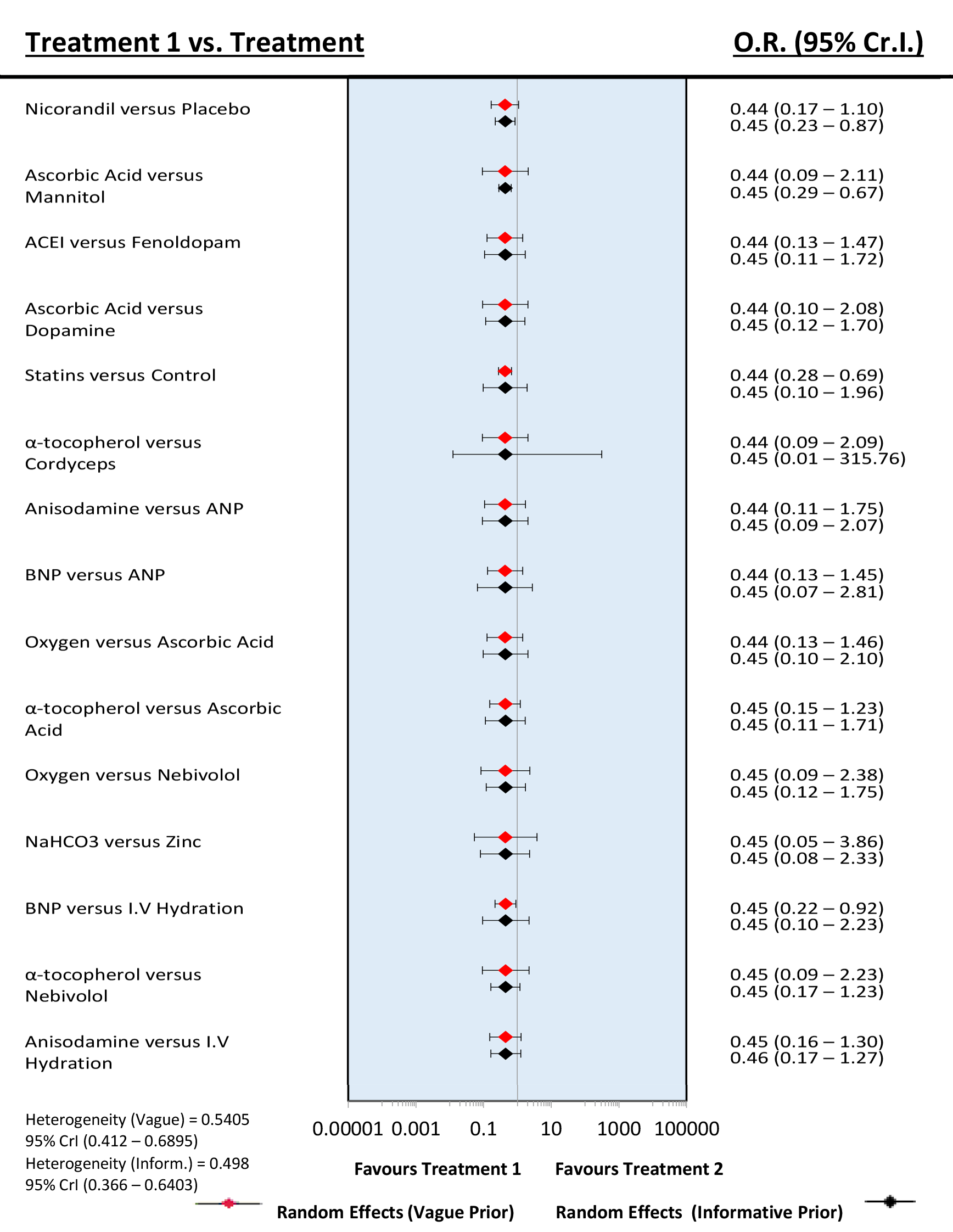


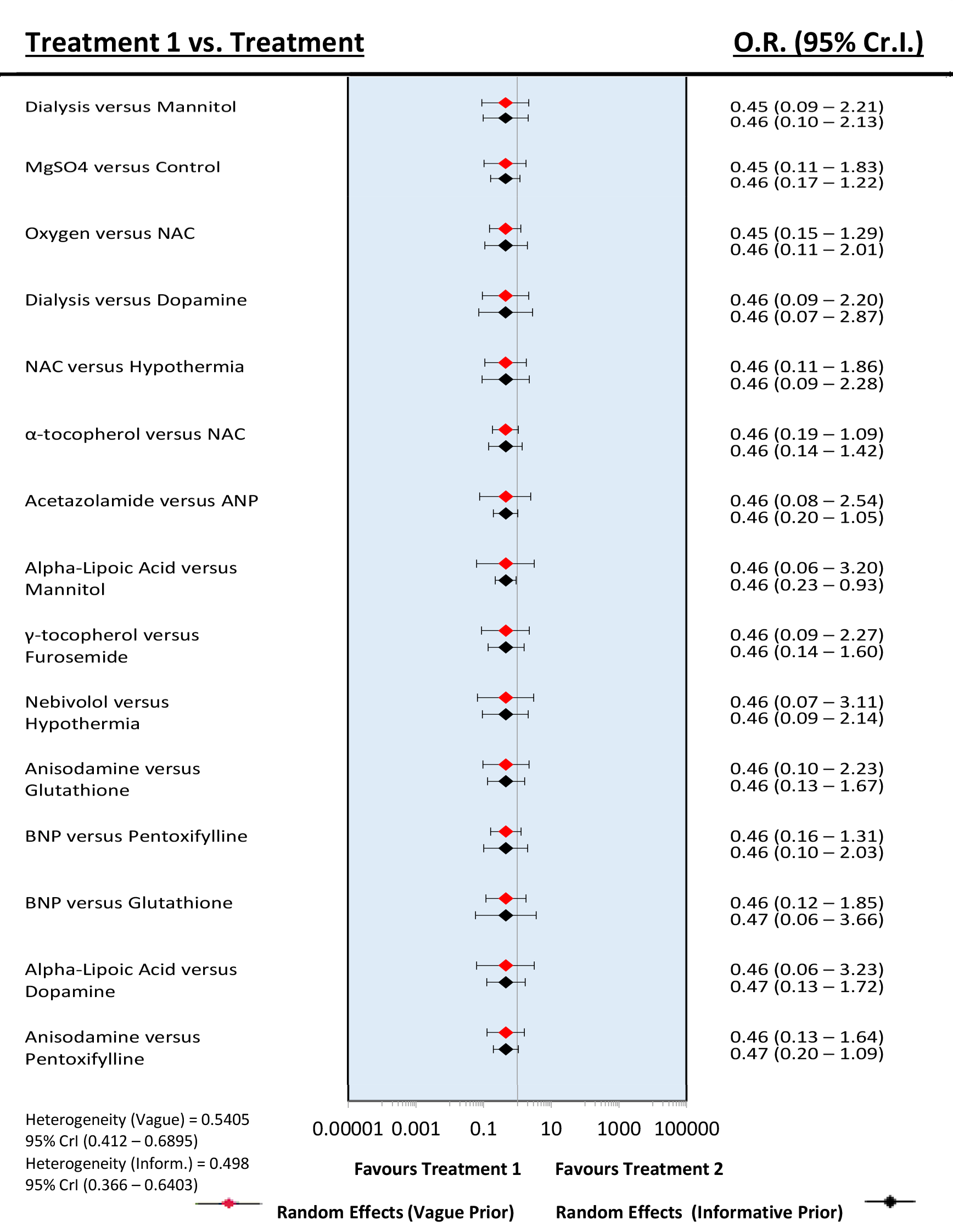


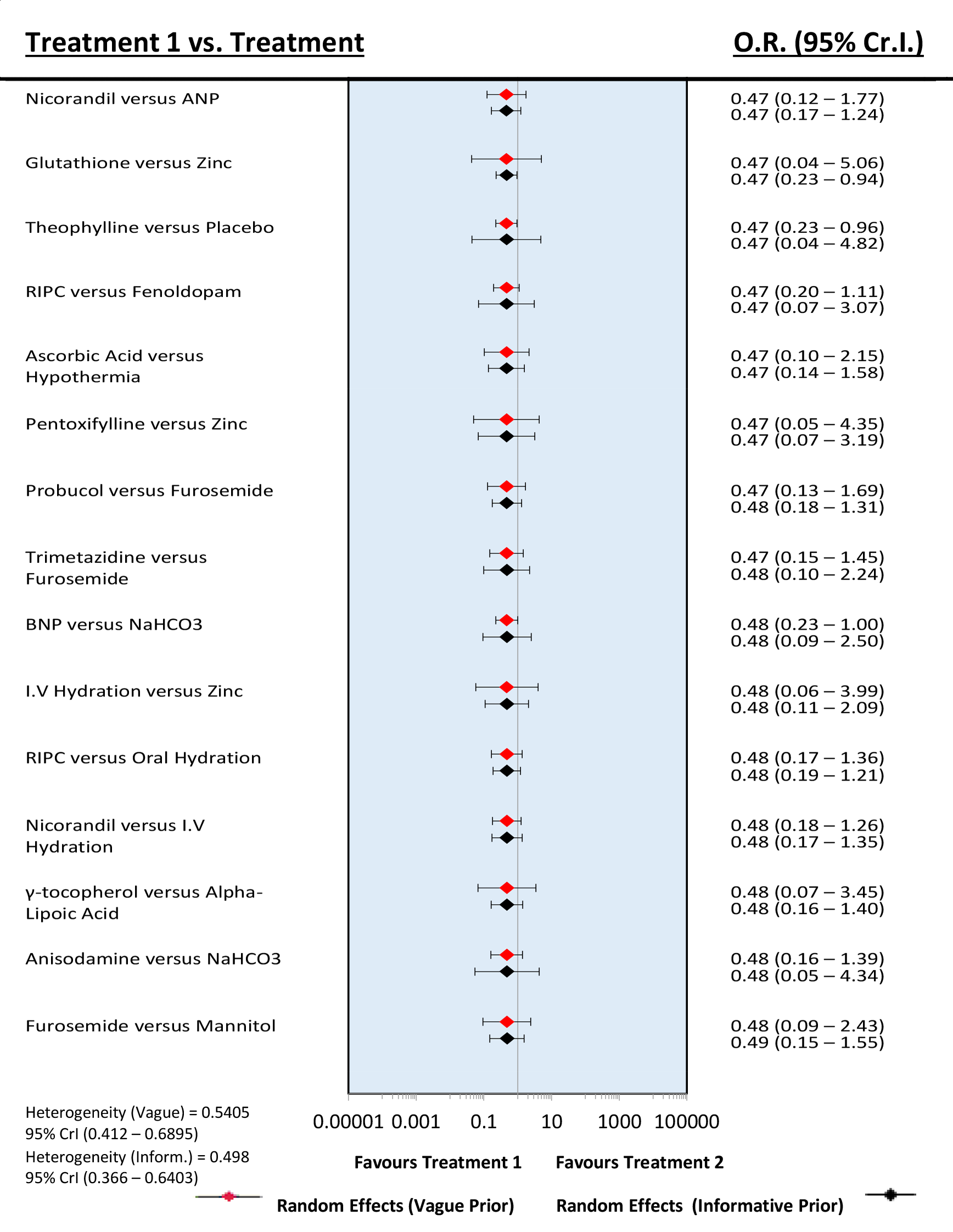


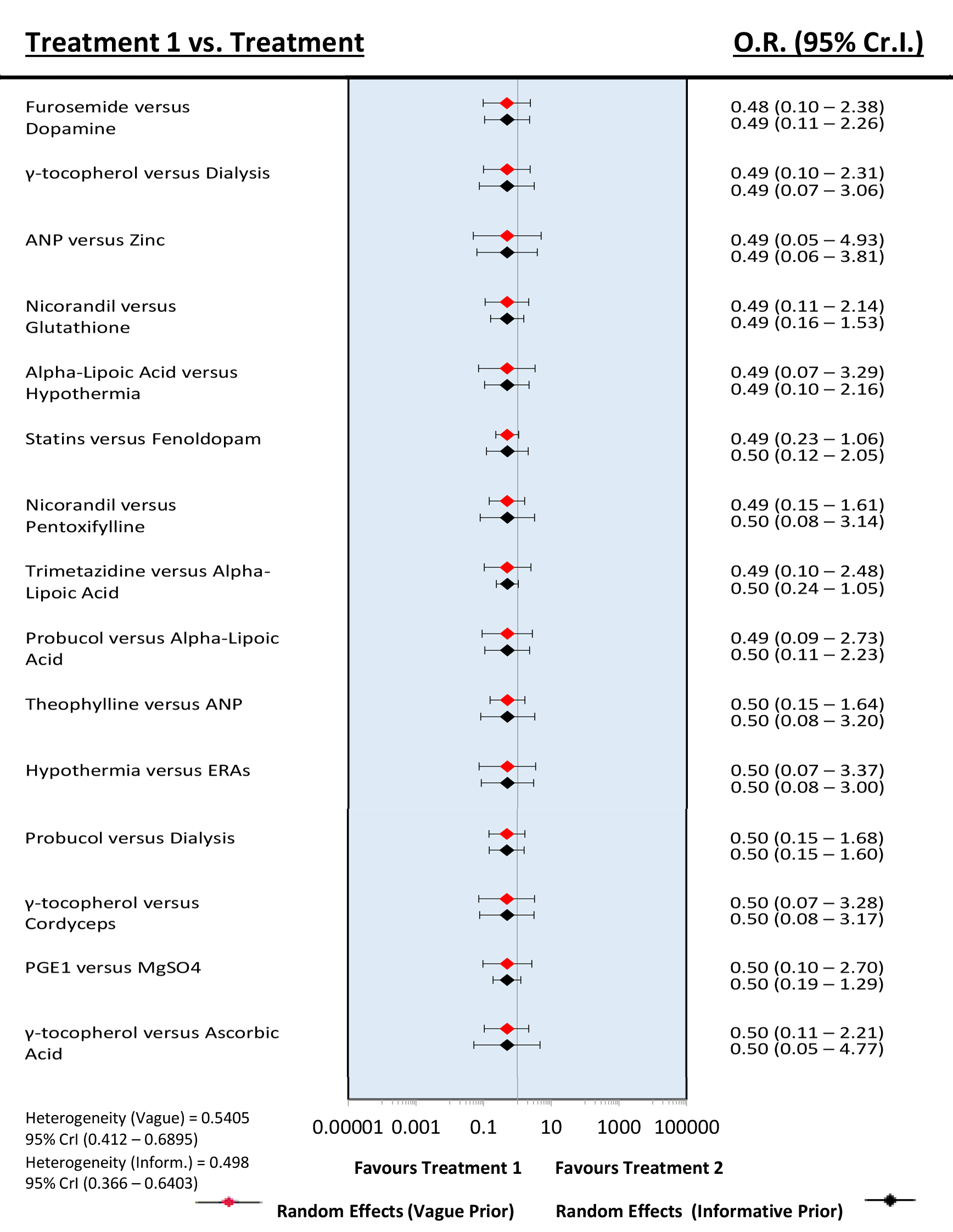


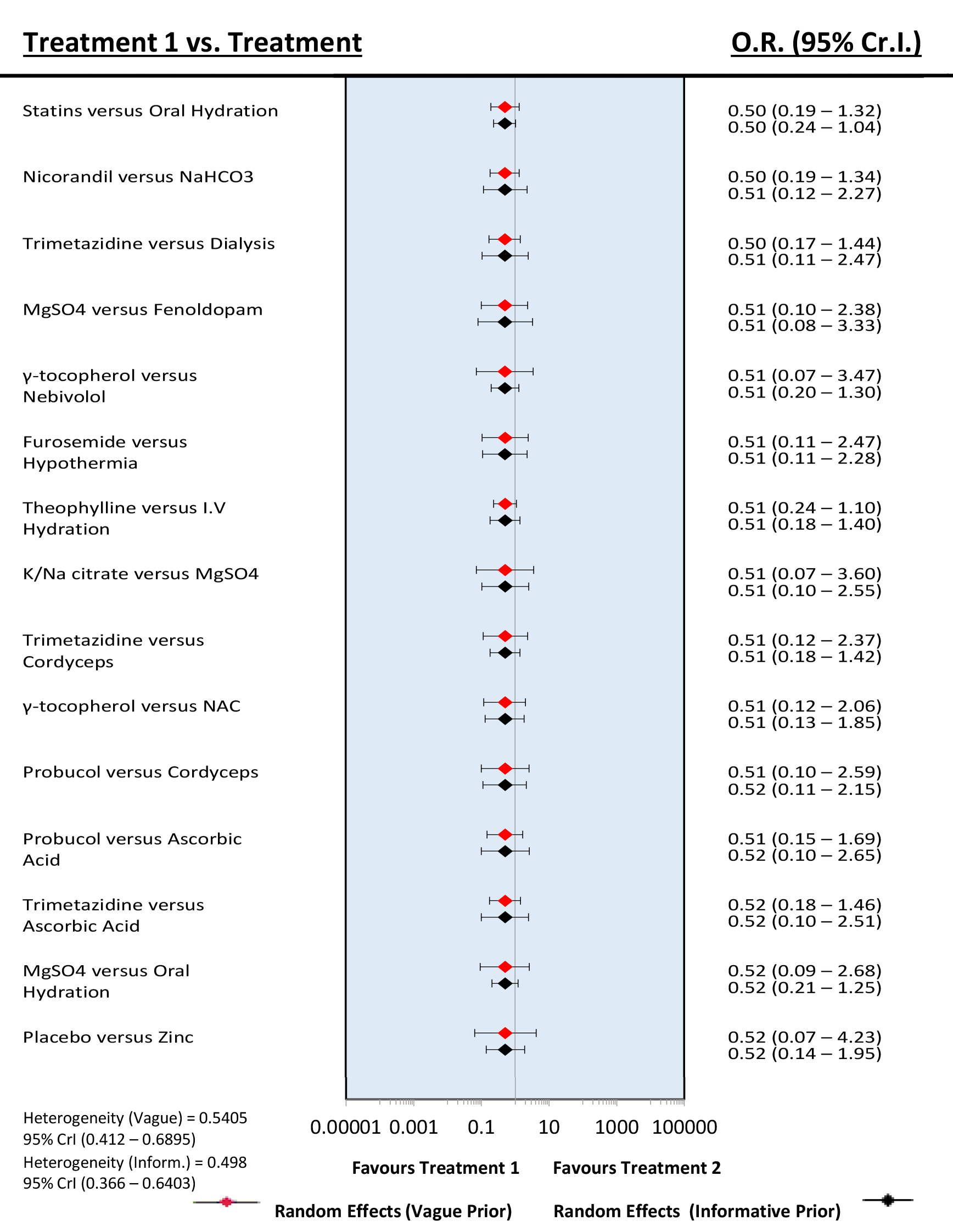


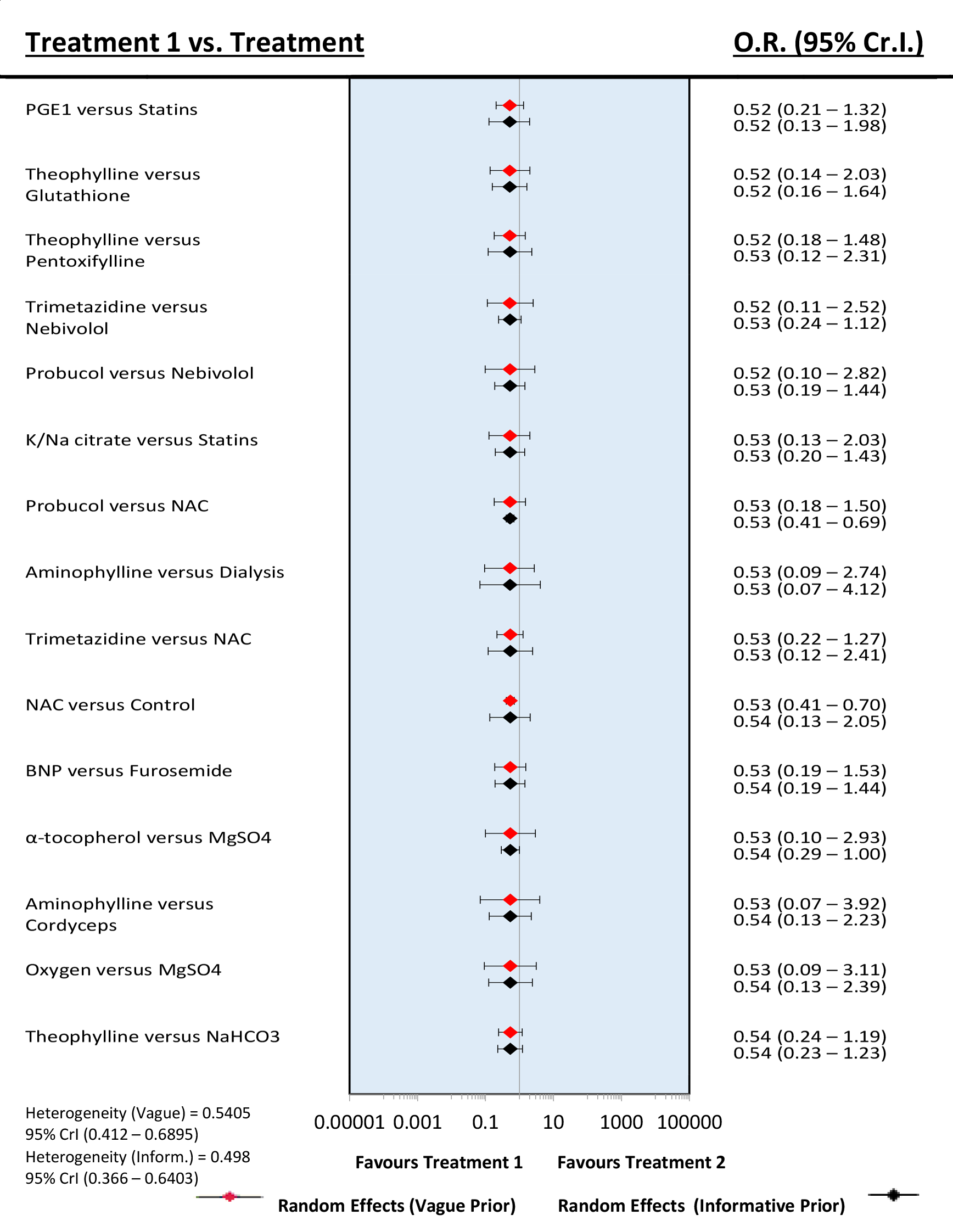


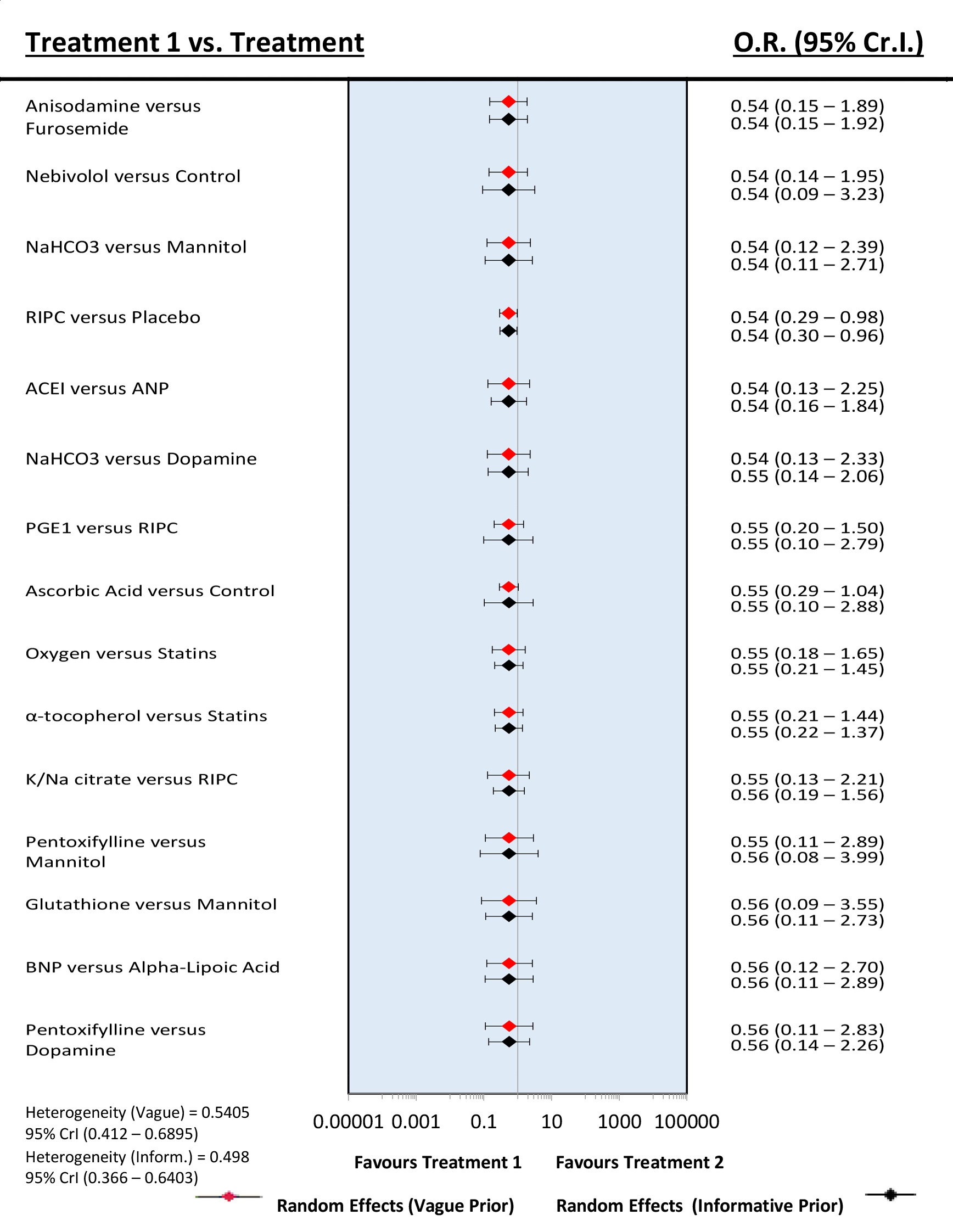


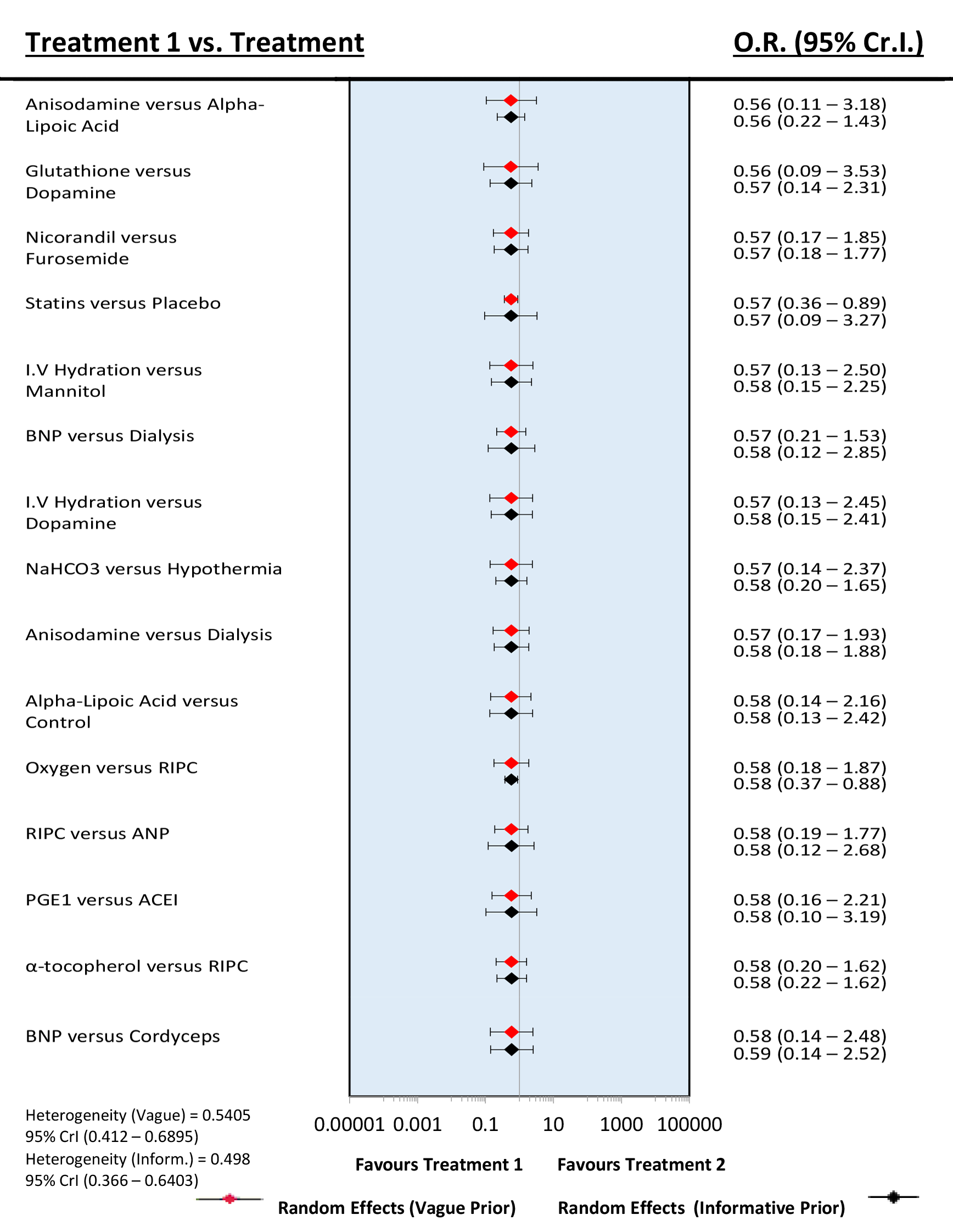


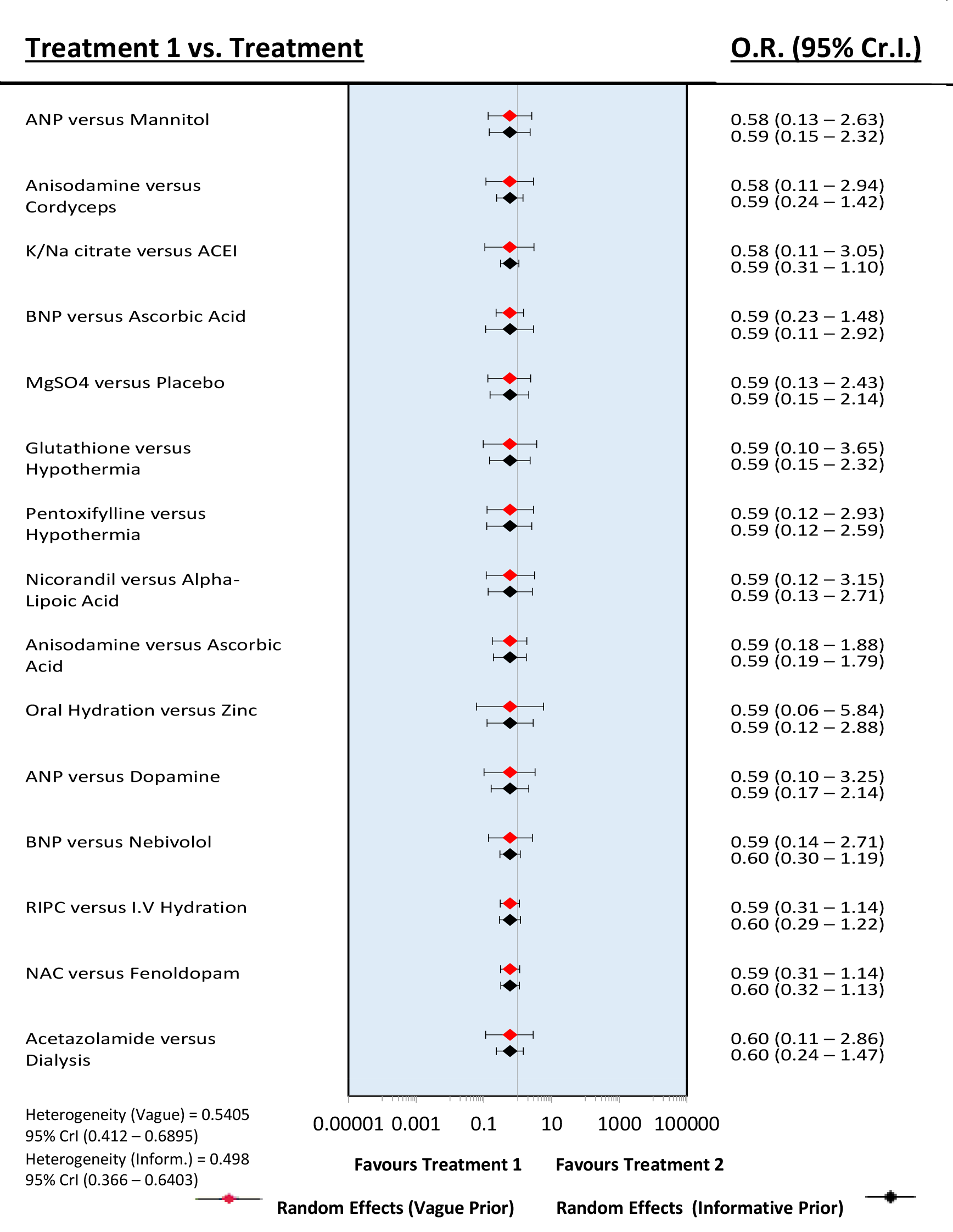


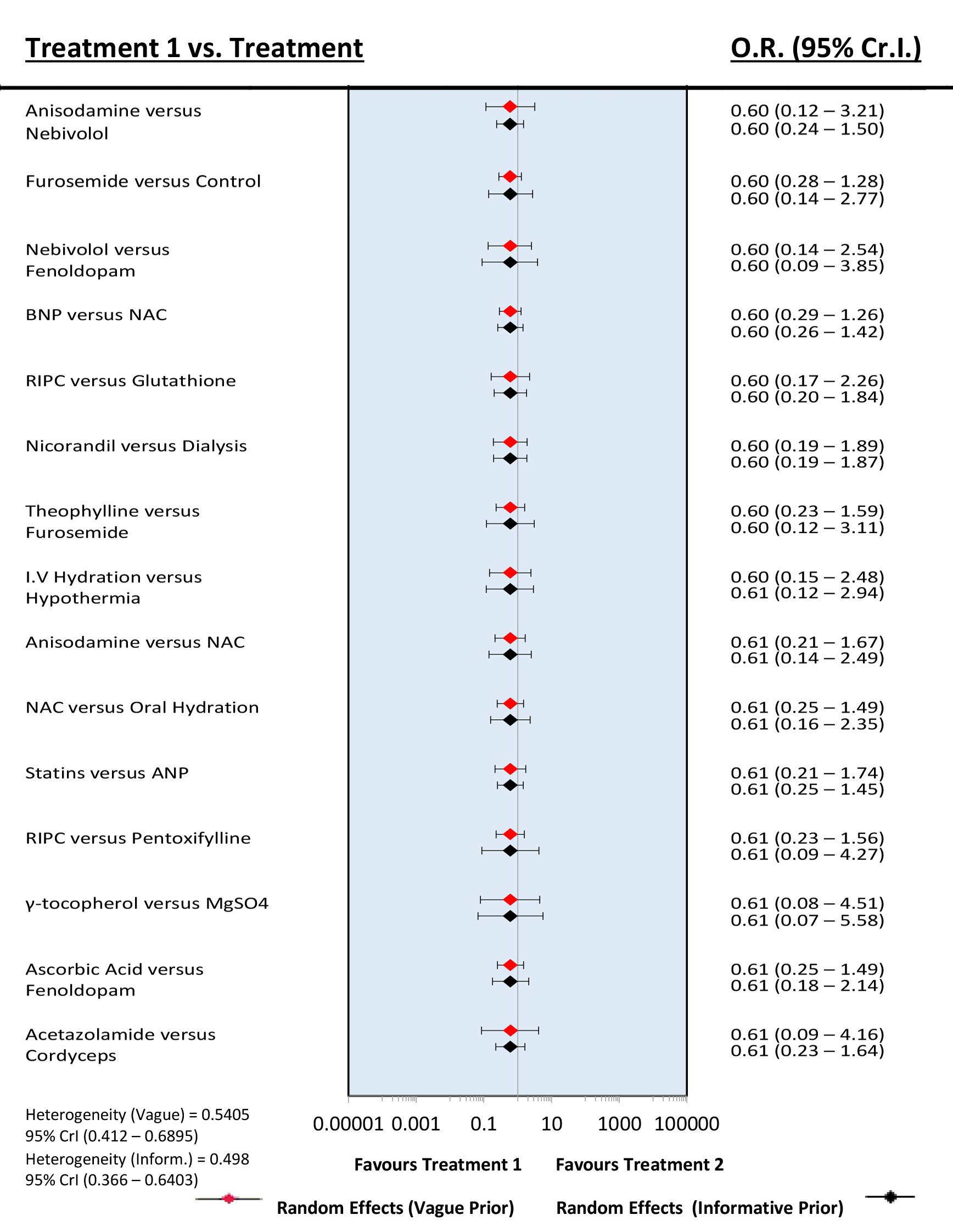


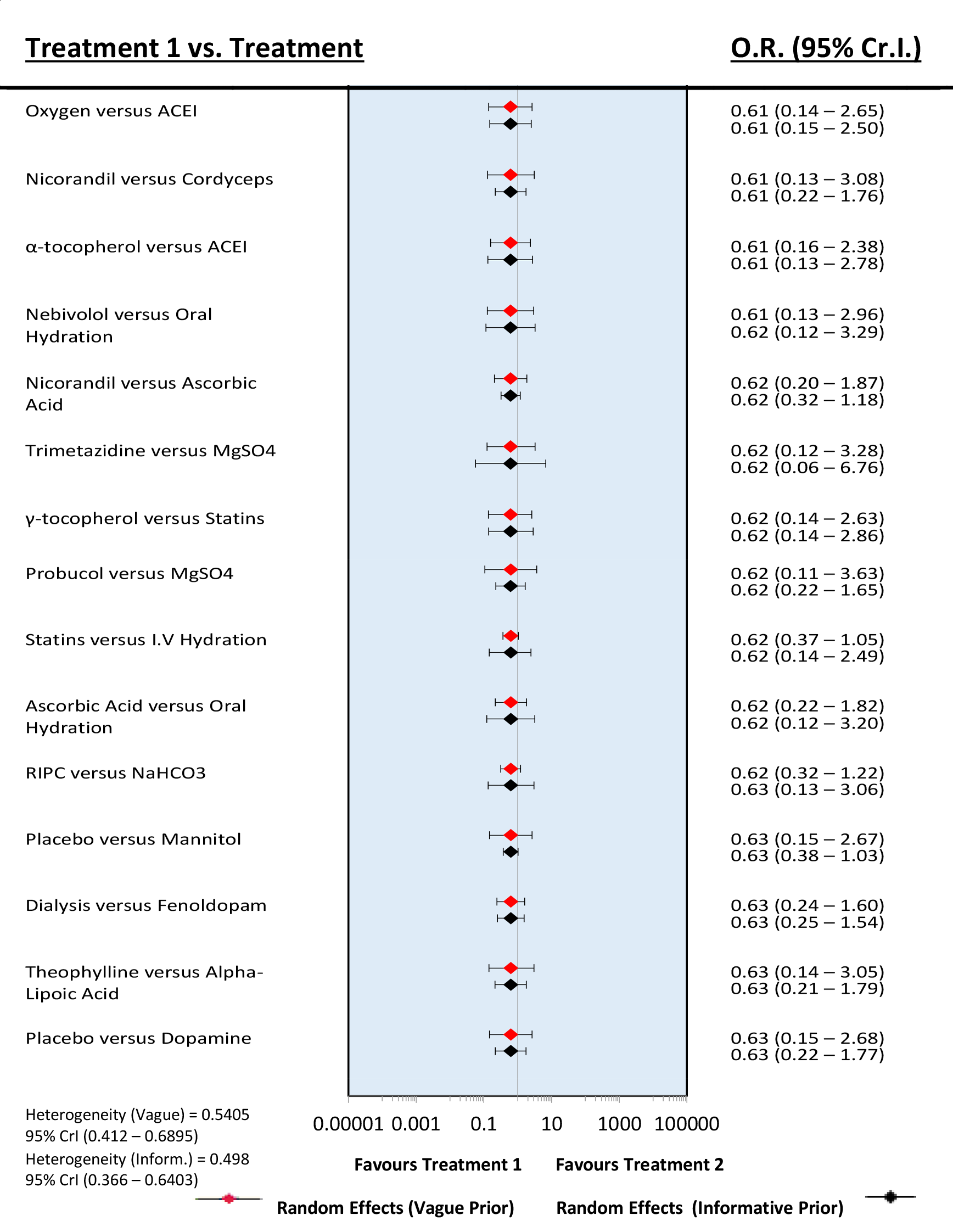


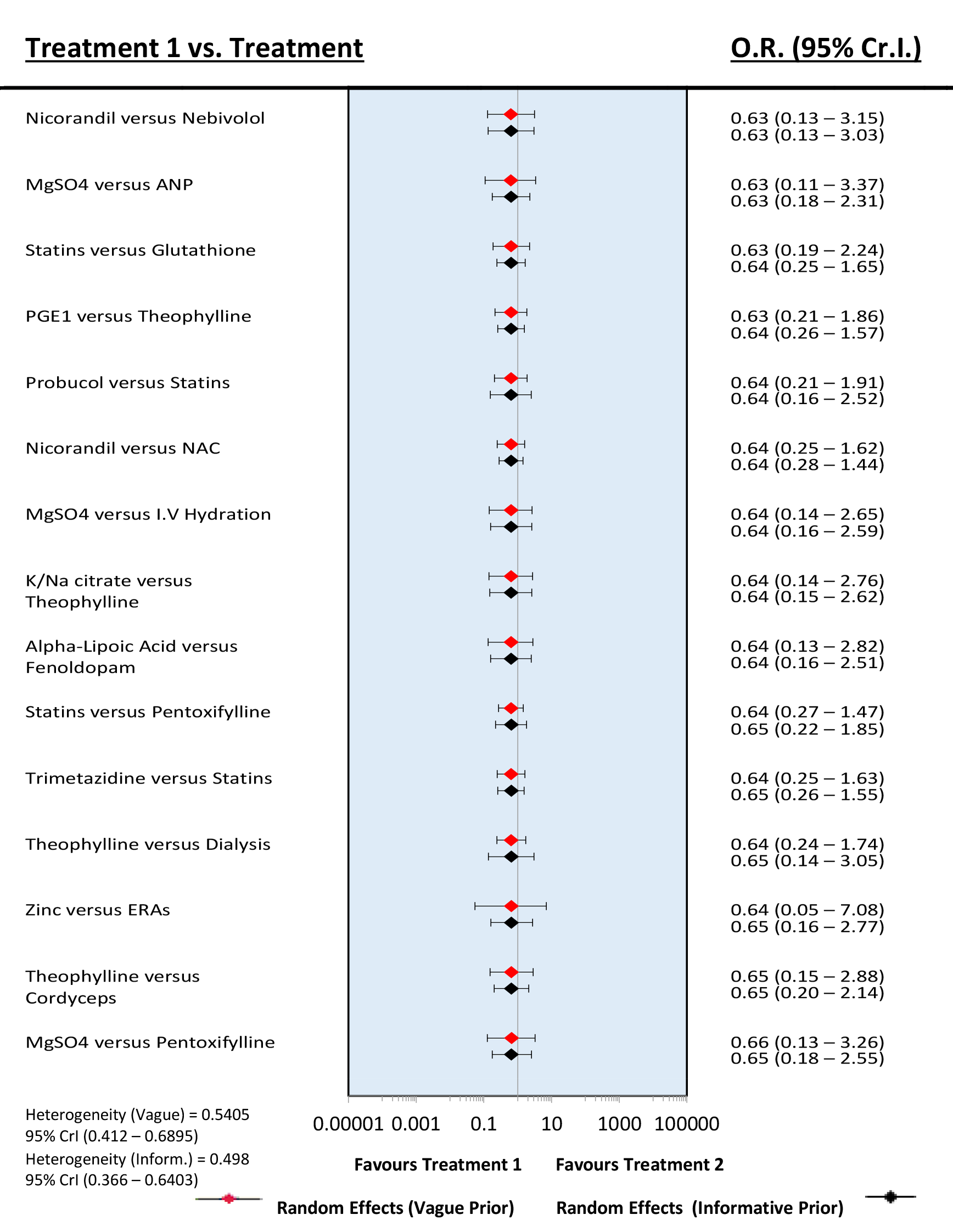


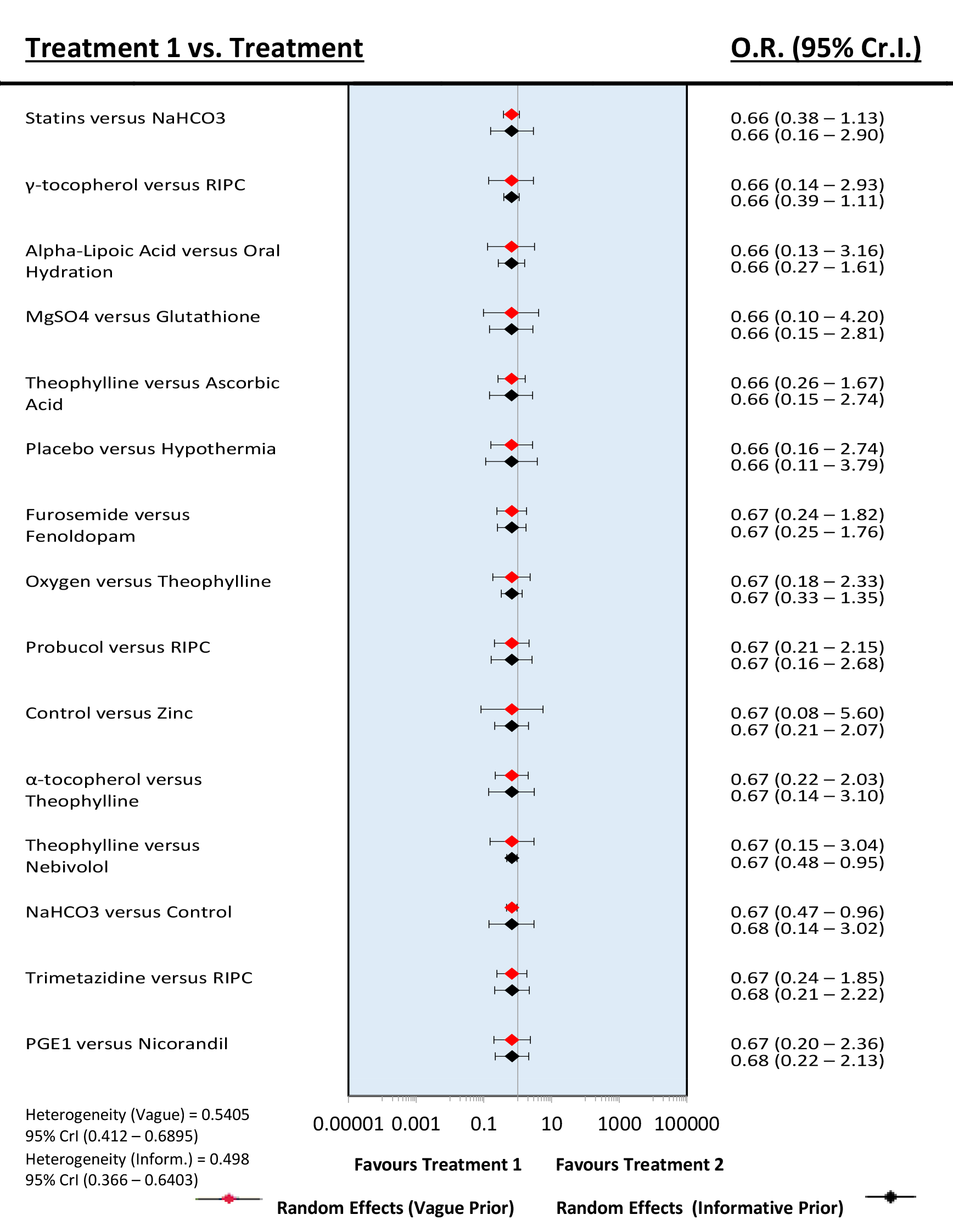


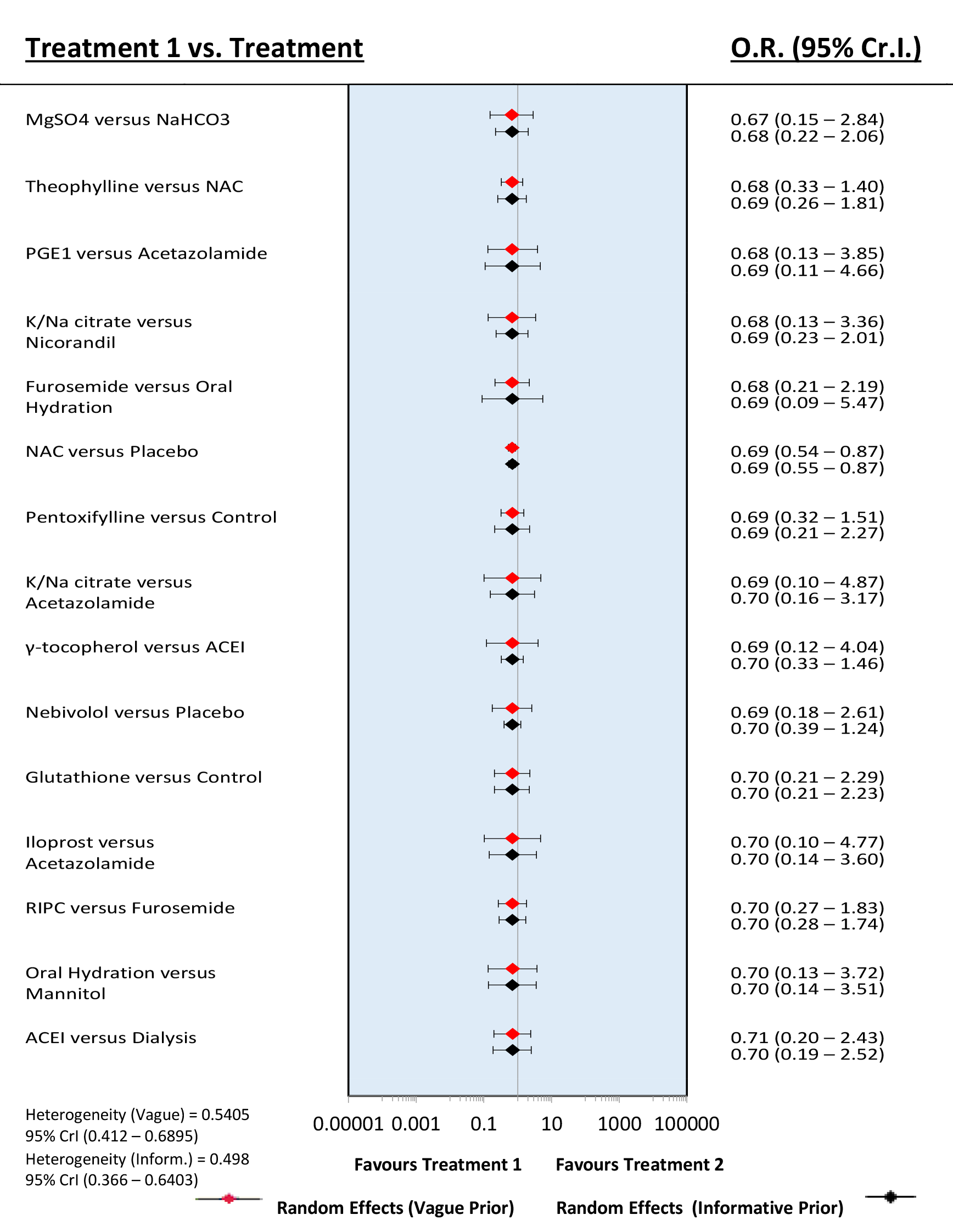


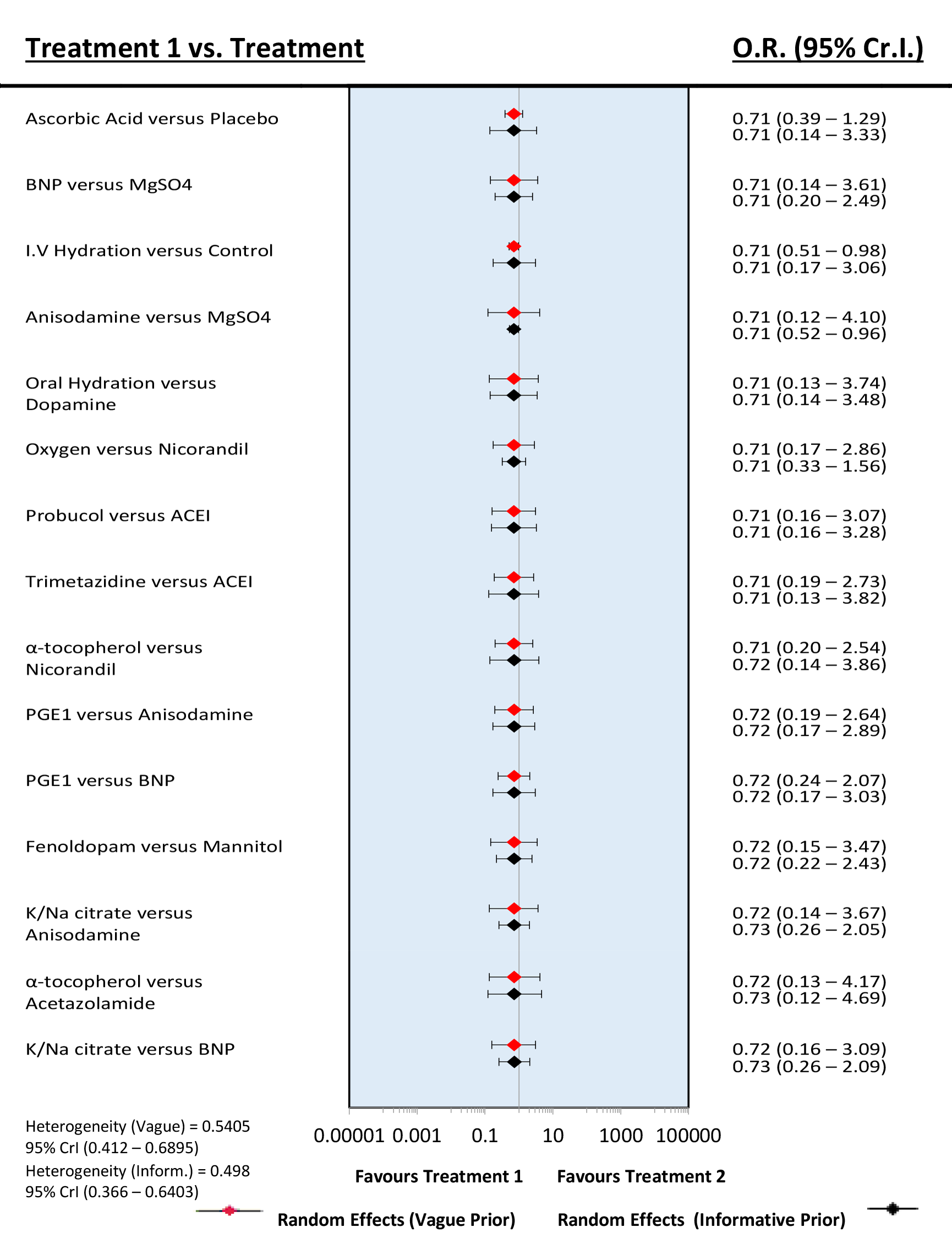


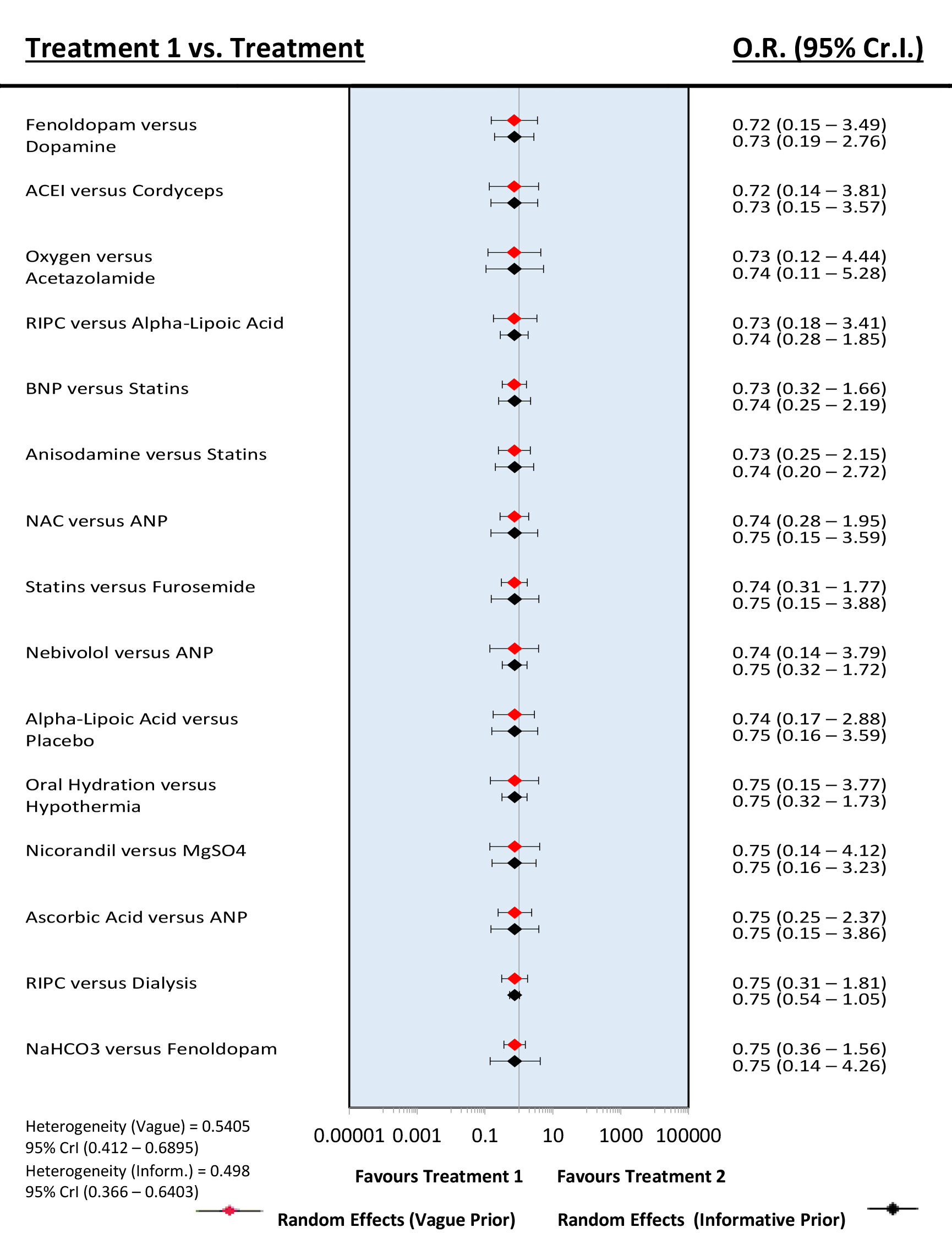


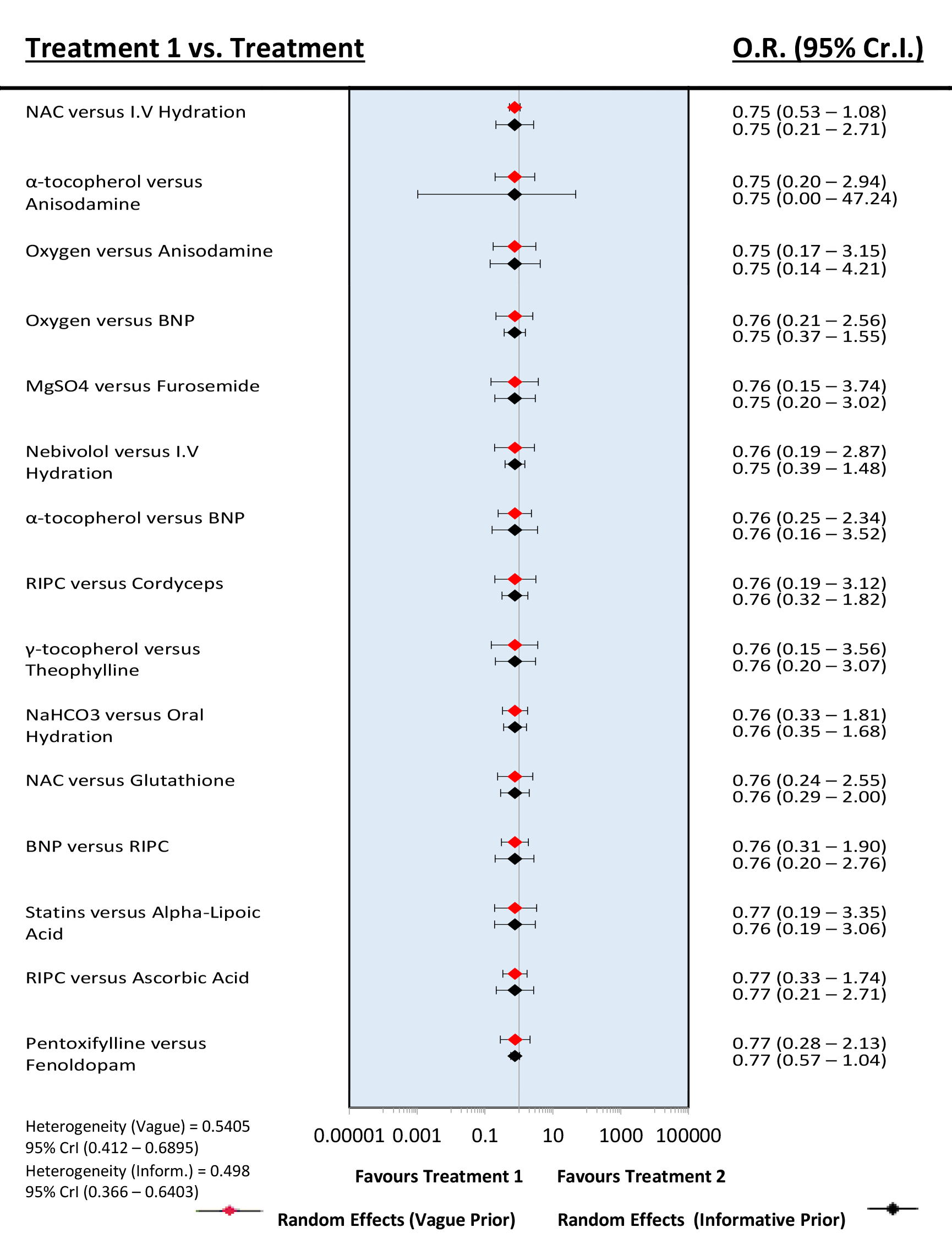


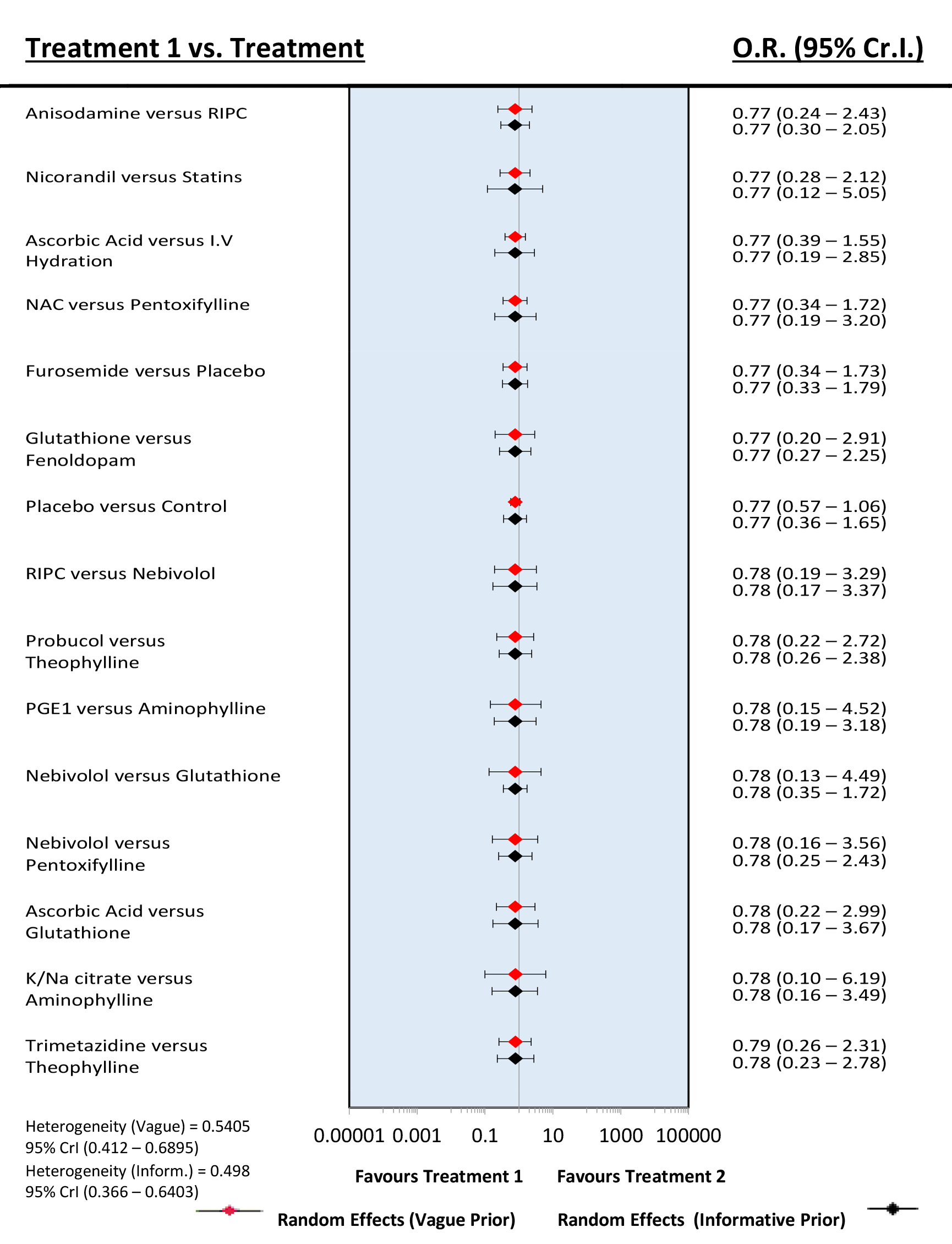


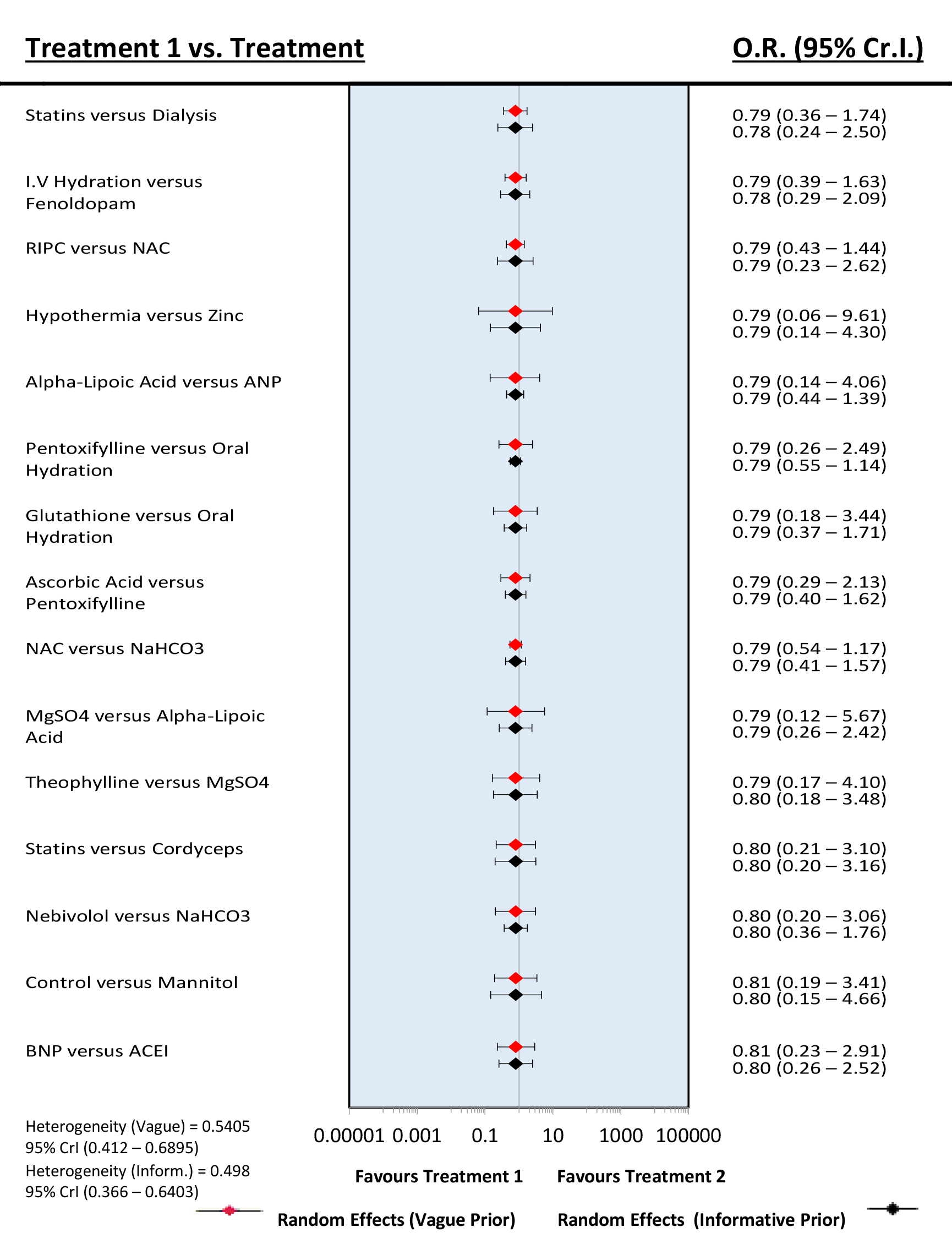


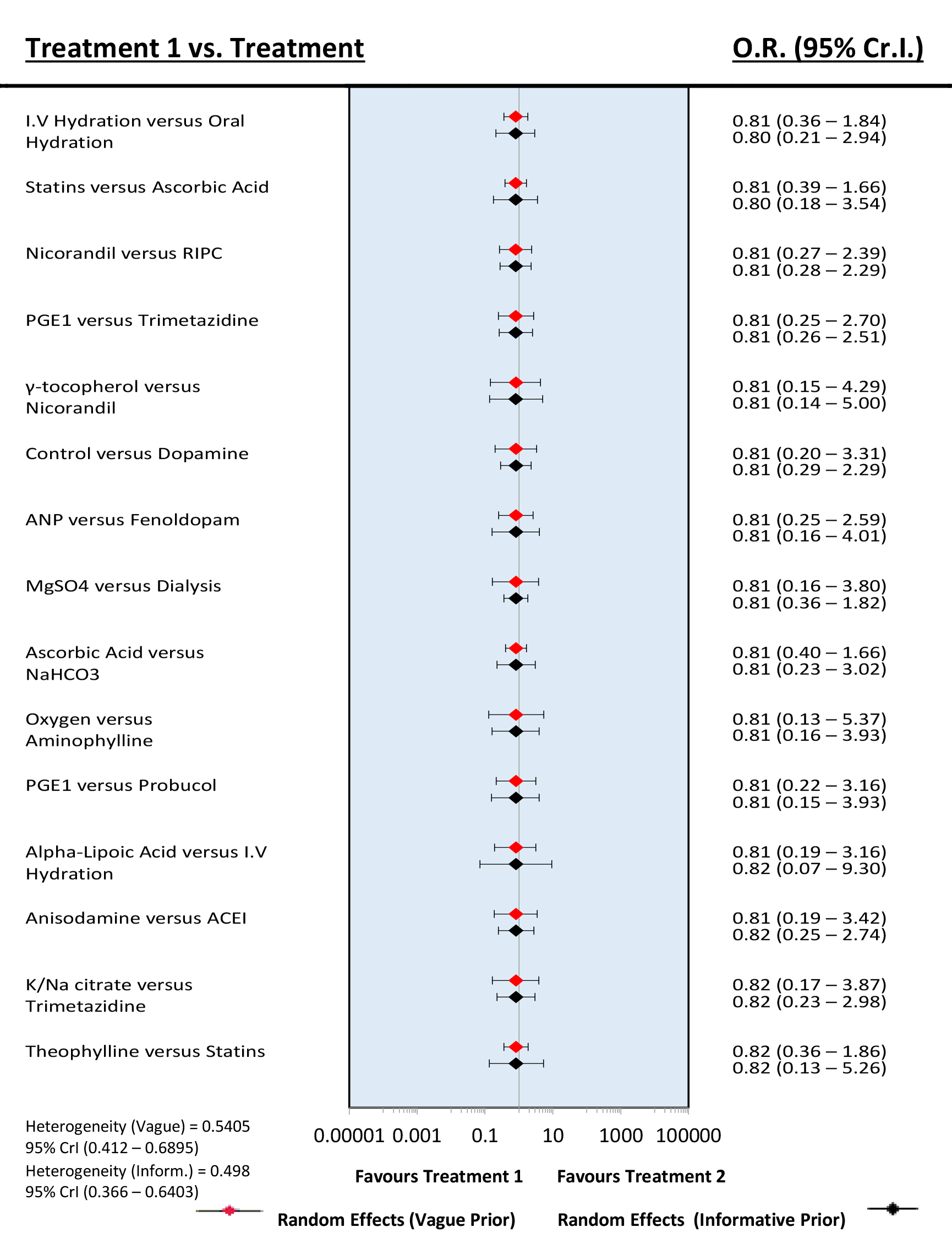


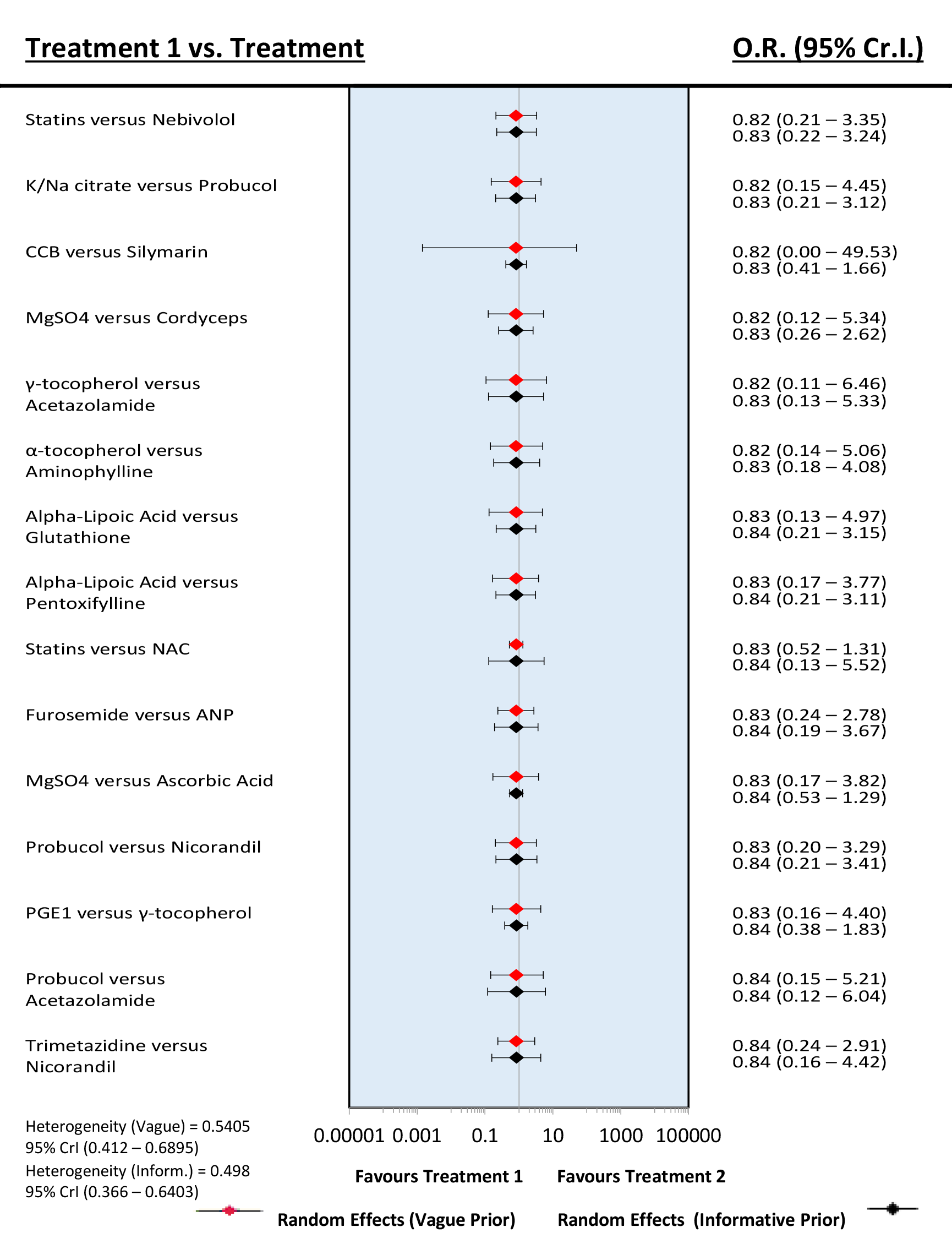


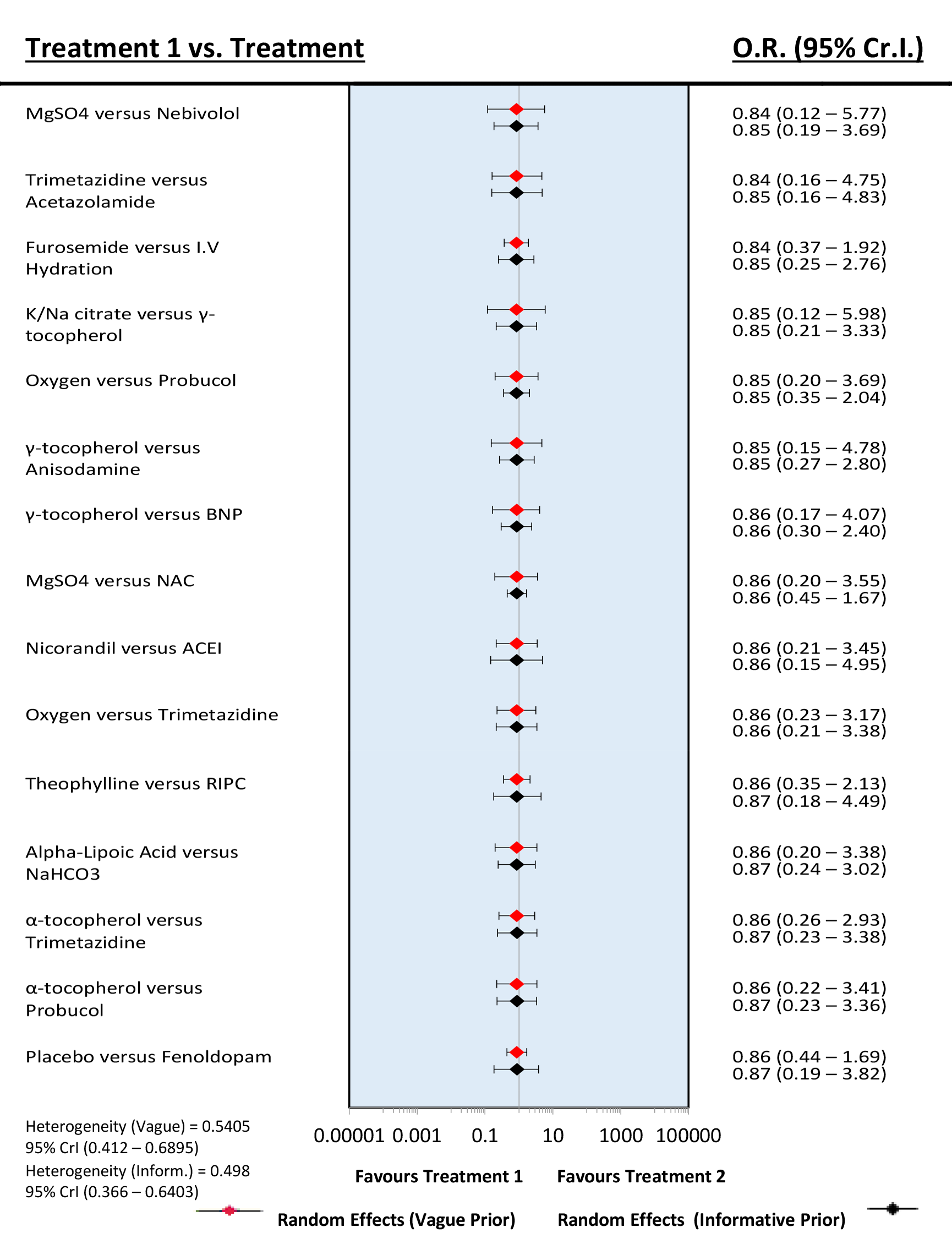


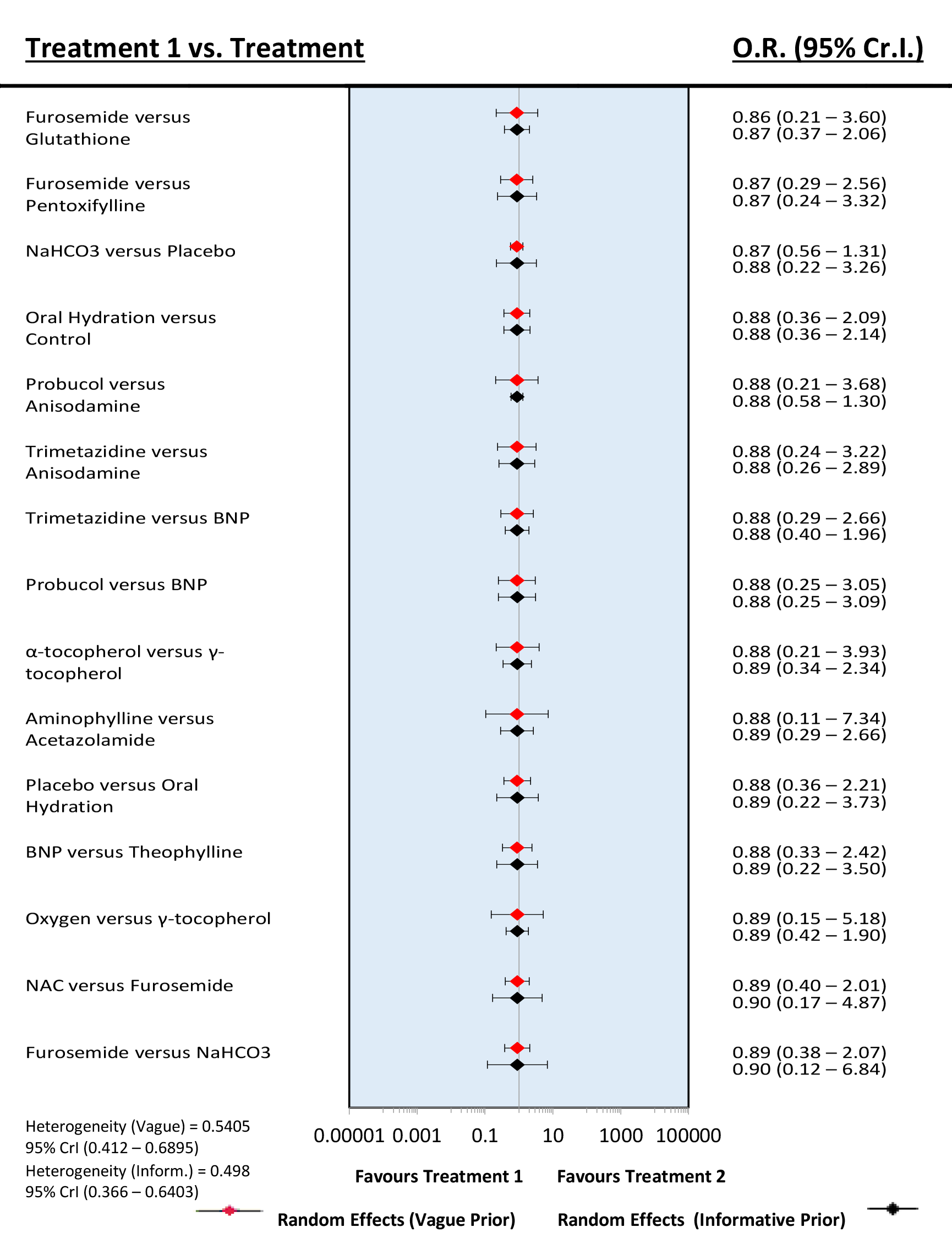


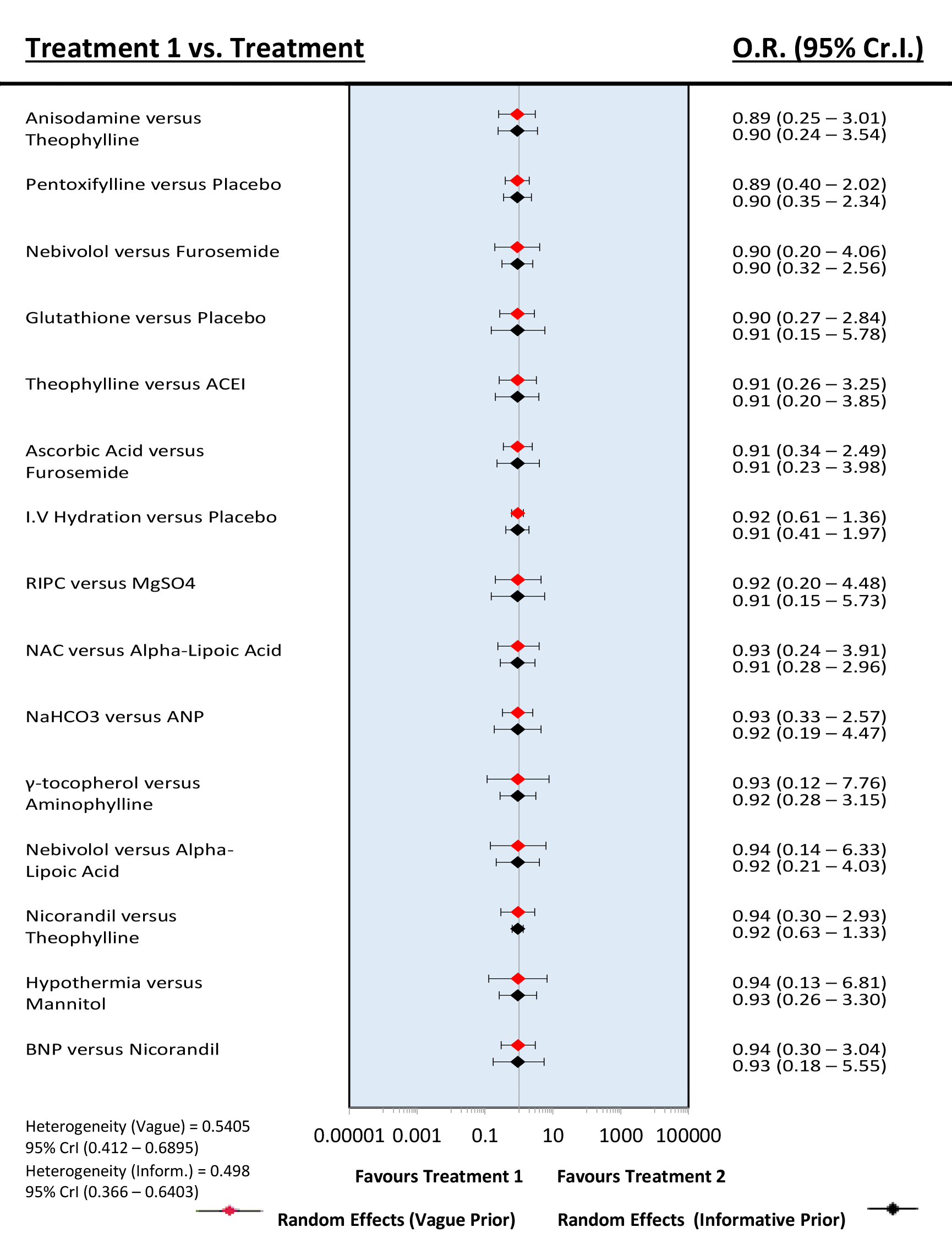


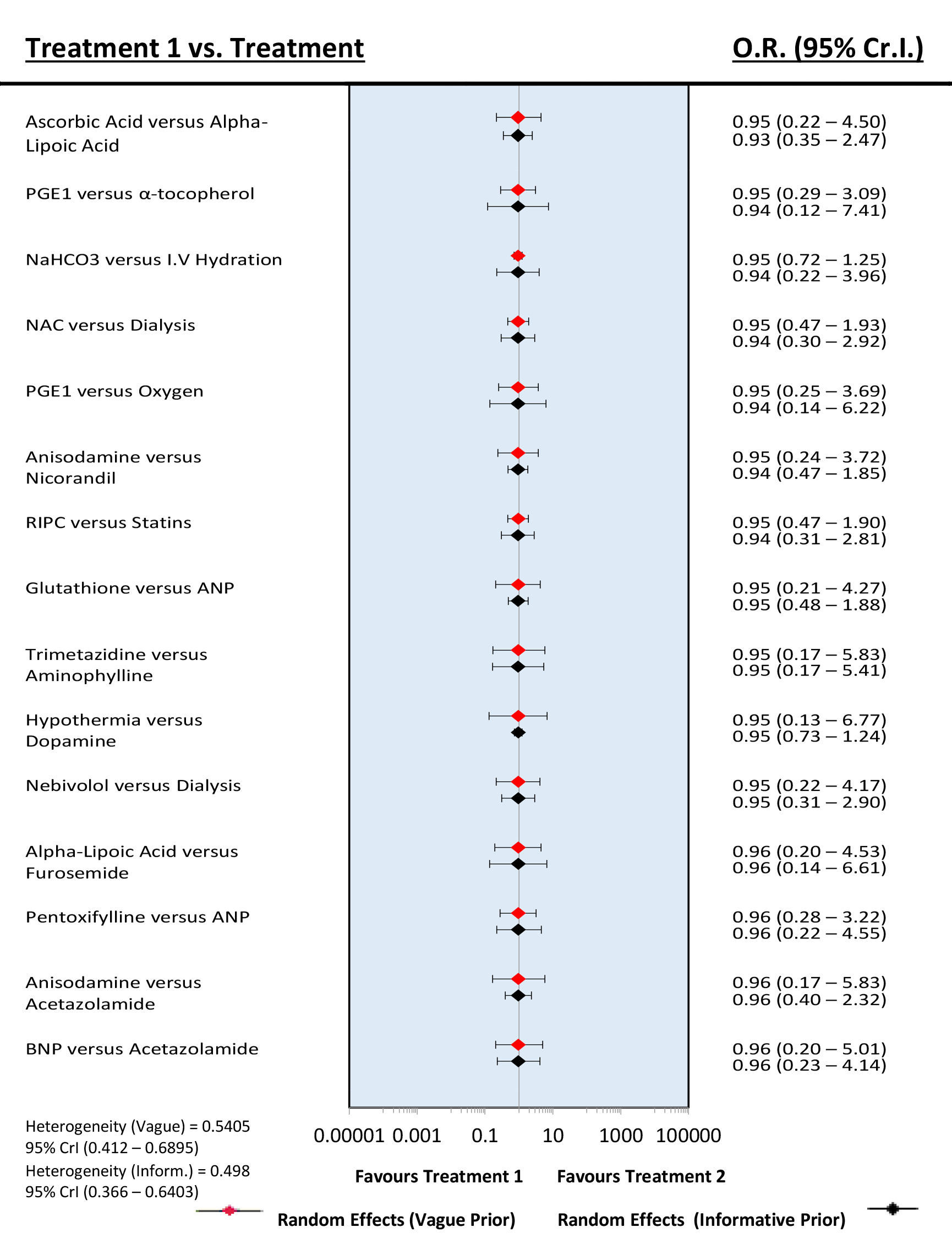


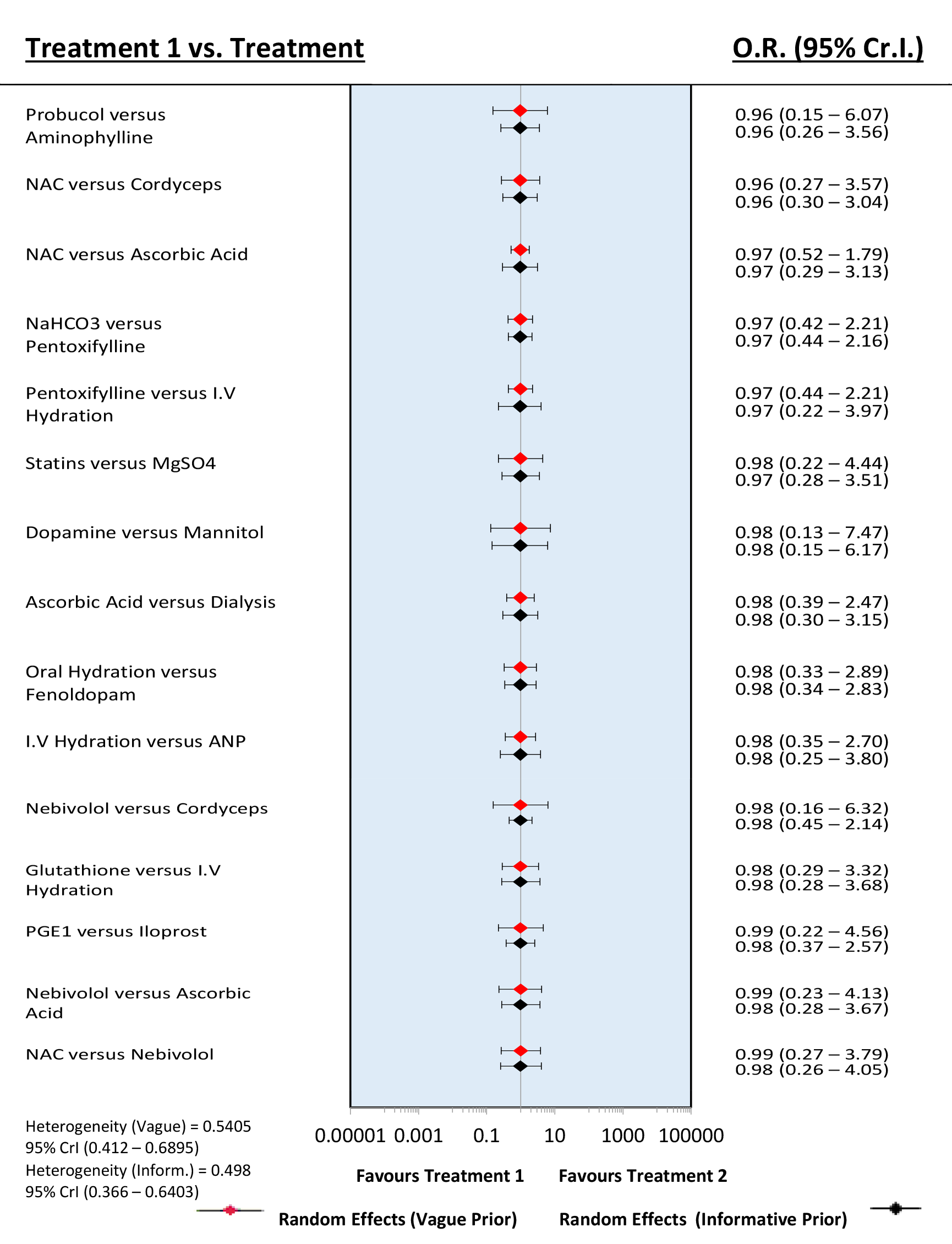


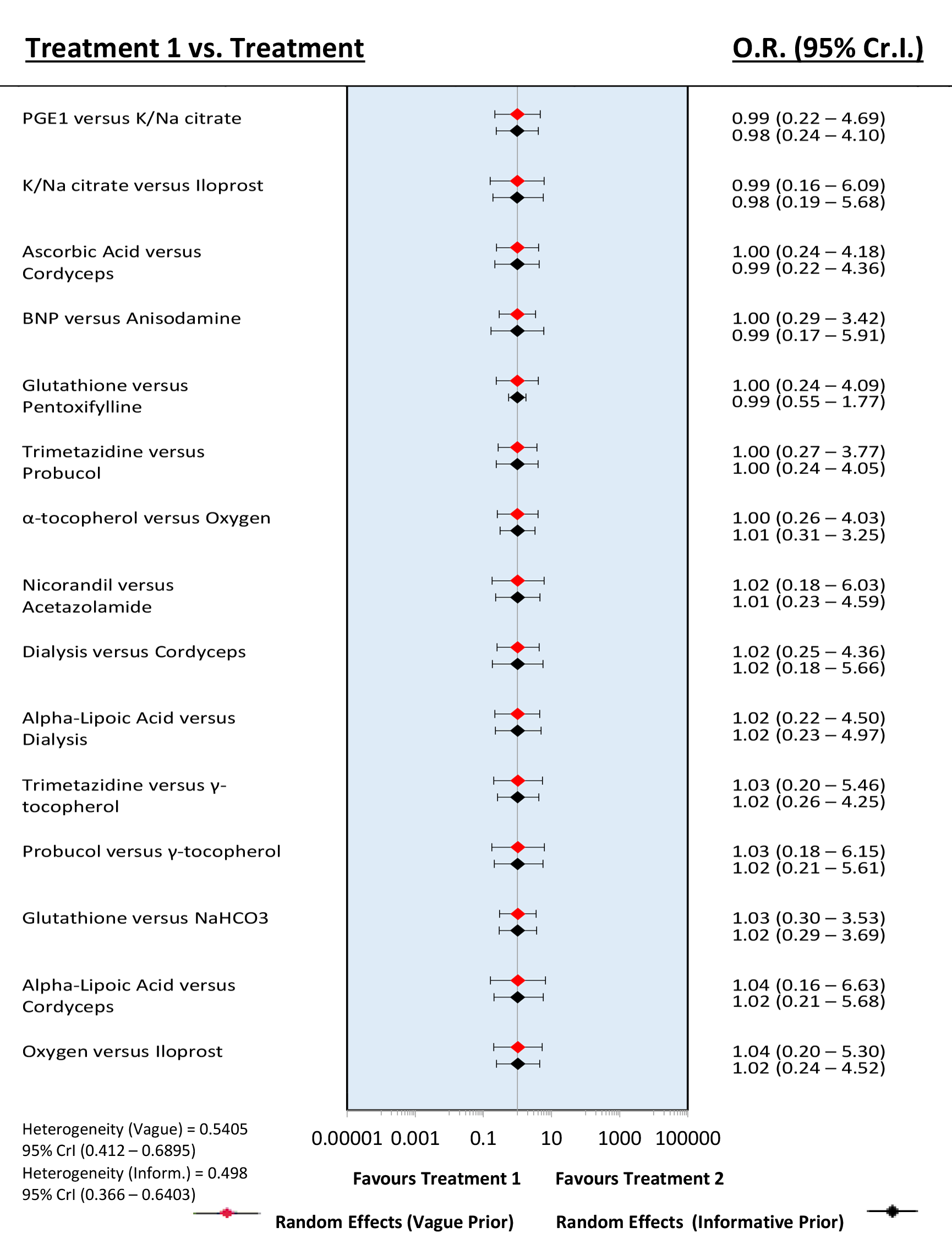


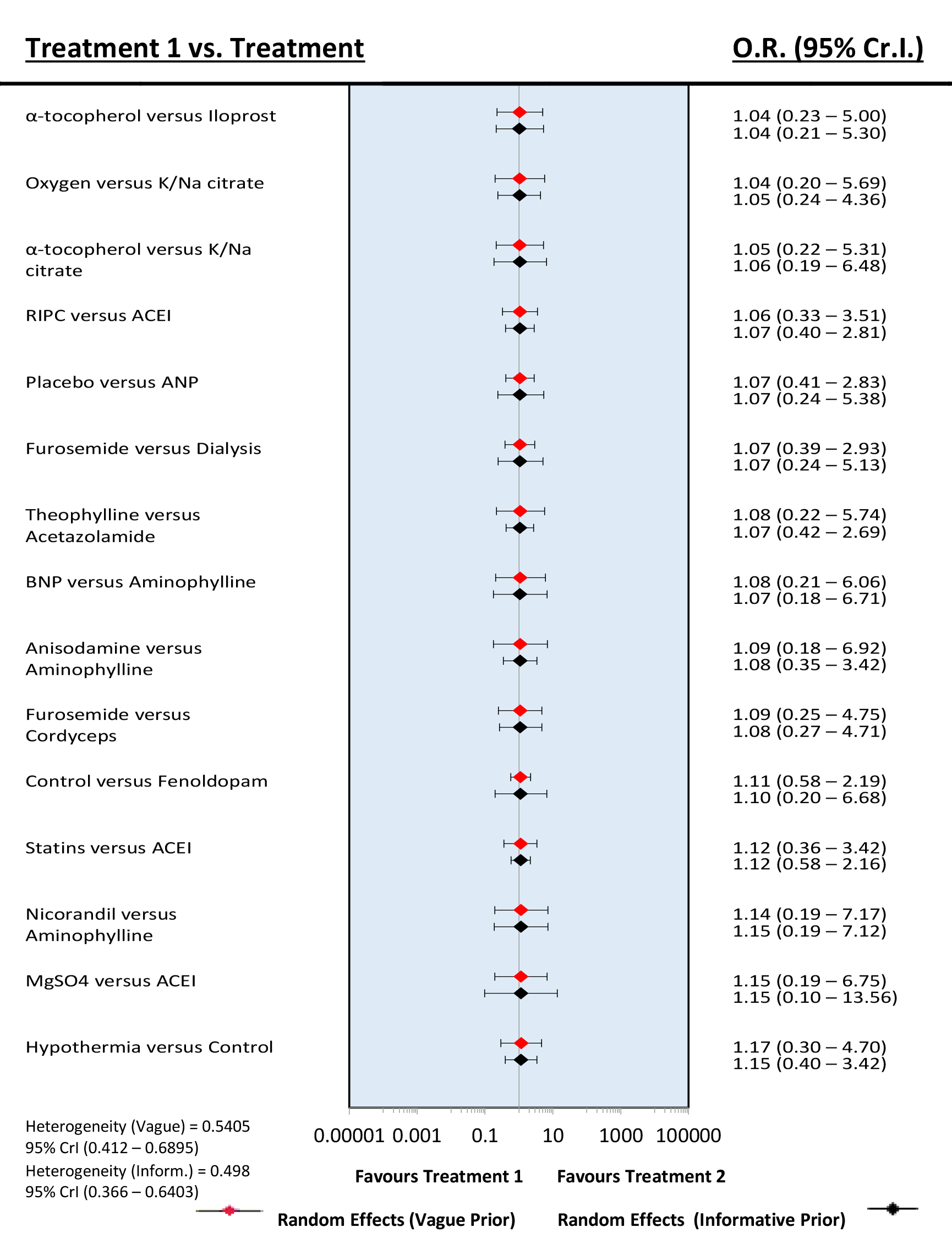


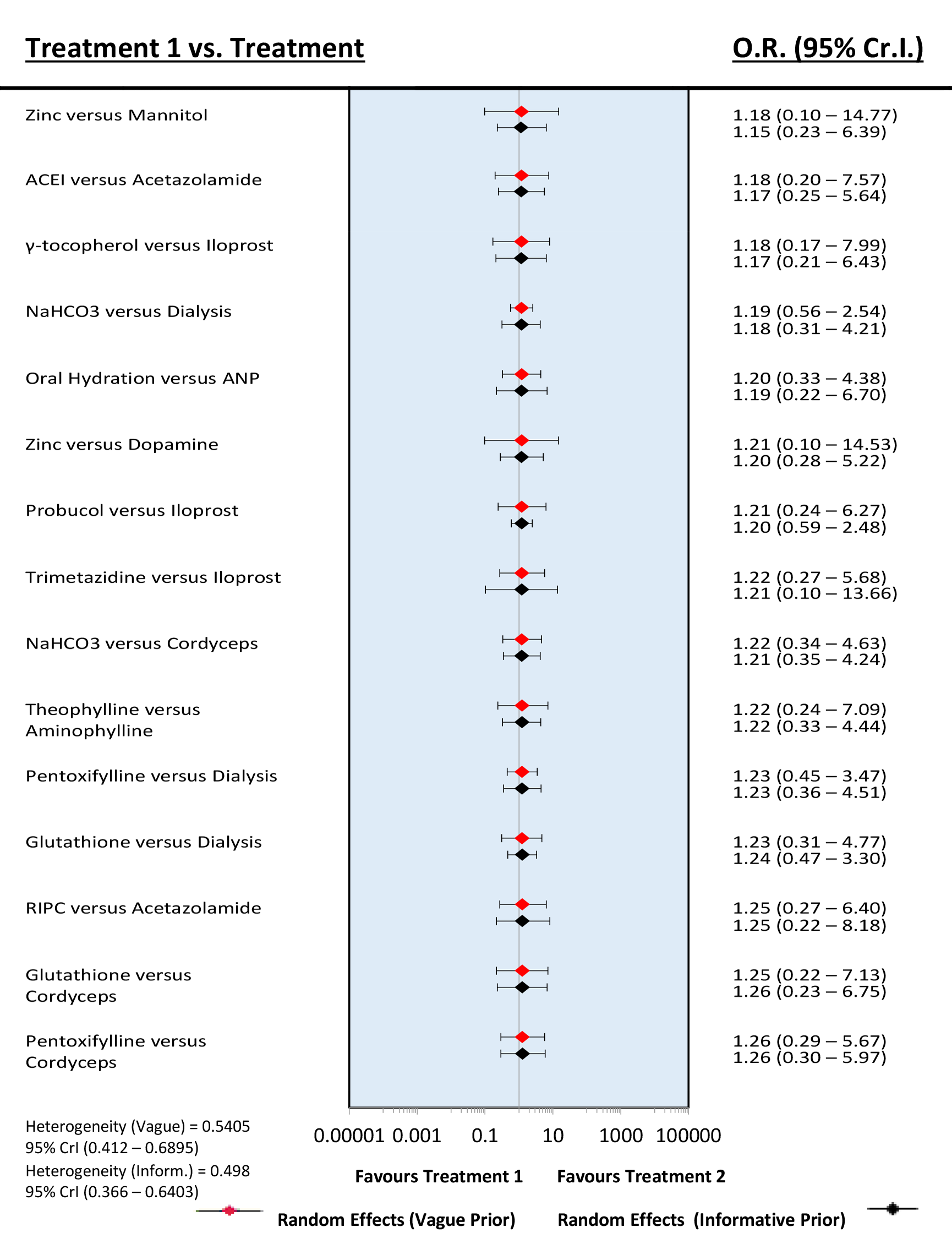


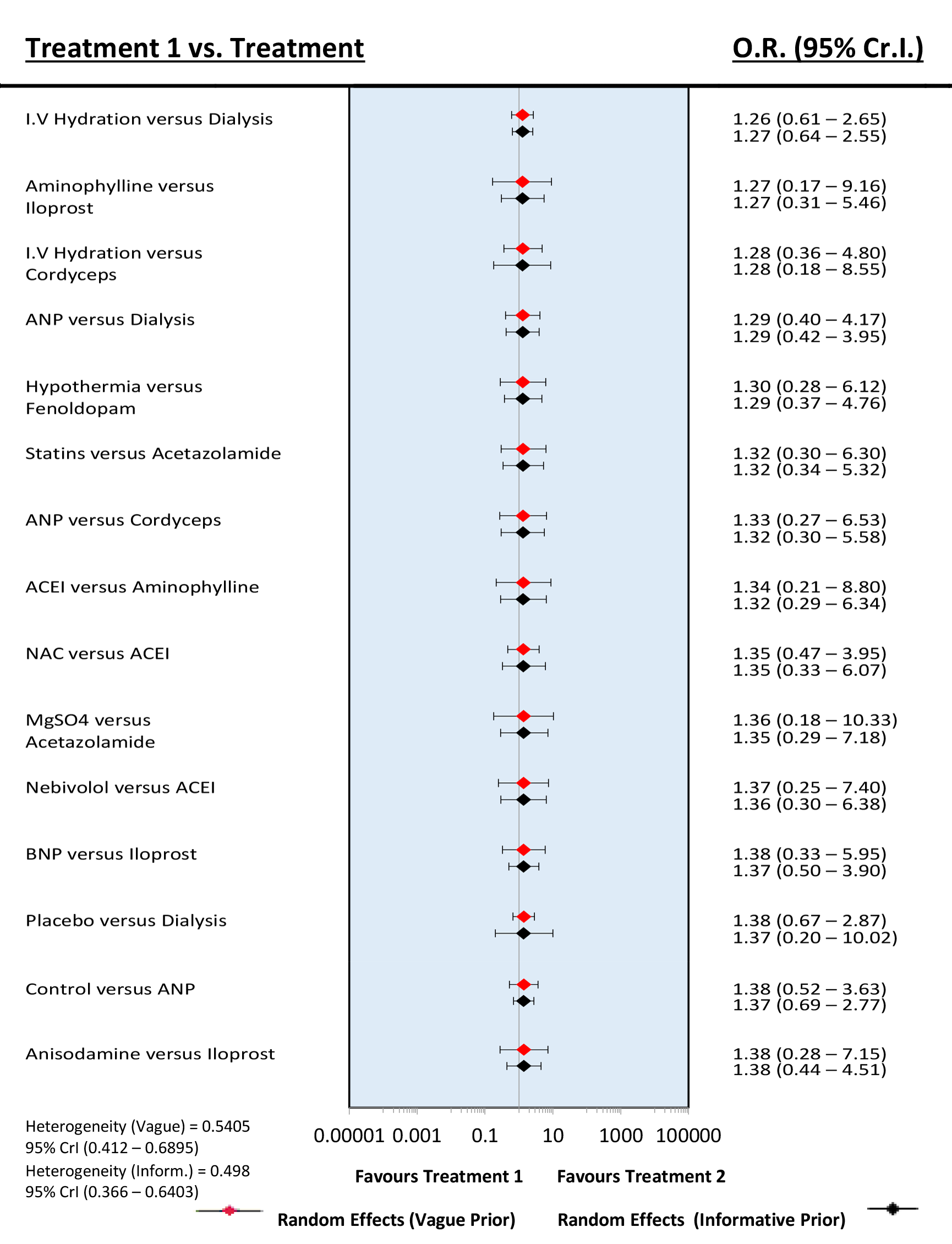


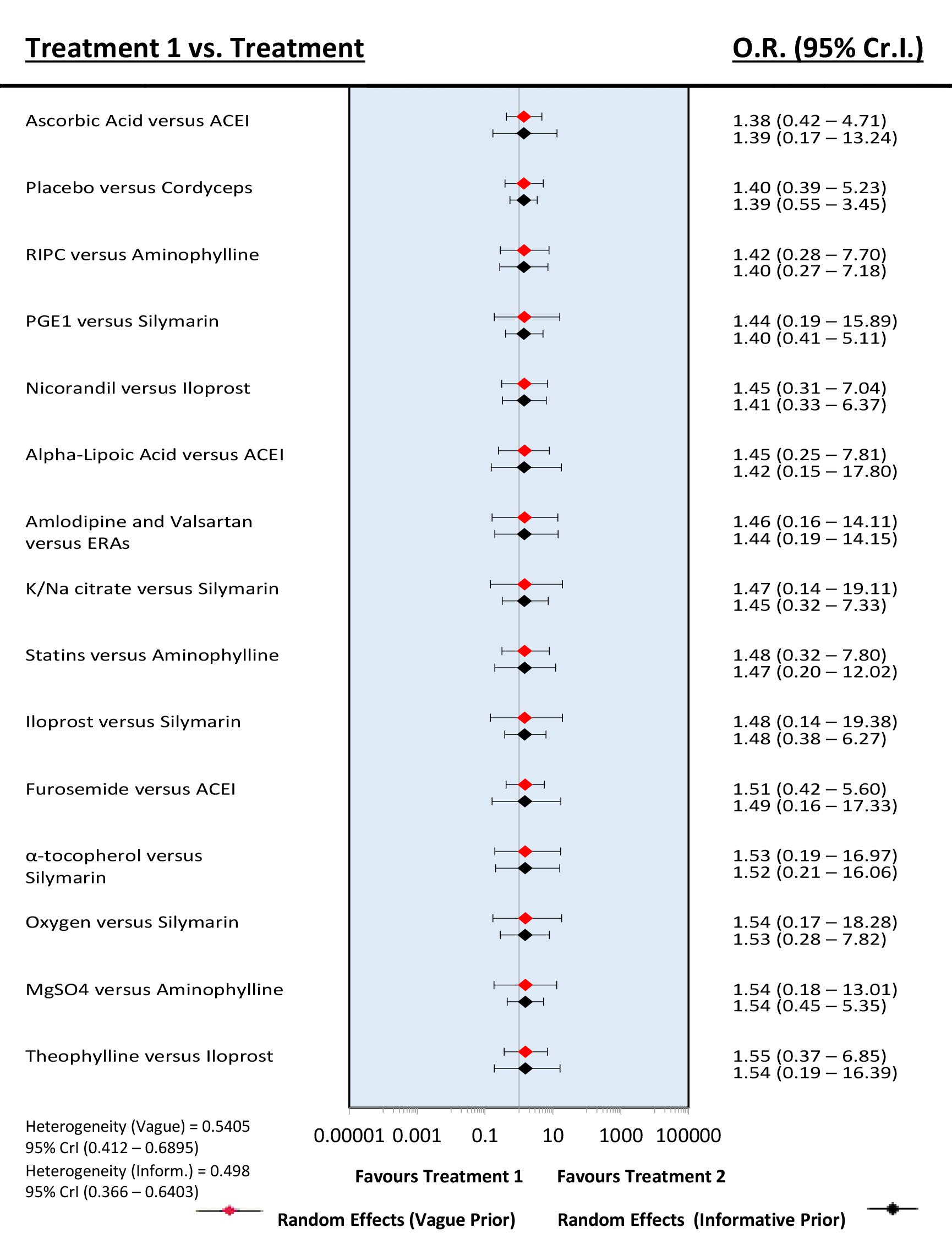


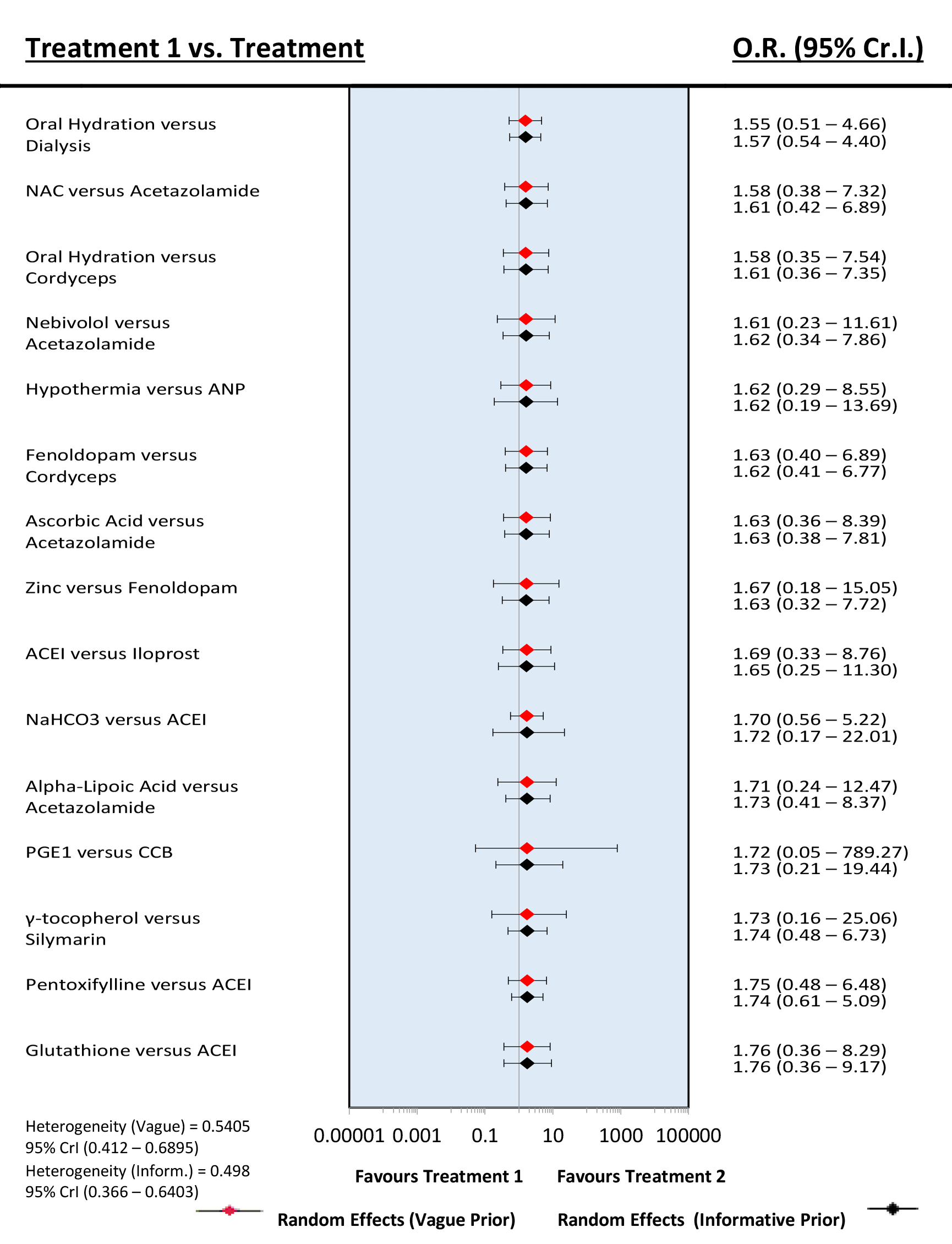


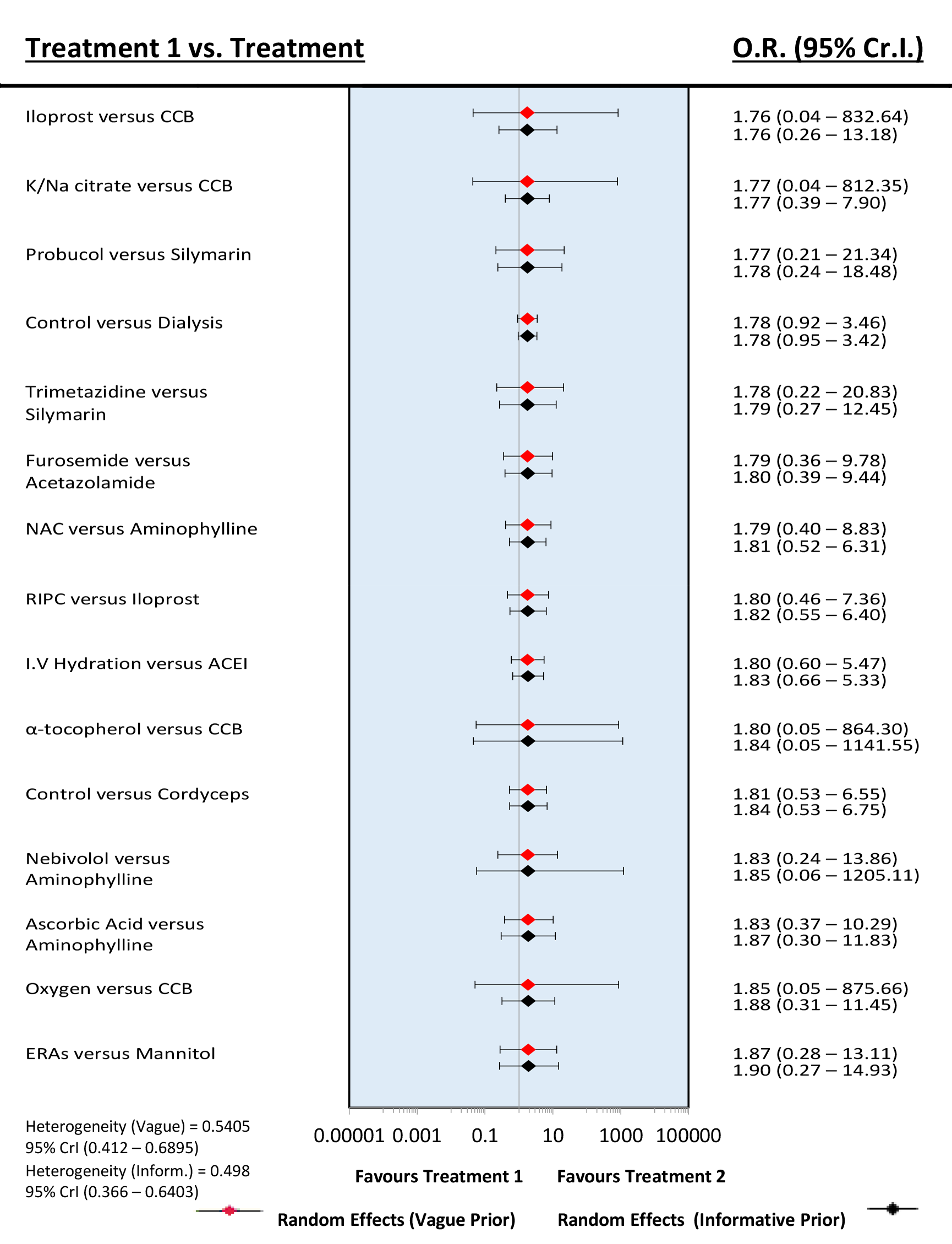


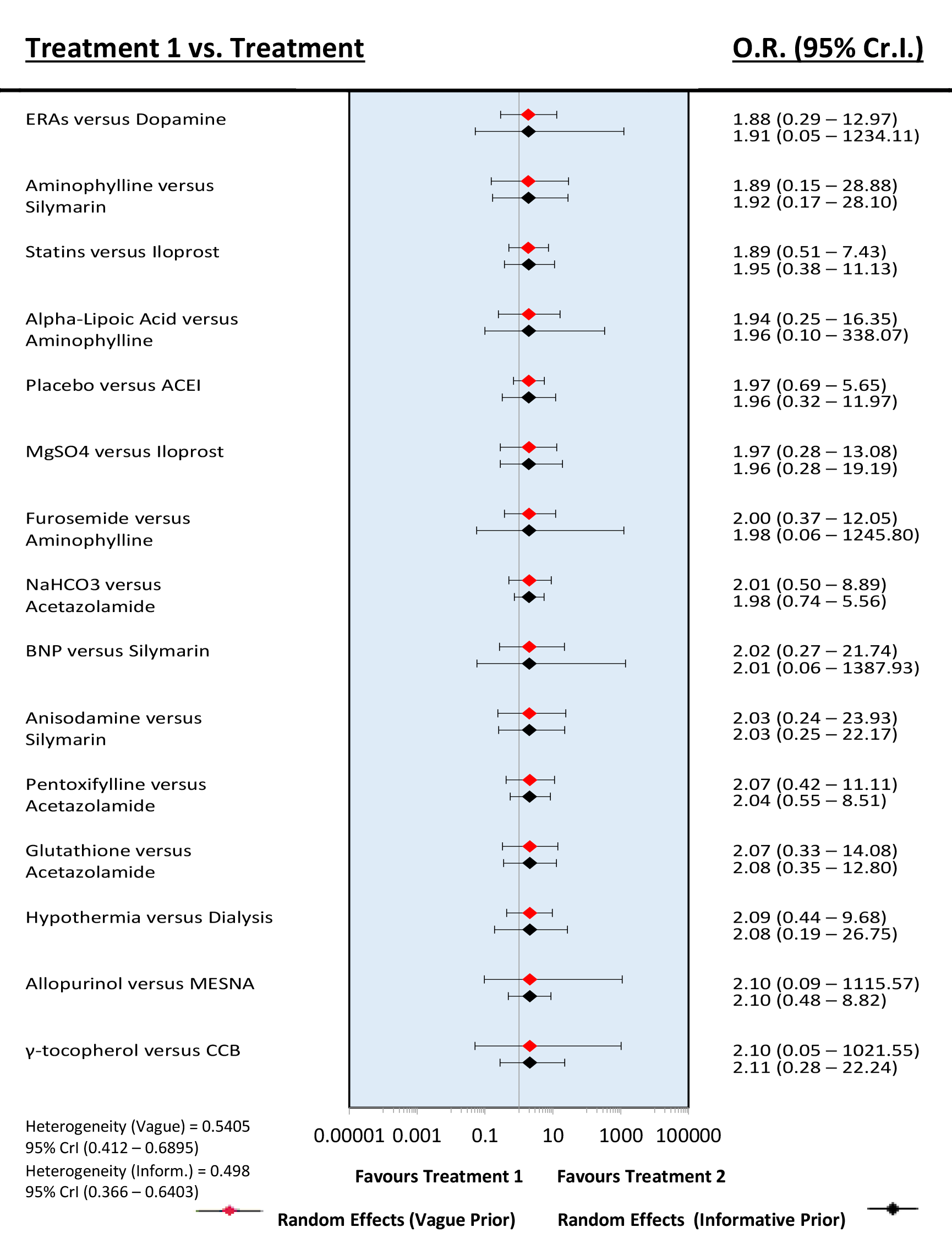


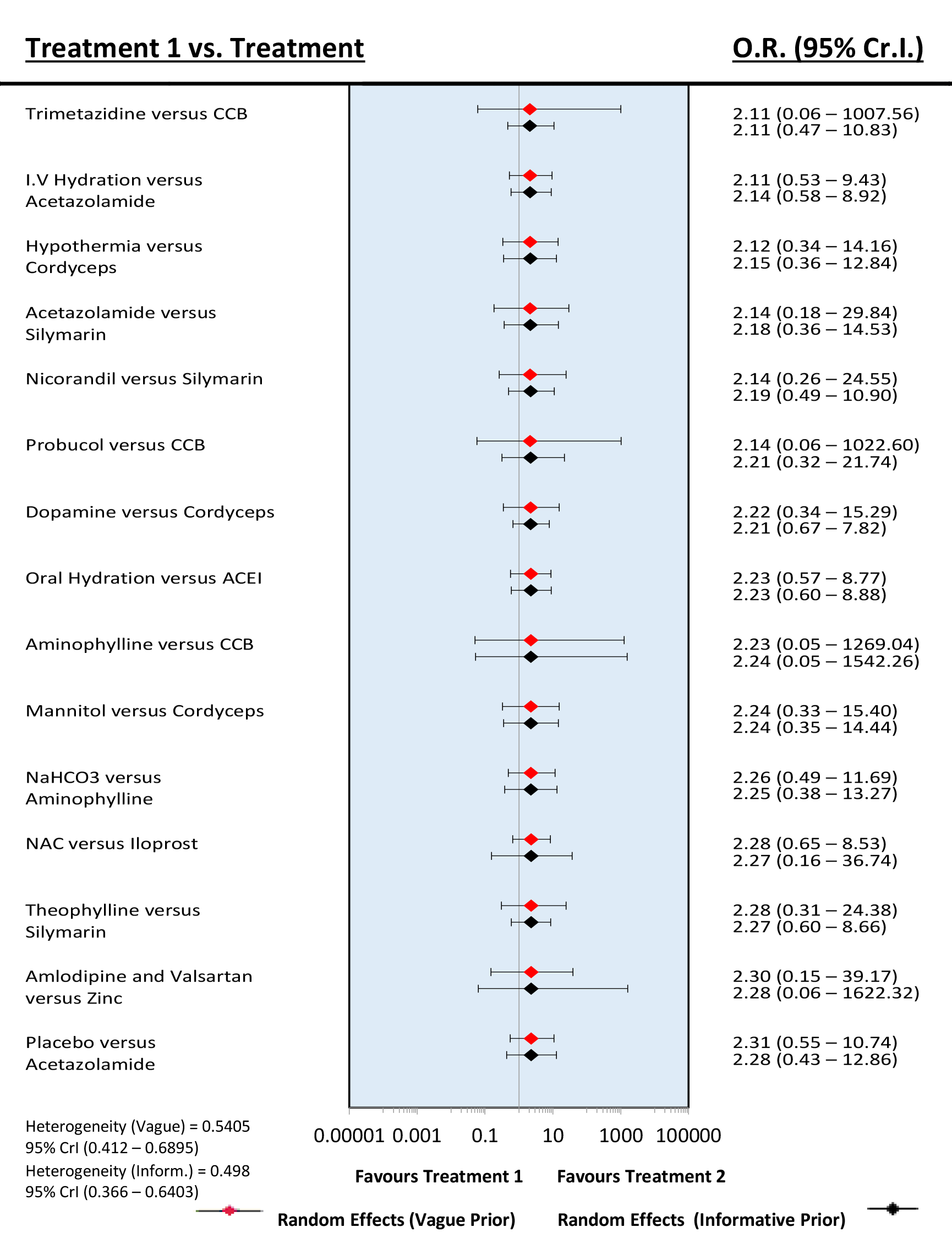


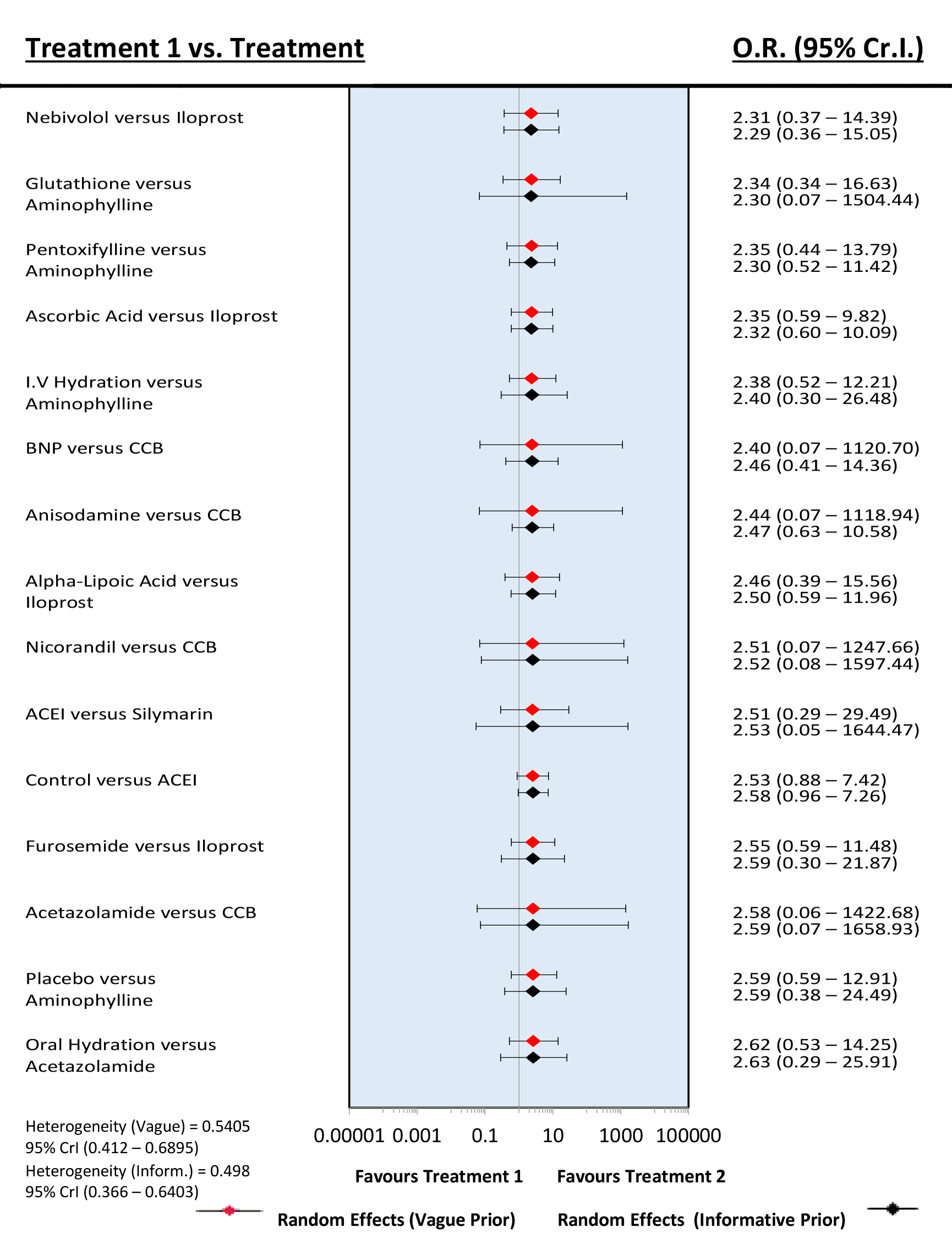


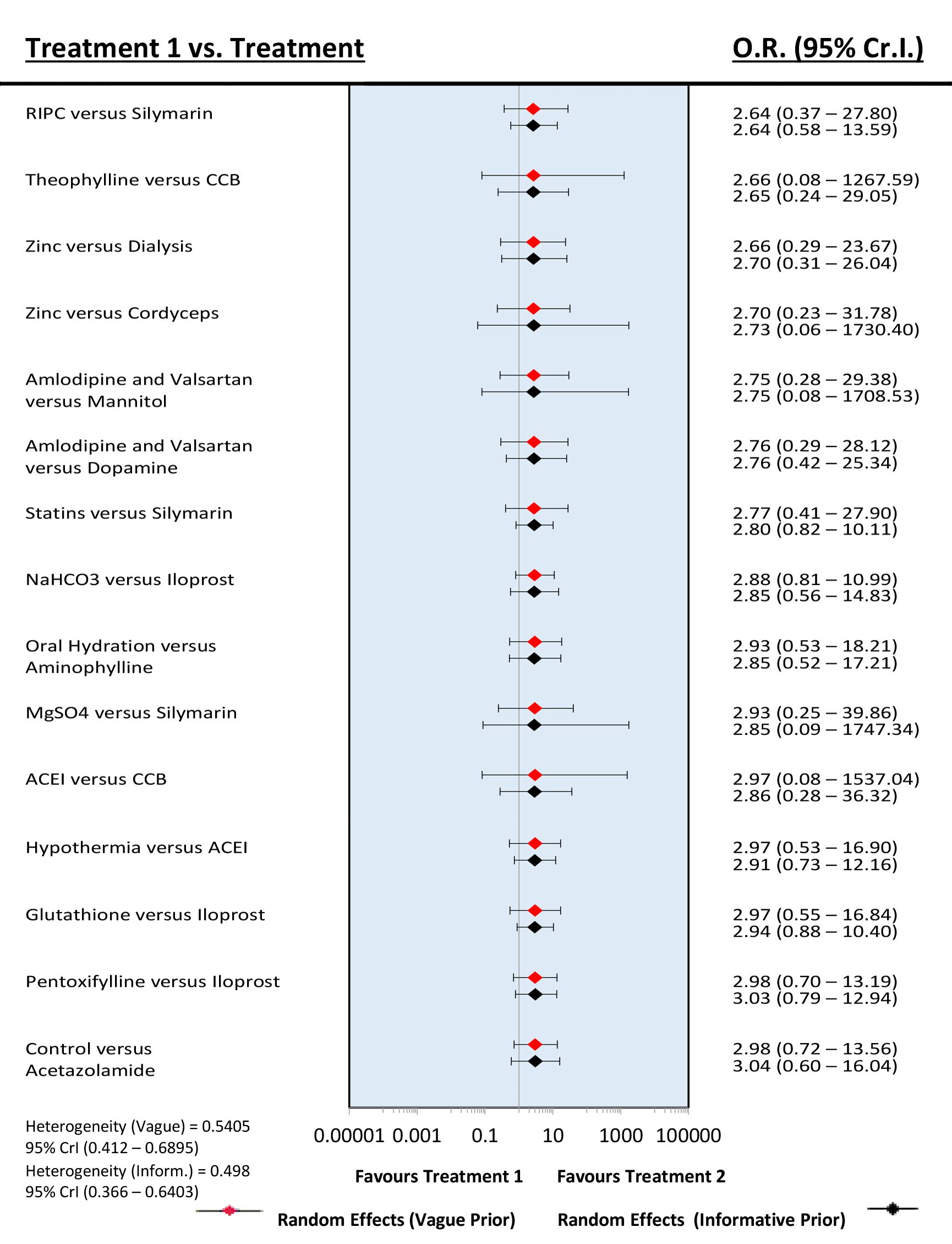


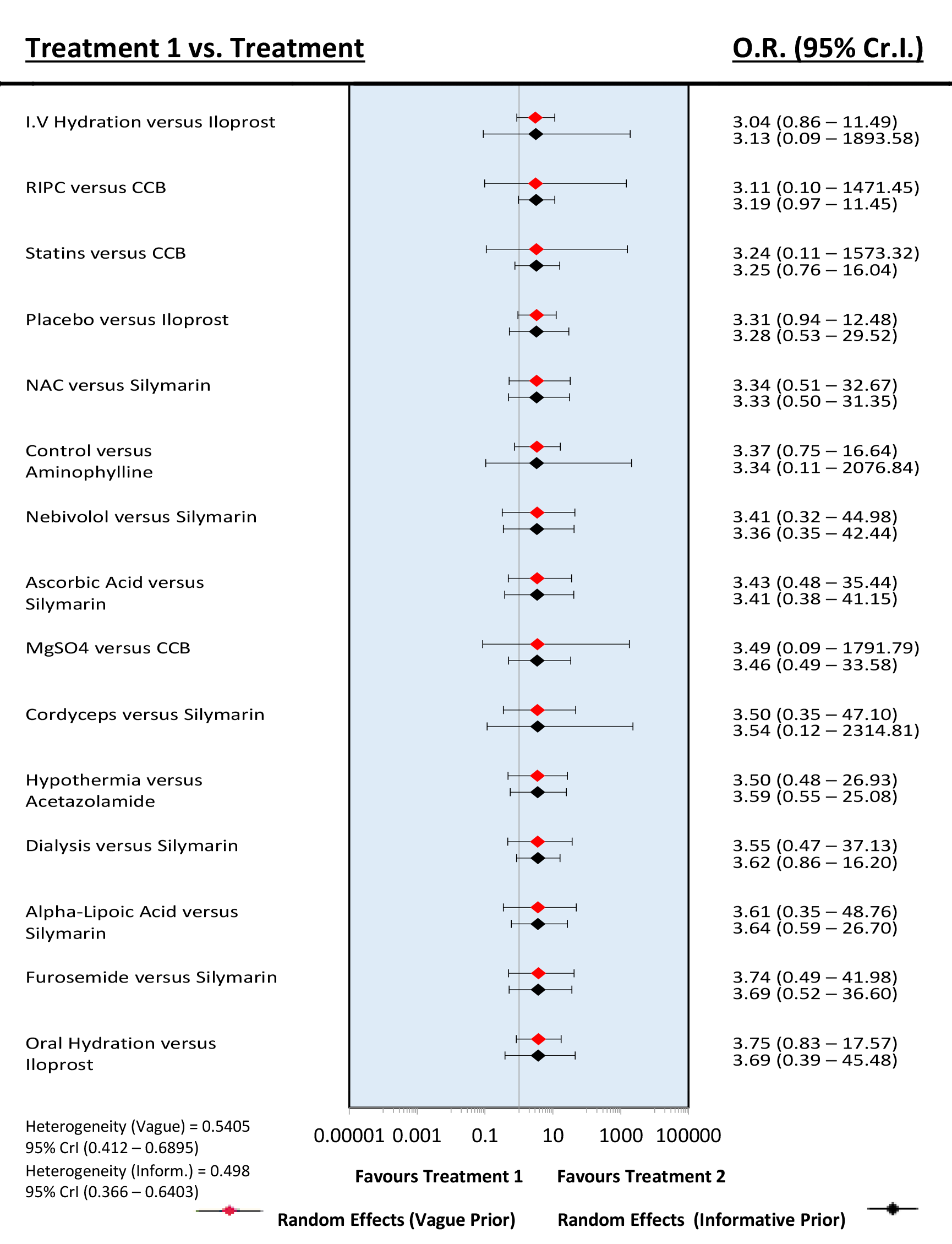


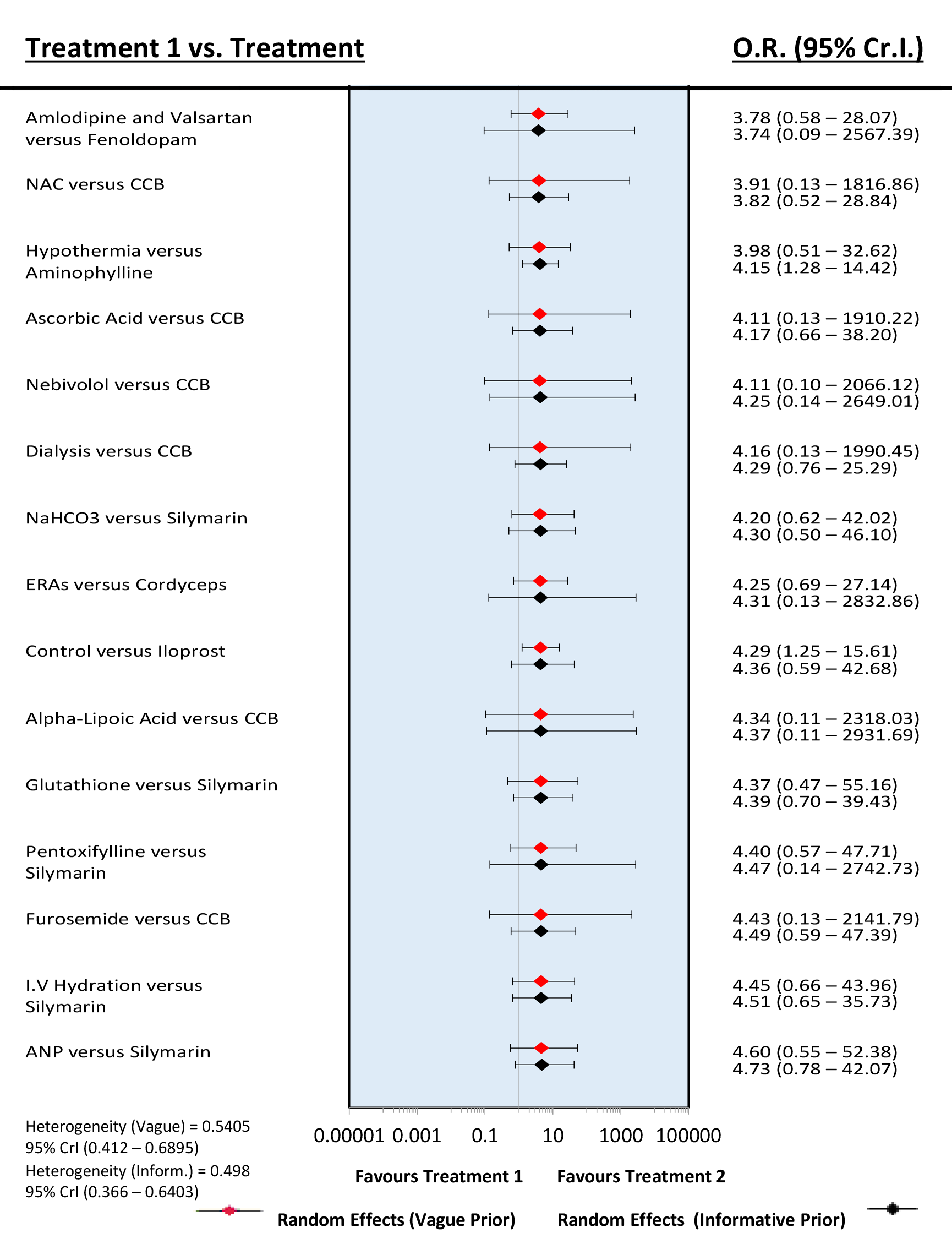


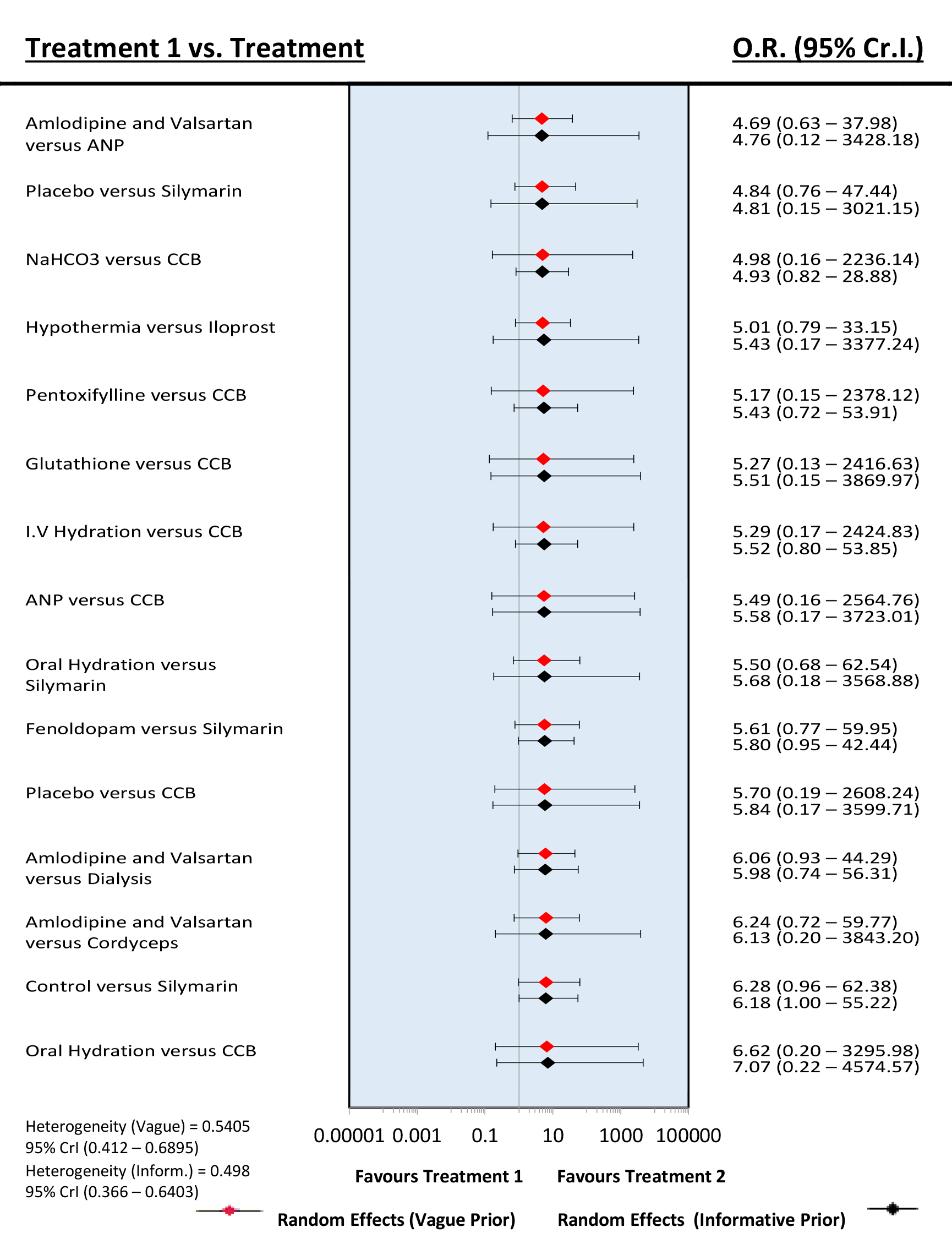


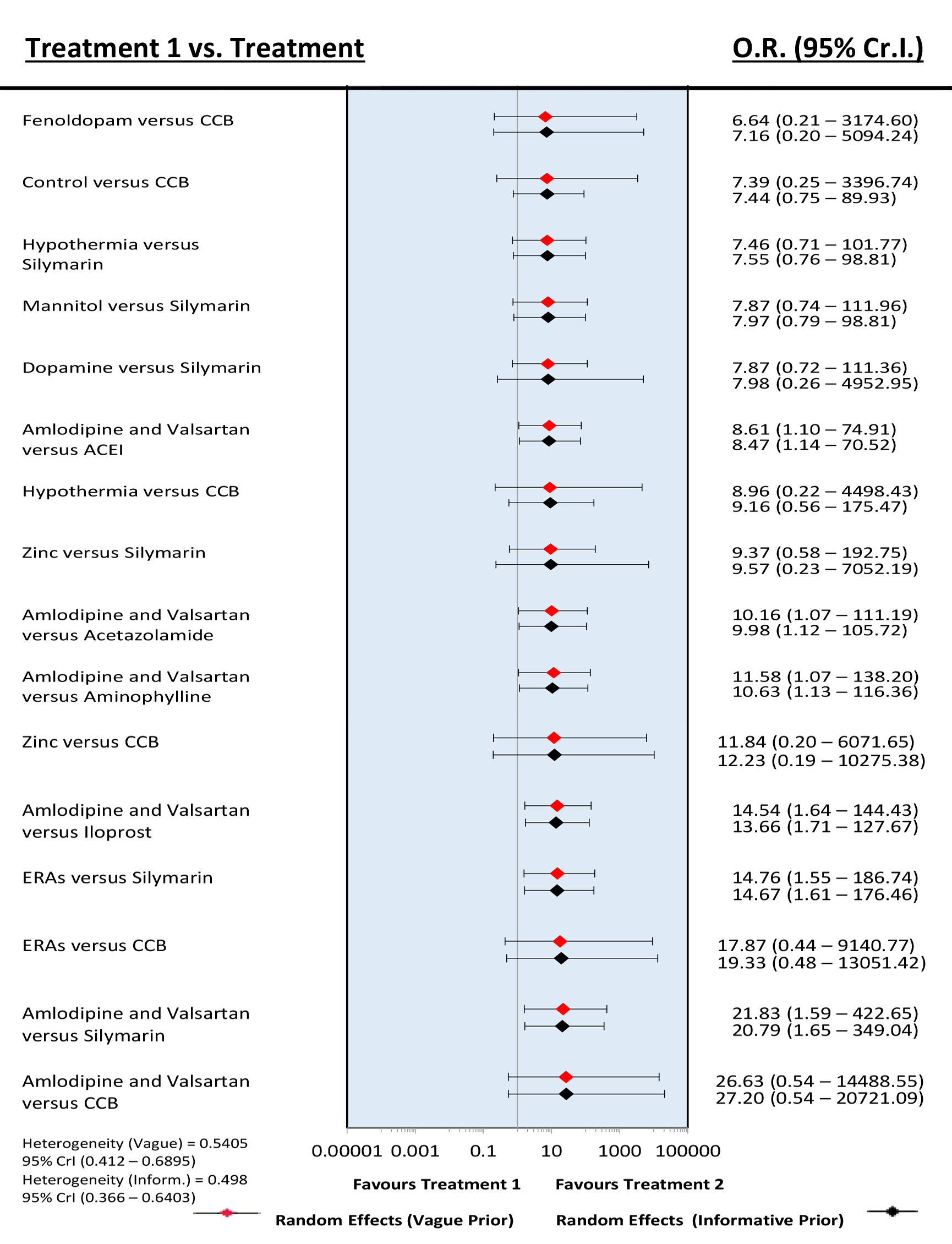


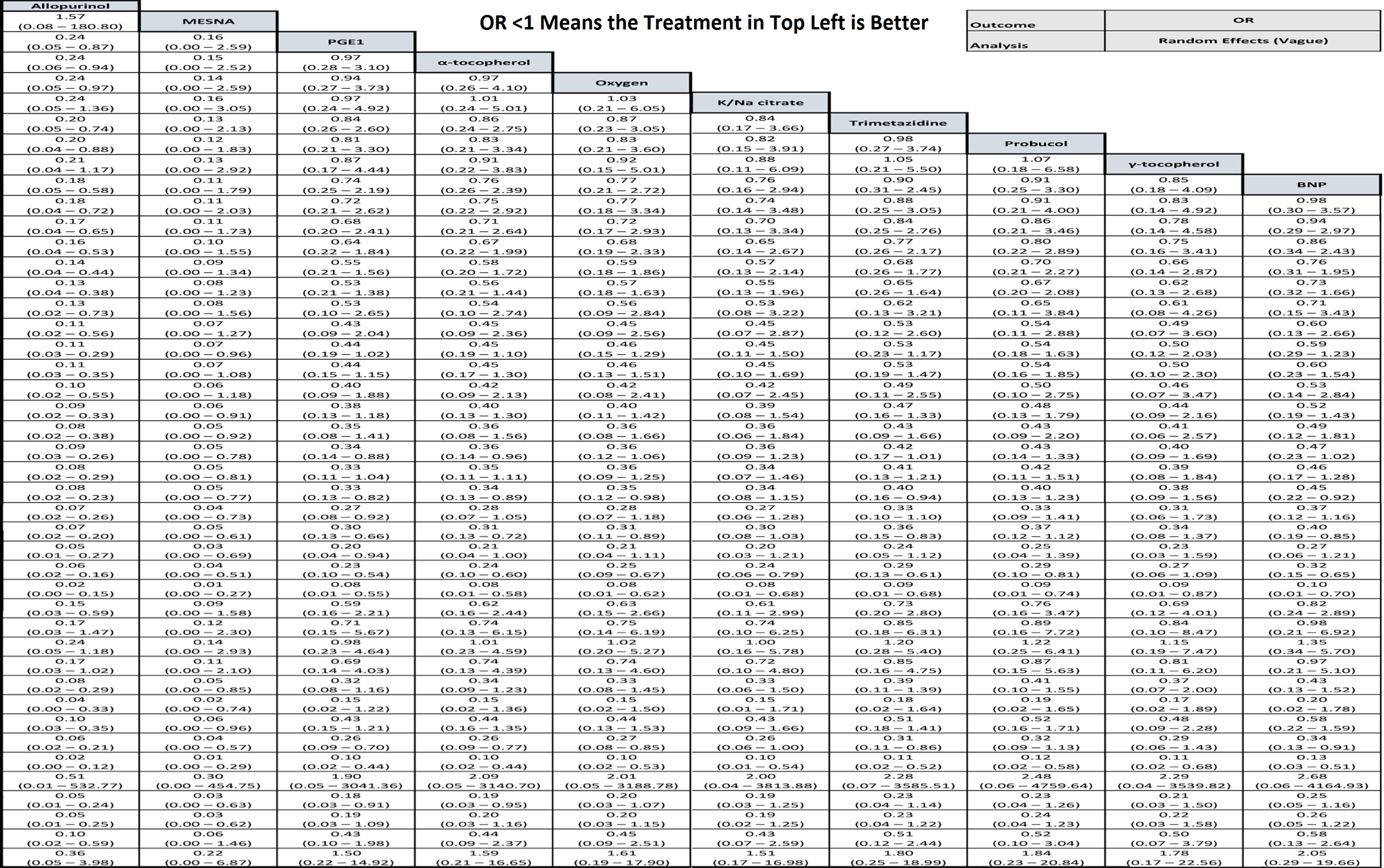


**Figure2 League Table**


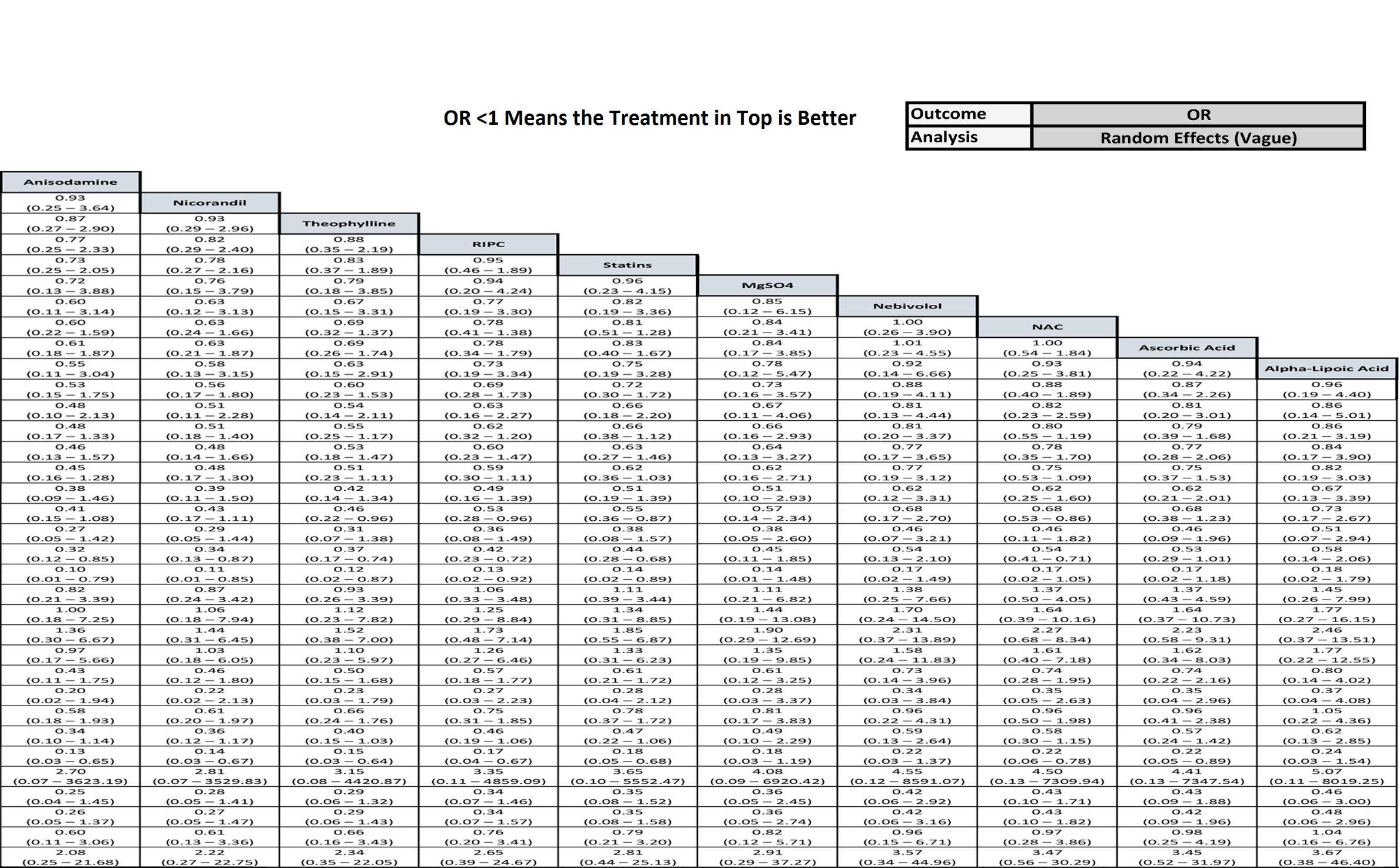


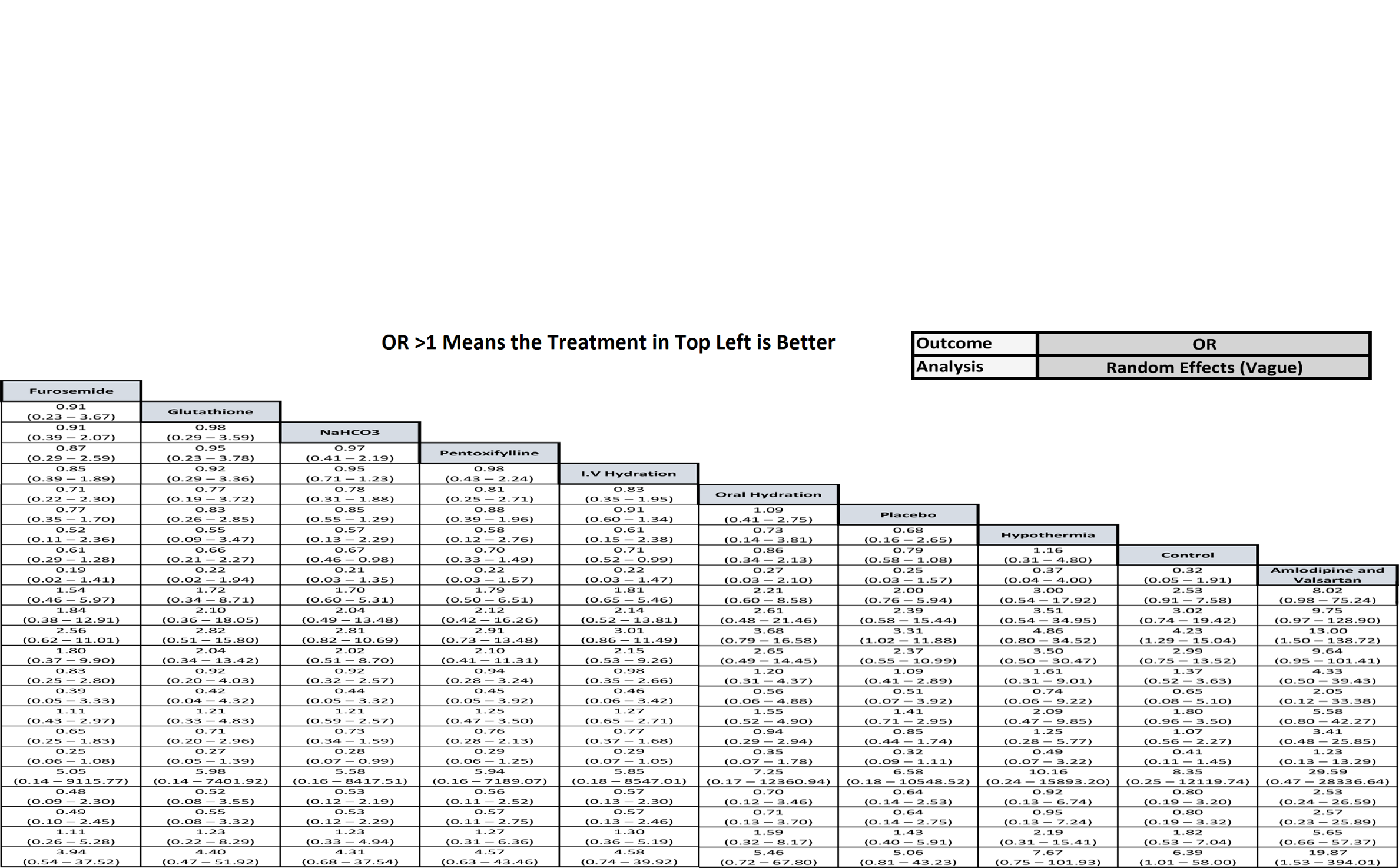


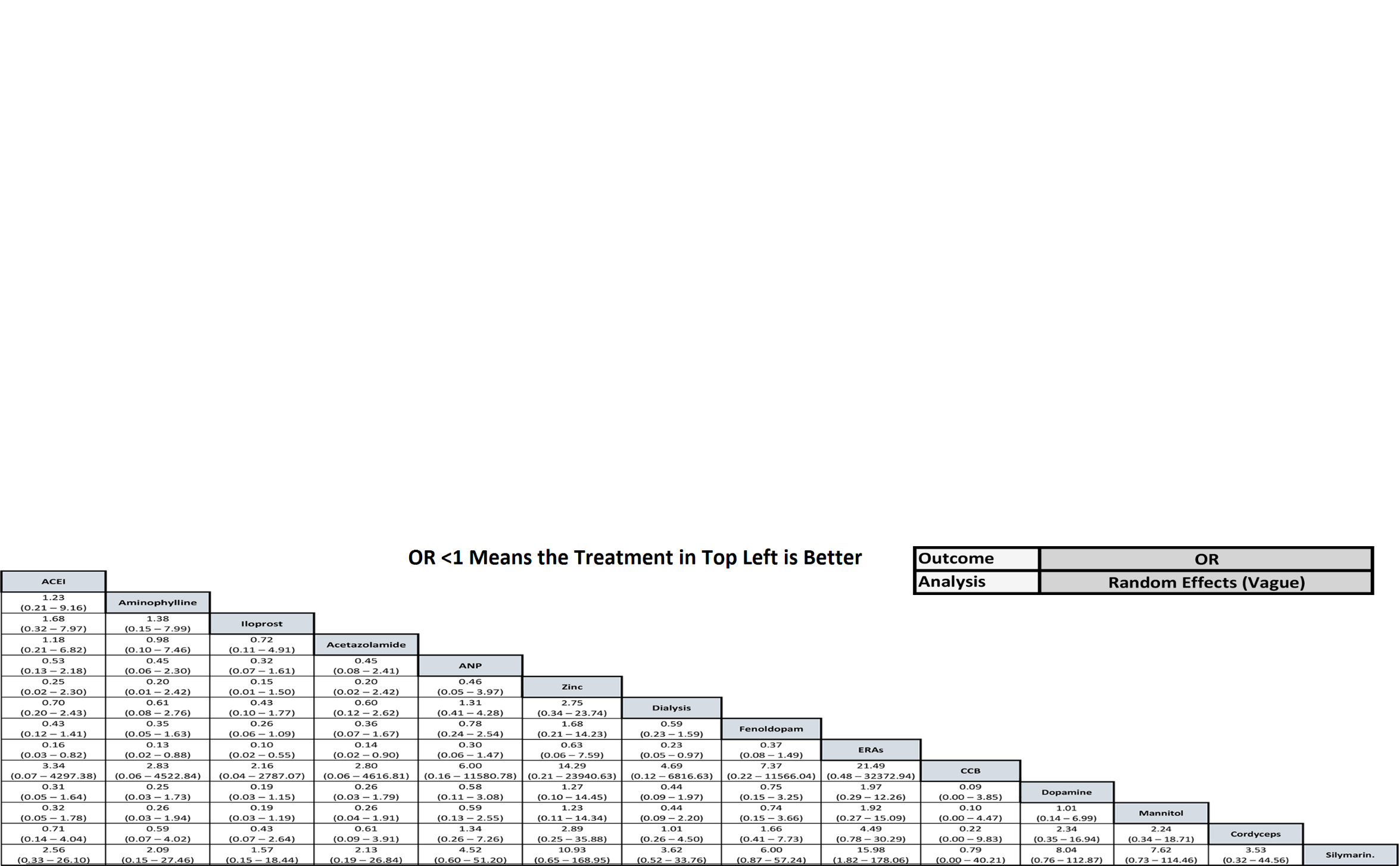


**Sub-Analysis after excluding studies with zero values**

Total Number of studies: 184 RCTs (

Excluded studies with zero events (total 16)

| Study | Interventions included |
| --- | --- |
| Bilasy, 2012 | Placebo, Theophylline |
| Efrati, 2003 | Placebo, NAC |
| Erol, 2013 | Control, Allopurinol |
| Khoury, 1995 | Control, CCB |
| Kinbara, 2010 | Control, NAC, Aminophylline |
| Kitzler, 2012 | Placebo, NAC, alpha tocopherol |
| Kotlyar, 2005 | Placebo, NAC |
| Kumar, 2014 (2 arms per contrast media) | NAC, Allopurinol, I.V Hydration |
| Ludwig, 2011 | Control, MESNA |
| Matejka, 2010 | Control, Theophylline |
| Moore, 2006 | Control, NAC |
| Saitoh, 2011 | Control, NAC, Glutathione |
| Sandhu, 2006 | Control, NAC |
| Sar, 2010 | Placebo, NAC |
| Spargias, 2006 | Placebo, Iloprost |

Figures and Tables:

1. Network Diagram

2. Tables:

A. Network Characteristics

B. Interventions Characteristics

C Direct comparisons characteristics

3. Rankogram

4. Ranking and probability of being the best (table)

5. Forest Plot

6. R Graphical diagnostics (Trace & Density)

| Software | Spec | Convergence | Analysis |
| --- | --- | --- | --- |
| Netmetaxl / WinBUGS14 version 1.4.3 | Burn 5000  Sim 10000 | good convergence (FE MC error 5% of the SD) | Random Effects (Vague)  Random Effects (Informative) |
| GeMTC R | Burn 5000  Sim 20000 | good convergence | Random Effects (Vague) |


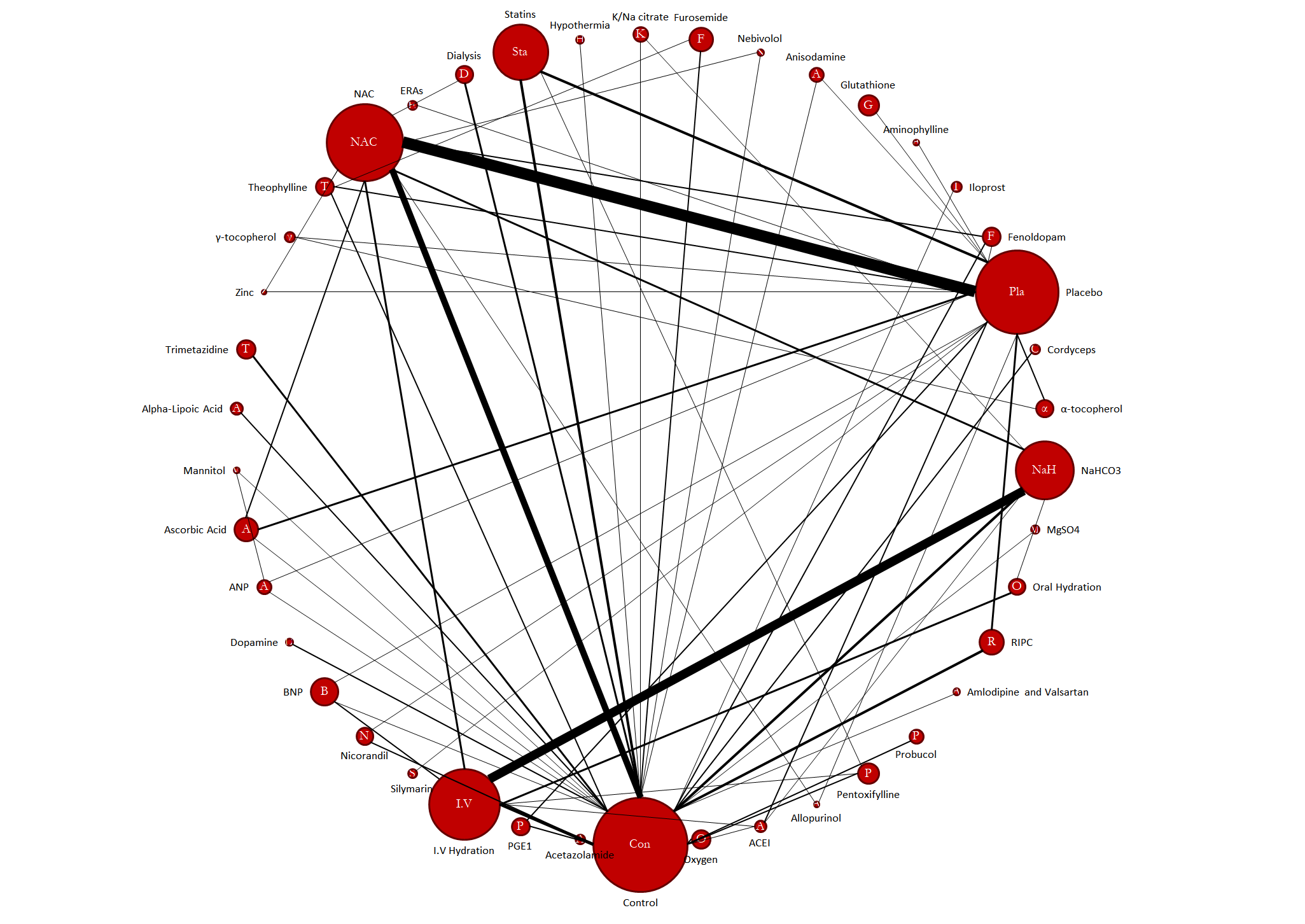


Figure 1Network Diagram

Table 1 Network Characteristics

| **Characteristic** | **Number** |
| --- | --- |
| **Number of Interventions** | 42 |
| **Number of Studies** | 184 |
| **Total Number of Patients in Network** | 41,187 |
| **Total Number of Events in Network** | 4,509 |
| **Total Possible Pairwise Comparisons** | 861 |
| **Total Number Pairwise Comparisons With Direct Data** | 71 |
| **Number of Two-arm Studies** | 168 |
| **Number of Multi-Arms Studies** | 16 |
| **Number of Studies With No Zero Events** | 184 |
| **Number of Studies With At Least One Zero Event** | 0 |
| **Number of Studies with All Zero Events** | 0 |

Table 2 Interventions Characteristics

| **Treatment** | **# Studies** | **# Events** | **# Patients** | **Aggregate Rate** |
| --- | --- | --- | --- | --- |
| **I.V Hydration** | 39 | 508 | 5046 | 0.1007 |
| **Statins** | 14 | 123 | 3040 | 0.0405 |
| **Furosemide** | 3 | 50 | 554 | 0.0903 |
| **NAC** | 58 | 642 | 5874 | 0.1093 |
| **Trimetazidine** | 4 | 17 | 352 | 0.0483 |
| **NaHCO3** | 32 | 366 | 3393 | 0.1079 |
| **PGE1** | 4 | 24 | 304 | 0.0789 |
| **MgSO4** | 1 | 9 | 62 | 0.1452 |
| **Pentoxifylline** | 4 | 30 | 438 | 0.0685 |
| **Placebo** | 64 | 924 | 6925 | 0.1334 |
| **Control** | 80 | 1218 | 8839 | 0.1378 |
| **Allopurinol** | 1 | 5 | 30 | 0.1667 |
| **BNP** | 4 | 52 | 744 | 0.0699 |
| **Probucol** | 2 | 12 | 198 | 0.0606 |
| **α-tocopherol** | 3 | 18 | 302 | 0.0596 |
| **γ-tocopherol** | 1 | 6 | 102 | 0.0588 |
| **Oxygen** | 2 | 33 | 346 | 0.0954 |
| **Amlodipine and Valsartan** | 1 | 8 | 45 | 0.1778 |
| **K/Na citrate** | 2 | 6 | 203 | 0.0296 |
| **Nicorandil** | 3 | 15 | 291 | 0.0515 |
| **Ascorbic Acid** | 7 | 52 | 552 | 0.0942 |
| **Alpha-Lipoic Acid** | 2 | 6 | 139 | 0.0432 |
| **Oral Hydration** | 5 | 23 | 254 | 0.0906 |
| **Nebivolol** | 1 | 8 | 40 | 0.2000 |
| **Anisodamine** | 2 | 17 | 192 | 0.0885 |
| **RIPC** | 10 | 38 | 608 | 0.0625 |
| **Theophylline** | 5 | 18 | 323 | 0.0557 |
| **Hypothermia** | 1 | 14 | 58 | 0.2414 |
| **Glutathione** | 1 | 21 | 414 | 0.0507 |
| **ACEI** | 3 | 10 | 129 | 0.0775 |
| **Aminophylline** | 1 | 4 | 30 | 0.1333 |
| **Iloprost** | 1 | 8 | 103 | 0.0777 |
| **Acetazolamide** | 1 | 5 | 94 | 0.0532 |
| **ANP** | 3 | 24 | 202 | 0.1188 |
| **Zinc** | 1 | 3 | 18 | 0.1667 |
| **Dialysis** | 5 | 43 | 293 | 0.1468 |
| **Fenoldopam** | 5 | 78 | 333 | 0.2342 |
| **ERAs** | 1 | 43 | 77 | 0.5584 |
| **Dopamine** | 2 | 9 | 48 | 0.1875 |
| **Mannitol** | 2 | 10 | 35 | 0.2857 |
| **Cordyceps** | 2 | 7 | 88 | 0.0795 |
| **Silymarin** | 1 | 2 | 69 | 0.0290 |

Table 3 Direct comparisons characteristics

| **Comparison** | **# Studies** | **# Patients** | **# Events** |
| --- | --- | --- | --- |
| **NAC vs. Placebo** | 32 | 8,048 | 940 |
| **Statins vs. Control** | 6 | 4,382 | 200 |
| **NaHCO3 vs. K/Na citrate** | 1 | 206 | 4 |
| **I.V Hydartion vs. NaHCO3** | 24 | 5,481 | 515 |
| **I.V Hydartion vs. Oral Hydration** | 5 | 509 | 43 |
| **NAC vs. Ascorbic Acid** | 3 | 583 | 88 |
| **Placebo vs. Ascorbic Acid** | 5 | 869 | 139 |
| **NAC vs. Control** | 17 | 2,304 | 355 |
| **NAC vs. Fenoldopam** | 3 | 359 | 44 |
| **Control vs. Fenoldopam** | 2 | 123 | 26 |
| **I.V Hydartion vs. Control** | 8 | 2,884 | 396 |
| **NAC vs. Theophylline** | 1 | 62 | 13 |
| **Statins vs. Placebo** | 7 | 1,508 | 125 |
| **NaHCO3 vs. Oral Hydration** | 1 | 43 | 3 |
| **Control vs. Alpha-Lipoic Acid** | 2 | 280 | 16 |
| **Furosemide vs. Control** | 3 | 1,089 | 131 |
| **Furosemide vs. Theophylline** | 1 | 159 | 18 |
| **Control vs. Theophylline** | 2 | 437 | 24 |
| **Control vs. RIPC** | 6 | 782 | 68 |
| **NAC vs. NaHCO3** | 5 | 805 | 211 |
| **Placebo vs. Theophylline** | 2 | 164 | 15 |
| **I.V Hydartion vs. NAC** | 4 | 536 | 60 |
| **Pentoxifylline vs. Control** | 2 | 461 | 46 |
| **Placebo vs. Nicorandil** | 1 | 240 | 29 |
| **MgSO4 vs. Control** | 1 | 126 | 26 |
| **Control vs. Dopamine** | 2 | 96 | 17 |
| **Control vs. Anisodamine** | 1 | 260 | 39 |
| **NAC vs. Nebivolol** | 1 | 80 | 17 |
| **Control vs. Nebivolol** | 1 | 80 | 19 |
| **Control vs. ACEI** | 1 | 71 | 12 |
| **Placebo vs. ACEI** | 2 | 202 | 19 |
| **Placebo vs. RIPC** | 4 | 427 | 43 |
| **NaHCO3 vs. Control** | 6 | 1,056 | 231 |
| **Control vs. Cordyceps** | 2 | 180 | 19 |
| **Statins vs. Pentoxifylline** | 1 | 220 | 9 |
| **NAC vs. Zinc** | 1 | 37 | 4 |
| **Placebo vs. Zinc** | 1 | 35 | 5 |
| **Control vs. Nicorandil** | 2 | 341 | 22 |
| **PGE1 vs. Placebo** | 2 | 392 | 74 |
| **ANP vs. Mannitol** | 1 | 20 | 8 |
| **Placebo vs. ANP** | 1 | 126 | 26 |
| **Control vs. Dialysis** | 5 | 588 | 112 |
| **Control vs. Probucol** | 2 | 409 | 50 |
| **PGE1 vs. Control** | 2 | 226 | 16 |
| **Control vs. BNP** | 1 | 209 | 23 |
| **I.V Hydartion vs. BNP** | 2 | 1,128 | 113 |
| **Trimetazidine vs. Control** | 4 | 714 | 71 |
| **Control vs. K/Na citrate** | 1 | 202 | 25 |
| **Control vs. Oxygen** | 2 | 697 | 115 |
| **Control vs. ANP** | 1 | 254 | 19 |
| **Control vs. Amlodipine and Valsartan** | 1 | 90 | 11 |
| **I.V Hydartion vs. Acetazolamide** | 1 | 190 | 21 |
| **NaHCO3 vs. Acetazolamide** | 1 | 190 | 9 |
| **NAC vs. Dialysis** | 1 | 275 | 11 |
| **Placebo vs. α-tocopherol** | 3 | 604 | 66 |
| **Placebo vs. Aminophylline** | 1 | 60 | 10 |
| **NAC vs. Allopurinol** | 1 | 65 | 12 |
| **Placebo vs. Allopurinol** | 1 | 60 | 16 |
| **Placebo vs. Silymarin** | 1 | 143 | 10 |
| **Control vs. Mannitol** | 1 | 53 | 10 |
| **Control vs. Iloprost** | 1 | 208 | 31 |
| **Placebo vs. Fenoldopam** | 1 | 283 | 90 |
| **Control vs. Hypothermia** | 1 | 128 | 29 |
| **Placebo vs. γ-tocopherol** | 1 | 203 | 21 |
| **α-tocopherol vs. γ-tocopherol** | 1 | 204 | 11 |
| **Placebo vs. ERAs** | 1 | 158 | 67 |
| **Placebo vs. Glutathione** | 1 | 825 | 41 |
| **Placebo vs. Anisodamine** | 1 | 126 | 17 |
| **I.V Hydartion vs. Pentoxifylline** | 1 | 199 | 12 |
| **Placebo vs. BNP** | 1 | 149 | 36 |
| **Control vs. Ascorbic Acid** | 1 | 156 | 10 |

Figure 2Rankogram: ranking the interventions for the probability of being the best, the interventions are colour coded; the first column represent the chance of being first best and 2nd column is the chance of being 2nd best and so on. The overall numerical value is presented in table 4


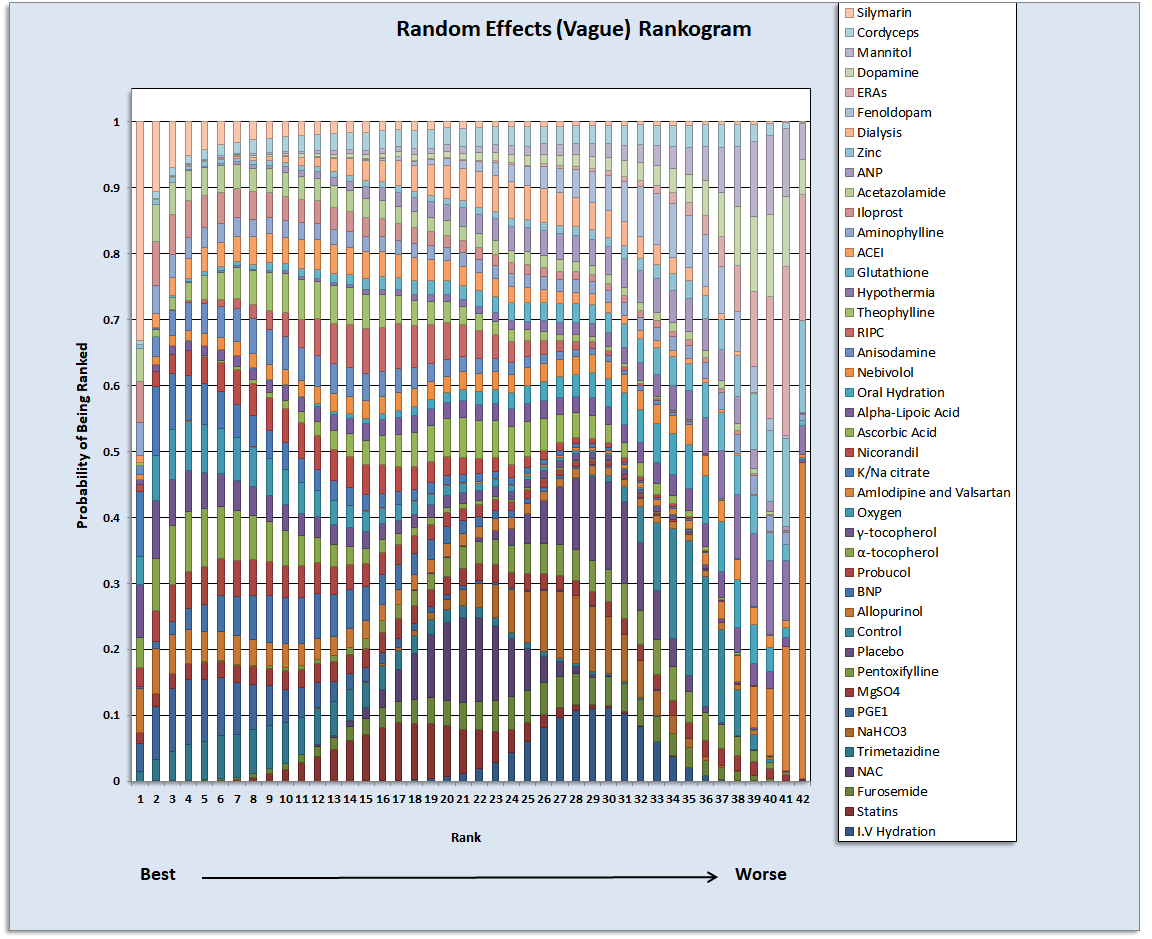


Table 4 Interventions ranking the treatments names column follow the league table (which arranges the presentation of summary estimates by ranking the treatments in order of most pronounced impact on the outcome under consideration) the numerical values represents the cumulative results of the probability of being best in which the highest score is 1 or 100% (see Rankogram)

| **Treatment** | **SUCRA** | **Treatment** | **SUCRA** |
| --- | --- | --- | --- |
| **PGE1** | 0.8382 | **NaHCO3** | 0.3564 |
| **α-tocopherol** | 0.8273 | **Pentoxifylline** | 0.352 |
| **K/Na citrate** | 0.7952 | **Glutathione** | 0.3362 |
| **Oxygen** | 0.7903 | **I.V Hydration** | 0.3257 |
| **Trimetazidine** | 0.7536 | **Placebo** | 0.3049 |
| **γ-tocopherol** | 0.748 | **Oral Hydration** | 0.2634 |
| **Probucol** | 0.7365 | **Hypothermia** | 0.2006 |
| **BNP** | 0.7265 | **Control** | 0.1682 |
| **Anisodamine** | 0.6984 | **Amlodipine and Valsartan** | 0.05304 |
| **Allopurinol** | 0.6974 | **Aminophylline** | 0.5531 |
| **Nicorandil** | 0.692 | **Iloprost** | 0.7117 |
| **Theophylline** | 0.6873 | **Acetazolamide** | 0.6628 |
| **ACEI** | 0.6406 | **ANP** | 0.3514 |
| **RIPC** | 0.6079 | **Zinc** | 0.2189 |
| **Statins** | 0.5806 | **Dialysis** | 0.4486 |
| **MgSO4** | 0.5285 | **Fenoldopam** | 0.2525 |
| **NAC** | 0.5054 | **ERAs** | 0.07113 |
| **Ascorbic Acid** | 0.4938 | **Dopamine** | 0.1919 |
| **Nebivolol** | 0.4745 | **Mannitol** | 0.1943 |
| **Alpha-Lipoic Acid** | 0.4437 | **Cordyceps** | 0.4611 |
| **Furosemide** | 0.4221 | **Silymarin** | 0.8345 |
| ***Analysis*** | **Random Effects (Vague)** | | |

Figure 3 Forest Plot


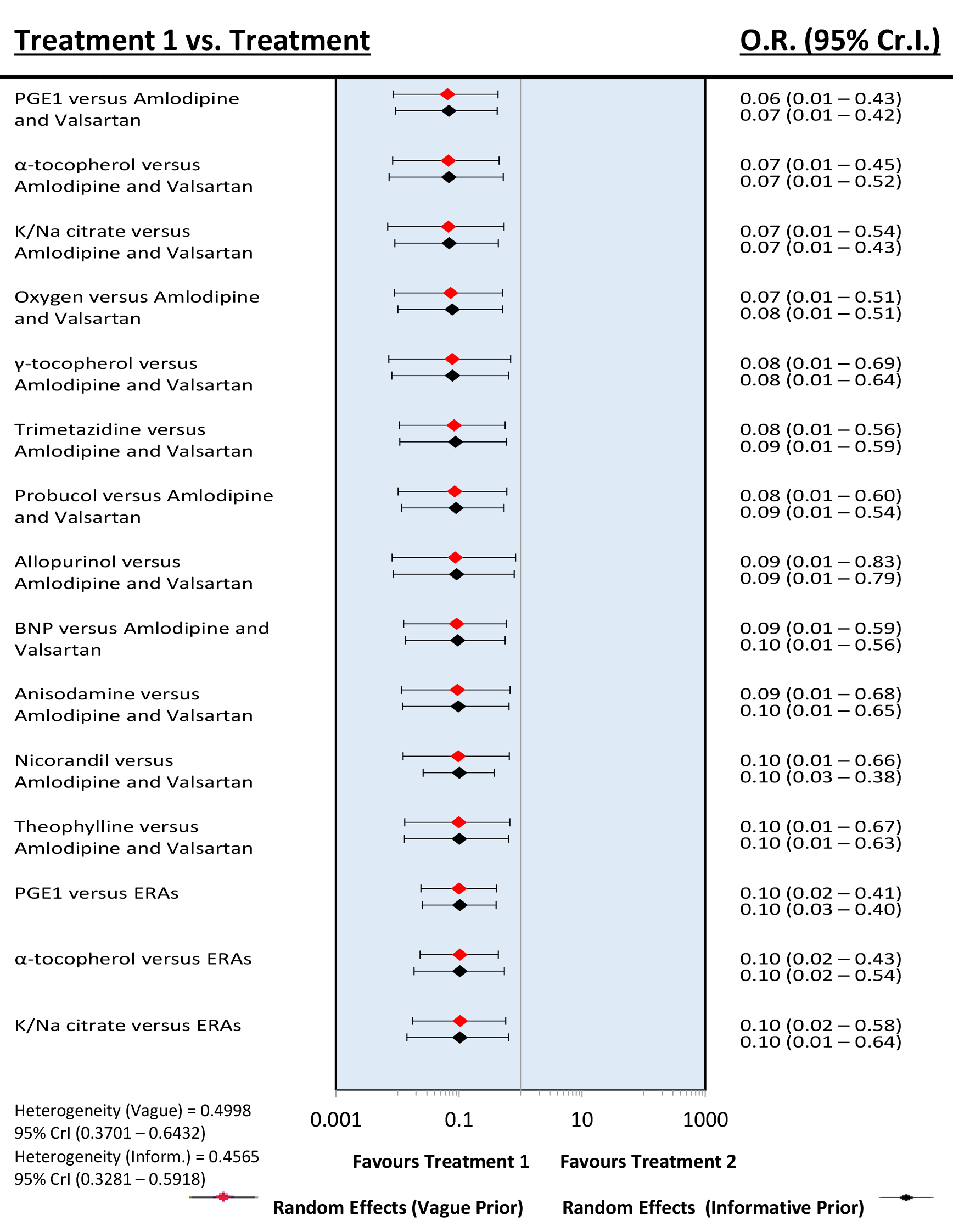


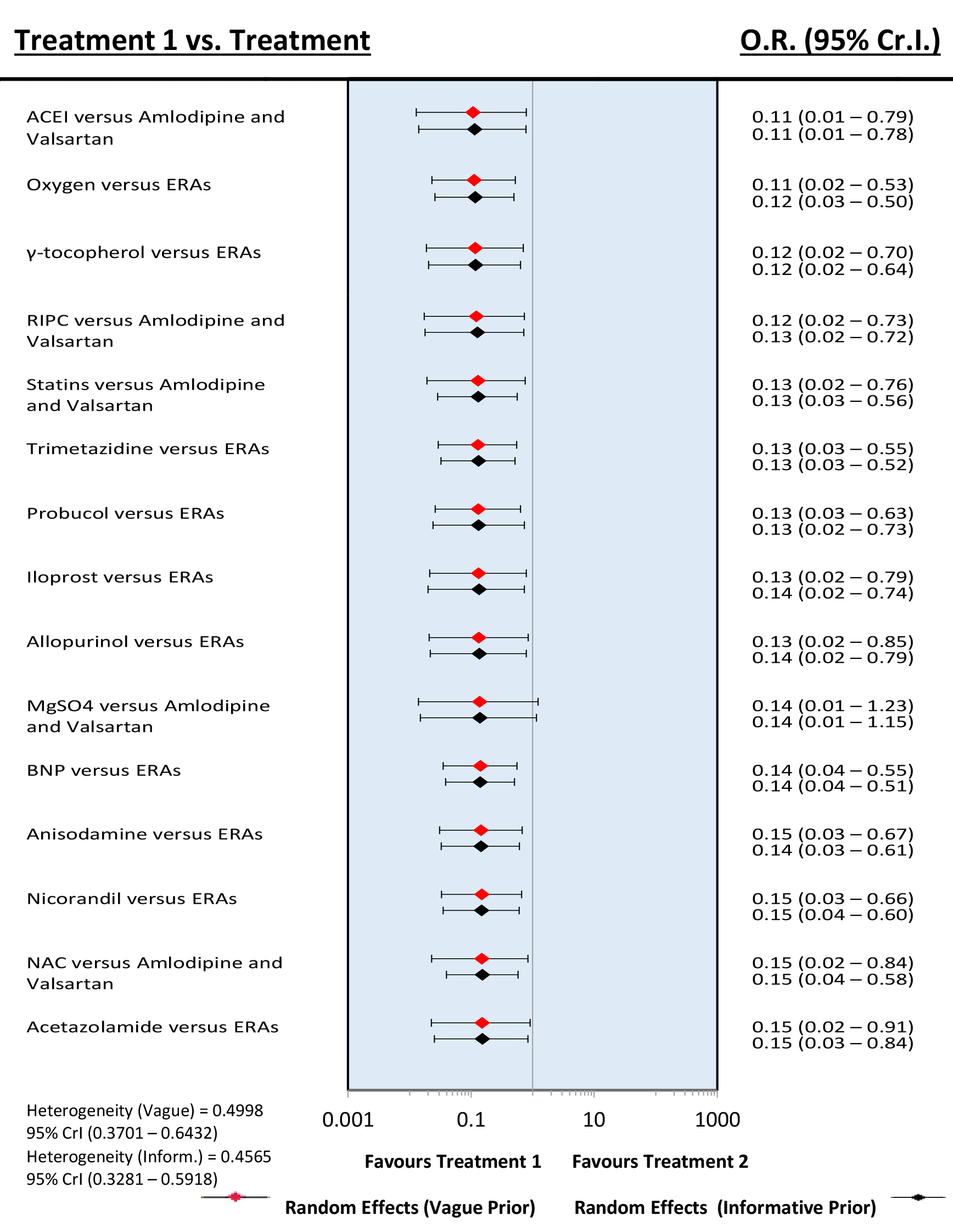


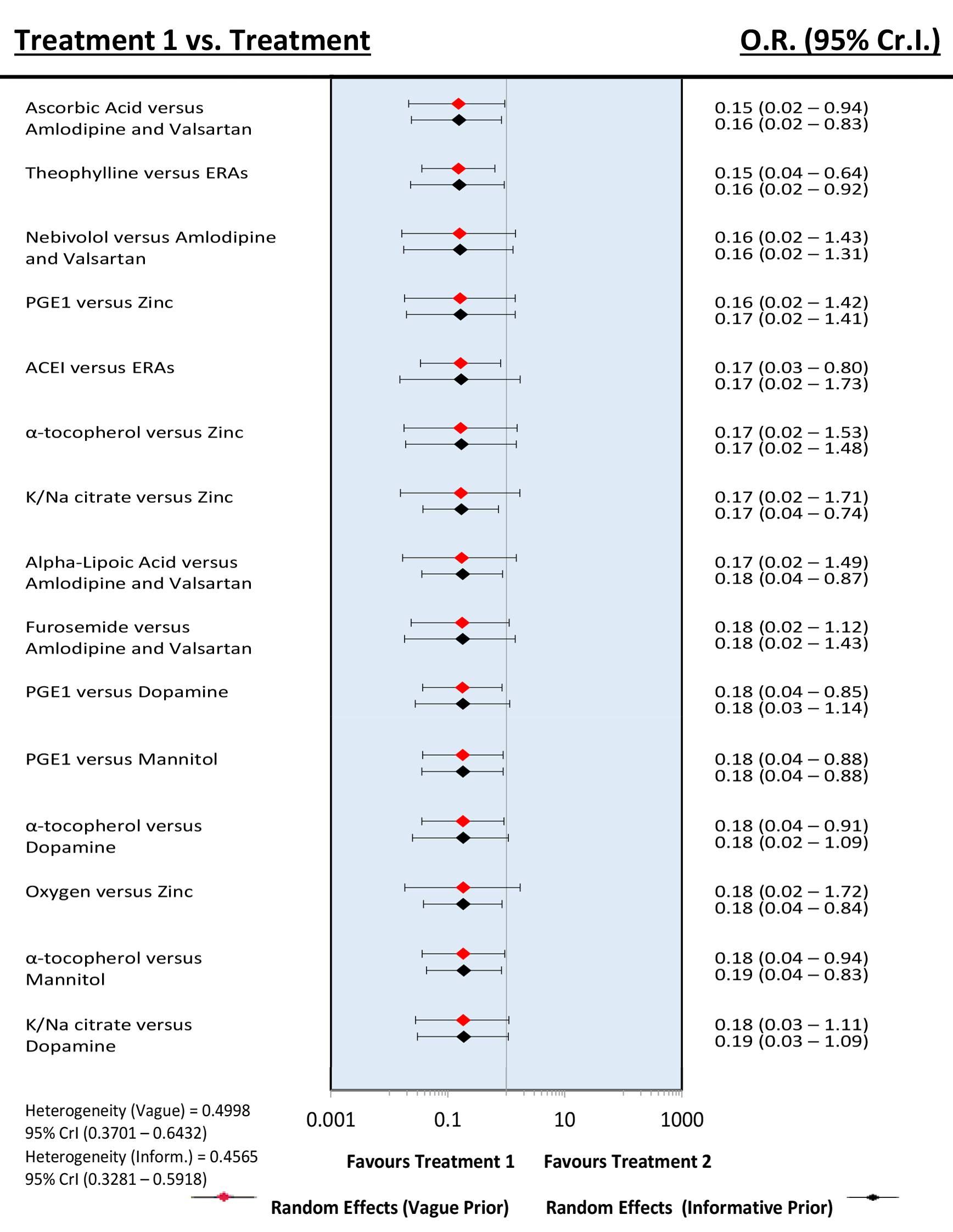


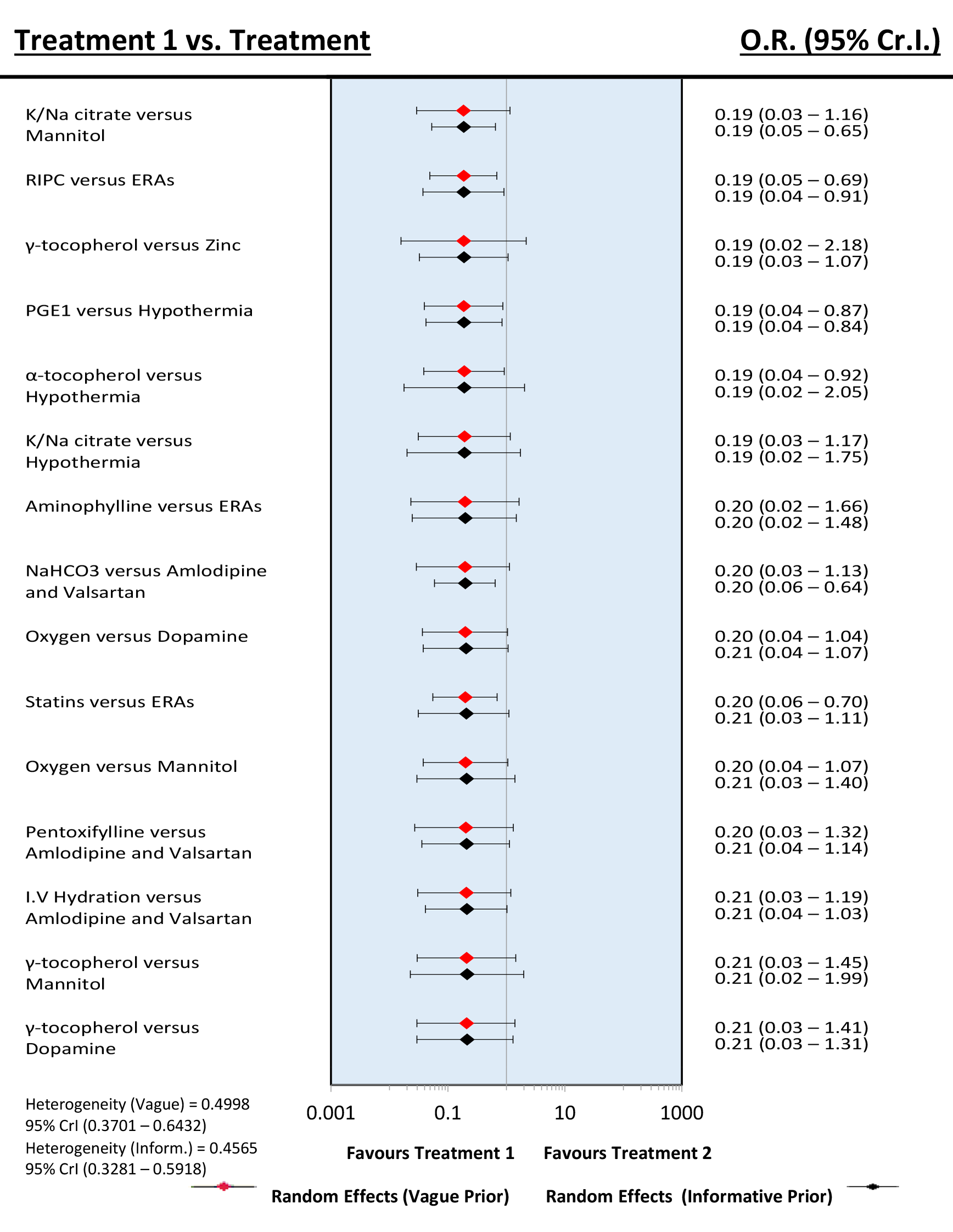


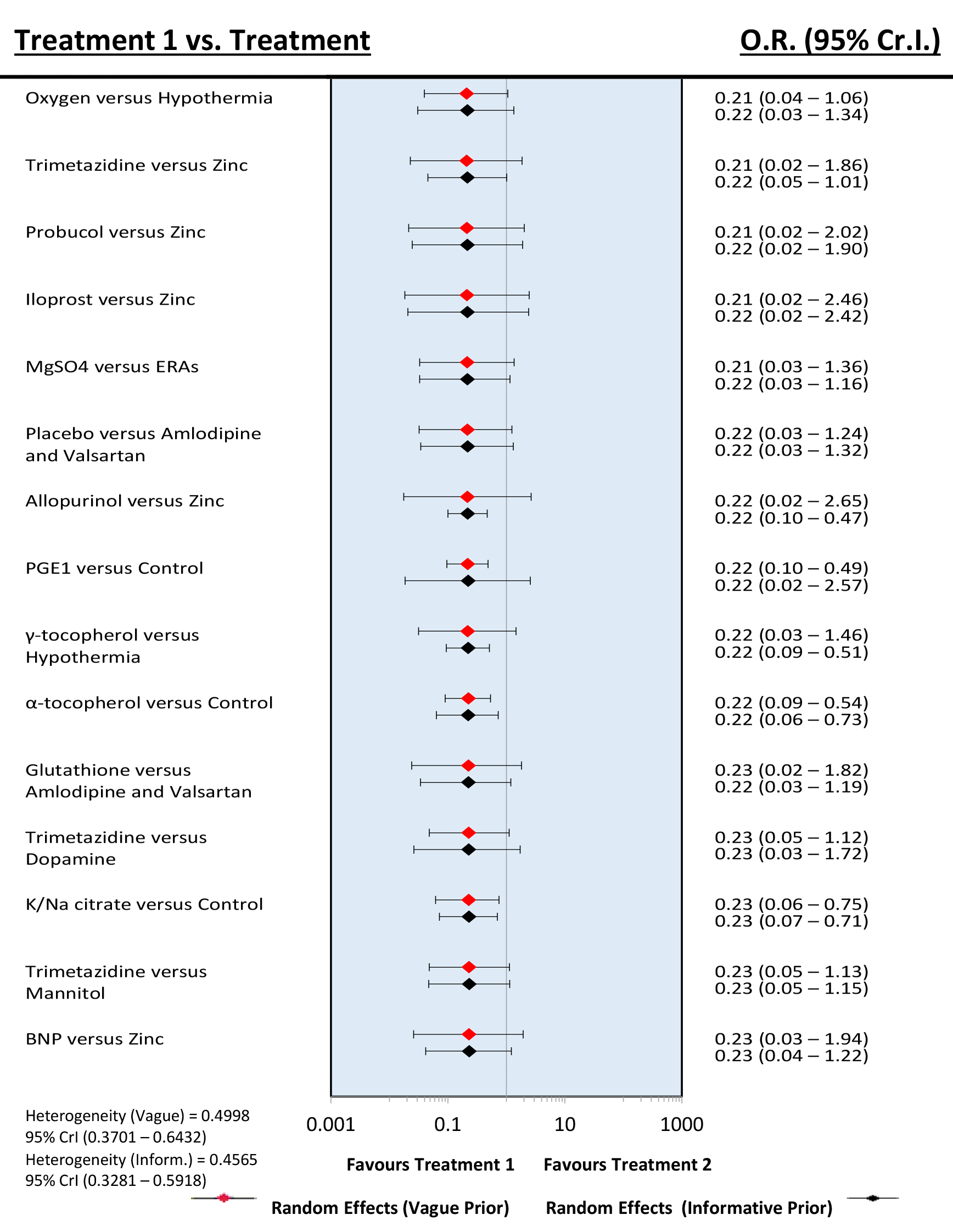


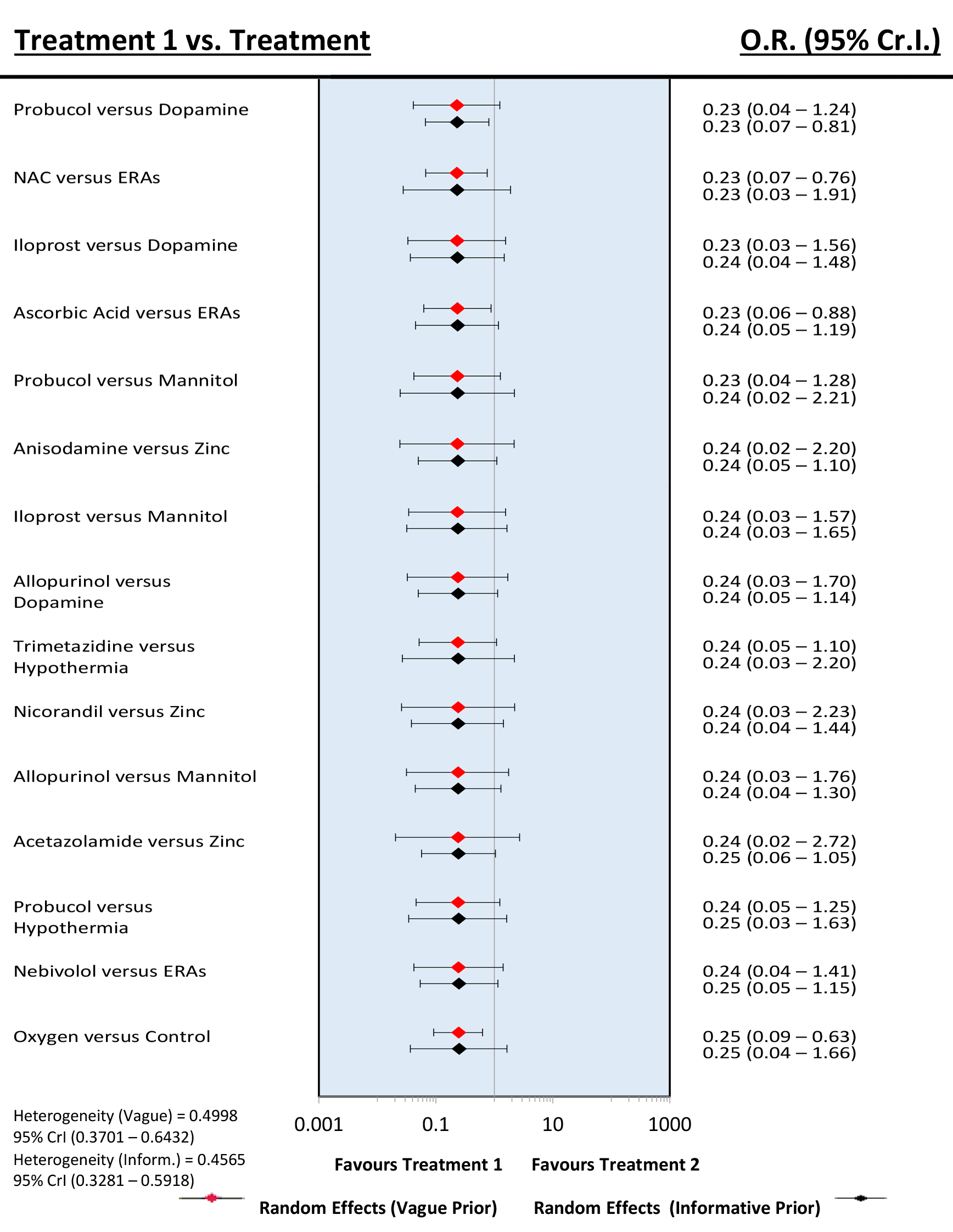


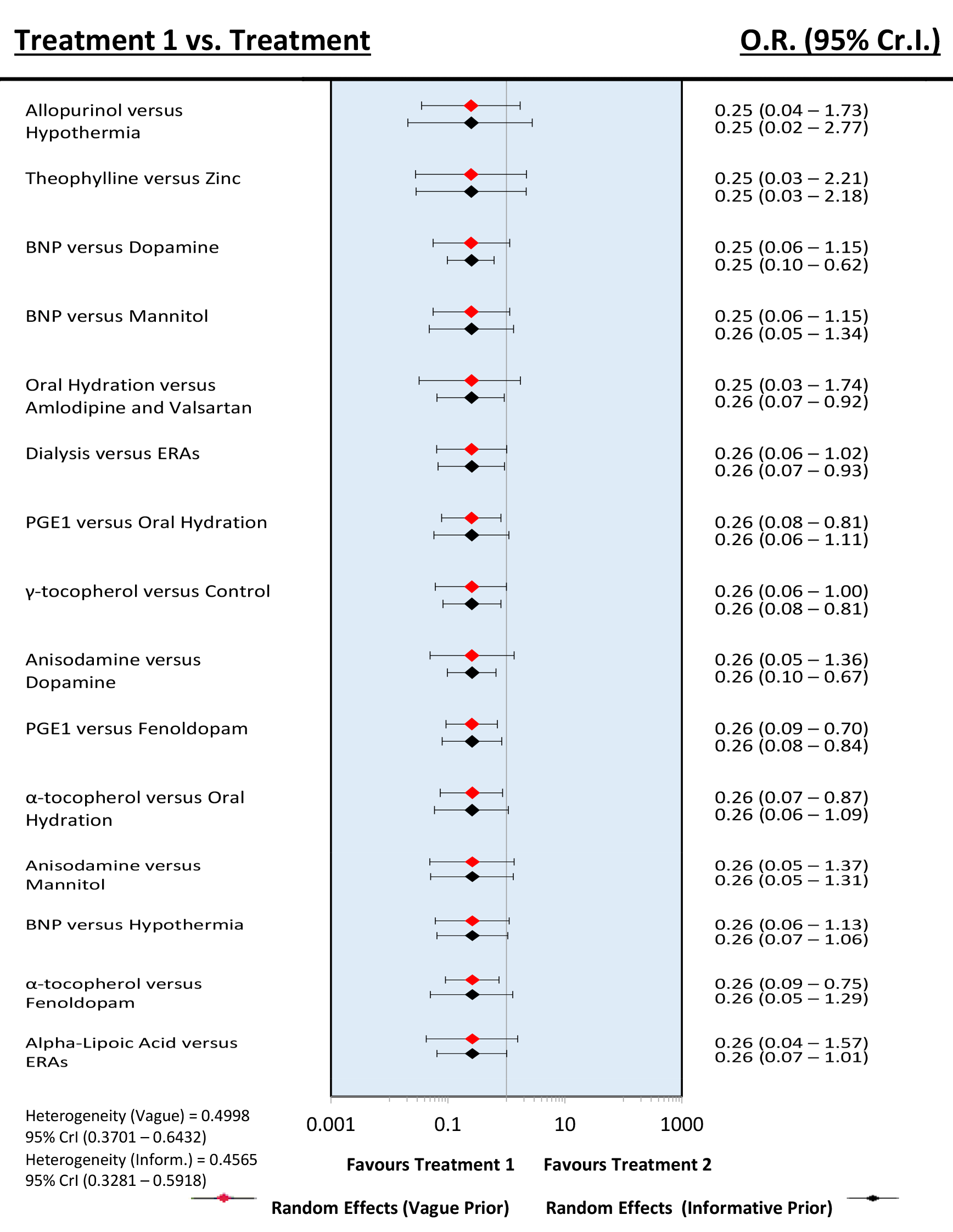


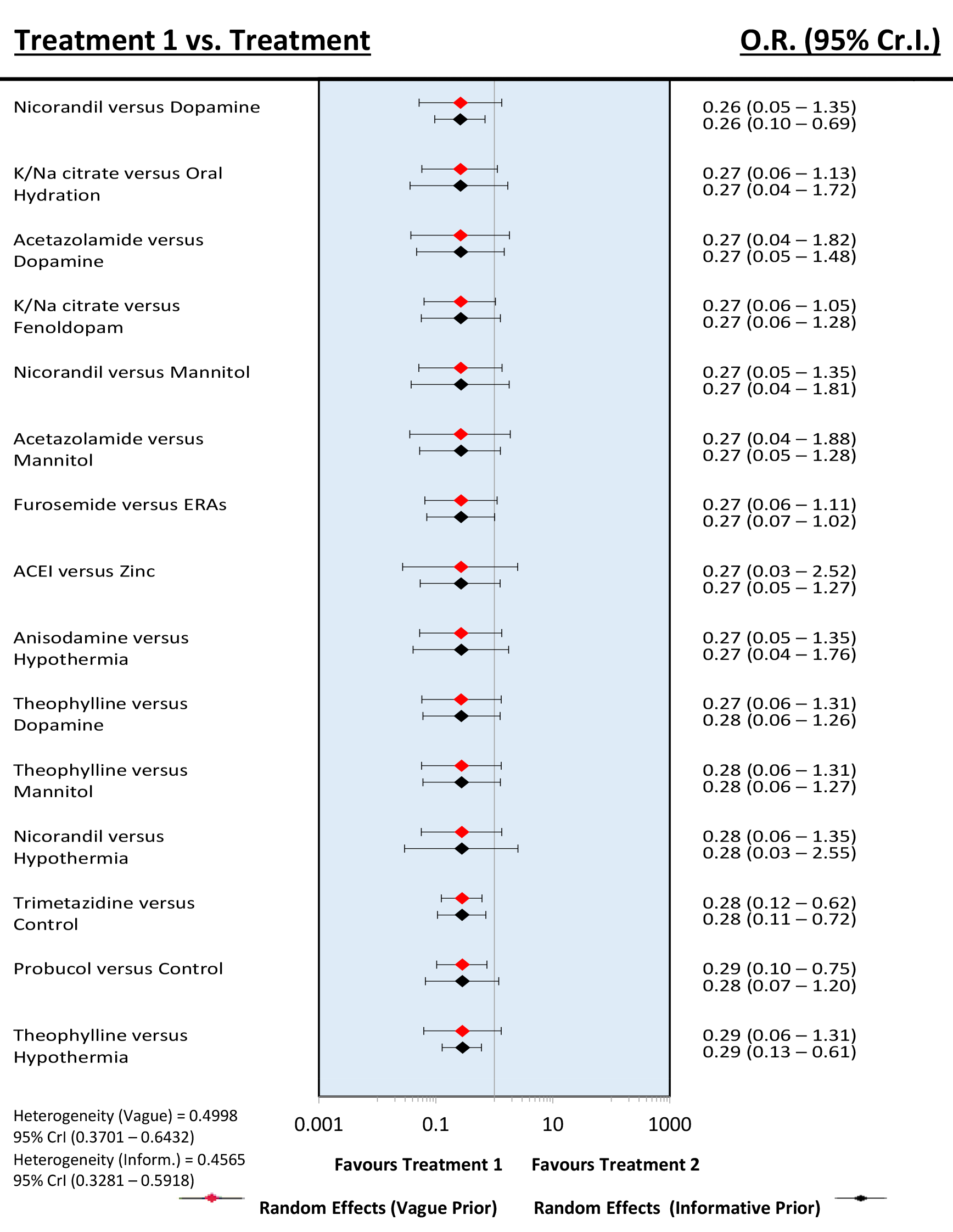


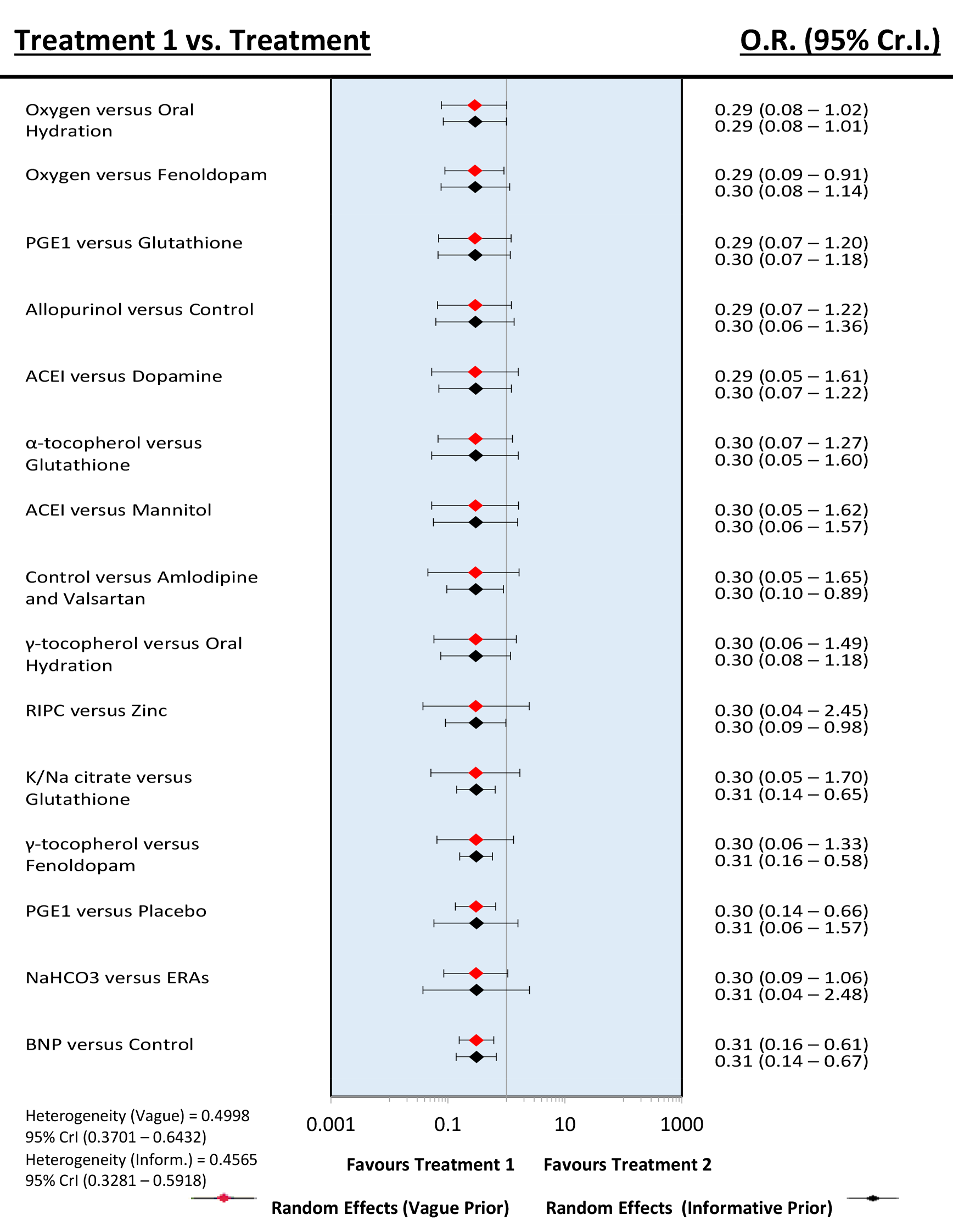


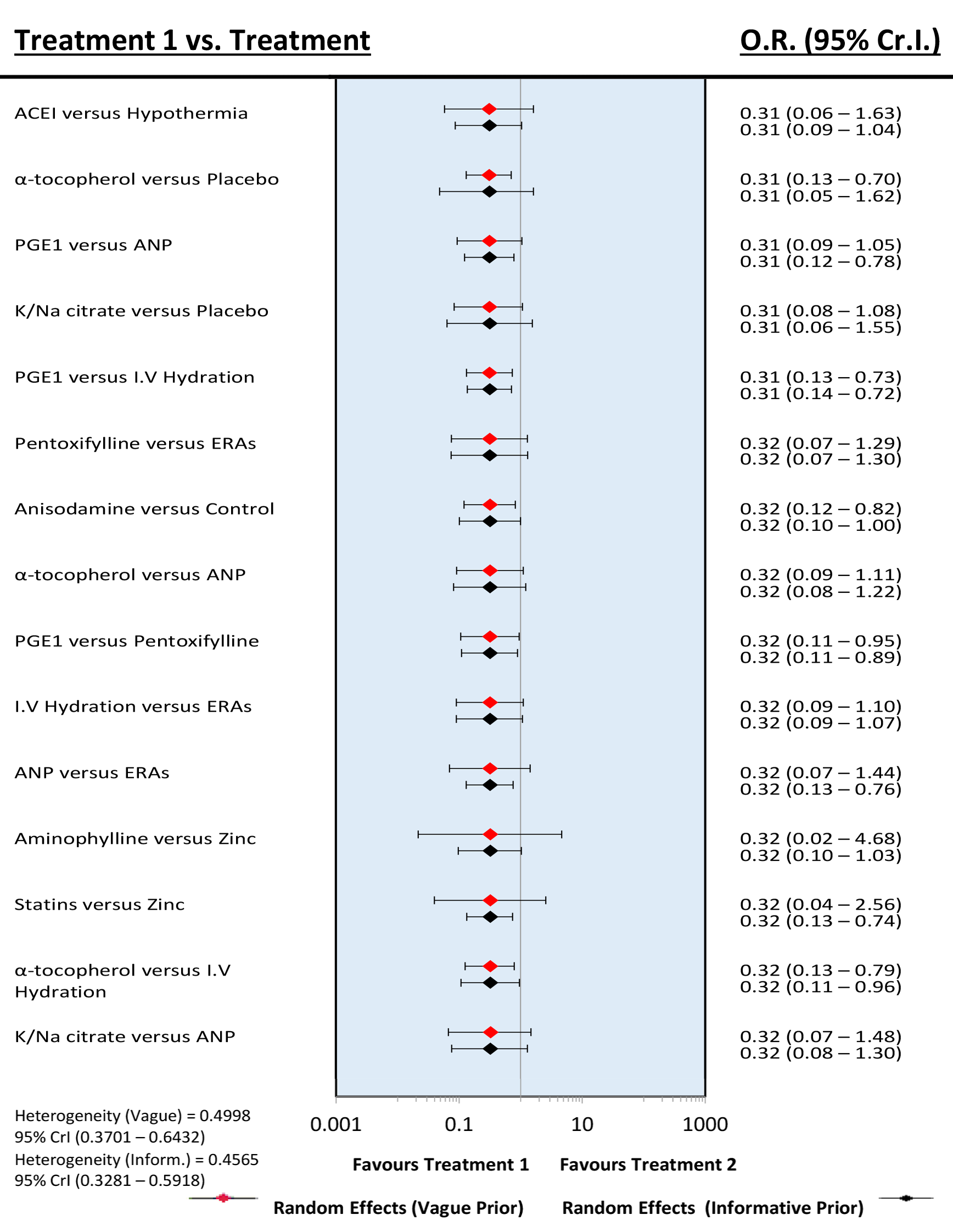


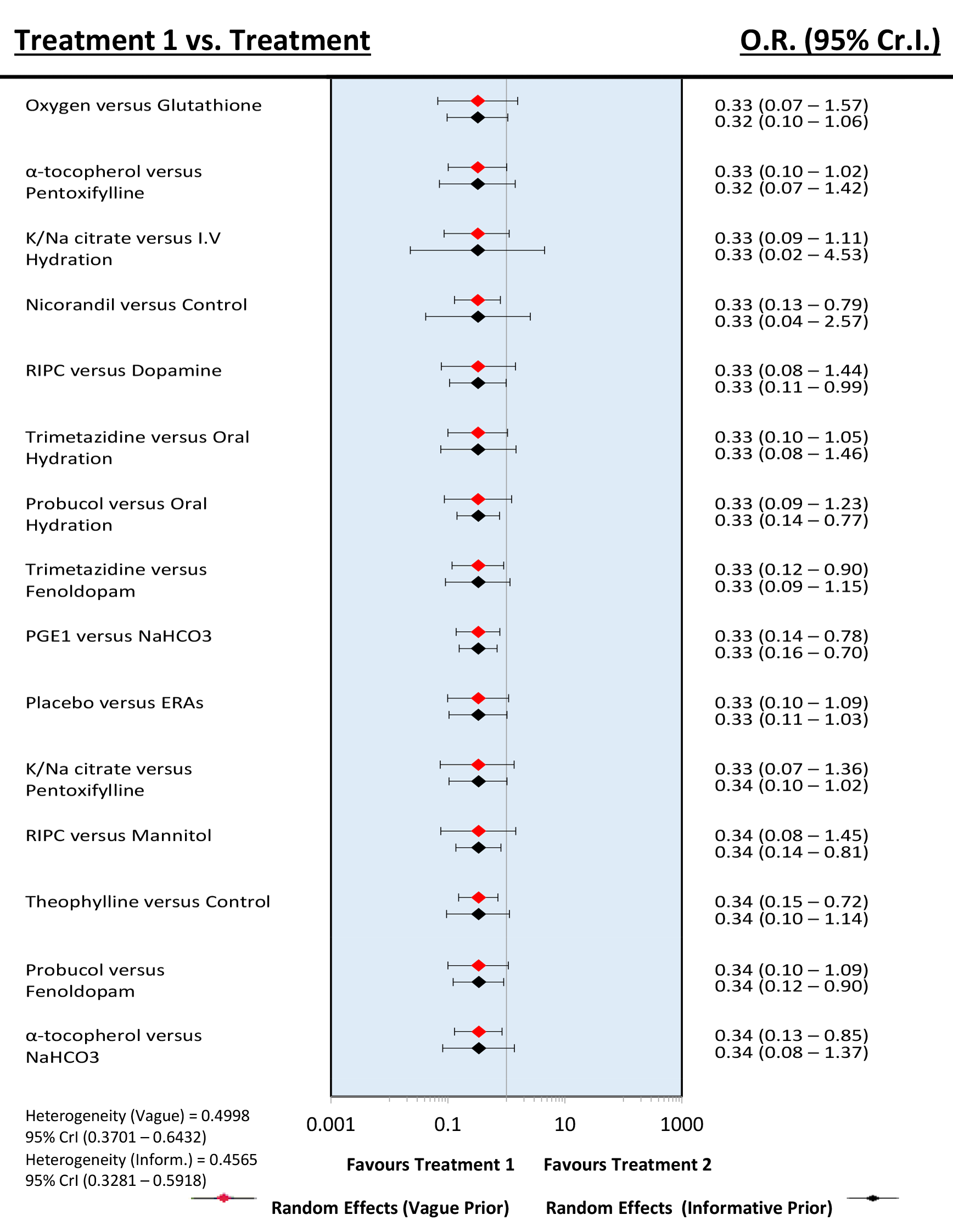


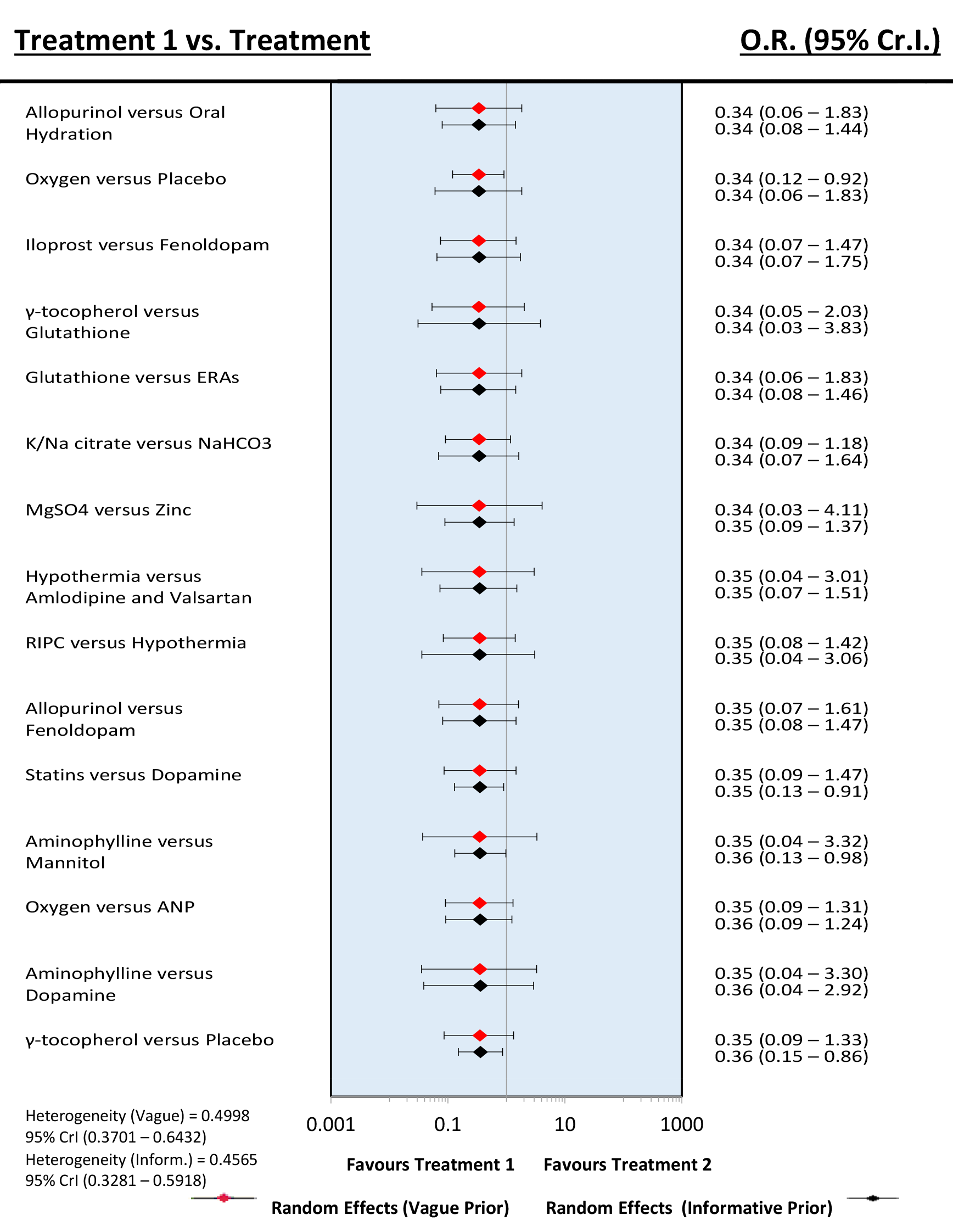


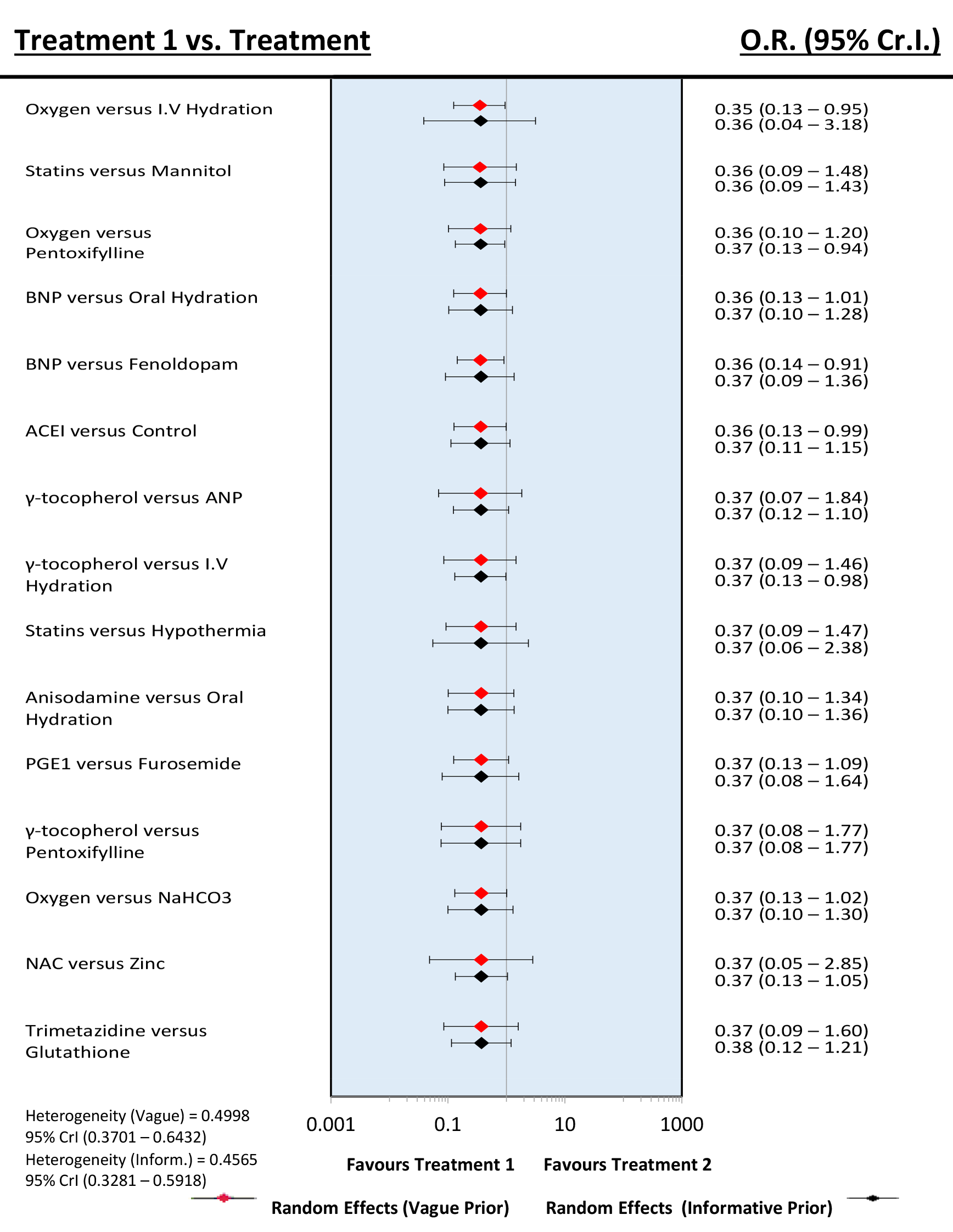


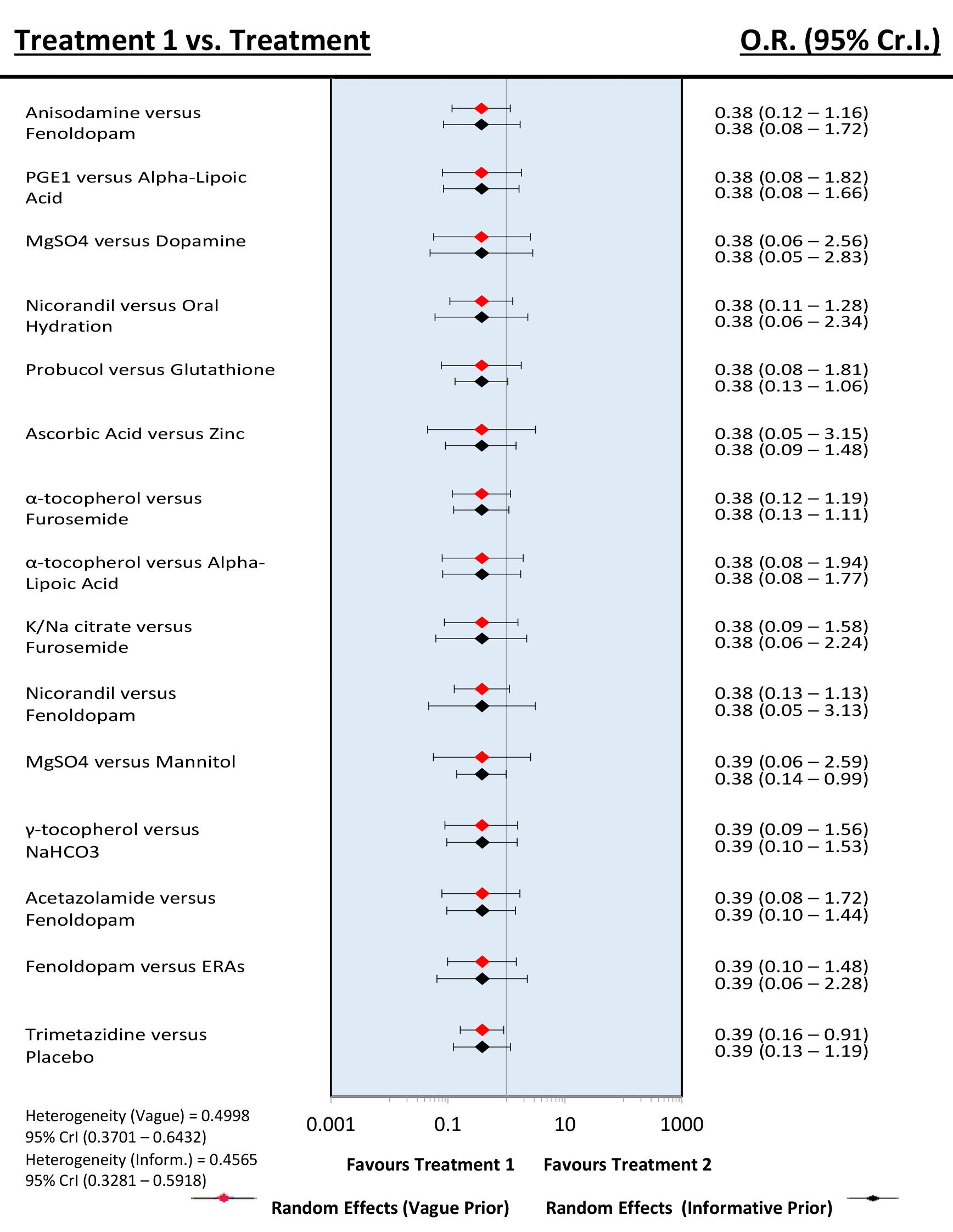


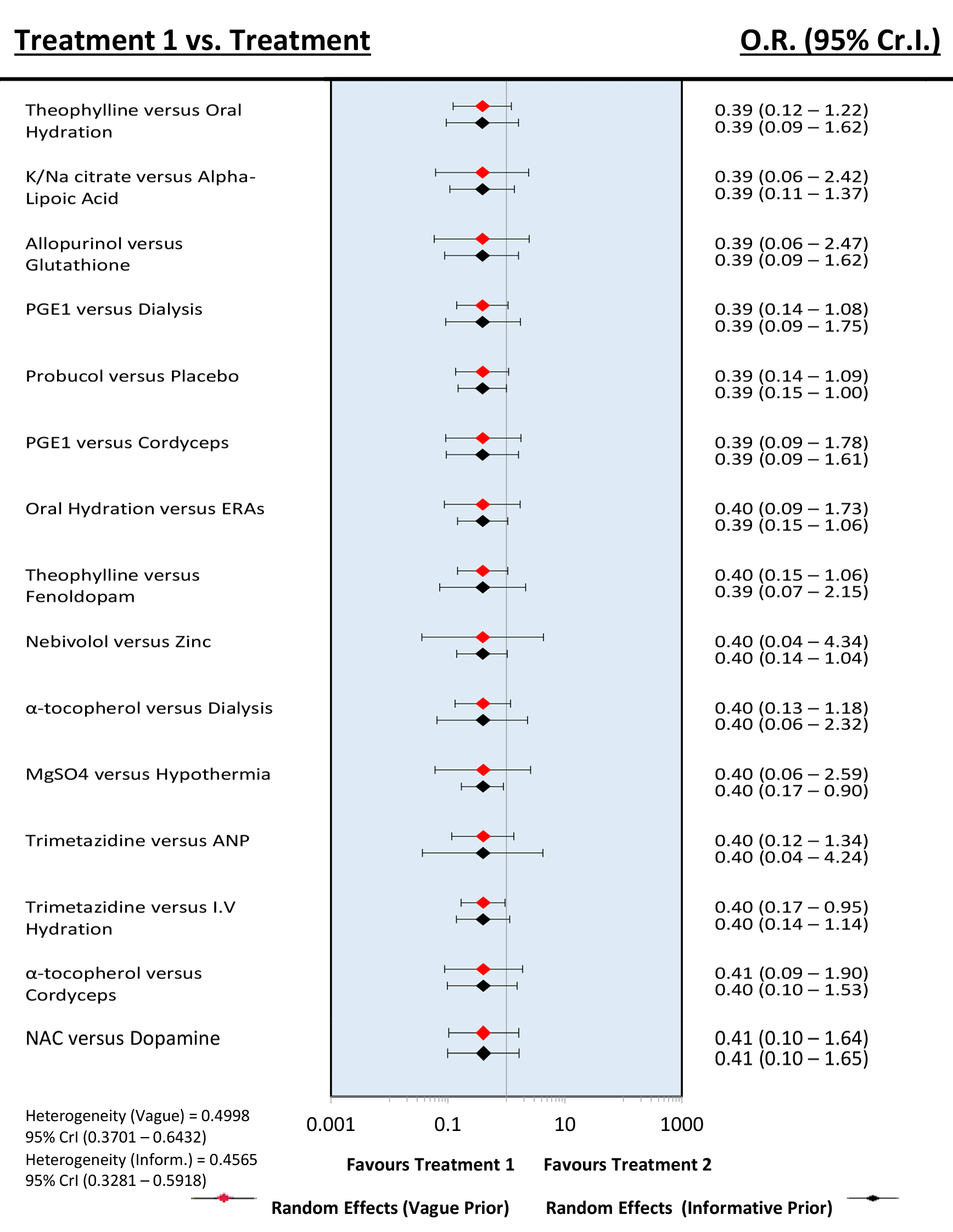


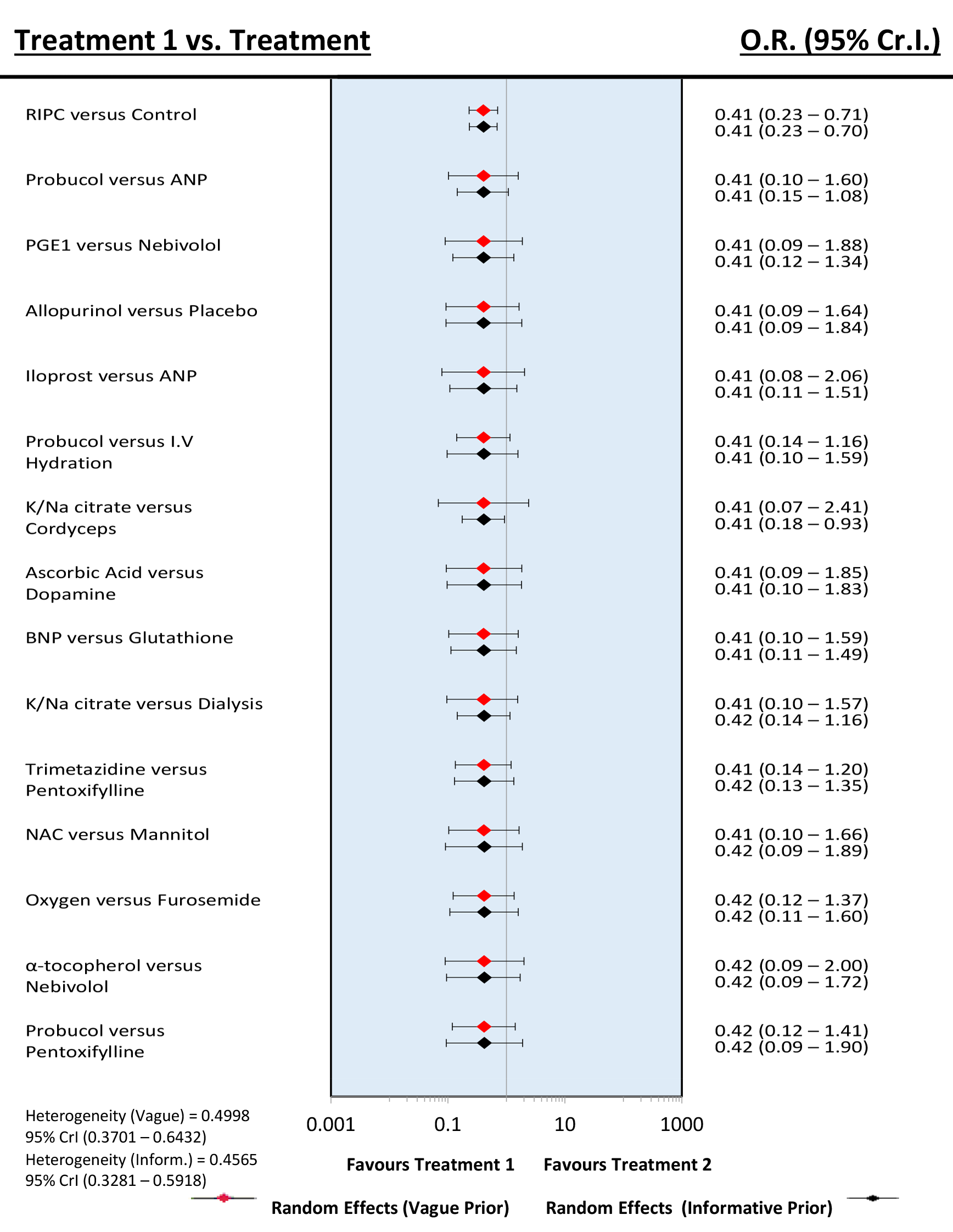


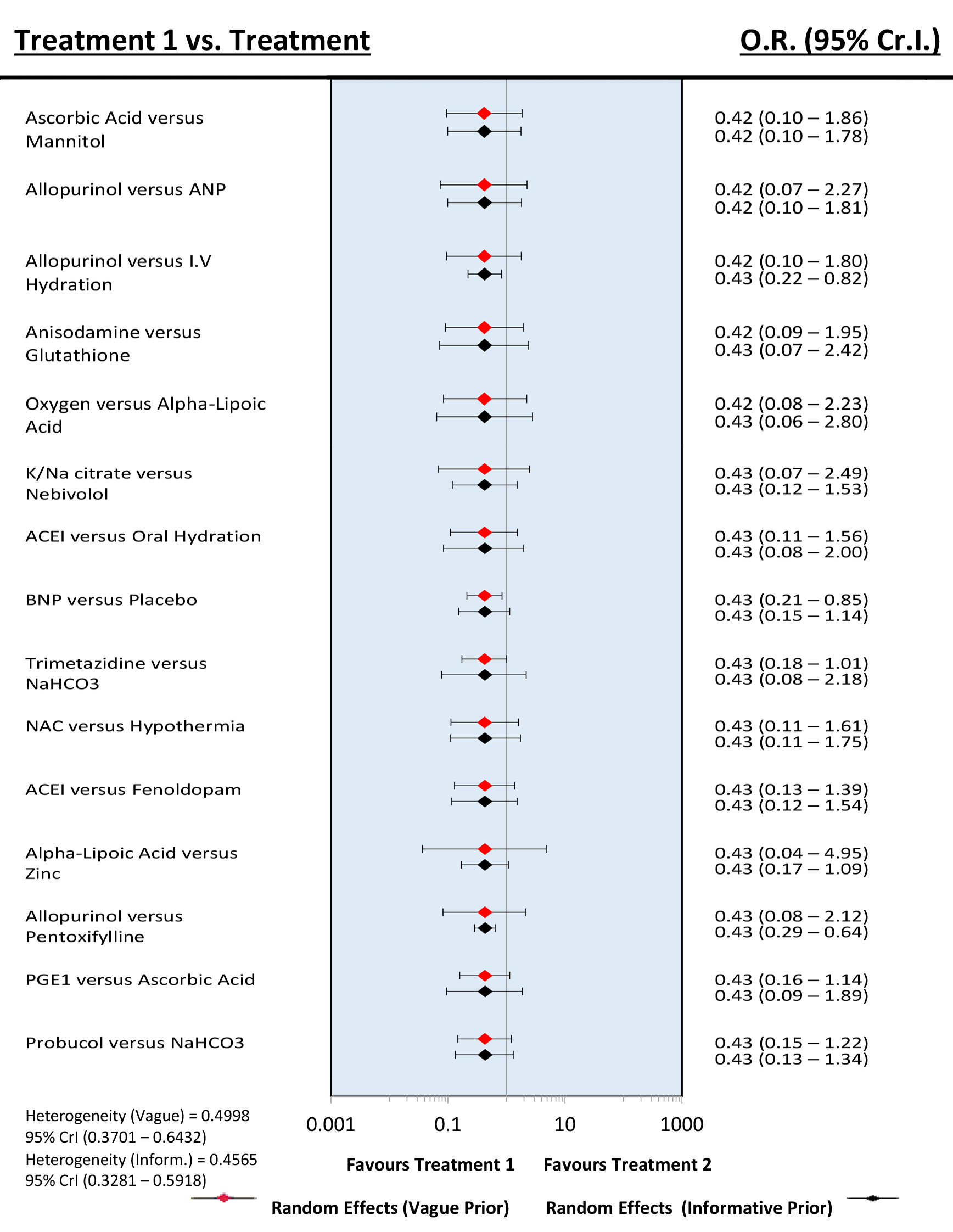


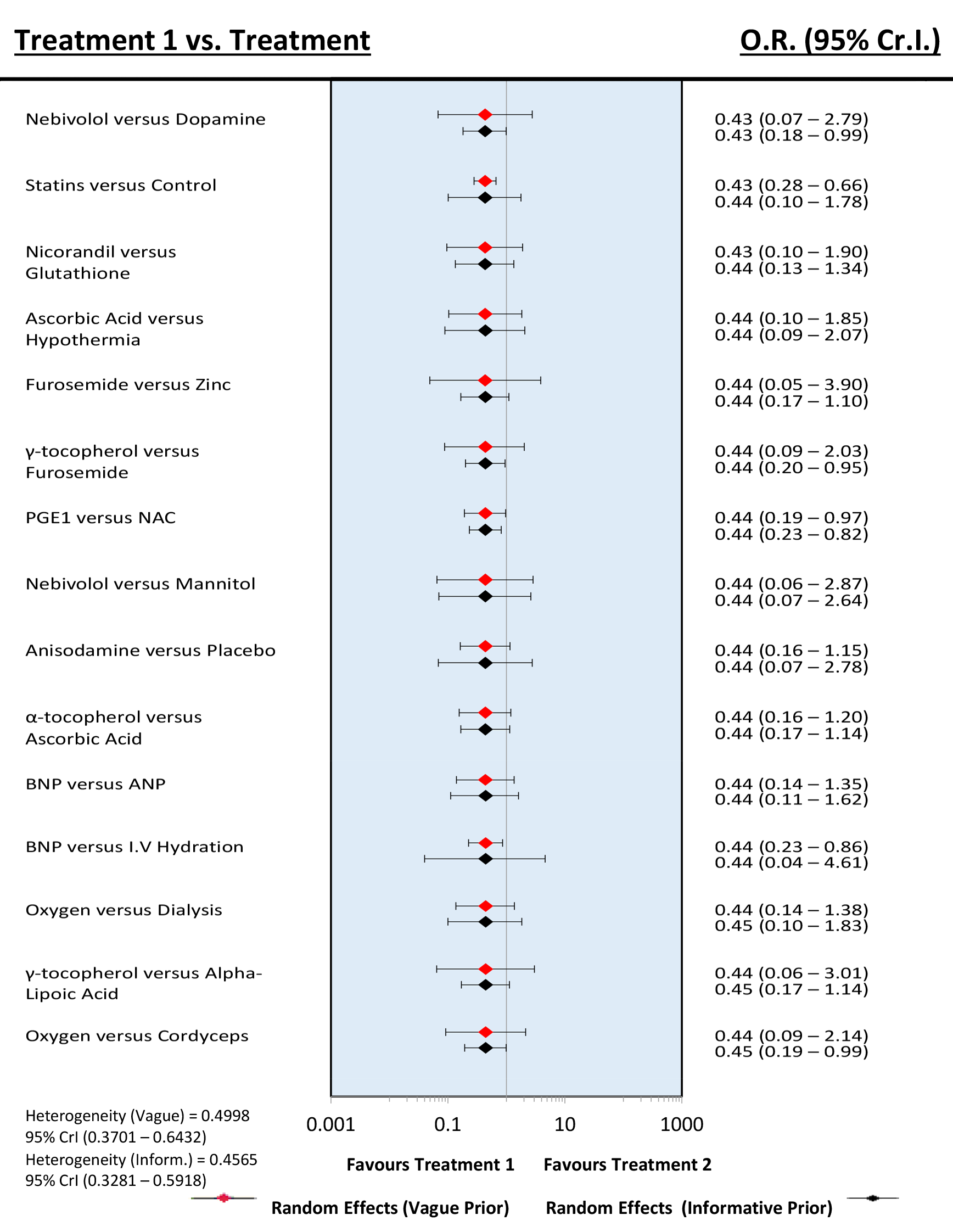


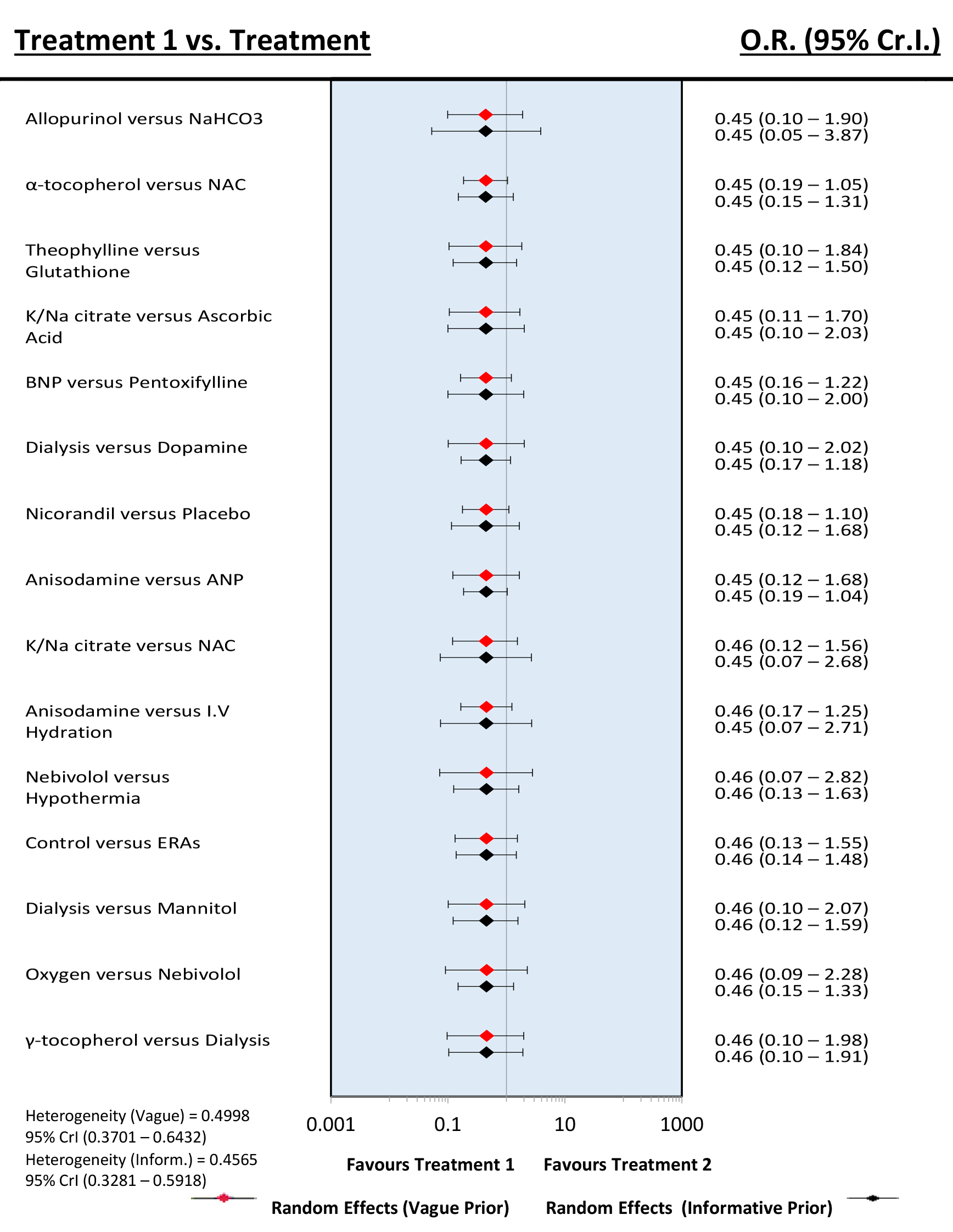


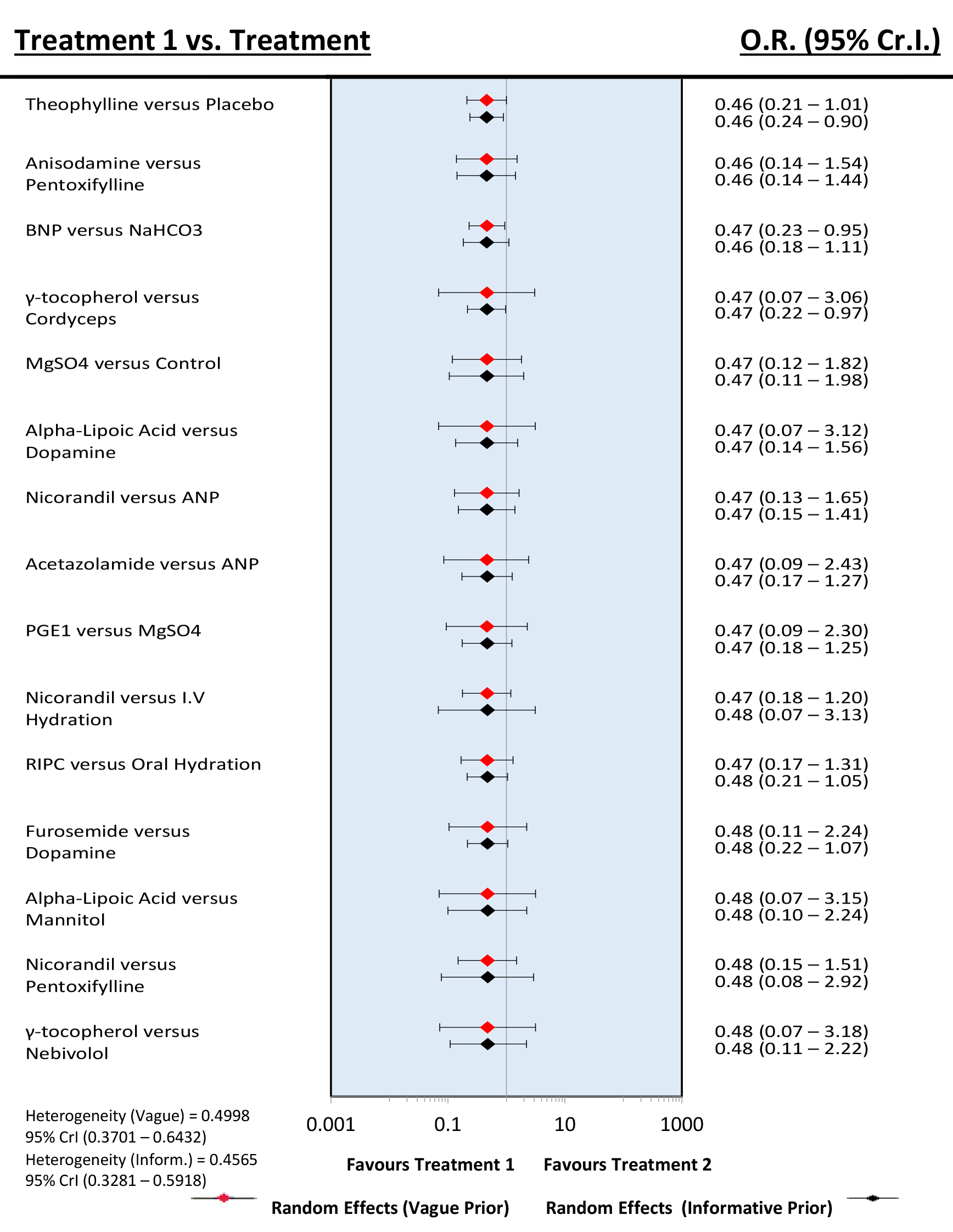


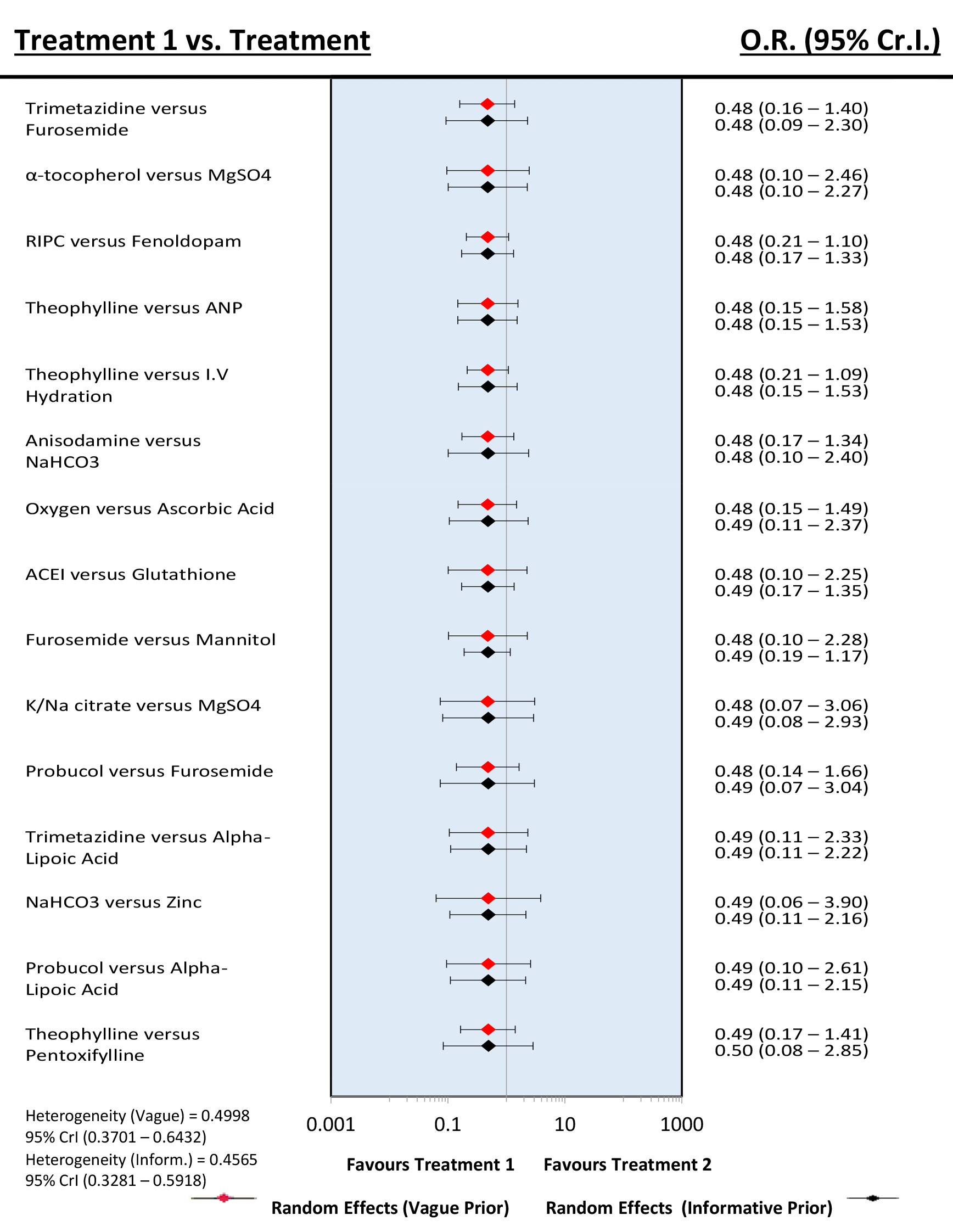


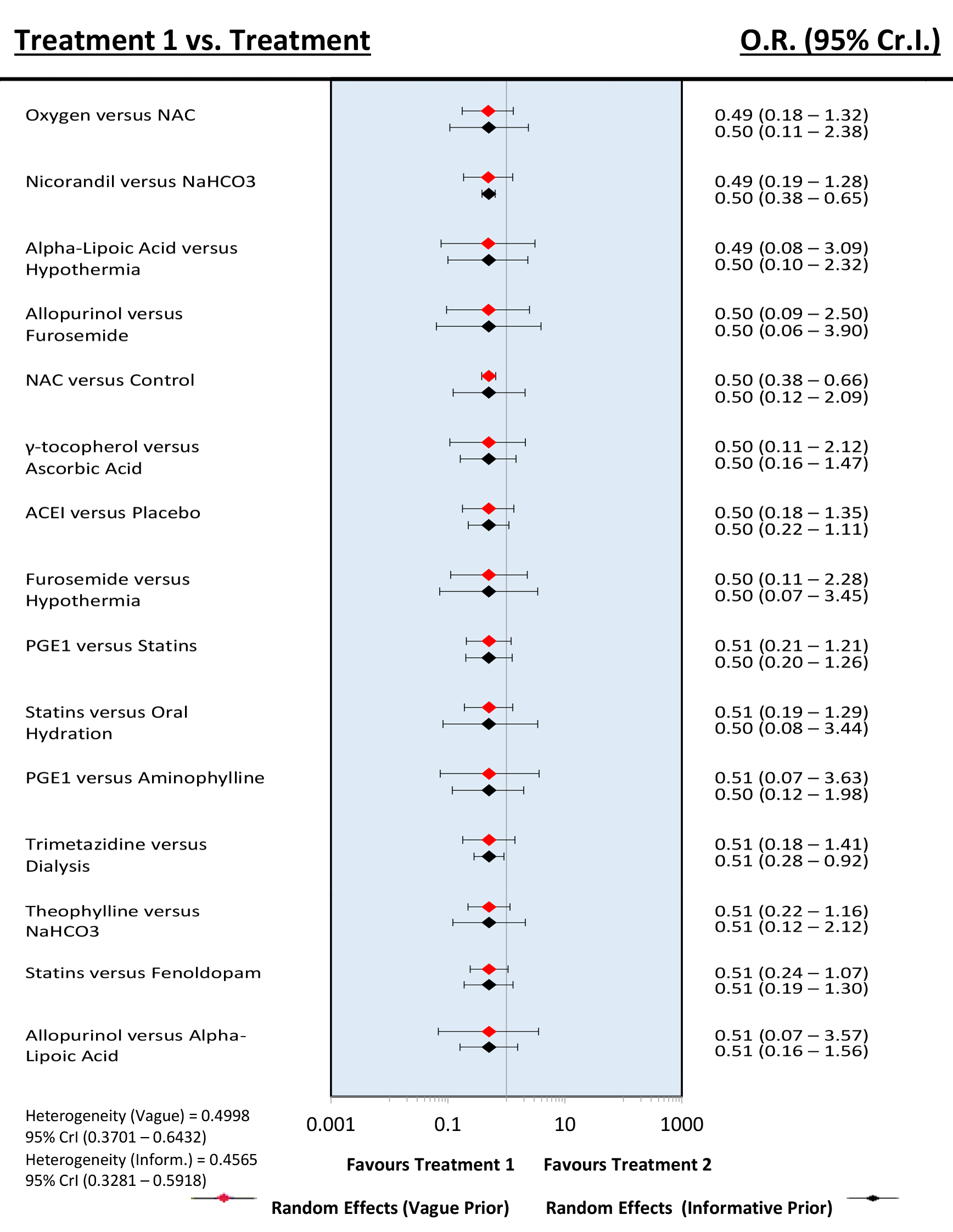


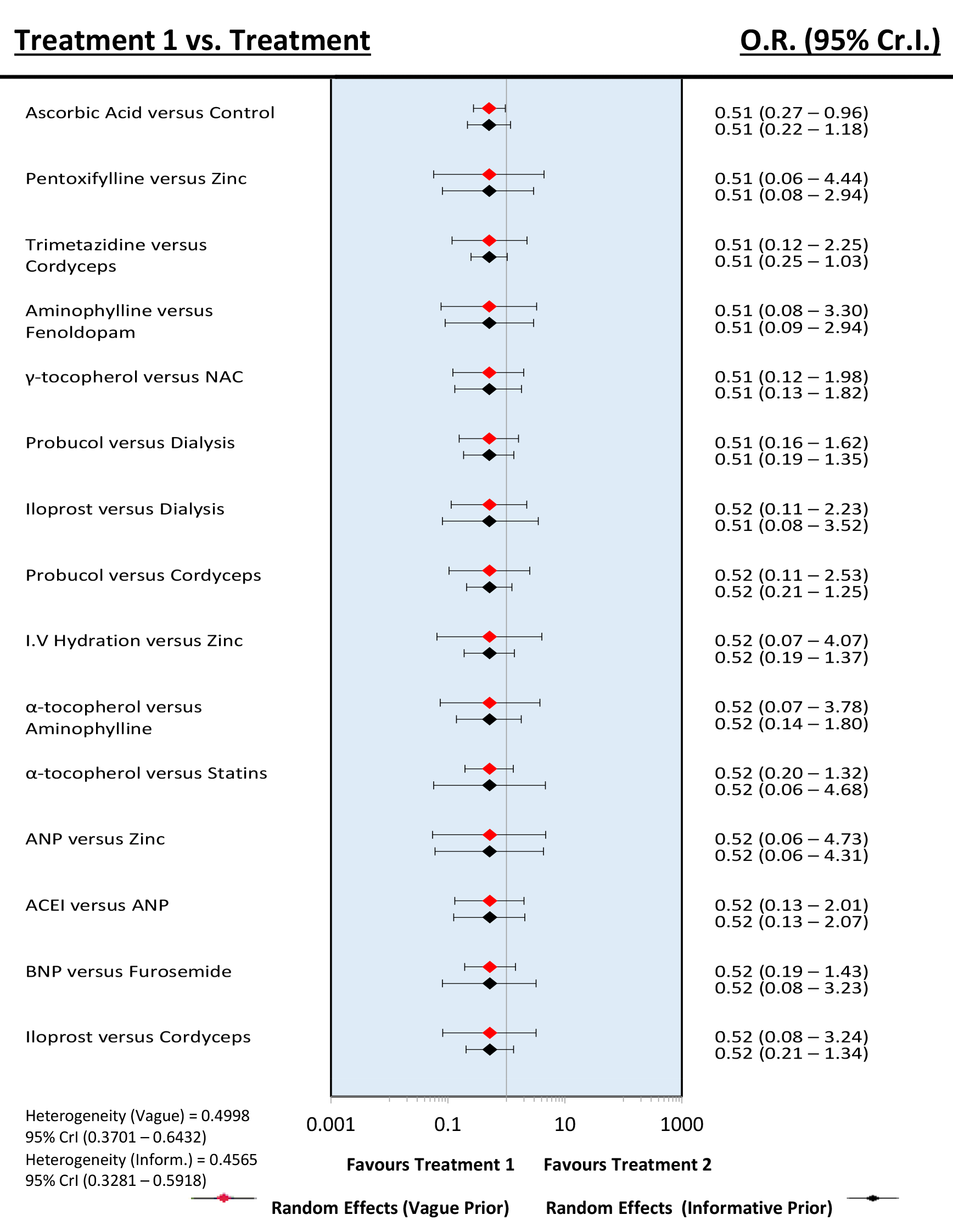


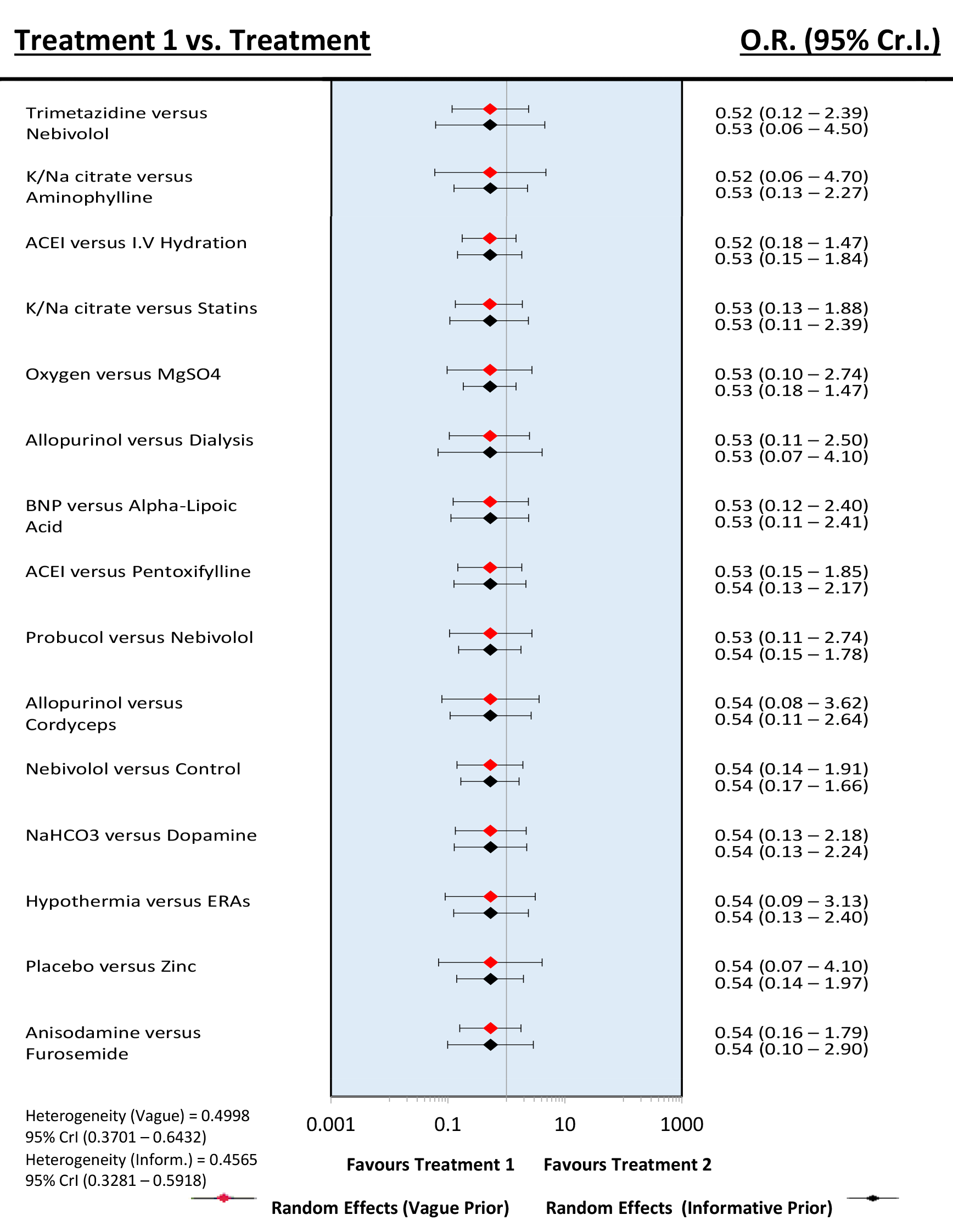


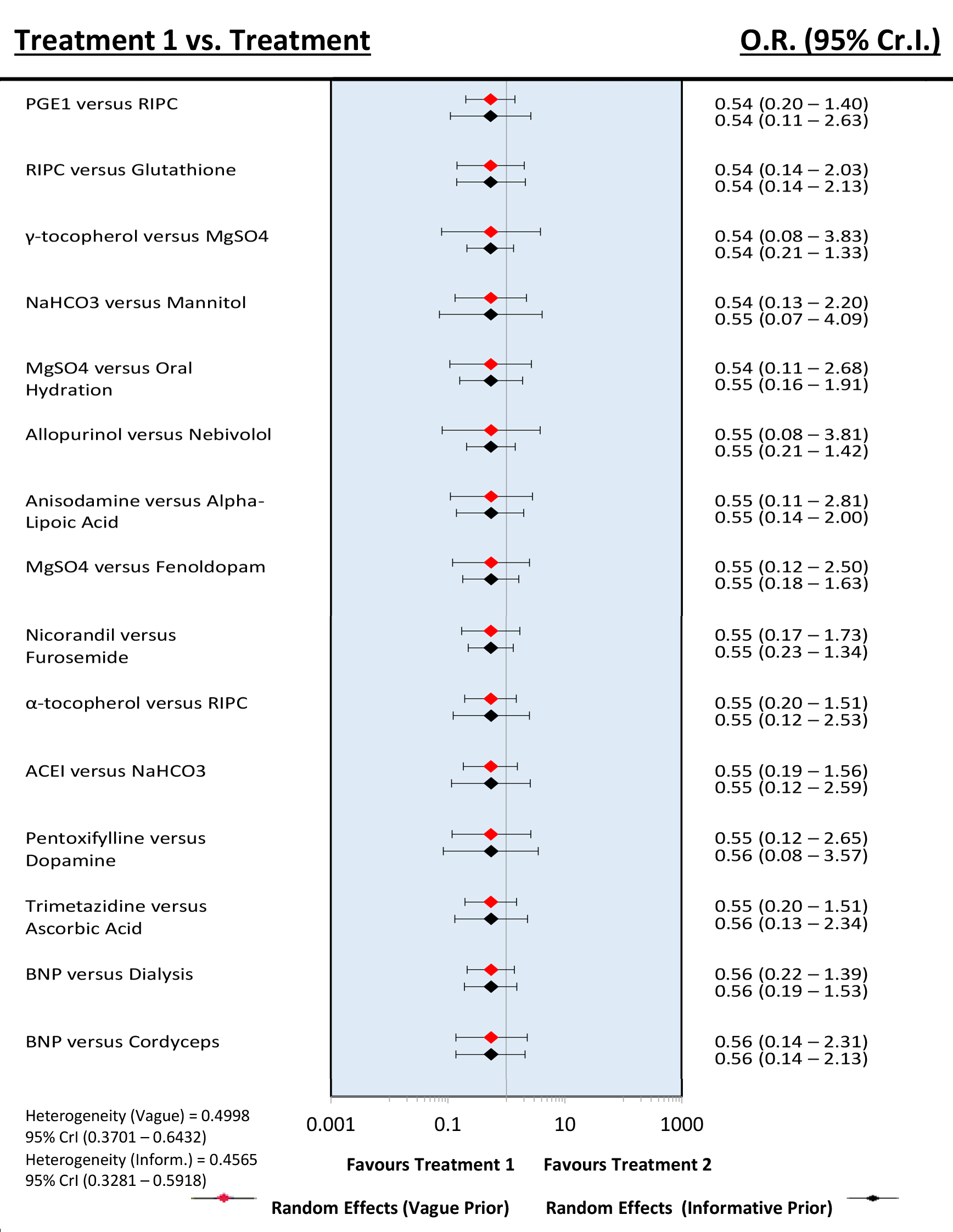


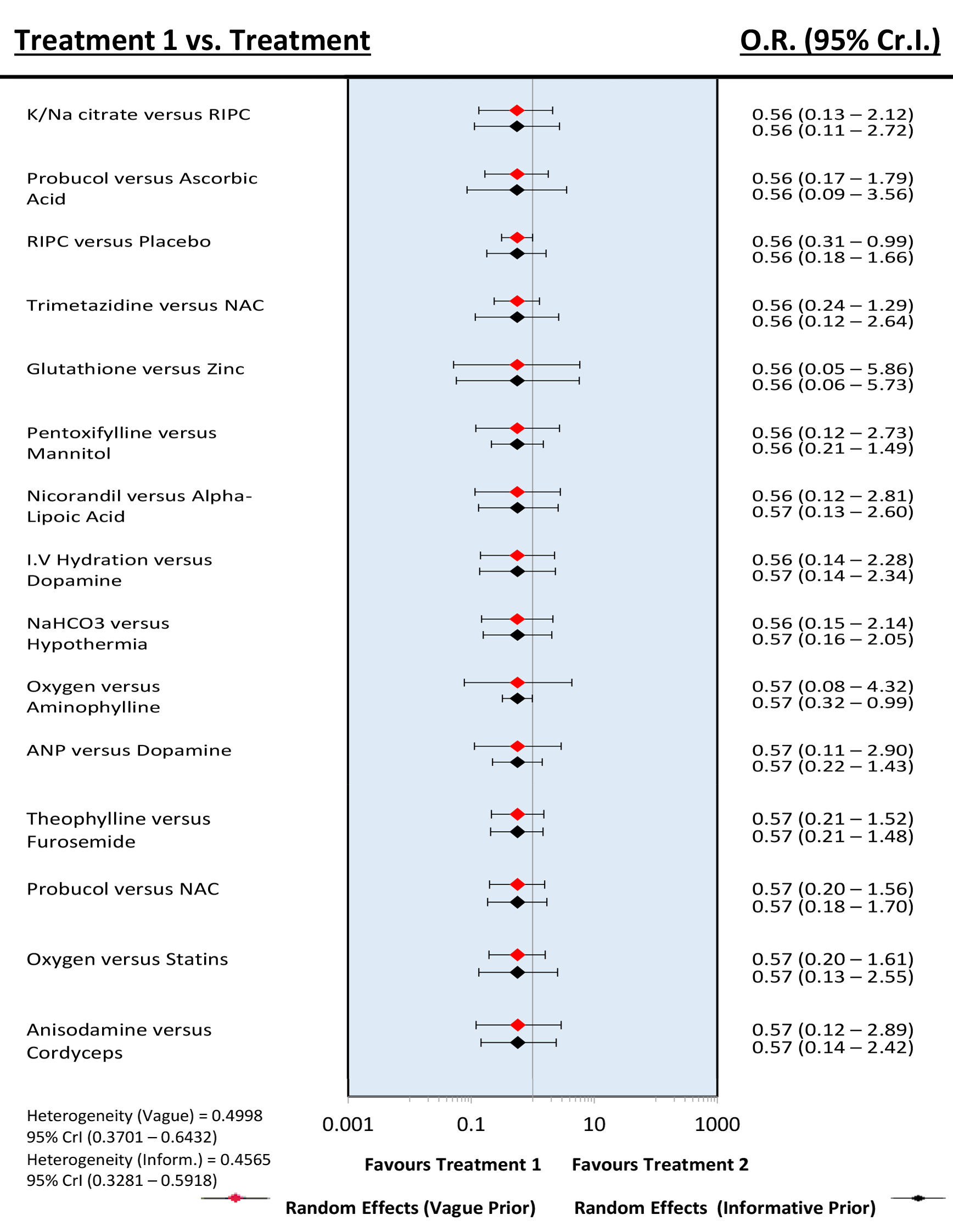


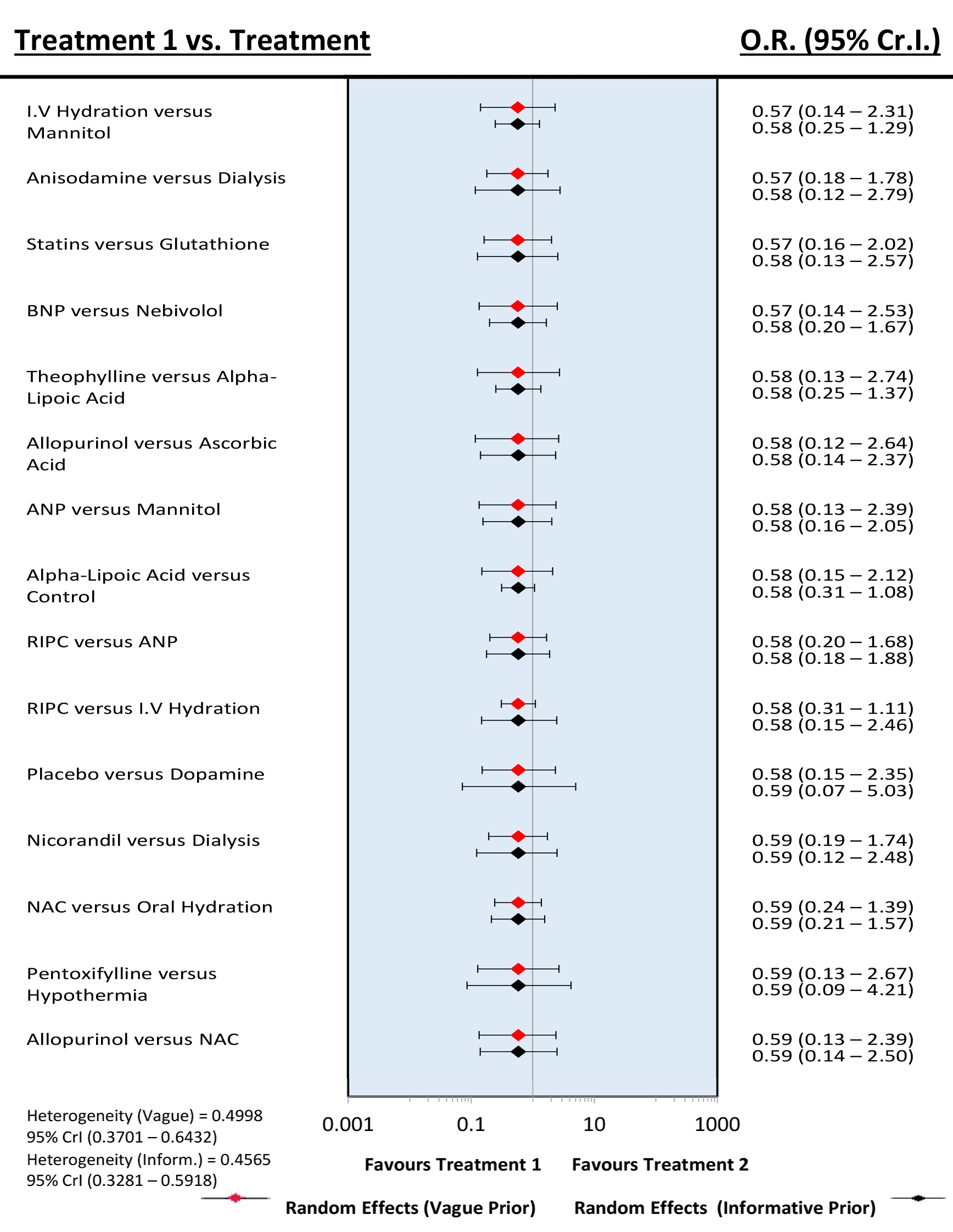


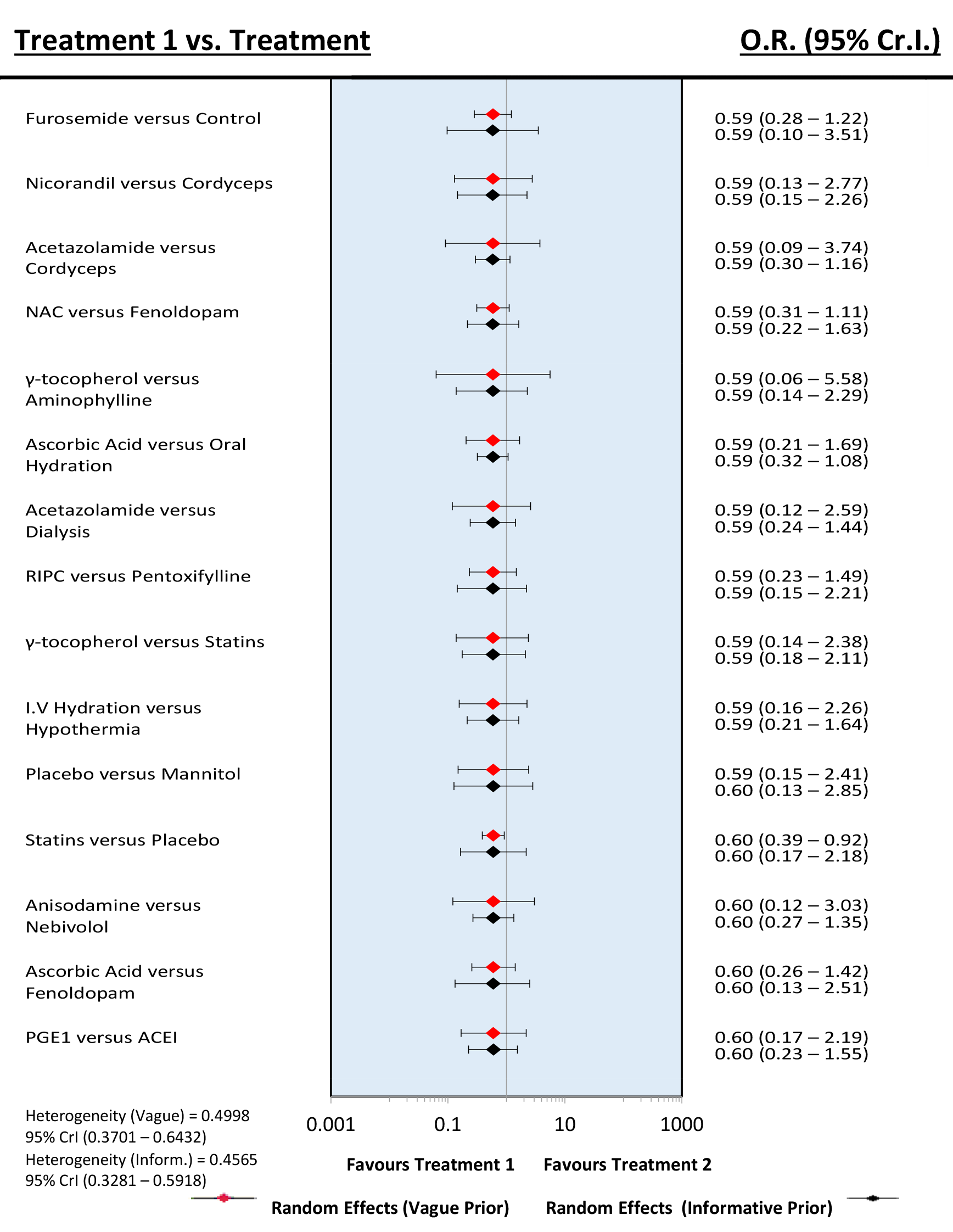


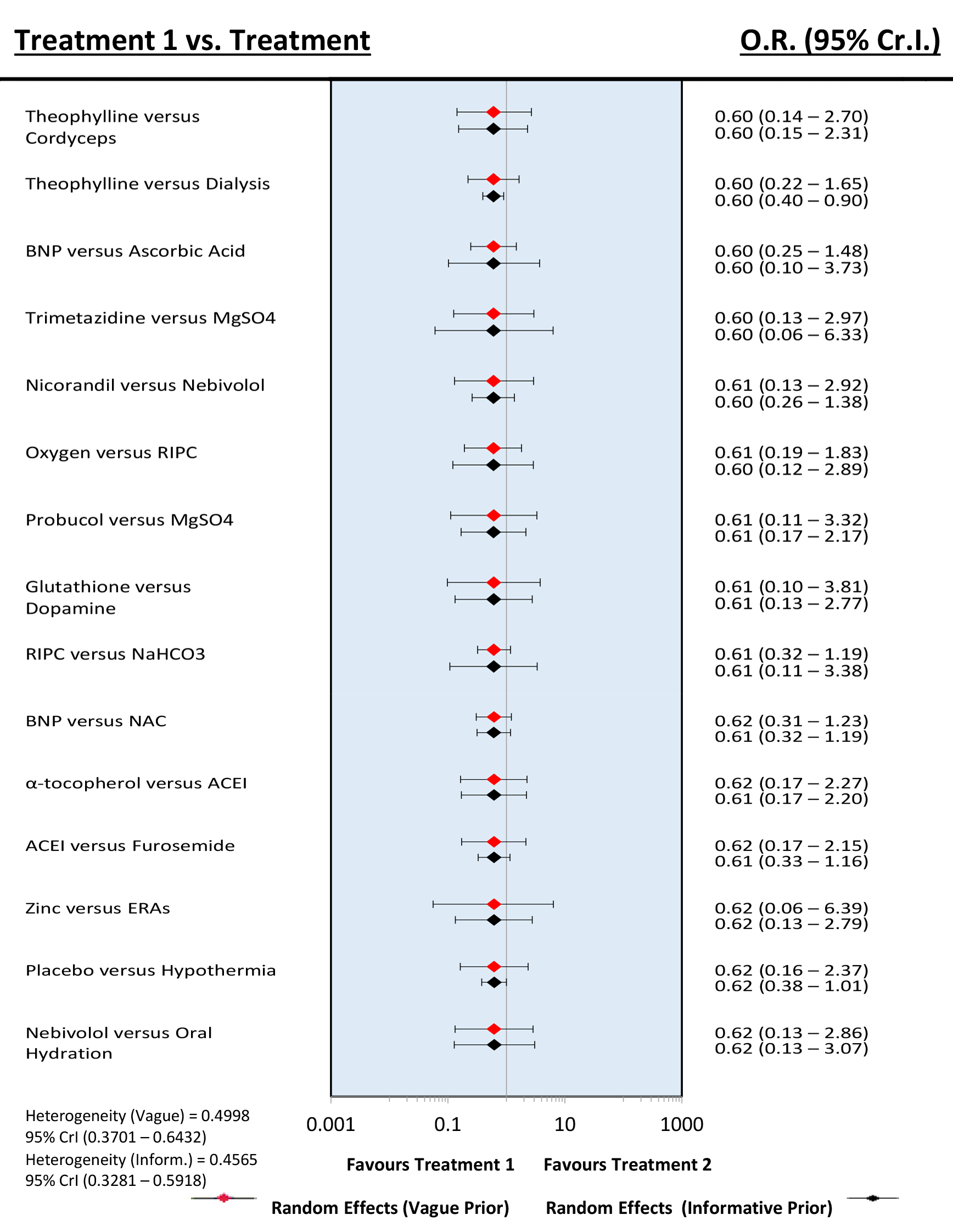


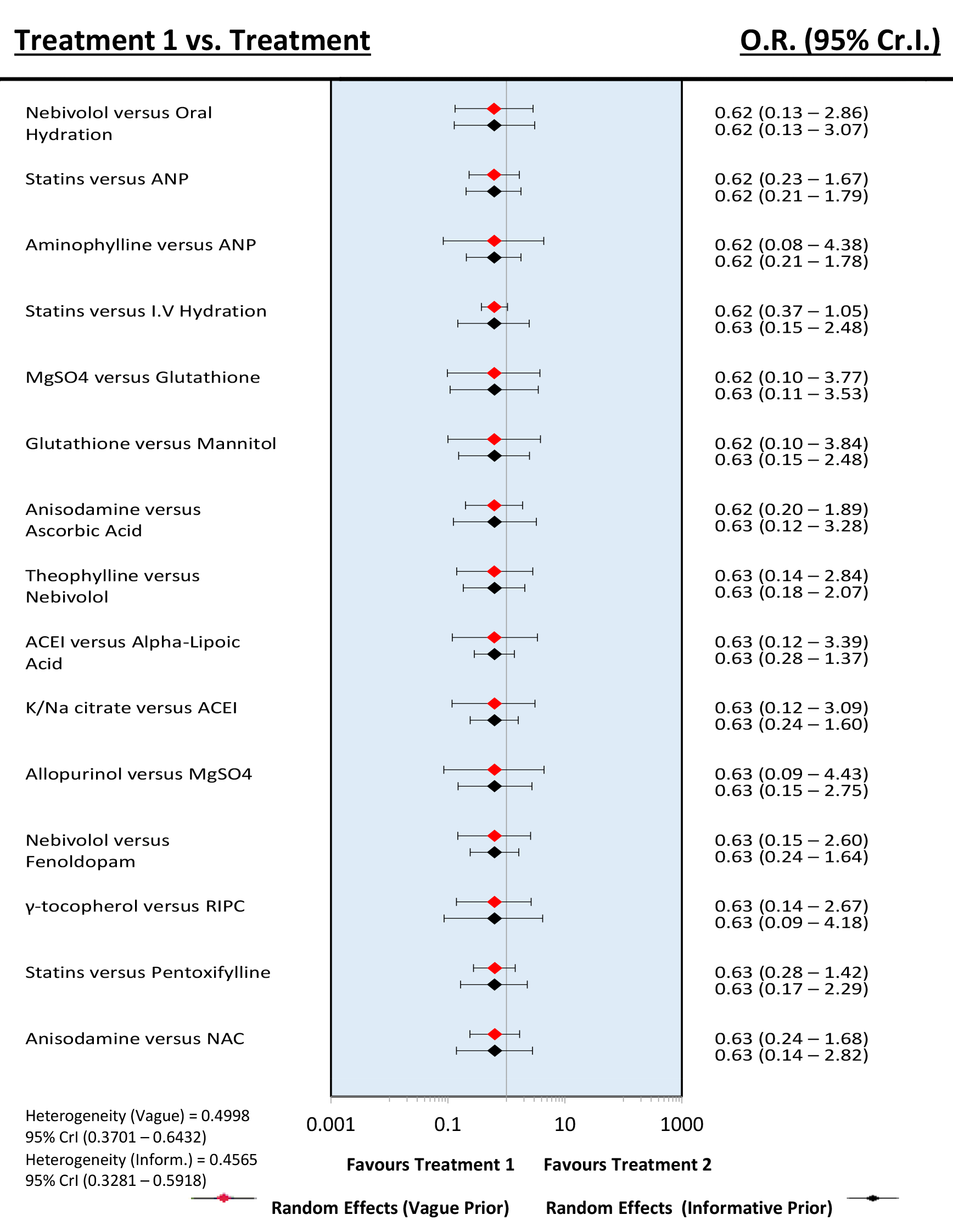


Figure 4Forest Plot (Results from R)

Figure 5Gelman and Rubin's convergence diagnostics

**Figure 6 League Table**
