# Supplementary material for: Effectiveness of contrast-associated acute kidney injury prevention methods; a systematic review and network meta-analysis
Source: BMC Nephrol. 2018 Nov 13;19:323. doi: 10.1186/s12882-018-1113-0 (PMC6234687; doi:10.1186/s12882-018-1113-0)
Supplement: Supplementary file 4 — Risk of Bias Table. (DOCX 1056 kb) [file 12882_2018_1113_MOESM4_ESM.docx]

| **Study Name & Year** | **Selection Bias** | | **Performance Bias** | **Detection Bias** | **Attrition Bias** | **Reporting Bias** |
| --- | --- | --- | --- | --- | --- | --- |
|  | Random Sequence Generation | Allocation Concealment | Blinding of participants and personnel | Blinding of Outcome Assessment | Incomplete Outcome Data | Selective Reporting |
| A.C.T Investigators, 2011 | 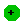 | 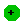 | 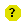 | 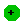 | 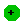 | 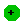 |
| Abaci, 2015 | 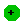 | 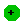 | 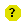 | 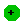 | 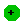 | 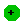 |
| Abouzeid 2016 | 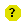 | 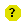 | 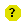 | 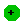 | 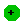 | 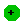 |
| Adolph, 2008 | 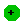 | 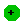 | 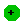 | 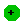 | 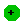 | 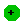 |
| Akyuz 2014 | 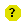 | 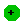 | 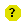 | 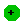 | 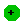 | 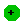 |
| Albabtain, 2013 | 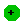 | 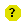 | 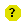 | 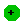 | 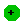 | 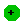 |
| Allaqaband, 2002 | 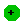 | 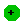 | 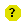 | 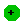 | 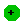 | 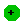 |
| Amini, 2009 | 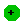 | 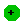 | 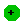 | 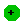 | 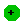 | 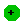 |
| Angoulvant, 2009 | 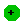 | 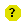 | 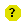 | 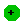 | 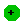 | 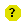 |
| Arabmomeni 2015 | 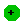 | 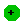 | 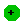 | 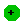 | 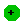 | 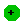 |
| Aslanger, 2012 | 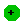 | 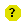 | 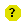 | 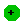 | 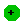 | 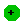 |
| Baker, 2003 | 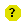 | 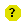 | 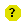 | 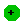 | 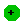 | 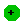 |
| Balderramo, 2004 | 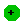 | 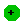 | 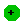 | 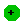 | 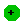 | 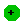 |
| Baskurt, 2009 | 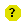 | 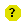 | 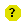 | 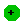 | 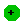 | 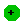 |
| Berwanger, 2013 | 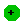 | 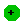 | 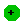 | 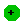 | 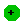 | 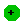 |
| Bidram, 2015 | 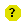 | 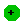 | 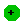 | 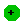 | 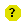 | 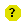 |
| Bilasy, 2012 | 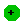 | 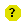 | 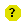 | 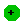 |  |  |
| Boscheri, 2007 |  |  |  |  |  |  |
| Boucek, 2013 |  |  |  |  |  |  |
| Brar, 2008 |  |  |  |  |  |  |
| Briguori 2002 |  |  |  |  |  |  |
| Briguori, 2004 |  |  |  |  |  |  |
| Briguori, 2007 |  |  |  |  |  |  |
| Brueck, 2013 |  |  |  |  |  |  |
| Burns, 2010 |  |  |  |  |  |  |
| Carbonell, 2007 |  |  |  |  |  |  |
| Carbonell, 2010 |  |  |  |  |  |  |
| Castini, 2010 |  |  |  |  |  |  |
| Chen, 2008 |  |  |  |  |  |  |
| Cho, 2010 |  |  |  |  |  |  |
| Cicek, 2013 |  |  |  |  |  |  |
| Coyle, 2006 |  |  |  |  |  |  |
| Durham, 2002 |  |  |  |  |  |  |
| Dussol, 2006 |  |  |  |  |  |  |
| Dvorsak, 2013 |  |  |  |  |  |  |
| Efrati, 2003 |  |  |  |  |  |  |
| Er, F. 2012 |  |  |  |  |  |  |
| Eric Chong 2015 |  |  |  |  |  |  |
| Erley, 1999 |  |  |  |  |  |  |
| Erol, 2013 |  |  |  |  |  |  |
| Erturk, 2014 |  |  |  |  |  |  |
| Eshraghi 2017 |  |  |  |  |  |  |
| Fan, Y 2016 |  |  |  |  |  |  |
| Ferrario, 2009 |  |  |  |  |  |  |
| Firouzi, 2012 |  |  |  |  |  |  |
| Firouzi, 2015 |  |  |  |  |  |  |
| Fung, 2004 |  |  |  |  |  |  |
| Gare 1999 |  |  |  |  |  |  |
| Geng, 2012 |  |  |  |  |  |  |
| Goldenberg, 2004 |  |  |  |  |  |  |
| Gomes, 2005 |  |  |  |  |  |  |
| Gomes, 2012 |  |  |  |  |  |  |
| Gu, 2013 |  |  |  |  |  |  |
| Gulel, 2005 |  |  |  |  |  |  |
| Gunebakmaz, 2012 |  |  |  |  |  |  |
| Gupta, 1999 |  |  |  |  |  |  |
| Hafiz, 2012 |  |  |  |  |  |  |
| Han 2014 |  |  |  |  |  |  |
| Hashemi 2005 |  |  |  |  |  |  |
| Healy, D 2015 |  |  |  |  |  |  |
| Heguilen, 2013 |  |  |  |  |  |  |
| Heng, 2008 |  |  |  |  |  |  |
| Hoole 2009 |  |  |  |  |  |  |
| Hsu, 2012 |  |  |  |  |  |  |
| Huber, 2003 |  |  |  |  |  |  |
| Inda-Filho, 2014 |  |  |  |  |  |  |
| Izani 2008 |  |  |  |  |  |  |
| Jaffery, 2012 |  |  |  |  |  |  |
| Jo 2008 |  |  |  |  |  |  |
| Jo, 2009 |  |  |  |  |  |  |
| Jo, 2013 |  |  |  |  |  |  |
| Jurado-Roman 2015 |  |  |  |  |  |  |
| Kai, Z 2015 |  |  |  |  |  |  |
| Kama, 2014 |  |  |  |  |  |  |
| Kay, 2003 |  |  |  |  |  |  |
| Kefer 2003 |  |  |  |  |  |  |
| Khosravi 2016 |  |  |  |  |  |  |
| Khoury, 1995 |  |  |  |  |  |  |
| Kimmel, 2008 |  |  |  |  |  |  |
| Kinbara, 2010 |  |  |  |  |  |  |
| Kitzler, 2012 |  |  |  |  |  |  |
| Klima, 2012 |  |  |  |  |  |  |
| Ko, 2013 |  |  |  |  |  |  |
| Koc, 2012 |  |  |  |  |  |  |
| Koc, 2013 |  |  |  |  |  |  |
| Koch, 2000 |  |  |  |  |  |  |
| Kong, 2012 |  |  |  |  |  |  |
| Kooiman 2014 |  |  |  |  |  |  |
| Kooiman, 2014a |  |  |  |  |  |  |
| Kotlyar, 2005 |  |  |  |  |  |  |
| Kumar, 2014 |  |  |  |  |  |  |
| Kurnik 1990 |  |  |  |  |  |  |
| Kurnik, 1998 |  |  |  |  |  |  |
| Lawlor, 2007 |  |  |  |  |  |  |
| Lee, 2011 |  |  |  |  |  |  |
| Lehnert, 1998 |  |  |  |  |  |  |
| Leoncini, 2014 |  |  |  |  |  |  |
| Li, 2009 |  |  |  |  |  |  |
| Li, 2011 |  |  |  |  |  |  |
| Li, 2014 |  |  |  |  |  |  |
| Liu, W 2015 |  |  |  |  |  |  |
| Liu, 2014 |  |  |  |  |  |  |
| Liu 2016 |  |  |  |  |  |  |
| Li W 2012 |  |  |  |  |  |  |
| Ludwig, 2011 |  |  |  |  |  |  |
| Luo 2014 |  |  |  |  |  |  |
| Luo S 2013 |  |  |  |  |  |  |
| MacNeill, 2003 |  |  |  |  |  |  |
| Maioli, 2008 |  |  |  |  |  |  |
| Maioli, 2011 |  |  |  |  |  |  |
| Malhis, 2010 |  |  |  |  |  |  |
| Marenzi, 2003 |  |  |  |  |  |  |
| Marenzi, 2006 |  |  |  |  |  |  |
| Marenzi, 2006a |  |  |  |  |  |  |
| Marenzi, 2012 |  |  |  |  |  |  |
| Markota, 2013 |  |  |  |  |  |  |
| Masuda, 2007 |  |  |  |  |  |  |
| Matejka, 2010 |  |  |  |  |  |  |
| Menting 2015 |  |  |  |  |  |  |
| Merten, 2004 |  |  |  |  |  |  |
| Miao, 2013 |  |  |  |  |  |  |
| Miner, 2004 |  |  |  |  |  |  |
| Minoo 2016 |  |  |  |  |  |  |
| Moore, 2006 |  |  |  |  |  |  |
| Morikawa, 2009 |  |  |  |  |  |  |
| Motohiro, 2011 |  |  |  |  |  |  |
| Nawa 2015 |  |  |  |  |  |  |
| Ng, 2006 |  |  |  |  |  |  |
| Nijssen 2017 |  |  |  |  |  |  |
| Ochoa, 2004 |  |  |  |  |  |  |
| Oguzhan, 2013 |  |  |  |  |  |  |
| Oldemeyer, 2003 |  |  |  |  |  |  |
| Onbasili, 2007 |  |  |  |  |  |  |
| Ozcan, 2007 |  |  |  |  |  |  |
| Ozhan, 2010 |  |  |  |  |  |  |
| Pakfetrat, 2009 |  |  |  |  |  |  |
| Patti, 2011 |  |  |  |  |  |  |
| Poletti, 2013 |  |  |  |  |  |  |
| Qiao, 2015 |  |  |  |  |  |  |
| Quintavalle, 2012 |  |  |  |  |  |  |
| Rahman, 2012 |  |  |  |  |  |  |
| Rashid, 2004 |  |  |  |  |  |  |
| Reinecke, 2007 |  |  |  |  |  |  |
| Rezaei 2016 |  |  |  |  |  |  |
| Rohani 2010 |  |  |  |  |  |  |
| Sadat, 2011 |  |  |  |  |  |  |
| Sadineni 2017 |  |  |  |  |  |  |
| Saitoh, 2011 |  |  |  |  |  |  |
| Sandhu, 2006 |  |  |  |  |  |  |
| Sanei, 2014 |  |  |  |  |  |  |
| Sar 2010 |  |  |  |  |  |  |
| Savaj 2014 |  |  |  |  |  |  |
| Sedighifard 2016 |  |  |  |  |  |  |
| Sekiguchi, 2013 |  |  |  |  |  |  |
| Seyon, 2007 |  |  |  |  |  |  |
| Shehata, 2014 |  |  |  |  |  |  |
| Shehata, 2015 |  |  |  |  |  |  |
| Shyu, 2002 |  |  |  |  |  |  |
| Singh 2016 |  |  |  |  |  |  |
| Solomon 1994 |  |  |  |  |  |  |
| Solomon 2015 |  |  |  |  |  |  |
| Spargias, 2004 |  |  |  |  |  |  |
| Spargias, 2006 |  |  |  |  |  |  |
| Spargias, 2009 |  |  |  |  |  |  |
| Stone, 2003 |  |  |  |  |  |  |
| Stone, 2011 |  |  |  |  |  |  |
| Sun, C 2015 |  |  |  |  |  |  |
| Tamura, 2009 |  |  |  |  |  |  |
| Tanaka, 2011 |  |  |  |  |  |  |
| Tasanarong, 2009 |  |  |  |  |  |  |
| Tasanarong, 2013 |  |  |  |  |  |  |
| Tepel, 2000 |  |  |  |  |  |  |
| Thiele, 2010 |  |  |  |  |  |  |
| Toso, 2010 |  |  |  |  |  |  |
| Traub, 2013 |  |  |  |  |  |  |
| Trivedi, 2003 |  |  |  |  |  |  |
| Tumlin, 2002 |  |  |  |  |  |  |
| Vasheghani-Farahani, 2009 |  |  |  |  |  |  |
| Vasheghani-Farahani, 2010 |  |  |  |  |  |  |
| Vogt, 2001 |  |  |  |  |  |  |
| Wang, C 2015 |  |  |  |  |  |  |
| Wang, Y. 2011 |  |  |  |  |  |  |
| Wang, 2000 |  |  |  |  |  |  |
| Webb, 2004 |  |  |  |  |  |  |
| Weisberg, 1993 |  |  |  |  |  |  |
| Wrobel, 2010 |  |  |  |  |  |  |
| Xu, R. H 2013 |  |  |  |  |  |  |
| Xu 2014 |  |  |  |  |  |  |
| Yamanaka 2015 |  |  |  |  |  |  |
| Yang 2014 |  |  |  |  |  |  |
| Yavari, 2014 |  |  |  |  |  |  |
| Yeganehkhah, 2014 |  |  |  |  |  |  |
| Yin, 2013 |  |  |  |  |  |  |
| Zagidullin 2017 |  |  |  |  |  |  |
| Zhang, 2010 |  |  |  |  |  |  |
| Zhao 2014 |  |  |  |  |  |  |
| Zhou, 2012 |  |  |  |  |  |  |
