# Supplementary material for: Effectiveness of contrast-associated acute kidney injury prevention methods; a systematic review and network meta-analysis
Source: BMC Nephrol. 2018 Nov 13;19:323. doi: 10.1186/s12882-018-1113-0 (PMC6234687; doi:10.1186/s12882-018-1113-0)
Supplement: Supplementary file 5 — Excluding hyperosmolar 173–159 RCTs. (DOCX 41962 kb) [file 12882_2018_1113_MOESM5_ESM.docx]

**Supplement:**

**Results from Analysis 2 (173 RCTs after excluding studies with any partial use of hyperosmolar contrast media)**

Number of Studies: 173 RCTs (see Manuscript)

Figures and Tables:

1. Network Diagram

2. Tables:

A. Network Characteristics

B. Interventions Characteristics

C Direct comparisons characteristics

3. Rankogram

4. Ranking and probability of being the best (included in the main manuscript)

5. Forest Plot

| Software | Spec | Convergence | Analysis |
| --- | --- | --- | --- |
| Netmetaxl / WinBUGS14 version 1.4.3 | Burn 5000  Sim 10000 | good convergence (FE MC error 5% of the SD) | Random Effects (Vague)  Random Effects (Informative) |


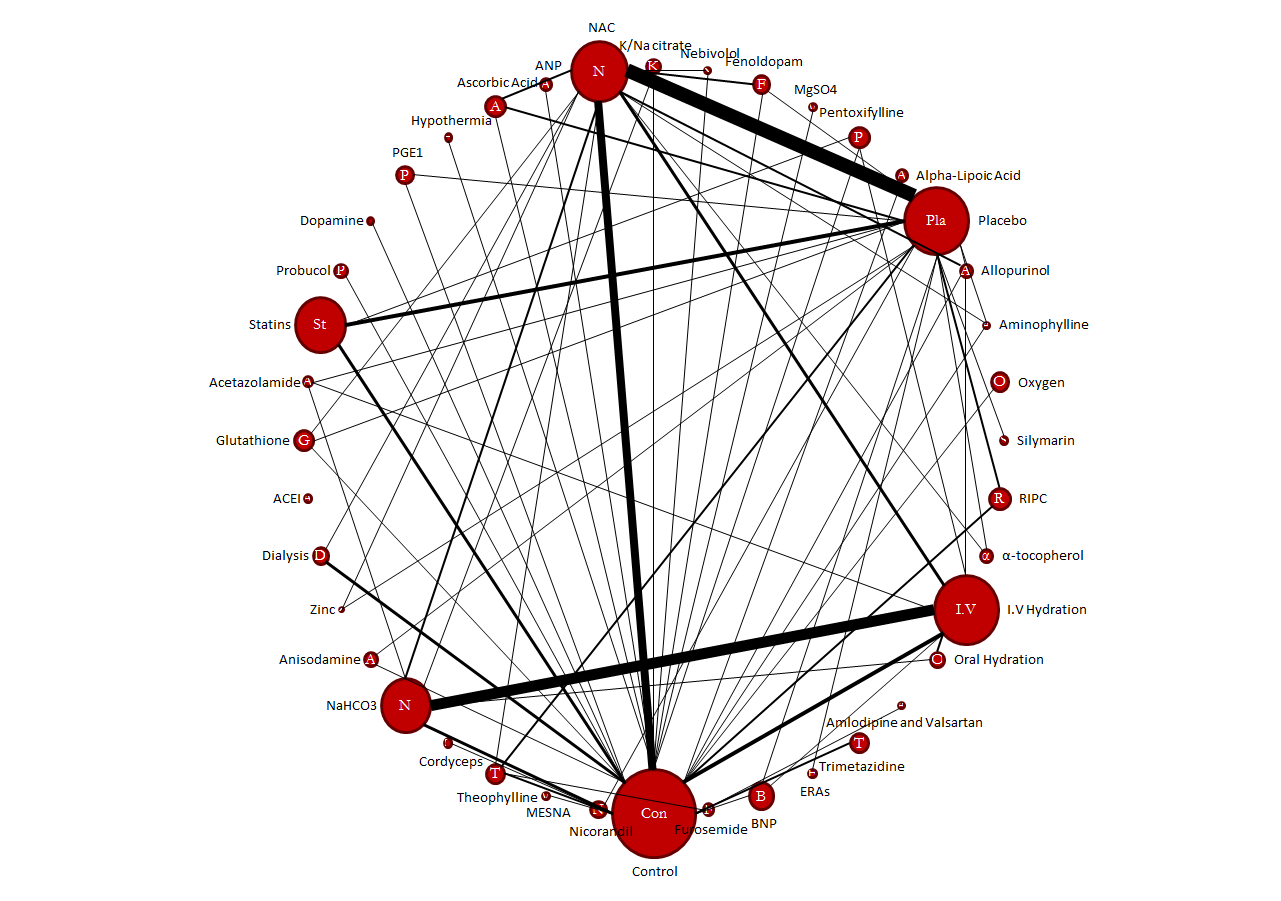


Figure 1 Network Diagram

Table 1Network Characteristics

| **Characteristic** | **Number** |
| --- | --- |
| **Number of Interventions** | 40 |
| **Number of Studies** | 173 |
| **Total Number of Patients in Network** | 34,549 |
| **Total Number of Events in Network** | 3,648 |
| **Total Possible Pairwise Comparisons** | 780 |
| **Total Number Pairwise Comparisons With Direct Data** | 72 |
| **Number of Two-arm Studies** | 154 |
| **Number of Multi-Arms Studies** | 19 |
| **Number of Studies With No Zero Events** | 159 |
| **Number of Studies With At Least One Zero Event** | 14 |
| **Number of Studies with All Zero Events** | 2 |

| **Treatment** | **# Studies** | **# Events** | **# Patients** | **Aggregate Rate** |
| --- | --- | --- | --- | --- |
| **I.V Hydration** | 38 | 513 | 4819 | 0.1065 |
| **Statins** | 14 | 123 | 3040 | 0.0405 |
| **Furosemide** | 2 | 16 | 166 | 0.0964 |
| **NAC** | 58 | 413 | 3674 | 0.1124 |
| **Trimetazidine** | 4 | 17 | 352 | 0.0483 |
| **NaHCO3** | 29 | 246 | 2938 | 0.0837 |
| **PGE1** | 4 | 24 | 304 | 0.0789 |
| **MgSO4** | 1 | 9 | 62 | 0.1452 |
| **Pentoxifylline** | 4 | 30 | 438 | 0.0685 |
| **Placebo** | 59 | 692 | 4797 | 0.1443 |
| **Control** | 77 | 1053 | 8136 | 0.1294 |
| **Allopurinol** | 4 | 5 | 204 | 0.0245 |
| **BNP** | 4 | 52 | 744 | 0.0699 |
| **Probucol** | 2 | 12 | 198 | 0.0606 |
| **α-tocopherol** | 2 | 10 | 159 | 0.0629 |
| **Oxygen** | 2 | 33 | 346 | 0.0954 |
| **Amlodipine and Valsartan** | 1 | 8 | 45 | 0.1778 |
| **K/Na citrate** | 2 | 6 | 203 | 0.0296 |
| **Nicorandil** | 3 | 15 | 291 | 0.0515 |
| **Ascorbic Acid** | 6 | 41 | 434 | 0.0945 |
| **Alpha-Lipoic Acid** | 2 | 6 | 139 | 0.0432 |
| **Oral Hydration** | 4 | 14 | 228 | 0.0614 |
| **Nebivolol** | 1 | 8 | 40 | 0.2000 |
| **Anisodamine** | 2 | 17 | 192 | 0.0885 |
| **RIPC** | 8 | 33 | 458 | 0.0721 |
| **Theophylline** | 7 | 21 | 384 | 0.0547 |
| **Hypothermia** | 1 | 14 | 58 | 0.2414 |
| **Glutathione** | 2 | 21 | 421 | 0.0499 |
| **MESNA** | 1 |  | 51 | 0.0000 |
| **ACEI** | 1 | 3 | 52 | 0.0577 |
| **Aminophylline** | 2 | 4 | 45 | 0.0889 |
| **Acetazolamide** | 1 | 5 | 94 | 0.0532 |
| **ANP** | 1 | 4 | 126 | 0.0317 |
| **Zinc** | 1 | 3 | 18 | 0.1667 |
| **Dialysis** | 5 | 43 | 293 | 0.1468 |
| **Fenoldopam** | 5 | 78 | 333 | 0.2342 |
| **ERAs** | 1 | 43 | 77 | 0.5584 |
| **Dopamine** | 1 | 4 | 33 | 0.1212 |
| **Cordyceps** | 2 | 7 | 88 | 0.0795 |
| **Silymarin** | 1 | 2 | 69 | 0.0290 |

Table 2 Interventions Characteristics

Table 3 Direct comparisons characteristics

| **Comparison** | **# Studies** | **# Patients** | **# Events** |
| --- | --- | --- | --- |
| **Statins vs. Control** | 6 | 4,382 | 200 |
| **NaHCO3 vs. K/Na citrate** | 1 | 206 | 4 |
| **I.V Hydartion vs. NaHCO3** | 23 | 5,113 | 471 |
| **I.V Hydartion vs. Oral Hydration** | 4 | 456 | 33 |
| **NAC vs. Placebo** | 31 | 4,408 | 593 |
| **NAC vs. Ascorbic Acid** | 3 | 583 | 88 |
| **Placebo vs. Ascorbic Acid** | 4 | 638 | 105 |
| **NAC vs. Control** | 18 | 2,006 | 240 |
| **NAC vs. Fenoldopam** | 3 | 359 | 44 |
| **Control vs. Fenoldopam** | 2 | 123 | 26 |
| **I.V Hydartion vs. Control** | 8 | 2,884 | 396 |
| **NAC vs. Theophylline** | 1 | 62 | 13 |
| **Statins vs. Placebo** | 7 | 1,508 | 125 |
| **Placebo vs. Theophylline** | 3 | 224 | 21 |
| **NaHCO3 vs. Oral Hydration** | 1 | 43 | 3 |
| **Control vs. Alpha-Lipoic Acid** | 2 | 280 | 16 |
| **Furosemide vs. Control** | 2 | 326 | 35 |
| **Furosemide vs. Theophylline** | 1 | 159 | 18 |
| **Control vs. Theophylline** | 3 | 493 | 27 |
| **Control vs. RIPC** | 4 | 486 | 55 |
| **Control vs. Allopurinol** | 1 | 159 | 6 |
| **Pentoxifylline vs. Control** | 2 | 461 | 46 |
| **Placebo vs. Nicorandil** | 1 | 240 | 29 |
| **MgSO4 vs. Control** | 1 | 126 | 26 |
| **Control vs. Dopamine** | 1 | 66 | 6 |
| **Control vs. Anisodamine** | 1 | 260 | 39 |
| **NAC vs. Nebivolol** | 1 | 80 | 17 |
| **Control vs. Nebivolol** | 1 | 80 | 19 |
| **NAC vs. NaHCO3** | 3 | 252 | 58 |
| **Placebo vs. RIPC** | 4 | 427 | 43 |
| **Control vs. Cordyceps** | 2 | 180 | 19 |
| **I.V Hydartion vs. NAC** | 5 | 476 | 89 |
| **Statins vs. Pentoxifylline** | 1 | 220 | 9 |
| **NAC vs. Zinc** | 1 | 37 | 4 |
| **Placebo vs. Zinc** | 1 | 35 | 5 |
| **NAC vs. Aminophylline** | 1 | 30 | 0 |
| **Control vs. Aminophylline** | 1 | 30 | 4 |
| **NAC vs. α-tocopherol** | 1 | 20 | 0 |
| **Placebo vs. α-tocopherol** | 2 | 318 | 31 |
| **Control vs. Nicorandil** | 2 | 341 | 22 |
| **PGE1 vs. Placebo** | 2 | 392 | 74 |
| **NaHCO3 vs. Control** | 5 | 806 | 95 |
| **I.V Hydartion vs. Allopurinol** | 2 | 185 | 31 |
| **NAC vs. Allopurinol** | 3 | 215 | 30 |
| **Control vs. Dialysis** | 5 | 588 | 112 |
| **Control vs. Probucol** | 2 | 409 | 50 |
| **Placebo vs. ACEI** | 1 | 114 | 9 |
| **PGE1 vs. Control** | 2 | 226 | 16 |
| **Control vs. BNP** | 1 | 209 | 23 |
| **I.V Hydartion vs. BNP** | 2 | 1,128 | 113 |
| **Trimetazidine vs. Control** | 4 | 714 | 71 |
| **Control vs. MESNA** | 1 | 100 | 7 |
| **Control vs. K/Na citrate** | 1 | 202 | 25 |
| **Control vs. Oxygen** | 2 | 697 | 115 |
| **Control vs. ANP** | 1 | 254 | 19 |
| **Control vs. Amlodipine and Valsartan** | 1 | 90 | 11 |
| **I.V Hydartion vs. Acetazolamide** | 1 | 190 | 21 |
| **NaHCO3 vs. Acetazolamide** | 1 | 190 | 9 |
| **NAC vs. Dialysis** | 1 | 275 | 11 |
| **Placebo vs. Aminophylline** | 1 | 60 | 10 |
| **Placebo vs. Allopurinol** | 1 | 60 | 16 |
| **NAC vs. Glutathione** | 1 | 14 | 1 |
| **Control vs. Glutathione** | 1 | 14 | 1 |
| **Placebo vs. Silymarin** | 1 | 143 | 10 |
| **Placebo vs. Fenoldopam** | 1 | 283 | 90 |
| **Control vs. Hypothermia** | 1 | 128 | 29 |
| **Placebo vs. ERAs** | 1 | 158 | 67 |
| **Placebo vs. Glutathione** | 1 | 825 | 41 |
| **Placebo vs. Anisodamine** | 1 | 126 | 17 |
| **I.V Hydartion vs. Pentoxifylline** | 1 | 199 | 12 |
| **Placebo vs. BNP** | 1 | 149 | 36 |
| **Control vs. Ascorbic Acid** | 1 | 156 | 10 |

Figure 2 Rankogram: ranking the interventions for the probability of being the best, the interventions are colour coded; the first column represent the chance of being first best and 2nd column is the chance of being 2nd best and so on. The overall numerical numerical value is presented in table 4


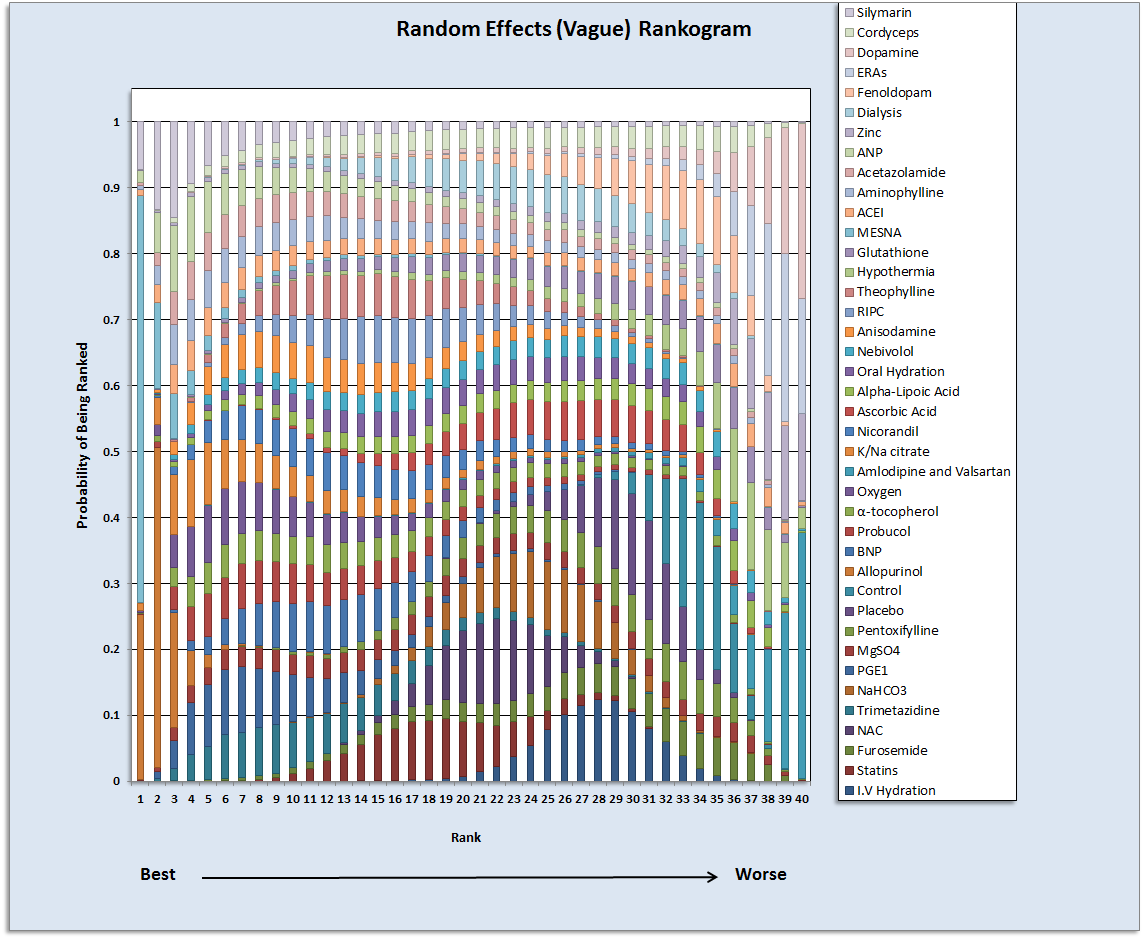


Table 4Interventions ranking the treatments names column follow the league table (which arranges the presentation of summary estimates by ranking the treatments in order of most pronounced impact on the outcome under consideration) the numerical values represents the cumulative results of the probability of being best in which the highest score is 1 or 100% (see Rankogram)

| **Treatment** | **SUCRA** | **Treatment** | **SUCRA** |
| --- | --- | --- | --- |
| **Allopurinol** | 0.9701 | **NaHCO3** | 0.4022 |
| **MESNA** | 0.946 | **Ascorbic Acid** | 0.3749 |
| **PGE1** | 0.7847 | **Glutathione** | 0.347 |
| **K/Na citrate** | 0.7634 | **Furosemide** | 0.3375 |
| **Oxygen** | 0.7568 | **Pentoxifylline** | 0.3338 |
| **Trimetazidine** | 0.7169 | **I.V Hydration** | 0.3115 |
| **Probucol** | 0.7079 | **Placebo** | 0.2593 |
| **BNP** | 0.6868 | **Hypothermia** | 0.1994 |
| **Anisodamine** | 0.6599 | **Control** | 0.1629 |
| **Nicorandil** | 0.648 | **Amlodipine and Valsartan** | 0.05976 |
| **Theophylline** | 0.6235 | **Aminophylline** | 0.6552 |
| **α-tocopherol** | 0.6192 | **Acetazolamide** | 0.6443 |
| **RIPC** | 0.5952 | **ANP** | 0.7384 |
| **Statins** | 0.5462 | **Zinc** | 0.1942 |
| **ACEI** | 0.5383 | **Dialysis** | 0.4266 |
| **MgSO4** | 0.5157 | **Fenoldopam** | 0.2277 |
| **Oral Hydration** | 0.4835 | **ERAs** | 0.06707 |
| **NAC** | 0.464 | **Dopamine** | 0.1218 |
| **Nebivolol** | 0.4534 | **Cordyceps** | 0.445 |
| **Alpha-Lipoic Acid** | 0.4226 | **Silymarin** | 0.7894 |
| ***Analysis*** | **Random Effects (Vague)** | | |

Figure 3 Forest Plot


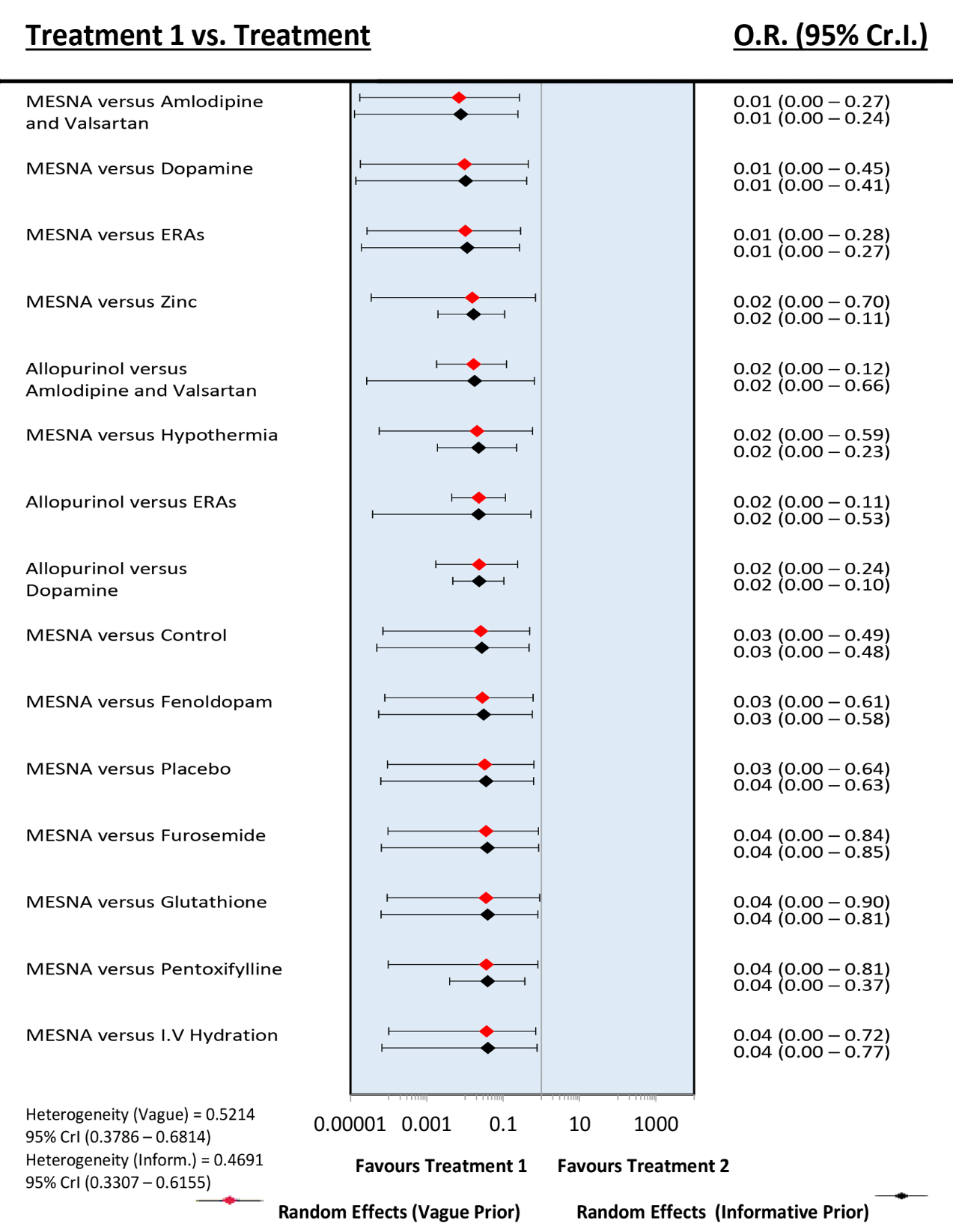


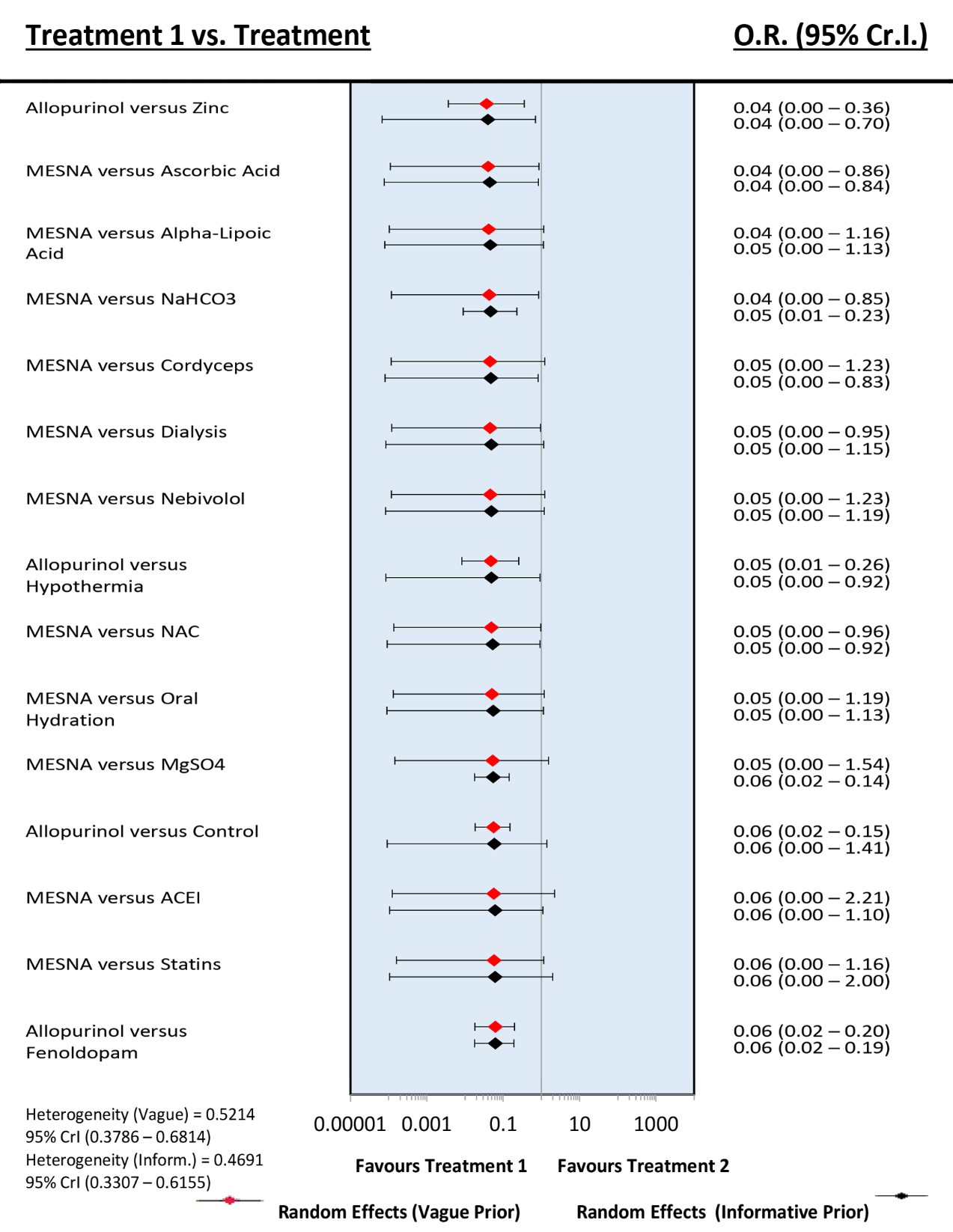


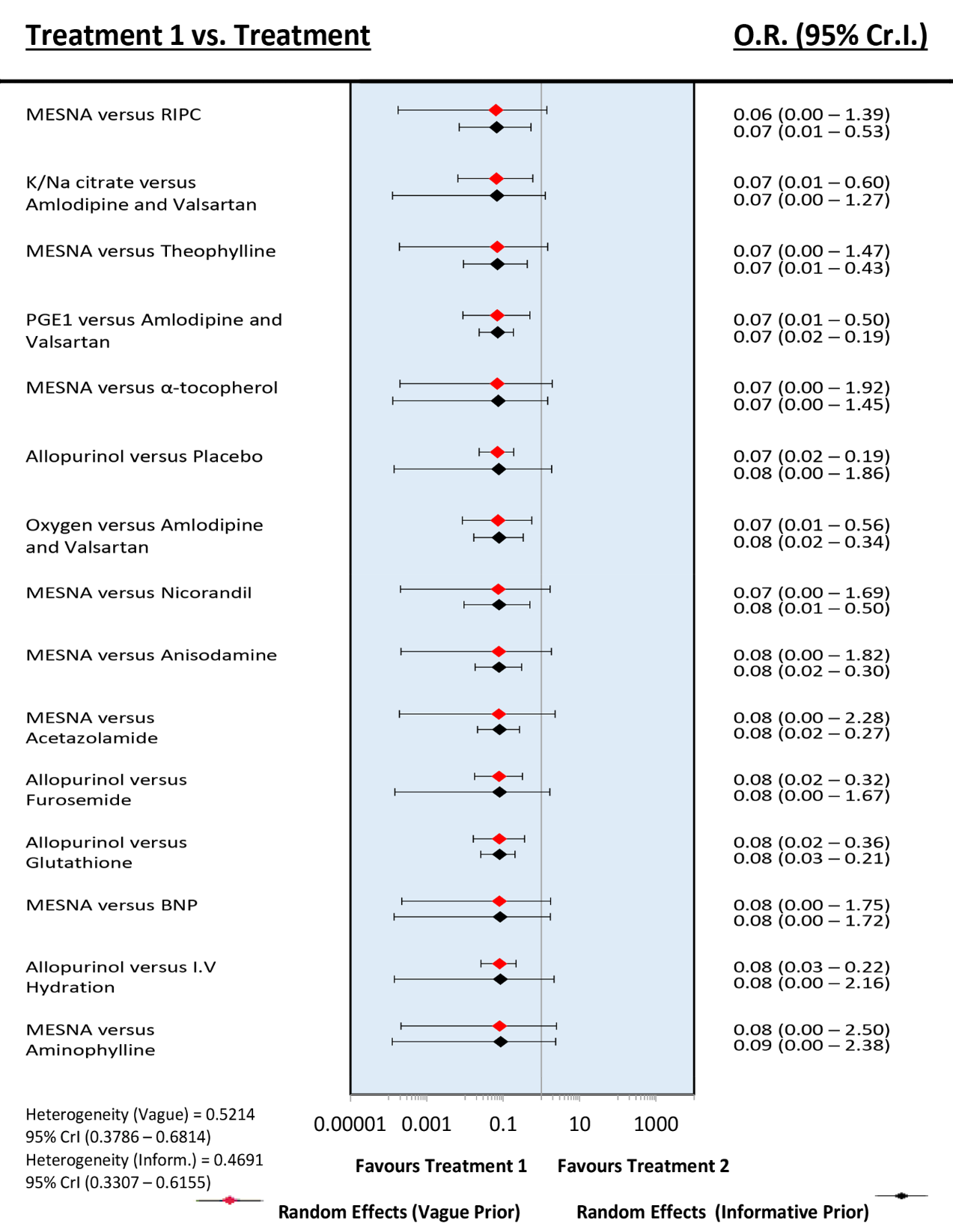


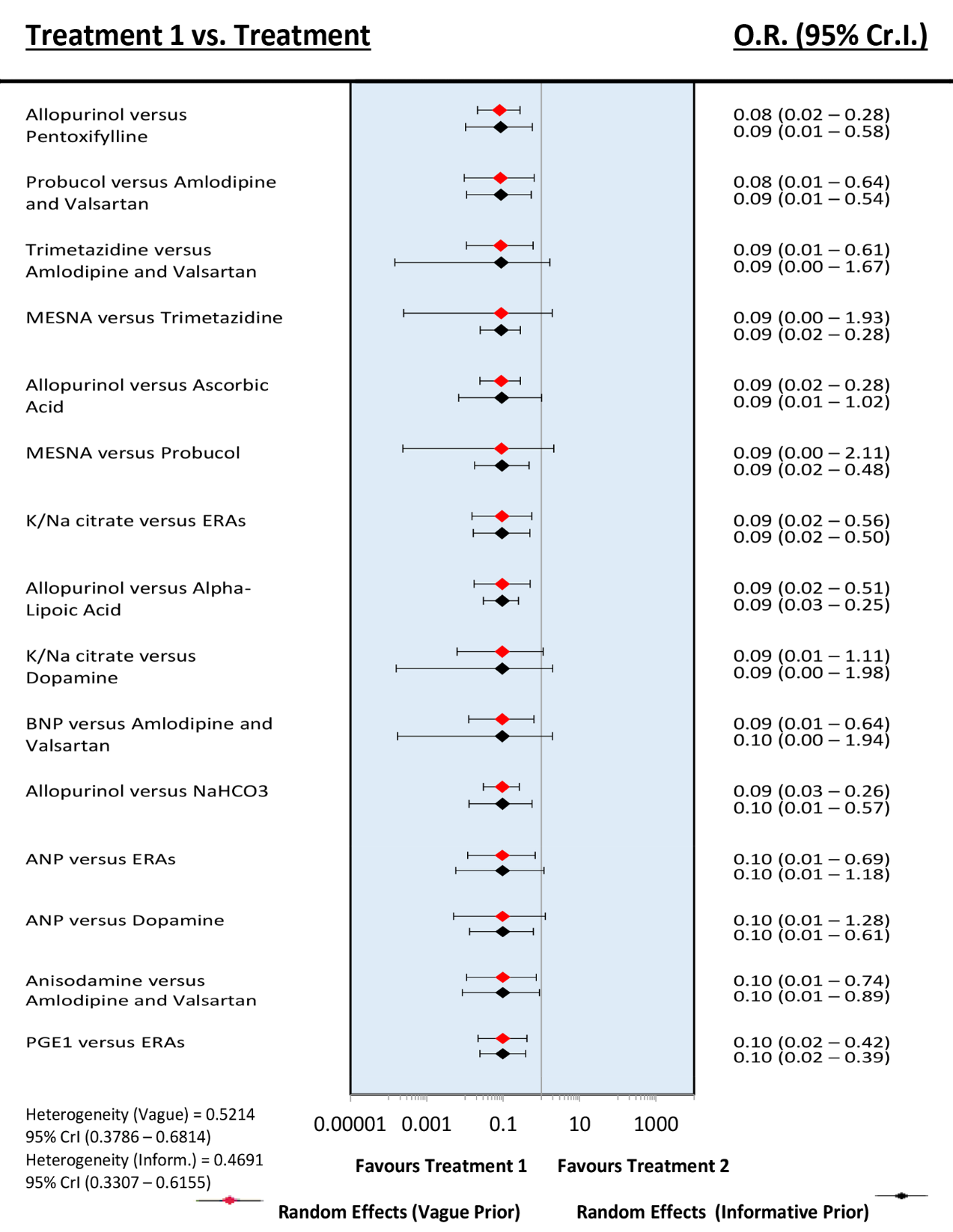


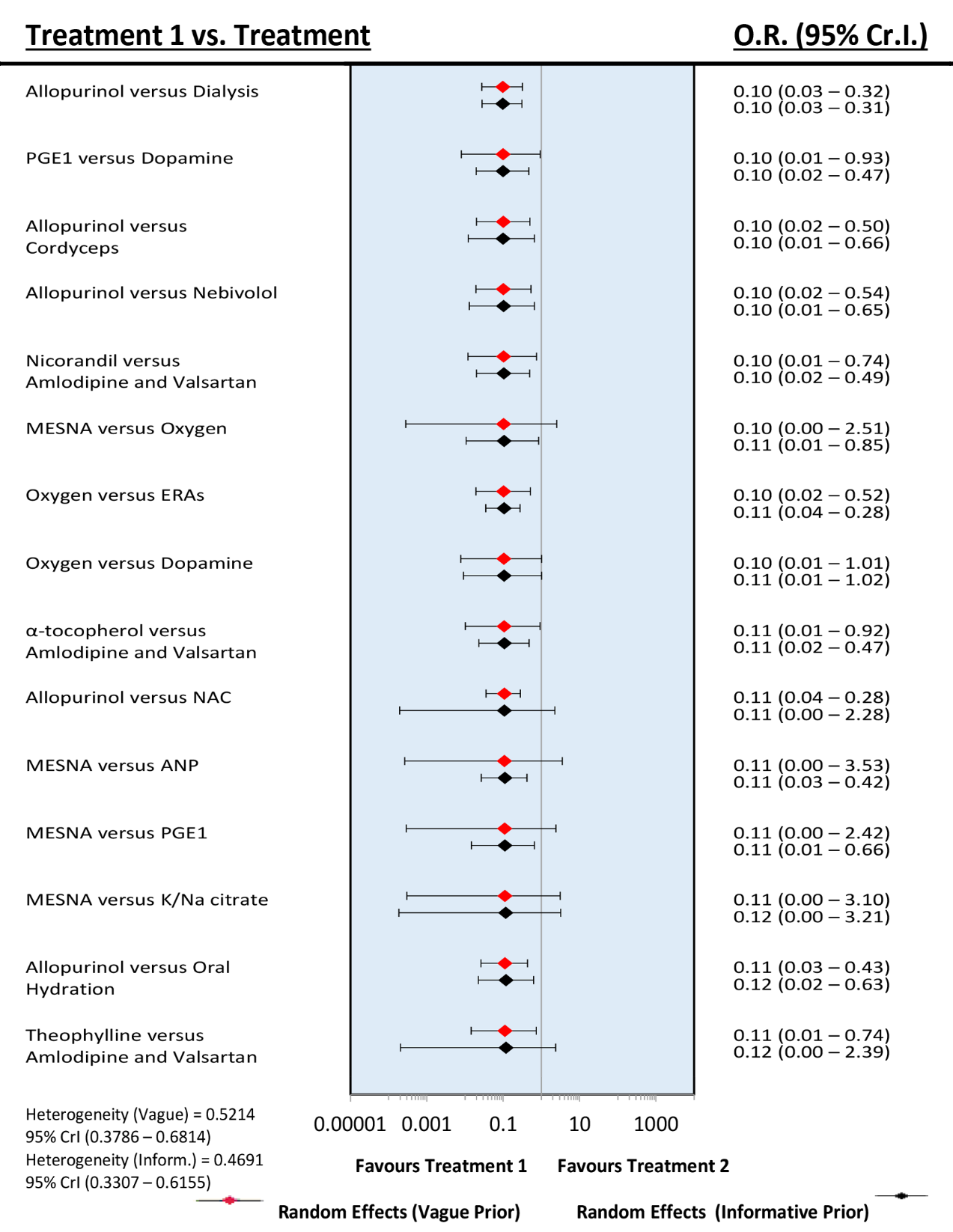


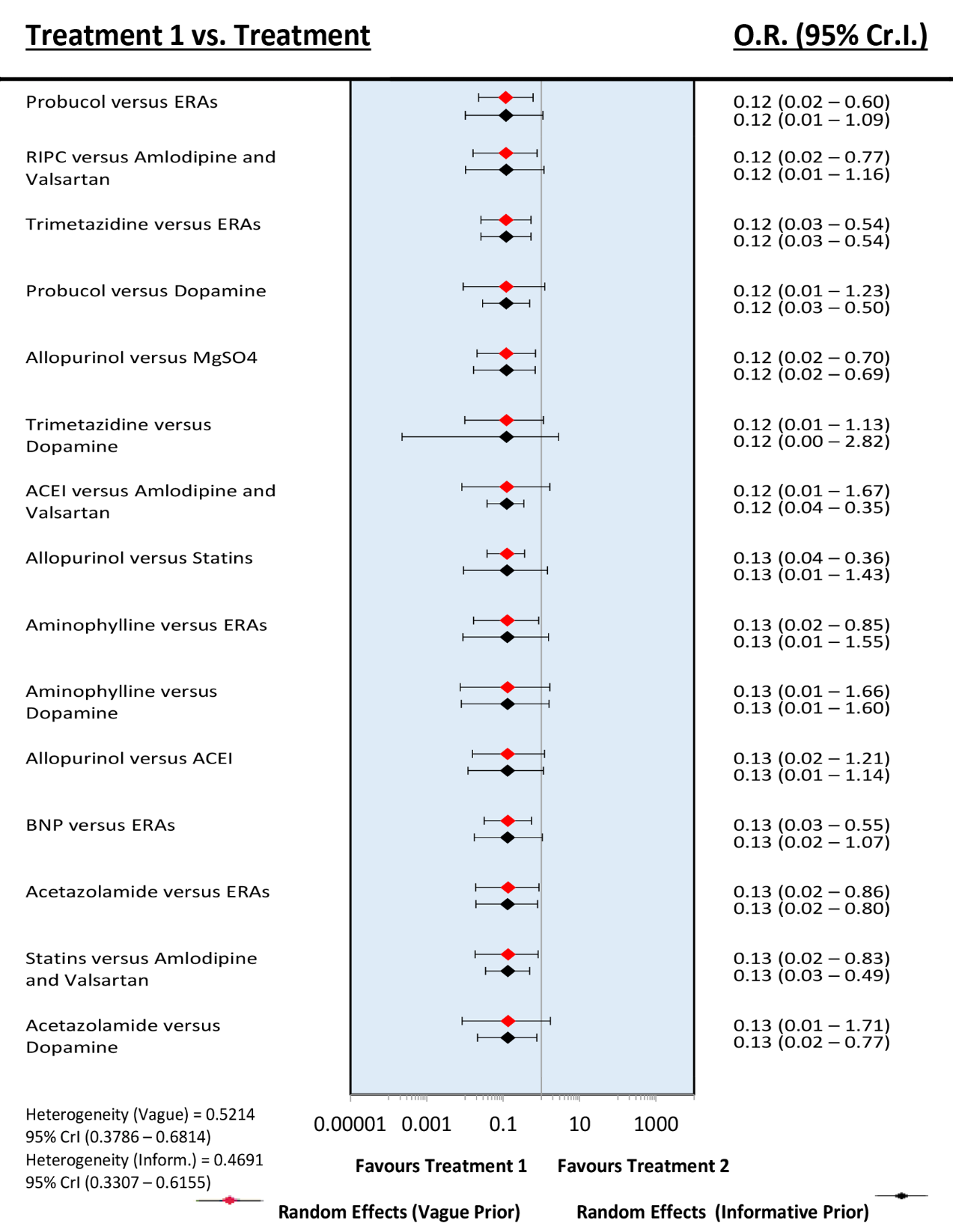


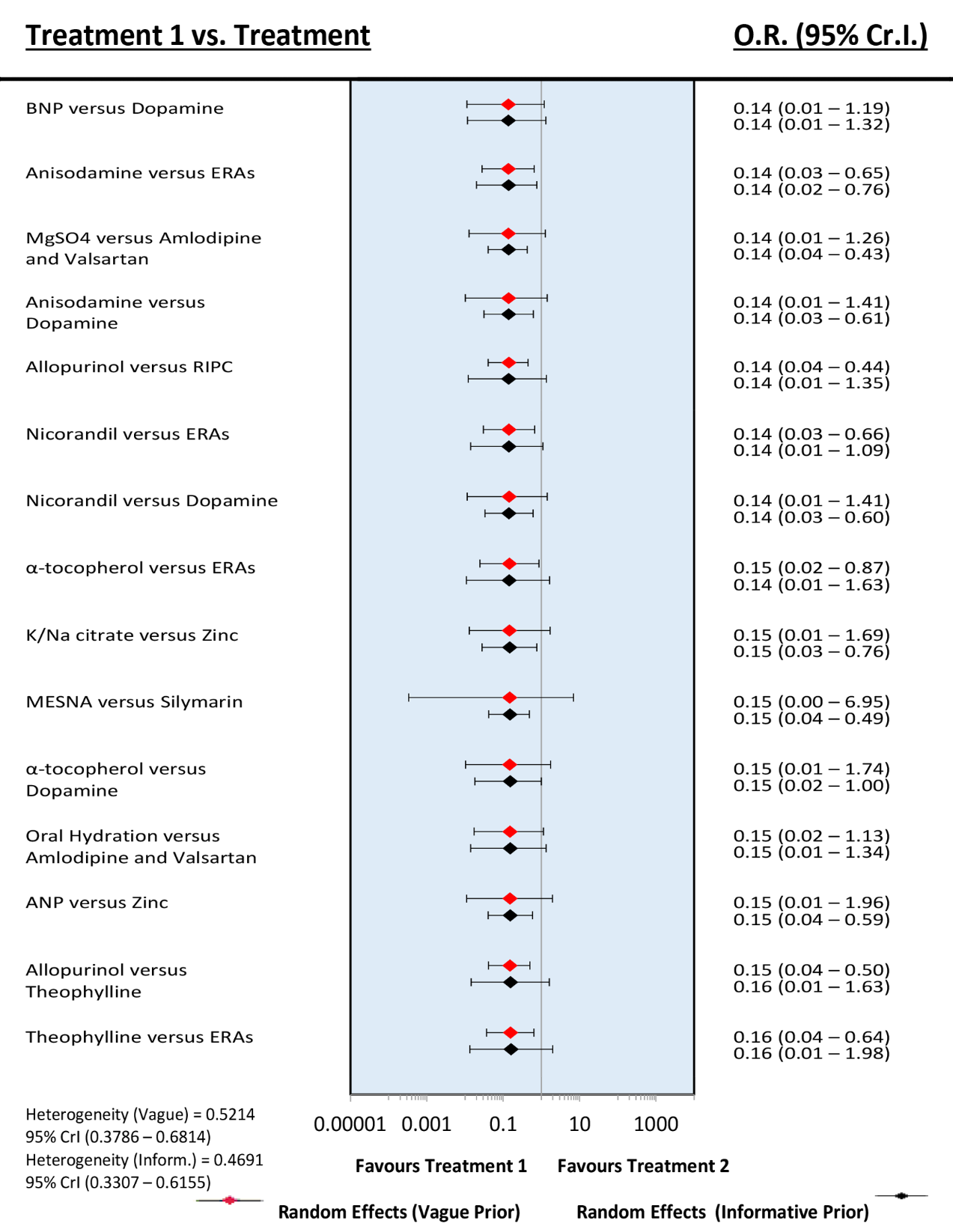


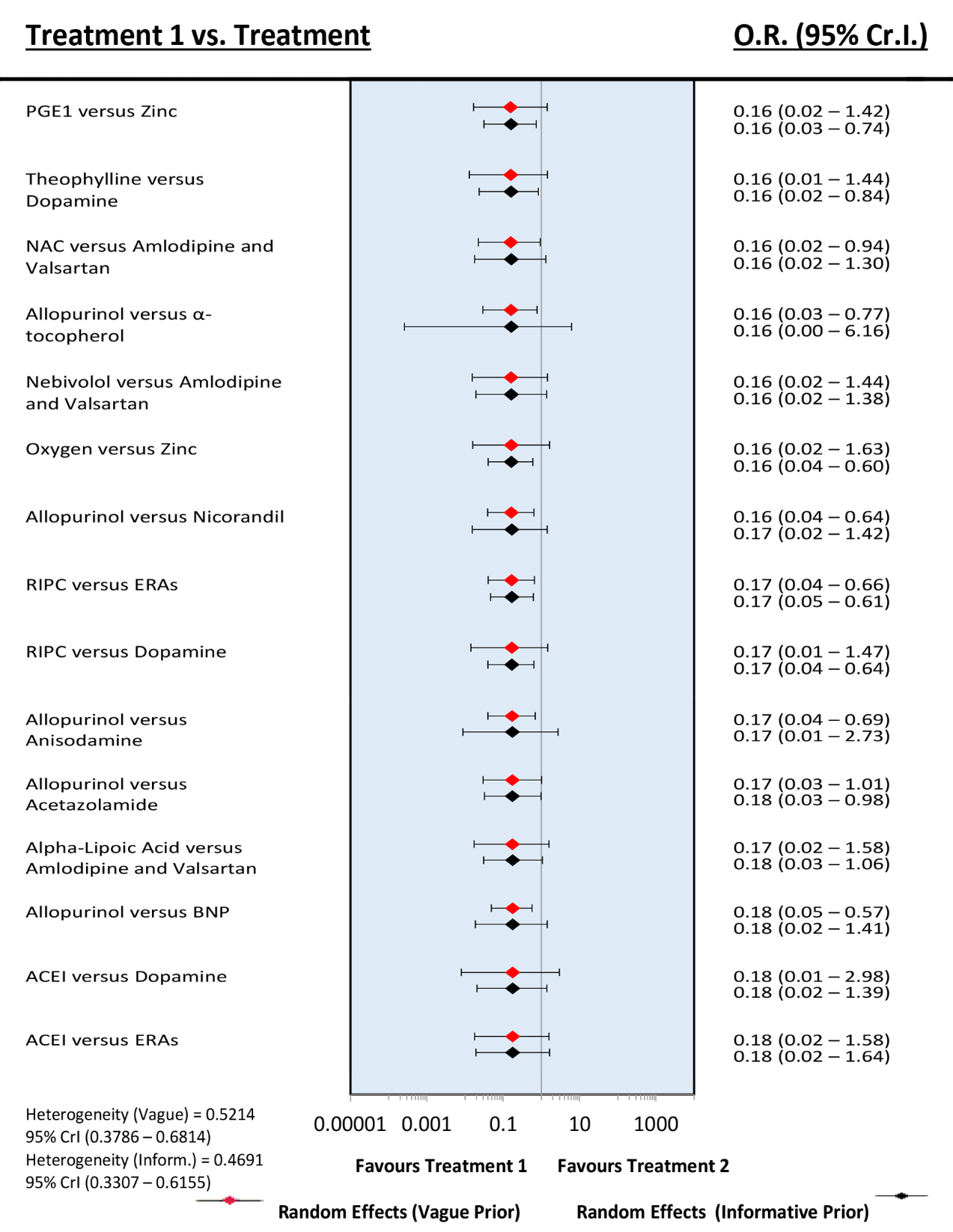


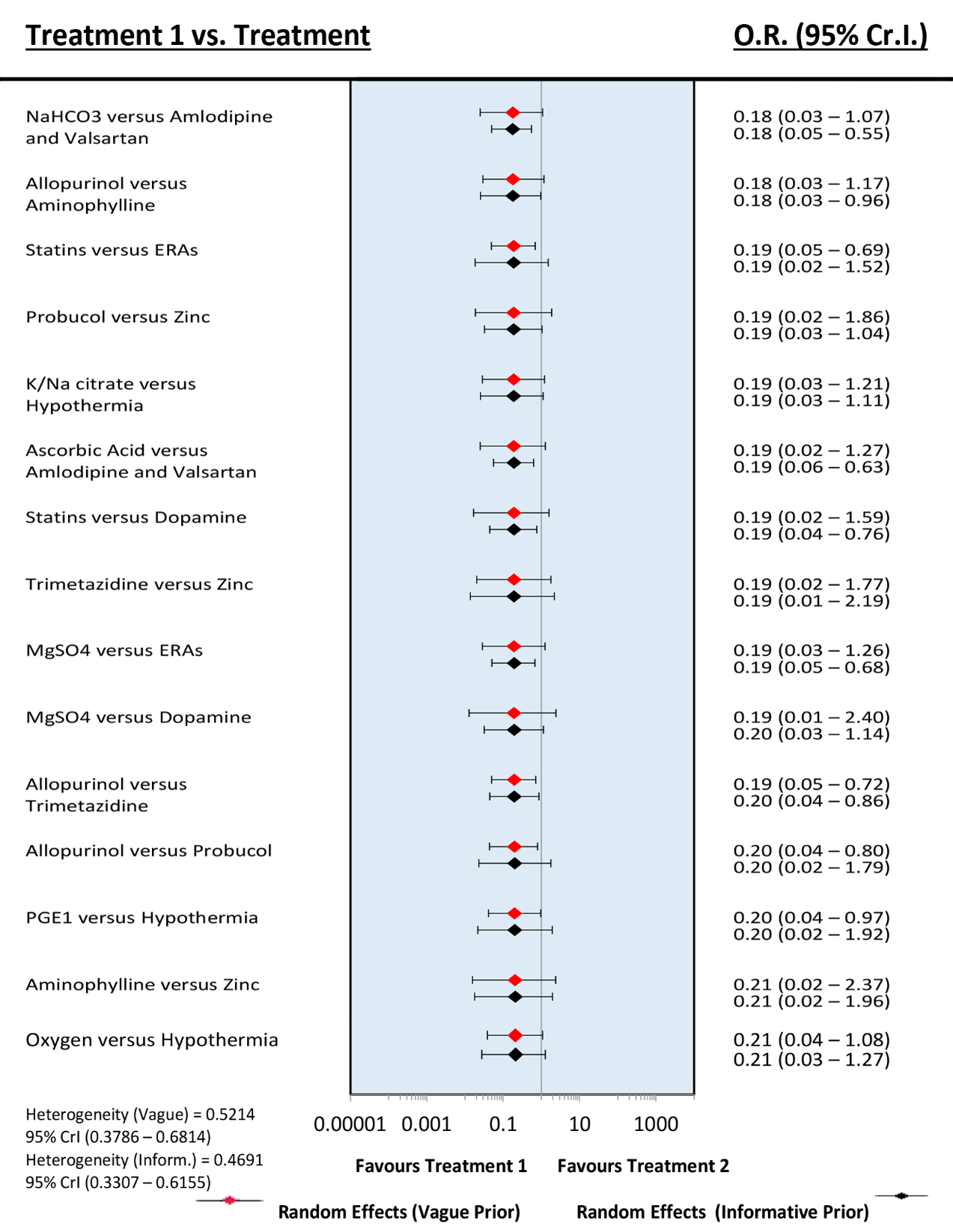


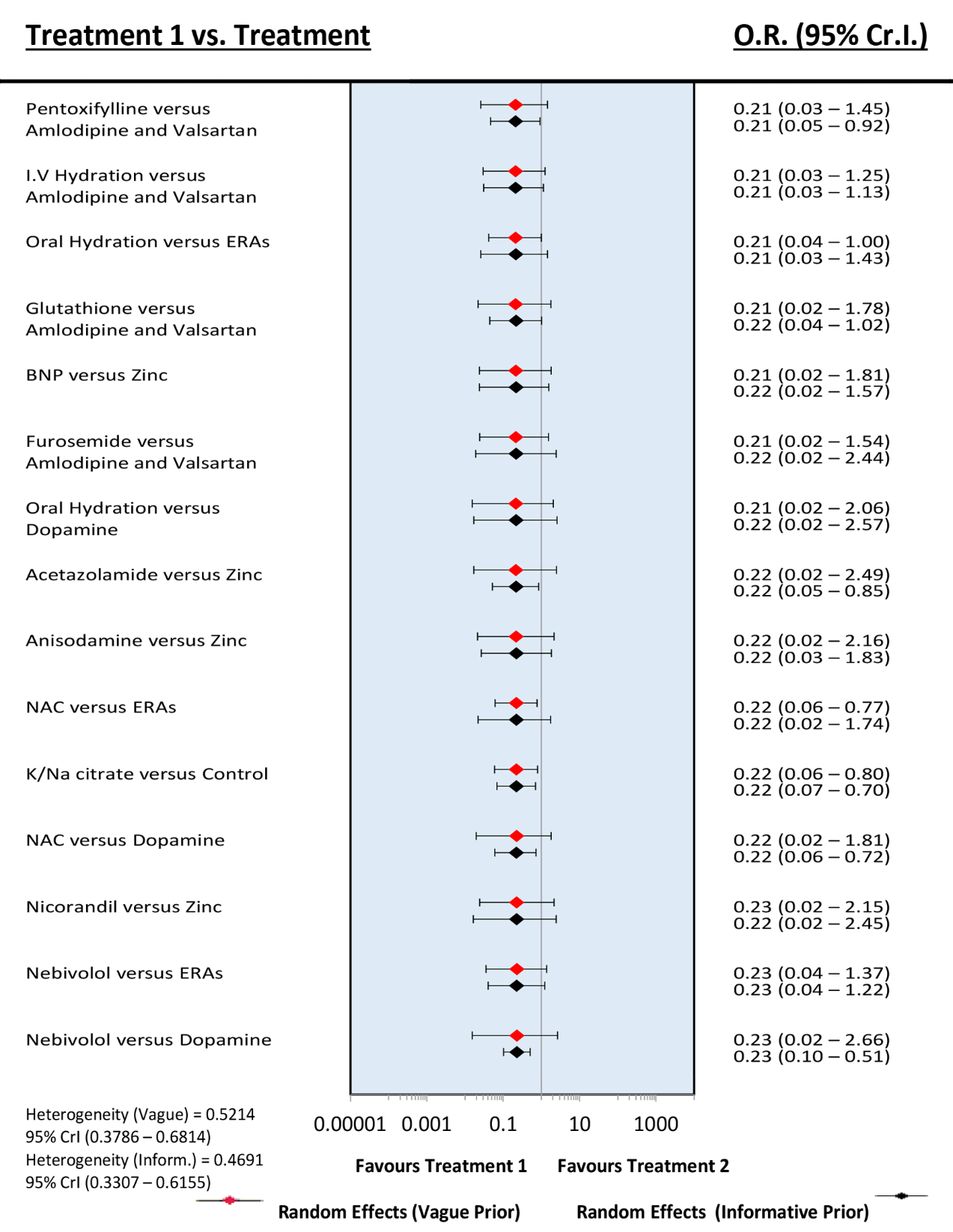


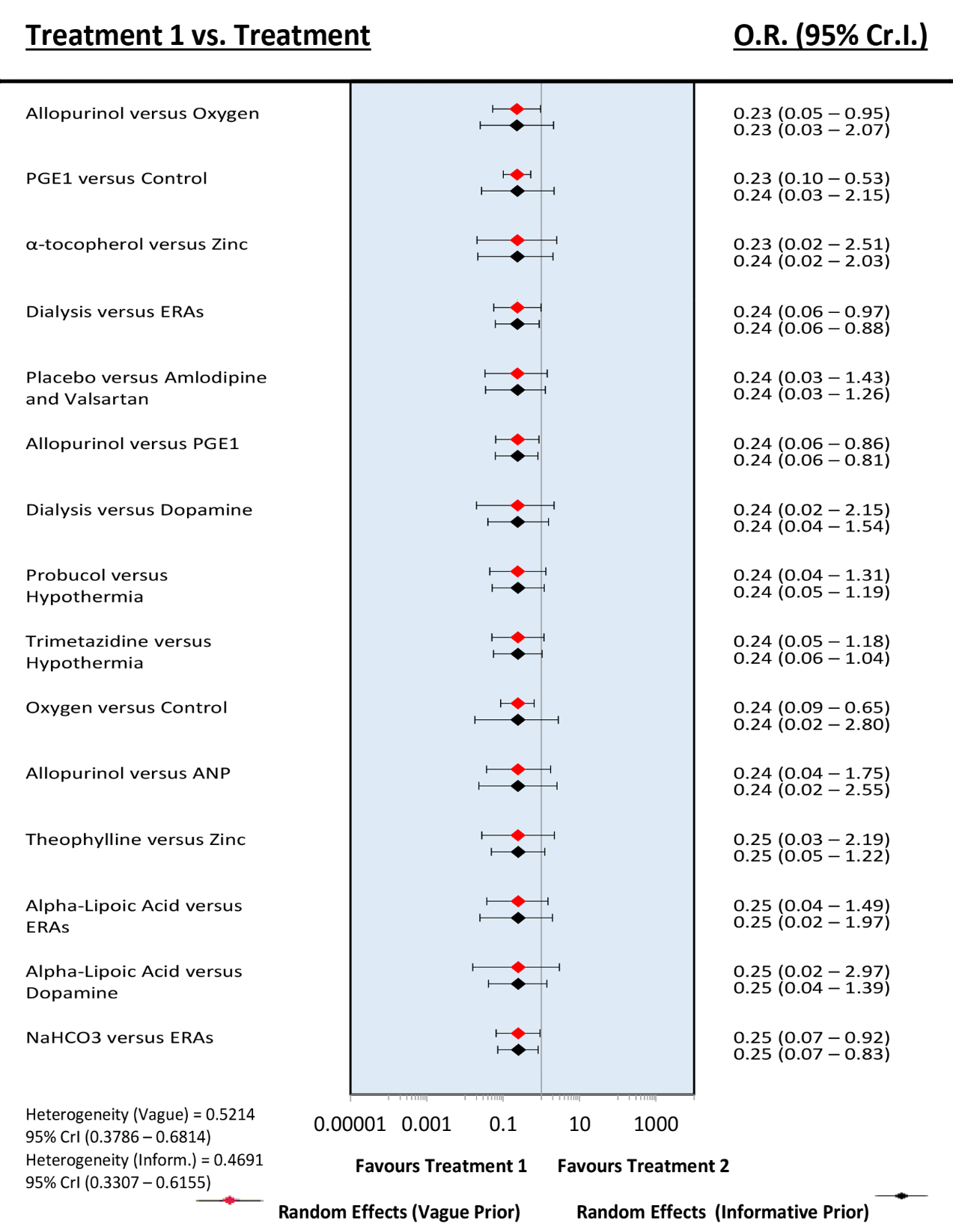


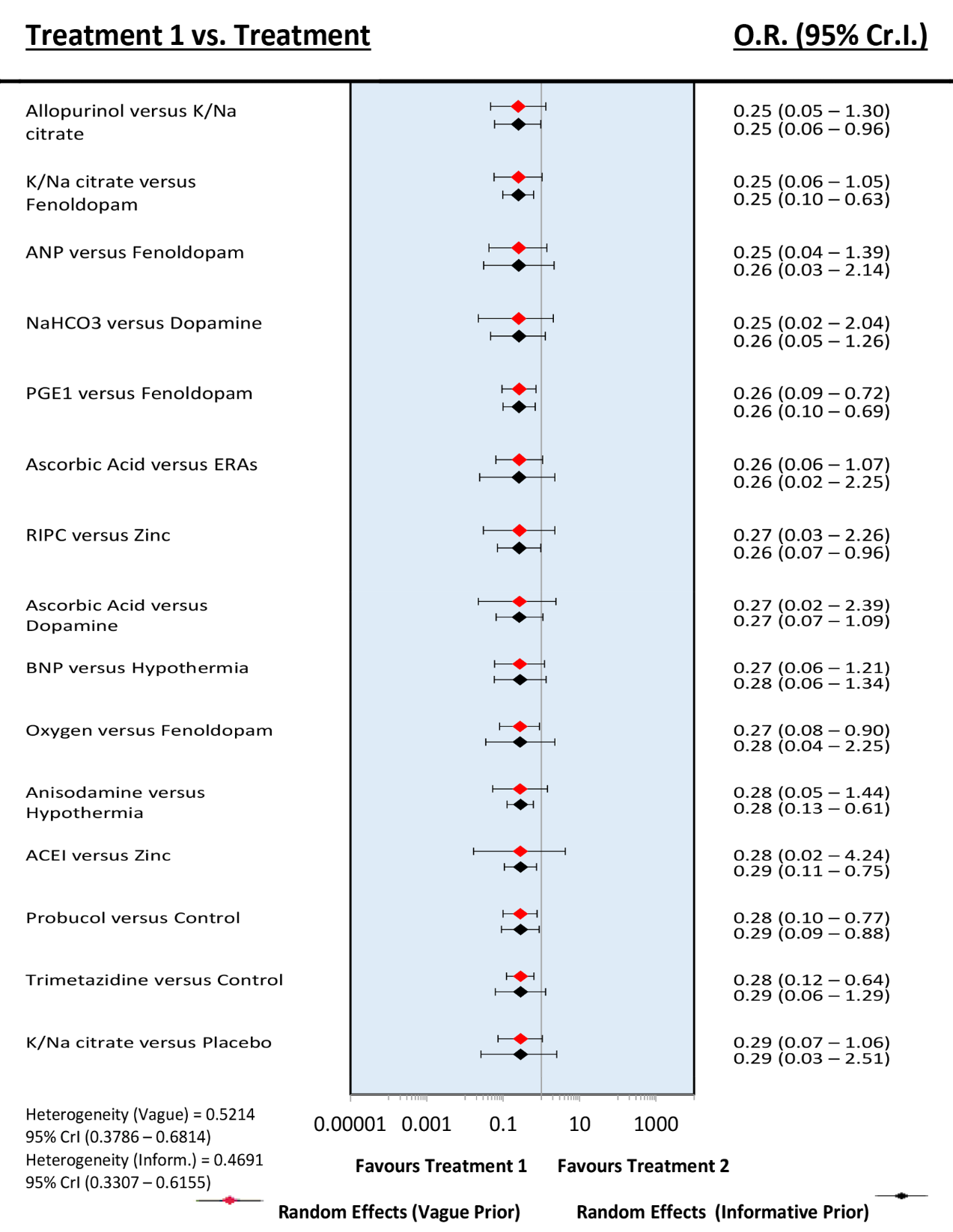


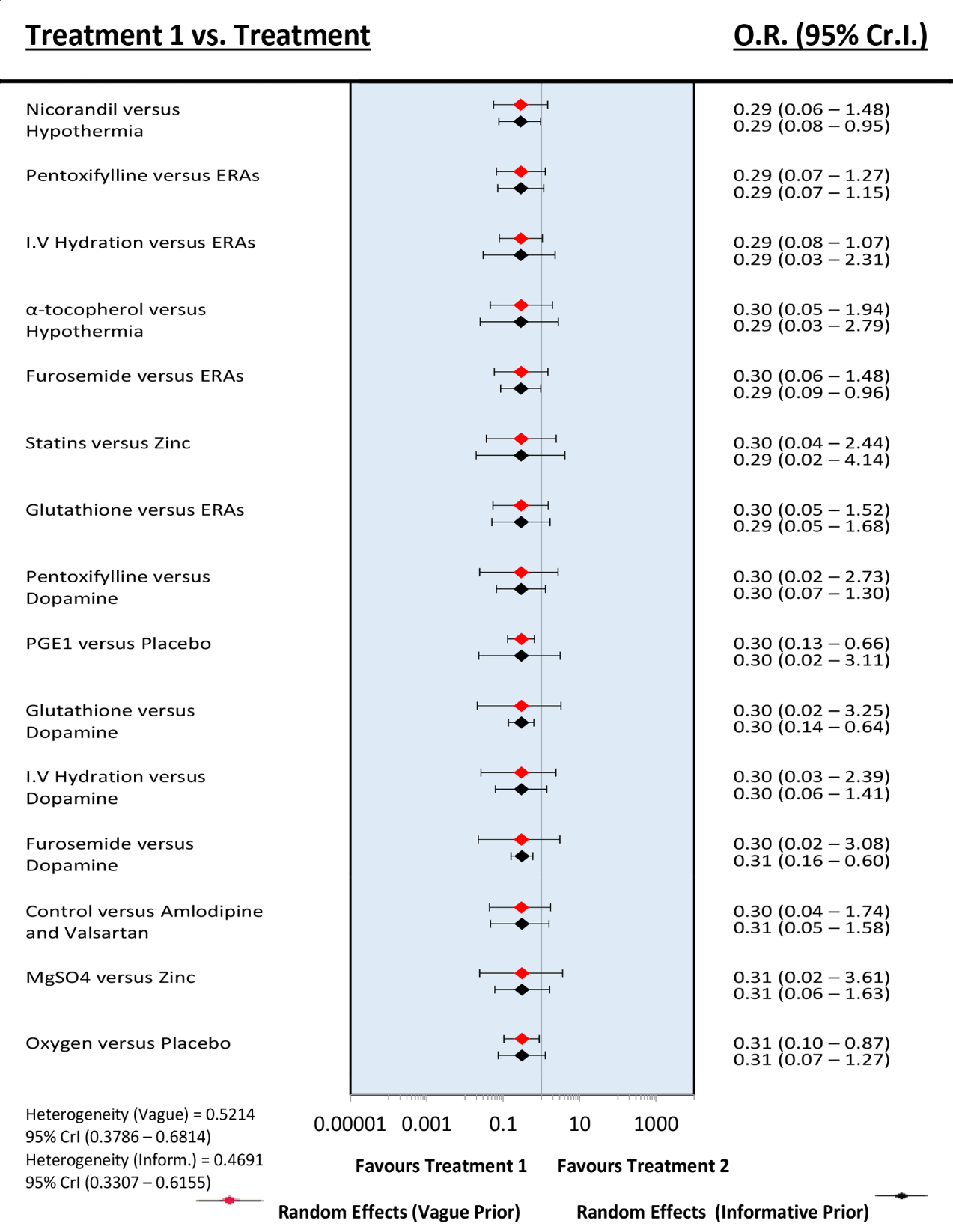


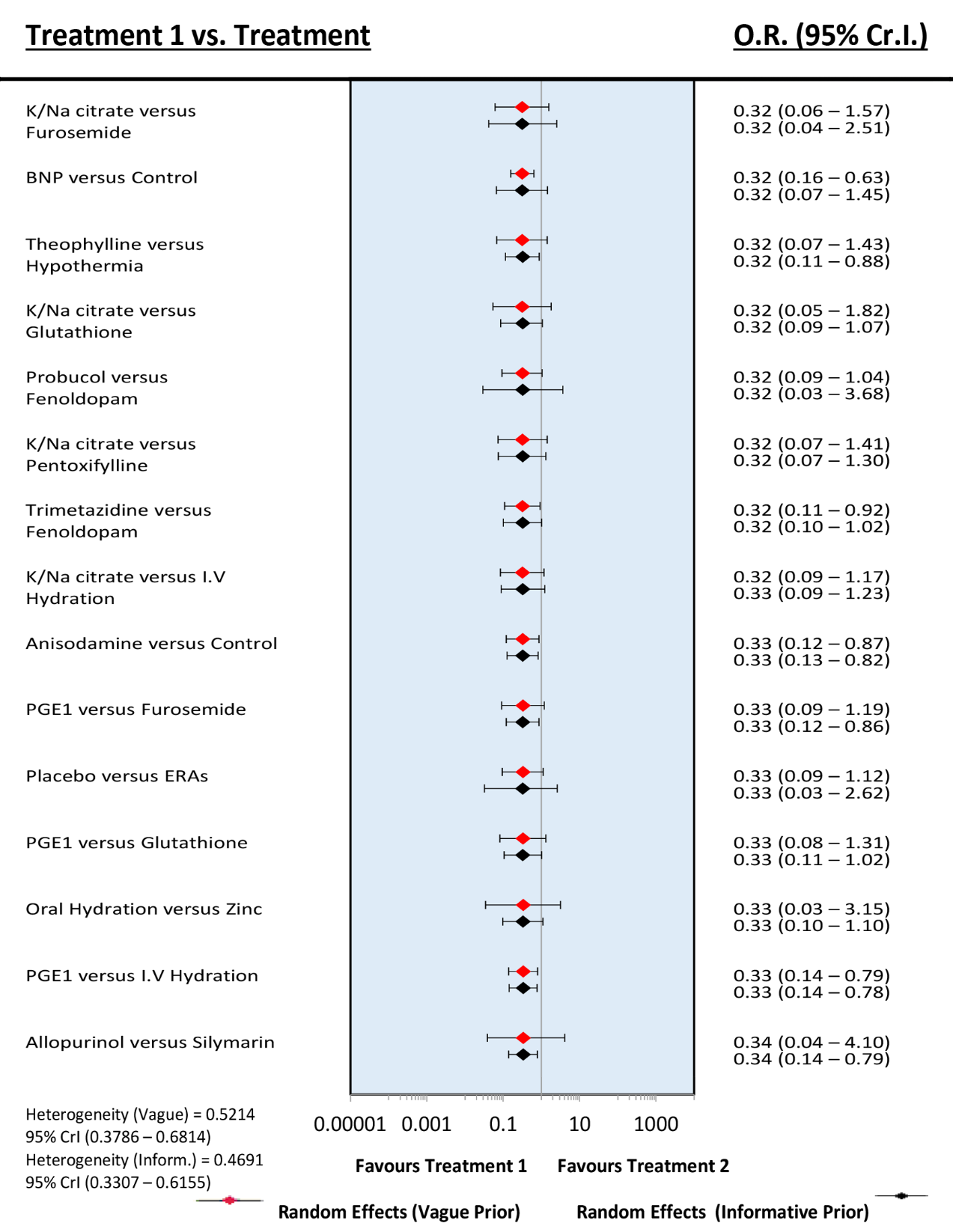


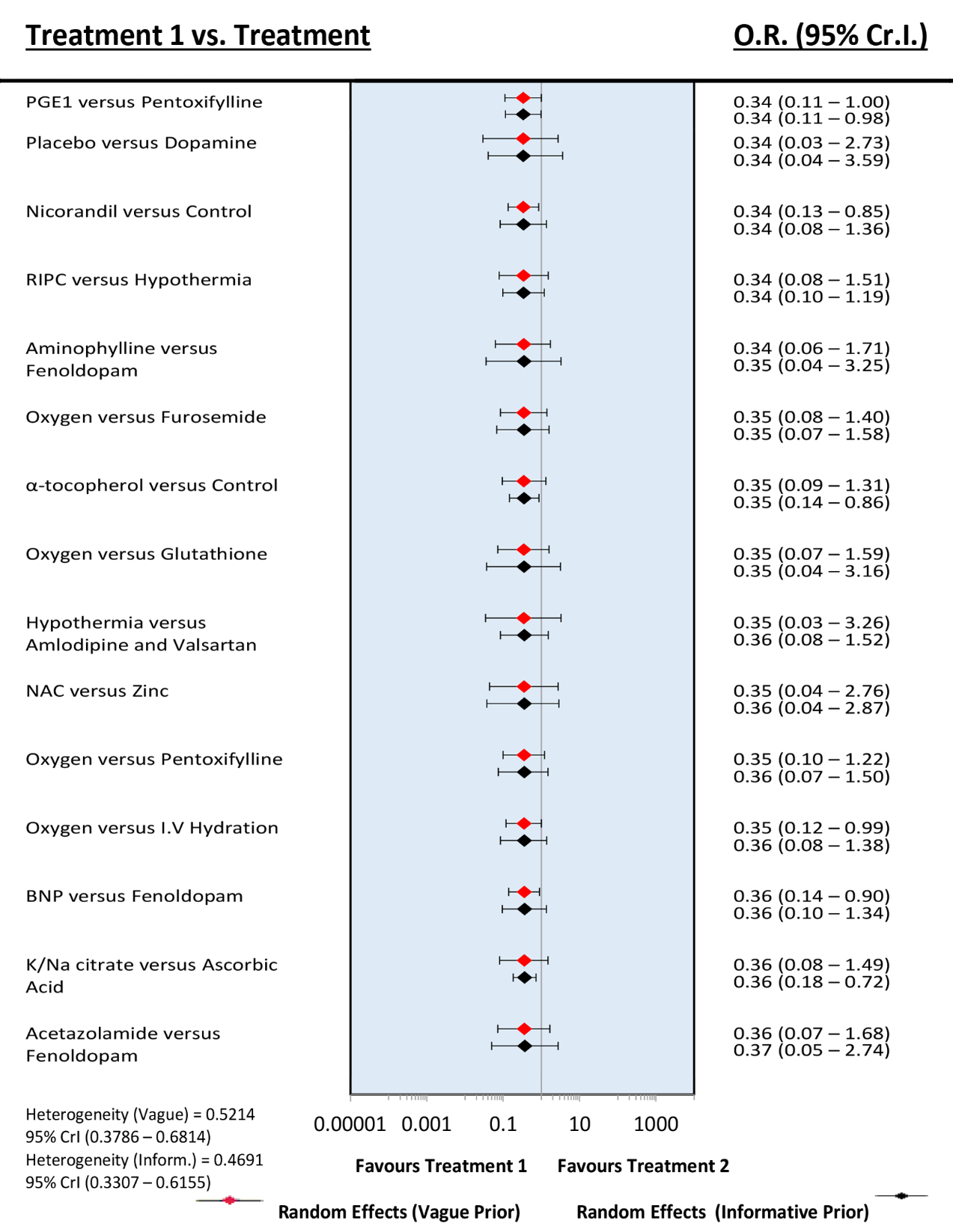


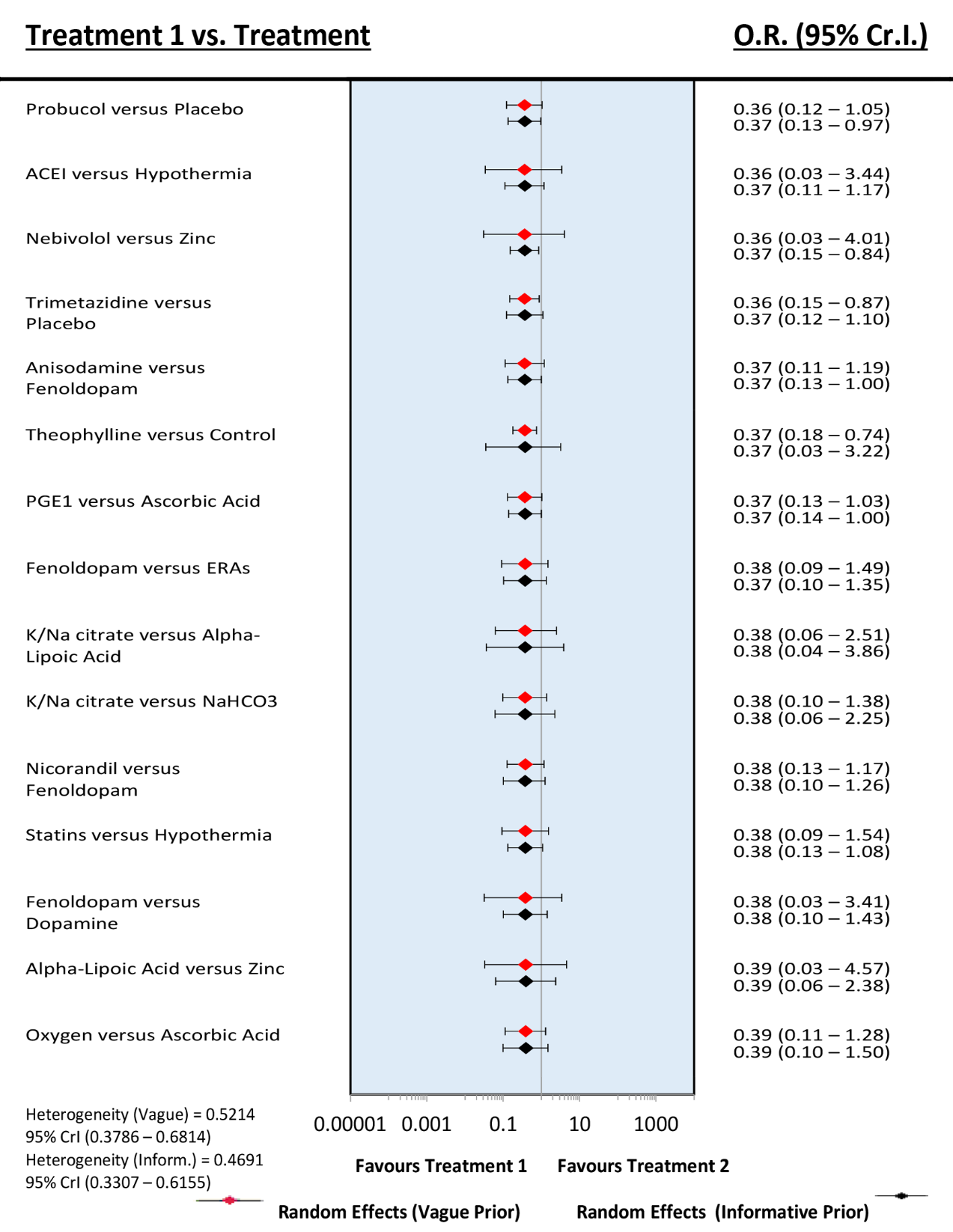


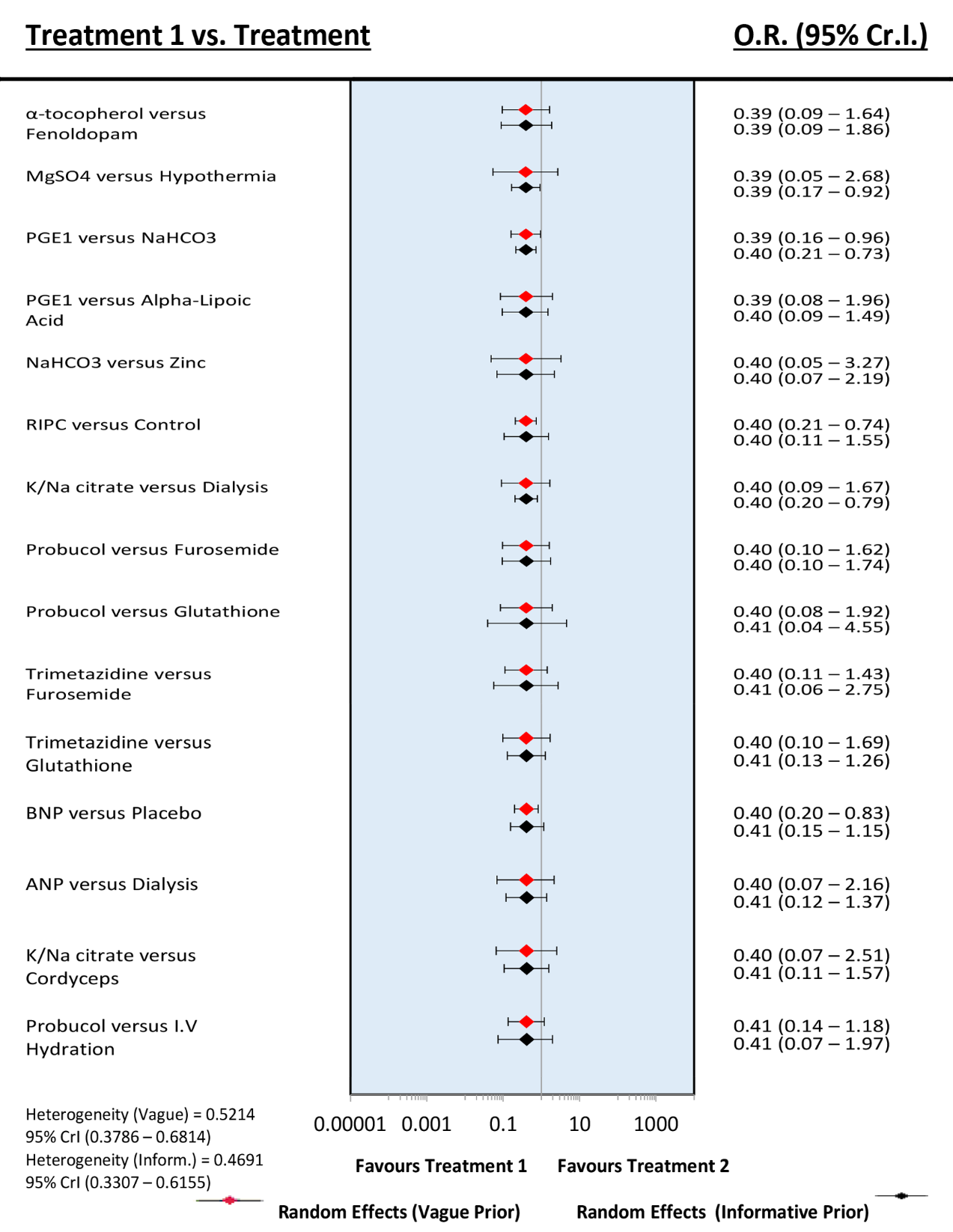


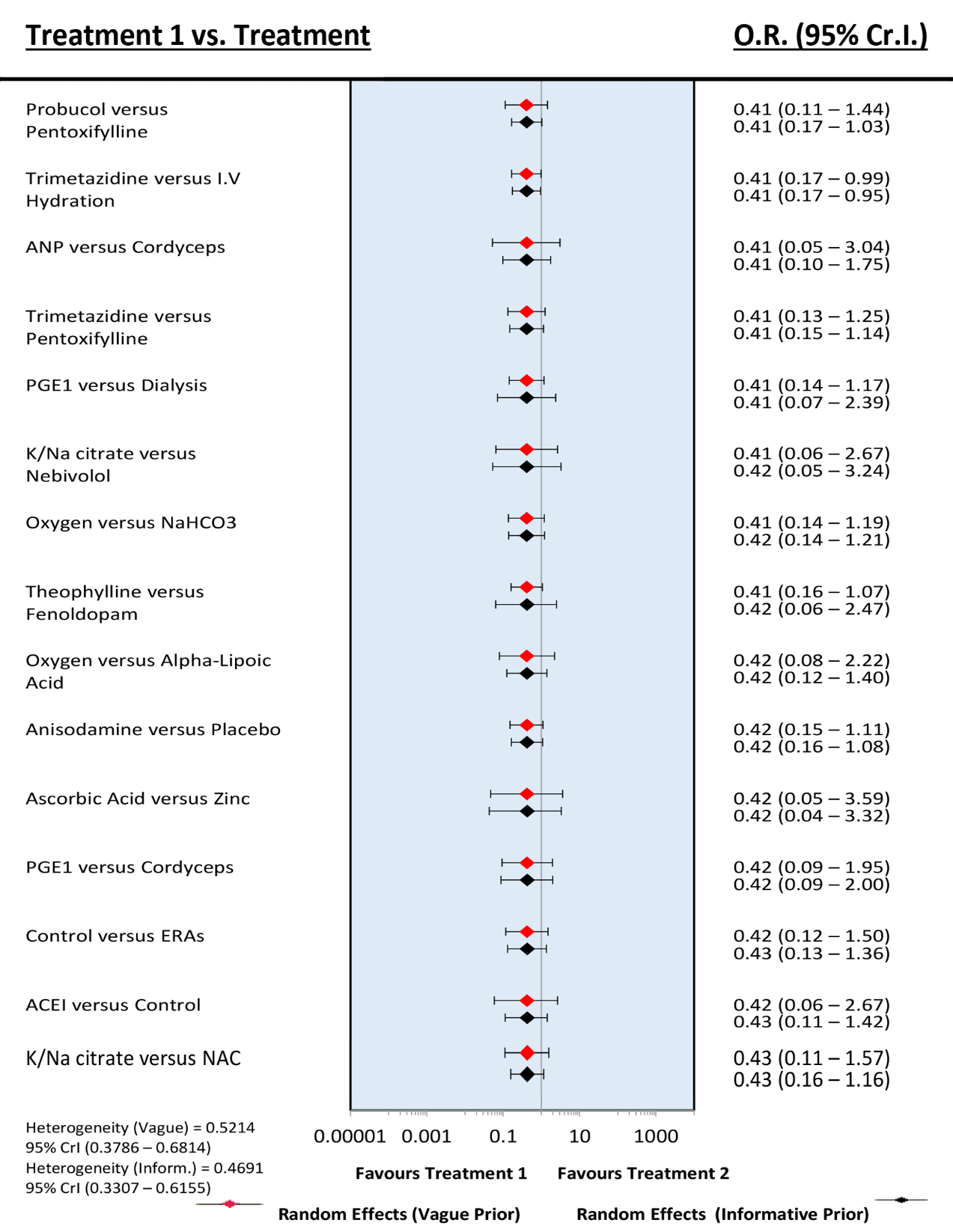


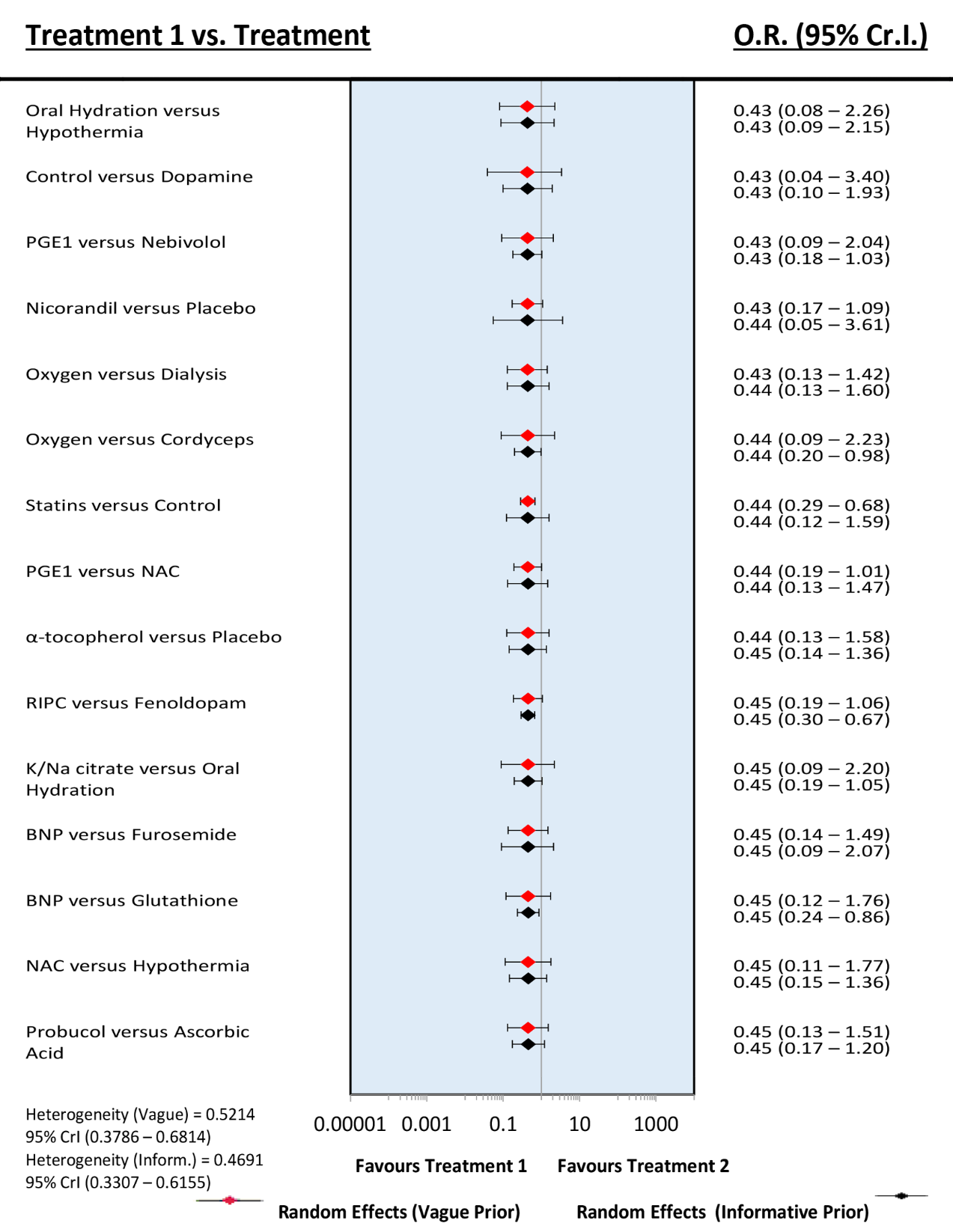


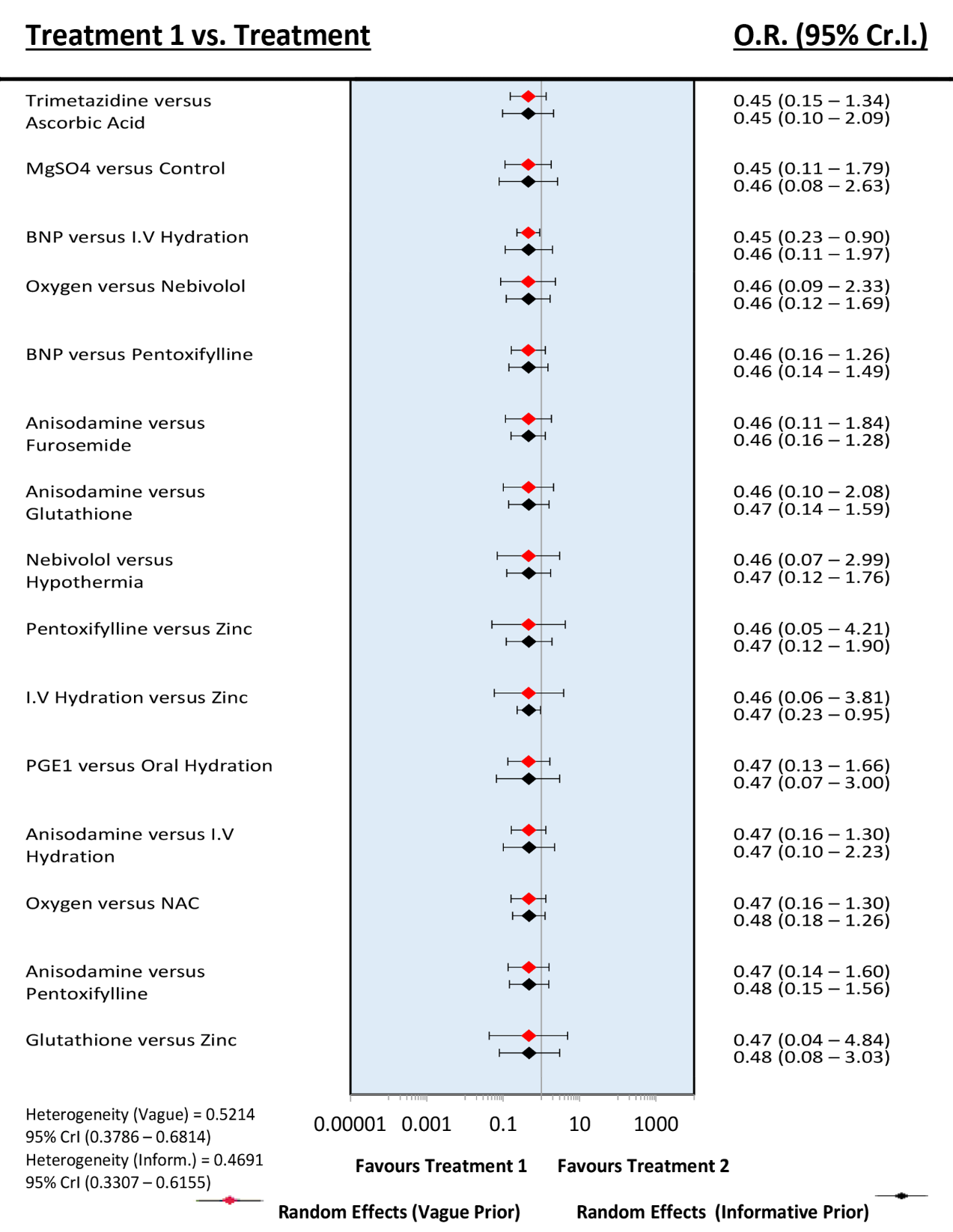


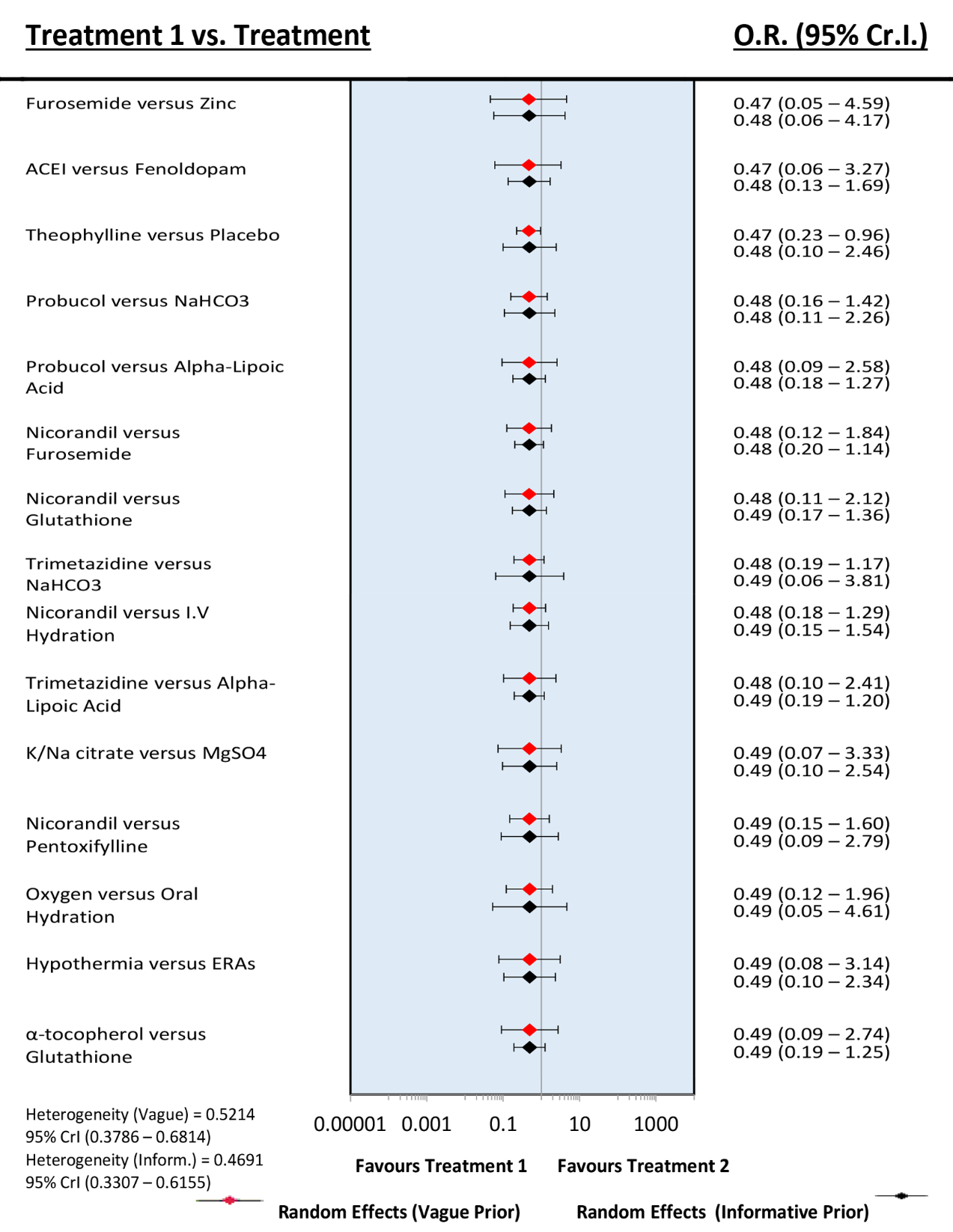


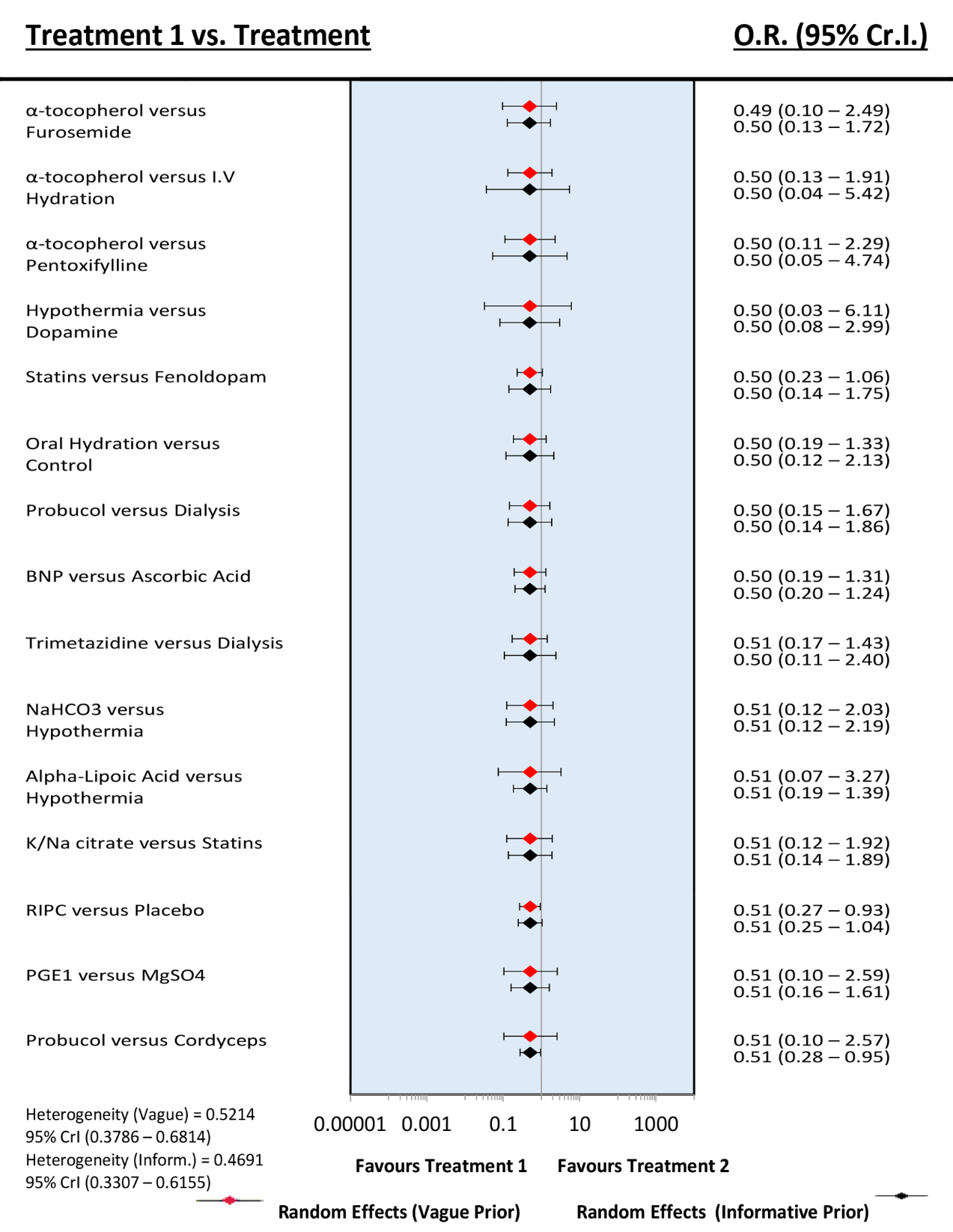


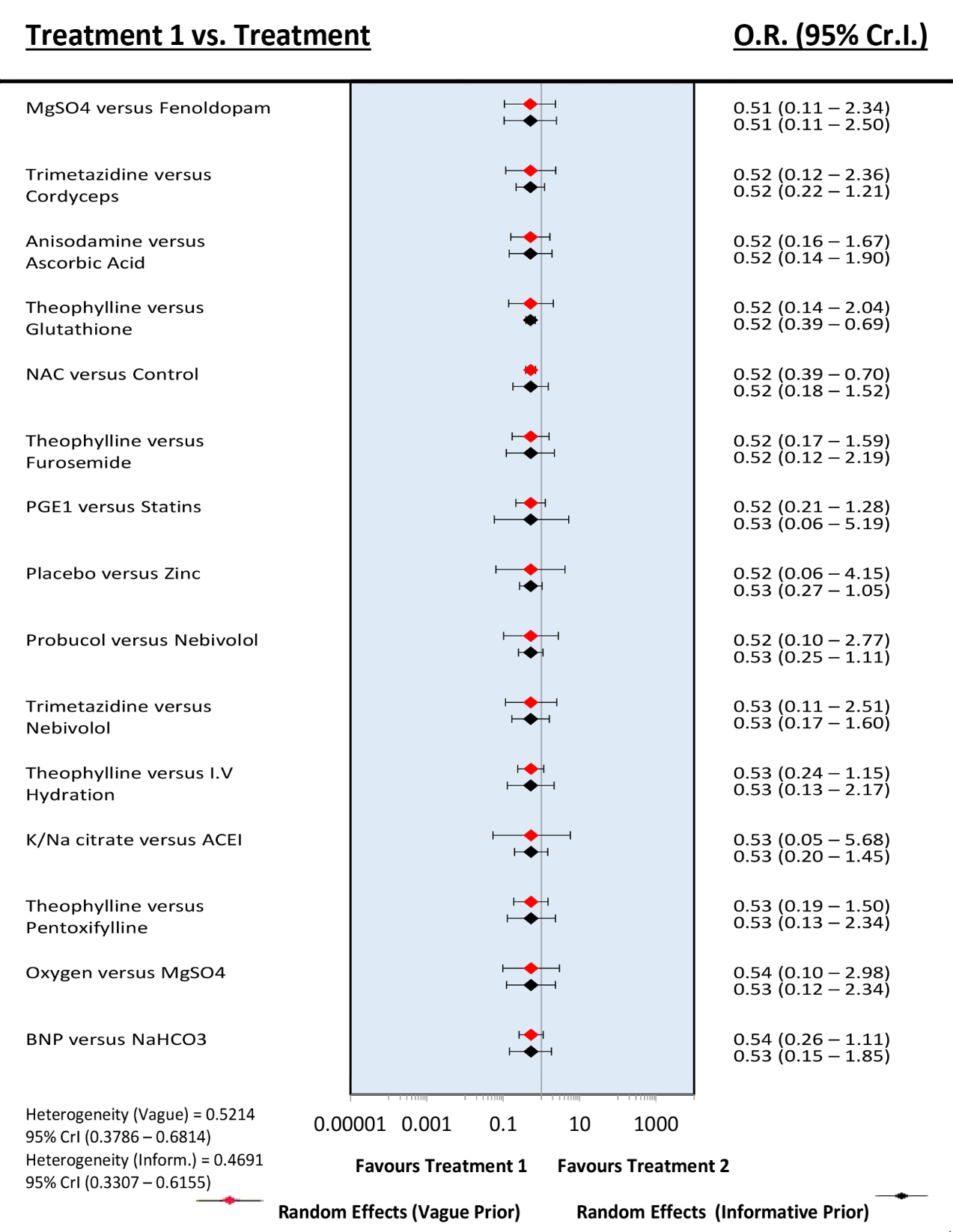


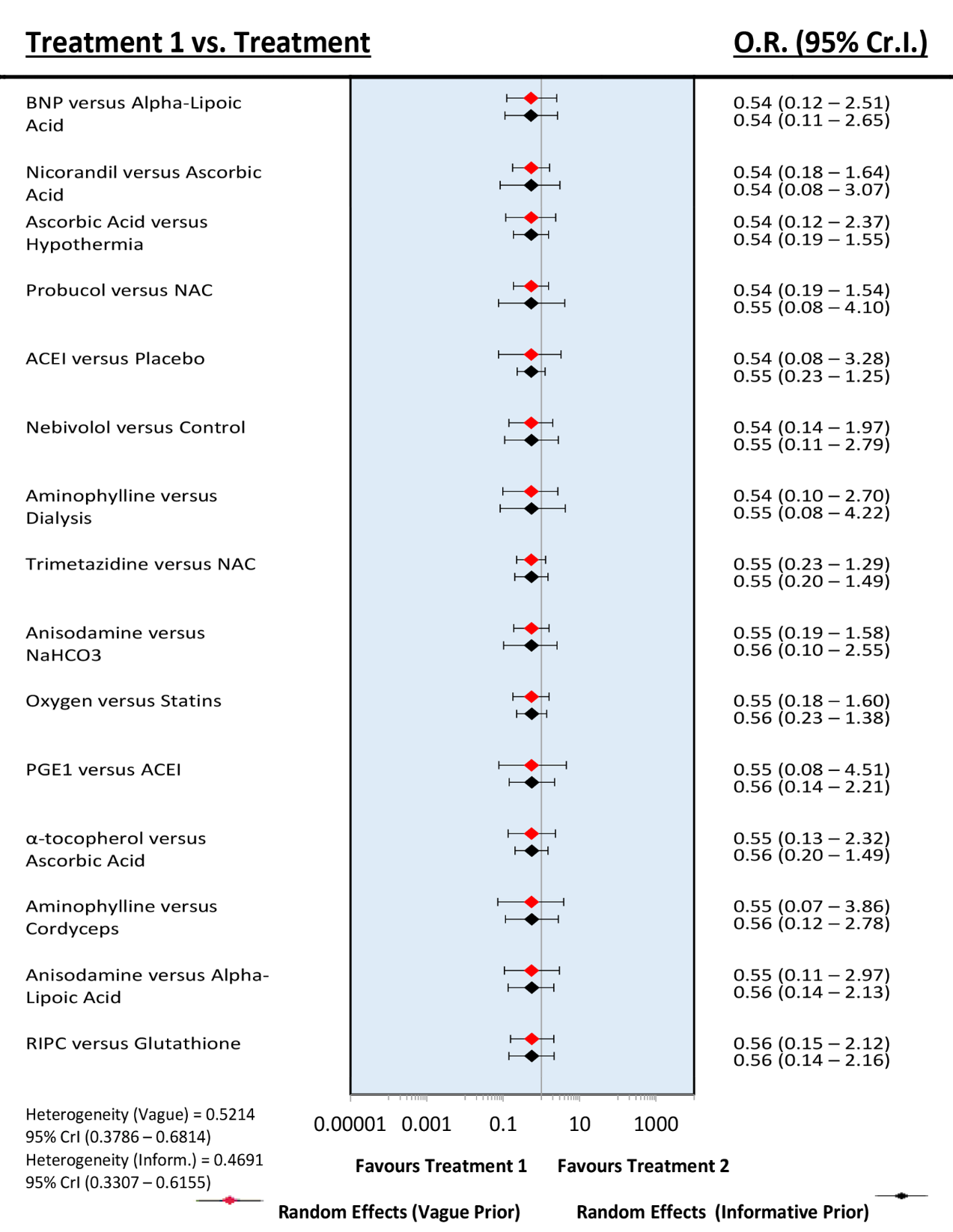


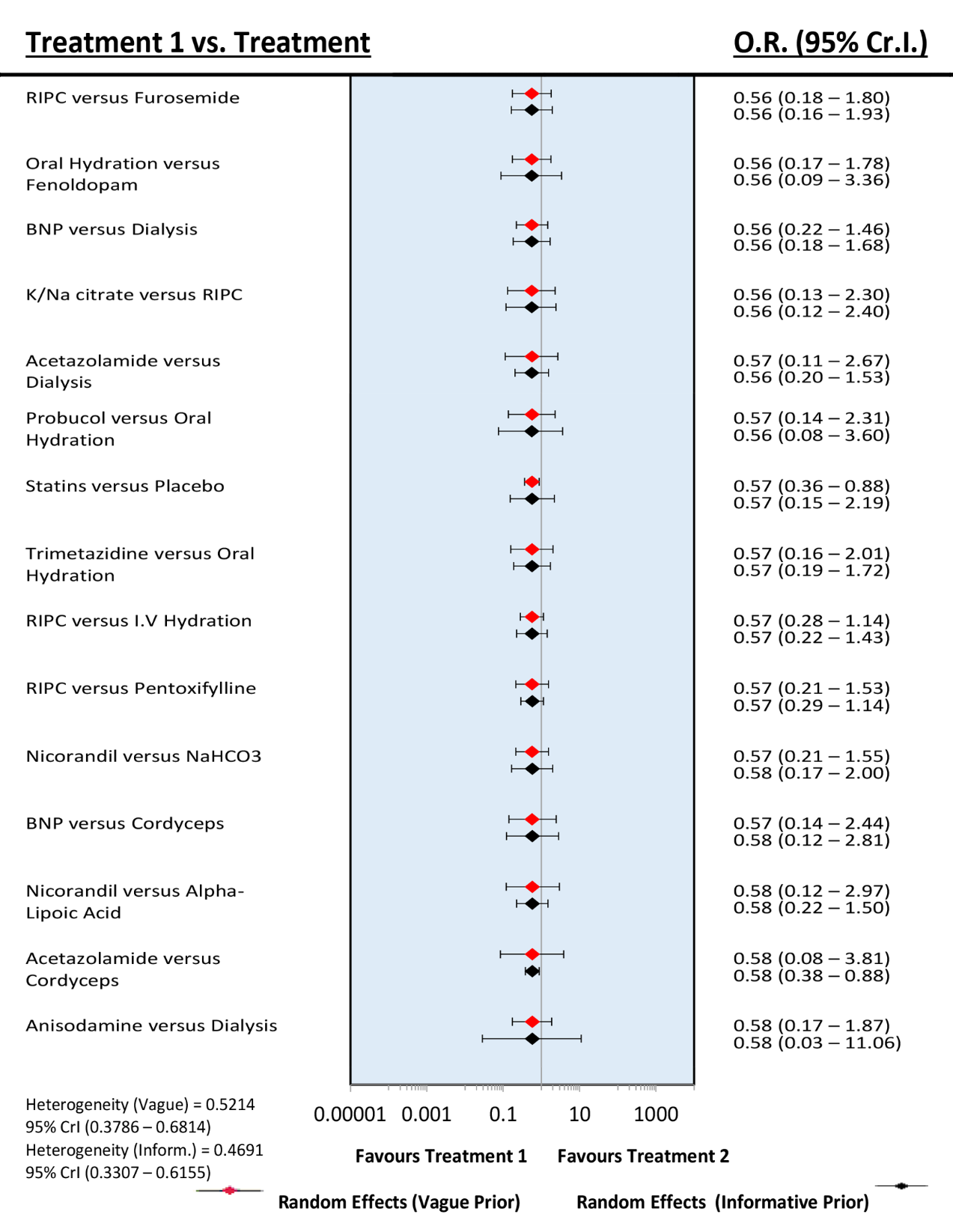


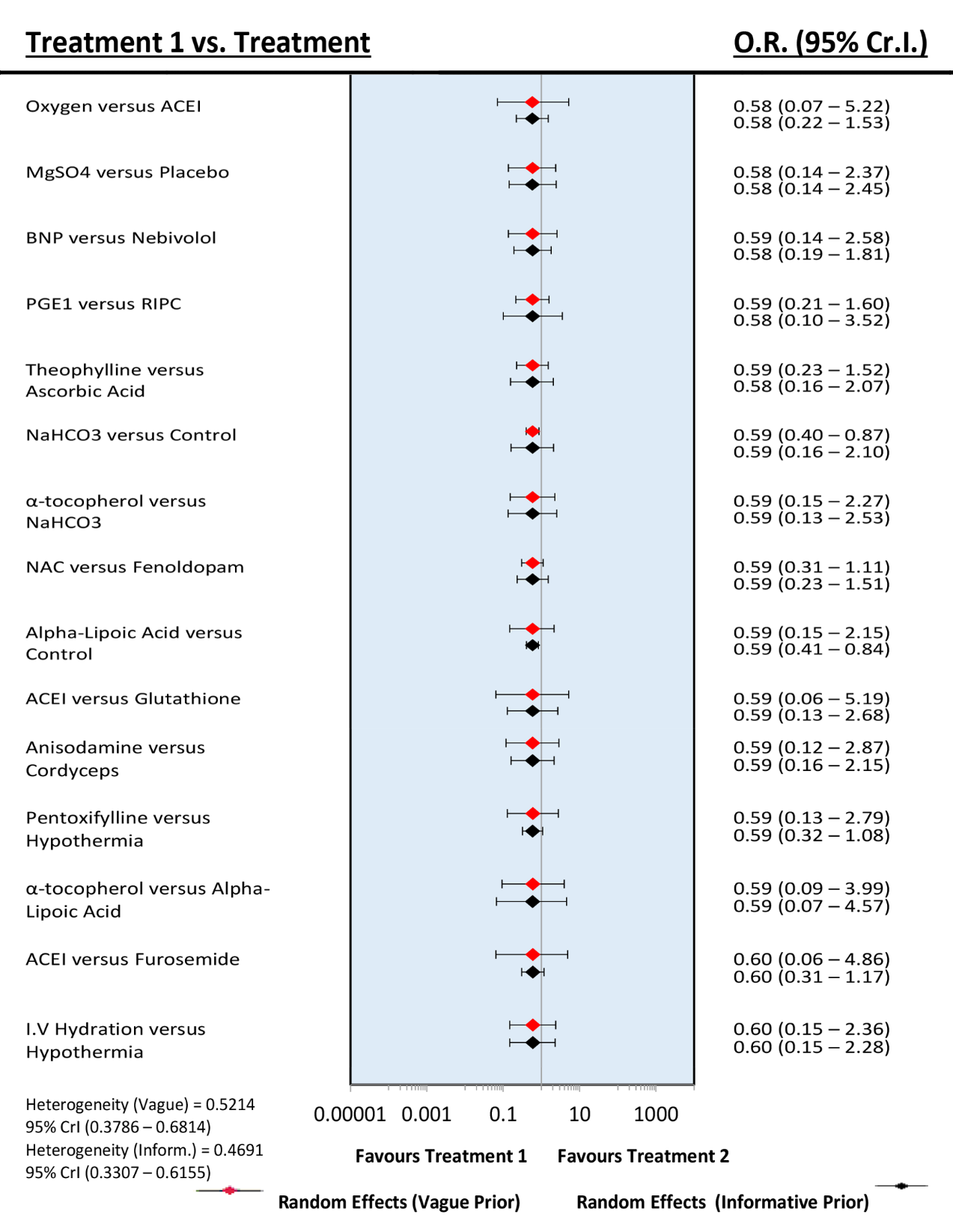


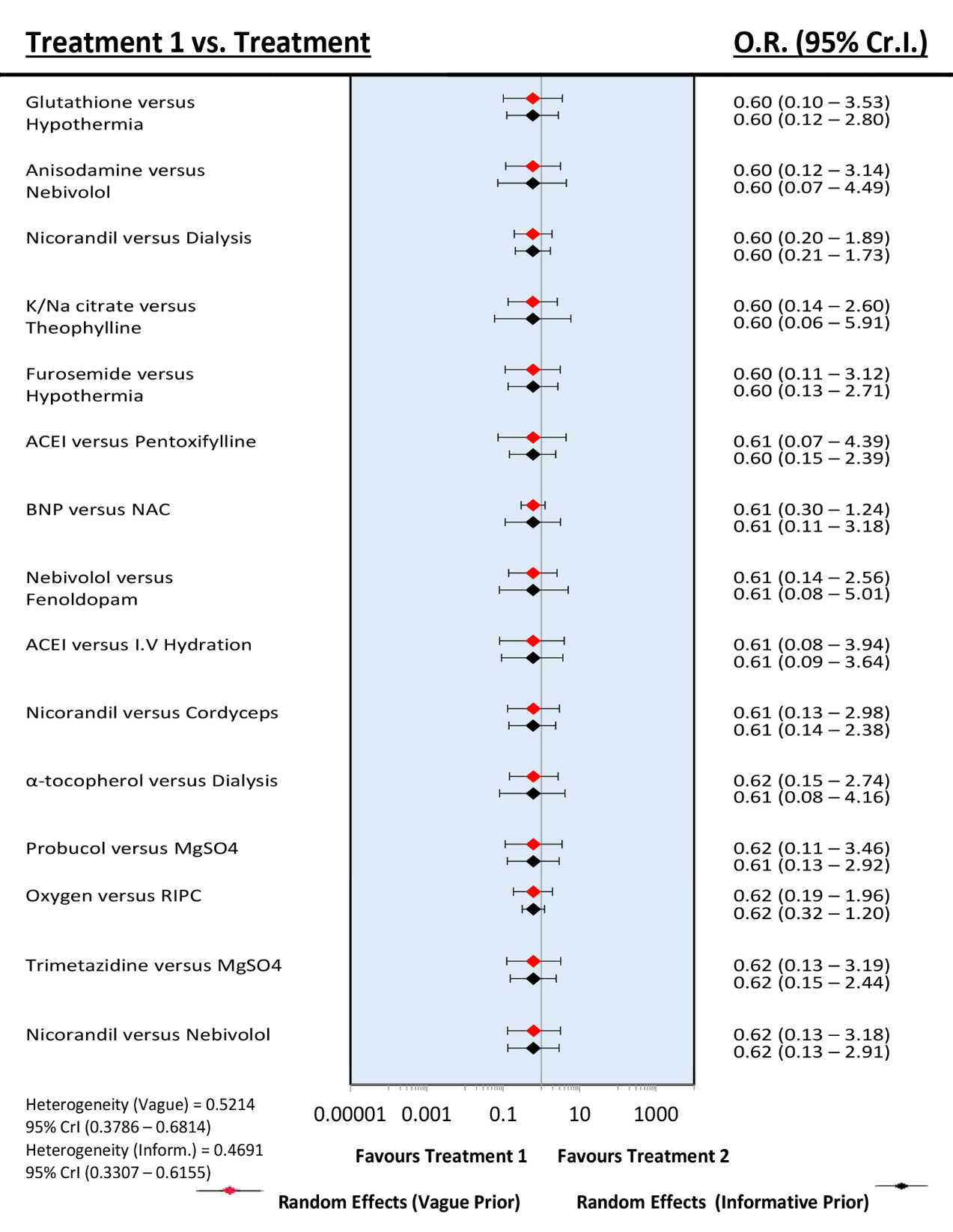


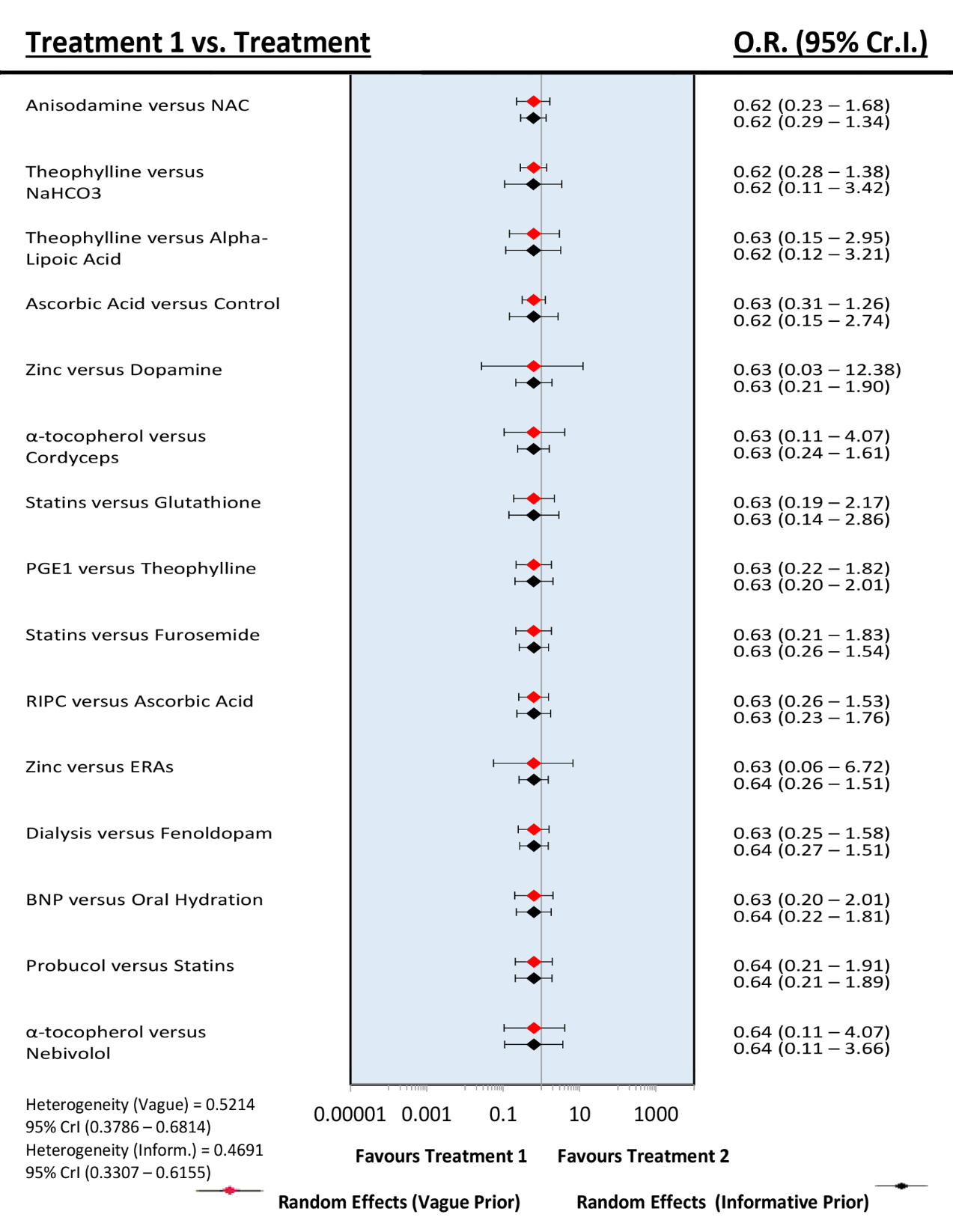


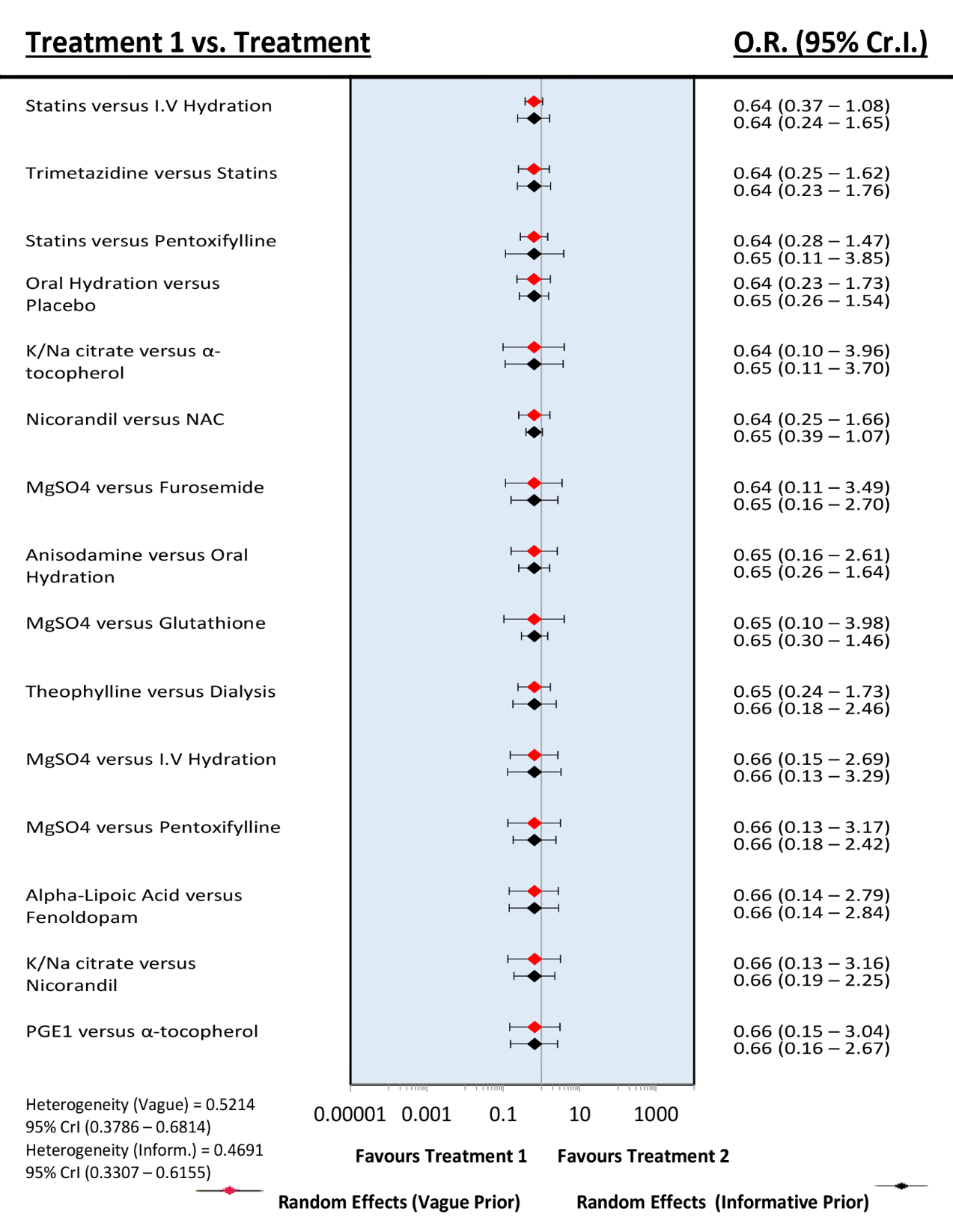


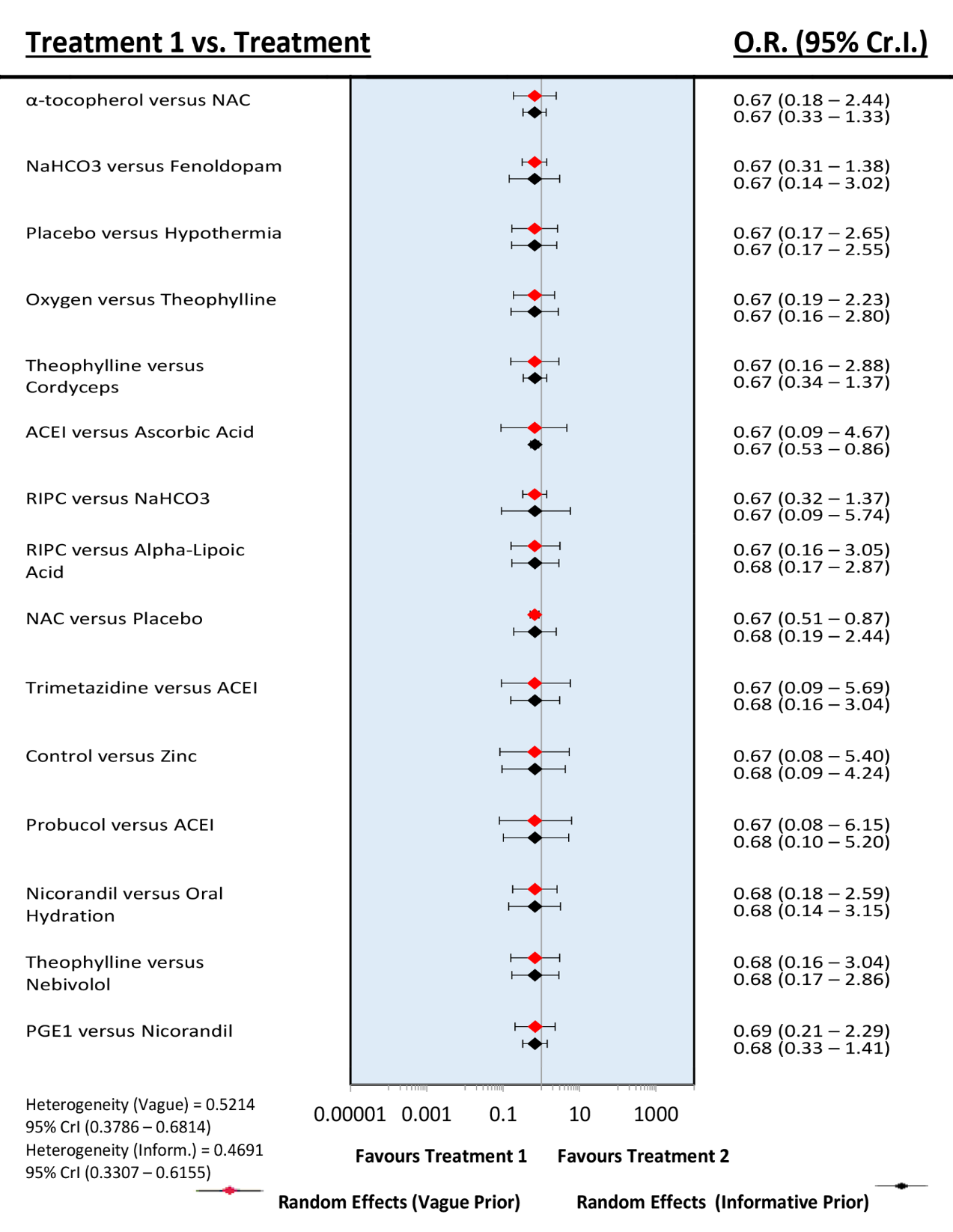


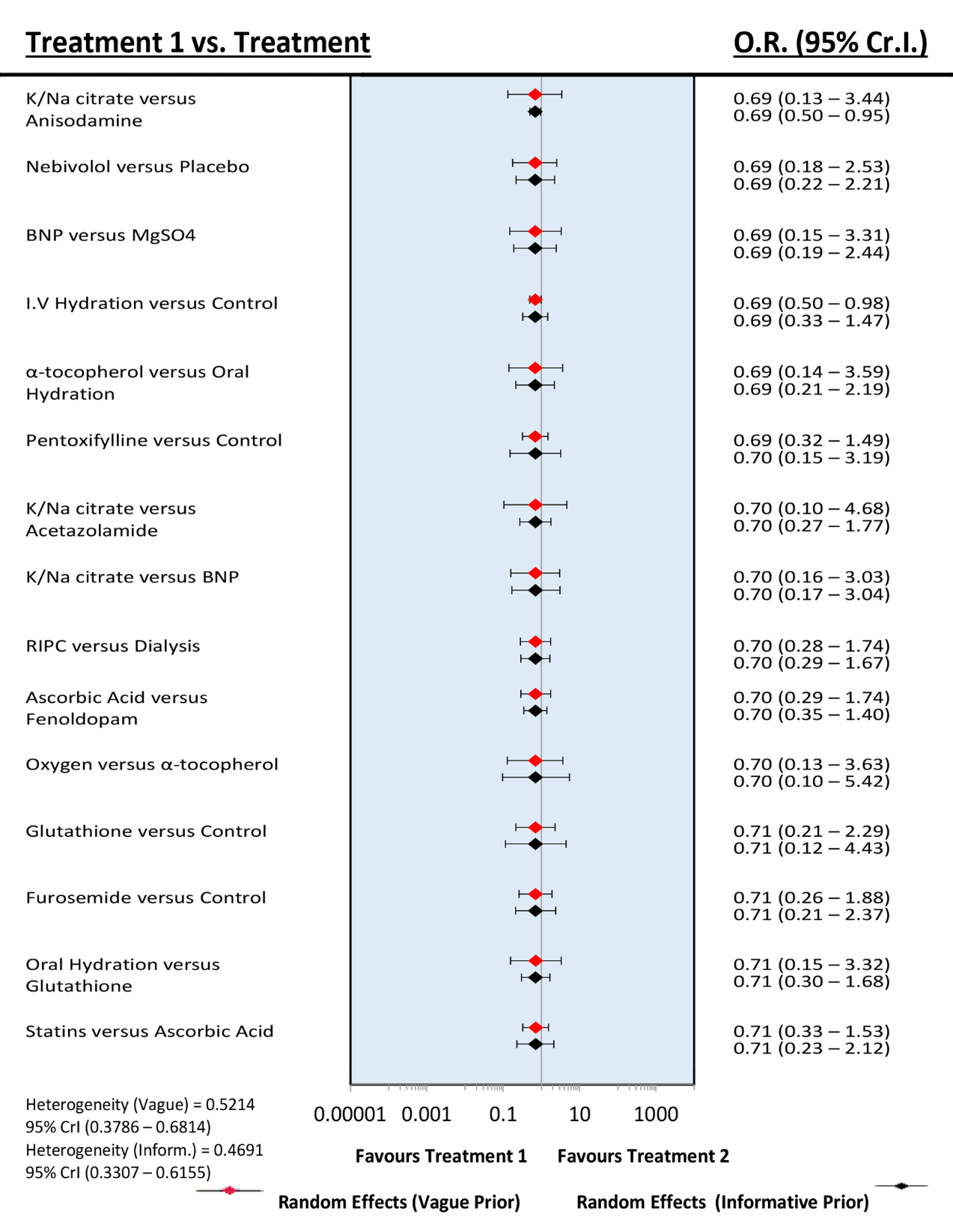


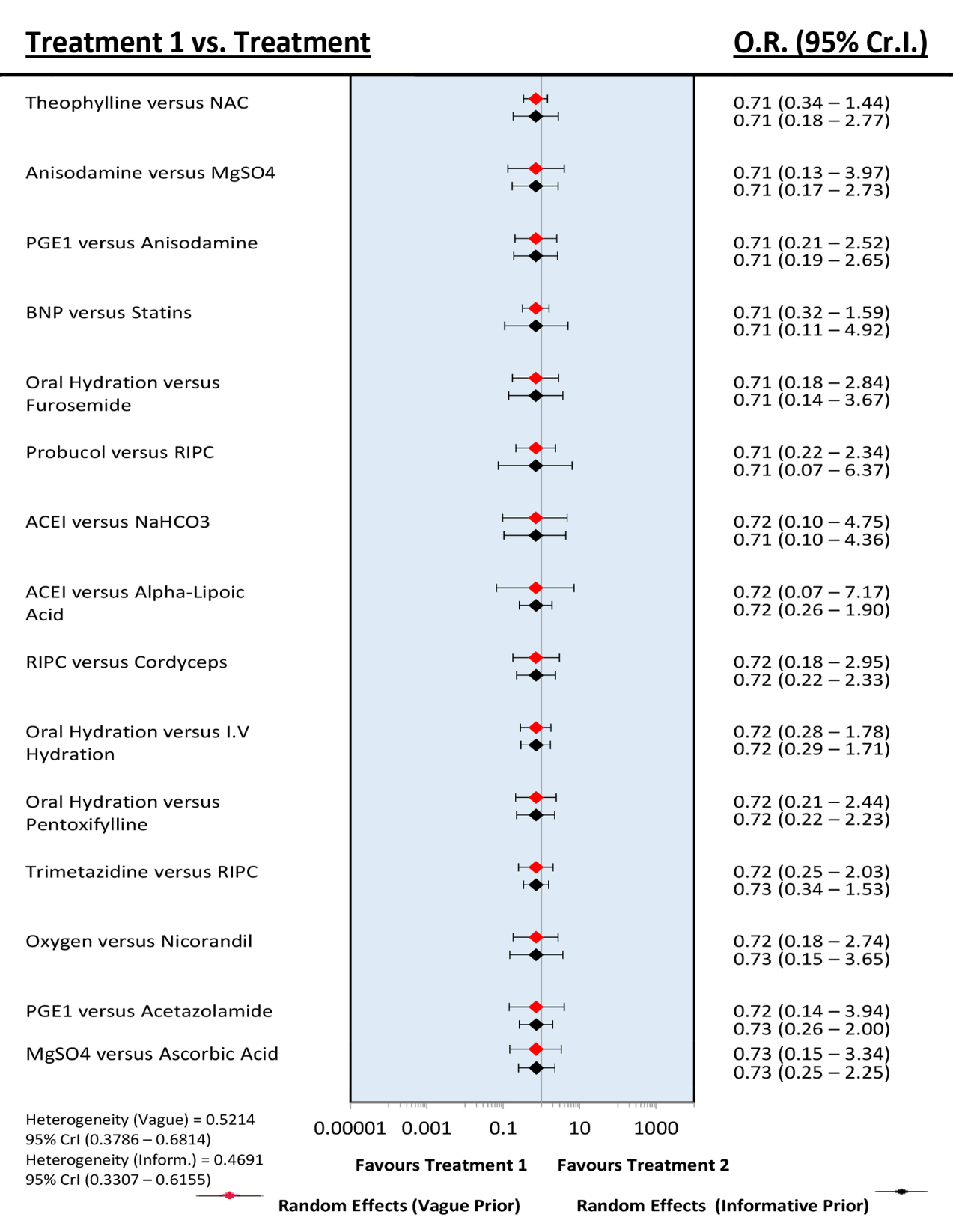


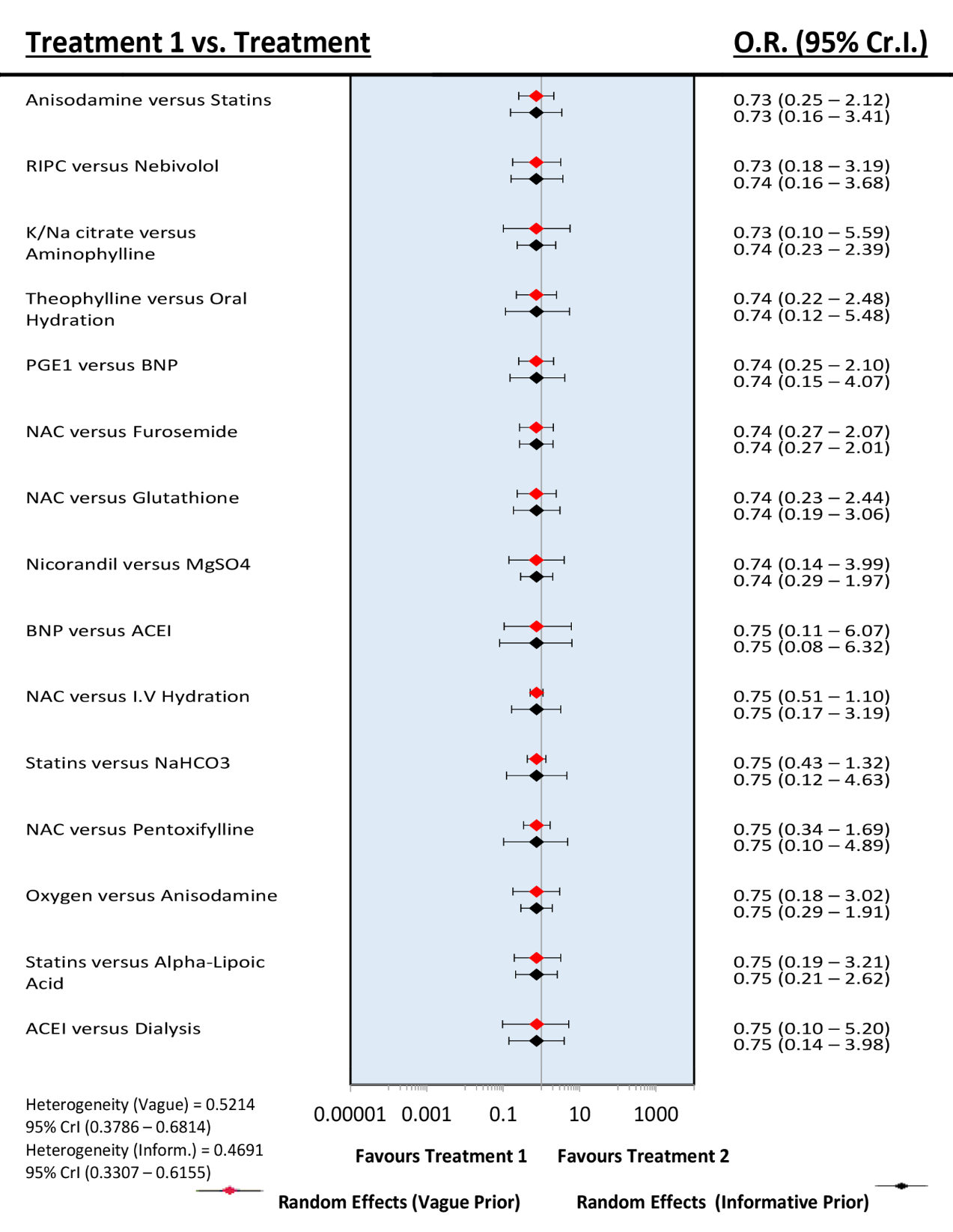


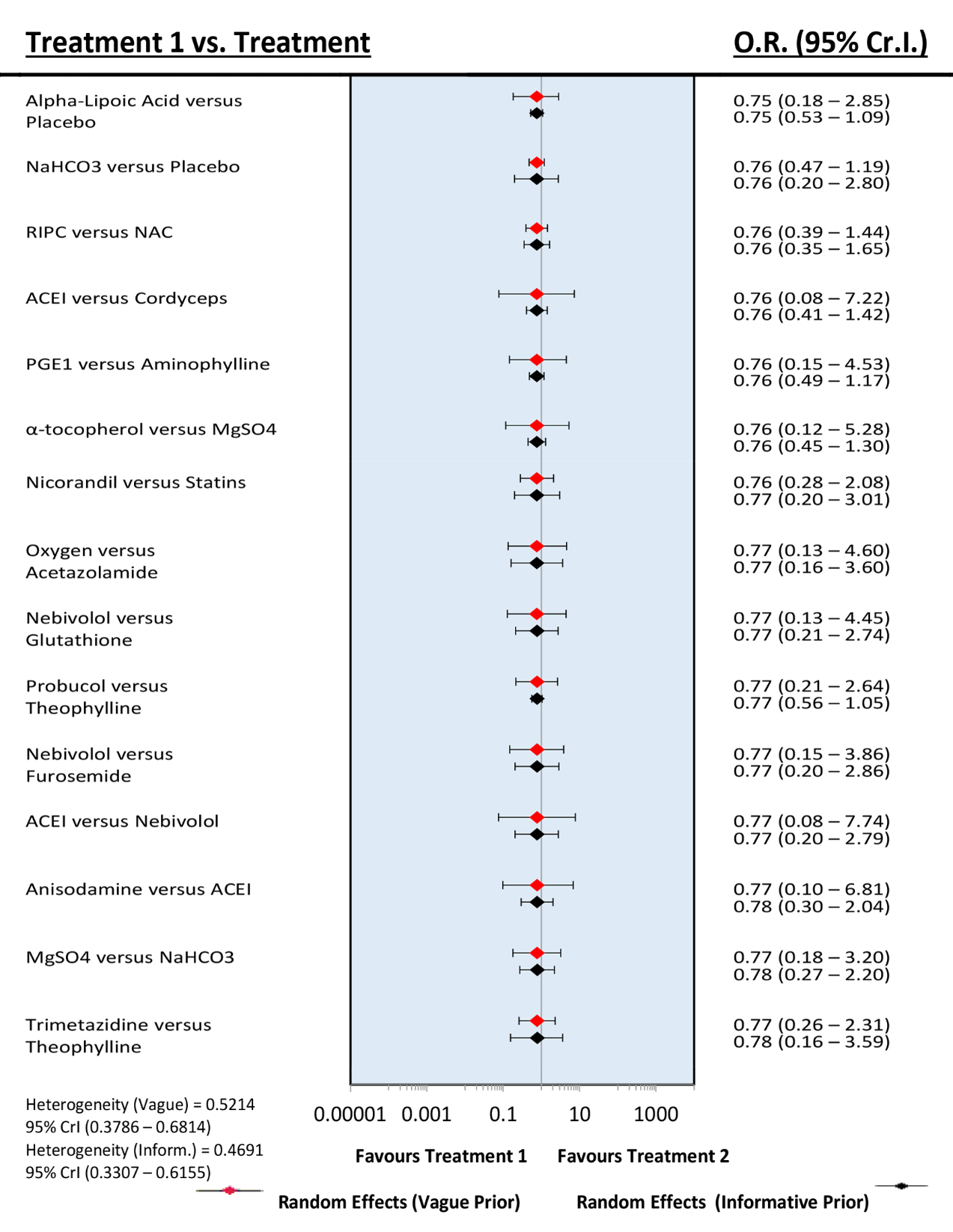


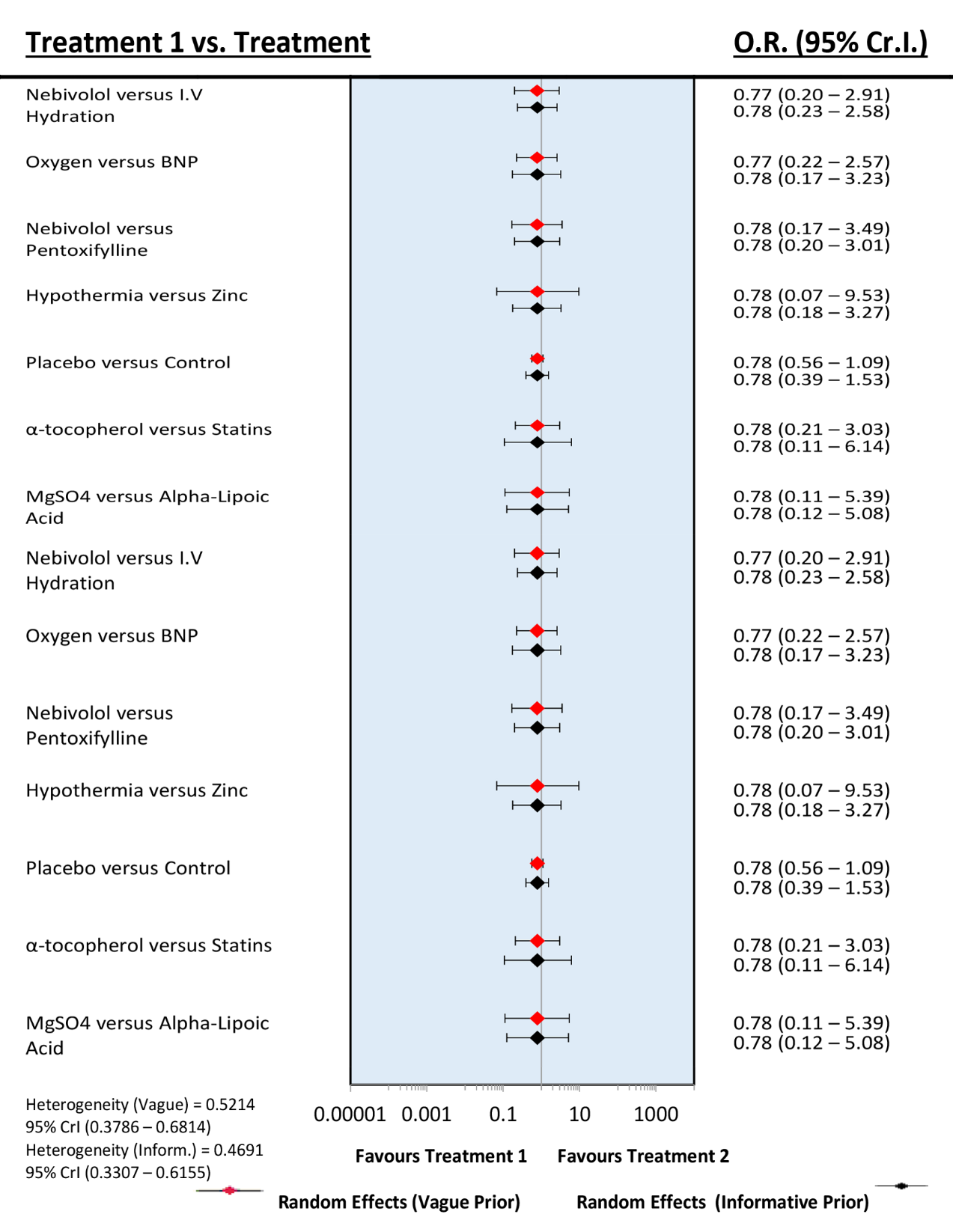


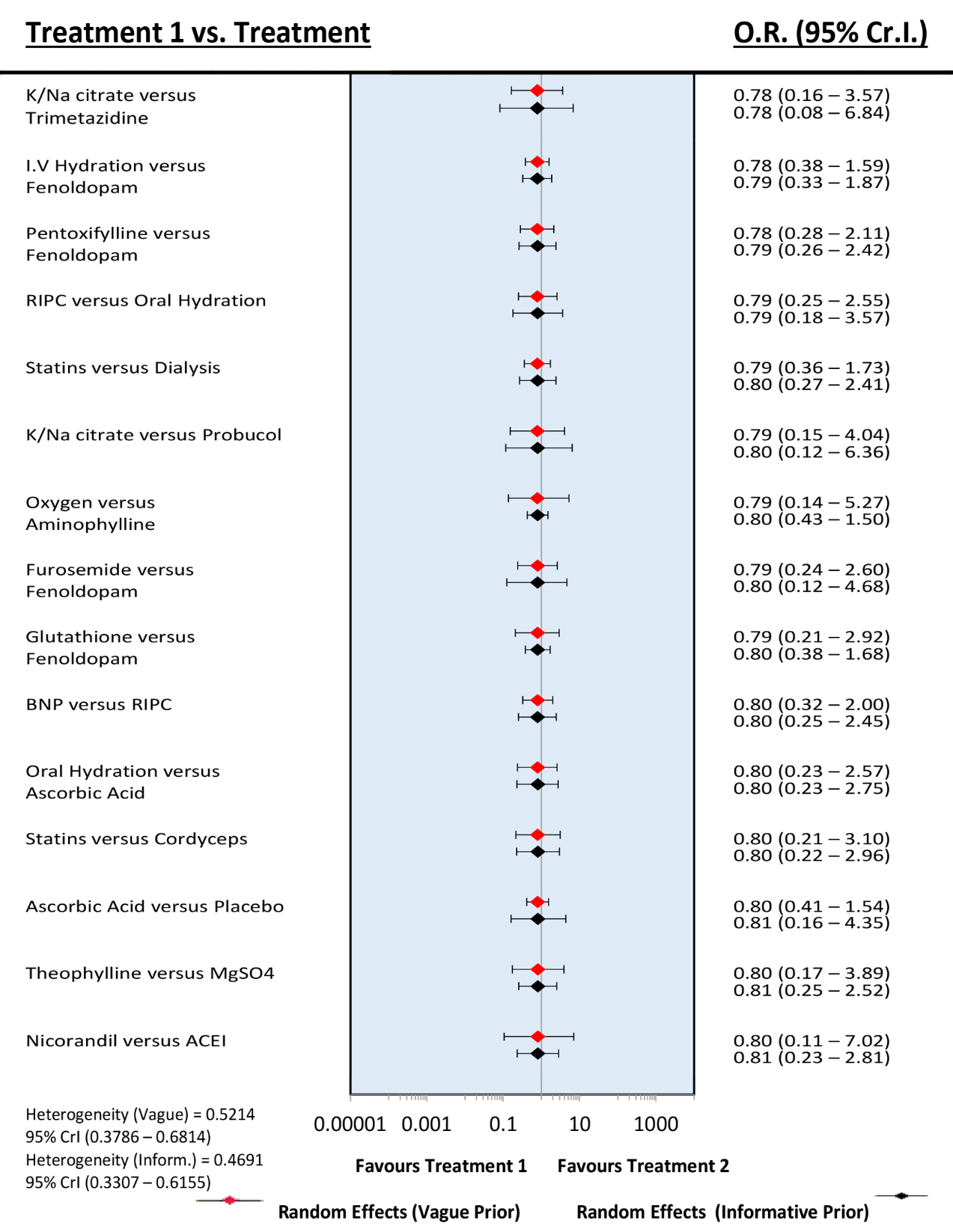


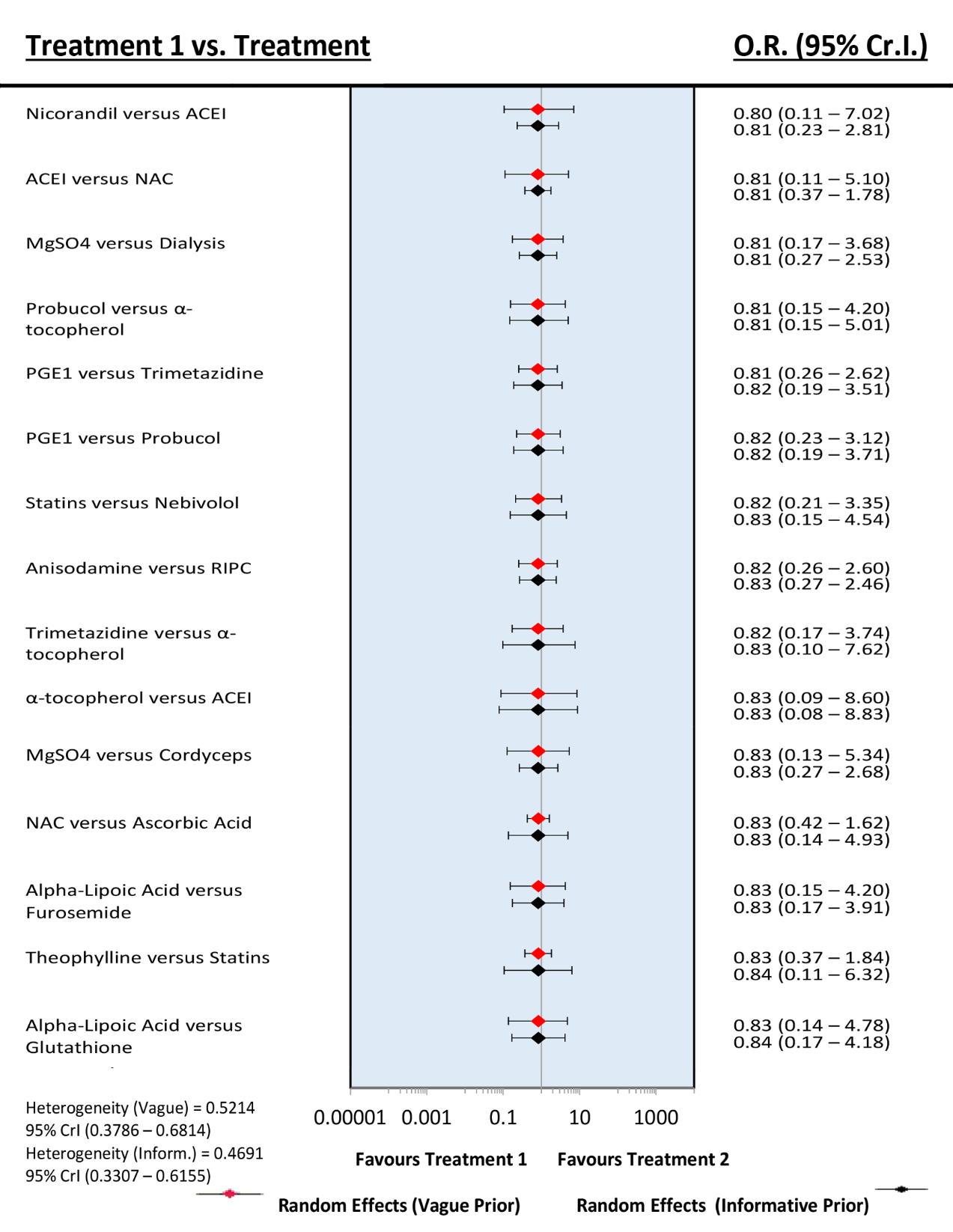


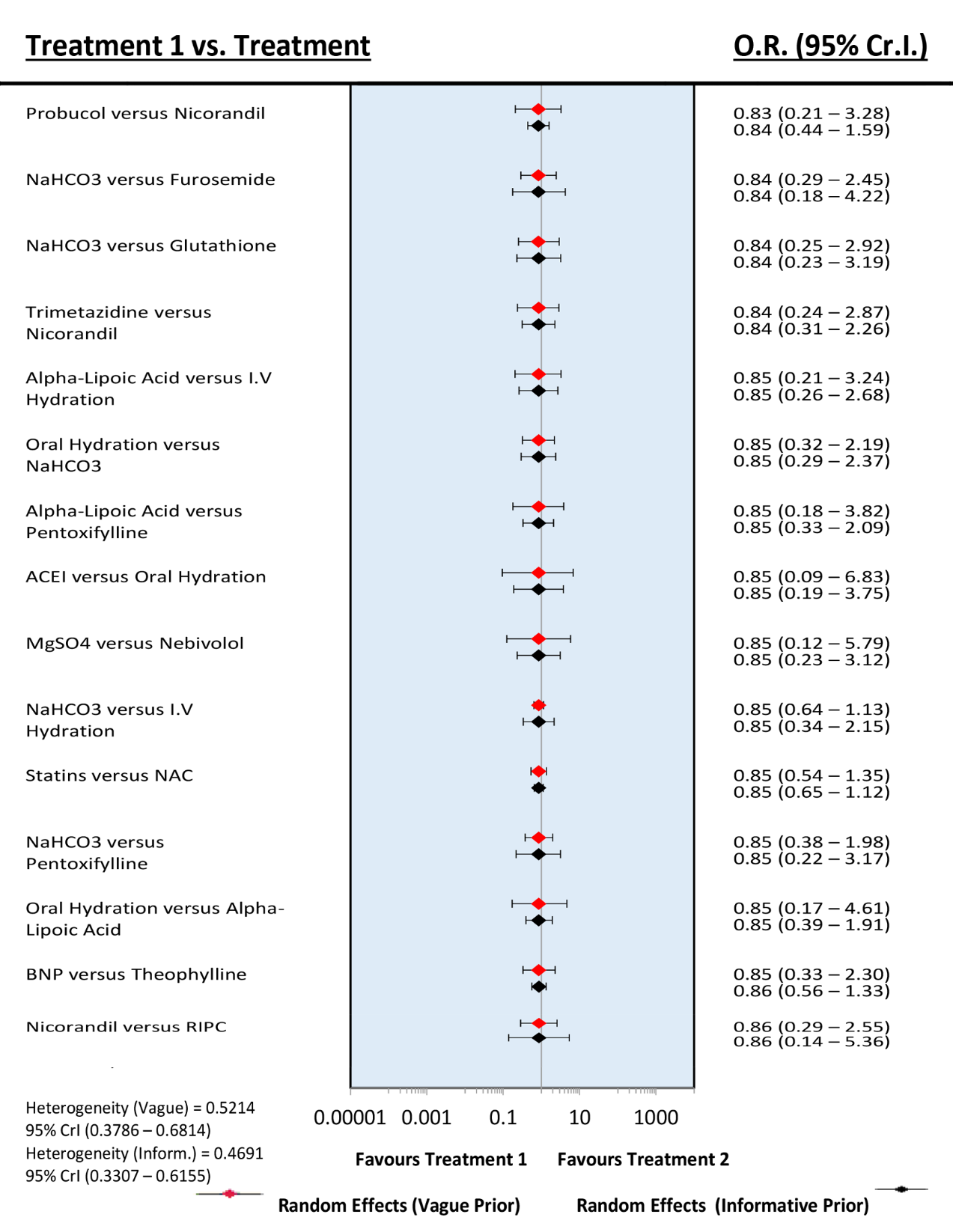


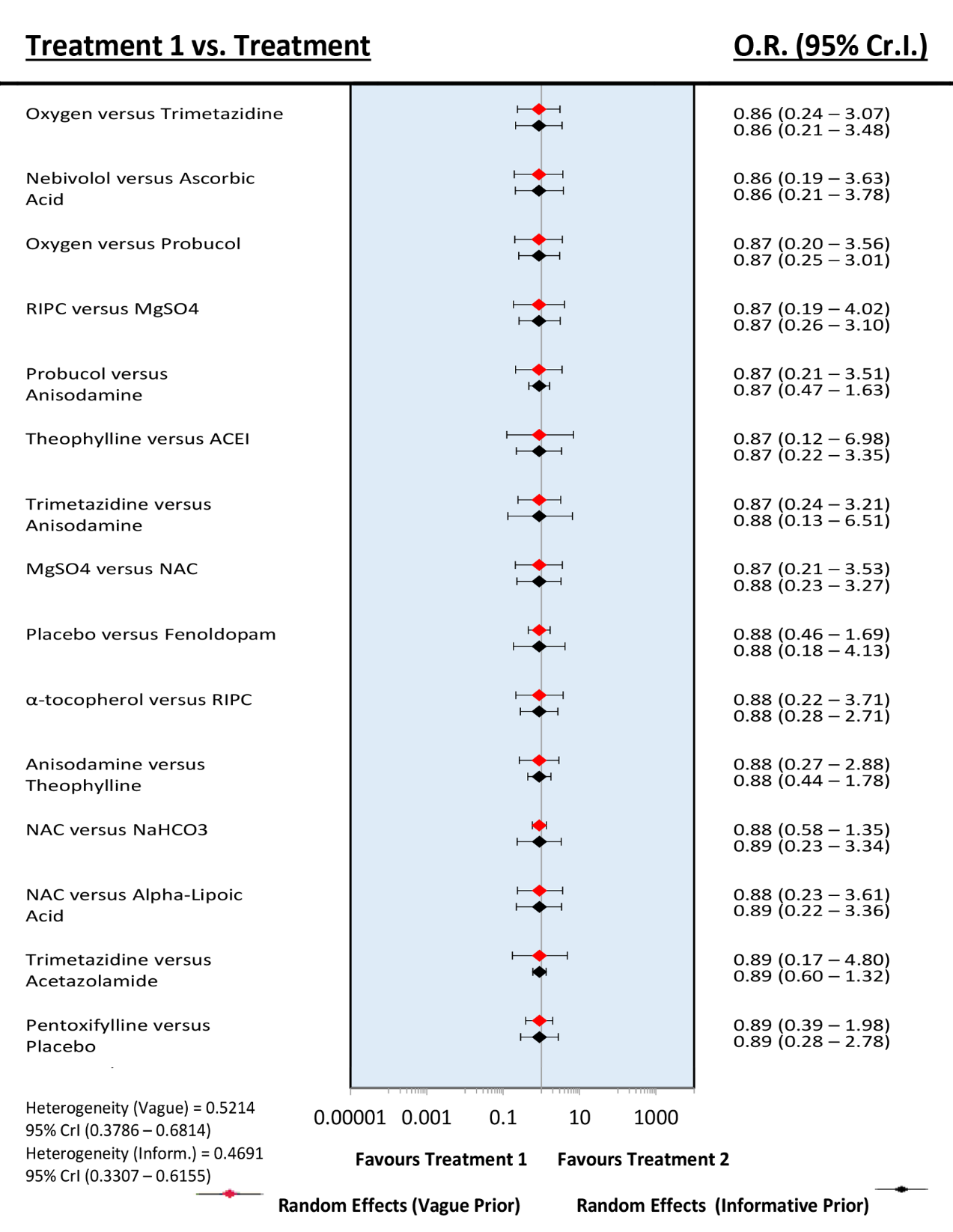


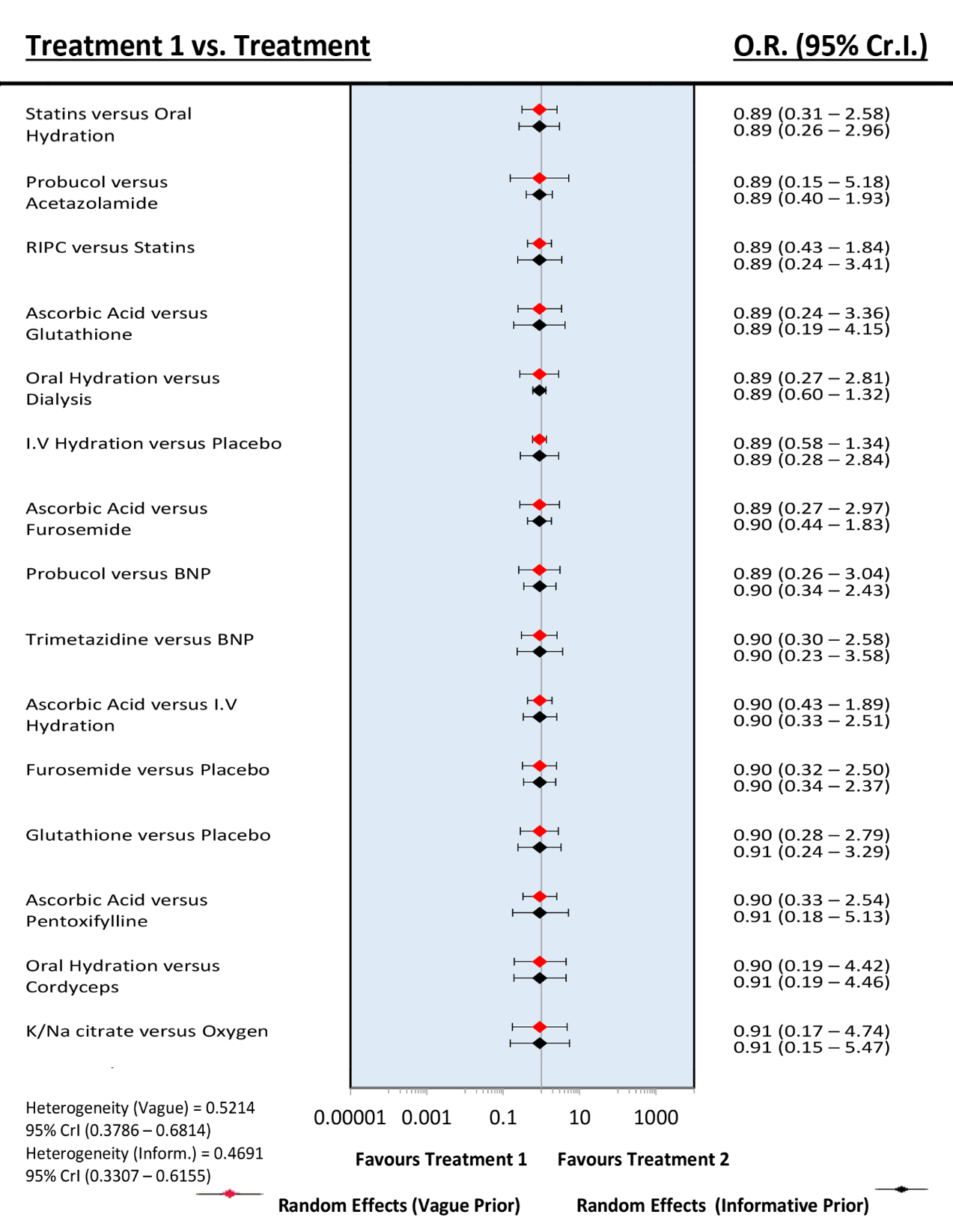


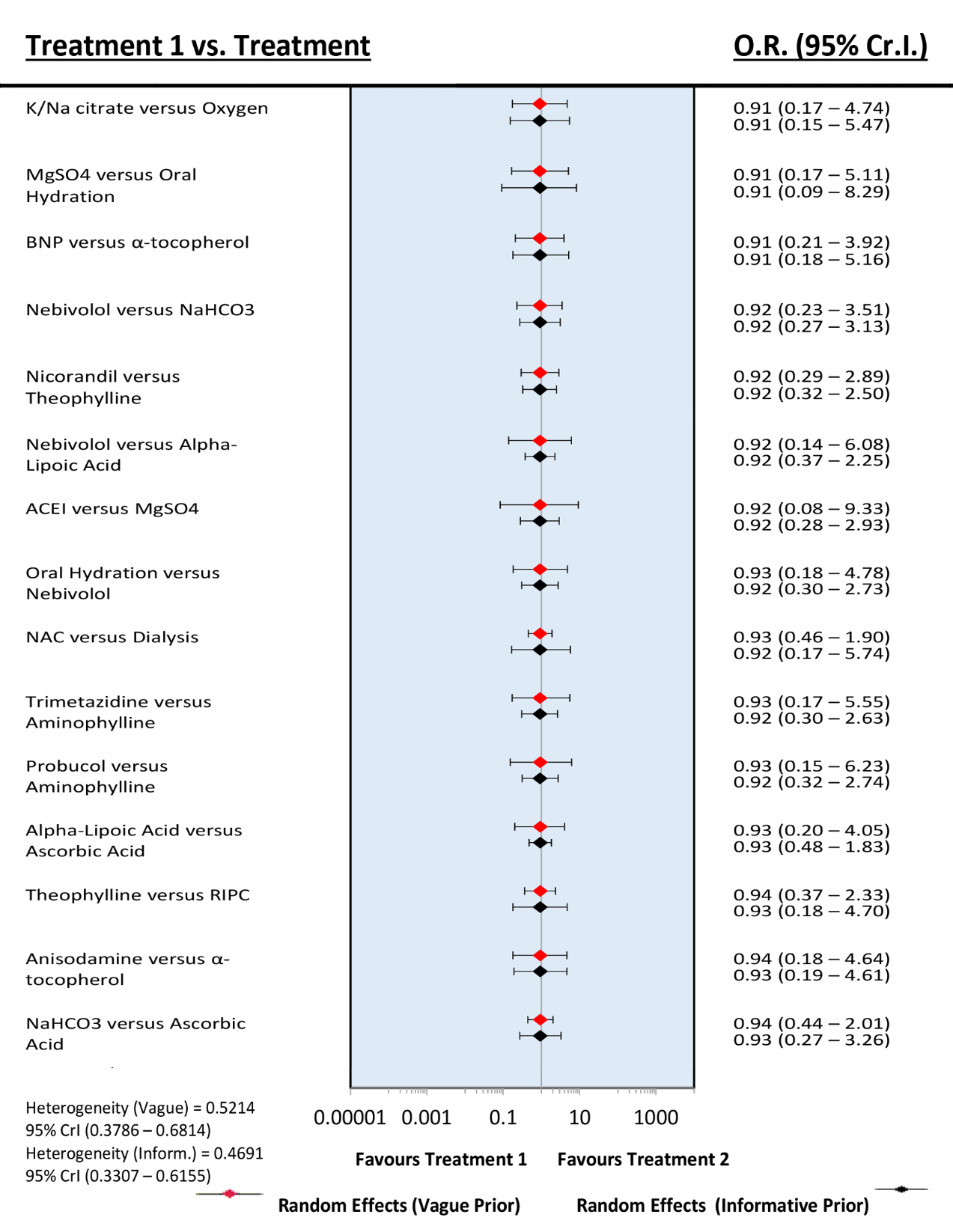


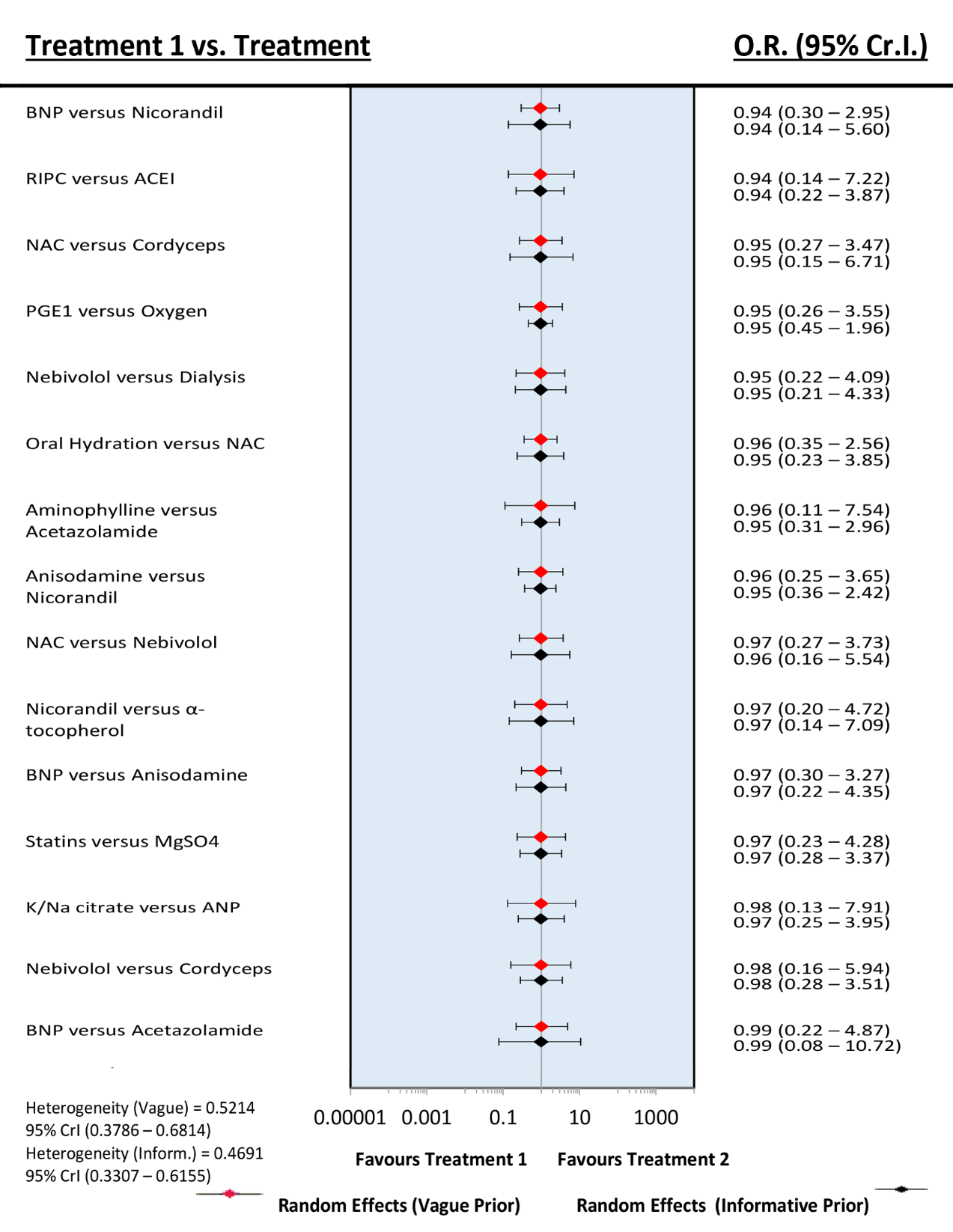


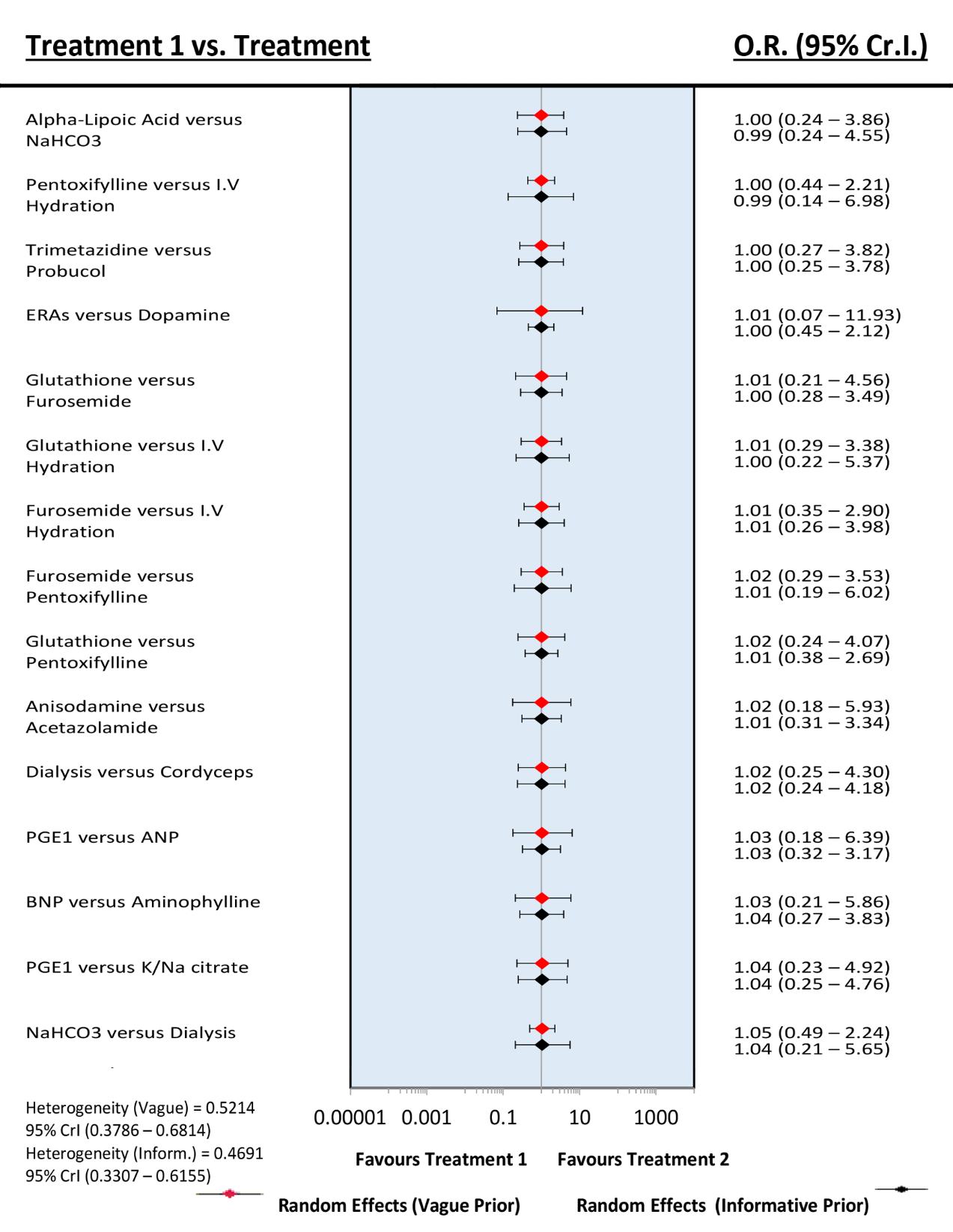


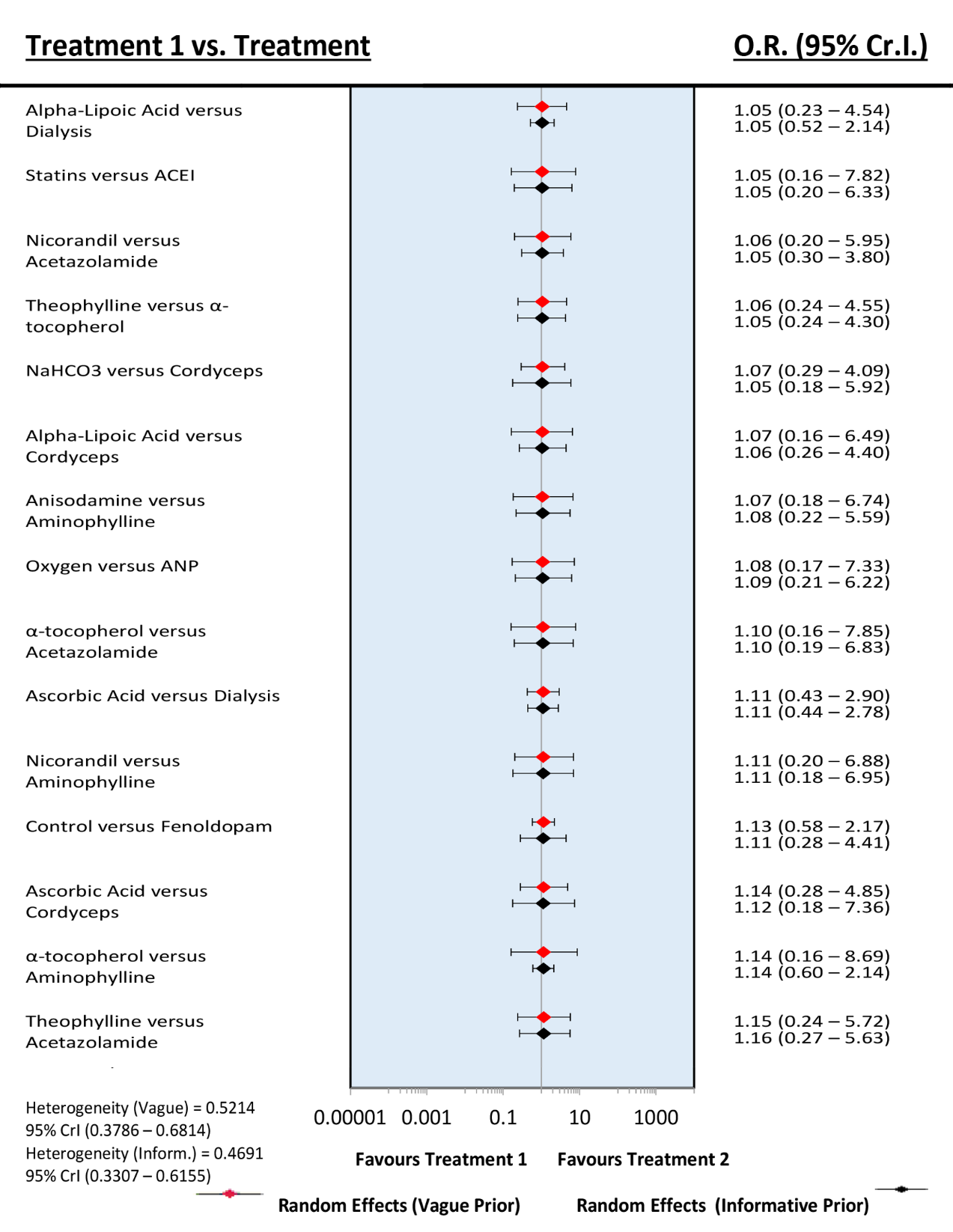


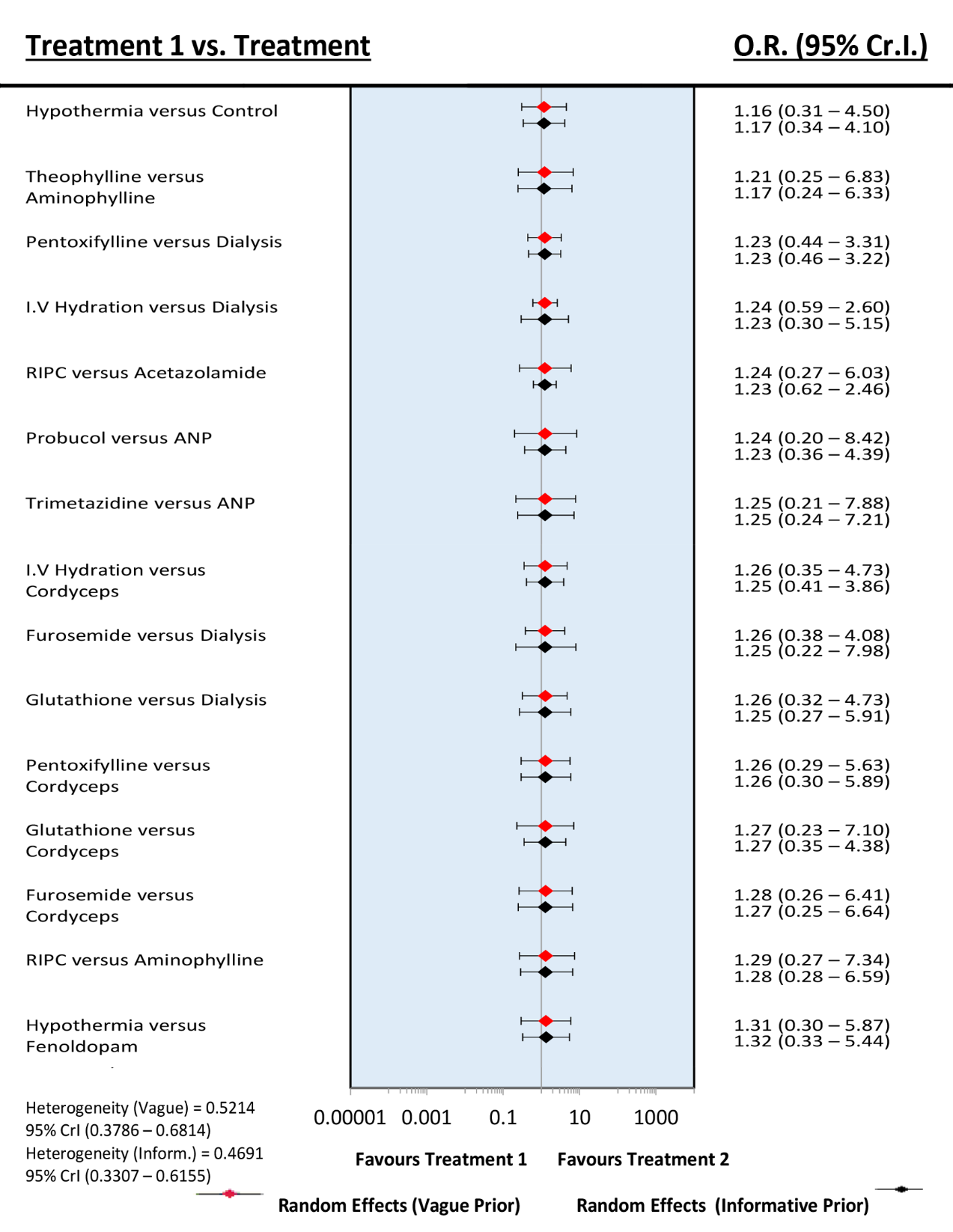


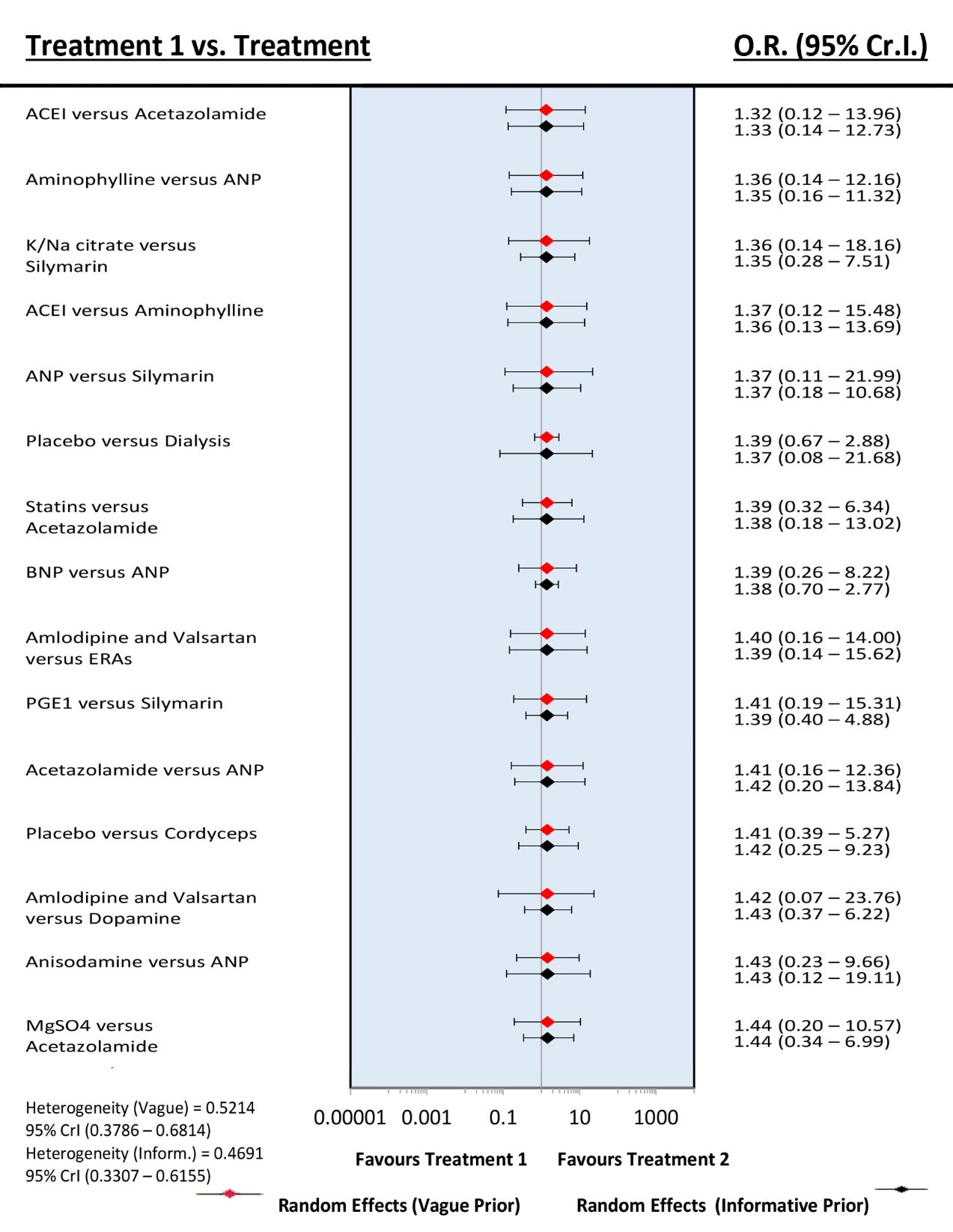


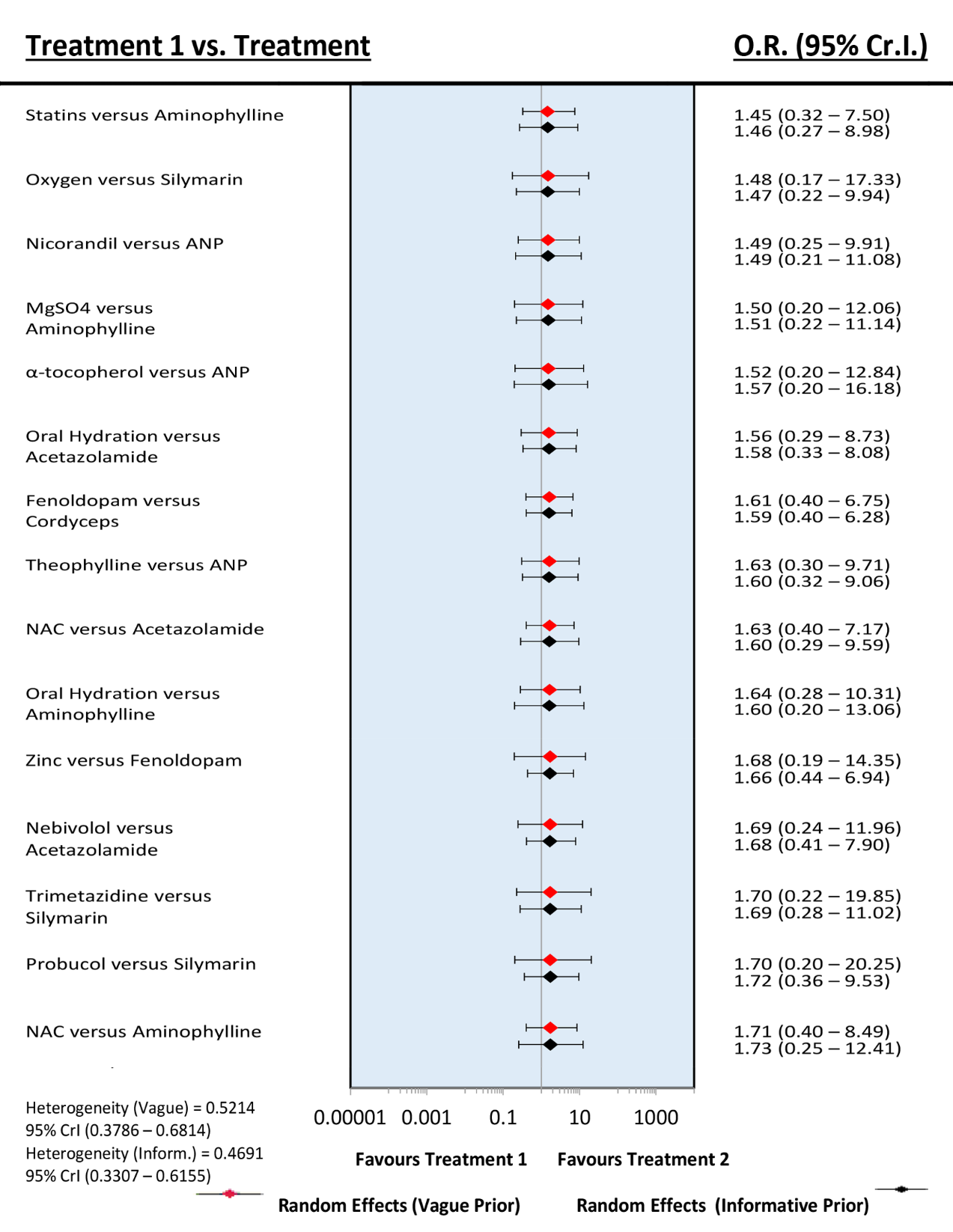


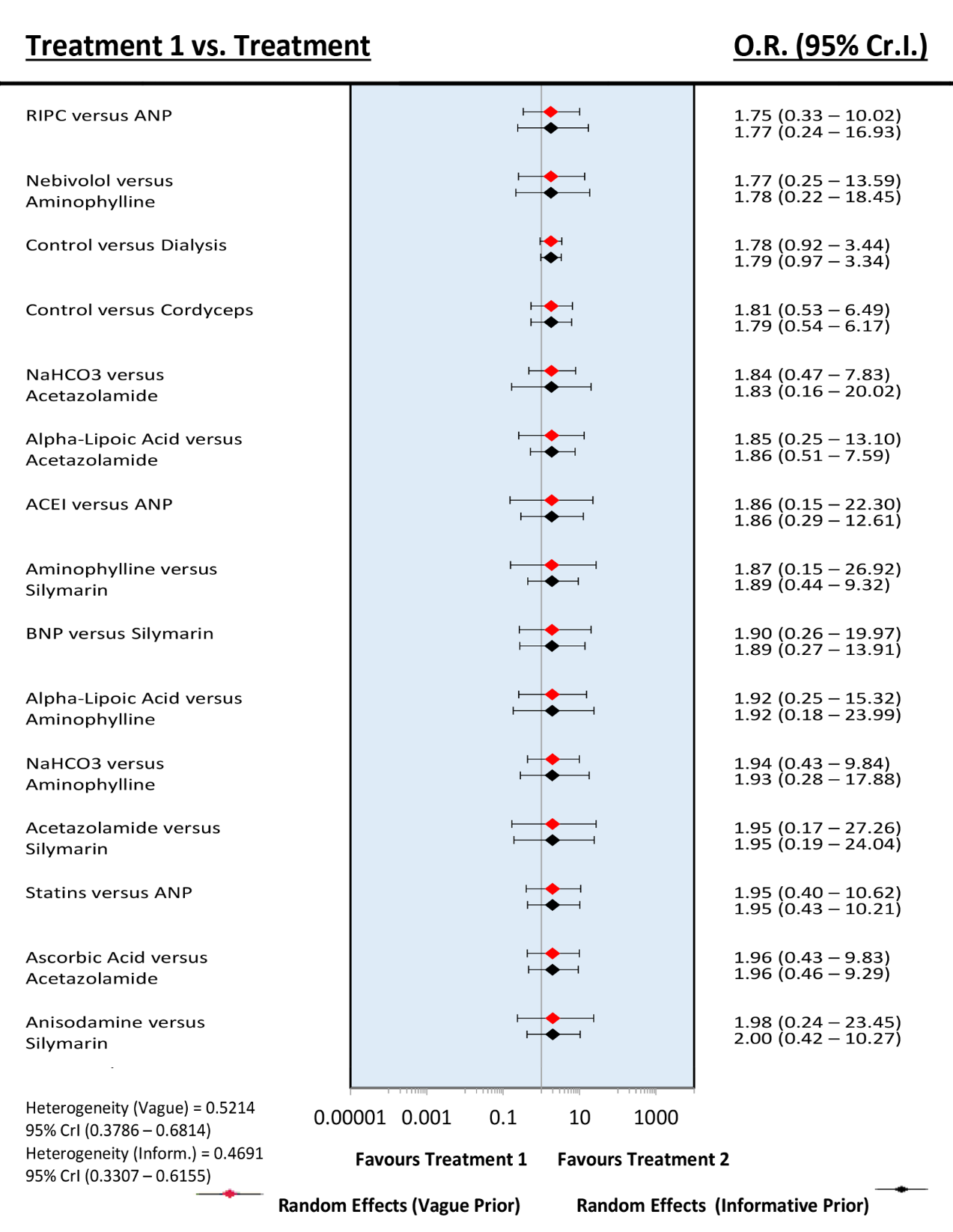


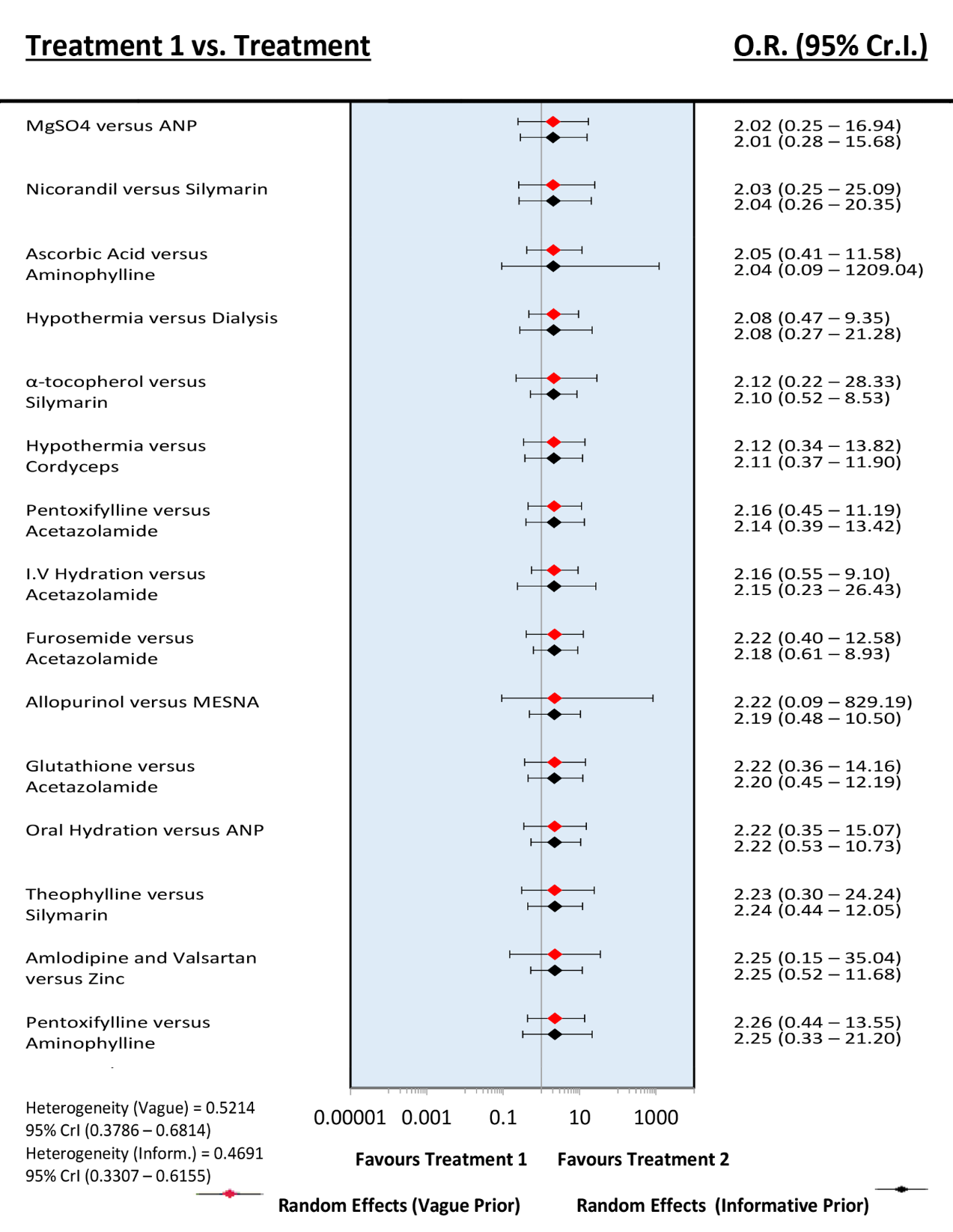


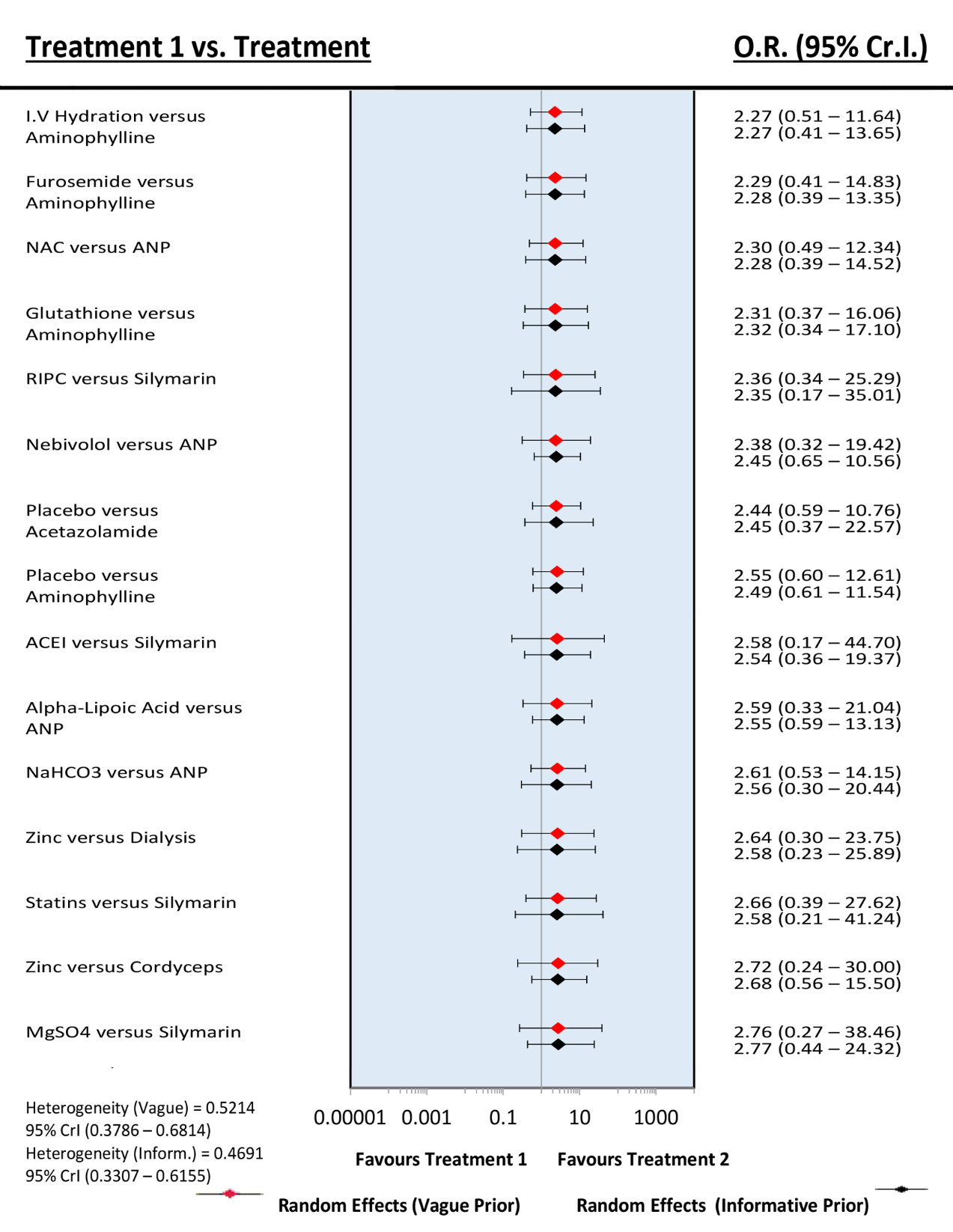


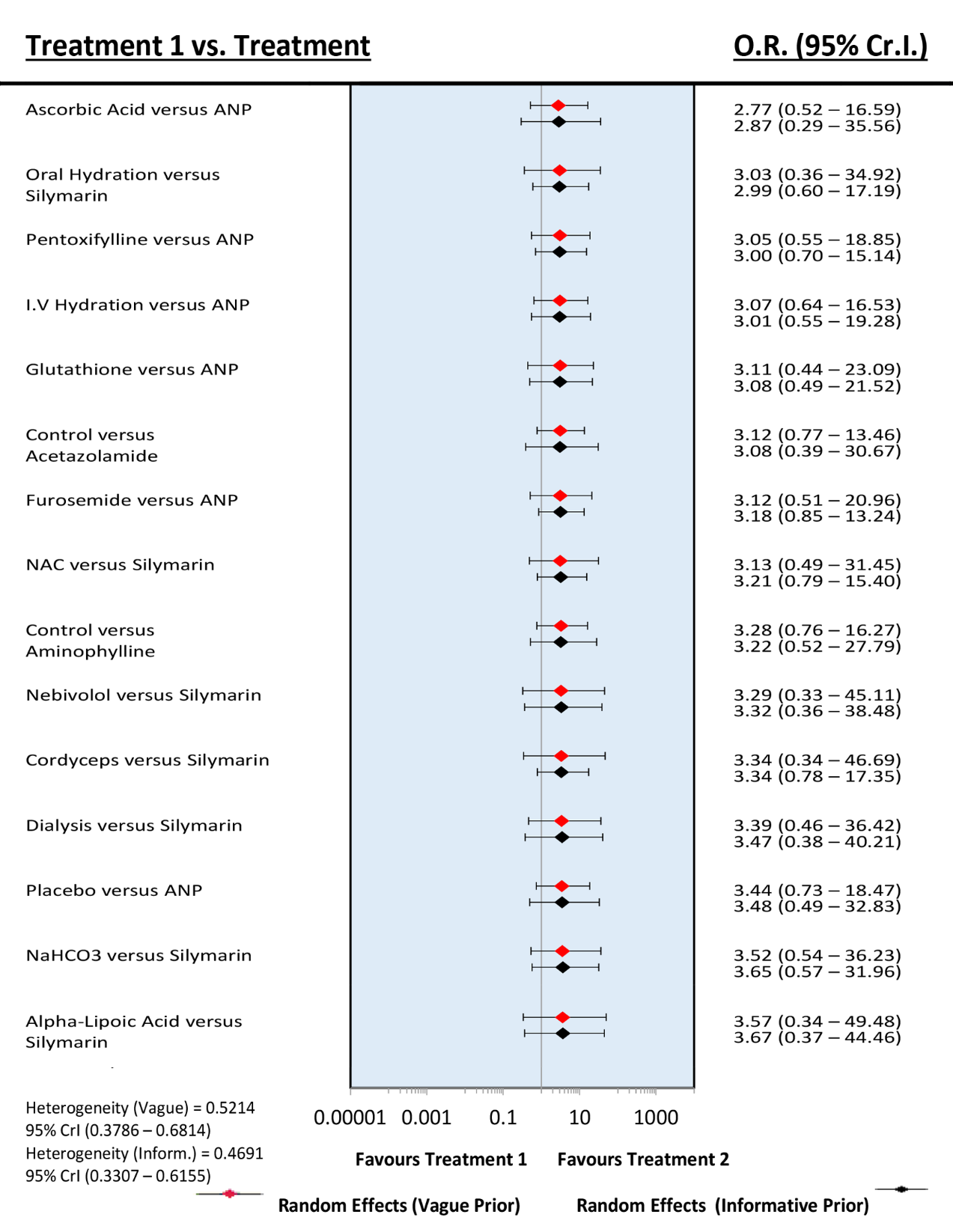


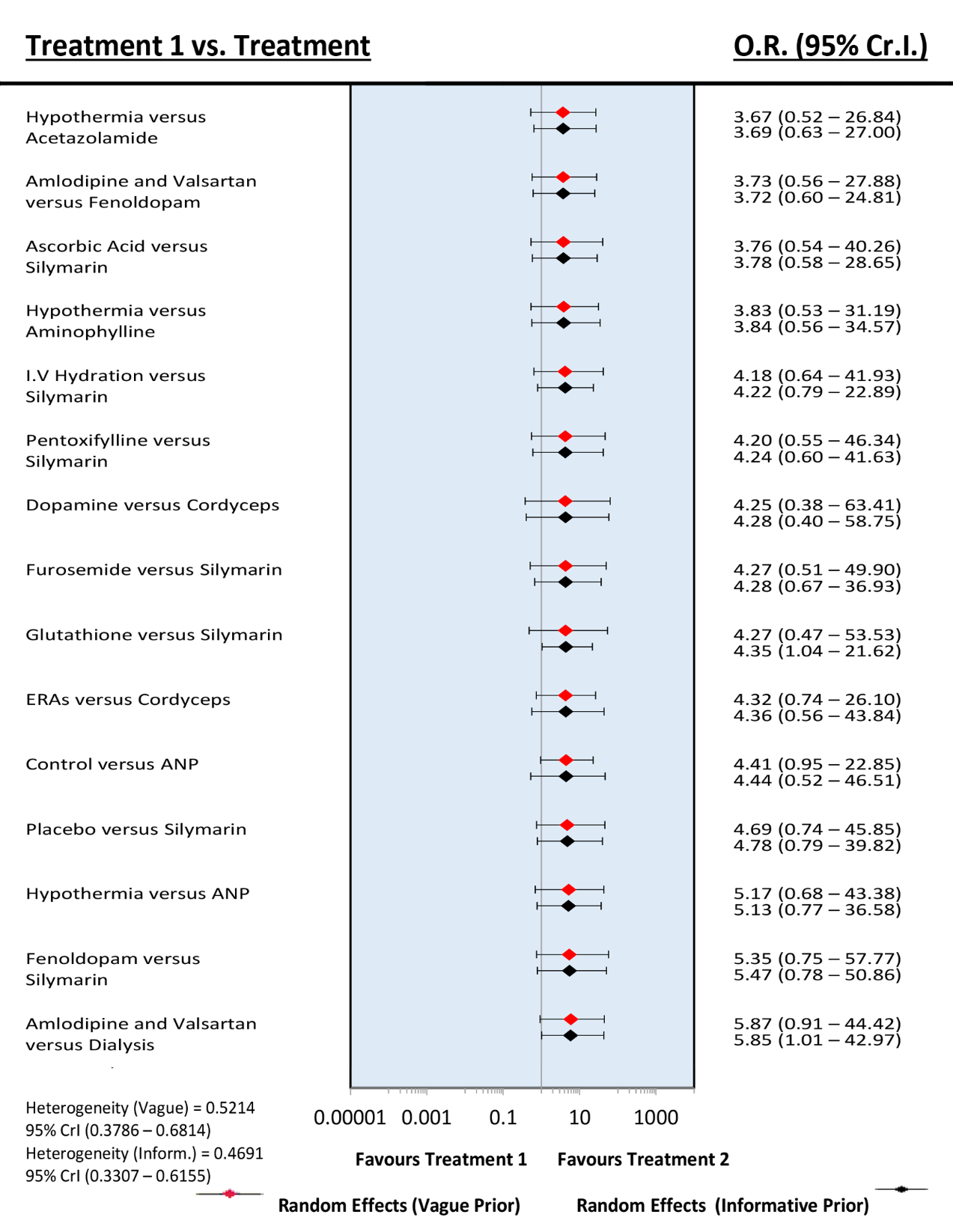


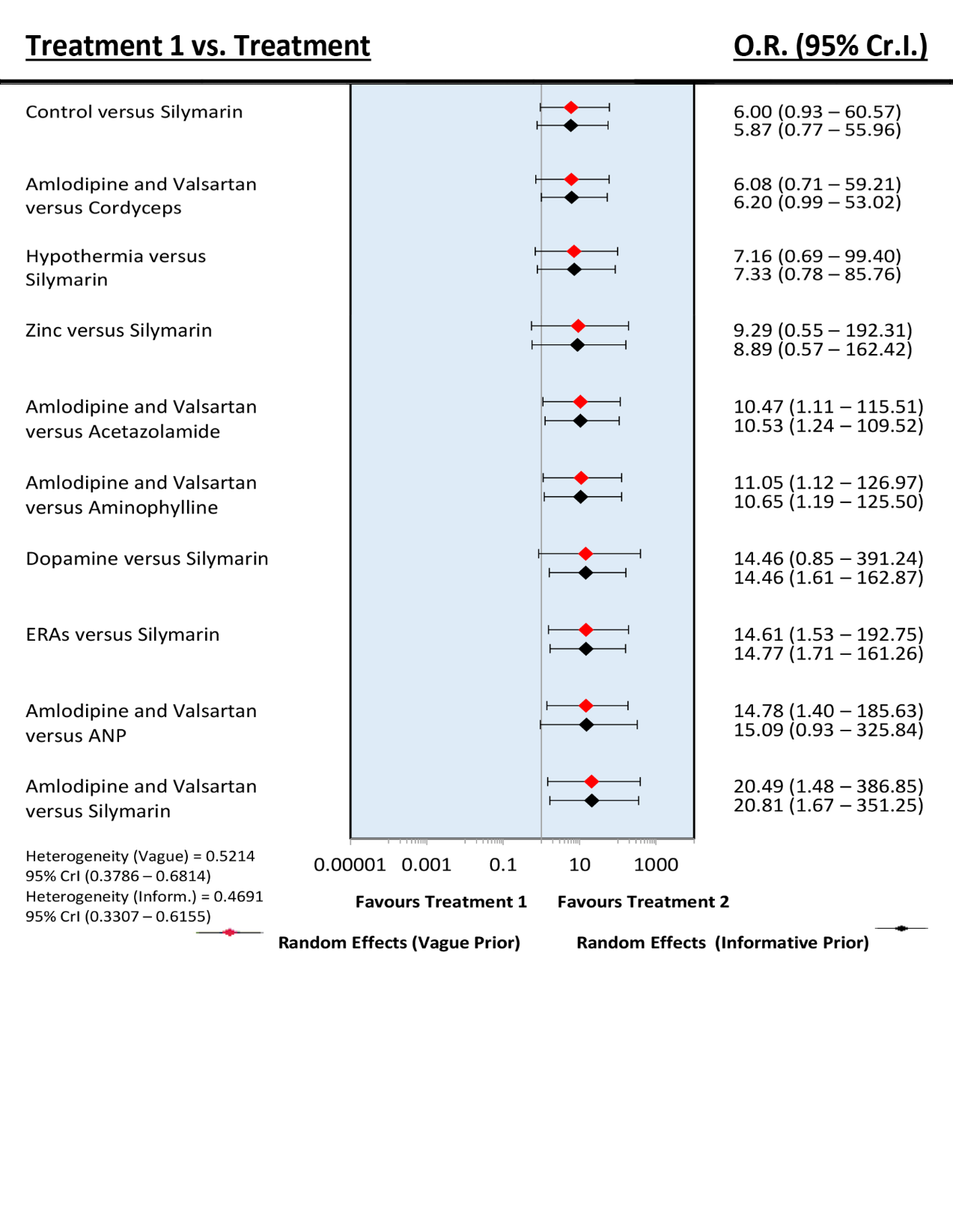


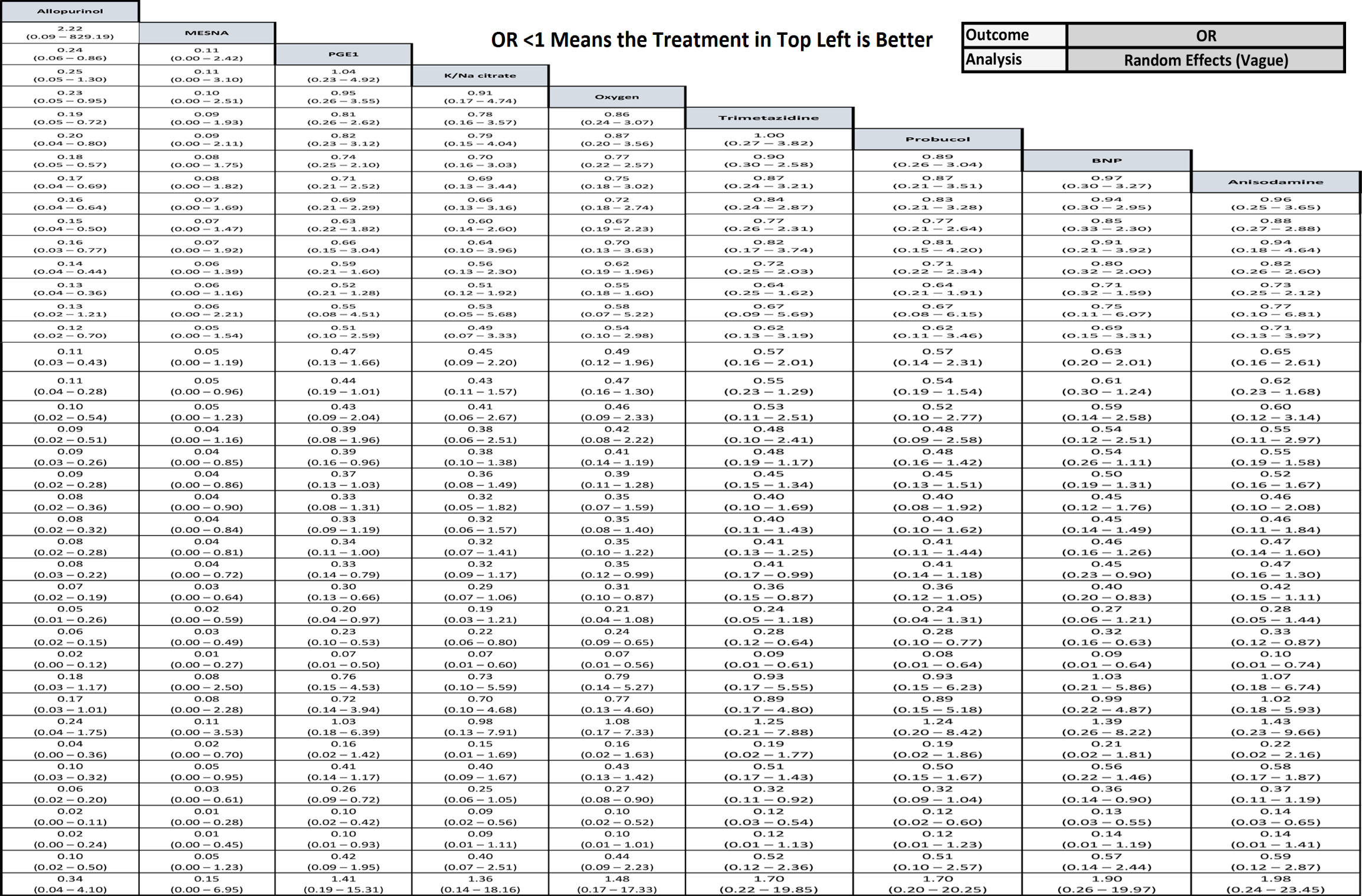


Figure 4 League Table


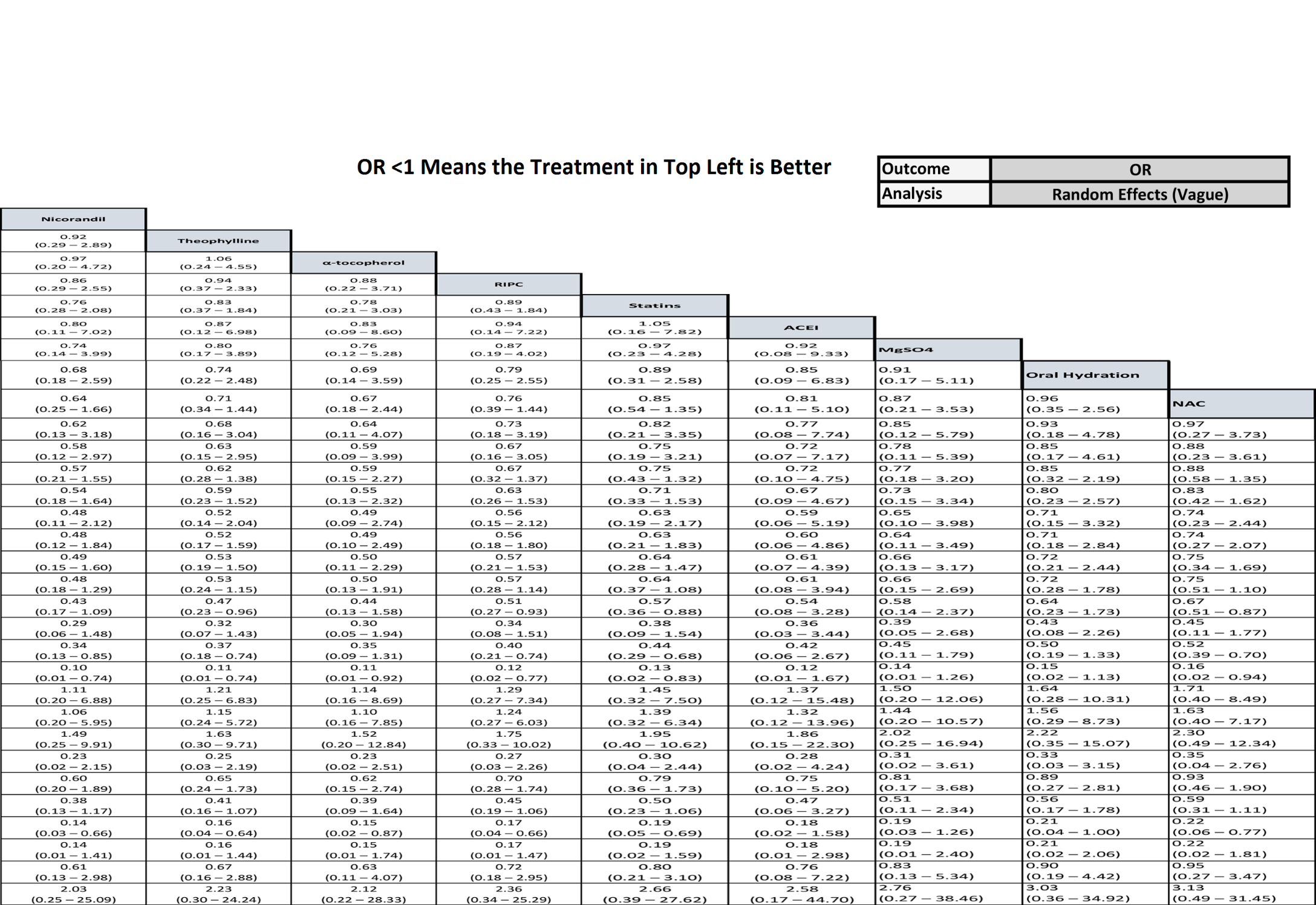


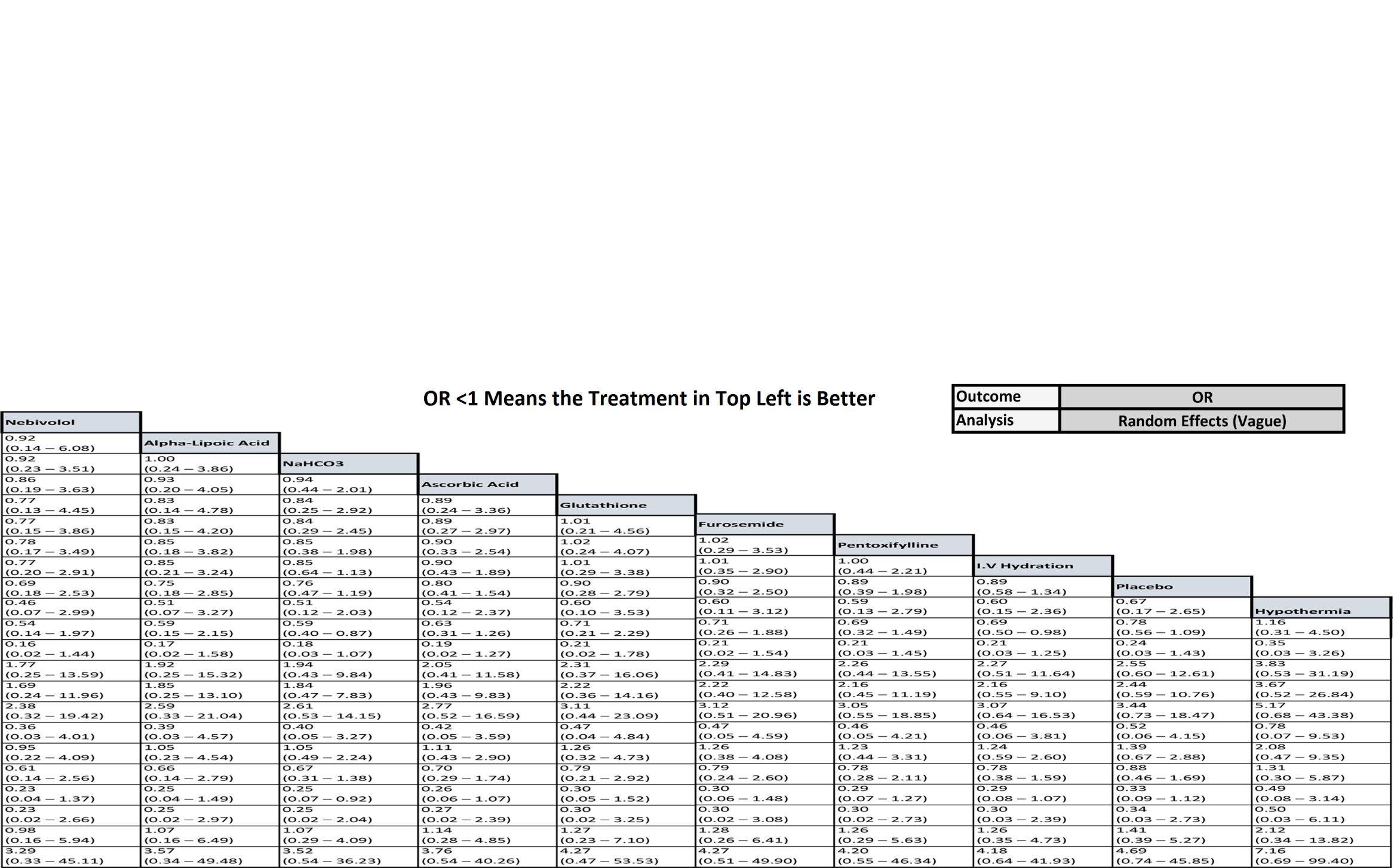


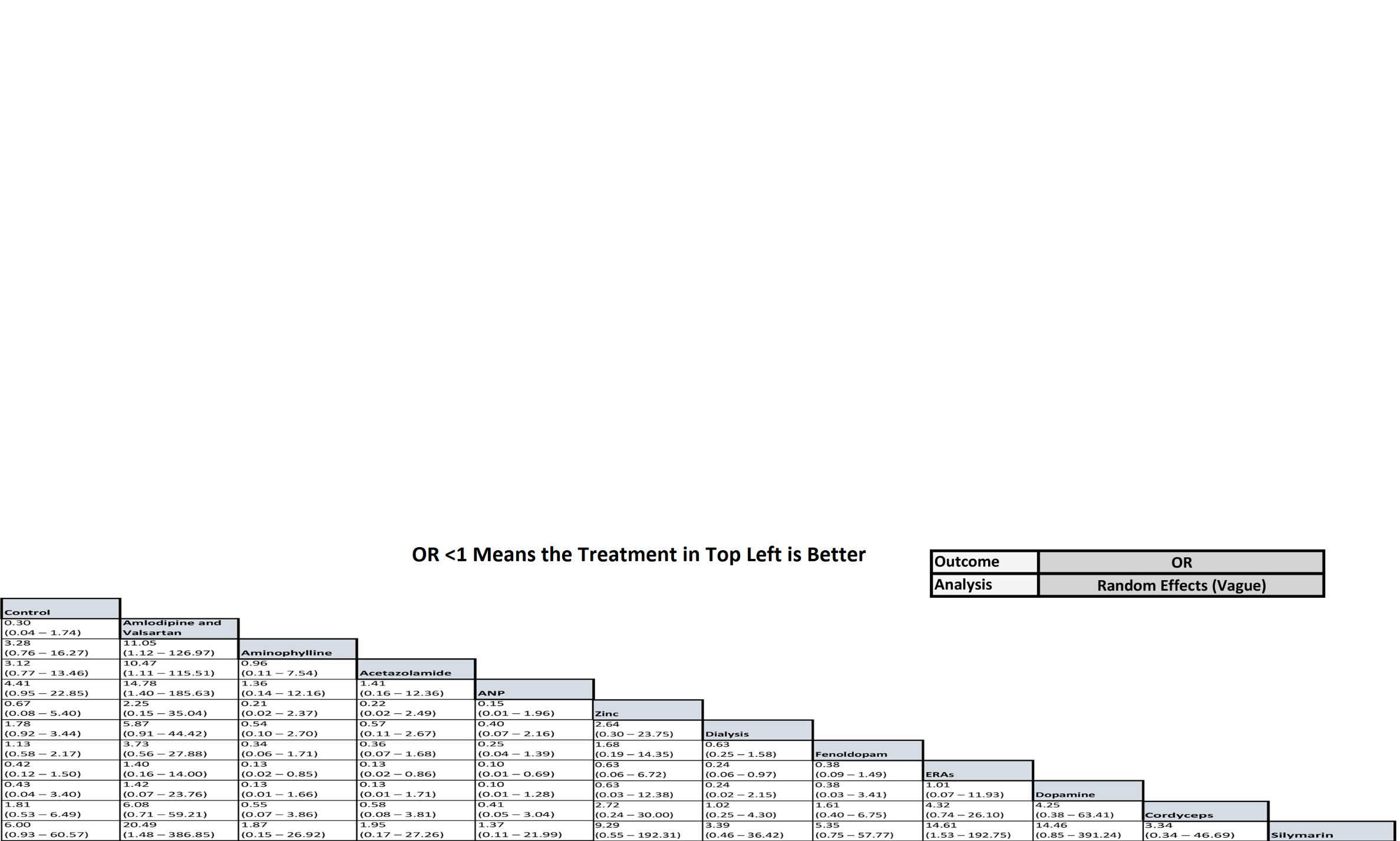


**Sub-Analysis after excluding studies with zero values**

Total Number of studies: 159 RCTs

Excluded studies with zero events (total 14)

| Study | Interventions included |
| --- | --- |
| Bilasy, 2012 | Placebo, Theophylline |
| Efrati, 2003 | Placebo, NAC |
| Erol, 2013 | Control, Allopurinol |
| Kinbara, 2010 | Control, NAC, Aminophylline |
| Kitzler, 2012 | Placebo, NAC, alpha tocopherol |
| Kotlyar, 2005 | Placebo, NAC |
| Kumar, 2014 (2 arms per contrast media) | NAC, Allopurinol, I.V Hydration |
| Ludwig, 2011 | Control, MESNA |
| Matejka, 2010 | Control, Theophylline |
| Moore, 2006 | Control, NAC |
| Saitoh, 2011 | Control, NAC, Glutathione |
| Sandhu, 2006 | Control, NAC |
| Sar, 2010 | Placebo, NAC |

Figures and Tables:

1. Network Diagram

2. Tables:

A. Network Characteristics

B. Interventions Characteristics

C Direct comparisons characteristics

3. Rankogram

4. Ranking and probability of being the best (table)

5. Forest Plot

6. R Graphical diagnostics (Trace & Density)

| Software | Spec | Convergence | Analysis |
| --- | --- | --- | --- |
| Netmetaxl / WinBUGS14 version 1.4.3 | Burn 5000  Sim 10000 | good convergence (FE MC error 5% of the SD) | Random Effects (Vague)  Random Effects (Informative) |
| GeMTC R | Burn 5000  Sim 20000 | good convergence | Random Effects (Vague) |


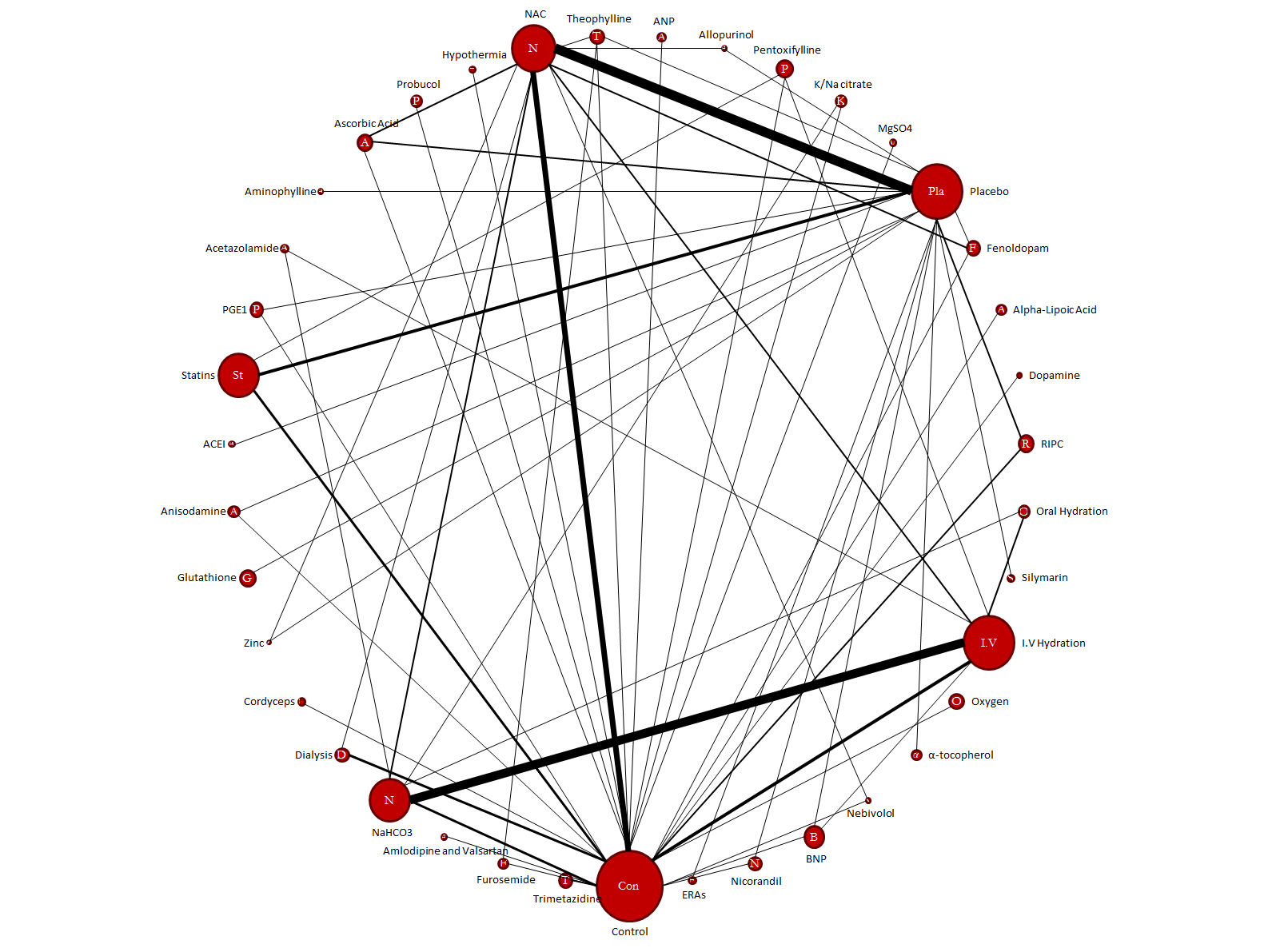


Figure 1 Network Diagram

Table 1 Network Characteristics

| **Characteristic** | **Number** |
| --- | --- |
| **Number of Interventions** | 39 |
| **Number of Studies** | 159 |
| **Total Number of Patients in Network** | 33,578 |
| **Total Number of Events in Network** | 3,560 |
| **Total Possible Pairwise Comparisons** | 741 |
| **Total Number Pairwise Comparisons With Direct Data** | 64 |
| **Number of Two-arm Studies** | 145 |
| **Number of Multi-Arms Studies** | 14 |
| **Number of Studies With No Zero Events** | 159 |
| **Number of Studies With At Least One Zero Event** | 0 |
| **Number of Studies with All Zero Events** | 0 |

Table 2 Interventions Characteristics

| **Treatment** | **# Studies** | **# Events** | **# Patients** | **Aggregate Rate** |
| --- | --- | --- | --- | --- |
| **I.V Hydration** | 36 | 482 | 4729 | 0.1019 |
| **Statins** | 14 | 123 | 3040 | 0.0405 |
| **Furosemide** | 2 | 16 | 166 | 0.0964 |
| **NAC** | 48 | 388 | 3453 | 0.1124 |
| **Trimetazidine** | 4 | 17 | 352 | 0.0483 |
| **NaHCO3** | 29 | 246 | 2938 | 0.0837 |
| **PGE1** | 4 | 24 | 304 | 0.0789 |
| **MgSO4** | 1 | 9 | 62 | 0.1452 |
| **Pentoxifylline** | 4 | 30 | 438 | 0.0685 |
| **Placebo** | 54 | 681 | 4693 | 0.1451 |
| **Control** | 70 | 1035 | 7898 | 0.1310 |
| **Allopurinol** | 1 | 5 | 30 | 0.1667 |
| **BNP** | 4 | 52 | 744 | 0.0699 |
| **Probucol** | 2 | 12 | 198 | 0.0606 |
| **α-tocopherol** | 1 | 10 | 149 | 0.0671 |
| **Oxygen** | 2 | 33 | 346 | 0.0954 |
| **Amlodipine and Valsartan** | 1 | 8 | 45 | 0.1778 |
| **K/Na citrate** | 2 | 6 | 203 | 0.0296 |
| **Nicorandil** | 3 | 15 | 291 | 0.0515 |
| **Ascorbic Acid** | 6 | 41 | 434 | 0.0945 |
| **Alpha-Lipoic Acid** | 2 | 6 | 139 | 0.0432 |
| **Oral Hydration** | 4 | 14 | 228 | 0.0614 |
| **Nebivolol** | 1 | 8 | 40 | 0.2000 |
| **Anisodamine** | 2 | 17 | 192 | 0.0885 |
| **RIPC** | 8 | 33 | 458 | 0.0721 |
| **Theophylline** | 5 | 18 | 323 | 0.0557 |
| **Hypothermia** | 1 | 14 | 58 | 0.2414 |
| **Glutathione** | 1 | 21 | 414 | 0.0507 |
| **ACEI** | 1 | 3 | 52 | 0.0577 |
| **Aminophylline** | 1 | 4 | 30 | 0.1333 |
| **Acetazolamide** | 1 | 5 | 94 | 0.0532 |
| **ANP** | 1 | 4 | 126 | 0.0317 |
| **Zinc** | 1 | 3 | 18 | 0.1667 |
| **Dialysis** | 5 | 43 | 293 | 0.1468 |
| **Fenoldopam** | 5 | 78 | 333 | 0.2342 |
| **ERAs** | 1 | 43 | 77 | 0.5584 |
| **Dopamine** | 1 | 4 | 33 | 0.1212 |
| **Cordyceps** | 2 | 7 | 88 | 0.0795 |
| **Silymarin** | 1 | 2 | 69 | 0.0290 |

Table 3 Direct comparisons characteristics

| **Comparison** | **# Studies** | **# Patients** | **# Events** |
| --- | --- | --- | --- |
| **Statins vs. Control** | 6 | 4,382 | 200 |
| **NaHCO3 vs. K/Na citrate** | 1 | 206 | 4 |
| **I.V Hydartion vs. NaHCO3** | 23 | 5,113 | 471 |
| **I.V Hydartion vs. Oral Hydration** | 4 | 456 | 33 |
| **NAC vs. Placebo** | 27 | 4,254 | 588 |
| **NAC vs. Ascorbic Acid** | 3 | 583 | 88 |
| **Placebo vs. Ascorbic Acid** | 4 | 638 | 105 |
| **NAC vs. Control** | 14 | 1,836 | 228 |
| **NAC vs. Fenoldopam** | 3 | 359 | 44 |
| **Control vs. Fenoldopam** | 2 | 123 | 26 |
| **I.V Hydartion vs. Control** | 8 | 2,884 | 396 |
| **NAC vs. Theophylline** | 1 | 62 | 13 |
| **Statins vs. Placebo** | 7 | 1,508 | 125 |
| **NaHCO3 vs. Oral Hydration** | 1 | 43 | 3 |
| **Control vs. Alpha-Lipoic Acid** | 2 | 280 | 16 |
| **Furosemide vs. Control** | 2 | 326 | 35 |
| **Furosemide vs. Theophylline** | 1 | 159 | 18 |
| **Control vs. Theophylline** | 2 | 437 | 24 |
| **Control vs. RIPC** | 4 | 486 | 55 |
| **Placebo vs. Theophylline** | 2 | 164 | 15 |
| **Pentoxifylline vs. Control** | 2 | 461 | 46 |
| **Placebo vs. Nicorandil** | 1 | 240 | 29 |
| **MgSO4 vs. Control** | 1 | 126 | 26 |
| **Control vs. Dopamine** | 1 | 66 | 6 |
| **Control vs. Anisodamine** | 1 | 260 | 39 |
| **NAC vs. Nebivolol** | 1 | 80 | 17 |
| **Control vs. Nebivolol** | 1 | 80 | 19 |
| **NAC vs. NaHCO3** | 3 | 252 | 58 |
| **Placebo vs. RIPC** | 4 | 427 | 43 |
| **Control vs. Cordyceps** | 2 | 180 | 19 |
| **I.V Hydartion vs. NAC** | 3 | 331 | 40 |
| **Statins vs. Pentoxifylline** | 1 | 220 | 9 |
| **NAC vs. Zinc** | 1 | 37 | 4 |
| **Placebo vs. Zinc** | 1 | 35 | 5 |
| **Control vs. Nicorandil** | 2 | 341 | 22 |
| **PGE1 vs. Placebo** | 2 | 392 | 74 |
| **NaHCO3 vs. Control** | 5 | 806 | 95 |
| **Control vs. Dialysis** | 5 | 588 | 112 |
| **Control vs. Probucol** | 2 | 409 | 50 |
| **Placebo vs. ACEI** | 1 | 114 | 9 |
| **PGE1 vs. Control** | 2 | 226 | 16 |
| **Control vs. BNP** | 1 | 209 | 23 |
| **I.V Hydartion vs. BNP** | 2 | 1,128 | 113 |
| **Trimetazidine vs. Control** | 4 | 714 | 71 |
| **Control vs. K/Na citrate** | 1 | 202 | 25 |
| **Control vs. Oxygen** | 2 | 697 | 115 |
| **Control vs. ANP** | 1 | 254 | 19 |
| **Control vs. Amlodipine and Valsartan** | 1 | 90 | 11 |
| **I.V Hydartion vs. Acetazolamide** | 1 | 190 | 21 |
| **NaHCO3 vs. Acetazolamide** | 1 | 190 | 9 |
| **NAC vs. Dialysis** | 1 | 275 | 11 |
| **Placebo vs. α-tocopherol** | 1 | 298 | 31 |
| **Placebo vs. Aminophylline** | 1 | 60 | 10 |
| **NAC vs. Allopurinol** | 1 | 65 | 12 |
| **Placebo vs. Allopurinol** | 1 | 60 | 16 |
| **Placebo vs. Silymarin** | 1 | 143 | 10 |
| **Placebo vs. Fenoldopam** | 1 | 283 | 90 |
| **Control vs. Hypothermia** | 1 | 128 | 29 |
| **Placebo vs. ERAs** | 1 | 158 | 67 |
| **Placebo vs. Glutathione** | 1 | 825 | 41 |
| **Placebo vs. Anisodamine** | 1 | 126 | 17 |
| **I.V Hydartion vs. Pentoxifylline** | 1 | 199 | 12 |
| **Placebo vs. BNP** | 1 | 149 | 36 |
| **Control vs. Ascorbic Acid** | 1 | 156 | 10 |


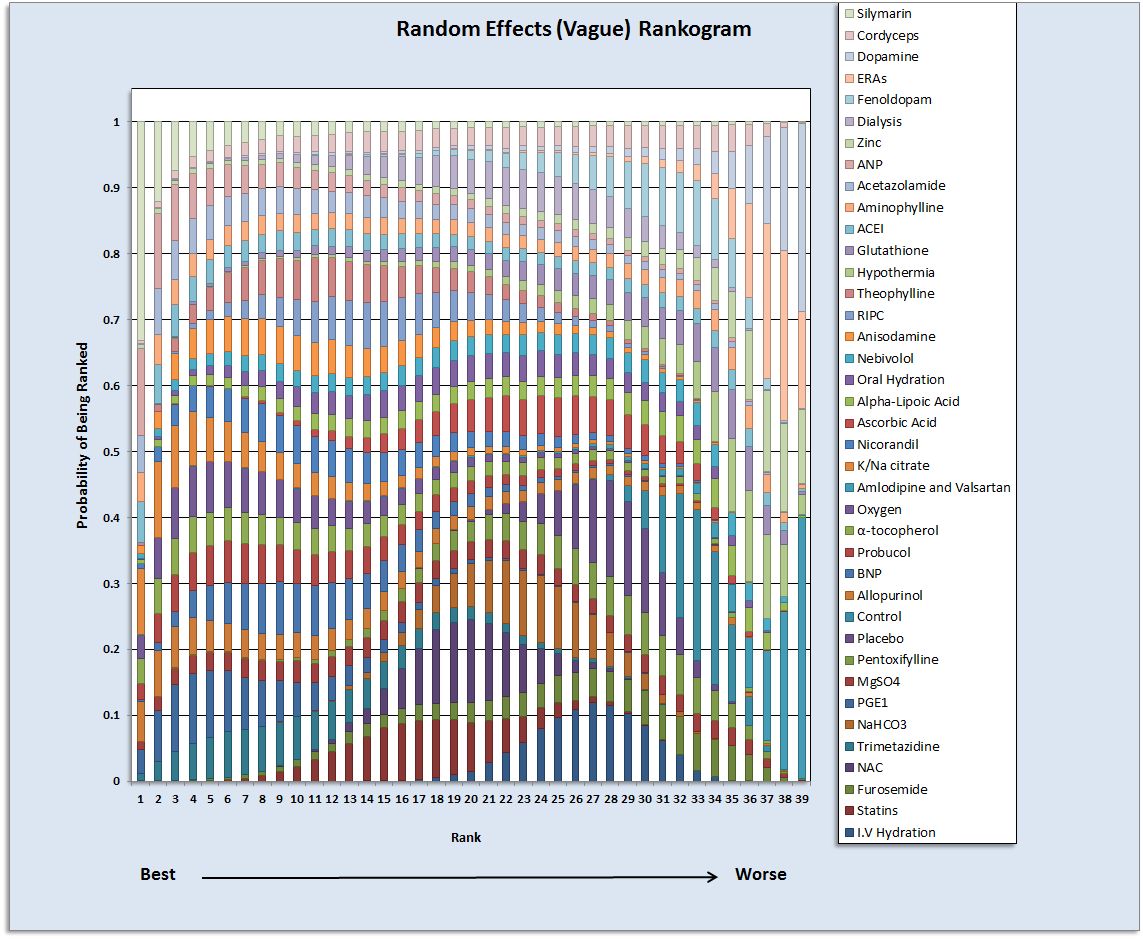


Figure 2 Rankogram: ranking the interventions for the probability of being the best, the interventions are colour coded; the first column represent the chance of being first best and 2nd column is the chance of being 2nd best and so on. The overall numerical value is presented in table 4

Table 4 Interventions ranking the treatments names column follow the league table (which arranges the presentation of summary estimates by ranking the treatments in order of most pronounced impact on the outcome under consideration) the numerical values represents the cumulative results of the probability of being best in which the highest score is 1 or 100% (see Rankogram)

| **Treatment** | **SUCRA** | **Treatment** | **SUCRA** |
| --- | --- | --- | --- |
| **PGE1** | 0.8322 | **NaHCO3** | 0.416 |
| **K/Na citrate** | 0.8032 | **Ascorbic Acid** | 0.4073 |
| **Oxygen** | 0.7792 | **Furosemide** | 0.3487 |
| **Trimetazidine** | 0.7389 | **Pentoxifylline** | 0.3387 |
| **BNP** | 0.7305 | **I.V Hydration** | 0.3228 |
| **Probucol** | 0.7292 | **Glutathione** | 0.3226 |
| **Anisodamine** | 0.6905 | **Placebo** | 0.2877 |
| **Allopurinol** | 0.6883 | **Hypothermia** | 0.1948 |
| **Nicorandil** | 0.6833 | **Control** | 0.1618 |
| **α-tocopherol** | 0.6807 | **Amlodipine and Valsartan** | 0.05371 |
| **Theophylline** | 0.6705 | **Acetazolamide** | 0.6908 |
| **RIPC** | 0.6237 | **ANP** | 0.7743 |
| **ACEI** | 0.5877 | **Zinc** | 0.2194 |
| **Statins** | 0.5666 | **Dialysis** | 0.4361 |
| **Aminophylline** | 0.5436 | **Fenoldopam** | 0.244 |
| **MgSO4** | 0.526 | **ERAs** | 0.07035 |
| **Oral Hydration** | 0.5081 | **Dopamine** | 0.1166 |
| **NAC** | 0.5055 | **Cordyceps** | 0.4561 |
| **Nebivolol** | 0.475 | **Silymarin** | 0.8378 |
| **Alpha-Lipoic Acid** | 0.4373 |  |  |
| ***Analysis*** | **Random Effects (Vague)** | | |

Figure 3 Forest Plot


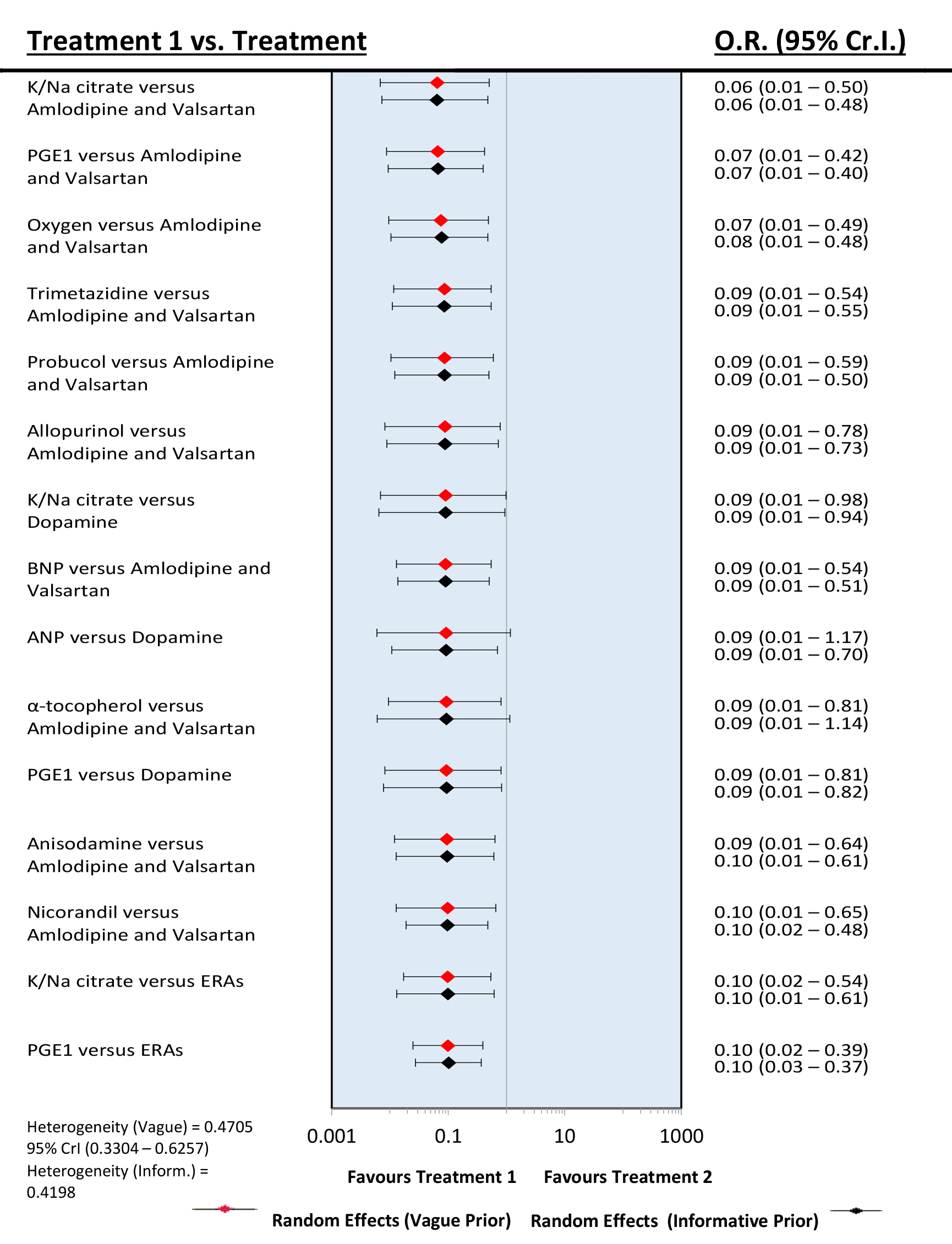


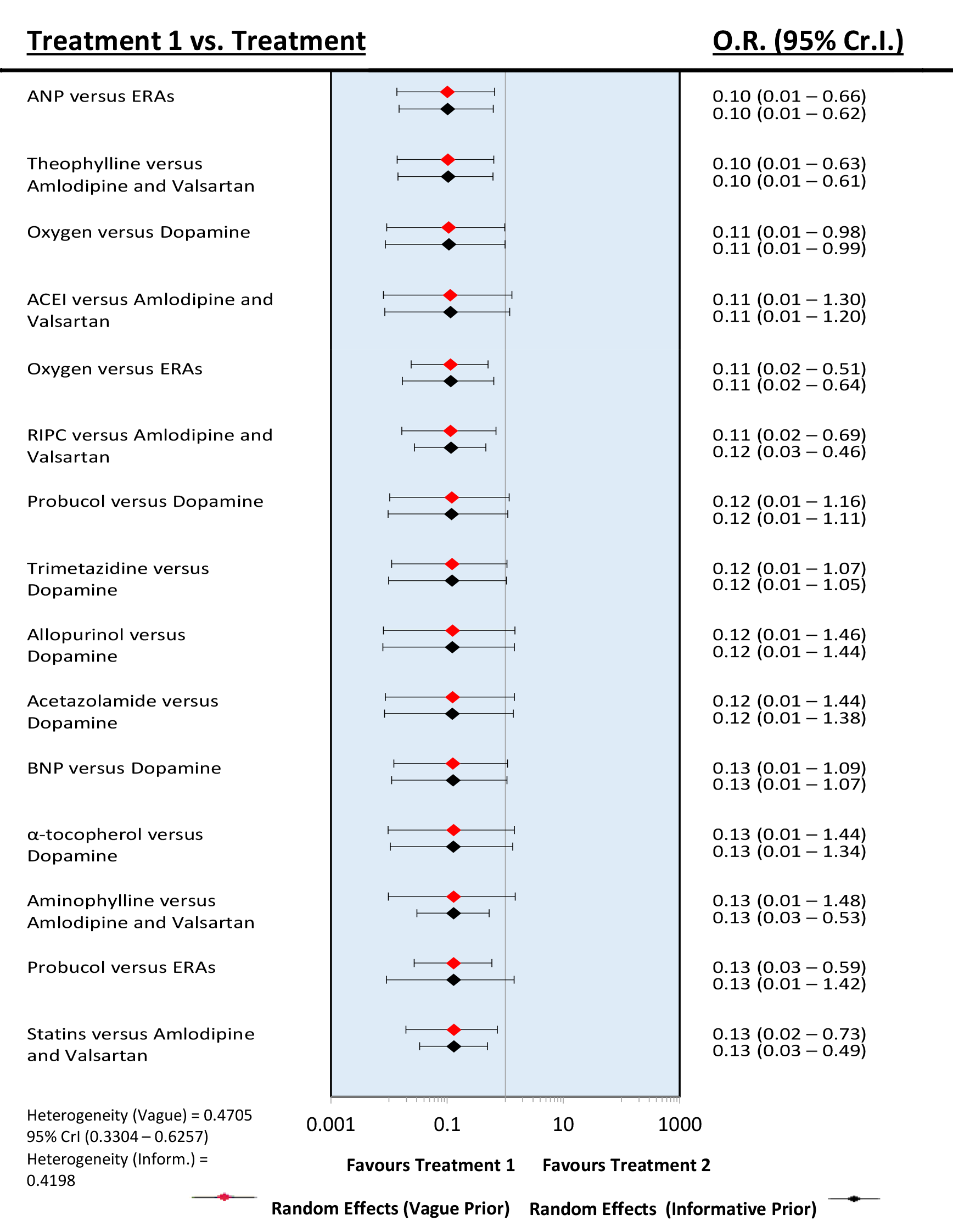


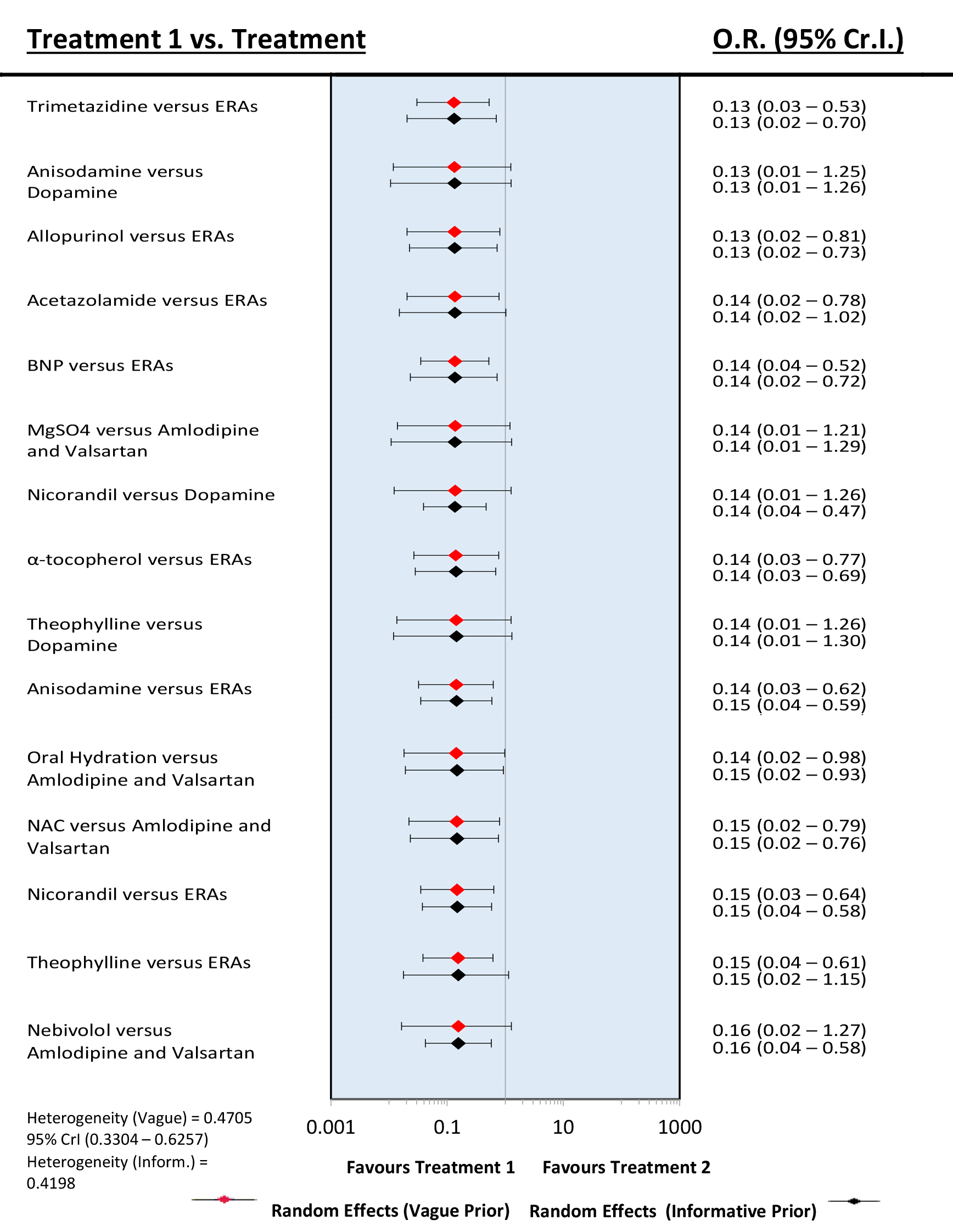


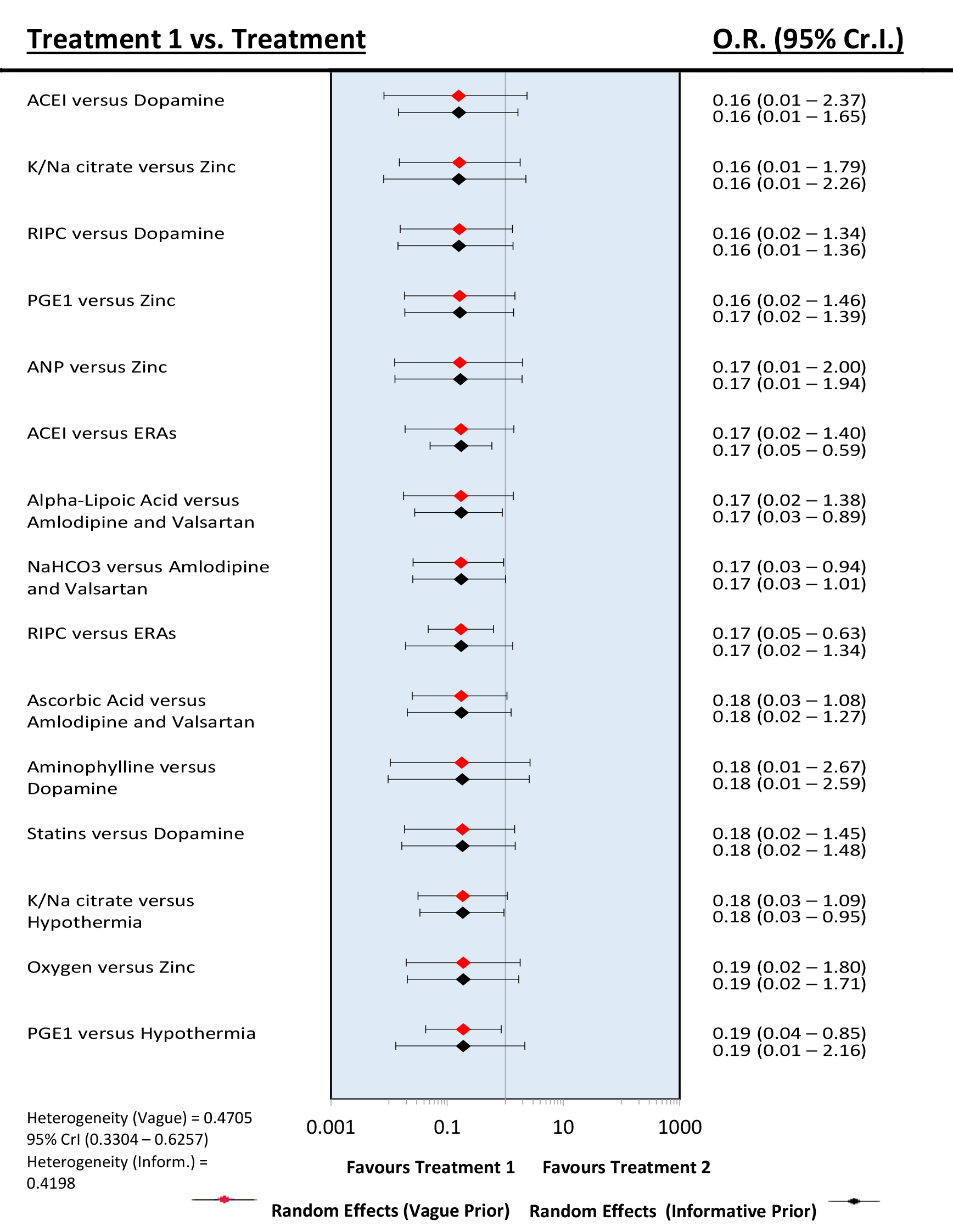


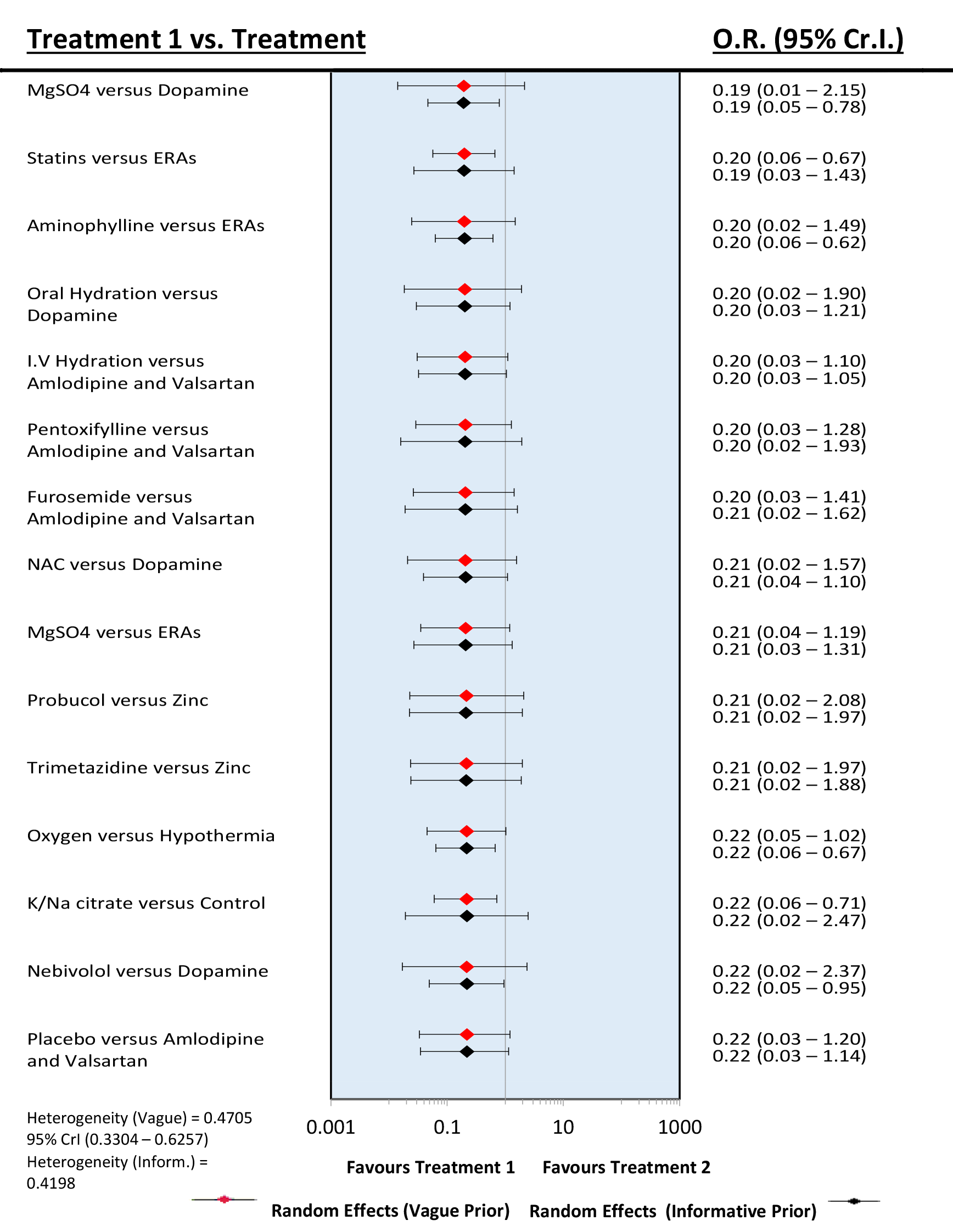


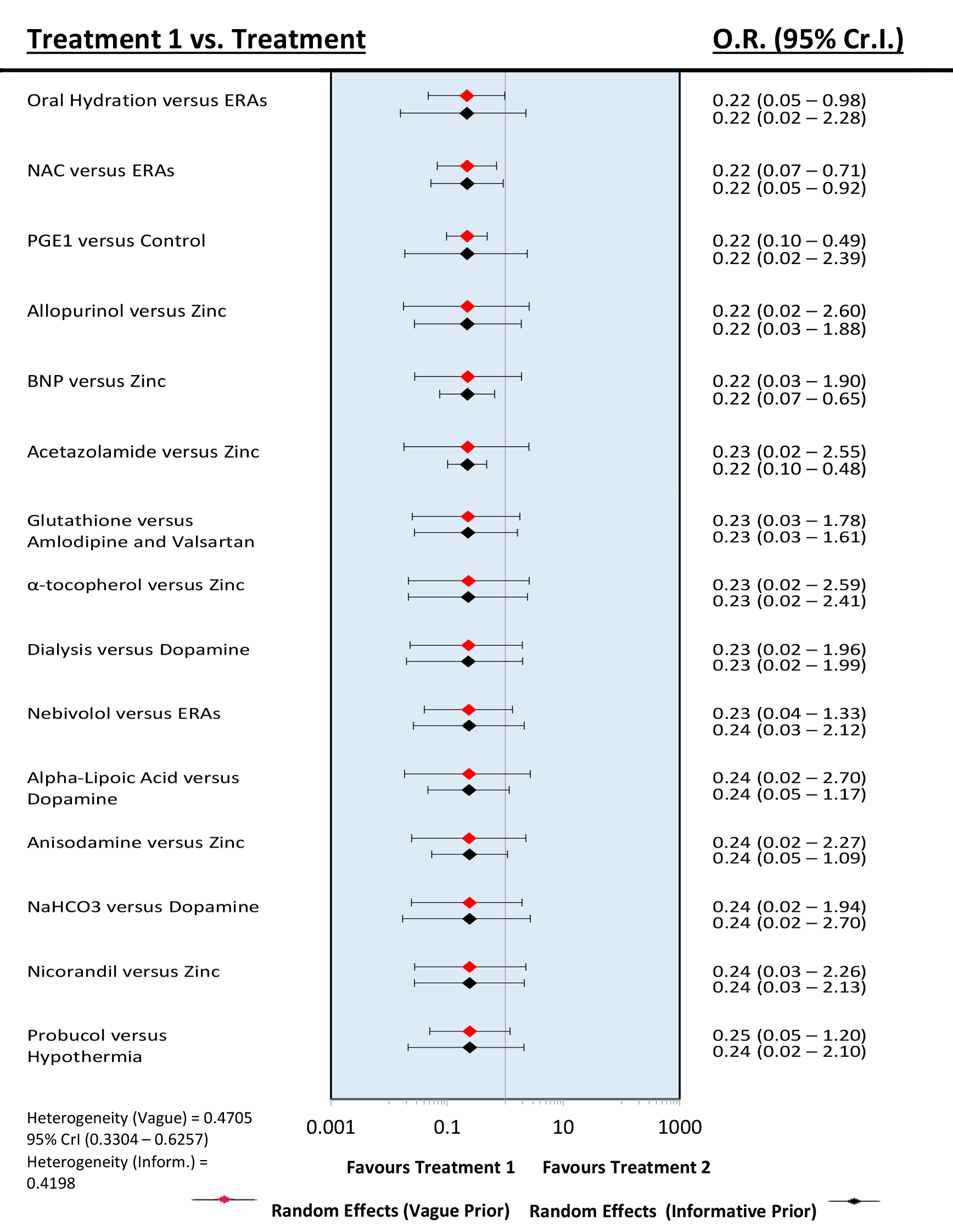


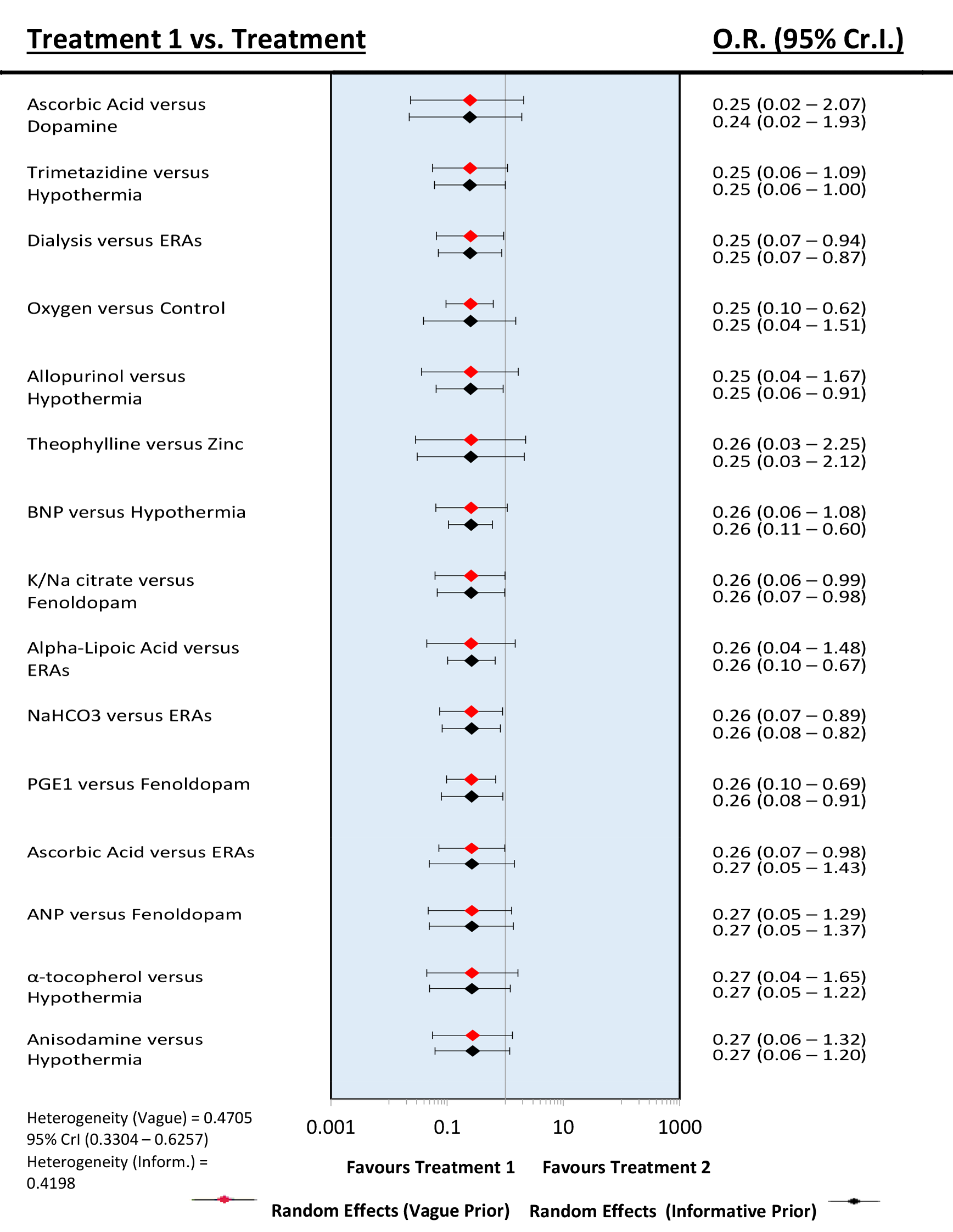


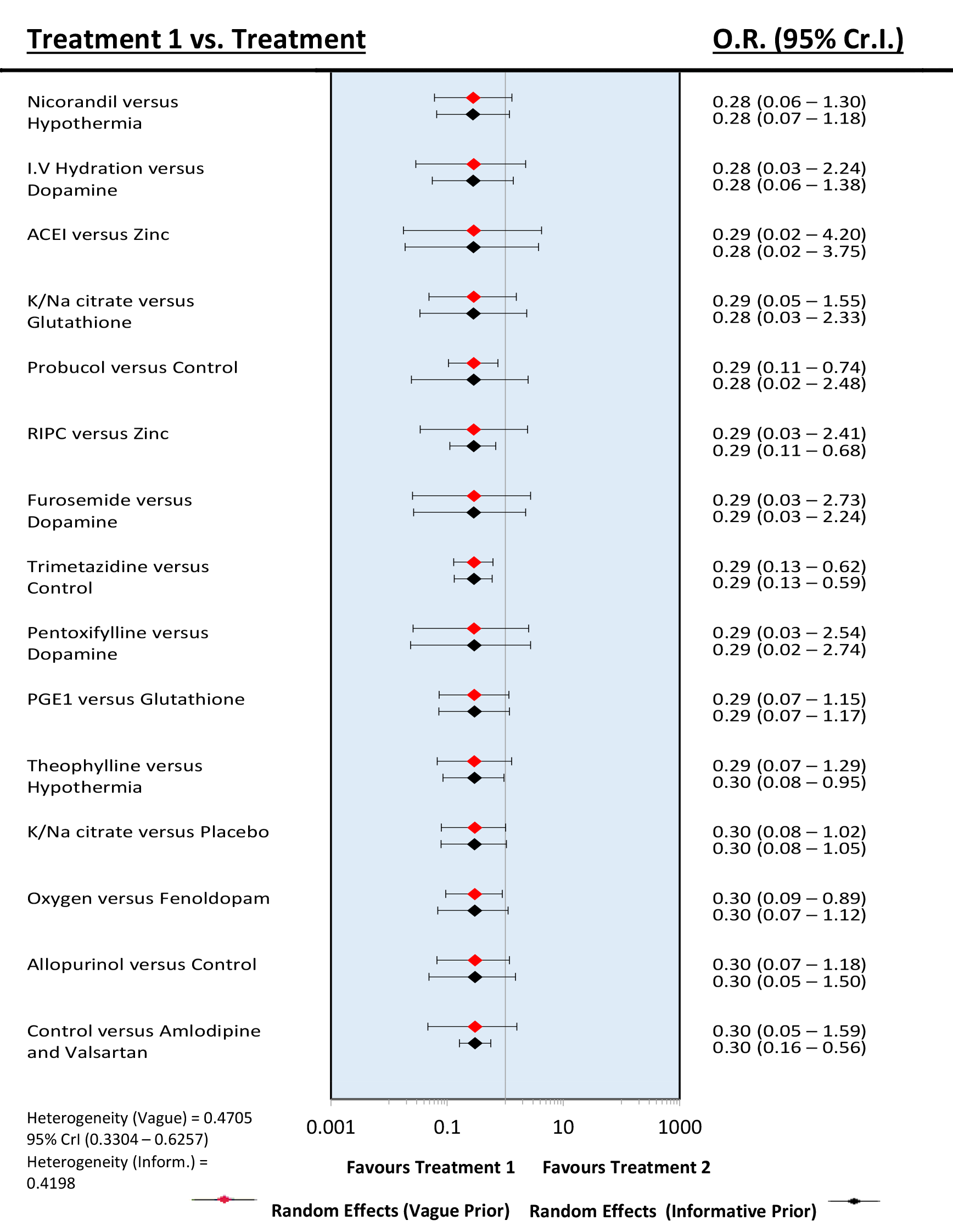


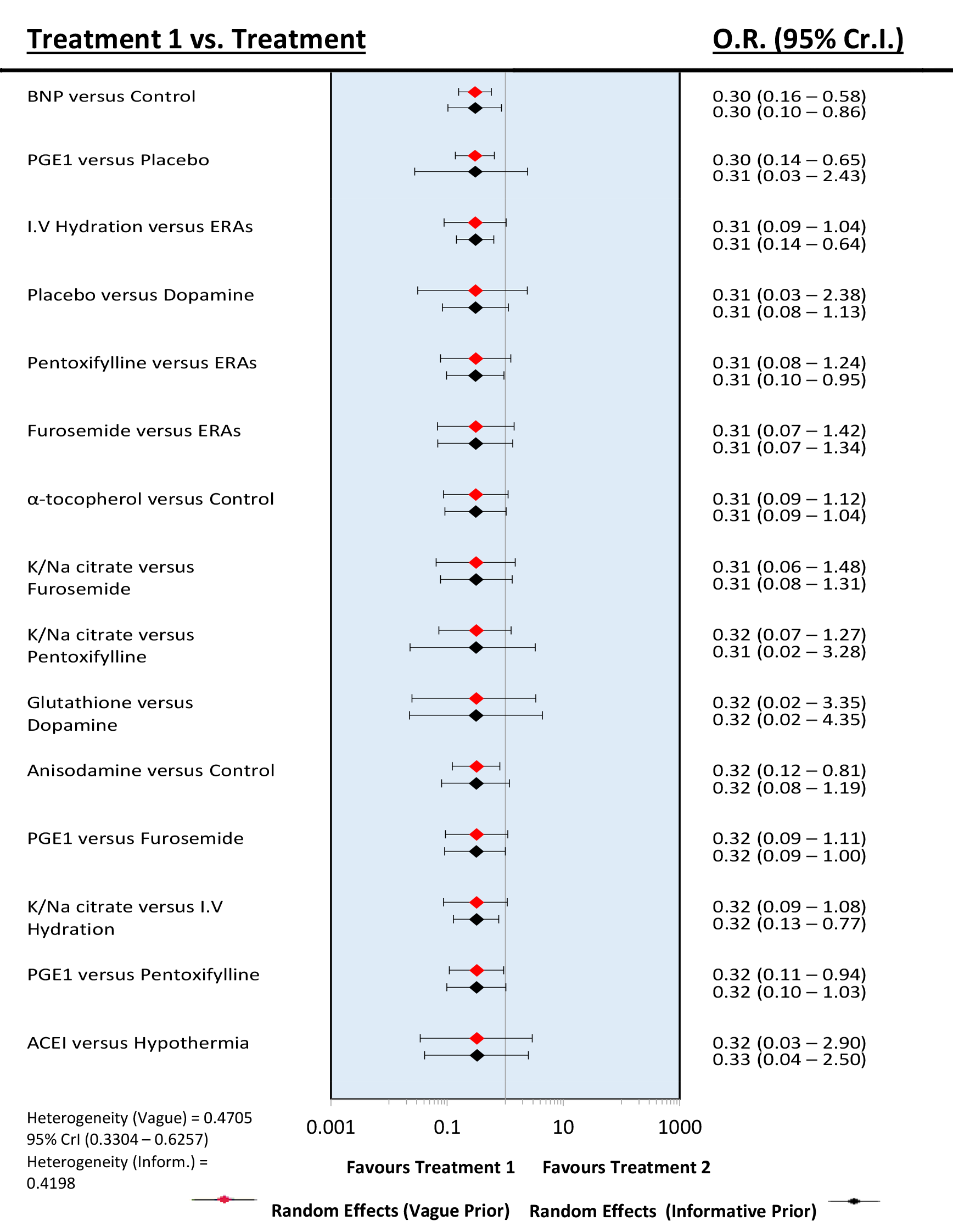


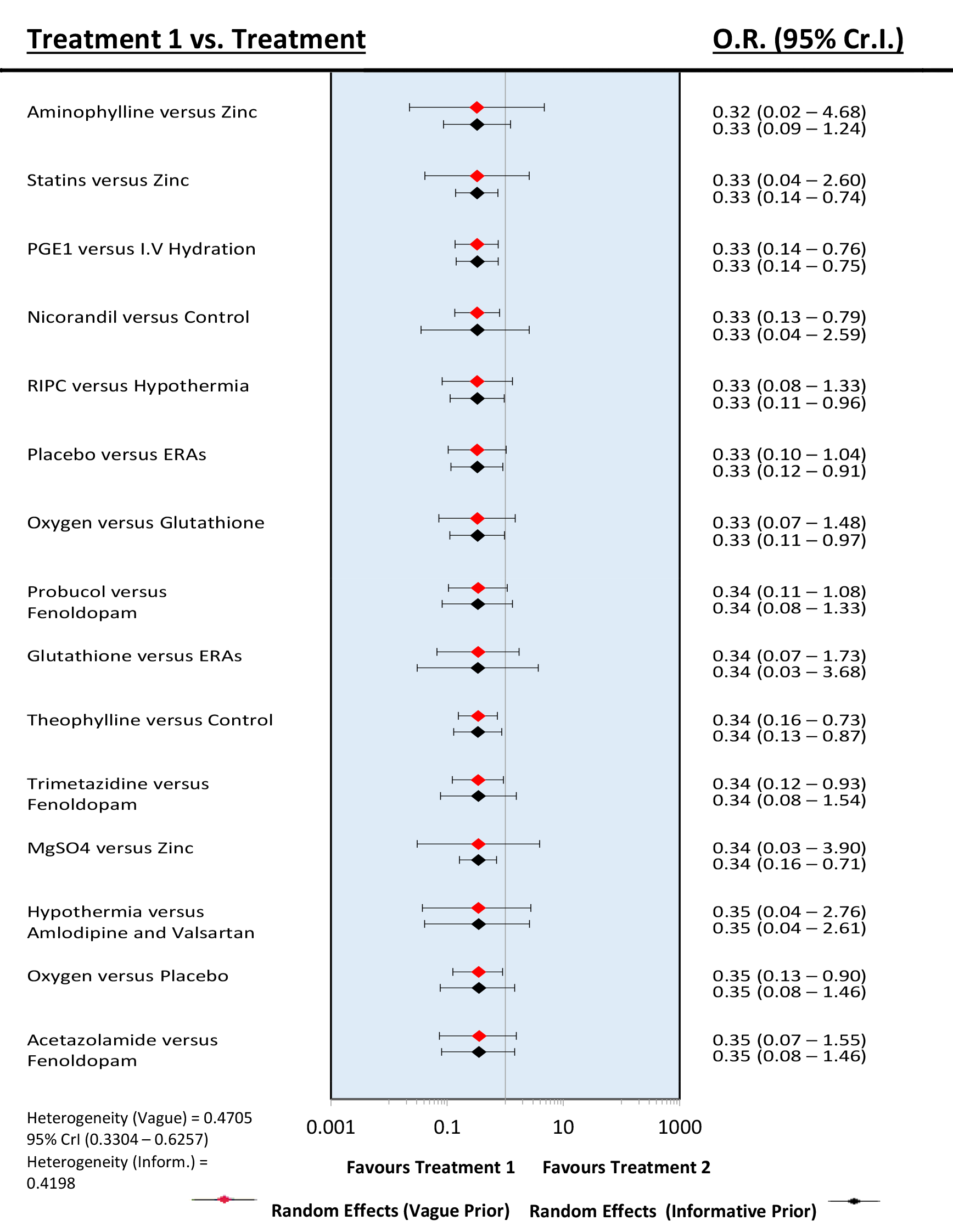


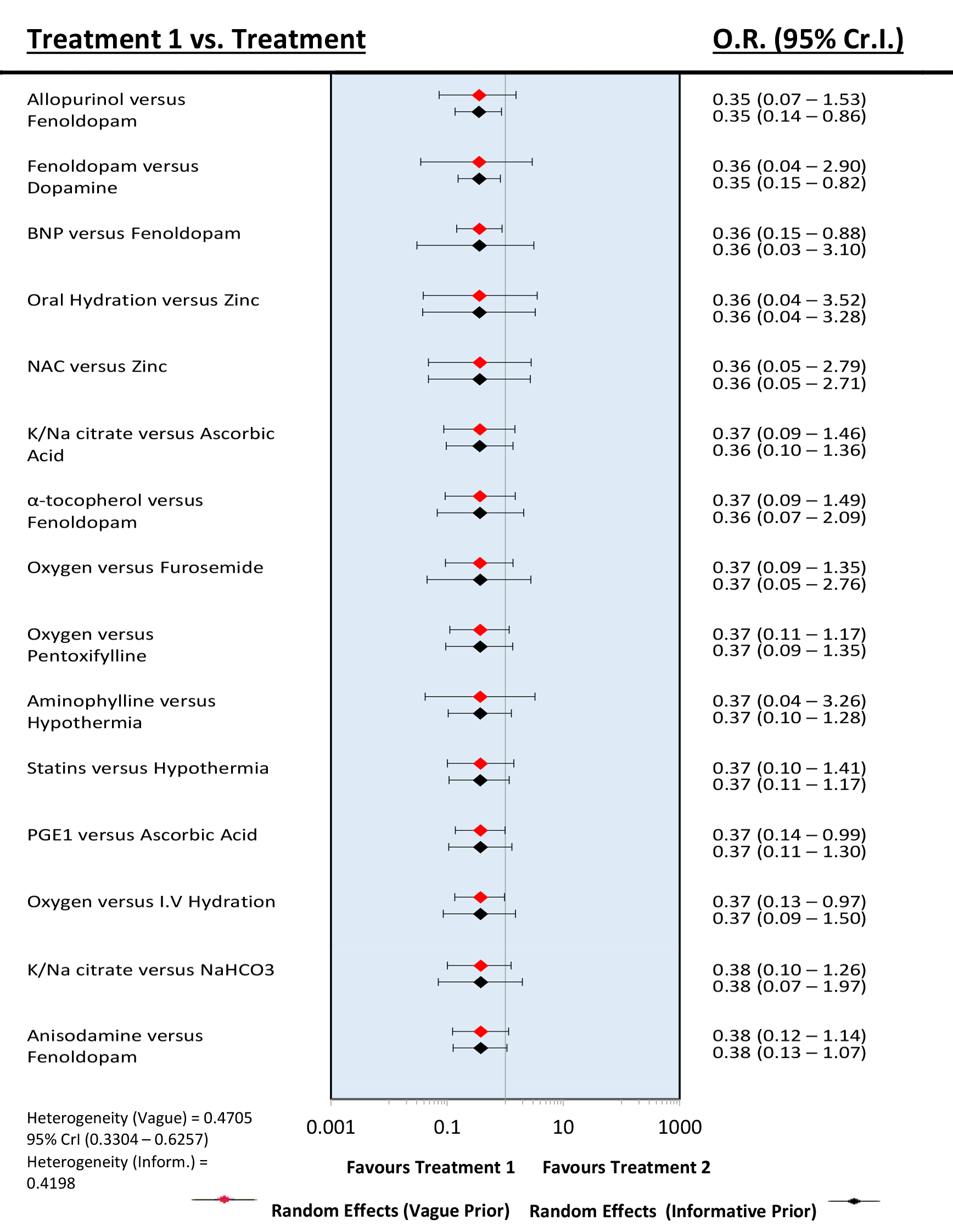


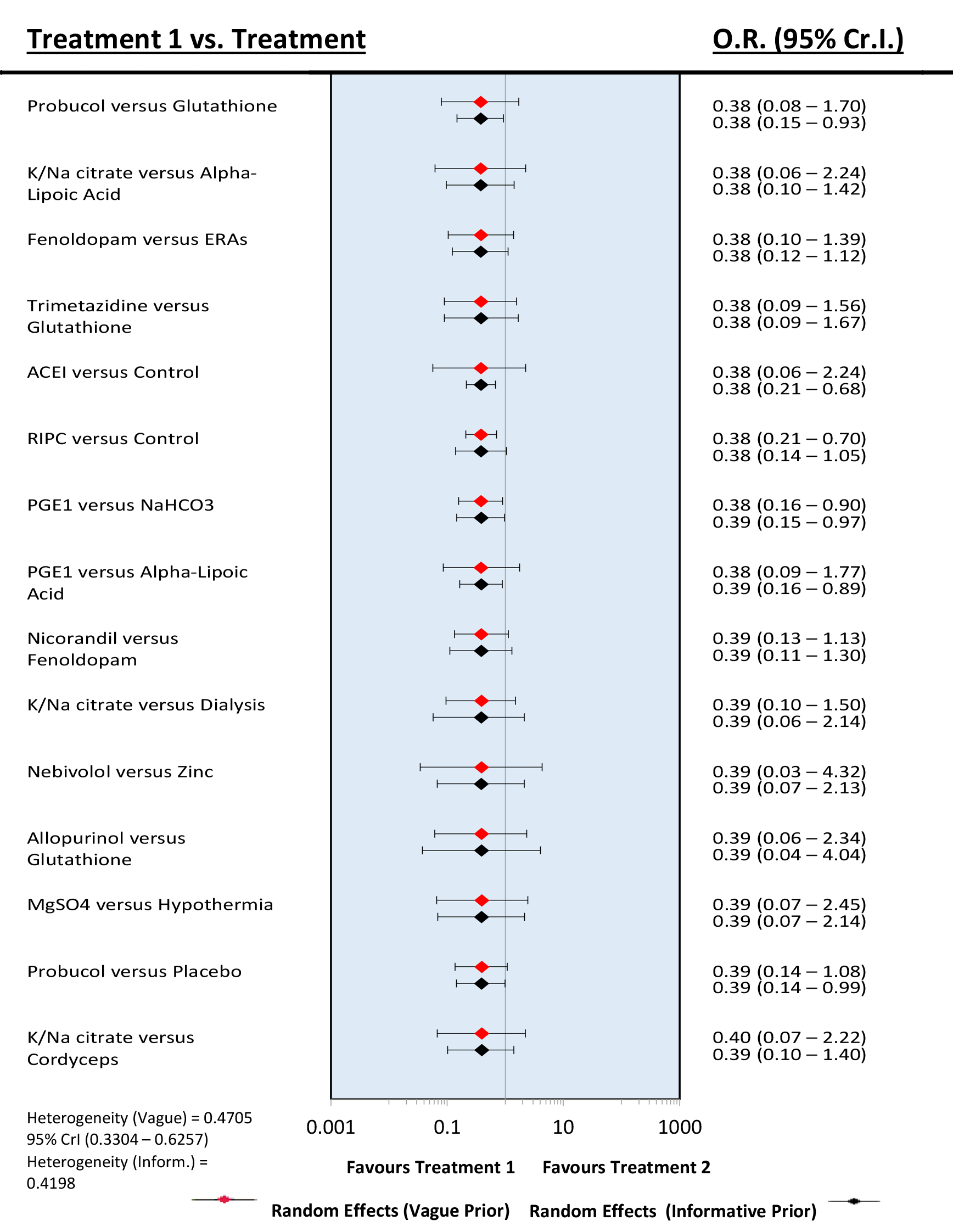


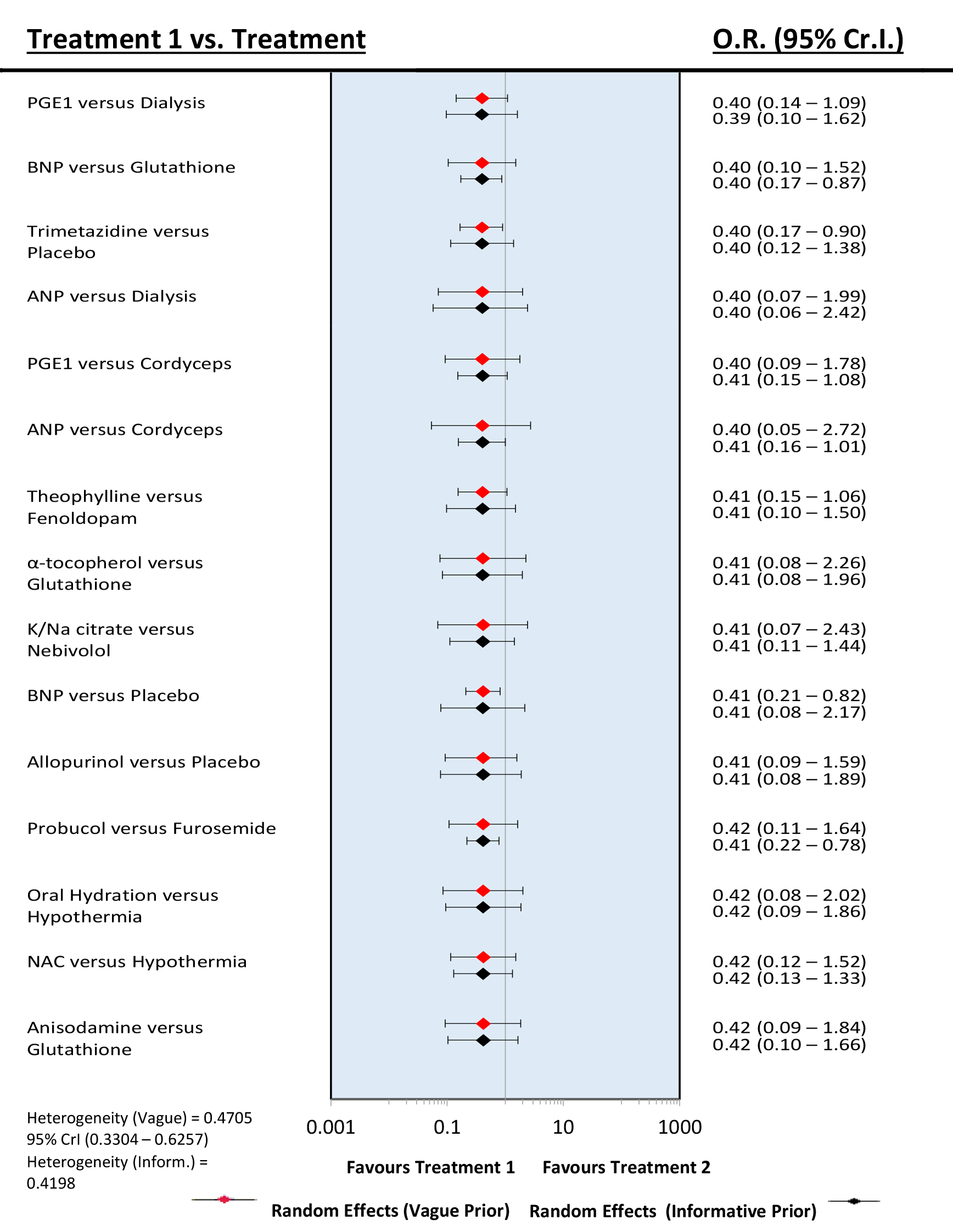


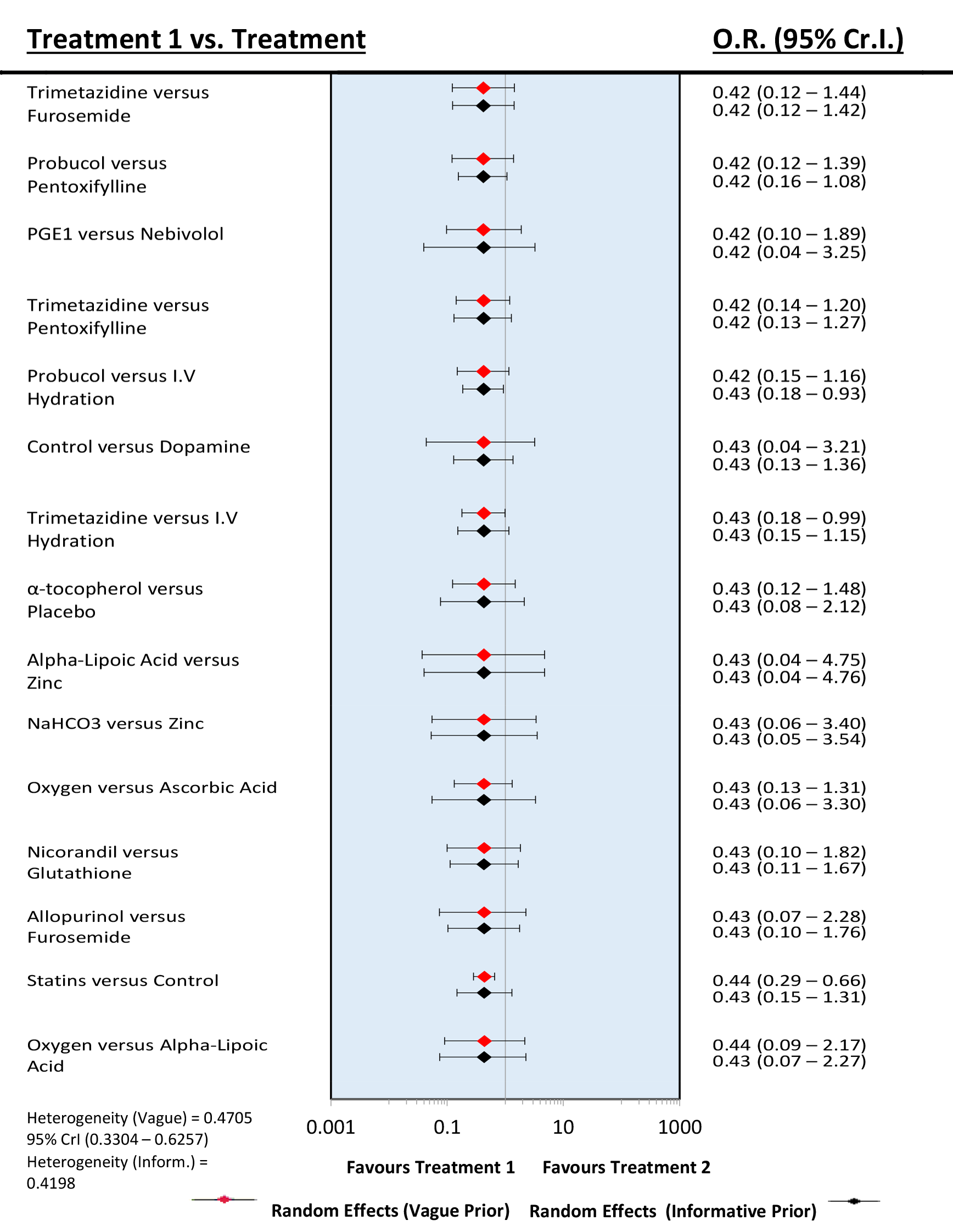


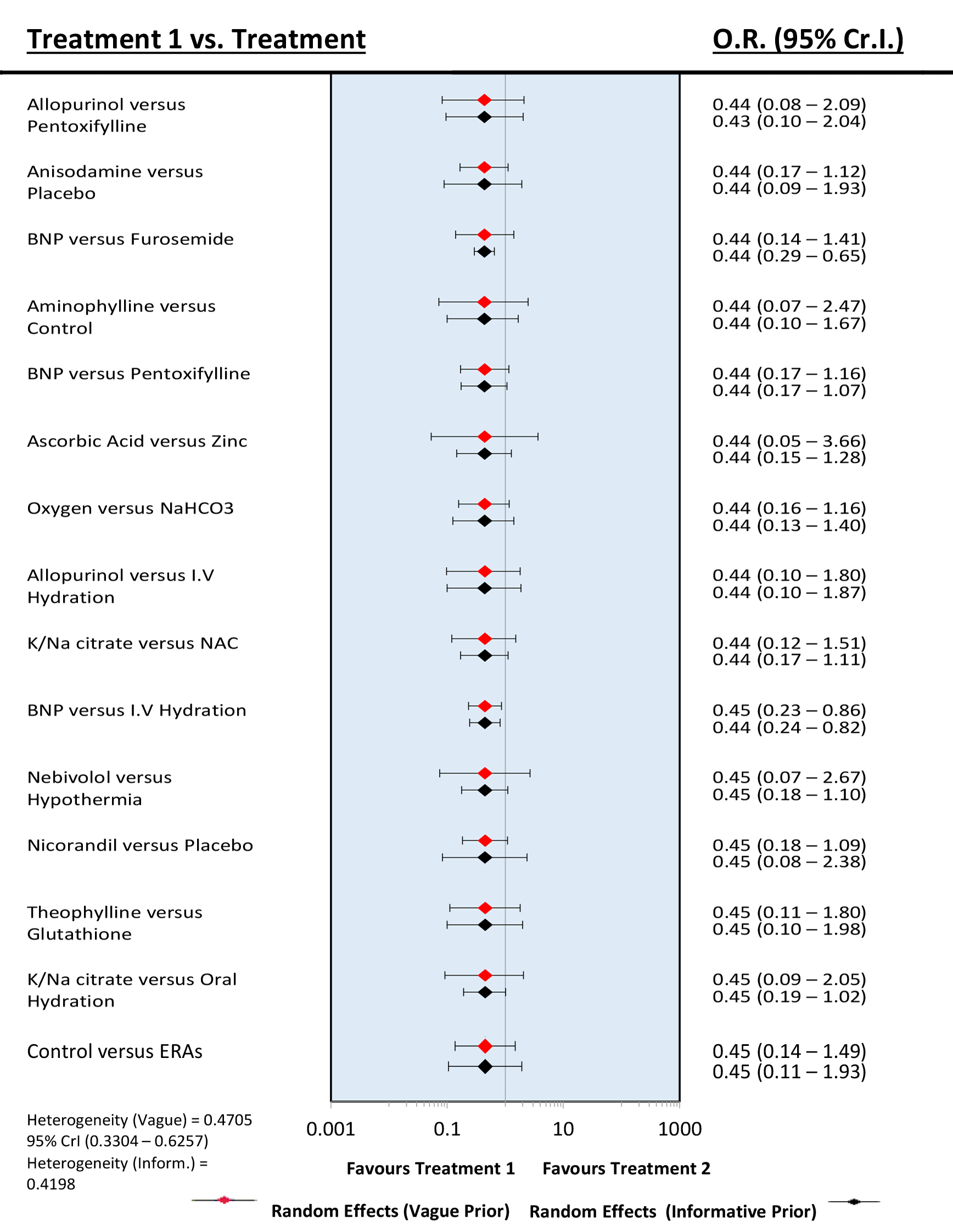


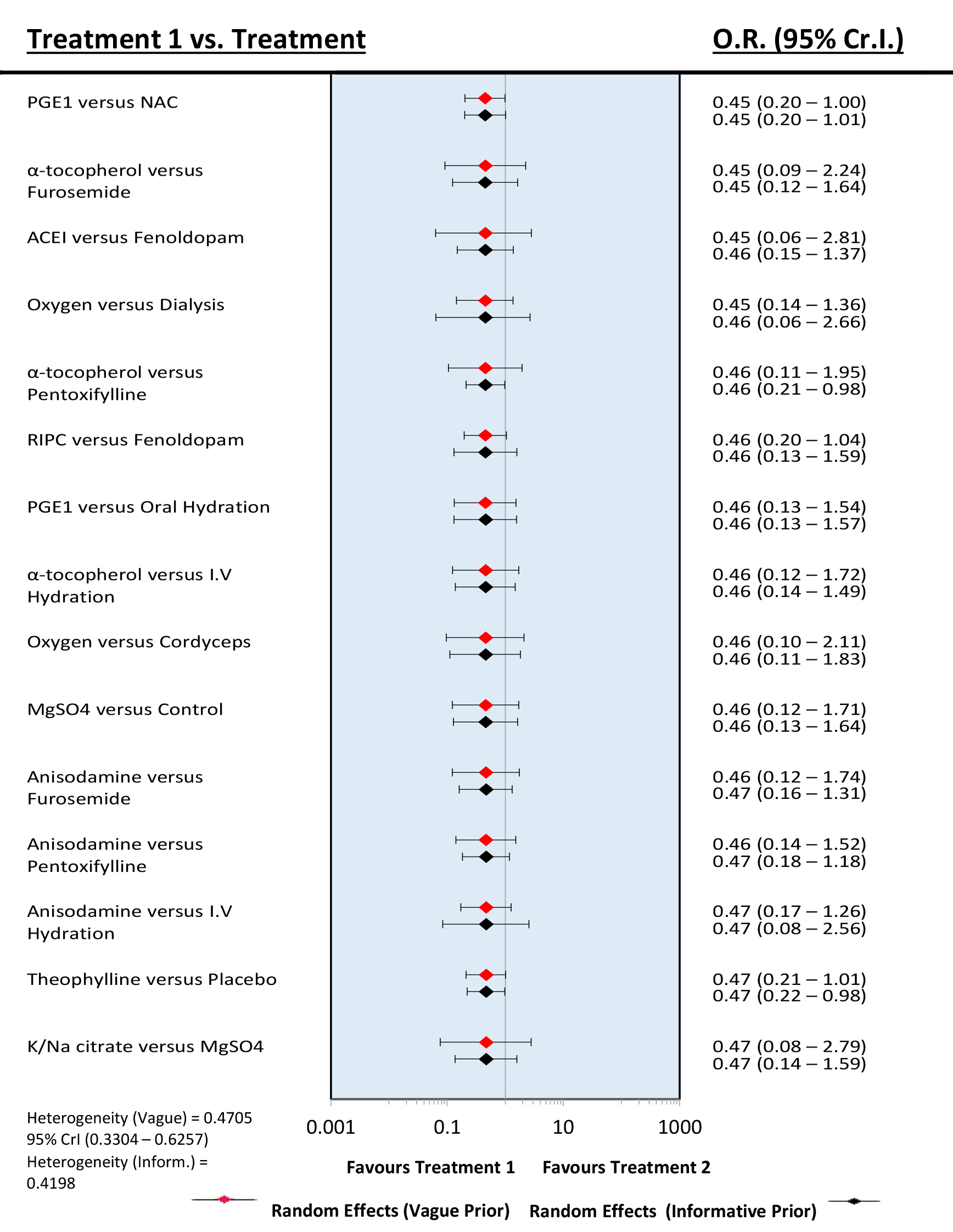


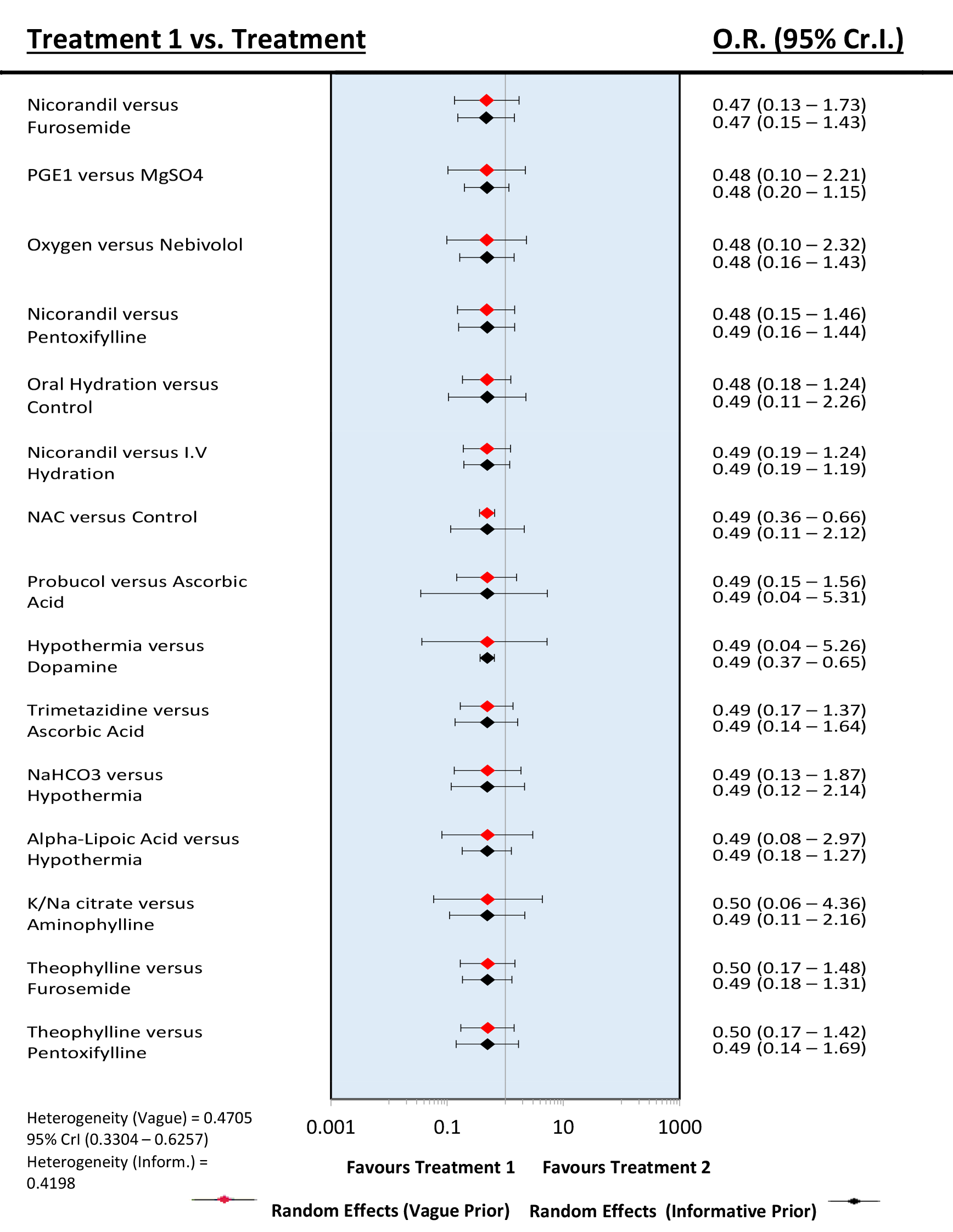


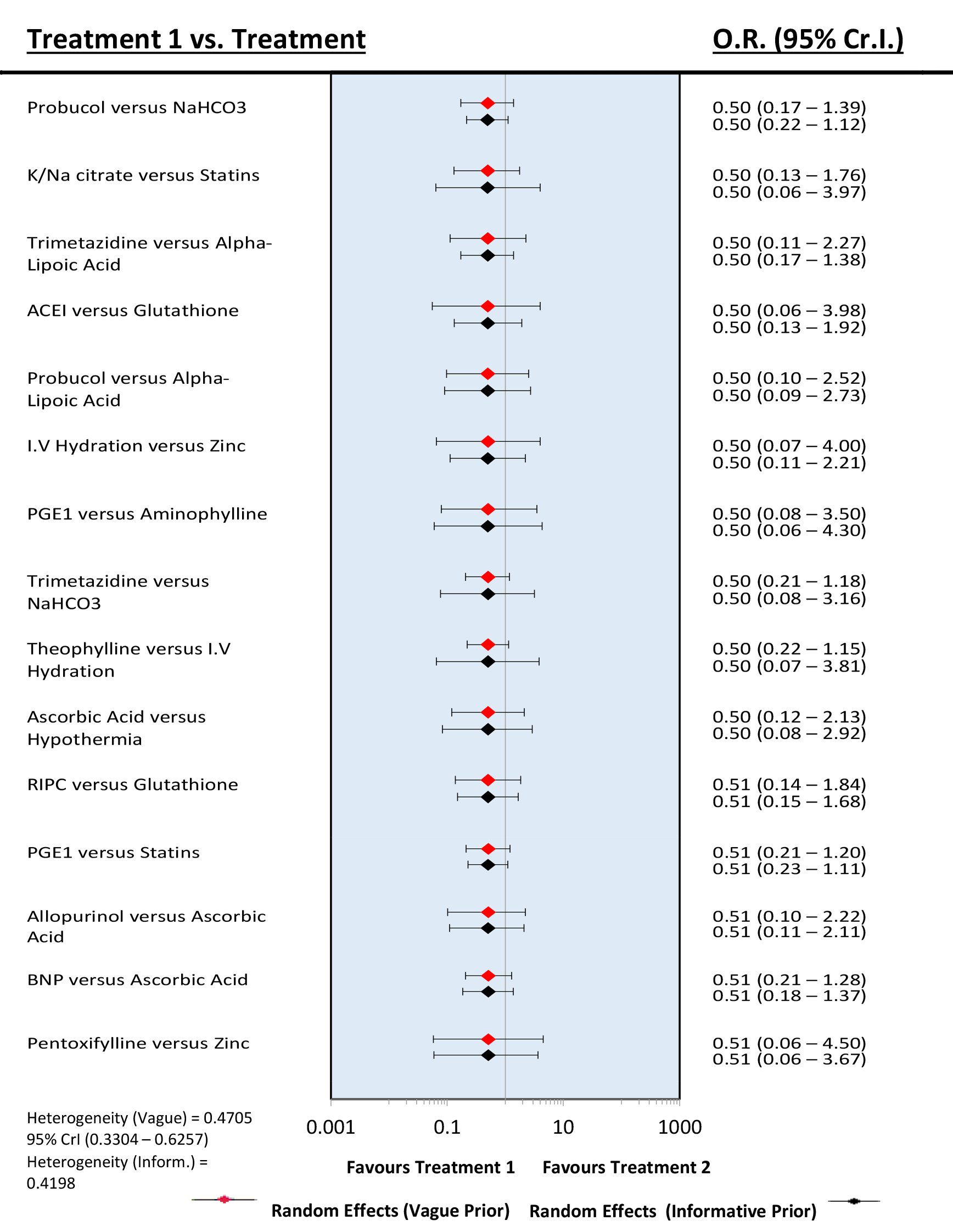


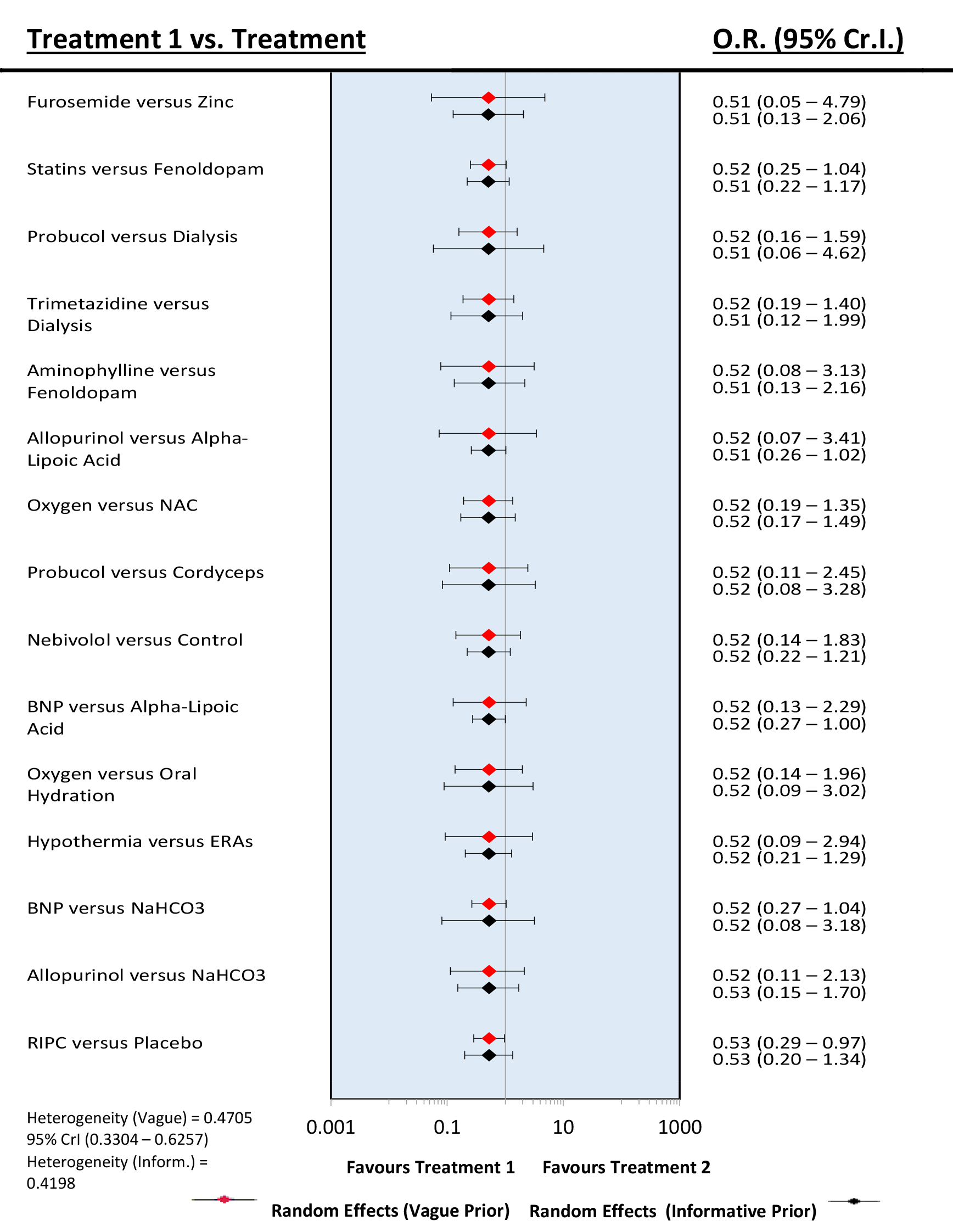


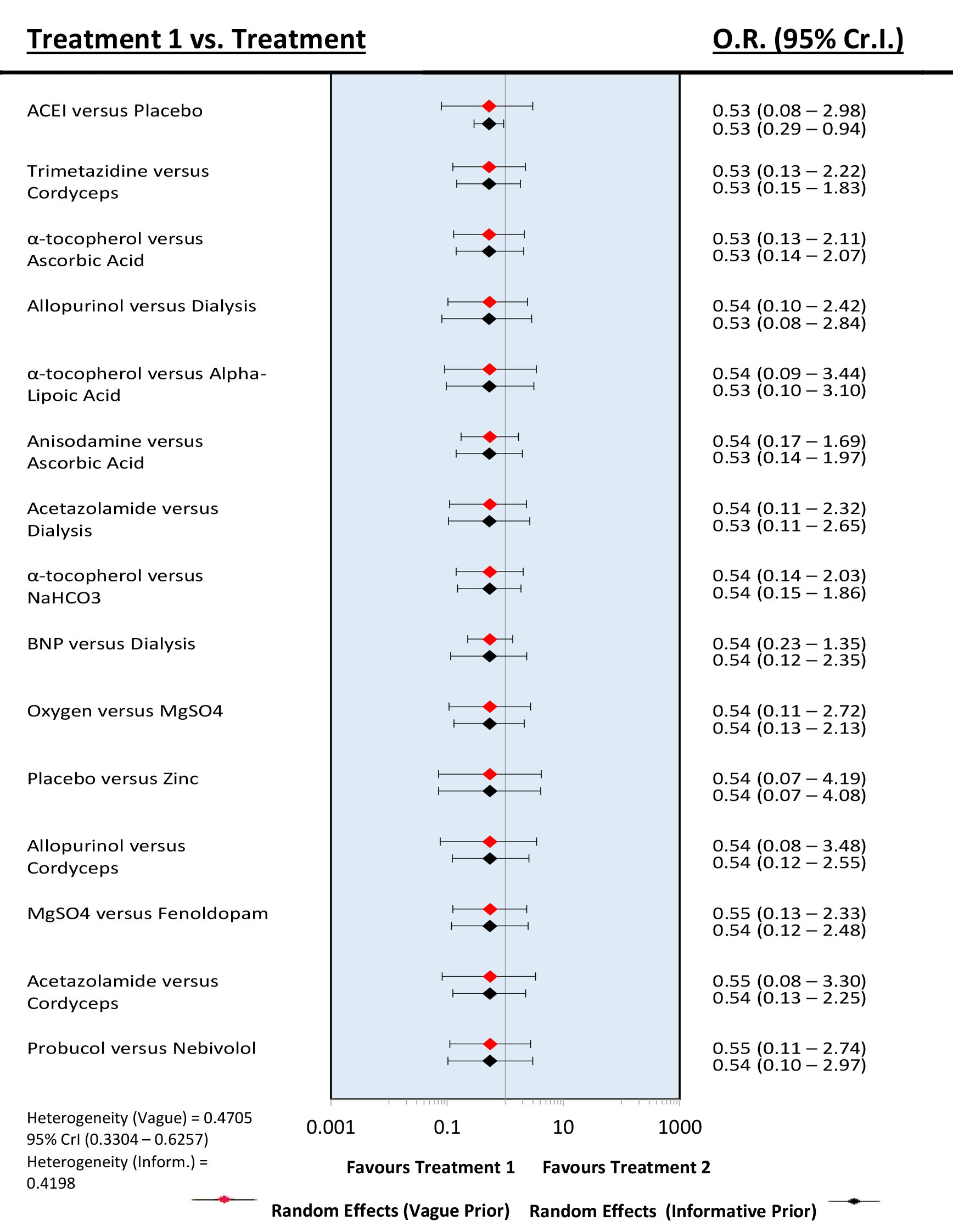


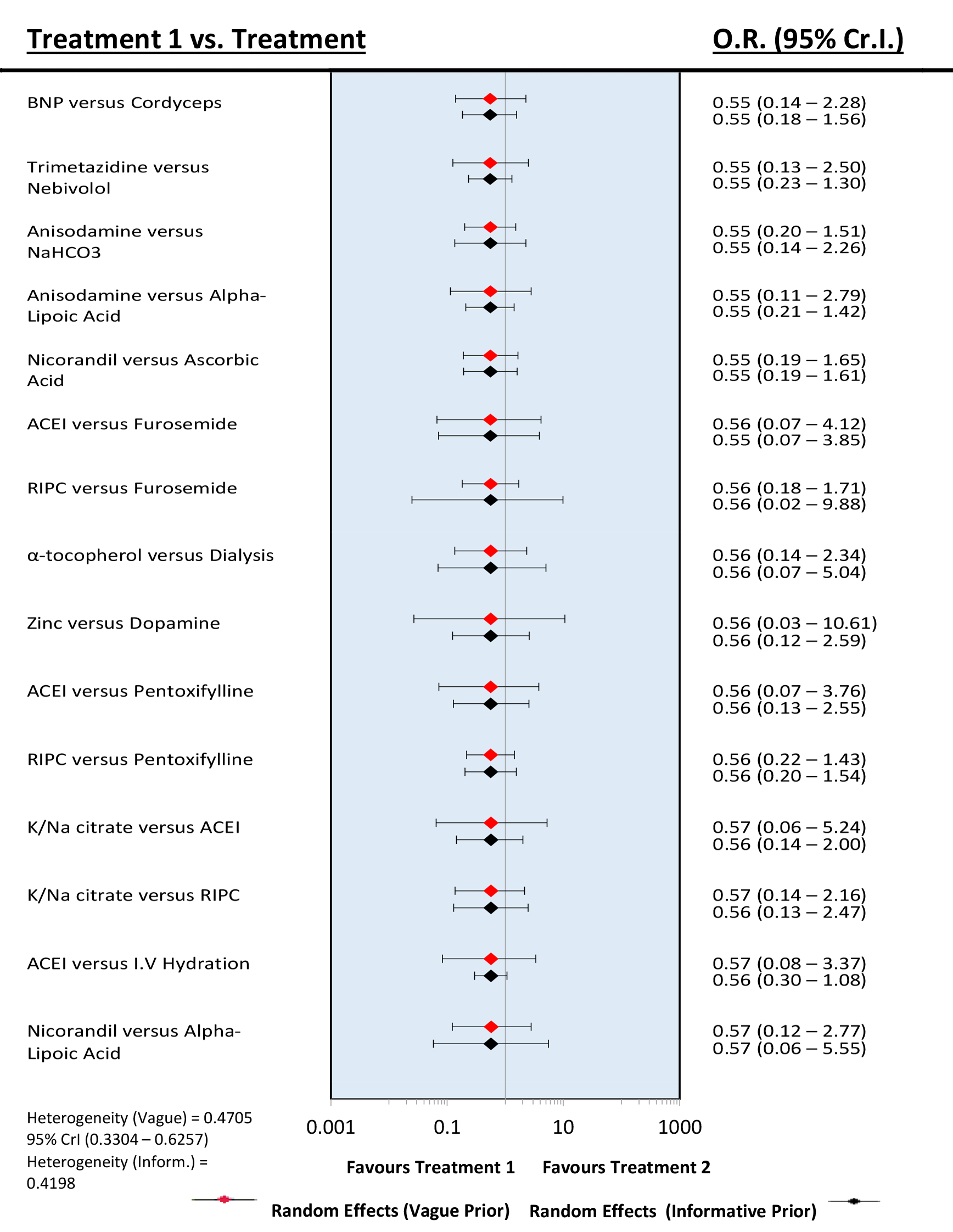


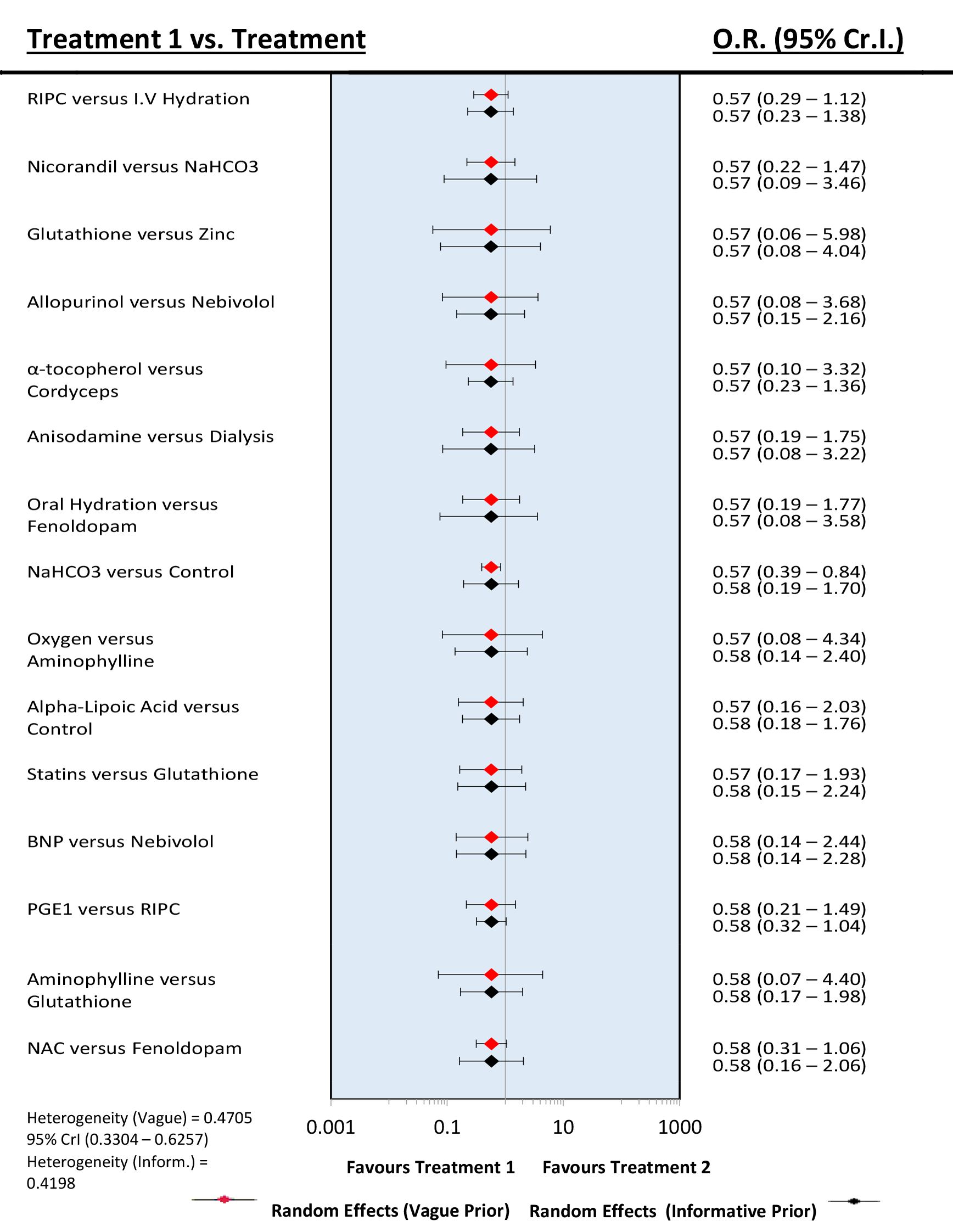


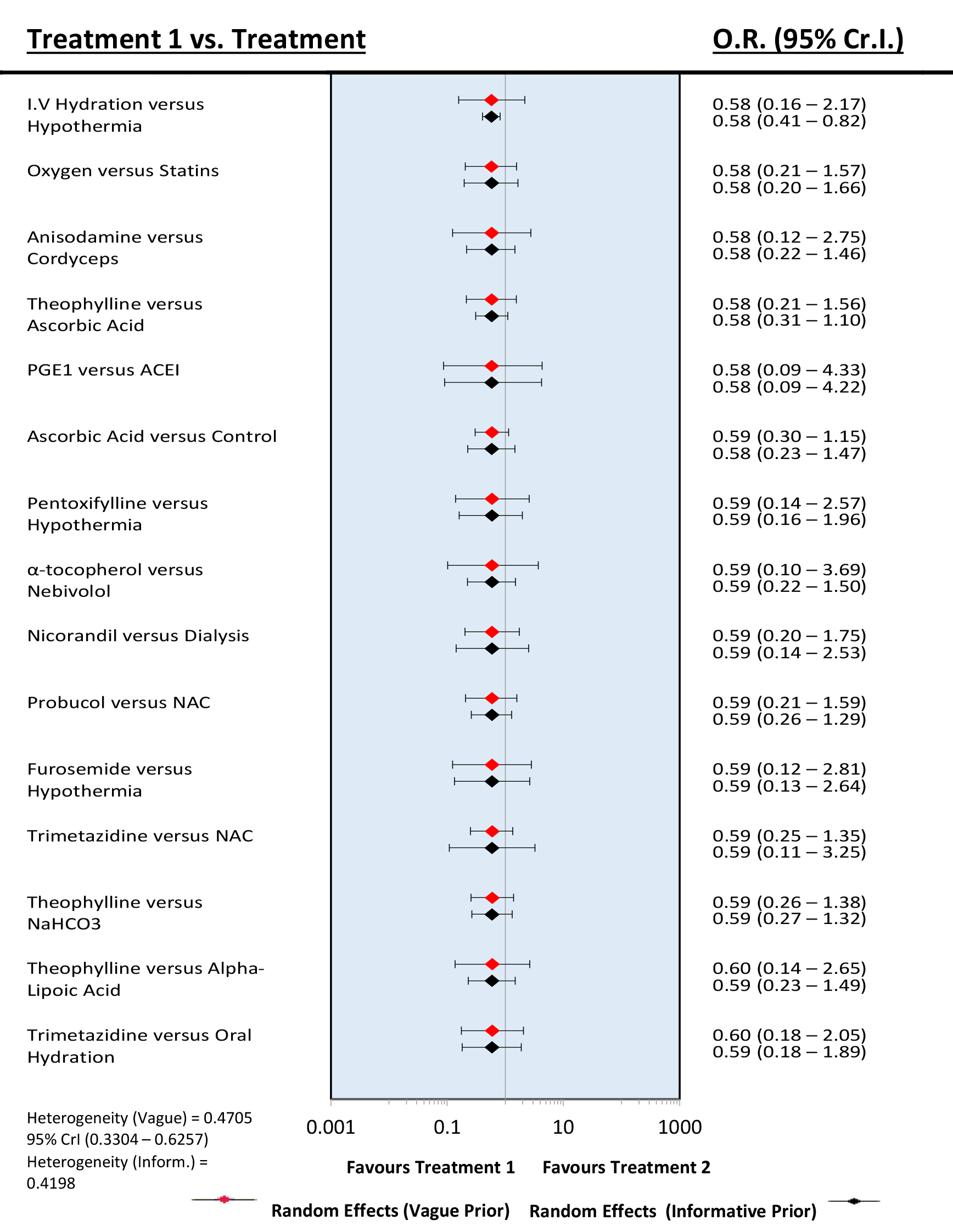


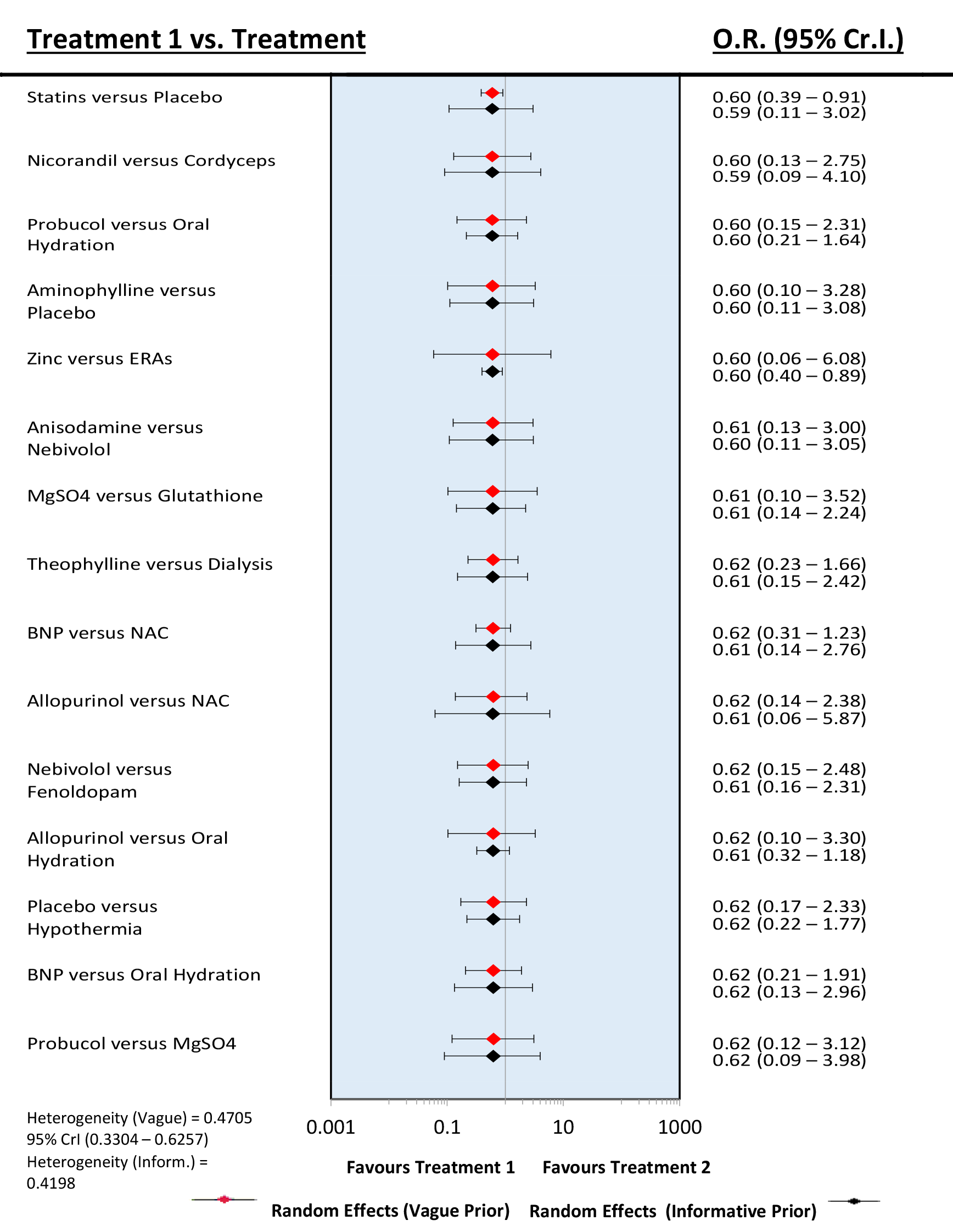


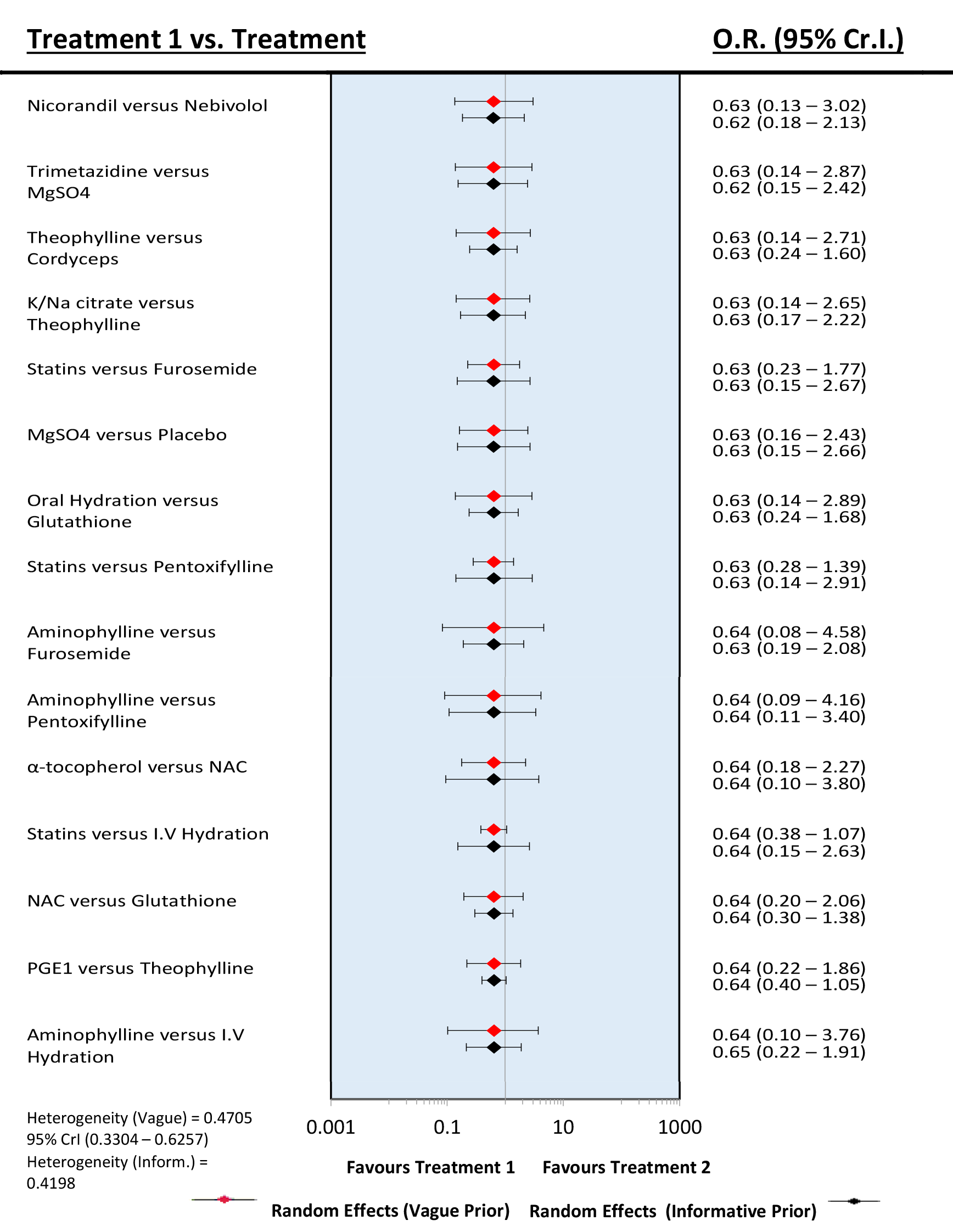


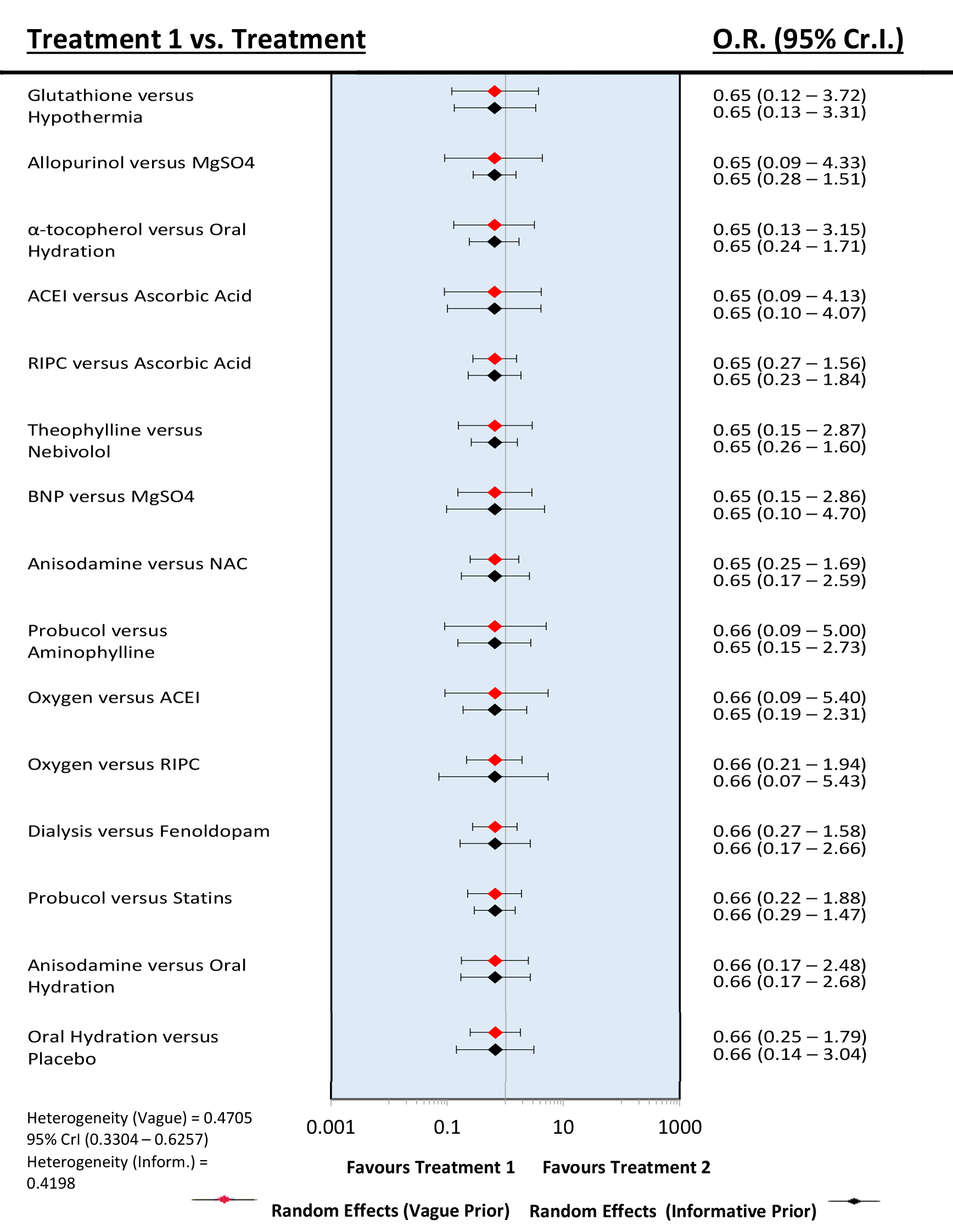


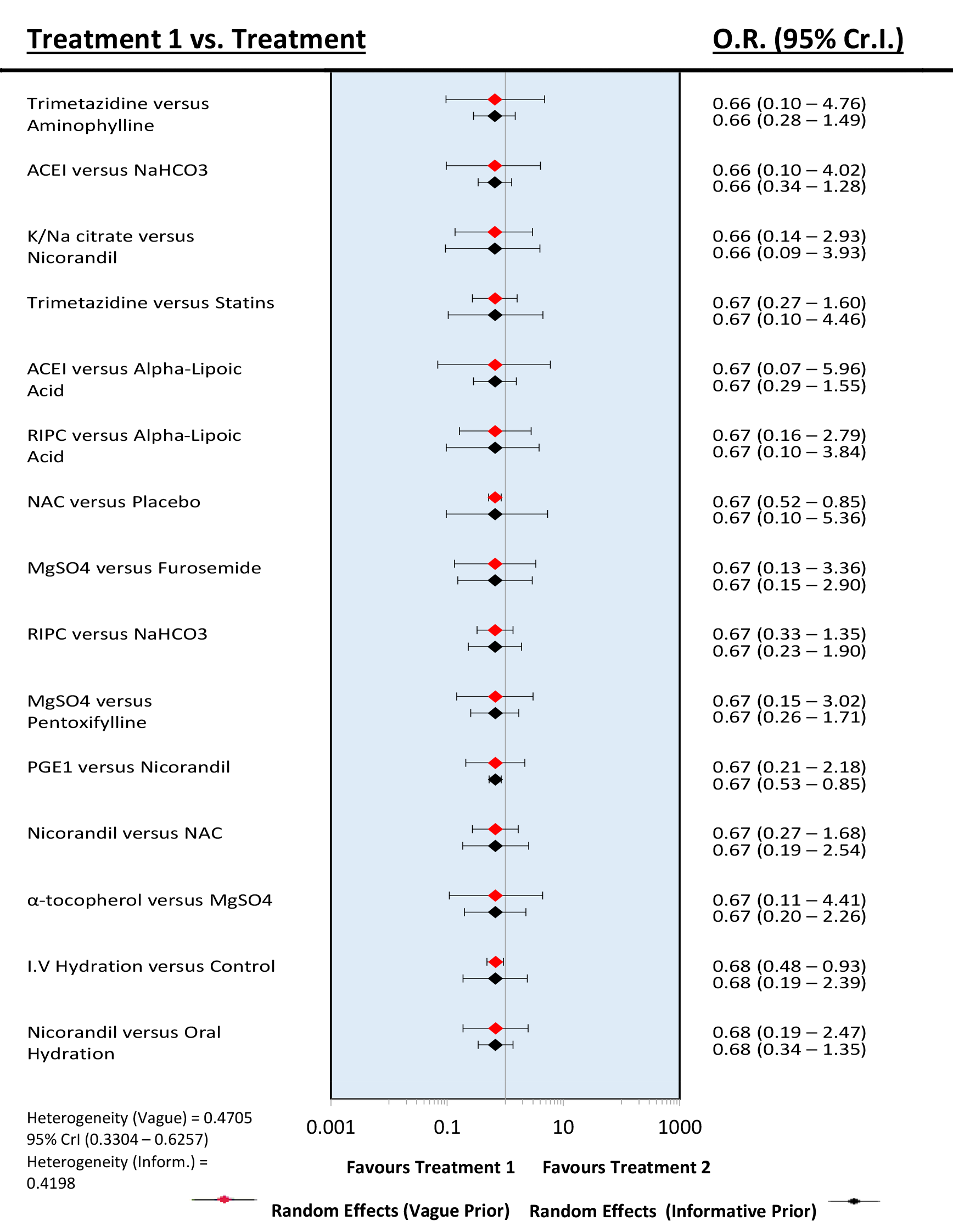


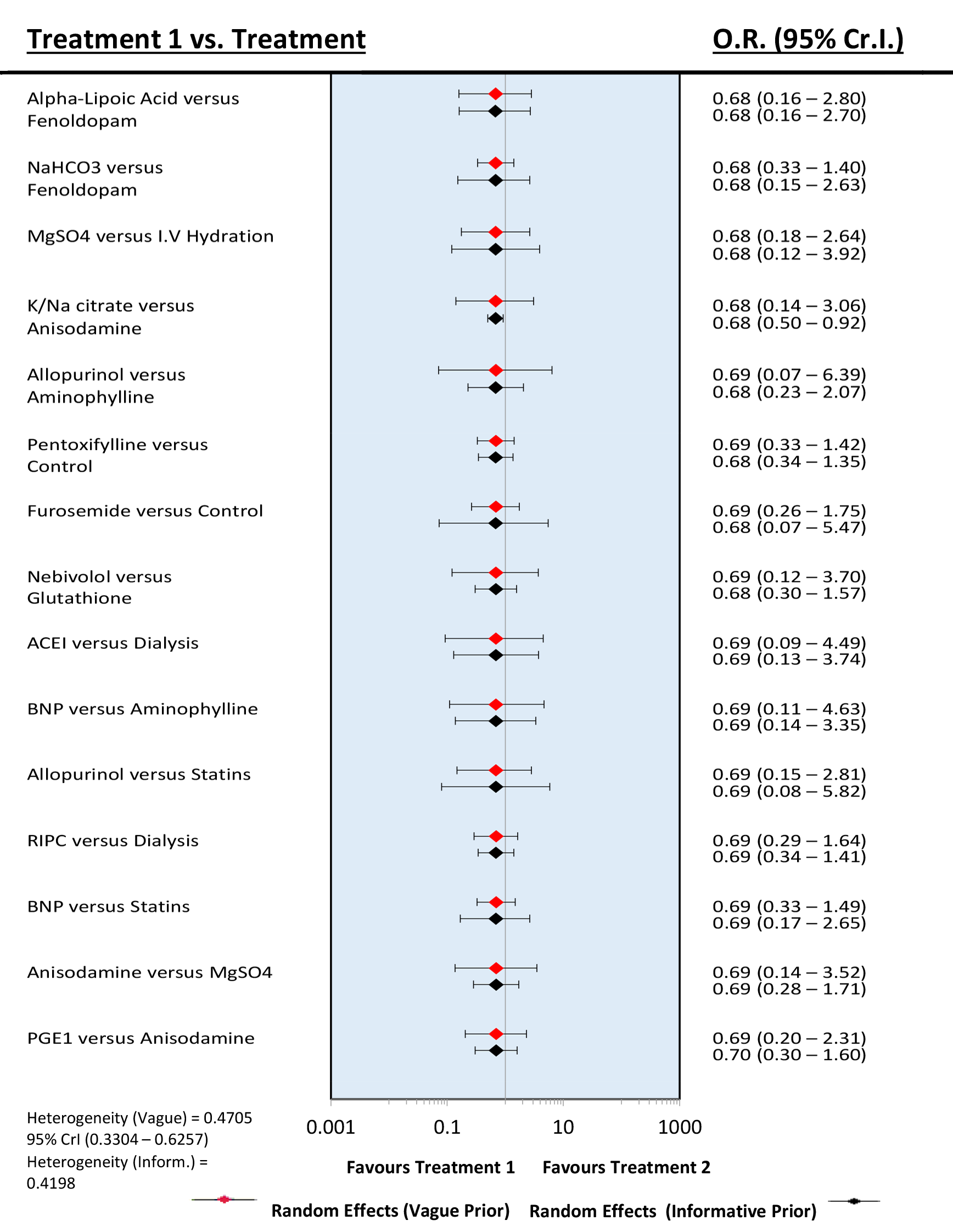


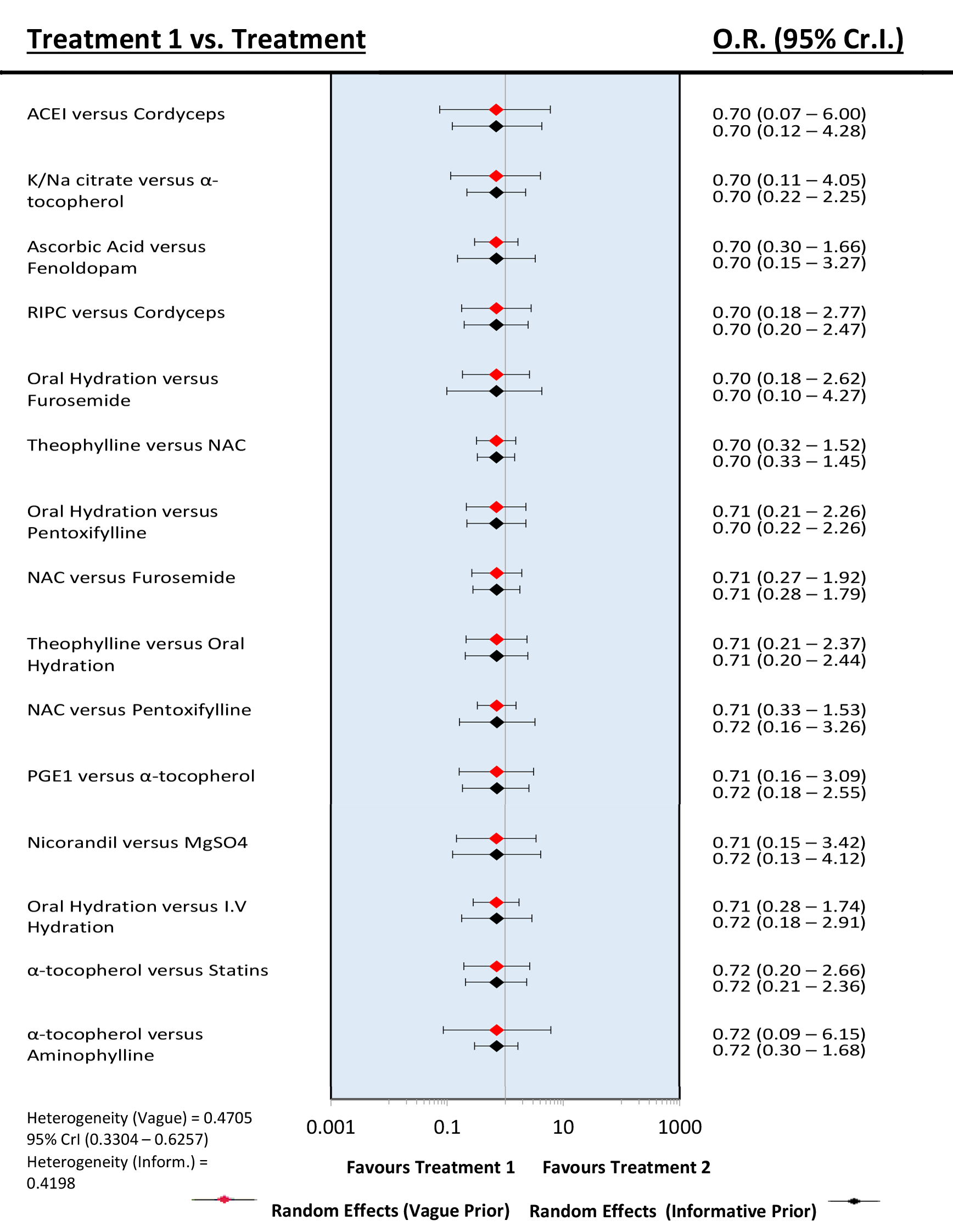


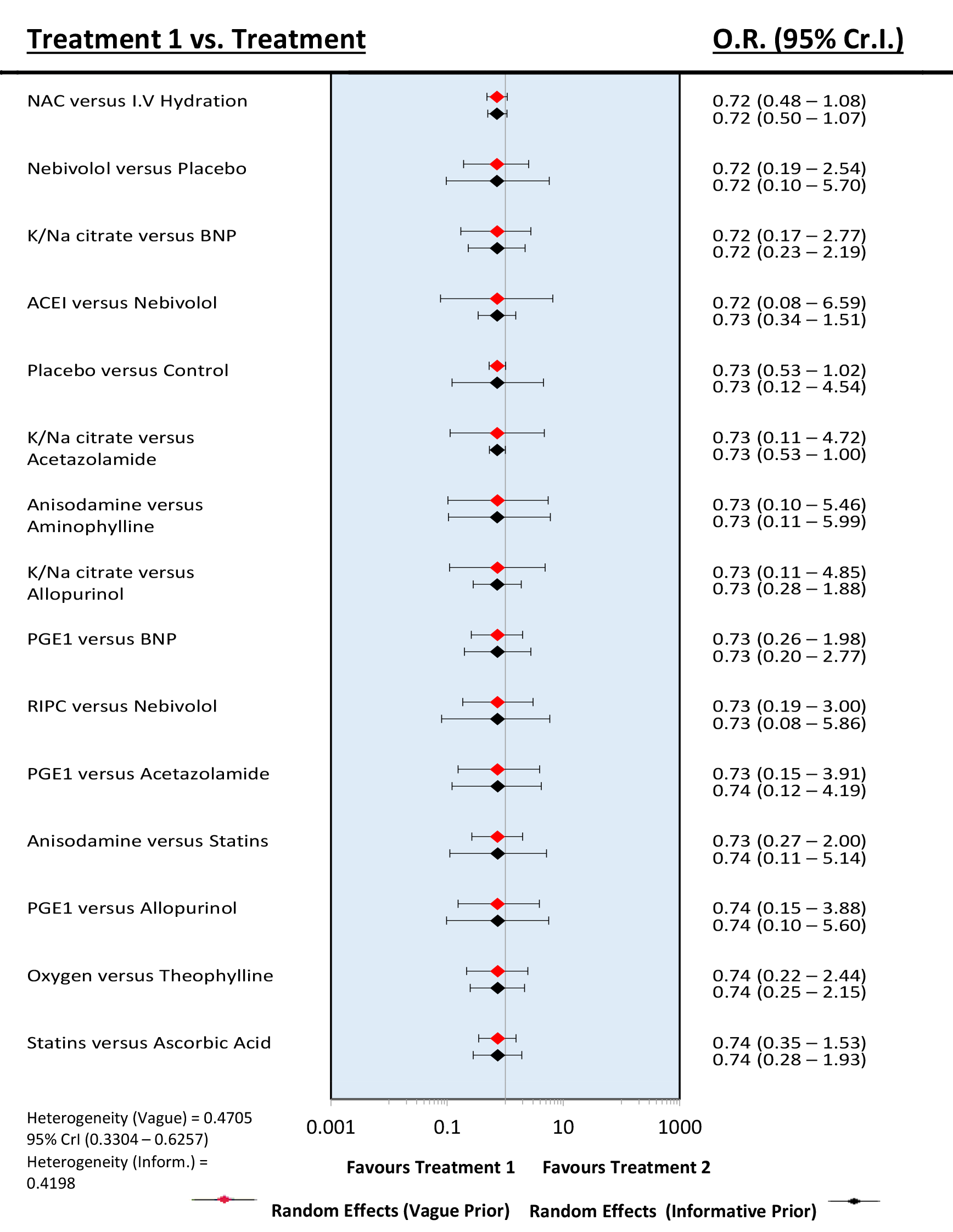


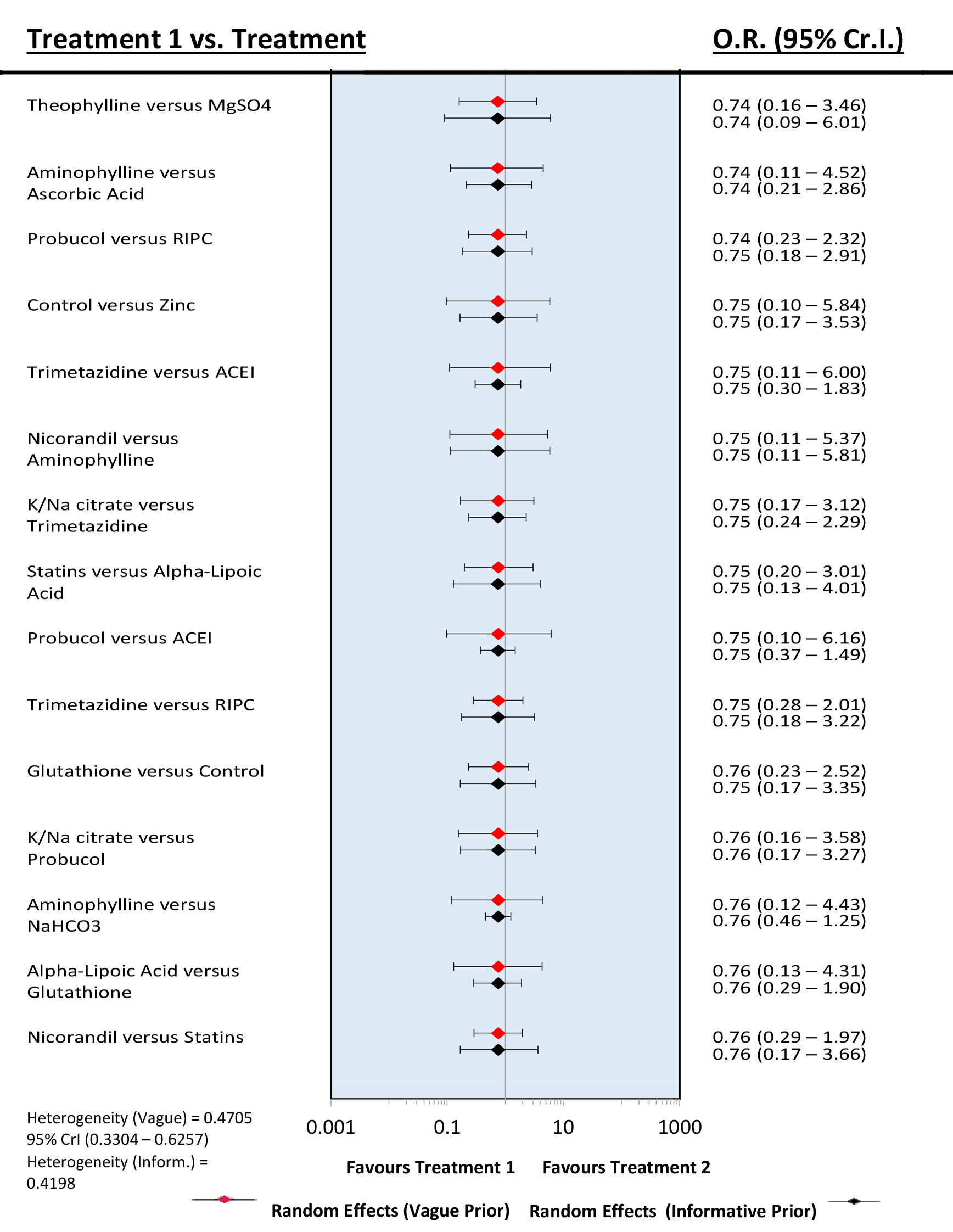


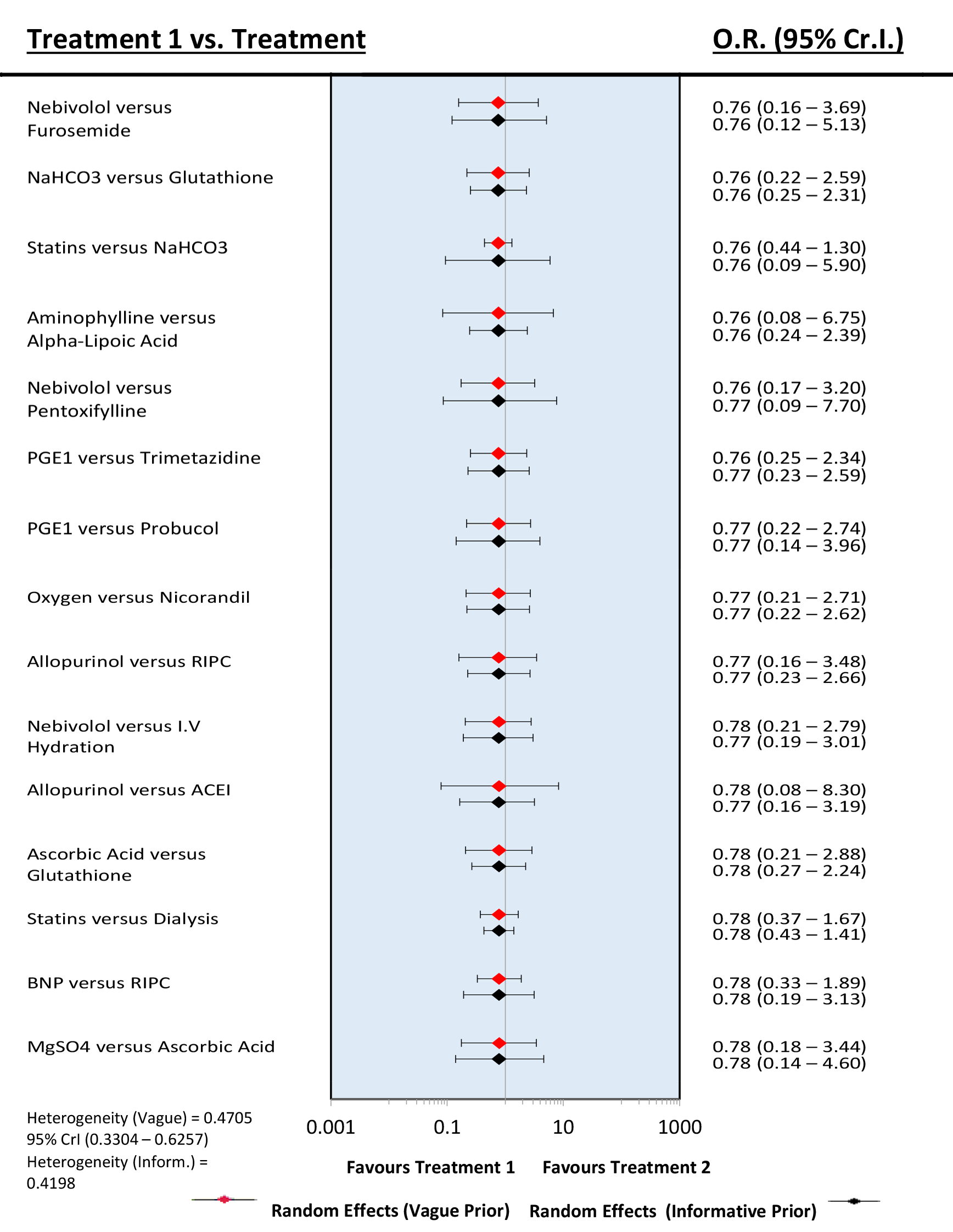


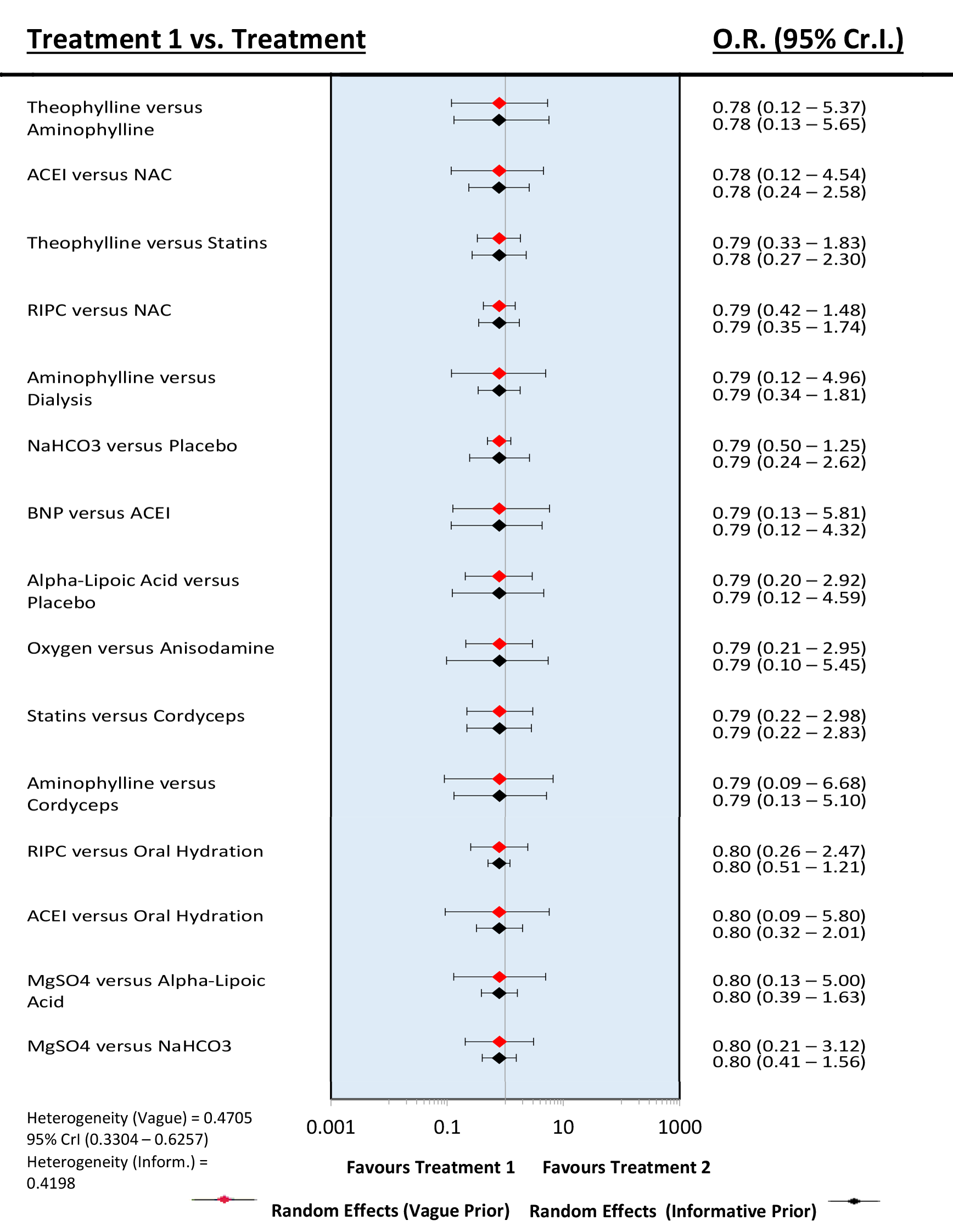


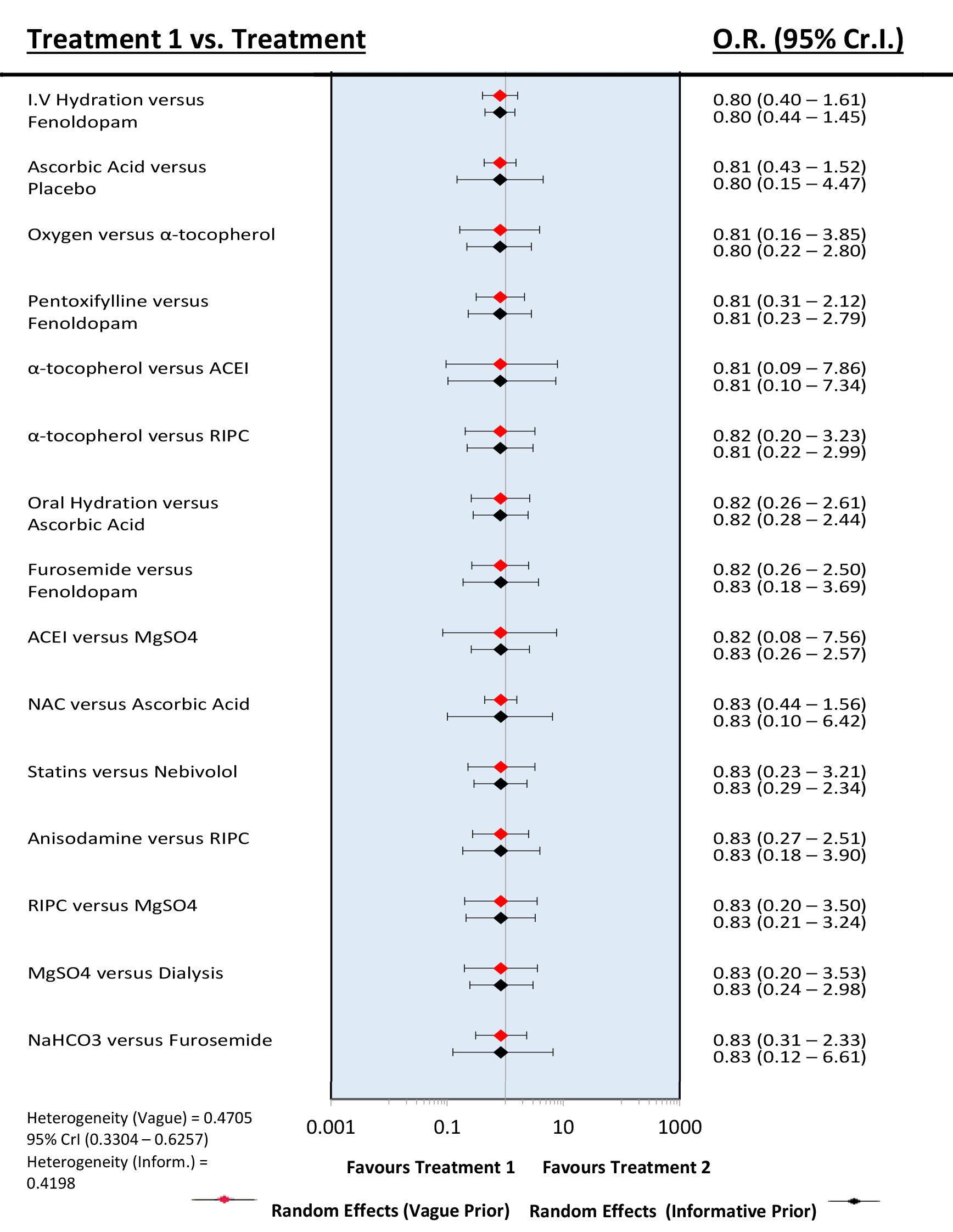


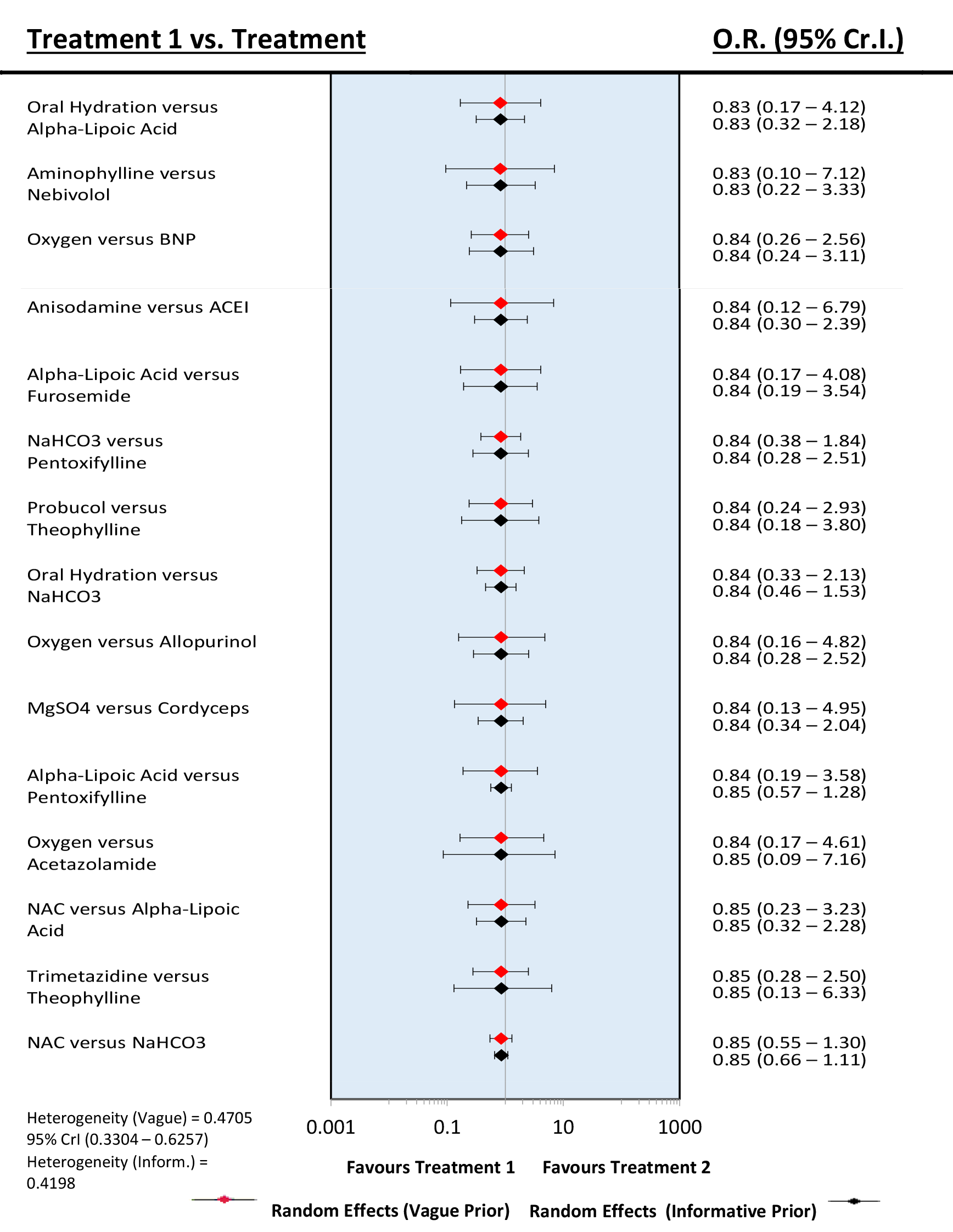


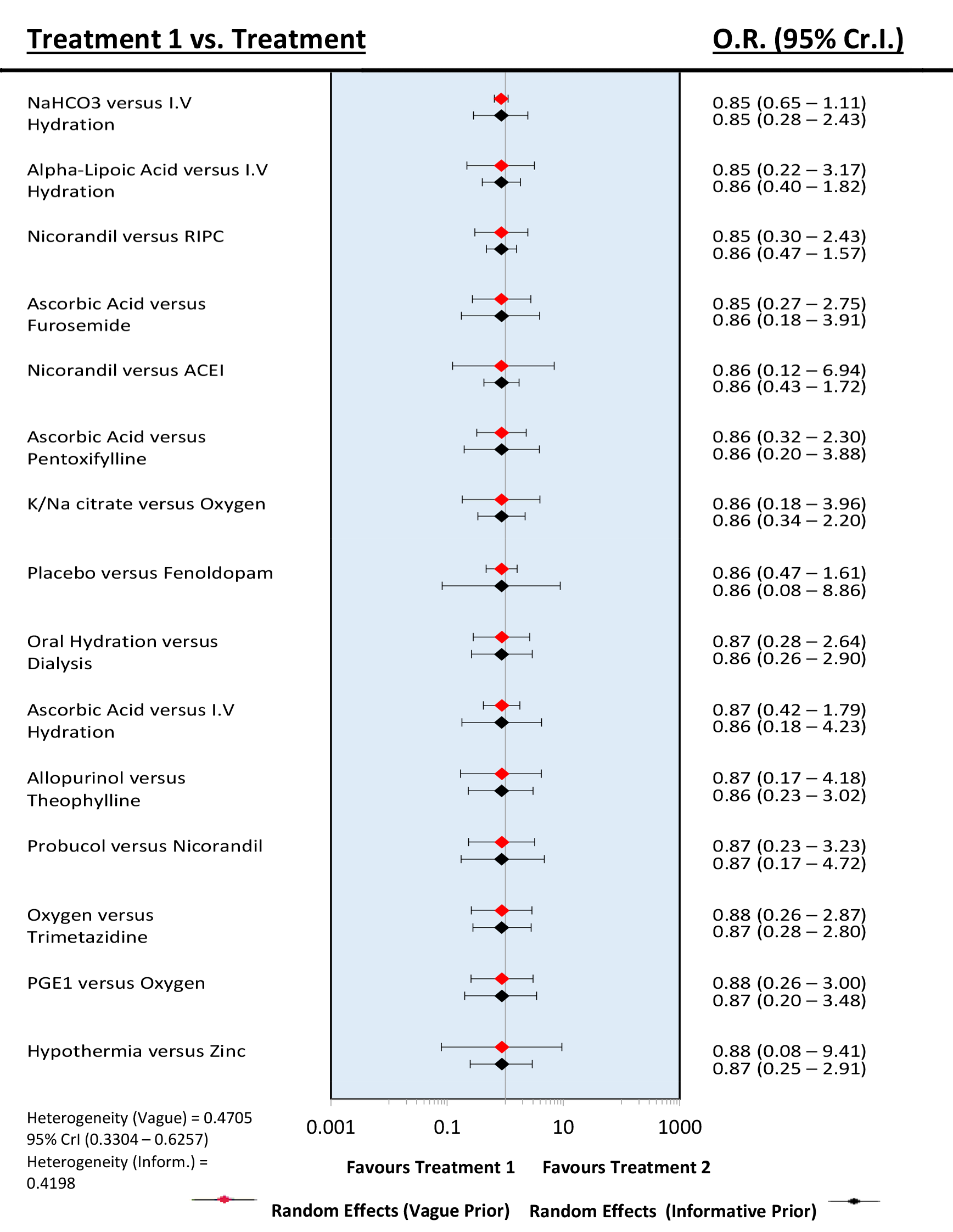


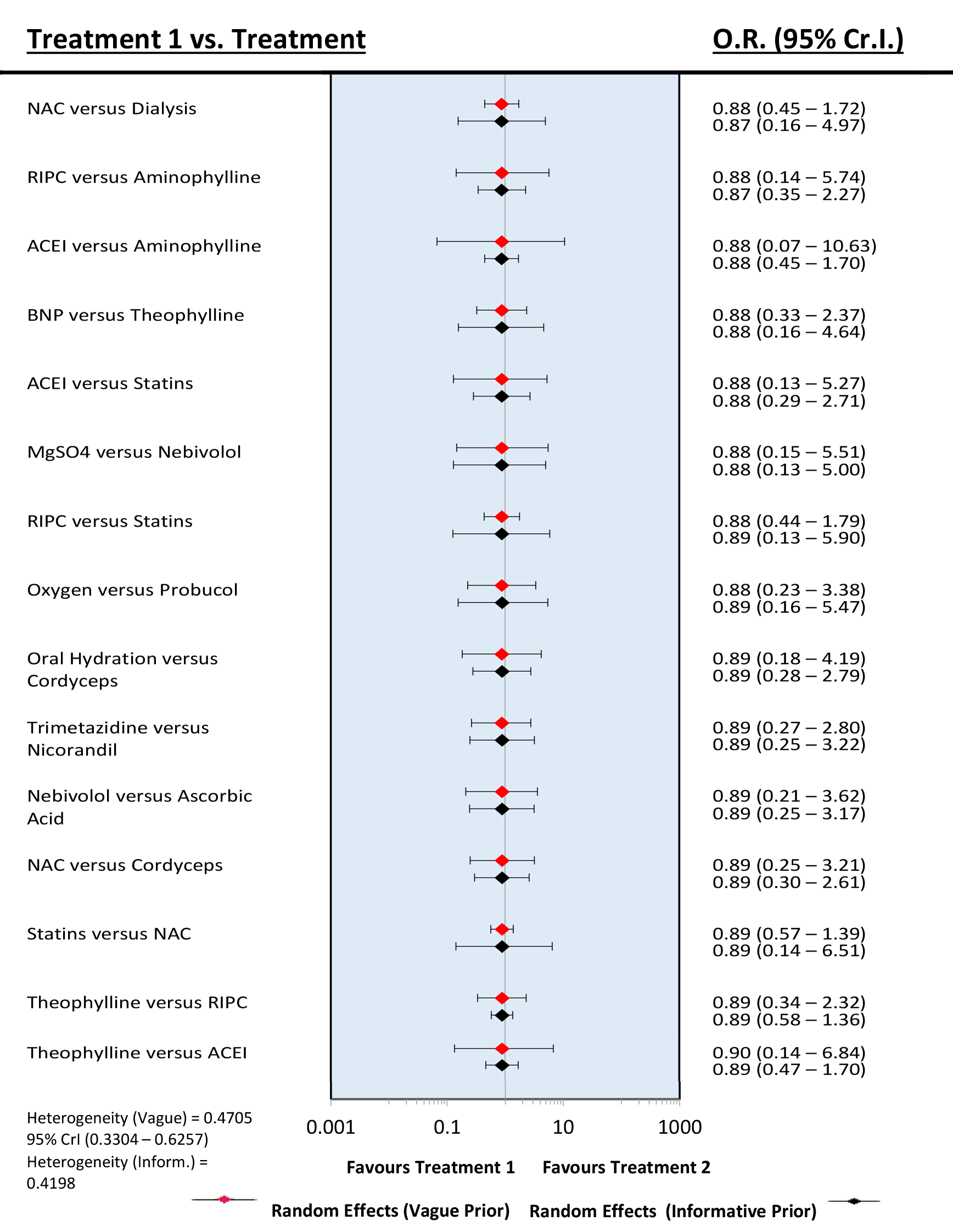


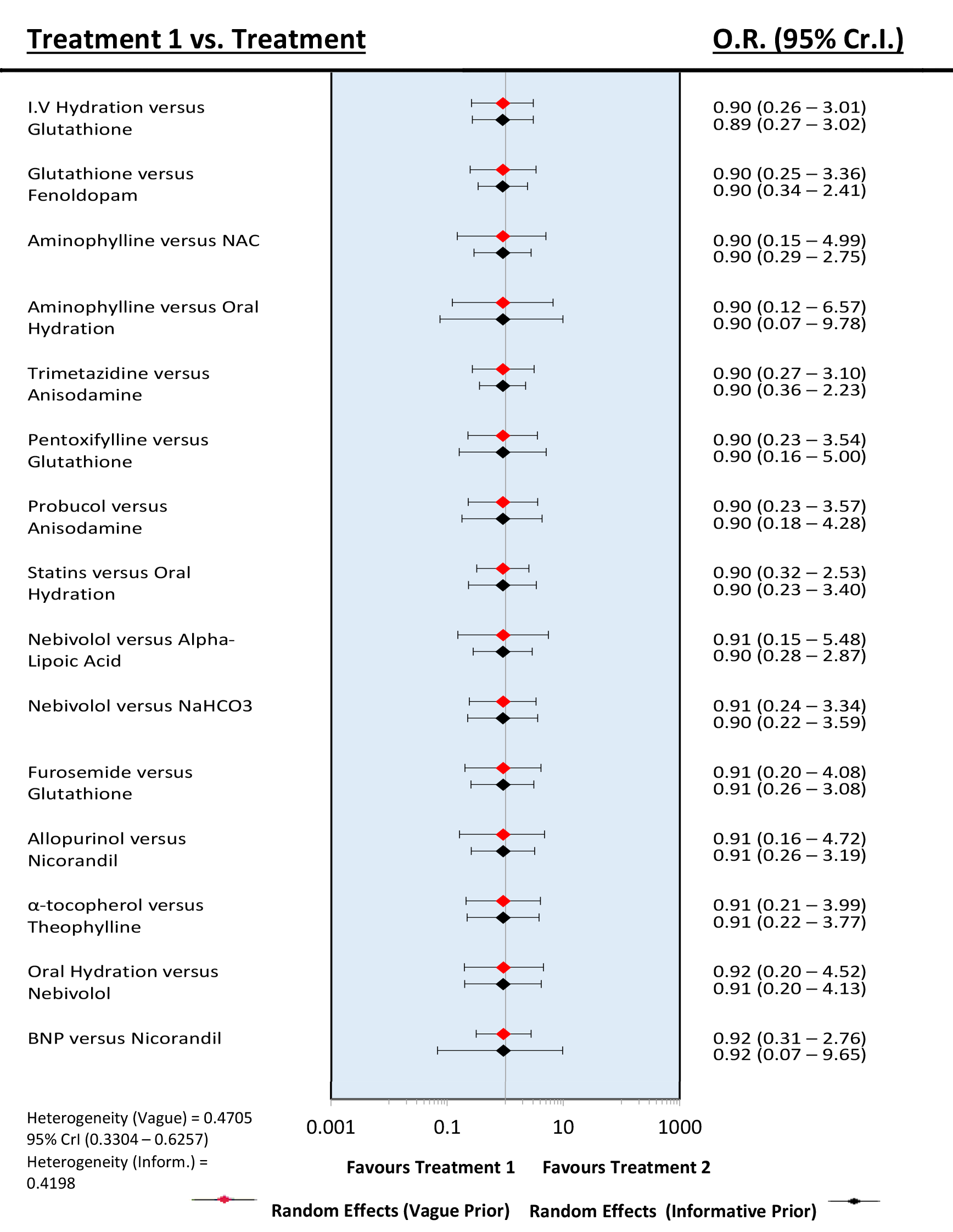


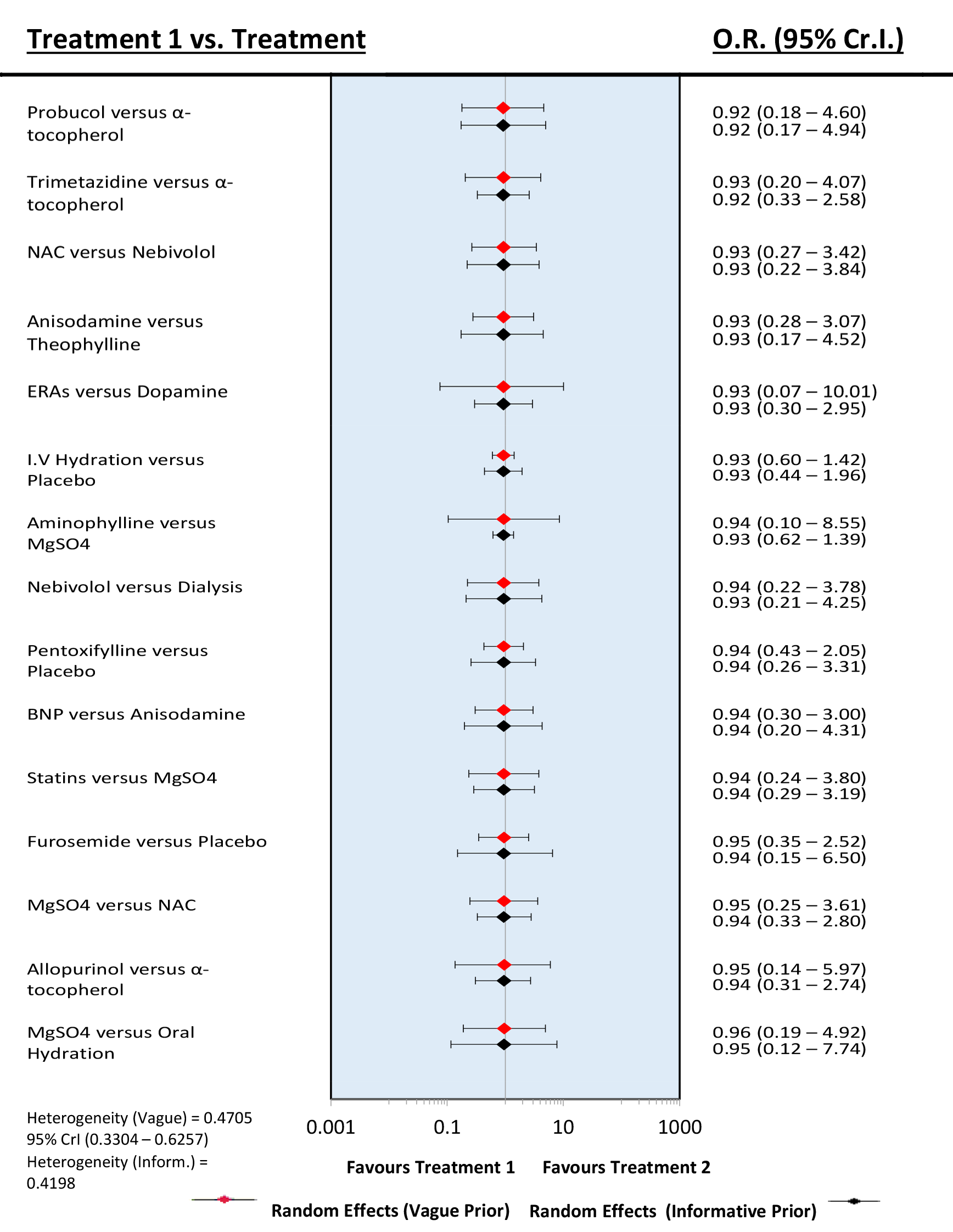


Figure 4Forest Plot (Results from R)

Figure 5Gelman and Rubin's convergence diagnostics

**Figure 6 League Table**
