# Supplementary material for: Effectiveness of contrast-associated acute kidney injury prevention methods; a systematic review and network meta-analysis
Source: BMC Nephrol. 2018 Nov 13;19:323. doi: 10.1186/s12882-018-1113-0 (PMC6234687; doi:10.1186/s12882-018-1113-0)
Supplement: Supplementary file 6 — High Baseline Renal Profile 112–105 RCTs. (DOCX 32278 kb) [file 12882_2018_1113_MOESM6_ESM.docx]

**Supplement:**

**Results from Analysis 3 (RCTs with high baseline renal Function)**

(Creatinine > 1.3 mg/dl (114 mmol/L) and or eGFR < 60 mls/min/1.73m^2)^

Number of Studies: 112 RCTs (see Manuscript)

Figures and Tables:

1. Network Diagram

2. Tables:

A. Network Characteristics

B. Interventions Characteristics

C Direct comparisons characteristics

3. Rankogram

4. Ranking and probability of being the best (table)

5. Forest Plot

| Software | Spec | Convergence | Analysis |
| --- | --- | --- | --- |
| Netmetaxl / WinBUGS14 version 1.4.3 | Burn 5000  Model 10000 | good convergence (FE MC error 5% of the SD) | Random Effects (Vague)  Random Effects (Informative) |

**112 RCTs before exclusion**


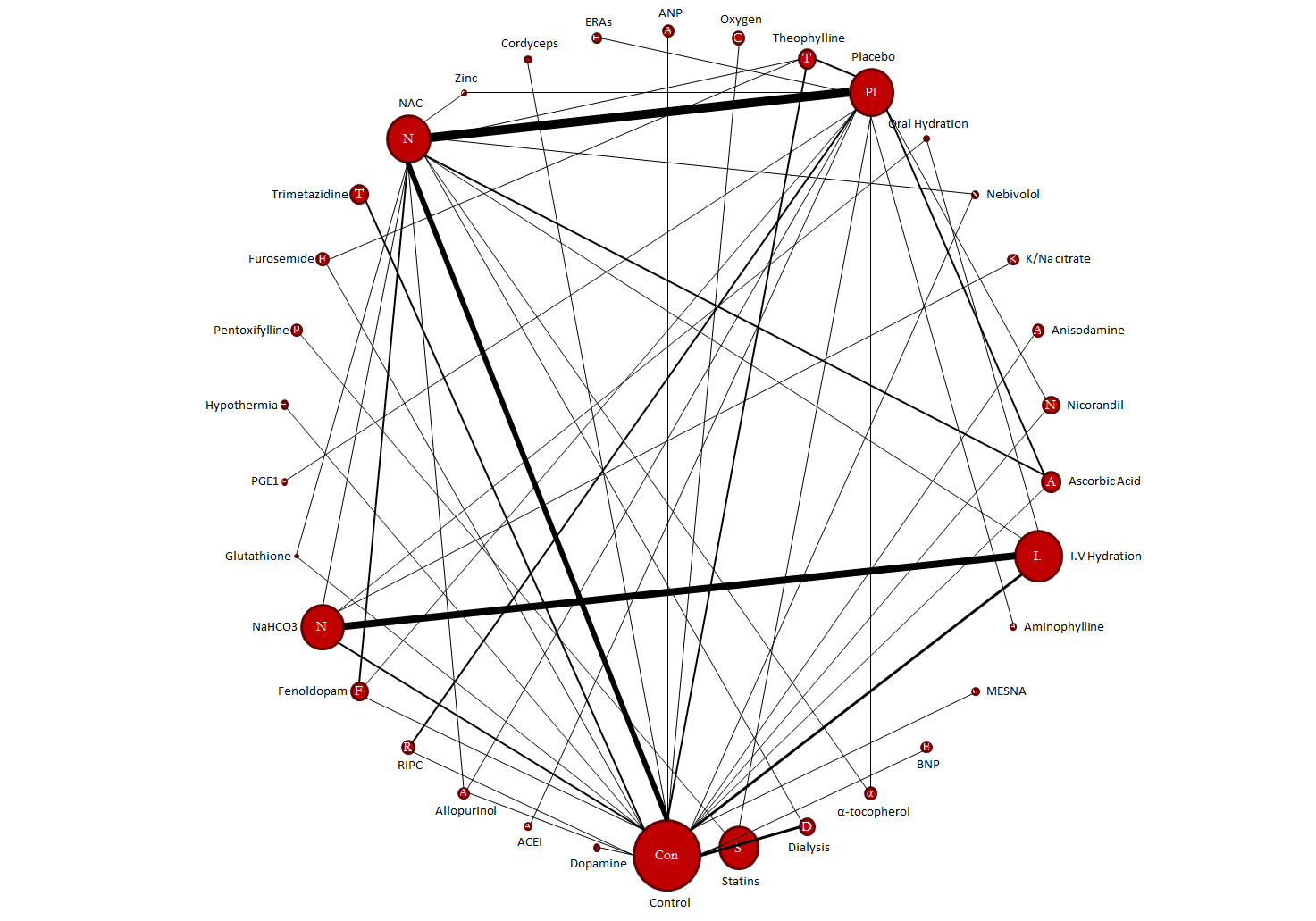


Figure 1 Network Diagram

Table 1Network Characteristics

| **Characteristic** | **Number** |
| --- | --- |
| **Number of Interventions** | 34 |
| **Number of Studies** | 112 |
| **Total Number of Patients in Network** | 21,422 |
| **Total Number of Events in Network** | 2,142 |
| **Total Possible Pairwise Comparisons** | 561 |
| **Total Number Pairwise Comparisons With Direct Data** | 54 |
| **Number of Two-arm Studies** | 99 |
| **Number of Multi-Arms Studies** | 13 |
| **Number of Studies With No Zero Events** | 105 |
| **Number of Studies With At Least One Zero Event** | 7 |
| **Number of Studies with All Zero Events** | 1 |

Table 2 Interventions Characteristics

| **Treatment** | **# Studies** | **# Events** | **# Patients** | **Aggregate Rate** |
| --- | --- | --- | --- | --- |
| **I.V Hydration** | 22 | 265 | 2835 | 0.0935 |
| **Statins** | 6 | 58 | 1961 | 0.0296 |
| **Furosemide** | 2 | 16 | 166 | 0.0964 |
| **NAC** | 40 | 255 | 2438 | 0.1046 |
| **Trimetazidine** | 4 | 17 | 352 | 0.0483 |
| **NaHCO3** | 23 | 173 | 2239 | 0.0773 |
| **PGE1** | 1 | 6 | 33 | 0.1818 |
| **Pentoxifylline** | 1 | 6 | 110 | 0.0545 |
| **Placebo** | 38 | 408 | 2449 | 0.1666 |
| **Control** | 50 | 600 | 5605 | 0.1070 |
| **Allopurinol** | 2 | 5 | 109 | 0.0459 |
| **BNP** | 1 | 6 | 106 | 0.0566 |
| **α-tocopherol** | 2 | 10 | 159 | 0.0629 |
| **Oxygen** | 1 | 1 | 174 | 0.0057 |
| **K/Na citrate** | 1 | 2 | 103 | 0.0194 |
| **Nicorandil** | 3 | 15 | 291 | 0.0515 |
| **Ascorbic Acid** | 6 | 41 | 434 | 0.0945 |
| **Oral Hydration** | 1 | 1 | 22 | 0.0455 |
| **Nebivolol** | 1 | 8 | 40 | 0.2000 |
| **Anisodamine** | 1 | 13 | 132 | 0.0985 |
| **RIPC** | 4 | 16 | 163 | 0.0982 |
| **Theophylline** | 7 | 21 | 384 | 0.0547 |
| **Hypothermia** | 1 | 14 | 58 | 0.2414 |
| **Glutathione** | 1 |  | 7 | 0.0000 |
| **MESNA** | 1 |  | 51 | 0.0000 |
| **ACEI** | 1 | 3 | 52 | 0.0577 |
| **Aminophylline** | 1 | 4 | 30 | 0.1333 |
| **ANP** | 1 | 4 | 126 | 0.0317 |
| **Zinc** | 1 | 3 | 18 | 0.1667 |
| **Dialysis** | 5 | 43 | 293 | 0.1468 |
| **Fenoldopam** | 5 | 78 | 333 | 0.2342 |
| **ERAs** | 1 | 43 | 77 | 0.5584 |
| **Dopamine** | 1 | 4 | 33 | 0.1212 |
| **Cordyceps** | 1 | 3 | 39 | 0.0769 |

Table 3 Direct comparisons characteristics

| **Comparison** | **# Studies** | **# Patients** | **# Events** |
| --- | --- | --- | --- |
| **Statins vs. Control** | 3 | 3,338 | 116 |
| **NaHCO3 vs. K/Na citrate** | 1 | 206 | 4 |
| **I.V Hydartion vs. NaHCO3** | 17 | 3,699 | 323 |
| **NAC vs. Placebo** | 21 | 2,622 | 373 |
| **NAC vs. Ascorbic Acid** | 3 | 583 | 88 |
| **Placebo vs. Ascorbic Acid** | 4 | 638 | 105 |
| **NAC vs. Control** | 13 | 1,575 | 170 |
| **NAC vs. Fenoldopam** | 3 | 359 | 44 |
| **Control vs. Fenoldopam** | 2 | 123 | 26 |
| **I.V Hydartion vs. Control** | 5 | 1,960 | 196 |
| **NAC vs. Theophylline** | 1 | 62 | 13 |
| **Placebo vs. Theophylline** | 3 | 224 | 21 |
| **I.V Hydartion vs. Oral Hydration** | 1 | 49 | 7 |
| **NaHCO3 vs. Oral Hydration** | 1 | 43 | 3 |
| **Furosemide vs. Control** | 2 | 326 | 35 |
| **Furosemide vs. Theophylline** | 1 | 159 | 18 |
| **Control vs. Theophylline** | 3 | 493 | 27 |
| **Control vs. RIPC** | 1 | 100 | 26 |
| **Control vs. Allopurinol** | 1 | 159 | 6 |
| **Placebo vs. Nicorandil** | 1 | 240 | 29 |
| **Control vs. Dopamine** | 1 | 66 | 6 |
| **Control vs. Anisodamine** | 1 | 260 | 39 |
| **NAC vs. Nebivolol** | 1 | 80 | 17 |
| **Control vs. Nebivolol** | 1 | 80 | 19 |
| **NAC vs. NaHCO3** | 2 | 152 | 32 |
| **Statins vs. Placebo** | 2 | 366 | 25 |
| **Control vs. Cordyceps** | 1 | 80 | 9 |
| **I.V Hydartion vs. NAC** | 2 | 231 | 27 |
| **Statins vs. Pentoxifylline** | 1 | 220 | 9 |
| **NAC vs. Zinc** | 1 | 37 | 4 |
| **Placebo vs. Zinc** | 1 | 35 | 5 |
| **NAC vs. α-tocopherol** | 1 | 20 | 0 |
| **Placebo vs. α-tocopherol** | 2 | 318 | 31 |
| **Control vs. Nicorandil** | 2 | 341 | 22 |
| **PGE1 vs. Placebo** | 1 | 62 | 21 |
| **NaHCO3 vs. Control** | 4 | 506 | 36 |
| **Control vs. Dialysis** | 5 | 588 | 112 |
| **Placebo vs. ACEI** | 1 | 114 | 9 |
| **Control vs. BNP** | 1 | 209 | 23 |
| **Trimetazidine vs. Control** | 4 | 714 | 71 |
| **Control vs. MESNA** | 1 | 100 | 7 |
| **Placebo vs. RIPC** | 3 | 225 | 27 |
| **Control vs. ANP** | 1 | 254 | 19 |
| **NAC vs. Dialysis** | 1 | 275 | 11 |
| **Placebo vs. Aminophylline** | 1 | 60 | 10 |
| **NAC vs. Allopurinol** | 1 | 65 | 12 |
| **Placebo vs. Allopurinol** | 1 | 60 | 16 |
| **NAC vs. Glutathione** | 1 | 14 | 1 |
| **Control vs. Glutathione** | 1 | 14 | 1 |
| **Control vs. Oxygen** | 1 | 349 | 10 |
| **Placebo vs. Fenoldopam** | 1 | 283 | 90 |
| **Control vs. Hypothermia** | 1 | 128 | 29 |
| **Placebo vs. ERAs** | 1 | 158 | 67 |
| **Control vs. Ascorbic Acid** | 1 | 156 | 10 |

Figure 2 Rankogram: ranking the interventions for the probability of being the best, the interventions are colour coded; the first column represent the chance of being first best and 2nd column is the chance of being 2nd best and so on. The overall numerical value is presented in table 4


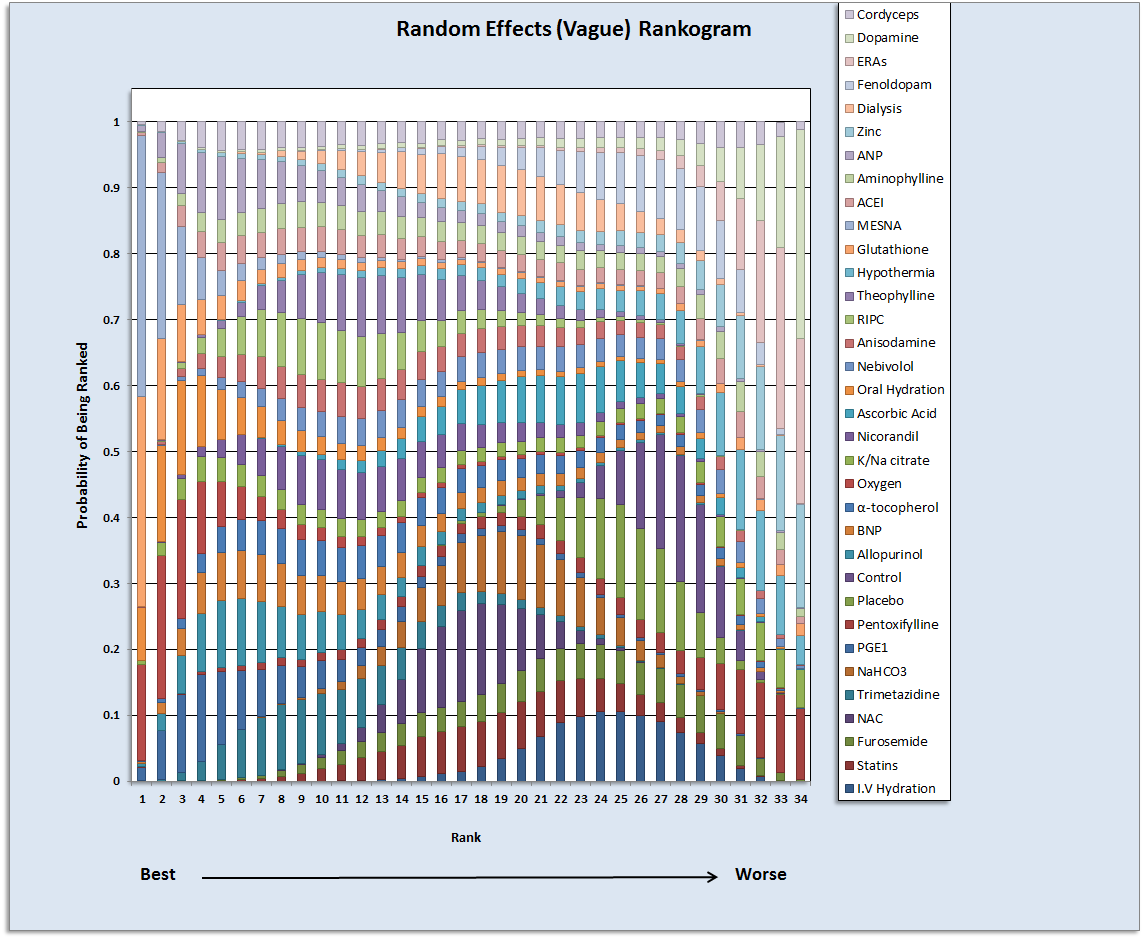


Table 4 Interventions ranking the names column follow the league table (which arranges the presentation of summary estimates by ranking the treatments in order of most pronounced impact on the outcome under consideration) the numerical values represents the cumulative results of the probability of being best in which the highest score is 1 or 100% (see Rankogram)

| **Treatment** | **SUCRA** | **Treatment** | **SUCRA** |
| --- | --- | --- | --- |
| **MESNA** | 0.9319 | **Nebivolol** | 0.4708 |
| **Oxygen** | 0.8727 | **Dialysis** | 0.4582 |
| **Oral Hydration** | 0.7916 | **Statins** | 0.4514 |
| **PGE1** | 0.7897 | **K/Na citrate** | 0.4451 |
| **Glutathione** | 0.7892 | **NaHCO3** | 0.4367 |
| **Allopurinol** | 0.7496 | **Ascorbic Acid** | 0.3861 |
| **ANP** | 0.7221 | **Furosemide** | 0.3671 |
| **Trimetazidine** | 0.7052 | **I.V Hydration** | 0.2946 |
| **RIPC** | 0.6731 | **Placebo** | 0.2623 |
| **BNP** | 0.6658 | **Pentoxifylline** | 0.2404 |
| **Nicorandil** | 0.6314 | **Hypothermia** | 0.2237 |
| **Theophylline** | 0.6148 | **Zinc** | 0.2064 |
| **α-tocopherol** | 0.5822 | **Control** | 0.1978 |
| **Anisodamine** | 0.5586 | **Fenoldopam** | 0.2554 |
| **ACEI** | 0.5207 | **ERAs** | 0.06881 |
| **NAC** | 0.5008 | **Dopamine** | 0.1314 |
| **Aminophylline** | 0.4858 | **Cordyceps** | 0.5184 |
| ***Analysis*** | **Random Effects (Vague)** | | |

Figure 3 Forest Plot


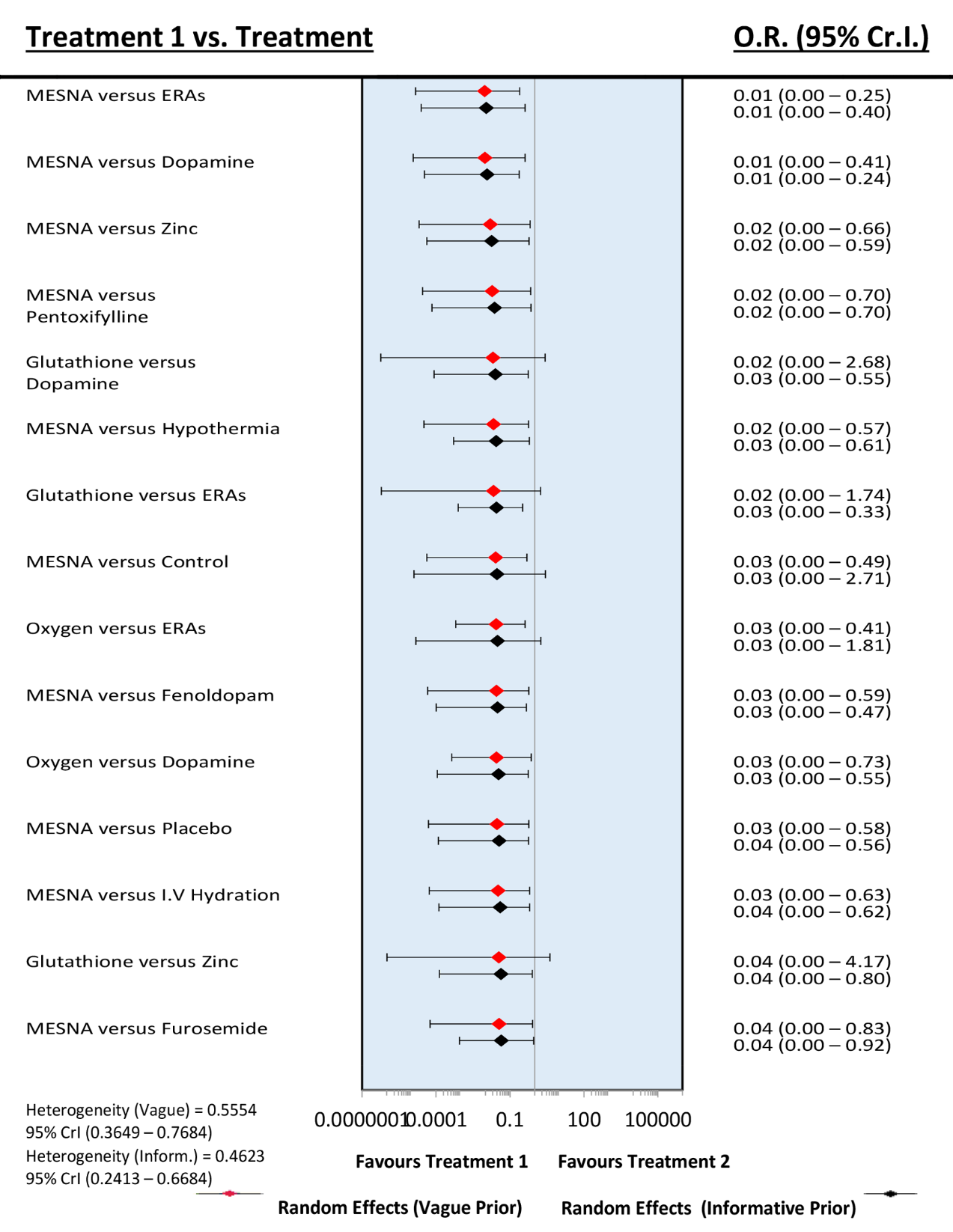


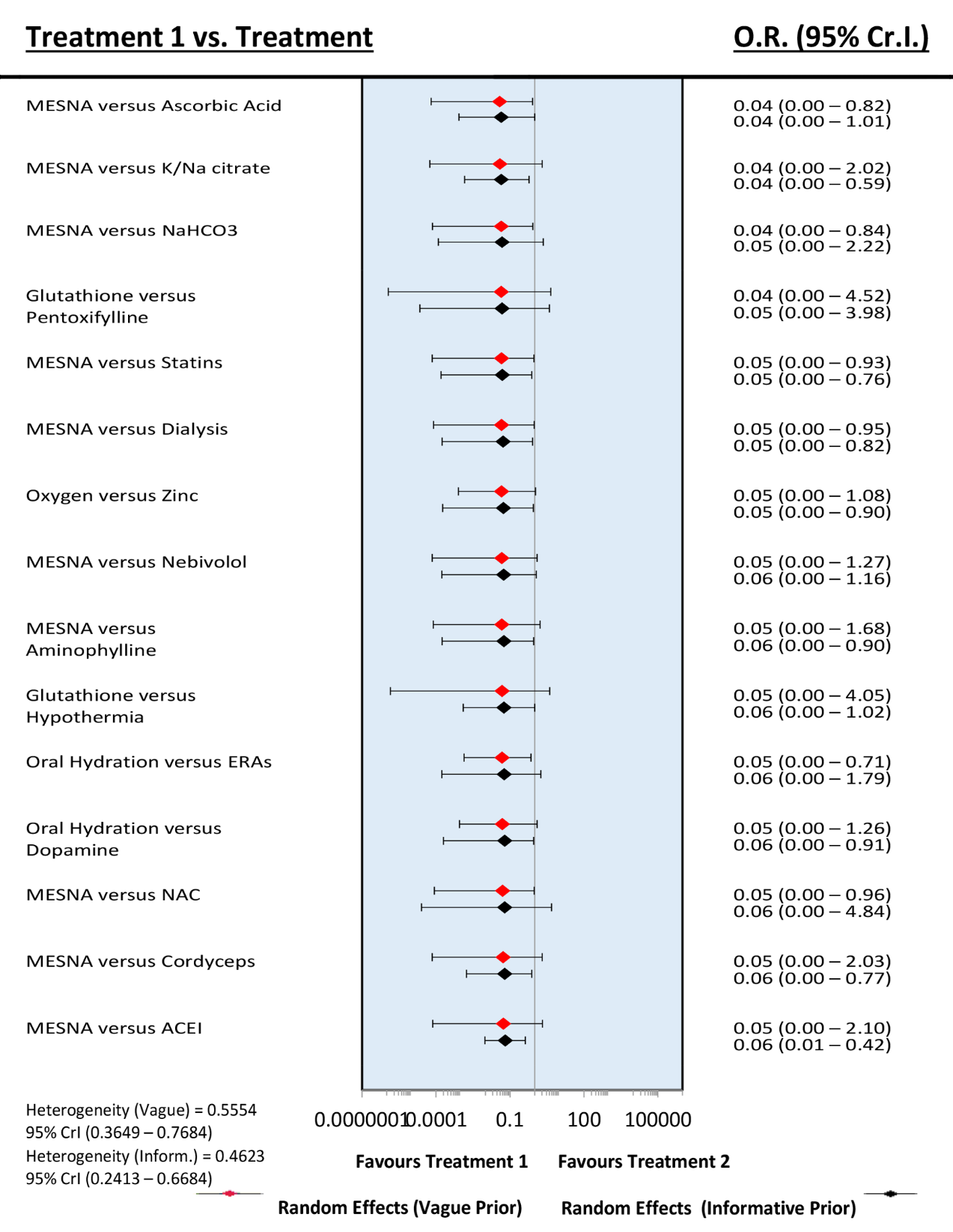


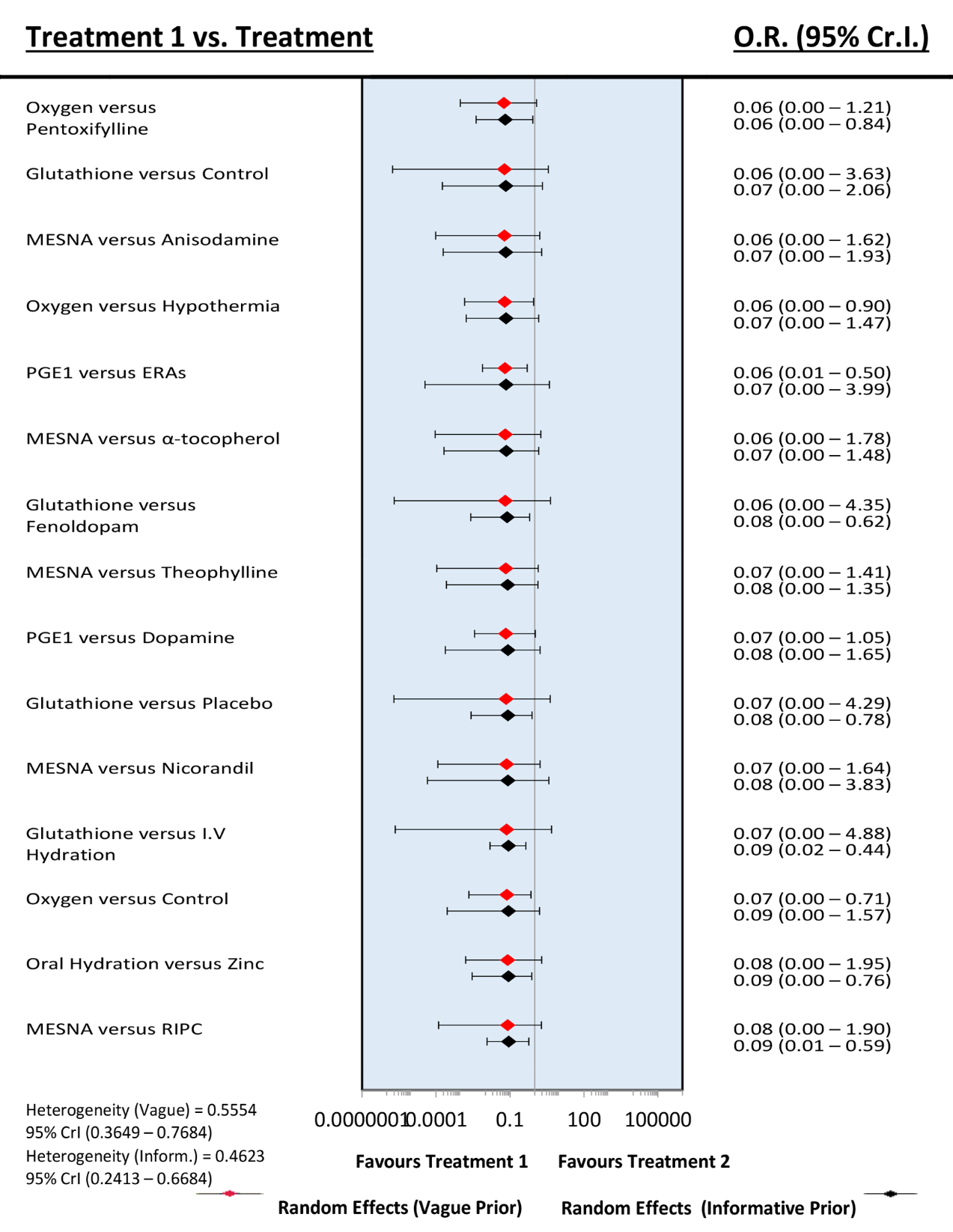


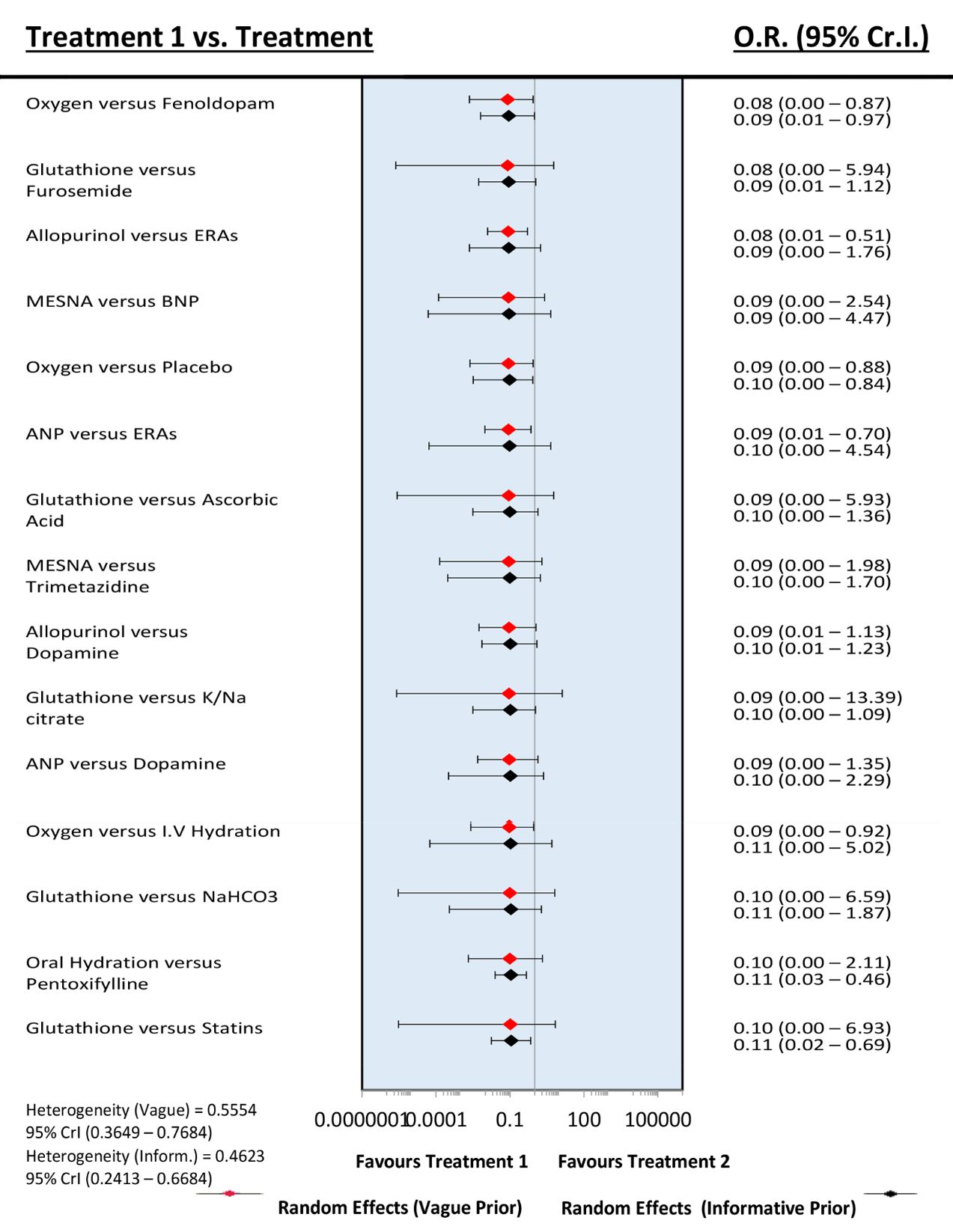


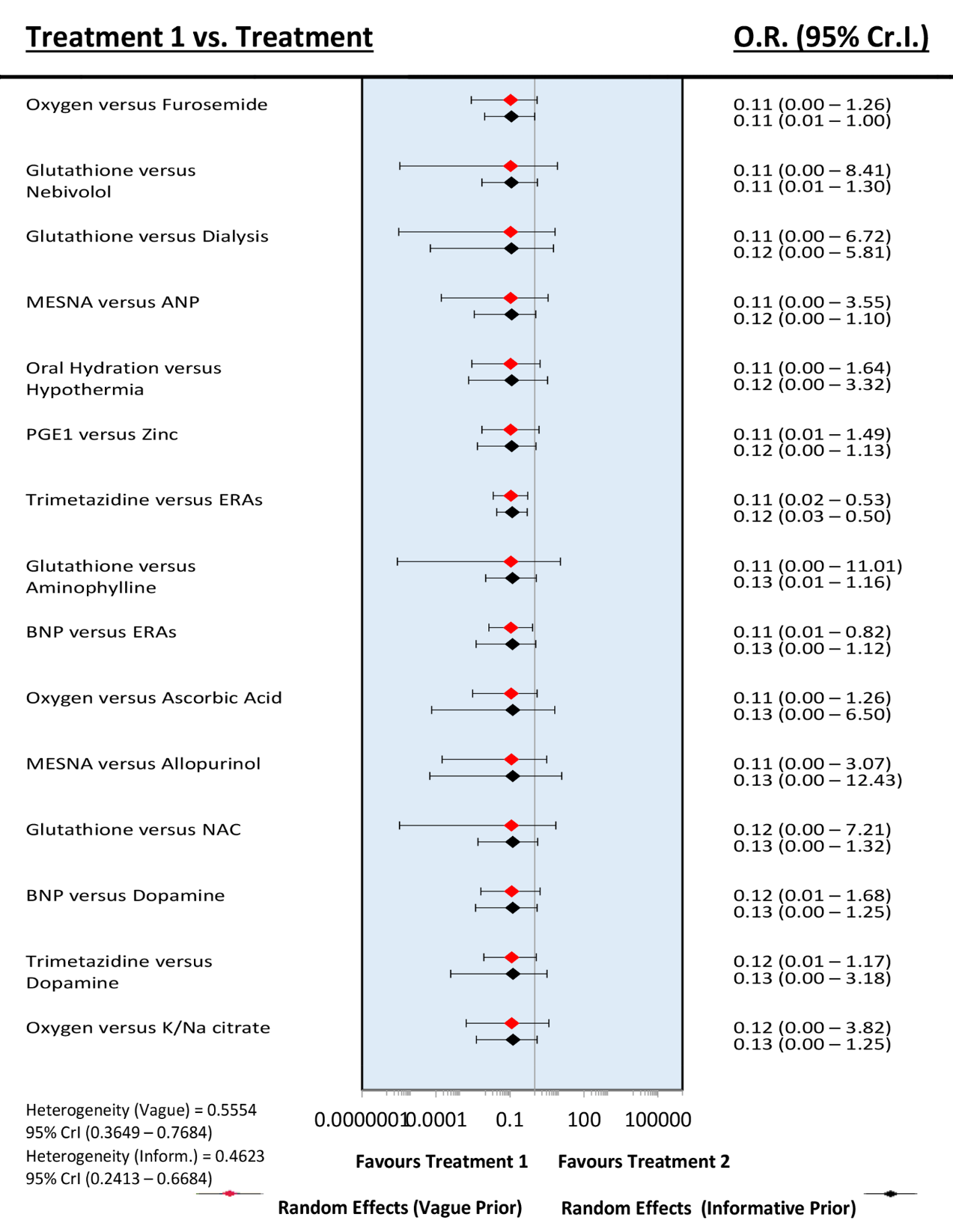


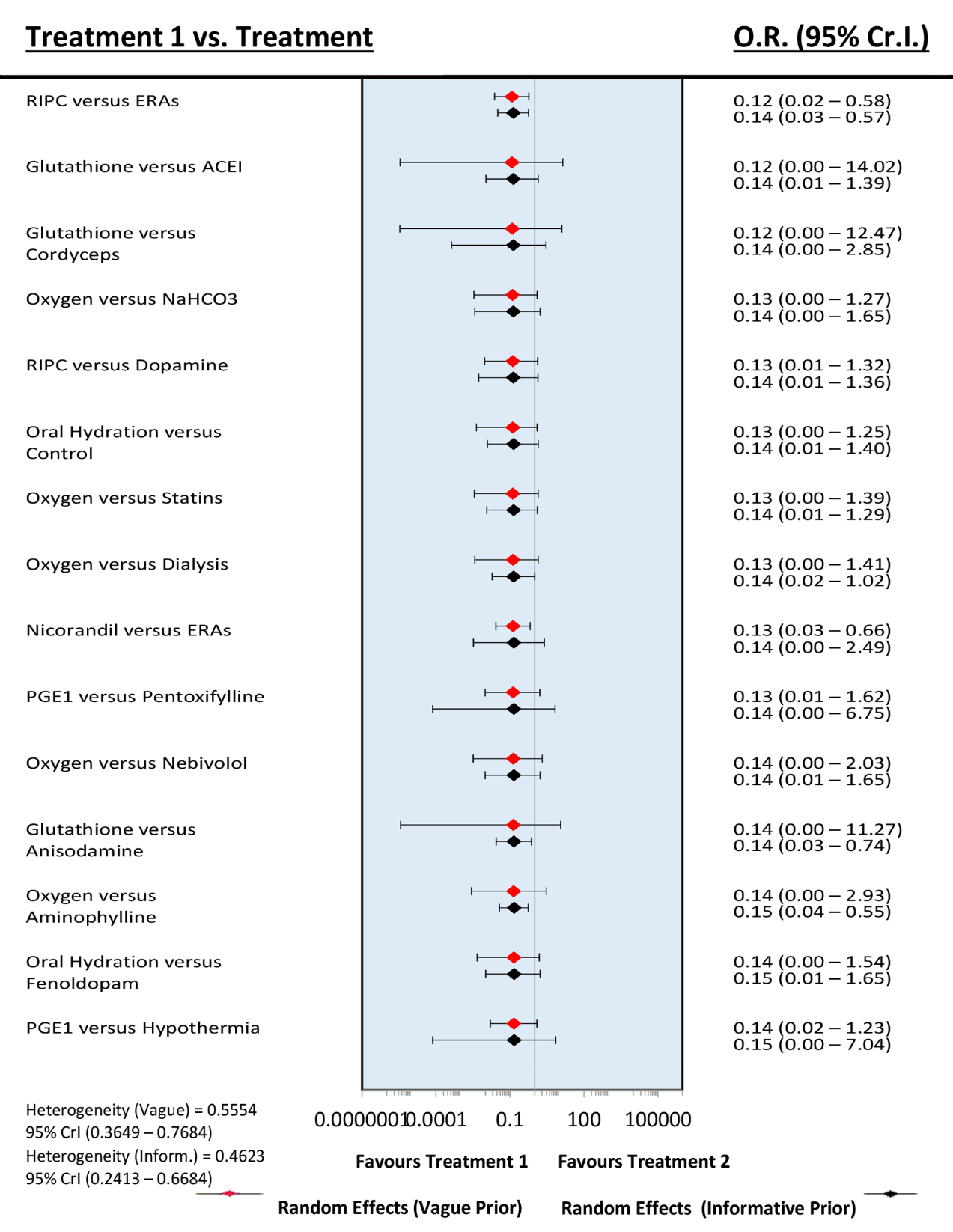


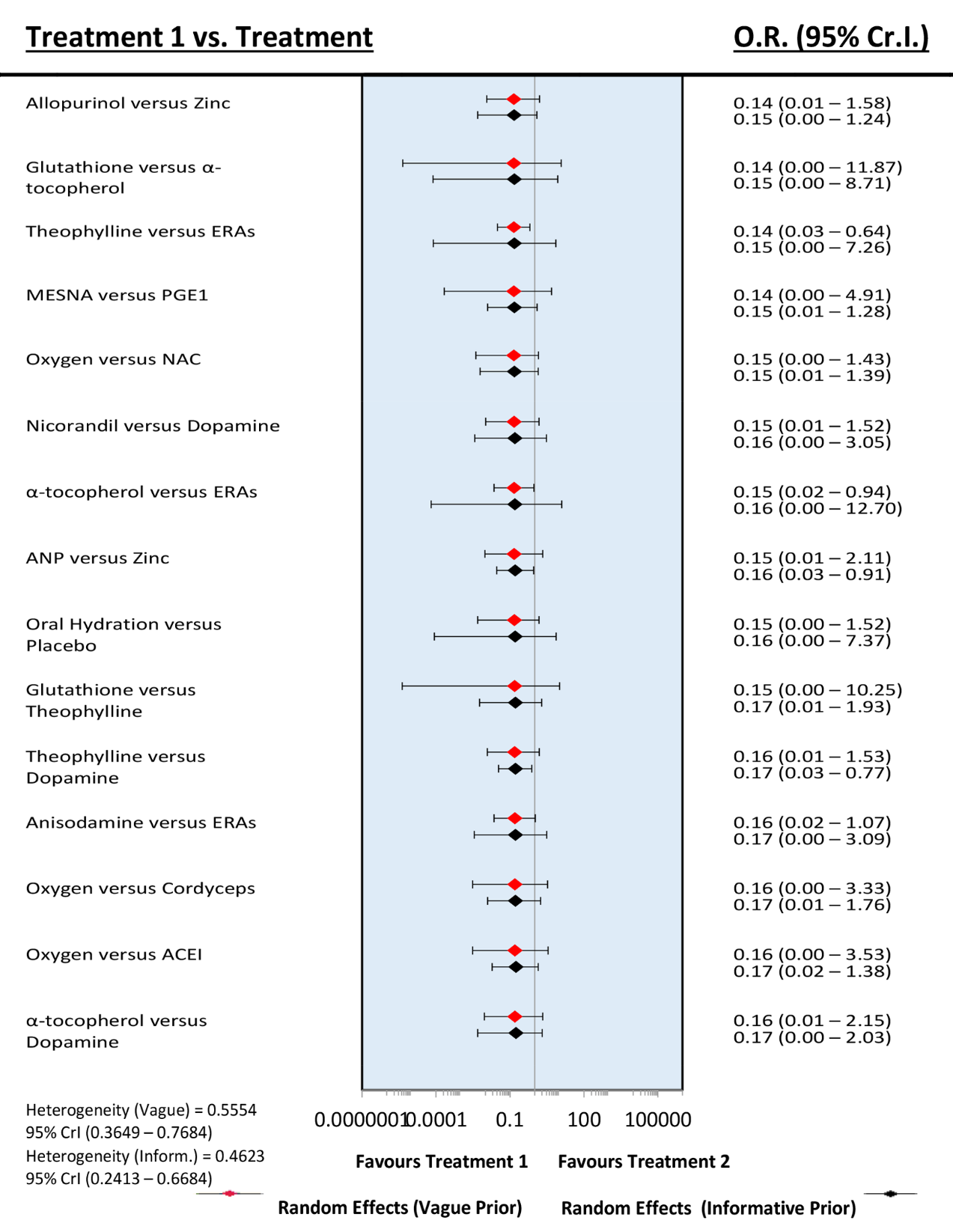


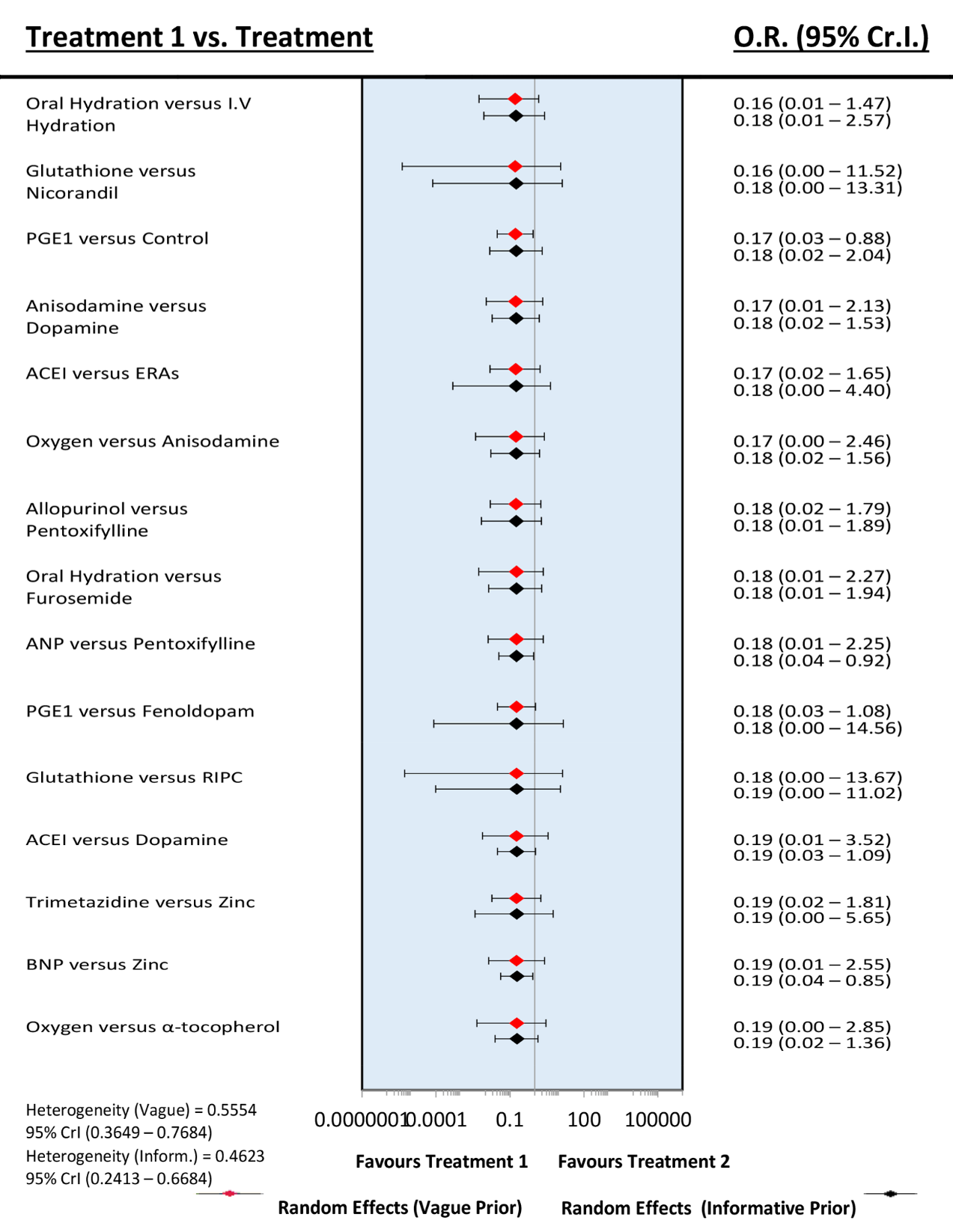


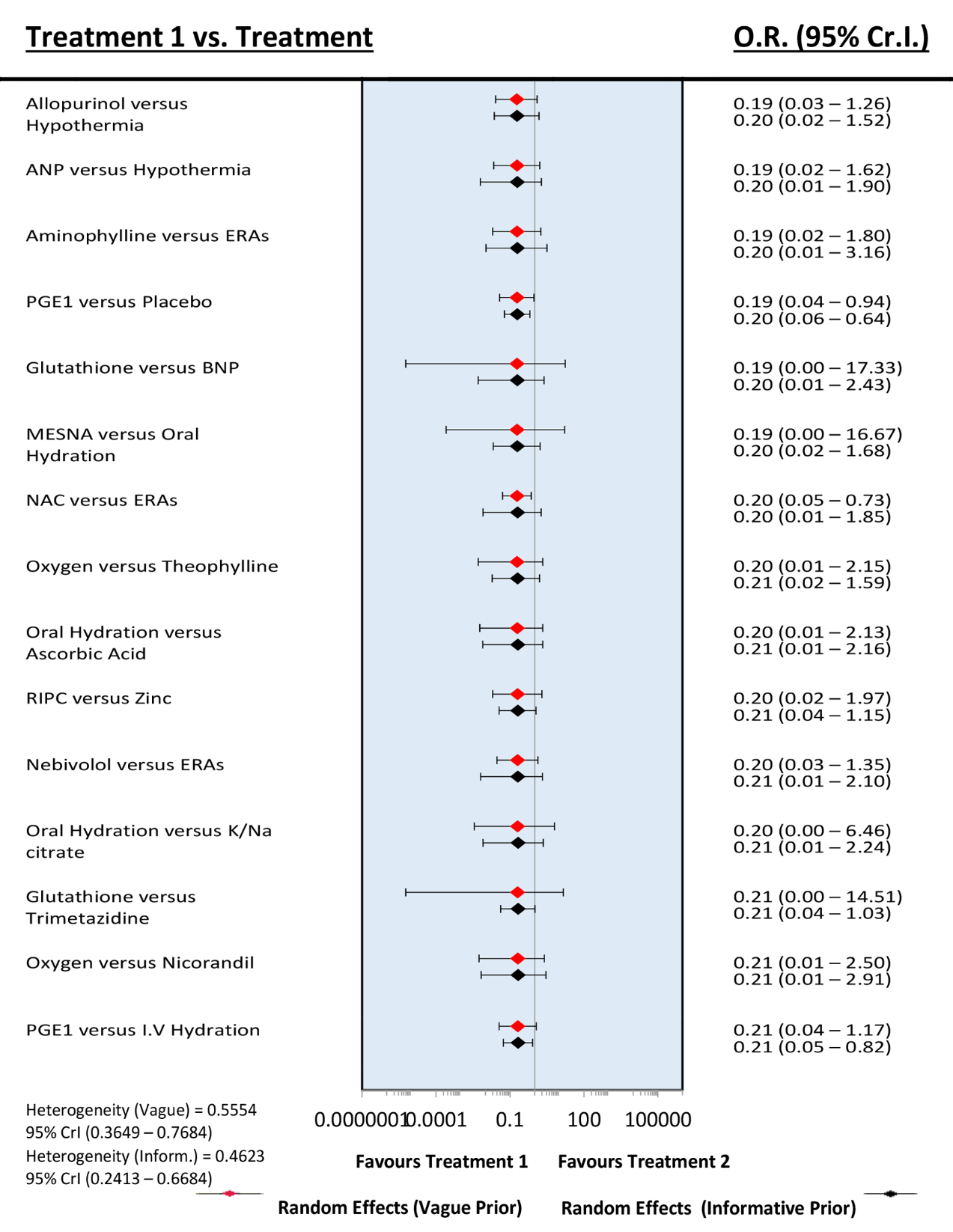


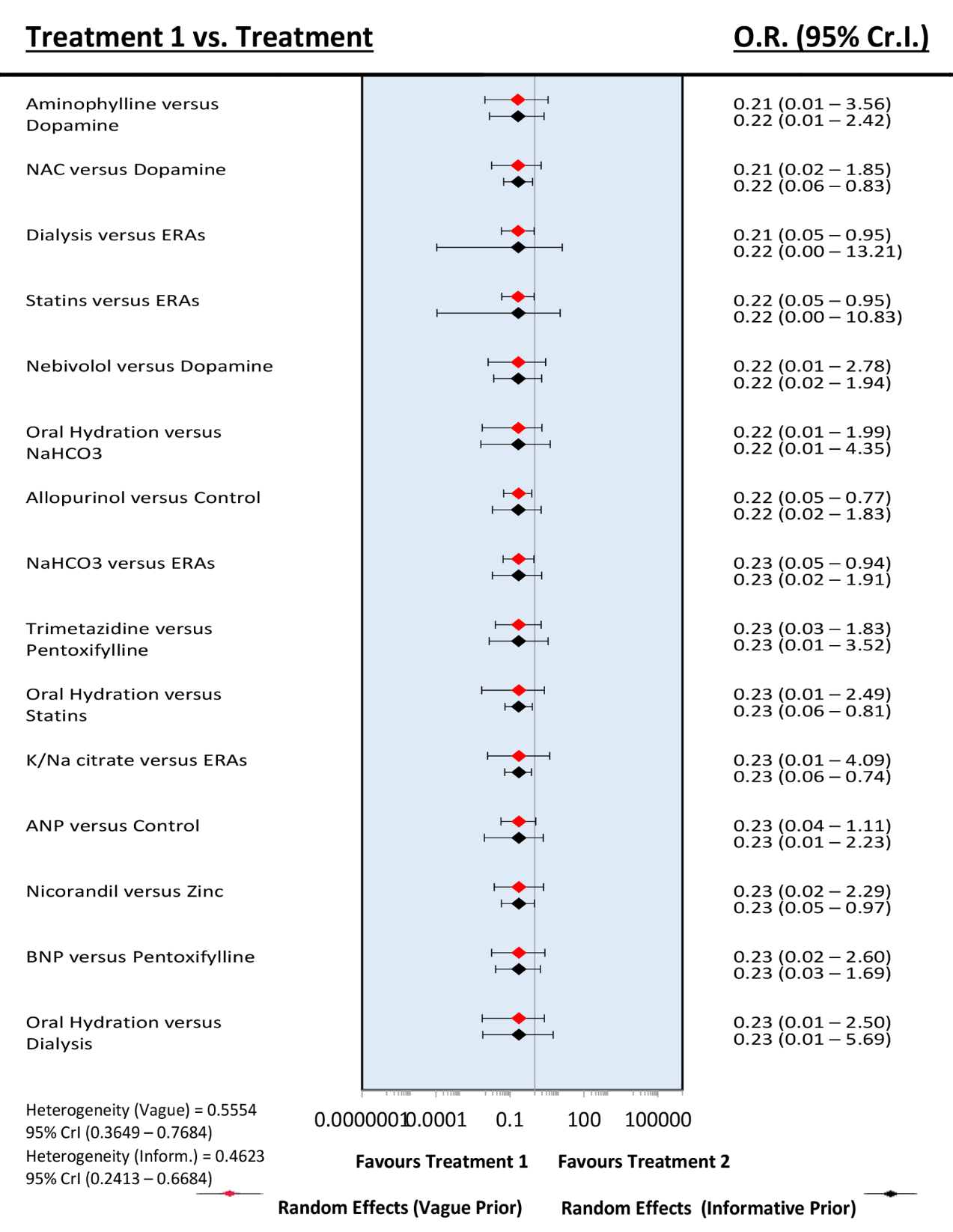


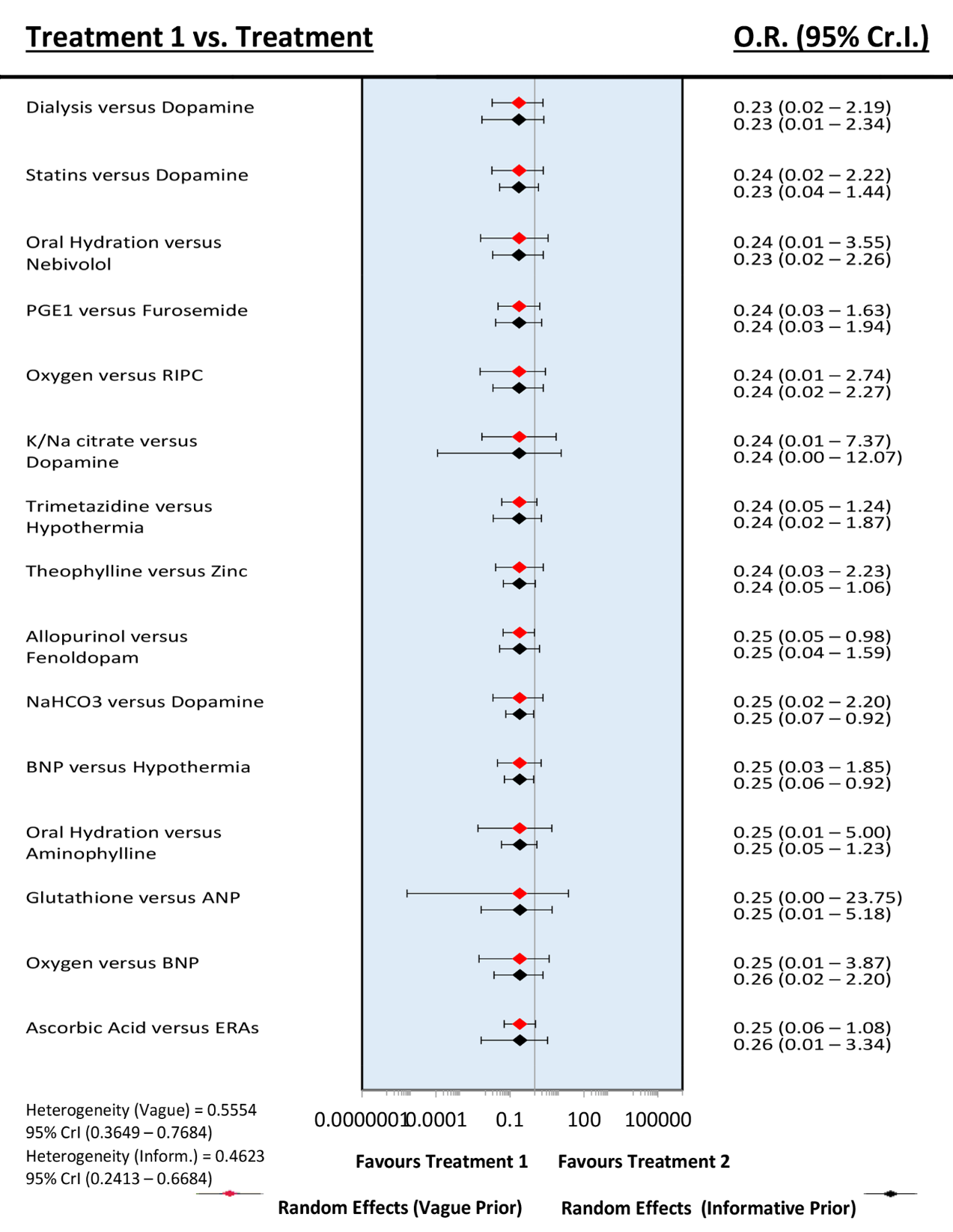


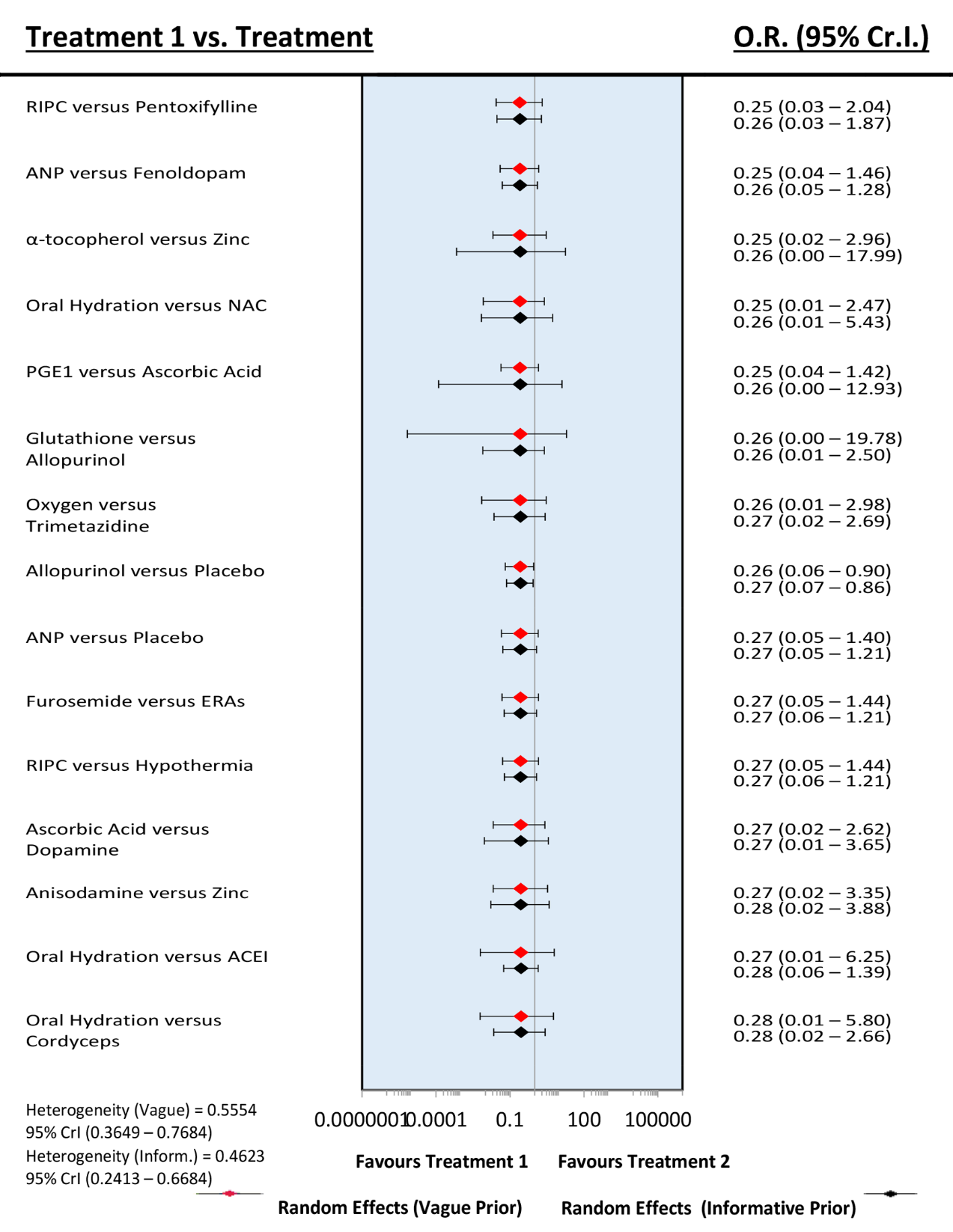


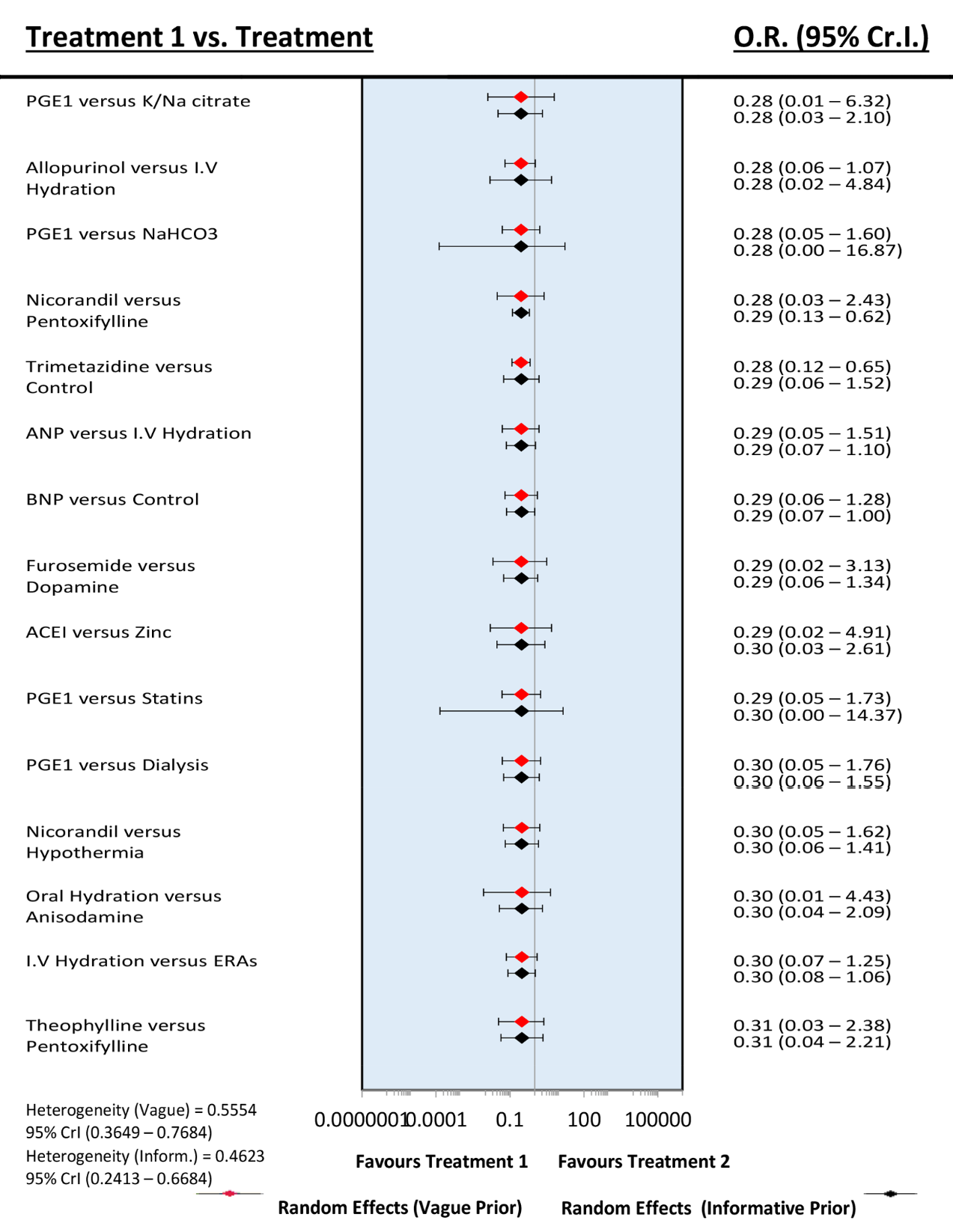


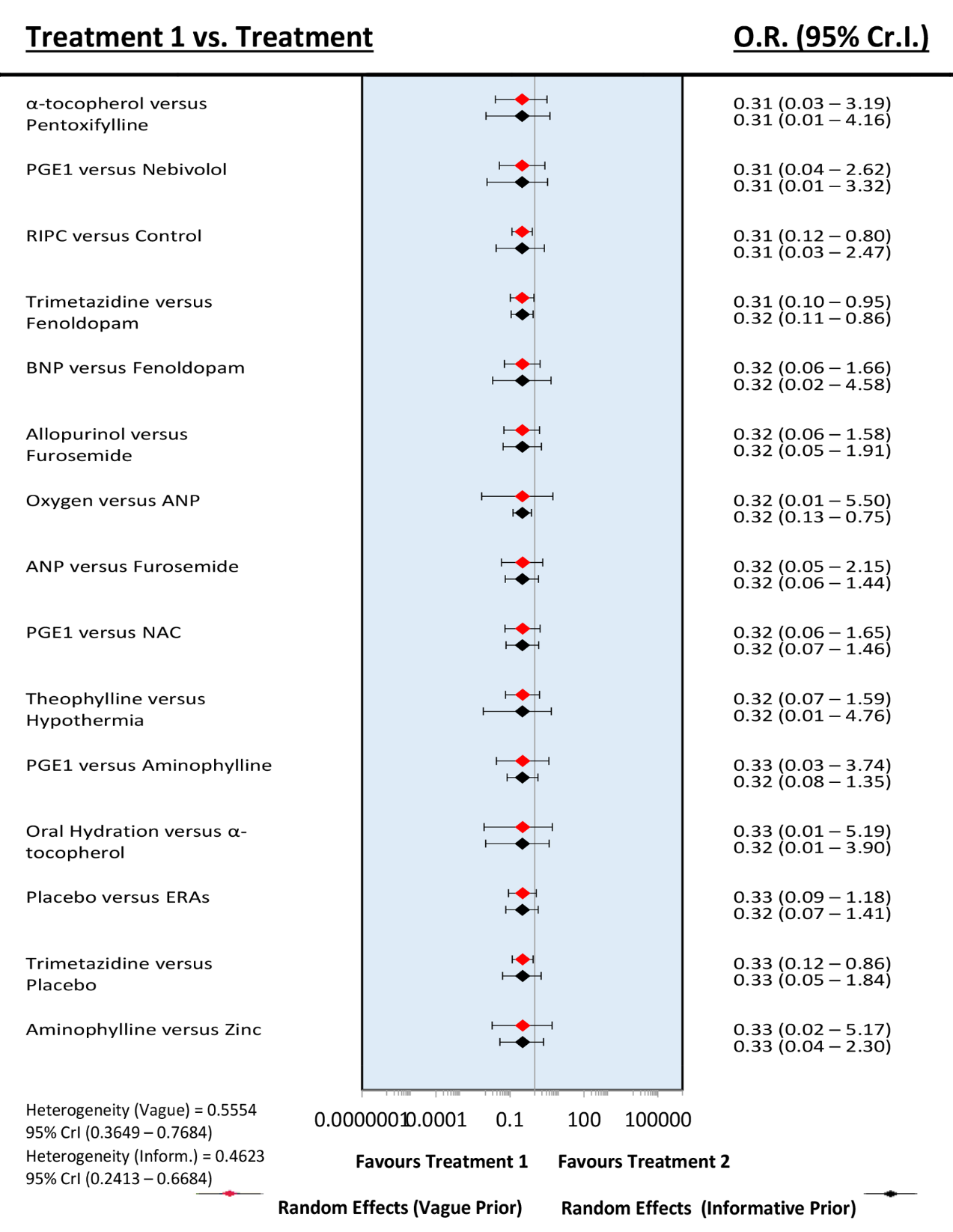


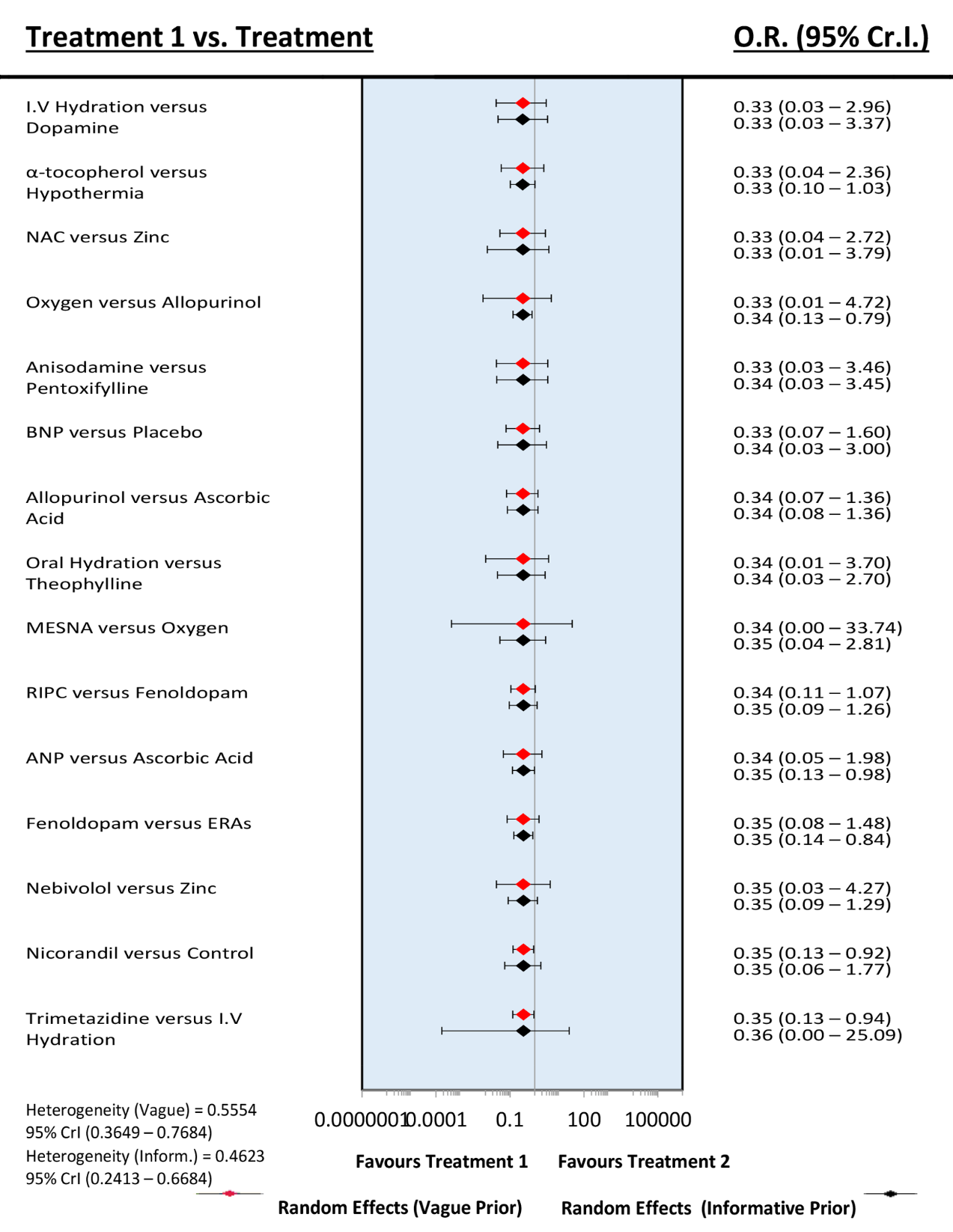


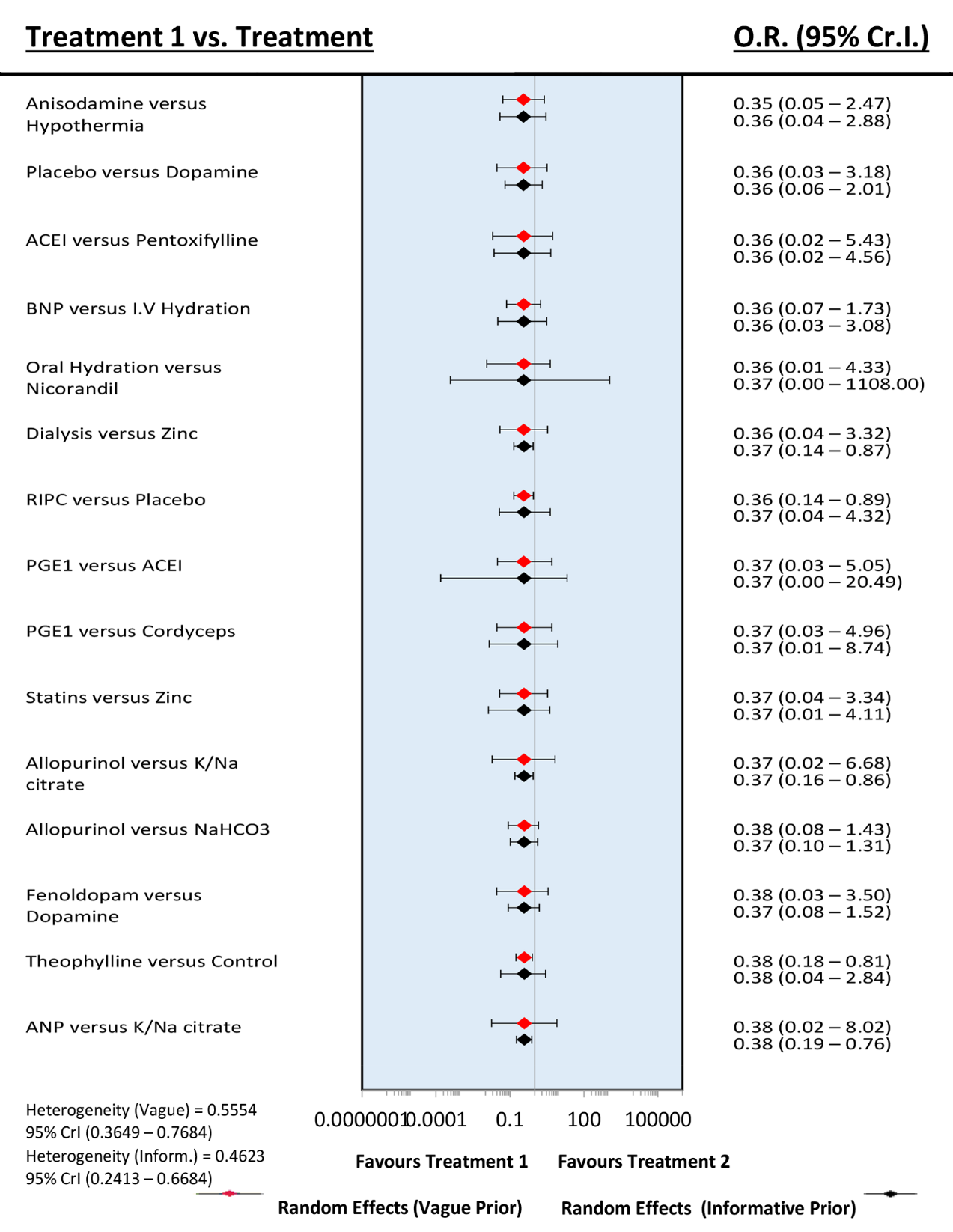


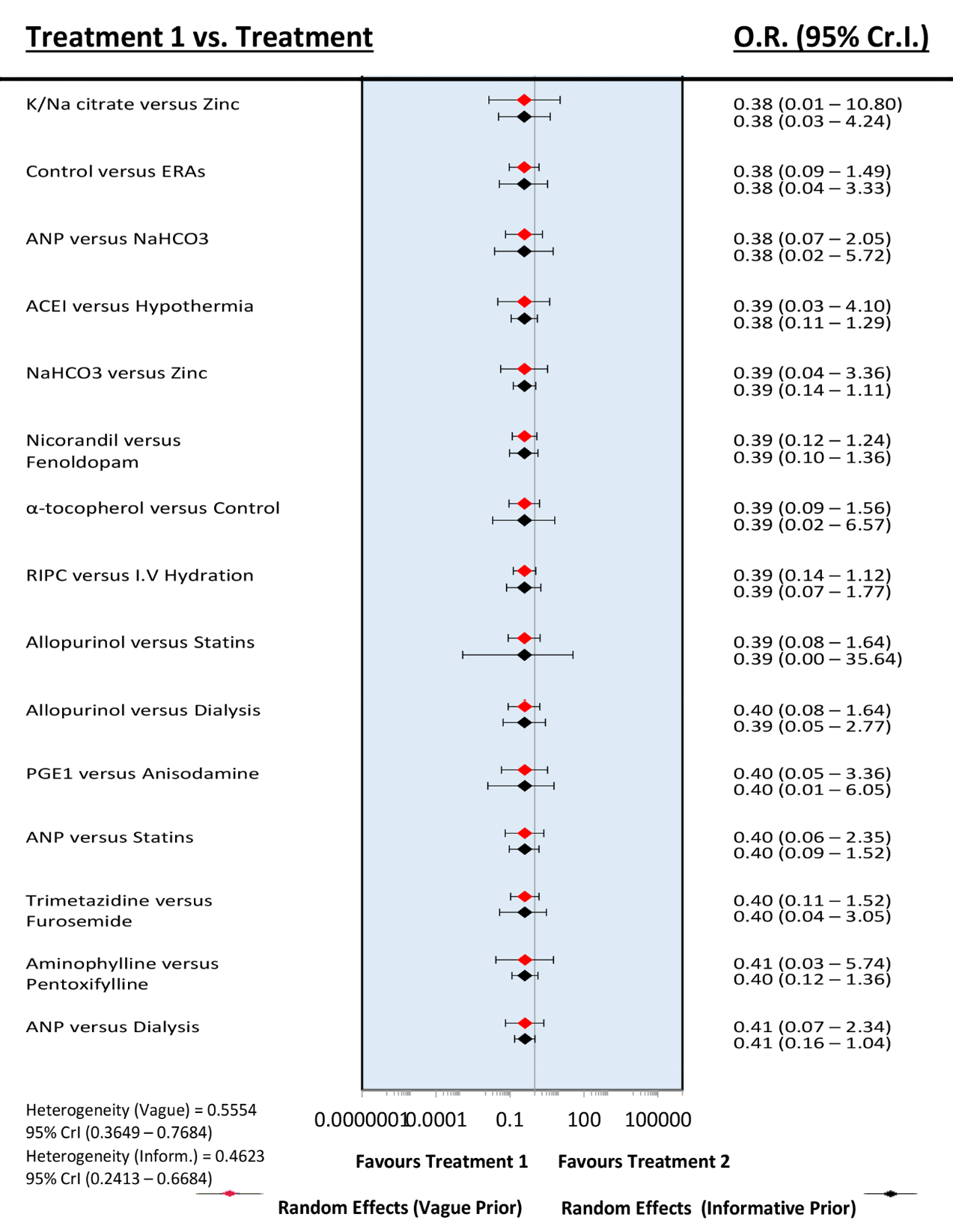


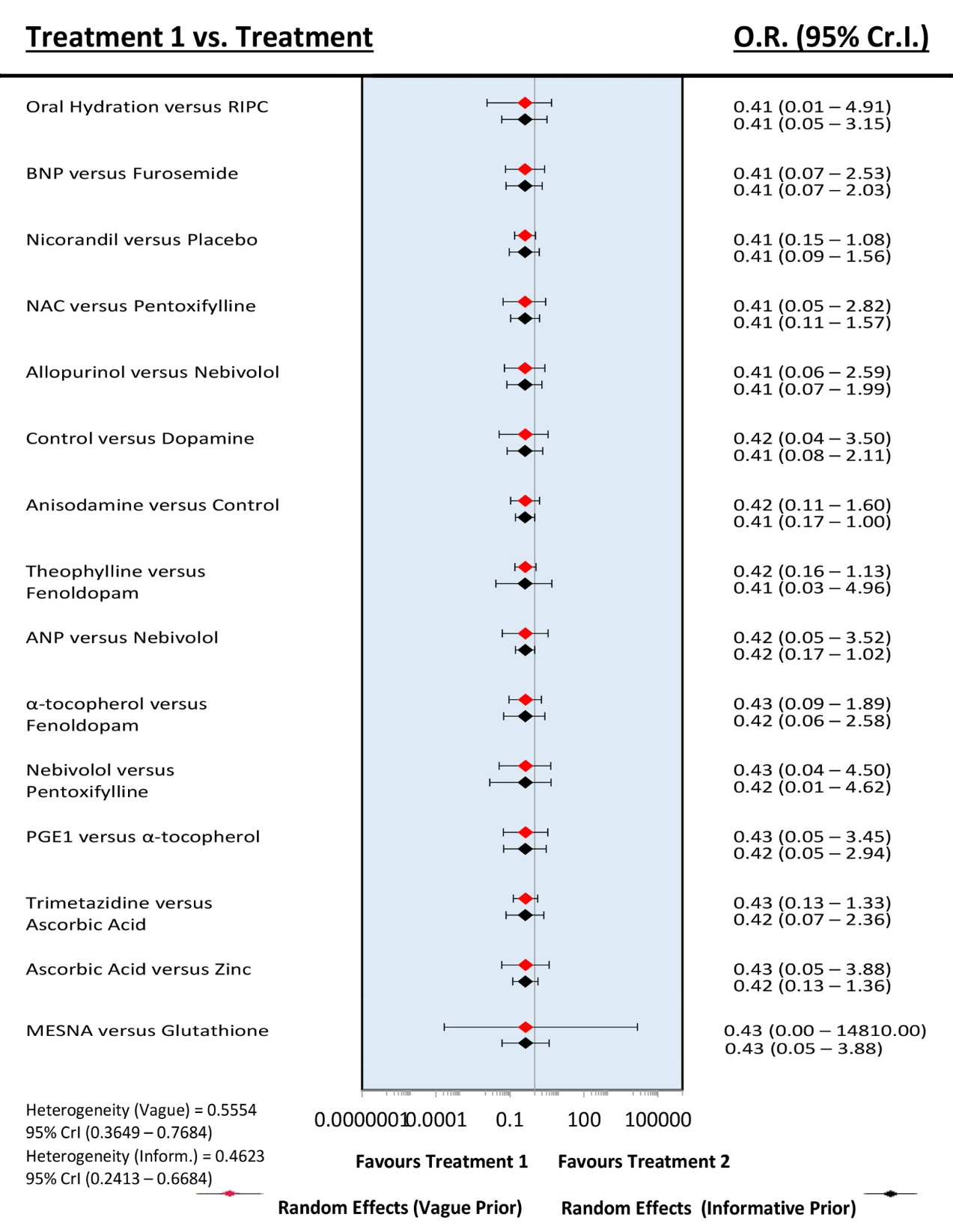


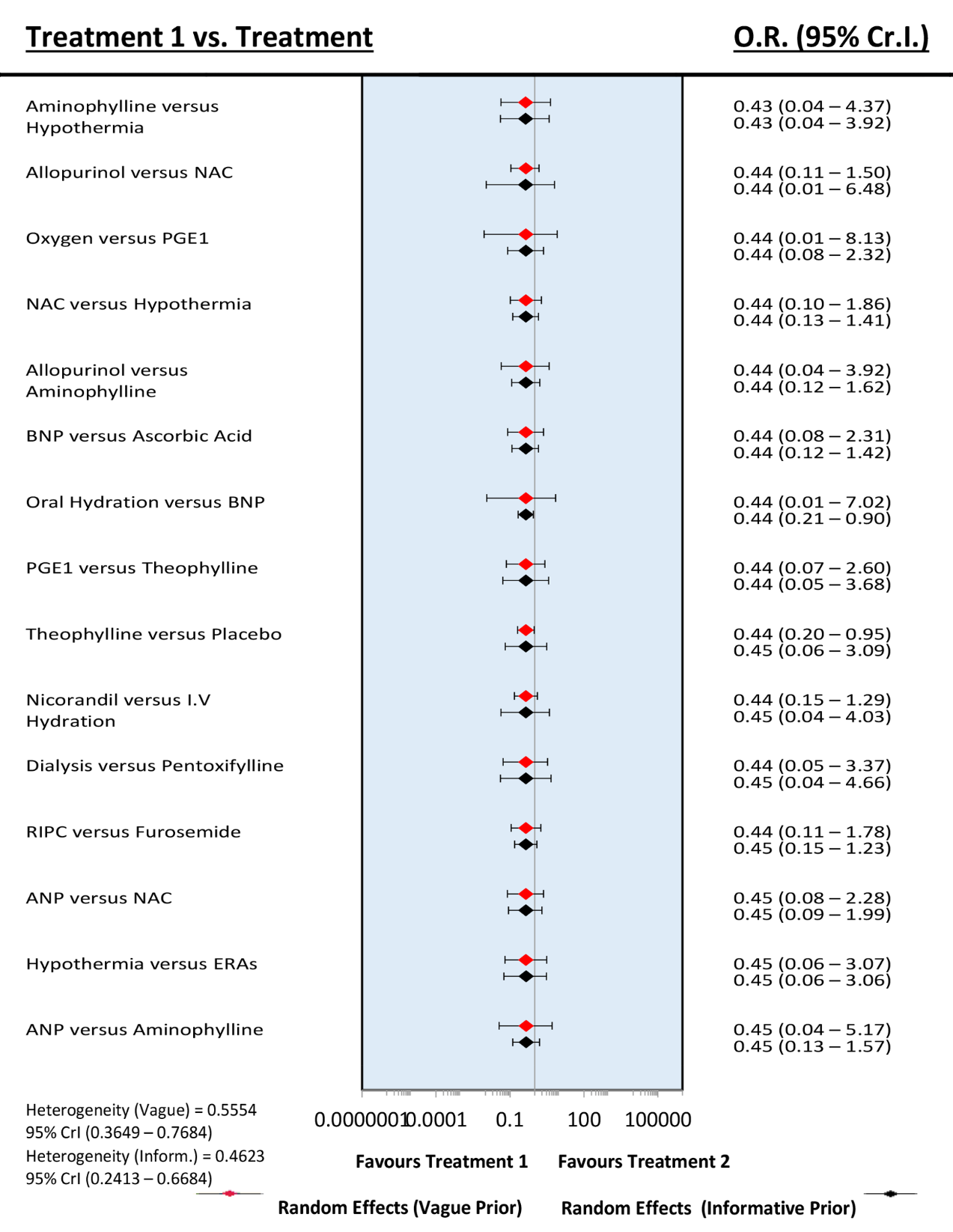


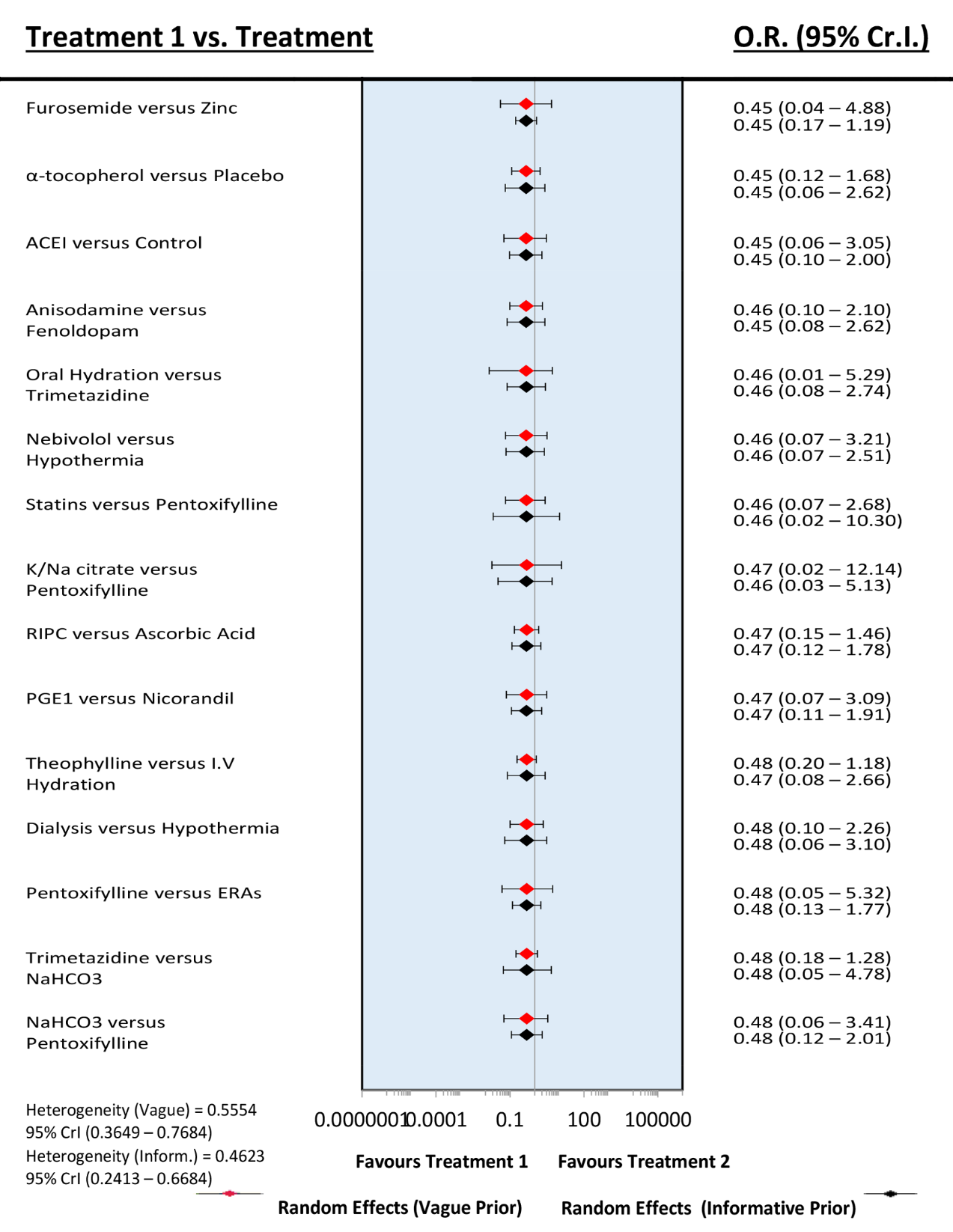


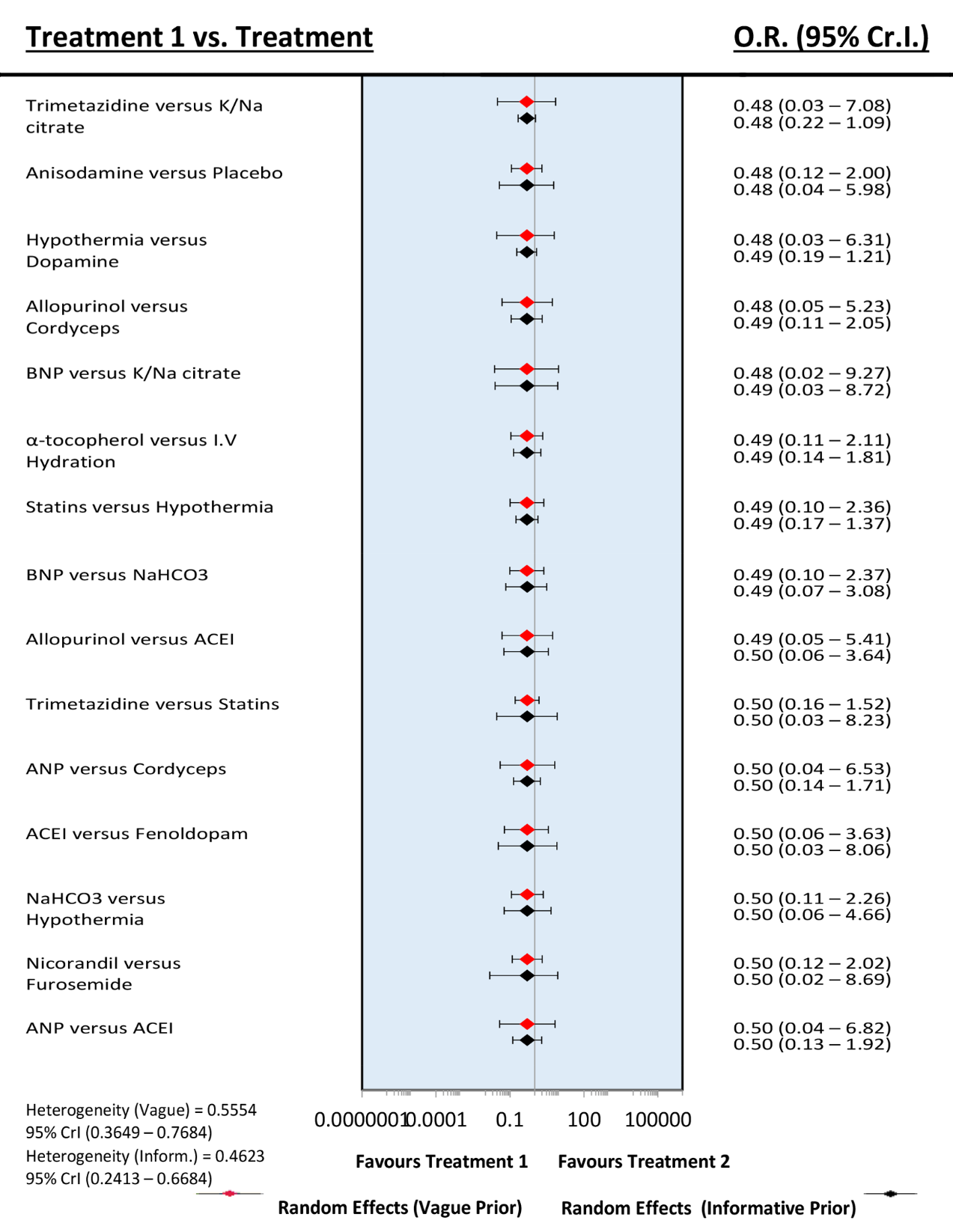


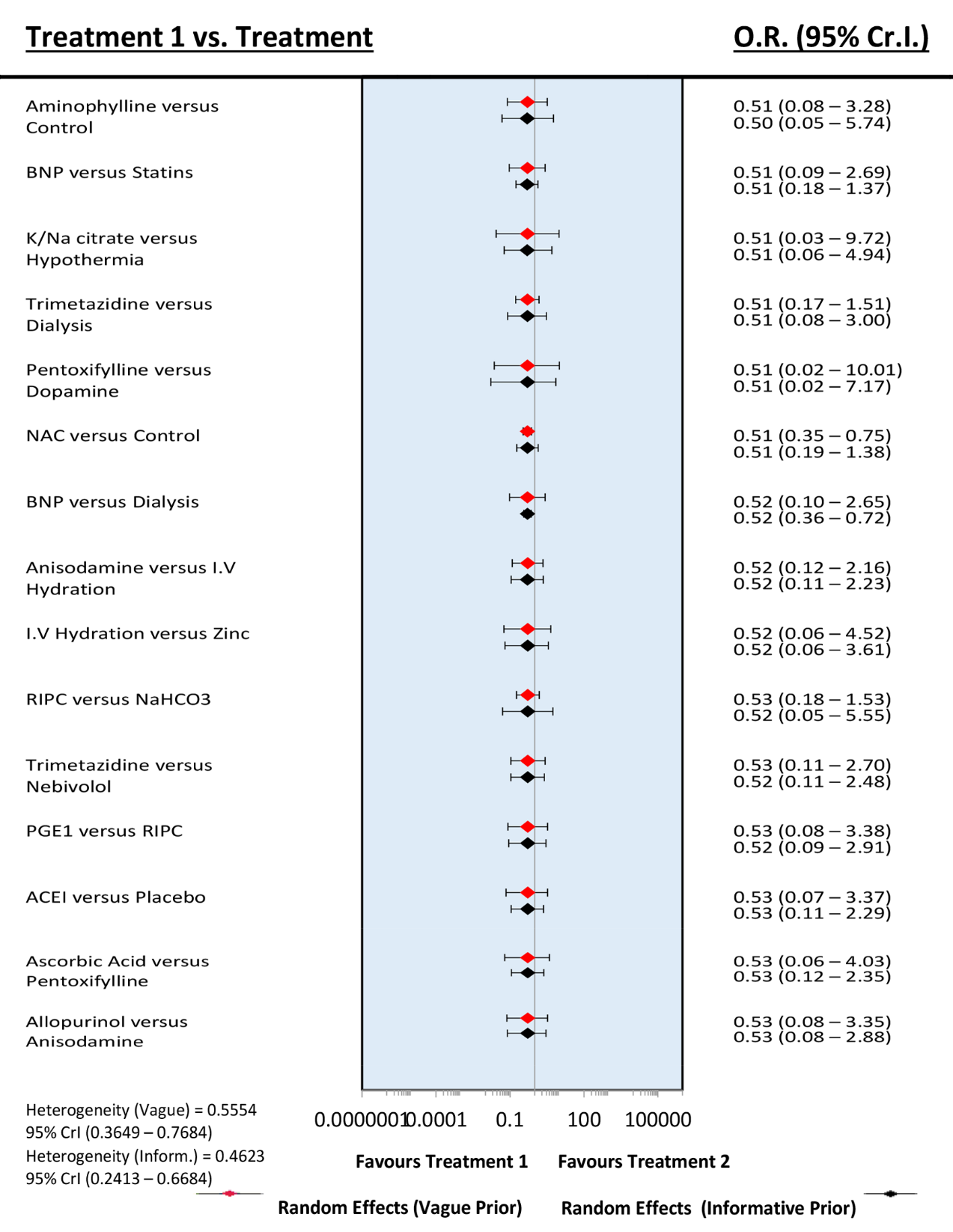


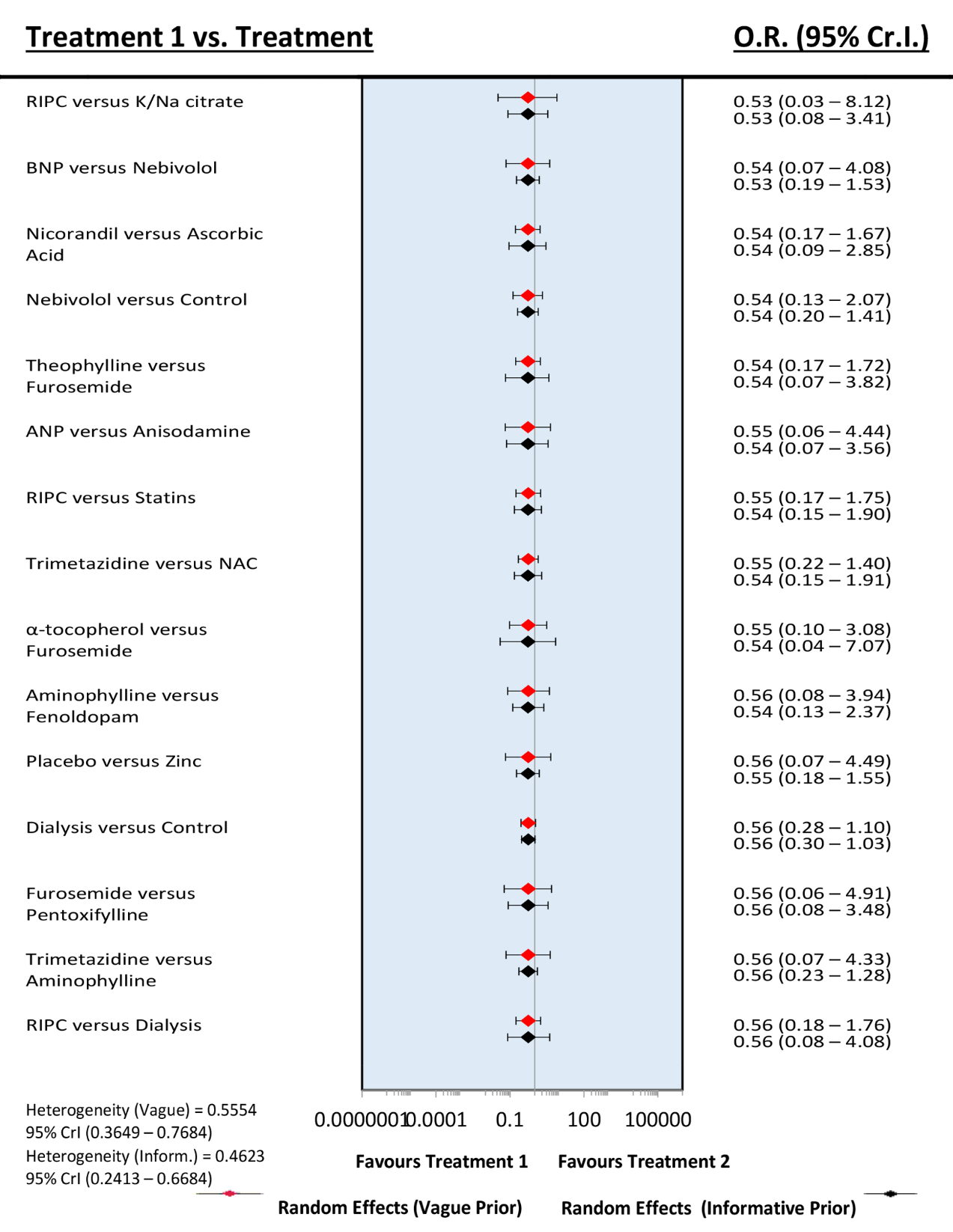


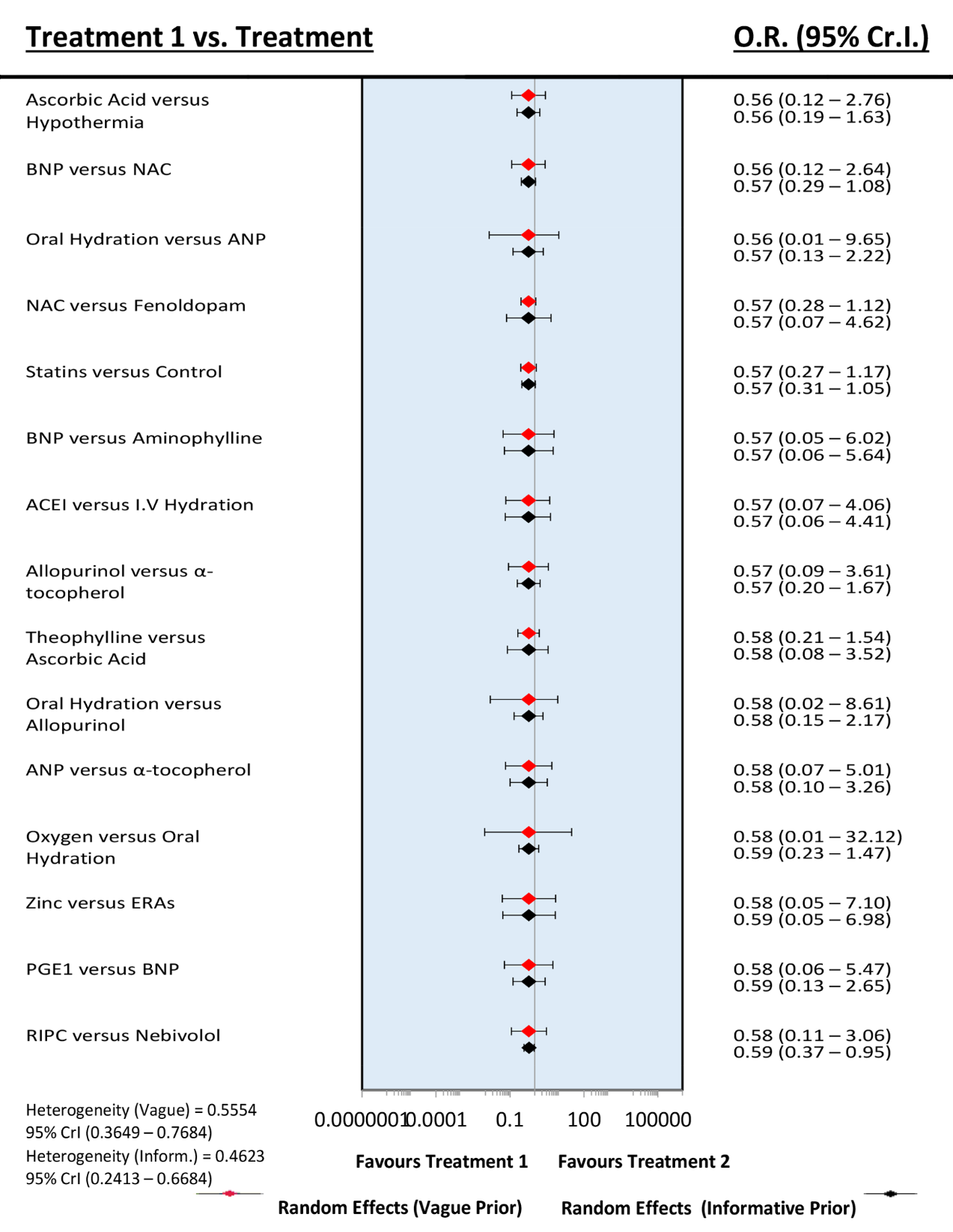


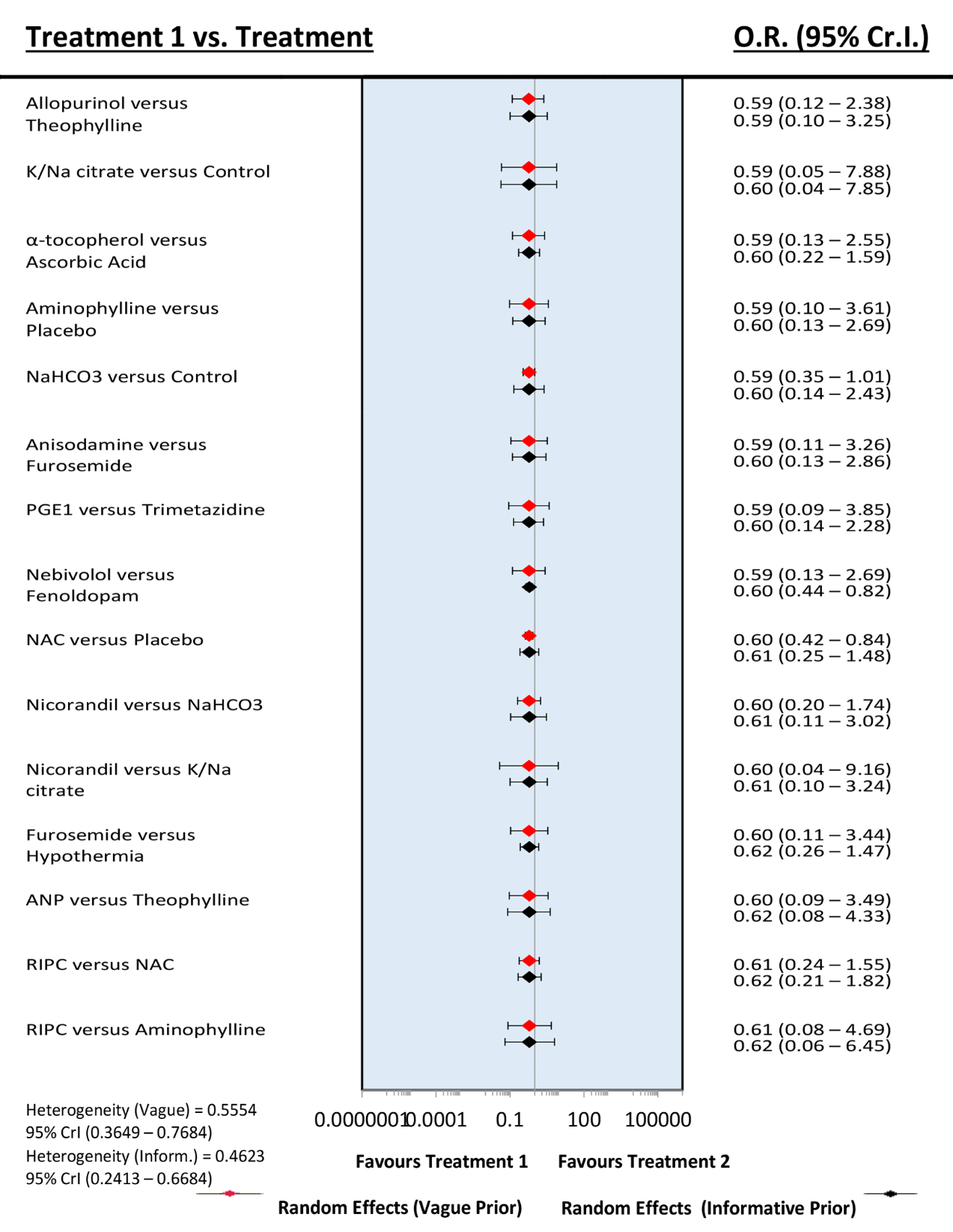


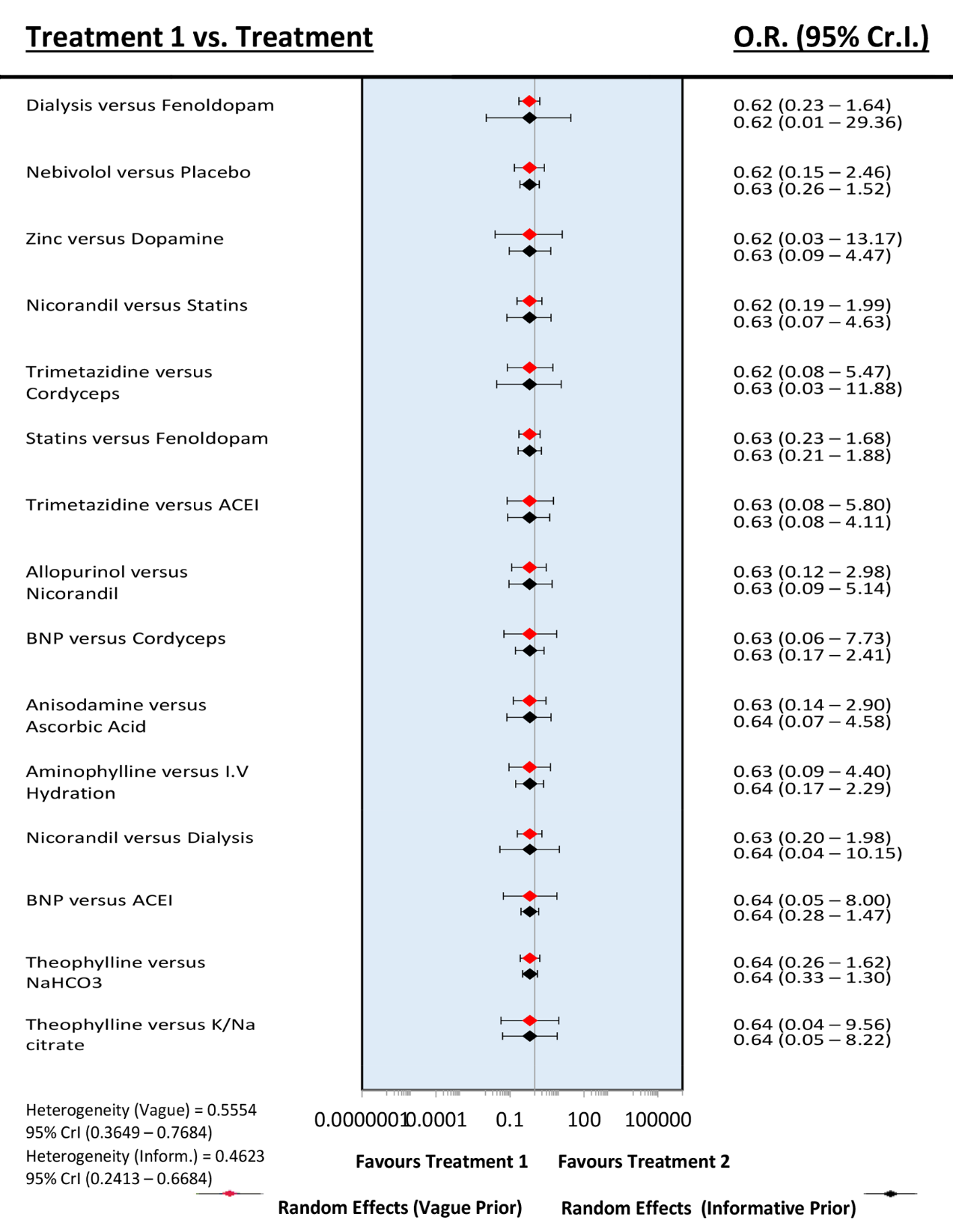


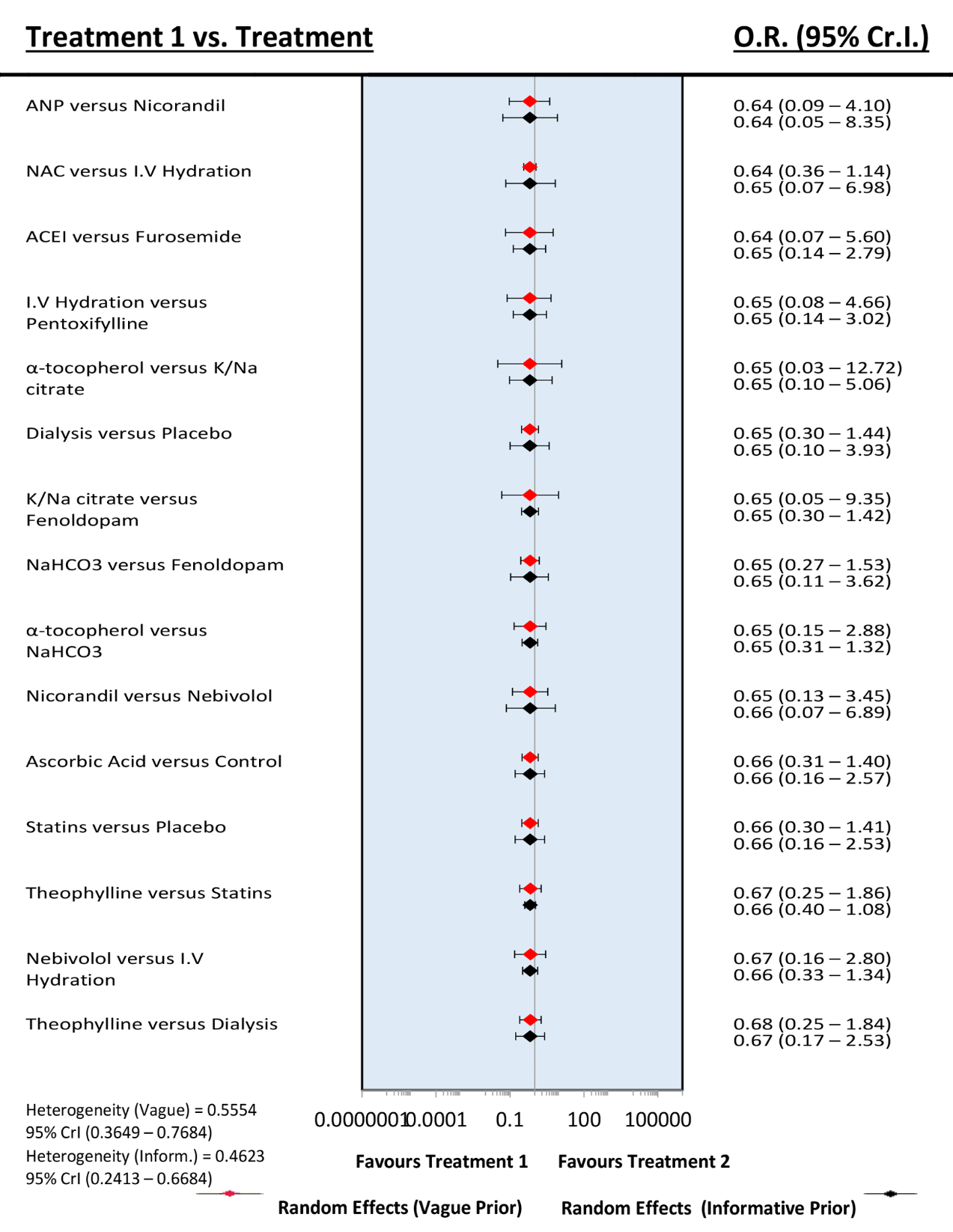


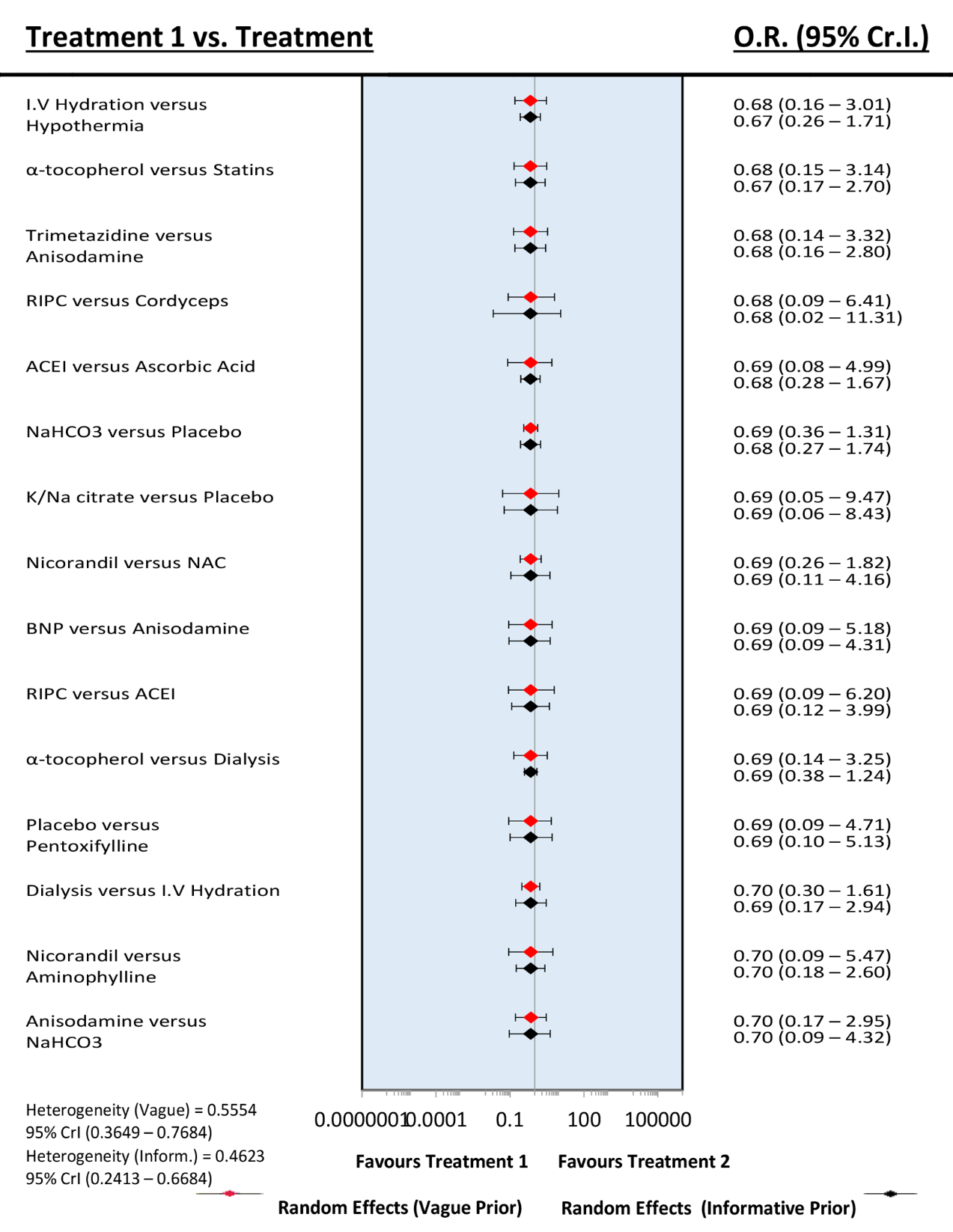


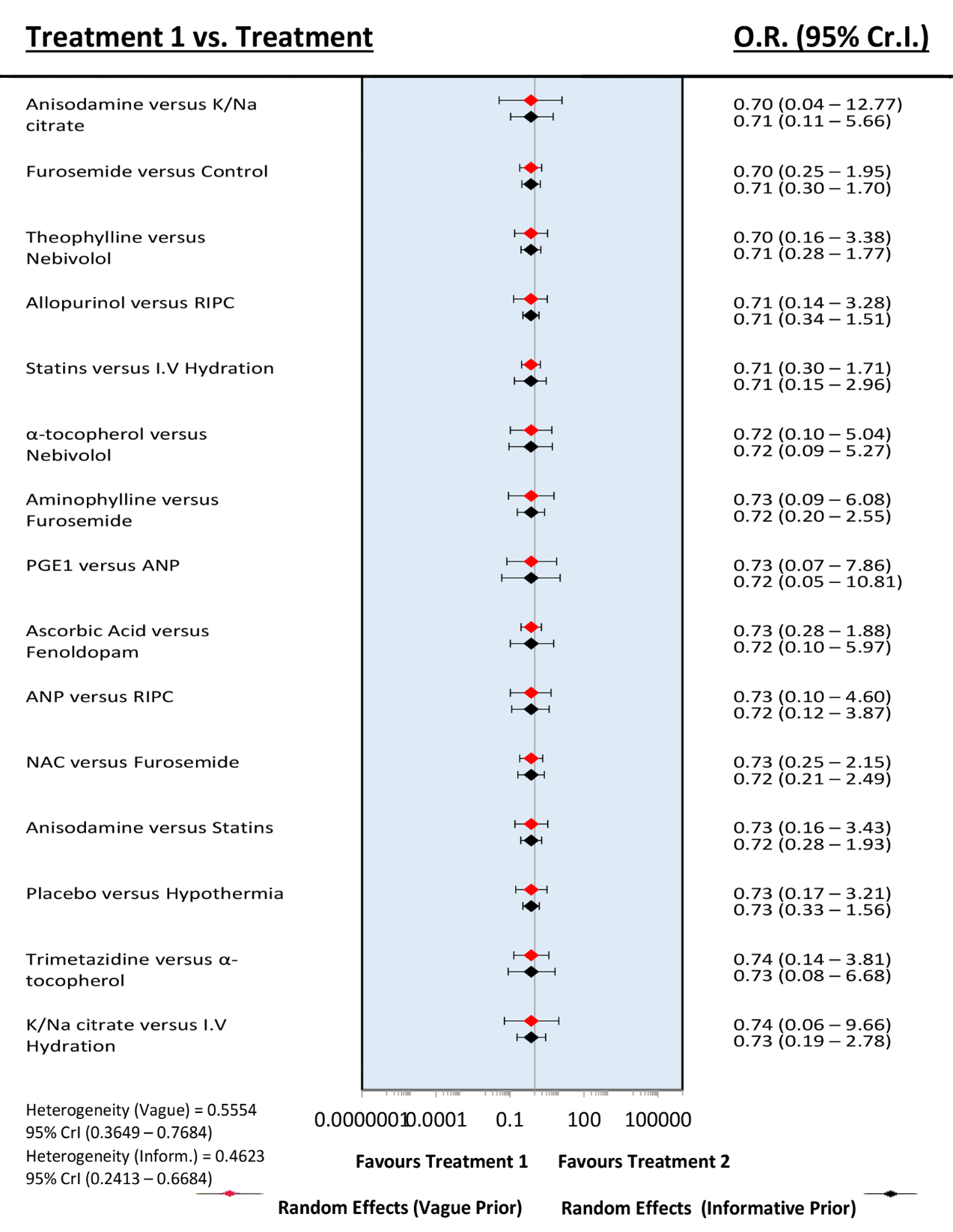


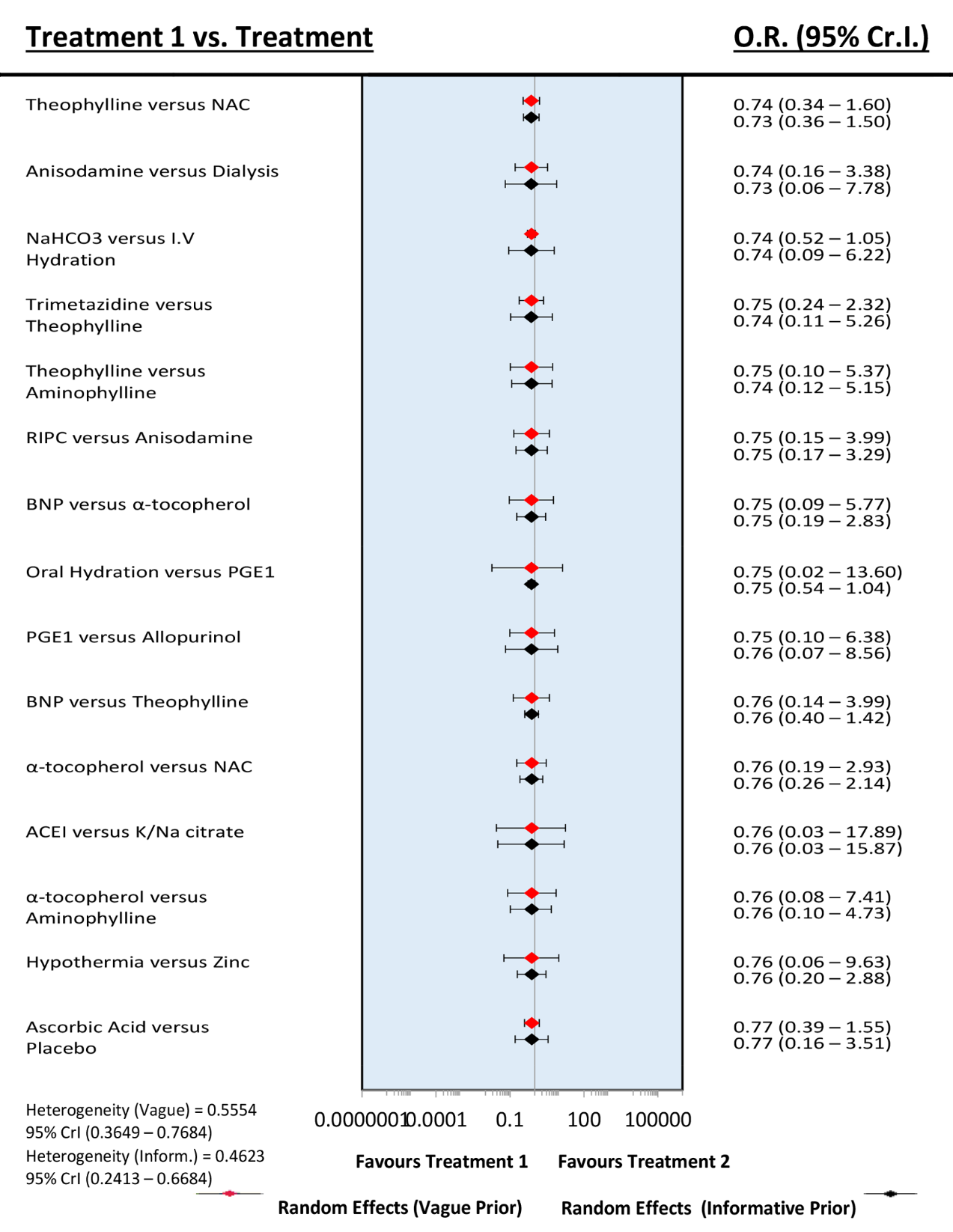


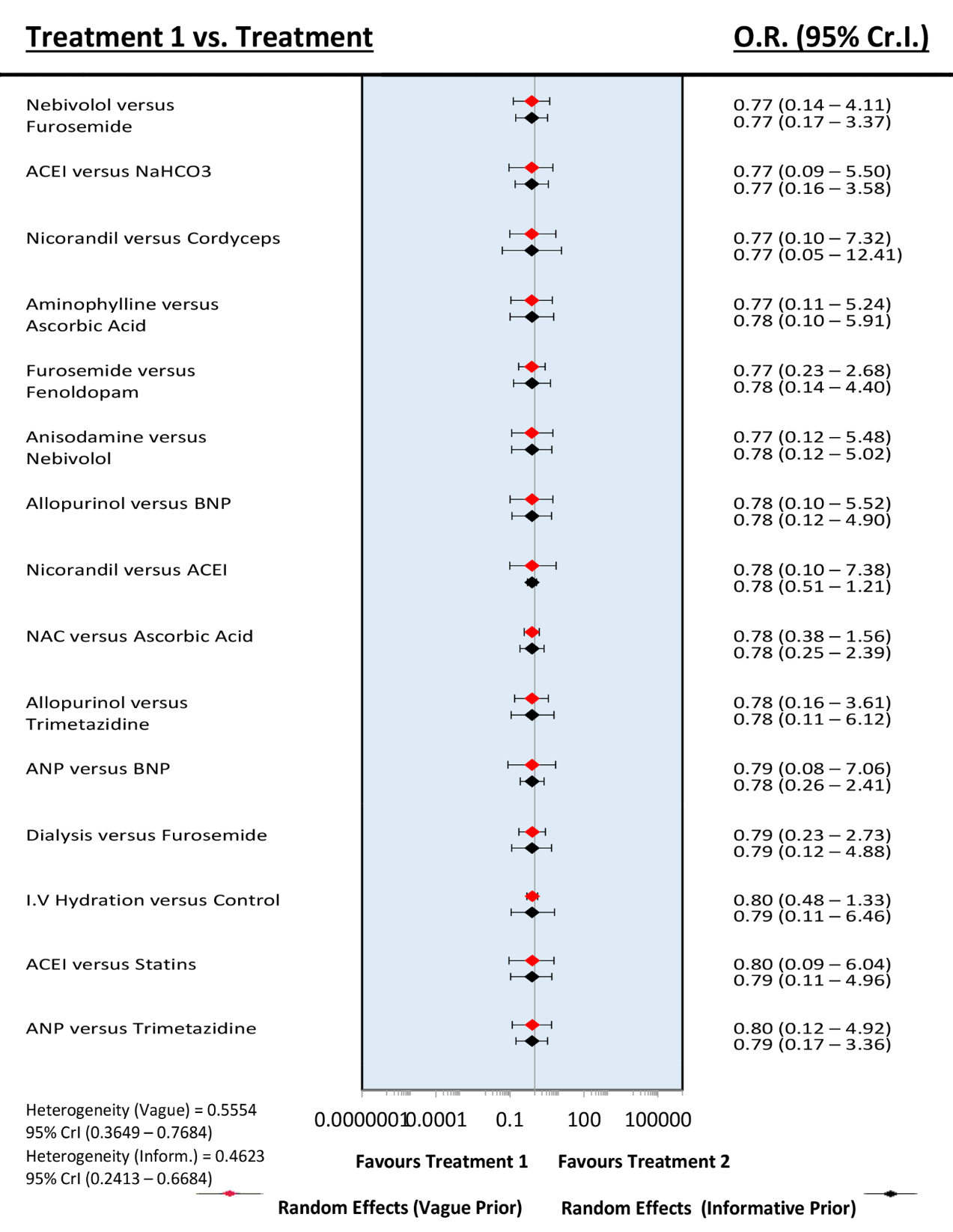


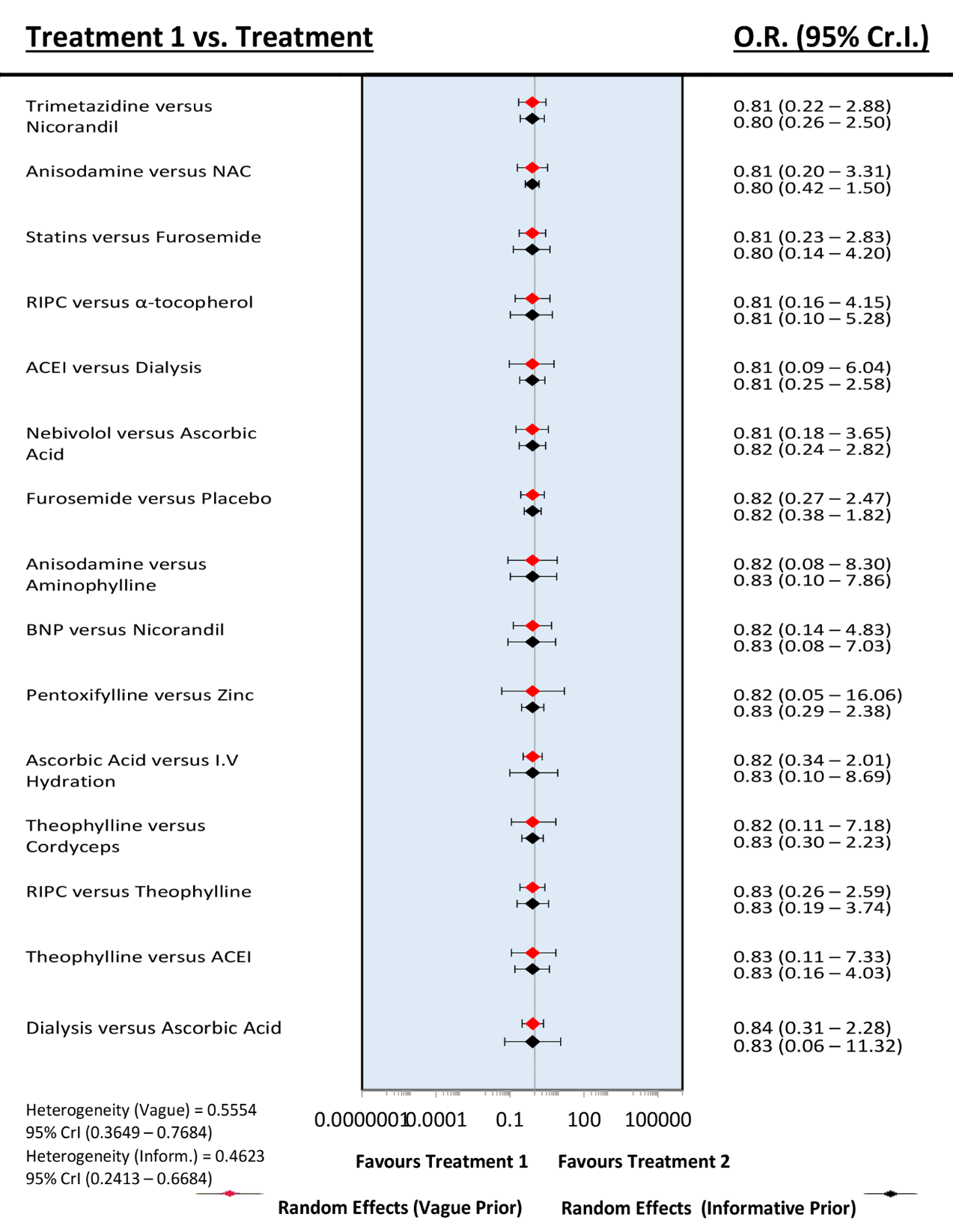


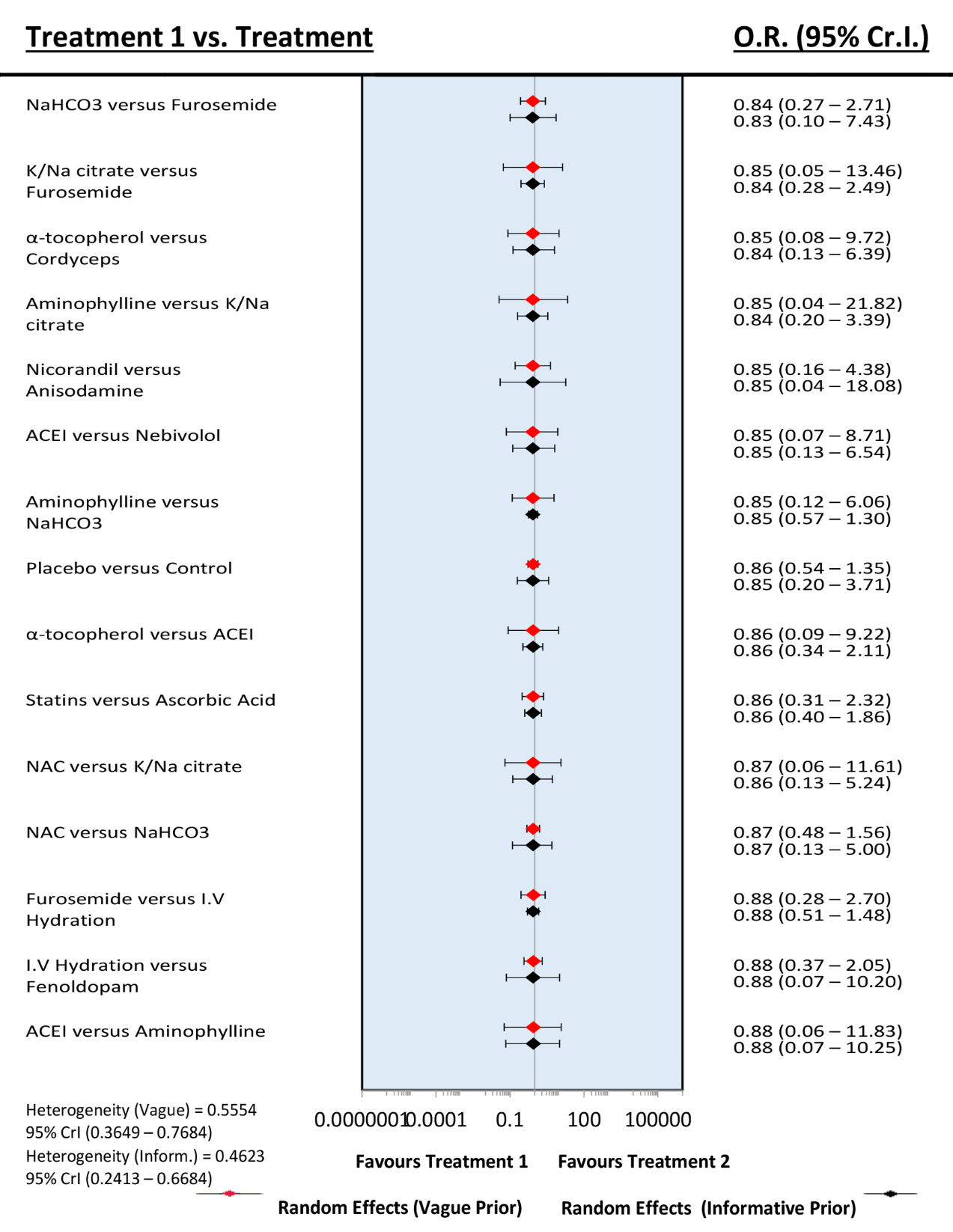


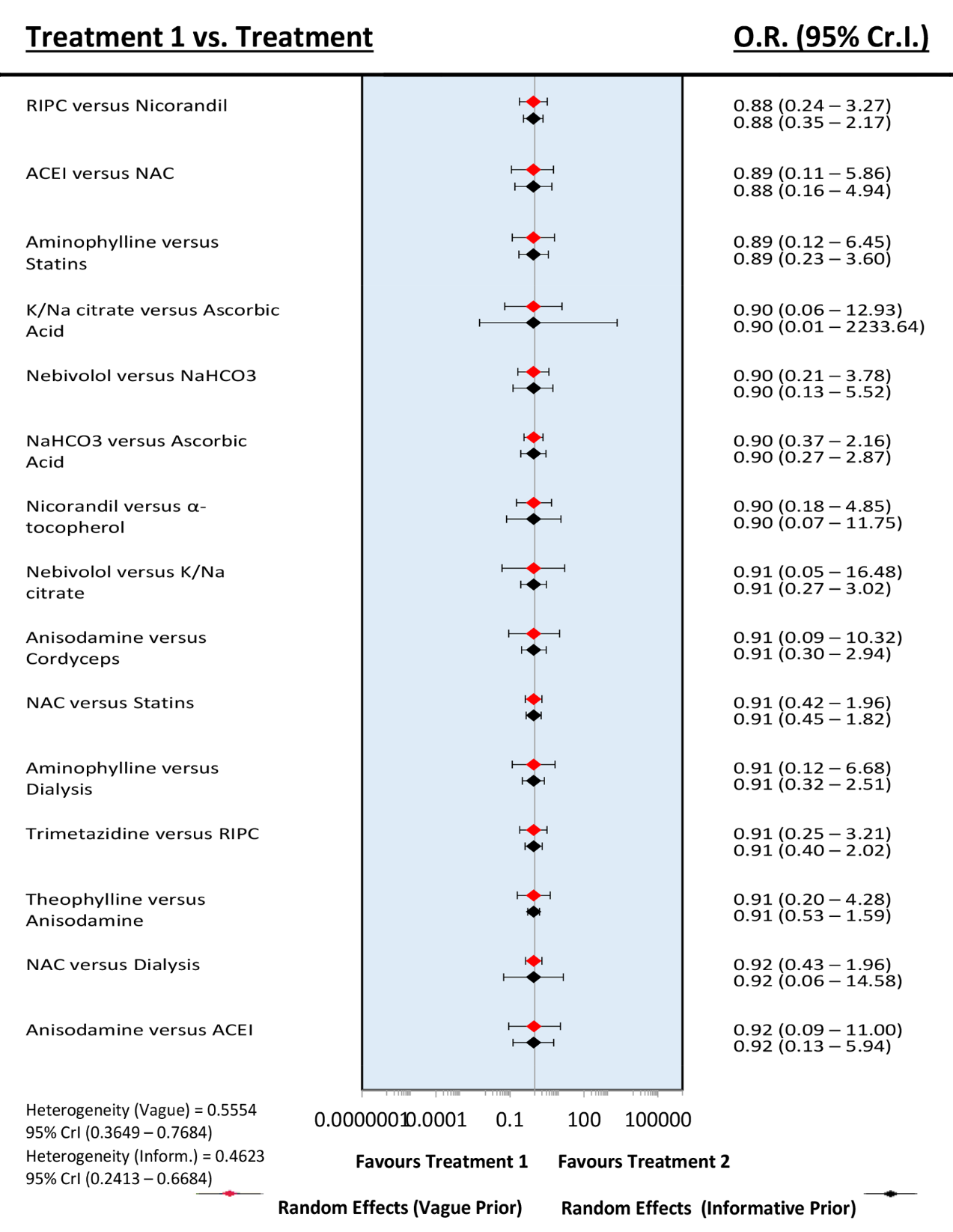


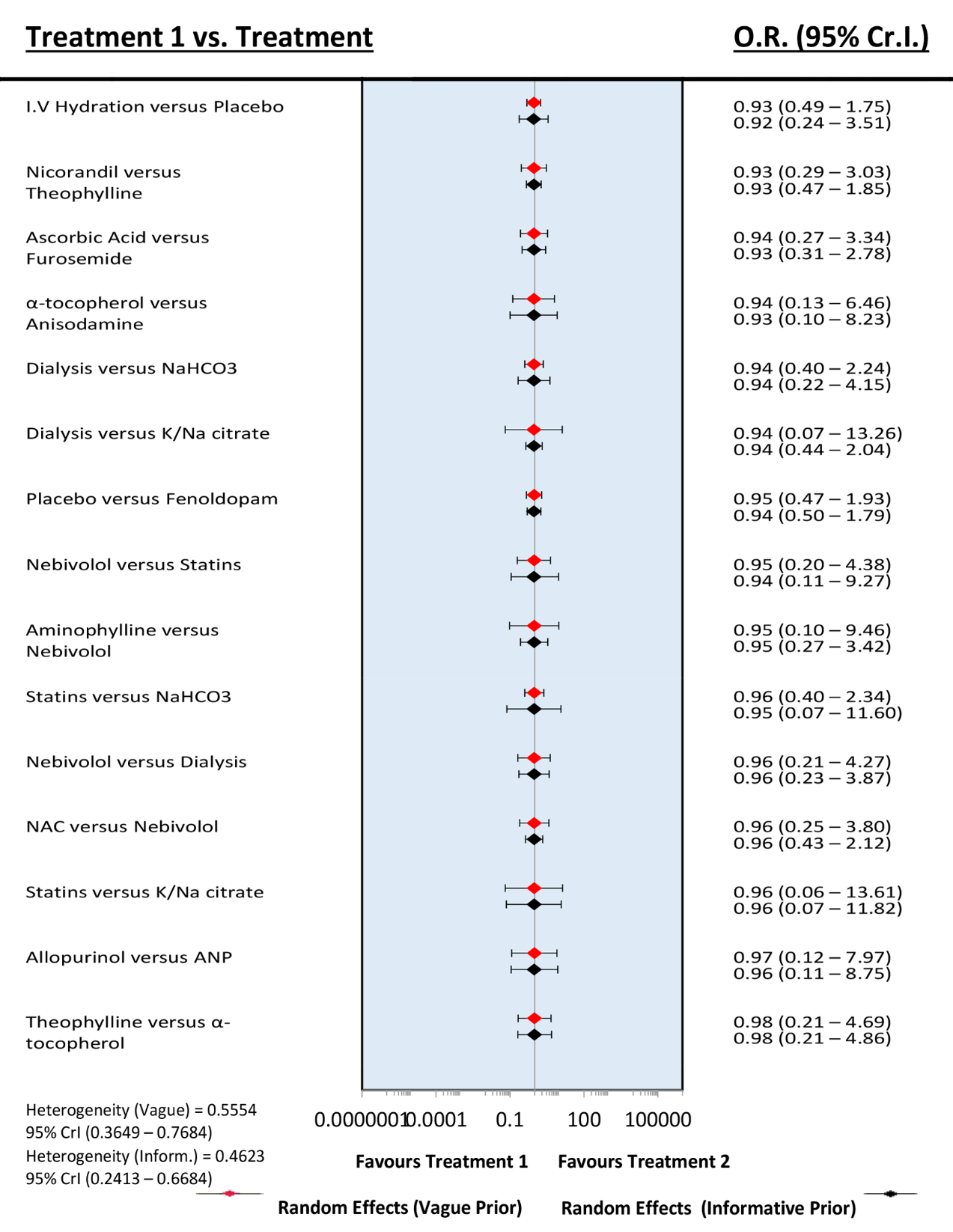


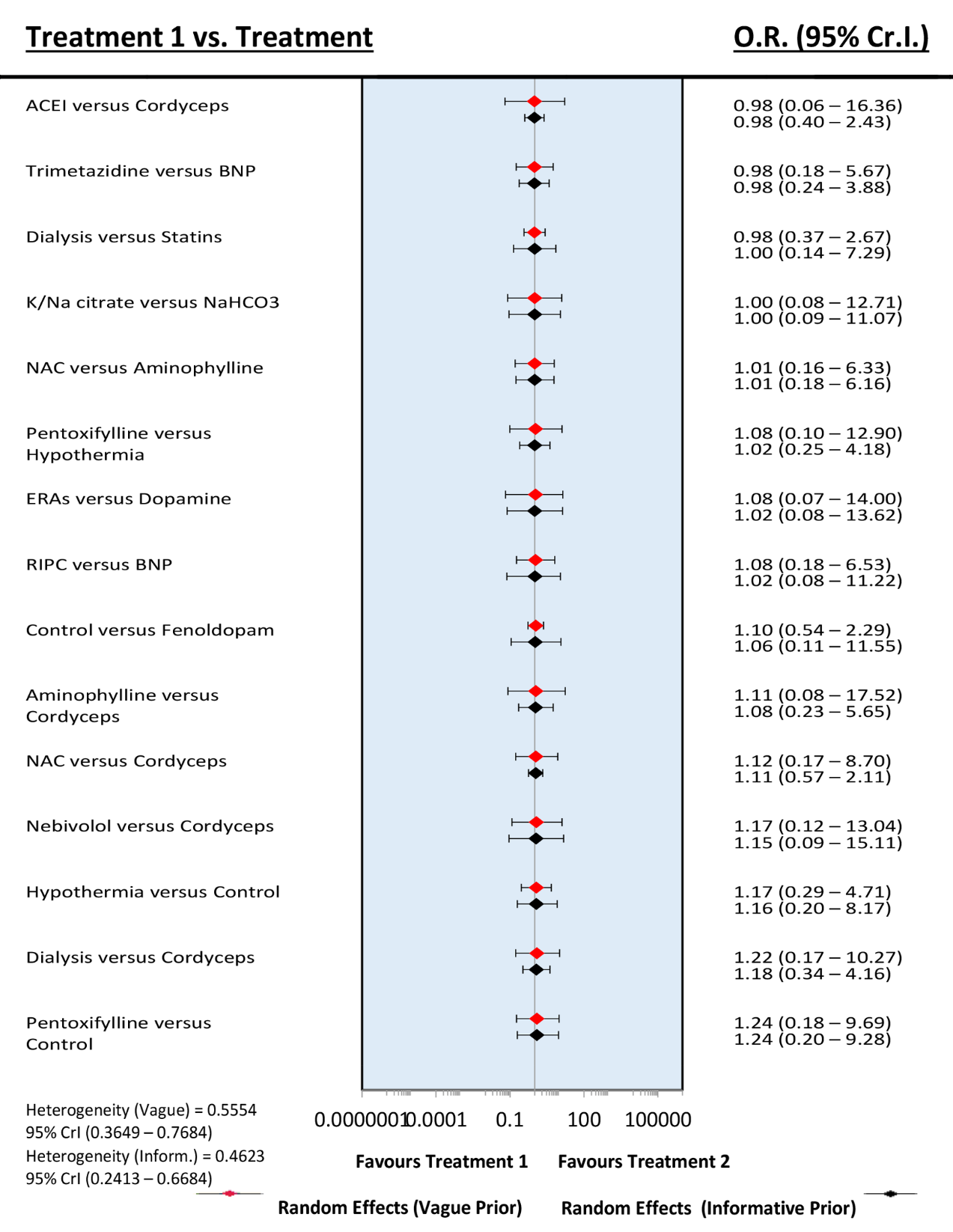


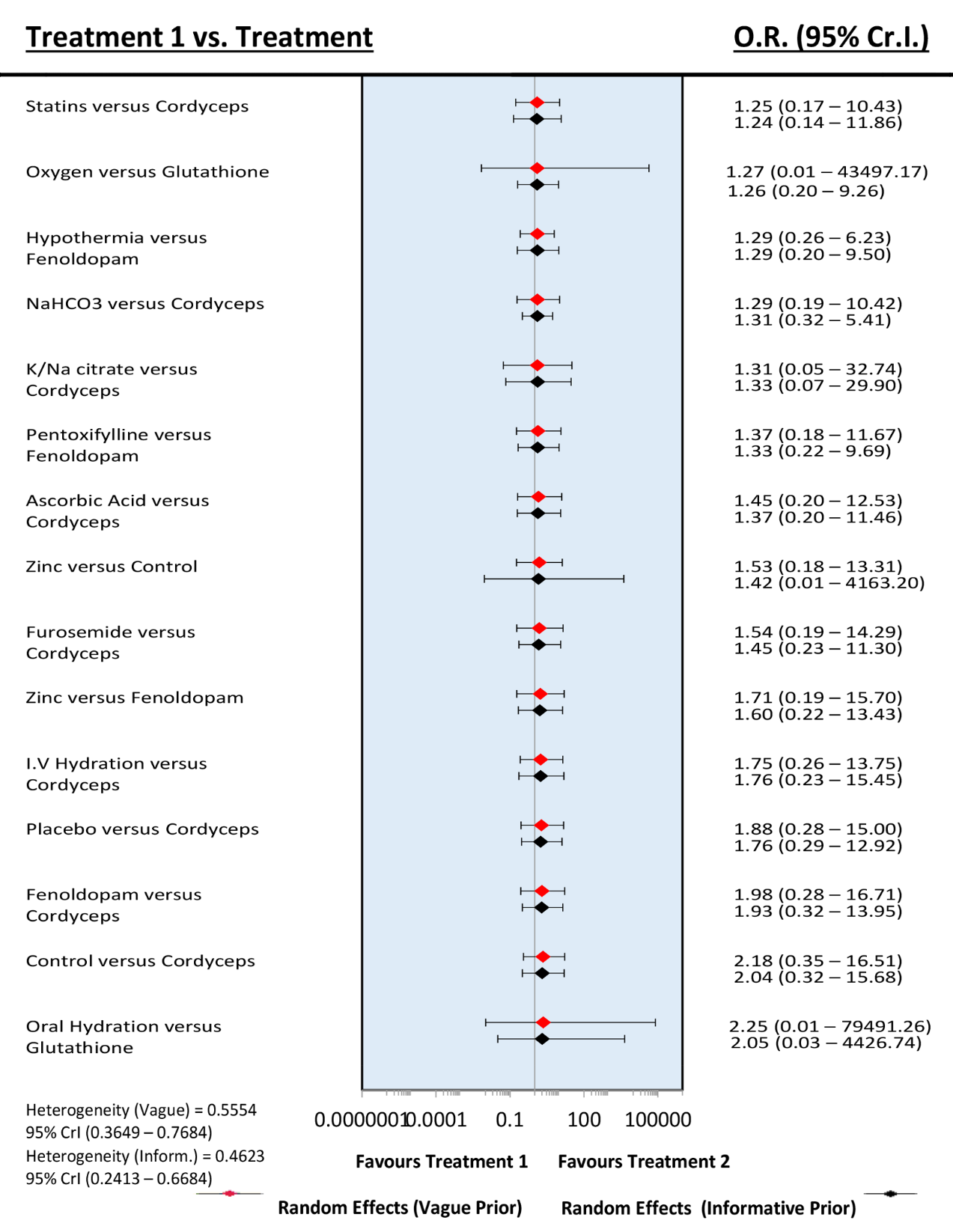


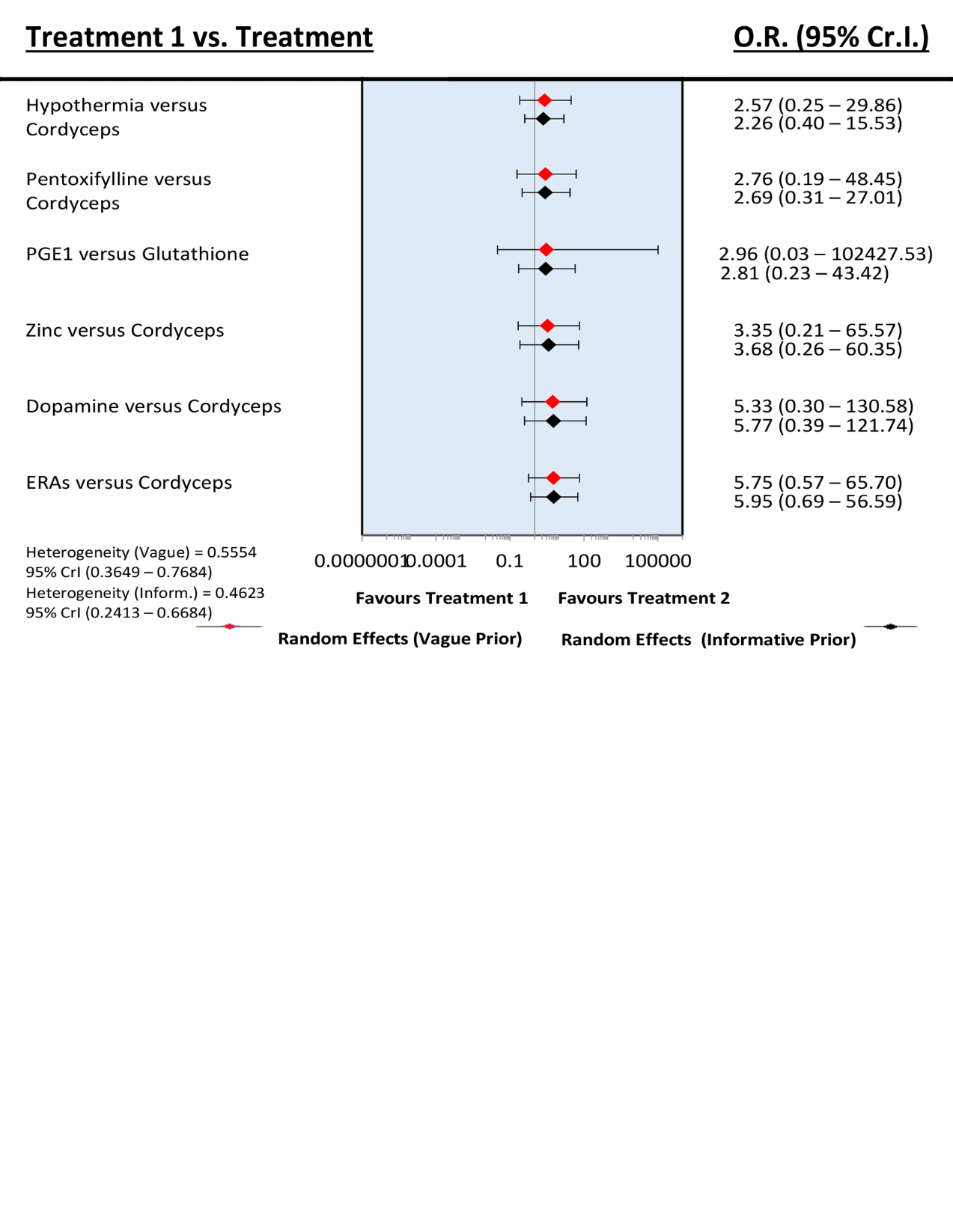


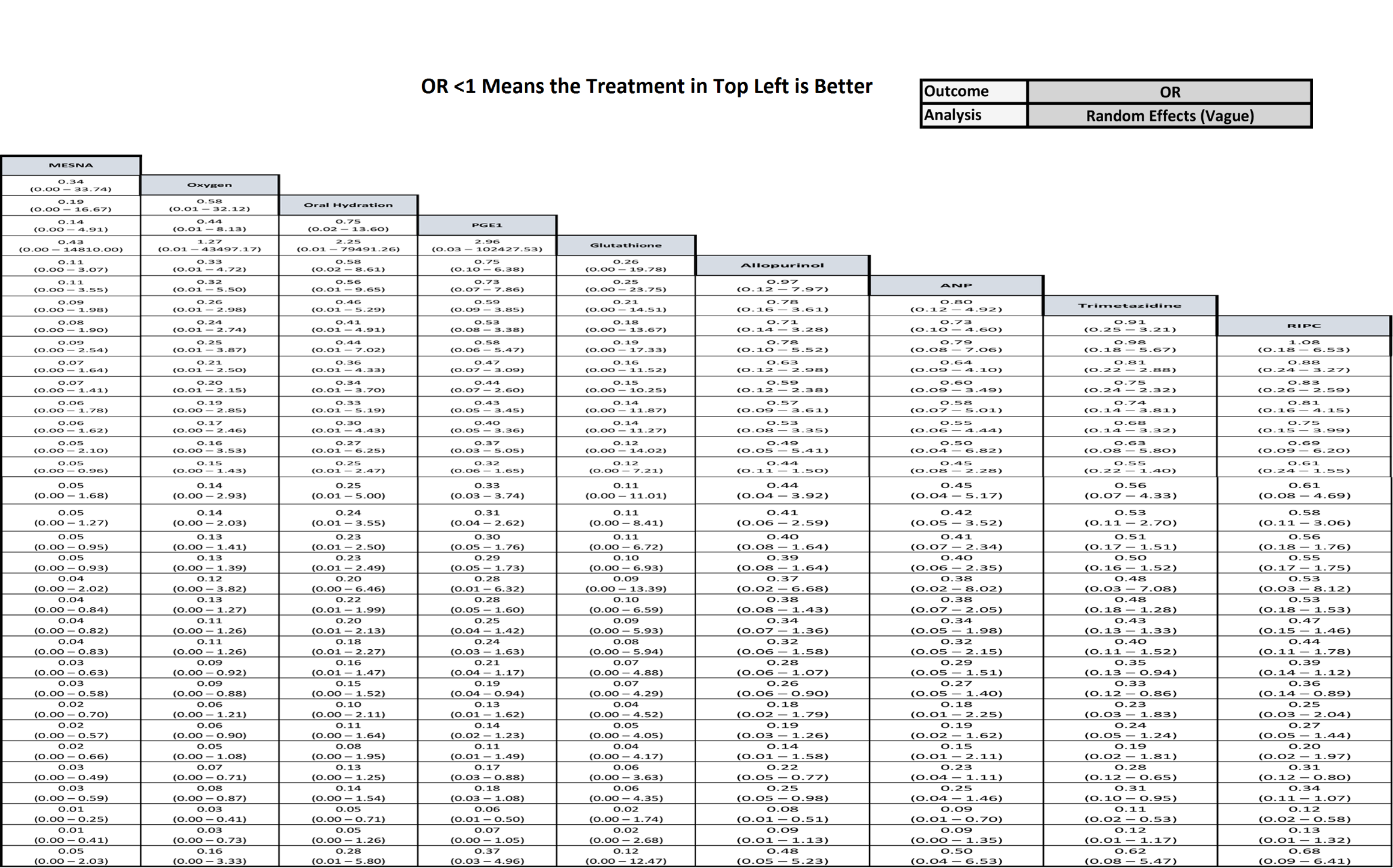


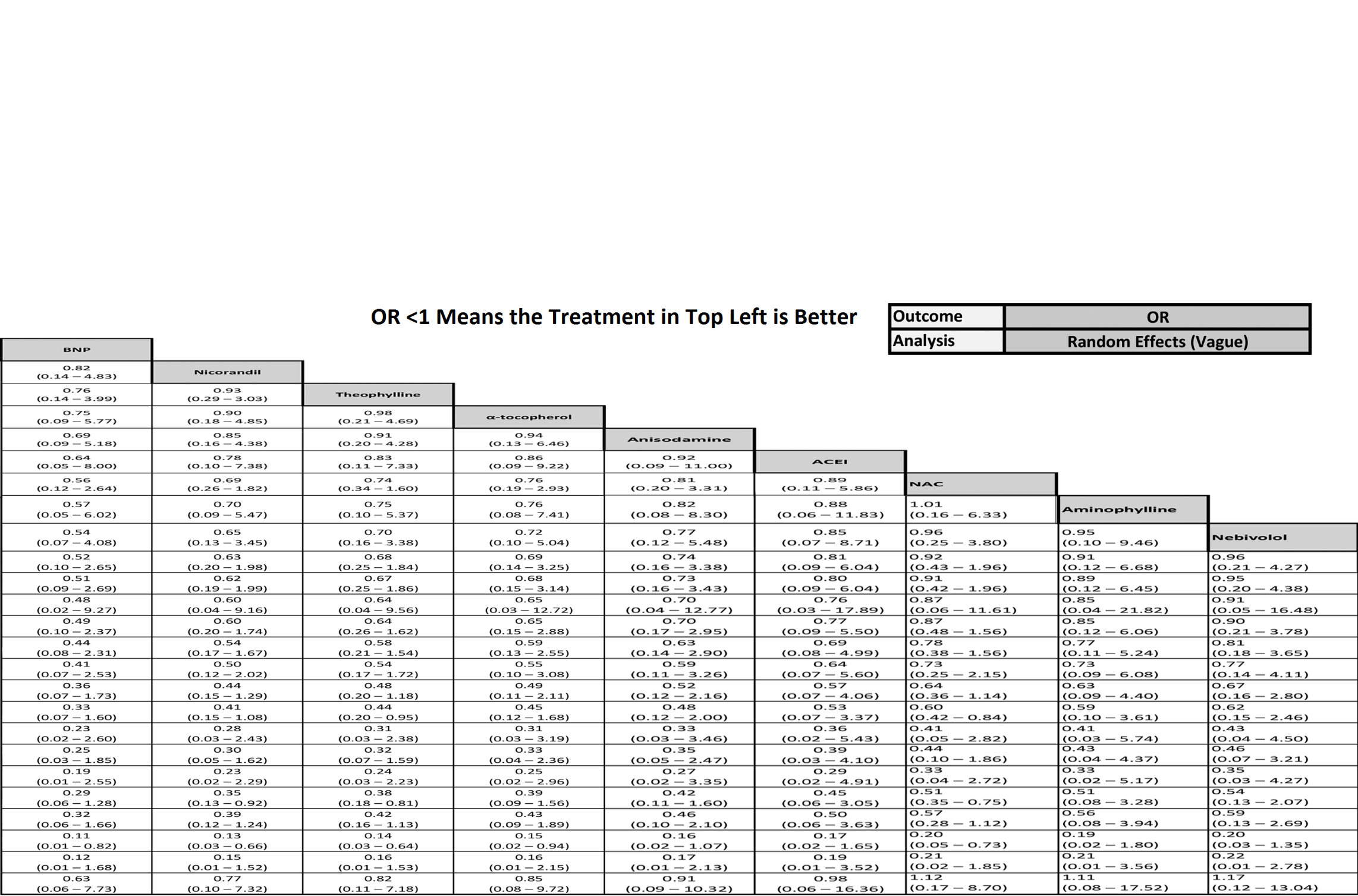


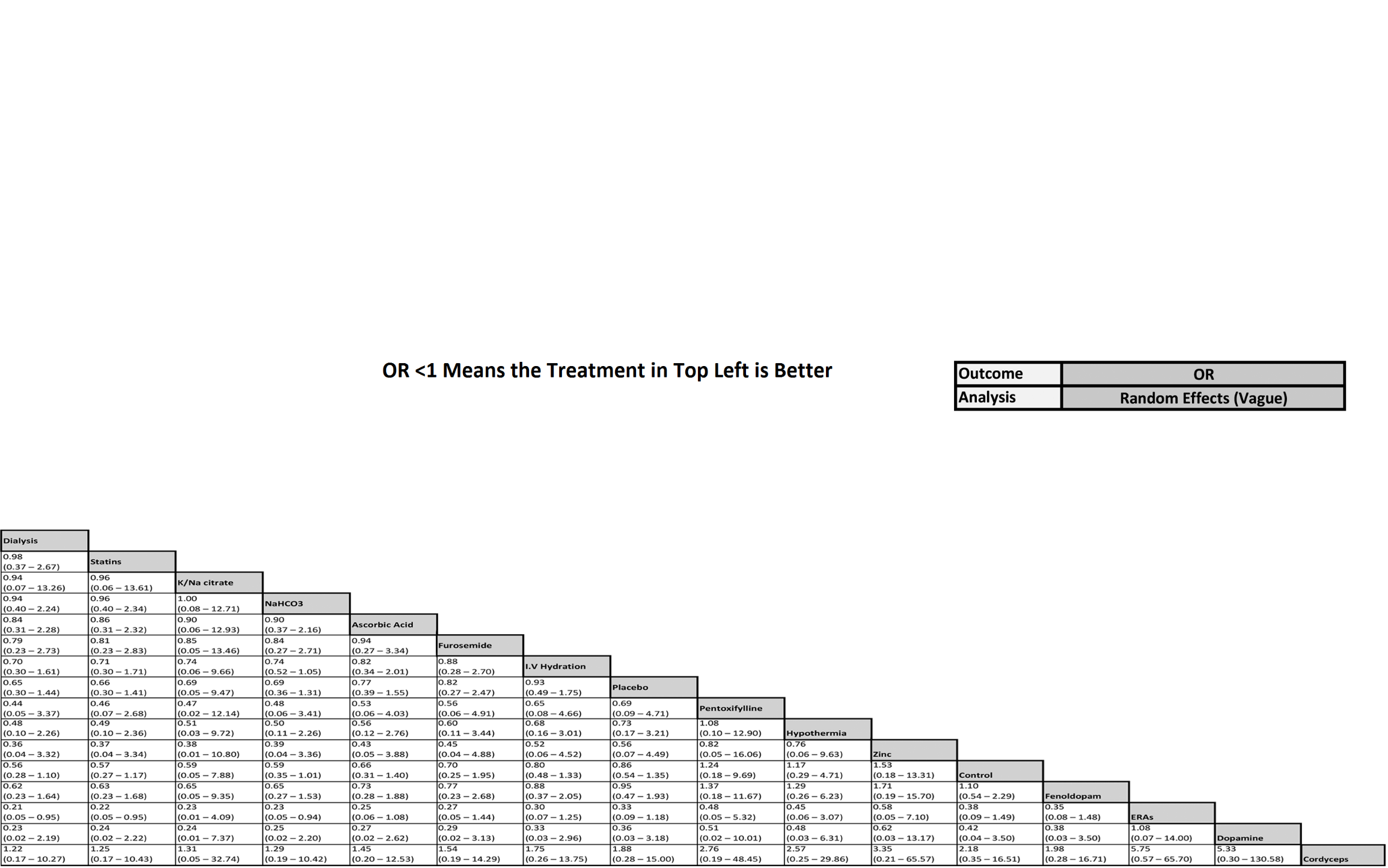


**Sub-Analysis after excluding studies with zero values**

Total Number of studies: 105 RCTs

Excluded studies with zero events (total 7)

| Study | Interventions included |
| --- | --- |
| Bilasy, 2012 | Placebo, Theophylline |
| Efrati, 2003 | Placebo, NAC |
| Erol, 2013 | Control, Allopurinol |
| Kitzler, 2012 | Placebo, NAC, alpha tocopherol |
| Ludwig, 2011 | Control, MESNA |
| Matejka, 2010 | Control, Theophylline |
| Saitoh, 2011 | Control, NAC, Glutathione |

Figures and Tables:

1. Network Diagram

2. Tables:

A. Network Characteristics

B. Interventions Characteristics

C Direct comparisons characteristics

3. Rankogram

4. Ranking and probability of being the best (table)

5. Forest Plot

6. R Graphical diagnostics (Trace & Density)

| Software | Spec | Convergence | Analysis |
| --- | --- | --- | --- |
| Netmetaxl / WinBUGS14 version 1.4.3 | Burn 5000  Sim 10000 | good convergence (FE MC error 5% of the SD) | Random Effects (Vague)  Random Effects (Informative) |
| GeMTC R | Burn 5000  Sim 20000 | good convergence | Random Effects (Vague) |


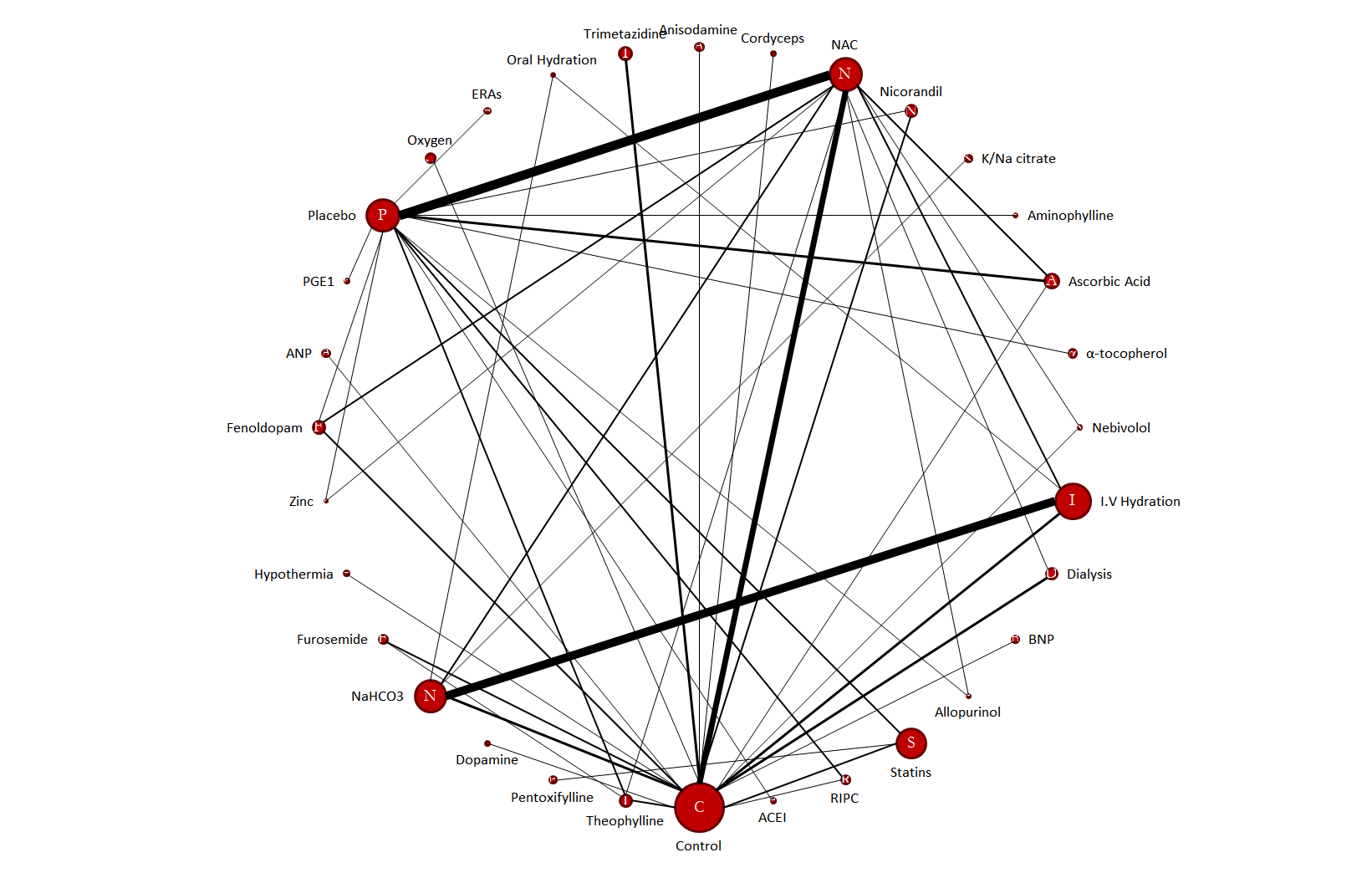


Figure 1 Network Diagram

Table 1 Network Characteristics

| **Characteristic** | **Number** |
| --- | --- |
| **Number of Interventions** | 32 |
| **Number of Studies** | 105 |
| **Total Number of Patients in Network** | 20,947 |
| **Total Number of Events in Network** | 2,116 |
| **Total Possible Pairwise Comparisons** | 496 |
| **Total Number Pairwise Comparisons With Direct Data** | 49 |
| **Number of Two-arm Studies** | 94 |
| **Number of Multi-Arms Studies** | 11 |
| **Number of Studies With No Zero Events** | 105 |
| **Number of Studies With At Least One Zero Event** | 0 |
| **Number of Studies with All Zero Events** | 0 |

Table 2 Interventions Characteristics

| **Treatment** | **# Studies** | **# Events** | **# Patients** | **Aggregate Rate** |
| --- | --- | --- | --- | --- |
| **I.V Hydration** | 22 | 265 | 2835 | 0.0935 |
| **Statins** | 6 | 58 | 1961 | 0.0296 |
| **Furosemide** | 2 | 16 | 166 | 0.0964 |
| **NAC** | 37 | 254 | 2397 | 0.1060 |
| **Trimetazidine** | 4 | 17 | 352 | 0.0483 |
| **NaHCO3** | 23 | 173 | 2239 | 0.0773 |
| **PGE1** | 1 | 6 | 33 | 0.1818 |
| **Pentoxifylline** | 1 | 6 | 110 | 0.0545 |
| **Placebo** | 35 | 400 | 2384 | 0.1678 |
| **Control** | 46 | 586 | 5444 | 0.1076 |
| **Allopurinol** | 1 | 5 | 30 | 0.1667 |
| **BNP** | 1 | 6 | 106 | 0.0566 |
| **α-tocopherol** | 1 | 10 | 149 | 0.0671 |
| **Oxygen** | 1 | 1 | 174 | 0.0057 |
| **K/Na citrate** | 1 | 2 | 103 | 0.0194 |
| **Nicorandil** | 3 | 15 | 291 | 0.0515 |
| **Ascorbic Acid** | 6 | 41 | 434 | 0.0945 |
| **Oral Hydration** | 1 | 1 | 22 | 0.0455 |
| **Nebivolol** | 1 | 8 | 40 | 0.2000 |
| **Anisodamine** | 1 | 13 | 132 | 0.0985 |
| **RIPC** | 4 | 16 | 163 | 0.0982 |
| **Theophylline** | 5 | 18 | 323 | 0.0557 |
| **Hypothermia** | 1 | 14 | 58 | 0.2414 |
| **ACEI** | 1 | 3 | 52 | 0.0577 |
| **Aminophylline** | 1 | 4 | 30 | 0.1333 |
| **ANP** | 1 | 4 | 126 | 0.0317 |
| **Zinc** | 1 | 3 | 18 | 0.1667 |
| **Dialysis** | 5 | 43 | 293 | 0.1468 |
| **Fenoldopam** | 5 | 78 | 333 | 0.2342 |
| **ERAs** | 1 | 43 | 77 | 0.5584 |
| **Dopamine** | 1 | 4 | 33 | 0.1212 |
| **Cordyceps** | 1 | 3 | 39 | 0.0769 |

Table 3 Direct comparisons characteristics

| **Comparison** | **# Studies** | **# Patients** | **# Events** |
| --- | --- | --- | --- |
| **Statins vs. Control** | 3 | 3,338 | 116 |
| **NaHCO3 vs. K/Na citrate** | 1 | 206 | 4 |
| **I.V Hydartion vs. NaHCO3** | 17 | 3,699 | 323 |
| **NAC vs. Placebo** | 19 | 2,553 | 371 |
| **NAC vs. Ascorbic Acid** | 3 | 583 | 88 |
| **Placebo vs. Ascorbic Acid** | 4 | 638 | 105 |
| **NAC vs. Control** | 12 | 1,561 | 168 |
| **NAC vs. Fenoldopam** | 3 | 359 | 44 |
| **Control vs. Fenoldopam** | 2 | 123 | 26 |
| **I.V Hydartion vs. Control** | 5 | 1,960 | 196 |
| **NAC vs. Theophylline** | 1 | 62 | 13 |
| **I.V Hydartion vs. Oral Hydration** | 1 | 49 | 7 |
| **NaHCO3 vs. Oral Hydration** | 1 | 43 | 3 |
| **Furosemide vs. Control** | 2 | 326 | 35 |
| **Furosemide vs. Theophylline** | 1 | 159 | 18 |
| **Control vs. Theophylline** | 2 | 437 | 24 |
| **Control vs. RIPC** | 1 | 100 | 26 |
| **Placebo vs. Theophylline** | 2 | 164 | 15 |
| **Placebo vs. Nicorandil** | 1 | 240 | 29 |
| **Control vs. Dopamine** | 1 | 66 | 6 |
| **Control vs. Anisodamine** | 1 | 260 | 39 |
| **NAC vs. Nebivolol** | 1 | 80 | 17 |
| **Control vs. Nebivolol** | 1 | 80 | 19 |
| **NAC vs. NaHCO3** | 2 | 152 | 32 |
| **Statins vs. Placebo** | 2 | 366 | 25 |
| **Control vs. Cordyceps** | 1 | 80 | 9 |
| **I.V Hydartion vs. NAC** | 2 | 231 | 27 |
| **Statins vs. Pentoxifylline** | 1 | 220 | 9 |
| **NAC vs. Zinc** | 1 | 37 | 4 |
| **Placebo vs. Zinc** | 1 | 35 | 5 |
| **Control vs. Nicorandil** | 2 | 341 | 22 |
| **PGE1 vs. Placebo** | 1 | 62 | 21 |
| **NaHCO3 vs. Control** | 4 | 506 | 36 |
| **Control vs. Dialysis** | 5 | 588 | 112 |
| **Placebo vs. ACEI** | 1 | 114 | 9 |
| **Control vs. BNP** | 1 | 209 | 23 |
| **Trimetazidine vs. Control** | 4 | 714 | 71 |
| **Placebo vs. RIPC** | 3 | 225 | 27 |
| **Control vs. ANP** | 1 | 254 | 19 |
| **NAC vs. Dialysis** | 1 | 275 | 11 |
| **Placebo vs. α-tocopherol** | 1 | 298 | 31 |
| **Placebo vs. Aminophylline** | 1 | 60 | 10 |
| **NAC vs. Allopurinol** | 1 | 65 | 12 |
| **Placebo vs. Allopurinol** | 1 | 60 | 16 |
| **Control vs. Oxygen** | 1 | 349 | 10 |
| **Placebo vs. Fenoldopam** | 1 | 283 | 90 |
| **Control vs. Hypothermia** | 1 | 128 | 29 |
| **Placebo vs. ERAs** | 1 | 158 | 67 |
| **Control vs. Ascorbic Acid** | 1 | 156 | 10 |

Figure 2 Rankogram: ranking the interventions for the probability of being the best, the interventions are colour coded; the first column represent the chance of being first best and 2nd column is the chance of being 2nd best and so on. The overall numerical value is presented in table 4


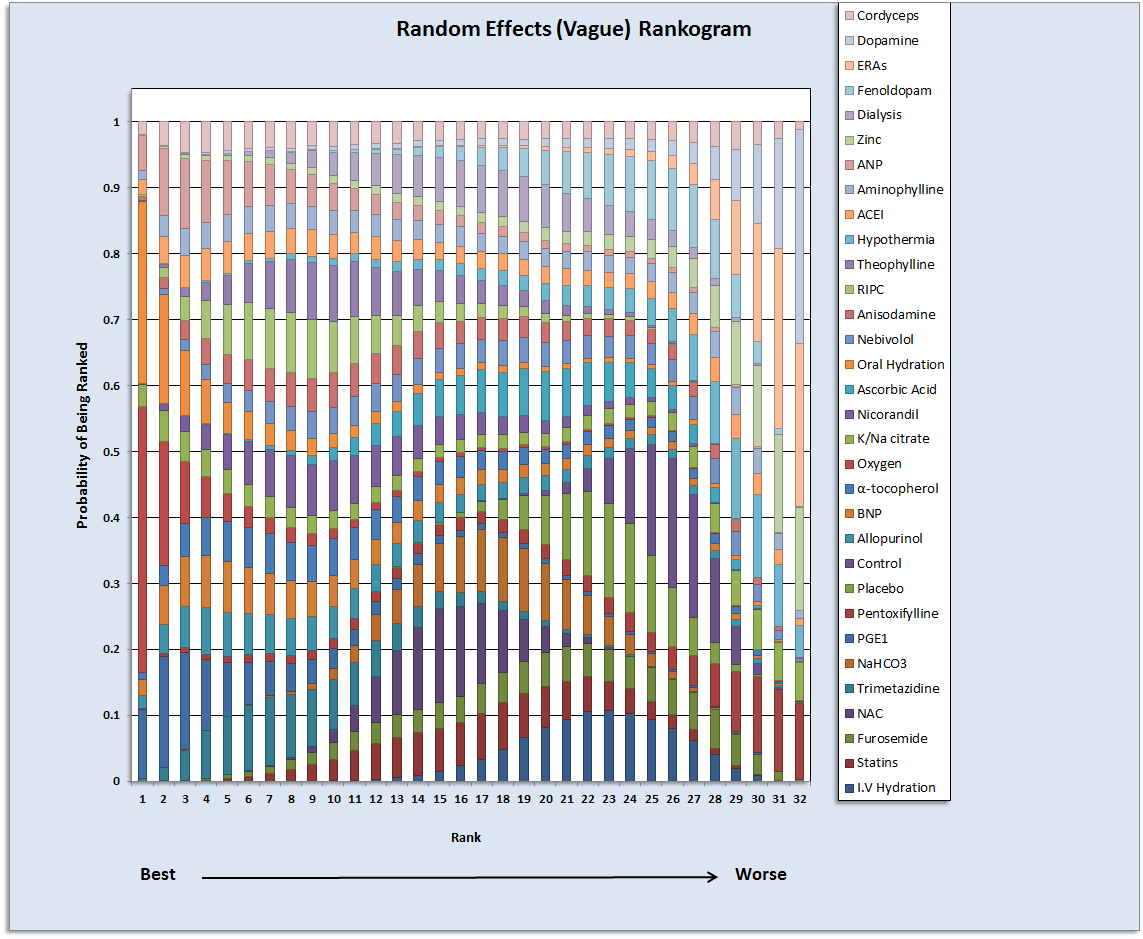


Table 4 Interventions ranking the treatments names column follow the league table (which arranges the presentation of summary estimates by ranking the treatments in order of most pronounced impact on the outcome under consideration) the numerical values represents the cumulative results of the probability of being best in which the highest score is 1 or 100% (see Rankogram)

| **Treatment** | **SUCRA** | **Treatment** | **SUCRA** |
| --- | --- | --- | --- |
| **Oxygen** | 0.9017 | **Statins** | 0.4792 |
| **Oral Hydration** | 0.8323 | **K/Na citrate** | 0.475 |
| **PGE1** | 0.8322 | **Dialysis** | 0.473 |
| **ANP** | 0.7567 | **NaHCO3** | 0.4605 |
| **Trimetazidine** | 0.7423 | **Ascorbic Acid** | 0.4187 |
| **RIPC** | 0.7143 | **Furosemide** | 0.3815 |
| **BNP** | 0.6992 | **I.V Hydration** | 0.3079 |
| **Nicorandil** | 0.665 | **Placebo** | 0.2903 |
| **Allopurinol** | 0.663 | **Fenoldopam** | 0.2728 |
| **Theophylline** | 0.6621 | **Pentoxifylline** | 0.2541 |
| **α-tocopherol** | 0.6409 | **Hypothermia** | 0.2324 |
| **Anisodamine** | 0.5797 | **Zinc** | 0.2158 |
| **ACEI** | 0.5524 | **Control** | 0.2001 |
| **NAC** | 0.5334 | **ERAs** | 0.07549 |
| **Aminophylline** | 0.523 | **Dopamine** | 0.1359 |
| **Nebivolol** | 0.4912 | **Cordyceps** | 0.5379 |
| ***Analysis*** | **Random Effects (Vague)** | | |

Figure 3 Forest Plot


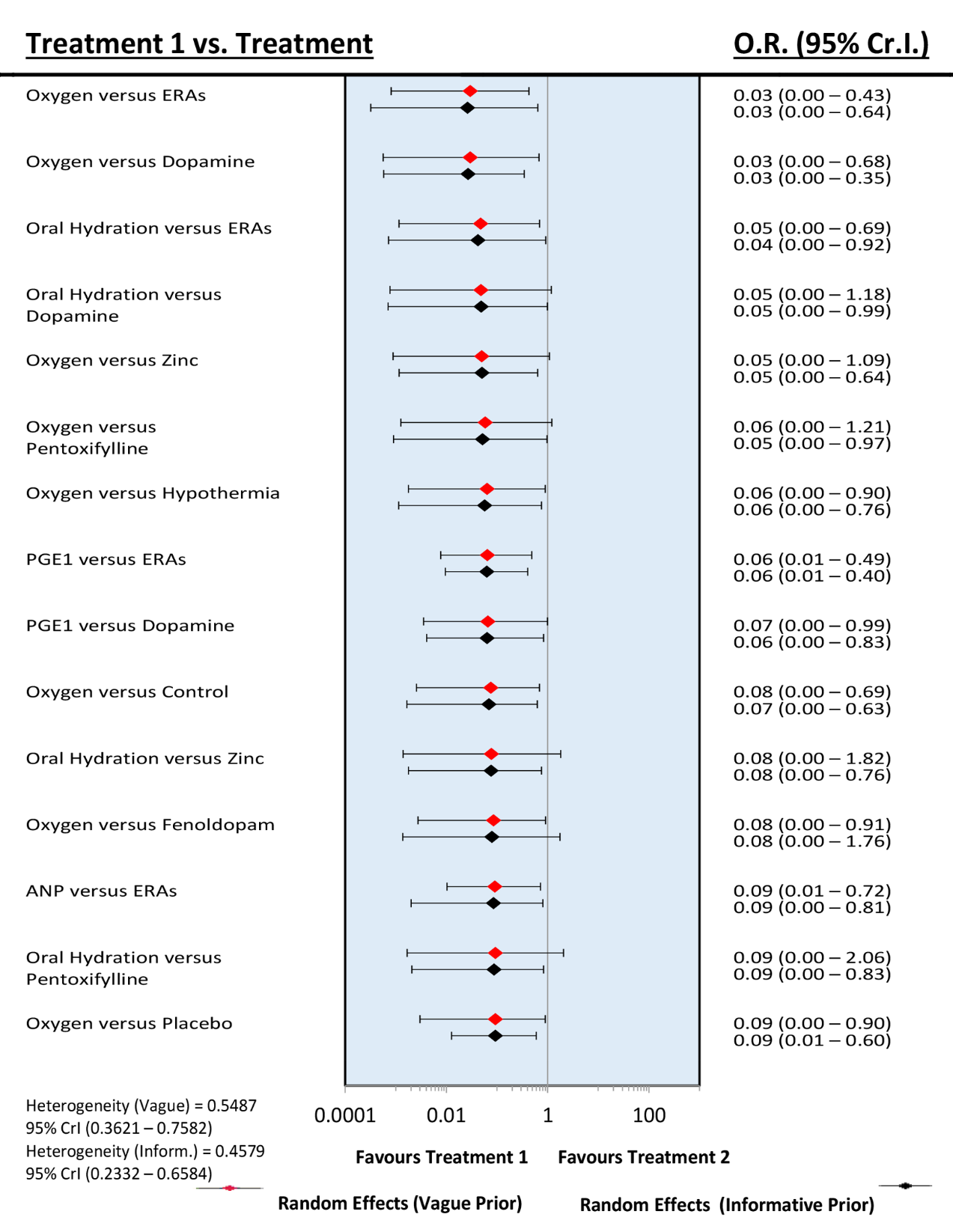


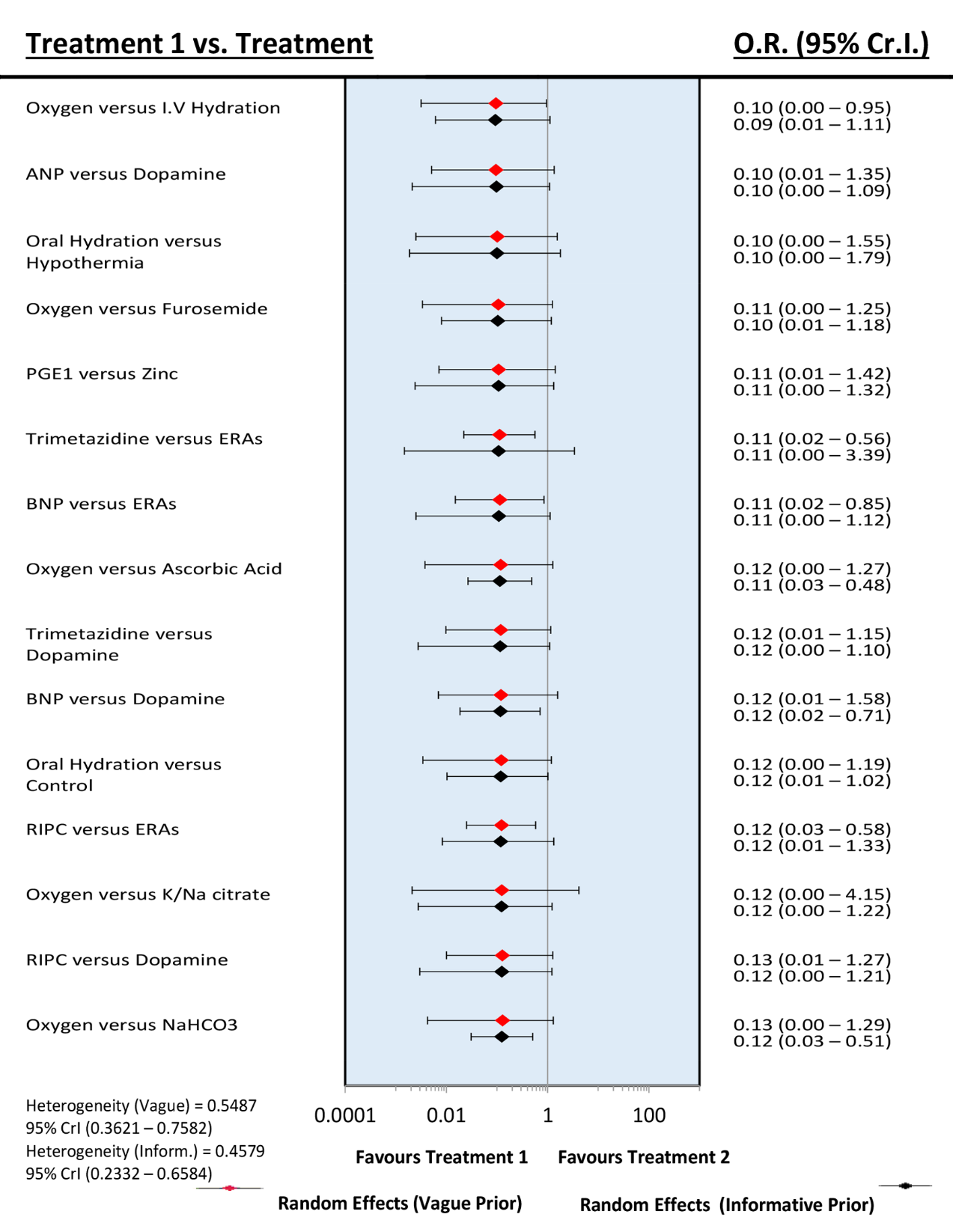


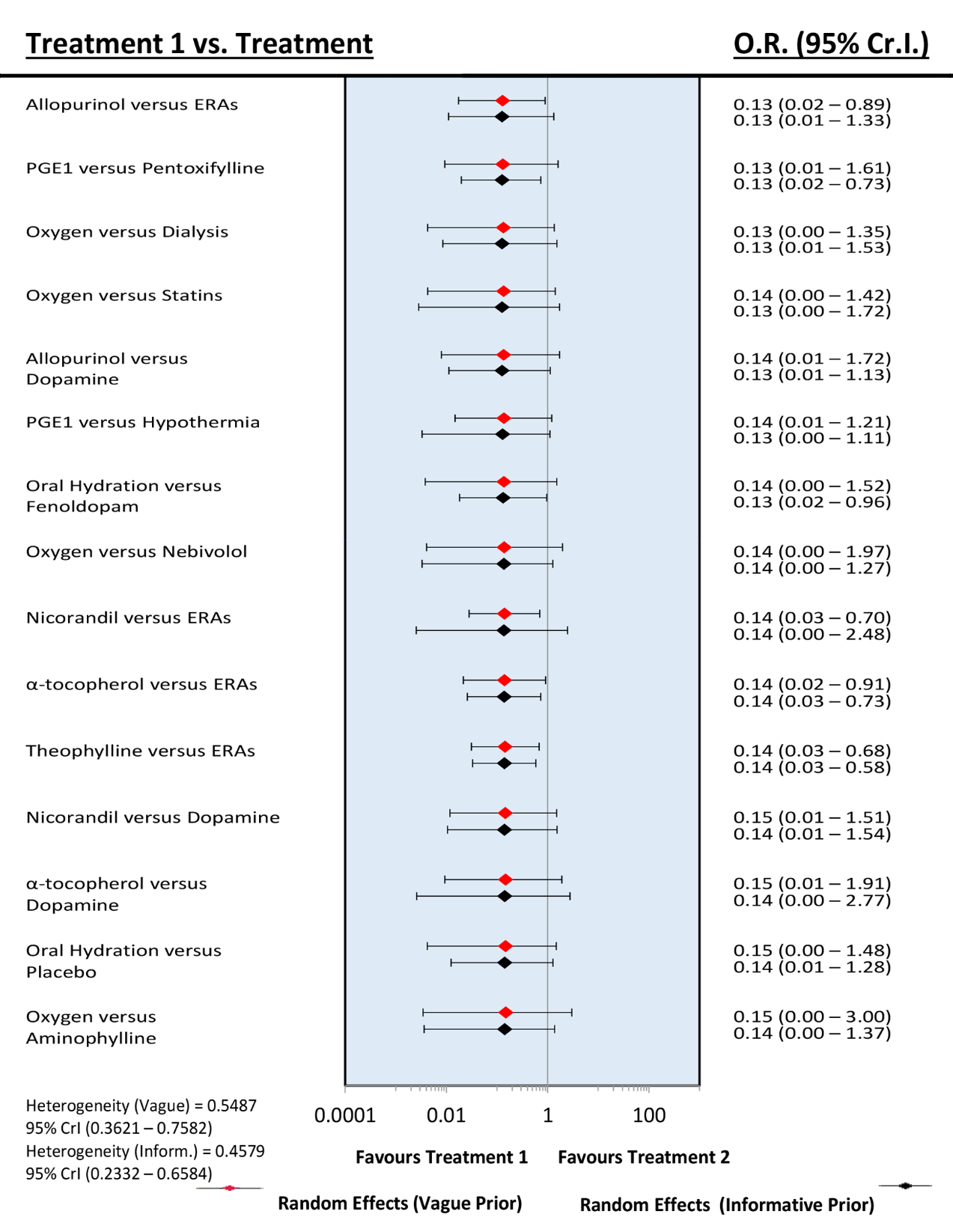


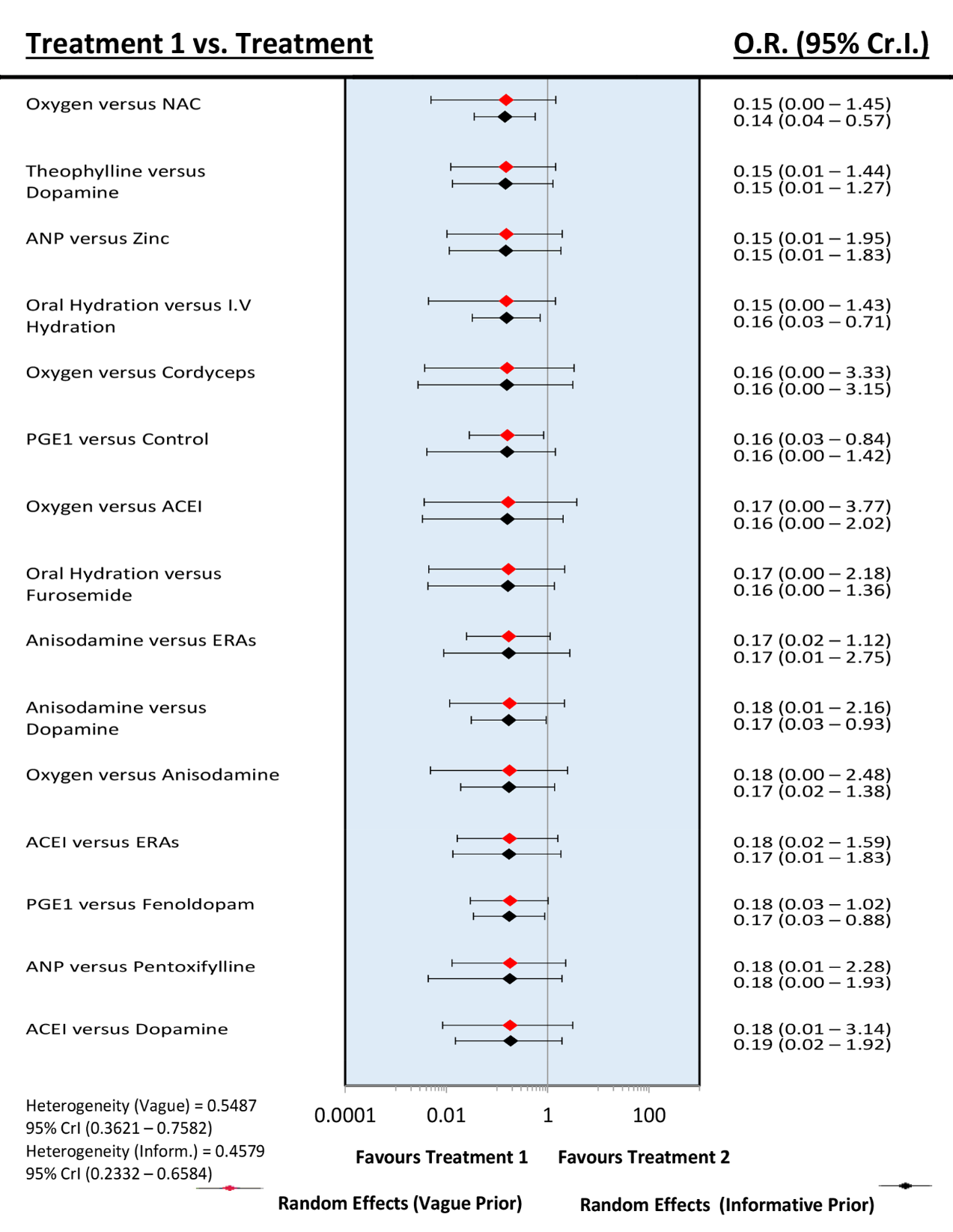


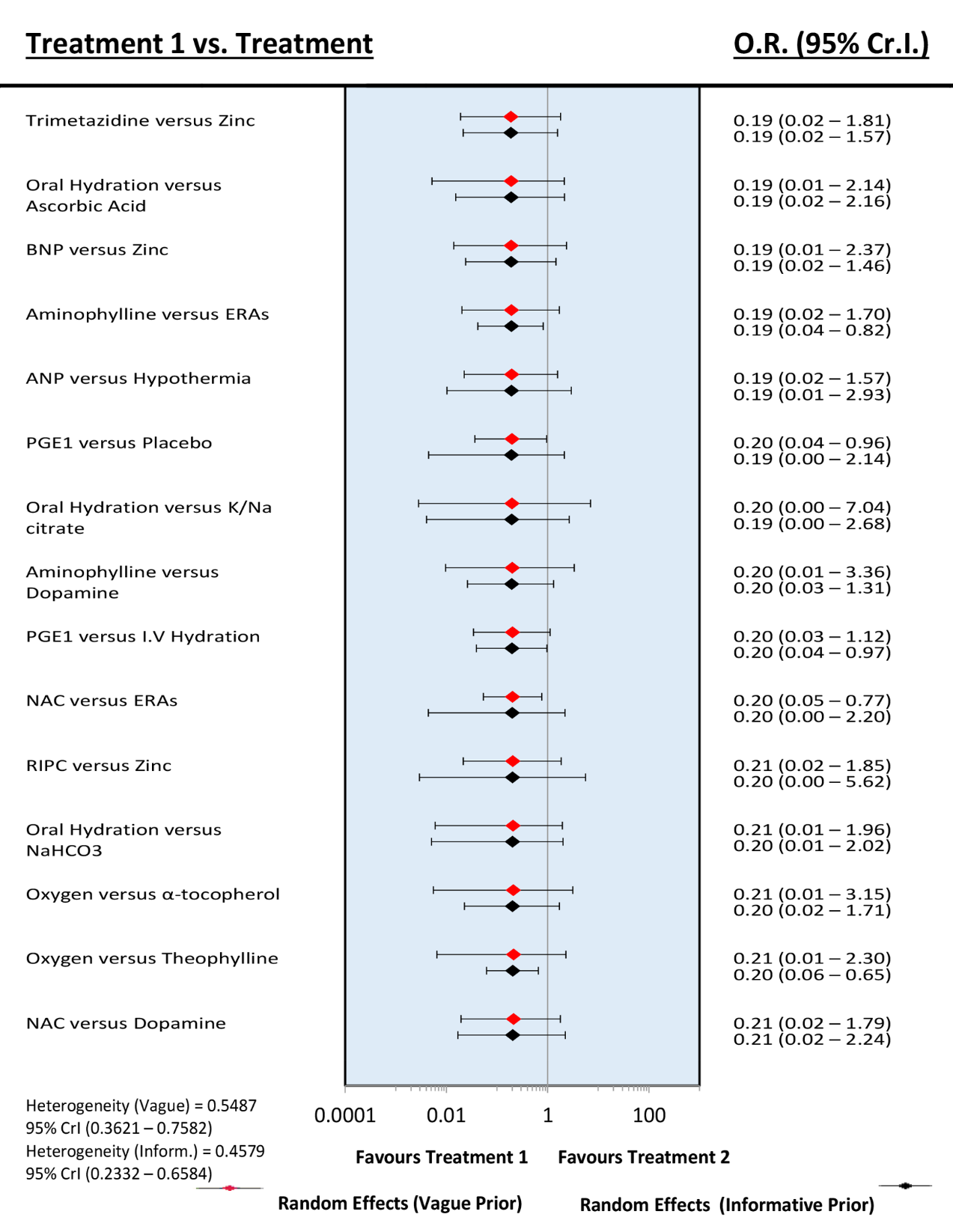


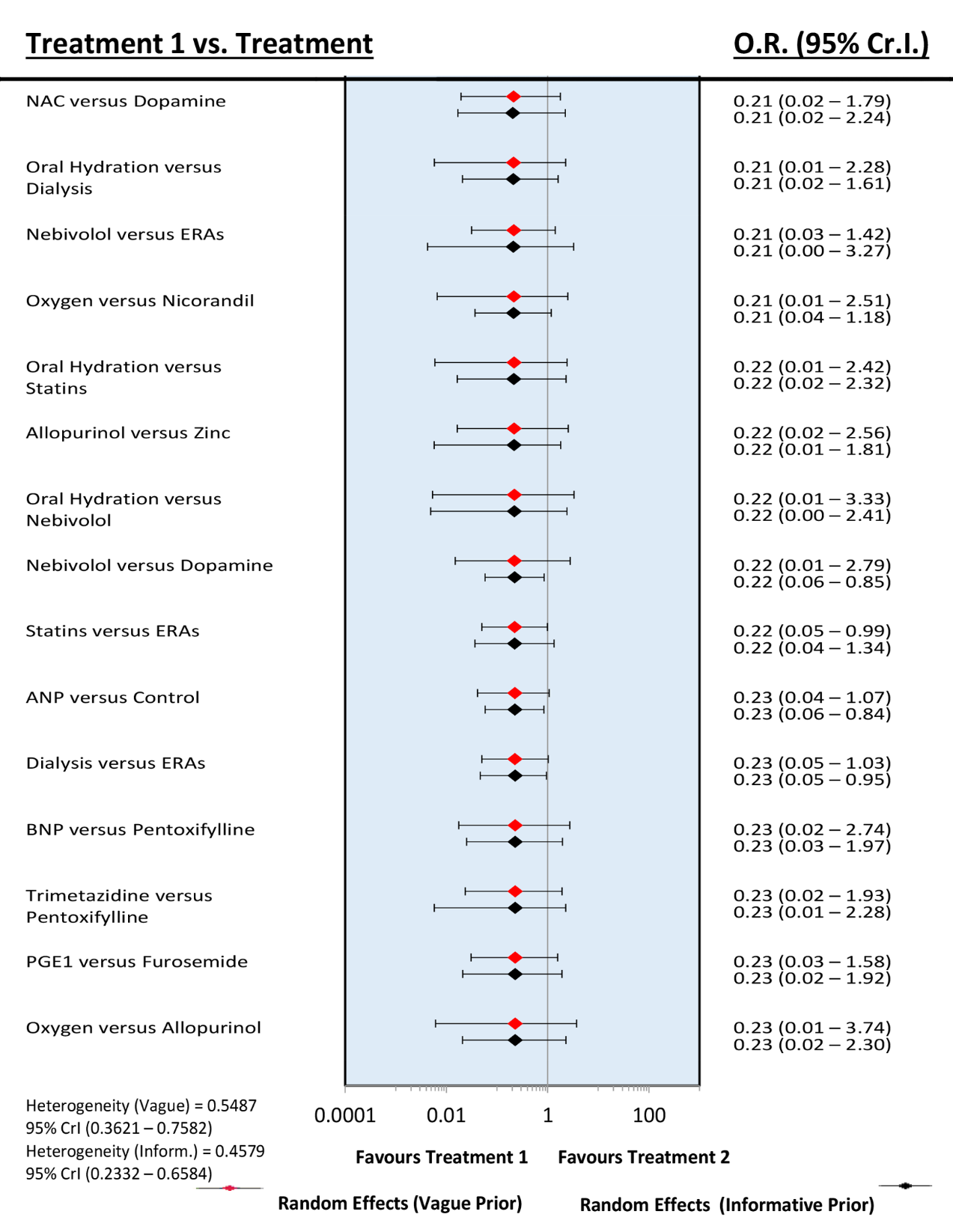


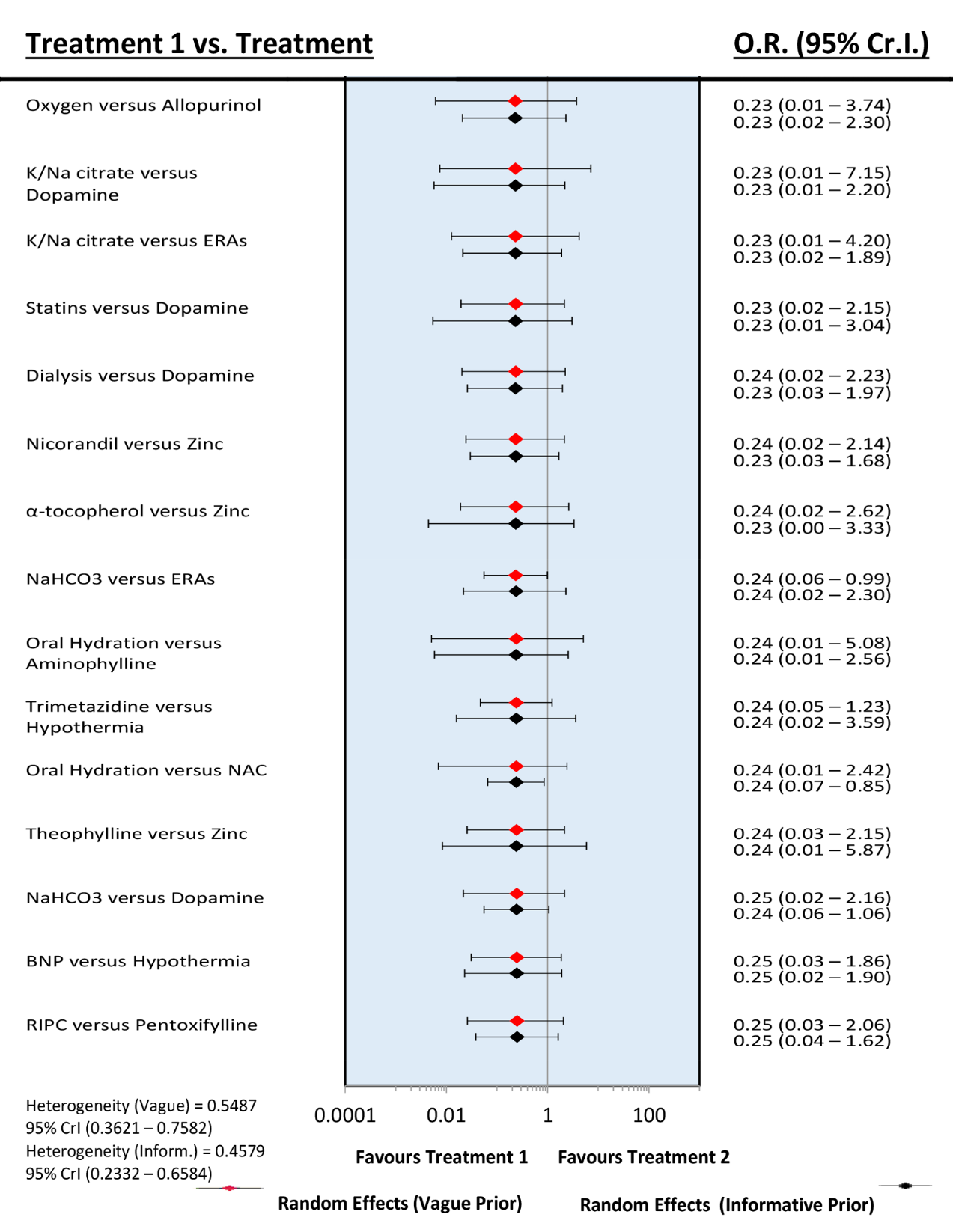


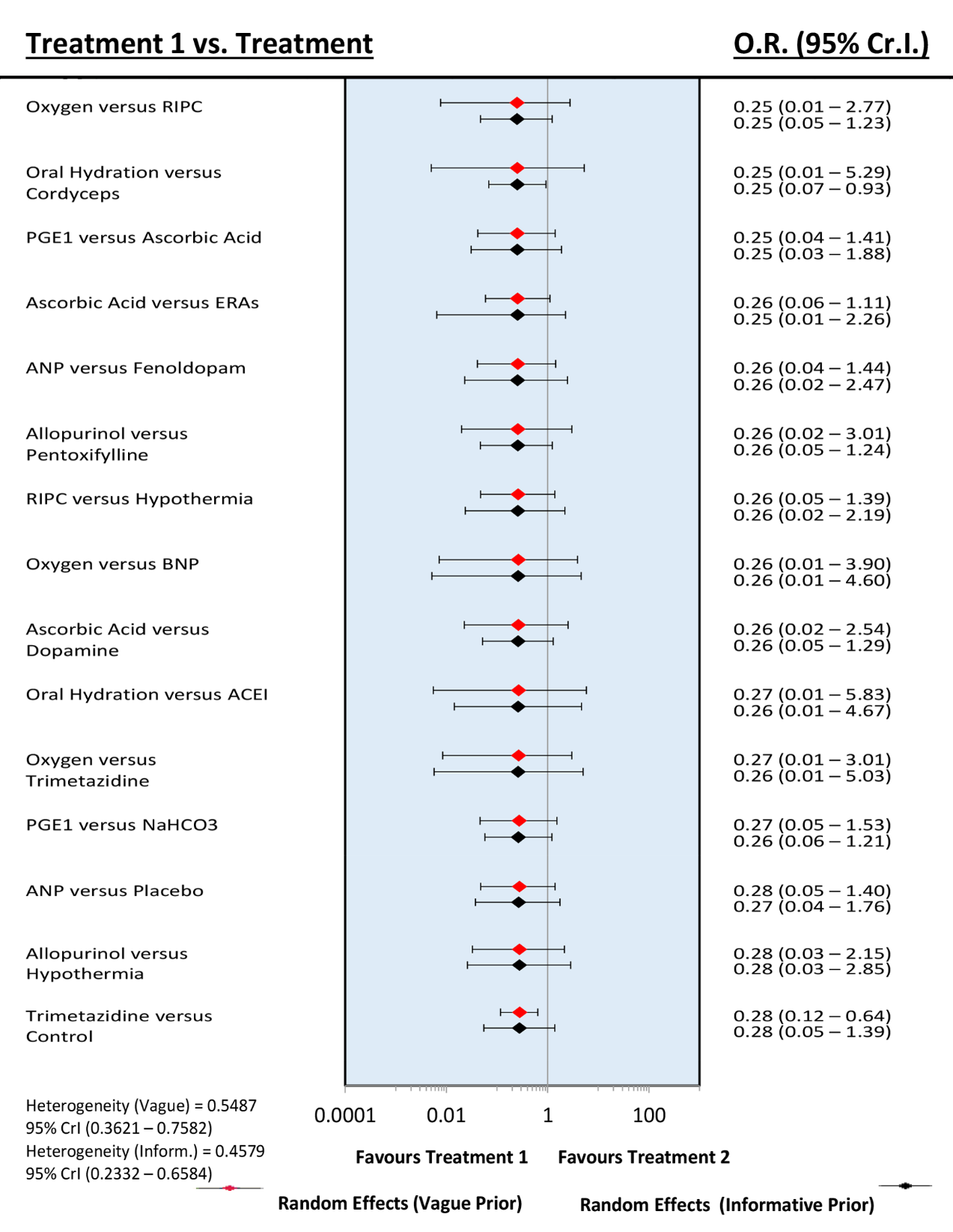


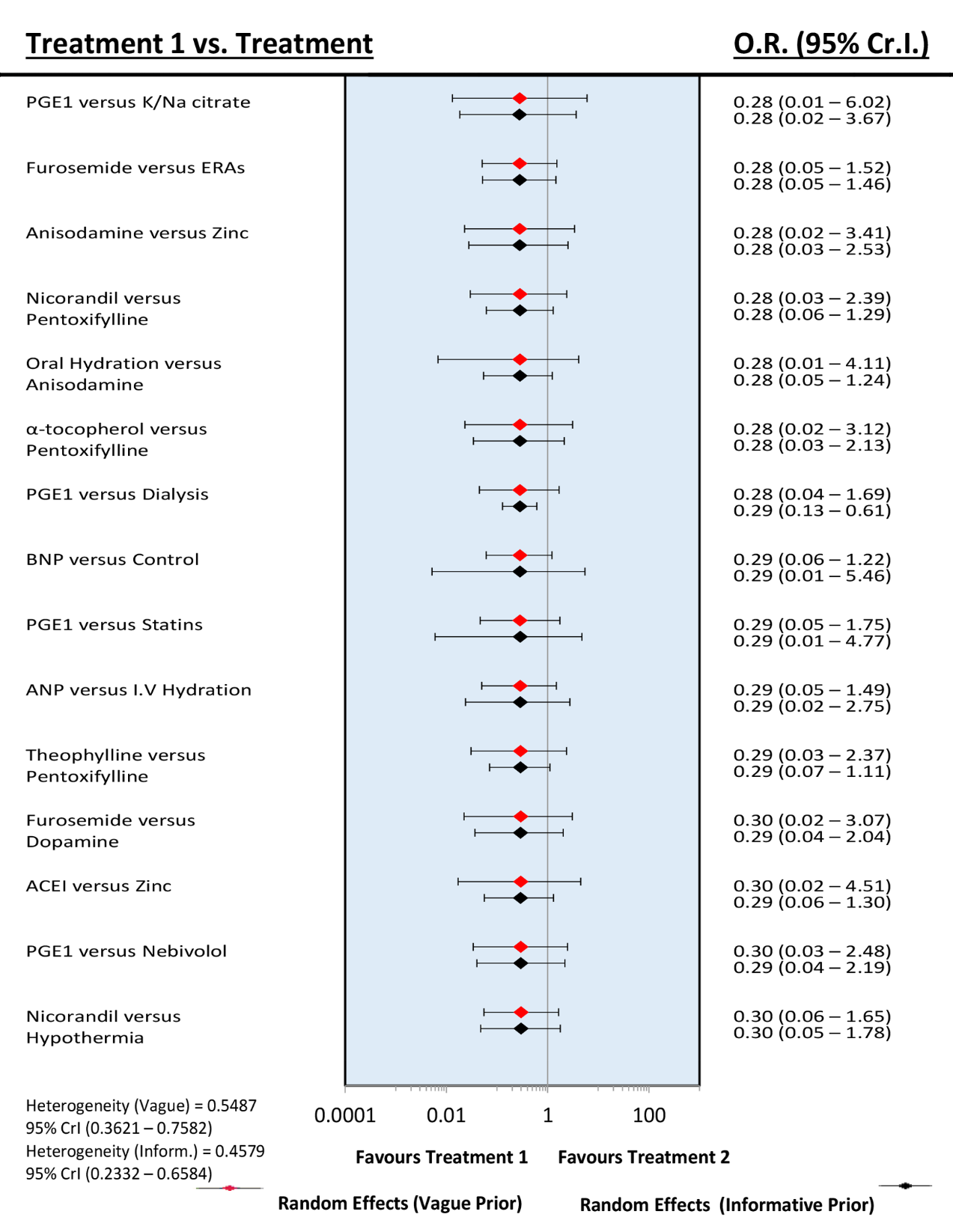


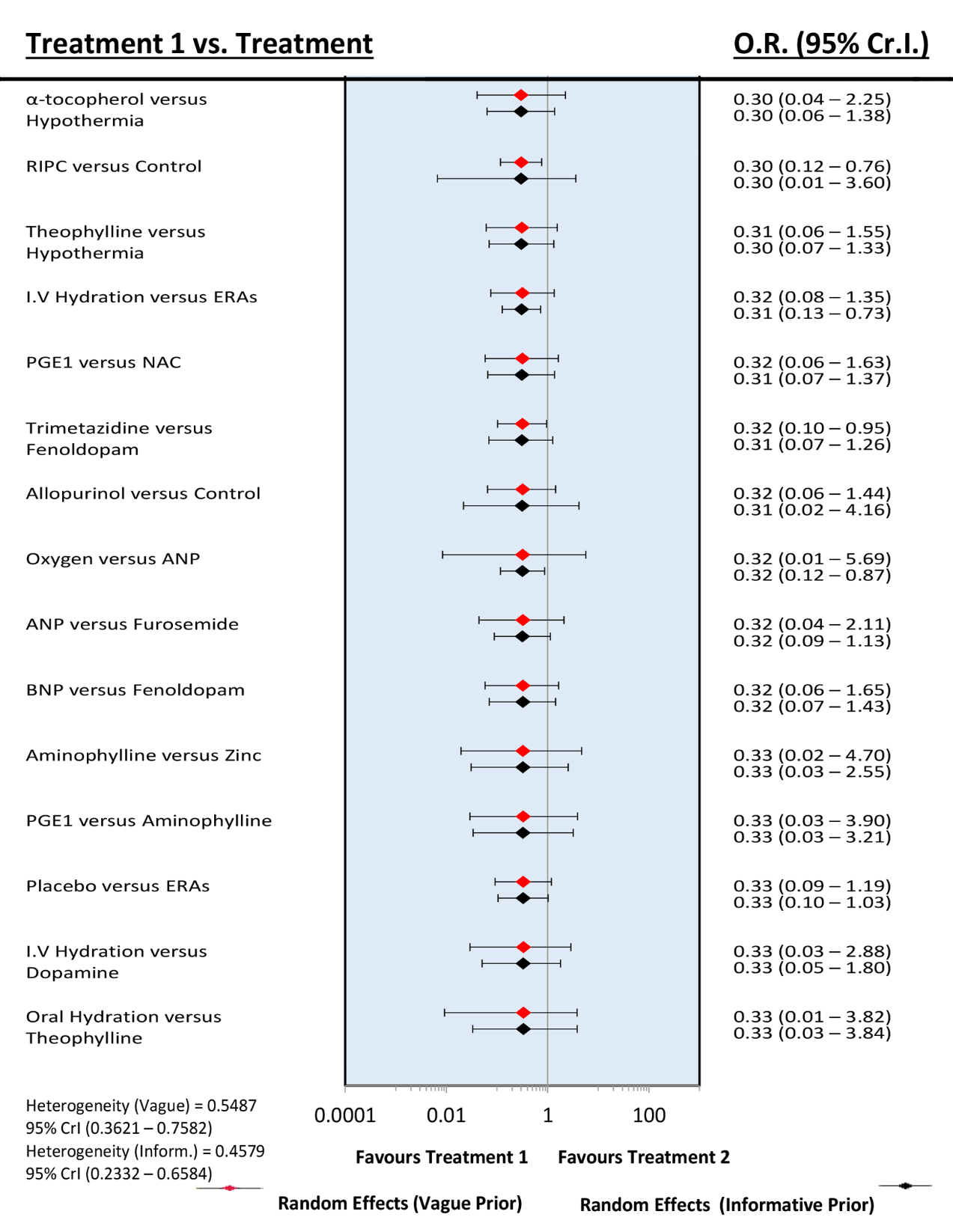


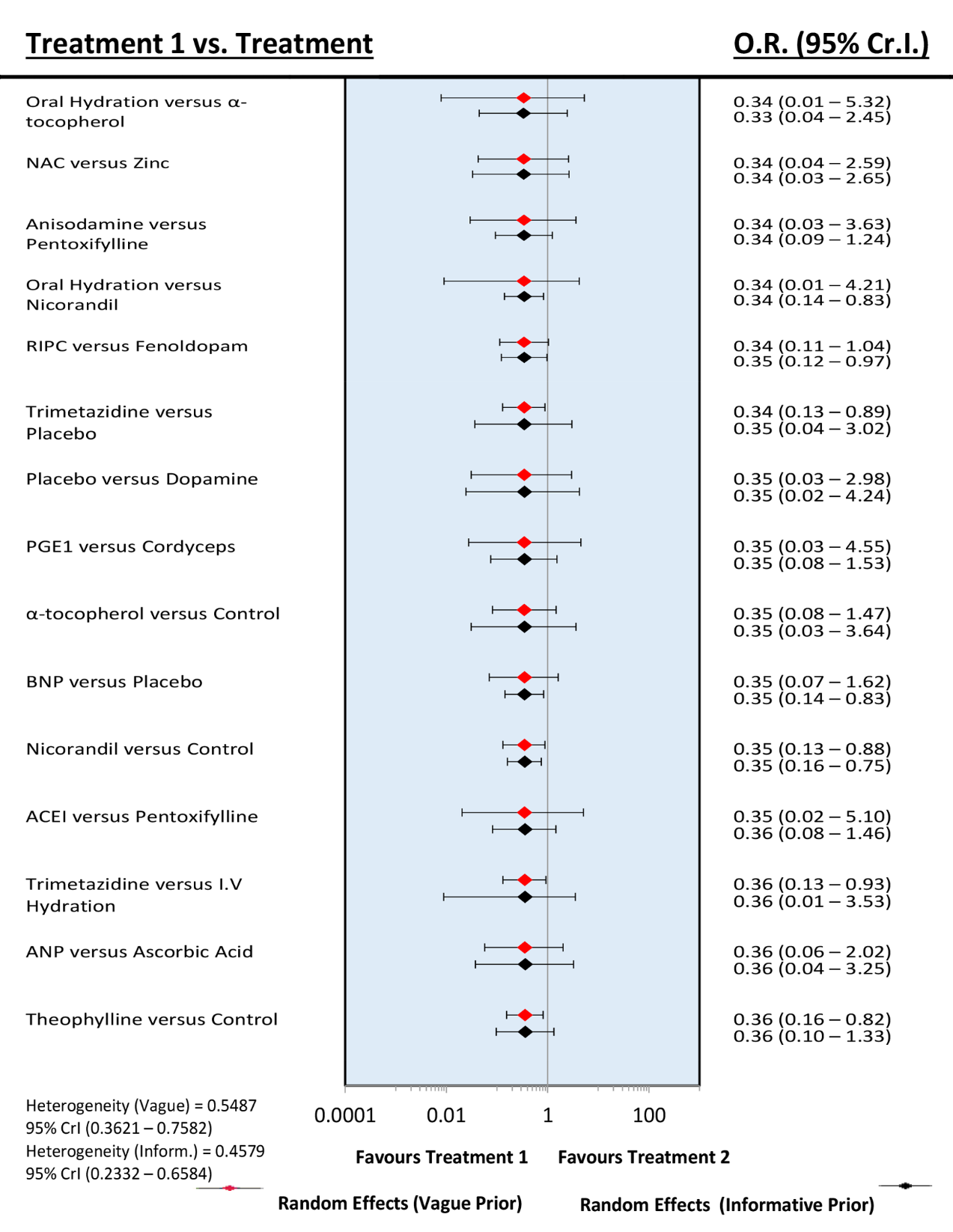


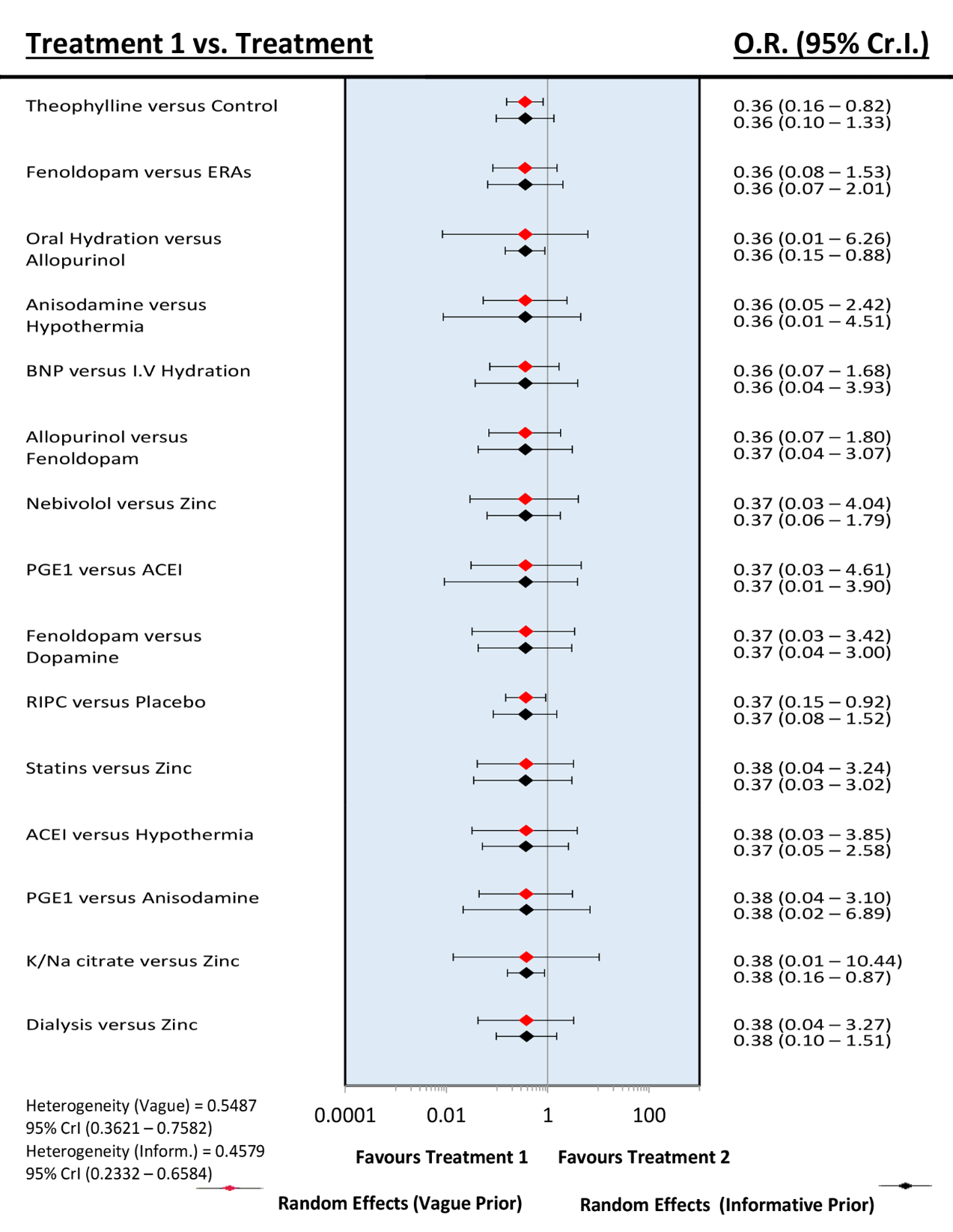


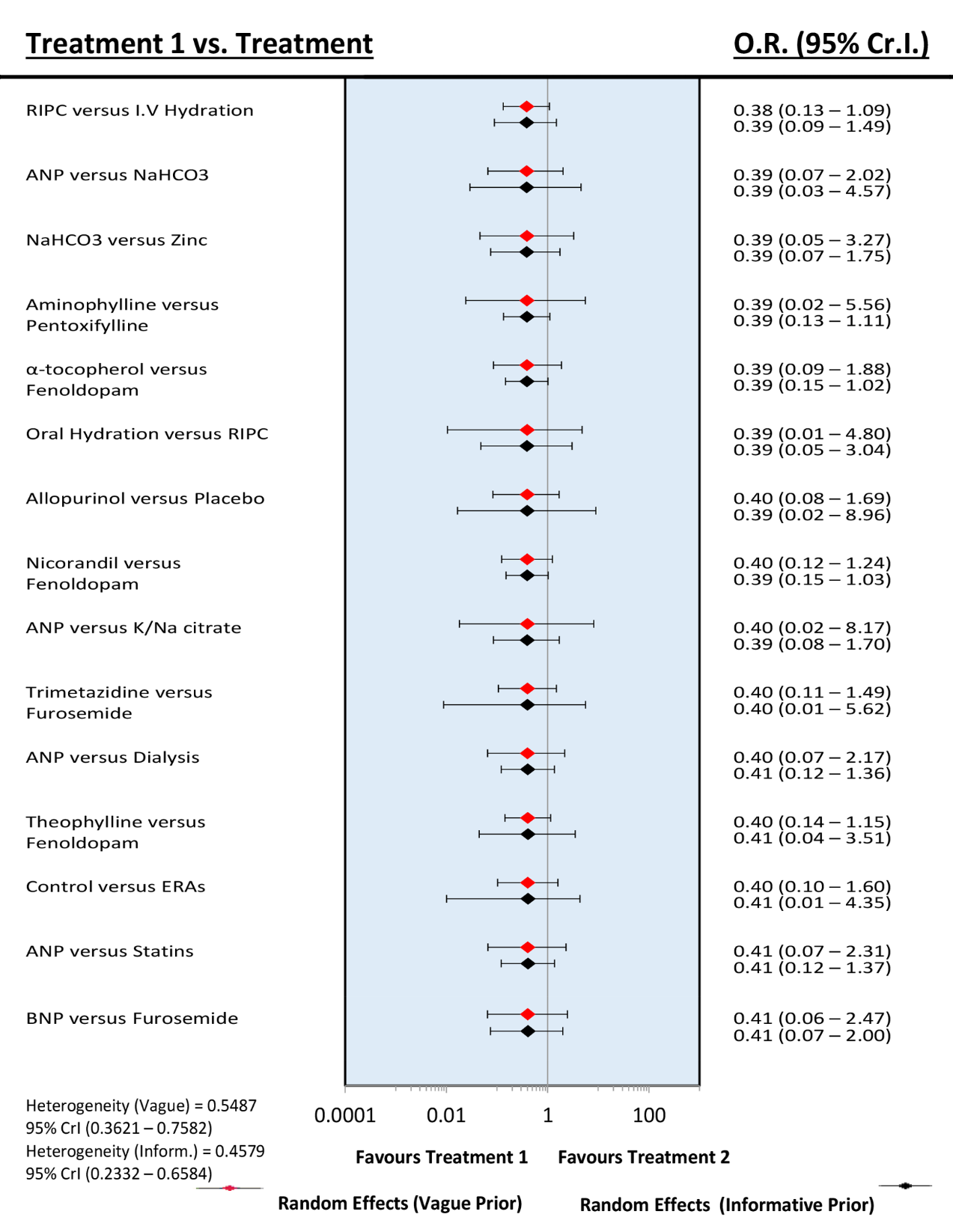


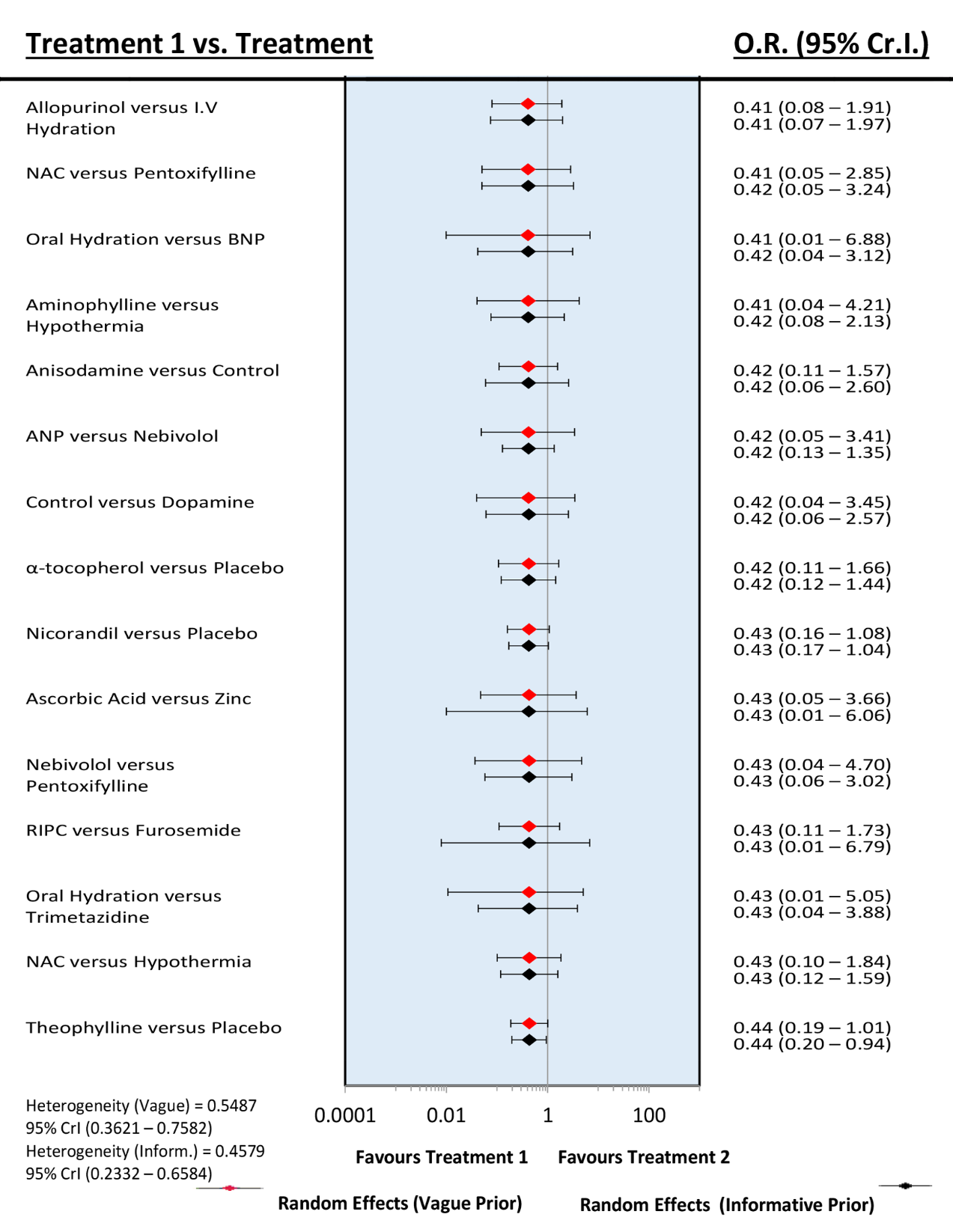


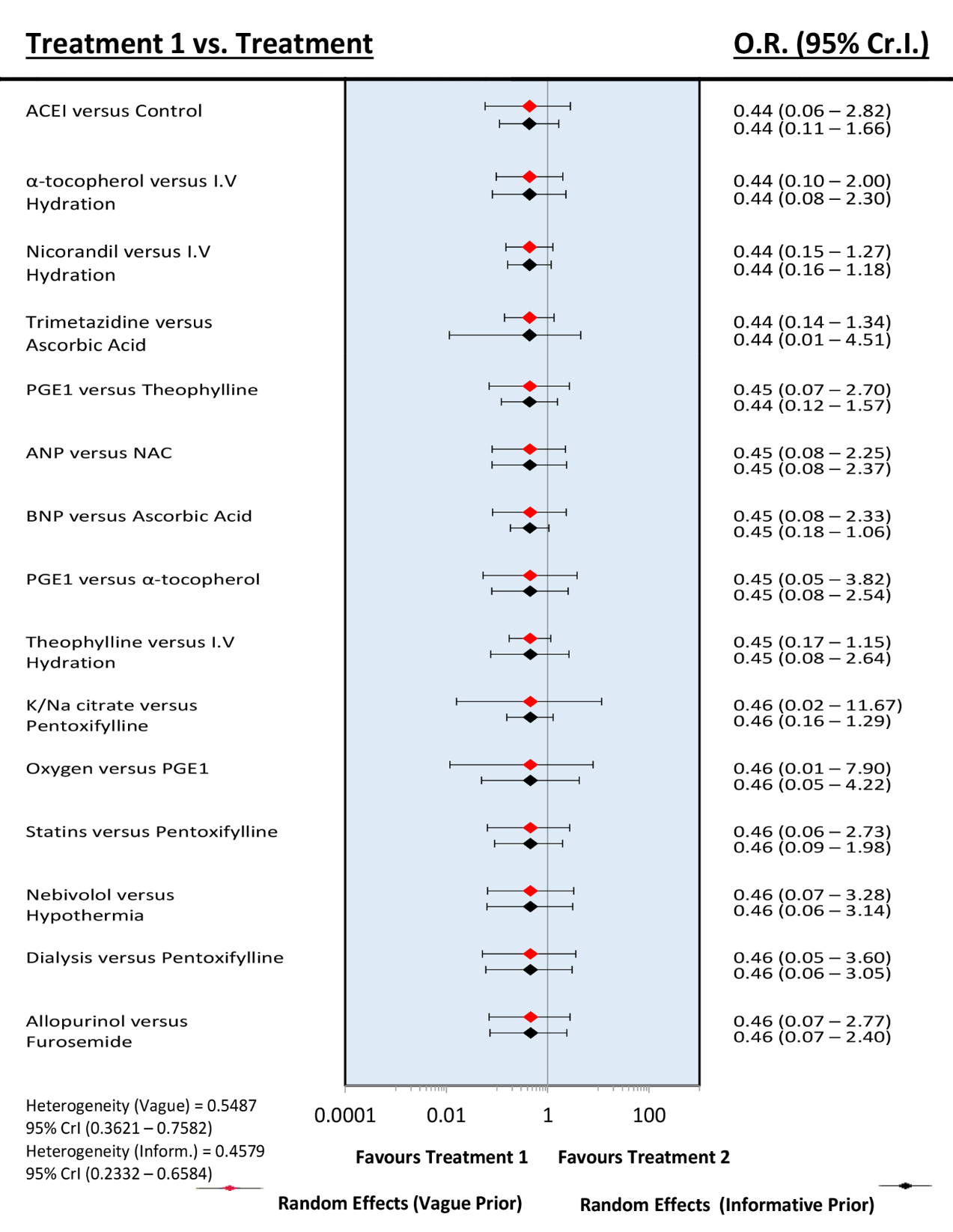


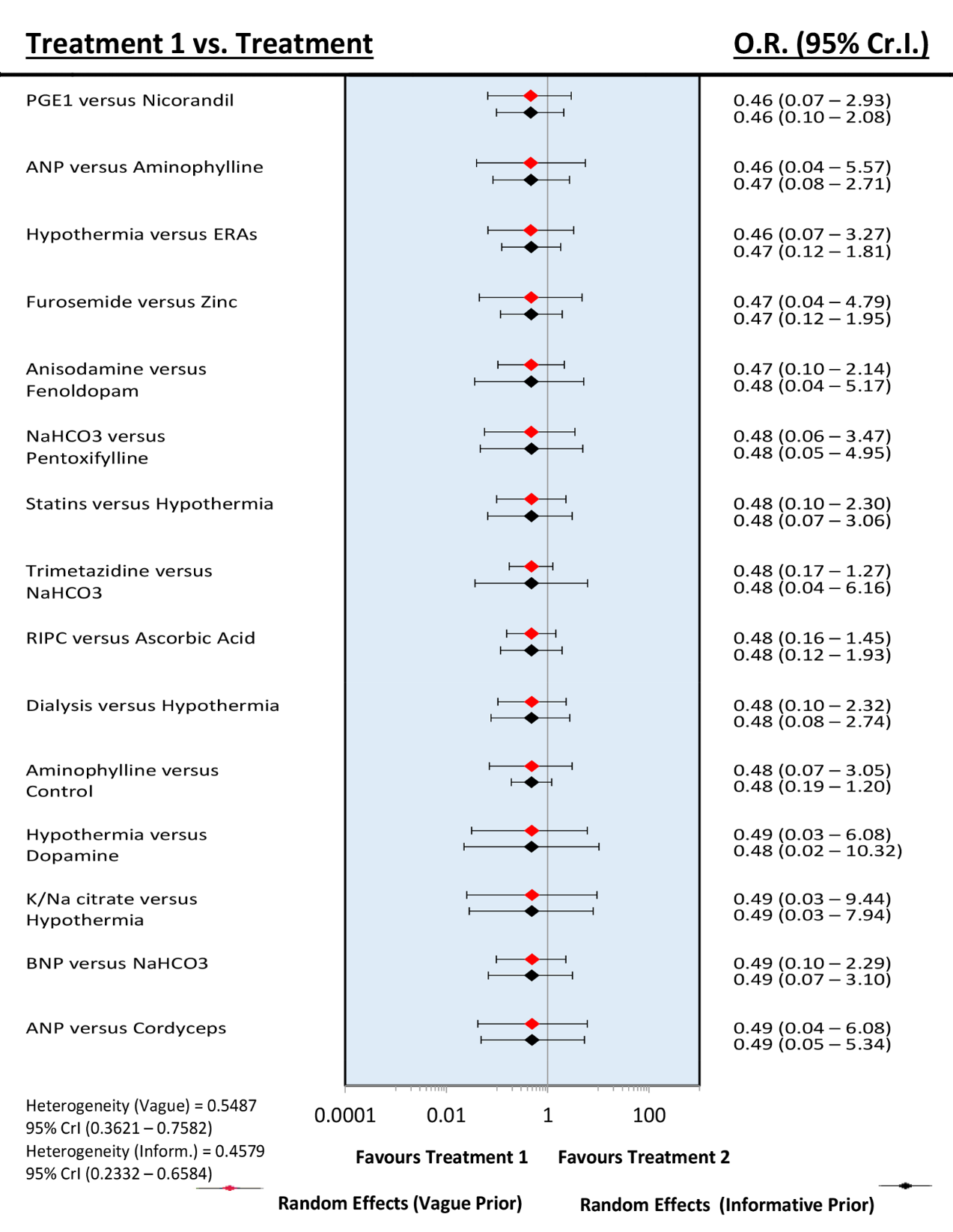


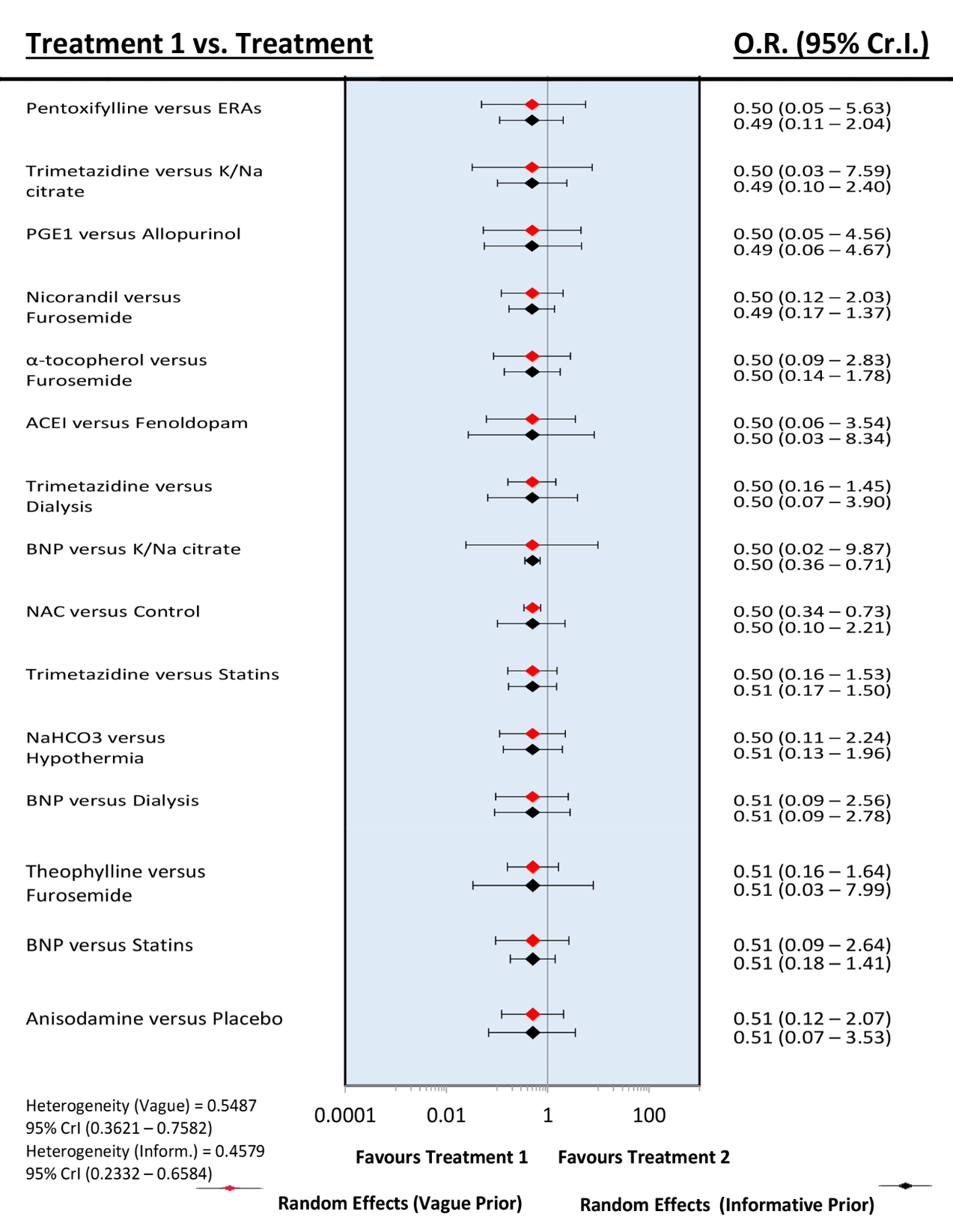


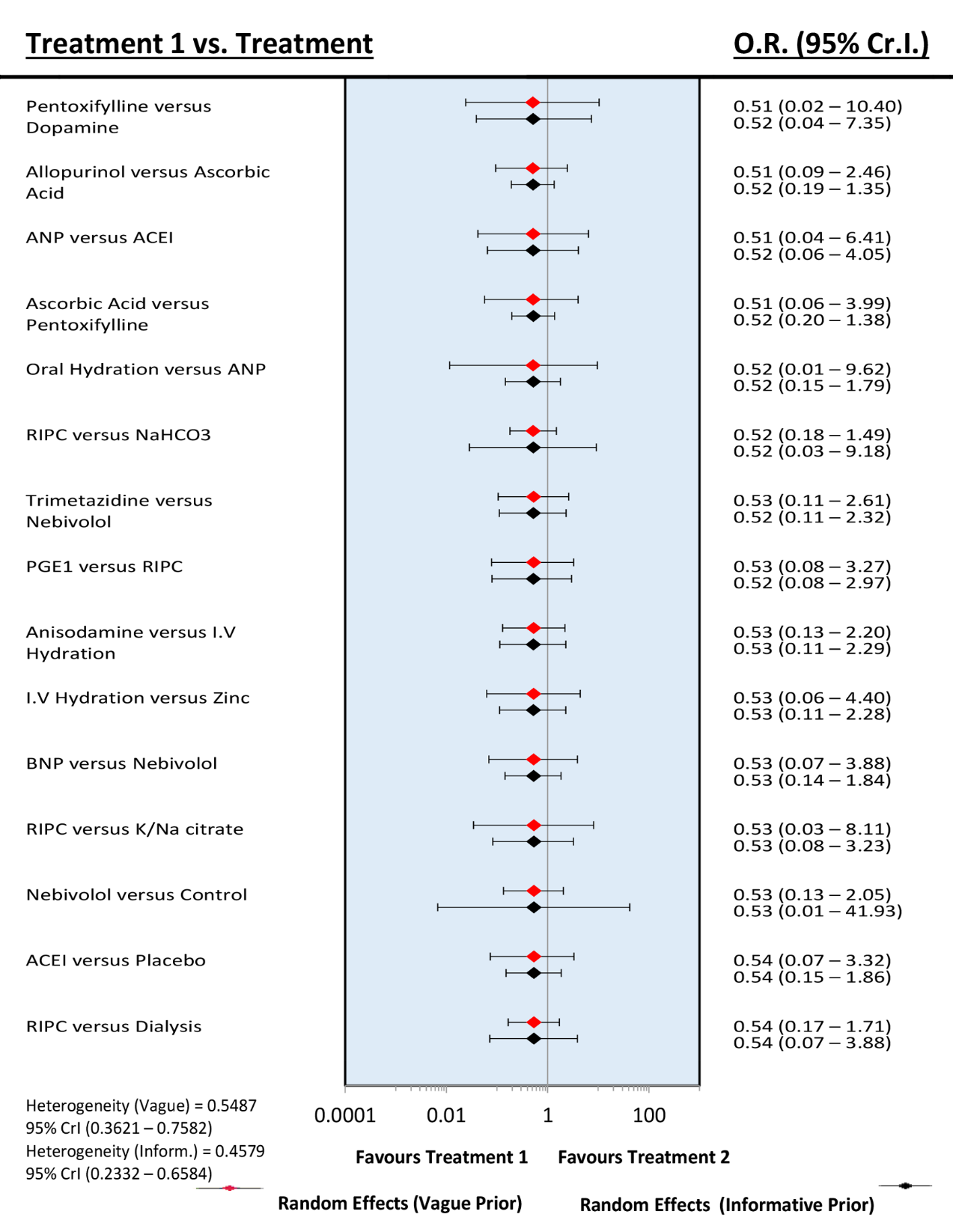


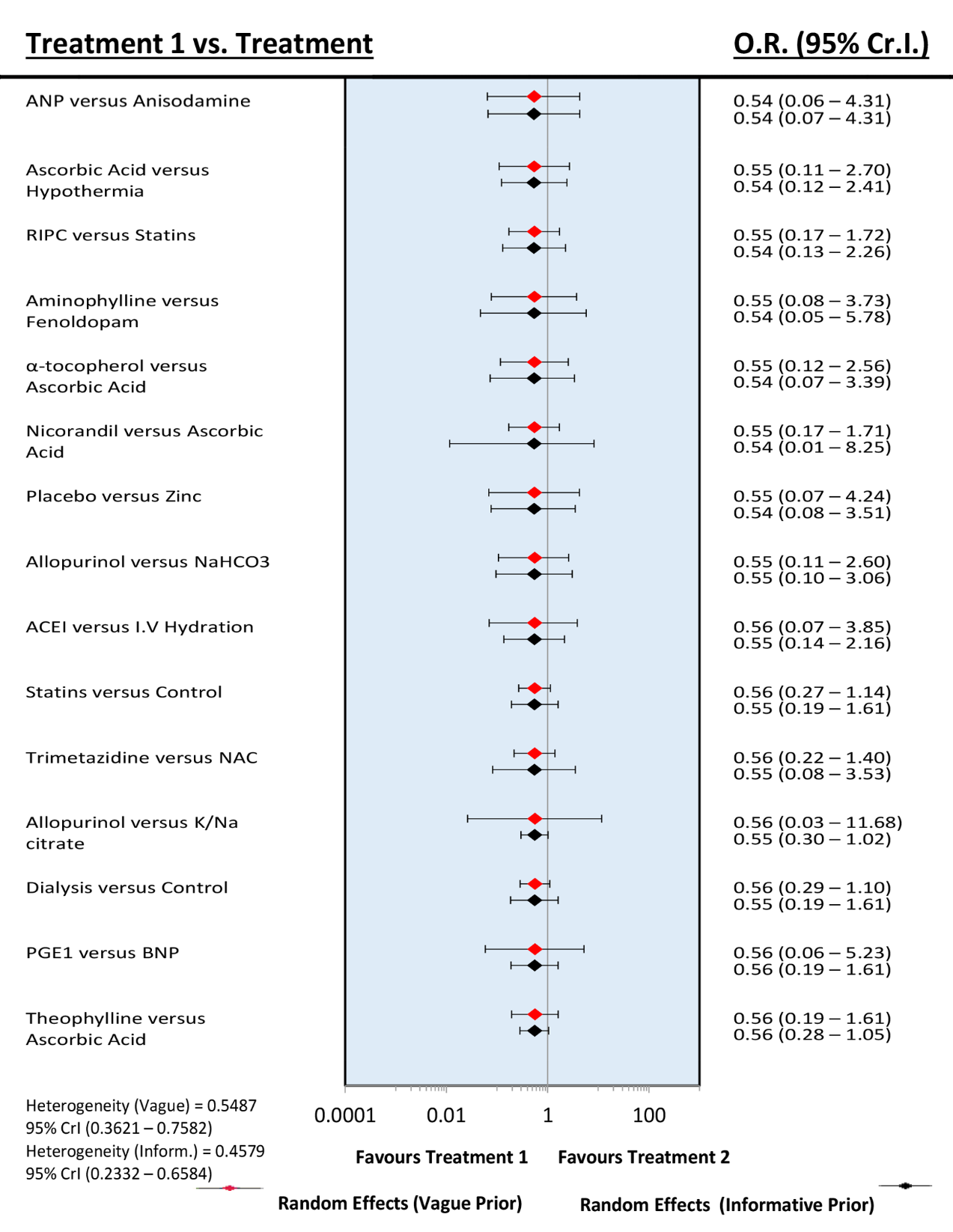


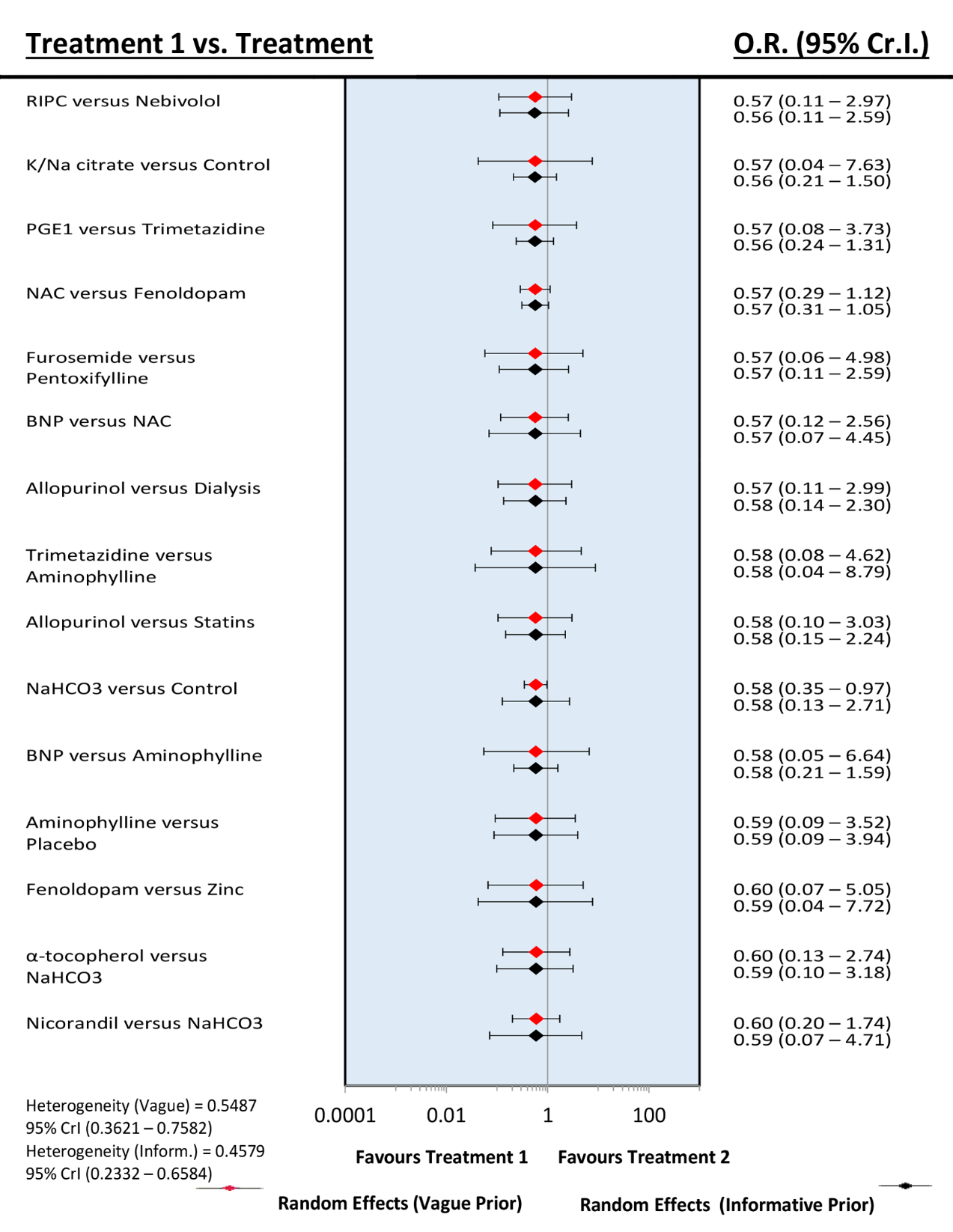


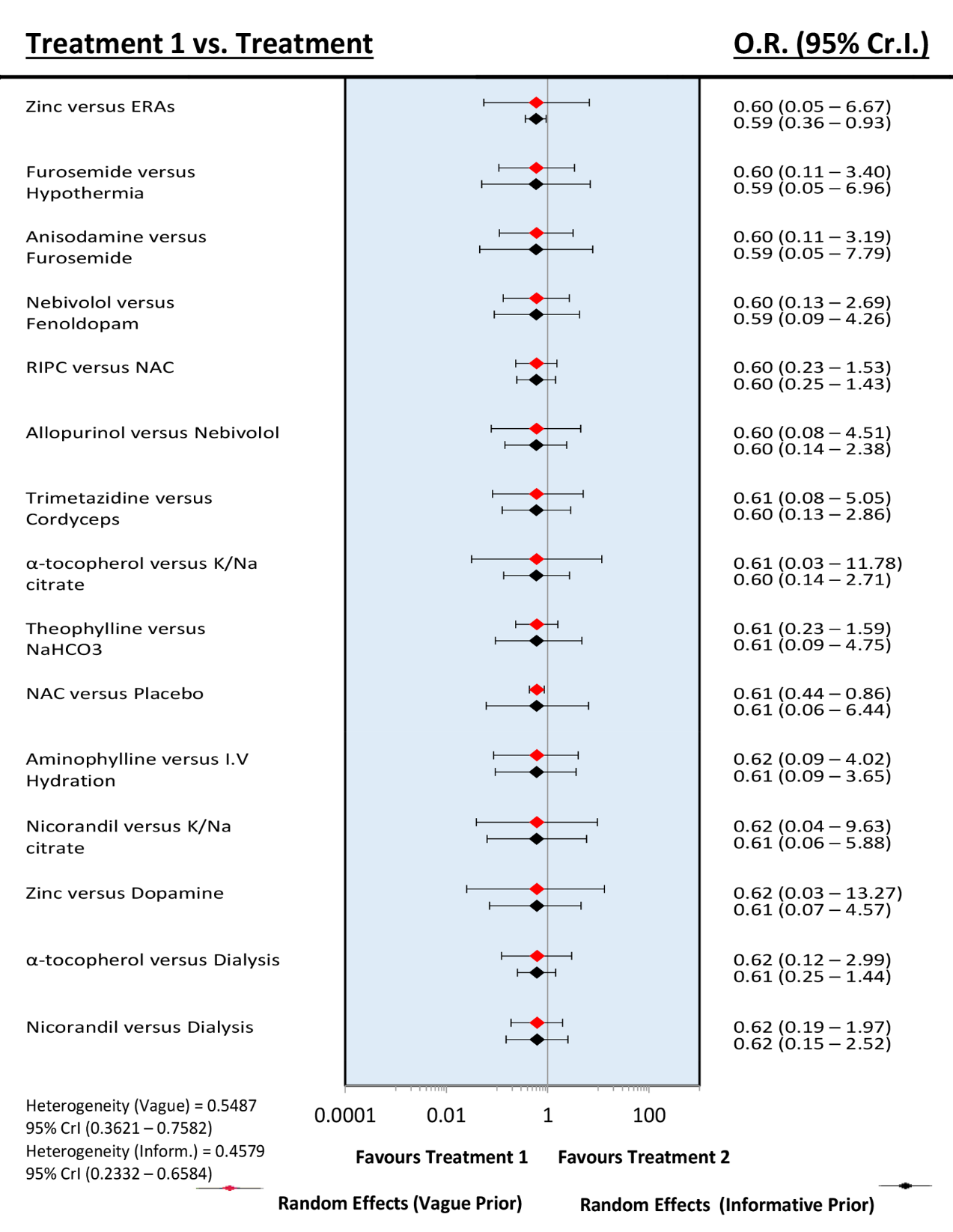


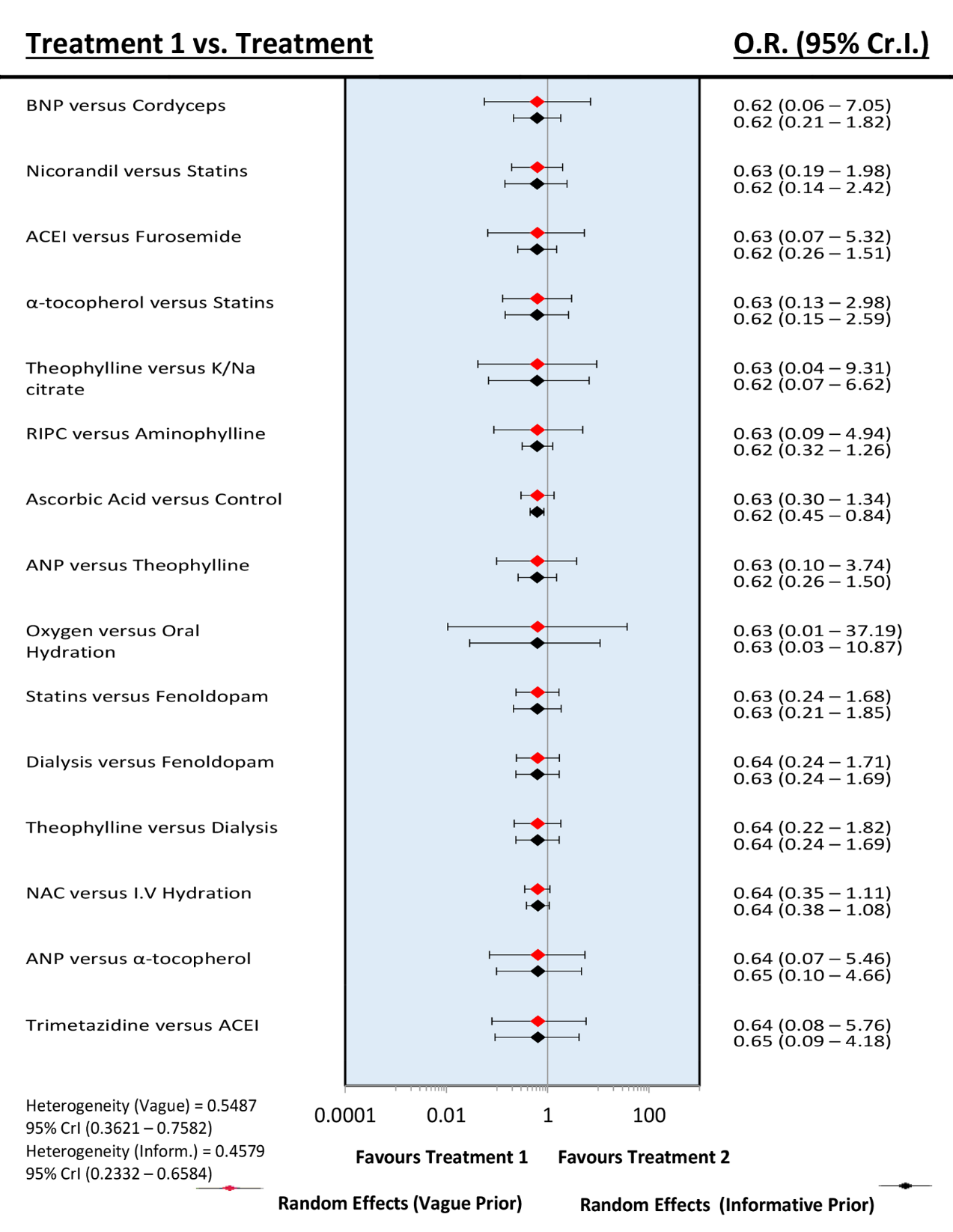


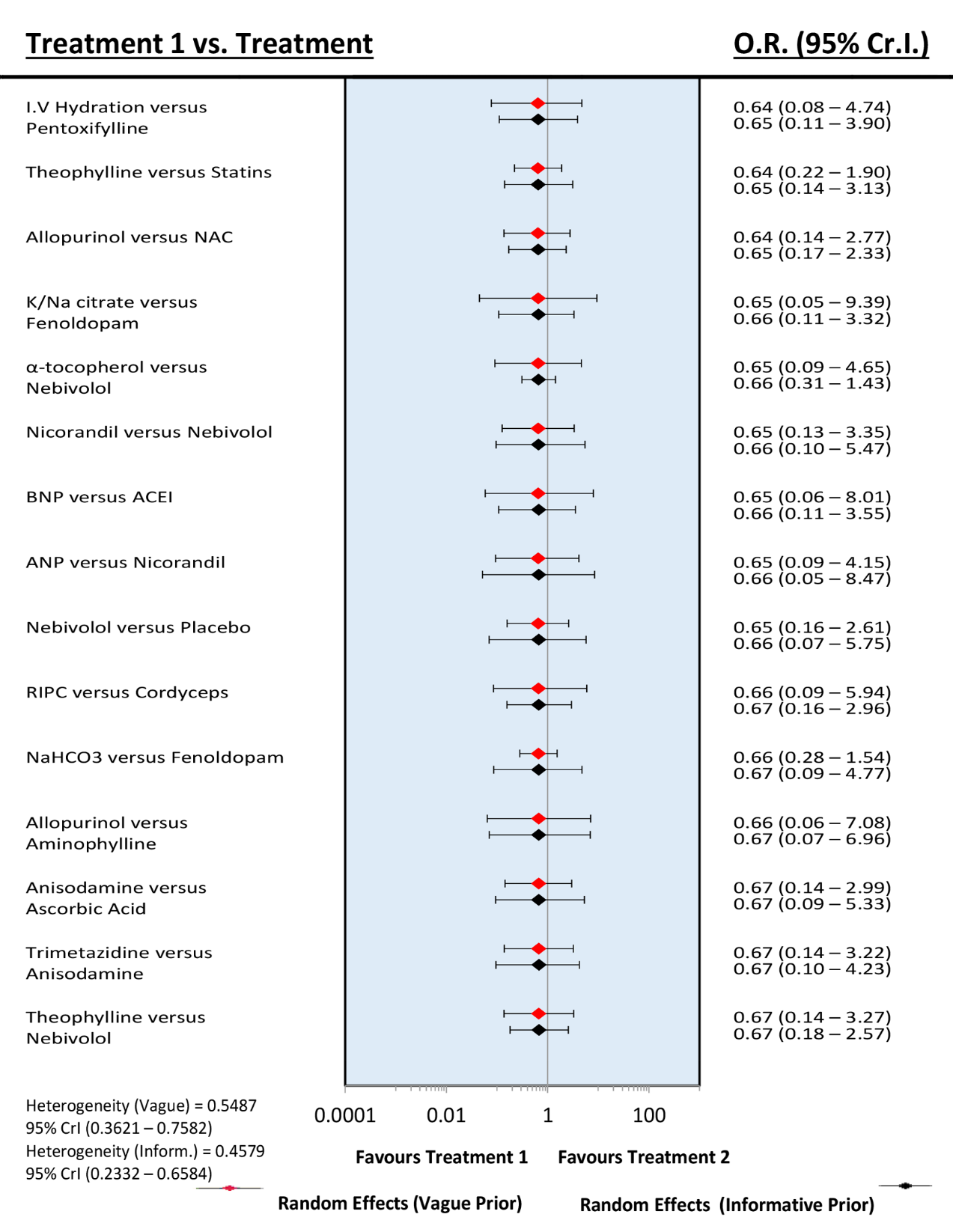


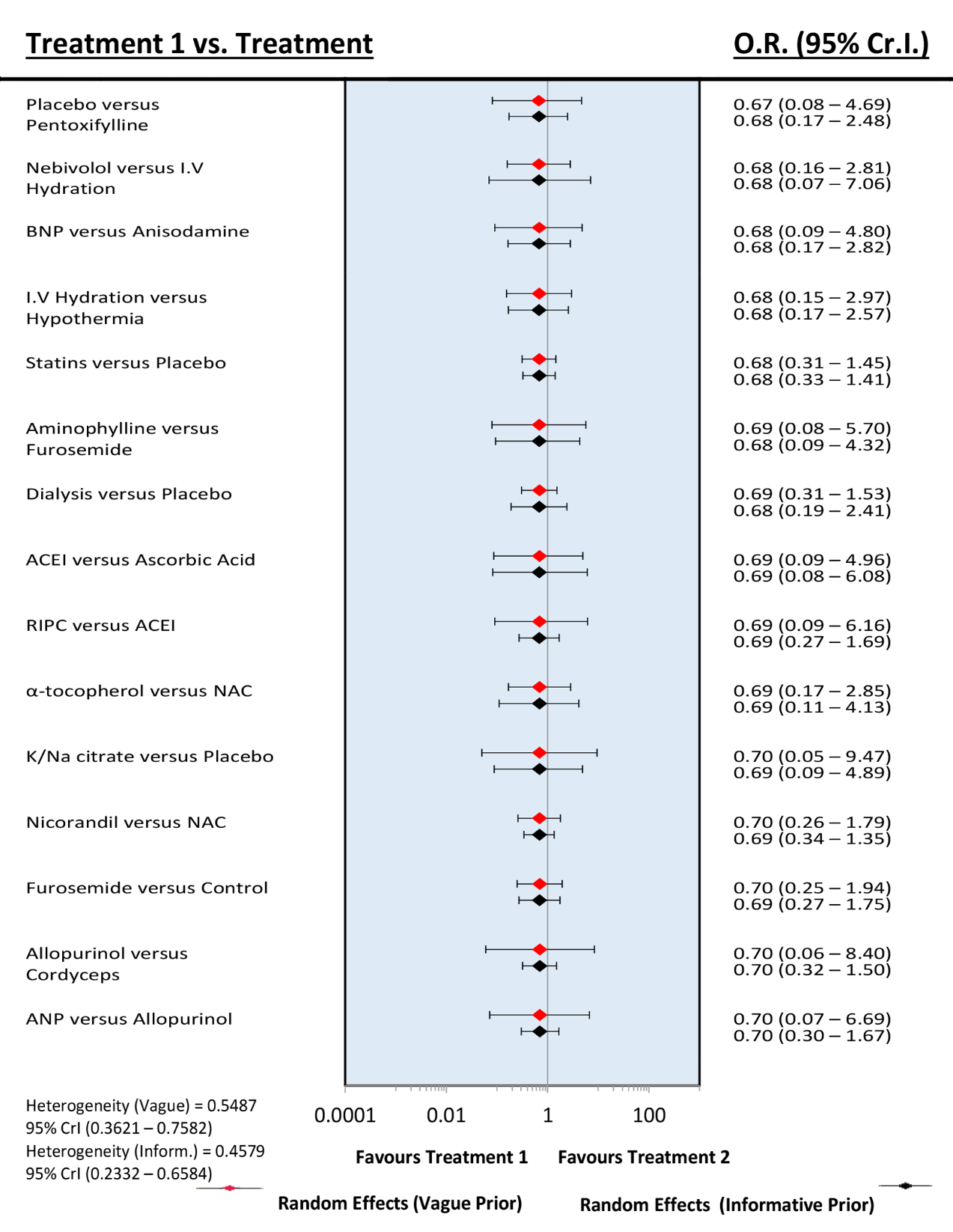


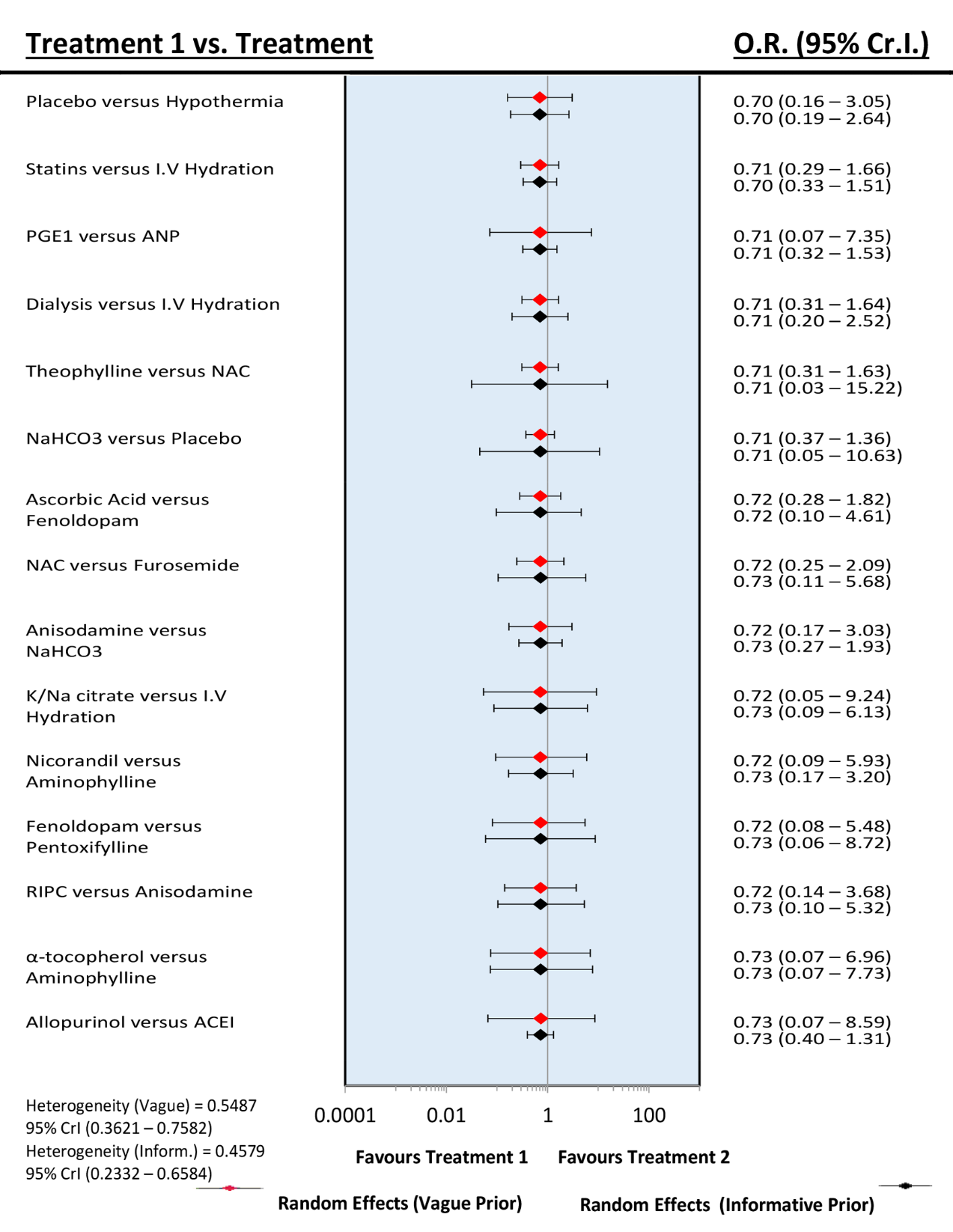


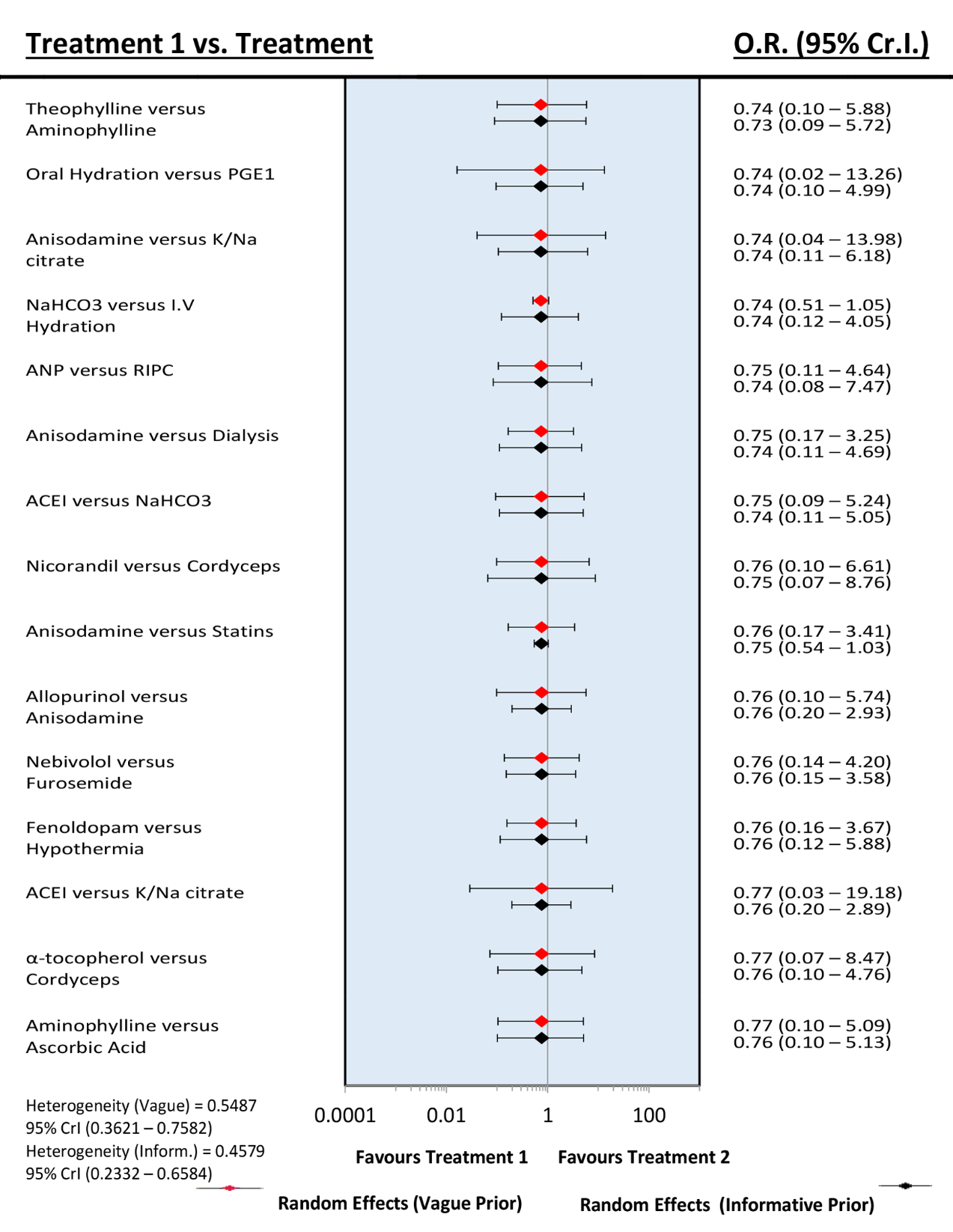


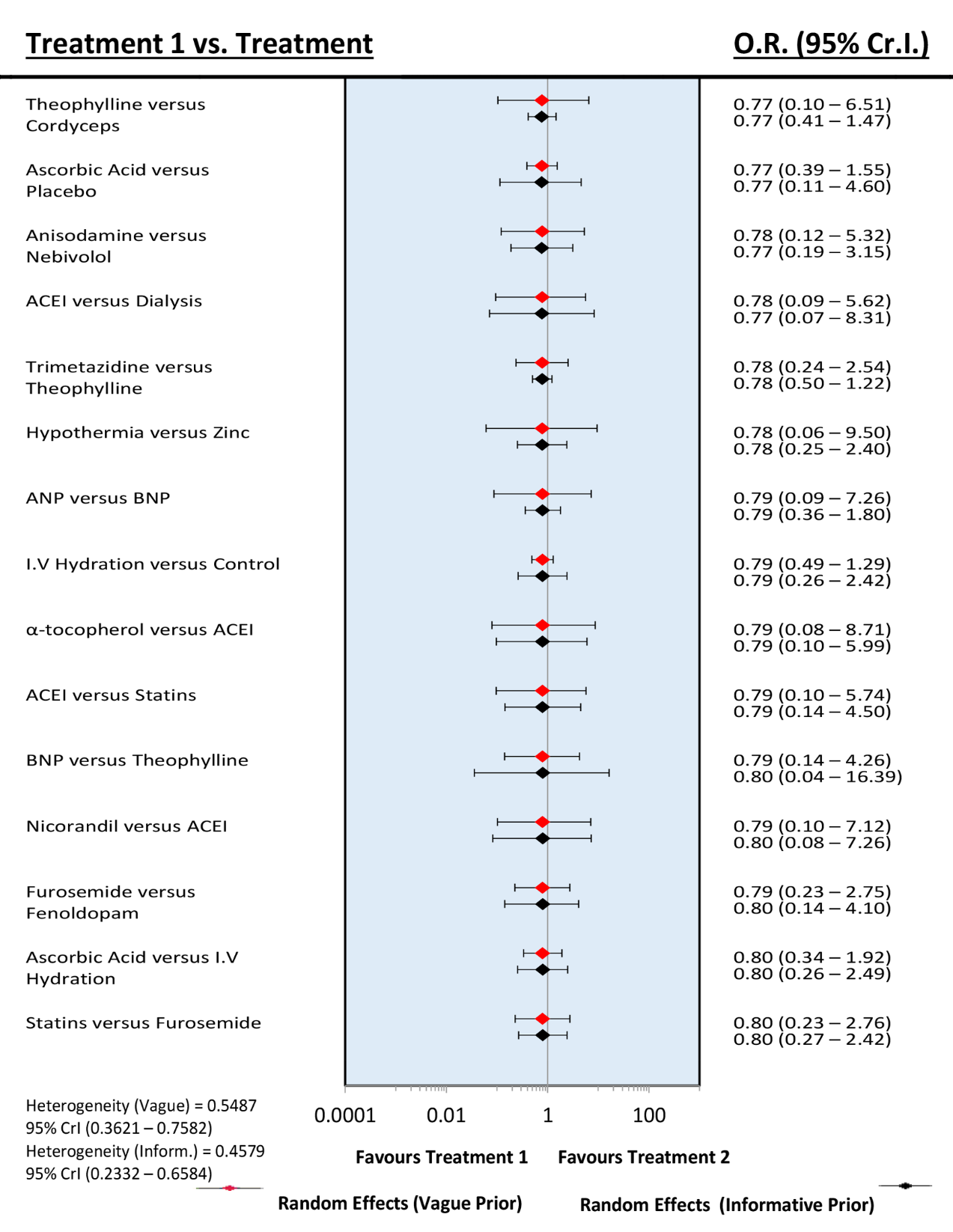


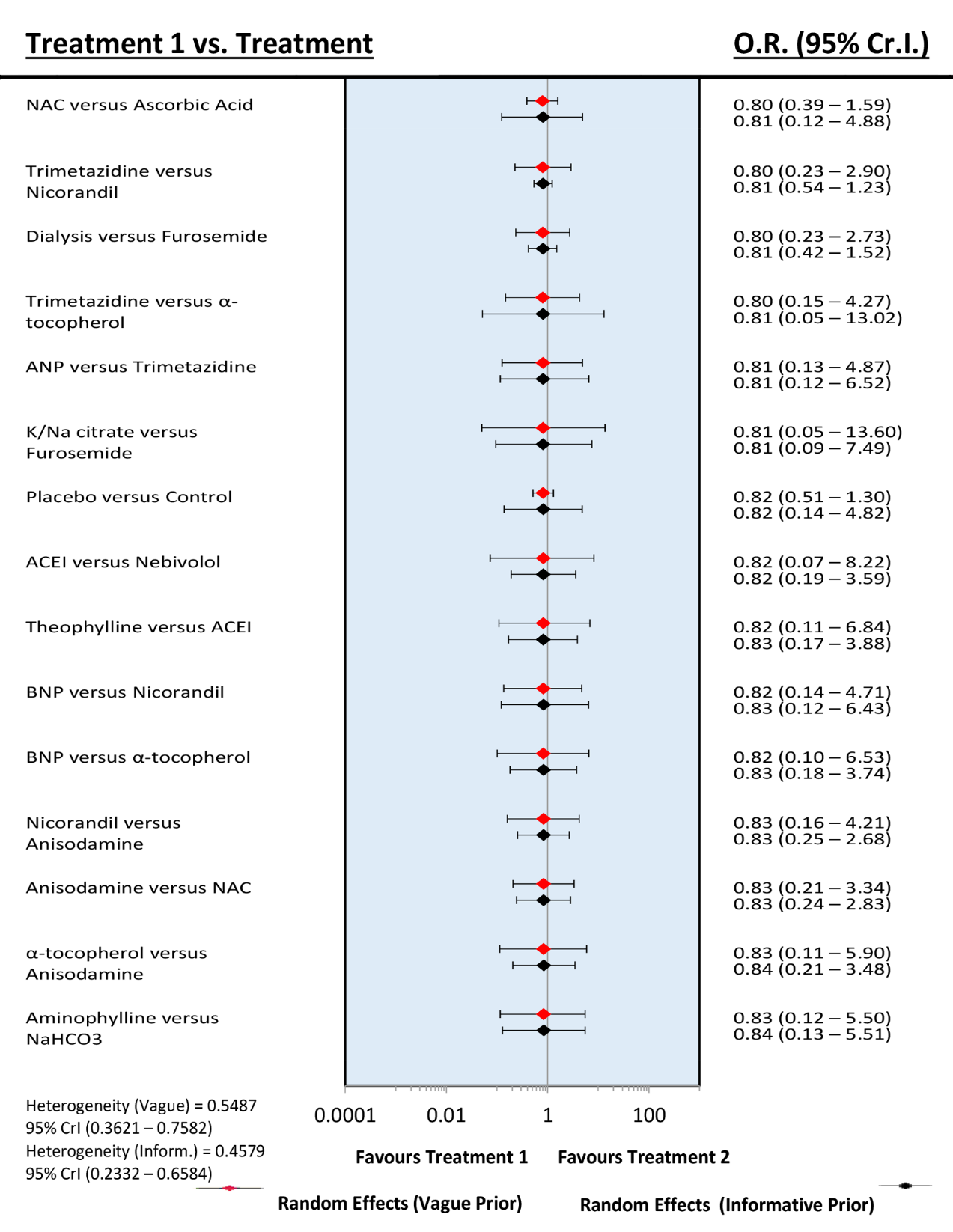


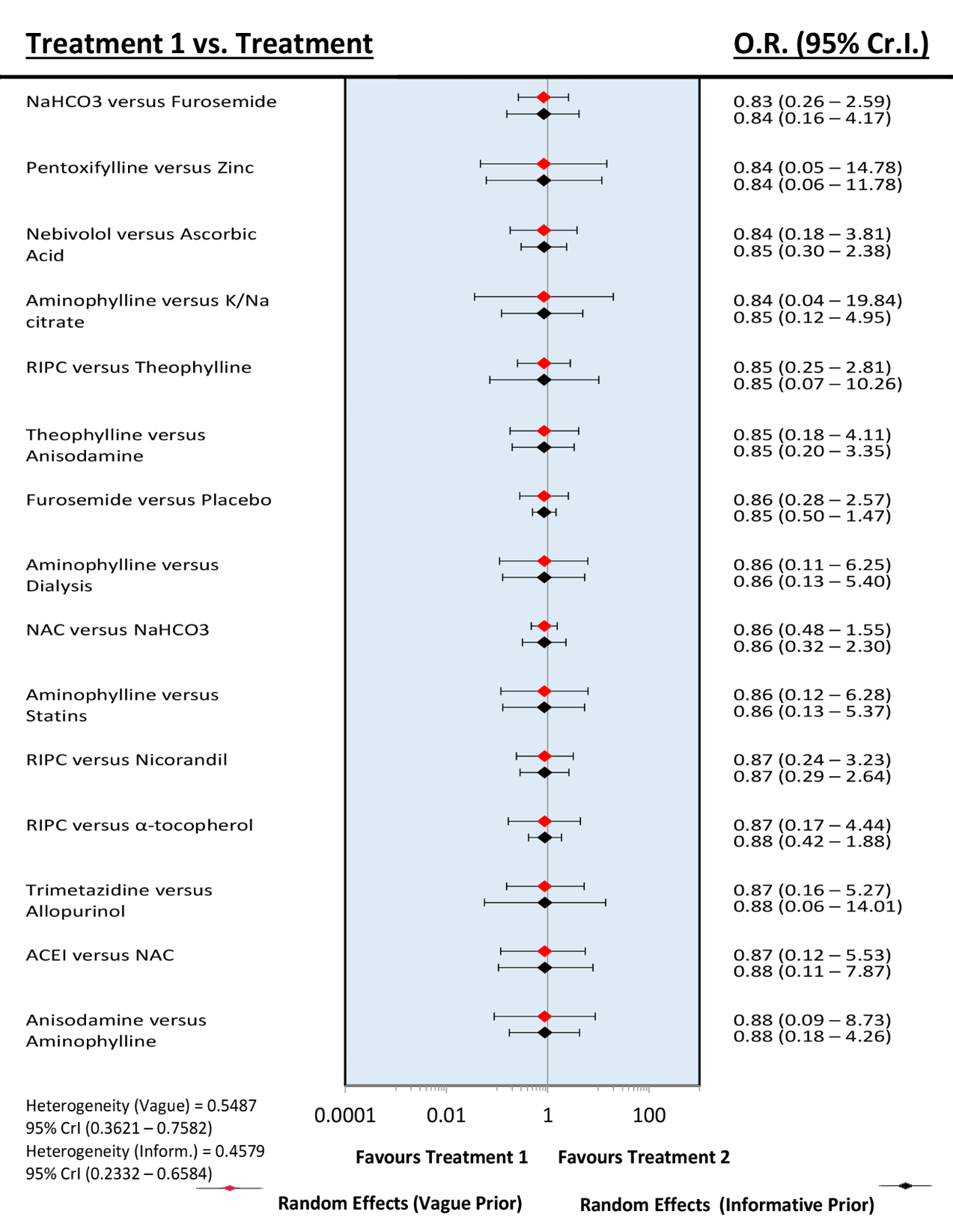


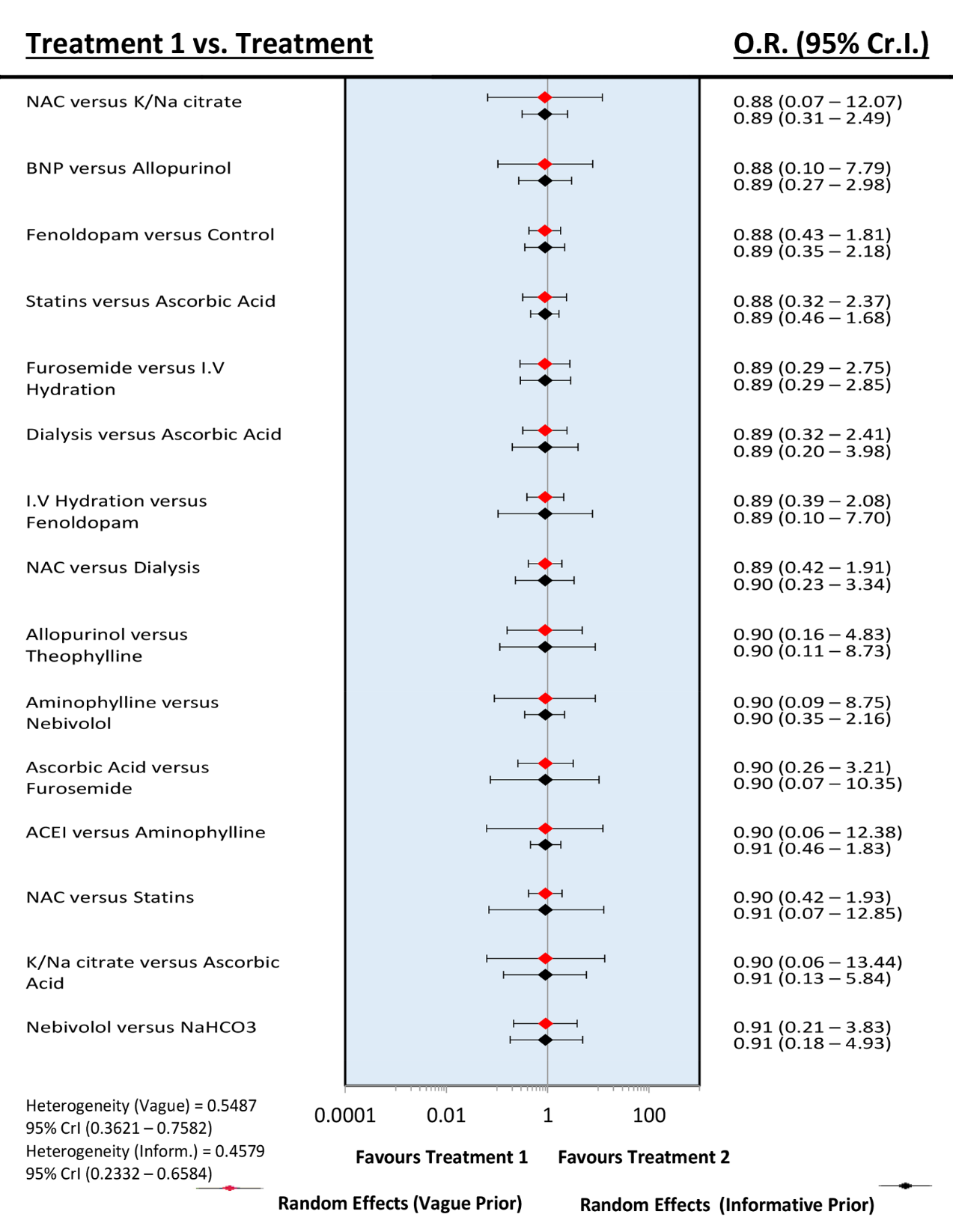


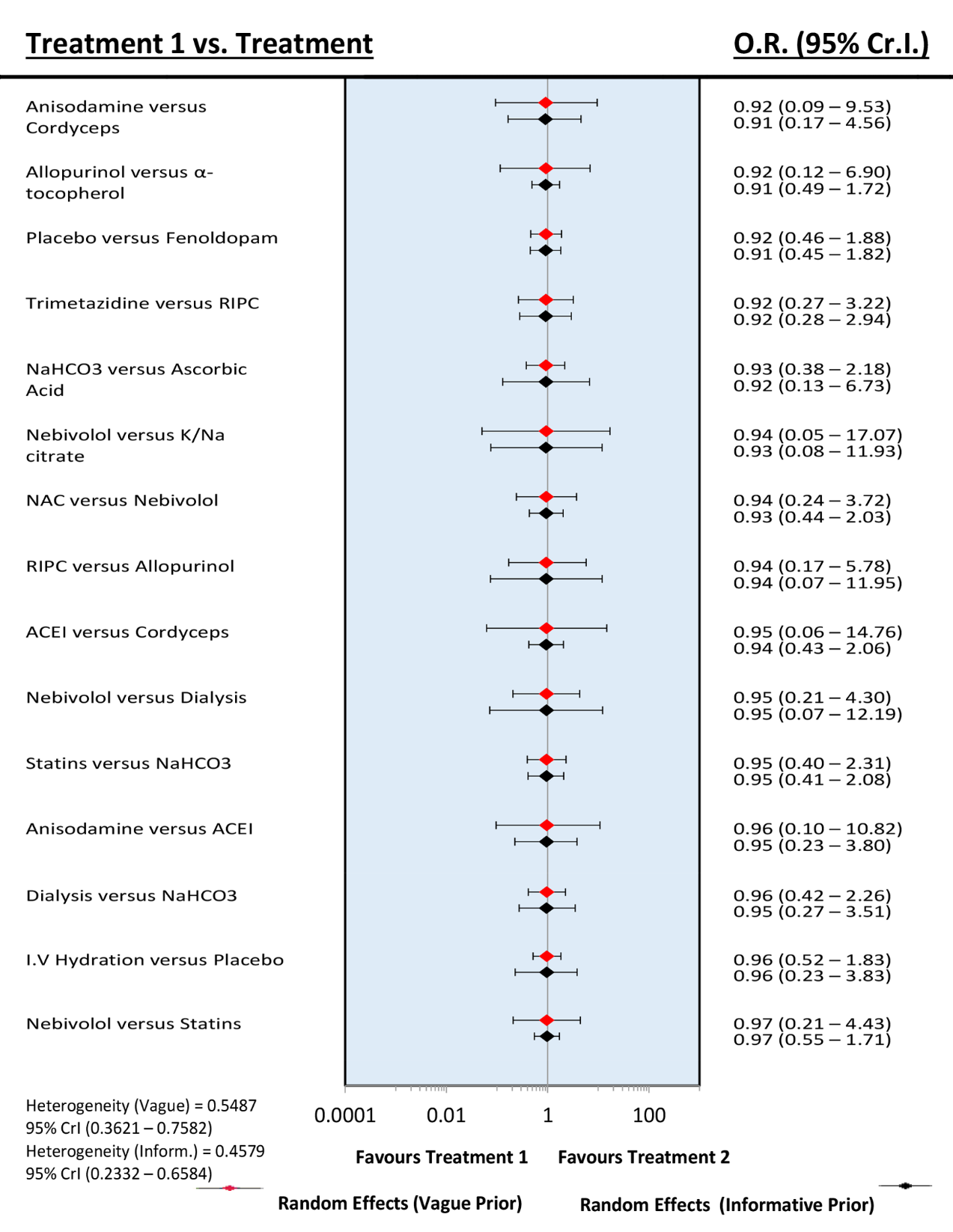


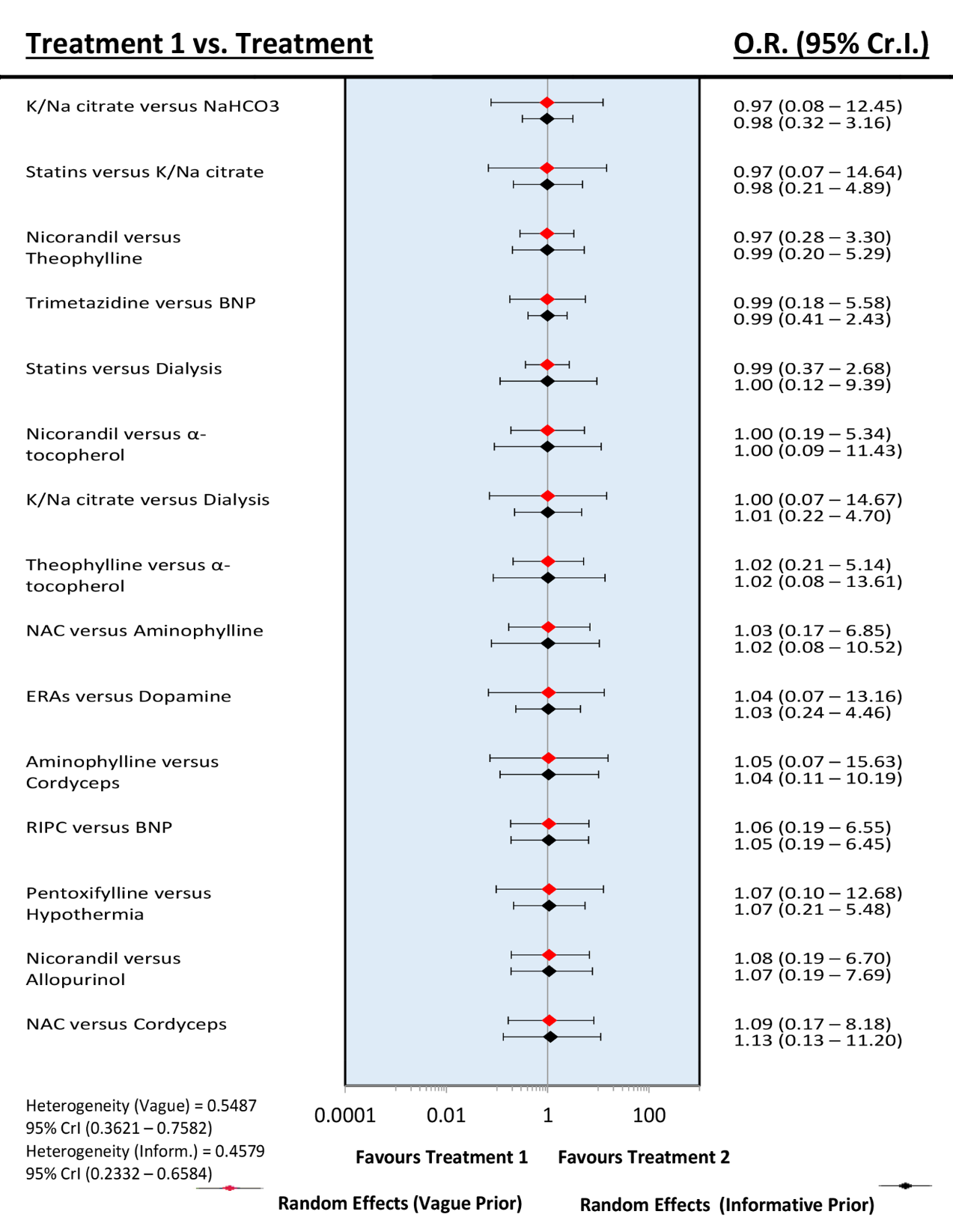


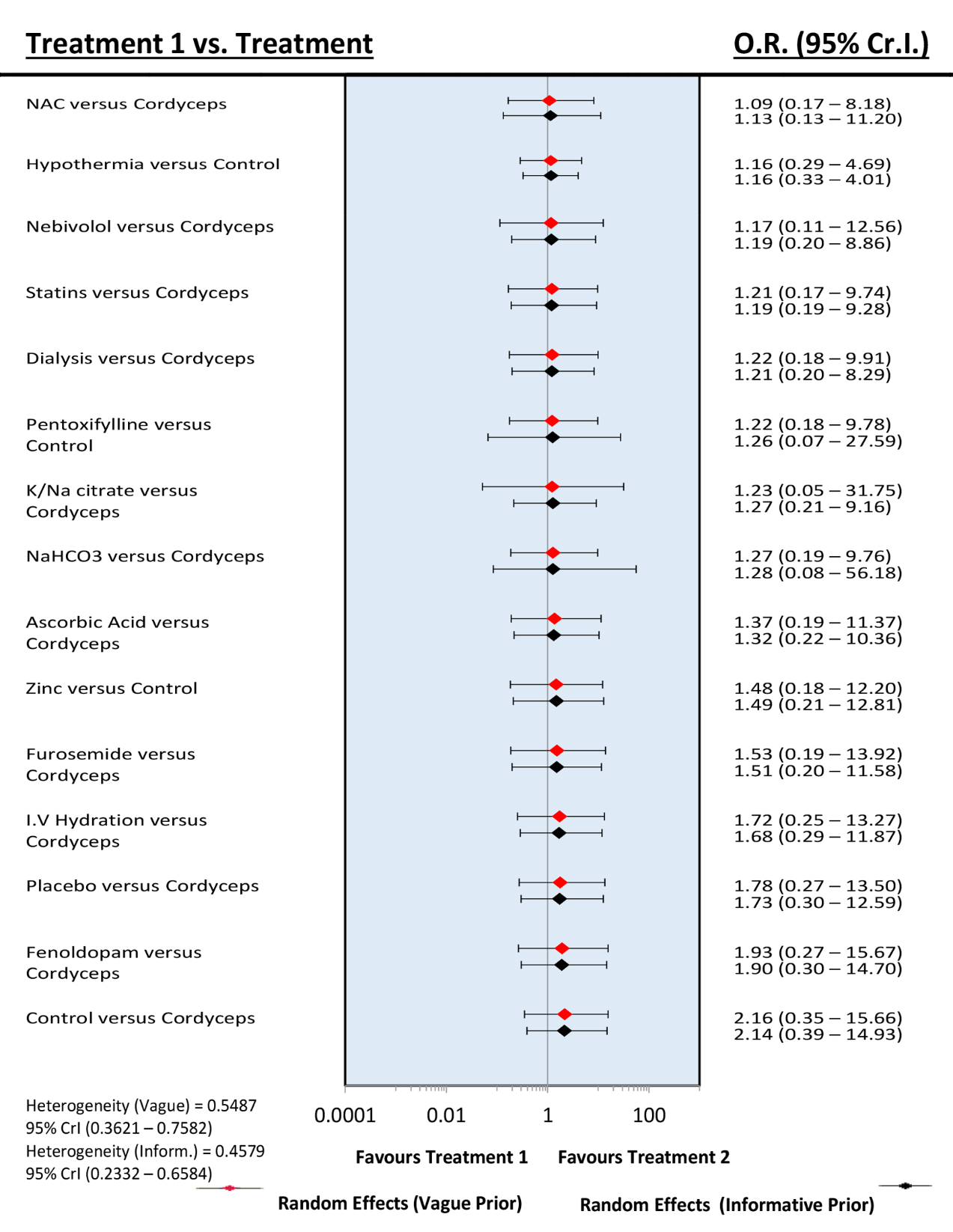


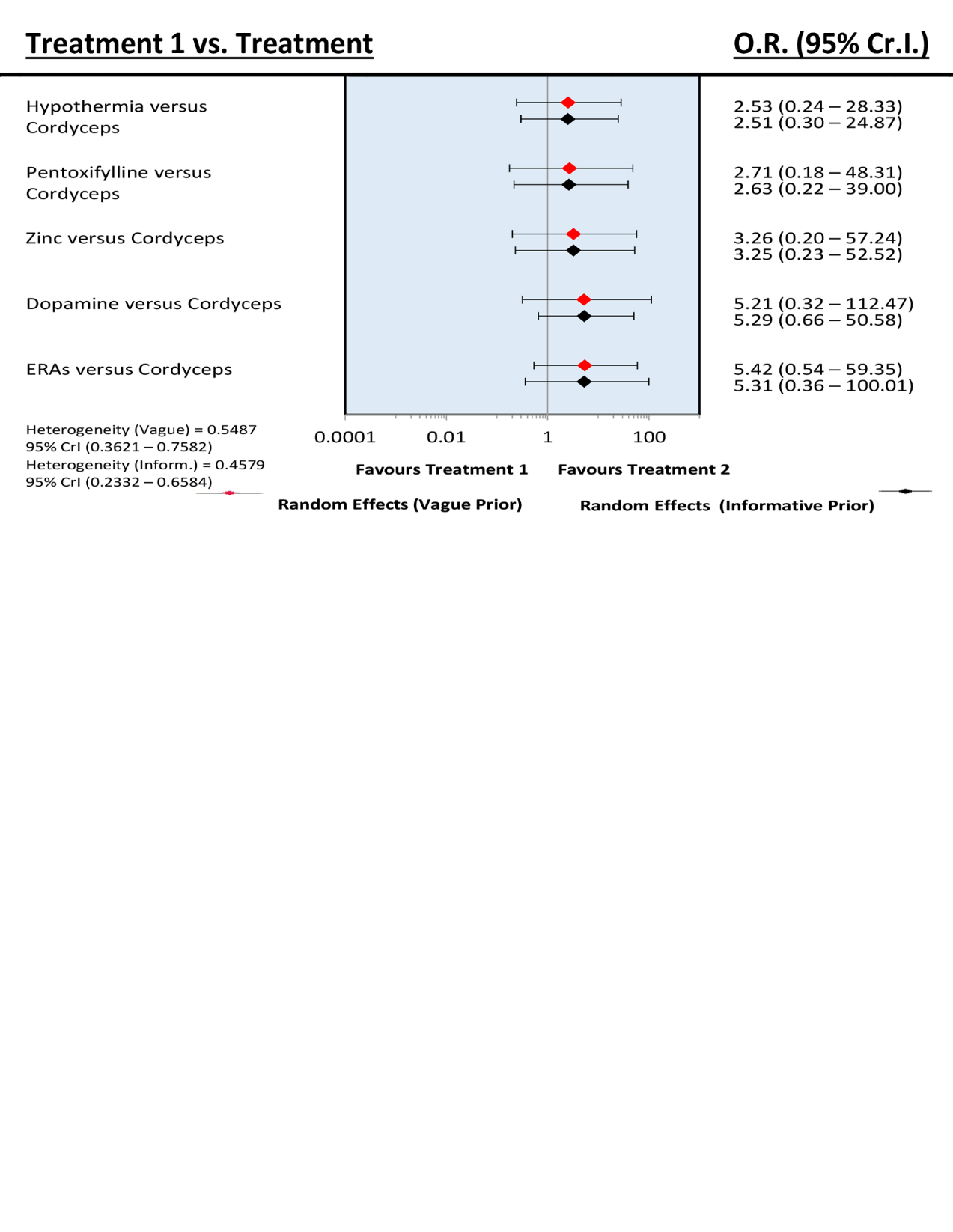


Figure 4Forest Plot (Results from R analysis)


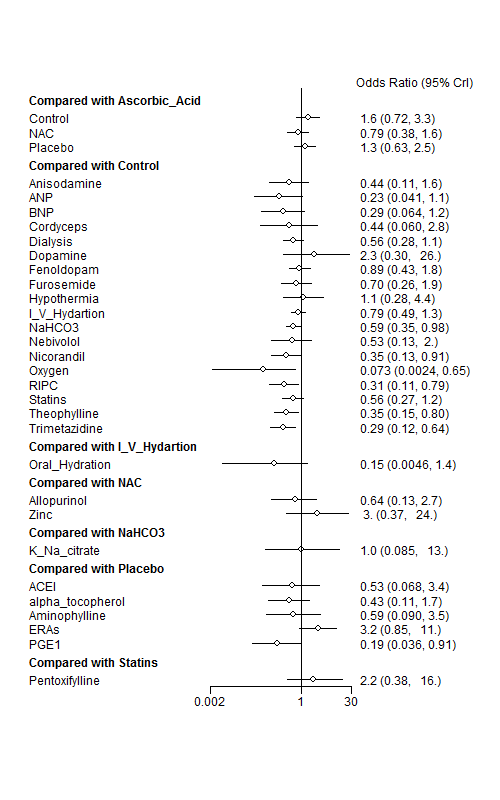


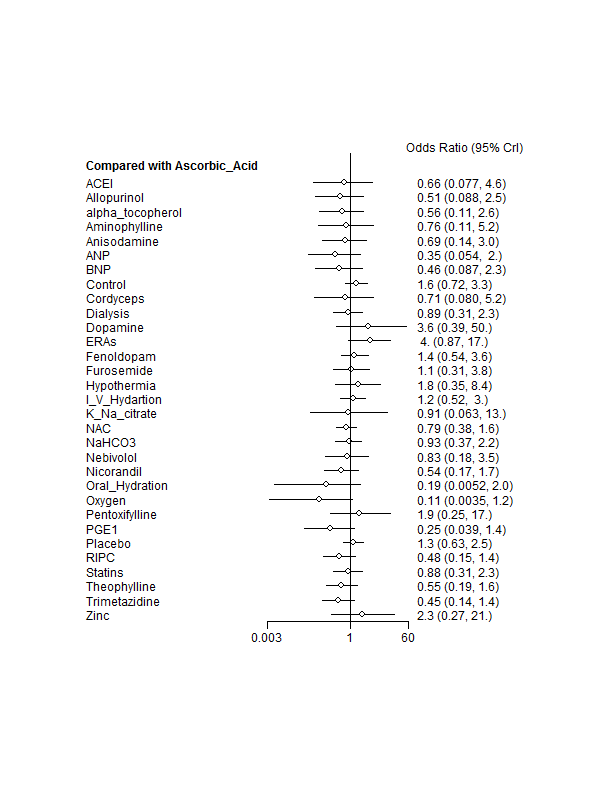

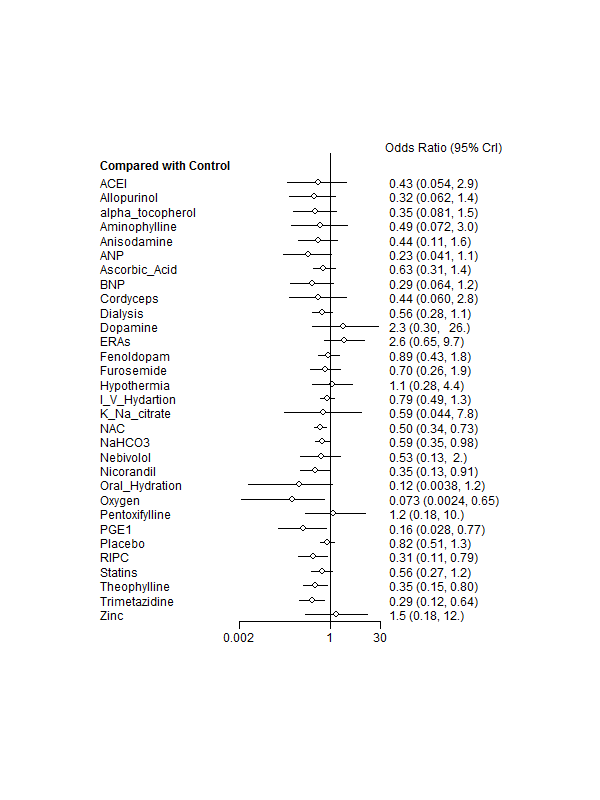

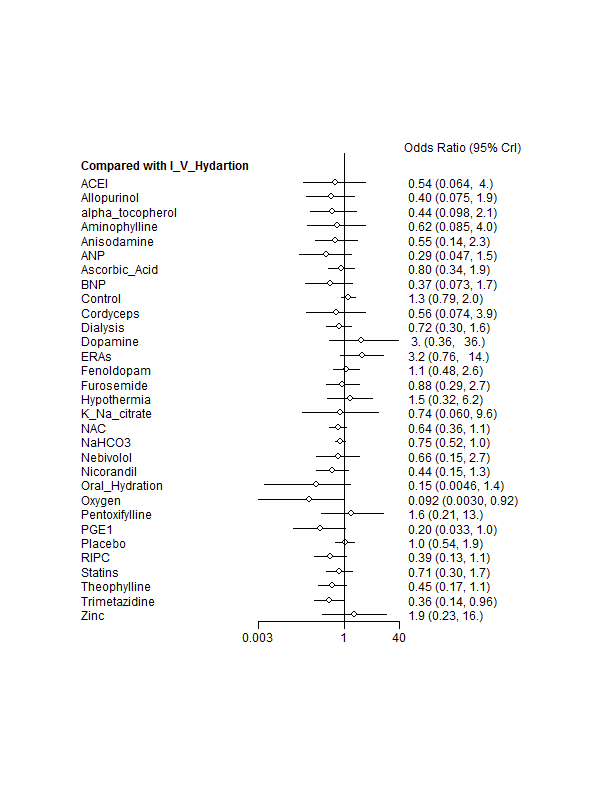

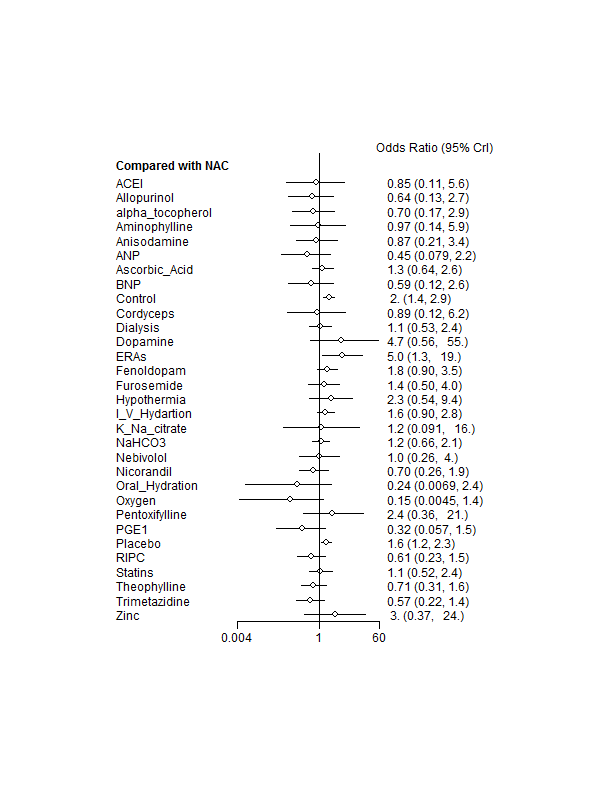

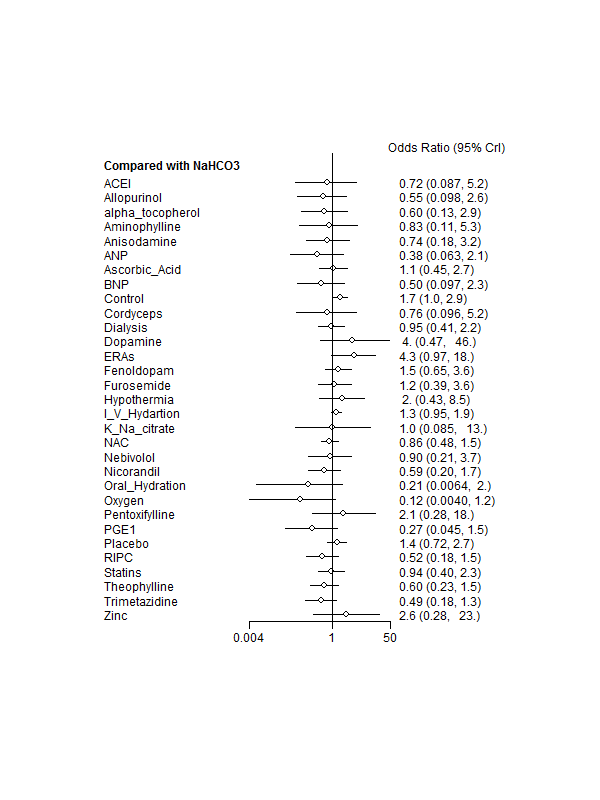

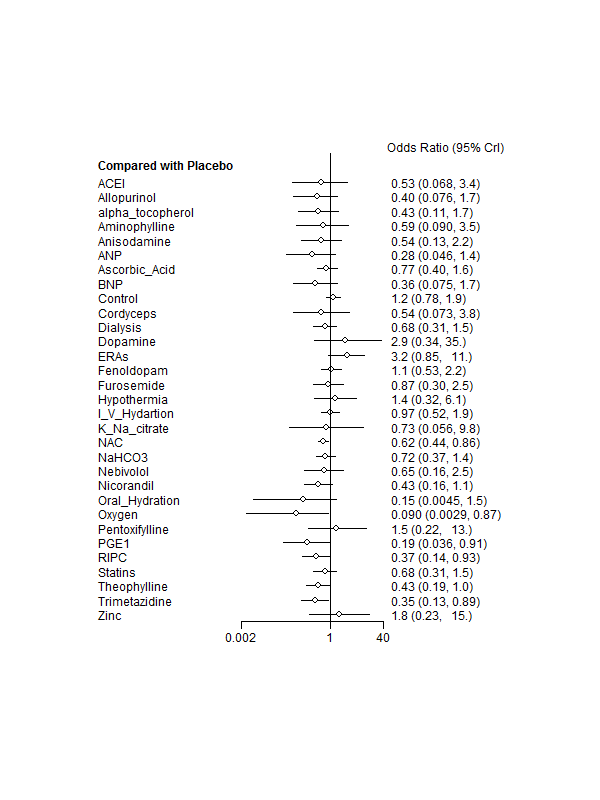

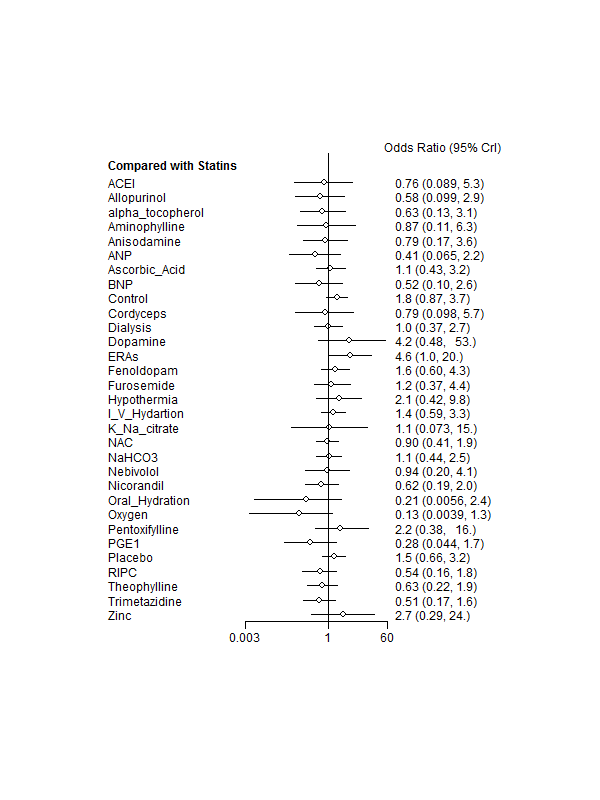

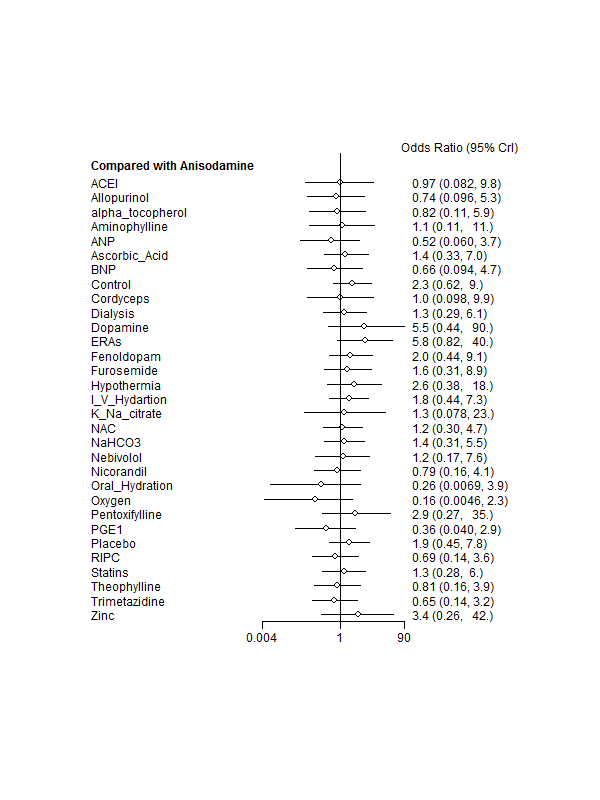

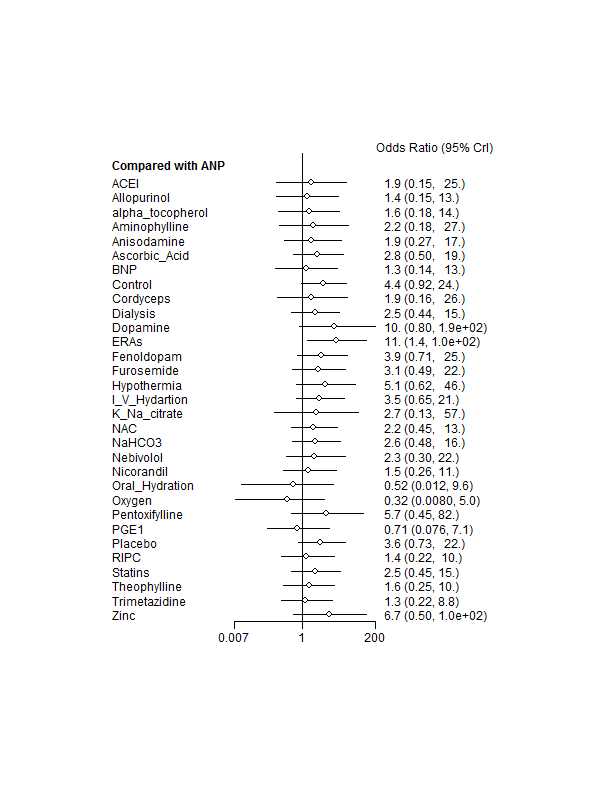

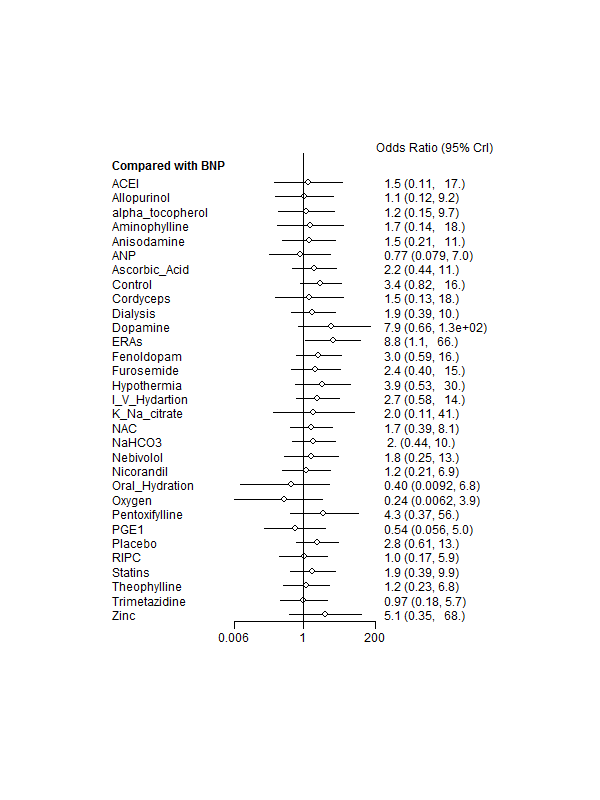

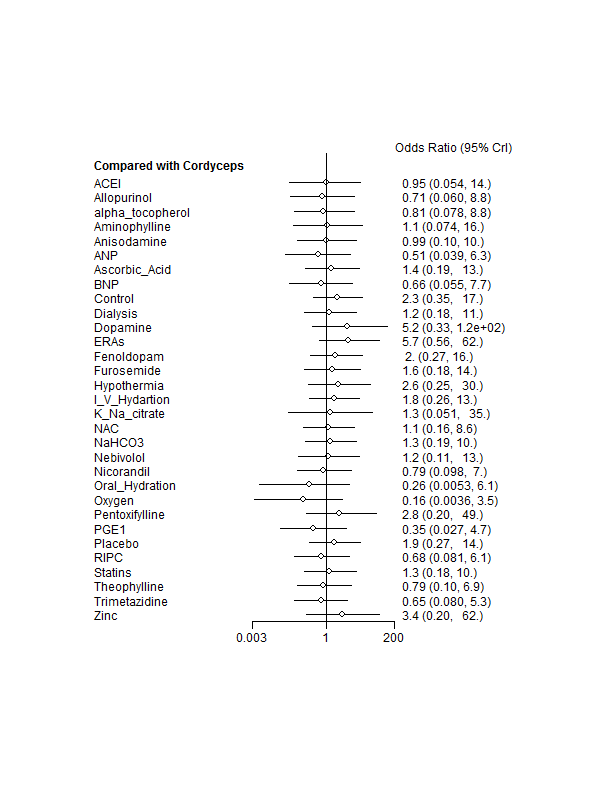

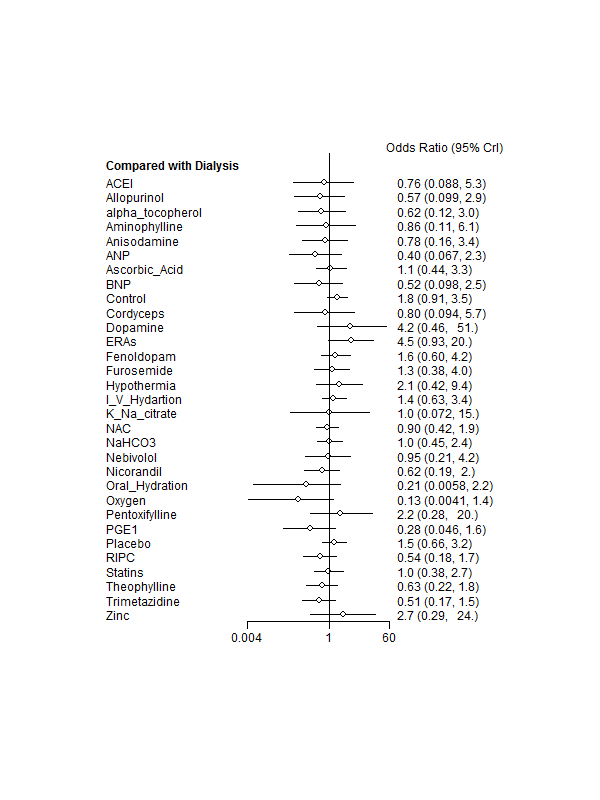

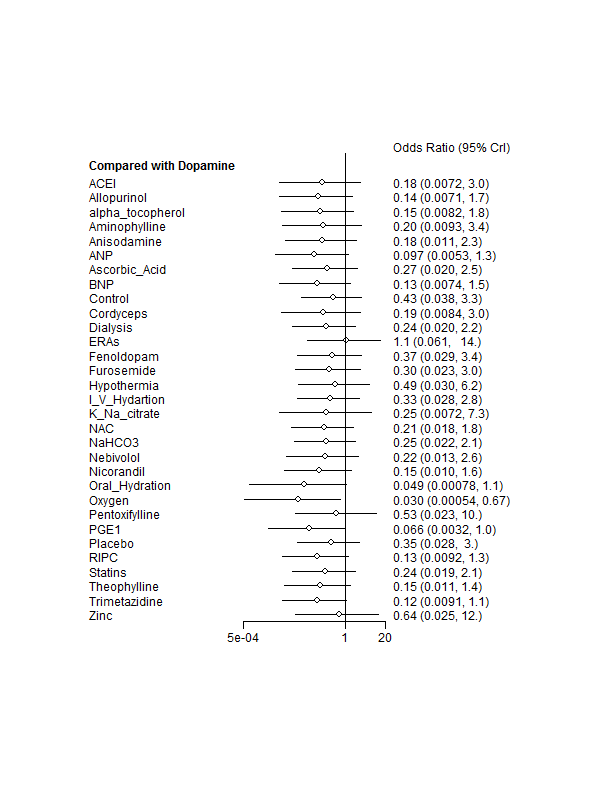

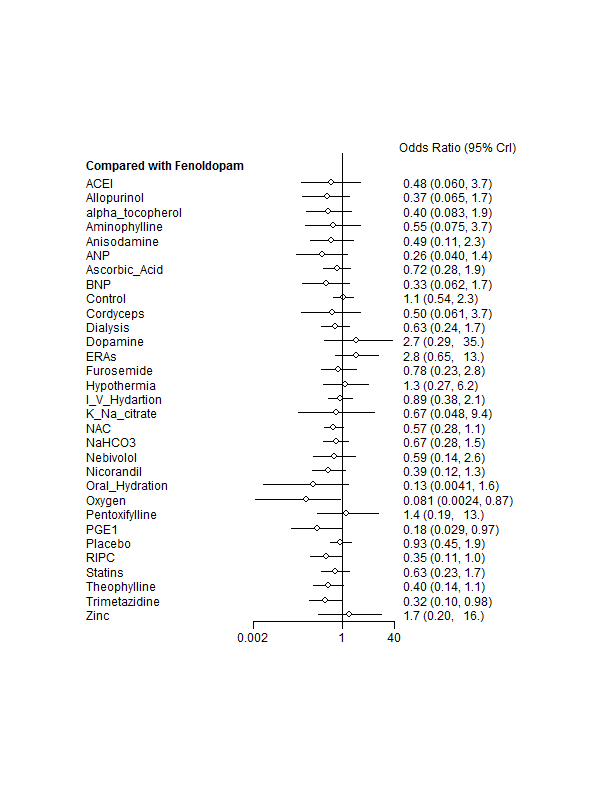

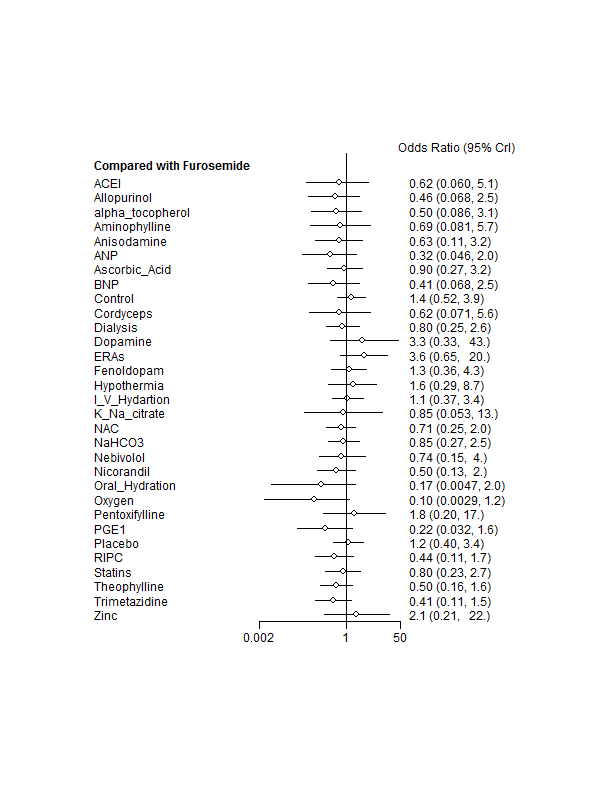

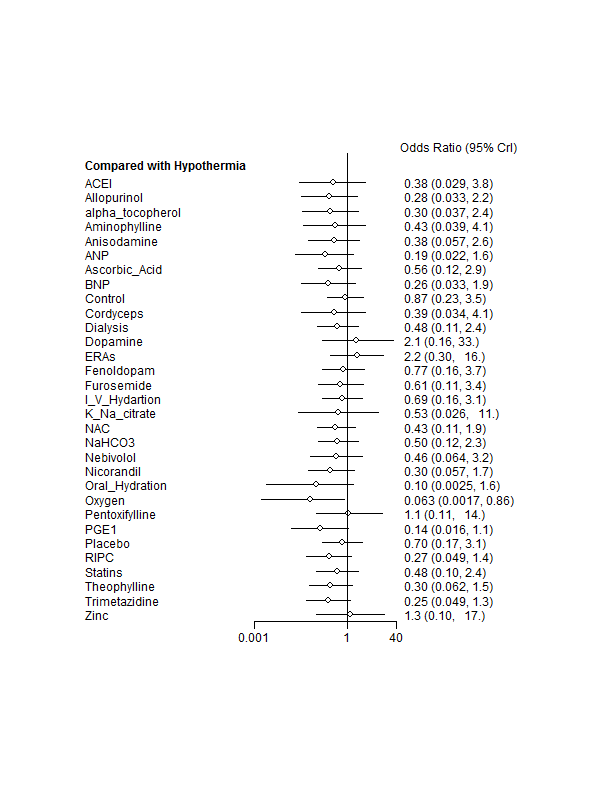

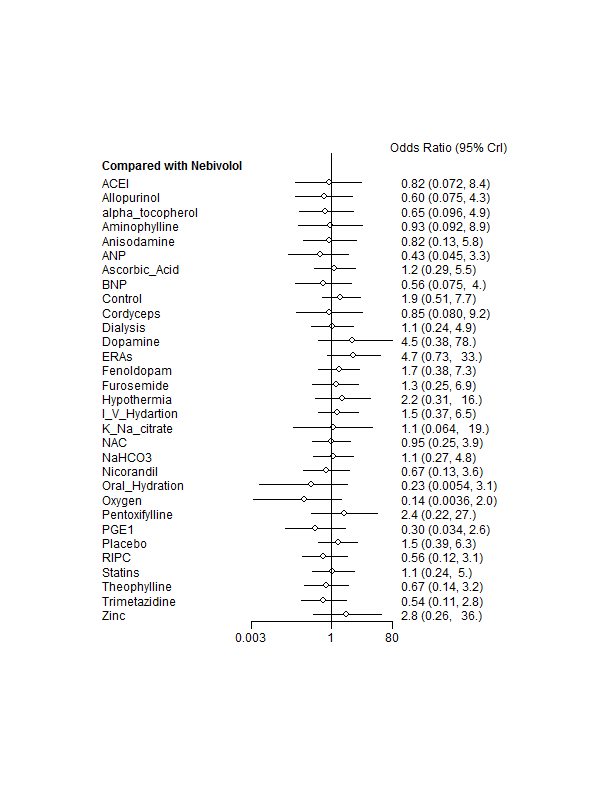

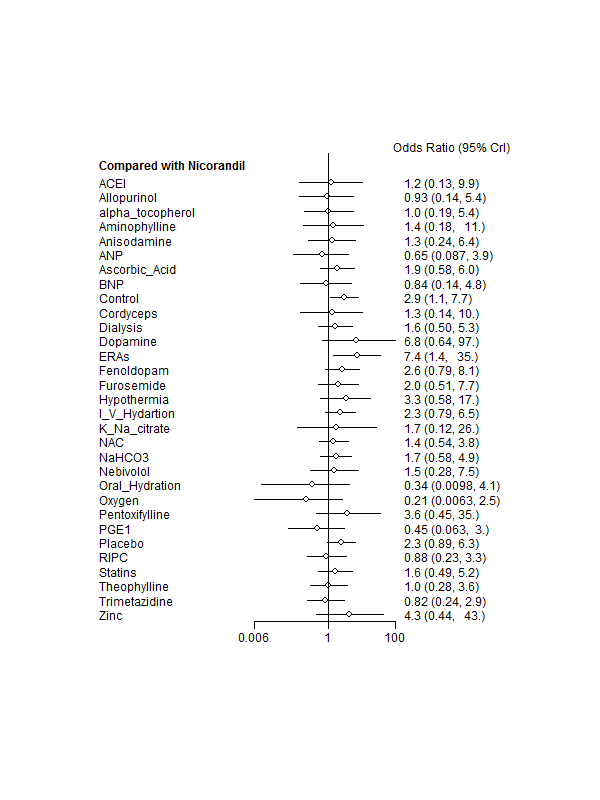

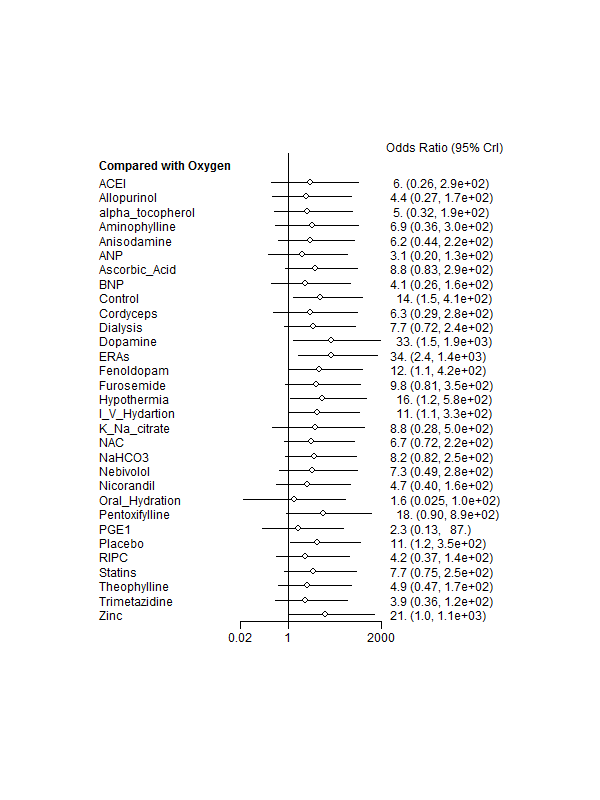

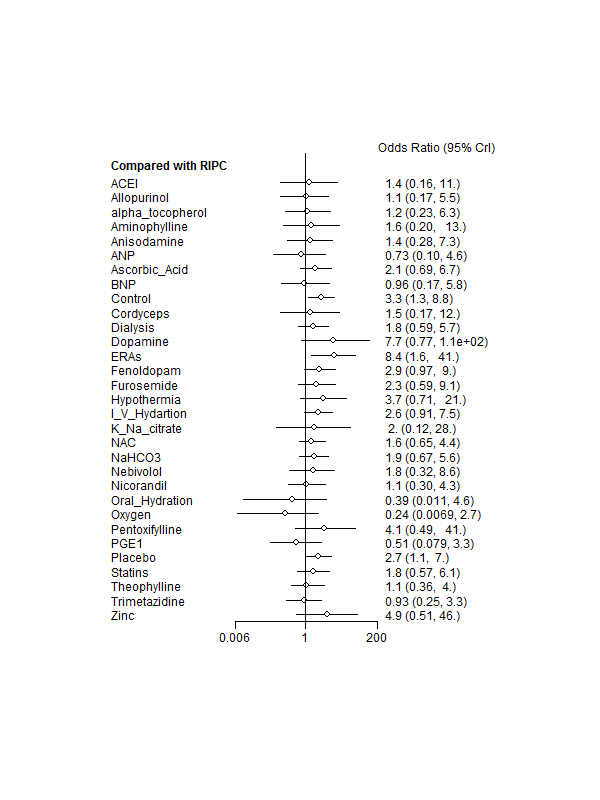


Figure 5 Gelman and Rubin's convergence diagnostics
