# Supplementary material for: Effectiveness of contrast-associated acute kidney injury prevention methods; a systematic review and network meta-analysis
Source: BMC Nephrol. 2018 Nov 13;19:323. doi: 10.1186/s12882-018-1113-0 (PMC6234687; doi:10.1186/s12882-018-1113-0)

**Supplement:**

**Results from Analysis 4 (60 RCTs with normal baseline Renal Profile)**

Number of Studies: 60 RCTs (see Manuscript)

Figures and Tables:

1. Network Diagram

2. Tables:

A. Network Characteristics

B. Interventions Characteristics

C Direct comparisons characteristics

3. Rankogram

4. Ranking and probability of being the best (included in the main manuscript)

5. Forest Plot

| Software | Spec | Convergence | Analysis |
| --- | --- | --- | --- |
| Netmetaxl / WinBUGS14 version 1.4.3 | Burn 5000  Model 10000 | good convergence (FE MC error 5% of the SD) | Random Effects (Vague)  Random Effects (Informative) |

Figure 1 Network Diagram


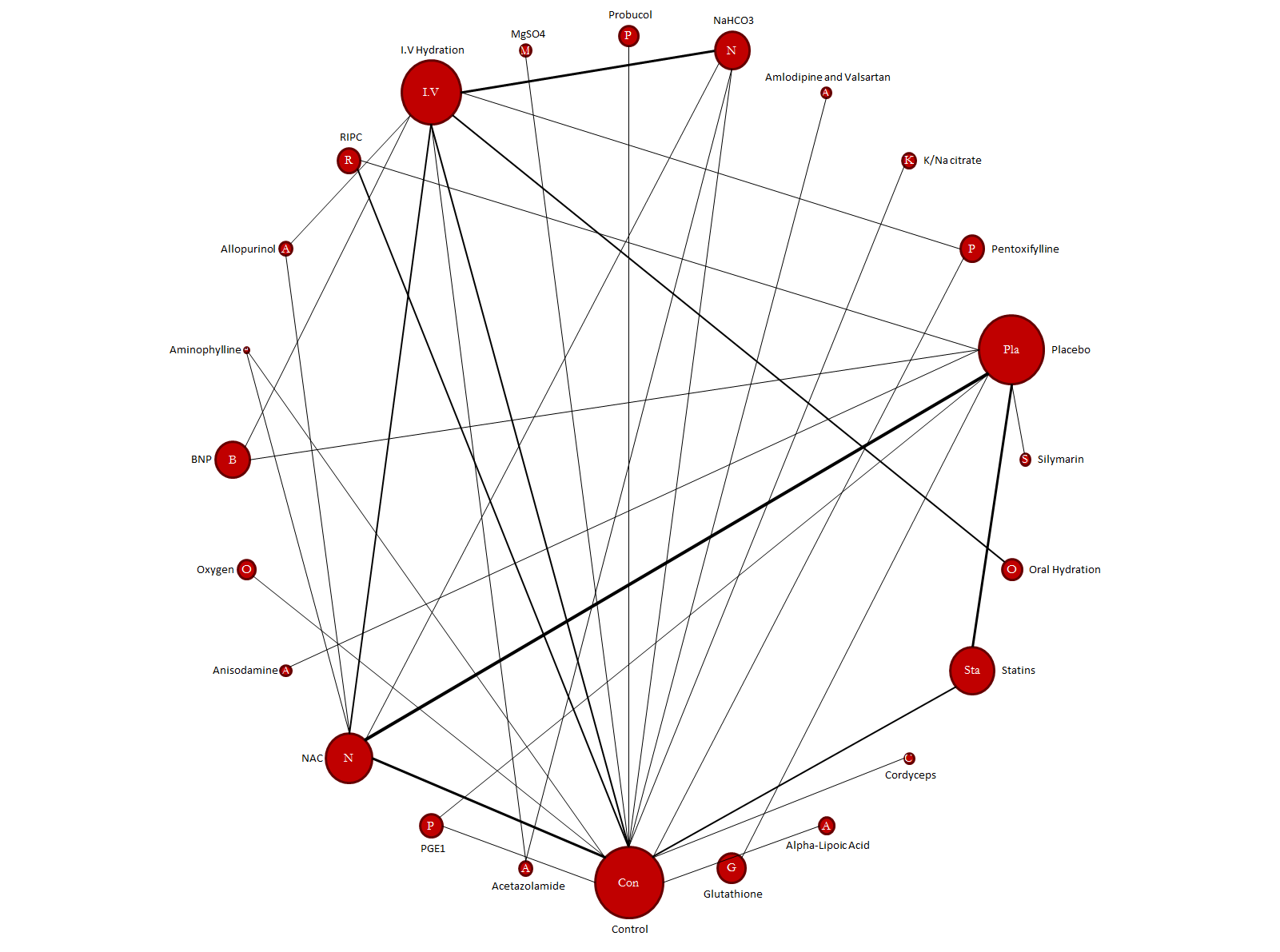


Table 1 Network Characteristics

| **Characteristic** | **Number** |
| --- | --- |
| **Number of Interventions** | 24 |
| **Number of Studies** | 60 |
| **Total Number of Patients in Network** | 13,023 |
| **Total Number of Events in Network** | 1,501 |
| **Total Possible Pairwise Comparisons** | 276 |
| **Total Number Pairwise Comparisons With Direct Data** | 34 |
| **Number of Two-arm Studies** | 54 |
| **Number of Multi-Arms Studies** | 6 |
| **Number of Studies With No Zero Events** | 53 |
| **Number of Studies With At Least One Zero Event** | 7 |
| **Number of Studies with All Zero Events** | 1 |

Table 2 Interventions Characteristics

| **Treatment** | **# Studies** | **# Events** | **# Patients** | **Aggregate Rate** |
| --- | --- | --- | --- | --- |
| **I.V Hydration** | 16 | 248 | 1984 | 0.1250 |
| **Statins** | 8 | 65 | 1079 | 0.0602 |
| **NAC** | 17 | 156 | 1183 | 0.1319 |
| **NaHCO3** | 6 | 73 | 699 | 0.1044 |
| **PGE1** | 3 | 18 | 271 | 0.0664 |
| **MgSO4** | 1 | 9 | 62 | 0.1452 |
| **Pentoxifylline** | 3 | 24 | 328 | 0.0732 |
| **Placebo** | 20 | 281 | 2297 | 0.1223 |
| **Control** | 27 | 453 | 2531 | 0.1790 |
| **Allopurinol** | 2 |  | 95 | 0.0000 |
| **BNP** | 3 | 46 | 638 | 0.0721 |
| **Probucol** | 2 | 12 | 198 | 0.0606 |
| **Oxygen** | 1 | 32 | 172 | 0.1860 |
| **Amlodipine and Valsartan** | 1 | 8 | 45 | 0.1778 |
| **K/Na citrate** | 1 | 4 | 100 | 0.0400 |
| **Alpha-Lipoic Acid** | 2 | 6 | 139 | 0.0432 |
| **Oral Hydration** | 3 | 13 | 206 | 0.0631 |
| **Anisodamine** | 1 | 4 | 60 | 0.0667 |
| **RIPC** | 4 | 17 | 295 | 0.0576 |
| **Glutathione** | 1 | 21 | 414 | 0.0507 |
| **Aminophylline** | 1 |  | 15 | 0.0000 |
| **Acetazolamide** | 1 | 5 | 94 | 0.0532 |
| **Cordyceps** | 1 | 4 | 49 | 0.0816 |
| **Silymarin** | 1 | 2 | 69 | 0.0290 |

Table 3 Direct comparisons characteristics

| **Comparison** | **# Studies** | **# Patients** | **# Events** |
| --- | --- | --- | --- |
| **I.V Hydartion vs. Oral Hydration** | 3 | 407 | 26 |
| **NAC vs. Placebo** | 9 | 1,682 | 215 |
| **Statins vs. Placebo** | 5 | 1,142 | 100 |
| **Control vs. Alpha-Lipoic Acid** | 2 | 280 | 16 |
| **Pentoxifylline vs. Control** | 2 | 461 | 46 |
| **MgSO4 vs. Control** | 1 | 126 | 26 |
| **Control vs. RIPC** | 3 | 386 | 29 |
| **Placebo vs. RIPC** | 1 | 202 | 16 |
| **I.V Hydartion vs. Control** | 3 | 924 | 200 |
| **NAC vs. Control** | 5 | 431 | 70 |
| **NAC vs. Aminophylline** | 1 | 30 | 0 |
| **Control vs. Aminophylline** | 1 | 30 | 4 |
| **I.V Hydartion vs. NaHCO3** | 6 | 1,414 | 148 |
| **I.V Hydartion vs. NAC** | 3 | 245 | 62 |
| **I.V Hydartion vs. Allopurinol** | 2 | 185 | 31 |
| **NAC vs. Allopurinol** | 2 | 150 | 18 |
| **Statins vs. Control** | 3 | 1,044 | 84 |
| **Control vs. Probucol** | 2 | 409 | 50 |
| **PGE1 vs. Control** | 2 | 226 | 16 |
| **I.V Hydartion vs. BNP** | 2 | 1,128 | 113 |
| **NaHCO3 vs. Control** | 1 | 300 | 59 |
| **Control vs. K/Na citrate** | 1 | 202 | 25 |
| **PGE1 vs. Placebo** | 1 | 330 | 53 |
| **Control vs. Oxygen** | 1 | 348 | 105 |
| **Control vs. Amlodipine and Valsartan** | 1 | 90 | 11 |
| **I.V Hydartion vs. Acetazolamide** | 1 | 190 | 21 |
| **NaHCO3 vs. Acetazolamide** | 1 | 190 | 9 |
| **Placebo vs. Silymarin** | 1 | 143 | 10 |
| **Placebo vs. Glutathione** | 1 | 825 | 41 |
| **Placebo vs. Anisodamine** | 1 | 126 | 17 |
| **I.V Hydartion vs. Pentoxifylline** | 1 | 199 | 12 |
| **NAC vs. NaHCO3** | 1 | 100 | 26 |
| **Placebo vs. BNP** | 1 | 149 | 36 |
| **Control vs. Cordyceps** | 1 | 100 | 10 |

Figure 2 Rankogram: ranking the interventions for the probability of being the best, the interventions are colour coded; the first column represent the chance of being first best and 2nd column is the chance of being 2nd best and so on. The overall numerical value is presented in table 4


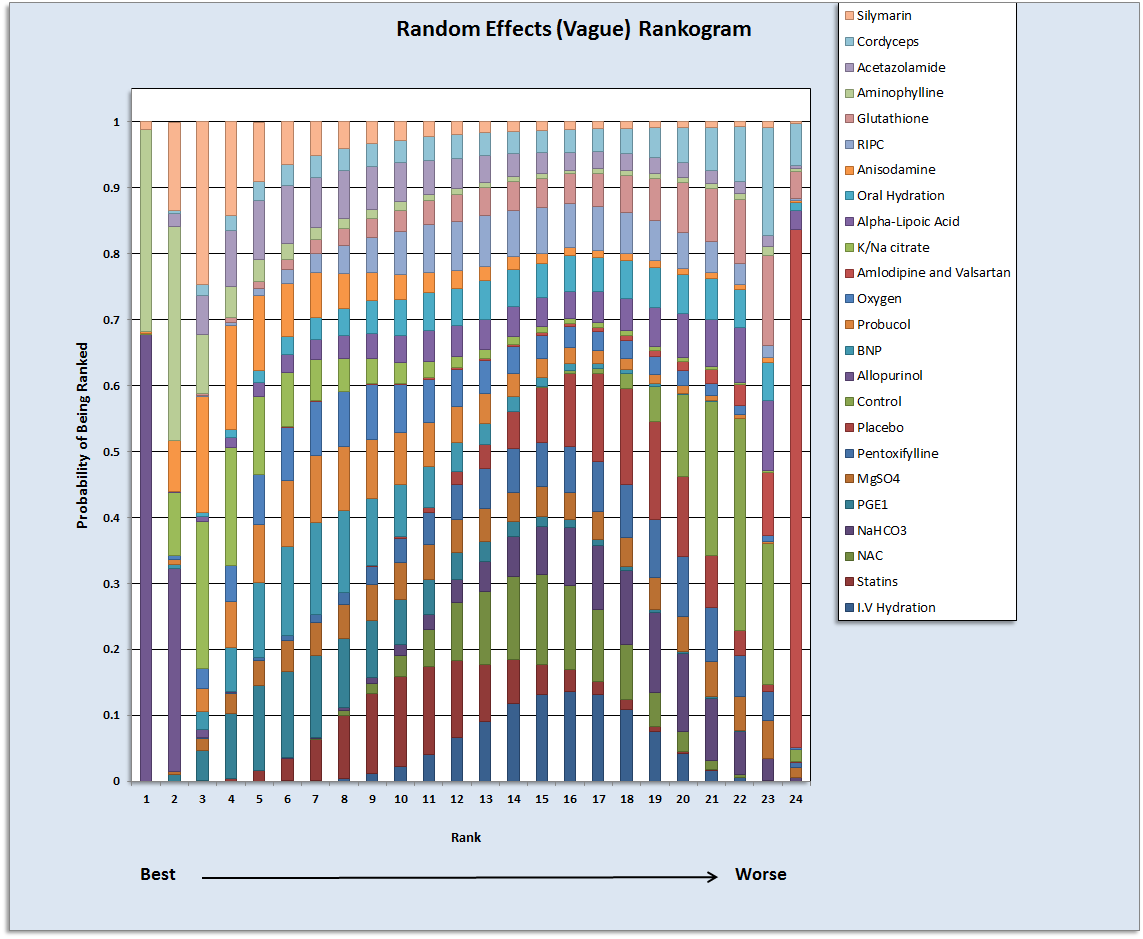


Table 4 Interventions ranking the treatments names column follow the league table (which arranges the presentation of summary estimates by ranking the treatments in order of most pronounced impact on the outcome under consideration) the numerical values represents the cumulative results of the probability of being best in which the highest score is 1 or 100% (see Rankogram)

| **Treatment** | **SUCRA** | **Treatment** | **SUCRA** |
| --- | --- | --- | --- |
| **Allopurinol** | 0.9852 | **RIPC** | 0.4204 |
| **Aminophylline** | 0.8601 | **Oral Hydration** | 0.3962 |
| **K/Na citrate** | 0.7858 | **NAC** | 0.3954 |
| **Silymarin** | 0.7731 | **I.V Hydration** | 0.3699 |
| **Anisodamine** | 0.7417 | **Alpha-Lipoic Acid** | 0.3545 |
| **PGE1** | 0.7006 | **Cordyceps** | 0.3398 |
| **BNP** | 0.689 | **Pentoxifylline** | 0.332 |
| **Probucol** | 0.6384 | **Glutathione** | 0.2993 |
| **Acetazolamide** | 0.6111 | **NaHCO3** | 0.2828 |
| **Oxygen** | 0.5887 | **Placebo** | 0.2774 |
| **Statins** | 0.5673 | **Control** | 0.1114 |
| **MgSo4** | 0.4479 | **Amlodipine and Valsartan** | 0.03205 |
| ***Analysis*** | **Random Effects (Vague)** | | |

Figure 3 Forest Plot


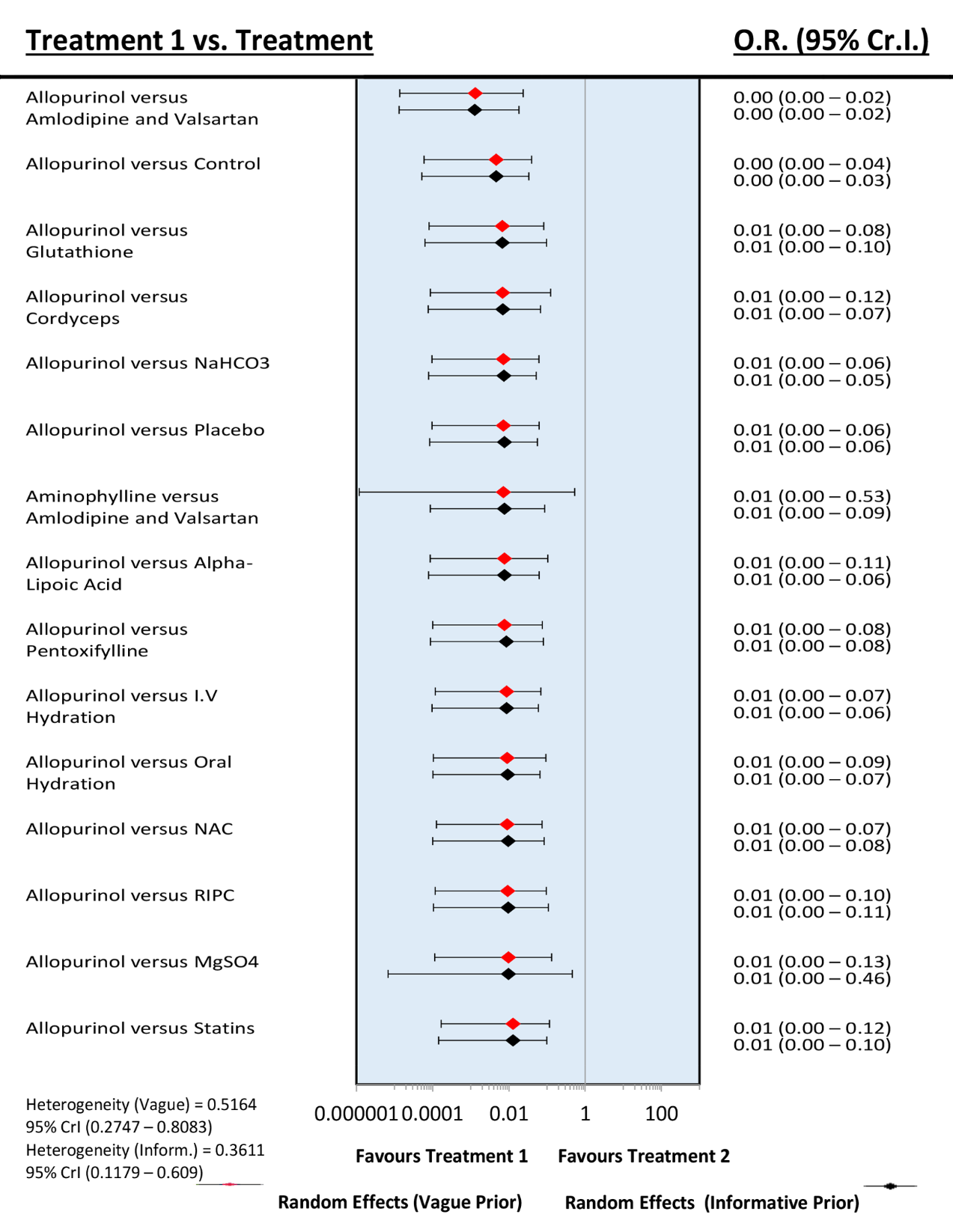


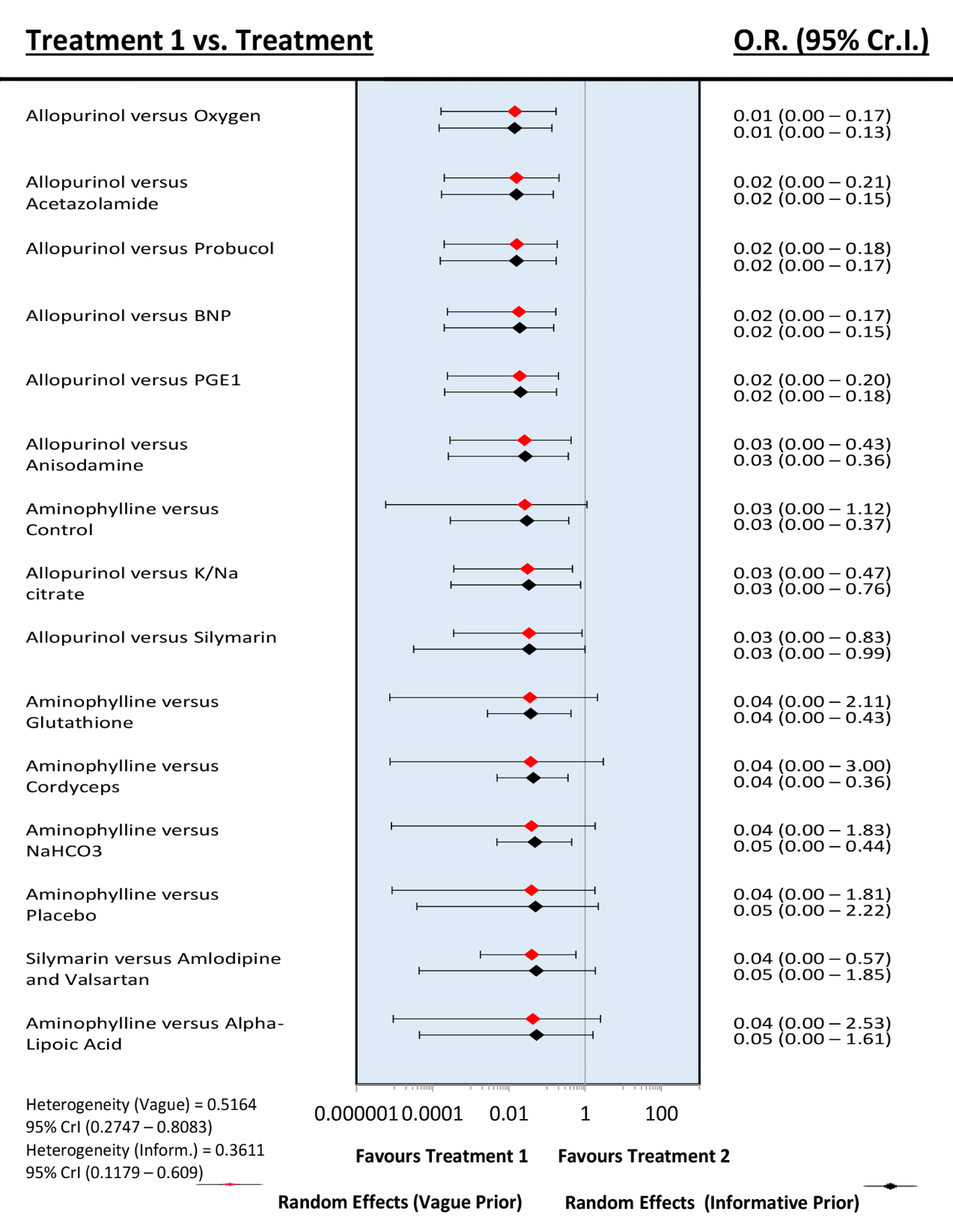


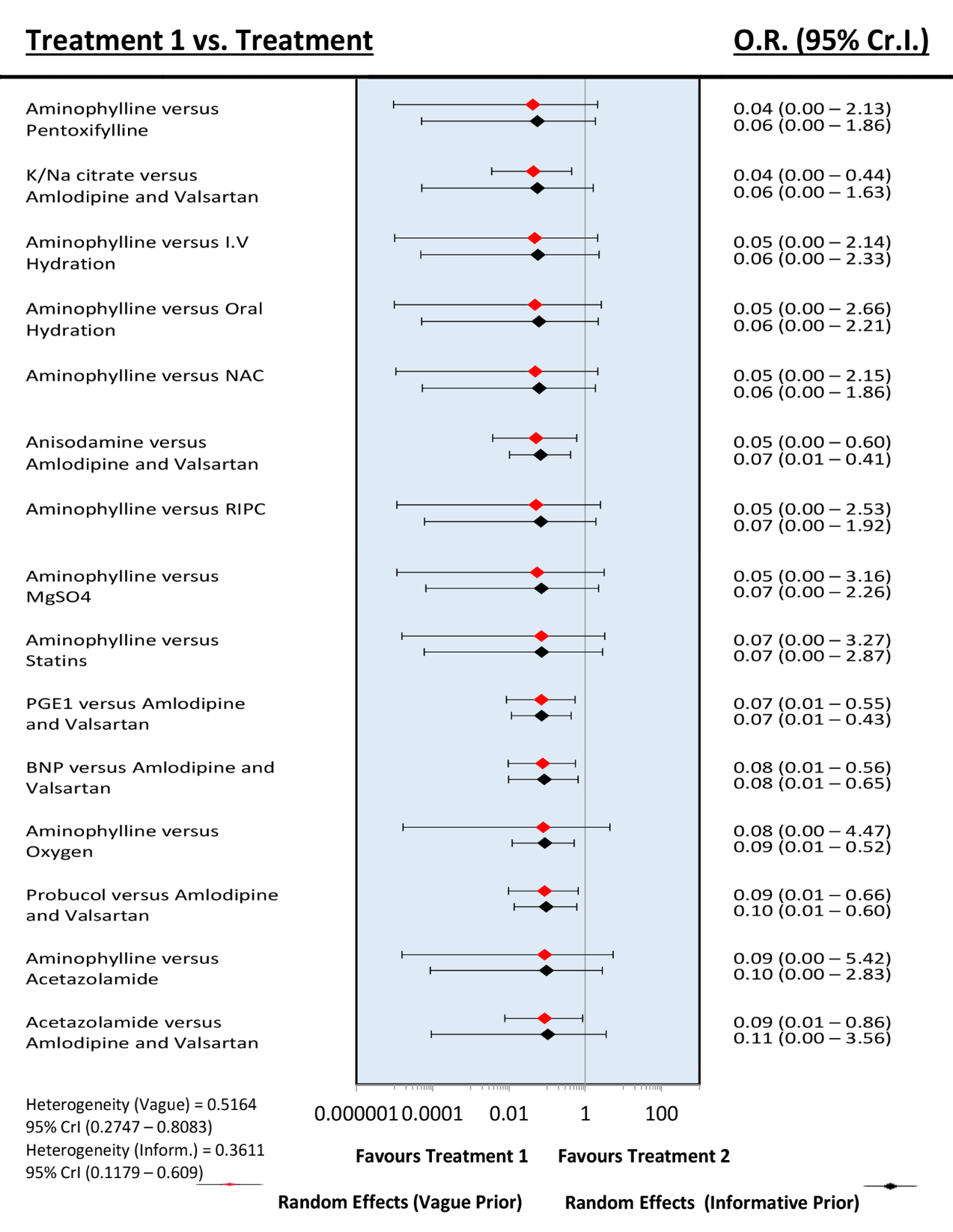


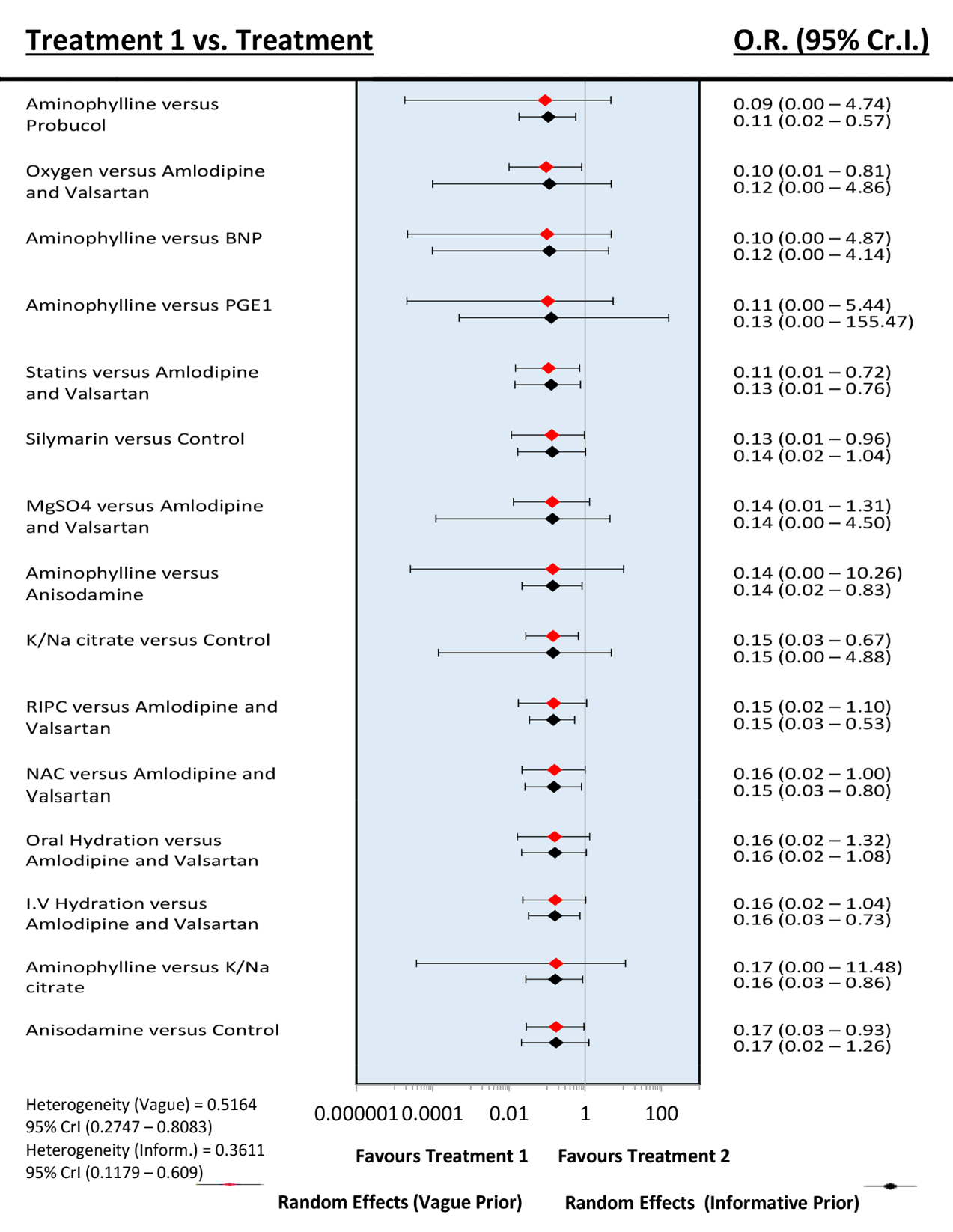


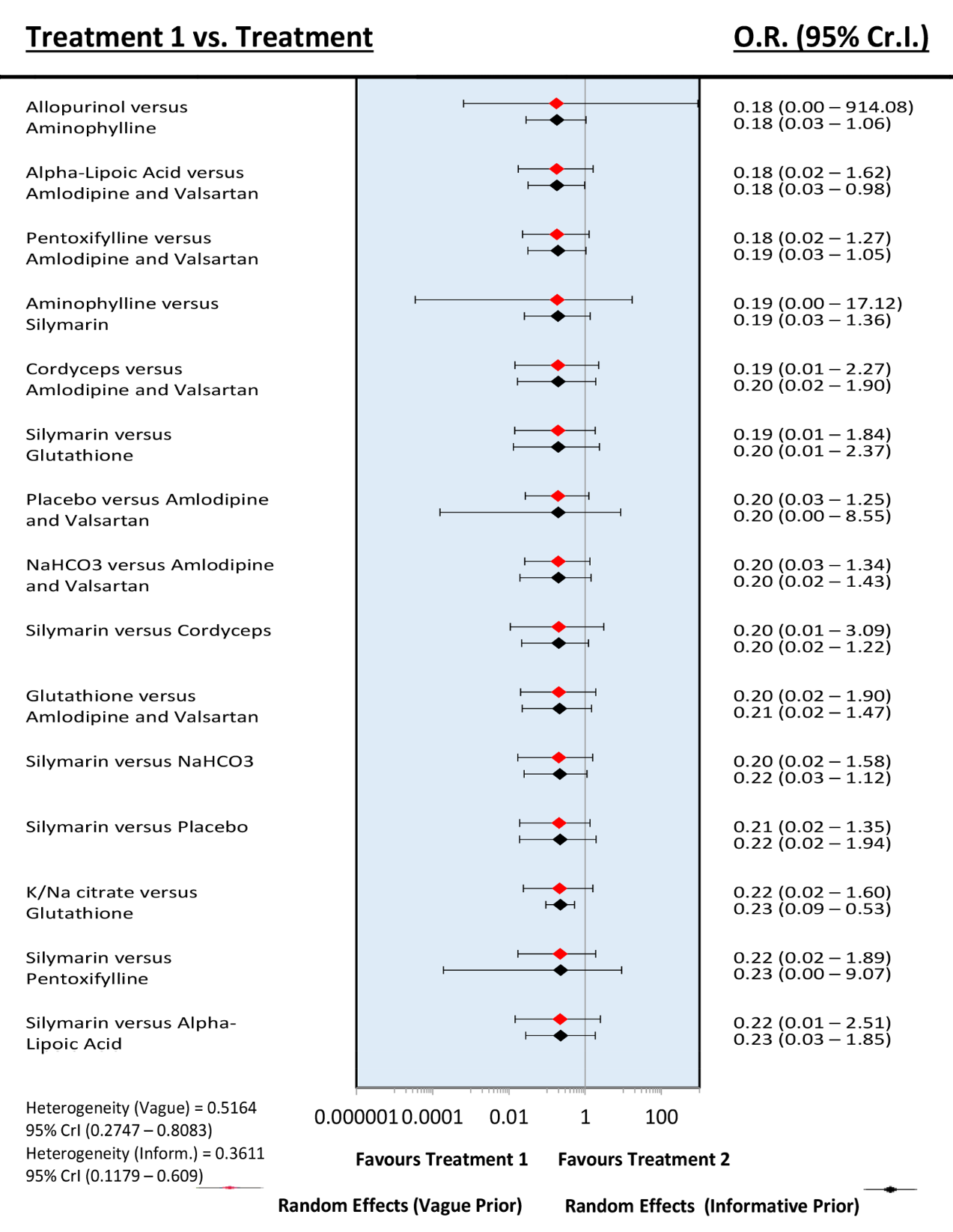


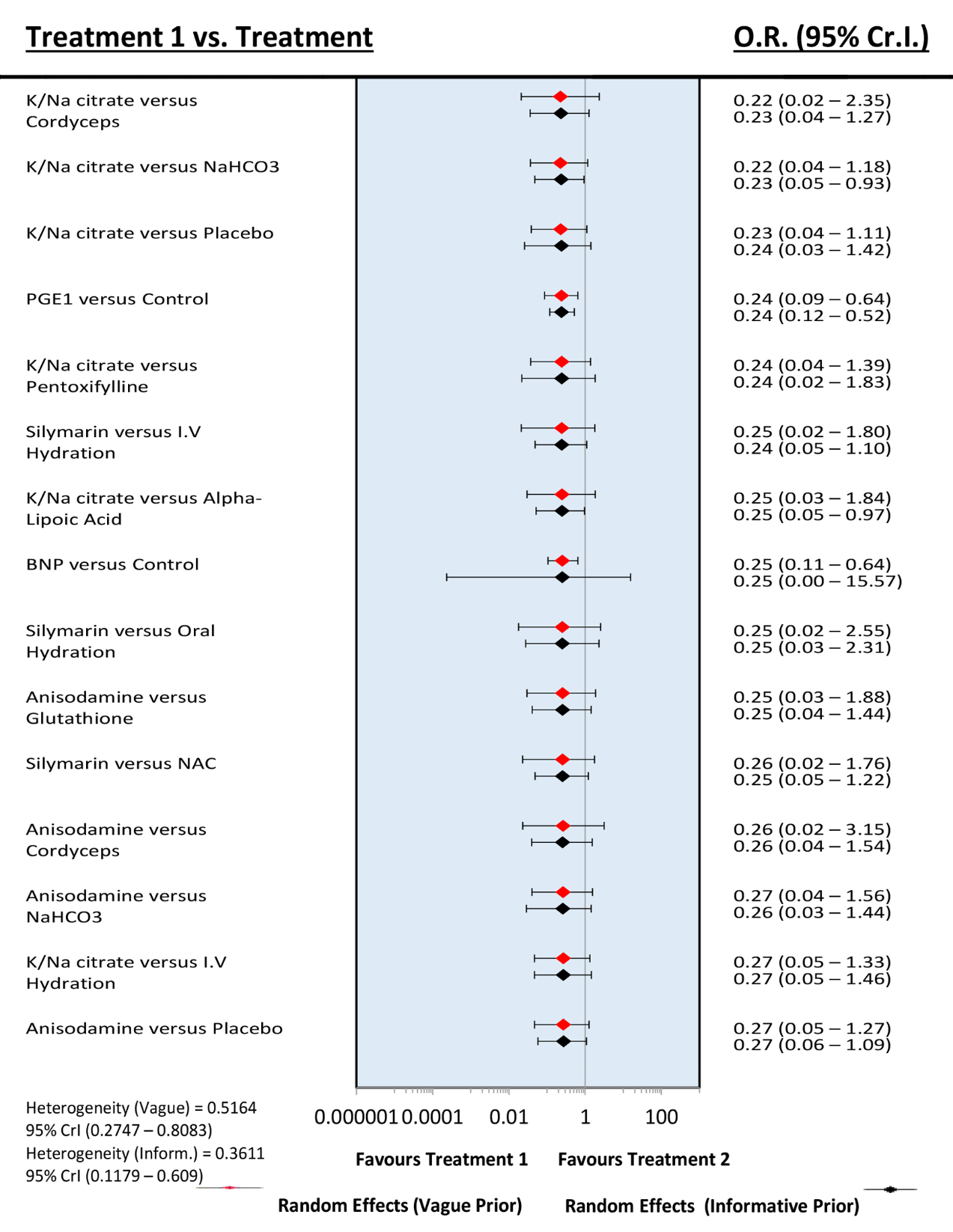


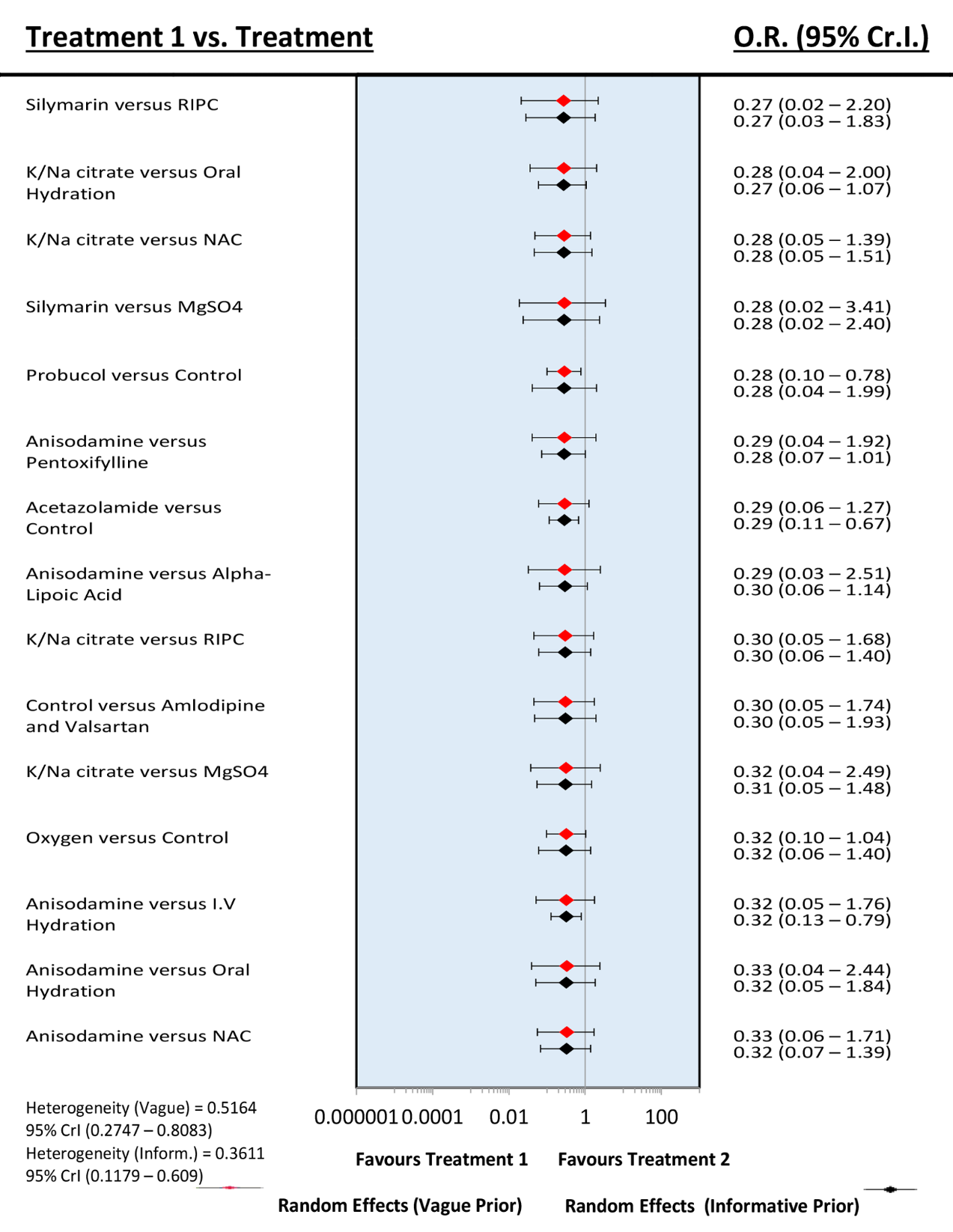


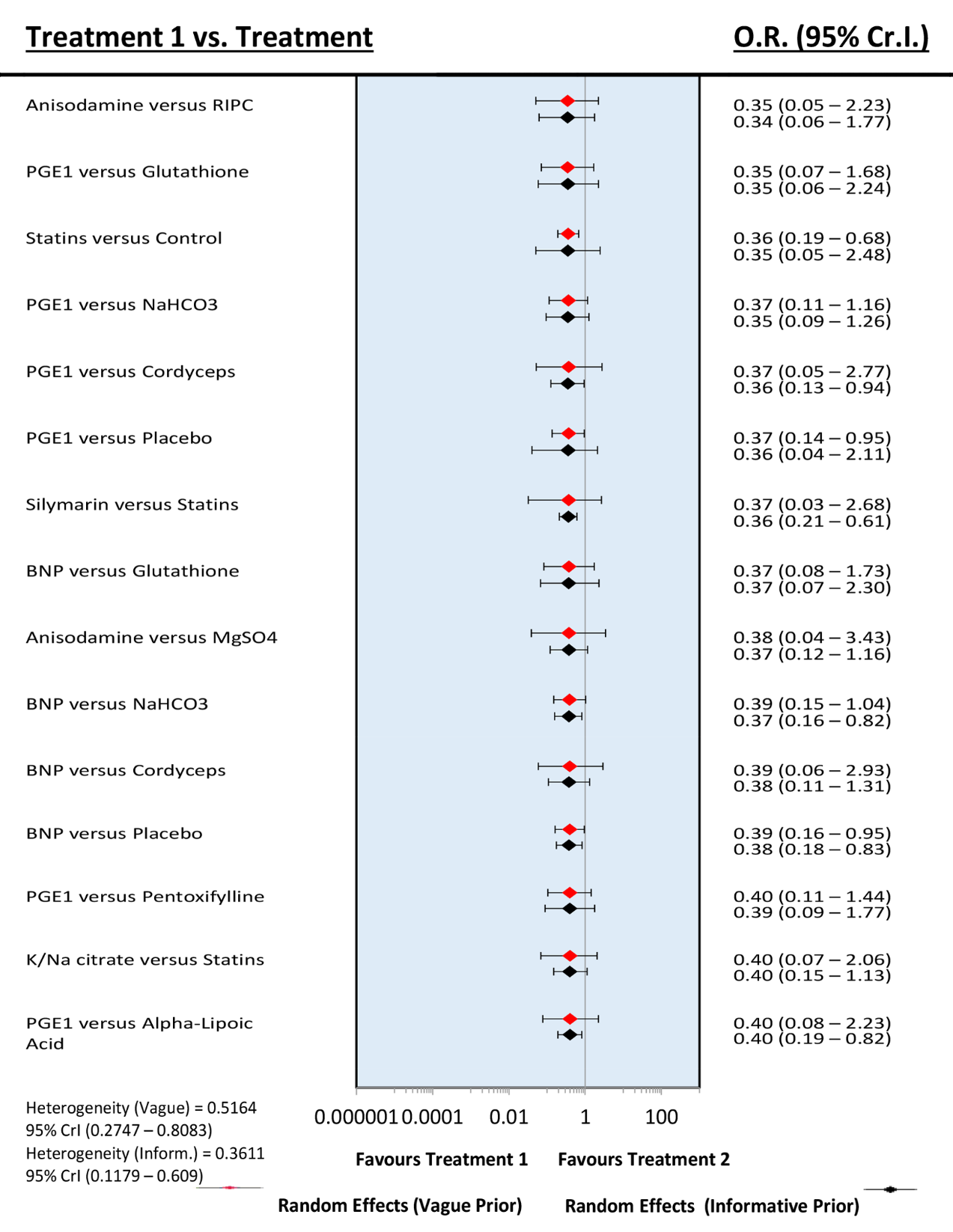


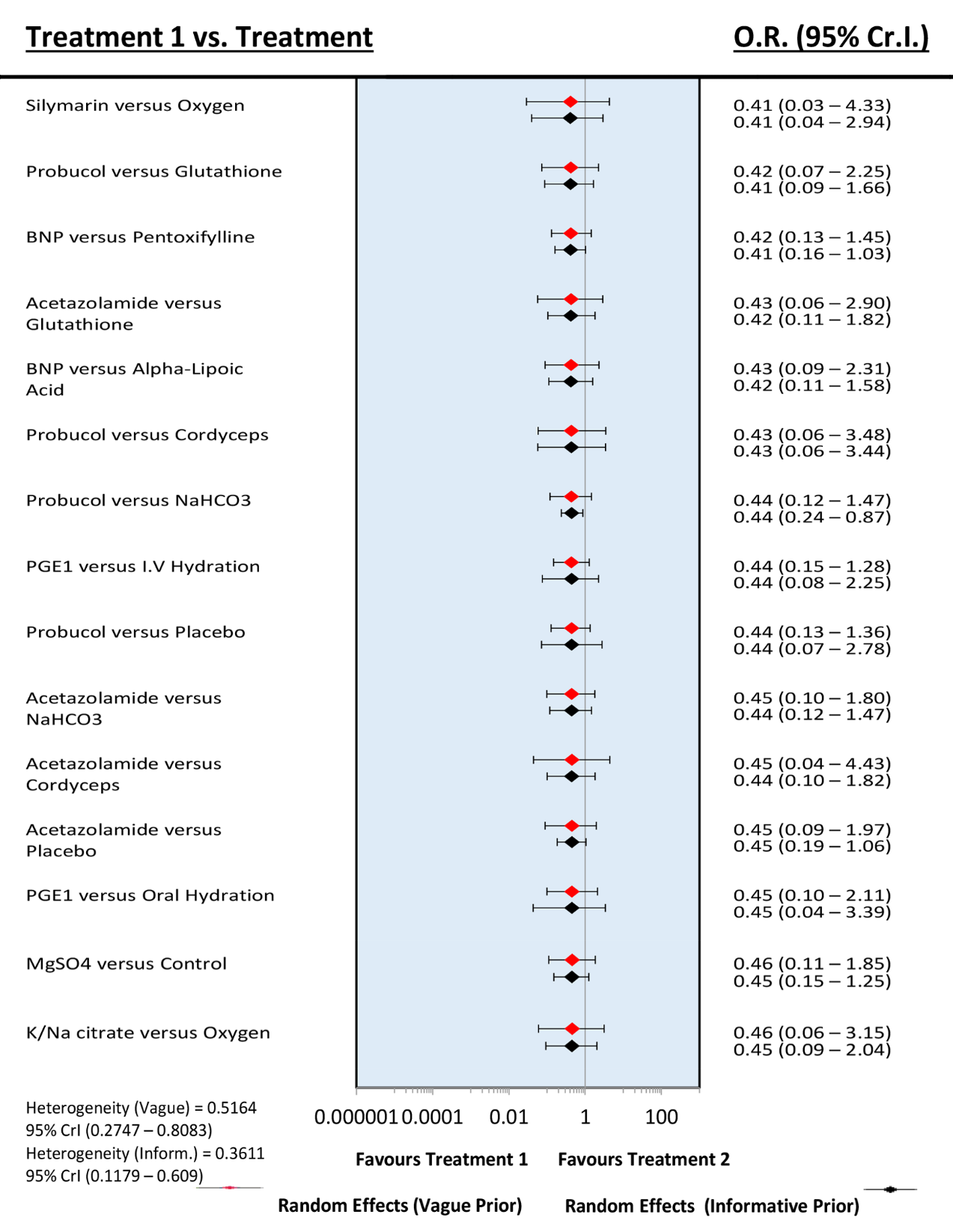


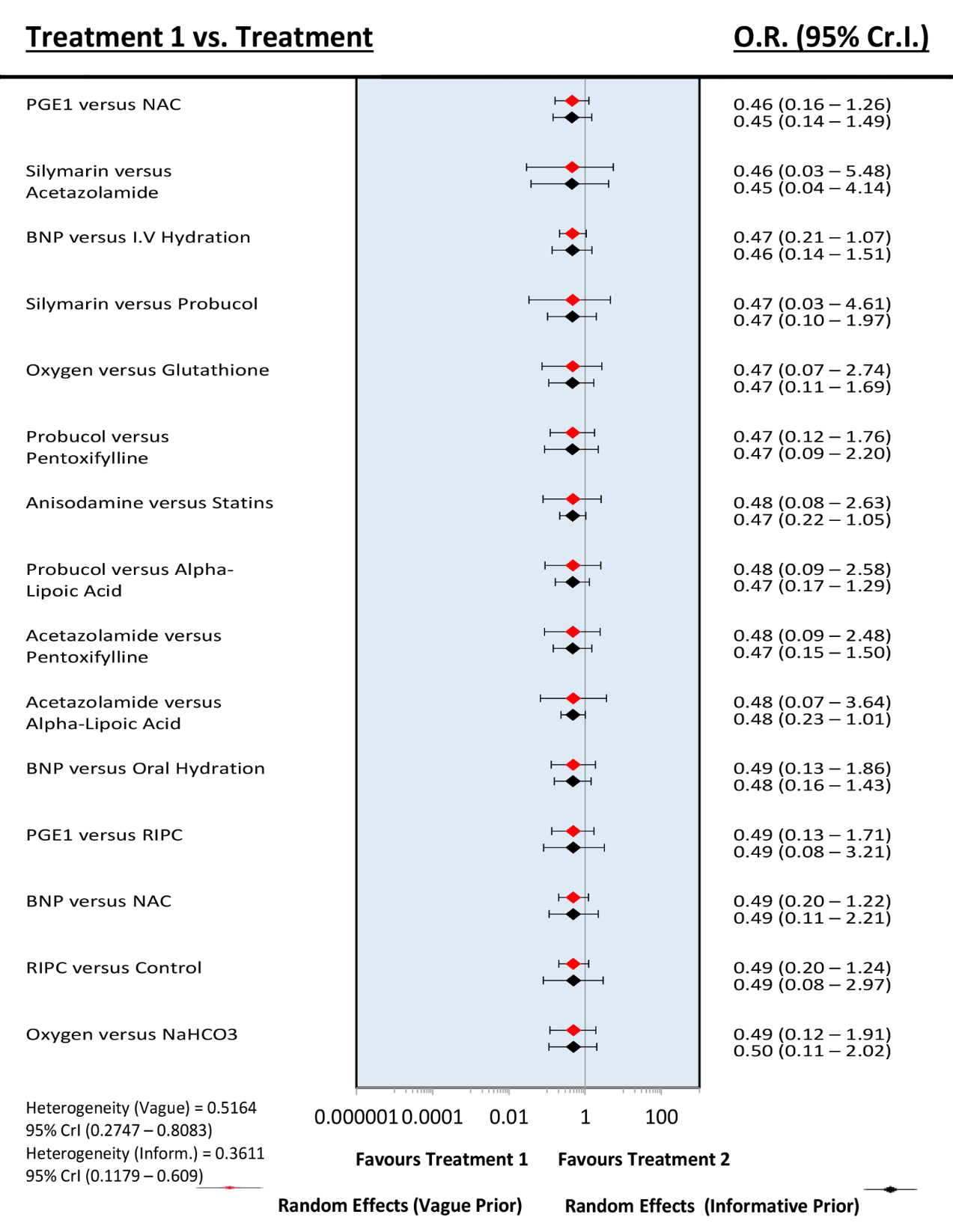


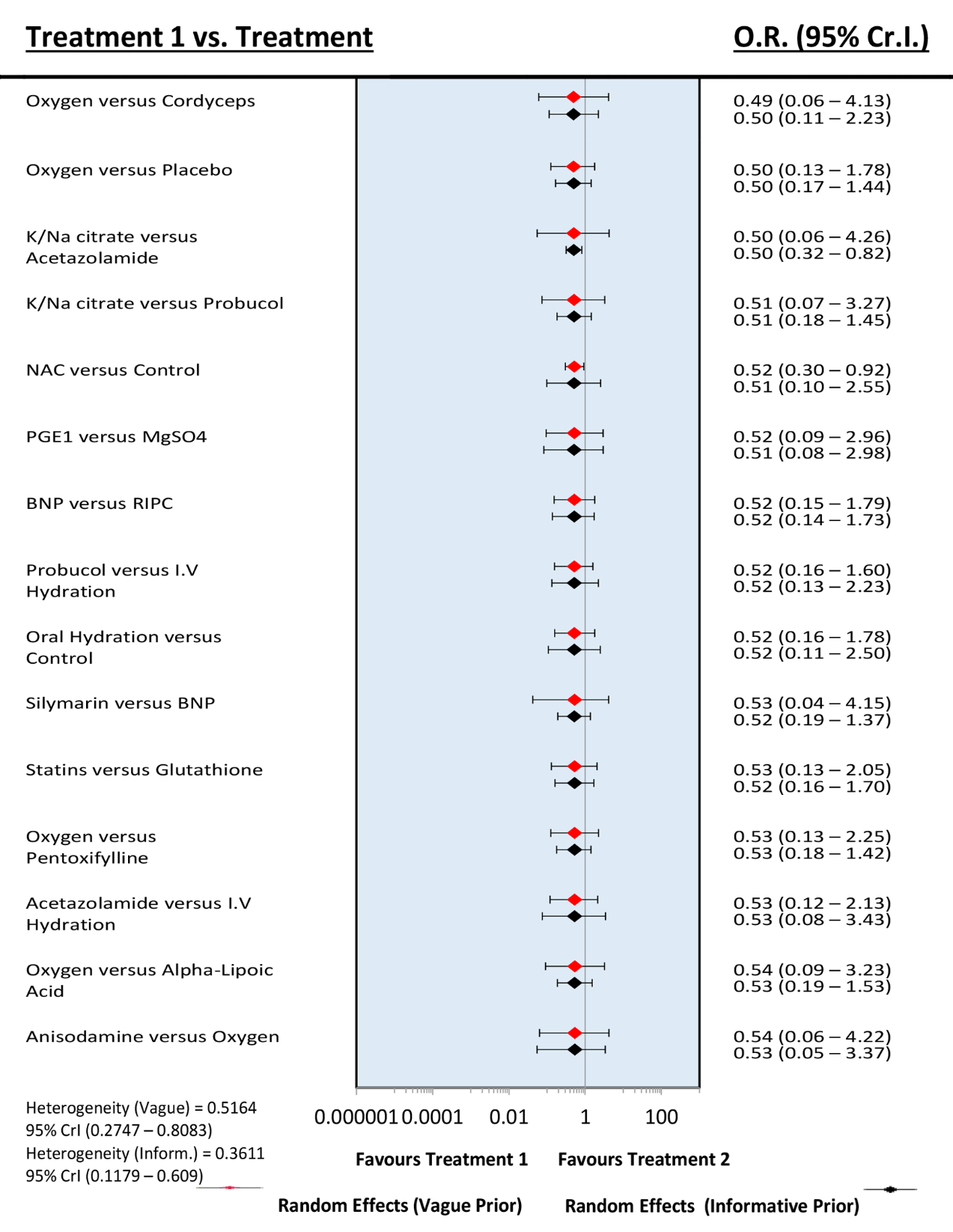


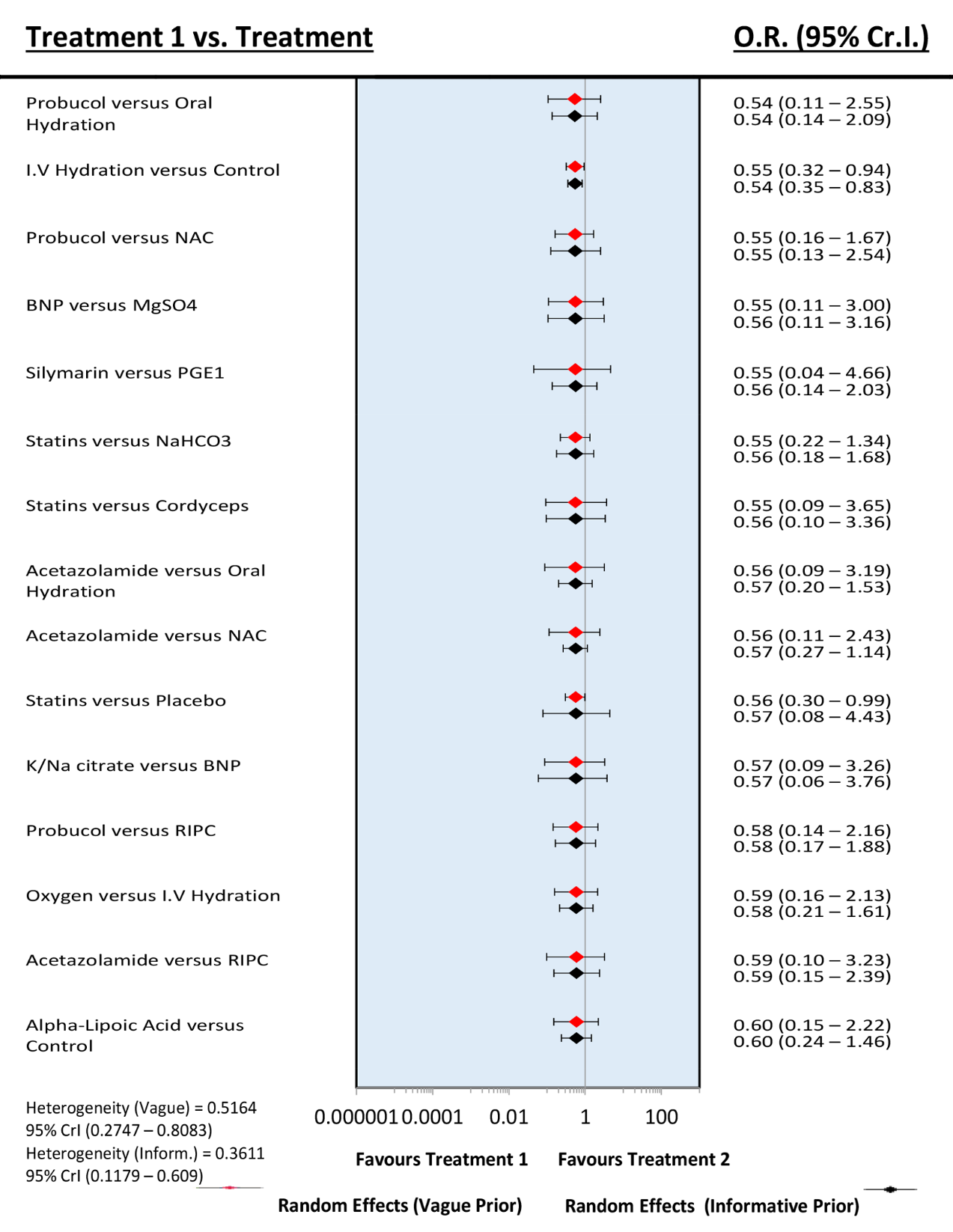


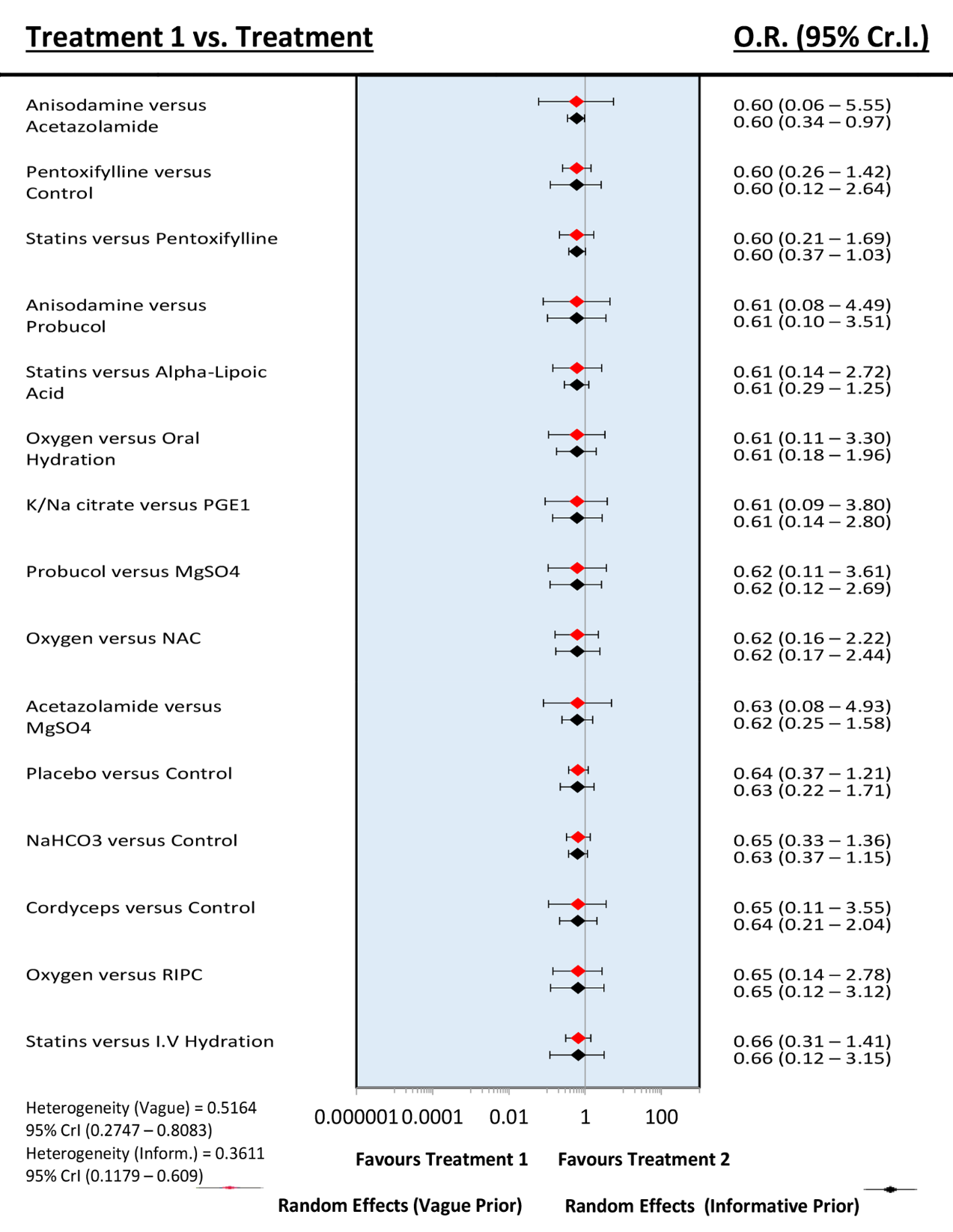


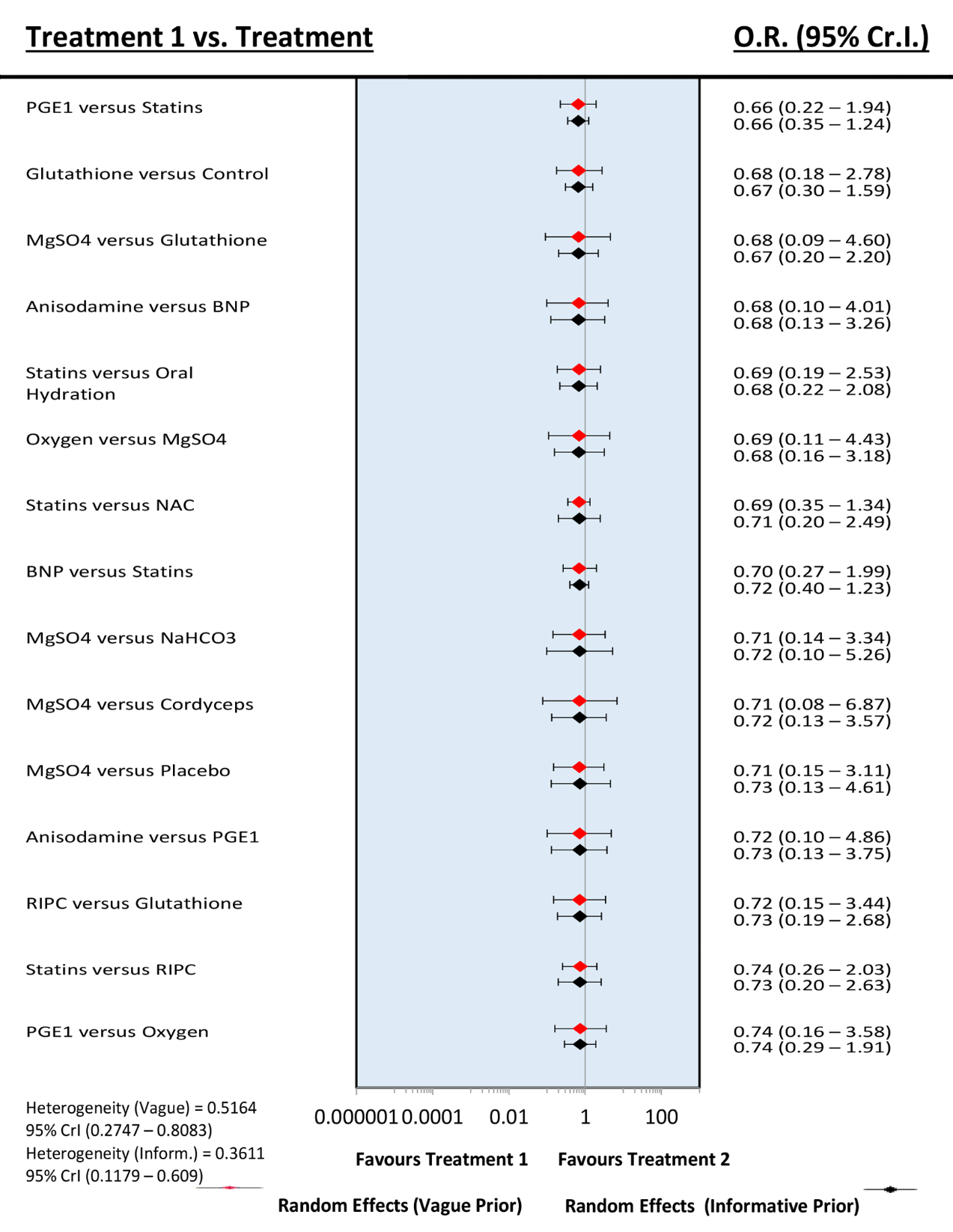


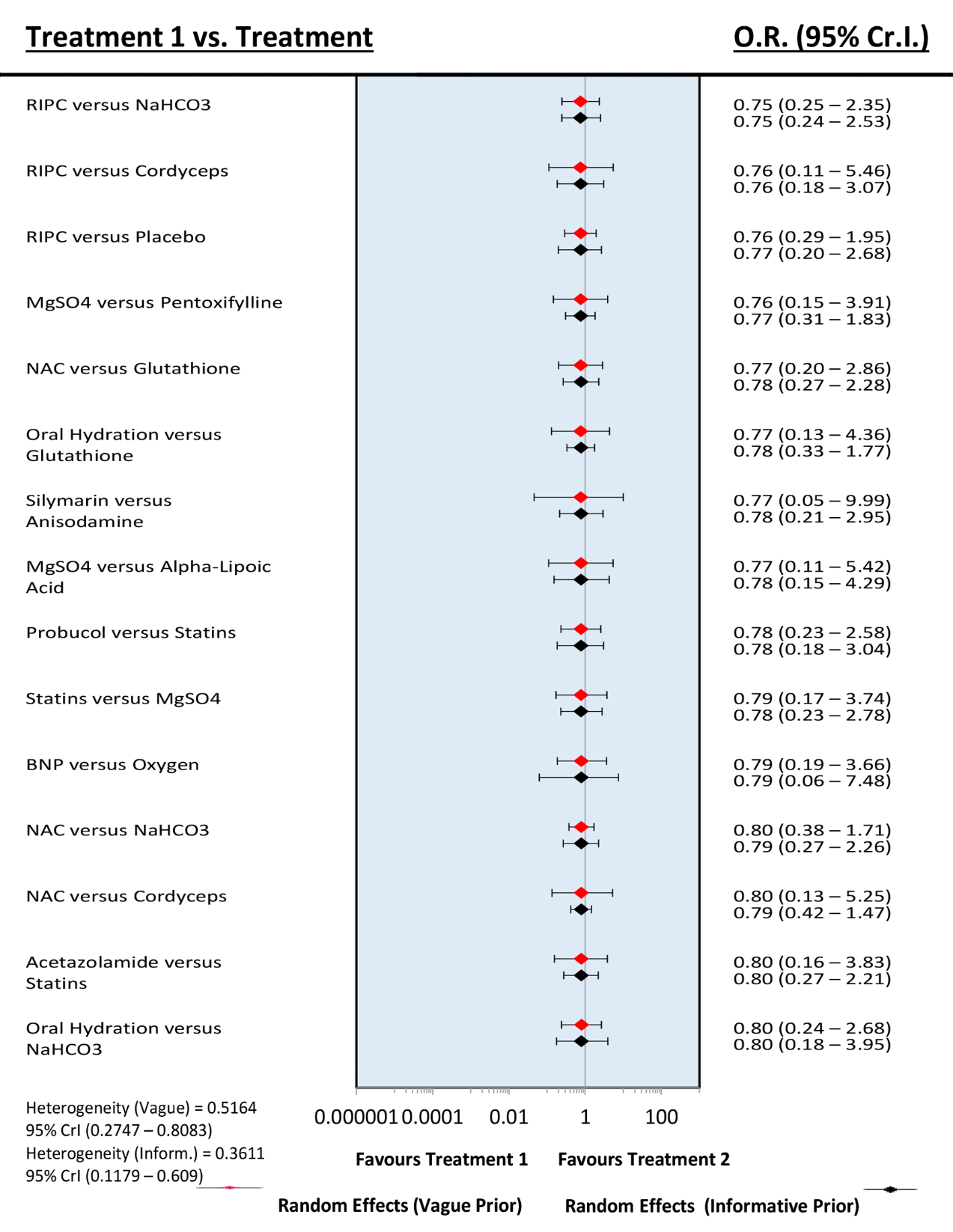


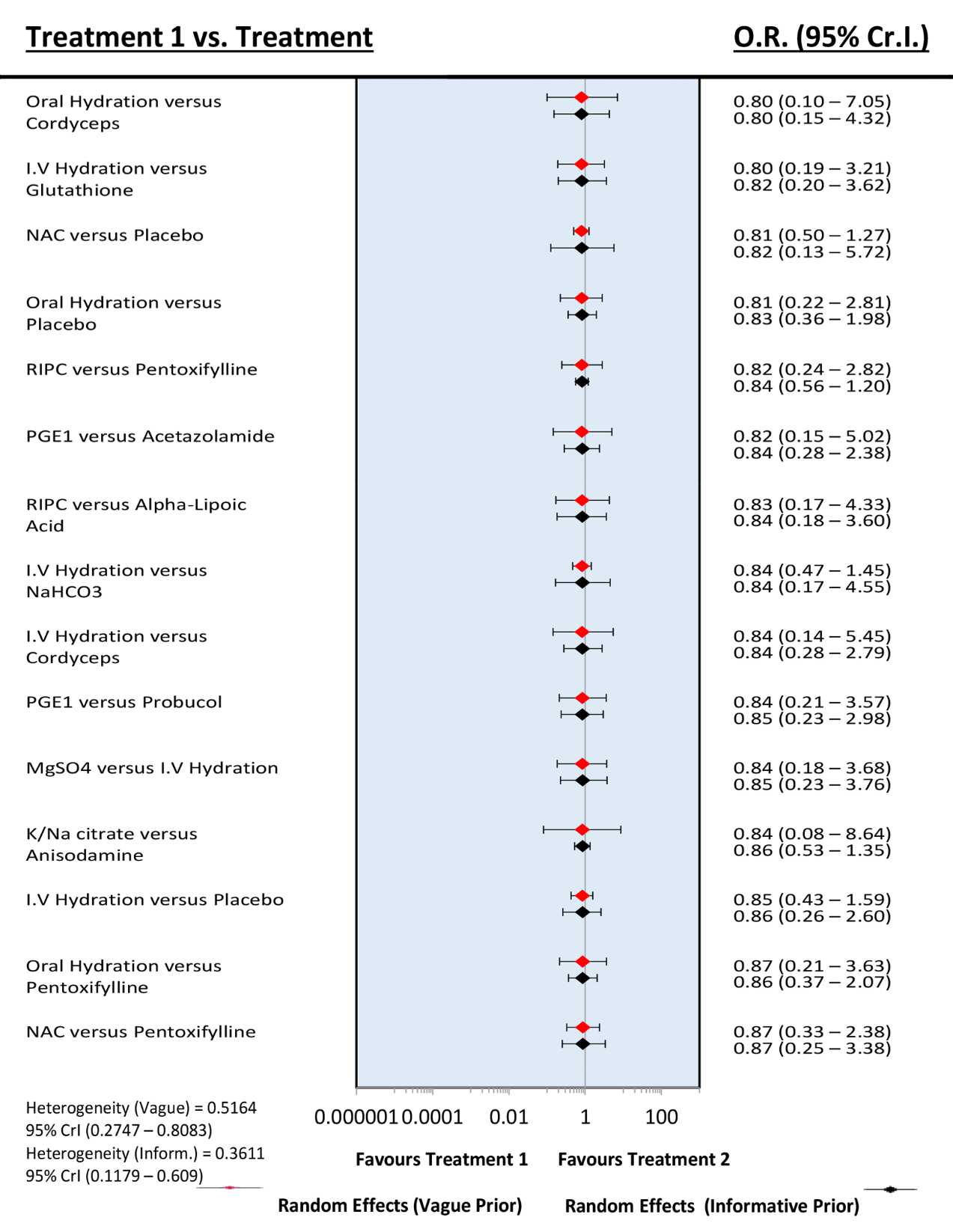


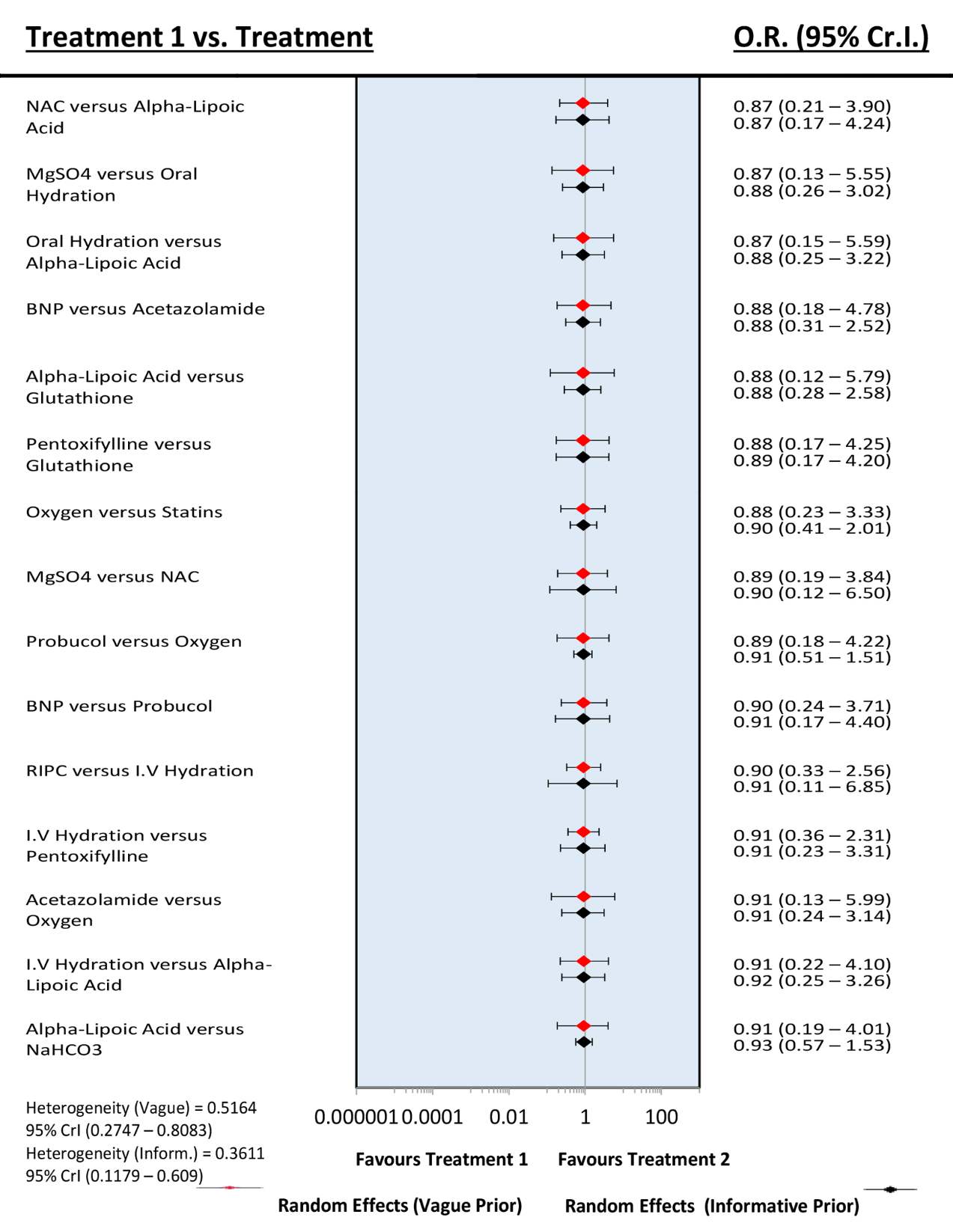


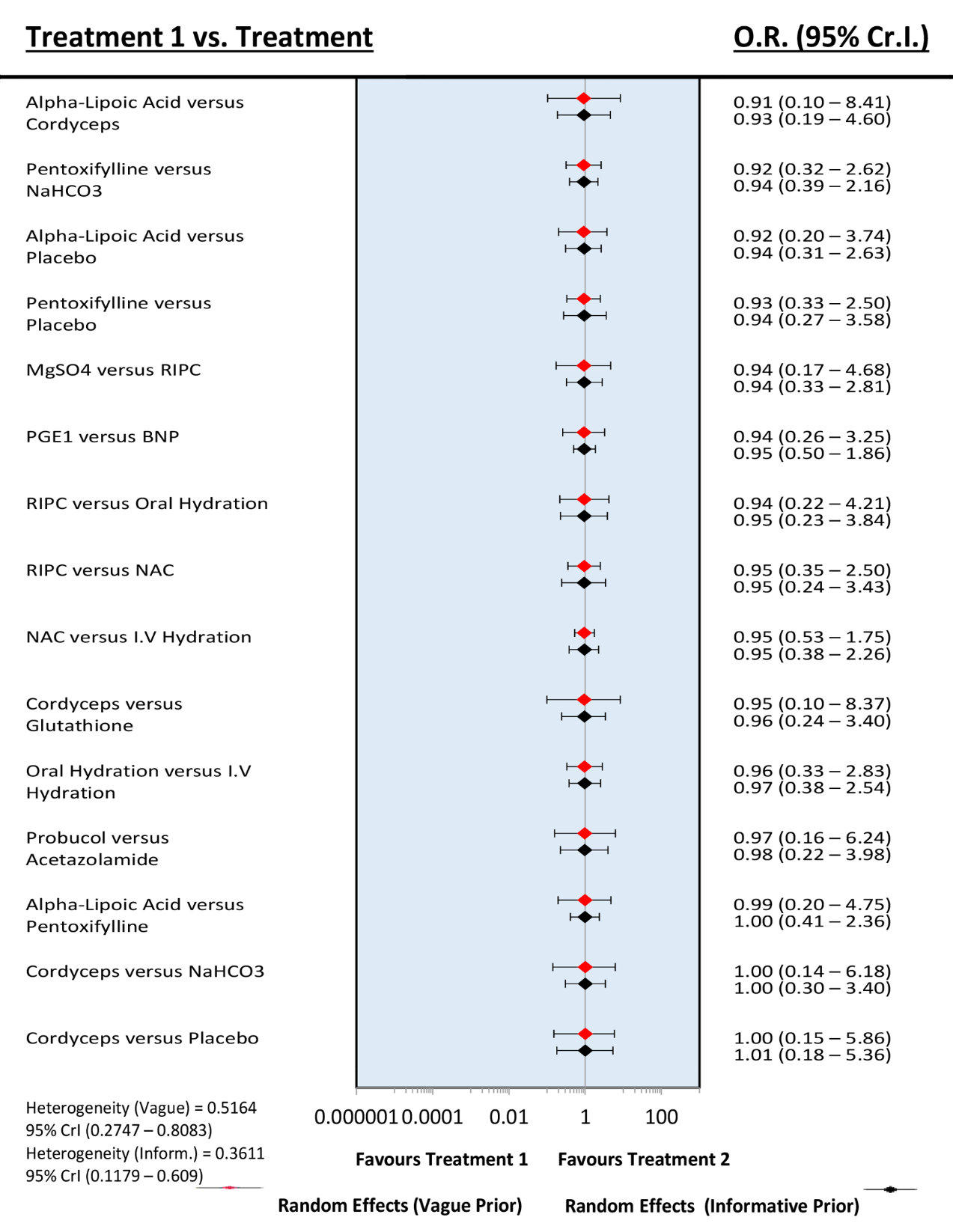


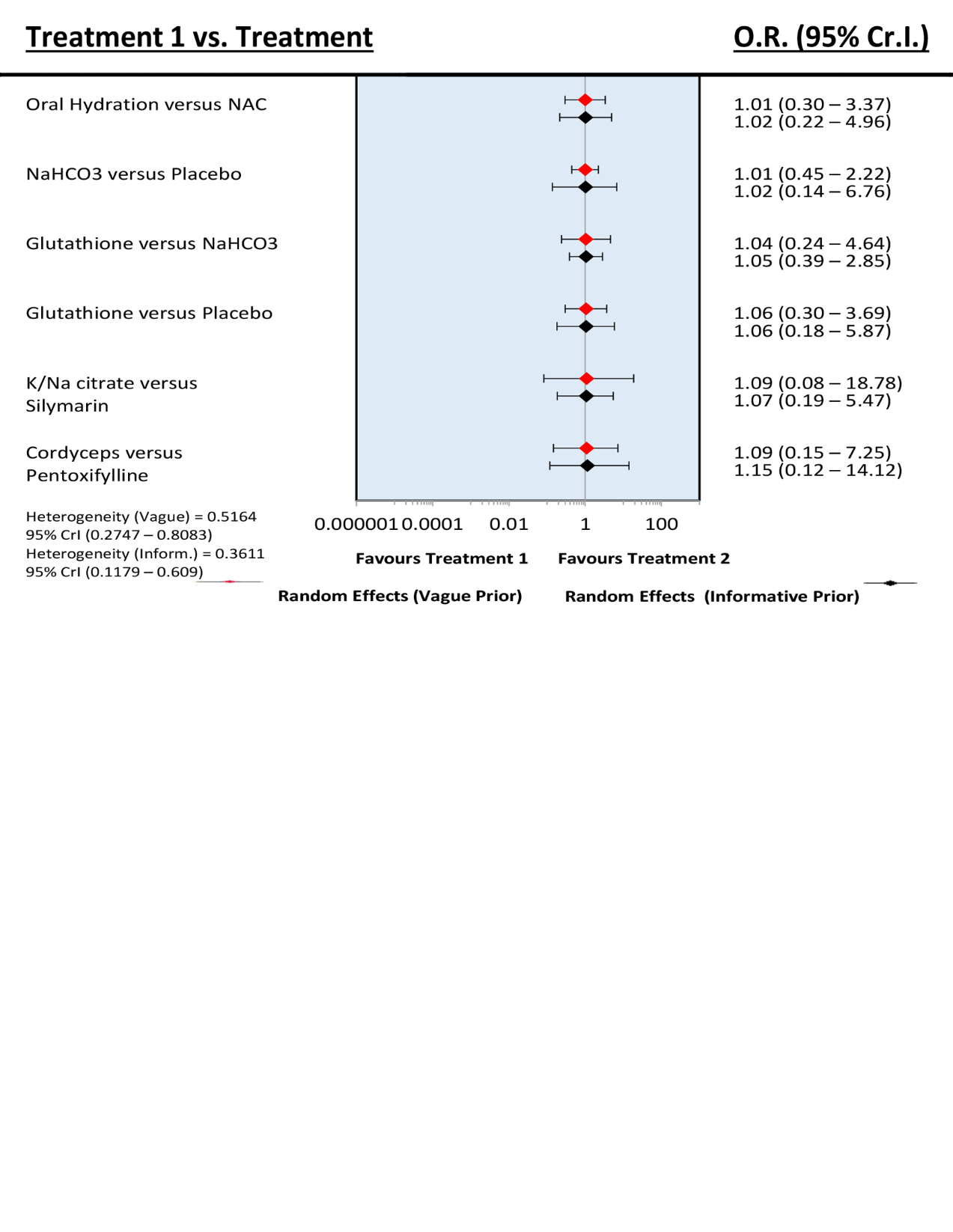


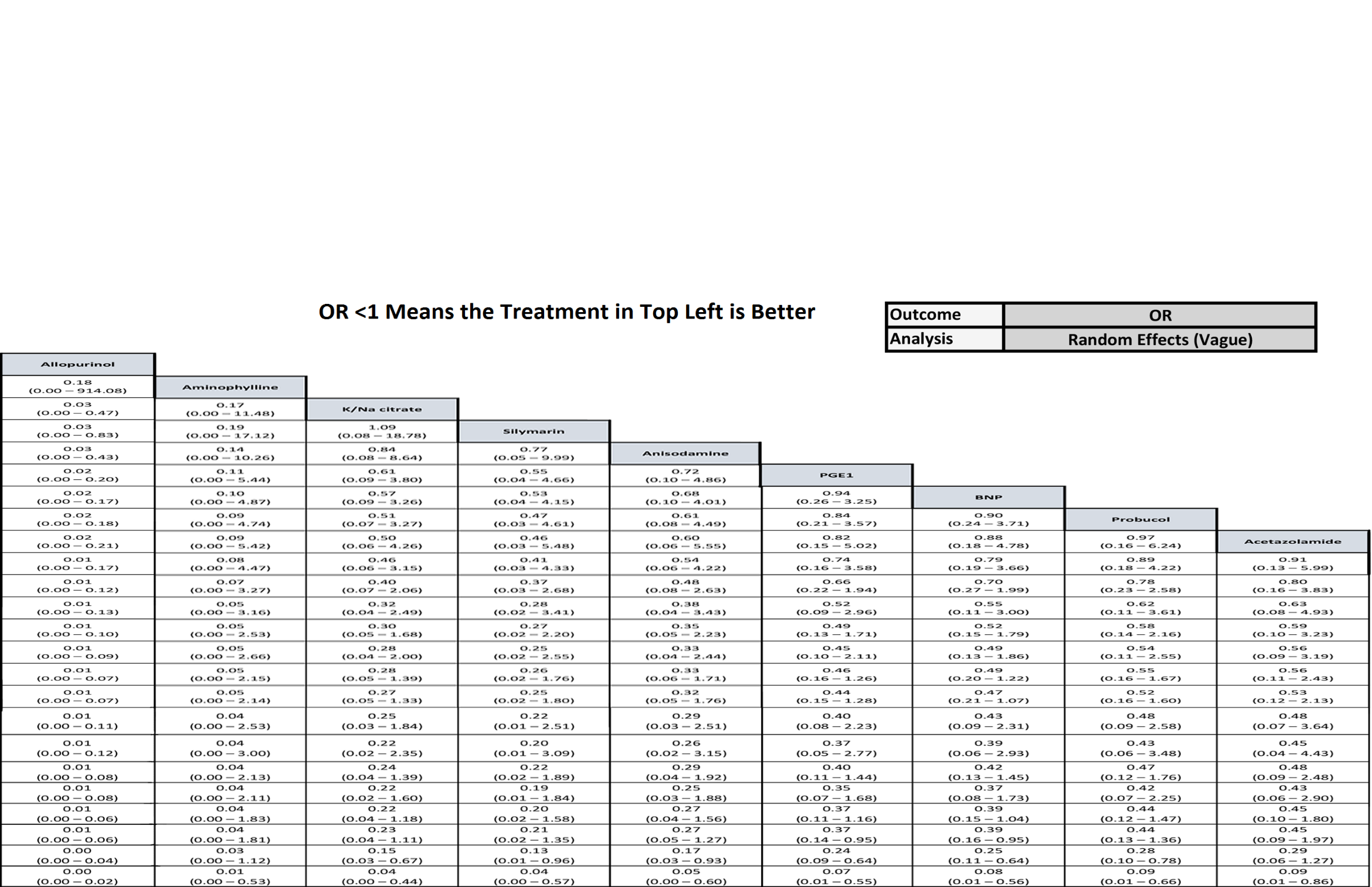


**Figure 4 League Table**


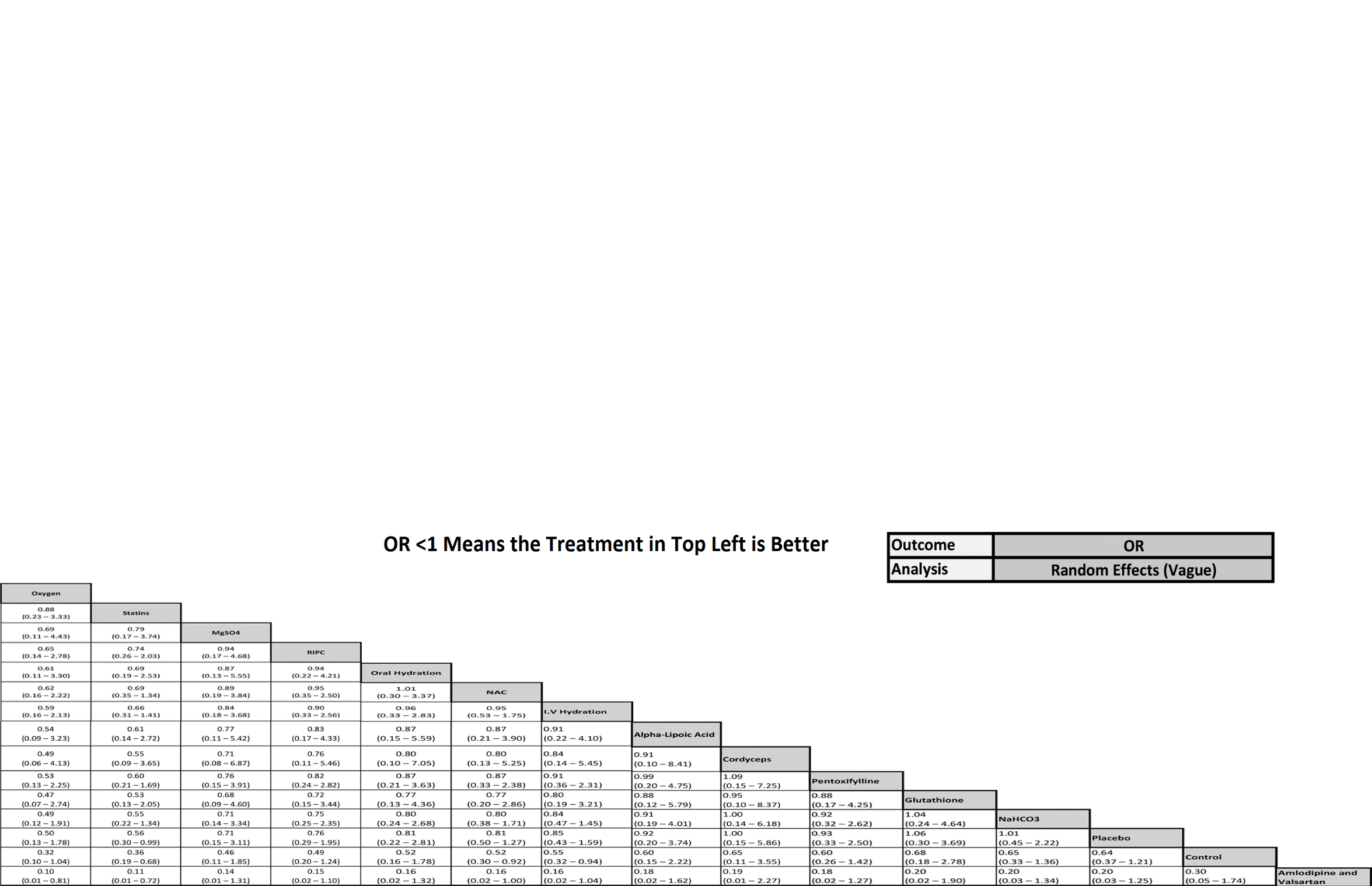


**Sub-Analysis after excluding studies with zero values**

Total Number of studies: 53 RCTs (

Excluded studies with zero events (total 7)

| Study | Interventions included |
| --- | --- |
| Kinbara, 2010 | Control, NAC, Aminophylline |
| Kotlyar, 2005 | Placebo, NAC |
| Kumar, 2014 (2 arms per contrast media) | NAC, Allopurinol, I.V Hydration |
| Moore, 2006 | Control, NAC |
| Sandhu, 2006 | Control, NAC |
| Sar, 2010 | Placebo, NAC |

Figures and Tables:

1. Network Diagram

2. Tables:

A. Network Characteristics

B. Interventions Characteristics

C Direct comparisons characteristics

3. Rankogram

4. Ranking and probability of being the best (table)

5. Forest Plot

6. R Graphical diagnostics (Trace & Density)

| Software | Spec | Convergence | Analysis |
| --- | --- | --- | --- |
| Netmetaxl / WinBUGS14 version 1.4.3 | Burn 5000  Sim 10000 | good convergence (FE MC error 5% of the SD) | Random Effects (Vague)  Random Effects (Informative) |
| GeMTC R | Burn 5000  Sim 20000 | good convergence | Random Effects (Vague) |


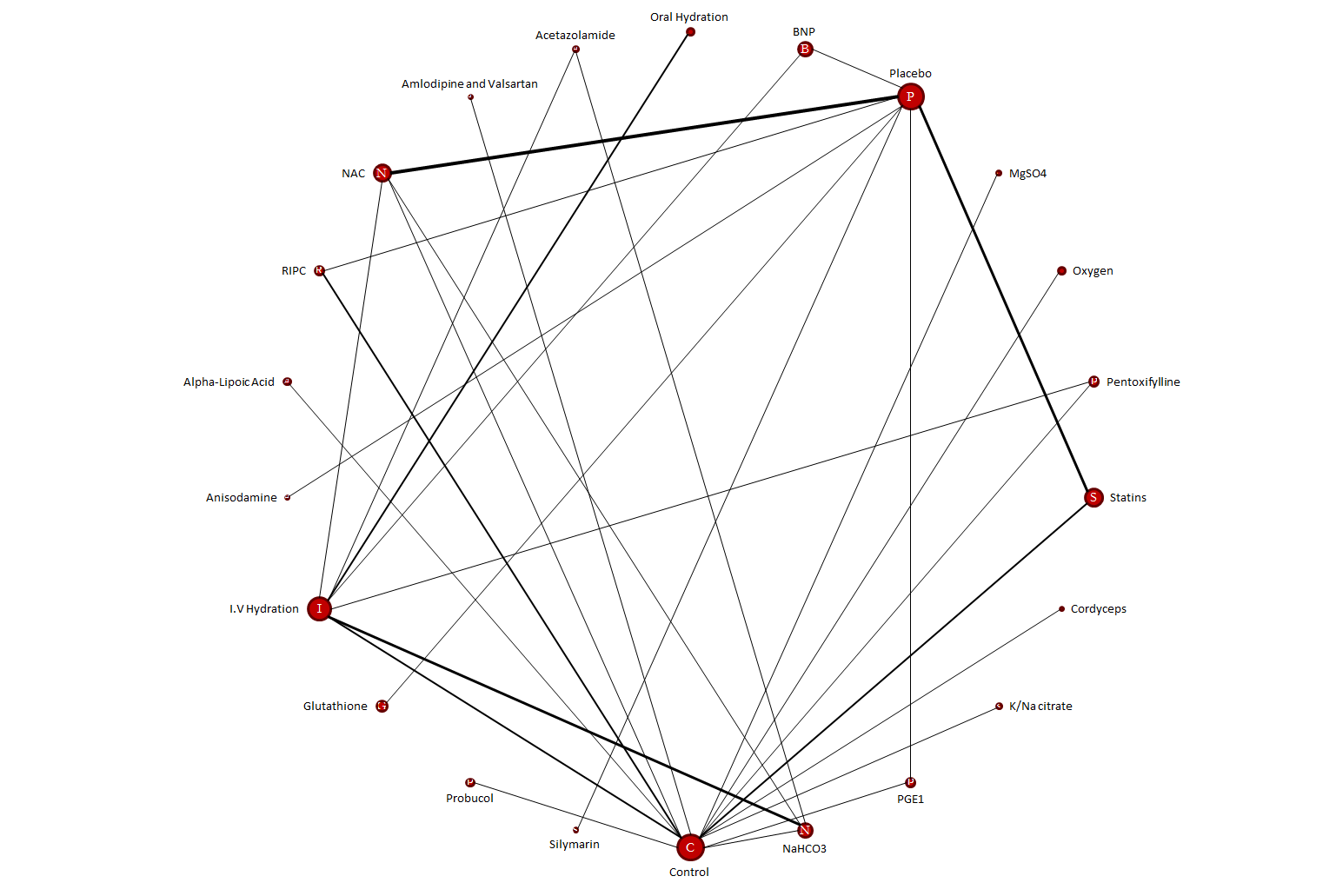


Figure 1 Network Diagram

Table 1 Network Characteristics

| **Characteristic** | **Number** |
| --- | --- |
| **Number of Interventions** | 22 |
| **Number of Studies** | 53 |
| **Total Number of Patients in Network** | 12,527 |
| **Total Number of Events in Network** | 1,439 |
| **Total Possible Pairwise Comparisons** | 231 |
| **Total Number Pairwise Comparisons With Direct Data** | 30 |
| **Number of Two-arm Studies** | 50 |
| **Number of Multi-Arms Studies** | 3 |
| **Number of Studies With No Zero Events** | 53 |
| **Number of Studies With At Least One Zero Event** | 0 |
| **Number of Studies with All Zero Events** | 0 |

Table 2 Interventions Characteristics

| **Treatment** | **# Studies** | **# Events** | **# Patients** | **Aggregate Rate** |
| --- | --- | --- | --- | --- |
| **I.V Hydration** | 14 | 217 | 1894 | 0.1146 |
| **Statins** | 8 | 65 | 1079 | 0.0602 |
| **NAC** | 10 | 132 | 1003 | 0.1316 |
| **NaHCO3** | 6 | 73 | 699 | 0.1044 |
| **PGE1** | 3 | 18 | 271 | 0.0664 |
| **MgSO4** | 1 | 9 | 62 | 0.1452 |
| **Pentoxifylline** | 3 | 24 | 328 | 0.0732 |
| **Placebo** | 18 | 278 | 2258 | 0.1231 |
| **Control** | 24 | 449 | 2454 | 0.1830 |
| **BNP** | 3 | 46 | 638 | 0.0721 |
| **Probucol** | 2 | 12 | 198 | 0.0606 |
| **Oxygen** | 1 | 32 | 172 | 0.1860 |
| **Amlodipine and Valsartan** | 1 | 8 | 45 | 0.1778 |
| **K/Na citrate** | 1 | 4 | 100 | 0.0400 |
| **Alpha-Lipoic Acid** | 2 | 6 | 139 | 0.0432 |
| **Oral Hydration** | 3 | 13 | 206 | 0.0631 |
| **Anisodamine** | 1 | 4 | 60 | 0.0667 |
| **RIPC** | 4 | 17 | 295 | 0.0576 |
| **Glutathione** | 1 | 21 | 414 | 0.0507 |
| **Acetazolamide** | 1 | 5 | 94 | 0.0532 |
| **Cordyceps** | 1 | 4 | 49 | 0.0816 |
| **Silymarin** | 1 | 2 | 69 | 0.0290 |

Table 3 Direct comparisons characteristics

| **Comparison** | **# Studies** | **# Patients** | **# Events** |
| --- | --- | --- | --- |
| **I.V Hydartion vs. Oral Hydration** | 3 | 407 | 26 |
| **NAC vs. Placebo** | 7 | 1,597 | 212 |
| **Statins vs. Placebo** | 5 | 1,142 | 100 |
| **Control vs. Alpha-Lipoic Acid** | 2 | 280 | 16 |
| **Pentoxifylline vs. Control** | 2 | 461 | 46 |
| **MgSO4 vs. Control** | 1 | 126 | 26 |
| **Control vs. RIPC** | 3 | 386 | 29 |
| **Placebo vs. RIPC** | 1 | 202 | 16 |
| **I.V Hydartion vs. Control** | 3 | 924 | 200 |
| **I.V Hydartion vs. NaHCO3** | 6 | 1,414 | 148 |
| **Statins vs. Control** | 3 | 1,044 | 84 |
| **Control vs. Probucol** | 2 | 409 | 50 |
| **PGE1 vs. Control** | 2 | 226 | 16 |
| **I.V Hydartion vs. BNP** | 2 | 1,128 | 113 |
| **NaHCO3 vs. Control** | 1 | 300 | 59 |
| **NAC vs. Control** | 2 | 275 | 60 |
| **Control vs. K/Na citrate** | 1 | 202 | 25 |
| **PGE1 vs. Placebo** | 1 | 330 | 53 |
| **Control vs. Oxygen** | 1 | 348 | 105 |
| **Control vs. Amlodipine and Valsartan** | 1 | 90 | 11 |
| **I.V Hydartion vs. Acetazolamide** | 1 | 190 | 21 |
| **NaHCO3 vs. Acetazolamide** | 1 | 190 | 9 |
| **Placebo vs. Silymarin** | 1 | 143 | 10 |
| **Placebo vs. Glutathione** | 1 | 825 | 41 |
| **Placebo vs. Anisodamine** | 1 | 126 | 17 |
| **I.V Hydartion vs. Pentoxifylline** | 1 | 199 | 12 |
| **I.V Hydartion vs. NAC** | 1 | 100 | 13 |
| **NAC vs. NaHCO3** | 1 | 100 | 26 |
| **Placebo vs. BNP** | 1 | 149 | 36 |
| **Control vs. Cordyceps** | 1 | 100 | 10 |


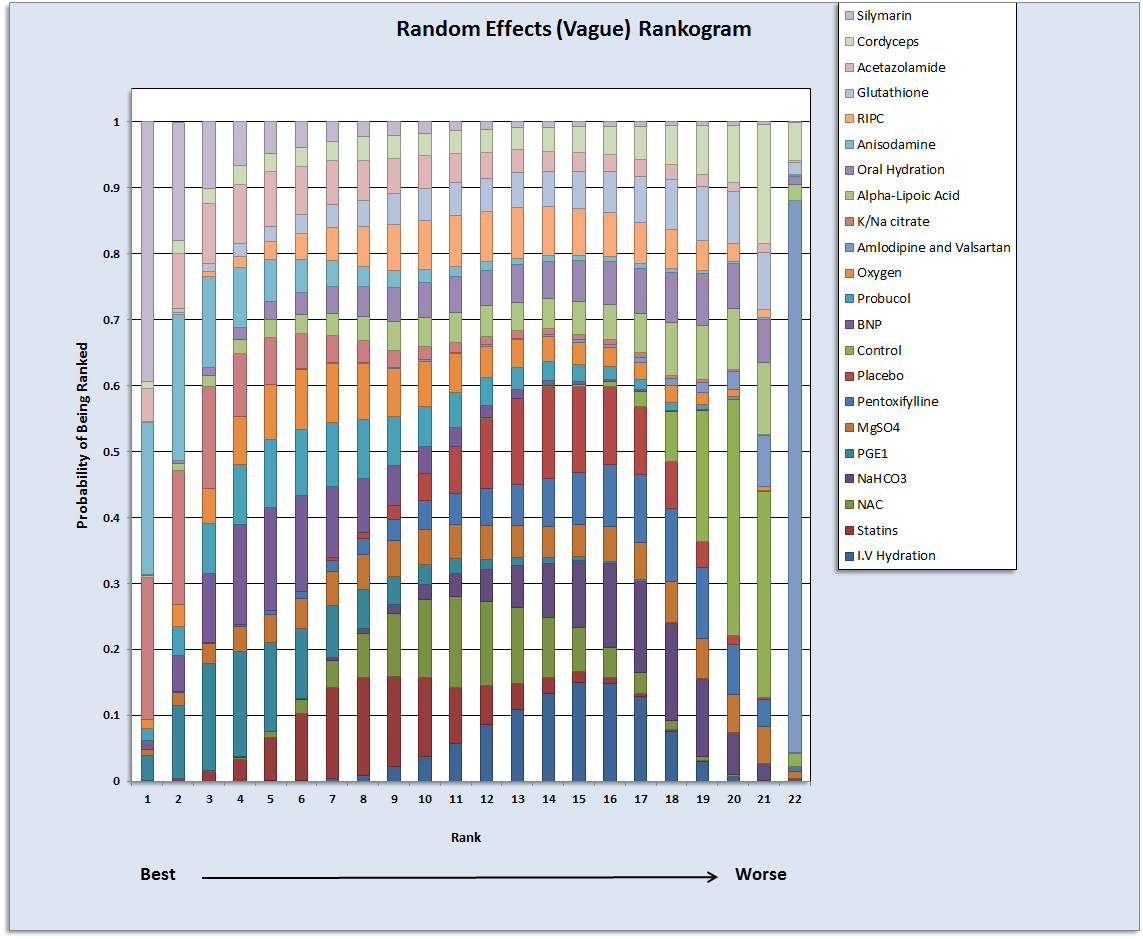


Figure 2 Rankogram: ranking the interventions for the probability of being the best, the interventions are colour coded; the first column represent the chance of being first best and 2nd column is the chance of being 2nd best and so on. The overall numerical value is presented in table 4

Table 4 Interventions ranking the treatments names column follow the league table (which arranges the presentation of summary estimates by ranking the treatments in order of most pronounced impact on the outcome under consideration) the numerical values represents the cumulative results of the probability of being best in which the highest score is 1 or 100% (see Rankogram)

| **Treatment** | **SUCRA** | **Treatment** | **SUCRA** |
| --- | --- | --- | --- |
| **Silymarin** | 0.8671 | **RIPC** | 0.4541 |
| **Anisodamine** | 0.8415 | **Oral Hydration** | 0.3887 |
| **K/Na citrate** | 0.8403 | **Glutathione** | 0.3707 |
| **PGE1** | 0.7902 | **Placebo** | 0.3649 |
| **BNP** | 0.7563 | **Alpha-Lipoic Acid** | 0.3614 |
| **Probucol** | 0.671 | **I.V Hydration** | 0.3563 |
| **Acetazolamide** | 0.6607 | **Cordyceps** | 0.3429 |
| **Statins** | 0.6331 | **Pentoxifylline** | 0.3228 |
| **Oxygen** | 0.6185 | **NaHCO3** | 0.2856 |
| **NAC** | 0.4937 | **Control** | 0.1009 |
| **MgSo4** | 0.4555 | **Amlodipine and Valsartan** | 0.02378 |
| ***Analysis*** | **Random Effects (Vague)** | | |

Figure 3 Forest Plot


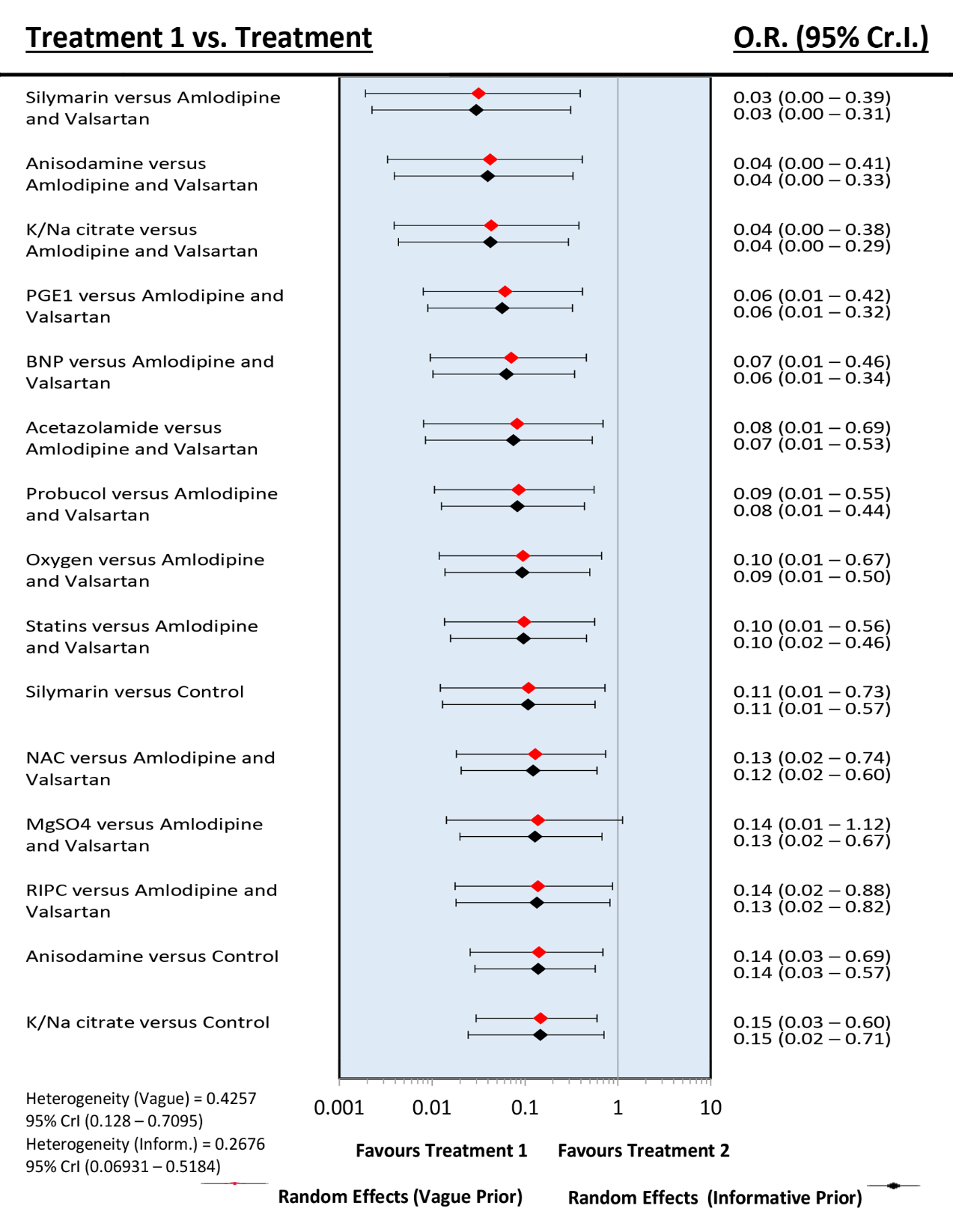


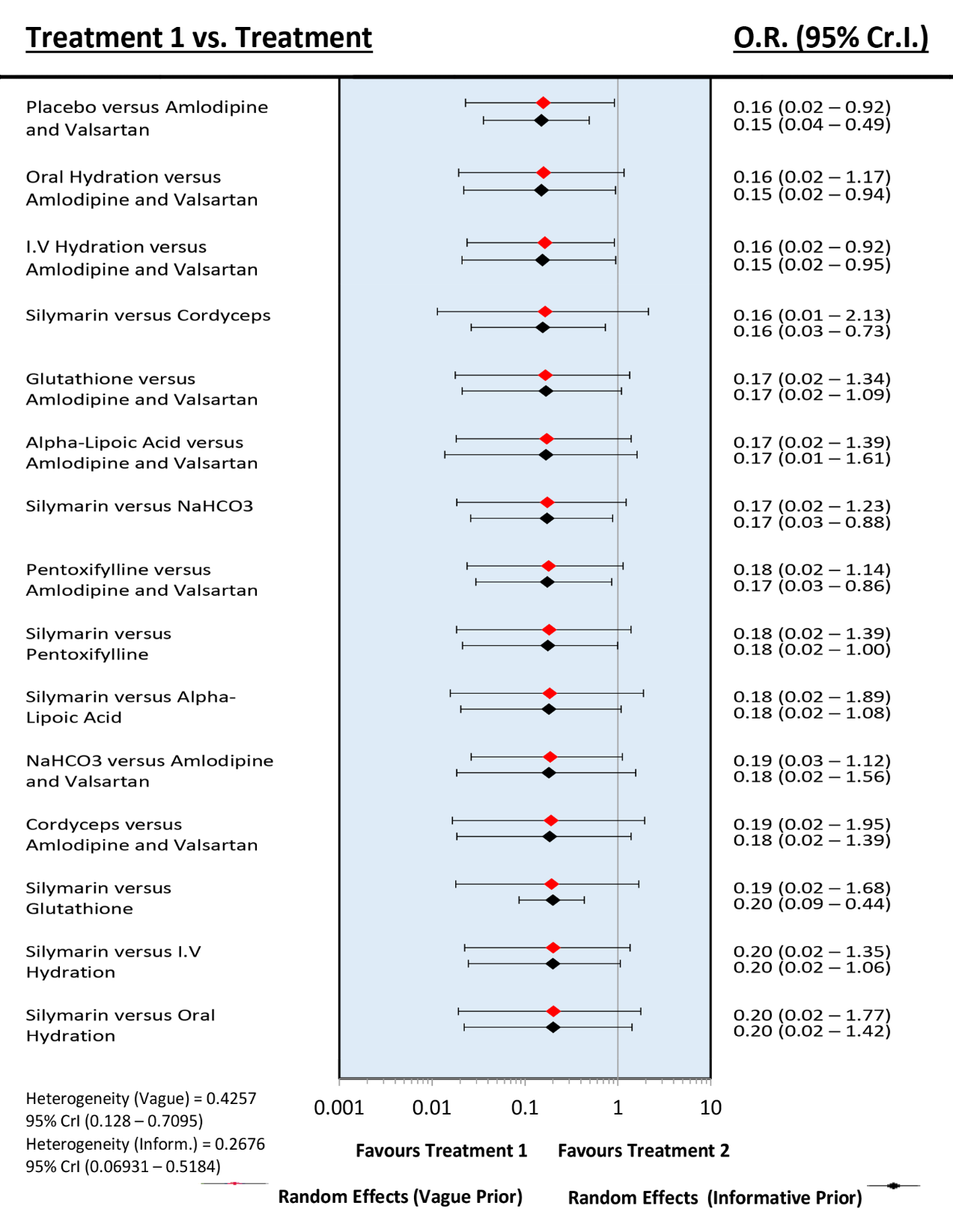


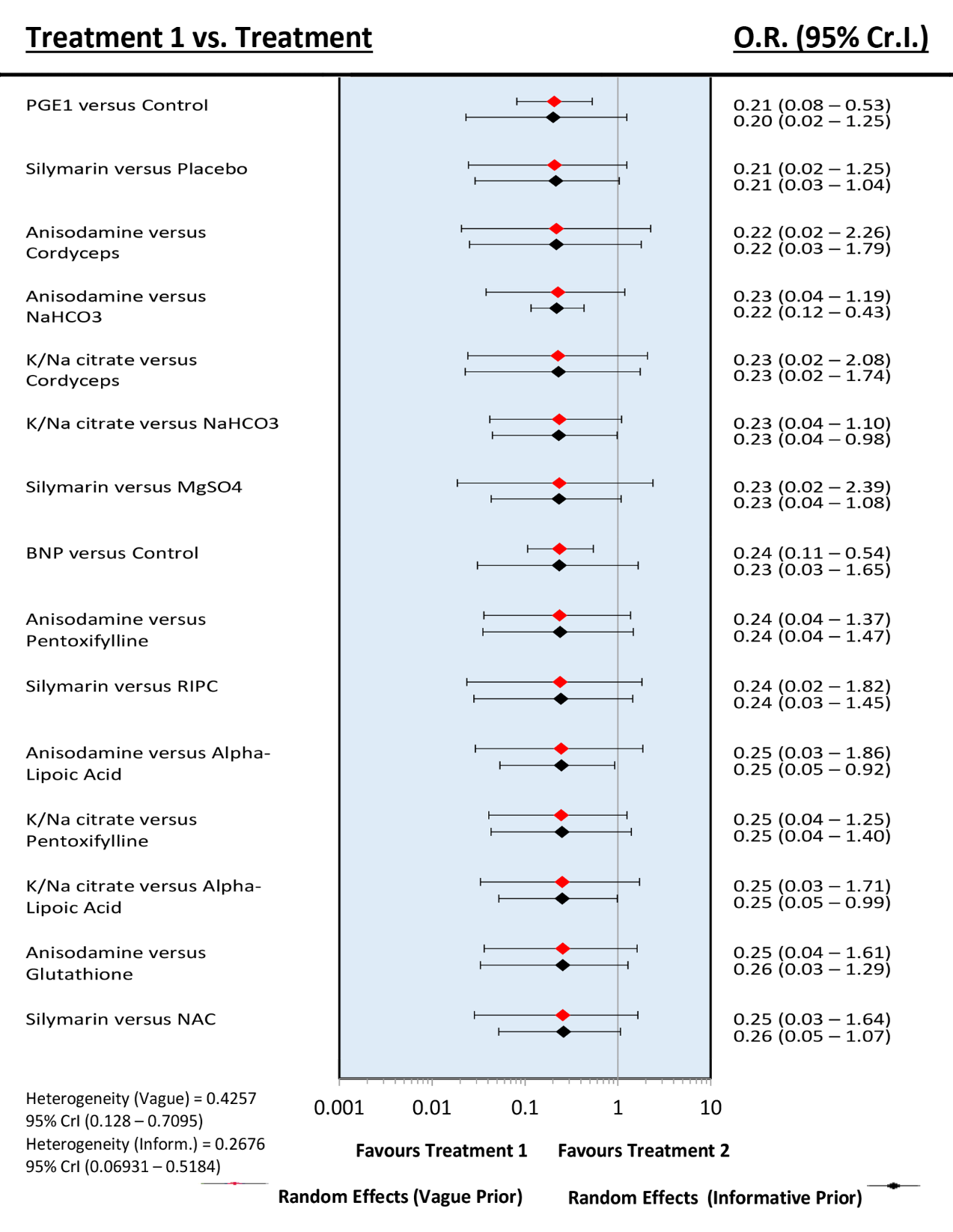


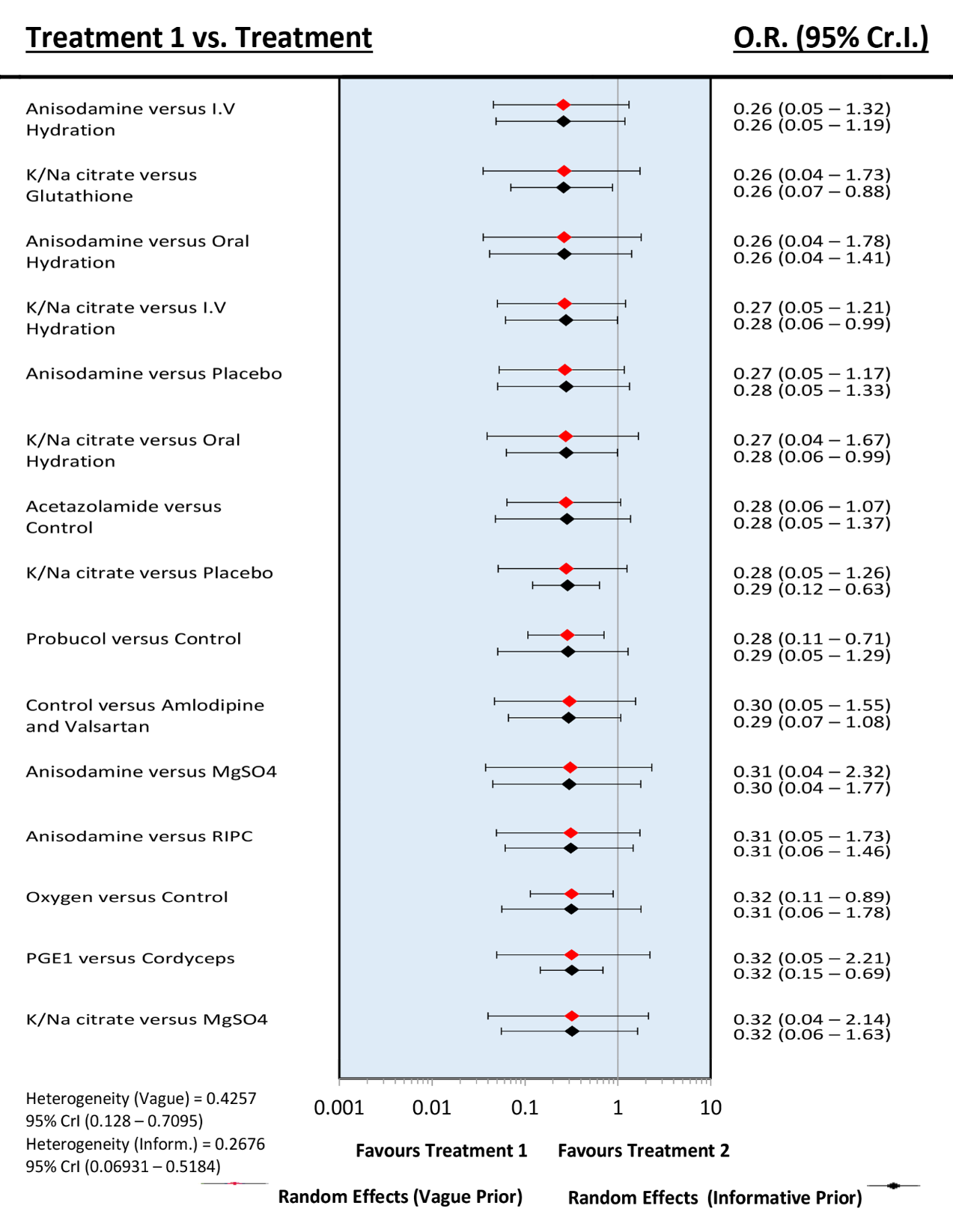


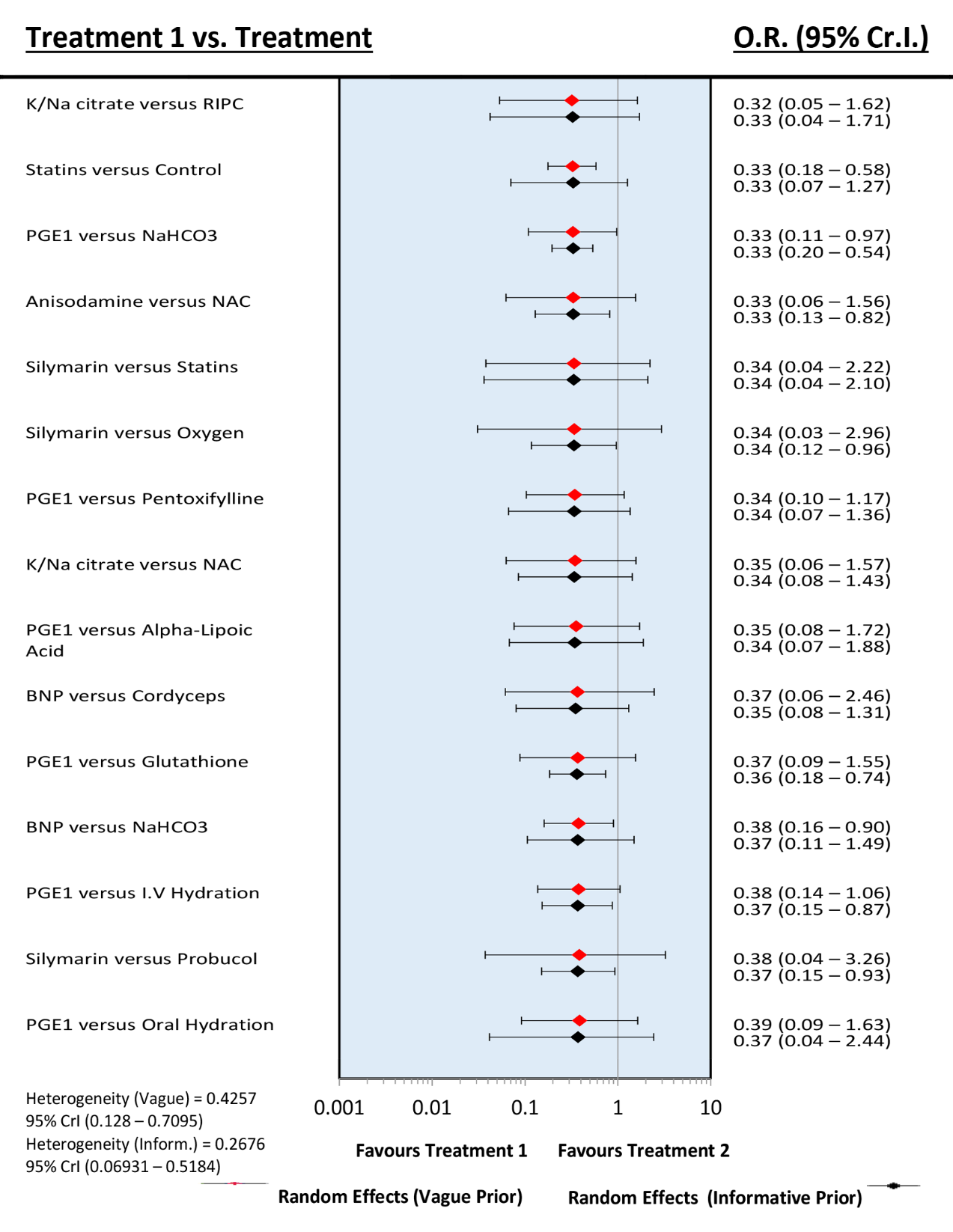


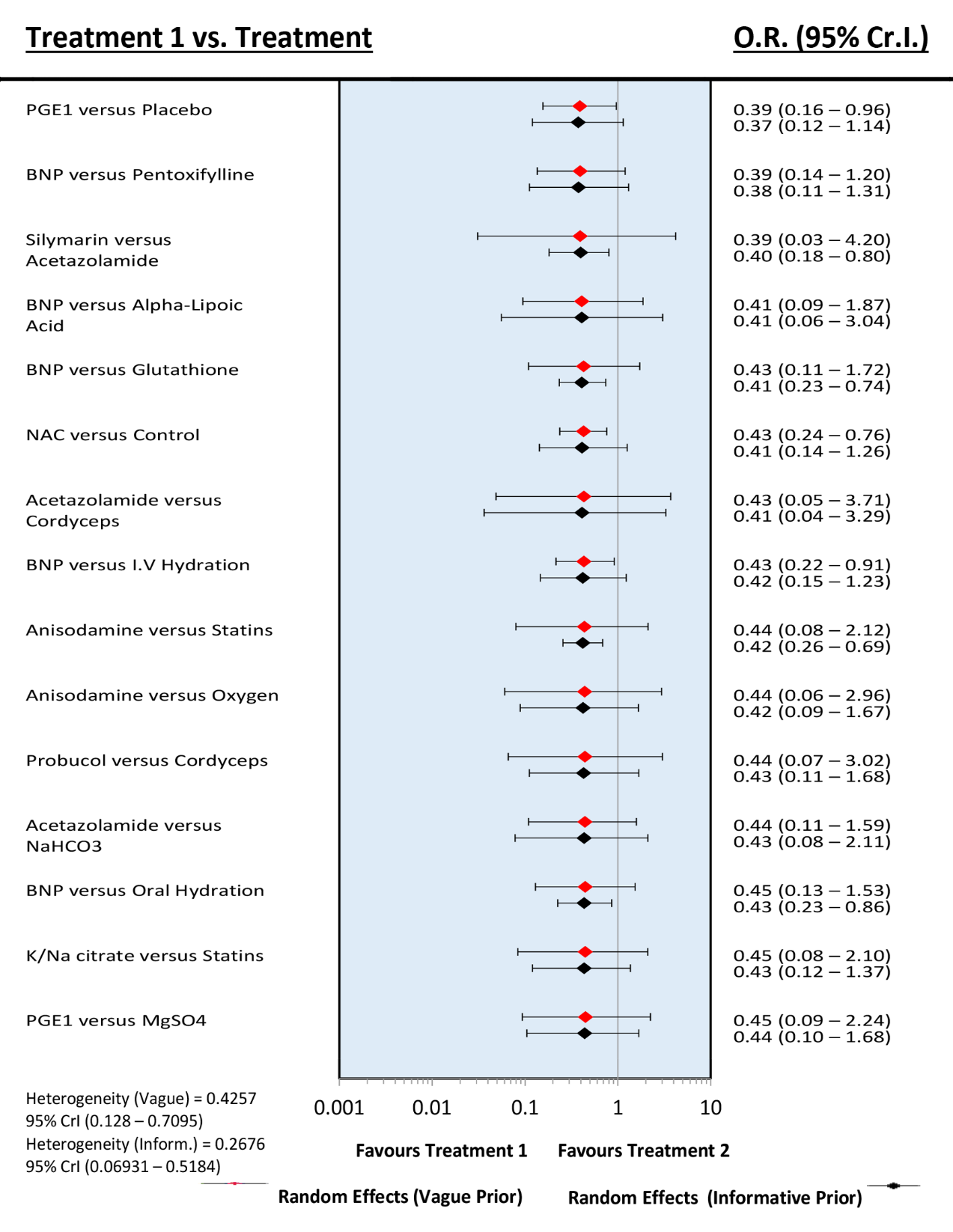


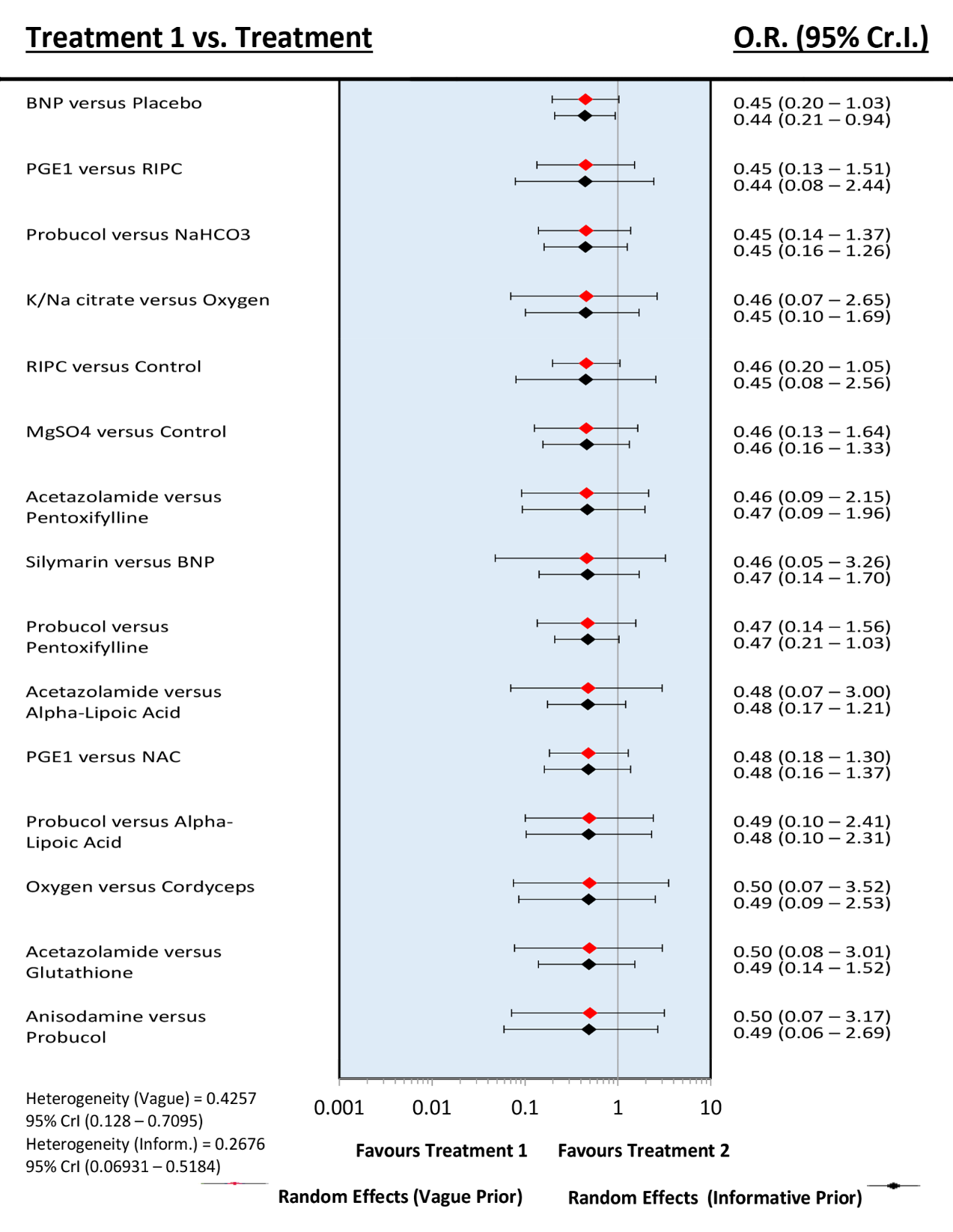


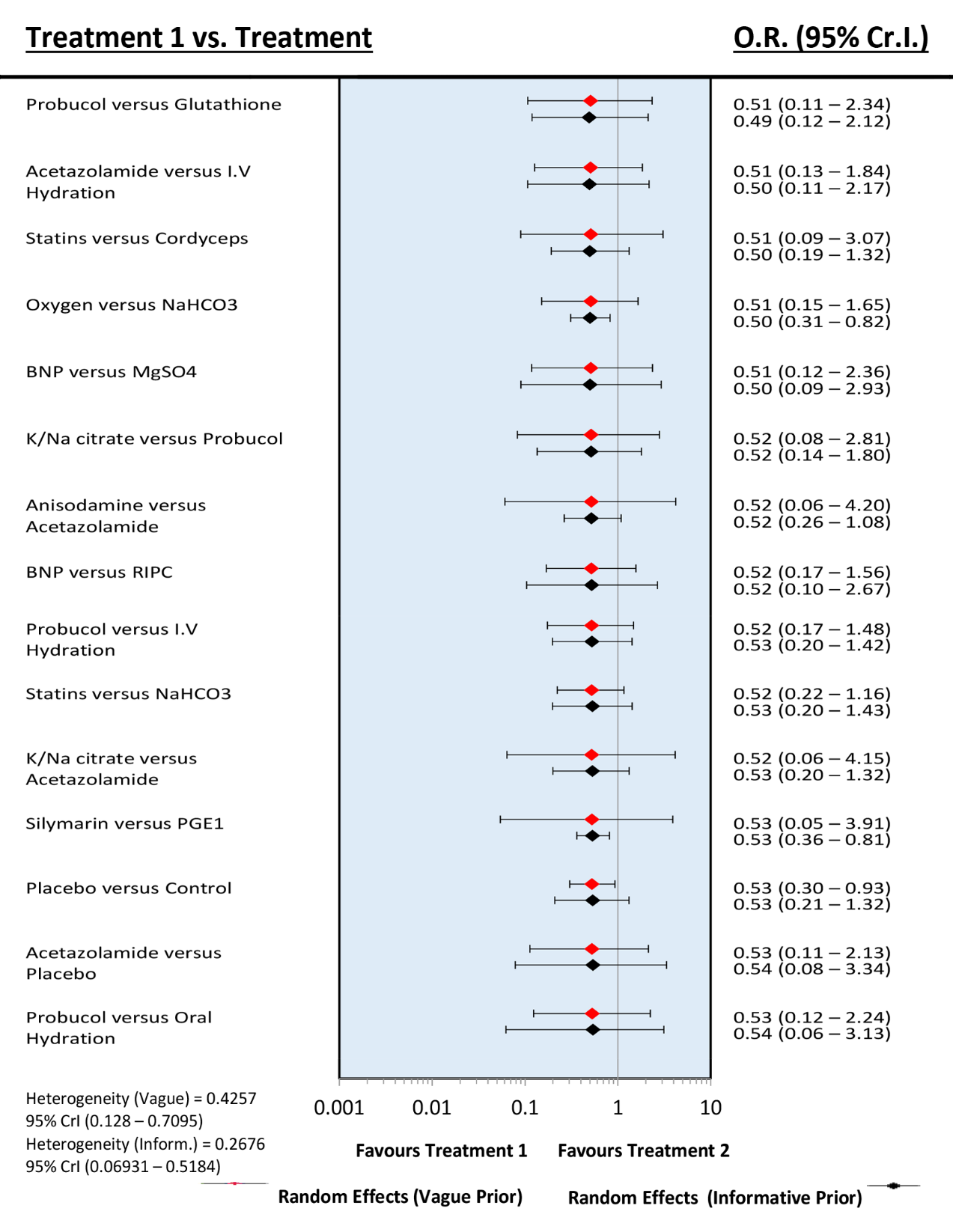


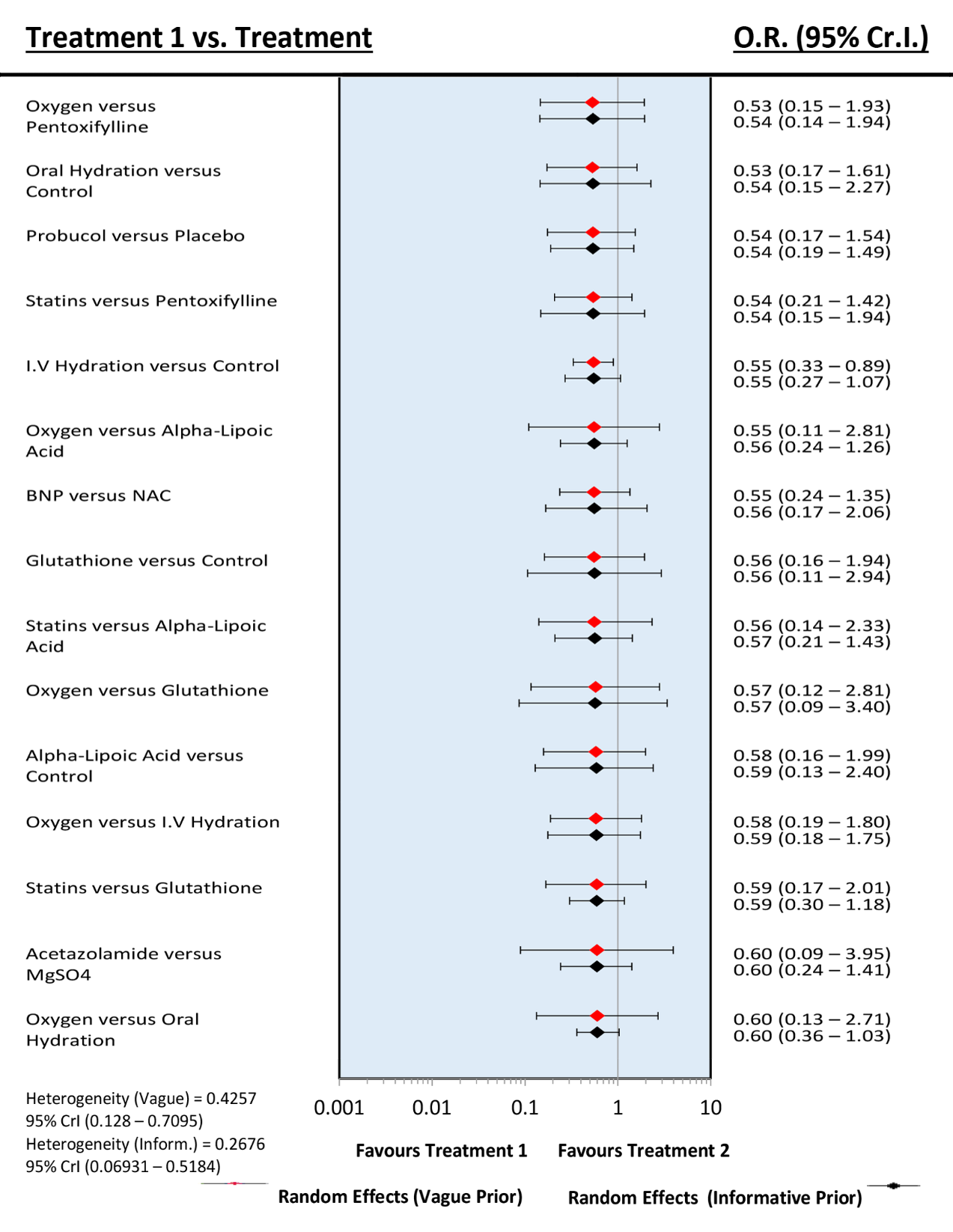


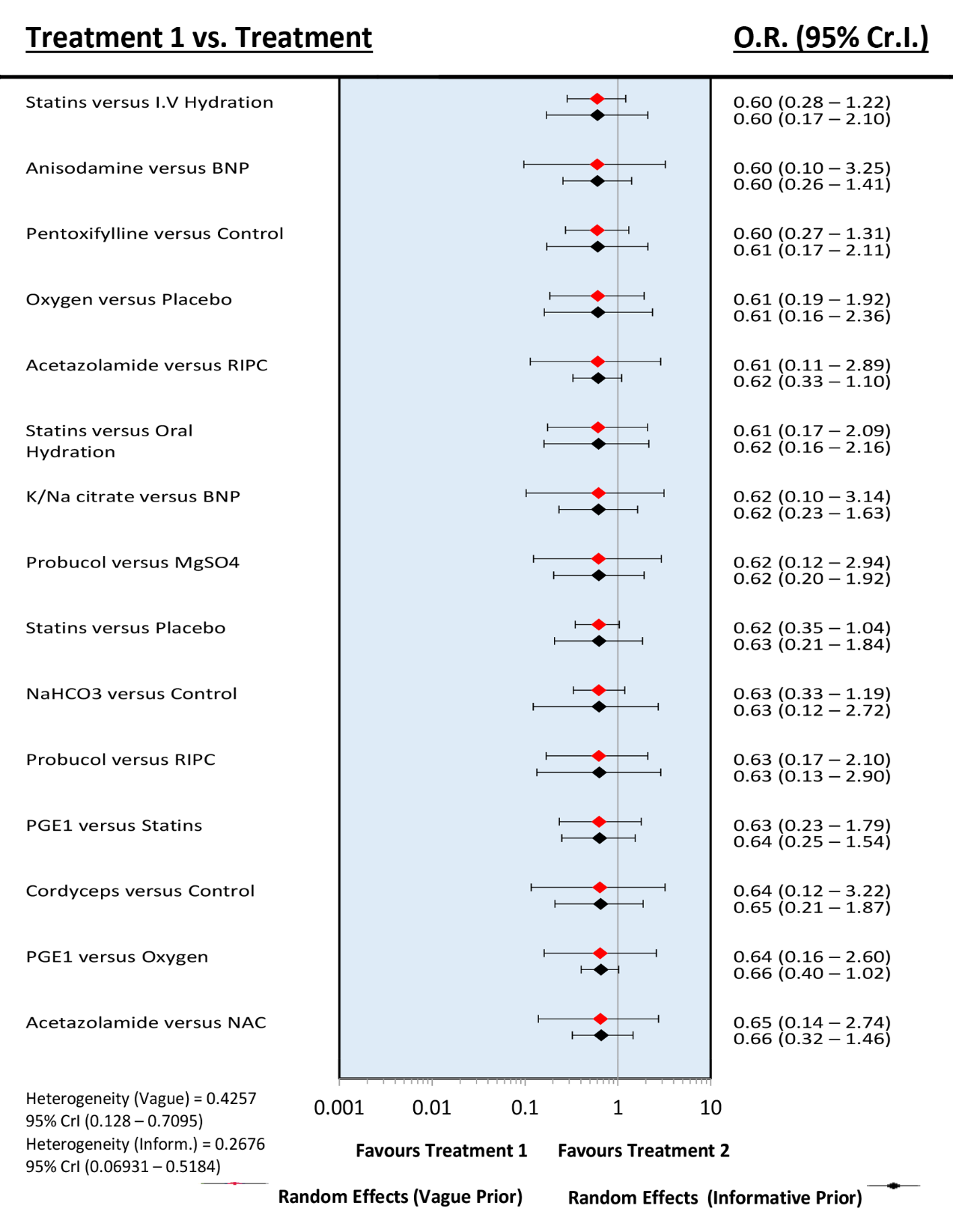


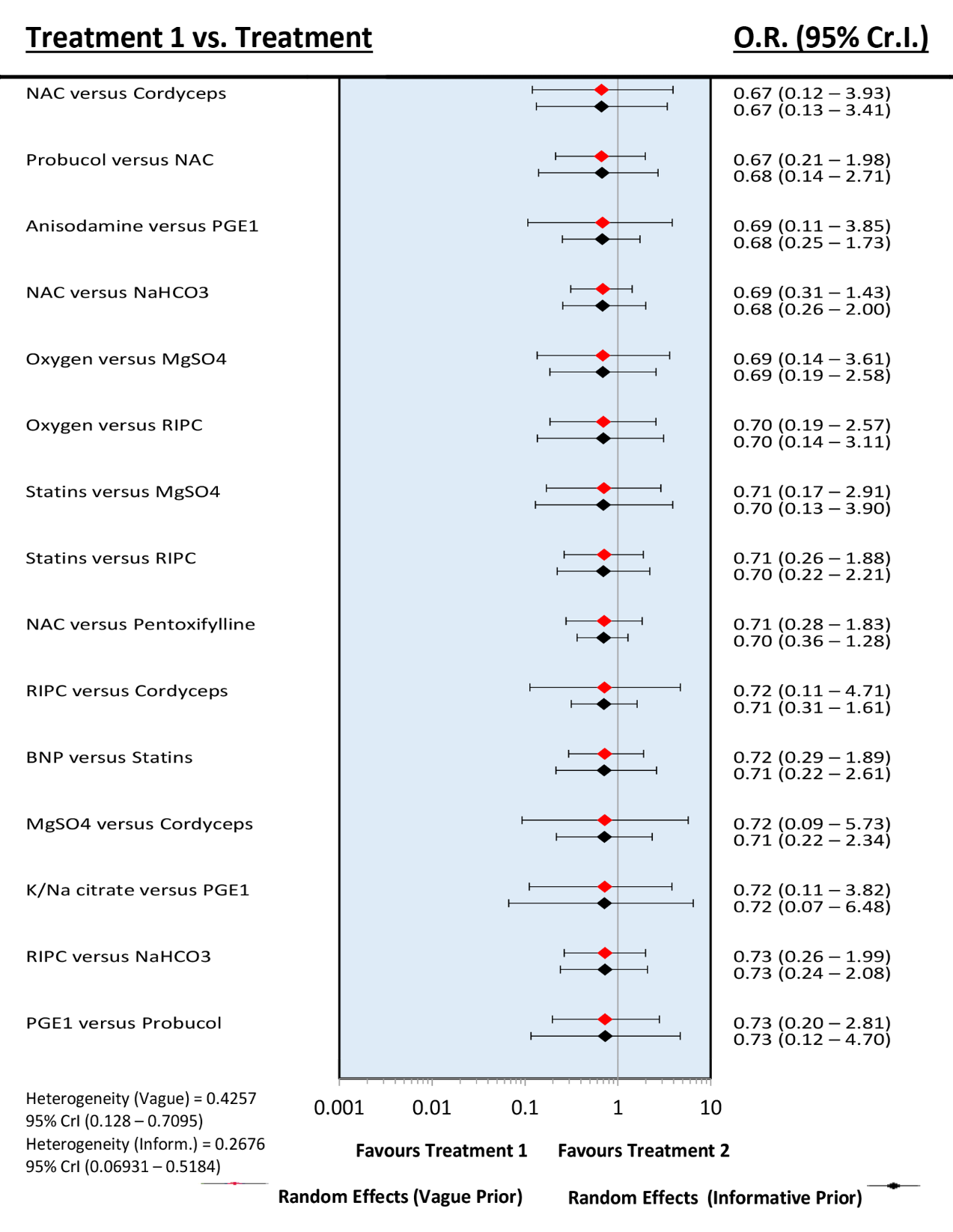


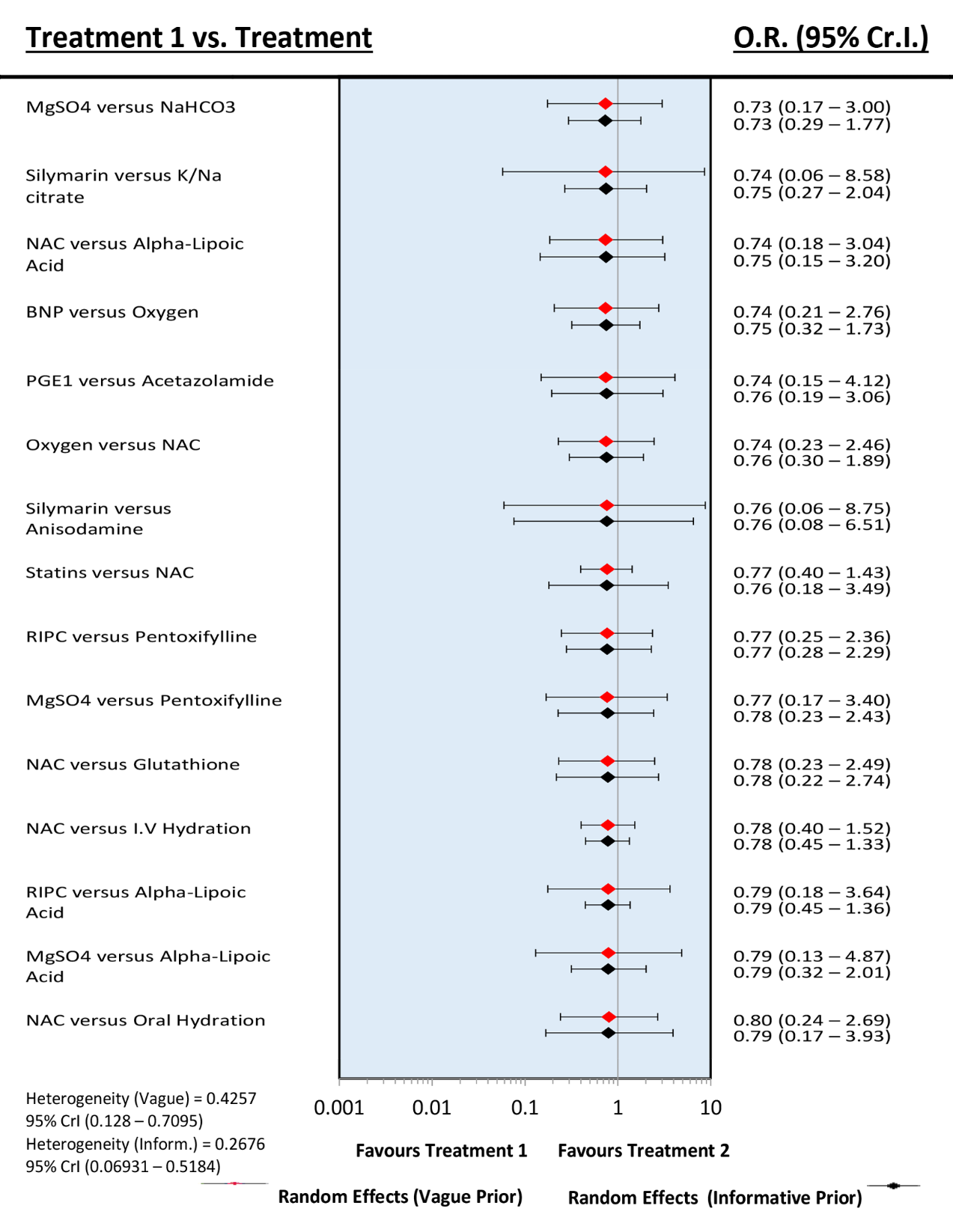


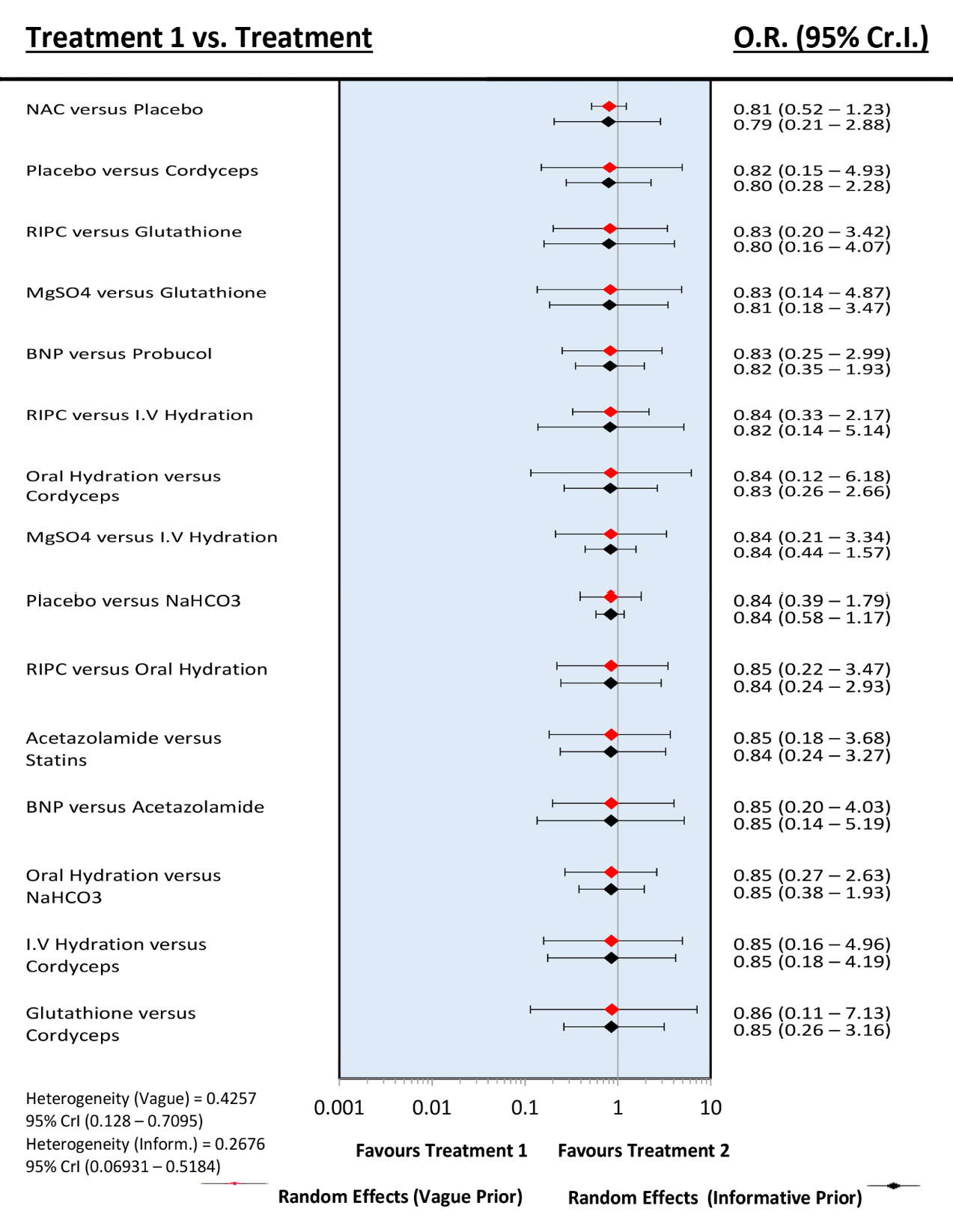


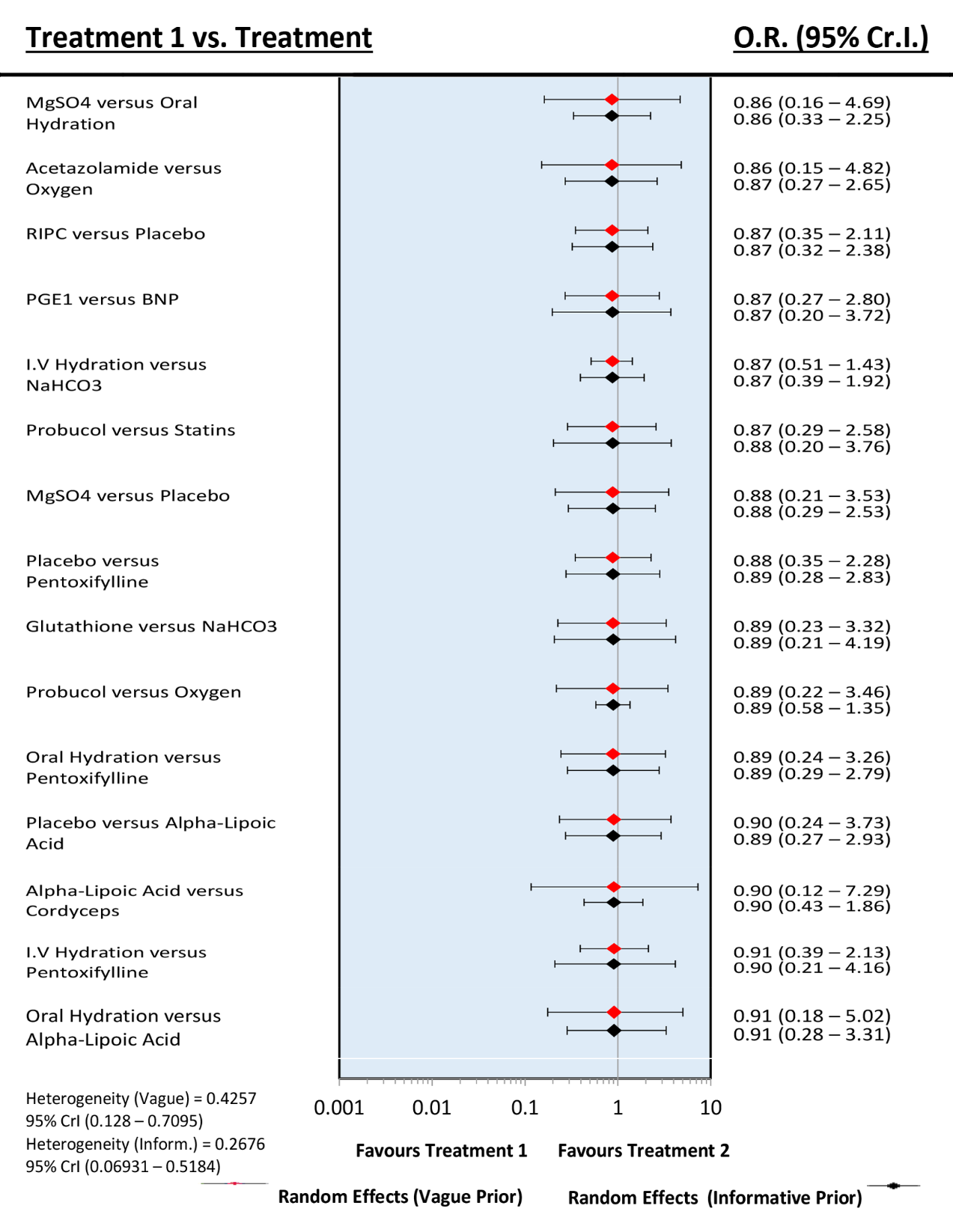


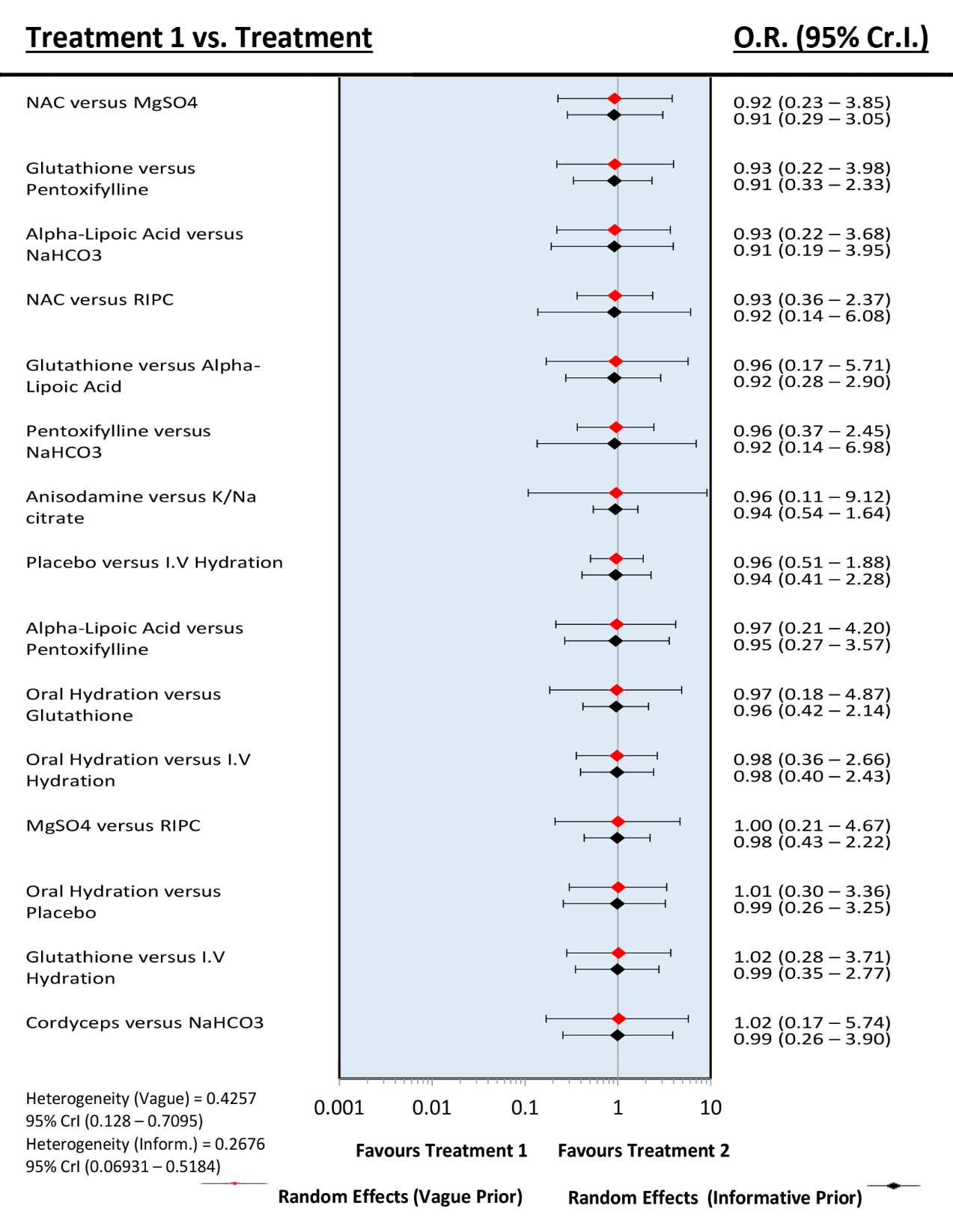


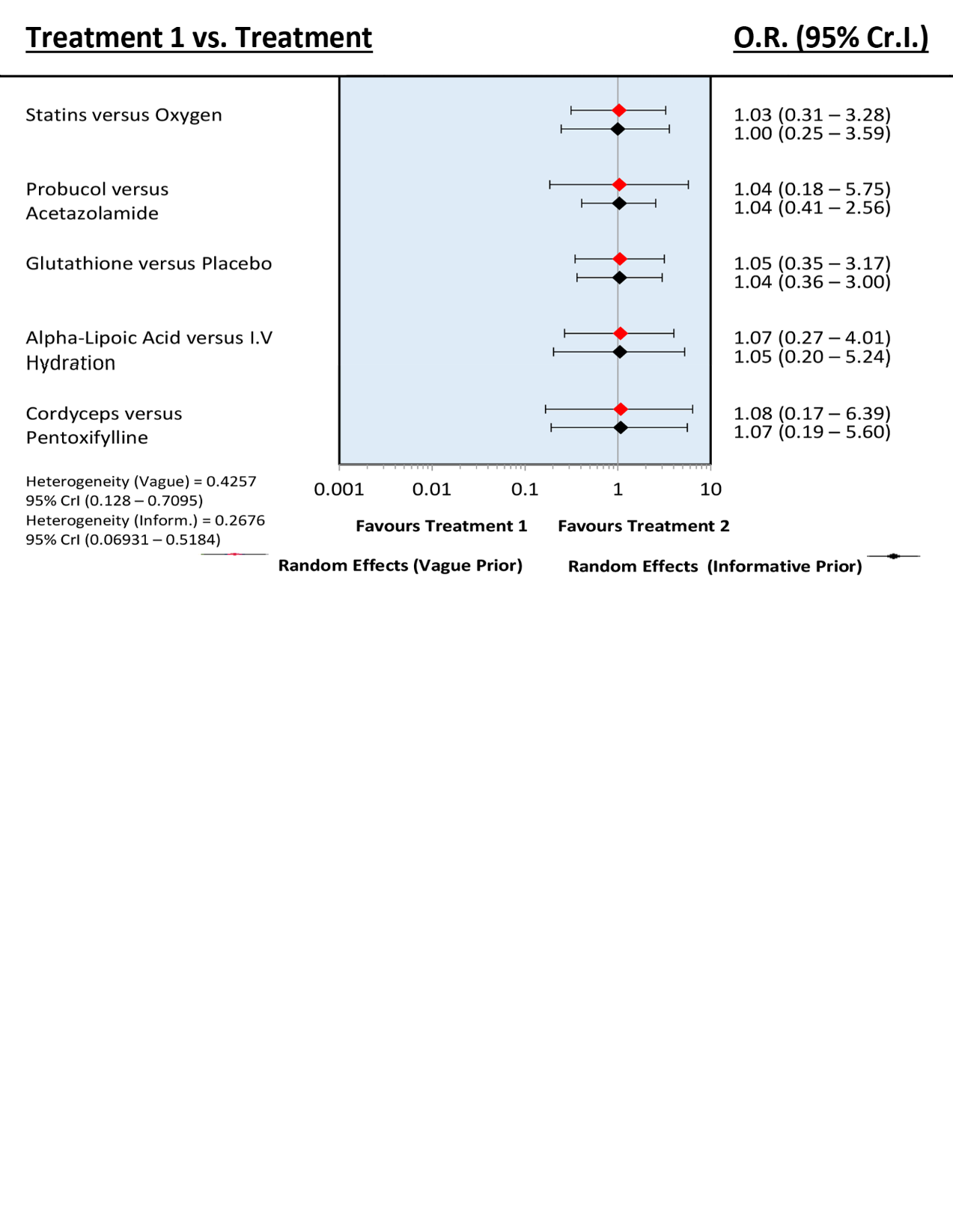


Figure 4 Forest Plot (Results from R)


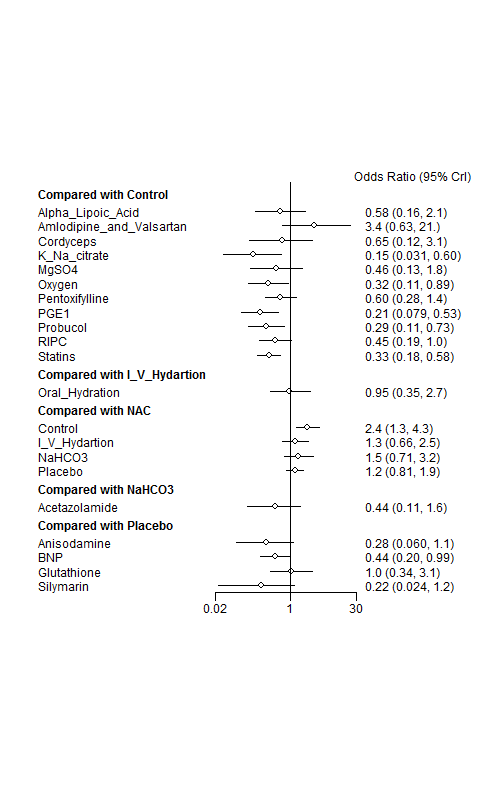


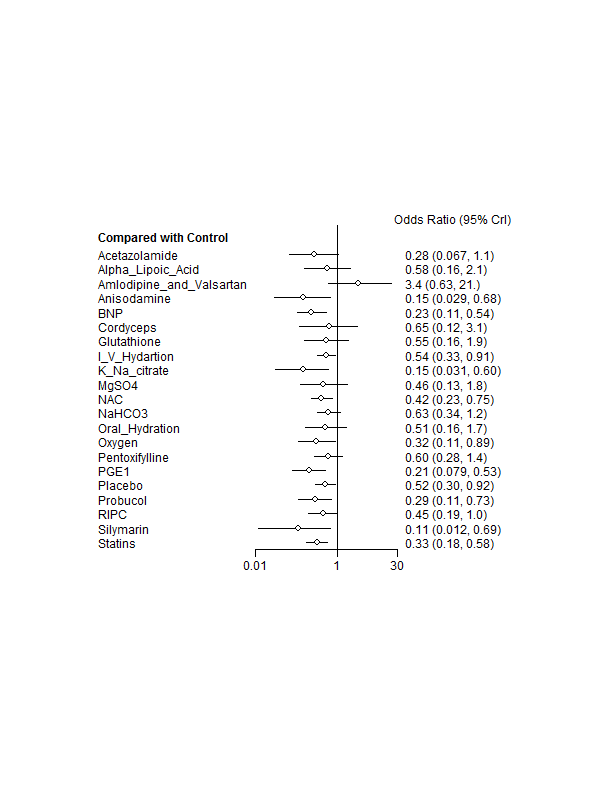

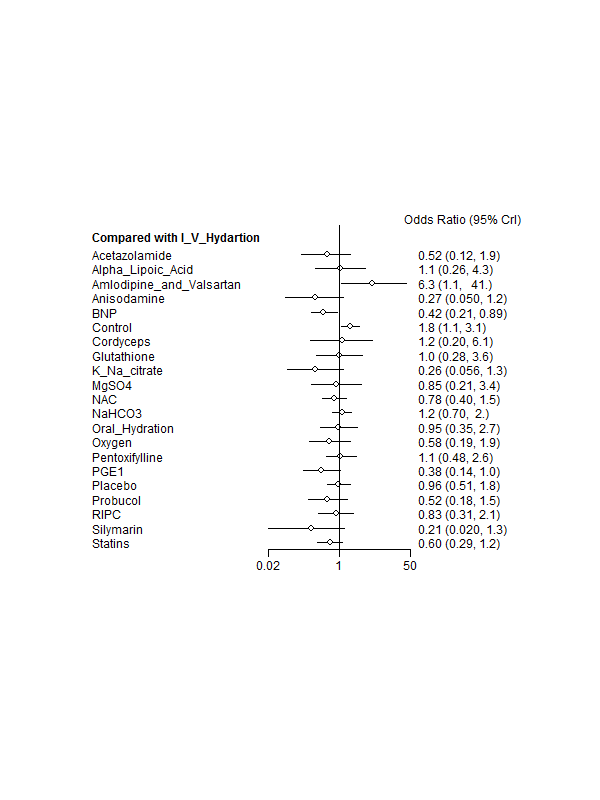

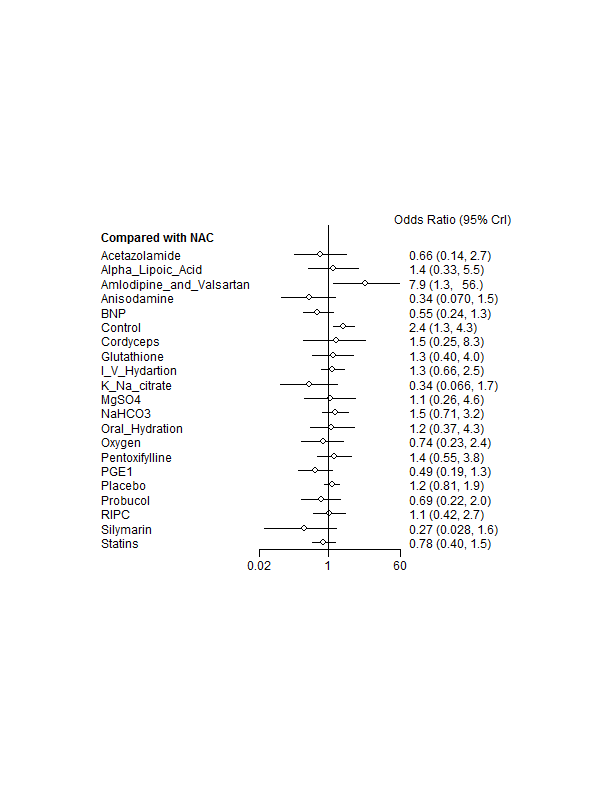

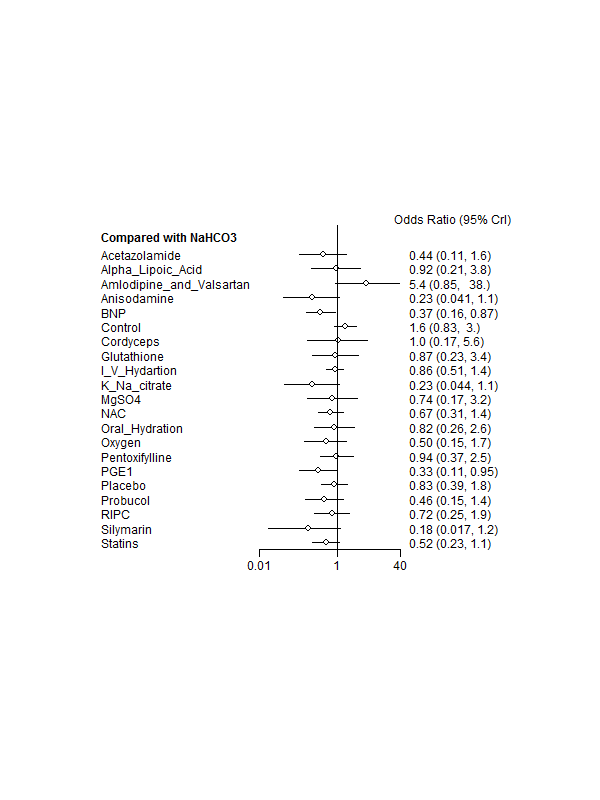

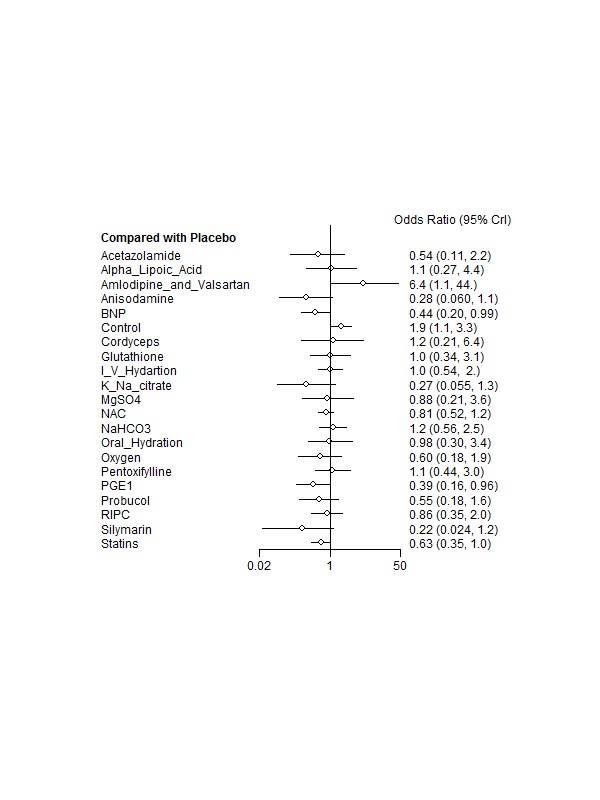

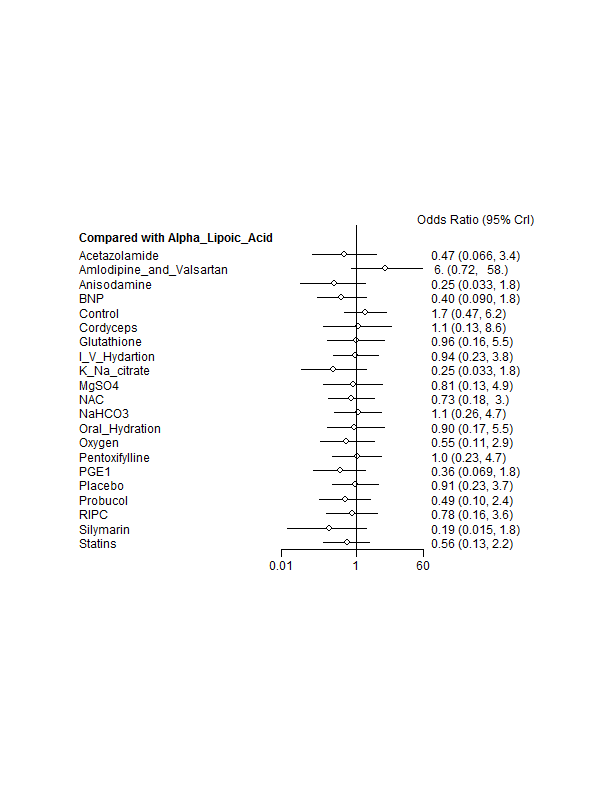

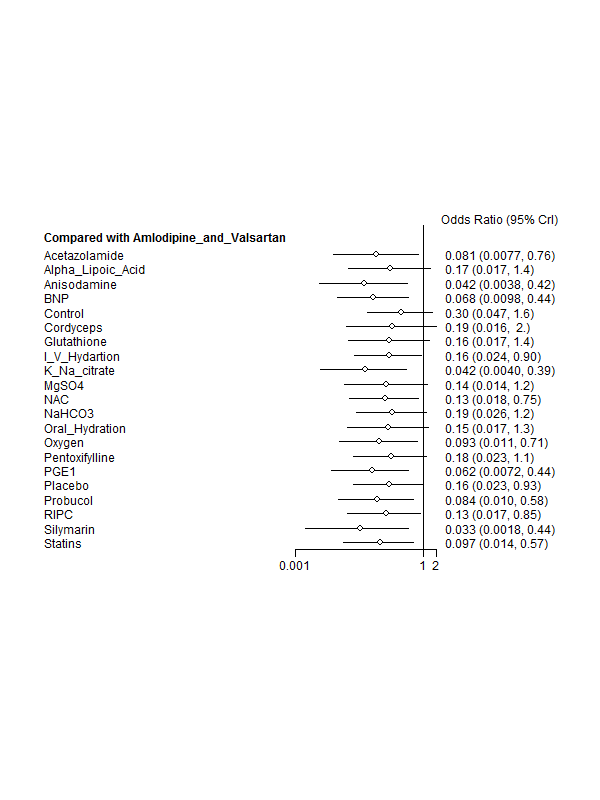

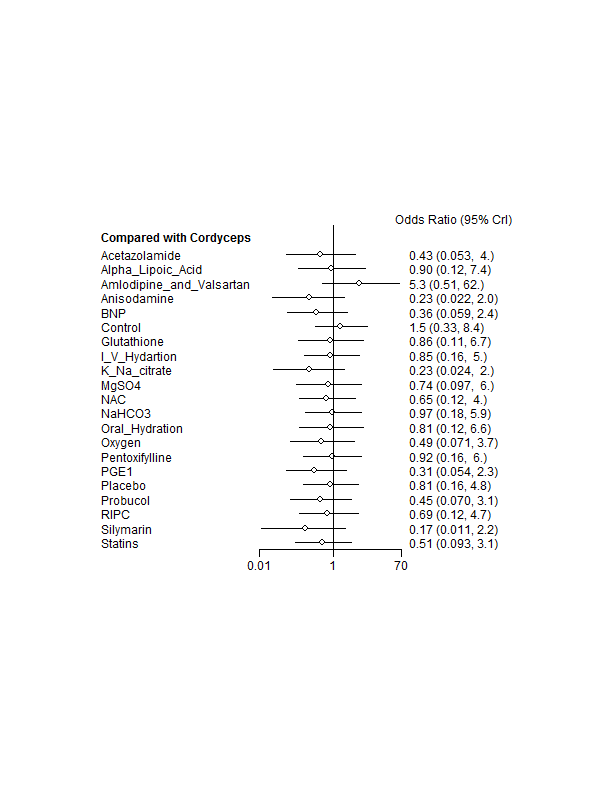

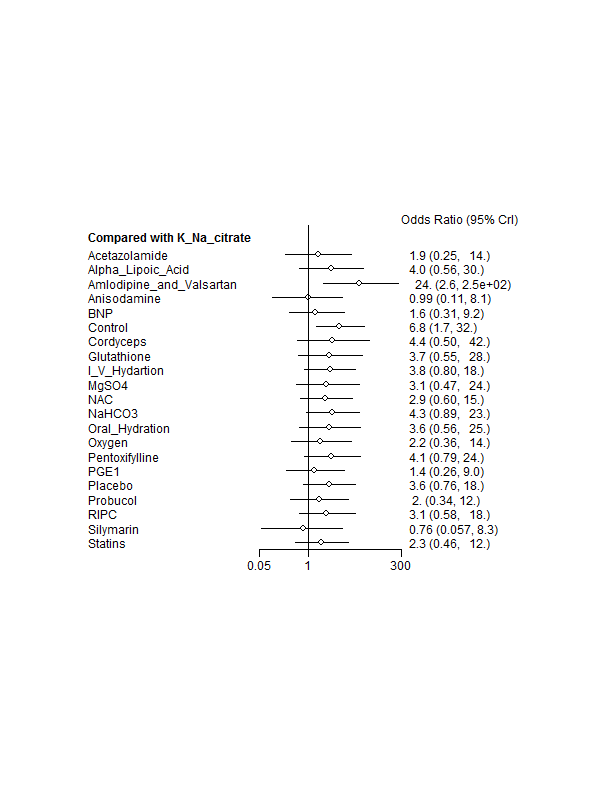

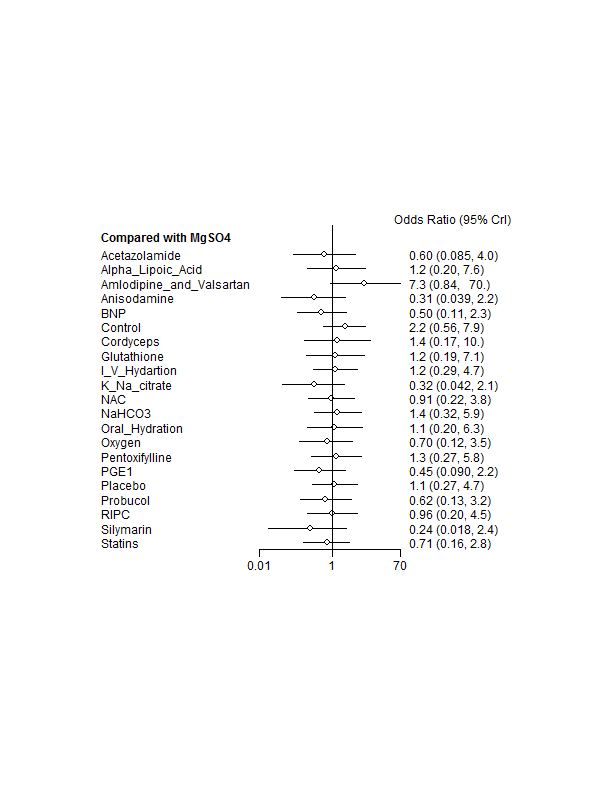

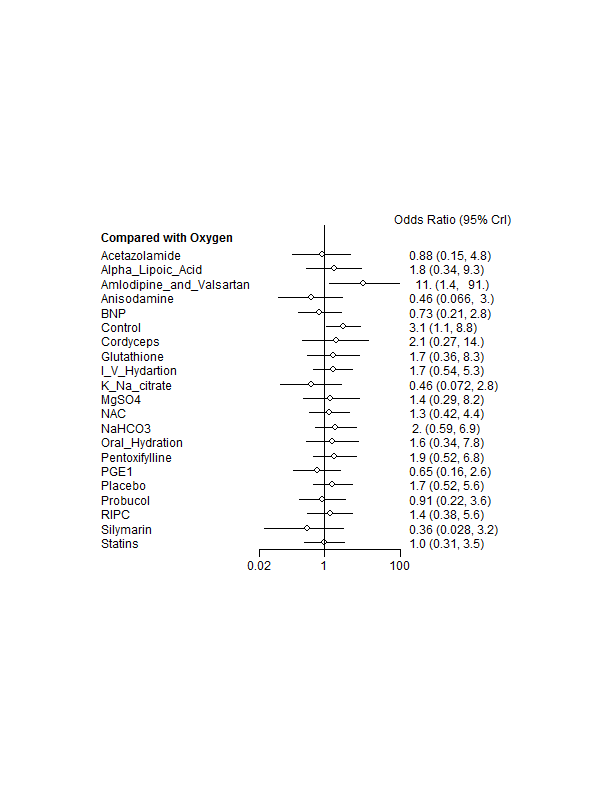

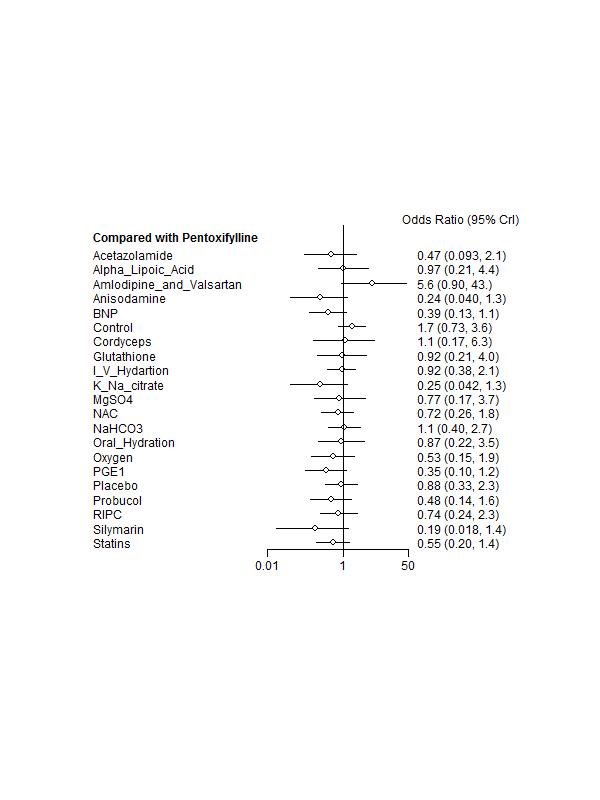

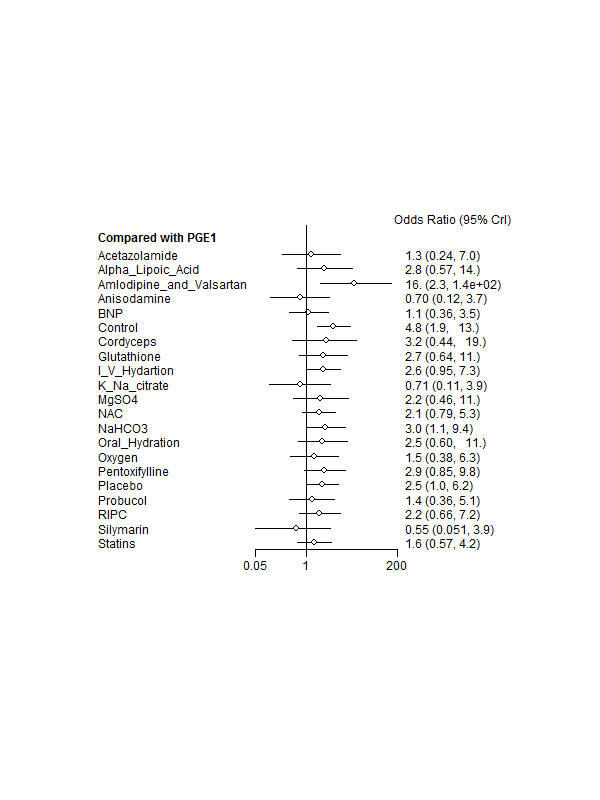

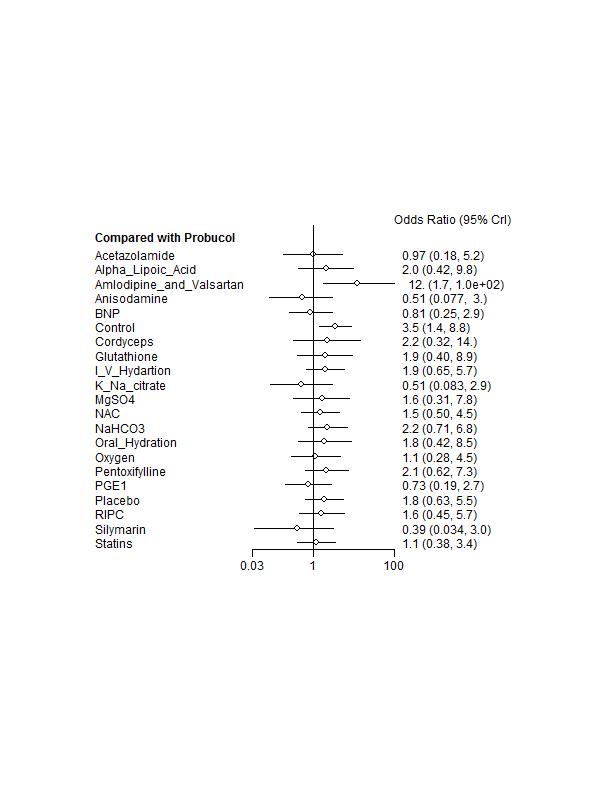

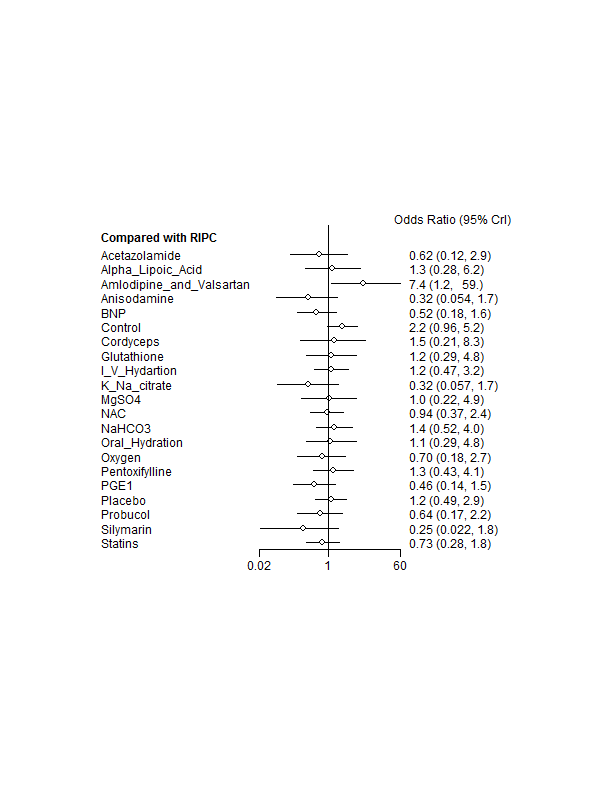

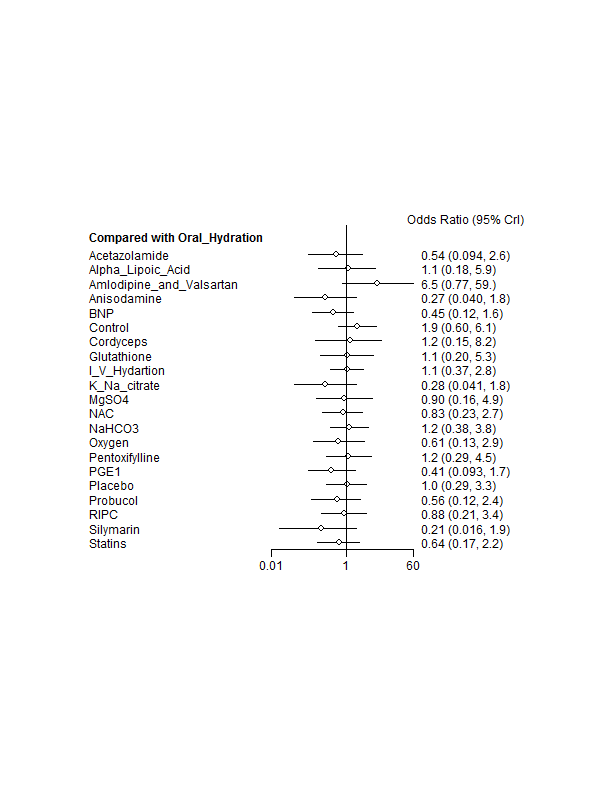

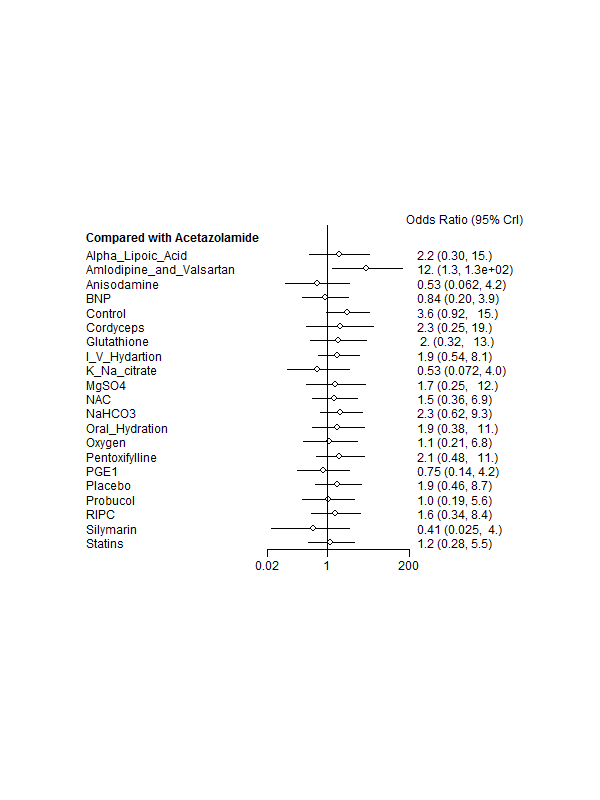

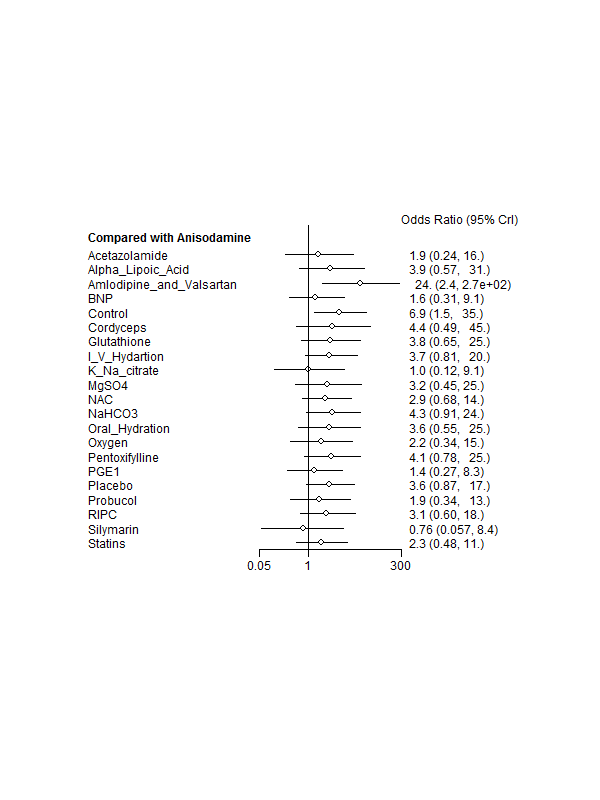

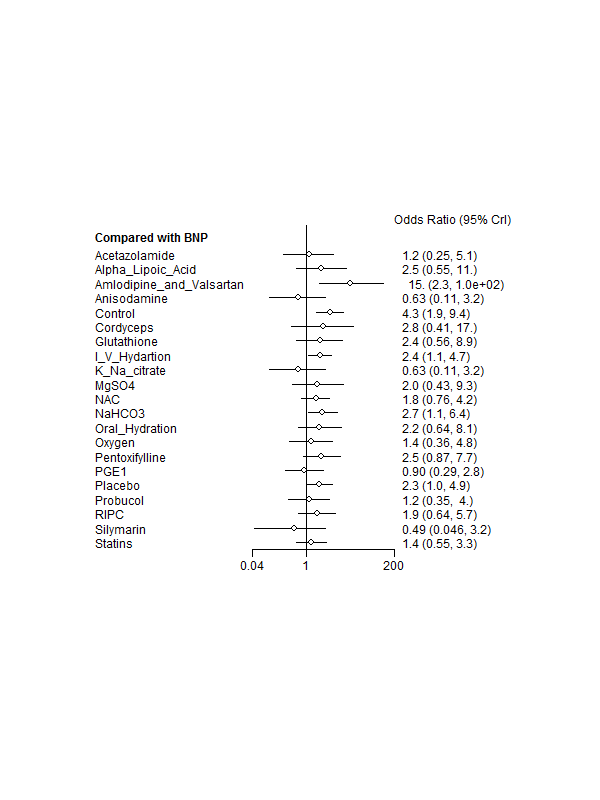

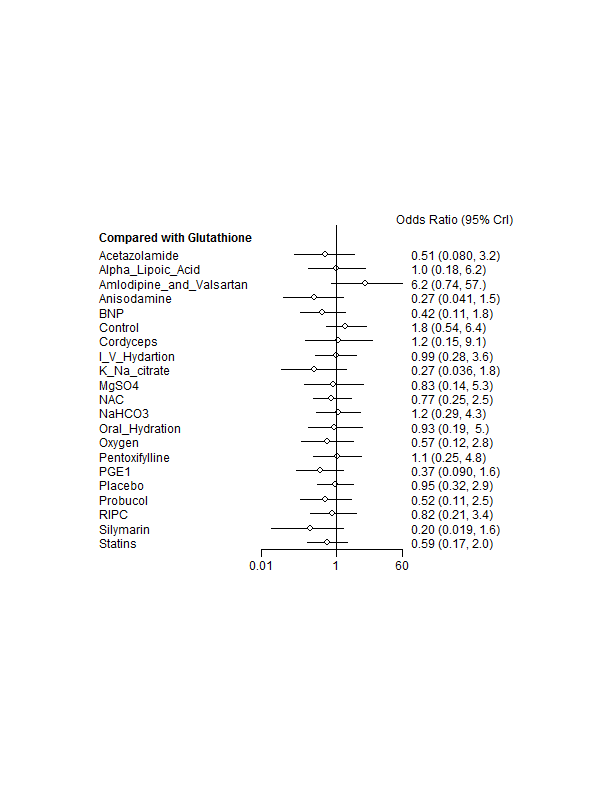

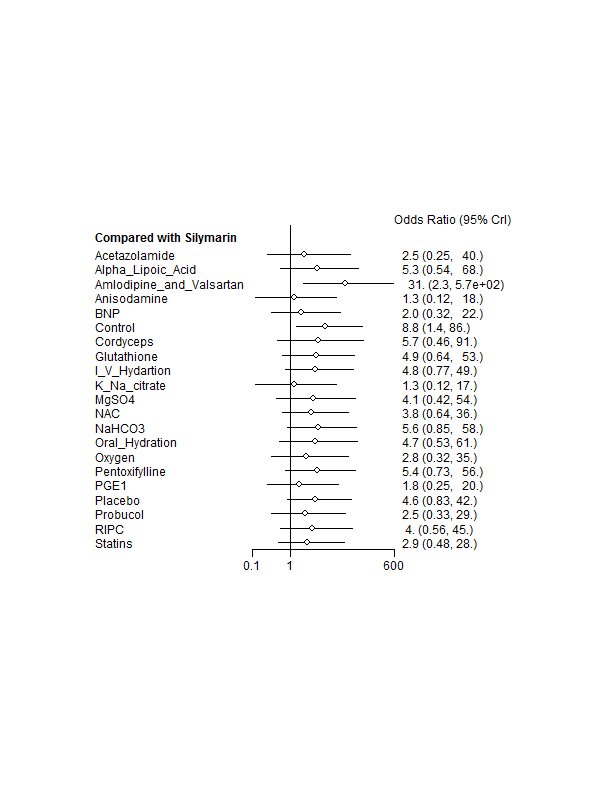


Figure 5 Gelman and Rubin's convergence diagnostics


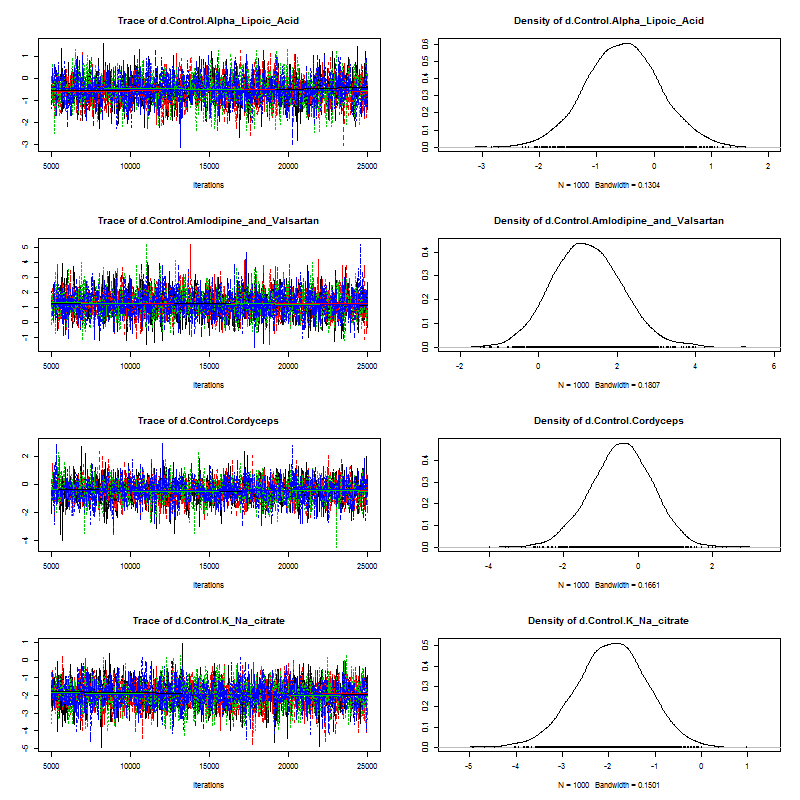


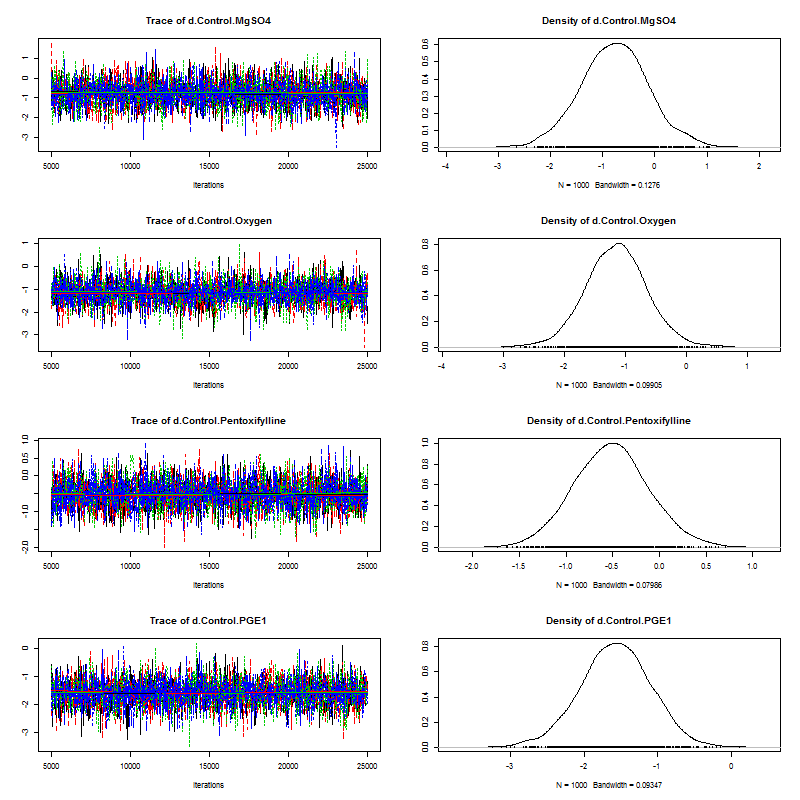

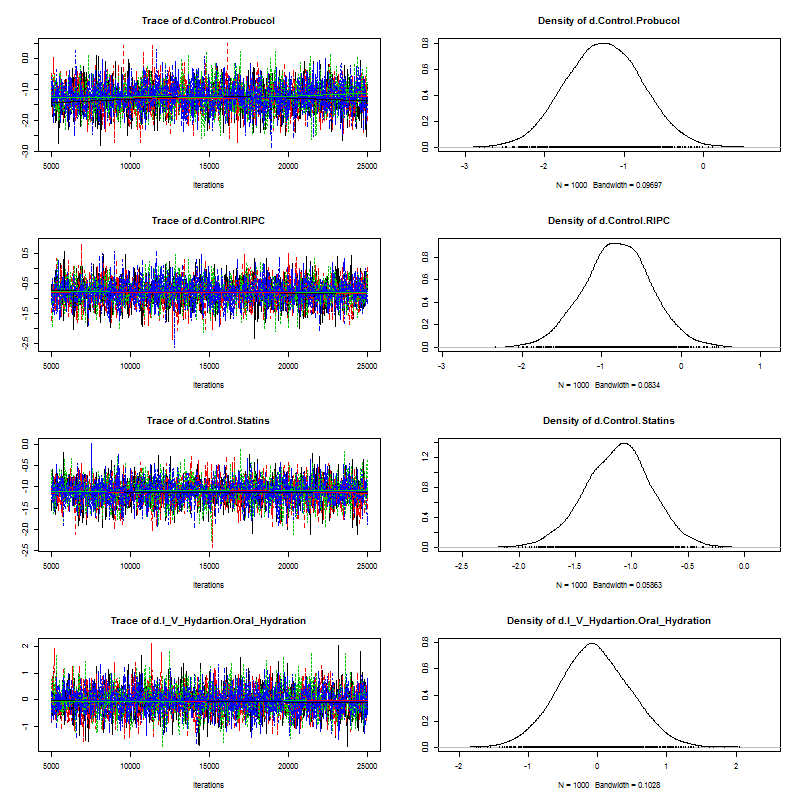

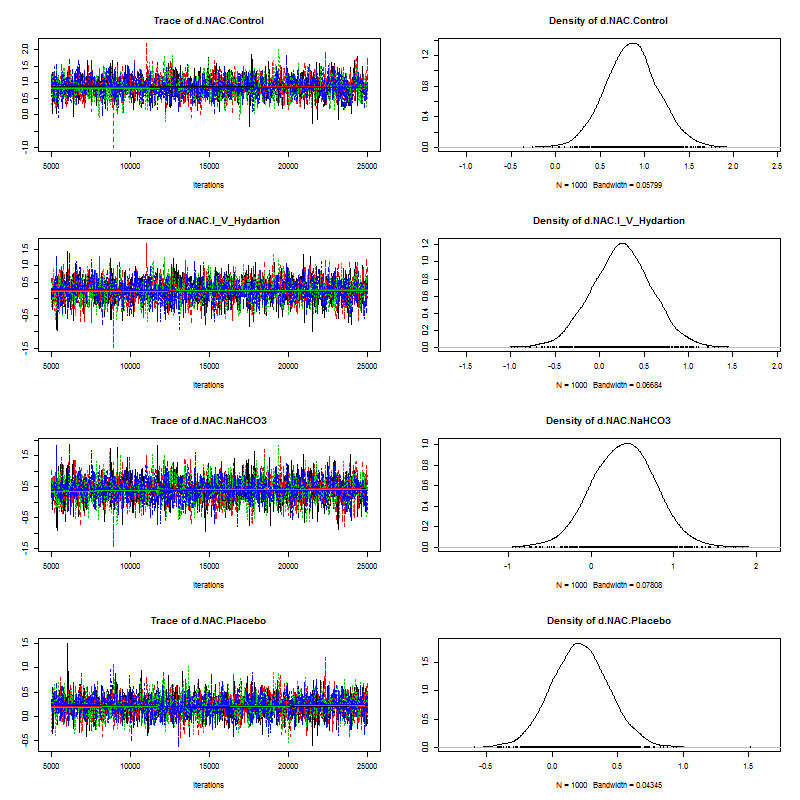

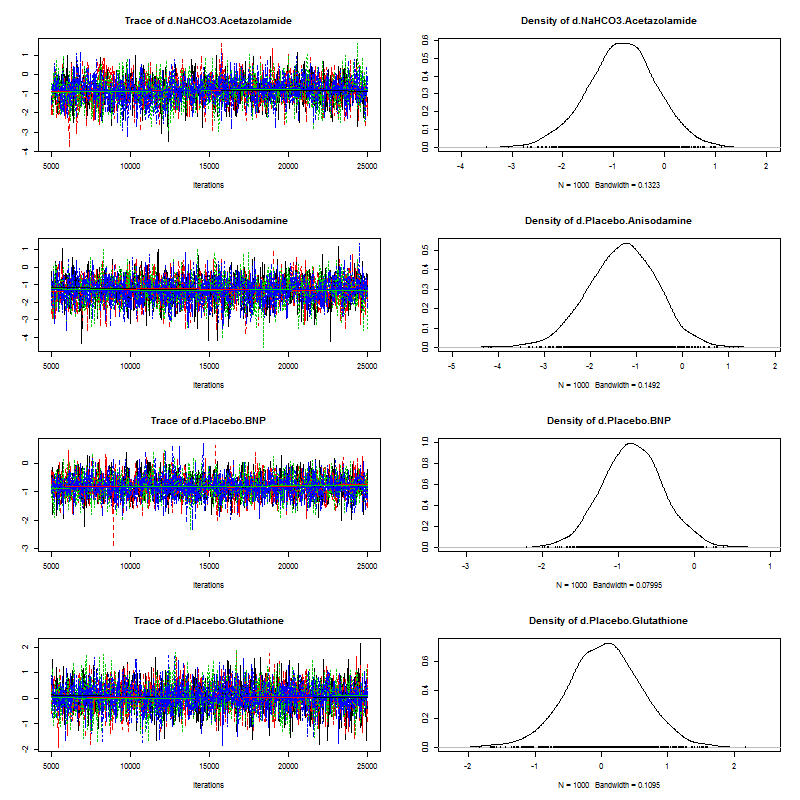

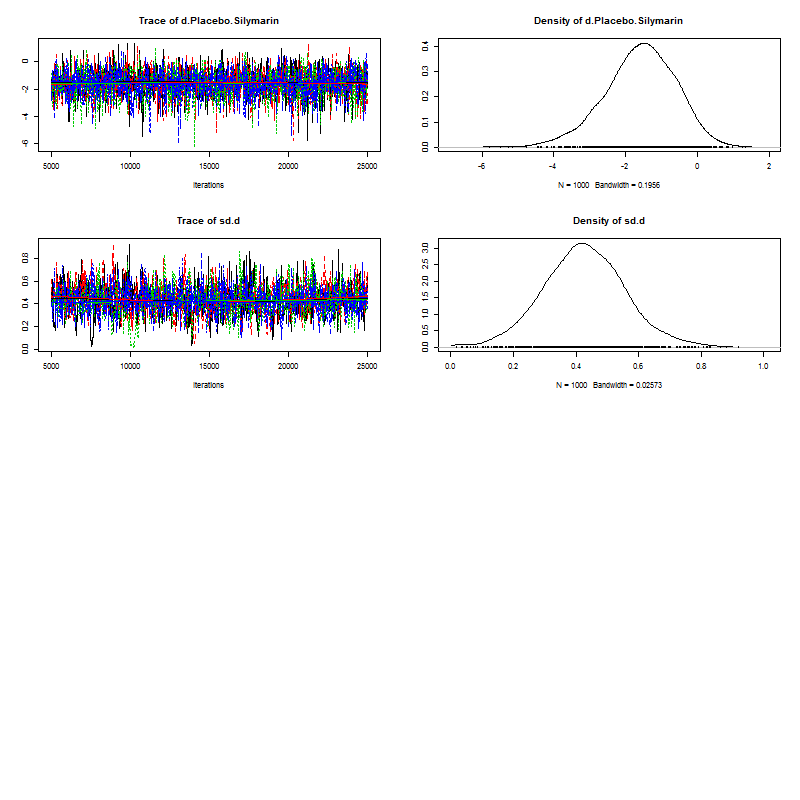


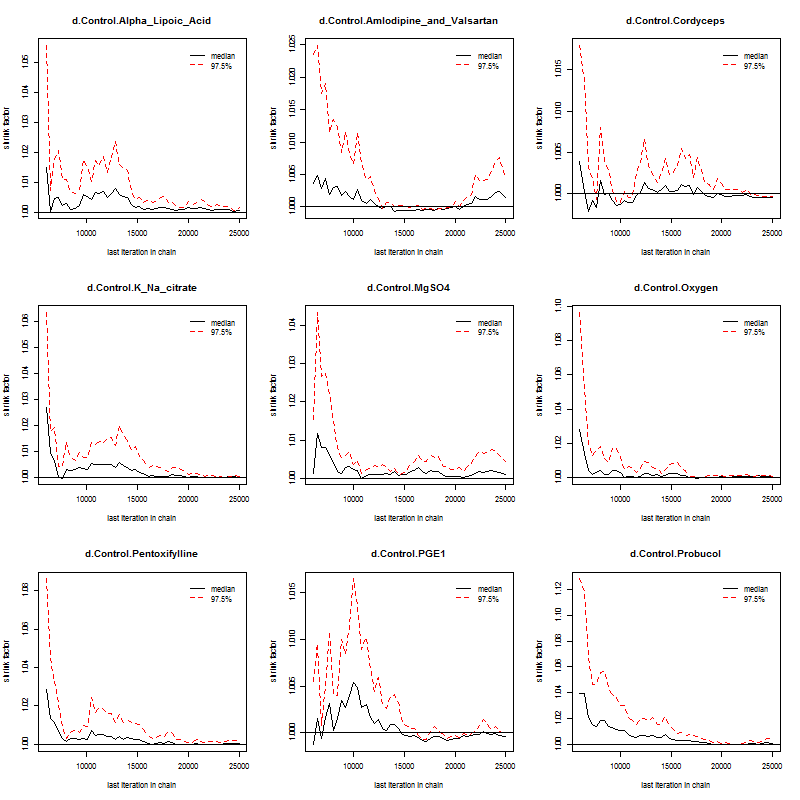

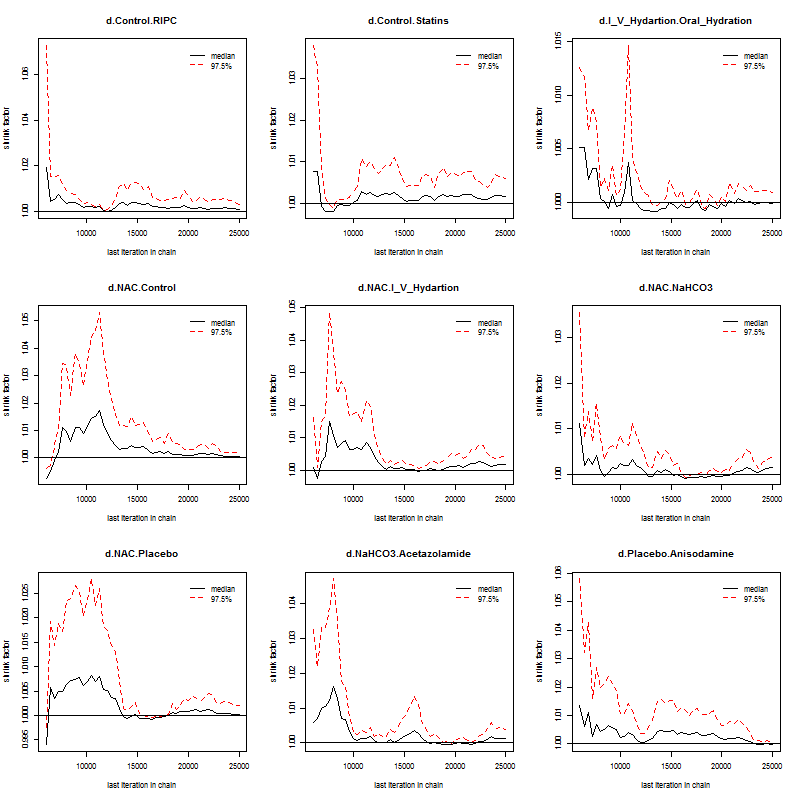

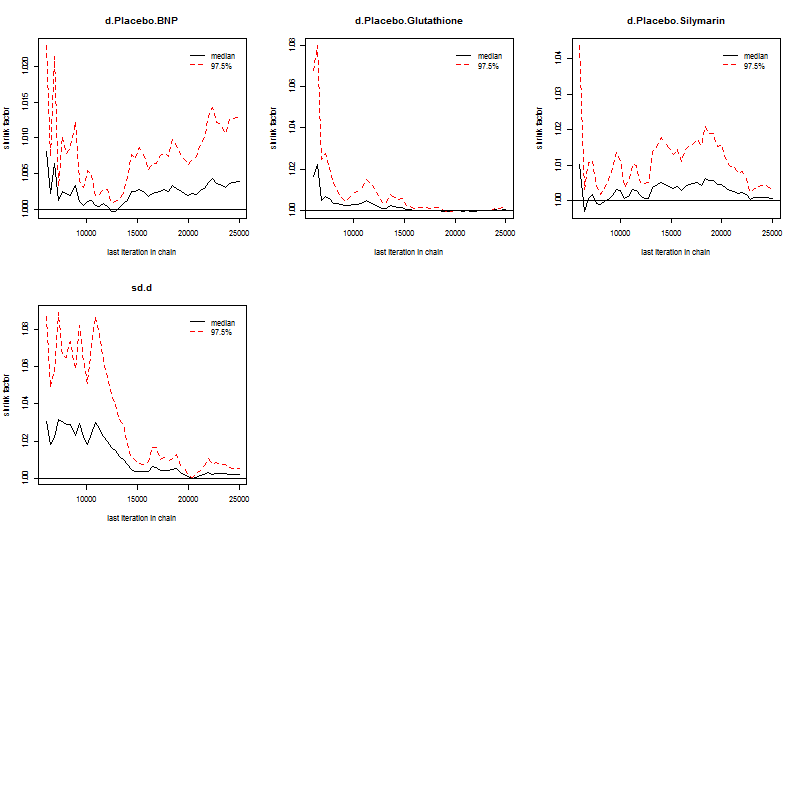


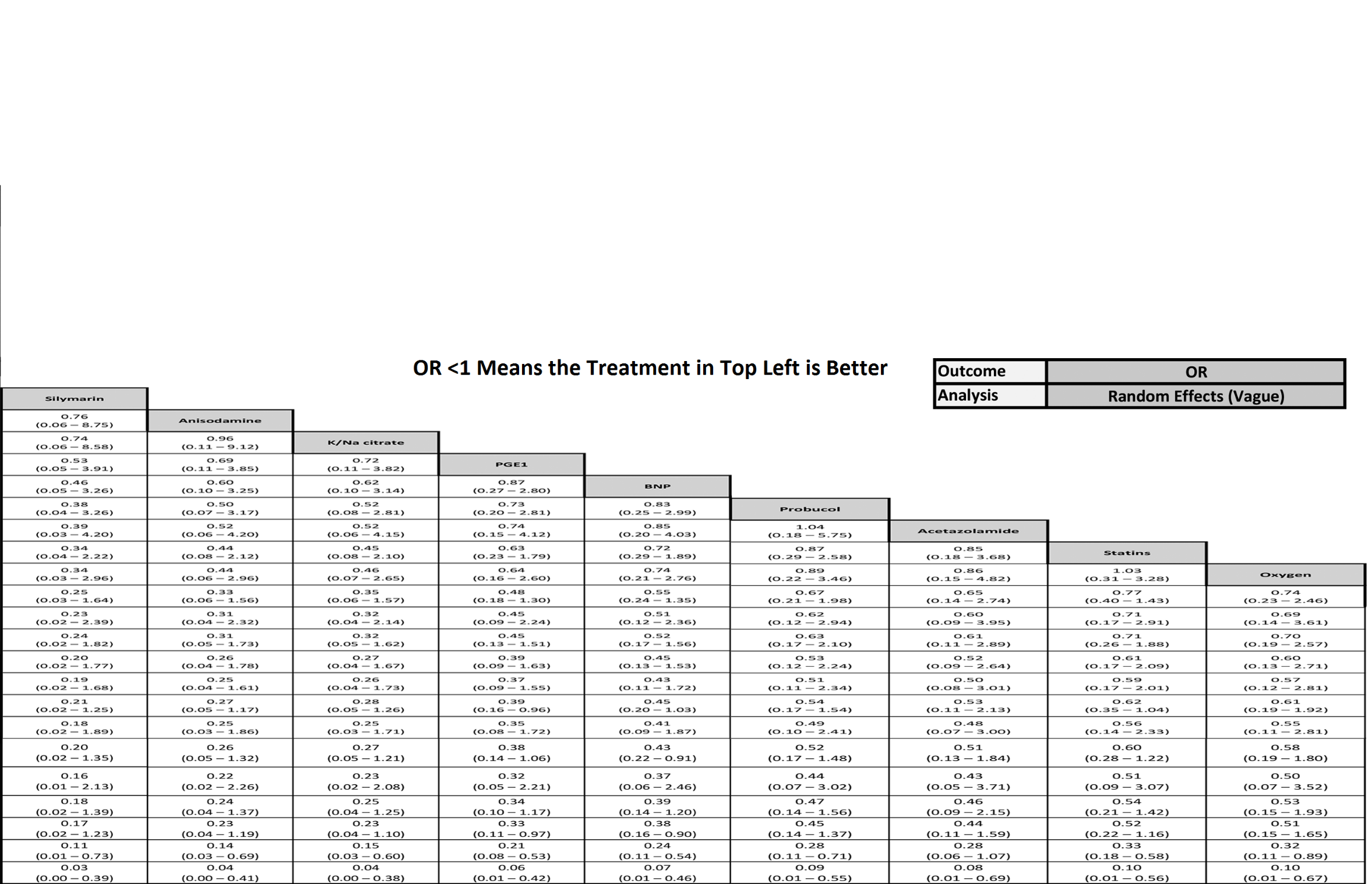


**Figure 6 League Table**


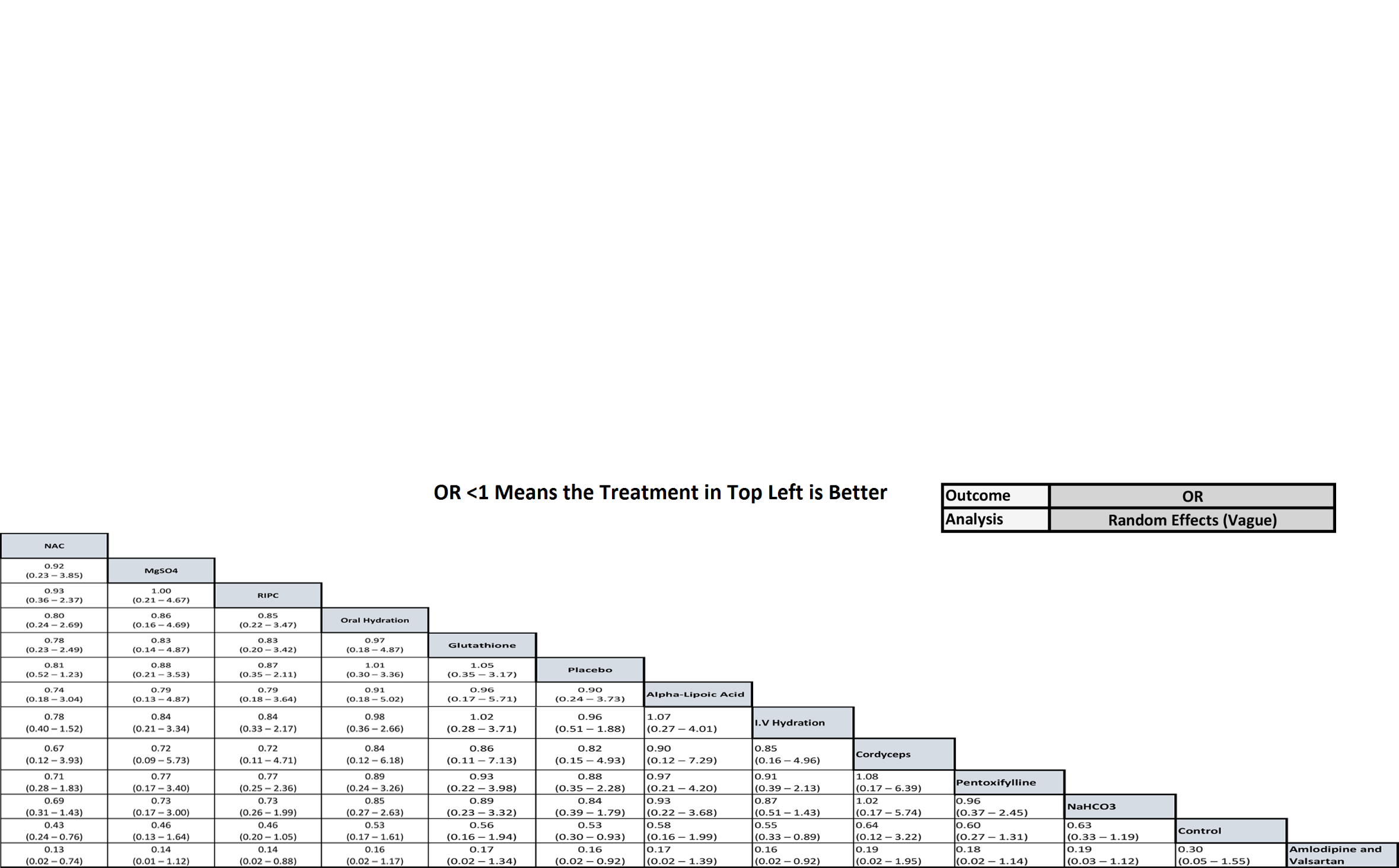

Supplement: Supplementary file 7 — Normal Baseline Renal Profile 60–53 RCTs. (DOCX 16081 kb) [file 12882_2018_1113_MOESM7_ESM.docx]
